# ISEV2018 abstract book

**DOI:** 10.1080/20013078.2018.1461450

**Published:** 2018-04-30

**1.**
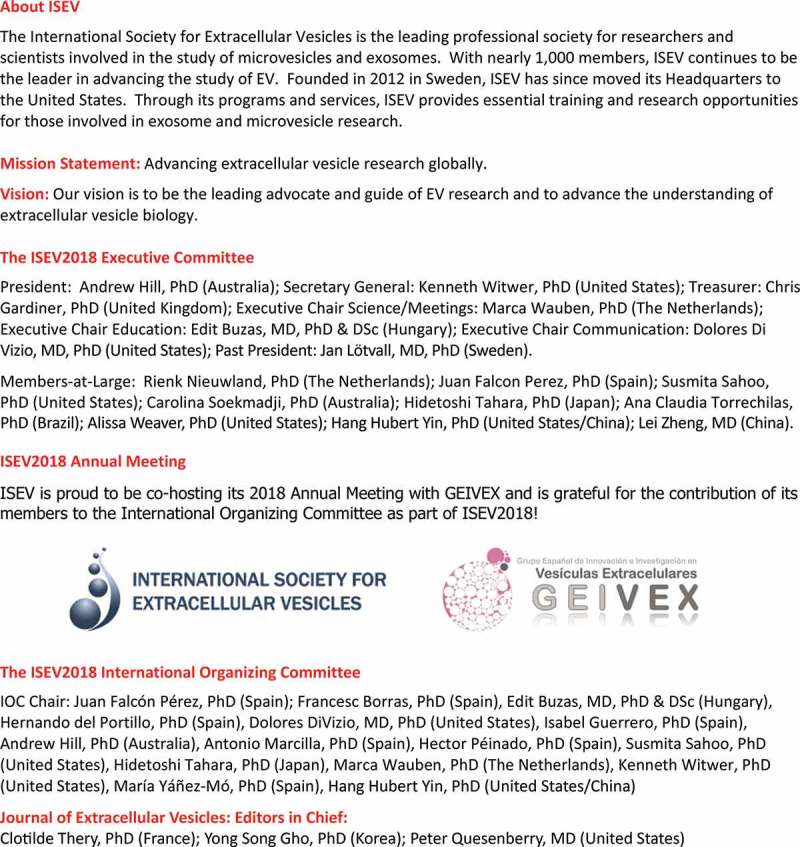

**2.**
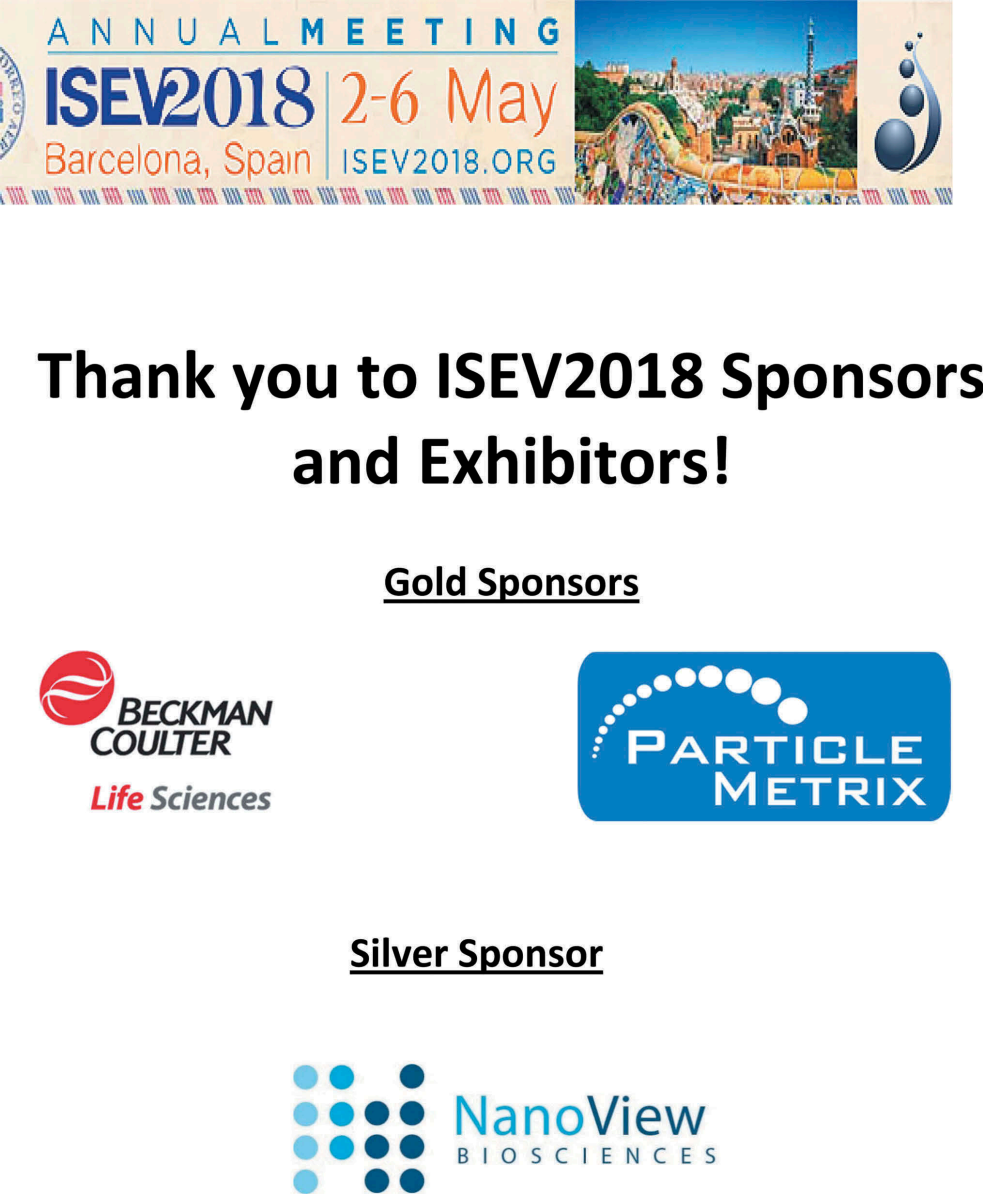

**3.**
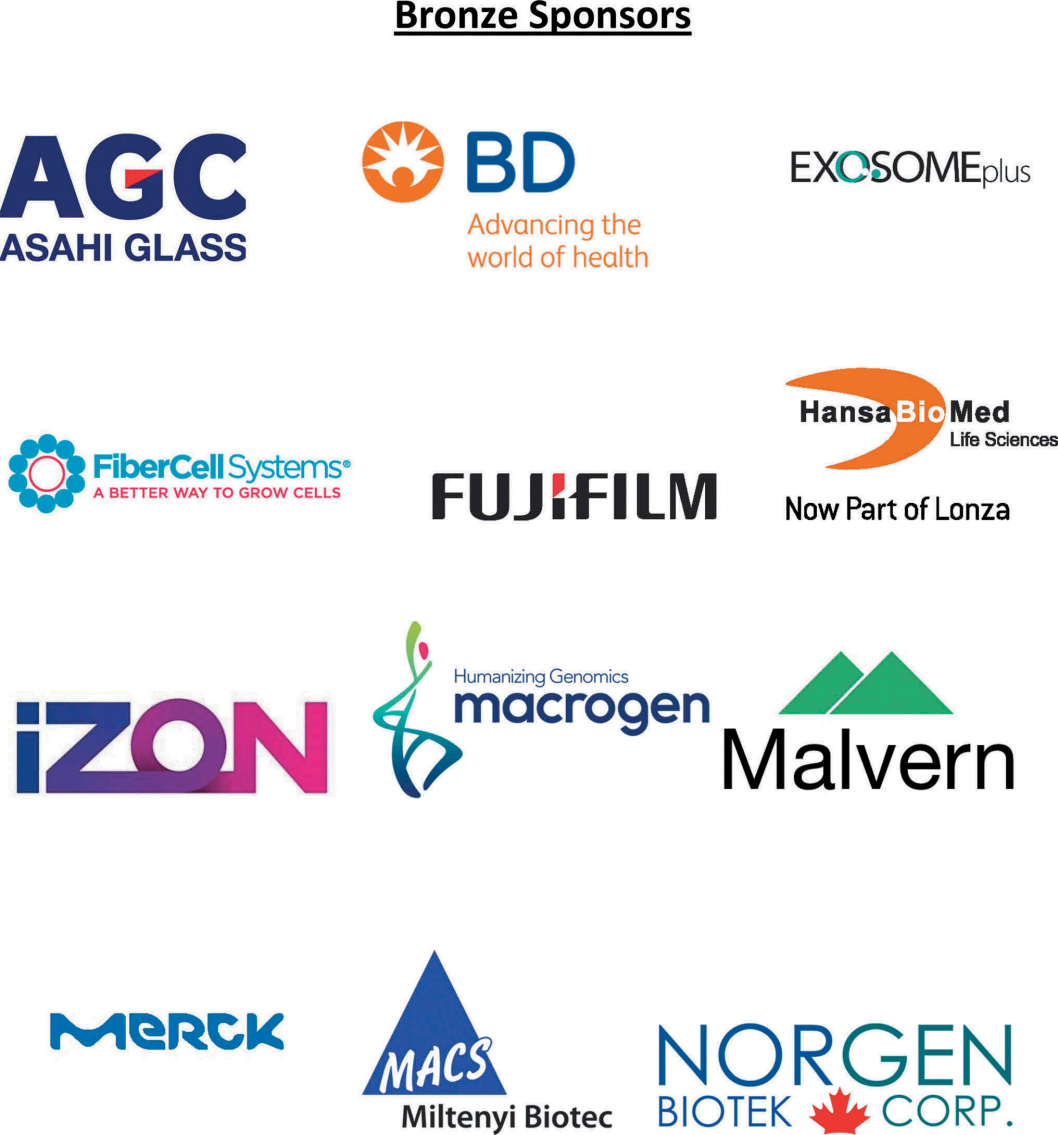

**4.**
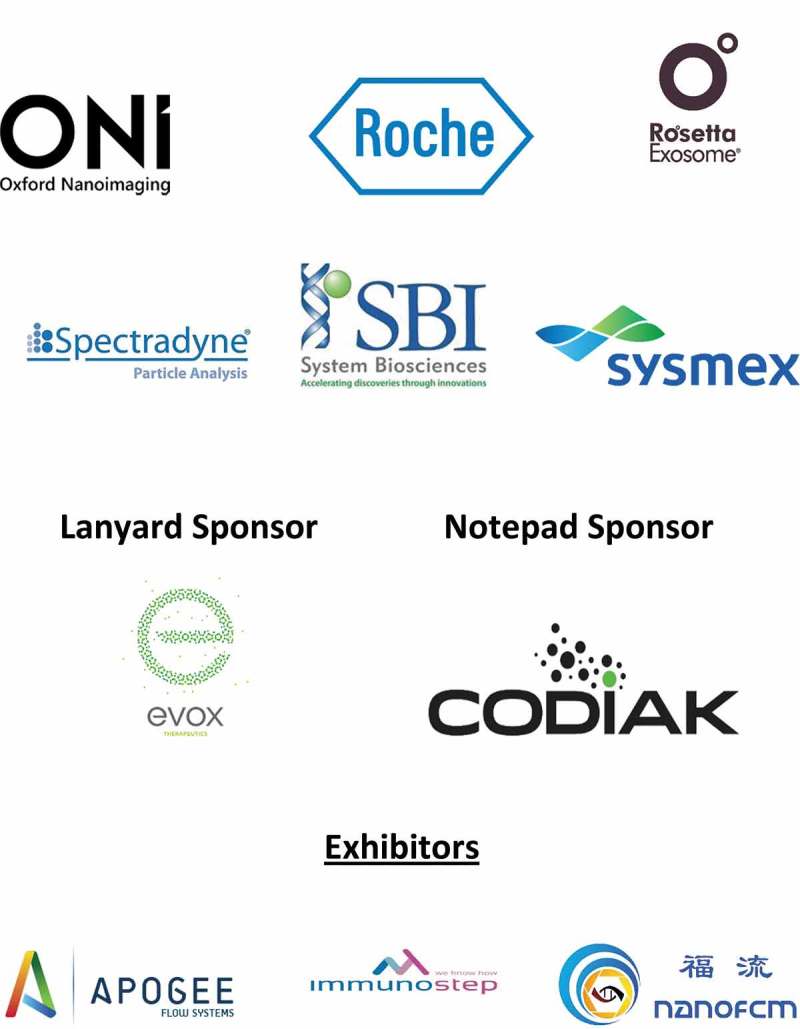

## 

Scientific Program ISEV2018Thursday, 02 May 2018PL 01: Plenary Session 1: Autophagy, Metabolism and AgeingChairs: Juan Falcón-Pérez; Andrew Hill Location: Auditorium09:30–10:30

PL 1

The Mechanism and Regulation of Autophagy

Daniel Klionsky

University of Michigan, Ann Arbor, USA

**Background**: Macroautophagy/autophagy is a process of cellular self-digestion that plays a critical role in cytoprotective responses to stress. Defects in autophagy in humans are associated with a wide range of pathologies including cancer, neurodegeneration, diabetes, and heart disease. Designing effective therapies for these pathophysiologies will require a greater understanding of the mechanism and regulation of autophagy. The overall pathway and the protein components of autophagy are highly conserved from yeast to human; over forty autophagy-related (ATG) genes have been identified in yeast, and homologs exist for many of them in more complex eukaryotes. Many questions concerning the molecular basis of the autophagy pathway remain unanswered. For example, how is the initial sequestering compartment, the phagophore, nucleated? What is the origin of the membrane used for expansion of the phagophore to form the autophagosome? What are the roles of the various Atg proteins in the process of autophagosome biogenesis?

We have been analyzing the regulation of autophagy in Saccharomyces cerevisiae. Two of the central autophagy-related proteins are Atg8 and Atg9: The amount of Atg8 determines the size of autophagosomes, whereas the Atg9 level controls the rate of autophagosome formation; therefore, we are interested in the transcriptional and post-transcriptional processes that regulate their function. The ATG8 gene in particular is controlled through a complex network that involves negative regulation through several distinct mechanisms; this ensures an appropriate level of homeostatic autophagy, while preparing cells to rapidly induce autophagy when they encounter stress.

**Funding**: This work is supported by NIH grant GM053396.

PL 2

A Way Out When Selective Autophagy Fails in Aging

Ana Maria Cuervo

Albert Einstein College of Medicine, New York, USA

Autophagy encompasses a series of intracellular pathways that mediate the delivery and degradation of cytosolic components – organelles and proteins – in lysosomes. Three types of autophagy have been described in mammalian cells: macroautophagy, microautophagy and chaperonemediated autophagy (CMA). Malfunctioning of these systems contribute in large extend to the abnormal accumulation of those altered components in cells and tissues in numerous diseases and in aging. Our recent studies have focused primarily on the degradation of proteins in lysosomes through two selective forms of autophagy in mammals, endosomal microautophagy (eMI) and CMA, where substrate proteins are delivered to the degradative compartment by chaperones. Hsc70, the same chaperone involved in substrate targeting to CMA, contributes to the delivery of substrates for selective e-MI.

In recent years, the better molecular characterization of CMA and the development by our group of mouse models with selective blockage of CMA has considerably advanced our understanding of the physiological role of this pathway in aging and in age-related disorders where CMA malfunctioning has been described. Furthermore, we have identified active cross-communication between both pathways whereby a blockage on CMA leads to re-routing of cytosolic proteins toward eMI. This shifting from one autophagic pathway to the other is normally an effective compensation. However, in some pathological conditions failure to degrade the rerouted proteins leads to their release to the extracellular media and may contribute to extracellular proteotoxicity and disease propagation. In this talk, I will describe our recent findings on the consequences of the functional decline of CMA with age on brain aging and on the progression of different neurodegenerative disorders as result of this failure. I will also share some of our current efforts to modulate CMA activity either genetically or chemically with neuroprotective purposes in aging.

Symposium Session 1 – EVs in Metabolic DisordersChairs: Juan Falcón-Pérez; Susmita Sahoo Location: Auditorium 10:45–12:15

OT01.01

The bystander effect of exosomes in ageing

Michela Borghesan; Juan Fafian-Labora; Paula Carpintero-Fernández; Ana O’Loghlen

Queen Mary University of London (UK), London, United Kingdom

**Background**: Ageing is a process of tissue function decline characterized by the presence of senescent cells. Senescent cells are permanently cell cycle arrested cells with a particular secretory phenotype denominated senescence-associated secretory phenotype (SASP) that influences the microenvironment. Here, we report for the first time that exosomes form part of the SASP and transmit the senescent phenotype to neighbouring cells.

**Methods**: In this study, we have used a combination of functional assays, super-resolution imaging, reporter systems followed by single-cell imaging, high-throughput screens and proteomic and transcriptomic analysis to identify a role for exosomes in senescence and ageing.

**Results**: We have found that blocking exosome biogenesis by the use of small molecular inhibitors or siRNA targeting key proteins regulating the endocytic pathway prevents the activation of paracrine senescence. A comparative analysis of the soluble and the exosome fraction shows that both are responsible for intercellular communication. In fact, the treatment of normal human primary diploid fibroblasts with an equal number of exosomes derived from control and senescent cells induces paracrine senescence in primary and cancer cell lines. By taking advantage of a Cre-loxP reporter system, we can confirm at a single-cell level that the cells internalizing exosomes derived from senescent cells activate this program, showing direct functionality. Proteomic analysis of the exosome content from control and senescent exosomes followed by an siRNA functional screen identify the activation of a non-canonical interferon (IFN) pathway mediated by exosomes purified from senescent cells.

**Summary/conclusion**: In summary, here we are showing a functional role for exosomes as part of the senescent secretome being mediators of paracrine senescence. In fact, our data could explain why the SASP has pleiotropic activity in cancer in addition to being key in contributing to systemic age-related tissue decline.

**Funding**: AO’s lab is supported by the BBSRC (BB/P000223/1). MB is funded by the MRC (MR/K501372/1). PCF and JFL are funded by the Xunta de Galicia Fellowships (Spain).

OT01.02

Protective effects of caloric restriction on Alzheimer’s disease progression: role for choroid plexus derived extracellular vesicles?

Charysse Vandendriessche ^1^; Sriram Balusu^2^; Caroline Van Cauwenberghe^1^; Marjana Brkic^1^; Griet Van Imschoot^1^; Elien Van Wonterghem^1^; Roosmarijn E. Vandenbroucke^1^

^1^VIB-UGent Center for Inflammation Research, Ghent, Belgium; ^2^VIB-KU Leuven Center for Brain & Disease Research, Leuven, Belgium

**Background**: The brain is protected against external insults by the presence of tight barriers. The blood-cerebrospinal fluid (CSF) barrier is formed by choroid plexus epithelial (CPE) cells, a single layer of epithelial cells situated at the interface between blood and the CSF-containing ventricular cavities. We found that in response to systemic inflammation, the CPE cells secrete more extracellular vesicles (EVs) into the CSF. We are currently studying this process in the context of Alzheimer’s disease (AD), the most common progressive form of dementia. Amyloid β oligomers (AβO) are now recognized as one of the main players in the pathology of AD.

**Methods**: We mimic AD by the intracerebroventricular (icv) injection of AβO in wildtype mice. Quantification of the amount of vesicles is done using Nanoparticle Tracking Analysis and the importance of the CPE cells as the source of EVs is studied using immunostainings, transmission electron microscopy and primary CPE cultures. Cognition is analysed using the novel object recognition test.

**Results**: We found that in the presence of AβO, the CPE cells secrete more EVs into the CSF. Interestingly, we observed that the AβO-induced increase in EV secretion into the CSF can be blocked by a short period of caloric restriction (CR), i.e. the reduction of food intake without causing under-nutrition. By performing cognitive tests, we were able to show that the injection of AβO results in cognitive decline, while a short period of CR before the icv injection protects against the observed memory deficits.

**Summary/conclusion**: Our data show that CR prevents AβO-induced EV secretion by the CPE cells, and further research is needed to determine whether this partially explains the protective effects of CR on the AβO-induced memory decline.

**Funding**: Research Foundation Flanders (FWO Vlaanderen).

OT01.03

Diabetes mellitus drives extracellular vesicle secretion and promotes increased internalization by circulating leukocytes

Nicole Noren Hooten; David Freeman; Erez Eitan; Jamal Green; Nicolle Mode; Monica Bodogai; Yongqing Zhang; Elin Lehrmann; Alan Zonderman; Arya Biragyn; Josephine Egan; Kevin Becker; Mark Mattson; Ngozi Ejiogu; Michele K. Evans

National Institute on Aging, National Institutes of Health, Baltimore, USA

**Background**: The rising incidence of diabetes mellitus represents an important global challenge. Type 2 diabetes is a chronic degenerative metabolic disease that is characterized by hyperglycemia and hyperinsulinemia resulting from cellular insulin resistance. Accumulating data suggest that circulating factors may contribute to numerous diseases, including diabetes. Intriguing data from mouse models and *in vitro* cell culture indicate that extracellular vesicles (EVs) may be important in diabetes through altering insulin signalling.

**Methods**: We isolated plasma EVs from cross-sectional and longitudinal cohorts of euglycemic, pre-diabetic and diabetic participants of the Healthy Aging in Neighborhoods of Diversity across the Life Span study. We tested the effects of insulin resistance on EVs using primary neurons and measured EV internalization as well as gene expression in human leukocytes.

**Results**: Individuals with diabetes had significantly higher levels of circulating EVs than euglycemic controls. Using primary cortical neurons, we observed that insulin resistance increases EV concentration through modulating the autophagy pathway. Furthermore, using a proteomic approach, we examined the expression of EV proteins involved in insulin signalling and inflammation. We found alterations in EV protein levels in individuals with high levels of insulin resistance and β-cell dysfunction. Using an assay to measure EV internalization, we discovered that EVs from diabetic individuals were preferentially internalized and influenced gene expression in circulating leukocytes. EVs from diabetic individuals affected cellular pathways related to cell survival, oxidative stress and immune function.

**Summary/conclusion**: Our data indicate that insulin resistance promotes EV secretion and affects the internalization of EVs by circulating leukocytes. This knowledge may further our understanding of the pathophysiological role of EVs in diabetes and might facilitate development of novel diagnostic and therapeutic approaches. In addition, this work furthers our insight into the contribution of EVs to age-related disease.

**Funding**: This study was supported by the Intramural Research Program of the National Institute on Aging, National Institutes of Health.

OT01.04

Extracellular vesicles in type 1 diabetes: comparison of immunological properties of diverse subpopulations of extracellular vesicles of the pancreatic beta-cell secretome

Khem Giri^1^; Steffi Bosch ^1^; Laurence De Beaurepaire^1^; Dominique Jegou^1^; Mathilde Mosser^1^; Romain Fleurisson^2^; Laurence Dubreil^2^; Jean-Marie Bach^2^; Gregoire Mignot^1^

^1^IECM, ONIRIS, USC1383 INRA, Nantes, France, Nantes, France; ^2^INRA, UMR703 PAnTher, Nantes, France, Nantes, France

**Background**: Type 1 diabetes (T1D) results from the autoimmune destruction of the insulin producing beta cells in the pancreas. Sustained beta-cell stress and defective clearance of dying cells has been associated to the release of self-components recognized by immune receptors. Apoptotic beta cells release extracellular vesicles (EV) that further fuel beta-cell failure and death. We showed earlier that some beta-EV microRNA (miRokines) can directly interact with the immune receptor Toll-like 7 (TLR7) initiating immune responses independently of RNA interference. Here, we aim to explore the distribution of miRokines inside distinct beta-EV subpopulations (apoptotic bodies (AB), microvesicles (MV) and small nanosized vesicles (sEV)) and their role in the modulation of immune responses.

**Methods**: EV released *in vitro* by murine pancreatic beta cells (MIN6) under normal or situations of cellular stress (pro-inflammatory (TNFα, IL1-β, IFNγ), pro-apoptotic (UV radiation) or hypoxic (1% O_2_)) were isolated using differential centrifugation (AB 2k pellet, MV 16k pellet), and size-exclusion chromatography (sEV). EV were characterized by TRPS, western blot and qPCR analysis of miRokine-expression (miR-7a, miR-21, miR-29a/b, let-7b/c). Their aptitude to activate immune cells from non-obese diabetic mice (spleen cells, dendritic cells, macrophages) *in vitro* was assessed by flow cytometry, ELISA and qPCR.

**Results**: Pancreatic beta cells exposed to stress rapidly undergo apoptosis as shown by time-lapse caspase-3/7 microscopy. While no changes were observed for the secretion of sEV, pro-apoptotic conditions led to a significant elevation of large vesicles (2k, 16k). MiRokine expression decreased in cells in parallel to an increase in the secretome. The amount of miRokines per vesicle remained constant in large vesicles but increased in sEV after cytokine exposure. Exposure of immune cells to equal amounts of EV lowered the expression of TLR7 and IL-2 for sEV obtained under pro-inflammatory conditions. Results on EV derived from a constant number of cells are pending.

**Summary/conclusion**: We demonstrated that stress favours export of miRokines in EV. Large and small beta-EV differ in their aptitude to ferry miRokines and to modulate immune responses which might be relevant for the development of vesicle-based immune tolerance induction.

**Funding**: Pays de la Loire & ANR-10-IBHU-005.

OT01.05

Higher levels of extracellular vesicles in type 1 diabetes, a cohort study of 236 patients

Sara Tehrani; Karin Bergen; Gun Jörneskog; Håkan Wallén; Fariborz Mobarrez

Karolinska Institutet, Dept of Clinical Sciences, Danderyd University Hospital, Stockholm, Sweden

**Background**: Type 1 diabetes is associated with high risk of vascular complications in both men and women, as women with type 1 diabetes lose their natural protection against cardiovascular disease (CVD). We investigated procoagulant extracellular vesicles (EVs) in patients with type 1 diabetes, with regard to sex differences and clinical microangiopathy.

**Methods**: We included 236 patients (107 women) with type 1 diabetes and 100 healthy controls matched for age, sex and body mass index. Clinical microangiopathy was found in 106 patients, while 130 patients had no vascular complications. Plasma EV levels were assessed by flow cytometry, and lactadherin was used to detect expression of procoagulant phosphatidylserine (PS) on EVs. The concentration of PS on EVs was assessed by lactadherin mean fluorescence intensity (MFI).

**Results**: Plasma EV levels were significantly higher among patients than in controls (median 41.5 (IQR 24.6–68.5) versus 23.2 (15.3–31.8) × 10(9)/L, *p* < 0.0001). The proportion of PS-positive EVs was lower in patients compared to controls (31% (25–40) vs. 44% (43–47), *p* < 0.0001), while PS concentration on EVs (lactadherin MFI) was higher in patients than in controls (11.5 (6.3–19.2) vs 7.7 (4.7–10.9), *p* < 0.0001). No differences in levels of plasma EVs in total or PS positive EVs were found between patients with and without microangiopathy. Among patients, the PS concentration on EVs was similar in men and women as well as in patients with and without microangiopathy, while healthy women had lower PS concentration on EVs compared to corresponding men (6.2 (3.6–8.9) vs. 9.0 (6.3–12.5), *p* = 0.0003).

**Summary/conclusion**: Patients with type 1 diabetes have higher levels of circulating EVs compared to controls. Surprisingly, we found no differences in EV levels between patients with and without clinical microangiopathy. Although patients had a higher proportion of PS-negative EVs compared to controls, PS concentration on EVs was significantly higher in patients. Female controls had lower PS concentration on EVs compared to male controls, which could indicate a less procoagulant EV phenotype in healthy women. This favuorable phenotype was not found in women with type 1 diabetes and may be associated to the loss of female protection against CVD in type 1 diabetes.

OT01.06

Characterization of the exosomal proteins and their potential as regulators of systemic metabolism

Ruben Garcia Martin; Emrah Altindis; Bruna B. Brandao; Thomas Thomou; C. Ronald Kahn

Section on Integrative Physiology and Metabolism, Joslin Diabetes Center and Harvard Medical School, Boston, USA

**Background**: Intercellular communication is essential for metabolic processes. Our lab recently showed that tissues can communicate *in vivo* through secretion of exosomal miRNAs which induce changes in gene expression in other tissues. In addition to miRNAs, exosomes are loaded with proteins. However, little is known about how these vary depending on tissue source or their role in the physiological regulation of metabolism. In this study, we aimed to identify both common and unique proteins in exosomes secreted by white/brown adipocytes, hepatocytes, muscle and endothelial cells, and identify the pathways that might be regulated by these proteins.

**Methods**: Murine brown and white adipocytes, AML12 hepatocytes, C2C12 muscle cells and vascular endothelial cells were grown in culture, and exosomes released into the media isolated by ultracentrifugation. Protein cargo was identified by using tandem mass tag labelling and liquid chromatography-tandem mass spectrometry. Results were confirmed by immunoblotting and compared to cellular content.

**Results**: By comparing the exosomal proteome released by different cell types, we identified general and cell-type-specific exosomal proteins. Thus, adiponectin and lysophospholipid were only present in white adipocyte exosomes, whereas SPARC and IGFBP5 were only in myotube exosomes. Similarly, Epidermal Growth Factor Receptor (EGFR), myosin-9 and thrombospondin-5 were uniquely in exosomes of hepatocytes, endothelial cells and brown adipocytes, respectively. When compared to the relative abundance of these proteins in cells, it was clear that loading of proteins into exosomes was selective, and that some proteins were enriched in different cell types. For example, several exosomal proteins secreted by hepatocytes were also secreted by muscle cells, including members of the Serpin family, some complement factors and proteins involved in iron/copper metabolism. In contrast, white and brown adipocyte- and endothelial cell-secreted proteins that were similar included proteins of carbohydrate metabolism (PGK1 and UGP2) and proteosomal proteins. Several exosomal proteins identified here have been linked to the development of diseases such as obesity and diabetes including SPARC and LGALS3BP.

**Summary/conclusion**: Exosomes contain novel and cell-type-specific proteins that could be involved in tissue communication in healthy and disease.

Symposium Session 2 – EVs and the Immune System Chairs: Francesc Borras; Esther Nolte’t-Hoen Location: Room 5 10:45–12:15

OT02.01

Exosomal transfer of microRNAs during immune synapsis contributes to the fine-tuning of immune responses

Lola Fernández Messina ^1^; Ana Rodríguez-Galán^2^; Francisco Sánchez-Madrid^1^; Virginia G. de Yébenes^2^; Almudena R. Ramiro^2^

^1^Hospital de la Princesa, Madrid, Spain; ^2^Centro Nacional de Investigaciones Cardiovasculares (CNIC), Madrid, Spain

**Background**: MicroRNAs have emerged as potent modulators of the immune response. Previous work in the laboratory demonstrated that the formation of the immune synapse promotes the unidirectional transfer of functional microRNA-bearing exosomes from the T cell to the antigen-presenting cell.

**Methods**: To identify the specific microRNAs transferred during immune synapsis and their role in the fate and function of recipient antigen-presenting cells, we have set up an experimental model using DICER-deficient B cells isolated from CD19Creki/+ DICERfl/fl mice. These cells contain virtually no mature microRNAs as they lack this enzyme necessary for miRNA biosynthesis. *In vitro* coculture of isolated DICER-deficient B cells with OT-II-derived CD4+ T cells, which express a transgenic OVA-specific TCR, in the presence or absence of the OVA peptide allows the study of microRNAs transferred from the T cell to the B cell during immune synapsis, and their impact on the recipient cell function.

**Results**: We have identified a specific set of microRNAs transferred from the T cell to the B cell after immune synapse formation, which target key molecules for B-cell biology, including Bim and Pten. Furthermore, exosomal microRNA transfer has been shown to modulate B-cell activity, promoting class switch and proliferation.

**Summary/conclusion**: This work contributes to the understanding of the regulation of the early phases of the immune response after antigen recognition and may open new avenues for the treatment of immune malignancies.

**Funding**: SAF2014-55579-R

InmunoRegulatory Molecules in the Inflammatory Response: Role of Exosomes in Cell-Cell Commmunication

PI: Francisco Sánchez-Madrid.

OT02.02

DNA outside and inside of EVs and its role in phosphorylation of interferon regulatory factor 3

Elisa Lázaro-Ibáñez ^1^; Ganesh V. Shelke^2^; Rossella Crescitelli^2^; Su Chul Jang^3^; Anaís García^2^; Cecilia Lässer^2^; Jan Lötvall^2^

^1^Discovery Biology, Discovery Sciences, IMED Biotech Unit, AstraZeneca, Gothenburg, Sweden, Mölndal, Sweden; ^2^Krefting Research Centre, Institute of Medicine, University of Gothenburg, Sweden, Gothenburg, Sweden; ^3^Krefting Research Centre, Institute of Medicine, University of Gothenburg, Boston, USA

**Background**: In contrast to the multiple studies addressing the role of RNAs in extracellular vesicles (EVs), the presence of DNA represents a more unexplored but highly appealing source of information. EV-associated DNA may hold remarkable value in clinical diagnostics as a biomarker, since it can provide information about the characteristics of a disease, but it can also act as an immune sensor activator mediating, e.g. autoimmunity or anti-viral responses. Here, we described that DNA is associated both inside and outside of EVs and that the DNA outside EVs can activate innate immune signalling in recipient cells.

**Methods**: EVs were purified from mast (HMC-1) and erythroleukemic (TF-1) cells by differential centrifugation followed by separation by bottom-loaded iodixanol density gradients. EVs with different density were characterized by western blotting, transmission electron microscopy and particle concentration, followed by total DNA extraction and analysis. The association of the dsDNA inside or outside EVs and its coverage was evaluated by enzymatic DNase treatment followed by whole genome sequencing (WGS) of the DNA inside and outside of EVs. The innate immune activation mediated by EV-DNA in recipient cells was assessed by the phosphorylation of interferon regulatory factor 3 (IRF-3).

**Results**: EV subsets with low and high densities showed differential dsDNA profiles analysed by a bioanalyser. Low-density EVs carried small quantities of dsDNA mainly unprotected from enzymatic degradation. Instead, high-density EVs contained larger quantities of dsDNA, which was partly protected from enzymatic degradation. WGS results showed that the entire genome was present both in the total DNA and in the DNA protected from enzymatic degradation. Regardless, from 77% to 97% of the total DNA was removed by DNase treatment, arguing that most of the DNA was present on the outside of the EVs. DNase treatment of the EVs eliminated their ability to induce phosphorylation of IRF-3 in recipient cells.

**Summary/conclusion**: EVs from several cell types carry DNA on their surface, but the inability of DNase to remove all DNA suggests that some of the genetic material is still on the inside of the EVs. EVs with DNA on their surface can induce the phosphorylation of IRF-3, which suggests a role in innate immunity.

OT02.03

Cigarette smoke-exposed monocyte-derived dendritic cells release EVs that elicit Th1/Th17 responses in autologous T-cells

Zhaohao Liao^1^; Mercedes Tkach^2^; Tine H. Schøyen^2^; Matias Ostrowski^3^; Clotilde Thery^4^; Kenneth Witwer ^1^

^1^The Johns Hopkins University School of Medicine, Baltimore, MD, USA; ^2^Institut Curie, Inserm U932, Paris, France; ^3^INBIRS Institute, School of Medicine, University of Buenos Aires, Buenos Aires, Argentina, Buenos Aires, Argentina; ^4^Institut Curie / PSL Research University / INSERM U932, Paris, France

**Background**: Cigarette smoking has well-documented adverse effects on health and is more prevalent in certain populations. For example, smoking is two- to four-fold more prevalent in HIV-1-positive populations and may exacerbate non-AIDS diseases associated with chronic inflammation. In this study, we evaluated the potential of EVs from monocyte-derived dendritic cells exposed or not to cigarette smoke to elicit different immune phenotypes in autologous T-cells.

**Methods**: Peripheral blood mononuclear cells were obtained from healthy donors by venipuncture and gradient processing or leukapheresis. Cell subsets were negatively selected by immunoaffinity. Cigarette smoke extract (CSE) was prepared by passing smoke from standard tobacco cigarettes (US National Institute of Drug Abuse) through medium prepared serum-free or with EV-depleted serum. EVs were enriched by stepped ultracentrifugation from monocyte-derived dendritic cell (DC)-conditioned medium after exposure or not to CSE. Dilutions of DC EVs were applied to autologous CD4+ T-cells. T-helper responses were assessed by flow cytometry (LSR Fortessa) 1–3 days after EV treatment.

**Results**: EVs derived from CSE-exposed DCs activated autologous CD4+ T-cells (CD69) and heavily skewed T-cells towards TH1/TH17 responses. T-bet and RORgamma-T were significantly increased, as was production of IL-2 and IL-17A expression. IL-4 and IL-13 production were unchanged or slightly but non-significantly decreased, and GATA-3 expression was unaffected. Changes in NF-kappaB expression were variable and did not reach significance. Dose dependence of all positive results was observed.

**Summary/conclusion**: EVs from cells exposed most directly to cigarette smoke and its by-products may transmit inflammatory signals to other cells via EVs. We are currently investigating this phenomenon in the context of HIV infection and disease.

**Funding**: This research was supported in part by the US National Institutes of Health through DA040385 (to KWW, MO, and CT).

OT02.04

Mesenchymal stromal cell extracellular vesicles modulate innate and adaptive immune cells at multi-organ level in a model of bronchopulmonary dysplasia

Monica Reis ^1^; Gareth R. Willis^2^; Angeles Fernandez-Gonzalez^2^; Nahal Mansouri^2^; Alex Mitsialis^2^; Stella Kourembanas^2^

^1^Department of Pediatrics, Harvard Medical School, Boston, Massachusetts, USA, Boston, USA; ^2^Division of Newborn Medicine & Department of Medicine, Boston Children’s Hospital, Boston, Massachusetts, USA

**Background**: Bronchopulmonary dysplasia (BPD) is a multifactorial chronic disease that occurs predominantly in preterm infants receiving oxygen therapy and mechanical ventilation, and is characterized by lung growth arrest, diminished alveolar and blood vessel development and impaired pulmonary function. Using a murine model in hyperoxia-induced BPD, we recently showed that a bolus dose of MSC extracellular vesicles (MEx) improved lung architecture and lung function and that this therapeutic effect was associated with modulation of lung macrophage phenotypes. However, BPD is a disease with multi-organ effects. Thus, we extend our studies in this BPD model to investigate the immunomodulatory effects of MEx on the innate and adaptive immune responses at the multi-organ level.

**Methods**: Extracellular vesicles were collected from the conditioned media of human Wharton’s Jelly-MSCs and purified through density flotation in Iodixanol. Newborn mice were exposed to hyperoxia on postnatal day 1 (PN1) (75% O2), treated with MEx on PN4 and returned to room air on PN7. Treated animals and appropriate controls were harvested on PN7 and PN14 for histologic and cytometric assessment of lungs, spleen and thymus.

**Results**: Hyperoxia-exposed mice presented significant lung damage and alveolar simplification as well as medullary involution of the thymus. Injection of MEx into hyperoxic-mice improved lung histology and restored thymic cortico-medullary ratios to levels akin to their normoxic counterparts. At PN7, MEx treatment modulated macrophages into an anti-inflammatory phenotype and mobilized inflammatory LY6ChiCCR2+ monocytes in the lungs and spleens. At PN14, MEx treatment induced a multi-organ reduction of inflammatory monocytes with a shift to a regulatory phenotype. Specifically, MEx altered T-cell subpopulation levels, inducing a reduction in CD8+ lymphocytes and an increase in CD4+ lymphocytes, and promoting the generation of CD4+CD25hiFoxP3+ regulatory T cells.

**Summary/conclusion**: Using a hyperoxia-induced BPD model, we show that MSC extracellular vesicle treatment results in a profound multi-organ effect on the immune system and promotes a tolerogenic T-cell phenotype that plays a crucial role in the amelioration of BPD-associated pulmonary damage.

OT02.05

Annexin a5(An5)-bound extracellular vesicles (EVs) from mesenchymal stromal cells (MSCs) show enhanced and specific anti-inflammatory effects

Anna Maria Tolomeo^1^; Martina Piccoli^1^; Michela Pozzobon^2^; Michele Grassi^1^; Chiara Franzin^2^; Marcin Jurga^3^; Alessandra Fierabracci^4^; Melania Scarpa^5^; Andrea Porzionato^1^; Ignazio Castagliuolo^1^; Maurizio Muraca ^1^

^1^University of Padova, Italy, Padova, Italy; ^2^Stem Cells and Regenerative Medicine Lab, Fondazione Istituto di Ricerca Pediatrica Città della Speranza, Padova, Italy; ^3^The Cell Factory BVBA (Esperite NV), Niel, Belgium, Niel, Belgium; ^4^Children’s Hospital Bambino Gesù, Infectivology and Clinical Trials Area, Type 1 Diabetes Centre, Roma, Italy; ^5^Istituto Oncologico Veneto, Padova, Padova, Italy

**Background**: An5 is a physiological molecule with tolerogenic properties. Since An5 can bind to negatively charged phospholipids like phosphatidylserine on the surface of EVs, we reasoned that this molecule could affect the well-recognized immune regulatory properties of MSC-EVs. Therefore, we compared the immunological effects of MSC-EVs and of the AnV-MSC-EVs complex both *in vitro* and *in vivo*.

**Methods**: An5 was bound to human Wharton Jelly-derived MSC-EVs using a commercial kit. Our *in vitro* assay consisted of a mixed lymphocyte reaction with splenocytes from two different mice strains (C57BL/6 and BALB/c). Mice with dextran sulphate sodium (DSS)-induced colitis (3% in drinking water for 5 days) were used to test the anti-inflammatory effect *in vivo* of EVs given via enema.

**Results**: *In vitro*, both MSC-EVs and An5-MSC-EVs exhibited a suppressive effect both on B and T lymphocytes, although the latter showed a more pronounced effect. *In vivo*, MSC-EV administration via enema had no effect on body weight, disease activity index, colon length and histological parameters. In contrast, enema administration of AnV-MSC-EVs dramatically improved both clinical and morphometric/histological scores. Free (unbound) An5 administration had no effect on colitis severity. MSC-EVs and An5-MSC-EVs induced a different pattern of cytokine expression in colon mucosa.

**Summary/conclusion**: In conclusion, An5 binding enhanced the anti-inflammatory activity of MSC-EVs both *in vitro* and *in vivo*. The dissimilar pattern of cytokine expression observed *in vivo* following treatment with MSC-EVs or An5-MSC-EVs suggests that An5 binding affects the mechanisms of action of MSC-EVs.

**Funding**: The BROAD MEDICAL RESEARCH PROGRAM AT CCFA supported this work.

OT02.06

Human mesenchymal stem cells or extracellular vesicles derived from these cells administered intra-arterially modulate immune response in a rat model of ischemic brain injury

Sylwia Dabrowska ^1^; Anna Andrzejewska^1^; Miroslaw Janowski^2^; Maurizio Muraca^3^; Barbara Lukomska^1^

^1^NeuroRepair Department, Mossakowski Medical Research Centre, PAS, Warsaw, Poland; ^2^Russel H. Morgan Department of Radiology and Radiological Science, Division of MR Research, The Johns Hopkins University School of Medicine, Baltimore, USA; ^3^University of Padova, Italy, Padova, Italy

**Background**: Mesenchymal stem cells (MSCs) are a potential tool for cell-based therapies in regenerative medicine. Recent studies suggest that therapeutic functions of MSCs are linked to the production of extracellular vesicles (EVs). *The aim of the study* was to compare the immunomodulatory properties of human bone marrow mesenchymal stem cells (hBM-MSCs) and extracellular vesicles derived from them (hBM-MSC-EVs) in a rat model of ischemic brain injury.

**Methods**: hBM-MSCs (Lonza) and hBM-MSC-EVs isolated from the culture media of these cells were used in our studies. 5 × 10^5^ hBM-MSCs labelled with superparamagnetic iron oxide nanoparticles conjugated with rhodamine (Molday ION, BioPAL) or 1.3 × 10^9^ hBM-MSC-EVs stained with lipophilic dye PKH26 (Sigma) were transplanted into the right internal carotid artery of Wistar rats with focal brain injury caused by stereotactic injection of 1 μl/50nmol ouabain into the right hemisphere, 48 h after the ischemic insult. The inflow and localization of infused hBM-MSCs was monitored using MRI. Additionally, the presence of hBM-MSCs or hBM-MSC-EVs in rat brain was detected by confocal microscopy analysis. The cellular and humoral immune response in the brain of experimental animals was evaluated immunohistochemically and with Bio-Plex ProTM Cytokine, Chemokine and Growth Factor Assay (BioRad).

**Results**: We observed that both hBM-MSCs and hBM-MSC-EVs injected i.a. into focal brain injured rats migrated into insulted hemisphere and were visible near the lesion. Immunohistochemical analysis of different cell subsets in the rat brain revealed that transplantation of hBM-MSCs or hBM-MSC-EVs reduced the number of activated astrocytes (GFAP+), microglia (ED1+) and leukocytes (CD45RA+) evoked by ischemia. Moreover, the decrease of pro-inflammatory cytokines, IL-1alfa, IL-1beta, IL-6, IFN-γ, and chemokines, CXCL-1, MIP-1α, MIP-3α, MCP-1, after 1, 3 and 7 days of hBM-MSCs or hBM-MSC-EVs infusion was observed in comparison to non-treated rats with ischemic brain injury.

**Summary/conclusion**: Our analysis reveals that hBM-MSCs and hBM-MSC-EVs transplanted intra-arterially modulate immune response in rat brain caused by focal cerebral ischemia. In this experimental model, hBM-MSC-derived EVs appear to have the same anti-inflammatory effects as their cells of origin.

**Funding**: Supported by MMRC statutory grant no 6.

Symposium Session 3 – EVs as Therapeutic Agents Chairs: Yong Song Gho; Ewa Zuba Surma Location: Room 610:45–12:15

OT03.01

Extracellular vesicles released by mesenchymal stem cells represent a novel therapeutic option in systemic sclerosis

Pauline Rozier^1^; Marie Maumus^1^; Alexandre Maria^2^; Karine Toupet^3^; Christian Jorgensen^3^; Philippe Guilpain^3^; Daniele Noel ^1^

^1^Inserm, Montpellier, France; ^2^CHU Montpellier, Montpellier, France; ^3^Université Montpellier, Montpellier, France

**Background**: Systemic sclerosis (SSc) is a rare intractable autoimmune disease, with unmet medical need. Cell therapy using mesenchymal stem cells (MSC) is a promising approach, and we recently reported its efficacy in a murine model of SSc induced by hypochlorite (HOCl). Since MSC act primarily through the secretion of soluble factors released within extracellular vesicles (EV), the use of EV instead of cells seems an attractive alternative. Herein, we compared the effects of two types of EV, exosomes and microparticles, in HOCl-induced SSc.

**Methods**: BALB/c mice were challenged with daily intradermal HOCl injections for 6 weeks to induce SSc. Each group was treated at mid-experiment with infusions of 2.5 × 10^5^ murine MSC, 250 ng of exosomes or microparticles isolated from IFNγ-activated or non-activated (NA) MSC. We measured skin thickness every week. At euthanasia (d42), we analysed the expression of fibrotic and inflammatory markers (collagens 1 and 3, αSma, TGFβ, MMP 1 and 9, TIMP1, IL1β, IL6, TNFα) in lungs and skin samples using RT-qPCR.

**Results**: Mice treated with each subtype of EV displayed lower clinical scores, less histological lesions, lower expression of fibrotic and inflammatory markers, with enhanced expression of remodelling parameters in skin and lung tissues. The observed effects were similar to those obtained with MSC. No difference was noted between NA and IFNγ-activated EV.

**Summary/conclusion**: MSC-derived EV display potent antifibrotic properties in murine SSc. This new acellular therapy represents a promising approach in this disease.

OT03.02

Neoadjuvant chemotherapy elicits a pro-metastatic cascade through EVs and monocytes

Ioanna Keklikoglou ^1^; Chiara Cianciaruso^1^; Esra Guc^2^; Mario Leonardo Squadrito^1^; Simon Tazzyman^3^; Luisa Iruela-Arispe^1^; Claire E. Lewis^3^; Jeffrey W. Pollard^2^; Michele De Palma^1^

^1^Swiss Institute for Experimental Cancer Research (ISREC), School of Life Sciences, École Polytechnique Fédérale de Lausanne (EPFL), Lausanne, Switzerland; ^2^College of Medicine and Veterinary Medicine, Queen’s Medical Research Institute, University of Edinburgh, Edinburgh, UK; ^3^Department of Oncology, University of Sheffield, Medical School, Sheffield, UK

**Background**: Neoadjuvant (pre-operative) chemotherapy may produce pathological complete responses (pCR) associated with survival benefit in breast cancer. However, most of the treated patients do not achieve a pCR and may develop metastatic disease. Increasing evidence suggests that primary tumours release extracellular vesicles (EVs) that can facilitate the seeding and growth of metastatic cancer cells in distant organs. We asked whether neoadjuvant chemotherapy could influence breast cancer metastasis through modulation of EV release from cancer cells.

**Methods**: 4T1 and PyMT mammary tumours were used in most studies. EVs were isolated from medium conditioned by murine mammary cancer cells using sequential ultracentrifugation, and were analysed by NTA and western blotting. For chemotherapy treatment of tumour-bearing mice, paclitaxel and doxorubicin were administered at doses of 10 and 8 mg/kg, respectively. For metastasis colonization assays in tumour-free mice, EV delivery was performed by intravenous injection of two, 2-day spaced preparations of tumour-derived EVs (15 μg in saline), followed by systemic administration of cancer cells.

**Results**: We found that regimens of neoadjuvant chemotherapy involving paclitaxel (a taxane) and doxorubicin (an anthracycline) increase spontaneous breast cancer metastasis in mice. Chemotherapy-elicited EVs (Ch-EVs) are released into the circulation of tumour-bearing mice and interact with pulmonary vascular endothelial cells (ECs). Uptake of Ch-EVs promotes EC upregulation of CCL2, a chemoattractant for metastasis-promoting monocytes. Purified Ch-EVs are sufficient to enhance the recruitment of CCR2+ monocytes to the lung of tumour-free mice and to promote metastasis from circulating breast cancer cells. Proteomic analysis of purified EVs identified a molecular regulator of Ch-EVs-induced metastasis.

**Summary/conclusion**: These findings suggest that neoadjuvant chemotherapies might also enhance the propensity of breast cancer to metastasize via the induction of pro-metastatic EVs.

**Funding**: This work was funded by grants from the Swiss Cancer League (KFS-3007-08-2012) and the Swiss National Foundation (SNF 31003A-143978 and SNF 31003A-165963) to M.D.P.

OT03.03

Shedding of bevacizumab in tumour cells-derived extracellular vesicles as a new therapeutic resistance mechanism in glioblastoma

Thomas Simon ^1^; Soritia Pinioti^2^; Franz Wendler^1^; Justin Stebbing^3^; Georgios Giamas^1^

^1^University of Sussex, Brighton, UK; ^2^CCB-KU Leuven Center for Cancer Biology, Leuven, Belgium; ^3^Imperial College, London, UK

**Background**: Glioblastoma (GBM) is the most aggressive type of primary brain tumours in humans. Anti-angiogenic therapies (AAT) such as bevacizumab, an anti-VEGF-A antibody, have been developed to target the tumour blood supply. However, mechanisms of GBM resistance to bevacizumab have been observed. Among them, an effect of AAT directly on GBM cells has been speculated but still remains unknown. In addition, bevacizumab has been shown to alter the intercellular communication of GBM cells with their direct microenvironment. Extracellular vesicles (EVs) have been recently described as main acts in the GBM microenvironment, allowing tumour and stromal cells to exchange genetic and proteomic material. The objective of this study was to examine and describe any alterations in the EVs produced by GBM cells upon treatment with bevacizumab.

**Methods**: Conditioned medium from bevacizumab-treated GBM cells was collected and EVs were isolated. Further nanoparticle tracking, mass spectrometry (MS) and western blotting (WB) analyses were performed on the GBM cells-derived EVs. Bevacizumab interaction with U87 GBM cells and respective EVs was also assessed by immunofluorescence and WB. Furthermore, effects on cell viability of bevacizumab combination with EVs production inhibitor GW4869 were also studied.

**Results**: Interestingly, bevacizumab that is able to neutralize GBM cells-derived VEGF-A was found to be directly bound to GBM cells and their respective EVs. Moreover, one of the core components for this binding appeared to be fibronectin, which was also identified as a main cargo of GBM cells-derived EVs via MS analysis. In addition, we observed that treatment with bevacizumab can induce changes in the EVs protein content, which could be potentially associated with tumour progression and therapeutic resistance. Similarly, inhibition of EVs production by GBM cells improved the anti-tumour effect of bevacizumab.

**Summary/conclusion**: Taken together, this data suggests of a potential new mechanism of GBM resistance to bevacizumab. Thus, according to our data, targeting EVs-based intercellular communication in the GBM microenvironment might constitute a new way to counteract bevacizumab resistance in GBM.

OT03.04

Synergistic effect of extracellular vesicles loaded with oncolytic viruses and paclitaxel for cancer drug delivery

Mariangela Garofalo ^1^; Heikki Saari^2^; Petter Somersalo^2^; Daniela Crescenti^3^; Lukasz Kuryk^4^; Laura Aksela^5^; Cristian Capasso^6^; Mari Madetoja^7^; Katariina Koskinen^8^; Timo Oksanen^5^; Antti Mäkitie^9^; Matti Jalasvuori^8^; Vincenzo Cerullo^6^; Paolo Ciana^3^; Marjo Yliperttula^2^

^1^Division of Pharmaceutical Biosciences, University of Helsinki, Milan, Italy; ^2^Division of Pharmaceutical Biosciences, University of Helsinki, Helsinki, Finland; ^3^Department of Oncology and Hemato-Oncology, Center of Excellence on Neurodegenerative Diseases, University of Milan, Italy, Milan, Italy; ^4^Targovax Oy, R&D, Clinical Science, R&D, Helsinki, Finland; ^5^Division of Pharmaceutical Biosciences and Centre for Drug Research, University of Helsinki, Helsinki, Finland, Helsinki, Finland; ^6^Laboratory of ImmunoViroTherapy, Centre for Drug Research, Division of Pharmaceutical Biosciences, University of Helsinki, Helsinki, Finland; ^7^Made Consulting, Turku, Finland; ^8^Biological and Environmental Science, University of Jyväskylä, Finland, Jyväskylä; ^9^Department of Otorhinolaryngology - Head and Neck Surgery, Helsinki University Hospital and University of Helsinki, Helsinki, Finland

**Background**: Cancer standard of care is commonly a combination of surgery with chemotherapy and/or radiotherapy. However, in advanced cancer patients, this approach is inefficient and may cause many side effects, including severe complications and even death. Oncolytic viruses exhibit different anti-cancer mechanism compared to conventional therapies, since they are specifically engineered to preferentially infect, replicate in and kill cancer cells instead of normal cells where their normal functions are restricted. Here, we investigated the systemic delivery of oncolytic adenoviruses and paclitaxel encapsulated in extracellular vesicles (EV) formulation in order to utilize them as carriers for cancer drug delivery

**Methods**: The *in vivo* efficacy of EV-Virus-Paclitaxel complex was tested in Balb/c nude mice after intravenous injection. Transcriptomic analysis carried out on the explanted xenografts from the different treatment groups was used to examine synergistic effect observed between the EV delivered virus and Paclitaxel into the tumours.

**Results**: We found that the obtained EV-Virus and EV-Virus-Paclitaxel formulations reduced the *in vivo* tumour growth in xenograft model of human lung cancer. Indeed, combined treatment of oncolytic adenovirus and paclitaxel encapsulated in EV showed synergistic anticancer effects both *in vitro* and *in vivo* lung cancer models. Transcriptomic analysis carried out on the explanted xenografts from the different treatment groups failed to show a simple additive effect of paclitaxel on the genetic programmes triggered by the EV virus administration indicating that a *de novo* genetic programme is triggered by the presence of the encapsulated paclitaxel.

**Summary/conclusion**: Our work provides a promising approach combining anti-cancer drugs and viral therapies by intravenous EV delivery as new therapeutic strategy aimed at treating lung cancer

**Funding**: Post doc pool foundation and Tekes 3D-Nano-MiniT-project (M.G.), Tekes EV-Extra-Tox and 3D-Nano-MiniT projects (P.S), Orion Foundation, Professor pool (M.Y.), Alfred Kordelin and Eemil Aaltonen Foundations (H.S.), Italian Association for Cancer Research grant IG-11903 (P.C).

OT03.05

Milk exosomes – a “platform” nano-carrier for siRNA delivery

Ramesh C. Gupta ^1^; Farrukh Aqil^2^; Jeyaprakash Jeyabalan^3^; Ashish kumar Agrawal^3^; Al-Hassan Kyakulaga^4^; Radha Munagala^2^

^1^Department of Pharmacology and Toxicology and JG Brown Cancer Center, University of Louisville, Louisvilleq, USA; ^2^Department of Medicine and JG Brown Cancer Center, University of Louisville, Louisville, USA; ^3^JG Brown Cancer Center, University of Louisville, Louisville, USA; ^4^Department of Pharmacology and Toxicology, University of Louisville, Louisville, USA

**Background**: siRNAs have been considered as viable therapeutics for gene silencing and the disease inhibition. Interest in the siRNA therapy has ever been increasing from both academia and industry. Recent data suggest that nano-carriers such as viruses, polymeric nanoparticles, liposomes and exosomes derived from cell culture can be loaded with siRNAs to achieve gene silencing. However, progress in this field towards translatability is hampered due to unavailability of a scalable and biocompatible source of nanomaterials. Here, we report that bovine milk exosomes, which are biocompatible, scalable and cost-effective, can be developed as a “platform” nano-carrier for siRNA-mediated gene silencing as shown in different cancer cell types.

**Methods**: Exosomes were isolated from bovine milk by differential centrifugation, and siRNA was loaded into the exosomes by either electroporation or chemical transfection reagent, ExoFectR. Following transfection of human lung, breast, ovarian and pancreatic cancer cells by the exosomal-siRNA (Exo-siRNA) formulation for 24 or 48 h, cells were harvested, and the cell lysates were analysed by western blot. Test siRNAs included siEGFR, siVEGF, siAkt, siSurvivin, siKras and siMAPK. Anti-proliferative activity of Exo-siKrasG12S was determined against A549 lung cancer cells by MTT assay.

**Results**: siAkt incorporated by electroporation when tested in H1299 lung cancer cells showed >80% gene silencing. siEGFR when incorporated by ExoFectR reagent showed dose-dependent gene silencing in H1299 lung cancer cells. The other siRNAs tested in H1299 and A549 lung cancer cells included siAkt, siVEGF, siKras, siSur and siMAPK all of which silenced target genes significantly. Significant gene silencing also occurred for siVEGF in pancreatic MiaPaCa cancer cells, for siVEGF and siKras in A549 lung cancer cells, for siSur in ovarian A2780 cancer cells and for siSur in MCF-7 and MDA-MB-231 breast cancer cells. The exosome and siRNAs alone treatment showed no significant effect on the gene expression. Exo-siKrasG12S showed dose-dependent anti-proliferation of the A549 cells.

**Summary/conclusion**: Our data suggest that the milk exosomes loaded with various siRNAs can lead to significant target gene silencing, and that the system can be advanced as a platform technology.

**Funding**: From Duggan Endowment and Helmsley Trust Fund.

OT03.06

Bovine milk-derived extracellular vesicles can inhibit catabolic and inflammatory mediators in articular chondrocytes and fibroblast-like synoviocytes from osteoarthritis patients

Bartijn Pieters ^1^; Onno Arntz^1^; Danny Kartoidjojo^1^; Anouk Feitsma^2^; Joost van Neerven^2^; Peter van de Kraan^1^; Fons van de Loo^1^

^1^Experimental Rheumatology, Radboudumc, Nijmegen, The Netherlands; ^2^FrieslandCampina, Amersfoort, The Netherlands

**Background**: Osteoarthritis (OA) is an age-related musculoskeletal disease characterized by low-grade synovial inflammation and articular cartilage degeneration. Currently, there is no cure and limited drugs to slow disease progression. Previous studies have shown the anti-inflammatory potential of bovine milk-derived EVs (MEVs) in mice. However, little is known how this translates to the human situation. In this study, we investigated the effects of MEVs on articular chondrocytes and synovial fibroblasts from OA patients.

**Methods**: MEVs were isolated from commercial skimmed cow milk using a standard differential ultracentrifugation protocol. Particle concentration, size and floating density were assessed by NTA analysis and sucrose density gradient, respectively. Articular chondrocytes and primary fibroblast-like synoviocytes (FLS) were stimulated for 24 and 48 h with MEVs and gene expression profiles were studied by RT-qPCR. Additionally, short stimulations (2 h) were performed to study direct TGFβ-receptor activation.

**Results**: Stimulation with 10–100 µg/ml MEVs was able to effectively reduce expression of catabolic enzymes (ADAMTS5, MMP1, MMP3) and inflammatory mediators (IL6, IL8, TNFα) in articular chondrocytes. Additionally, we observed a significant increase in expression of the metalloproteinase-inhibitor TIMP3. Stimulation of primary FLS showed similar results, with marked reduction of catabolic enzymes (ADAMTS5, MMP1) and also increased in TIMP3 levels. The reduction in inflammatory mediators was, however, not found, and, in contrast, IL6 was significantly increased in FLS after exposure to MEVs. Short exposure of chondrocytes to MEVs led to induction of early TGFβ response genes (*JUNB, SMAD7, PAI*), which was completely blocked using an anti-TGFβ1,2,3 antibody.

**Summary/conclusion**: Human articular chondrocytes and synovial fibroblasts exposed to MEVs show reduced destructive and inflammatory potential. The induction of early TGFβ response genes after short incubations confirms the presence of active TGFβ, which could explain, in part, the anti-inflammatory and reduced catabolic profiles found. These findings highlight the therapeutic potential of MEVs in OA, where inflammatory and catabolic mediators are responsible for joint pathology and subsequent loss of mobility.

**Funding**: FrieslandCampina R0003572.

Symposium Session 4 – EVs as Cancer Biomarkers Chairs: Takahiro Ochiya; Carla Oliveira Location: Auditorium 13:30–15:00

OT04.01

EV analysis of clinical urine samples from prostate cancer patients

Thomas A. Hartjes^1^; Janne Leivo^2^; Mirella Vredenbregt - van den Berg^1^; Joke Veldhoven-Zweistra^3^; Chris Bangma^3^; Adriaan B. Houtsmuller^4^; Wytske van Weerden^3^; Guido W. Jenster^1^; Martin E. van Royen ^4^

^1^Erasmus Medical Center, Rotterdam, The Netherlands; ^2^University of Turku, Turku, Finland; ^3^Department of Urology, Erasmus MC, Rotterdam, The Netherlands; ^4^Department of Pathology, Erasmus Optical Imaging Centre, Erasmus MC, Rotterdam, The Netherlands

**Background**: Urinary extracellular vesicles (EVs) are a promising source of biomarkers for urogenital cancers, but EV analysis in clinical samples remains challenging. We recently developed two assays that enable quantification and characterization EVs in urine (EVQuant and TR-FIA) and applied these on minimally processed urines of prostate cancer (PCa) patients.

**Methods**: Urinary EVs from patients with (*n* = 80) and without (*n* = 80) PCa, men after radical prostatectomy (*n* = 10) and females (*n* = 10) were compared. EVs were quantified using the EVQuant assay and EVs were analysed for CD9, CD63 and PSMA (FolH1) surface biomarkers using TR-FIA, and compared to clinical data (incl. urinary PSA (uPSA)).

**Results**: EV concentration and the CD63 markers were higher in urine from men that received digital rectal examination (DRE), indicating an increase of prostate EV after DRE. EV concentration and CD9/CD63 levels are lower in urine from men with PCa and no differences were found in EV PSMA using the TR-FIA. Higher EV counts in men without PCa could be caused by more prostate fluid in the urine due to an enlarged prostate in this control group. As a marker for prostate fluid in urine, uPSA was indeed higher in men without PCa. When marker levels are corrected for uPSA prostate fluid levels in urine, the EV concentration and CD9, CD63 and PSMA levels in the TR-FIA were elevated in the presence of PCa.

**Summary/conclusion**: EVQuant was able to quantify EVs in clinical urine samples, indicating that DRE before collection increases the number of EVs in urine. In this particular cohort of men with and without PCa, the dominant difference was uPSA, indicating higher concentration of prostate fluid in urine after DRE in men without PCa, likely caused by enlarged prostates in this group. The concentration of prostate fluid represented by uPSA fluctuates among men after DRE, affecting the numbers of prostate-derived EVs and is an important correction factor for future clinical assays. The ratio of EV numbers or TR-FIA signals divided by uPSA is higher in men with PCa and expected to be a more consistent indicator for the presence of PCa. Together, this indicates that both EVQuant and TR-FIA corrected for uPSA have diagnostic potential for PCa, but also shows the need for PCa-specific markers to enable direct detection of PCa-derived EVs in urine for clinical use.

**Funding**: Dutch Cancer Society, Alpe d’HuZes EMCR 2015-8022.

OT04.02

A novel strategy to liquid biopsy for early diagnosis of lethal prostate cancer employing palmitoyl-proteomics of extracellular vesicles

Javier Mariscal ^1^; Bo Zhou^1^; Peter De Hoff^2^; Desmond Pink^3^; Tatyana Vagner^4^; Mandana Zandian^5^; John D. Lewis^3^; Louise C. Laurent^2^; Wei Yang^1^; Andries Zijlstra^6^; Dolores Di Vizio^7^

^1^Cedars Sinai Medical Center, Los Angeles, USA; ^2^University of California, San Diego, San Diego, USA; ^3^University of Alberta, Edmonton, Canada; ^4^Department of Surgery, Cedars-Sinai Medical Center, Los Angeles, USA; ^5^Cedars-Sinai Medical Center, Los Angeles, USA; ^6^Department of Pathology, Microbiology and Immunology, Vanderbilt University Medical Center, Nashville, TN, USA; ^7^Departments of Surgery, Biomedical Sciences, and Pathology and Laboratory Medicine, Cedars-Sinai Medical Center, Los Angeles, USA

**Background**: Efficient risk assessment of prostate cancer patients is crucial for improved management. Tumour extracellular vesicles (EVs) have been shown to be carriers of abundant tumour material which can be used as evidence of disease. Recent discoveries highlight the complexity of the origin and function of EVs. A large subpopulation of EVs, large oncosomes (LO), has been identified so far as the only subtype uniquely secreted by migratory tumour cells, thus making an excellent platform for the discovery of robust biomarkers. In addition, LO cargo shows a significant enrichment in protein susceptible of S-palmitoylation. S-palmitoylation is a post-translational modification involved in vesicle trafficking and protein secretion and whose malfunction has been extensively reported in cancer.

**Methods**: Differential centrifugation, iodixanol gradient, tunable resistive pulse sensing, size exclusion chromatography, electrical sensing zone, micro-flow cytometry, mass spectrometry, palmitoyl-protein Identification.

**Results**: We refined the methods for the isolation and characterization of EVs to improve specificity and yield. Most of these vesicles have a size of 2–4 µm and are enriched in CK18 and HSPA5 markers in contrast to small EVs (80–120 nm), which are enriched in CD81 and Tsg101 markers. Our preliminary results show a strong association of LO cargo with tumour cell survival mediated by the protein ubiquitination and unfolded protein response pathways. Interestingly, there is a significant enrichment of proteins susceptible of palmitoylation and associated to pro-survival pathways frequently activated in tumour cells. Accordingly, some of these proteins have been previously proposed as biomarkers in a plethora of diseases including cancer but their palmitoylation status have not been considered.

**Summary/conclusion**: Assessment of palmitoylated biomarkers in LO represents a promising strategy in the liquid biopsy of lethal prostate cancer.

**Funding**: National Institutes of Health NIH R01 CA218526.

OT04.03

Development of a multiplex-to-single exosome analysis (MT-SEA) pipeline to characterize exosomes associated with tumour progression and responses to treatment

Joshua A. Welsh ^1^; Julia Kepley^2^; Alexis Barfield^2^; Jason Savage^2^; Milos Miljković^2^; André Görgens^3^; Thomas Waldmann^2^; Kevin Conlon^2^; Katherine McKinnon^2^; Samir El-Andaloussi^3^; Kevin Camphausen^2^; Veronica Galli^2^; Veffa Franchini^2^; Jay Berzofsky^2^; Jennifer Jones^4^

^1^Molecular Immunogenetics and Vaccine Research Section, Vaccine Branch, CCR, NCI, NIH, Bethesda, MD, USA; ^2^National Institutes of Health, Bethesda, USA; ^3^Clinical Research Center, Department for Laboratory Medicine, Karolinska Institutet, Stockholm, Sweden; ^4^National Cancer Institute, Bethesda, USA

**Background**: Extracellular vesicles (EVs) have potential as non-invasive biomarkers. We developed a first-in-class pipeline to characterize EV heterogeneity and provide high-sensitivity quantification of informative EVs in biofluids throughout treatment. By combining multiplex assays with high-resolution, single EV flow cytometric methods together into a mutiplex-to-single EV analysis (Mt-SEA) pipeline, we are able to characterize a broad range of EV subsets, while also measuring the concentration of specific EV populations. Exploratory studies presented here validate the Mt-SEA method by confirming strong correlations of liquid biopsy EV repertoires with tumour burden and responses to treatment. 

**Methods**: Plasma was obtained before and after treatment (*n* = 5 treatment courses) from Adult T-cell leukemia/lymphoma patients receiving palliative radiation. Multiplex EV capture beads were used with additional detection antibodies to identify 37 major EV subsets. General exosome and EV detection epitopes included CD63, CD9 and CD81. Tumour-specific epitopes for each patient included CD4, CD5 and CD25, based on available histo-/cyto-pathology results. High-resolution single EV analyses were performed with nanoFACS sorting and a prototype nanoFCM analyser.

**Results**: ATLL-derived EVs were detected in each pretreatment sample, with reduced specific ATLL-derived EV subsets concentrations at the end of treatment. Furthermore, ATLL-specific EVs from patients with progressive systemic disease prior to treatment were found to carry CD44 and other stemness-associated epitopes, consistent with increasing tumour aggressiveness. Responses to treatment that were clinically evident mirrored changes in the Mt-SEA EV profiles, and Mt-SEA identified new candidate prognostic EV profiles associated with clinical outcomes.

**Summary/conclusion**: Our exploratory study demonstrates that Mt-SEA provides unexpected insights into tumour biology, along with robust estimations of concentrations of EV subsets of interest. Detection of tumour-associated EVs and detection of EV repertoire changes during treatment paves the way to future evaluation of the Mt-SEA pipeline for personalized therapies in a wider range of tumour types.

OT04.04

Plasma extracellular vesicle imaging by high resolution flow cytometry in patients presenting for diagnostic EUS-guided pancreatic FNA

Terry K. Morgan ^1^; Kevin Judge^2^; Philip Streeter^1^

^1^OHSU, Portland, USA; ^2^BD Biosciences, San Jose, USA

**Background**: Our group employs high-resolution flow cytometry (HRFC) to visualize, quantitate, and sort cell- and size-specific extracellular vesicles (EVs) in patient plasma. Our objective in this pilot study was to test whether we could visualize and quantitate epithelial marker (EpCAM)-positive EVs, platelet EVs and total nano-sized events (50–1000 nm) in a prospective series of plasma samples collected from patients before diagnostic endoscopic ultrasound-guided fine needle aspiration (EUS-FNA) biopsies of clinically suspicious pancreatic lesions.

**Methods**: Blood samples were collected into EDTA tubes prior to EUS-FNA. Platelet poor plasma was banked at -80 °C. Samples were tested on a BD FacsAria Fusion using settings optimized for HRFC and Megamix polystyrene beads (100, 160, 200, 240, 300, 500 900 nm) to standardize sizing relative to log-scale side scatter (SSC-H). Platelet-related EVs present within all plasma samples served as internal positive controls. EpCAM-positive events were identified using anti-EpCAM-APC-Cy7 (Abcore, clone 323/A3). The volume of plasma tested for each case was normalized relative to the number of 200 nm FITC-conjugated beads spiked into 0.1 um filtered PBS (plasma samples diluted 1:100 in bead buffer). Outcomes were determined by FNA diagnoses, resection specimens and 1-year clinical follow-up. All samples were tested in triplicate. EpCAM+ EVs were FACs sorted and validated by electron microscopy and mir21 qRT-PCR.

**Results**: Outcomes were classified into ductal adenocarcinoma (*n* = 16), pancreatitis (*n* = 8) and IPMN (*n* = 3). Total nano-sized events/ml of plasma (mean 1 × 10^9^/ml) were not significantly different between adenocarcinoma, IPMN and pancreatitis. However, the number of EpCAM+ EVs/ml was significantly greater in cancer cases (2 × 10^5^) compared with pancreatitis (similar to PBS stained background~5 × 10^4^/ml) (*p* = 0.002). IPMN levels were not different than pancreatitis. Sorted EpCAM+ EVs were ~100 nm in size by cryoEM and enriched for mir21.

**Summary/conclusion**: Pancreatic cancer EVs in patient blood may be detected, counted and sorted by HRFC. There are significant differences in counts/ml in patients with cancer compared with pancreatitis.

**Funding**: Cancer Early Detection Advanced Research Center [CEDAR], Oregon Health & Science University; NICHD RFA-HD-16-037.

OT04.05

Exosomes and microvesicles contain more tumour RNA than platelets

Kay Brinkmann ^1^; Lisa Meyer^1^; Anne Krug^1^; Daniel Enderle^1^; Carola Berking^2^; Mikkel Noerholm^1^; Johan Skog^3^

^1^Exosome Diagnostics GmbH, Martinsried, Germany; ^2^Department of Dermatology and Allergology, University Hospital of Munich (LMU), Munich, Germany; ^3^Exosome Diagnostics Inc., Waltham, USA

**Background**: Recently, the notion of tumour-educated platelets has emerged as a novel source of tumour RNA biomarkers. We sought to confirm the suitability of the platelet blood fraction for liquid biopsy approaches. Since publications have claimed that tumour RNA and other tumour-derived material is transferred from tumour cells to the platelets and that tumor-derived transcripts can be detected in platelets, we chose to focus on RNA carrying a mutation as being of bona fide tumour origin.

**Methods**: Prospective blood samples from a cohort of 10 melanoma patients with tissue-confirmed BRAF V600E mutation were collected after informed consent, according to an ethics committee-approved protocol. Each specimen was processed using three different protocols in parallel isolating exosomes and other extracellular vesicles (EVs), platelet poor plasma (PPP) and platelets, respectively. The EV fraction was prepared using a commercial protocol for spin column-based isolation of extracellular vesicles, followed by purification of the RNA, whereas platelets and PPP were processed by centrifugation protocols reported in literature, followed by a similar RNA purification. The RNA from each fraction was analysed by a highly sensitive ARMS RT-qPCR with a wild-type blocker for detection of BRAF V600E mutations. This assay enables the quantification of the mutant allele fraction (%MAF) of BRAF V600E down to 0.01% in a background of up to 100,000 wild-type copies.

**Results**: Comparing all three fractions, only the EVs contained detectable BRAF V600E in 10 out of 10 patients and showed a substantially higher %MAF of BRAF V600E than the other two fractions. In three patients with the overall lowest mutant signal, BRAF V600E was only detectable in the EV fraction, but not in platelets or PPP. The platelet fraction from all 10 patients contained too high amounts of wild-type BRAF signal to accurately quantify any mutation signal above the elevated background noise.

**Summary/conclusion**: Our observations suggest that if tumour RNA is indeed transferred to platelets, this phenomenon occurs below detection limit, since even a very sensitive qPCR assay did not allow for a reliable detection of BRAF V600E in the platelet fraction. In contrast, the EV fractions from the same patients allowed for detection of BRAF V600E in all 10 specimens.

OT04.06

Clinical relevance of the defining the most abundant fraction of mutated oncogenic DNA and RNA among different EV subtypes

Jennifer Klump^1^; Ulrike Phillipp^2^; Marie Follo^2^; Nikolas von Bubnoff^2^; Irina Nazarenko ^1^

^1^Institute for Infection Prevention and Hospital Epidemiology; Medical Center - University of Freiburg, Faculty of Medicine, University of Freiburg, Freiburg, Germany; ^2^Department of Medicine I, Medical Center - University of Freiburg, Faculty of Medicine, University of Freiburg, Freiburg, Germany

**Background**: The need to develop better approaches allowing comprehensive molecular characterization of a progressing tumour has led to development of the “Liquid Biopsy” concept, based on the application of body fluids as a biomarker source. In view of the attractiveness of both free circulating fractions and different EV subtypes, an important biological and clinical question arises: in which of the components the majority of mutated DNA or RNA molecules are located?

**Methods**: We established a robust method allowing separation of different EV subtypes and fc fractions from one blood sample. For method validation, we tested for presence of the BRAFV600E mutation in EVs and fc of the HT29 colorectal carcinoma cells. To address clinical relevance of tumour RNA and DNA, we performed a pilot clinical study, testing patients with advanced stage melanoma, harbouring the BRAFV600E mutation and patients with systemic aggressive mastocytosis harbouring the cKITD816V mutation, and determined copy numbers of wild-type and mutated oncogenes in the fc and EV fractions using digital PCR.

**Results**: Both EV and fc fractions contained DNA and RNA. However, significantly higher amounts of the double-stranded DNA was located in EV. In contrast to that, comparative amounts of total RNA were determined in EV and fc fractions. Importantly, the portion of mutated oncogenes in different subtypes of EV and fc fractions was strongly dependent on the tumour type and stage. Thus, in patients with aggressive form of the diseases, such as stage IV melanoma and systemic mastocytosis, over 10-fold more wild-type and mutant BRAF and cKIT copies were detected in the fc as compared to the EV fractions. In contrast, mutated RNA transcripts were mostly located in the EV fractions.

**Summary/conclusion**: Our results show that different tumour types at different stages may contain varying portions of potential biomarkers in the free circulating fraction and in different EV subtypes. This finding indicates high relevance of a comparative analysis of different blood compartments prior to the choice of the most appropriate source and technique for the liquid biopsy-based diagnostic.

Different EV types transport DNA and RNA *in vitro* as well as *in vivo*. More DNA of mutant alleles BRAFV600E and cKITD816V is present in the fc fractions than in the EVs of patients with advanced melanoma and mastocytosis.

Symposium Session 5 – EVs in Stem Cell and Cancer Growth Chairs: Lorraine O’Driscoll; Peter Quesenberry Location: Room 5 13:30–15:00

OT05.01

Onogenic EGFR/Src signalling modulates the functionality of exosomal β4 integrin for tumour malignancy and organotropic metastasis

Yu-Ling Tai^1^; Chia-Yu Yang^2^; Ko-Chien Chen^3^; Tang-Long Shen ^4^

^1^Dept of Plant Pathology and Microbiology, National Taiwan University, Taipei, Taiwan (Republic of China); ^2^National Taiwan University, Taipei, Taiwan (Republic of China); ^3^Department of Life Sciences, National Taiwan University, Taipei, Taiwan (Republic of China); ^4^Department of Plant Pathology and Microbiology, National Taiwan University, Taipei, Taiwan (Republic of China)

**Background**: Metastasis is the most devastating outcome of cancer, accounting for the major cancer death. Although the expression of β4 integrin in both tumour cells and tumour-derived exosomes is crucial for malignant development and organotropic metastasis of cancer, the regulatory mechanism of β4 integrin expression in tumour cells and tumour-derived exosomes is still unclear.

**Results**: Here, we revealed that N-glycosylation of β4 integrin is mandatory for β4 integrin loading into exosomes. Mechanistically, oncogenic EGFR/Src signal is required for N-glycosylation of β4 integrin. Authentically, EGFR/Src signal-mediated N-glycosylation of tumour-derived exosomes facilitates tumour-derived exosomes uptake by normal human lung fibroblast cells, which in turn promotes the formation of cancer-associated fibroblasts. Moreover, cancer-associated fibroblasts contribute to the cancer malignant development.

**Summary/conclusion**: Our study uncovers a function of N-glycosylation of β4 integrin in tumour-derived exosomes, which is important for malignant development.

**Funding**: Ministry of Science and Technology, Taiwan.

OT05.02

The role of large oncosomes in leukaemia

Valentina R. Minciacchi; Rahul Kumar; Parimala Sonika Godavarthy; Christina Karantanou; Daniela S. Krause

Georg-Speyer Haus, Institute for Tumor Biology and Experimental Therapy, Frankfurt, Germany

**Background**: In the last years, the tumour supportive bone marrow microenvironment (BMM) has been suggested to be involved in several aspects of leukemia including drug resistance, leukemia cell survival and disease progression. Leukemia cells, similar to what has been described for other tumours, might establish a bidirectional communication with the cells in the BMM leading to the establishment of a permissive environment. Recent data have proved the contribution of leukemia-derived nano-sized extracellular vesicles (EVs) to the education of the BMM. Large oncosomes (LO) are atypically large EVs shed by aggressive, highly motile tumour cells that have acquired an amoeboid phenotype. Due to recent data supporting a role of LO in conditioning of the tumour microenvironment and due to the high deformability of leukemia cells which may support LO shedding, LO may provide an attractive means of communication between leukemia cells and the BMM.

**Methods**: Leukemia mouse models; differential and density gradient centrifugation; flow cytometry analysis and sorting; western blot; fluorescent microscope imaging.

**Results**: We adapted and optimized a protocol to isolate leukemia-specific LO from the bone marrow of mice which were suffering from chronic or acute myeloid leukemia or B-cell acute lymphoblastic leukemia, induced by the retroviral transduction/transplantation model. This was possible due to GFP and oncogene expression in leukemia cells, which could be detected in isolated LO. We further assessed the interaction of leukemia-specific LO with primary niche cells obtained from the BMM and we observed a dose-dependent uptake of LO. However, there seemed to be differences not only between target cell types but among LO isolated from different leukemias.

**Summary/conclusion**: In this study, we were able to successfully isolate leukemia-derived LO from the bone marrow of mice with leukemia. Moreover, the differences observed in the interaction between LO from different leukemia subtypes with the niche cells suggest specific activity of the LO according to the original leukemia subtype.

**Funding**: EMBO long term fellowship to V.R.M.

OT05.03

Oral administration of bovine milk-derived extracellular vesicles reduce primary tumour burden but accelerate cancer metastases

Suresh Mathivananan

La Trobe University, Melbourne, Australia

**Background**: It has been proposed that extracellular vesicles (EVs) from the diet can be absorbed by the intestinal tract of the consuming organism, be bioavailable in various organs and exert phenotypic changes. However, the concept was challenged by few well-controlled studies emphasizing the instability of nucleic acids which ultimately succumb to the membrane barriers and nucleases of the mammalian gastrointestinal tract. Furthermore, the observations were often criticised as diet-responsive endogenous RNAs or artefacts due to non-adherence of rigorous procedures.

**Methods**: EVs were isolated from raw and commercial milk by ultracentrifugation and OptiPrep density gradient. Quantitative proteomics and RNA-seq of EVs. DIR labelled EVs biodistribution was monitored by IVIS imaging. Quantitative proteomics of mouse liver Tissues. EVs were orally administered to various models including xenograft, cachexic, E-cadherin biosenor and metastatic mice models.

**Results**: Here, we orally administered bovine milk-derived EVs to mice and demonstrated that milk-derived EVs can survive the harsh degrading conditions of the gut and subsequently be detected in multiple organs. Interestingly, oral administration of milk-derived EVs reduced the primary tumour burden in various cancer models and attenuated cancer cachexia. Intriguingly, in spite of the reduction in primary tumour growth, milk-derived exosomes accelerated metastasis in breast and pancreatic cancer mice models. Timing of exosome administration was critical as oral administration after resection of the primary tumour reversed the pro-metastatic effects of milk-derived exosomes in breast cancer.

**Summary/conclusion**: Taken together, our study provides novel context-based and opposing role of milk-derived exosomes as metastasis inducers and as metastasis blocker.

OT05.04

3D culture of cancer cells in a polysaccharide-based hydrogel drastically alters the protein and RNA cargo of EVs

Christopher Millan ^1^; Daniel Eberli^1^; Flurina Clement^2^

^1^University of Zurich Hospital, Schlieren, Switzerland; ^2^ETH Zurich, Zurich, Switzerland

**Background**: Previously, we have introduced a 3D culture platform based on the polysaccharides chitosan and alginate that confers an altered morphology and phenotype to encapsulated cells. Compared to the same cells cultured on tissue culture plastic (2D), cancer cells cultured in 3D exhibit enrichment of tumour-associated antigens and resistance to treatment by conventional chemotherapeutics, hallmarks of advanced cancers. Here, we examined how the encapsulation of cells in 3D affects their behaviour related to EV production looking at both cancerous and healthy cell types.

**Methods**: Cells were cultured in either 2D or 3D and EVs were isolated from supernatants via size exclusion chromatography (SEC). EVs were then characterized by Bradford assay, TEM, nanoparticle tracking analysis (NTA), LC-MS/MS, and next-generation sequencing (NextSeq).

**Results**: All cell types evaluated exhibited an increase of 2–5× in production of EVs when cultured in 3D. This difference was not attributed to alterations of EV sizes as NTA and TEM results indicated similar EV diameters (means of 130 ± 20 nm) independent of cell type or culture condition. However, striking differences were observed in proteomics and genomics data. Culture of cells in 3D resulted in the expression of 300–400 extra proteins that were not found in EVs of the same cells cultured in 2D – a trend consistent for each cell type tested. Approximately 10% of these “extra proteins” have never before been reported as EV cargo to our knowledge (e.g. in ExoCarta). Similar dramatically altered expression at the RNA level was observed in NextSeq results.

**Summary/conclusion**: These results indicate that the *in vitro* model used to produce EVs for downstream analysis plays a profound role in the characteristics of vesicles obtained. In next steps, we plan to validate certain proteins/RNAs uncovered by 3D culture as potential biomarkers in a small, retrospective clinical study involving a cohort of 25 prostate cancer patients with varying degrees of tumour burden.

**Funding**: Swiss Commission for Technology and Innovation grant no. 26691.1 PFLS-LS.

OT05.05

lncRNA 19 from human adipose stem cells derived exosomes promote the regeneration of hepatocytes by up-regulating HGF/c-Met pathway and significantly improve survival of rats with acute liver failure

Yinpeng Jin; Hongchao Li; Xi Wang; Qingchun Fu

Shanghai Public Health Clinical Center, Fudan University, Shanghai, China (People’s Republic)

**Background**: Stem cells can promote the regeneration of damaged tissue through paracrine effect, but the mechanism is still unclear. We collected exosomes of human adipose stem cells (hASCs), and treated D-gal-induced rat model of acute liver failure with ASC, ASC-derived exosomes and ASC lysate, respectively, and compare their efficacy.

**Methods**: (1) To obtain ASC from healthy human abdominal subcutaneous fat tissues through collagenase I digestion and purify the cells through adherent culture. (2) Collect exosome by ultra filtration concentration centrifugation, and evaluate ingredients including proteins and RNAs in the exosome via protein mass spectrometry and gene sequencing. (3) Treat the acute liver failure rats with ASC, low-concentration lysate solution, high-concentration lysate solution, low-concentration exosome and high-concentration exosome through vena femoralis injection. Observe the survival of the rats and evaluate the rats and human RNA expression differentiations in the rats’ liver tissues in high-concentration exosome group and PBS-controlled group. (4) Analyse the key genes that function in the treatment procedures of acute liver failure with ASC exosome by bioinformatics methods.

**Results**: (1) The survival of the rats in ASC group, low- or high-concentration lysis solution group, low- or high-concentration exosome group were 37.5%, 25%, 50%, 62.5% and 100%, respectively, whereas in PBS-controlled group, the survival of the rats was only 27.3%. (2) The expression of hepatocyte growth factor and c-Met in liver tissue were both up-regulated in exosomes-treated group. Second-generation RNA sequencing analysis showed that human lncRNA H19 was significantly increased in rats’ liver in exosomes-treated group. Interestingly, the survival rate of high concentration of exosomes-treated group decreased to 40% when lncRNA H19 was knockdown, suggesting that human lncRNA H19 released from hASCs-derived exosomes can promote the regeneration of hepatocytes by up-regulating HGF/c-Met pathway, thereby improving the survival rate of rats.

**Summary/conclusion**: High-concentration ASC exosomes have good curative effect for acute liver failure rats and could improve their survival. lncRNA H19 is probably the key genes that function in the treatment procedures for acute liver failure.

OT05.06

Increased haematopoietic extracellular RNAs and vesicles in the lung during allergic airway responses

Heather H. Pua ^1^; Hannah H. Happ^2^; Ni-Ting Chiou^2^; Carleigh J. Gray^1^; Laura E. Hesse^1^; K. Mark Ansel^2^

^1^Department of Pathology, Microbiology and Immunology, Vanderbilt University Medical Center, Nashville, USA; ^2^Department of Microbiology and Immunology, University of California San Francisco, San Francisco, USA

**Background**: Extracellular microRNAs (ex-miRNAs) are present in body fluids. The goal of this study was to characterize the composition, forms and cellular sources of ex-miRNAs in bronchoalveolar lavage fluid (BALF) both at steady state and after the induction of allergic airway inflammation.

**Methods**: miRNA sequencing was performed comparing ex-miRNAs in BALF and serum as well as cellular miRNAs from lung epithelial brushings and haematopoietic cell rich pellets from bronchial washings. Fluids and cells were isolated from control mice and mice challenged with allergen in lung. Serial ultracentrifugation followed by qPCR analysis was used to test whether miRNAs were present in vesicle-enriched fractions. Nanoparticle tracking, electron microscopy and cell-type specific labelling of membranes *in vivo* followed by vesicle flow cytometry were used to characterize BALF vesicles and their cellular sources.

**Results**: Ex-miRNAs were abundant in BALF and had a composition that was unique from serum. The ex-miRNA profile of BALF correlated most highly with the miRNA content of the airway lining epithelium, the most prevalent cell type in the local tissue environment. Extracellular vesicles were present within BALF, and ex-miRNAs were contained within vesicle-enriched fractions from this fluid. Using cell-type specific membrane tagging and single vesicle flow cytometry, we identified that 80% of fluorescence positive vesicles were of epithelial origin and 10–15% were of haematopoietic origin in the lungs of control mice. After the induction of allergic type inflammation in the lungs, there was twofold increase in the number of haematopoietic cell derived vesicles as well as an increase in miRNAs selectively expressed by haematopoietic cells including miR-223 and miR-142a.

**Summary/Conclusion**: Together these results provide evidence that the infiltration of immune cells into tissues during inflammatory responses leads to detectable changes in the composition of local body fluids. Determining the cellular sources and composition of extracellular vesicles and RNA *in vivo* are essential first steps in understanding their function in pathologic processes including allergic asthma.

**Funding**: This work was supported by NIH U19CA179512 (KMA), NIH K08AI116949 (HHP) and the Department of Pathology, Microbiology and Immunology at Vanderbilt University Medical Center (HHP).

Symposium Session 6 – EV Analysis by Microfluidics and Flow Cytometry Chairs: Jennifer Jones; Victor Ugaz Location: Room 6 13:30–15:00

OT06.01

Microfluidic device provides a solution for a time-course analysis of EV secretion

Takanori Ichiki ^1^; Takanori Akagi^2^

^1^Department of Materials Engineering, School of Engineering, The University of Tokyo, Bunkyo, Japan; ^2^University of Tokyo, Bunkyo, Japan

**Background**: The function and state of cells change dynamically in several hours. Time-course analysis of extracellular vesicles (EVs) is required to understand the mechanism of the EV secretion. However, most researchers analyse EVs collected from a large amount of culture supernatants after cultivated for several tens of hours. Here, we present the successful evaluation of EVs collected from a small sample volume using microfluidic devices that enable a time-course analysis of EVs.

**Methods**: A human acute leukemia HL60 cells were used as a model sample. After HL60 cells of 1 × 10^7^ particles were cultivated with an EV-depleted medium for 2 h, culture supernatants were centrifuged at 300×g for 10 min, at 2000× g for 20 min and at 10,000× g for 100 min. The clarified supernatant was further centrifuged at 100,000× g for 200 min. Vesicles in resulting pellet were suspended and diluted to approximately 10 times by 10 mM HEPES solution.

**Results**: The EV sample was introduced into a microchannel. Particle electrophoresis was performed immediately after Brownian motion analysis for obtaining both diameter and zeta potential of each EV. The mean with standard deviation of diameter and zeta potential of EVs were 127 with 77 nm and −12.5 with 5.4 mV, respectively.

**Summary/conclusion**: This methodology requires only 1 × 10^7^ particles per measurement and hence, nables a time-course characterization of EV population at 10-min intervals, in principle.

**Funding**: This research was supported by the Center of Innovation Program (COI STREAM), by the program for creating start-ups from advanced research and technology (START Program) from Japan Science and Technology Agency (JST), and by JSPS KAKENHI Grant Number 16K04915 (to TA).

OT06.02

Rapid quantification and characterization of individual urinary EVs using a microfluidic assay

Serhii Mytnyk ^1^; Guido W. Jenster^2^; Thomas A. Hartjes^2^; Martin E. van Royen^3^; Volkert van Steijn^1^

^1^Delft University of Technology, Delft, The Netherlands, Delft, The Netherlands; ^2^Erasmus Medical Center, Rotterdam, The Netherlands; ^3^Department of Pathology, Erasmus Optical Imaging Centre, Erasmus MC, Rotterdam, The Netherlands

**Background**: To optimally use the potential of extracellular vesicles (EVs) as biomarkers for various diseases, there is a need for fast, inexpensive and accurate methods for EV quantification and characterization in clinical samples. Our aim is to develop a simple assay that requires limited resources and expertise, enabling its wide dissemination across the community. Here, we present an epifluorescence microscopy-based microfluidic assay for simultaneous determination of the concentration and the size distribution of urinary EVs in minimally processed clinical samples.

**Methods**: EVs were labelled using general fluorescent lipid membrane stains and/or specific immunofluorescent antibodies. After adding fluorescent labels and 5-min incubation, the samples were injected into PDMS microchannels without any further processing. By employing shallow channels with a depth comparable to the focal depth of the microscope, the measurement volume was precisely defined allowing us to determine EV concentration. At the same time, we determined their size distribution by tracking 2D diffusion of individual EVs, without the need of using advanced equipment such as a confocal microscope.

**Results**: We have successfully validated our approach by applying it to suspensions of fluorescent nanoparticles of defined sizes and known concentrations. When applied to EVs, the developed method allowed accurate concentration measurements over a wide range (10^6^–10^9^ EV/ml), as confirmed by comparison with data obtained from nanosight tracking analysis. Moreover, our microfluidic assay provides a quick and accurate diameter estimate of individual urinary EVs.

**Summary/conclusion**: We have developed an assay for EV concentration and size measurement using user-friendly methodology eliminating the need for complex equipment, significantly decreasing analysis times and making this method a promising tool in diagnostics in a clinical setting. Extending this approach to immunofluorescently labelled EVs enables detection of subpopulations of EVs and further development of the microfluidic assay as a non-invasive tool for EV analysis in biofluids in health and disease.

**Funding**: IMMPROVE project is funded by Dutch Cancer Society (KWF) in collaboration with Alpe d’HuZes.

OT06.03

Exosome nanoarray for the next generation single-exosome analysis platform

Kyohei Okubo; Hiromi Kuramochi; Shusuke Yokota; Akiko Iwaya; Rei Okamura; Takanori Ichiki

Department of Materials Engineering, School of Engineering, The University of Tokyo, Bunkyo, Japan

**Background**: Exosomes, one of extracellular vehicles (EVs), have recently attracted much attention as promising biomarkers for an early-stage diagnostic test. Exosomes are heterogeneous in size ranging from 30 to 150 nm in diameter. Quantitative evaluation of single exosome is a challenging issue because the coexistence of several other kinds of EVs in biofluids affects the accurate analysis of exosomes, therefore establishing a method to isolate exosomes is of great importance. Here, we propose an assay platform called exosome array, in which exosomes are separately immobilized and analysed in the similar manner as DNA array.

**Methods**: To attach exosomes on the Si substrate, polyethyleneglycol (PEG)-lipid modified nanodot-array is formed on the substrate by electron beam (EB) lithography and selective chemical modification using aqueous 3-aminopropyltriethoxysilane solution, followed by lift-off process. PEG-lipid derivative has an oleyl group at its end, at which exosomes are attached via hydrophobic interaction. As for exosome suspension, after cultivation with a serum-free medium for 48 h, culture supernatants of leukemia HL60 cells were centrifuged (300× g, 10 min; 2000× g, 20 min; 10,000× g, 100 min), then the clarified supernatant was further centrifuged at 100,000× g, 200 min.

**Results**: A microfluidic system was accompanied with the nanoarray to demonstrate the immobilization of individual exosome. A flow of exosome suspension was exposed over the nanoarray, and the suspension was incubated while a micro-peristatic pump in the microfluidic device generated continuous flow. After the chip was uninstalled out of the device, the topography of the nanodot array was observed using an atomic force microscope, revealing single exosome was successfully immobilized on the dot. Larger exosomes showed greater deformation that the calculated values, indicating the presence of strong hydrophobic interactions between the PEG-lipid and exosomes.

**Summary/conclusion**: Exosome nanoarray obtained by a combination of EB lithography, amino-modification and lift-off process was demonstrated to show the possibility of immobilizing and analyzing individual exosomes.

OT06.04

Hollow organosilica beads as reference particles for optical detection of extracellular vesicles

Zoltan Varga ^1^; Edwin van der Pol^2^; Marcell Palmai^1^; Raul Garcia-Diez^3^; Christian Gollwitzer^3^; Michael Krumrey^3^; Jean-Luc Fraikin^4^; Najat Hajji^5^; Ton G. van Leeuwen^6^; Rienk Nieuwland^7^

^1^Biological Nanochemistry Research Group, Institute of Materials and Environmental Chemistry, Research Centre for Natural Sciences, Hungarian Academy of Sciences, Budapest, Hungary, Budapest, Hungary; ^2^Biomedical Engineering & Physics, Academic Medical Center, University of Amsterdam, Amsterdam, The Netherlands, Amsterdam, The Netherlands; ^3^Physikalisch-Technische Bundesanstalt (PTB), Berlin, Germany; ^4^Spectradyne LLC, Torrance, USA; ^5^Laboratory of Experimental Clinical Chemistry, Academic Medical Centre of the University of Amsterdam, Amsterdam, The Netherlands; ^6^Department of Biomedical Engineering and Physics, and Vesicle Observation Center, Academic Medical Centre of the University of Amsterdam, Amsterdam, The Netherlands; ^7^Laboratory of Experimental Clinical Chemistry, and Vesicle Observation Center, Academic Medical Center, University of Amsterdam, Amsterdam, The Netherlands, Amsterdam, The Netherlands

**Background**: The concentration of extracellular vesicles (EVs) in body fluids is a promising biomarker for disease, and flow cytometry remains the clinically most applicable method for measuring it. To compare concentration measurements of EVs between flow cytometers, solid polystyrene reference beads are used. However, these polystyrene beads lead to false size determination of EVs due to the mismatch in refractive index between the beads and EVs. The objective of this study is to prepare, characterize and test hollow organosilica beads (HOBs) with nominal diameters with 200 nm (HOB200) and 400 nm (HOB400) as reference beads to set EV size gates in flow cytometry investigations.

**Methods**: HOBs were prepared by a hard template sol-gel method and extensively characterized for morphology, size distribution and colloidal stability. The applicability of HOBs as reference particles was investigated by flow cytometry using HOBs and platelet-derived EVs.

**Results**: The HOBs proved monodisperse with homogeneous shell thickness with mean diameters of (189 ± 2) nm and (374 ± 10) nm for HOB200 and HOB400, respectively, with a polydispersity below 15%. Two-angle light scattering measurements proved that the scattering intensity of HOBs overlaps with the scattering intensity expected from EVs. To demonstrate that HOBs can be used independent of the light scattering collection angles of a flow cytometer, we determined the concentration of platelet-derived EVs using the FSC or SSC detector within size gates set by HOBs. The percentage difference in the gated concentration relative to the mean concentration is smallest for the gates set by HOBs compared to solid beads, suggesting that HOBs outperform solid beads to standardize EV flow cytometry.

**Summary/conclusion**: Because HOBs resemble the structure and the light scattering properties of EVs, HOBs can be used to set size gates in nanometers independent from the optical configuration of a flow cytometer, thus making HOBs an ideal reference material which may facilitate the comparison of EV measurements between instruments and institutes.

**Funding**: This work was supported by the National Research, Development and Innovation Office (Hungary) under grant numbers PD 121326 and NVKP_16-1-2016-0007. Part of this work was supported by the Cancer-ID program and the MEMPHISII program of The Netherlands Technology Foundation STW.

OT06.05

Bioengineered nanovesicles as extracellular vesicle-mimetics: a novel standard for extracellular vesicle analysis

Estefanía Lozano-Andrés ^1^; Sten F.H.M. Libregts^2^; Félix Royo^3^; Sara Morales-López^4^; Soraya López-Martín^4^; Mar Valés^5^; Hugh T. Reyburn^5^; Marca H.M. Wauben^2^; Juan M. Falcón-Pérez^3^; María Yáñez-Mó^6^

^1^Molecular Biology Department, Autonomous University of Madrid (UAM), Madrid, Spain; ^2^Department of Biochemistry and Cell Biology Faculty of Veterinary Medicine, Utrecht University, Utrecht, The Netherlands; ^3^CIC bioGUNE, CIBERehd, Bizkaia Science and Technology Park, Derio, Bizkaia, Spain, Derio, Spain; ^4^Molecular Biology Center Severo Ochoa (CBM), Instituto de Investigación Sanitaria Princesa (IIS-IP), Madrid, Spain; ^5^Immunology and Oncology Department, Biotechnology National Center (CNB-CSIC), Madrid, Spain; ^6^Departamento de Biología Molecular. UAM, Madrid, Spain

**Background**: Adequate detection of extracellular vesicles (EVs) is difficult due to their size, low refractive index and polydispersity, as well as the lack of proper standards or reference materials for equipment setup. Our aim was to construct suitable standards for EV analyses by modifying synthetic nanovesicles (niosomes) with the antigenic regions of tetraspanins, classical EV markers.

**Methods**: Large extracellular loops (LELs) of human tetraspanins CD9, CD63 and CD81, tagged at both ends with BirA-biotin ligase target sequences, were cloned into pGEX4T2 expression vectors and co-transformed with a BirA expression vector into a protease-deficient *E. coli* strain. After culture amplification, GST fusion proteins were purified by affinity chromatography and released from GST using thrombin.

Biotinylated tetraspanin recombinant LELs were then incubated with fluorescent or non-fluorescent (strept)avidin-coated niosomes, and unbound LEL peptide was removed by size-exclusion chromatography. Collected fractions were subsequently analysed by dot blot, western blot, nanoparticle tracking analysis (NTA), transmission electron microscopy (TEM) and flow cytometry.

**Results**: NTA of decorated niosome-containing fractions confirmed the presence of nanovesicles with a size between 100 and 200 nm. Bead-assisted flow cytometry using specific antibodies verified the presence of recombinant tetraspanins on niosomes within samples. Cryo-TEM revealed the presence of vesicles with a heterogeneous morphology, whereas antibody immunogold labelling confirmed the presence of LEL tetraspanins on the surface of niosomes. Finally, using high-resolution flow cytometry, expression of recombinant tetraspanins was further confirmed at the single niosome-level.

**Summary/conclusion**: We here describe the production of tetraspanin-decorated nanovesicles. Using various isolation and detection techniques, we show that these nanovesicles have similar biophysical properties to EVs and are suited for antibody-staining techniques, making these bioengineered nanovesicles an effective standard and reference material for various EV-detection techniques.

**Funding**: Grants from Fundación Ramón Areces and Ministerio de Economía y Competitividad (BFU2014-55478-R, REDIEX. SAF2015-71231-REDT). E.L. was supported by the ESF, GEIVEX Mobility and UAM STS fellowships.

OT06.06

Isolation of microvesicles and exosomes by fluorescence-triggered FACS

Celine Gounou^1^; Sisareuth Tan^1^; Nicolas Arraud^2^; Alain R. Brisson ^3^

^1^UMR-5248 CNRS - of Bordeaux, Pessac, France; ^2^Laboratoire de Cytométrie en Flux, Hôpitaux Universitaires de Genève, Geneva, Switzerland; ^3^University of Bordeaux, Pessac, France

**Background**: The isolation of extracellular vesicles (EV) constitutes a major challenge in the EV field, mainly due to the heterogeneity of EV suspensions and the difficulty of EV detection. We showed earlier that the detection of EVs was significantly improved by fluorescence-triggered flow cytometry (FL-FCM) as compared to conventional light-scatter triggering (1).

The objective of this study was two-fold: (1) to sort selected EV populations by FL FACS and (2) to compare the sorting efficiency of the two main EV populations, namely large (100 nm to 1 µm) microvesicles (MV) derived from plasma membranes and small (50–100 nm) exosomes derived from multivesicular bodies.

**Methods**: MV were obtained by hypotonic lysis of erythrocytes, while EX derived from reticulocytes were obtained from sickle cell disease plasma. EV sorting was performed with a FACS-Aria-II (Becton Dickinson) using PE-labelled anti-CD235a and anti-CD71 IgGs and Cy5-annexin5 (Anx5). In parallel to FCM, immuno-cryo-electron microscopy was used to image EV before and after sorting (2).

**Results**: Before sorting, EV were first characterized by FCM and immuno-cryoEM. Erythrocyte-derived MV consist of 100–300 nm vesicles that expose both CD235a and phosphatidylserine, while reticulocyte-derived EX consist of 50–100 nm vesicles that express the transferrin receptor CD71 (3). The conditions of sorting were optimized for MV, using FL-FCM based either on PE-CD235a-IgG or on Cy5-Anx5 signal, and for EX using FL-FCM on PE-CD71-IgG. The sorted MV and EX suspensions were re-analysed by FCM and by immuno-cryoEM. A sorting yield was calculated, equal to the ratio of EV concentrations detected by FL-FCM before and after sorting. Sorting yields of 20–40% were found for CD235a+ and PS+ MV and 30% for CD71+ EX, respectively. Both EV suspensions were of high purity, as shown by immuno-cryoEM.

**Summary/conclusion**: We show here that fluorescence-triggered FACS is a powerful and general method for isolating EV, and for the first time, that EV as small as exosomes can be sorted with high efficiency using a standard FACS equipment. The isolation of selected EV populations constitutes a major step towards the determination of their omic composition and the elucidation of their specific function.

1- Arraud et al. Cytometry A 2016 9:184.

2- Arraud et al. J Thromb Haemost. 2014;12:614.

3- Harding et al. J Cell Biol. 1983;97:329.

Oral with Poster Session 1 Chair: Hernando del Portillo Location: Auditorium 15:30–16:30

OWP1.01 = PF04.01

Immunomodulatory function of human mesenchymal stromal cells-derived extracellular vesicles on Type-I interferon response in human plasmacytoid dendritic cells and lupus murine pDCs

Lin Kui ^1^; Godfrey CF Chan^2^; Pamela PW Lee^2^

^1^Department of Paediatrics and Adolescent Medicine, The University of Hong Kong, Hong Kong, Hong Kong; ^2^Department of Paediatrics & Adolescent Medicine, LKS Faculty of Medicine, The University of Hong Kong, Hong Kong, Hong Kong

**Background**: Immunoregulatory effect of mesenchymal stem cell (MSC) is attributed to extracellular vesicles (EV) secretion. Given its effectiveness in pre-clinical studies of autoimmune disease, no one has examined its effect on SLE pathogenesis, signified by excessive type-I IFN production by pDCs and animal models. We found that TSG-6, a key anti-inflammatory protein secreted by activated MSC, downregulates TLR7 and TLR9 activation in human pDC. Herein, we investigate the effect of MSC and MSC-EVs on regulating cytokines production in pDCs, and whether such effect is mediated by TSG-6.

**Methods**: htMSC (immortalized human MSCs) was cultured in CDPF medium for 48 h. EVs were isolated by ultracentrifugation at 100,000× g, 3 h, at 4°C and were characterized by transmission electron microscopy, nanosight tracking analysis, and western blot. Comparison of immunosuppressive function between htMSC-EV and TSG-6 knockdown htMSC on TLR9-mediated cytokine production in pDC was determined by GEN2.2, a human pDC cell-line, following activation by CpG-A, and analysis by qPCR and ELISA. Finally, we compared the IFN-α and TNF-α intracellular expression in pDCs of htMSC-EV-treated NZB W/F1 mice with PBS control group.

**Results**: Upon activation of TLR9 by CpG-A, IL-1ß, TNF-α and IFN-α transcription was upregulated in GEN2.2. Such response was reduced when CpG-A-primed GEN2.2 were co-cultured with htMSC. Knockdown of TSG-6 in htMSC dampened its capacity to suppress IL-1ß, TNF-α, IFN-α and IRF7 transcription in GEN2.2. To find out whether MSC exert its immunosuppressive effect by means of EV, we isolated EVs from hTERT MSCs and found htMSC-EV-contained TSG-6 protein. Coculture of htMSC-EV with CpG-A-primed GEN2.2 resulted in downregulation of IFN-α transcription and protein expression, mediated via reduction in total and phospho-IRF7. htMSC-EV treatment of NZB/W F1 mice resulted in augmentation of splenic and bone marrow pDCs, with a reduction in bone marrow pDCs TNF-α and splenic pDC IFN-α expression.

**Summary/conclusion**: For the first time, we showed that MSC downregulated TLR9 activation in human pDCs, in a TSG-6 dependent manner. Furthermore, htMSC-EV contain TSG-6 and suppress IFN-α response in CpG-A-activated pDCs by reducing total and phospho-IRF7. Finally, htMSC-EV treatment modulated pDCs in NZB/W F1 mice.

**Funding**: Edward and Yolanda Wong Foundation.

OWP1.02 = PT09.01

Role of CD44 in therapeutic mesenchymal stem cell-derived extracellular vesicles for joint diseases

Enrico Ragni ^1^; Carlotta Perucca Orfei^1^; Gaia Lugano^1^; Marco Viganò^1^; Alessandra Colombini^1^; Paola De Luca^1^; Daniele Zacchetti^2^; Valentina Bollati^3^; Laura de Girolamo^1^

^1^Orthopaedic Biotechnology Lab, IRCCS Galeazzi Orthopaedic Institute, Milano, Italy; ^2^Division of Neuroscience, San Raffaele Scientific Institute, Milano, Italy; ^3^EPIGET LAB, Department of Clinical Sciences and Community Health, Università degli Studi di Milano, Milan, Italy, Milano, Italy

**Background**: Mesenchymal stem cells (MSCs) exert protective effects in the treatment of joint diseases mainly via soluble mediators, which can be conveyed within extracellular vesicles (EVs). CD44, the hyaluronan receptor, is a key MSC marker on both cells and EVs. In MSC, CD44 expression *in vitro* can be augmented by hyaluronan exposure, a situation mimicking MSC injected in hyaluronic acid enriched cavity of synovial joints *in vivo*. Hyaluronan also coats the surface of joint cell types, as chondrocytes and synoviocytes, opening the possibility of an improved EVs docking through CD44 binding. We investigated the role of CD44 as a key molecule for MSC-EVs homing, together with the possibility of increasing EVs incorporation through modulation of CD44 abundance.

**Methods**: MSCs were obtained from adipose tissue (ASCs) and fibroblast-like synoviocytes from synovial membrane (hFLS) after informed consent. ASCs were cultured in presence or absence of hyaluronan. EVs were isolated by centrifugation. CD44 presence and modulation in both ASCs and EVs were scored by flow cytometry. EVs’ ability to fuse with hFLS was tested by FACS, ELISA and confocal microscopy. Role of CD44 in vesicles binding to hFLS was assessed by both CD44-block and hyaluronan coat removal from hFLS.

**Results**: ASCs cultured under hyaluronan exposure for 24 h resulted in a three-fold increase of CD44 expression. Similarly, released EVs showed a 1.6-gain. On the contrary, no differences in the number of secreted vesicles or in their physical properties were observed. EVs were able to efficiently fuse with hFLS, with vesicles having higher CD44 levels more prone (1.5-fold) to be incorporated. hFLS resulted to embody around 500 EVs per day. CD44 block or hyaluronan removal resulted in a decrease of EVs incorporation, suggesting the CD44-hyaluronan interaction as a player involved in EVs docking.

**Summary/conclusion**: This study opens the possibility that presence and modulation of CD44 amount in EVs secreting cells and released vesicles, as it may happen for MSCs injected in hyaluronan rich joint cavity could direct EV/target recognition and docking. Therefore, future strategies to increase EV-associated CD44 expression, as hyaluronic-acid-supplemented media, could lead to promising approaches to improve vesicle uptake, especially in cells presenting an extensive hyaluronan coat.

OWP1.03 = PF03.12

Osteoblast-secreted extracellular vesicles stimulate the expansion of CD34+ human umbilical cord blood cells

Jess Morhayim ^1^; Mariette ter Borg^1^; Rachel van Leeuwen^1^; Corina Ghebes^2^; Jeroen van de Peppel^3^; Bram van der Eerden^3^; Jan Cornelissen^1^; Johannes van Leeuwen^3^; Carlijn Voermans^2^; Eric Braakman^1^

^1^Department of Hematology, Erasmus University Medical Center, Rotterdam, The Netherlands, Rotterdam, The Netherlands; ^2^Department of Hematopoiesis, Sanquin Research, Amsterdam, The Netherlands, Amsterdam, The Netherlands; ^3^Department of Internal Medicine, Erasmus University Medical Center, Rotterdam, The Netherlands, Rotterdam, The Netherlands

**Background**: Osteolineage cells represent one of the critical bone marrow niche components that regulate self-renewal and differentiation of haematopoietic stem and progenitor cells (HSPCs). We previously demonstrated that osteoblast-derived extracellular vesicles (EVs) retain a HSPC-supporting capacity *ex vivo*, as revealed by long-term cultures and *in vivo* repopulation assays. In the present study, we focus on elucidating the key EV miRNAs and investigate their implications on HSPC-osteolineage-cell crosstalk.

**Methods**: We isolated EVs from the conditioned medium of human osteoblasts (hFOB 1.19 and SV-HFO cells) and mesenchymal stem cells (hMSC-TERTs) by a series of ultracentrifugation steps, and referred to them as hEVs, sEVs and mEVs, respectively. We investigated the potency of the different EVs to promote ex vivo expansion of human umbilical cord blood-derived CD34+ HSPCs and subsets by enumeration using single-platform flow cytometry. We compared the miRNA profiles of stimulatory and non-stimulatory EVs using next-generation sequencing to delineate candidate EV miRNAs that may stimulate HSPC support.

**Results**: We show that sEVs enhance proliferation (two-fold, *p* < 0.01) of CD34+ cells and their immature subsets in growth factor-driven ex vivo expansion cultures, while hEVs and mEVs do not promote HSCP support compared to the control. Comparison of sEV and hEV miRNA profiles reveals that 35 miRNAs are overrepresented (≥1.5-fold, *p* < 0.05) in sEVs suggesting that differential RNA expression is actively maintained between different EVs. Furthermore, sEV treatment of CD34+ cells alters the expression of candidate miRNA targets, such as HBP1, BCL2 and PTEN, involved in the regulation of haematopoietic proliferation. Currently, we are setting up reporter experiments to evaluate the function of candidate miRNAs. In parallel, we are developing a protocol to assess the involvement of miRNAs in EV function by using a detergent-permeabilization approach combined with RNase treatment to generate “empty EVs” devoid of miRNAs.

**Summary/conclusion**: In this study, we demonstrate a novel EV-mediated mechanism for regulation of HSPC proliferation and expansion. These discoveries provide a foundation for the utilization of EV-miRNAs for the development of cord blood-derived HSPC expansion strategies to treat hematological disorders.

OWP1.04 = PS06.13

Prostate cancer-derived extracellular vesicles facilitate osteoclast fusion and differentiation via enhancing filopodia formation in osteoclast precursors

Fumihiko Urabe ^1^; Nobuyoshi Kosaka^1^; Yusuke Yamamoto^1^; Yusuke Yoshioka^1^; Fumitaka Takeshita^2^; Shin Egawa^3^; Takahiro Ochiya^1^

^1^Division of Molecular and Cellular Medicine, National Cancer Center Research Institute, Chuo-ku, Japan; ^2^Fundamental Innovative Oncology Core Center, National Cancer Center Research Institute, Chuo-ku, Japan; ^3^Department of Urology, The Jikei University School of Medicine, Minato-ku, Japan

**Background**: Bone metastasis (BM) is one of the major concerns that causes skeletal-related events and increases mortality in prostate cancer (PCa) patients. Vicious cycle paradigm has been proposed to describe how PCa cells educate osteoblasts and osteoclasts to benefit the survival and growth of the PCa cells in the metastatic site. Although the concept of vicious cycle is widely accepted, the underlying mechanisms of BM in PCa remain obscure. Extracellular vesicles (EVs) are released from almost all types of cells, and it has been shown that cancer-cell-derived EVs control their microenvironmental cells for their benefit. Here, we show that EVs from PCa cells (PCa-EVs) are involved in the vicious cycle and contribute to progression of BM.

**Methods**: PCa-EVs were isolated by ultracentrifugation and characterized by western blot and nanoparticle tracking analysis. PCa-EVs were added to osteoclast precursors, and differentiation was assessed by Tartrate-resistant acid phosphatase (TRAP) stain. TRAP-positive cells containing three or more nuclei were counted as osteoclasts. Morphological changes after addition of EVs were evaluated by immunofluorescence staining. To reveal the change of cellular transcriptome during osteoclast differentiation, total RNA was extracted from EV-treated osteoclast precursors, and RNA sequence analyses were performed.

**Results**: We found that PCa-EVs promoted osteoclast differentiation in the presence of RANKL. Mitogenic activity of PCa-EVs was not shown in the OC precursors, and the PCa-EVs did not rescue apoptosis. On the other hand, the number of filopodia formation in osteoclast was significantly increased after the addition of PCa-EVs, resulting in the promotion of cell fusion among osteoclast precursor cells. RNA sequence analyses revealed that the expression of several genes, which regulated formation of filopodia and osteoclastogenesis, was upregulated by addition of PCa-EVs. Interestingly, RANKL was not detected in PCa-EVs, suggesting that PCa-EVs have synergistic effects to promote osteoclastgenesis with RANKL.

**Summary/conclusion**: PCa-EVs synergistically activate osteoclastogenesis. PCa-EVs will be the novel diagnostic and therapeutic target for BM in PCa, leading the great improvement of quality of life in PCa patients.

OWP1.05 = PF02.02

Investigating the roles of macrophage colony-stimulating factor (CSF-1) and carbonic anhydrase 9 (CAIX) in neratinib resistant HER2+ breast cancer cell lines and extracellular vesicles

Michelle C. Lowry ^1^; Susan Breslin^2^; Lorraine O’Driscoll^2^

^1^School of Pharmacy and Pharmaceutical Sciences, Trinity College Dublin, Pearse St, Ireland; ^2^School of Pharmacy and Pharmaceutical Sciences, Trinity College Dublin, Dublin, Ireland

**Background**: In July 2017, the FDA approved neratinib for the extended adjuvant treatment of adult patients with early-stage HER2+ breast cancer. Although neratinib is proving efficacious, *de novo* and acquired neratinib-resistance (NR) is an evolving issue and the mechanisms need to be deciphered.

**Methods**: NR cell line variants (HCC1954-NR and SKBR3-NR) were previously established. Ultracentrifugation was used to purify extracellular vesicles (EVs) released from each cell variant. EVs were characterized by immunoblotting, TEM and NTA. Olink Proteomics was performed on cell lines and their respective EVs. Kaplan–Meier plots were created using BreastMark. Immunoblots and ELISAs were utilized to validate the proteomic results (macrophage colony-stimulating factor (CSF-1) and carbonic anhydrase 9 (CAIX)). Cells were treated with deferoxamine to induce CAIX and determine the levels in all cell variants. To determine if CAIX plays a role in neratinib resistance, acid phosphatase assays were performed using combinations of CAIX inhibitor (S4) and increasing concentrations of neratinib for 72 h.

**Results**: EVs were successfully isolated and characterized. Using BreastMark, high expression of CAIX correlated with decreased overall survival (*p*-value = 0.002) in HER2+ patients, similarly, this trend was also evident in lymph node-negative HER2+ patients (*p*-value = 0.01). No significant changes in CSF-1 were detected between cell line variants using immunoblots (detects one isoform). However, using ELISA (detects 3 isoforms), CSF-1 was significantly increased in HCC1954-NR cell lines and SKBR3-NR EVs (*p*-value = 0.043 and 0.002, respectively). CAIX protein was significantly increased in SKBR3-NR cells compared to the parent cell line (*p*-value < 0.01), a similar trend was observed in HCC1954 variants. Following the treatment of S4 and neratinib, Compusyn software determined that the S4 and neratinib combination is synergistic in HCC1954 and SKBR3 variants.

**Summary/conclusion**: Further studies are warranted to validate these findings, to further investigate the functional relevance of CSF-1 and CAIX in NR and subsequently progress our findings to further analysis of EVs and specimens from cohorts of breast cancer patients.

**Funding**: Irish Cancer Society’s support of Breast-Predict and H2020 support of BM1202 ME-HaD.

OWP1.06 = LBS07.16

Extracellular vesicles isolated from cardiosphere-derived cells and mesenchymal stem cells elicit distinct immunomodulatory properties *in vitro* and *in vivo*

Ann-Sophie Walravens; Sasha Smolgovsky; Lauren Kelly; Kiel Peck; Linda Marbán; Geoffrey de Couto; Luis R.-Borlado

Capricor Therapeutics, Inc., Beverly Hills, USA

**Background**: Cardiosphere-derived cells (CDCs) possess cardioprotective, regenerative and immunomodulatory characteristics when delivered to the heart post-myocardial infarction (MI). These effects are recapitulated by CDC extracellular vesicles (EVs; CDC-EVs) in acute and chronic models of MI. It has been reported that mesenchymal stem cell (MSC) extracellular vesicles (MSC-EVs) confer some immunomodulatory effects in different indications. Thus, here we compared CDC-EVs to MSC-EVs by examining their RNA cargo and testing their ability to modulate macrophage function *in vitro* and *in vivo*.

**Methods**: CDCs and MSCs were cultured for 15 days in serum-free media and then conditioned media collected, filtered and concentrated by ultrafiltration (10 kDa MWCO) to isolate EVs. Differences in CDC-EV (*n* = 12) and MSC-EV (*n* = 4) RNA cargo was determined by small RNA-seq (NextSeq 500, Illumina). The functional effect of EVs was tested on macrophages both *in vitro* and *in vivo*. For our *in vitro* assays, activated peritoneal macrophages were treated with vehicle, CDC-EVs or MSC-EVs and then assessed for proinflammatory gene expression by qPCR. For our *in vivo* assays, mice were stimulated with zymosan (intraperitoneal injection) and then treated with vehicle, CDC-EVs or MSC-EVs (intravenous injection). Forty-eight hours later, peritoneal macrophages were isolated and analyzed by flow cytometry.

**Results**: RNA-seq analysis revealed a greater overall abundance of Y RNA fragments and distinct miR composition in CDC-EVs compared to MSC-EVs. When examining the origin of EV-derived Y RNA fragments, a greater proportion of Y4-derived (*p* < 0.05), but lower amount of Y5-derived (*p* < 0.05), Y RNA were observed in CDC-EVs. *In vitro*, macrophages treated with CDC-EVs (*n* = 5), in contrast to MSC-EVs (*n* = 4), induced a dose-dependent increase in anti-inflammatory genes (*p* < 0.01). *In vivo*, CDC-EVs (*n* = 6) significantly reduced (*p* < 0.05) the accumulation of CD11b+F4/80+ peritoneal macrophages compared to MSC-EVs (*n* = 4).

**Summary/conclusion**: Here, we show that CDCs and MSCs produce intrinsically different EV populations. We demonstrate that both the RNA composition and the functional effects exerted on macrophages are distinct. Together, these data support the therapeutic utility of CDC-EVs in a range of inflammatory diseases.

OWP1.07 = LBS07.17

Role of Wnt4 exosomes in thymic ageing

Krisztina Banfai ^1^; Kitti Garai^1^; David Ernszt^2^; Judit E. Pongracz^1^; Krisztian Kvell^1^

^1^Institute of Pharmaceutical Biotechnology, Faculty of Pharmacy, University of Pecs, Pecs, Hungary, Pécs, Hungary; ^2^Institute of Physiology, Faculty of Medicine, University of Pecs, Pecs, Hungary, Pécs, Hungary

**Background**: Wnt4 plays a crucial role in promoting the development and halting the ageing of the thymus. During ageing Wnt4 is down-regulated, while PPARγ is up-regulated and triggers adipose involution. However, miR27b was described to suppress PPARγ. Our goal was to prove the presence of Wnt4 in exosomes, to detect its effect and follow its path both *in vitro* and *in vivo*.

**Methods**: Exosomes were harvested from control and Wnt4 over-expressing TECs (thymic epithelial cells) for further experiments. Exosomes were visualized by transmission electron microscopy. Exosomal miR27b levels were measured by TaqMan qPCR, while Wnt4 protein content was assayed by ELISA. DiI-labelled exosomes were applied on mouse and human thymus sections and also iv-injected into mouse for *in vivo* tracking.

**Results**: Transmission electron microscopy showed exosomes ranging 50–100 nm in size. TaqMan miRNA assay measured elevated miR27b levels, while ELISA showed high Wnt4-content in Wnt4-exosomes compared to control exosomes. For functional studies steroid (Dx)-induced TECs were used as cellular ageing model. Dx accelerated ageing, but Wnt4-containing exosomes could efficiently counteract Dx-induced senescence. We have obtained diverse staining patterns using DiI-labelled Wn4-exosomes on sections of young and aged samples. Finally, *in vivo* injected DiI-labelled Wnt4-exosomes showed detectable homing to the thymus.

**Summary/Conclusion**: According to our results Wnt4 and miR27b are present in TEC exosomes. Our findings indicate that Wnt4 is a key inhibitor thymic involution potentially via miR27b. However, further experiments are required for possible applications.

**Funding**: Scientific research support was provided by PTE AOK KA-2016-16, PTE Pharmaceutical Talent Center program and the PTE Viral Pathogenesis Talent Center program via KK. The Janos Bolyai Scholarship of the Hungarian Academy of Sciences also supported KK.

OWP1.08 = LBF04.07

Impact of pathogenic microbes and healthy microbiota by *Lactobacillus*-derived extracellular vesicles

Bao-Hong Lee ^1^; Wei-Hsuan Hsu^2^; Tang-Long Shen^3^

^1^Division of Hematology and Oncology, Department of Internal Medicine, Taipei Medical University Hospital, Taipei, Taiwan, New Taipei City, Taiwan (Republic of China); ^2^Industrial Technology Research Institute, New Taipei City, Taiwan (Republic of China); ^3^Department of Plant Pathology and Microbiology, National Taiwan University, Taipei, Taiwan (Republic of China)

**Background**: The potential of *Lactobacillus* strains against pathogenic microbialinfection has been investigated over past decades. Extracellular vesicles (EVs) are membrane-based structure secreted from various microbes,including lactic acid bacteria. EVs serve as vehicles to carry different types of cellular cargo, such as lipids, proteins, receptors and effector moleculesto therecipient cells. Recent studies have demonstrated that *Lactobacillus*-derived EVs modulate microbiota, suppress cancer cells and regulate dendritic cells, whereas their detail mechanisms remain unclear.

**Methods**: We have attempted to investigate the characteristic and anti-microbe activity of *Lactobacillus*-derived EVs.

**Results**: Our data showed that under a similar growth condition, the EVs production and size distribution among these three *Lactobacillus* strains, including *Lactobacillus acidoplilus, Lactobacillus plantarum* and *Lactobacillus reuteri*, were clearly distinct. Nevertheless, these EVs were prominently capable of suppressing the growth of pathogenic microbe *Escherichia coli, Staphylococcus aureus* and *Vibrio parahaemolyticus*.

**Summary/Conclusion**: These results indicated that *Lactobacillus*-derived EVs may be applied as novel agents for maintaining or regulating healthy microbiota.

OWP1.09 = LBS08.07

Catching the hedgehog: unravelling hedgehog secretion during filopodia-mediated transport

Gustavo Aguilar ^1^^,^^2^; Markus Affolter^2^; Isabel Guerrero^1^

^1^Centro de Biología Molecular Severo Ochoa (CSIC-UAM), Madrid, Spain; ^2^Biozentrum, University of Basel, Switzerland.

**Background**: During embryonic development, cells acquire different fates, proliferate and die in a tightly controlled manner. To orchestrate these processes, cell-to-cell communication occurs via signalling molecules that instruct cell behaviour at a distance. Among these secreted molecules, signalling by morphogens is thought to be able to subdivide a developing tissue in a concentration dependent fashion. Therefore, the dispersal of morphogens is a key event in the formation of the concentration gradients during “patterning” processes. The lipid-modified Hedgehog (Hh) is one of these morphogens, proposed to disperse via exovesicles presented by filopodia-like structures (called signalling filopodia or cytonemes) that protrude from producing towards receiving cells. The receiving cells also extend filopodia towards presenting cells, exposing the receptor to the Hh morphogen.

**Methods**: We have analysed the mechanisms for receptor and ligand exchange and also the trafficking machinery implicated. To do so, we are implementing new contact-dependent exocytosis sensors to visualize ligand and receptor secretion. We have also developed synthetic binders to membrane-trap these molecules upon presentation for reception. We are combining these tools to elucidate the basis for morphogen transport and contact-dependent cell signalling using the *in vivo* model of *Drosophila* epithelial morphogenesis.

**Results**: Our results support the model of basolateral long-distance presentation of the membrane anchored Hh by signalling filopodia in a polarized epithelium, in opposition to the apical diffusion model. We also suggest that these filopodia are the active sites for receptor presentation and ligand exchange.

**Summary/conclusion**: The use of novel tools in a multicellular organism provides a unique information to resolve the cellular basis of paracrine signalling events during tissue patterning. Our data support a model of filopodia mediated cell–cell signalling, discarding previous models of free diffusion of morphogens during epithelial development.

Oral with Poster Session 2 Chair: Francesc Borras Location: Room 5 15:30–16:30

OWP2.01 = PS09.14

Isolation and phenotype characterization of microvesicle subpopulations from mixed cells in an *in vitro* model of lung microvascular injury

Nikhil Tirlapur; Kieran P. O’Dea; Michael Wilson; Masao Takata

Section of Anaesthetics, Pain Medicine and Intensive Care, Faculty of Medicine, Imperial College London, Chelsea and Westminster Hospital, London, United Kingdom, London, United Kingdom

**Background**: Methods to isolate microvesicle (MV) subpopulations derived from a mixed parent cell population, while preserving MV biological function, are not clearly established. We present a novel method of isolating endothelial- and monocyte-derived MVs from an *in vitro* model of lung microvascular injury using immunomagnetic bead separation.

**Methods**: Human lung microvascular endothelial cells were grown to confluence on flexible-bottomed plates. Primary human monocytes were incubated for 2 h with pre-activated endothelial cells (LPS 20 ng/ml, 24 h). Cells then underwent cyclic stretching for 16 h to model pulmonary microvascular injury were seen clinically in ventilator-induced lung injury. Culture media were harvested and underwent differential centrifugation to isolate MVs. Separation of MV subpopulations was performed by negative immunomagnetic bead separation, using beads coated either with anti-CD146 (binding endothelial-derived MVs) or with anti-CD11b (binding monocyte-derived MVs). Phenotypes of isolated MV subpopulations were confirmed by flow cytometry, and their biological function tested by MV (1 × 10^6^) incubation with human umbilical vein endothelial cells (HUVECs) for 6 h, followed by flow cytometric analysis of their surface activation markers (E-selectin/ICAM-1/VCAM-1).

**Results**: Endothelial- and monocyte-derived MV subpopulations were successfully separated in our model, with >95% purity, negligible contamination with other MV subtypes, and recovery yield of 80–95% for endothelial-derived (CD146+ve) MVs and 70–85% for monocyte-derived (CD11b/CD45+ve) MVs. Monocyte-derived MVs, but not endothelial-derived MVs, induced significant HUVEC activation.

**Summary/conclusion**: Negative immunomagnetic bead separation provided efficient isolation of mixed MV subpopulations, preserving their individual phenotypes and biological function while maintaining reasonable recovery and purity. This methodology may be beneficial for functional analysis of individual MV subpopulations in samples from other *in vitro* models or *in vivo*/clinical samples.

**Funding**: Medical Research Council.

OWP2.02 = PS05.01

Detection and characterization of different neuronal and glial populations of exosomes by surface plasmon resonance imaging

Silvia Picciolini ^1^; Alice Gualerzi ^1^; Andrea Sguassero^1^; Furio Gramatica^1^; Massimo Masserini^2^; Marzia Bedoni^1^

^1^Laboratory of Nanomedicine and Clinical Biophotonics (LABION), IRCCS Fondazione Don Carlo Gnocchi, Milan, Italy; ^2^Nanomedicine Center NANOMIB, School of Medicine and Surgery, University of Milano-Bicocca, Monza, Italy

**Background**: The use of exosomes for diagnostic and disease monitoring purposes is becoming particularly appealing, considering that the pathological status greatly affects the exosomes content. Moreover, brain-derived exosomes present in blood plasma could be seen as a direct read-out of the condition of the CNS and can thus be studied as peripheral biomarkers of neurological disorders. Inspired by remarkable development of plasmonic biosensors having the ability to detect exosomes, we have designed an antibody array using surface plasmon resonance imaging (SPRi) with the aims to detect CNS-derived exosomes present in human plasma and to characterize them according to the presence and the relative amount of membrane molecules. 

**Methods**: Exosomes were isolated from plasma of healthy volunteers by size-exclusion chromatography and characterized by nanoparticles tracking analysis, transmission electron microscopy, western blot and a nanoplasmonic assay to check the sample purity. The SPRi array was optimized for the detection of exosomes subpopulations, by using a suitable surface chemistry and specific antibodies for each class of vesicle to be detected.

**Results**: Exosomes were detected and adsorbed on the SPRi chip, demonstrating the possibility to simultaneously distinguish exosomes derived specifically from neurons (Ephrin), microglia (IB4), astrocytes (GLAST) and oligodendrocytes (PLP) using the multiplexing SPRi approach. Moreover, the presence and relative amount of another membrane constituent (GM1) were then evaluated using a sandwich approach, showing a different composition of exosomes according to their cellular origin.

**Summary/conclusion**: SPRi can be used to discriminate the neuronal and the different glial populations of exosomes circulating in the peripheral blood and to perform their concomitant characterization. The optimized SPRi biosensor represents a promising platform for the characterization of exosomes involved in neurodegenerative and cerebrovascular diseases and for their possible use as clinical biomarkers.

**Funding**: Italian Ministry of Health, Ricerca corrente 2017–2018.

OWP2.03 = PS04.03

Microscale electrophoretic separations of exosomes

Yuliya Shakalisava; Delaram Zahouri; Roy Kreekel; Thomas Hankemeier

Leiden Academic Center for Drug Research, Leiden University, Leiden, The Netherlands

**Background**: Exosomes have gathered interest due to their diagnostic and therapeutic potential. They are present in blood, urine and saliva, which make them an appealing resource for non-invasive etiological and diagnostic research. Undoubtedly, size and optical properties are the most studied, which is reflected in the current isolation methods dominating the research field. Our research makes a contribution to further investigation of electrophoretic properties of exosomes. For the first time we report a microscale separation method capillary electrophoresis (CE) for characterisation of exosomes. The aim was to further explore electrophoretic behaviour of exosomes and investigate the electrophoretic signatures of exosomes in CE format.

**Methods**: CE was employed to study the electrophoretic migration of standards of exosomes in the narrow bore capillary under the electric field. Laser-induced fluorescent detector was used and different fluorescent markers were investigated for labelling of exosomes. Capillary zone electrophoresis (CZE) and capillary isotachophoresis(cITP) modes of CE were used in this study. To improve the resolution of exosomal fractions in cITP mode, various spacer compounds were investigated. The method was applied to the human exosomes samples.

**Results**: The multiple zones of exosomes can be seen in the electropherogram of exosomes standards. These indicate the subpopulations of exosomes within the commercial sample of purified exosomes. These subpopulations show differences in their electrophoretic mobility which are based on their size and charge properties. Different fluorescent markers provided an informative insight into the migration of different fractions of exosomes depending on the mechanisms of labelling. cITP approach was superior to CZE in terms of sensitivity and resolution. The analysis of human exosomes samples revealed unique signatures of exosomal fractions. The results of the healthy vs disease samples will be presented.

**Summary/conclusion**: Electrophoretic signatures of exosomes were successfully investigated in CE format. Electrophoretic properties of exosomes can offer an insightful method of characterization.

**Funding**: This project has received funding from the European Union’s Horizon 2020 research and innovation programme under grant agreement No 709077 Marie Skłodowska-Curie Individual Fellowship.

OWP2.04 = PF05.02

Normalization of urinary extracellular vesicles

Charles J. Blijdorp ^1^; Thomas A. Hartjes^1^; Martin E. van Royen^2^; Guido W. Jenster^1^; Robert Zietse^1^; Ewout J. Hoorn^1^

^1^Erasmus Medical Center, Rotterdam, The Netherlands; ^2^Department of Pathology, Erasmus Optical Imaging Centre, Erasmus MC, Rotterdam, The Netherlands

**Background**: Urinary extracellular vesicles (uEVs) have emerged as a powerful non-invasive tool to study renal epithelial transport in humans. However, the optimal method to quantify and normalize uEVs remains unclear, especially for spot urines. 

**Methods**: Four healthy subjects were subjected to overnight thirsting (10 pm to noon) followed by water loading (20 ml/kg in 30 min). Spot urines were collected during thirsting (T1–2) and after water loading (WL1–4, noon to 7 pm). Subsequently, 4 uEV quantification techniques were compared: (1) nanoparticle tracking analysis (NTA), (2) uEV isolation by ultracentrifugation followed by immunoblotting of CD9, CD63, CD81, ALIX, and TSG101, (3) a time-resolved fluorescence immunoassay (TRFIA) that captures CD9+ uEVs, and (4) EVQuant, a novel technique which counts individual fluorescently labelled EVs after immobilization in a matrix. A Bland–Altman analysis was used to compare methods using NTA as reference.

**Results**: As expected, urine osmolality was near-maximal during thirsting, decreased after water loading and then increased again. The results of the 4 uEV quantification methods showed similar dynamics as urine osmolality suggesting that uEV number changes in proportion to urinary concentration. Of interest, EVQuant identified 2.4 ± 0.5 times more uEVs than NTA. Using NTA as reference, the Bland–Altman analysis showed that EVQuant had the best agreement (SD of bias 16%) followed by TRFIA (SD of bias 22%). Of the uEV-markers, CD9 agreed best with NTA (SD of bias 28%). uEV number correlated strongly with urine creatinine (R2 0.9, *p* < 0.0001).

**Summary/conclusion**: uEV number is proportional to urinary concentration and urine creatinine can be used to normalize spot urines for uEV number. EVQuant is a promising alternative to NTA and appears more sensitive for uEV detection. These uEV quantification methods can also be used to analyze if changes in a uEV protein of interest are the result of more protein per uEV or the excretion of more uEVs containing this protein.

**Funding**: Dutch Kidney Foundation.

OWP2.05 = PF01.20

Comparative analysis of Raman signals between non-small cell lung cancer (NSCLC) cell derived exosomes and their potential protein markers

Hyunku Shin ^1^; Hyesun Jung^2^; Jaena Park^1^; Sunghoi Hong^3^; Yeonho Choi^4^

^1^Department of Bio-convergence Engineering, Korea University, Seoul, Republic of Korea, Seoul, Republic of Korea; ^2^School of Biosystem and Biomedical Science, Department of Public Health Sciences, Korea University, Seoul, Republic of Korea;^3^School of Biosystem and Biomedical Science, College of Health Science, Korea University, Seoul, Republic of Korea; ^4^School of Biomedical Engineering, Korea University, Seoul, Republic of Korea

**Background**: Surface proteins of exosomes are of great interest for cancer diagnosis. Surface-enhanced Raman spectroscopy (SERS) is one of the useful methods for investigating the surface proteins. Here, we demonstrated identification of unique SERS spectrum of non-small cell lung cancer (NSCLC) cell-derived exosomes and their proteins contributing to the unique spectral patterns. We identified unique Raman spectrum of cancerous exosomes by principal component analysis and quantitative analysis and compared SERS spectra of the five surface protein markers with the unique SERS spectrum of cancerous exosomes.

**Methods**: Exosomes in cell culture media were prepared by size-exclusion column chromatography. SERS substrate was prepared by drying gold nanoparticles, followed by coating with linker for exosomes and proteins. After exosomes or proteins were dropped, spectra were measured.

**Results**: We measured Raman spectra of exosomes derived from normal alveolar cells, NSCLC cell lines-derived exosomes, and PBS as a control. We identified major spectral patterns by principal component analysis and quantitative analysis of mixtures containing normal and cancerous exosomes. The intensities of 15 peaks generally increased with the density of cancerous exosomes.

Several protein markers had a correlation with these peaks in a comparative study on Raman spectrum. Interestingly, this concordance was consistent with immunoblotting results. The protein markers which had uniquely overlapping peaks showed higher expression on cancerous exosomes. This result indicates that these proteins could contribute to the Raman spectrum of cancerous exosomes.

**Summary/conclusion**: In conclusion, we compared unique Raman spectrum of lung cancer cell-derived exosomes and their protein markers. We estimated unique Raman spectral peaks and compared to Raman spectra of five protein markers. Finally, we could identify the protein markers likely contributing to the Raman spectrum of the cancerous exosomes.

**Funding**: This research was supported by a grant from the Korea Health Technology R&D Project through the Korea Health Industry Development Institute, funded by the Ministry of Health & Welfare, Republic of Korea (Grant Nos. HR14C0007).

OWP2.06

Development of high sensitivity flow cytometry for sizing and molecular profiling of individual extracellular vesicles down to 40 nm

Ye Tian^1^; Manfei Gong^1^; Haisheng Liu^1^; Wenqiang Zhang^1^; Ling Ma^2^; Shaobin Zhu^2^; Xiaomei Yan^1^

^1^Department of Chemical Biology, Xiamen University, Xiamen, China; ^2^NanoFCM Inc., Xiamen, China

**Background**: Though of great importance, sizing and molecular profiling of individual extracellular vesicles (EVs) are technically challenging due to their nanoscale particle size, minute quantity of analytes, and overall heterogeneity. Our laboratory has developed high sensitivity flow cytometry (HSFCM) that allows light scattering detection of single silica nanoparticles (SiNPs) and viruses as small as 24 and 27 nm in diameter, respectively. Here we report a HSFCM-based approach for quantitative multiparameter analysis of single EVs down to 40 nm.

**Methods**: EVs were extracted from cell cultured medium and human blood samples by ultracentrifugation. Employing SiNPs as the size reference standards and upon refractive index mismatch correction based on the Mie theory, accurate sizing of EVs can be obtained by direct measurement of the scattered light from individual EVs. The subpopulation of EVs expressing specific surface proteins were analyzed upon immunofluorescent staining and single particle enumeration by the HSFCM. Lipid dyes such as PKH 26 and Dil, and nucleic acid dyes such as SYTO 9 and RNA Select were also used to stain the EVs. The glycoproteins on the surface of single EVs were quantified via metabolic incorporation of azide-modified monosaccharides which were then chemoselectively coupled to complementary alkyne-functionalized fluorophores.

**Results**: HSFCM provides accurate sizing of single EVs down to 40 nm with an analysis rate up to 10,000 particles per minute, and the resolution is comparable to that of cryo-TEM. The population of EVs expressing CD9, CD63, or CD81 will be reported along with their copy number distributions on single EVs. Meanwhile, the staining ratios of lipid membrane dyes, nucleic acid dyes, and glycoproteins will be reported against side scattering measurements. When HSFCM was used to analyze blood samples, a significantly elevated level of CD147+ EVs was identified in colorectal cancer patients compared to healthy donors (P < 0.001).

**Summary/conclusion**: HSFCM expands the capability of flow cytometry for single-cell analysis to single EVs as small as 40 nm. HSFCM enables us to create an objective benchmark to insight into heterogeneous EV populations, which is highly desirable to decipher the biology of EVs and promote the development of EV-based liquid biopsy and therapeutics.

OWP2.07 = LBT03.07

Immunofluorescence flow cytometry of extracellular vesicle surface proteins

John Nolan ^1^; Erika Duggan^1^

^1^Scintillon Institute, San Diego, USA

**Background**: Like the cells that produce them, extracellular vesicles (EVs) bear surface molecules that can give clues to identity and function. Unlike cells, surface proteins on EVs are present in numbers that challenge the sensitivity of conventional flow cytometers, which presents challenges to quantitative and reproducible measurements. We have adapted calibration and standardization approaches from quantitative IF of cells to enable quantitative and reproducible measurement of EV surface proteins.

**Methods**: Erythrocytes and platelets (RBCs, PLTs) were washed, treated with ionophore (A23187) in the presence of Ca+2, and centrifuged (2×, 2500× g, 15 min) to remove cells and large debris. Cell lines were cultured for 48 h in EV-free media and the media were collected, centrifuged to remove cells and large debris, and concentrated ~100-fold by centrifugal ultrafiltration and stored at −80°C. Vesicle flow cytometry (VFC) was performed using a vesicle measurement kit comprised of a vesicle staining solution and a synthetic vesicle size standard. EV samples were stained with fluorescent antibodies to various surface markers and measured by flow cytometry using a fluorescence trigger. Fluorescence intensity was calibrated using commercial MESF intensity standards, custom intensity standards and antibody-capture standards.

**Results**: VFC measures the number, size and FL-Ab staining of individual EVs, to ~70 nm in diameter and ~30–50 PE-Abs. We performed VFC with IF on RBC and PLT EVs using antibodies to abundant cell surface proteins, with antigen-free vesicles and non-specific IgGs serving as controls. RBC EVs were 75–300 nm in diameter (median 160 nm) and bound 90–6000 PE-Abs (median 2200 MESF) to CD235ab. PLT EVs were 75–500 nm in diameter (median 175 nm) and bound 90–9000 PE-Abs to CD41 (median 900 MESF), 50–6000 CD61 (median 480 MESF) and 50–3000 PE-Abs (median 625 MESF) to CD9. Antibody capture beads with calibrated numbers of Ab-binding sites allow quantitative assessment of different fluorescent conjugates for suitability in EV IF.

**Summary/conclusion**: By observing the basic tenets of quantitative FC, including using appropriate controls, standards, calibration protocols and experimental design, EV IF can be performed quantitatively and reproducibly.

LBT01.15 = OWP2.08

Free flow electrophoresis allows preparation of extracellular vesicles fractions with high recovery and purity rates

Gerhard Weber^1^; Simon Staubach^2^; Christian Reiter ^1^; Bernd Giebel^2^

^1^FFE Service GmbH, Feldkirchen, Germany; ^2^Institute for Transfusion Medicine, University Hospital Essen, Essen, Germany

**Background**: Free flow electrophoresis (FFE) is a well-established (micro)preparative method to separate analytes with inherent difference of charge density and/or difference of pI-value. Run with media of different pH values (pH = 8 to pH = 4.8), FFE has classically been optimized to effectively separate amphoteric analytes, like proteins and peptides, from non-amphoteric analytes, like lipid vesicles, DNA and RNA.

**Methods**: According to the need to isolate pure EVs especially from plasma samples, we took the challenge and optimized the FFE for the EV purification, either as a stand alone method or in combination with a second separation method, the size exclusion chromatography (SEC), being performed before FFE. Obtained FFE fractions (48 per run) were analysed for their protein content (fluorescence 280−350 nm), the sizes of their proteins (SDS PAGE, Coomassie and silver staining) and for the presence of EV markers in dot blot and Western blot analyses.

**Results**: Initially, EV markers were recovered in FFE fractions which also contained high concentrations of non-EV-associated proteins such as albumin. By changing several parameters, we have optimized FFE and maximized the sample throughput at a minimum dilution, both in a continuous and in an interval mode.

Now, the largest content of negatively charged EVs from plasma and serum samples can be enriched in 1–3 fractions. These fractions are diluted 1:3 only and contain less than 1% of total sample protein. Coomassie staining of SDS PAGEs confirmed that their protein profiles differ from that of EV-free FFE fractions. Particles within the EV fractions could be quantified by nanoparticle tracking analysis (NTA) without prior concentration. The true EV nature of the harvested particles was confirmed by western blot analysis. Of note, maybe due to the high heterogeneity of EVs in given samples, a minor proportion of vesicles has been detected in other FFE fractions, which will be characterized in the future. Now, with the improved continuous separation protocol, two plasma or serum samples can be processed in parallel at a throughput of 5 ml per hour each.

**Summary/conclusion**: In summary, FFE provides a powerful method, to purify and fractionize EVs from plasma and serum samples as well as from other liquids. If required, it can be combined with other EV processing technologies like SEC.

LBT01.13 = OWP2.09

Isolation of extracellular vesicle-associated small RNA from canine mitral valve interstitial cells using ultracentrifugation and tangential flow filtration with size exclusion chromatography

Vicky Yang ^1^; Dawn Meola^1^; Kristen Thane^1^; Andrew Hoffman^1^

^1^Tufts University Cummings School of Veterinary Medicine, North Grafton, USA

**Background** : Myxomatous mitral valve disease is a highly prevalent canine cardiac disease that can lead to congestive heart failure. Histologic changes in the valves include greater prevalence of valvular interstitial cells (VIC) with myofibroblastic phenotype. These changes and the functional consequences are virtually identical to mitral valve prolapse in people. Our previously published work shows that, compared to VIC harvested from normal mitral valves, VIC from diseased valves had decreased cellular expression of let-7c, miR-17, miR-20a and miR-30d. However, the miRNA content of extracellular vesicles (EV) from normal and diseased VIC have not yet been analyzed.

**Methods** : VIC were isolated by enzymatic digestion from normal and diseased valves (*n* = 5/group). Passage 2 VIC were cultured in defined chemical media, and the conditioned media were collected every 24 h for 3 days. EV were then isolated using ultracentrifugation (UC) (300× g, 10 min; 2000× g, 10 min; 10,000× g, 30 min; 100,000× g, 70 min) followed by size exclusion chromatography (HPLC), or using tangential flow filtration (TFF) (100kDa MWCO PES filters) followed by HPLC. EV were further characterized using nanoparticle tracking analysis, TEM and Western blot for CD9 and TSG101. RNA from VIC were isolated using the mirVana miRNA isolation kit and from EV using the Qiagen miReasy kit. Isolated RNA concentrations were determined by the Agilent Bioanalyzer.

**Results** : HPLC showed a single peak corresponding to the EV fraction for samples first processed by UC, whereas those first processed by TFF showed two distinct peaks (F1 and F2 fractions). Average total particle yield was higher by TFF+HPLC vs. UC+HPLC (7.8 × 109 ± 7.3 × 10^9^ vs. 1.5 × 109 ± 6.0 × 10^8^), with 74% of the TFF+HPLC particles residing in the F1 vs. F2 fraction. TFF+HPLC yielded on average more small RNA than UC+HPLC (9.4 ± 7.4 μg/μl vs. 6.3 ± 10.1 μg/μl), with 59% of the total RNA residing in the F1 fraction. Western blot showed that F1 EV were positive for TSG101 while F2 EV were not.

**Summary/conclusion** : Compared to UC+HPLC, TFF+HPLC yielded higher RNA concentrations and was able to separate two different EV populations. The miRNA content of the 2 EV fractions and of the VICs will be further analysed by RNA sequencing to better understand the miRNA expression differences between the cellular and EV populations.

**Funding** : Shipley Foundation.

Oral with Poster Session 3 Chair: Maria Yañez-Mó Location: Room 6 15:30−16:30

OWP3.01 = PS03.01

Sarco/endoplasmic reticulum ATPase inhibition activates calcium signalling pathways for microvesicle biogenesis

Jack D. Taylor ^1^; Michael Johnson^2^; Gregory Monteith^3^; Mary Bebawy^4^

^1^University of Technology Sydney, Sydney, Australia; ^2^School of Life Sciences, University of Technology Sydney, NSW, Sydney, Australia; ^3^The School of Pharmacy, The University of Queensland, Brisbane, Australia; ^4^The Graduate School of Health, The University of Technology Sydney, Sydney, Australia

**Background**: An increase in intracellular Ca^2+^ is a key initiator of microvesicle (MV) biogenesis. The Ca^2+^-signalling pathway(s) implicated in this are currently unknown. This study aims to elucidate the Ca^2+^ pathways involved in MV biogenesis in malignant and non-malignant cells in an attempt to identify selective drug targets for vesicle inhibition. 

**Methods**: Interrogation of the Ca2+ signalling pathway was done using the SERCA inhibitor, thapsigargin (TG), the Calpain inhibitor II (ALLM) and the inhibitor of store-operated Ca2+ entry (YM58483). AFM was used to study cell surface topography in response to inhibitors in HBEC-D3, MCF-7 and MCF-7/Dx cells (see Taylor et al., 2017). MV isolation and flow cytometric quantification were done as per Roseblade et al. (2015). Real-time deconvolution (DeltaVision personalVD, Elite) and super-resolution (DeltaVision OMX Blaze) microscopy were used for live cell imaging using CellLight Plasma Membrane-RFP, Bacmam 2.0®.

**Results**: ALLM selectively inhibited vesiculation in malignant cells confirming a basal Ca2+-calpain dominant pathway. This was not observed for non-maligant cells confirming an alternative vesiculation pathway independent of calpain (Taylor et. al., 2017).

Depletion of endoplasmic reticulum stores by TG alone resulted in slight and significant increases in vesiculation in malignant and non-malignant cells respectively, suggesting a maintained level of Ca2+ through a SOCE pathway. In the presence of YM58483 alone, we saw no significant effect above basal levels in both cell types. In the presence of TG and YM58483, we observed inhibition of vesiculation, consistent with a SERCA/SOCE-mediated regulation of vesiculation. Consequently, only differentiator in vesiculation in malignant vs. non-malignant cells appears to be the involvement of calpain rather than Ca2+ signalling through SECRA/SOCE.

In visualizing the morphology of the cells using both AFM and live cell imaging, we observed vesiculation to be perinuclear, clustered and polarized in MCF-7 cells at rest and upon activation in both cell types.

**Summary/conclusion**: We show for the first time the involvement of SERCA/SOCE Ca2+ signalling in MV vesiculation. Differences in basal vesiculation in malignant and non-malignant cells are at the level of calpain rather than the SERCA/SOCE pathway.

OWP3.02 = PT08.17

Origin of extracellular vesicles released during exhaustive exercise

Alexandra Brahmer ^1^; Perikles Simon^2^; Eva-Maria Krämer-Albers^3^

^1^University of Mainz, IDN, Molecular Cell Biology, Mainz, Germany; ^2^University of Mainz, Department of Sports Medicine, Rehabilitation and Prevention, Mainz, Germany; ^3^IDN, Molecular Cell Biology, Johannes Gutenberg University Mainz, Mainz, Germany

**Background**: Extracellular vesicles (EVs) represent versatile entities with body-wide signalling functions as they pass barriers and deliver complex biomolecules between cells and tissues. We recently demonstrated that exosome-like EVs are secreted into the circulation during an early phase of exercise. Physical activity is known to exhibit a wide range of beneficial properties regarding cardiovascular and immune functions as well as the ageing process. Determination of the source and target cell populations is essential to examine the potential systemic effects of EVs released during physical activity. Here, we performed a detailed characterization of exercise-EVs (“ExerVs”).

**Methods**: Healthy male athletes were subjected to an incremental cycling test until exhaustion. Blood was drawn before, during and immediately after the test and distinct subclasses of EVs were purified from EDTA blood plasma by different isolation methods: size exclusion chromatography and CD9-, CD63- or CD81-immunobead isolation. Isolated EVs were characterized by western blot using common EV-markers and MACSPlex analysis of a variety of characteristic surface epitopes. Functional tests include stimulation of THP1 cells as well as HUVEC cells using ex vivo ExerVs from exhausted athletes. The proteomic cargo of ExerVs will be evaluated by quantitative mass spectrometry.

**Results**: Western blot analysis of tetraspanin (CD9, CD63, CD81) kinetics confirmed EV release during incremental cycling, showing highest levels at peak exercise. MACSPlex surface marker analysis of the same EV samples showed comparable increases of vesicles bearing markers of antigen presenting cells, endothelial cells, lymphocytes and platelets. These observations suggest involvement of several subclasses of exercise EVs in angiogenesis, coagulation, immune function and tissue repair, currently assessed in cell culture experiments involving ex vivo ExerVs.

**Summary/conclusion**: EVs released during physical exercise (ExerVs) comprise a mixed population of vesicles derived from antigen-presenting cells, lymphocytes, platelets and endothelial cells. These findings enable further investigation of ExerVs with regard to multi-systemic signalling associated with health benefits to evaluate their diagnostic and therapeutic potential for various lifestyle-associated diseases.

OWP3.03 = PS05.02

Extracellular vesicles as mediators of periphery-to-brain communication in inflammation-associated brain disorders

Nagiua Haymour ^1^; Alekhya Mazumdar^2^; Mea Holm^3^; Martin Schwab^3^; Irene Knuesel^4^; Christopher Pryce^1^; Giorgio Bergamini^1^

^1^Preclinical Laboratory for Translational Research into Affective Disorders, Department of Psychiatry Psychotherapy and Psychosomatics Psychiatric Hospital, University of Zurich, Zurich, Switzerland; ^2^Department of Orthopaedics, Balgrist University Hospital, Zürich, Switzerland; ^3^Brain Research Institute, University of Zurich and ETH Zurich, Zurich, Switzerland; ^4^Roche Pharmaceutical Research and Early Development, Roche Innovation Center, Basel, Switzerland

**Background**: Substantial evidence shows that inflammation is important in the aetiology of several psychiatric disorders, including major depressive disorder (MDD). Furthermore, MDD symptoms are often observed in patients with infection and autoimmune diseases. Stress and inflammation have been proposed to affect emotion and cognition in part through their inhibitory effects on the brain dopaminergic system. We have demonstrated that chronic social stress (CSS) induces MDD-relevant behavioural states in mice including decreased motivation for rewards. CSS mice exhibit an inflammatory response in the periphery and brain and dysregulation of the dopamine system. We have also shown that a systemic inflammatory challenge (i.e. lipopolysaccharide (LPS)) induces MDD-relevant sickness behaviours. We hypothesize here that extracellular vesicles (EVs) released from peripheral immune cells constitute a pathophysiological pathway via which peripheral inflammatory signalling (e.g. miRNAs) can be communicated to brain, to trigger neuropsychiatric disorders.

**Methods**: We use mouse models of (a) LPS and (b) CSS-induced brain-behaviour dysfunction. To investigate the effect of LPS and CSS on EVs, plasma EVs are isolated and miRNA content is analysed using qPCR. Transgenic mice, exposed to either LPS or CSS, are used to investigate the effects of inflammation on EVs-mediated signalling.

**Results**: Using TEM, western blots and NTA, we show that EVs can be isolated from plasma using a polymer-based protocol. The expression of inflammation-associated miRNAs is measured in EVs treated with proteinase K and RNAse. LPS increases EVs expression of mir-155 and mir-146a at 5 h post-injection. Using transgenic mice, we will investigate if LPS and CSS increase periphery-to-brain communication, with Cre-mediated recombination rate in brain cells as marker for EVs-mediated signalling.

**Summary/conclusion**: These experiments indicate that the inflammatory effects on the systemic milieu include changes in miRNAs content of blood EVs. Furthermore, we will investigate if EVs transduce peripheral immune signals to the brain under inflammatory conditions. Future experiments will investigate the pathophysiological role of EVs in MDD-relevant brain and behavioural dysfunctions, allowing the identification of therapeutic targets for inflammation-associated behavioural disorders.

OWP3.04 = PS09.01

Extracellular vesicles deformation on surface: some tracks to limit it

Ksenia Maximova ^1^; Sameh Obeid^2^; Thierry Burnouf^3^; Wilfrid Boireau^1^; Celine Elie-caille^1^

^1^FEMTO-ST Institute, UBFC, Besancon, France; ^2^French National Institute for Agricultural Research | INRA, Rennes, France; ^3^College of Biomedical Engineering Taipei Medical University, Taipei, Taiwan, Tapei, Taiwan (Republic of China)

**Background**: Despite the booming development of multiple characterization techniques of extracellular vesicles (EVs), reliable nanocharacterization of the EVs still remains a challenge due to the large variety of their size and cell origin.

**Methods**: In this context, our efforts are aimed at the development of a NanoBioAnalytical (NBA) platform, which combines multiple characterization techniques, including atomic force microscopy (AFM) − a source of information about EVs metrology. Our principle goal is to create a versatile biochip–instrument interface, which opens the possibility to multi-technique and multi-scale investigations that in its turn bring complete information about the different EVs populations. Our NBA platform consists in a biochip, which is biofunctionalized in a multiplexed format, through the grafting of different relevant and specific ligands. This biochip behaves like a “EVs smart carrier”, since it first enables the biodetection and capture of EVs subsets, thanks to a surface plasmon resonance instrument, while EVs size and morphology are achieved on the same biochip by AFM in the next place.

**Results**: Nevertheless, EVs are known to be soft and deformable, thus their dimensions and morphology obtained by AFM measurements may vary, among other things, according to support constraints. Depending on whether EVs simple “passive” adsorption or immunocapture on a substrate, and even function of the antibody density grafted on it, EVs may deform pretty much and possibly loose partly their functionality. In addition, multiple AFM imaging modes and parameters can also impact the metrological analysis of EVs, some of them being really important to warrant a confident EVs nanocharacterization. Finally, taking care about these surface and imaging experimental conditions, a correlation between 2D (on the surface) and 3D (in solution) EVs characterization was achieved.

**Summary/conclusion**: Thus, this current communication, through highlighting the influence of certain biointerface and imaging experimental parameters on the whole EVs subsets qualification, could contribute by giving sort of guidelines for EVs characterization by AFM.

**Funding**: This work was realized thanks to a CNRS interdisciplinary call (Défi instrumentation aux limites) and funds from the Franche-Comte region obtained in 2017.

OWP3.05 = PF01.01

Comparison of generic fluorescent dyes for detection of extracellular vesicles by flow cytometry

Leonie de Rond ^1^; Edwin van der Pol^2^; Chi M. Hau^3^; Zoltan Varga^4^; Auguste Sturk^5^; Ton G. van Leeuwen^2^; Rienk Nieuwland^5^; Frank A.W Coumans^6^

^1^Academic Medical Center, University of Amsterdam, Amsterdam, The Netherlands; ^2^Biomedical Engineering & Physics, Academic Medical Center, University of Amsterdam, Amsterdam, The Netherlands, Amsterdam, The Netherlands; ^3^Laboratory Experimental Clinical Chemistry, Academic Medical Center, University of Amsterdam, Amsterdam, The Netherlands, Amsterdam, The Netherlands; ^4^Biological Nanochemistry Research Group, Institute of Materials and Environmental Chemistry, Research Centre for Natural Sciences, Hungarian Academy of Sciences, Budapest, Hungary, Budapest, Hungary; ^5^Laboratory of Experimental Clinical Chemistry, and Vesicle Observation Center, Academic Medical Center, University of Amsterdam, Amsterdam, The Netherlands, Amsterdam, The Netherlands; ^6^Department of Biomedical Engineering and Physics, and Vesicle Observation Center, Academic Medical Centre of the University of Amsterdam, Amsterdam, The Netherlands

**Background**: Because extracellular vesicles (EVs) in plasma are potential biomarkers of disease, a generic fluorescent dye specifically staining EVs is desirable. Here, we evaluated five commonly used generic dyes for flow cytometry.

**Methods**: EVs from MCF7-conditioned culture medium and human plasma were stained with calcein AM, calcein violet, CFSE, di-8-ANEPPS or lactadherin. The concentration of EVs detected by generic dyes was measured by flow cytometry (A60-Micro, Apogee). EVs were identified by immunostaining EpCAM for MCF7-EVs and CD61 for platelet EVs. Scatter triggering was applied as a reference, and the influence of non-EV components was evaluated.

**Results**: Di-8-ANEPPS, lactadherin and side scatter detected 100% of EpCAM+ MCF7-EVs. In plasma, di-8-ANEPPS inefficiently stained EVs due to protein binding, which improved by protein removal. Lactadherin and side scatter detected 33% and 61% of CD61+ EVs, respectively. Because all generic dyes stained proteins, the overall sensitivity to detect platelet EVs in plasma was 33% at best. Calcein AM, calcein violet and CFSE were either inefficient at detection of EVs in both samples, or suffered from swarm detection and/or insufficient event rates.

**Summary/conclusion**: None of the generic dyes detected all and only EVs in plasma. Side scatter triggering detected the highest concentration of plasma EVs on our flow cytometer, followed by lactadherin. The choice between scatter or lactadherin primarily depends on the sensitivity of the flow cytometer used.

**Funding**: We acknowledge funding from The Netherlands Organisation for Scientific Research - Domain Applied and Engineering Sciences (NWO-TTW), research programs VENI 13681 (FC) and Perspectief CANCER-ID 14195 (LR).

OWP3.06 = LBS08.06

Role of calcium signalling in the biogenesis of different types of extracellular vesicles derived from the same cell

Ákos Lőrincz ^1^; Balázs Bartos^1^; Dávid Szombath^1^; Dániel Veres^2^; Ágnes Kittel^3^; Erzsébet Ligeti^1^

^1^Department of Physiology, Semmelweis University, Budapest, Hungary; ^2^Department of Biophysics, Semmelweis University, Budapest, Hungary; ^3^Institute of Experimental Medicine, Hungarian Academy of Sciences, Budapest, Hungary

**Background**: It has been reported for several cell types that initiation of a sharp calcium signal by application of artificial means such as calcium ionophores induces generation of extracellular vesicles (EVs). However, the role and requirement of calcium signals triggered by natural stimuli in production of different types of EVs released from the same cell is largely unknown.

**Methods**: Medium-sized EVs were obtained in two centrifugation and filtration steps from neutrophils (PMN) isolated from human peripheral blood or murine bone marrow. Murine PMN-EVs were characterized in detail using dynamic light scattering and electron microscopy. EVs were quantitated by flow cytometry and protein measurements.

**Results**: EV production from human neutrophilic granulocytes occurring spontaneously (sEV) and upon stimulation with opsonized particles (aEV) was compared in the absence and presence of extracellular calcium. Generation of aEV was seriously impaired by calcium deficiency whereas release of sEV was not affected. These results were supported in similar experiments carried out on neutrophils isolated from murine bone marrow. Murine neutrophils deficient in phospholipase γ2, the key enzyme for intracellular calcium signalling, were also impeded in release of aEVs whereas sEV production proceeded undisturbed.

**Summary/conclusion**: Requirement for extracellular calcium supply and intracellular calcium signalling strongly diverges in generation of different types of EVs from the same cell. These findings supply molecular data on the existence of distinguishable cellular pathways of EV production.

**Funding**: NKFIH K119236, Hungary.

OWP3.07 = LBS08.05

Unravelling the distribution of extracellular vesicles *in vivo* using recombinant tetraspanins

Stefan Vogt ^1^; Madhusudhan Reddy Bobbili^1^; Carolina Patrioli^1^; Samir Barbaria^1^; Markus Schosserer^2^; Lucia Terlecki-Zaniewicz^2^; Elsa Arcalis^3^; Dietmar Pum^3^; Severin Muehleder^4^; Wolfgang Holnthoner^4^; Christopher Kremslehner^5^; Florian Gruber^6^; Johannes Grillari^2^

^1^Department of Biotechnology, University of Natural Resources and Life Sciences, Vienna, Austria., Vienna, Austria; ^2^CDL for Biotechnology of Skin Aging BOKU - Department of Biotechnology, Vienna, Austria; ^3^Department of Applied Genetics and Cell Biology, University of Natural Resources and Life Sciences, Vienna, Austria., Vienna, Austria; ^4^Ludwig Boltzmann Institute for Experimental and Clinical Traumatology, AUVA Research Centre, Endothelial Cell Croup, Vienna, Austria., Vienna, Austria; ^5^Department of Dermatology, Medical University of Vienna, Austria; Christian Doppler Laboratory for Biotechnology of Skin Aging, Austria., Vienna, Austria; ^6^CDL for Biotechnology of Skin Aging Medical University of Vienna, Vienna, Austria

**Background**:Extracellular vesicles (EVs) which were considered as garbage bags of cells came into view only a decade ago and are now increasingly recognized for their importance in cell-to-cell communication. Its their apparent natural ability to transfer cargo from donor cell to recipient cell thereby conferring messages in paracrine or endocrine manner. Over a decade, lot of research has been done to understand the omics, mode of secretion and uptake mechanisms. However, especially the trafficking of EVs *in vivo* is still poorly understood. 

**Methods**: We here generated the tetraspanins CD63 and CD81 C-terminally fused to a snorkel tag (1) that adds an additional transmembrane domain to the four existing ones to be able to attach further tags facing the extracellular space. Due to their extravesicular orientation, these tags can be used as a future tool to understand trafficking of EVs *in vivo*. As a first step, we aimed to give proof of principle that our constructs allow to track and isolate functional recombinant EVs from cultured cells. We therefore established a method to isolate functional EVs carrying our recombinant tetraspanins using a combination of anti-hemagglutinin affinity matrix and precission protease cleavage to isolate EVs without damaging the EV membrane and without losing the CLIP and FLAG tags which are preceding to precission protease site and HA tag.

**Results**: Indeed, we were able to purify the EVs by this strategy. To further proof that these EVs are able to transfer intact and active cargo to recipient cells, we additionally loaded the EVs with Cre recombinase mRNA (2). Therefore, we stably expressed recombinant tetraspanins and Cre recombinase in donor HeLa cells and fluorescent colour switch LoxP system in recipient HEK293 cells (3). Indeed, snorkel tagged EVs were taken up in this experiment. Using an *in vivo* mimicking 3D cell culture model (4), we also observed a crosstalk from human dermal fibroblasts to keratinocytes with snorkel tag containing EVs.

**Summary/conclusion**: Finally, we are currently testing if snorkel tag containing EVs from the stable HeLa cell line introduced into a xenograft mouse model can be isolated from plasma and tissues to understand the distribution of tumour derived EVs in different tissues. We therefore pave the ground for using snorkel-tagged EVs as a valuable tool to understand EV trafficking *in vivo*.

OWP3.08 = LBS08.04

Evidence for selective mRNA sorting into cancer exosomes

Mohammad Arshad Aziz ^1^; Fatima Qadir^2^; Ahmad Waseem^2^; Muy-Teck Teh^2^

^1^University of Otago, Dunedin, New Zealand; ^2^Centre for Oral Immunobiology & Regenerative Medicine, Institute of Dentistry, Barts & The London School of Medicine and Dentistry, Queen Mary University of London, England, United Kingdom

**Background**: Exosomes are membrane-bound vesicles released by cells into their extracellular environment. It has been shown that cancer cells exploit this mechanism for local and/or distant oncogenic modulation. As it is not clear if oncogenic mRNA molecules are sorted selectively or randomly into exosomes, this study investigated using a cell culture model.

**Methods**: Exosomes were isolated using an established ultracentrifugation method from cell culture supernatant of a premalignant buccal keratinocyte (SVpgC2a) and a malignant (SVFN10) cell line. Exosome and cell debris pellets were then subjected to RNase A and proteinase K protection assays prior to extraction of total RNA for reverse transcription quantitative PCR (RT-qPCR) to quantify mRNA of 15 expressed genes.

**Results**: RNA in cell debris pellet were sensitive to RNase A treatment but exosomal RNA were resistant to RNase A. Pre-incubation of exosome pellet with Triton-X to solubilize membranes rendered exosomal RNA sensitive to RNase A, indicating that exosomal RNA was protected within exosomal membranes. RT-qPCR showed that mRNA were present within exosomes. Of the 15 genes selected for RT-qPCR in this study, two (FOXM1 and HOXA7) were found to be more abundant in exosomes secreted from the malignant SVFN10 cells compared to the premalignant SVpgC2a cells. RNase A pretreatment on exosomal pellet did not degrade FOXM1 and HOXA7 mRNA suggesting that these mRNA were protected within exosomes. Interestingly, one gene (*ITGB1*), although abundantly expressed in parental cell, was not resistant to RNase A pretreatment indicating that not all mRNA purified from the exosomal pellet were sorted into the vesicles.

**Summary/conclusion**: In conclusion, this study presented the first evidence that mRNA molecules were found to be protected within exosomes secreted by human buccal keratinocytes. Furthermore, we presented evidence for selective sorting of specific mRNA molecules into exosomes which is independent of parental cell mRNA concentration. This suggests that tumour cells preferentially package certain oncogenes in their exosomes as a potential intercellular vehicle for reprograming target cells. Signature of mRNA contents within cancer exosomes may have clinical applications for diagnostic and therapeutic purposes.

OWP3.09 = LBF05.04

Alterations in the miRNA cargo of HIV-infected macrophage-derived extracellular vesicles promote pulmonary smooth muscle proliferation

Himanshu Sharma; Navneet K. Dhillon; Mahendran Chinnappan; Stuti Agarwal; Pranjali Dalvi

University of Kansas Medical Center, Kansas City, USA

**Background**: Our previous studies consistently demonstrate enhanced pulmonary vascular remodelling in HIV-1-infected individuals, simian immunodeficiency virus-infected macaques and HIV-transgenic rats exposed to illicit drugs. We reported significant perivascular inflammation around the remodelled vessels; however, the exact role of these inflammatory cells in the development of pulmonary vascular remodelling remains unknown. Our recent *in vitro* findings revealed that HIV-1-infected and cocaine (H+C)-treated human monocyte-derived macrophages (MDMs) secrete higher number of extracellular vesicles (EVs) compared to mono-treatments. We now hypothesize that dual hit of HIV-1 and cocaine may alter miRNA cargo of macrophage-derived EVs in a way that promotes smooth muscle proliferation.

**Methods**: EVs were isolated by ultracentrifugation from supernatants collected from HIV-1Bal infected and cocaine (H+C)-treated MDMs at 4 days post-infection and used for analysis of miRNA expression. We selected five PI3/AKT signalling-associated miRNAs for analysis based on small RNA seq findings. Human primary pulmonary arterial smooth muscle cells (HPASMCs) were treated with EVs or MDM supernatants followed by proliferation assay.

**Results**: We observed significant increase in the expression of miR130a and 27a in EVs derived from H+C-treated MDMs compared to untreated group with significantly elevated miR130a levels in H+C EVs when compared to only HIV-1 or only cocaine mono-treatments. Examining the effect of EVs on HPASMCs showed that both mRNA and protein expression of PTEN, TSC-1 and TSC-2 were significantly reduced in cells exposed to H+C EVs and this corresponded to increased activation of PI3K-AKT signalling and proliferation of smooth muscle cells. Furthermore, inhibition of miRNA130a in HPASMCs with antagomir-130a blocked the EV-mediated decrease in PTEN mRNA expression, thus confirming direct role of miR130a in modulating PTEN expression and therefore potentiating the PI3/AKT signalling-mediated cell proliferation.

**Summary/conclusion**: In summary, our findings suggest a pivotal role of EVs derived from HIV-1-infected and cocaine-treated macrophages in modulating pulmonary smooth proliferation and this may play a crucial role in development of HIV-associated pulmonary arterial hypertension.

**Funding**: R01DA034542, R01DA042715 and R01HL129875.

Blood EV’s Roadmap Auditorium 16:30–17:15

Meet the Journal Editors Room 5 Chair: Hector Peinado; Marca Wauben16:30–17:15

Meet the National Societies and Outreach Strategies Room 6Chair: Isabel Guerrero18:30–20:00

Meet the Expert Session: RNA and EVs: What, Why and Where of their InteractionAuditoriumChair: Andrew Hill 18:30–20:00

Meet the Expert Session: Regulatory Aspects of EVs to Reach the Clinic Room 5Chair: Susmita Sahoo18:30–20:00

Poster Session PT01: EVs, Pathogens and Cross-organism Communication Parasitic Infections Chairs: Martin Jaular Lorena; Elena MercadeLocation: Exhibit Hall 17:15–18:30

PT01.01

GP63-enriched *Leishmania* exosomes concur to cutaneous leishmaniasis development

Alonso da Silva Lira Filho; Pauline Clement; Martin Olivier

McGill University, Montreal, Canada

**Background**: Protozoan parasites of the genus *Leishmania* are transmitted by the bite of infected sand flies leading to a wide range of diseases called leishmaniasis. Depending on the species involved, it can produce a self-healing wound to a potentially lethal visceral infection. Recently, we published a seminal work demonstrating that leishmanial exosomes (*Leish Exo*) were released in the lumen of the sand fly midgut and to be co-egested with the parasite during the blood meal. *Leish Exo* were found to stimulate an inflammatory response conducting to exacerbated cutaneous leishmaniasis, also it was shown that these vesicles cargo important virulence factors like GP63; Based on this, our actual aim was to analyse the impact of GP63-enriched *Leish Exo* on the modulation of macrophage inflammatory response and its infection in mice.

**Methods**: Using *Leish Exo* isolated from *Leishmania amazonensis* expressing different levels of GP63 (WT, GP63low, GP63high), we tested their capacity to induce the expression of various inflammatory cytokines (e.g. TNF, IL-6) and chemokines (e.g. CXCL2). In addition, LC-MS/MS analyses of these various Leish Exo preparations have been performed.

**Results**: Results obtained revealed that presence of GP63 differentially influences their expression in macrophages. Of interest, the presence of GP63 was confirmed and to influence the level of *Leishmania* arginase being enriched in *Leish Exo*.This latter being important in the regulation of NO activity, it was thus of further interest to test how these different Leish Exo preparations could influence the infection progression *in vivo*. Therefore, to test this, Balb/c mice were infected in their hind footpad with stationary *L. amazonensis* WT or GP63low with or without *Leish Exo* from each three groups of parasites.

**Summary/conclusion**: Data obtained from this study will be further discussed during the poster presentation, as well as the final conclusion in regard to the critical role played by *Leishmania* GP63 in *Leish Exo*.

**Funding**: This work was funded by CIHR and CNPq-Brazil.

PT01.02

Extracellular vesicles released by B-1 cells modulates macrophages response and alters the course of experimental *Leishmania* (*Leishmania*) *amazonensis* infection

Mayte dos Santos Toledo^1^; André Cronemberger-Andrade^2^; Natasha Ferraz de Campos Reis^1^; Talita Vieira Dupin^3^; Ana Claudia. Torrecilhas^4^; Patricia Xander ^3^

^1^Departamento de Ciências Farmacêuticas, Universidade Federal de São Paulo campus Diadema, Diadema, Brazil; ^2^Programa de Pós Graduação em Infectologia, Universidade Federal de São Paulo campus São Paulo, São Paulo, Brazil; ^3^Departamento de Ciências Farmacêuticas, Universidade Federal de São Paulo campus Diadema, São Paulo, Brazil; ^4^UNIFESP, São Paulo, Brazil

**Background**: B-1 cells consist in a subtype of B cells that are distinct in development, localization, phenotype and function from the majority B-cell population. Our group demonstrated that these cells are able to phagocytose *L. (L.) amazonensis* promastigotes and participate in immunity against the parasite in murine model of infection by *Leishmania (Leishmania) amazonensis*. However, the mechanisms underlying this protection have not yet been uncovered. In this study, we evaluated the release of extracellular vesicles by B-1 cells uninfected or infected with *L. (L.) amazonensis*, the role of these particles on macrophages functions and in the course of experimental infection with the parasite. 

**Methods**: B-1 cells were purified from peritoneal cavities of BALB/c mice by using antibodies anti-CD23 and anti-CD19 coupled with magnetic microbeads. Purified B-1 cells were infected with *L. (L.) amazonensis* promastigotes for 24 h. Extracellular vesicles were obtained from supernatant by ultracentrifugation. *In vitro* studies were performed with macrophages differentiated from bone marrow stimulated with EVs from B-1 cells. The experimental infection was carried out with BALB/c mice after approval of the study by the ethics and research committee of UNIFESP.

**Results**: Nanotracking analysis (NTA) and scanning electron microscopy showed that uninfected B-1 cells spontaneously released EVs but the parasite stimulated an increase in EVs releasing. The expression of the IL-6 and IL-10 cytokines was significantly higher in macrophages treated with EVs from infected B-1 cells, compared to macrophages stimulated with EVs from uninfected B-1 cells. Footpads of BALB/c mice treated with EVs from B-1 cells prior challenge with the parasites showed differences in parasite load and histological findings, as compared with mice that received no treatment.

**Summary/conclusion**: This study showed that EVs from B-1 cells can modulate macrophage activation and influence the course of experimental infection with *L. (L.) amazonensis*.

**Funding**: Supported by FAPESP, CNPq and CAPES.

PT01.03

Trypanosoma cruzi induce macrophage expression of inflammatory factors and release of EVs that modulate infection by the parasite

Andre Andrade^1^; Natalia Lima Pessoa^2^; Marco Antonio Campos^2^; Patricia Xander^3^; Rodrigo Pedro Soares^2^; Ana Claudia. Torrecilhas ^1^

^1^UNIFESP, São Paulo, Brazil; ^2^Centro de Pesquisa René Rachou, Fiocruz, Belo Horizonte, Brazil; ^3^Departamento de Ciências Farmacêuticas, Universidade Federal de São Paulo campus Diadema, São Paulo, Brazil

**Background**: The infection by the protozoan *Trypanosoma cruzi*, a parasite that cause Chagas’ disease, modulates host innate immune response. The objective of our study was to determine if during infection, or after incubation of human macrophages with parasite extracellular vesicles (EV), these cells released EVs with immunomodulatory activity or with the capacity to affect cell invasion by the parasite. 

**Methods**: We employed THP-1 lineage which were differentiated by the addition of Phorbol-12-myristate-13-acetate (PMA) for 24 h. The cells were either directly infected with the parasites, or incubated with parasite EVs, or EVs isolated from non-infected cells. mRNA was extracted from the cells and submitted to NGS transcriptome analysis. The released EVs from macrophages submitted to the different treatments were analysed by scanning electronic microscope and NTA or used to treat TLR2- or TLR4-transfected CHO cells, and these receptors’ activation is measured by expression of CD25.

**Results**: We detected 106 genes with increased expression, 15 of them related to the host pro-inflammatory response in infected cells or cells treated with parasite EVs compared to control macrophages. Concomitantly, the incubation of cells for 1, 6 and 24 h with the parasites produced an increased release of EVs from the plasma membrane of THP-1 cells. These EVs had similar size of EVs released from non-infected macrophages but only the EVs produced by infected cells, or cells incubated with parasite EVs induced preferentially TLR2 than TLR4 activation on CHO reporter cells. The TLR2 CHO cells become also more infected than TLR4 or non-transfected cells when incubated with similar amounts of EVs.

**Summary/conclusion**: Either infection or the presence of *T. cruzi* EVs induce the inflammatory responses in macrophages and the release of EVs that can modulate the innate host response through activation mainly of TLR2. These EVs also promote an increased infection. These results demonstrate an orchestration of signalling response by the release of EVs from the parasite and host cells.

**Funding**: Supported by FAPESP and CNPq.

PT01.05

A total transcriptome analysis of extracellular vesicles derived from the intestinal protozoa *Giardia intestinalis*

Bruno Gavinho^1^; Maria Amorim^2^; Izadora Rossi^3^; Diana Nunes^2^; Ingrid Evans-Osses^4^; Emmanoel Dias-Neto^2^; Marcel I. Ramirez ^3^

^1^Universidad Federal de Parana, Curitiba, Brazil; ^2^Laboratory of Medical Genomics, AC Camargo Cancer Center, São Paulo, SP, Brazil; ^3^Instituto Oswaldo Cruz-Fiocruz, Curitiba, Brazil; ^4^Instituto Oswaldo Cruz, Curitiba, Brazil

**Background**: *Giardia intestinalis* (GI) is an anaerobic protozoa and aetiological agent of giardiasis, one of the most causes of diarrheal disease in humans worldwide, infecting hundreds of millions of people every year (Tejman-Yarden, 2011).The parasite induces a loss of epithelial barrier function and damage to the enterocyte, diarrhea, lower inflammation and other symptoms. We have observed recently a release of extracellular vesicles (EVs) during the interaction with mammalian host cell (Evans-Osses, et al. 2017) that should have a role in the unclear pathogenesis of Giardiasis. Here, we have seen that EVs contain miRNAs that could modulate host cells. This work aims to characterize the role of nucleic acid contained in EVs from GI.

**Methods**: *G. intestinalis* isolate WB (ATCC 50803) culture, EV production and purification were defined as Evans-Osses et al (2017). Total RNA from purified MVs was obtained through Direct-zol™ RNA and MiniPrep (Zymo Research), and RNA-seq was obtained through illumina platform. Results were analysed with MirDEEP2.

**Results**: RNA extraction from EVs released by trophozoites contains small RNAs. Transcriptome analysis from Evs revealed that rRNA was the most abundant class of RNAs in the EVs followed by coding RNAs. RNA-seq analysis detected 50 miRNAs already described in Giardia (Zhang et al, 2009) and other candidates to novel miRNA. Mature gla-miR-13, gla-miR-33 and gla-miR 49 were the most abundant miRNA in EVs. Protein coding was the most diverse category, being the Protein 21.1 transcript, followed by high cysteine membrane protein Group 2 (HCM2) the most abundant.

In silico analyses of the known miRNA were made though BlastN searches with the seed regions of each miRNA against GI annotated coding sequences (GiardiaDB), and the highest putative targets were Protein 21.1, Kinases NEK, Variable Surface Proteins and HCM2.

**Summary/conclusion**: Here, we present for the first time the presence of nucleic acids on EVs of GI. It is possible that the abundant presence of miRNAs in GI extracellular vesicles regulates gene function of parasite or host cells genes, interfering on adhesion, proliferation and immunomodulation. Bioinformatics for target prediction on host cells are under investigation

**Funding**: Fellow from CNPq level I - Fiocruz - Capes Brazil.

PT01.06

Biodistribution and proteomics analysis of plasma-derived EVs from *Fasciola hepatica* infections

Alicia Galiano ^1^; Joan Segui-Barber^2^; Miriam Diaz-Varela^2^; Susana Garcia-Silva^3^; Maria Trelis^4^; Héctor Peinado^3^; Fernando Cantalapiedra^5^; Dolores Bernal^4^; Hernando A. del Portillo^6^; Antonio Marcilla^1^

^1^Departament de Farmàcia I Tecnologia Farmacéutica i Parasitologia, Universitat de Valéncia, Spain, BURJASSOT (VALENCIA), Spain; ^2^ISGlobal, Hospital Clínic - Universitat de Barcelona, Barcelona, Spain, Barcelona, Spain; ^3^Microenvironment and Metastasis Group, Molecular Oncology Program, Spanish National Cancer Research Centre (CNIO), Madrid, Spain, Madrid, Spain; ^4^Universitat de Valencia, BURJASSOT (VALENCIA), Spain; ^5^Centre de Salud Publica de Manises, Manises (Valencia), Spain; ^6^ISGlobal, Hospital Clínic - Universitat de Barcelona. Institute for Health Sciences Trias I Pujol (IGTP), Badalona, Spain. Catalan Institution for Research and Advanced Studies (ICREA), Barcelona, Spain., Barcelone, Spain

**Background**: Several studies have described the release of extracellular vesicles (EVs) in parasites, where they play an important role in the interaction between parasites and their hosts, and have been implicated in maintaining a balance with the immune system. Here, we identified proteins from the parasitic helminth *Fasciola hepatica* in EVs derived from plasma of naturally infected cows and analyse their *in vivo* biodistribution in a mouse model.

**Methods**: EVs from plasma of infected and non-infected cows were isolated by size-exclusion chromatography. Fractions containing EVs were identified using bead-based assays and quantified by NTA. Identification of EVs proteins was carried out after Orbitrap Fusion™ Tribrid™ Mass Spectrometry (MS) and Blast using public databases. To determine the *in vivo* distribution, EVs derived from plasma were labelled with NIR815 and administered by retroorbital injection to C57BL/6 mice. Intensity of the fluorescent signal was measurement by Odyssey Imaging System.

**Results**: About 42 proteins of *F. hepatica* were identified in infected cows by MS. Interestingly, some parasite proteins were found in cows diagnosed as free of infection, suggesting that identified proteins can be used in early diagnosis. *In vivo* distribution in C57BL/6 mice showed a rapid migration of EVs to both liver and spleen. Moreover, there were significant differences in the signals obtained with EVs from controls and healthy cows.

**Summary/conclusion**: Plasma-derived EVs from *F. hepatica* infections in cows contain parasite proteins whose biodistribution showed an early migration of exosomes to the liver and the spleen of infected mice. Identification of such parasite proteins and the interaction with the spleen should facilitate future efforts to use them as novel biomarkers of disease and therapeutic agents.

**Funding**: Supported by the Conselleria d’Educació, Cultura i Esports, Generalitat Valenciana, Valencia, Spain (PROMETEO/2016/156 to A.M.) and REDIEX-Spanish Ministry of Economy and Competitiveness to A.M., H.P., and HAP. This work received specific support from the Fundación Ramón Areces, 2014. “Investigación en Ciencias de la Vida y de la Materia”, Project “Exosomas: Nuevos comunicadores intercelulares y su aplicabilidad como agentes terapéuticos en enfermedades parasitarias desatendidas”.

PT01.07

T-lymphocytes are not involved in the preventive effect of *Fasciola hepatica* EVs in DSS-induced acute ulcerative colitis

Alicia Galiano ^1^; Javier Roig^2^; Maria Laura Sainz^3^; Maria Trelis^4^; Fernando Cantalapiedra^5^; Carlos Monteagudo^6^; Elisa Giner^4^; Rosa M. Giner^4^; M. Carmen Recio^4^; Dolores Bernal^4^; Francisco Sánchez-Madrid^7^; Antonio Marcilla^1^

^1^Departament de Farmàcia I Tecnologia Farmacéutica i Parasitologia, Universitat de Valéncia, Spain, BURJASSOT (VALENCIA), Spain; ^2^Universidad Europea de Valencia, Valencia, Spain; ^3^Centro Nacional de Investigaciones Cardiovasculares (CNIC), Madrid, Spain; ^4^Universitat de Valencia, BURJASSOT (VALENCIA), Spain; ^5^Centre de Salud Publica de Manises, Manises (Valencia), Spain; ^6^Universitat de Valencia, Valencia, Spain; ^7^Hospital de la Princesa. Madrid. Spain., Madrid, Spain

**Background**: The complexity of the pathogenesis of inflammatory bowel disease has led to the quest of empirically drug therapies, combining immunosuppressant agents, biological therapy and modulators of the microbiota. Helminth parasites have been proposed as an alternative treatment of these diseases based on the hygiene hypothesis, but ethical and medical problems arise. The identification of extracellular vesicles on those secreted products opens a new field of investigation, since they exert potent immunomodulating effects. 

**Methods**: Adult parasites were cultured *in vitro* and secreted extracellular vesicles were purified and used for immunizing both wild-type C57BL/6 and RAG1−/− mice. Control and immunized mice groups were treated with dextran sulphate sodium 7 days after last immunization to promote experimental colitis. The severity of colitis was assessed by disease activity index and histopathological scores. Mucosal cytokine expression was evaluated by ELISA. The activation of NF-kB, COX-2 and MAPK was evaluated by immunoblotting.

**Results**: Injection of extracellular vesicles from *F. hepatica* (FhEVs) ameliorated the pathological symptoms induced by DSS in C57BL/6 mice measured by disease activity index, altering pro-inflammatory molecules in the intestine (TNF-α, IL-6 and IL-17A), and interfering with both MAPK and NF-kB pathways. RAG1−/− mice treated with FhEVs showed preservation of tissue architecture whereas colitic mice displayed large disruption areas of the colonic architecture.

**Summary/conclusion**: Our results indicate that extracellular vesicles from parasitic helminths can modulate immune responses in DSS-induced colitis, exerting a protective effect that should be mediated by other cells distinct from B- and T-lymphocytes.

**Funding**: Supported by the Conselleria d’Educació, Cultura i Esports, Generalitat Valenciana, Valencia, Spain (PROMETEO/2016/156 to A.M.), Fundación Ramón Areces and REDIEX-Spanish Ministry of Economy and Competitiveness (MINECO) to A.M. and F.S.-M. F.S-M was supported by MINECO (SAF2014-55579-R), Comunidad de Madrid, Spain (INDISNET-S2011/BMD-2332), and the European Research Council (ERC-2011-AdG 294340-GENTRIS). JR is supported by a Generalitat Valenciana (Valencia, Spain) predoctoral fellowship. MLS is supported by FPI programme (Spanish Ministry of Economy).

PT01.09

Extracellular vesicles from the parasitic nematode Trichuris muris: new insights into host–parasite communications

Ramon M. Eichenberger^1^; Hasanuzzaman Talukder^2^; Matthew A. Field^3^; Phurpa Wangchuk^1^; Paul R. Giacomin^1^; Alex Loukas^1^; Javier Sotillo Javier Sotillo ^1^

^1^Centre for Biodiscovery and Molecular Development of Therapeutics, Australian Institute of Tropical Health and Medicine, James Cook University, Australia, Cairns, Australia; ^2^Department of Parasitology, Faculty of Veterinary Science, Bangladesh Agricultural University, Mymensingh, Bangladesh; ^3^Australian Institute of Tropical Health and Medicine, James Cook University, Cairns, QLD, Australia, Cairns, Australia

**Background**: *Trichuris muris* is a nematode parasite that lives in the mouse colon and has been widely used to study human whipworm infections, a parasitic disease affecting more than 500 million people worldwide. These nematodes secrete a multitude of compounds that interact with host tissues where they orchestrate a parasitic existence. Until now, there was no evidence that *T. muris* secreted extracellular vesicles (EVs).

**Methods**: We isolated EVs from the secretory products of *T. muris* after ultracentrifugation and further purification using Optiprep density gradient. We characterized the proteomic and nucleic acid (miRNA and mRNA) contents of the vesicles and used confocal microscopy to demonstrate the internalisation of parasite EVs by murine colonic organoids.

**Results**: A total of 364 proteins, including tetraspanins and other exosome markers, were identified in *T. muris* -secreted EVs. In addition, 56 miRNAs and 475 full-length mRNA transcripts mapping to *T. muris* gene models were also identified. Many of the miRNAs putatively mapped to mouse genes involved in regulation of inflammation, implying a role in parasite-driven immunomodulation. Furthermore, we demonstrated that *T. muris* EVs can be actively internalized by mouse colonic organoids, suggesting a role in host–parasite communication. Summary/conclusion: Understanding how parasites interact with their hosts is crucial to develop new control measures. This first characterization of the proteins and nucleic acids from the EVs secreted by *T. muris* provides important information on whipworm–host communication and forms the basis for future studies.

**Funding**: This work was supported by a program grant from the National Health and Medical Research Council (NHMRC) [program grant number 1037304] and a Principal Research fellowship from NHMRC to AL. RME was supported by an Early Postdoc Mobility Fellowship (P2ZHP3_161693) from the Swiss National Science Foundation. MHT was supported by an Endeavour Research Fellowship. The funders had no role in study design, data collection and analysis, decision to publish, or preparation of the manuscript. The authors declare no competing financial interests.

PT01.10

Cathepsin B cysteine protease of *L. donovani*: role in the modulation of parasitic exosomal proteins and TGF-β1 and arginase activities in macrophages

Camila dos Santos Meira; Asel Faiq Murtatha; Lashitew Gedamu

Department of Biological Sciences, University of Calgary, Calgary, Canada

**Background**: *Leishmania donovani* is an intracellular parasite that causes visceral leishmaniasis, a chronic disease with no effective treatment. Cathepsin B cysteine protease (catB) is a *Leishmania* virulence factor involved in the activation of transforming growth factor (TGF)-β1 in macrophages. Active TGF-β1 is suggested to increase *Leishmania* survival by modulating arginase activity and nitric oxide (NO) production in macrophages. Moreover, *catB* disruption was shown to induce proteome remodelling in *L. donovani*, affecting proteins secreted into exosomes. Here, we aimed to investigate the effect of catB on the expression of exosomal proteins associated with the pathogenesis of *Leishmania* and to determine the role of *L. donovani* exosomes in modulating TGF-β1 and arginase activities in macrophages.

**Methods**: In this study, we used *L. donovani* catB wild-type (wt), catB null mutants (ko) and episomally complemented catB ko (cm) parasites. Exosomes were isolated from stationary phase cultures and characterized by nanoparticle tracking analysis, transmission electron microscopy, mass spectrometry and immunoblotting against selected virulence factors (Elongation Factor (EF)-1α, Peroxidoxin (Pxn)-4 and catB). To assess the role of exosome-derived catB, J774.1 and PMA-activated U937 cells were treated with purified exosomes, and the concentration of active TGF-β1 and NO and the arginase activity was evaluated 24 and 48 h post-incubation.

**Results**: We identified a total of 787 proteins, 51% of which were shared among wt, ko and cm exosomes. We observed EF-1α, heat shock and antioxidant proteins among the most abundant proteins in each group. We also validated the exosome-based secretion of Pxn4, EF-1α and catB, and the expression of Pxn4 and EF-1α was downregulated in ko exosomes. Wt exosomes were able to cleave TGF-β1 *in vitro* and induced high levels of active TGF-β1 in J774.1 and U937 cells. Enhanced arginase activity in J774.1 cells was also detected 48 h post-incubation with wt exosomes.

**Summary/conclusion**: CatB modulates the expression of exosomal proteins that are associated with *L. donovani* pathogenesis. The high level of active TGF-β1 detected in macrophages incubated with wt exosomes points to a role for exosome-derived catB in TGF-β1 activation and arginase activity.

**Funding**: NSERC

Alberta Innovates-Technology Futures.

PT02: EVs in Reproduction and Pregnancy Chairs: Eva Colas; María Yáñez-Mó Location: Exhibit Hall17:15–18:30

PT02.01

Paving the way to the profiling of endometrial extracellular vesicles (EV)/exosomes as a source of non-invasive biomarkers for guiding the successful embryo implantation

Natasa Zarovni ^1^; Francesca Loria^1^; Alice Luddi^2^; Valentina Pavone^2^; Laura Governini^2^; Bianca Semplici^2^; Camilla Marrocco^2^; Paola Piomboni^2^

^1^Exosomics Siena, Siena, Italy; ^2^Department of Reproductive and Developmental Medicine, Siena University, Siena, Italy

**Background**: Lack of accurate synchronization with uterine receptivity is one of the major causes of recurring embryo implantation failure in patients undergoing assisted reproductive treatment (ART). Current histological and morphological methods are invariably unsatisfactory and invasive. Endometrial EVs emerged as key players in a dynamic endometrium-embryo cross-talk. Project aim is to develop a minimally invasive, affordable and patient-tailored EV molecular profile-based method, enabling the definition of the proper time window for efficient embryo transfer.

**Methods**: Upon institutional approval, we started the enrollment of fertile women and women undergoing ART and collected uterine fluid, cervical brushes, urine and serum at different points of natural cycle. EVs were isolated by peptide-based affinity and chemical precipitation coupled to EV RNA extraction and analysis. EV content was assessed by BCA assay, nanoparticle tracking analysis and ELISA. EV RNA yield and integrity were evaluated by BioAnalyzer. RTqPCR panel including EV quantification/normalization RNAs and endometrium-specific mRNAs was assessed.

**Results**: A compendium of clinically compliant preanalytical protocols is established for sample handling and intact EV recovery. Different sample types varied in relative and absolute EV content. RNase prewash did not affect EV RNA profiles; poor EV RNA yield warranted the use of an ad-hoc protocol including a preamplification of the target genes. Small set of endometrium-specific mRNAs was evaluated and proven to fluctuate according to the source sample and the functional endometrial status.

**Summary/conclusion**: Provided sample flow enables a set up of a sensitive EV-based platform for analysis of endometrium-associated mRNAs. Candidate mRNAs are currently verified in a relevant cohort to identify informative combination of a sample type and a RNA panel with a potential to drive a single embryo transfer to increase ART efficiency.

**Funding**: Funded under GFI scheme by Merck.

PT02.02

Characterization of extracellular vesicles produced by single human embryos at early stages of development

Stoyan Tankov^1^; Arina Lavrits ^2^; Freddy Lattekivi^1^; Kaarel Krjutskov^3^; Anu Sikut^4^; Sulev Koks^1^; Aneta Andronowska^5^; Andres Salumets^3^; Alireza Fazeli ^1^

^1^Institute of Biomedicine and Translational Medicine, Department of Pathophysiology, University of Tartu, Tartu, Estonia; ^2^Institute of Cell and Molecular Biology, University of Tartu, Tartu, Estonia; ^3^Competence Centre on Health Technologies, Tartu, Estonia; ^4^Tartu University Hospital’s Women’s Clinic, Tartu, Estonia; ^5^Polish Academy of Sciences, Institute of Animal Reproduction and Food Research, Warsaw, Poland

**Background**: Extracellular vesicles (EVs) are recognized as potent vehicles for intercellular communication. To date, there is little information available regarding the role of EVs during the early stages of human embryonic development. The aim of this study was to develop techniques for the recovery of EVs secreted by a single human embryo in an *in vitro* culture system. The EVs were characterized according to size, concentration and electrical surface properties (zeta potential), in order to understand the role of EVs production in human embryos for determination of their quality at early stages of development.

**Methods**: Human embryos were produced by *in vitro* fertilization (IVF) for 24 h in fertilization medium, cultured individually for 48 h (3 days) in cleavage medium and additionally 48 h in blastocyst medium (day 5). Conditioned media, at days 3 and 5 post-IVF, was collected and EVs were isolated using a series of centrifugations and size-exclusion chromatography. The size, concentration and zeta potential of EVs were characterized using a nanoparticle tracking analysis.

**Results**: Using this method of isolation, we were able to collect and characterize EVs produced by a single human embryo. Analysis confirmed the presence of EVs at early stages of development, with the concentration of EVs being higher in early blastocysts (day 5), as compared to 4–8 cell-stage embryos (day 3). Moreover, already at day 3, we were able to discriminate between embryos that were properly developing and those that were later visually determined as degrading at day 5. The data indicates that embryos following normal development at day 3 but degrading at later stages (day 5) were producing significantly higher number of EVs (with size range of 100–160 nm) compared with those developing properly at day 3 and later progressing to early blastocysts at day 5.

**Summary/conclusion**: In conclusion, we have developed a sensitive protocol for the isolation of EVs from human embryos cultured individually. We have demonstrated that human embryos secrete EVs in varying amounts and sizes during the early stages of their development. Further investigations are needed to establish EV characteristics of early human embryo as a quality marker for human clinical embryology.

**Funding**: The TransGeno project has received funding from the EU’s Horizon 2020 research and innovation programme under grant agreement no. 668989.

PT02.03

Small extracellular vesicles from follicular fluid of growing follicles from bovine are enriched of precursor miRNAs

Ana Clara Ávila; Gabriella Andrade; Felipe Perecin; Flávio Meirelles; Juliano C. da Silveira

Department of Veterinary Medicine, Faculty of Animal Sciences and Food Engineering, University of São Paulo, Pirassununga, Brazil

**Background**: Extracellular vesicles (EVs) from follicular fluid (FF) are involved in intercellular communication within follicle environment. EVs contain microRNAs (miRNAs), which can modulate cellular responses by mature miRNAs acting with the silencing complex. Mature miRNAs are originated by primary and precursor miRNAs. The objective of this study was to evaluate relative levels of mature and precursor miRNAs in small EVs in ovarian FF from bovine growing follicles. 

**Methods**: Slaughterhouse ovaries were collected in pairs and classified in early estrus cycle by corpus luteum appearance and progesterone concentration. Follicles between 3 and 6 mm were aspirated. FF (*n* = 4) was centrifuged at 300× g and 2000× g for 10 min and 16,500× g for 30 min to pellet cells and cellular debris. FF was filtrated (0.22 µm) and then centrifuged at 11,9700× g for 70 min twice. The EVs pellet was resuspended and used to characterize EVs as well as RNA analysis. For this, reverse transcription for mature miRNAs was obtained using miScript HiSpec Bufffer while precursor and mature miRNAs were obtained using miScript HiFlex Buffer. Relative levels of 384 miRNAs were determined by real-time PCR using miR-99b as normalizer. Differences in relative levels of mature and precursor/mature miRNAs were determined by Student’s *t*-test, considering significant *p*-value of ≤0.05.

**Results**: Progesterone concentration in FF samples was 63.62 ± 6.79 ng/ml, confirming the early estrus cycle stage. EVs from FF presented a size (113.8 ± 4.70 nm) and concentration mean (5.2 × 1011 ± 1.03 × 1011 particles/ml) corresponding to small EVs. A total of 165 mature miRNAs were found in these small EVs, while 330 precursor/mature miRNAs were found in the same EV samples. When mature and precursor/mature miRNA forms were compared, we observed 142 differently detected, and from these, 140 miRNAs were upregulated in precursor/mature miRNA forms. These different miRNAs are involved in regulation of signalling pathways like PI3K-Akt, RNA transport, oocyte meiosis and TGF-beta.

**Summary/conclusion**: Based on the results, we identified that small EVs from FF of growing follicles are enriched of precursor miRNAs forms, suggesting its role in intercellular molecular regulation.

**Funding**: FAPESP (grant numbers 2014/22887-0; 2015/ 21829-9; 2017/02037-0).

PT02.04

Differential expression of miRNAs in urinary extracellular vesicles from pregnant women with and without preeclampsia

Muthuvel Jayachandran; John Lieske; Pritha Chanana; Vesna Garovic

Mayo Clinic Rochester, Rochester, USA

**Background**: The pathophysiology of preeclampsia (hypertensive pregnancy disorder with proteinuria) remains elusive. Emerging studies show that extracellular vesicles (EVs) containing miRNAs regulate physiologic and pathophysiological processes, since the potential role of miRNAs in the pathophysiology of preeclampsia-related renal injury is largely unknown. This study compared the EV-associated miRNAs between pregnant women with and without preeclampsia.

**Methods**: Bio-banked cell-free urine samples from pregnant women without (*n* = 5) and with (*n* = 5) preeclampsia were used in this study. Urinary EVs were isolated by ExoQuicKTc and miRNAs were quantitated using an XRNA Exosome RNA-Seq Library Kit (System Biosciences, Palo Alto, CA). Differentially expressed miRNAs with a *p*-value 0.05 or lower were selected for pathway analysis.

**Results**: A group of miRNAs that contribute to glomerular and tubular injury, ischemia perfusion injury, oxidative stress, cell proliferation and growth, acute kidney injury, renal fibrosis, inflammatory processes and hypertension were increased 8- to180-fold in preeclampsia women including miR-18, miR-92, miR-126, miR-143, miR-155, miR-194, miR-194, miR-199, miR-204, miR-378, miR-429, miR-451, miR-454, miR-664, miR671, miR-754, miR-4516 and miR-4488, whereas miRNAs that contribute to tumour suppression, decreased cell proliferation, migration, and invasion, anti-inflammation, regulation of kidney progenitors and osteoblast differentiation were decreased 4- to 42.2-fold in preeclampsia women including miR-30b, miR-95, miR106, miR203, miR365, miR-412, miR-432, miR-3679 and miR3960.

**Summary/conclusion**: Our previous studies demonstrated that glomerular podocyte damage was greater in preeclampsia compared to normotensive pregnant women. The differential expression of specific miRNAs associated with urinary EVs that we identified may provide new insights into the mechanisms of renal injury in preeclampsia, and suggest new biomarkers for screening, diagnosis and risk stratification of preeclampsia.

**Funding**: NIH AG44170; U54DK083908; Mayo Clinic O’Brien Urology Research Center (U54 DK100227).

PT02.05

Heterogeneity of cell-free foetal DNA in different types of placenta-derived extracellular vesicles

Matthew Kang ^1^; Julie Wang^1^; Lawrence W. Chamley^2^

^1^Department of Obstetrics and Gynaecology, University of Auckland, Auckland, New Zealand; ^2^University of Auckland, Auckland, New Zealand

**Background**: The placenta is a foetal organ. The placental surface is bathed in maternal blood and is lined by a single multinucleated cell, the syncytiotrophoblast, which has a surface area of 11–13 m^2^ at the end of pregnancy. During pregnancy, the syncytiotrophoblast sheds three sizes of extracellular vesicles (EVs) into the maternal blood: macro-, micro- and nano-EVs. These EVs have been shown to carry the cell-free foetal DNA (cffDNA) in the maternal circulation that is detected in non-invasive prenatal testing. We hypothesized that there is heterogeneity in the cffDNA carried by the three different types of placental EVs.

**Methods**: Placental explant culture system was used to obtain placenta-derived EVs (*n* = 5). Sequential centrifugation was used to isolate macro-, micro-, nano-EVs, as well as retaining the final supernatant. Qubit and Tapestation analyses were performed to quantify and qualitate the fragment sizes of cffDNA extracted from each fraction.

**Results**: The quantity of DNA (normalized to the weight of the donor placental explant) was different for each type of placental EVs: macro-EVs, which contain intact nuclei, yielded 0.16 ng/mg explant, micro-EVs 0.15 ng/mg explant, nano-EVs 0.38 ng/mg explant and supernatant 0.54 ng/mg explant. DNA fragment lengths were also different between the four fractions: macro-EVs contained large DNA in the range of 13–29 kb, micro- and nano-EVs contained up to four sizes ranging from large fragments (9–12 kb) to several smaller fragments (411–468, 688–733, 989–1120 bp) and the supernatant contained only small fragments (173–177, 404–473, 769–1070 bp).

**Summary/conclusion**: The different fragment lengths of cffDNA in macro-, micro-, and nano-EVs most likely reflect differing vesiculation routes of each EV type. The large fragment size in macro-EVs reflects the presence of multiple intact nuclei in these structures. The existence of cffDNA in the supernatant indicates that approximately half of the cffDNA is carried in EVs.

**Funding**: Marsden-funded project.

PT02.06

Morphology characteristics and miRNA of extracellular vesicles secreted during blastulation discriminate competent bovine blastocysts

Edwin A Mellisho^1^; Fidel Ovidio Castro^1^; Lleretny Rodríguez-Alvarez ^2^

^1^Universidad de Concepcion, Chillan, Chile; ^2^Department of Animal Science. Faculty of Veterinary Sciences. Universidad de Concepcion, Chillan, Chile

**Background**: Embryo development is a complex process that relies on the bidirectional interaction between the embryo and the maternal side. It has been seen that embryos secrete extracellular vesicles (EVs) that might participate in this interaction. Here, we hypothesized that morphological characteristics and miRNA content of secreted EVs will vary depending on embryo quality.

**Methods**: Bovine embryos were produced by *in vitro* fertilization, cultured in groups (25 zigote per well) until day5 (morulae stage). Morulae were selected and individually cultured in EVs depleted media until day7.5. Embryos were monitored and the formation of blastocele verified (early blastulation at day 6.5 (EB) and late blastulation at day 7.5 (LB)). At day 7.5, culture medium was collected, blastocysts were transferred to fresh media until day11 to assess their post-hatching competence. At day 11, blastocysts were classified as more competent (MC); size > 270 μm; non-competent (NC); size < 160 μm. Culture media from day7.5 were retroactively grouped as G1:EB-MC (*n* = 73); G2:EB-NC (*n* = 68); G3:LB-MC (*n* = 61) and G4:LB-NC (*n* = 52).The EVs from culture media were analysed using a nanoparticle tracking analysis and the miRNA cargo determined by smallRNA sequencing.

**Results**: Early blastocysts had a higher probability of post -atching development (EB: 39.3%; LB: 10.5%; *p* < 0.001). Embryos classified as EB-MC secreted EVs with higher mean size (G1: 123, G2: 81: G3: 87, G4: 90 nm; *p* < 0,001) while EB-NC had the higher concentration of EVs (G1: 21, G2: 57, G3: 25, G4: 22 × 108 xml; *p* < 0,001). Cluster and principal component analysis of concentration and size of EVs allowed to discriminate MC and NC embryos in EB (G1 and 2) but not in LB (G3 and 4). Total RNA yield was higher in NC embryos (not statistically: G1: 12, G2: 20, G3: 2, G4:16 ng/μl) but similar abundance of microRNA was obtained in all groups. Several miRNAs from EVs were different among groups with fold change bigger than 2 (*p* < 0.05; FDR < 0.05). The more significant miRNAs were miR-124a, miR-124b, miR-2334, miR-2382-3p, miR-let7g and miR-425-5p.

**Summary/conclusion**: Characteristics and miRNA cargo of EVs secreted during blastulation discriminate embryos with different developmental competence and may be a non-invasive tool for embryo selection.

**Funding**: Grants Fondecyt 1170310 and Corfo 17Cote-72437, Government of Chile.

PT02.07

Effects of temperature on placental extracellular vesicle characteristics

Cherie Blenkiron ^1^; Julie Wang^2^; Matthew Kang^2^; Lawrence W. Chamley^3^

^1^Department of Molecular Medicine and Pathology, University of Auckland, Auckland, New Zealand; ^2^Department of Obstetrics and Gynaecology, University of Auckland, Auckland, New Zealand; ^3^University of Auckland, Auckland, New Zealand

**Background**: Enzymatic treatment of isolated extracellular vesicles (EV) is often required in order to deplete a preparation of contaminating protein aggregates or extra-vesicular nucleic acids. Many of these reactions have extended heating steps which we hypothesized could interfere with EV stability. Here, we assessed the effects of heat treatments on the size and number of EVs isolated from placental tissues.

**Methods**: EVs were isolated from 24 h placental explant culture media (*n* = 5) by sequential centrifugation at 2000 g (debris discarded), 20,000 g for Micro- (150–500 nm) and 100,000 g for nano-EVs (20–150 nm). Isolated EVs were treated at a range of temperatures prior to analysis by nanoparticle tracking analysis (analysed at different threshold and cameral level settings for micro- and nano-EVs) or transmission electron microscopy (TEM, as a holistic size snapshot).

**Results**: Heating of micro- and nano-EVs at 25°C, 37°C, 56°C, 70°C and 90°C did not change the mean and mode sizes (nm) significantly. However, the range of sizes seen for the Micro-EV broadened at the higher two temperatures and nano-EV trended towards increases in mode size from 56°C upwards. The concentration of micro- and nano-EV (per gram of donor placenta) dropped significantly after heating at 90°C but only the micro-EVs were affected at a lower 70°C treatment. Single-vesicle characterization by TEM at 70°C showed that the micro-EVs become more variable in size (46–352 nm at 25°C and 55–676 nm at 70°C), whereas nano-EVs become larger (from mean 126 nm, range 39–377 nm at 25°C up to mean 196 nm, range 47–571 nm at 70°C) suggesting that particle fusion may occur in the latter.

**Summary/conclusion**: Heating causes instability of placental micro- and nano-EVs, particularly at higher temperatures. These effects may also occur in EVs from other sources. We caution that isolation/purification procedures requiring heating can affect the stability and therefore the behaviour of EVs in downstream molecular or functional assays.

**Funding**: Marsden Fund.

PT02.08

Maternal serum extracellular RNA as noninvasive biomarkers associated with abnormally invasive placenta

Victoria Fratto^1^; Srimeenakshi Srinivasan ^1^; Cuong To^1^; Peter De Hoff^1^; Vy Tran^1^; Allison O’Leary^2^; Melissa Westermann^3^; Mary Norton^2^; Deborah Wing^3^; Gladys Ramos^1^; Louise C. Laurent^1^

^1^University of California San Diego, San Diego, USA; ^2^University of California San Francisco, San Francisco, USA; ^3^University of California Irvine, Irvine, USA

**Background**: Use of ultrasound and magnetic resonance imaging to diagnose abnormally invasive placenta (AIP) is costly, imprecise and requires specialized training. Extracellular RNAs (exRNAs) secreted under both physiological and pathological conditions regulate gene expression post-transcriptionally. Recent studies have focused on the potential use of exRNAs as biomarkers in various human diseases including placental disorders. Here, we hypothesize that levels of specific miRNA in the maternal blood will differ among women with AIP, previa and normal placentation (NP) and could be used as biomarkers in predicting and/or monitoring these conditions.

**Methods**: Sixty women with suspected AIP (17), previa (15) or NP (28) were prospectively recruited. AIP was confirmed by pathologic evaluation. RNA was extracted from maternal serum using miRNeasy micro kit and subjected to small RNA sequencing using the NEBNext small RNA Library Preparation kit. The percent abundance of miRNA, piRNA, and tRNA and rRNA fragments, and levels of individual miRNAs were compared. Chi square, Kruskal–Wallis, Mann–Whitney U, and Fishers Exact tests were used as appropriate. Differential Rank Conservation (DIRAC) was used to identify pairs of miRNAs that were inversely correlated in NP and AIP.

**Results**: The median gestational age at sample collection was 30 weeks and 3 days and did not differ among groups (*p* = 0.13). The abundance of total miRNA reads as a percentage of all reads in the small RNA sequencing data was highest among women with AIP and lowest in NP. DIRAC analysis identified pairs of miRNAs that had inversely correlated expression in AIP and previa, as well as AIP and NP and was validated by qPCR.

**Summary/conclusion**: Thus, we believe that exRNA from maternal serum have the potential to serve as biomarkers for accurate antenatal diagnosis of AIP. Studies in larger cohorts for validation of these results are needed.

PT02.09

Analysis of exosome concentration in blastocyst culture media by microfluidic resistive pulse sensing correlates with embryo implantation capacity: a pilot study

Jean-Luc Fraikin ^1^; Marcy Maguire^2^; Franklin Monzon^1^; Richard Scott^2^

*^1^Spectradyne LLC, Torrance, USA; ^2^IVI-RMA Global, Basking Ridge, USA*

**Background**: Advances in *in vitro* fertilization have allowed top-rated fertility clinics to promise an approximately 70% chance of live birth from transfer of a single euploid embryo. Despite these great improvements, approximately one third of euploid embryos fail to implant.

Exosomes have recently been suggested to play roles in embryo implantation. However, because embryos are grown in a low volume of complex media (typically < 25 µl), accurate quantification of exosomes in embryo culture has been challenging. In this early-stage pilot study, microfluidic resistive pulse sensing (MRPS) was used to predict embryo implantation by quantifying exosomes in the spent culture media of 20 human embryos.

**Methods**: Informed consent was obtained for use of materials in this study. Spent media from blastocysts grown in single culture was collected and stored at −80°C. Spent media from 10 embryos that successfully implanted and 10 embryos that failed to implant were submitted for blinded analysis by MRPS. Samples were thawed to room temperature and 3 µl taken from each for analysis. Total nanoparticle concentration was measured over the size range 250–2000 nm diameter and was used to predict pregnancy outcome using a threshold established from the data. As a preliminary assessment of variability in the MRPS measurements, one sample was measured in triplicate.

**Results**: MRPS analysis predicted pregnancy outcome with 80% sensitivity and 80% specificity. Particle concentration showed an approximate power-law dependence on size in each sample. Total nanoparticle concentration across samples clustered in two groups spanning approximately 1.2 E7 to 7.3 E7 particles/ml, with higher concentration in media from successfully implanted embryos. Preliminary assessment of variability in concentration measurements using one sample showed CV < 3%.

**Summary/conclusion**: In this pilot study, exosome concentrations in spent culture media measured by MRPS correlated strongly with embryo implantation potential – a tantalizing result. However, more in-depth validation is required, and consistency of measurement results must still be demonstrated more broadly. If these metrics can be satisfied, MRPS could prove a valuable tool in predicting embryo implantation potential.

PT02.10

Isolation and characterization of human seminal plasma exosomes: vehicles involved in spermatozoa motility properties and capacitation

Valentina Murdica^1^; Greta Cermisoni^2^; Alessandro Bartolacci^2^; Elisa Giacomini^2^; Alessandra Alteri^2^; Natasa Zarovni^3^; Andrea Salonia^4^; Paola Viganò^2^; Riccardo Vago ^4^

^1^Urological Research Institute, IRCCS San Raffaele Scientific Institute, Milan, 20132, Italy, Miano, Italy; ^2^Reproductive Sciences Laboratory, Division of Genetics and Cell Biology, IRCCS San Raffaele Scientific Institute, Milano,20132, Italy, Milano, Italy; ^3^Exosomics Siena, Siena, Italy; ^4^Urological Research Institute, IRCCS San Raffaele Scientific Institute, Milan, 20132, Italy, Milano, Italy

**Background**: Infertility affects men and women equally, and sperm number, morphology and motility play a critical role in the fertilization process. Thus, an impaired sperm motility (a condition called asthenozoospermia) is a common cause of male infertility. Recently, a certain attention has been directed to the role of exosomes in spermatozoa maturation and in conferring overall fertilization capacity by the transfer of key molecules along the male reproductive tract.

**Methods**: The study was approved by the institutional ethical committee and informed consent was obtained by patients. We collected seminal plasma from normozoospermic and asthenozoospermic patients, isolated and characterized exosomes by nanoparticle tracking analysis, transmission electron microscopy and western blotting. The uptake of labelled exosomes by spermatozoa was monitored by immunofluorescence and flow cytometry. The effect of exosomes on spermatozoa was determined by the evaluation of progressive motility and capacitation, the latter assessed by tyrosine phosphorylation and acrosome reaction.

**Results**: Human seminal plasma contains a discrete population of exosomes displaying canonical protein markers such as CD9, CD63, Alix and TSG101. In addition, they carry proteins involved in the spermatozoa maturation and fertilization capacity and in the mechanism of anti-oxidative protection. Exosomes can be captured by sperm cells indicating that they are still receptive even after ejaculation and can continue to receive vesicle-delivered cargos. The uptake of exosomes isolated from normozoospermic patients improved progressive motility and capacitation of spermatozoa, while those from astenozoospermic men failed to exert any function.

**Summary/conclusion**: Exosomes play a strategic role in sperm maturation and capacitation along the male reproductive tract, but also after ejaculation, opening new perspectives for the assisted reproductive technology.

**Funding**: The project was funded by intramural grant program.

PT02.11

Maternal-placental messaging through extracellular vesicles is impacted by particulate air pollution exposure

Valentina Bollati^1^; Laura Cantone^1^; Simona Iodice^2^; Jacopo Mariani ^1^; Laura Pergoli^1^; Mirjam Hoxha^1^; Vincenza Dolo^3^; Nicola Persico^4^

^1^EPIGET LAB, Department of Clinical Sciences and Community Health, Università degli Studi di Milano, Milan, Italy, Milano, Italy; ^2^EPIGET - Epidemiology, Epigenetics and Toxicology Lab Department of Clinical Sciences and Community Health, University of Milan, Milan, Italy; ^3^Department of Life, Health and Environmental Sciences, University of L’Aquila, L’Aquila, Italy; ^4^Department of Obstetrics and Gynaecology ‘L. Mangiagalli’, Fondazione IRCCS Ca Granda Ospedale Maggiore Policlinico, Milan, Italy

**Background**: Growing evidences have shown that maternal exposure to particulate matter (PM) might be associated with an impaired foetal development and adverse birth outcomes but to date the biological mechanisms are still unknown. Extracellular vesicles (EVs) might be the ideal candidate to mediate the effects of PM exposure on pregnancy: potentially they could be responsible for the complex signalling occurring between the maternal tissues representing the primary target of PM exposure, such as the lungs and the developing foetus. The main objective of the present study was thus to determine the effects of short-term (day before blood drawing) and long-term (90 days) exposure to PM on EV production in a cross-sectional sample of 199 healthy pregnant women recruited at the 11th week of pregnancy.

**Methods**: Ambient concentrations PM2.5 were obtained from the regional air quality monitoring network. Size and cellular origin of plasma EVs were characterized by nanoparticle tracking analysis and flow cytometry analysis. Association between PM exposure and EVs was evaluated by multivariable regression model adjusted for age, BMI, smoking habits, season, apparent temperature and warm/cold months.

**Results**: Short-term exposure to PM2.5 (10 µg/m^3^ increase) was associated with increased release of CD14+ EVs (Δ% = +6.5%, *p* = 0.008) derived from macrophages/ monocytes, as well as hERV-w+ EVs (Δ% = +6.6%, *p* = 0.008).

Long-term exposure to PM2.5 was in turn associated with lower concentration of CD14+ EVs (Δ% = −62.5%, *p* < 0.001) and hERV-w+ EVs (Δ% = −62.7%, *p* < 0.001). In addition, CD66+ EVs produced from neutrophils were reduced (Δ% = −53.6%, *p* < 0.001).

**Summary/conclusion**: Our study sheds light on the potential mechanisms underlying the adverse effects of air pollution exposure during pregnancy. The positive effect of short-term exposure to PM is suggestive of a pro-inflammatory reaction. This reactive ability might be desensitized once the high exposure is maintained for a long period (i.e. 90 days).

**Funding**: PRIN 2015 (Italian Ministry of Research), Project INSIDE, 20152T74ZL_004.

PT02.12

Isolation and small RNA sequencing of follicular fluid exosomes from single-ovarian follicles

Brandon A. Wyse; Mugundhine Sangaralingam; Stewart Russell; Karen Menezes; Sahar Jahangiri; Isabel Wiesenfeld; Clifford L. Librach

CReATe Fertility Centre, Toronto, Canada

**Background**: The ovarian follicle is the basic female reproductive unit consisting of the oocyte, somatic cells and follicular fluid (FF). Proper signalling between these compartments is required for optimal folliculogenesis. Recently, the miRNA cargo of human FF exosomes (*folliculosomes* (FFE)) has been described as potential biomarkers for polycystic ovarian syndrome, blastocyst quality, and pregnancy outcome. However, assessing the FFE whole snRNA cargo at the single follicle level has not been explored, limiting the ability to use FFE snRNA as a predictive biomarker of *in vitro* fertilization (IVF). The aim of this study was to determine the minimal FF volume requirements to enable the assessment of snRNAs from single follicles.

**Methods**: This study was approved by the University of Toronto Ethics Board. FF was collected from individual mature follicles at ovum retrieval from 18 consenting patients. Three pools of FF were aliquoted into 4, 2, 1, 0.5 and 0.25 ml fractions and FFEs were isolated using ExoQuick. Particles were quantified using NTA, total protein, and the purity confirmed by western blotting. RNA was isolated and sequencing libraries generated using the Small RNA Kit (Norgen) and sequenced on a NextSeq. Pearson correlations were conducted to determine the impact FF input has on snRNA detection.

**Results**: A linear relationship (*r* = 0.88) was observed between the FF input volume and protein concentration (10.8–44.9 ug/ul). We observed a similar relationship with the concentration of particles, RNA yield, and CD9 and CD63 levels. We produced sequencing libraries from FFEs isolated from as little as 0.25 ml of FF. When comparing the snRNA targets between the titrations, we observed moderately strong correlations between the 4 ml input down to 0.5 ml (*r* = 0.38) but deteriorated in the 0.25 ml samples. Several of the top expressed snRNAs across all titrations have been previously implicated in folliculogenesis including miR-143, miR-30e, miR-27a and miR-146b.

**Summary/conclusion**: We were able to reliably sequence FFEs from as low as 0.5 ml of FF, which represents a quarter the volume of FF isolated from a mature follicle. This study not only allows us to interrogate the entire “small RNAome” from single follicles but also to correlate this snRNA profile with IVF outcomes associated with a given oocyte retrieved for IVF.

PT02.13

Protein profiling of extracellular vesicles from the oviductal fluid of sows before and after ovulation

Inga Weiss; Sergio E. Palma-Vera; Andreas Vernunft; Jennifer Schoen; Shuai Chen

Institute of Reproductive Biology, Leibniz Institute for Farm Animal Biology (FBN), Dummerstorf, Germany, Dummerstorf, Germany

**Background**: Extracellular vesicles (EV) present in the oviduct fluid (OF) have been suggested to deliver oviductal signals to gametes/embryo, thereby mediating the gamete/embryo–maternal crosstalk. Aim of this pilot study was to characterize the protein profile of oviductal EVs collected from sows before and after ovulation.

**Methods**: We isolated EVs from OF collected from sows in mid and late estrus (*n* = 2/group) and in metestrus (d2 post ovulation, *n* = 4), using a two-step polyethylene glycol precipitation followed by ultracentrifugation. EVs were visualized by transmission electron microscopy (TEM). BCA assay and mass spectrometry were performed to analyse their protein content. Differential protein expression analysis was performed using the R/Bioconductor package “Differential Enrichment analysis of Proteomics data” (DEP).

**Results**: TEM proved the presence of EVs (cup-shaped structure and size smaller than 150 nm) after isolation. Mass spectrometry analysis identified 1002 proteins which were expressed in all samples. The top 500 variable proteins produced cycle stage-dependent clusters after principal component analysis, which was corroborated by hierarchical clustering analysis of the differentially expressed proteins (5% FDR). Due to the low number of biological replicates, we observed a small number of differentially expressed proteins. The comparison between late-estrus (around LH peak) and metestrus showed eight up-regulated and three downregulated proteins, while the comparison between mid-estrus (before LH peak) and metestrus indicated six upregulated and six downregulated proteins. Seven significantly up-regulated proteins were detected when comparing the two estrus stages. Interestingly, upregulated proteins of the contrasts late-estrus versus mid-estrus and late-estrus versus metestrus intersected in six common proteins CRYM, RHOA, SP17, RS20, AKAP9 and ELMO3. In comparison to both other stages, HEM2 was upregulated in metestrus.

**Summary/conclusion**: These first results indicate that the protein content of oviductal EVs is dynamically adapted during the periovulatory period. These regulated EV proteins are potentially involved in gamete/embryo–maternal interactions.

**Funding**: Inga Weiss is supported by the H. Wilhelm Schaumann Foundation, Hamburg, Germany.

PT02.14

Treatment with intravenous immunoglobulin increases the level of small EVs in plasma of pregnant women with recurrent spontaneous abortions

Rikke Baek ^1^; Malene M. Jørgensen^1^; Kim Varming^1^; Ole Bjarne Christiansen^2^

^1^Department of Clinical Immunology, Aalborg University Hospital, Aalborg, Denmark; ^3^Department of Obstetrics and Gynecology, Aalborg University Hospital, Aalborg, Denmark

**Background**: Recurrent spontaneous abortion (RSA) is the cause of childlessness in 2–5% of reproducing couples. Immunological mechanisms have been proposed as an aetiology in some cases of RSA. Various forms of immunotherapy have been attempted in individuals thought to have an immunologic mechanism associated with RSA. Intravenous immunoglobulin has been tested in a placebo-controlled trial of women with RSA, and the effect of plasma small EV (sEV) phenotypes and levels were investigated during the pregnancy.

**Methods**: Twelve pregnant women with RSA who participated in the aforementioned trial were included in this study. In a blinded set-up, five of the women were given treatment with intravenous immunoglobulin and the rest were given placebo (human albumin). Venous peripheral blood (EDTA) was obtained from the women at several time points during their pregnancy.

Small EV concentration and composition were analysed by the EV Array (Jørgensen et al., 2013, JEV) using 29 selected surface markers. The antibodies used to capture the EVs included antibodies against EVs in general (CD9, CD63, CD81, Alix, Flotilin-1 etc.) and placental and immunological markers (PLAP, HLA ABC, HLA DR/DP/DQ, HLA G, FSHR, LHR, TSHR etc.).

**Results**: The first of the sequential samples (obtained before the first infusion in pregnancy week 5) from each woman were used as reference point to which the rest of the samples were normalized in order to detect the change over time. Already at the second sampling point (after 11–21 days), the level of sEVs carrying CD9 and CD81 increased massively (2–4 fold). After 30–40 days, this increase stops and remains stable during the rest of the pregnancy.

**Summary/conclusion**: A larger cohort/study is needed for increasing the statistical power. However, the tendencies are notably that the treatment with intravenous immunoglobulin has an effect on the level of sEVs in plasma.

PT02.15

The role of extracellular vesicles in mediating placental responses to maternal cellular stress 

Catherine Evans; Thomas Rice; Beate Kampmann; Beth Holder

IMPERIAL COLLEGE LONDON, London, United Kingdom

**Background**: During pregnancy, the placenta acts as the interface between the maternal and foetal circulations. The placenta sheds extracellular vesicles (EVs), including exosomes, into the maternal circulation, which interact with maternal immune cells. We have recently demonstrated that this trafficking of EVs is bidirectional, with trafficking of EVs from immune cells to the placenta. EVs shed by stressed cells can elicit a “bystander effect” in recipient cells. We therefore investigated the functional impact of EVs released by stressed monocytes on placental trophoblast cells.

**Methods**: THP-1 cells were exposed to oxidative stress by hydrogen peroxide treatment. EVs were isolated by differential centrifugation and characterized by nanosight tracking analysis. EVs were added to BeWo trophoblast cells, which were then either left unstressed, or were subjected to oxidative stress.

**Results**: Oxidative stress induced by hydrogen peroxide had no effect on EV size nor concentration. Pretreatment with EVs from stressed or unstressed cells caused a small reverse in reduction of trophoblast viability in response to oxidative stress.

**Summary/conclusion**: EVs from maternal immune cells may help increase placental resistance to oxidative stress.

**Funding**: NIHR Imperial Biomedical Research Centre

MRC The Gambia

PT03: EV-OMICS Chairs: Armando Menezes-Neto; Muller Fabbri Location: Exhibit Hall 17:15–18:30

PT03.01

A proteome-wide catalog of viable renal cell carcinoma tissue-derived EVs, towards development of cancer liquid biopsy diagnostics

Atsushi Ikeda^1^; Kentaro Jingushi^2^; Naomi Ohnishi^1^; Motohide Uemura^3^; Kazutake Tsujikawa^2^; Koji Ueda ^1^

^1^Cancer Proteomics Group, Cancer Precision Medicine Center, Japanese Foundation for Cancer Research, Tokyo, Japan, Koto-ku, Japan; ^2^Laboratory of Molecular and Cellular Physiology, Graduate School of Pharmaceutical Sciences, Osaka University, Suita, Japan; ^3^Department of Therapeutic Urologic Oncology, Graduate School of Medicine, Osaka University, Osaka, Japan, Osaka, Japan

**Background**: Early detection of cancer is one of the most fundamental strategies to improve therapeutic outcomes and reduce cancer-related mortality rate. Here, we propose a new strategy to explore targets for cancer EV diagnostics, which allowed high-purity EV isolation even from a tiny viable tissue section of early staged cancer.

**Methods**: We extracted tissue-exudative EVs (Te-EVs) from serum-free media of freshly resected renal cell carcinoma (RCC) tissues and adjacent normal tissues using ultracentrifugation method (*n* = 20). Te-EV proteome was then comprehensively identified and quantified by high-resolution LC/MS system and Expressionist proteomics server. A couple of RCC-EV specific proteins were further validated by serum EV sandwich ELISA (*n* = 104) and analysed individually for their biological significance.

**Results**: Comprehensive LC/MS analysis identified 3871 Te-EV proteins, in which 106 proteins showed significant upregulation in EVs from RCC tissue (*p* < 0.05, fold-change > 2.0) compared to those from kidney normal tissues. Particularly, azurocidin (AZU1) and TME19 exhibited highly RCC-specific load on EVs (*p* = 2.85E-3, fold change = 31.6 and *p* = 1.18E-4, fold change = 17.4, respectively). Importantly, serum EV-AZU1 level demonstrated stage-dependent escalation in EV sandwich ELISA even from stage I. AZU1-overexpressed EVs drastically collapsed vascular endothelial cell sheet structure, suggesting that EV-AZU1 may promote hematogenous metastasis of RCC (Int J Cancer, 142: 607, 2018). On the other hand, EV-TME19 directly induced transformation from patient-derived renal fibroblasts to cancer-associated fibroblasts (CAFs).

**Summary/conclusion**: Our Te-EV proteome catalog can provide lots of new and reliable insights regarding relationship between behaviours of EVs and cancer biology, which could lead to development of novel diagnostics and therapy of cancer.

PT03.02

Characterization of extracellular vesicles from glioblastoma brain tumours

Gwennan André-Grégoire; Nicolas Bidère; Julie Gavard

CRCINA - INSERM, CNRS, University, Nantes, Nantes, France

**Background**: Glioblastoma multiforme (GBM) is the most aggressive primary tumour within the brain and the most common and lethal cerebral cancer, mainly because of treatment failure. Indeed, tumour recurrence is inevitable and fatal in a short period of time. Glioblastoma stem-like cells (GSCs) are thought to participate in tumour initiation, expansion, resistance to treatments, including the alkylating chemotherapeutic agent temozolomide, and relapse. Here, we assessed whether extracellular vesicles (EVs) released by GSCs could disseminate factors involved in the resistance mechanisms.

**Methods**: We first characterized EVs both circulating in peripheral blood from newly diagnosed patients and released by patient-derived chemotherapy-resistant GSCs.

**Results**: We found that EVs were mainly composed of particles homogeneous in size (50–100 nm) and were more abundant in liquid biopsies from GBM patients, as compared to healthy donors. Further mass spectrometry analysis unveils that EVs from control and temozolomide-treated GSCs shared core components of EVs, as well as ribosome- and proteasome-associated networks. More striking, temozolomide treatment led to the enrichment of EVs in cargoes involved in cell adhesion processes.

**Summary/conclusion**: Thus, while relatively inefficient in killing GSCs *in vitro*, temozolomide could instead increase the release of pro-migratory information that might ultimately participate to GBM invasiveness.

**Funding**: Fondation de France ;

Ligue nationale contre le cancer, comité de Loire-Atlantique ;

Région Pays de la Loire et Nantes Métropole under Connect Talent Grant.

PT03.03

Proteomic and metabolomic profiling of large microvesicles for their use as cancer biomarkers

Kerstin Menck ^1^; Annalen Bleckmann^2^; Matthias Schulz^2^; Júlia Perera Bel^3^; Judith Büntzel^2^; Hanibal Bohnenberger^4^; Christof Lenz^5^; Gry Helene Dihazi^5^; Frank Streit^5^; Claudia Binder^5^

^1^INSERM, U1068, Centre de Recherche en Cancérologie de Marseille, Marseille, France; ^2^University Medical Center Goettingen, Dept. of Hematology/Medical Oncology, Göttingen, Germany; ^3^University Medical Center Goettingen, Dept. of Medical Statistics, Goettingen, Germany; ^4^University Medical Center Goettingen, Institute for Pathology, Goettingen, Germany; ^5^University Medical Center Goettingen, Dept. of Clinical Chemistry, Goettingen, Germany

**Background**: Among extracellular vesicles (EV) especially the larger microvesicles (MV, diameter 100–1000 nm) are poorly characterized, and the mechanism of their biogenesis remains largely elusive. Tumour cells are known to secrete high numbers of MV which can be detected in cancer patients’ blood through the definition of tumour-specific markers and can be used as prognostic biomarkers. The aims of this study are (1) the characterization of MV via metabolomic and proteomic profiling and (2) the comparison of MV expression profiles to smaller EVs in order to find MV-specific proteins and metabolites that could give hints about their biogenesis and to define markers that could be used for the detection of tumour MV in blood.

**Methods**: Since MV from different tumour subtypes differ in their specific profiles, this study focused on one tumour subtype which is breast cancer. Vesicles were isolated by differential ultracentrifugation (MV = 14k pellet (P14), smaller EV = 110k pellet (P110)) from human MCF7 and SK-BR-3 breast cancer cells as well as from peripheral blood of breast cancer patients and were characterized by mass spectrometry (proteomics: label-free and SILAC; metabolomics).

**Results**: Comparison of P14 and P110 by proteomics revealed more than 2000 proteins that were significantly differentially expressed between both populations. While P110 expressed high levels of tetraspanins and proteins of the Syntenin-Alix pathway, P14 showed very heterogeneous protein expression patterns with many cytoskeleton-associated proteins. Membrane proteins were often expressed in both fractions. Equally, the metabolome of P14 and P110 differed significantly, in particular regarding the lipidome. Using this metabolite profile, tumour-derived P14 could be detected at concentrations of as low as 2% in total P14 extracts from human plasma samples.

**Summary/conclusion**: These results suggest that although the proteome and metabolome of P14 and P110 are to a certain extent overlapping; thorough characterization and comparison reveals subtype-specific markers that hint to different biogenesis mechanisms. In addition, the definition of tumour-specific profiles allows the detection of tumour vesicles in complex mixtures such as human plasma and paves the way for the use of these techniques in cancer diagnostics.

PT03.04

Proteomic analysis of acute myeloid leukaemia-derived extracellular vesicles

Hyoseon Kim ^1^; Ka-Won Kang^2^; Kwang Pyo Kim^3^; Woojune Hur^4^; Yong Park^5^

^1^Kyung Hee university, Seoul, Republic of Korea; ^2^Korea university, seoul, Republic of Korea; ^3^Kyung-Hee University, Yongin, Republic of Korea; ^4^Korea university, Seoul, Republic of Korea; ^5^Korea University School of Medicine, Seoul, Republic of Korea

**Background**: Acute myeloid leukemia (AML) is a malignant disease categorized by blocking monocyte differentiation and maturation as haematopoietic cells. AML is divided into 8 subtypes according to French-American-British (FAB) classification which mainly depends on cell maturity and differentiation. Extracellular vesicles (EV) are known to perform critical physiological and pathological functions as an emerging of communication in mammalian cells. Only a few proteomic studies on subtype-specific AML have been reported. As EVs perform multifaceted pathological functions in intercellular signalling and communication, it is essential to profile and compare EV-proteome changes for understanding the pathophysiology of AML differentiation. 

**Methods**: To elucidate the proteomic characteristics of the EVs from AML, we isolated EVs from human dermal fibroblast, human bone marrow-derived mesenchyme stem cells and AML such as acute promyelocytic leukemia (HL60), acute myelomonocytic leukemia (KG-1), and acute monocytic leukemia (THP-1). Proteome profiles of isolated EVs were analysed by using liquid chromatography-tandem mass spectrometry (LC-MS/MS) analyses.

**Results**: A total of 1554 proteins were identified in all groups. It is worthy to note that the commonly identified proteins were enriched in the cellular components of extracellular exosome and membrane, and engaged in the pathways of leucocyte surface antigen as well as myeloid-associated differentiation. EV proteins from different cell types revealed differentially expressed proteins.

**Summary/conclusion**: We compared each group of proteomes and observed changes in leukocyte-genesis mechanism and proteoglycan mechanism in AML that could explain differentiation of AML from the bone marrow. Our study might help to understand the intracellular/extracellular of AML differentiation pathways that could explain physiological regulation factors in AML groups.

PT03.05

Proteomic analysis of breast cancer-derived extracellular vesicles

Stamatia Rontogianni^1^; Donna Olivia Debets ^1^; Maarten Altelaar^2^; Wei Wu^1^

^1^Utrecht University, Utrecht, The Netherlands; ^2^Biomolecular Mass Spectrometry and Proteomics Group, Bijvoet Center for Biomolecular Research and Utrecht Institute for Pharmaceutical Sciences, Utrecht, The Netherlands

**Background**: Extracellular vesicles (EVs) are released by a variety of cell types. EVs derived from cancer cells can promote cell migration, invasion, proliferation and cancer growth. They carry cell-specific proteins, RNA and lipids. This is interesting from a clinical perspective since EVs are known to circulate in a variety of bio fluids, such as blood and urine. Circulating EVs present therefore a rich source of disease biomarkers allowing the development of novel, non-invasive screening tests. In this study, we used mass spectrometry analysis to unravel the proteomic profiles of EVs derived from different breast cancer subtypes. 

**Methods**: We performed proteomic comparisons of EVs derived from different cell lines of the three main breast cancer subtype classes; clinical subtyping is based on the abundance of receptors on the cell surface. Three important receptors for subtyping are the human epidermal growth factor receptor 2 (HER2), estrogen receptor (ER) and progesterone receptor (PR). Breast cancer cells that have low abundances of all of these receptors are referred to as triple-negative breast cancer (TNBC). In this study, we used four HER2+ breast cancer cell lines, four triple negative breast cancer cell lines, one ER+/PR+ breast cancer cell line and one normal breast epithelial cell line. We isolated the extracellular vesicles by ultracentrifugation and subsequently performed LC-MS/MS analysis.

**Results**: In this study, we identified a total of 4661 vesicular proteins across the different cell lines. Proteomic analysis revealed distinct subtype-specific protein signatures, which reflect the unique biology of each subtype. For example, proteins enriched for pathways such as cell motility, migration and angiogenesis are significantly upregulated in the proteomes of the TNBC cell lines compared to the other cell lines. This is in agreement with the invasive nature of this subtype.

**Summary/conclusion**: We believe that our data set shows the biomarker potential of extracellular vesicles in the subtyping of breast cancer patients, including treatment selection and response monitoring.

PT03.06

The contribution of chronic intermittent hypoxia to OSAHS: from the perspective of serum extracellular microvesicle proteins

Huina Zhang ^1^; Xinliang Ma^2^; Yongxiang Wei^3^

^1^Beijing Institute of Heart Lung and Blood Vessel Disease, Capital Medical University, Beijing, China (People’s Republic); ^2^Thomas Jefferson University, Philadelphia, USA; ^3^Beijing An Zhen Hospital, Beijing, China (People’s Republic)

**Background**: Obstructive sleep apnea hypopnea syndrome (OSAHS) is an independent risk factor for many clinical complications and chronic intermittent hypoxia (CIH) is a main property of OSAHS. However, specific contribution of CIH to overall OSAHS-initiated pathological complications remains unclear. By utilizing an unbiased proteomic approach, current study attempted to determine whether OSAHS may alter protein profiles in serum extracellular microvesicles (SEMVs) and how CIH contribute to these alterations.

**Methods**: Tandem mass tagging (TMT)-labelled quantitative proteomics assay was used to compare the differentially expressed proteins (DEPs) in SEMVs from OSAHS patients and non-OSAHS subjects, and the same strategy of comparative proteomics study was performed in SEMVs from CIH and normoxia rats. The similarity and disparity of DEPs and DEPs-related functions predicted by bioinformatics tools were compared using two different models, and some of the DEPs were further verified by ELISA or western blotting.

**Results: About** 560 human SEMV proteins were identified by TMT-labelled quantitative proteomics assay, with 32 DEPs found in OSAHS patient SEMVs. Four of DEPs, including CRP, HP, FN1 and PF4, were further verified by ELISA and three of them (CRP, FN1 and Hp) showed significant difference between OSAHS and non-OSAHS. In rat SEMVs, 121 DEPs were identified. Among the DEPs analysed by two different models, three proteins (CRP and FN1 and F13a1) were identical with the same variation tendency in SEMVs from OSAHS patients and CIH rats, which were further verified by western blotting. Computational functional analysis further revealed the similar and different DEP-involved pathways under OSAHS and CIH status.

**Summary/conclusion**: This study provides the first evidence that OSAHS causes significant alteration in SEMV protein content, which may contribute to OSAHS-initiated multiple organ injury and organ-organ communication. Moreover, CIH was proved as the primary contributor for increased inflammatory protein in SEMV. As CRP is being increasingly recognized not only as a marker for but also function as a mediator of inflammatory response to tissue injury, increased SEMV CRP in CIH/OSAHS may play a significant role in OSAHS-induced tissue injury, suggesting SEMV CRP might be a therapeutic target against OSAHS-related complications.

PT03.07

*In vitro* characterization of cardiac extracellular vesicles involved in transport of the circulating biomarkers of heart failure miR-21-5p, miR-23a-3p and miR-222-3p

Henri Charrier ^1^; Emilie Dubois-Deruy^1^; Olivia Besème^1^; Paul Mulder^2^; Maggy Chwastyniak^1^; Philippe Amouyel^1^; Vincent Richard^2^; Florence Pinet^1^

^1^INSERM U1167 - Université de Lille Nord de France - Institut Pasteur de Lille, Lille, France; ^2^Inserm U1096 - UFR Santé Université de Rouen Normandie, Rouen, France

**Background**: After myocardial infarction (MI), 30% of patients develop left ventricular (LV) remodelling which may lead to heart failure (HF) and death. In an experimental rat model of HF induced by left coronary ligation, the circulating biomarkers miR-21-5p, miR-23a-3p and miR-222-3p are modulated in LV of HF rats (Dubois-Deruy, *Sci Reports*, 2017). *In vitro*, these miRNAs are detected in extracellular vesicles (EVs) isolated from cardiofibroblasts (EVs-Fib) and H9c2 cardiomyoblasts (EVs-H9c2). The aim of this project is to characterize the profile of these cardiac EVs.

**Methods**: We use filtration-ultracentrifugation of serum free culture medium to purify cardiac EVs-Fib and EVs-H9c2 and non-cardiac EVs from smooth muscle cells (EVs-SMC).

**Results**: Nanoparticle tracking analysis showed that EVs-Fib and EVs-H9c2 have a similar concentration (about 1010 particles/ml), a similar small size (<150 nm) confirmed by electronic microscopy but a different size distribution. Although EVs-H9c2 are enriched in RNAs compared to EVs-Fib (x18.2, *p* = 0.02), the levels of miR-21-5p, miR-23a-3p and miR-222-3p between RNAs from EVs-H9c2 and EVs-Fib are not significantly different, suggesting that EVs-H9c2 and EVs-Fib may be both involved in the transport of these miRNAs.

EVs-Fib and EVs-H9c2 express classical markers of endogenous biogenesis like CD63 and CD81 but not CD9, unlike EVs-SMC (used as control) which expresses CD9, suggesting that EVs-Fib and EVs-H9c2 have a distinct protein profile. Proteomic analysis (1D gel and Maldi-TOF/TOF) of EVs-Fib and EVs-SMC highlighted 98 proteins with 17 shared proteins and 46 proteins detected only in EVs-fib. These results suggest that EVs-fib may have cell-specific set of proteins. In silico analysis (DAVID) of these proteins predicts an exosomal origin (*p* < 6.4.10–21) for these vesicles and confirmed a role in molecular binding (p < 6.3.10–10).

**Summary/conclusion**: Small EVs from cardiac fibroblasts and myoblasts are involved in the transport of HF biomarkers miR-21-5p, miR-23a-3p and miR-222-3p. Proteomic analysis of these EVs underlined potential markers of cardiac EVs which may allow their identification in the circulation.

PT03.08

Liver-specific miRNAs are detected in exosomes from HIV/HCV patients

Óscar Brochado Kith^1^; Alicia Gómez Sanz^1^; Luz María Medrano de Dios^1^; Luz Martín Carbonero^2^; María Ángeles Jimenez Sousa^1^; Sara Monzón Fernández^3^; Isabel Cuesta de la Plaza^3^; Verónica Briz Sebastián^1^; Salvador Resino García^1^; Amanda Fernández Rodríguez ^1^

^1^Institute of Health Carlos III (ISCIII), National Center for Microbiology (CNM), Majadahonda, Spain; ^2^Servicio de Medicina Interna, Hospital Universitario La Paz, Madrid,, Majadahonda, Spain; ^3^Institute of Health Carlos III (ISCIII), Bioinformatics unit, Majadahonda, Spain

**Background**: Liver disease is the most common cause of death HIV/HCV coinfected patients. To know the progression of liver fibrosis is essential to determine prognosis. Therefore, the availability of accurate techniques to monitor liver disease progression is essential, as well as the identification of markers to predict the clinical evolution of the patients. HCV and HIV hijack exosomal machinery as an additional mechanism of infection and to evade immune system. Exosomal RNAs are involved in the transcriptional regulation of immune system and antiviral response against HIV and HCV. In addition, exosomes are essential in liver physiology, and they reflect the liver modifications that follow to liver disease progression. Thus, we aim to analyse exosome RNA profile for the identification of new liver disease biomarkers.

**Methods**: Plasma from HCV/HIV coinfected patients was used to isolate exosomes with six different isolation protocols (Plasma/Serum Exosome and Free-Circulating RNA Isolation Kit (Norgen), exosome isolation kit (Mircury), Total exosome Isolation kit (Thermo Fisher) and polyethylene glycol (PEG) of 6/10 KDa, and ultracentrifugation). The RNA was isolated with miRNeasy (Qiagen) and Plasma/Serum Circulating and Exosomal RNA Purification Kit (Norgene). High-throughput sequencing (HTS) of total RNA was performed in a HiSeq 2500 with the SeqMatic TailorMix library. miRpath v.3 was used to identify pathways involved.

**Results**: Similar miRNAs profiles were detected with all protocols, with the precipitation-based methods yielding higher number of miRNAs (especially PEG 6 and 10kDa). About 243 miRNAs were identified, 45 out of them captured in all protocols. These miRNAs mainly belong to fatty acid metabolism and cell signalling pathways. One of the most detected miRNAs was the hsa-miR-122-5p, whose expression is specific to the liver and plays an important positive role in HCV infection. Most of the miRNAs were abundantly expressed in liver (such as -let-7a-5p, miR-21-5p, miR-22-3p and miR-199a-3p) and upregulated in liver diseases.

**Summary/conclusion**: Liver-specific miRNAs were detected in plasma exosomes of HIV/HCV patients. These molecules are an excellent material to study liver disease prognosis and evolution.

**Funding**: This work has been supported by grants given by Institute of Health Carlos III (MPY-1404/15;MPY-1144/16).

PT03.09

*In situ* microarray-based detection of intravesicular proteins in exosome-like extracellular vesicles

Rosalie Martel ^1^; Philippe DeCorwin-Martin^2^; Eun Hae Oh^2^; Frederic Normandeau^1^; David Juncker^2^

^1^Biological & Biomedical Engineering Program, McGill University, Montreal, Quebec, Canada, Montreal, Canada; ^2^Biomedical Engineering Department, McGill University, Montreal, Quebec, Canada, Montreal, Canada

**Background**: Knowledge of the protein content of exosome-like extracellular vesicles (ELEVs) can be leveraged for profiling and identification of biomarkers. While transmembrane protein studies are often performed on whole ELEVs, most current methods, such as ELISA, employ lysis when looking at exosome intravesicular proteins. Here, we propose a microarray-based, minimally disruptive method that allows vesicles with specific markers to be enriched on microarray spots and probed for intravesicular proteins, making it straightforward to correlate extravesicular and intravesicular markers.

**Methods**: IgGs targeting known transmembrane exosome markers (i.e. CD63, CD9, CD81) were inkjet-printed on an aldehyde-functionalized glass slide in a microarray format. The slide was passivated with BSA and incubated overnight with size exclusion chromatography-purified ELEV samples from CD63-GFP-expressing A431 cells. After washing, the captured vesicles were fixed and permeabilized, and intravesicular proteins were detected using oligonucleotide-conjugated IgGs. Padlock probe-based rolling circle amplification and hybridization with fluorescently labelled probes was performed, followed by imaging using a fluorescent microarray scanner.

**Results**: The intravesicular GFP tag was detected in proof-of-concept experiments to validate the proposed method. The GFP detection signal of vesicles captured on antibody spots was quantified and compared with the direct GFP signal. Seven capture combinations involving antibodies against CD63, CD9 and CD81 were thus tested, and a clear correlation was shown between the GFP fluorescence and the amplified fluorescent detection signal.

**Summary/conclusion**: The intravesicular GFP tag of A431-GFP ELEVs was quantified and compared to known transmembrane markers with a method enabling signal amplification and minimal disruption. This new approach has the potential to open the way to more efficient detection of internal targets in ELEV biomarker research.

**Funding**: This work was supported by Genome Canada, the Natural Sciences and Engineering Research Council of Canada (NSERC), and the Fonds de recherche du Québec – Nature et technologie (FRQNT).

PT03.10

The extracellular RNA-Seq processing pipeline of the Extracellular RNA Communication Consortium

Joel Rozowsky ^1^; Robert R. Kitchen^2^; Jonathan Park^1^; Timur Galeev^3^; James Diao^4^; Jonathan Warrell^3^; William Thistlethwaite^5^; Sai Lakshmi Subramanian^6^; Aleksandar Milosavljevic^6^; Mark B. Gerstein^4^

^1^Yale University, New Haven, USA; ^2^Exosome Diagnostics, Boston, USA; ^3^Department of Molecular Biophysics & Biochemistry, Yale University, New Haven, USA; ^4^Yale, New Haven, USA; ^5^Baylor College of Medicine, Houston, USA; ^6^Department of Molecular & Human Genetics, Baylor College of Medicine, Houston, USA

**Background**: We will present the tools of the Extracellular RNA Communication Consortium that have been developed for the analysis of extracellular RNA-Seq data and have been used in the construction of a comprehensive atlas of exRNAs in human body fluids. Due to the process of extracting, purifying and sequencing of RNA from extracellular biofluids, exRNAs are more vulnerable to contamination than cellular RNA samples. To address this, we have developed the extracellular RNA processing tool (exceRpt), optimized for the analysis of exRNA-seq data.

**Methods**: This involves three steps: (1) pre-processing: support for random-barcoded libraries, spike-in sequences for calibration or titration, and explicit removal of common laboratory contaminants and ribosomal RNAs. (2) Endogenous processing: alignment and quantitation of the full set of annotated, potentially spliced, endogenous RNA transcripts including all known miRNAs, tRNAs, piRNAs, snoRNAs, lincRNAs, mRNAs, retrotransposons and circular RNAs. (3) Exogenous processing: alignment to annotated exogenous miRNAs in miRBase and exogenous rRNA sequences in the RDP and alignments to the full genomes of all sequenced bacteria, viruses, plants, fungi, protists, metazoa and selected vertebrates.

**Results**: We have developed a novel algorithm for characterizing alignments to all available exogenous genomes in terms of the NCBI taxonomy tree. Existing approaches that remove degenerate sequences (i.e. those that co-occur across multiple species) result in a loss of potentially valuable data as the occurrence of reads aligning to multiple species/strains is very frequent. This is done independently for exogenous reads aligning to exogenous rRNA as well as exogenous genome sequences.

**Summary/conclusion**: The exceRpt pipeline (available at genboree.org and github.gersteinlab.org/exceRpt) generates a variety of sample-level quality control metrics, produces abundance estimates for various RNA biotypes, including detailed reports of this processing. The exceRpt pipeline (including endogenous and exogenous processing steps) has been used to uniformly process all ~2500 exRNA-Seq data sets that are in the ERCC exRNA Atlas (exrna-atlas.org). We will also present the quality control metrics as applied to the current available extracellular RNA-Seq data sets of the ERCC in the exRNA Atlas.

**Funding**: NIH Common Fund.

PT03.11

Integrating long and short sequencing data for a global overview of the endothelial extracellular vesicle RNA landscape

Tina O’Grady ^1^; Makon-Sébastien Njock^2^; Michelle Lion^1^; Ingrid Struman^3^; Franck Dequiedt^4^

^1^Laboratory of Protein Signaling and Interactions, GIGA-R (Molecular Biology of Diseases), University of Liège, Liège, Belgium; ^2^Laboratory of Molecular Angiogenesis, GIGA-R, University of Liège, Belgium, Liège, Belgium; ^3^Laboratory of Molecular Angiogenesis, GIGA-R (Cancer), University of Liège, Liege, Belgium; ^4^Laboratory of Protein Signaling and Interactions, GIGA centre, University of Liège (Belgium), Liège, Belgium

**Background**: Although extracellular vesicles (EVs) are well known to be enriched in miRNAs and other short RNA species, long RNA transcripts such as mRNA and lncRNA have been reported by several groups. We use RNA-Seq analysis in conjunction with wet bench techniques to provide a global analysis of the RNA content of endothelial cell EVs.

**Methods**: EVs from primary human umbilical vein endothelial cell (HUVEC) cultures were isolated by sequential ultracentrifugation and immunocapture. RNA was extracted from cells and EVs, then short and long RNA libraries were prepared and sequenced. Reads were mapped to the human genome and transcriptome and mapped reads were analysed to determine transcript type and differential abundance between cells and EVs. RT-PCR was used to investigate integrity of long RNA transcripts. Gene ontology analyses were performed to determine enrichment of functional terms.

**Results**: RNA in primary endothelial EVs is highly diverse in terms of length, type and abundance. As expected, endothelial EV RNA content is dominated by short RNA molecules, in particular snRNA and piRNA. Additionally, long rRNA, mRNA and lncRNA transcripts are present. Many of these transcripts are intact, putatively functional transcripts and are detectable at robust levels. Analysis of differential abundance between EVs and cells reveals significant differences in miRNA, snRNA, piRNA, mRNA and lncRNA profiles. LncRNAs in particular show a striking distribution, with about 13 times more lncRNA transcripts being enriched in EVs than in cells. Few of these lncRNAs have been fully functionally characterized, but gene ontology analysis of EV-enriched mRNA transcripts reveals an overabundance of genes coding for ribosomal proteins, elongation factors and other translation-related proteins.

**Summary/conclusion**: Endothelial EVs are enriched for short and long regulatory RNA with the potential to control gene expression at both transcriptional and post-transcriptional levels.

**Funding**: Grants from the Belgian National Fund for Scientific Research (FNRS) and ULiège.

PT03.12

Improving specificity and diagnostic power in extracellular vesicles – Easily incorporate isomiR analyses in existing pipelines

Benedikt Kirchner ^1^; Dominik Buschmann^1^; Gustav Schelling^2^; Anja Maria. Gumpp^3^; Alexander Karabatsiakis^3^; Iris-Tatjana Kolassa^3^; Michael W. Pfaffl^1^

^1^Division of Animal Physiology and Immunology, School of Life Sciences Weihenstephan, Technical University of Munich, Germany, Freising; ^2^Department of Anesthesiology, University Hospital, Ludwig-Maximilians-University Munich, Germany, München; ^3^Clinical and Biological Psychology, Institute of Psychology and Education, Ulm University, Ulm, Germany

**Background**: The untapped potential of miRNA isoforms (isomiRs) to uncover important aspects of EV physiology has recently been demonstrated by a number of publications. Although a few isomiR detection tools exist, their universal applicability is unfortunately limited as restriction to specific species, aligners or isoforms and inflexible work flows make comparison of results across studies difficult. To facilitate isomiR analyses in all kinds of study backgrounds, we developed a small suite of bioinformatic tools that can be easily incorporated in any pipeline. Starting from any set of reference miRNA sequences, isomiRs derived from up to 3nt additions and 6nt trimmings on 5' and 3' ends in any combination as well as polymorphic isomiRs can be detected from NGS data.

**Methods**: To highlight the limitations of conventional miRNA analysis and the potential advantages of isomiRs, we sequenced small RNA in whole blood and serum EVs from healthy volunteers and patients suffering from depression (*n* = 16 each). Expression profiles of miRNAs and isomiRs were generated by incorporating our bioinformatic suite into a conventional analysis pipeline.

**Results**: Despite detecting copious miRNA reads and clear differences between whole blood and extracellular samples, conventional analysis failed to discriminate between volunteers and patients, and could not establish any statistically significant changes in gene expression across groups. Analysing isomiRs, on the other hand, not only increased mapping output and effect sizes (fold change), but also detected 62 differentially expressed isoforms. Moreover, isomiRs in EVs, but not whole blood, allowed for the construction of a statistically cross-validated model that classified healthy and depressive individuals with high confidence (AUC of ROC: 0.9961). Additionally, isomiR expression distinguished patient subgroups with moderate and severe depression from volunteers (AUC of ROC: 0.9852 and 0.9913, respectively).

**Summary/conclusion**: The separation of single miRNAs into individual isoforms not only increases the potential pool of biomarker candidates in itself, but also strengthens discrimination between study-related signal and noise. With the help of our bioinformatic suite, researchers can seamlessly integrate isomiR analyses in their existing workflows with minimal effort and maximum comparability.

PT03.13

Determination of biological and technical variability at protein level in isolated urinary extracellular vesicles of healthy individuals

Eline Oeyen ^1^; Inge Mertens^2^; Hanny Willems^3^; Lucien Hoekx^4^; Stefan De Wachter^4^; Filip Ameye^5^; Geert Baggerman^2^

^1^University of Antwerp/ VITO, Mol, Belgium; ^2^University of Antwerp, Antwerp, Belgium; ^3^VITO, Antwerp, Belgium; ^4^University Hospital of Antwerp, Antwerp, Belgium; ^5^Hospital Maria Middelares Ghent, Ghent, Belgium

**Background**: The origin and function of extracellular vesicles (EVs) and their presence in easily accessible body fluids render EVs a promising potential as source of biomarkers. The cargo of urinary EVs provide a targeted view into the urogenital tract to enhance the ability to detect urological diseases or tumours as they are released by the epithelia of the complete urogenital tract. In biomarker discovery studies, determining the variability is necessary for a correct experimental design with sufficient statistical power. This results in biologically significant disease-specific differential proteins. We determined the variability at protein level in urinary EVs of healthy individuals aged above 50 years.

**Methods**: Urine samples of healthy individuals were collected and in compliance with the Declaration of Helsinki. Informed consent was obtained and the study was approved by the medical ethics committee of the University Hospital of Antwerp. Different experimental set-ups of variation were used to determine the total variation of urinary EV proteins which includes the inter-individual variation, intra-individual variation and the technical variation.

**Results: Seventy-five percent** least variable peptides of the total variation set-up with a two-sided 0.001 significance level with 90% power leads to a standard deviation of 1.35. This value can be used to calculate the sample size with a giving fold change to result in significant disease specific differential proteins.

**Summary/conclusion**: To avoid false discoveries driven by underpowered quantitatieve proteomics experiment, it is essential to determine the global variation in real clinical samples. We determined the variability arising from biological and technical variation of isolated urinary EVs. We concluded that the 75% least variable peptides of the total variation lead to a standard deviation of 1.35 which can be used for power calculation.

**Funding**: This PhD research was funded by the Flemish Institute for Technological Research.

PT03.14

Unravelling the mechanism of action of milk-derived EV by linking their proteome to relevant signalling pathways using an unbiased comprehensive bioinformatics approach

Martijn J.C. van Herwijnen ^1^; Marijke I. Zonneveld^2^; Soenita Goerdayal^3^; Esther N.M Nolte-’t-Hoen^4^; Johan Garssen^5^; Maarten Altelaar^3^; Frank A. Redegeld^5^; Marca H.M. Wauben^4^

^1^Utrecht University, Utrecht, The Netherlands; ^2^Autophagy lab, department of Radiotherapy, GROW - school for Oncology & Developmental Biology, Maastricht University, Maastricht, The Netherlands; ^3^Biomolecular Mass Spectrometry and Proteomics Group, Bijvoet Center for Biomolecular Research and Utrecht Institute for Pharmaceutical Sciences, Utrecht, The Netherlands; ^4^Department of Biochemistry & Cell Biology, Faculty of Veterinary Medicine, Utrecht University, Utrecht, The Netherlands, Utrecht, The Netherlands; ^5^Division of Pharmacology, Department of Pharmaceutical Sciences, Faculty of Science, Utrecht University, Utrecht, The Netherlands, Utrecht, The Netherlands

**Background**: EV are multisignaling components and their functionality is likely to occur from the combined actions of their constituents, rather than single molecules. Upon deciphering their functional effects *in vitro*, the major challenge is to define which molecules are responsible for their mode of action. Previously, we have published the human milk-derived EV proteome and we have shown that milk-EV can enhance epithelial cell migration on the one hand and suppress T-cell activation on the other hand. While various bioanalytical approaches are available that reveal the general functions of proteins in a data set, none of these show comprehensive signalling cascades. In this study, we aimed to select and combine the relevant tools to reconstruct the mechanism of action of milk-EV.

**Methods**: Protein–protein interaction networks were made from the common milk-EV proteome (367 proteins) using STRING. Functional enrichment analysis in STRING was used to determine which proteins were involved in any of the relevant biological processes studied in the *in vitro* assays. Then, the signalling pathways were constructed using Uniprot entries and their associated sources from the individual proteins, supplemented with a general literature search (including KEGG and MetaCore pathways). Finally, interacting proteins were linked to these pathways.

**Results**: Interestingly, individual proteins and protein clusters could be linked to specific signalling events and their function (activation or inhibition) fitted the observed *in vitro* data. For instance, proteins were identified that can stimulate the P38 migration pathway and cytoskeleton remodelling. Additionally, the milk-EV proteome contained a great number of proteins that are known to inhibit T cells via suppression of PI3K/AKT, RAS/RAF and MAPK pathways. Depending on the specific pathway, regulation can take place early in the signalling cascade or throughout the entire signalling pathway.

**Summary/Conclusion**: By integrating various bioanalytical approaches we were able to identify relevant proteins and determine their action and position in distinct signalling pathways. As expected, milk-derived EV contain a cluster of proteins of which their combined actions are likely to regulate intercellular communication.

PT03.15

New sample preparation method for exosome proteome analysis

Zhigang Sui; Huiming Yuan; Lihua Zhang; Yukui Zhang

Dalian Institute of Chemical Physics, Chinese Academy of Sciences, Dalian, China (People’s Republic)

**Background**: Emerging evidences show that exosomes represent a rich source of biomarkers in the diagnosis and prognosis of diseases. Currently, the most widely used method for exosome isolation is differential centrifugation. But, it requires large quantities of starting material. Size-based approaches and affinity-based approaches have also been proposed for isolation and purification of exosomes. However, they are limited to low-throughput applications. Besides, several commercial exosome isolation kits have been launched for fast recovery of exosomes. However, these kits are expensive, especially if a large number of biological samples are to be processed. Here, we demonstrated a PEG-based approach, which could harvest exosomes without specialized equipment at minimal cost, coupled with cysteine-capturing aided sample preparation method, enabling a single-run shotgun quantitative proteomic workflow of exsosomes within 6 h.

**Methods**: Firstly, PEG (8 kDa, Sigma) was thoroughly mixed with 2 ml conditioned media (CM) or urine to a final PEG concentration of 10%. After incubation for 20 min, the samples were centrifuged at 4000 g for 10 min. The exosome pellet was harvested for downstream analysis. To improve the identification of plasma membrane proteins, 4% SDS was used to extract proteins from exosomes, combining a cysteine-capturing aided strategy to remove the reference of SDS on proteome analysis. After sample preparation (almost 4.5 h), the digested peptides was analyzed by 1-h proteome analysis.

**Results**: The developed PEG-based and cysteine-capturing aided approach enabled single run of SILAC-labelled exosome lysates to identify an average of 550 proteins, which is better than the efficacies of several commercial exosome isolation kits. Meanwhile, proteome profiling of urinary exosomes showed more than 1500 proteins in 2 ml urine within 6 h in a single run, providing us a potential strategy to distinguish the patients with early IgA glomerulonephritis from healthy individuals.

**Summary/conclusion**: The developed method allows short workflow time, facile preparation procedure and good compatibility towards subsequent MS analysis and requires small quantity of sample. We expect that our approach will facilitate the study of in-depth proteome profiling of exosomes and provide technical supports for clinical diagnosis.

PT04: Tumour–Stroma Interactions by EVs Chairs: Carla Mazzeo; Michiel Pegtel Location: Exhibit Hall 17:15–18:30

PT04.01

The role of extracellular vesicle-mediated miR-10a transfer in bone marrow microenvironment of patients with multiple myeloma

Tomohiro Umezu ^1^; Satoshi Imanishi^2^; Seiichiro Yoshizawa^1^; Kazuma Ohyashiki^1^; Junko H. Ohyashiki^2^

^1^Department of Hematology, Tokyo medical university, Shinjyuku, Japan; ^2^Institute of Medical Science, Tokyo Medical University, Shinjyuku, Japan

**Background**: Multiple myeloma (MM) is refractory hematologic malignancy. Bone marrow stromal cells (BMSCs) interact with MM cells in the bone marrow (BM), and also create a permissive microenvironment for MM cell growth and survival. Recent evidence indicated that extracellular vesicles (EVs)-mediated MM cell–BMSC communication plays an important role in the MM microenvironment. In this study, we investigated the biological property of the EVs and EV-miRNAs derived from BMSCs, aiming to establish the emerging strategies to target MM microenvironment to prevent tumour growth and spread.

**Methods**: BM samples were obtained from MM patients (age 56–82, *n* = 21) in accordance with the Declaration of Helsinki and using protocols approved by the research Ethics Committee of Tokyo Medical University (IRB No. 2648), and BMSCs derived form MM patients (MM-BMSCs) were isolated by the classical adhesion method. BMSCs from healthy donors (normal BMSCs) were purchased from Lonza Inc. The EVs were isolated from conditioned medium of BMSCs using Exoquick-TC Reagent (System Biosciences). To check the tumour-supportive effect of EVs derived from MM-BMSCs (MM-BMSC-EVs), we added the EVs to the cultured MM cell lines (RPMI8226). After 48 h, cell viability assays were performed using WST-8 (Dojindo). EV-miRNA profiling was done using a TaqMan low-density array (Applied Biosystems). For functional analysis of candidate miRNAs, miRNA mimics (Ambion) were transfected into RPMI8226 using HiPerFect (Qiagen).

**Results**: There were no significant differences in size and amount of EVs among normal BMSCs and MM-BMSCs. We found that the MM-BMSC-EVs enhanced the cell proliferation of RPMI8226. The EV-miRNA expression was different between MM-BMSCs and normal BMSCs, and some miRNAs, including miR-10a, were significantly up-regulated in the MM-BMSC-EVs. We then visualized with an *in vitro* model the uptake of Cy3-labelled miR-10a into RPMI8226 via EVs. To identify the function of miR-10a in MM cells, miR-10a mimic was transfected into RPMI8226 cells. Of note is that the overexpression of miR-10a enhanced MM cell growth and survival mediated through regulation of MAP3K7 and BTRC.

**Summary/conclusion**: While tumour cell growth was regulated by various factors, the EV-miR-10a derived from MM-BMSCs might therefore be one of promising target for controlling tumour proliferation in MM.

PT04.02

Optimization of an explant culture model to characterize cancer-associated exosomes in canine osteosarcoma

Anita Luu ^1^; Rachel Macdonald^1^; Michelle Oblak^2^; Brigitte Brisson^2^; Alicia Viloria-Petit^1^

^1^Department of Biomedical Sciences, Ontario Veterinary College, University of Guelph, Guelph, Canada; ^2^Department of Clinical Studies, Ontario Veterinary College, University of Guelph, Guelph, Canada

**Background**: Osteosarcoma is the most common bone tumour in canines and in humans. Previous studies have shown that both tumour cells and tumour-associated cells can promote osteosarcoma progression through extracellular vesicle secretion, such as exosomes. Various factors within the environment, such oxygen level, overall pH and matrix stiffness, can impact exosomal content. The latter is particularly important when considering osteosarcoma, due to the overall stiffness of the bone environment. The goal of this research was to develop an explant culture model to purify and characterize exosomes from canine osteosarcoma tumour tissue. This will allow for a more accurate representation of tumour exosomes *in vivo*, thus enhancing the potential for clinical translation.

**Methods**: With owner consent, tumour tissue and healthy bone samples (control) were obtained using a sterile saw and biopsy tools following limb amputation. Tissue samples were washed with PBS, mechanically dissociated and incubated in antibiotic-supplemented culture media under standard conditions overnight. The next day, the medium was changed and the explants were incubated for additional 72 h. After this, explant medium was recovered and centrifuged to remove cell debris. The supernatant was collected and stored at −80°C until further use. qEV size exclusion columns were used to isolate exosomes from the explant media, following manufacturer’s instructions. Exosomes were characterized via immunoblotting.

**Results**: Media collected from both tumour tissue and healthy tissue contained exosomes, which were predominately found in fractions 7, 8 and 9. Immunoblotting analyses showed different marker profiles in exosomes from control versus normal tissue. Further optimization steps are being implemented to improve exosome yield and purity prior to mass spectrometry.

**Summary/conclusion**: Various cell types within the tumour release exosomes that contribute to osteosarcoma progression. Microenvironmental factors impact tumour exosome features, and this is not adequately addressed by current *in vitro* models. This explant culture model provides a novel approach to characterize and study the role of exosomes in osteosarcoma.

**Funding**: Ontario Veterinary College (OVC) Pet Trust.

PT04.03

Multiple myeloma-derived exosomes carry EGFR ligand and are responsible for the uncoupled bone remodelling

Stefania Raimondo ^1^; Laura Saieva^1^; Emanuela Vicario^1^; Federica Costa^2^; Nicola Giuliani^2^; Riccardo Alessandro^1^

^1^Dipartimento di Biopatologia e Biotecnologie Mediche, University of Palermo, Palermo, Italy; ^2^Myeloma Unit, Department of Medicine and Surgery, University of Parma, Parma, Italy, Parma, Italy

**Background**: Multiple myeloma (MM) is a hematologic malignancy associated with osteolytic bone disease. We had previously shown that MM exosomes are involved in osteolytic lesions but the underlying mechanism is still understood. We hypothesize that the epidermal growth factor receptor ligand Amphiregulin (AREG) can be delivered by multiple myeloma-derived exosomes and participate in modulating the response of the bone microenvironment to the tumour. 

**Methods**: Exosomes were isolated from the conditioned medium of MM1 cell line and from BM plasma samples of patients. In order to test whether MM exosomes could affect osteoclastogenesis through the activation of the EGFR pathway, primary CD14+ monocytes and a murine cell line (RAW264.7) were used as osteoclast (OC) models. Mesenchymal stromal cells (MSC) were used to evaluate the role of MM exosomes in affecting osteoblast (OB) differentiation. Cells were treated with exosomes from both MM1 and plasma samples, pretreated or not with anti-AREG neutralizing antibodies; OC and OB specific markers were measured by real-time PCR and ELISA.

**Results**: We found that AREG was specifically enriched in exosome samples, leading to the activation of EGFR in pre-OC. In addition, we showed a significant increase of the expression of the OC markers Cathepsin K, matrix metalloproteinases 9 and tartrate-resistant acid phosphatase in RAW 264.7 and CD14+ cells after treatment with MM-derived exosomes as compared to the control. The effects of MM-derived exosomes on OC activation were significantly abrogated by exosome pretreatment with anti-AREG neutralizing Ab. Finally, we found that the treatment of MSC with exosomes reduces the expression of OB markers, leading to the inhibition of cell differentiation.

**Summary/conclusion**: Our data indicate that MM-derived exosomes affect both osteoclast and osteoblast differentiation and are responsible for the uncoupled bone remodelling. In this context, AREG packed into MM-derived exosomes is a new player in MM-induced bone resorption.

**Funding**: This work was supported by a grant from the Associazione Italiana per la Ricerca sul Cancro (AIRC) to Riccardo Alessandro (grant n°18783). Stefania Raimondo is supported by a AIRC fellowship.

PT04.04

The role of extracellular vesicles miRNAs of AML patients in the regulation of tumour bone marrow microenvironment

Inna Tzoran; Anat Aharon; Benjamin Brenner

Rambam Health Care Campus, Haifa, Israel

**Background**: Acute myeloid leukemia (AML) is characterized by rapid growth of abnormal blast cells that accumulate in the bone marrow (BM) and interfere with the production of normal blood cells. MVs contain cytokines and micro-RNA (miRNA) that are critical for cell development, proliferation and apoptosis. MVs are the major transport vehicle and serve as a unique mode of genetic exchange between cells. The current study aimed to explore the potential role of MV miRNAs in AML disease progression and to study the effects of MVs on the BM leukemic niche.

**Methods**: Blood and BM samples were collected from healthy controls and patients with AML at diagnosis and upon achievement of first remission. MV effects on the BM mesenchymal stem cells (BM-MSC) and endothelial cells were studied using confocal microscopy, migration and proliferation assays and the RT-PCR method. miRNA expression was screened by NanoString technology and validated by RT-PCR.

**Results**: Blood and bone marrow samples were collected from 43 AML patients and 24 random healthy volunteers. Screening of patient BM-MVs demonstrated that the expression of some miRNAs was high at diagnosis but decreased in remission, while other miRNAs exhibited an opposite trend. Notably, these alterations were observed in miRNA-181a, which is known to play a role in normal and malignant haematopoiesis. Specifically, miRNA-181a levels in BM-MVs of AML patients were at least 10 times higher at diagnosis than in remission or in healthy controls (*p* < 0.05). Similar results were found in miRNA-181a of circulating MVs of these patients, particularly in those who were alive at 1 year of follow-up, but not in the plasma of the patients.

**Summary/conclusion**: EV-miRNAs of AML patients are involved in the regulation of tumour BM microenvironment, affecting BM-MSC migration, proliferation and gene expression. MV-miRNAs reflect and affect AML progression and may serve as a biomarker of disease dynamics.

PT04.05

Tumour-derived exosomes contribute to a pro-tumourigenic inflammatory microenvironment in cancer

Laurence Sarte; Rie Nakata; Hiroyuki Shimada; Esteban Fernandez; Yves A. DeClerck

Children’s Hospital Los Angeles, Los Angeles, USA

**Background**: Inflammation plays an important contributory role in cancer progression through multiple mechanisms. Among those is the ability of tumour cells to induce the expression of pro-tumourigenic cytokines and chemokines by stromal cells in the tumour microenvironment. Here, we have examined the role of tumour-derived exosomes in the induction of inflammation in neuroblastoma (NBL), the second most common solid malignancy in children.

**Methods**: Exosomes from human NBL cells lines were purified by differential ultracentrifugation (DUC), optiprep density gradient centrifugation (ODGC) and size exclusion chromatography (SEC) and characterized by electron microscopy, nanoparticle tracking analysis and western blot analysis (presence of syntenin, ALIX, CD-9, 63 and 81 and absence of GM-130 and calnexin). Exosomes were labelled with green or red lipophilic dyes (PKH67 and PKH 26). Human NBL cells were also engineered to express green fluorescent protein (GFP) labelled exosomes (NBL-X-PACK-GFP). The activity of these tumour-derived exosomes was examined *in vitro* and their capture *in vitro* and *in vivo*

**Results**: NBL-derived exosomes induced the expression of several pro-tumourigenic cytokines such as IL-6, IL-8, MCP-1 and VEGF with exomes purified by SEC having the highest specific activity. The capture of exosomes by stromal cells was affected by Galectin-3-binding protein present at their surface. *In vivo*, purified NBL-derived exosomes injected intravenously or exosomes produced by NBL-X-Track tumour cells implanted orthotopically in immunodeficient mice were captured by CD-45 and F4/80-positive myeloid cells in the lungs, liver and bone marrow and by few CD-105-positive mesenchymal stromal cells in the bone marrow.

**Summary/conclusion**: Tumour-derived exosomes provide a contact-independent mechanism of communication between tumour cells and stromal cells that contribute to the induction of a pro-inflammatory reaction favourable for cancer progression.

**Funding**: National Cancer Institute/National Institutes of Health USA; St Baldrick’s Foundation.

PT04.06

Cell communication via microRNA exchange between endothelial and tumour cells during anti-cancer neoadjuvant therapy

Stella Dederen ^1^; Ingrid Struman^2^

^1^Laboratory of Molecular Angiogenesis, GIGA-R, University of Liège, Belgium, Liège, Belgium; ^2^Laboratory of Molecular Angiogenesis, GIGA-R (Cancer), University of Liège, Liege, Belgium

**Background**: The interaction between tumour cells and their microenvironment is an essential aspect of tumour development. Therefore, understanding how this microenvironment communicates with tumour cells is crucial for the development of new anti-cancer therapies.

The aim of this study is to identify microRNAs (miRNA) mediating tumour-endothelial cell (HUVEC) communication, and involved in tumour response to neoadjuvant chemotherapy. In particular, we focus on the transfer of miRNAs in endothelial exosomes.

**Methods**: Exosomes were purified by differencial ultracentrifugation. Exosomal markers were analysed by western blotting. miRNA content of exosomes was determined using qRT-PCR miRNA profiling.

**Results**: In order to determine the concentration of chemotherapeutic drugs to use, we performed survival and apoptosis assays. Results showed that when the HUVECs were treated for 2 h with paclitaxel 20 ng/ml or epirubicin 1 µg/ml, half of the cells survive after 72 h. Similar treatment does not lead to endothelial cell apoptosis. We analysed whether the treatment affects endothelial cells exosomes properties. We found that the treatments did not modify the size of the vesicles using dynamic light scattering analysis. Analyses did not reveal any modification on exosomal marker TGS101, CD63, CD81 and CD9, nor the endothelial-cell-specific marker CD31. We then isolated RNA from exosomes and from producing cells to make a profiling of their miRNA content. Analysis of the impact of treatment on the sorting of miRNA in exosome has been done. Four miRNAs (miR-373-3p, miR-887-3p, miR-122-5p and miR-129-5p) have been selected for further studies, based on their increased level in exosomes from chemotherapy-treated HUVECs. In parallel, we also found that exosomes from HUVECs treated with epirubicin or paclitaxel affected the expression of genes known to participate in drug resistance.

**Summary/conclusion**: Future work will attempt to evaluate the effects of these four exosomal miRNAs on cancer cells.

**Funding**: This work is supported by the FRIA, the FNRS, the fondation contre le cancer, the centre anti-cancéreux, the fonds Léon Frédéricq and ULiege.

PT04.07

Phenotypic heterogeneity in activated fibroblasts created by extracellular vesicles

Minami Kumazaki; Yutaka Naito; Yusuke Yamamoto; Takahiro Ochiya

Division of Molecular and Cellular Medicine, National Cancer Center Research Institute, Chuo-ku, Japan

**Background**: Tumour tissues are comprised of not only cancer cells but also stromal cells such as fibroblasts, so-called cancer-associated fibroblasts, and immune cells, and their intercellular communication via secreted factors and extracellular vesicles (EVs) plays an important role in cancer progression and metastasis.

**Methods**: We employed co-culture system of fibroblasts with high-metastatic diffuse type gastric cancer (DGC) cells or low-metastatic DGC cells. By comparing transcriptome profiles of fibroblasts co-cultured with high- and low-metastatic DGC cells, we sought to understand how high-metastatic DGC cells created an optimal microenvironment for the metastasis.

**Results**: Whole transcriptomic analysis revealed that high-metastatic DGC cells strongly induced the expressions of alpha-smooth muscle actin (α-SMA), a typical marker of myofibroblasts, and inflammatory chemokines in the fibroblasts. When fibroblasts were treated with TGFβ, a key factor of myofibroblast differentiation, α-SMA was clearly induced but suppressed inflammatory chemokine expression in the fibroblasts. In contrast, when treated with EVs from high-metastatic DGC, inflammatory chemokines such as CXCL1 and CXCL8 were significantly induced in the fibroblasts, but it is not observed in the fibroblast treated with EVs from low-metastatic DGC. Immunocytochemical analysis stained by CXCL8 and α-SMA revealed distinct populations of activated fibroblasts in the co-culture with high-metastatic DGC, suggesting that phenotypic heterogeneity was generated by EVs. Also, we found metabolic reprogramming of glycolytic pathways in fibroblasts by co-culture with high-metastatic DGC cells.

**Summary/conclusion**: Our findings suggest the cellular crosstalk between high-metastatic DGC and fibroblasts via EVs contributes to the inflammatory chemokine induction, establishing the appropriate tumour microenvironment towards the metastasis.

PT04.08

Extracellular vesicles induce fibroblasts metalloproteinases expression in thyroid tumour microenvironment

Rocío del Carmen Bravo Miana^1^; María L. Guantay^1^; Mónica B. Gilardoni^1^; Ana L. De Paul^2^; Graciela A. Borioli^3^; Claudia G. Pellizas^1^; Ana C. Donadio ^1^

^1^Dpto. Bioquímica Clínica. Centro de Investigaciones en Bioquímica Clínica e Inmunología (CIBICI-CONICET). Facultad de Ciencias Químicas. Universidad Nacional de Córdoba, Córdoba, Argentina; ^2^Centro de Microscopía Electrónica. Facultad de Ciencias Médicas. Universidad Nacional de Córdoba. Instituto de Investigaciones en Ciencias de La Salud (INICSA-CONICET/UNC), Córdoba, Argentina; ^3^Dpto. Química Biológica. Centro de Investigaciones en Química Biológica de Córdoba (CIQUIBIC-CONICET). Facultad de Ciencias Químicas. Universidad Nacional de Córdoba., Córdoba, Argentina

**Background**: The tumour microenvironment (TME), conformed by cellular and non-cellular components, promotes tumourigenesis in many solid malignancies. Extracellular vesicles (EVs) are related with intercellular communication in the TME. Fibroblasts (Fb), present in the TME, foster tumour growth stimulating cell proliferation and secreting extracellular matrix-remodelling proteases such as matrix metalloproteinases (MMPs). CD147, a glycoprotein implicated in MMPs expression, is related to thyroid tumour progression. Previous studies showed that thyroid tumour cell–Fb interactions promoted the secretion of MMPs to culture supernatants (CMs) and a migratory phenotype in tumour cells. Our goal was to identify EVs production and their role as mediators of MMPs expression in thyroid TME.

**Methods**: As an *in vitro* tumour–stroma cell interaction model, non-tumour cells (NThyOri), thyroid papillary carcinoma cells (TPC-1) and thyroid anaplastic cells (8505c) were co-cultured with normal Fb. EVs, obtained by ultracentrifugation of cell and cell-Fb CMs, were characterized by transmission electron microscopy and dynamic light scattering. MMPs were studied by zymography in EVs and CMs of Fb incubated with EVs. CD147 expression was analysed in EVs by gold-immunocytochemistry and western blot.

**Results**: EVs ranged between 50 and 600 nm. No MMP activity was detected in EVs, but Fb incubated with EVs secreted MMP9 and MMP2 to CMs. Interestingly, an increase in MMP9 and active MMP2 was observed in Fb treated with EVs collected from tumour cell–Fb co-cultures. Equally, an increased MMP2 and MMP9 expression was previously detected by immunocytochemistry in Fb cocultured with thyroid tumour cells. CD147 expression was detected in EVs and significantly increased in TPC-1-Fb and 8505c-Fb cocultures, without changes in NThyOri-Fb cocultures.

**Summary/conclusion**: The results suggest that EVs play an active role in intercellular communication events in thyroid TME, stimulating the Fb release of MMPs and the consequent tumour cell migration.

**Funding**: Secretaría de Ciencia y Tecnología de la Universidad Nacional de Córdoba (SECyT-UNC). Consejo Nacional de Investigaciones Científicas y Técnicas (CONICET). Fundación Sales (Fundación para la Investigación en Cáncer).

PT04.09

ANXA6+-EVs from CAFs support pancreatic cancer aggressiveness through a PRT99-dependent internalization process

Jeremy Nigri ^1^; Julie Leca^2^; Sebastien Martinez^2^; Sophie Lac^1^; Stephane Audebert^1^; Luc Camoin^1^; Veronique Secq^1^; Pascale Zimmermann^3^; Juan Iovanna^1^; Richard Tomasini^3^

^1^Cancer Research Center of Marseille (CRCM), Marseille, France; ^2^Cancer Research Center of Marseille, Toronto, Canada; ^3^University of Marseille, Marseille, France

**Background**: Pancreatic ductal adenocarcinoma (PDA) is an aggressive cancer. It is the fourth leading cause of cancer-related death in the Western world with a 5-year survival rate under 5%. Recently, we have shown (Leca *et al*, JCI, 2016) that Annexin A6^+^ extracellular vesicles (ANXA6^+^-EVs) from cancer-associated-fibroblast (CAF) enhance pancreatic cancer cells aggressiveness. We further demonstrated that absence of ANXA6 in those EVs limits their uptake by cancer cells.

This study aims at determining how Annexin A6 silencing in CAF limits EVs internalization by pancreatic cancer cells.

**Methods**: Immortalized or primary human CAF from resected pancreatic cancer have been used for all the *in vitro* experiments following co-cultures with murine macrophages cells (RAW264.7) in order to mimic intra-tumoural microenvironment. EVs from those co-cultures were isolated by ultracentrifugation then stained with PKH26 for uptake assays.

**Results**: Using mass spectrometry we compared the protein content of EVs from CAF shANXA6 and CAF shCtrl. One of the most significantly downregulated protein in CAF shANXA6 EVs was PRT99, known to be an exosome marker and also reported to be linked to exosome internalization. We demonstrated by qPCR, IHC and IF that PRT99 is expressed by the stromal compartment *in vivo* and further increased *in vitro* following physiopathologic culture conditions demonstrated in our previous study (Leca et al, JCI, 2016). More importantly, we demonstrated that PRT99 is physically linked to the ANXA6 complex discovered in our previous study. Then using PRT99 blocking antibody, we confirmed the implication of PRT99 in EVs uptake by reducing EVs internalization in pancreatic cancer cells and a consequent reduced migratory ability. Preliminary results suggest that following ANXA6+ EVs uptake by pancreatic cancer cells in a PRT99-dependent process enhances their migratory ability through p38 signalling pathway activation.

**Summary/conclusion**: Our results deepen the understanding of EVs internalization mode and demonstrate that PRT99 is a crucial component of ANXA6+ EVs uptake by cancer cells and their consequent gain in migratory ability. Limiting or impairing the action of PRT99 offers a new window to limit the oncogenic dialogue between stromal and cancer cells in pancreatic cancer.

**Funding**: INCa PLBIO13-134, ERC, SIRIC.

PT04.10

GABARAPL1 is required for the secretion of pro-angiogenic extracellular vesicles during hypoxia

Tom G.H. Keulers ^1^; Marijke I. Zonneveld^1^; Sten F.H.M. Libregts^2^; Marca H.M. Wauben^2^; Kasper M.A. Rouschop^1^

^1^Autophagy lab, department of Radiotherapy, GROW - school for Oncology & Developmental Biology, Maastricht University, Maastricht, The Netherlands; ^2^Department of Biochemistry and Cell Biology Faculty of Veterinary Medicine, Utrecht University, Utrecht, The Netherlands

**Background**: Hypoxia is a hallmark of solid tumours and is associated with tumour progression and therapy resistance. In response to hypoxia, tumour cells secrete pro-angiogenic factors to induce blood vessel formation and restore oxygen supply to the tumour. Extracellular vesicles (EVs) are emerging as mediators of intercellular communication in the tumour microenvironment and mediate intercellular communication by shuttling biological information such as miRNA’s, mRNA, proteins and growth factors to recipient cells. Previously, we demonstrated that the expression of GABARAPL1, a member of the LC3/GABARAP protein family, is induced during hypoxia. Now, we demonstrate that GABARAPL1 is required for secretion of pro-angiogenic EVs during hypoxia.

**Methods**: Ht29 and U87 doxycycline-inducible GABARAPL1 knockdown cell lines were exposed to hypoxia (16 h, <0.02% O2). EVs were isolated by sucrose density gradient isolation and analysed by western blot, qNANO or high-resolution flow cytometry. Angiogenic potential of cells was assessed by tube formation assays. Xenografts were implanted subcutaneously at the lateral flanks of NMRInu/nu mice and tumour size was measured by calliper.

**Results**: GABARAPL1 is expressed on the EV surface and can be targeted with antibodies. This results in blockade of pro-angiogenic responses *in vitro*. Silencing GABARAPL1 with inducible knockdown models perturbs EV secretion and results in decreased tumour growth due to decreased vascularisation and enhanced necrosis. Additionally, targeting GABARAPL1 directly after tumour irradiation resulted in enhanced tumour regrowth delay. Furthermore, we demonstrate that GABARAPL1+ EVs are detectable and increased in blood of cancer patients.

**Summary/conclusion**: Here, we reveal that hypoxic tumour cells secrete a unique EV subset, marked by GABARAPL1 expression. These EVs control tumour progression, are targetable and are therefore interesting to pursue as biomarker and therapeutic target.

**Funding**: This work was financially supported by the Dutch Cancer Society (KWF Grants UM 2012-5506 and 2015-7735 to K.R.), worldwide cancer research fund 16-0265 (to K.R.).

PT04.11

MiRNA-146a-5p in extracellular vesicles facilitate the angiogenesis in high-grade bladder cancer

Marta Prieto Vila ^1^; Wataru Usuba^1^; Nobuyoshi Kosaka^2^; Fumitaka Takeshita^3^; Hideo Sasaki^4^; Tatsuya Chikaraishi^4^; Takahiro Ochiya^2^

^1^Division of Molecular and Cellular Medicine, National Cancer Center Research Institute, Tokyo, Japan; ^2^Division of Molecular and Cellular Medicine, National Cancer Center Research Institute, Chuo-ku, Japan; ^3^Fundamental Innovative Oncology Core Center, National Cancer Center Research Institute, Chuo-ku, Japan; ^4^Department of Urology, St. Marianna University School of Medicine, Kawasaki, Japan

**Background**: High recurrence is an everlasting problem in bladder cancer (BCa).The classical method for detecting recurrence in BCa is cystoscopy, which is a highly invasive technique. Thus, novel methods with high reliability and low-invasiveness are needed. To overcome this problem, biomarkers in the urine such as microRNA (miRNA) in extracellular vesicles (EVs) are proposed. Although the usefulness of miRNA in body fluid from cancer patients has been reported, their contribution to the cancer progression has not been shown clearly. Since we previously detected high urinary levels of miR-146a-5p in patients with BCa, in this study, we further analysed the function of miRNA-146a-5p in EVs from BCa.

**Methods**: High-grade bladder cancer cell line, UMUC-3, with miR-146a overexpression was established and orthotropically transplanted in SCID mice.Tumour size was measured by *in vivo* imaging and immunohistochemical analysis was performed to evaluate angiogenesis in the tumour. Cellular proliferation, migration and invasion were assessed in human umbilical vein cell (HUVEC) after the addition of EVs from BCa. Uptake of EVs from BCa was also evaluated by PKH67-lavelled EVs.

**Results**: Urinary miR-146a-5p level was higher in patients with high-grade BCa and in correlation with the depths of invasiveness. Tumours generated by UMUC-3-luc miR-146a o/e cells transplanted in mice presented fast tumour growth with angiogenesis. We found that the cell proliferation of HUVEC was significantly increased both under transfection with miR-146a mimic and treatment with miR-146a-5p- enriched EVs. This proliferation and the uptake of EVs efficiency by HUVEC were in harmony with the depth of invasiveness of BCa.

**Summary/conclusion**: Our findings indicate that EVs containing miR-146a-5p from BCa promoted the proliferation of endothelial cells in tumour. These results demonstrate that miRNAs in body fluid, even within EVs, which has been widely reported for diagnosis, could contribute for the cancer progression. Therefore, miRNAs in EVs will be not only a target molecule for diagnosis but also for therapy.

**Funding**: Grand-in-Aid from the Project for Cancer Research and Therapeutic Evolution (P-CREATE) from the Japan Agency for Medical Research and Development (AMED).

PT04.12

Exosomes secreted by human osteosarcoma cells promote angiogenesis

Francesca Perut ^1^; Laura Roncuzzi^1^; Annamaria Massa^1^; Elena Torreggiani^1^; Nicoletta Zini^2^; Nicola Baldini^1^

^1^Orthopaedic Pathophysiology and Regenerative Medicine Unit, Rizzoli Orthopaedic Institute, Bologna, Italy; ^2^CNR - National Research Council, Institute of Molecular Genetics, Bologna, Italy

**Background**: Angiogenesis is a pivotal process in osteosarcoma (OS) development and progression. Angiogenesis involves a number of different players among which exosomes have been recently proposed as efficient cargo of pro-angiogenic mediators. Acidity is a hallmark of malignancy in a variety of cancers, including sarcomas, as a result of an increased energetic metabolism. In cancer other than sarcoma, tumour-induced extracellular acidity has been associated with an increased exosome release and uptake. In this study, we investigated the role of OS-derived exosomes on tumour angiogenesis, and the influence of acidity of tumour microenvironment in this process.

**Methods**: Exosomes were isolated by differential centrifugation of culture media from 143B OS cells grown at different pH (6.5 or 7.4). Exosome morphology was assessed by TEM.

To test the effect of exosomes on angiogenesis, HUVEC cells were stimulated with exosomes, and their uptake, cell proliferation, migration and tubule-like structure formation were analysed. The expression profiles of angiogenesis-related proteins were evaluated by an angiogenesis array on OS-derived exosomes. The ability of exosomes to induce new blood vessel growth *in vivo* was assessed on chicken chorioallantoic membrane (CAM).

**Results**: Exosomes isolated by OS cells displayed the expected size range (30–100 nm). The release of exosomes by OS cells was significantly increased at acidic compared to neutral pH (*p* = 0.009). HUVEC proliferation and migration was not significantly affected by the treatment with OS-derived exosomes. OS-derived exosomes significantly promoted the tubulogenesis by HUVEC (*p* = 0.034). Exosomes induced new blood vessel growth on CAM vascular bed *in vivo*. The lengh of vessels and the number of branch points was significantly higher for exosomes derived from OS cells cultured at acidic pH (*p* = 0.018 and *p* = 0.0026). Angiogenesis-related proteins (i.e. SerpinE1, TIMP1, Thrombospondin -1, uPA, VEGF, PTX3, CD105) were detected in OS-derived exosomes.

**Summary/conclusion**: Our findings suggest that human OS cells secrete exosomes both in acidic and in neutral conditions. Acidity increases the release of exosomes. OS-derived exosomes induce angiogenesis, both *in vitro* and *in vivo*, and this activity is prompted by the acidity of tumour microenvironment.

**Funding**: Supported by The Italian Association for Cancer Research (IG 15608).

PT04.13

Unravelling Notch implication in exosome-mediated angiogenesis of MDA 231

Hernan González-King^1^; Nahuel Aquiles. García^2^; María Ciria^3^; Rafael Sánchez^1^; Sandra Tejedor^3^; Pilar Sepulveda ^1^

^1^Instituto de Investigación Sanitaria La Fe., Valencia, Spain; ^2^Cedars-Sinai, La Jolla, USA; ^3^Fundación para La Investigación La Fe/ Centro de Investigación Príncipe Felipe de Valencia, Valencia, Spain

**Background**: Tumour-derived exosomes are emerging mediators of tumourigenesis and tissue-specific metastasis. Dysregulated Notch receptor activity has been implicated in breast cancer but the mechanisms by which Notch contributes to the tumourigenic process are not yet clear and even less its role mediated by exosomes. 

**Methods**: We have used several *in vitro* techniques to study the presence of Notch pathway components in exosomes of the tumourigenic breast cancer cell line MDA 231, the functionality of these components and its implication on the angiogenic process triggered by these cells.

**Results**: We found that MDA 231 exosomes are loaded with ligands of the Notch signalling pathway, the Notch receptor 1 and also the activated domain of this receptor (N1ICD). The addition of exosomes from MDA 231 cultures to endothelial cells triggered transcriptional changes in Notch target genes and induced angiogenesis in an *in vitro* model of capillary-like tube formation. Both effects were maintained in the presence of an inhibitor of the NOTCH pathway that blocks the release of N1ICD by activation and cleavage of the Notch receptor 1.

**Summary/conclusion**: All together, these results indicate that exosomes derived from MDA 231 have an angiogenic capacity in part by the packaging of NICD, which could be a potential target for antitumoural drugs.

**Funding**: ISCIII: PI16/00107, RD16/0011/0004.

PT04.14

Exosomes contain Wnt signals that regulate vascularization in lung cancer

Judith Miskei ^1^; Kitti Garai^2^; Krisztina Banfai^2^; Emoke Papp^1^; Zsofia Torok^1^; Krisztian Kvell^2^; Veronika Sarosi^1^; Judit E. Pongracz^2^

^1^University of Pecs, Pecs, Hungary; ^2^Institute of Pharmaceutical Biotechnology, Faculty of Pharmacy, University of Pecs, Pecs, Hungary, Pécs, Hungary

**Background**: Angiogenesis is important both in normal tissue function and in disease and represents a key target in lung cancer (LC) therapy. Unfortunately, the two main subtypes of non-small-cell lung cancers (NSCLC), namely, adenocarcinoma (AC) and squamous cell carcinoma (SCC), respond differently to anti-angiogenic, e.g. anti-vascular endothelial growth factor (VEGF)-A treatment with life-threatening side effects, often pulmonary haemorrhage in SCC. The mechanisms behind such adverse reactions are still largely unknown although peroxisome proliferator activator receptor (PPAR) gamma and Wnt-s have been named as molecular regulators of the process.

**Methods**: Oncosomes and exosomes were isolated from supernatants of lung cancer (adeno and squamous cell carcinoma) cell lines. PPARgamma, Wnt5a, Wnt4, miR27b levels were determined using various techniques, including ELISA, TaqMan PCR and microarray. Exosomes were stained and organ homing was identified in mice.

**Results**: Wnt5a was identified as one of the major protein content of the isolated exosomes of SCC cell lines.

**Summary/conclusion**: During carcinogenesis, the Wnt microenvironment alters, which can downregulate PPARgamma leading to increased VEGF-A expression. Wnt5a is the characteristically highly expressed Wnt in cancers with squamous histology and increased Wnt5a levels are readily detectable in exosomes of SCC cancer cell lines. Differences in the Wnt microenvironment in AC and SCC cell lines can provide a potential diagnostic tool to differentiate AC and SCC type vascularization from patients’ sera in lung cancers that can determine future therapy.

PT04.15

The association of total and vesicular blood HLA-G levels with disease stage and circulating tumour cells in ovarian cancer patients

Esther Schwich ^1^; Rafael T Michita^2^; Paul Buderath^3^; Peter A. Horn^1^; Rainer Kimmig^3^; Sabine Kasimir-Bauer^3^; Vera Rebmann^1^

^1^Institute for Transfusion Medicine, University Hospital Essen, Essen, Germany; ^2^Genetics Department, Universidade Federal do Rio Grande do Sul (UFRGS), Porto Alegre, Brazilil; ^3^Department for Gynecology and Obstetrics, University Hospital Essen, Essen, Germany

**Background**: The non-classical human leucocyte antigen-G (HLA-G) expression promotes cancer invasiveness and metastatic progression. HLA-G can exist as cell surface molecule or in soluble forms (sHLA-G) including secreted or shed molecules or released molecules via extracellular vesicles (EVs). In this study, we addressed the question how sHLA-G subcomponents impact the clinical parameters and disease outcome in epithelial ovarian cancer (EOC). 

**Methods**: For this, we (i) quantified the total amount of sHLA-G (sHLA-Gtot) and vesicular sHLA-G (HLA-GEV) in histologically confirmed EOC patients through ELISA and (ii) analysed the impact of sHLA-G on the clinical parameters of EOC.

**Results**: Levels of both, sHLA-Gtot and sHLA-GEV were significantly increased in serous EOC patients compared to healthy donors (HD, *p* < 0.0001). Further, elevated levels were associated with advanced disease stage (*p* < 0.0001) mirroring the tumour burden. Strikingly, release of vesicular sHLA-G was promoted in EOC (*p* = 0.0003) and the share of sHLA-Gtot on sHLA-GEV was already observed in early stages of disease (*p* < 0.01). Of note, sHLA-GEV was strongly associated with the presence of circulating tumour cells (*p* < 0.01).

**Summary/conclusion**: Our data suggest that EOC promotes the release of vesicular sHLA-G which links to a deteriorated course of disease indicating that discrimination of sHLA-G subcomponents is a potential tool for the diagnosis and prognosis of EOC.

PT04.16

Arginase-1-containing exosomes induce suppression of antitumour *in vitro* and *in vivo* immune response

Malgorzata Czystowska-Kuzmicz ^1^; Anna Sosnowska^1^; Justyna Chlebowska-Tuz^1^; Kavita Ramji^1^; Marta Szajnik^1^; Slawomir Gruca^1^; Artur Stefanowicz^2^; Dominika Nowis^3^; Jakub Golab^1^

^1^Dept. of Immunology, Center of Biostructure Research, Medical University of Warsaw, Warsaw, Poland; ^2^Dept. of Gynecology and Obstetrics, “Praski” Hospital, Warsaw, Warsaw, Poland; ^3^Genomic Medicine, Medical University of Warsaw, Warsaw, Poland

**Background**: We have shown previously that exosomes derived from ascites of patients with ovarian cancer (OvCa) and from OvCa cell lines (TEX) contain enzymatically active Arg-1 which activity correlates with worse prognosis. In this study, we used TEX isolated from OvCa cells transfected with V5-tagged Arg-1 to discriminate tumour-derived Arg-1 from endogenous Arg-1. We investigated the influence of these exosomes on the antitumour effector mechanisms of immune response in *in vitro* and *in vivo* experiments.

**Methods**: TEX were isolated by ultracentrifugation and verified by western blotting, NanoSight and TEM. Effects of exosomal Arg-1 on specific immune response were analysed in *in vitro* proliferation assays and *in vivo* by adoptive transfer of OVA-antigen specific OT-I T cells. Effects of Arg-1 on tumour growth were investigated in a syngeneic OvCa model in immunocompetent mice.

**Results**: Arg-1-expressing tumours developed faster, led to faster ascites accumulation and shorter survival in an OvCa mouse model. We detected a lower percentage of activated CD8+ and CD4+ T cells isolated from ascites positive for OvCa-derived Arg1-TEX in comparison to T cells isolated from ascites containing mock-TEX. T cells from Arg1-TEX-positive ascites expressed lower levels of CD3-zeta and CD69 upon *in vitro* re-stimulation. Administration of an Arg-1 inhibitor led to slower tumour development and increased percentage of activated T cells and dendritic cells (DCs) in the peritoneal cavity. Co-culture of bone-marrow-derived DCs with Arg1-TEX resulted in the transfer of functionally active Arg-1 and inhibition of DCs-primed proliferation. Similarly, OVA-antigen-specific proliferation of OT-I T cells *in vivo* was inhibited by Arg1-TEX. All these *in vitro* and *in vivo* effects were reversed by the Arg-1 inhibitor.

**Summary/conclusion**: Our findings provide the first evidence for the role of Arg-1 in the formation of an immunosuppressive microenvironment in OvCa. We identify a novel mechanism of exosomal Arg-1 distribution from the tumour cells to antigen-presenting cells. Inhibition of Arg-1 activity may be an attractive novel anti-cancer strategy in ovarian carcinoma.

**Funding**: National Science Center - OPUS 6 Program 2013/11/B/NZ6/02790 (MC) and OPUS 12 2016/23/B/NZ6/03463 (DN), National Center for Research and Development - STRATEGMED2/265503/3/NCBIR/15 (JG).

PT04.17

The effect of IFN-γ treatment on extracellular vesicles metabolite composition in breast cancer cells

Hiroko Tadokoro ^1^; Ryuhei Kudo^2^; Akiyoshi Hirayama^2^; Yusuke Yoshioka^3^; Masahiro Sugimoto^2^; Takahiro Ochiya^3^

^1^Division of Molecular and Cellular Medicine, National Cancer Center Research Institute, Tokyo, Japan; ^2^Institute for Advanced Biosciences Keio University, Tsuruoka, Japan; ^3^Division of Molecular and Cellular Medicine, National Cancer Center Research Institute, Chuo-ku, Japan

**Background**: The functions of extracellular vesicles (EVs) in cancer relate to tumour survival, such as immunosuppression. EVs contain various molecular constituents, including metabolites. The functions of metabolites in EVs remain largely unknown. Indoleamine-2,3-dioxygenase1 (IDO) is a tryptophan(Trp) catabolic enzyme which is induced by cytokines such as IFN-γ. Because of IDO-induced Trp depletion and production of metabolites that exert immunoregulatory functions, IDO in tumours create an immunosuppressive microenvironment. The mechanisms of IDO-induced immunosuppression in tumours are still incompletely understood. Therefore, we aim to identify IDO-induced metabolites that are associated with immunosuppressive functions in breast cancer cell-derived EVs.

**Methods**: The breast cancer cell line MDA-MB-231-D3H2LN (D3H2LN) was cultured in the presence and absence of IFN-γ. EVs were purified from cell supernatant by ultracentrifugation. Metabolome analyses of cell and EVs were performed on D3H2LN treated with or without IFN-γ, using CE-TOFMS and IC/LC-QE. To investigate the cytotoxic effects of EVs derived from D3H2LN treated with IFN-γ (IFN-γ_EVs) on immune cells, the cell viability assay was performed using human leukaemia monocyte cell line (THP-1) treated with IFN-γ.

**Results**: Treatment with IFN-γ enhanced IDO expression in D3H2LN. Higher amounts of uracil, uridine, adenosine and guanosine were detected in IFN-γ_EVs. Cell viability of THP-1 treated with IFN-γ stimulated by IFN-γ_EVs was significantly reduced after 72 h, as compared with cells stimulated by EVs derived from D3H2LN treated without IFN-γ.

**Summary/conclusion**: Trp catabolism via the kynurenine pathway produces adenosine diphosphate ribose. Therefore, it can be speculated that adenosine was produced by treatment of IFN-γ in cell and sorted into EVs. Our results indicate that IFN-γ_EVs have cytotoxic effects on THP-1.

PT04.18

Extracellular vesicles derived from natural killer cells use multiple cytotoxic proteins and killing mechanisms to target cancer cells

Chun-Hua Wu; Robert Seeger; Muller Fabbri; Larry Wang; Alan Wayne; Ambrose Y. Jong

Children’s Hospital of Los Angeles, Los Angeles, USA

**Background**: Extracellular vesicles (EVs) are secreted membrane vesicles that play complex physiological and pathological functions in intercellular communication. We have recently isolated natural killer cell-derived EVs (NK-EVs) from *ex vivo* expansion of NK cell cultures. The isolated NK-EVs contained cytotoxic proteins and activated caspases, and they induced apoptosis in target cells. However, the detailed mechanisms of NK-EV associated cell killing are not completely understood.

**Methods**: We used ELISA to detect the level of cytotoxic proteins from isolated NK-EVs, and immunofluorescence microscope and western blots to monitor the impacts on the targeted cancer cells.

**Results**: Our results showed that the mean values of perforin (PFN, 550 ng/ml), granzyme A (Gzm-A, 185 ng/ml), granzyme B (Gzm-B, 23.4 ng/ml), granulysin (GNLY, 56 ng/ml) and Fas ligand (FasL 2.5 ng/ml) were obtained from >60 NK-EV isolates. The correlation between cytotoxicity and cytotoxic protein levels was examined by linear regression. PFN, Gzm-A, Gzm-B, GNLY all had a positive, moderate correlation with cytotoxicity (*R* ^2^ = 0.2~0.4), suggesting that there is not a single cytotoxic protein dominantly involved in killing, but that all may contribute to cytotoxicity. To further explore the possible killing mechanisms, targeted cell lysates treated with NK-EVs were assessed by western blotting. The levels of Gzm-A substrates, SET and HMG2, were diminished in target cells, indicating that Gzm-A induces a caspase-independent death pathway. In addition, immunofluorescence microscopic images showed that cytochrome C was released from mitochondria, a central hallmark of caspase-dependent pathways. Several ER-associated proteins were altered, e.g. increase of Ero1-Lalpha, PERK and phosphorylated-elF2alpha, suggesting that NK-EVs-induced ER stress may result in apoptosis.

**Summary/conclusion**: Our results support that multiple killing mechanisms are activated by NK-derived EVs, including caspase-independent and caspase-dependent cell death pathways, resulting in the killing of targeted cancer cells.

PT04.19

TEx-induced tDC

Sarah Renaud^1^; Chantal Havet^1^; Rami Mustapha^1^; Joshua Mason^2^; Zachary Fitzpatrick^3^; Benjamin Hennart^4^; Delphine Allorge^4^; Nadira Delhem^1^; Olivier Morales ^1^

^1^CNRS UMR 8161 IRCV team, Lille, France; ^2^Palm Beach Atlantic University, West Palm Beach, USA; ^3^Department of Neurology, The Massachusetts General Hospital and NeuroDiscovery Center, Harvard Medical School, Boston, USA; ^4^Laboratoire de Toxicologie, CBP, CHRU Lille, Lille, France

**Background**: A characteristic of the nasopharyngeal carcinoma (NPC) micro-environment is the presence of immunosuppressive exosomes released by tumour cells. Our team has recently shown that NPC-derived exosomes, which carry Galectine-9, favour the recruitment and suppressive activity of human regulatory T cells (Treg), thus contributing to NPC immune escape (Mrizak et al, JNCI, 2015).

In this study, our objective is now to evaluate whether these NPC-derived exosomes could promote the emergence of tolerogenic semimature dendritic cells (tolDC) able to induce regulatory T cells from naive CD4+ T cells ultimately contributing to the tolerance of tumour cells.

**Methods**: We performed a complete phenotypical and functional study comparing the effect of NPC and healthy donor-derived exosomes on DC maturation. This study includes (i) cell morphological analysis by photonic microscopy, (ii) transcriptomic study by RTqPCR, (iii) flow cytometric analysis of the expression of specific makers (phenotypic DC and Treg markers), (iv) a preliminary DC functional study by western blotting (IDO) and HPLC dosage of tryoptophan metabolites, (v) a secretome analysis by ELISA (IL-10; TGF-β, TNF-α, IL-6 and IL-12) (vi) and finally a functional assay where the CNP exosome-exposed tolDCs are co-cultivated with naive T cells in order to determine the type of T cells generated.

**Results**: Taken together, our results strongly suggest that the presence of NPC-derived exosomes favours the emergence of semi-mature tolerogenic DCs.

**Summary/conclusion**: Despite the importance of immature DCs as mediators of cancer immune escape, no other studies have shown the impact of tumour exosomes on the maturation of human DCs. Thus, these promising results should open new prospects for antitumour immunotherapies based on the inhibition of factors involved in the emergence and activation of Tregs.

**Funding**: Cancéropole Nord-Ouest, Lille, France; Région-Hauts de France.

PT04.20

Cell-line specific influence of breast cancer-derived extracellular vesicles on the composition and immune response of peripheral cell subsets

Esther Schwich ^1^; Maike Giesing^1^; Lisa König^2^; Ann-Kathrin Bittner^2^; Oliver Hoffmann^2^; Rainer Kimmig^2^; Peter A. Horn^1^; Sabine Kasimir-Bauer^2^; Vera Rebmann^1^

^1^Institute for Transfusion Medicine, University Hospital Essen, Essen, Germany; ^2^Department for Gynecology and Obstetrics, University Hospital Essen, Essen, Germany

**Background**: Tumour-derived extracellular vesicles (EV) represent important elements for intercellular cross-talk operative in the tumour microenvironment or in the periphery. In breast cancer (BC), the expression status of the hormone receptors progesterone (PR), estrogen (ER) and human epidermal growth factor receptor 2 (HER2) define therapeutic decisions. Therapeutic options for patients lacking these receptors (triple negative, TN) are limited contributing to a worse outcome. However, little is known about the influence of steroid hormones on the modulatory capacity of BC-derived EV. Here, we focus on the immune-editing effects of EV derived from different BC cell lines cultured with/without steroid hormones.

**Methods**: EV derived from the TN cell line MDA-MB-231 (EVMDA), and the PR+/ER+/HER2- cell lines MCF-7 (EVMCF) and T47D (EVT47D), cultivated with/without progesterone and/or ß-estradiol for 24 h, were isolated by differential centrifugation. Sizes and particle numbers as well as EV-marker expression (Tsg101, CD81, Syntenin) were defined by nano-tracking analysis or SDS-PAGE/western blotting. Composition and activation status of peripheral blood cells (PBL) subsets after CD3/CD28 or PMA/Ionomycin stimulation in the absence/presence of the different EV preparations were analysed by flow cytometry.

**Results**: All EV preparations displayed typical EV markers. Particle size, particle numbers released per cell, altered composition of PBL subsets and their modified proliferation and cytokine responses in the presence of EV were found to be cell-lineage specific but independent of the hormonal treatment. Presence of EVMCF during CD3/CD28 or PMA/Ionomycin stimulation resulted in increased NK and CD4+ T-cell frequencies, and decreased frequencies of TNFa- and IFNg-producing cell subsets, whereas EVMDA provoked an increased IL-6 and proliferation response by T-cells.

**Summary/conclusion**: Thus, EV of different BC lines revealed a cell-line-specific capacity to modulate the immune response of PBLs, which were not influenced by hormonal treatment.

PT04.21

Mutant p53 controls tumour microenvironment via extracellular vesicles

Tomer Cooks ^1^; Ioannis S. Pateras^2^; Ana I. Robles^3^; Vassilis G. Gorgoulis^2^; Curtis C. Harris^3^

^1^national cancer institute, NIH, Bethesda, USA; ^2^Athens, Athens, Greece; ^3^NCI, NIH, Bethesda, USA

**Background**: TP53 mutants (mutp53) are involved in the pathogenesis of most human cancers. Specific mutp53 proteins gain oncogenic functions (GOF) distinct from the tumour suppressor activity of the wild-type protein. Tumour-associated-macrophages (TAM), a hallmark of solid tumours, are typically correlated with poor prognosis. We investigated cell-to-cell communication between cancer cells harboring mutp53 GOF and neighboring macrophages.

**Methods**: Primary human macrophages were co-cultured with colon carcinoma cell lines differing by their p53 status in a transwell system. We identified inflammatory and pro-tumoural phenotypes of co-cultured macrophages using qPCR, ELISA and various functional biological assays. Co-injection of macrophages and tumour cells in NOD-SCID mice was used to determine tumour-supportive characteristics using both xenografts and orthotopic models. Resected colon tumours from colorectal cancer patients were genotyped, divided into groups of wt vs. mutant p53 and analysed for the correlation with tumour-associated macrophages and survival.

**Results**: We report a non-cell-autonomous mechanism whereby human mutp53 cancer cells reprogram macrophages to a tumour supportive and anti-inflammatory state. The colon carcinoma cells harbouring GOF mutp53 selectively shed miR-1246-enriched exosomes. Uptake of these exosomes by neighbouring macrophages triggers their miR-1246-dependent reprogramming into a cancer-promoting state. Mutp53-reprogammed TAM favours anti-inflammatory immunosuppression with increased activity of TGF-β. These findings, associated with poor survival in colon cancer patients, strongly support a microenvironmental GOF role for mutp53 in actively engaging the immune system to promote cancer progression and metastasis.

**Summary/conclusion**: Genetic alterations in the tumour might exacerbate tumourigenesis mediated by extracellular vesicles transferred between tumour cells and associated macrophages. The transfer of miR-1246 shapes a tumour supporting microenvironment that could be targeted in the future, using anti-miR therapies.

**Funding**: National Cancer Institite.

PT05: EVs as Cancer Biomarkers-proteomics Chairs: Yves deClerck; Alicia Llorente Location: Exhibit Hall 17:15–18:30

PT05.01

Proteomics discovery of novel plasma exosome biomarkers for early detection of patients at risk for non-small cell lung cancer (NSCLC)

Esther Sok Hwee. Cheow ^1^; Win Lwin Thuya^1^; Amelia Lau^1^; Lingzhi Wang^1^; Ross Soo^1^; John Kit Chung Tam^2^; Boon Cher Goh^1^

^1^Cancer Science Institute of Singapore, National University of Singapore, Singapore, Singapore; ^2^Department of Surgery, Yong Loo Lin School of Medicine, Singapore, Singapore

**Background**: Non-small cell lung cancer (NSCLC) is the main cause of cancer mortality, with surgical intervention and radiotherapy having minimal impact on 5-year survival rates. The lack of precise biomarkers required for NSCLC screening contributed to the delay in early detection. Exosomes are constitutively secreted by almost all cell types into the plasma, and tumour cells are known to release more exosomes than normal proliferating cells. The ability of exosomes to deliver proteins to elicit a functional response made them desirable as biomarkers.

**Methods**: Written informed consent was obtained from all participants, approved by the National University Hospital Institutional Review Board. Plasma exosomes were isolated using ultracentrifugation and total exosome isolation reagent in the discovery and verification/validation phase, respectively. Tandem mass tag quantitative discovery proteomics was used to establish the differential proteome of pooled plasma exosomes from early-stage NSCLC, late-stage NSCLC and healthy subjects. In the verification/validation phase, antibody-based assays were used.

**Results: Fifty-six** differentially expressed (*p* <0.05) proteins were scrutinized through extensive literature mining, and based on their novelty and association with cancer progression, 10 markers were shortlisted for verification. Verification analyses on individual patients returned with a panel of six promising plasma exosome markers of NSCLC, with expressions significantly (*p* < 0.05) associated with both early- and late-stage NSCLC. Validation on the diagnostic efficiency of the six candidates will be conducted alongside with known NSCLC biomarkers, in larger cohort, to assess their reliability.

**Summary/conclusion**: To date, proteomics studies on circulatory exosomes in lung cancer research are under-explored. The interrogation of exosome proteome is a promising approach to uncover the wealth of biomarker information. The panel with the best combination derived at the end of this study will deliver a protein signature with added predictive value to complement with existing screening tools, to improve the diagnosis, stratification and long-term prognosis of NSCLC.

**Funding**: This research is supported by the National Research Foundation Singapore and the Singapore Ministry of Education under its Research Centres of Excellence initiative.

PT05.02

Identification of androgen-dependent glycosylations on the surface of extracellular vesicles derived from prostate cancer cell lines

Md Khirul Islam ^1^; Parvez Syed^1^; Jason P. Webber^2^; Guido W. Jenster^3^; Kim Pettersson^1^; Urpo Lamminmäki^1^; Janne Leivo^1^

^1^University of Turku, Turku, Finland; ^2^Tissue Microenvironment Group, Division of Cancer and Genetics, School of Medicine, Cardiff University, Cardiff, United Kingdom; ^3^Erasmus Medical Center, Rotterdam, The Netherlands

**Background**: Changes in glycans are common in cancer and play important role in identification of surface tumour markers. Majority of the surface tumour markers reported to date are either glycoproteins or glycolipids. In this study, we attempted to identify androgen-dependent glycosylations on the surface of extracellular vesicles (EVs) derived from prostate cancer (PCa) cell lines using a panel of lectins.

**Methods**: Biotinylated anti-CD63 antibody was immobilized on streptavidin-coated microtitre plate to capture EVs derived from androgen hormone-sensitive (VCaP & LNCaP) and hormone-insensitive (PC3 & DU145) PCa cell lines. The glycans present on the surface of the captured EVs were targeted by glycan-binding lectins, conjugated with Eu+3-doped nanoparticles (NPs). To ensure equal loading of EVs in these assays, 400 ng of total protein content was loaded.

**Results**: Among 35 lectins screened so far, lectins WFA (*Wisteria floribunda*), TJA-II (*Trichosanthes japonica*) and UEA (*Ulex europaeus*) showed significant signal intensities to the EVs derived from androgen hormone-sensitive cell lines compared to androgen hormone-insensitive cell lines. The signals obtained from the assay were normalized with the signals obtained from assay where antibodies against tetraspanins were conjugated with NPs. Our results give clue of a reciprocal link between androgen regulation and EV glycosylations, which can be detected with a simple bioaffinity assay.

**Summary/conclusion**: The relationship between glycosylations and androgen dependency in PCa is a well-known phenomenon. However, identification of such glycosylations is often laborious and tedious. By using our simple lectin-Eu3+-NPs technology, it is possible to identify disease-specific glycosylations on the surface of EVs. This approach might be useful for EVs-based diagnosis and prognosis of prostate cancer.

**Funding**: The research work was supported by Department of Biotechnology (DBT), Government of India; U. Lamminmäki, PROVATECT FINLAND funded by TEKES (decision number 40089/14); O. Carpen, Tekes funding.

PT05.03

Proteomic identification of exosome-derived FAM3C as a potential biomarker for non-small cell lung cancer

Win Lwin Thuya ^1^; Ross Soo^1^; Nicholas Syn^1^; Tiannan Guo^2^; Esther Sok Hwee. Cheow^1^; Ting Ting Wang^1^; Li Ren Kong^1^; Amelia Lau^1^; Richard Weijie Ong^3^; The Hung Huynh^3^; Andrea Li Ann Wong^1^; Henry Yang^1^; Paul Chi Lui Ho^4^; Newman Siu Kwan Sze^2^; Lingzhi Wang^1^; Boon Cher Goh^1^

^1^Cancer Science Institute of Singapore, National University of Singapore, Singapore, Singapore; ^2^School of Biological Sciences, Nanyang Technological University, Singapore, Singapore; ^3^National Cancer Centre, Singapore, Singapore, Singapore; ^4^Department of Pharmacy, National University of Singapore, Singapore, Singapore

**Background**: The discovery of biofluid-based biomarkers is urgently needed to improve early detection of lung cancer. Exosome-derived proteins are useful resources in biomarker identification.

**Methods**: Proteomic analysis of one normal fibroblast and three NSCLC cell-derived exosomes was conducted. Exosomes were isolated by ultracentrifugation and characterized by western blot, transmission electron microscopy and Zetasizer. Human plasma and tissues samples were used for validation of FAM3C as a novel lung cancer *in vivo* biomarker. Written informed consent was obtained from all participants.

**Results**: FAM3C was among the top 15 potential proteins highly expressed in cancer cell exosomes and chosen for further validation. In functional study, overexpression of FAM3C dramatically stimulated the epithelial-mesenchymal transition (EMT), migration, invasion, proliferation and colony formation of lung cancer cells while knockdown of FAM3C showed opposite effects. Further analysis showed that exosomes could serve as messengers in intercellular communication to promote metastasis in lung cancer cells. Injection of overexpressed FAM3C cells via the tail vein promoted lung metastasis in mouse models. The IHC staining indicated that FAM3C expression in lung cancer specimens was greatly increased compared to those in tumour adjacent and normal lung tissues. Moreover, granular FAM3C staining was significantly associated with improved lung cancer specific survival in squamous cell carcinoma patients. ELISA assay revealed that plasma exosome FAM3C was significantly elevated in NSCLC patients (*n* = 78) compared to healthy controls (*n* = 78) (*p* < 0.0001) with an AUC of 0.831, a sensitivity of 0.756 and a specificity of 0.744.

**Summary/conclusion**: These findings demonstrate that exosome-derived FAM3C is a potential biomarker which predicts lung cancer metastasis, and further large-scale clinical studies are warranted.

**Funding**: This research is supported by the National Research Foundation Singapore and the Singapore Ministry of Education under its Research Centers of Excellence initiative.

PT05.04

Regulation of exosome release in lung cancer cell lines by a lung cancer exosome-specific protein 1 (LESP1)

Hyesun Jeong ^1^; Byeong Hyeon Choi^2^; Jik Han Jung^3^; Jaena Park^4^; Yong Park^5^; Ji-Ho Park^3^; Yeonho Choi^6^; Hyun Koo Kim^7^; Sunghoi Hong^8^

^1^School of Biosystem and Biomedical Science; Korea Univ. Department of public health Sciences, Graduate School of Korea University,, Seoul, Republic of Korea; ^2^Department of Biomedical Sciences, College of Medicine, Korea University, Seoul, Republic of Korea; ^3^Department of Bio and Brain Engineering, Korea Advanced Institute of Science and Technology, Taejon, Republic of Korea; ^4^Department of Bio-convergence Engineering, Korea University, Seoul, Republic of Korea; ^5^Korea University School of Medicine, Seoul, Republic of Korea; ^6^School of Biomedical Engineering, Korea University, Seoul, Republic of Korea; ^7^Department of Thoracic and Cardiovascular Surgery, Korea University Guro Hospital, College of Medicine, Korea University, Seoul, Republic of Korea; ^8^School of Biosystem and Biomedical Science, College of Health Science, Korea University, Seoul, Republic of Korea

**Background**: Nanosize exosomes (30–150 nm) encapsulated by cell membrane play a key role for cell-to-cell communication, and are likely to carry genetic and molecular information from cell to cell. As according to the global cancer statistical reports, it was estimated that new lung cancer occurance was 222,500 and death was 155,870 in 2017. The mortality of lung cancer is the highest compared to other cancers, which reflects that the lung cancer would be better to be diagnosed at early stage.

**Methods**: Exosomes from non-small cell lung cancer (NSCLC) cells and human pulmonary artery endothelial cell (HPAEC) were isolated by column liquid chromatography and analysed by dynamic light scattering (DLS), nanoparticle tracking analysis (NTA) and western blotting (CD63). The exosomes were lysed and applied to proteomic analysis. Cancer cell lines transfected with pCMV-AC-GFP by Lipofectamine 2000 were transduced by lentiviral vectors containing LESP shRNA. The distribution and morphology of exosomes were examined by immunocytochemistry (ICC) and DLS.

**Results**: Five proteins were identified by the proteomics analysis of exosomes derived from NSCLC cell lines, but not HPAEC cell line. One protein was dramatically increased in NSCLC cell lines-derived and NSCLC patients-derived exosomes, but not normal HPAEC, by our quantitative RT-PCR and western blot analysis. The protein was named as lung cancer exosome-specific protein 1 (LESP1), which is involved in endosome-to-Golgi transport. Our study showed that cancer cells secrete more many exosomes than normal. In our LESP1 knock-down NSCLC cell line, the exosome secretion was dramatically decreased.

**Summary/conclusion**: The protein LESP1 was highly expressed in lung cancer exosomes and also can regulate the exosome release, which suggests that LESP1 may be a potential biomarker for early diagnosis of lung cancer.

**Funding**: This research was supported by a grant of the Ministry of Health & Welfare, Republic of Korea (HR14C0007) and from the Ministry of Science, ICT and Future Planning (2017M3A9C6026996) of the government of the Republic of Korea.

PT05.05

Exploiting lipidomics to unravel novel biomarkers for pancreatic cancer

Aikaterini Emmanouilidi ^1^; Peter J. Meikle^2^; Dino Paladin^1^; Marco Falasca^1^

^1^School of Pharmacy and Biomedical Sciences, Curtin Health Innovation Research Institute, Curtin University, Perth, Australia, Perth, Australia; ^2^Baker Heart and Diabetes Institute, Melbourne, Australia

**Background**: Pancreatic ductal adenocarcinoma (PDAC) accounts for approximately 90% of pancreatic malignancies, with a post-diagnosis 5-year survival rate of less than 5%. Due to lack of clinical symptoms, sufferers tend to be diagnosed late in the disease progression. In order to deliver effective therapies, specific biomarkers are needed for early detection. Lysophospholipids (LPLs) have been proposed as potential biomarkers for many forms of cancer. Furthermore, exosomes play a multifaceted role in cancer progression and are providing new opportunities for biomarkers discovery. We focus on the discovery of novel exosome-associated lipid biomarkers for PDAC and compare with prostate and ovarian cancer, to identify a specific PDAC signature.

**Methods**: Exosomes were successfully isolated from PDAC and normal pancreatic cell lines conditioned media, and together with their corresponding cells, they were subjected to lipidomic analysis using HPLC-ESI-MS/MS to detect over 700 lipid species from 25 lipid classes and subclasses. MS-based proteomic analysis was performed to verify the lipidomic findings.

**Results**: PDAC-derived exosomes were found to have a distinct lipid composition compared to their corresponding cells and exosomes derived from healthy cells. Exosome were greatly enriched in free and esterified cholesterol, compared to the derived cells, with a specific cholesteryl ester subclass being identified. Unusual lysolipid species were also detected, along with important proteins involved in lipid biology. A mutated form of the p53 protein was also verified, and its effect on the lipid metabolism was further explored.

**Summary/conclusion**: PDAC-derived exosomes not only carry precious lipidomic information which can be exploited for the discovery of novel prognostic and diagnostic biomarkers, but also provide us with crucial information about the tumour biology and progression of the disease.

**Funding**: Funded by Avner Pancreatic Cancer Foundation, AB Analitica and Biofield Innovation

PT05.06

Identification of human melanoma biomarkers by comparative exoproteoma analysis of melanocytes and melanoma cells

Andrea Agüera-Lorente; Aintzane Asumendi; Maria Dolores Boyano; Aintzane Apraiz

Department of Cell Biology and Histology, Faculty of Medicine and Nursing, University of the Basque Country, Leioa (vizcaya), Spain

**Background**: The metastatic capacity of tumours relies partially in their ability to modify local microenvironment and distant niches. Extracellular vesicles (EVs), and among them endosomal system-derived exosomes, have been shown to modulate other cells to favour metastasis providing them of special relevance in tumours such as malignant melanoma. The highly metastatic nature of malignant melanoma, even when identified in early stages (I–II), supports the need for molecular markers to accurately classified patients. In addition, EVs-based characterization of biomarkers could provide us with relevant information regarding essential communications molecules that could become therapeutic targets. Previous studies have focused on the characterization of the protein content of EVs derived from melanoma cells lines although there is no data comparing exoproteomes from melanocytes and malignant melanoma cells. The aim of this study is the identification of diagnostic and prognostic biomarkers that could represent key changes in EVs-mediated communication.

**Methods**: EVs derived from human melanocytes (HeMn-LP, HeMn-MP), primary melanoma (A375, MelHO) and metastatic melanoma (A2058, Colo800) cell lines were purified based on media concentration and differential ultracentrifugation. Exosomal content in the isolated EVs fraction was verified by electron microscopy, the presence of exosomal markers (e.g. TSG101, HSP90, CD81) and the absence of Calnexin. Samples were subjected to FASP and exoproteomes of the different cell lines determined by LC-MS/MS.

**Results**: Significant differences were obtained between the protein components enriched in the EVs of normal and tumour cells; exoproteomes derived from malignant cells were mainly enriched in proteins related to cell adhesion and the organization of the extracellular matrix, while cell–cell adhesion was the most enriched term in melanocytes. LC-MS/MS data revealed a malignancy-linked gradual increase on the extracellular matrix component SPP1 while cell–cell adhesion molecule CEACAM1 was decreased.

**Summary/conclusion**: Exoproteome analysis is able to discriminate melanocytes from malignant melanoma cells and identifies secreted SPP1 and CEACAM1 as putative disease biomarkers.

**Funding**: This study was supported by grants from the Basque Government (ELKARTEK 2017) and the UPV/EHU (IT-970-16).

PT05.07

Identification of diagnostic biomarker on circulating extracellular vesicles as a novel biomarker for colon cancer detection

Pyong-Gon Moon; Chan-Hyeong Lee; Eun-Ju Im; YouKyung Kim; Rakibul Alam; Moon-Chang Baek

Department of Molecular Medicine, Kyungpook National University School of Medicine, Jung-gu, Republic of Korea

**Background**: Extracellular vesicles (EVs) secreted from cancer cells have potential for generating cancer biomarker signatures. Currently, there are no molecular biomarkers for the detection of colon cancer. 

**Methods**: This study focused on identifying surface proteins found on circulating EVs for detecting colon cancer. In this study, isolated EVs from HT-29 and HCT-116 colon cancer cell lines were analysed using LC-MS/MS. Biomarker candidates among proteins that were identified in colon cancer cells were selected based on several filtering criteria.

**Results**: Five selected proteins were shown to be upregulated in colon cancer by western blot analysis. Tetraspanin-1 (TSPAN1), among the candidate proteins, was upregulated in small EVs from colon cancer patients compared to that of healthy controls. These results suggest that TSPAN1 is a potential noninvasive biomarker in detecting for colon cancer.

**Summary/conclusion**: This liquid biopsy to detect TSPAN1 on circulating EVs could be a promising method to detect colon cancer.

**Funding**: BK21 Plus KNU Biomedical Convergence Program,

Department of Biomedical Science, Kyungpook National University, Korea.

PT05.08

Exosomes as biomarkers for identification quantitation and stratification of chronic lymphocytic leukaemia

Sapir Cohen ^1^; Galia Luboshits^2^; Michael A. Firer^2^

^1^Ariel University, Qyriat Gat, Israel; ^2^Ariel University,Laboratory for Immunology and Cancer Biology, Ariel, Israel

**Background**: CLL is most common type of adult leukemia, molecular and clinically heterogeneous disease. CLL clinical staging is commonly made according to the Rai or Binet classifications. New molecular therapies for CLL have recently entered the clinic, but their long-term efficacy ultimately relies on correct and efficient stratification of patients. Additional biomarkers have also been tested but they are currently limited in their reliability and reproducibility. Research indicates that exosomes may play an important role in the development and progression of CLL, raising the prospect that easy detection of CLL-derived exosomes may lead to improved patient stratification and treatment.

**Methods**: The research was built on two *in vitro* models: mouse lymphoma line A-20 cells and human CLL plasma achieved from pretreatment CLL patients through collaboration with Professor Shpilberg (Assuta Medical Center Tel Aviv). For both models, exosomes were purified with size-exclusion Exo spin TM columns. Presence of exosomes was validated by western blot and SEM. The peptide discovery was achieved with phage display technology. A work flow process was deviced to ensure the removal from the phage pool of clones that bind exosomes in normal plasma, following plaque amplification and sequencing of phage DNA from third cycle. The resulting sequences were compared with normal peptide sequences.

**Results**: We discovered four peptides for the mouse model: SX1, SX3, SX4 and SX6. All of them have common motif of 4 amino acids that is different from the normal peptide discovered. We are now working on sequencing of the human peptide model. Following this, we predict discovering the novel biomarker on the membrane of CLL exosomes.

**Summary/conclusion**: The main aims of the research thus far have been to fulfil two goals.

1. To establish and calibrate the conditions required to isolate and identify leukemic cell-derived exosomes.

2. To establish proof-of-concept that phage display technology can be used to discover peptides that specifically bind leukaemic cell-derived exosomes. To do this, we began with mouse model systems using A-20 B-cell lymphoma cell cultures following the human CLL model. We expect this process to be validated with the arrival of discovered peptides from the mouse model which we await now and finishing the sequencing of the human peptide model.

PT05.09

Serum exosome concentration as a differential diagnosis marker for pulmonary tuberculosis and non-small cell lung cancer

Lei Zheng; Taixue An

Department of Laboratory Medicine, Nanfang Hospital, Southern Medical University, Guangzhou, China (People’s Republic)

**Background**: The 5-year survival rate of non-small cell lung cancer (NSCLC) patients was less than 16%. Pulmonary tuberculosis (pTB) is the disease most commonly misdiagnosed as lung cancer. A bulk of time and medical resources were consumed on distinguishing two diseases. Previous researches reported that EVs level will increase dramatically in tumourigenesis. However, the EVs level in pTB patients has not been determined. We suppose that serum EVs level of pTB patients may be different from cancer patients for their low immunity and weak physical conditions. Serum EVs concentration may sever as a diagnostic marker to distinguish two diseases.

**Methods**: We recruited volunteers from the Nang Fang Hospital, including three groups: NSCLC (*n* = 90), pTB (*n* = 55) and healthy individuals (*n* = 22). NSCLC patients were diagnosed by pathological biopsy, and pTB patients were diagnosed based on acid-fast staining of sputum smears. Subjects without lung shadows in X-ray tests, a history of tuberculosis or obvious symptoms of illness were enrolled into healthy group. Chemical reagent was utilized to precipitate EVs from serum. Isolated EVs were characterized by western bloting and electron microscope. The concentration and diameter were measured by the nanoparticle tracking analysis (NTA). Our research was approved and supervised by the Medical Ethics Committee of the hospital.

**Results**: We compared levels of serum EVs concentration in pTB, NSCLC and healthy group. Decreased EVs concentration in pTB patients and smaller EVs diameter in NSCLC patients with advanced stage were observed. With a cut-off of 7.7 × 1012 /ml, the AUC of serum EVs concentration reached 0.83 for discriminating pTB and total NSCLC, which is significantly higher than commonly used markers like CEA, CRP and WBC. It seems that serum EVs concentration is not correlated with individual age, sex, CEA, CRP and WBC.

**Summary/conclusion**: These findings suggest that the serum EVs concentration is significant different among pTB, NSCLC and healthy individuals, and may serve as a potential marker for the differential diagnosis of two diseases thereby aiding clinicians and decreasing the incidence of misdiagnosis.

**Funding**: This study was supported by grants from the National Natural Science Foundation of China (81371901).

PT05.11

Protein biomarker discovery in extracellular vesicles isolated from plasma for colorectal cancer

Keiko Kasahara ^1^; Ryohei Narumi^1^; Yuichi Abe^1^; Jun Adachi^1^; Kentaro Jingushi^2^; Yoshiharu Sakai^3^; Takeshi Tomonaga^1^

^1^Laboratory of Proteome Research, National Institute of Biomedical Innovation, Health and Nutrition, Ibaraki, Japan; ^2^Laboratory of Molecular and Cellular Physiology, Graduate School of Pharmaceutical Sciences, Osaka University, Suita, Japan; ^3^Department of Gastrointestinal Surgery, Graduate School of Medicine, Kyoto University, Kyoto, Japan

**Background**: The recent advance of mass-spectrometry-based technologies has been providing a quantitative proteomic analysis for biomarker discovery. However, it is still regarded as difficult to provide reproducibility in analysis for a small amount of protein in samples with high-complexity protein profile, such as plasma. Isolating extracellular vesicles (EVs) from plasma is one of ideal methods to overcome this problem in the point of reducing abundant proteins. We propose the challenges and considerations in EVs proteomics that may lead to identification of novel biomarkers.

**Methods**: In discovery phase, we isolated EVs from plasma in colorectal cancer (CRC) patients (*n* = 58) and healthy controls (*n* = 58) by using ultracentrifugation. Protein biomarker candidates for CRC were obtained by following strategies: (1) shotgun proteomics using LC-MS/MS to provide an overview of plasma EVs protein profile; (2) PubMed literature search for proteins that had been reported to be functionally correlated to the pathogenesis of CRC; (3) ExoCarta database research for proteins that had been reported their expression in EVs. LC-SRM/MS was used for targeted proteomics to obtain quantitative information for biomarker candidates. In validation phase, we used isolated EVs from plasma in CRC patients (*n* = 50) and healthy controls (*n* = 50) to evaluate the performance of biomarker candidates.

**Results**: The number of proteins was 1300 in (1), 900 in (2) and 5,400 in (3). We selected 400 proteins from PubMed literature search (2) on the basis of the identification by shotgun proteomics (1) or ExoCarta database search (3). We evaluated the performance of the candidates quantitatively by targeted proteomics using more than 1000 stable isotope-labelled synthetic peptides.

**Summary/conclusion**: These approaches would be efficient and powerful for biomarker discovery.

PT05.12

Protein signatures in exosome-like vesicles of uterine aspirates improve the diagnosis and stratification of endometrial cancer patients

Irene Campoy^1^; Cristian Moiola^1^; Marc Hirschfeld^2^; Jasmin Asberger^2^; Silvia Cabrera^3^; Xavier Matias-Guiu^4^; Jaume Reventos^1^; Eduard Sabidó^5^; Antonio Gil Moreno^6^; Pierre Thibault^7^; Eva Colas ^1^

^1^Group of Biomedical Research in Gynecology, Vall Hebron Institute of Research (VHIR), CIBERONC, Barcelona, Spain; ^2^Department of Obstetrics and Gynecology, University Medical Center, Albert-Ludwigs-University, Freiburg, Germany, Freiburg, Germany; ^3^Department of Gynecological Oncology, Vall Hebron University Hospital, Barcelona, Spain, Barcelona, Spain; ^4^Pathological Oncology Group and Pathology Department, University Hospital Arnau de Vilanova, and University Hospital Bellvitge, IRBLLEIDA and Idibell, University of Lleida. CIBERONC, Barcelona, Spain; ^5^Proteomics Unit, Universitat Pompeu Fabra (UPF) and Centre de Regulació Genòmica (CRG), Barcelona, Spain; ^6^Department of Gynecological Oncology, Vall Hebron University Hospital, CIBERONC, Barcelona, Spain, Barcelona, Spain; ^7^Proteomics and Bioanalytical Mass Spectrometry research unit. IRIC (Institute of Research in Immunology and Cancer), Montreal, Canada

**Background**: Endometrial cancer (EC) accounts for more than 10,000 deaths per year in the US alone. EC is divided into the more common and less aggressive type 1 and the type 2. There is an urgent need to develop non-invasive tests that can provide early detection of EC and that can discriminate EC subtypes. This study focuses on the identification of protein biomarkers in exosome-like vesicles (ELVs) isolated from uterine aspirates. Uterine aspirates are collected by a minimally invasive procedure and it represents the ideal body fluid since it is the closest to the neoplasic endometrium cells. 

**Methods**: Protein extracts from purified ELVs were obtained following ultracentrifugation of UAs from age-matched groups of control, type 1 and type 2 EC patients (10 patients/group). The quality of ELVs was monitored by nanoparticle tracking analysis and immunoblot. To profile protein abundance across different groups, we develop a super-SILAC approach where ELV proteins from three different EC cell lines grown in heavy amino acids were combined with ELV protein extracts of each patient. Proteins were separated by SDS-PAGE and 10 gel-isolated bands per patient were digested with trypsin and analysed by mass spectrometry. From 2138 proteins identified, we generated a list of 54 protein candidates that were further validated by selected reaction monitoring (SRM) in an independent cohort of 107 patients including type 1 EC (*n* = 45) EC, type 2 EC (*n* = 21) and controls (*n* = 41). A total of 86 unique peptides matching the proteins of interest were monitored. Protein quantitation was performed using a QTRAP 5500 Sciex instrument.

**Results**: Our targeted mass spectrometry approach confirmed that ELVs from uterine aspirates contain proteins that can discriminate between cancer patients and healthy individuals, and can classify EC in the different subtypes. A 2-protein signature, composed of Agrin and CD81, achieved an AUC = 0.935 for EC diagnosis. In addition, we also report a new protein signature, combining CLD6, and RAB8A, that can differentiate type 1 versus type 2 EC (AUC = 0.932). This study has important implications in early detection of EC and in patient stratification.

**Summary/conclusion**: A targeted mass spectrometry approach defines protein signatures for endometrial cancer diagnosis and stratification of patients in ELVs isolated from uterine aspirates.

PT05.14

Proteome analysis of glioma-derived extracellular vesicles isolated from neurosurgical aspirates provide markers for disease stage and progression

Susannah Hallal ^1^; Ben Russell^1^; Brindha Shivalingam^2^; Michael Buckland^2^; Kimberley Kaufman^1^

^1^The University of Sydney, Sydney, Australia; ^2^Royal Prince Alfred Hospital, Sydney, Australia

**Background**: Glioblastomas (GBM) are highly lethal brain tumours with limited treatment options available to patients. Non-invasive liquid biopsies that monitor GBM progression are important for developing personalized therapies for GBM. GBM extracellular vesicles (GBM-EVs) play key roles in GBM biology and are detectable in the peripheral circulation. However, profiling GBM-EVs from the blood remains an obstacle as they are a minor subset of the total blood EV population. We investigated whether our previously described *in vitro* GBM-EV proteome signature could be translated to GBM-EVs isolated from clinical sources that are rich in brain tumour EVs, i.e. Cavitron Ultrasonic Surgical Aspirate (CUSA) fluid. 

**Methods**: EVs were harvested from CUSA fluid by ultracentrifugation and enriched on a discontinuous iodixanol/sucrose gradient. Nanoparticle tracking analysis and transmission electron microscopy confirmed the presence of “exosome” sized (~100 nm) and vesicular-shaped particles in CUSA fluid, and the proteomes of enriched CUSA-EVs from GBM (*n* = 3) and low-grade astrocytoma (*n* = 3) were analysed by quantitative label-free LC-MS/MS. SLHD HREC approval was obtained and patients provided informed consent.

**Results**: Multiple proteins were identified in the CUSA-EVs that are associated with glioma biology (EGFR, IDH1, vimentin, CD53). There was a substantial overlap of the CUSA-EV proteins with our *in vitro* GBM-EV proteomic signature, with GBM CUSA-EVs sharing 76% of GBM signature proteins and low-grade astrocytoma CUSA-EVs sharing 60%. EV proteins previously correlated to GBM cell invasiveness *in vitro* (ANXA1, IGF2R, ITGB1, PDCD6IP, ACTR3, CALR, IPO5, MVP, PSMD2) were also significantly increased in GBM CUSA-EVs compared to low-grade astrocytomas. Interestingly, significantly higher levels of all molecular chaperone T-Complex Protein 1 Ring Complex (TRiC) subunits, which are associated with multiple oncogenes and play roles in tumour invasion, were identified in GBM CUSA-EVs.

**Summary/conclusion**: CUSA fluid constitutes a novel and rich source of brain tumour EVs, sufficient to elucidate and validate potential prognostic biomarkers. With further study, these targets could offer avenues for tumour staging and monitoring GBM progression via peripheral blood sampling of GBM-EVs.

PT05.15

Identification of novel targets for colorectal cancer liquid biopsy by proteome-wide profiling of EVs from cultured viable tumour tissues

Makoto Sumazaki ^1^; Kentaro Jingushi^2^; Hideaki Shimada^3^; Koji Ueda^4^

^1^Project for Personalized Cancer Medicine, Cancer Precision Medicine Center, Japanese Foundation for Cancer Research, Koto-ku, Japan; ^2^Laboratory of Molecular and Cellular Physiology, Graduate School of Pharmaceutical Sciences, Osaka University, Suita, Japan; ^3^Department of Clinical Oncology, Toho University Graduate School of Medicine, Ota-ku, Japan; ^4^Cancer Proteomics Group, Cancer Precision Medicine Center, Japanese Foundation for Cancer Research, Tokyo, Japan, Koto-ku, Japan

**Background**: Early detection of colorectal cancer (CRC) is essential for improvement of prognosis by enabling therapeutic intervention at early stage. Recently, it has been shown that extracellular vesicles (EVs) could have potential to be served as attractive biomarker carriers in any body fluids. To explore CRC-specific diagnostic antigens on EVs, we isolated EVs from cultured colorectal normal or tumour tissues and performed global quantitative proteome analysis.

**Methods**: Tissue-exudative EVs (Te-EVs) were obtained from serum-free media of freshly resected CRC tissues and adjacent normal mucosa using the sequential ultracentrifugation method (*n* = 20). A quantitative expression profile of Te-EV proteins was acquired using Orbitrap Fusion Lumos LC/MS system (Thermo Scientific) and MaxQuant software. A statistically valid biomarker candidate protein (TMAM) was further evaluated by plasma exosome sandwich ELISA (*n* = 30). Additional clinical and functional assessments were also performed including IHC staining and EV incorporation assays.

**Results**: Among 6371 identified Te-EV proteins, 616 proteins were significantly overexpressed (*p* < 0.05 and fold-change >4.0) in EVs from CRC tissues compared to those from paired normal mucosa. We especially focused on multi-pass transmembrane protein TMAM (*p* = 3.62 E-5, fold change = 7.0) which was known to be a key regulator of cell growth and also overexpressed in CRC cells. Importantly, TMAM level on plasma EVs from CRC patients (*n* = 20) were significantly higher than those from healthy donors (*n* = 10) in exosome sandwich ELISA using independent sample set (*p* = 0.036). IHC staining analysis also demonstrated that TMAM specifically overexpressed in CRC cells. Interestingly, TMAM overexpressed EVs decoyed its inhibitory ligand away from cancer cells, leading to growth upregulation.

**Summary/conclusion**: These results indicated that TMAM on EVs should have great potential as a novel target for CRC diagnosis and therapy.

PT05.16

Chloride intracellular channel protein 4 (CLIC4) is a serological cancer biomarker released from tumour epithelial cells via extracellular vesicles and required for metastasis

Vanesa C. Sanchez ^1^; Alayna Craig-Lucas^1^; Bih-Rong Wei^2^; Abigail Read^2^; Mark Simpson^2^; Ji Luo^1^; Kent Hunter^2^; Stuart Yuspa^1^

^1^National Institutes of Health (NIH), Bethesda, USA; ^2^LCBG NCI NIH, Bethesda, USA

**Background**: CLIC4 is a highly conserved metamorphic protein originally described as an ion channel. It translocates to the nucleus serving as an integral component of TGF-β signalling. In multiple cancers, CLIC4 is a tumour suppressor, excluded from the nucleus and lost from the cytoplasm of progressing cancer cells. In contrast, CLIC4 is upregulated in the tumour stroma acting as a tumour promoter. CLIC4 lacks a secretory sequence, but recent reports indicate that CLIC4 is detected in the circulation of cancer patients serving as possible biomarker and has been detected in extracellular vesicles (EVs).

**Methods**: EVs from cell culture supernatants or biological fluids were isolated by differential centrifugation, following ultracentrifugation and Optiprep density gradients. EV size distribution and concentration were analysed by NTA and TEM. The presence of prototypical markers and CLIC4 was analysed by immunoblot.

**Results**: CLIC4 was present in EVs released from primary normal and multiple ovarian and breast tumour cell lines. Substantial increases in CLIC4 were measured in EVs of tumour cells when compared to normal cells. TGF-β-induced myofibroblasts also increased CLIC4 in both the cells and the EVs they released. *In vivo*, CLIC4 levels increased in EVs released into the peritoneal cavity as tumour burden increased in a heterotopic xenograft ovarian cancer model. Moreover, CLIC4 levels in EVs isolated from plasma increased with tumour burden and lung metastatic load in orthotopic syngeneic mouse breast cancer models. To dissect the contribution of stromal vs tumour epithelial compartments as the source of the CLIC4-high EVs, CLIC4 was either deleted in tumour cells lines by CRISPR/Cas9 or CLIC4 KO females were implanted CLIC4 WT tumour cells. CLIC4 is reduced in circulating EVs from CLIC4 KO tumour bearing mice when compared to WT and it is present in circulating EVs from CLIC4 KO females bearing WT tumours, indicating that the major contribution of CLIC4 into circulation is from tumour epithelium. Moreover, CLIC4 KO females display no difference in primary tumour size and a significant reduction in both size and number of lung metastases.

**Summary/conclusion**: CLIC4 levels in EVs from biological fluids may have value as a cancer biomarker, in conjunction with other markers, to detect or analyse tumour progression or recurrence.

PT05.17

Bioinformatics analysis of metabolites present in urinary exosomes identify metabolic pathways altered in prostate cancer

Marc Clos-Garcia ^1^; Pilar Sanchez-Mosquera^2^; Patricia Zuñiga-García^2^; Ana R. Cortazar^2^; Verónica Torrano^2^; Ana Loizaga-Iriarte^3^; Aitziber Ugalde-Olano^3^; Isabel Lacasa^4^; Félix Royo^5^; Miguel Unda^3^; Arkaitz Carracedo^2^; Juan M. Falcón-Pérez^5^

^1^Exosomes Laboratory, CIC bioGUNE, Derio, Spain; ^2^CIC bioGUNE, Derio, Spain; ^3^Basurto University Hospital, Bilbao, Spain; ^4^Hospital Basurto, Bilbao, Spain; ^5^CIC bioGUNE, CIBERehd, Bizkaia Science and Technology Park, Derio, Bizkaia, Spain, Derio, Spain

**Background**: Metabolomics is an omics discipline with high potential to identify new biomarkers, but it is limited to metabolites, lacking of information on the context and/or integration into metabolic pathways. Previously, using metabolomics data obtained from urine EVs, we identified altered metabolites between prostate cancer (PCa) patients and benign hyperplasia (BPH) patients. In the current work, we developed a bioinformatics workflow to identify gene-encoding proteins involved in the metabolism of those metabolites and to map them into metabolic pathways. Using publicly available, gene expression for prostate cancer datasets, we identified several genes which regulation was altered, in agreement with the alterations observed at the metabolite level.

**Methods**: R scripts were developed for retrieving information from KEGG and HMDB database, specifically, enzymes and genes related to the metabolites of interest. Combining both genes and metabolites lists, the script searched for metabolic pathway that could be altered. Finally, gene expression data was analysed in available databases for those genes of interest.

**Results**: We detected 76 metabolites that were significantly different between prostate cancer and benign prostate hyperplasia. We identified 149 enzymes involved in the metabolism of those metabolites. From them, the levels of their encoding genes were evaluated in the PCa gene expression data sets. As a result, the levels of 7 gene-encoding enzymes were found altered in PCa and were in concordance with the metabolite levels observed in urinary EVs. Our results indicate that steroid hormones, leukotriene and prostaglandin, linoleate, glycerophospholipid and tryptophan metabolisms and urea and TCA cycles, are altered in PCa.

**Summary/conclusion**: In this work, we demonstrated that bioinformatics tools applied for combining of metabolomics and gene expression data are able to identify metabolic pathways that help to explain the metabolic phenotype of PCa, and provide novel therapeutics targets. This work also supports the study of urinary EVs as surrogate metabolic non-invasive markers for PCa tissue metabolism.

PT05.18

Optimization of storage conditions for extracellular vesicles

Michel Bremer ^1^; Verena Börger^1^; André Görgens^2^; Samir El-Andaloussi^2^; Bernd Giebel^3^

^1^Institute for Transfusion Medicine, University Hospital Essen, University of Duisburg-Essen, Essen, Germany; ^2^Clinical Research Center, Department for Laboratory Medicine, Karolinska Institutet, Stockholm, Sweden, Hälsovägen, Sweden; ^3^Institute for Transfusion Medicine, University Hospital Essen, Essen, Germany

**Background**: EVs transmit specific information from their cells of origin to specific target cells and are key factors in a novel form of intercellular communication. Regularly, the EVs functional properties are analysed following their purification and often they have been frozen and thaw before analysis. Storage, especially freezing and thawing, might critically affect the integrity and functionality of respective EVs. Indeed, preliminary data of our group showed that long-time storage can reduce the number of particles recovered after freezing and thawing. To optimize EV storage conditions, we have studied the impact of different buffers and storing containers on recovery rates of stored eGFP-labelled EVs.

**Methods**: In detail, eGFP-labelled EVs were harvested from supernatants of THP-1 cells, which had been transduced with CD63-eGFP encoding lentiviral particles. By ultracentrifugation purified EVs were resuspended in six different buffers and aliquots of resulting suspensions transferred into 12 different plastic/glass containers each. Containers were either stored at 4°C, −20°C or −80°C. Following a defined storage time and after multiple freezing and thawing cycles, stored samples were analysed by nanoparticle tracking analysis (NTA).

**Results**: We observed a reduced recovery of particles during most storage conditions. Independent of the storage temperatures, isotonic and pH-controlled buffers appeared preferable. Remarkably, the choice of storage containers and the storage temperature had massive effects on the particle concentrations and average size distributions of stored EVs. While EV rates were rather constant when stored at −80°C, EV numbers varied significantly when stored at −20°C or 4°C, respectively. Currently, we test for the functionality of stored EVs.

**Summary/conclusion**: Combinations of storage containers, buffers and temperature significantly affect recovery rates of stored EVs.

**Funding**: This research was funded by European Regional Development Fund 2014-2020 (EFRE) and European Union.

PT06: EVs in Cellular Differentation and Organ Development Chairs: Ana Gamez Valero; Guillaume van NielLocation: Exhibit Hall 7:15–18:30

PT06.01

Driving patched to the bottom: vesicular trafficking to polarize Hh reception

Ana C. Gradilla; Laura González-Méndez; Isabel Guerrero

Centro de Biologia Molecular Severo Ochoa (CSIC-UAM), Madrid, Spain

**Background**: The Hedgehog (Hh) signalling pathway is essential for early animal development and tissue maintenance in the adult. The lipid-modified Hh acts as a morphogen and signals in a graded manner, achieving differential responses in the receiving cell according to ligand concentration. Thus, the extracellular ligand distribution of the membrane anchored Hh is a key factor of signalling during morphogenesis. We have found that Hh is packed to be released in exovesicles associated to filopodia-like structures (cytonemes) that extend basolaterally in Drosophila polarized epithelia. Recycling of the ligand from the apical to the basolateral side of the epithelium has been demonstrated to be essential for Hh inclusion in MVBs and exovesicles. Furthermore, basolateral colocalization of Hh and the Hh receptor complex at cytoneme contacts has been shown. Here, we investigate the potential recycling mechanism for the extracellular presentation of the Hh receptor patched (Ptc) in cytonemes. 

**Methods**: We have performed mass spectrometry analysis of Ptc interactors after tagged overexpression and protein-trap isolation from *Drosophila* developing tissues. A broad genetic screening and phenotypic analysis have also been performed using RNAi treatment against vesicle trafficking regulators as ESCRT and Snare complexes.

**Results**: Loss-of-function clonal analysis has shown that both ESCRT and Snare are needed for Ptc basal extracellular presentation and Hh normal reception. Besides, Snare proteins such as Sec22 have been found in the Ptc interactome. We are currently assessing through electron microscopy the potential inclusion of Ptc into MVBs and the type of vesicles for its extracellular presentation.

**Summary/conclusion**: We demonstrate that Ptc recycles as Hh from the apical to the basolateral side of polarized developmental epithelia, previous to cytoneme-mediated distribution. This recycling process for Ptc extracellular presentation at the basolateral side and for normal Hh reception requires ESCRT and Snare proteins.

**Funding**: Grants BFU2014-59438-P to IG, BFU2015-73609-JIN to ACG and SAF2015-71231-REDT to REDiEX consortium, all from the Spanish Ministry of Economy and Competitiveness (MINECO).

PT06.02

Articular chondrocytes-derived EVs regulate osteoclastogenesis, but not osteogenesis

Yohei Sanada ^1^; Shigeru Miyaki^1^; Nobuo Adachi^2^

^1^Hiroshima University Hospital, Hiroshima, Japan; ^2^Hiroshima University, Hiroshima, Japan

**Background**: Osteoarthritis (OA) represents the most common musculoskeletal disorder. It is a whole joint disease, characterized by the degradation of articular cartilage, subchondral bone remodelling. Extracellular vesicles (EVs) such as exosomes have attracted attention as novel a mechanism of communication among joint tissues, but the fundamental mechanisms are still unknown. We hypothesized that EVs from articular chondrocytes (AC) function as a novel paracrine factor for joint homeostasis. The purpose of this study is to examine the function of EVs from cultured AC in osteogenesis and osteoclastogenesis.

**Methods**: Mouse AC were isolated by collagenase digestion of femoral head cartilage from 4-week-old male C57/B6 mouse. Mouse AC were cultured with 10% DMEM in 3~5 × 10^5^ cells/well for 48 h. Large EVs (10K) and small EVs (100K) from condition media (CM) were collected by differential ultracentrifugation. Supernatants also were collected as EVs-depleted CM. We confirmed isolated EVs by western blots, using an antibody against the commonly found EV marker proteins such as flotillin-1, tetraspanin CD9 and CD81. To evaluate the effect of AC-derived EVs (10K or 100K) on osteoclastogenesis and osteogenesis, mouse bone marrow derived cells (BMDCs) and osteoblastic cell line MC3T3-E1 were used. BMDCs and MC3T3-E1 were cultured with each differentiation media in the presence of EVs (10K or 100K). Osteoclast cells were stained with a commercial kit for tartrate-resistant acid phosphatase (TRAP), and multinucleated cells with >4 nuclei were counted as TRAP-positive osteoclast cells. Osteogenic differentiation was verified by alkaline phosphatase staining.

**Results**: Flotillin-1, CD9 and CD81 were highly expressed small size EVs (100K), but these protein levels in large size EVs (10K) were at low level. Although AC-derived EVs (10K and 100K) were inhibited osteoclastogenesis, EVs (100K) were significantly inhibited osteoclastogenesis compared with FBS derived control EVs and EVs (10K). On the other hand, AC-derived EVs (10K and 100K) had no effect on osteogenesis.

**Summary/conclusion**: This present study demonstrated that AC-derived EVs, small EVs (100K) regulate osteoclastogenesis, but not osteogenesis. AC-derived EVs are new communication factor in joint homeostasis, and might be involved in subchondral bone changes for OA development.

PT06.03

Mechanical force induced EV-miRNAs play a role in foetal lung development

Tanbir Najrana; Goldberg Laura; Peter Quesenberry; Juan Sanchez-Esteban

Brown University, Providence, USA

**Background**: During development, cells communicate each other for the growth in specific patterns of tissues/organs. Cells achieve this by sending and receiving the signals. Cell uses release of extracellular vesicles (EVs) as one of the developmental signals. EVs are membrane bound particles rich in miRNA with other bioactive molecules. Incomplete development of the lung can cause neonatal death and morbidity. There is no specific treatment that can stimulate the growth of the lung. Lung morphogenesis has significant dependence on mechanical signals. However, the mechanism by which mechanical force promotes lung development is not well-characterized. miRNAs have an important role in foetal lung development and have shown the expression is gradually increased and shifted from mesenchymal cells to epithelial cells as development progressed. Given that physiological mechanical signals release EVs and miRNAs are key components of the EVs cargo, we hypothesize that *mechanical force-induced EV-miRNA promotes foetal lung development*. **Purpose**: To identify the mechanical force EV-miRNA induced contributes to the lung development using mouse lung epithelial cell MLE12 *in vitro*. 

**Methods**: MLE12 culture was exposed to 5%, 10% and 20% cyclic mechanical stretch for 24 h in collagen-I-coated bioflex plate. Condition medium was collected and EVs were isolated using differential centrifugation. Cells in static condition were used as control. Size and quantity of EVs were determined by NanoSight device. Cell viability was analysed using live/dead cell reagent SYTOX Red. Equal amounts of EVs for stretch and static condition were used to isolate small RNA to subject to micro array assay to analyse the miRNA profile.

**Results: About** 1.5-, 2.5- and 10-fold increase of release of EVs from MLE12 cells were according to the increase of cyclic stretch. No cell death and injury were measured.

**Summary/conclusion**: As miRNA is a key cargo of EVs, we expect to identify that stretch induced EV-miRNA involves in lung development as we are completing the miRNA profile analysis. We tested before the presence of EVs in mouse faetal lung. Future studies will test this hypothesis using animal models.

**Funding**: COBRE for perinatal Biology Pilot Project Award Program

Oh-Zopfi Pilot Project Grant Program.

PT06.04

Cells interactions and cells modifications via exosomes

Alexandr Abramov ^1^; Alisa Petkevich^2^; Vadim Pospelov^1^; Kiselevskiy Mikhail^2^

^1^Scientific and Practical Center of children medical care, genetics department, Moscow, Russia; ^2^N. N. Blokhin Russian Cancer Reserach Center, Institute of experimental tumous diagnosis and treatment, laboratory of cell immunity, Moscow, Russia

**Background**: Exosomes play pivotal role in intercellular messaging and are still one of promissing ways of drugs and target molecules delivery and recently of vectors dlivery as well. Anyway, even non-enriched exosomes may show some impact on cells, this may clarify pathology basis of diseases and, moreover, make exosomes one of possible instrument for cells modifications, opening a new prospects for therapeutic strategies.

**Methods**: Cells of AML were incubated for 72 h by standard protocol; supernatant was analysed for exosomes by WB, exo-miRNAs and free miRNAs by qRT-PCR (let-7a, let-7b, mir-19a, mir-106a, mir-149, mir-155, mir-199a, mir-214, mir-221, mir-222). PAGE was performed; miRNA concentration was determined by NanoDrop. Bone marrow mononuclear cells (BMNC) were incubated with this supernatant for 72h. Immunophenotype (IFT) of BMNC was analysed by flow cytometry (mAb: CD45, CD34, CD14, CD127, CD3 (BD Biosiences, USA; Beckman Canto II) after 24, 48 and 72 h of incubation with supernatant of AML cells; there were control group and group with supernatant of K562 cells obtained by the same method. qRT-PCR for above-mentioned exo-miRNAs and free miRNAs was performed at the same time points. Simultaneously, there was an attempt to recreate en effect of proanthocyanidin obtained from *Vaccínium uliginósum* via exosomes provided by cells incubated with this substrate. Exosomes purifying from substrate was performed by multistep filtration and ultracentrifugation and assessed by high-performance liquid chromatography Agilent 1290 (AgilentTechnologies).

**Results**: There was shift in IFT of cells incubated with supernatant of AML cells and K562 for 48 and 72 h: CD45, CD127 and CD14 expression increased in comparison with control group, miRNA concentration in supernatant of cells incubated with AML supernatant also changed in comparison with control groups. There was similar effect on AML cells of proanthocyanidin and exosomes of cells incubated with proanthocyanidin according to IFT data (Ki67 expression decreased in comparison with control group).

**Summary/conclusion**: Obtained data show exosomes may have some impact on cells modifications and serve as putative instruments for novel therapeutic strategies. Anyway, further research is necessary to verify this effect and reveal specific mechanisms that maybe involved in its development.

PT06.05

Characterization of osteoblast-derived exosomes of distinct emryonic origin

Hadil Al Jallad ^1^; Monther Abu Hantash^2^; Reggie Hamdy^3^

^1^Shriners Hospital for Children-Canada, Montreal, Canada; ^2^McGill University-Department of Experimental Surgery, Montreal, Canada; ^3^McGill University/Shriners Hospital for Children, Montreal, Canada

**Background**: The bony tissue is a dynamic structure that has the capacity to self-remodel and damage repair with new bone formation. When the damage, however, results in a large segmental defect that exceeds bone’s ability to self-repair, a surgical intervention is required for healing to ensue. It has been reported that approximately 2–10% of bone fractures may develop non-union due to insufficient bone growth. The current “gold standard” treatment in the clinical settings promotes bone regeneration through the use of autologous and allogeneic bone grafting. However, approximately 20–30% of patients who undergo autologous bone grafts suffer from morbidity at the graft-harvesting site, and limited supply of graft material – a particular challenge in pediatric patients. Despite current advances in reconstructive orthopaedic techniques, managing bone non-union is challenging to the patient and the surgeon. Thus, the need to develop safe and effective bone regeneration therapy is of high demand. Exosomes ranging from 30 to 100 nm in diameter have been shown to induce osteogenesis *in vitro* and *in vivo*. We hypothesize that osteoblast embryonic origin is a critical factor in dictating the osteogenic potential of Ob-derived exosomes.

**Methods**: Two-day-old C57BL6 mouse pups will be used to isolate primary osteoblasts (OBs) from frontal, parietal and long bones. The bones will be scraped of periosteum and then sequentially digested using collagenase. OBs will be cultured in alpha MEM media supplemented with 10% exosome-free FBS, 1% pencilline and streptomycin; for osteoblast differentiation, cells will be grown in osteogenic medium containing 50 ug/ml ascorbic acid and 10 mM beta glycerophosphate. Exosomes will be purified as described previously by Thery et al. (2006).

Exosomal proteins will be identified by mass spectrometry, while RNA profile will be determined by RNA sequencing.

**Results**: OB embryonic origin dictates distinct OB exosomal content.

**Summary/conclusion**: Exosomes derived from osteoblasts of distinct embryonic origins exerted different osteogenic regenerative capacities.

**Funding**: This project is not funded.

PT06.06

Secretion mechanisms of Wnt proteins

Alena Ivanova; Oksana Voloshanenko; Jan Winter; Michael Boutros

Division of Signaling and Functional Genomics, German Cancer Research Center (DKFZ), Heidelberg, Germany

**Background**: The Wnt signalling pathway plays an important role during development, carcinogenesis and many other diseases. Wnt proteins – key players in intercellular signalling – can travel through extracellular space, but having lipid modifications rendering them insoluble, they need to use special carriers. According to the current understanding of Wnt secretion, Wnt proteins are transported with the cargo receptor Evi/WIs from the ER through Golgi to the plasma membrane. To contact responding cells, Wnts can migrate through heparan sulphate proteoglycan chains on cell surface and transported on filopodia. Additionally, Wnts can be solubilized by binding to interacting proteins or form micelle-like structures to travel in the intracellular space. Wnt proteins can be recycled through the endosomal compartment and secreted on exosomes [1].

[1] Gross JC, Chaudhary V, Bartscherer K, Boutros M. Active Wnt proteins are secreted on exosomes. Nat Cell Biol. 2012;14:1036–1045.

**Methods**: Here, we established genetic tools to identify genes which are important for the particular types of Wnt proteins secretory pathways. We use CRISPR/Cas9 screening technologies for targeted disruption of genes in combination with Wnt activity assays to identify genes that are required for the secretion of functional canonical Wnt proteins.

**Results**: With the described approach, a panel of possible secretory factors have been tested. Knock-out of several targeted genes led to reduction in the secretion of functional WNT3 protein. Observed phenotypes were validated with western blots and TCF4/Wnt reporter read-out.

**Summary/conclusion**: Obtained results indicate that established approach can be used to identify new positive WNT3 secretion regulators. In summary, the established tools will contribute towards the understanding of Wnt trafficking and their secretion routes.

PT06.07

Extracellular vesicular miRNAs in osteoblastogenesis

Clare (Chi-Chih) Chang ^1^; Jørgen Kjems^2^; Yan Yan^2^; Morten Venø^3^; Junyi Su^4^

^1^Aarhus University, Risskov, Denmark; ^2^Interdisciplinary Nanoscience Center (iNANO), Aarhus University, Denmark, Aarhus, Denmark; ^3^Department of Molecular Biology and Genetics, Aarhus University, Aarhus, Denmark, Aarhus, Denmark; ^4^Aarhus University, Aarhus C, Denmark

**Background**: Mesenchymal stem cell (MSC) therapy by infusion therapy has been studied both in pre-clinical models and in clinical trials, and it was shown that most systemically administered MSCs cleared within a week. Despite, the short lifespan, MSCs alleviated different pathologies such as myocardial infarction and graft versus host disease. Recent studies have shown that the therapeutic effects of MSCs are attributed to their paracrine factors. One subset of these factors is excreted via extracellular vesicles (EVs). The aim of this study is to identify microRNAs (miRNAs) in EVs before and after differentiation to osteoblasts to identify factors that may contribute towards bone regeneration.

**Methods**: Bone marrow MSCs are isolated from three donors and their EVs were collected before and after osteoinduction for 7 days. The EVs were characterized using nanoparticle analysis, western blotting and TEM. The small RNAs therein were then sequenced and analysed.

**Results**: miRNAs were differentially expressed in EVs after osteoinduction and the expression of miRNAs in EVs were distinct from those in cells after osteogenesis. This indicates that EVs are not simply small replicas of their parental cells. EVs had a smaller subset of miRNAs compared to their parental cells, and most of the miRNAs enriched or depleted in EVs were similar – regardless of the state of differentiation. A G**G motif was identified in EV enriched miRNAs, which suggests preferential loading of miRNAs containing this motif. Finally, for functional studies, exosomes were purified added to the osteoinduction media during differentiation. We found that D0 BMSC EVs increased the expression of RUNX2 compared to the control.

**Summary/conclusion**: In summary, we have profiled the miRNA cargo of bone marrow MSC EVs before and after 7 days of differentiation *in vitro*. The expression of the miRNAs in the EVs was distinct before and after differentiation. Furthermore, the miRNAs from the EVs were different from that of their parental cellular miRNAs, which suggests a functional role for the miRNAs that are selectively incorporated into EVs. Interestingly, the miRNAs incorporated had a G**G motif, which could contribute towards cargo selection.

PT06.08

Functionality of bone marrow mesenchymal stem cell-derived extracellular vesicles on self-renewal

Juan Antonio Fafian Labora ^1^; Miriam Morente-López^2^; Patricia Díaz-Barreiro^2^; Pablo Fernandez-Pernas^2^; María Arufe Gonda^2^

^1^Grupo de Terapia Celular y Medicina Regenerativa (TCMR-CHUAC), CIBER BBN/ISCIII, Instituto de Investigación Biomédica de A Coruña (INIBIC), Complexo Hospitalario Universitario de A Coruña (CHUAC), SERGAS, Departamento de Ciencias Biomédicas, Medicina y Fisioterapia, Facultade 1Grupo de Terapia Celular y Medicina Regenerativa (TCMR-CHUAC), CIBER BBN/ISCIII, Instituto de Investigación Biomédica de A Coruña (INIBIC), Complexo Hospitalario Universitario de A Coruña (CHUAC), SERGAS, Departamento de Ciencias Biomédicas, Medicina y Fisioterapia, Facultade de Oza, Universidade da Coruña (UDC), As Xubias, A Coruña, Spain. Oza, Universidade da Coruña (UDC), As Xubias, A Coruña, Spain., A Coruña, Spain; ^2^Grupo de Terapia Celular y Medicina Regenerativa (TCMR-CHUAC), CIBER BBN/ISCIII, Instituto de Investigación Biomédica de A Coruña (INIBIC), Complexo Hospitalario Universitario de A Coruña (CHUAC), SERGAS, Departamento de Ciencias Biomédicas, Medicina, A Coruña, Spain

**Background**: Mesenchymal stem cells (MSCs) are highly relevant for regeneration of mesoderm tissues. The promising role of MSCs in cell-based therapies and tissue engineering is limited due to a decline pluripotency, proliferation and immunogenic potential with increasing donor age. Extracellular vesicles (EVs) have been recognizing as potent vehicles of intercellular communication on different biological systems. MSC-derived EVs present advantages over cell-based therapy as it eliminates the safety concerns associated with injection of MSCs in patients and particularly useful for enhancing recovery from various diseases. Here, the influence of ageing on self-renewal functionality MSC-derived EVs using an *in vitro* model was studied.

**Methods**: Two age groups from bone marrow MSCs of male Wistar rats were used, old (3 months old) and young (14 days old). EVs were isolated using ultracentrifugation and characterized by nanoparticle tracking analysis, flow cytometry and electronic microscopy. MSCs from young individuals (yMSCs) were treated with MSC-derived EVs from old group (oEVs) and vice versa. The internalization of marked EVs with DiI was evaluated using fluorescence microscopy. Besides, self-renewal markers (Nanog, Oct4, Vinculin and lamin A/C) were evaluated at RNA and proteomic levels at several times of treatment (2, 3 and 6 days).

**Results**: It was observed internalizing of oEVs in yMSCs at 2 days and increase of EV uptake along the time, the similar result was observed in oMSCs treated with yEVs. With respect to pluripotency markers at RNA level, a statistically significant decrease of pluripotency markers (Nanog and Oct4) in yMSCs treated with oEVs and a statistically significant increase in oMSCs treated with yEVs were obtained. On the contrary, it was obtained with respect Vinculin expression. At proteomic level, a statistically significant increase of three isoforms of Lamin A/C in yMSCs treated with oEVs and statistically significant decrease in oMSCs treated with yEVs were obtained.

**Summary/conclusion**: MSC-derived EVs and their self-renewal capacity were influenced by age, as reveled by changes in the expression of self-renewal markers such as Nanog, Oct4, Vinculin and Lamin A/C. These findings are important to the understand the influence of the ageing on MSCs and to advance in the development EV-based therapies.

PT06.09

Ex vivo expansion of umbilical cord blood-derived haematopoietic stem cells using extracellular vesicles of bone marrow stromal origin

Corina Ghebes ^1^; Jess Morhayim^2^; Marion Kleijer^1^; Bram van der Eerden^3^; Jeroen van de Peppel^3^; Eric Braakman^2^; Carlijn Voermans^1^

^1^Department of Hematopoiesis, Sanquin Research, Amsterdam, The Netherlands; ^2^Department of Hematology, Erasmus University Medical Center, Rotterdam, The Netherlands; ^3^Department of Internal Medicine, Erasmus University Medical Center, Rotterdam, The Netherlands

**Background**: Ex vivo expansion of umbilical cord blood (UCB)-derived haematopoietic stem and progenitor cells (HSPCs) is a promising approach to promote engraftment and accelerate haematopoietic recovery in patients eligible for UCB stem cell transplantation. However, expansion of immature HSC has remained a challenge. Extracellular vesicles (EVs) represent uncovered means of intercellular communication via the transfer of bioactive lipids, proteins and RNAs that can regulate cell fate of EV-target cells. In the bone marrow niche, EVs may play an important role in the orchestration of hematopoiesis by bone marrow stromal cells. We hypothesize that mesenchymal stromal cells (MSCs)-derived EVs can promote the ex vivo expansion of both lineage restricted HPCs and multipotent HSCs.

**Methods**: UCB-derived CD34+ HSPCs were ex vivo cultured for 10 days in serum-free medium supplemented with cytokines (SCF and Flt-3) with or without EVs derived from foetal or adult MSCs. EVs were isolated from MSCs conditioned medium by sequential centrifugation steps at low (300g–2000 g) and high speed (20,000—1,00,000 g). We assessed the capacity of the HSPC to proliferate, to generate colony forming units (CFU) and to differentiate towards the various haematopoietic lineages based on the expression of surface markers.

**Results**: We found that foetal MSCs-derived EVs improve the ex vivo expansion of UCB-derived CD34+ HSPCs compared to cytokines only. Foetal MSCs-derived EVs and to a lesser extent adult MSCs-derived EVs enhance the proliferation of total nucleated cells, their CD34+ subset and their immature subset (CD34+CD90+CD38-CD45RA-), while retaining their CFU capacity. Based on surface maker expression, no skewing towards other lineages was observed. Preliminary ImageStream analysis indicates the uptake of PKH26-labelled EVs by a subset of CD34+ cells. RNAseq and proteomic analysis of MSC-derived EVs will allow to identify candidate miRNA and proteins present in EVs that contribute to mRNA regulation in EV-targeted HSPC, respectively, and whether they have known effects in proliferation, self-renewal and differentiation.

**Summary/conclusion**: Adult MSC-derived EVs, but in particular foetal MSC-derived EVs, show a potential use in development of a novel clinically relevant ex vivo expansion strategy for UCB-derived HSPCs.

PT06.10

Non-coding landscape of extracellular vesicles RNA from stem cells

Sippy Kaur^1^; Hanna Hiidenmaa^1^; Heidi Hongisto^2^; Riku Paananen^3^; Ahmed Abushahba^1^; Heli Skottman^2^; Riitta Seppänen-Kaijansinkko^1^; Bettina Mannerström ^1^

^1^Department of Oral and Maxillofacial Diseases, University of Helsinki and Helsinki University Hospital, Helsinki, Finland; ^2^BioMediTech, Faculty of Medicine and Life Sciences, University of Tampere, Tampere, Finland; ^3^University of Helsinki, Helsinki, Finland

**Background**: Extracellular vesicles (EVs) have been reported to be involved in stem cell maintenance, self-renewal and differentiation. Due to their bioactive cargoes influencing cell fate and function, interest in EVs in regenerative medicine has rapidly increased. Specifically, EV-derived miRNAs mimic the functions of the parent stem cells, regulating the maintenance and differentiation of stem cells, controlling the intercellular regulation of gene expression, which eventually affect the cell fate. The goal of this study was to analyse the EV-derived miRNAs and other non-coding RNAs released by adipose tissue stromal/stem cells (AT-MSCs) and pluripotent stem cells (PSCs) and to explore their biological relevance and their clinical potential.

**Methods**: Human PSC cells were cultured in serum-free medium and characterized for expression of pluripotency markers and spontaneous differentiation; AT-MSCs were cultured in EV-depleted FBS and characterized for MSC immunophenotype and multipotency. EV-miRNA sequencing was performed by Exiqon. Data analysis was performed using the edgeR package.

**Results**: The EV-miRNA sequencing showed that the profile of miRNA expression in PSC follows the profile reported for cell-derived miRNA; further, the miRNAs were found to originate from specific miRNA clusters (miR-17-92 miR-302, miR-371/372/373, CM19 microRNA cluster). For the AT-MSCs, the highly expressed miRNAs were found to be associated with osteogenesis and chondrogenesis (miR-10a, miR-100, miR-125/let-7cluster, miR-195, miR-199, miR-615). Additionally, abundant small nucleolar and nuclear RNA (SNORA, -D and RNU1) were detected in PSCs whereas Y- and tRNA were found in AT-MSCs.

**Summary/conclusion**: Identification of EV-miRNA and non-coding RNA signatures released by these stem cells will provide clues towards understanding the role of these EV-ncRNAs in intracellular communications, their clinical potential as well as their roles in maintaining the stem cell niche.

**Funding**: University of Helsinki and Helsinki University Hospital project funding.

PT06.11

Procardiomyogenic and proangiogenic properties of extracellular vesicles derived from genetically modified human induced pluripotent stem cells

Katarzyna Kmiotek-Wasylewska ^1^; Sylwia Bobis-Wozowicz^1^; Elzbieta Karnas^2^; Zbigniew Madeja^1^; Ewa K. Zuba-Surma^1^

^1^Department of Cell Biology; Faculty of Biochemistry, Biophysics and Biotechnology; Jagiellonian University; Krakow; Poland, Kraków, Poland; ^2^Laboratory of Stem Cell Biotechnology; Malopolska Centre of Biotechnology; Jagiellonian University; Krakow; Poland, Krakow, Poland

**Background**: Extracellular vesicles (EVs) are population of small (100–1000 nm) circular membrane vesicles secreted by most cell types. It has been recently reported that EVs may carry bioactive cargo including proteins, microRNAs and mRNAs. They also play a crucial role in cell-to-cell communication in both physiological and pathological conditions.

**Methods**: The aim of this study was to verify if treatment with EVs derived from hiPS cells overexpressing procardiomyogenic miR1 and miR199a as well as proangiogenic miR126 might have impact on various properties of human cardiac cells (CCs) and cardiac endothelial cells (CECs), respectively, including proliferation, migration, metabolic activity, differentiation and survival. EVs derived from wild type (WT) and copGFP overexpressing hiPS were used as a control. EVs were isolated from conditioned hiPS culture media using differential centrifugation followed by ultracentifugation. NHCF-V cells (Lonza) and HCAEC cells (Lonza) were used as a model of target CCs and CECs models, respectively. In each experimental set-up, cells were treated with 20 ng of EVs per 1000 cells.

**Results**: Our data indicate that hiPS-EVs may protect both types of cells from apoptosis and inhibit the progress of this process. They also had impact on NHCF-V cells proliferation, metabolic activity, migration and differentiation towards cardiomyocytes. Extracellular vesicles from hiPS cells had also impact on HCAEC cells capability for capillaries, their migration and metabolic activity.

**Summary/conclusion**: These results may suggest positive impact of EVs from hiPS cells overexpressing miR1, miR199a and miR126 in myocardium after ischemia, which needs to be tested in further experiments *in vivo*.

**Funding**: This study is funded by National Science Centre Poland (NCN) grants: SONATA BIS-3 (UMO-2013/10/E/NZ3/007500) to EZS and PRELUDIUM-11 (UMO-2016/21/N/NZ3/00363) to KKW.

Faculty of Biochemistry, Biophysics and Biotechnology of Jagiellonian University is a partner of the Leading National Research Center (KNOW) supported by the Ministry of Science and Higher Education

PT07: EV-inspired Therapeutics, Vaccines, and Clinical Trials Chairs: Shilpa Buch; Pia SiljanderLocation: Exhibit Hall 17:15–18:30

PT07.01

Extrusion of mesenchymal stromal cells produces EV-like vesicles that attenuate allergic airway inflammation

Elga Bandeira ^1^; Su Chul Jang^2^; Kyong-Su Park^1^; Kristina Johansson^1^; Cecilia Lässer^3^; Madeleine Rådinger^1^; Jan Lötvall^3^

^1^University of Gothenburg, Gothenburg, Sweden; ^2^Krefting Research Centre, Institute of Medicine, University of Gothenburg, Boston, USA; ^3^Krefting Research Centre, Institute of Medicine, University of Gothenburg, Gothenburg, Sweden

**Background**: Asthma is associated with airflow obstruction and hyper-responsiveness that arises from airway inflammation and remodelling. Cell therapy with mesenchymal stromal cells (MSC) has been shown to attenuate airway inflammation in asthma models. Recently, similar effects have been observed using extracellular vesicles (EVs) released by these cells. Nano-sized vesicles can also be artificially generated from MSC by extrusion, and we call them exosome-mimetic nanovesicles (NVs). In this study, we evaluated the effects of MSC-derived EVs and NVs in a murine model of allergic airway inflammation.

**Methods**: EVs were obtained through sequential centrifugation of media conditioned by human bone marrow MSC for 24 h. NVs were produced through serial extrusion of MSCs. Both vesicle types underwent density gradient purification and were quantified through nanoparticle tracking analysis. C57Bl/6 mice were sensitized to ovalbumin (OVA), randomly divided into OVA (intranasally exposed to 100 µg OVA on 5 consecutive days) and control (exposed to PBS) groups. The mice were further randomized into groups that received 2E09 EVs or NVs, following the first OVA/PBS exposure.

**Results**: Local administration of both EVs and NVs reduced the cellularity and number of eosinophils in bronchoalveolar lavage fluid (BALF) of OVA-exposed animals. In addition, NVs caused a decrease in the number of inflammatory cells within the lung tissue, which was associated with lower levels of CCL24 in BALF and lung tissue. The effectivity of NVs was similar when administered intraperitoneally or locally to the airways. Changing the administration route, nevertheless, led to remarkable differences in their biodistribution and to distinct attenuation especially of IL-13 and CCL24.

**Summary/conclusion**: Our results indicate that EVs and NVs derived from MSC have similar effects in a murine model of airway allergy. Furthermore, artificially generated vesicles can be effective upon different delivery routes, which, however, results in different immunomodulatory effects. Because of the higher yield of vesicles obtained by the extrusion process and the technical advantages it presents, we suggest that NVs can be an alternative to EVs in MSC-based therapies.

**Funding**: The Swedish Heart-Lung Foundation, Sahlgrenska University Hospital, Herman Krefting Foundation Against Asthma/Allergy, CODIAK Biosciences.

PT07.02

Specific targeting of challenging membrane proteins on exosomes and their multiple uses

Robert Z. Mamoun ^1^; Christian Leveque^2^; Oussama El Far^2^

^1^Ciloa SAS, Montpellier Cedex 5, France; ^2^Inserm, Marseille, France

**Background**: Membrane structures expressing fully native and mature transmembrane proteins are very useful tools to address several biological questions such as ligand/receptor binding but also for drug screening as well as for producing therapeutic antibodies and vaccines. Exosomes are native secreted membrane nanovesicles on which membrane protein topology is identical to the plasma membrane one.

**Methods**: We present our original approach to specifically address any types of membrane proteins to exosomal membranes. By merging a patented pilot peptide to the cytosolic domain of a chosen membrane protein, Ciloa technology allows the secretion by cells of exosomes harbouring this protein. We used such recombinant exosomes harbouring receptors to study ligand–receptor interaction and to develop highly efficient immunogens.

**Results**: The system allows the expression on exosomes of (i) fully native membrane proteins, (ii) more than one defined protein at the surface of the same exosome and (iii) homo- or hetero-oligomeric receptors and/or ion channels.

Our results demonstrate that these proteins on exosomes are fully functional for their specific ligand binding. In addition, viral envelope proteins presented by exosomes trigger strong immune response. The results reveal that these recombinant exosomes are highly efficient antigen presentators allow development of virus-free and adjuvant-free candidate vaccines.

**Summary/conclusion**: Our recombinant exosomes allow o immunization of animals against proteins known as “poor immunogens”. Such exsosomes are highly efficient antigen presentators allowing development of virus-free and adjuvant-free candidate vaccines.

**Funding**: Academic and private.

PT07.03

Discovery of an inhibitor for EV secretion in cancer cells using a small-molecule library approach

Yusuke Yoshioka ^1^; Akira Yokoi^2^; Takahiro Ochiya^1^

^1^Division of Molecular and Cellular Medicine, National Cancer Center Research Institute, Chuo-ku, Japan; ^2^National Cancer Center Research Institute, Chuo-ku, Japan

**Background**: Cancer cells release a wide variety of cancer cell-derived extracellular vesicles (EVs) that influence the behaviour of cells in the primary tumour microenvironment and at metastatic sites, resulting in the promotion of the initial steps for pre-metastatic niche formation. Therefore, inhibition of EV secretion from cancer cells can serve as a novel therapeutic tool to inhibit cancer metastasis. This study focused on the screening of small-molecule inhibitors for EV secretion in cancer cells.

**Methods**: We used an original screening system based on ExoScreen assay for monitoring CD9 positive EV secretion (Yoshioka Y et al., Nat Commun, 2014). In this assay system, EVs are captured by two types of antibodies, which are detected by photosensitizer beads. One is a biotinylated antibody and the other is an antibody conjugated to AlphaLISA acceptor beads. To observe the influence of small molecules on cell growth, a proliferation assay was undertaken using IncuCyte. The EV secretion rate of cells was normalized to cell growth rate. Using this screening system and a chemical compound library containing 1280 small molecules, inhibitors for EV secretion were identified in the ovarian cancer cell line ES-2. The particle number of EVs was determined using a NanoSight.

**Results**: Based on the first screening result, 76 small molecules were selected as putative inhibitors for EV secretion. These small molecules were further validated by ExoScreen. As a result of the validation, 9 small molecules were found to inhibit EV secretion in ovarian cancer cells. These 9 molecules did not affect ES-2 cell proliferation compared to untreated cells.

**Summary/conclusion**: Here, we identify inhibitors for EV secretion in ovarian cancer cells. Based on these results, we are now investigating whether these nine molecules have an effect on the EV secretion in normal cells. These results pave the way towards identifying new therapeutic targets for preventing metastatic spread.

**Funding**: This work was supported by JSPS KAKENHI Grant Number JP16K07189 and Project for Cancer Research and TherapeuticEvolution (P-CREATE) from the Japan Agency for Medical Researchand Development (AMED).

PT07.04

Microvesicles derived from gene-modified “mesenkillers”: isolation, characterization and anti-cancer potential

Filippo Rossignoli ^1^; Rita Leporati^2^; Giulia Rovesti^1^; Giulia Grisendi^3^; Carlotta Spano^3^; Massimo Dominici^4^

^1^University-Hospital of Modena and Reggio Emilia, Modena, Italy; ^2^University-Hospital of Modena and Reggio Emilia, Modena, Italy, Carpi, Italy; ^3^Rigenerand srl, Medolla, Modena, Reggio Emilia, Italy; ^4^University-Hospital of Modena and Reggio Emilia, Modena, Italy, Ferrara, Italy

**Background**: Mesenchymal stromal/stem cells (MSC) are a population of multipotent progenitor cells retaining proliferative potential able to differentiate into a variety of cell types. Their possible applications have been extensively investigated during the last 50 years, and their tumour tropism together with the possibility of genetic manipulation makes them a promising vector to deliver anti-cancer agents to tumour sites. In particular, gene-engineered MSC expressing TNF-related apoptosis-inducing ligand (TRAIL) death ligand demonstrated a significant tumour killing effect in several cancer models, such as pancreatic cancer and sarcomas. In recent years, several studies showed that secreted bioactive factors can play a pivotal role in the therapeutic action of MSC and extracellular vesicles (EV) could mediate some biological functions conventionally attributed to MSC. While this has been progressively established in regenerative medicine, little is known about the possible use of MSC-derived EV in anti-cancer strategies.

**Methods**: By an improved differential centrifugation-based protocol, we were able to isolate microvesicles (MV) from MSC expressing TRAIL variants. Released MVs were analysed by flow cytometry, their TRAIL content was assessed by ELISA and their cancer cell-killing efficacy was demonstrated by *in vitro* assays.

**Results**: The introduced protocol was suitable for isolation of MSC-derived EV and the preliminary results indicated how MV derived from TRAIL-armed AD-MSC could carry TRAIL variants inducing a rapid and selective apoptosis of sarcoma (A673) and pancreatic cancer (BXPC3) cell lines.

**Summary/conclusion**: These data highlight the potential of MSC-EV as tools for cell-free anti-cancer therapy delivering a very potent pro-apoptotic agent as a novel therapeutic approach for cancer.

**Funding**: This study was also possible by an unrestricted grant from ASEOP, Modena, Italy.

PT07.05

Optimizing loading and expression of HChrR6 mRNA in extracellular vesicles (EVs) for side effect-free prodrug-mediated treatment of HER2+ve breast cancer

Alexis V. Forterre ^1^; Jing-Hung Wang^1^; Reka Haraszti^2^; Anastasia Khvorova^2^; AC Matin^1^

^1^Stanford University School of Medicine, Stanford, USA; ^2^University of Massachusetts Medical School, Worcester, USA

**Background**: Lack of specific targeting and insufficient genetic material delivery has hampered gene-directed enzyme prodrug (GDEPT) therapies. We have developed EXO-DEPT/CNOB regimen that specifically targets and completely arrests the growth orthotopic implanted HER2+ve tumours in mice. These EVs specifically deliver *HchrR* mRNA to tumours to generate the HChrR6 enzyme, which converts the prodrug CNOB into cytotoxic MCHB; MCHB can be quantified from its fluorescence. mRNA is superior to DNA for gene delivery, being directly translated upon delivery to the cytosol. To enhance the efficacy of this regimen, enhancement of mHChrR6 loading and expression were undertaken.

**Methods**: Use of plasmids employed previously for loading mRNA can potentially introduce harmful genetic material in the EVs. We have therefore shifted to using *in vitro* transcribed (IVT) HchrR mRNA to load HEK293FT cells. Cholesterol-Teg-oligos, complementary to the HchrR mRNA coding region, were tested to facilitate loading into the EVs. Functionality was assessed by measuring MCHB fluorescence after CNOB addition; MTT assay measured cell viability.

**Results**: Use of the IVT HchrR6 mRNA instead of the plasmid (XPort/HChrR6) improved the loading of mHChrR6, decreasing the number of EVs required to deliver one mRNA copy from 5000 to 20. BT474 cells receiving the mRNA from these EXO-DEPTs retained the ability to convert CNOB into MCHB for up to 4 days. Whether this is because of stability of the mRNA or the HChrR6 protein is under investigation. Use of Cholesterol-Teg oligos permitted loading of HChrR6 IVT mRNA in EVs without using transfection reagents; this likely occurred via the endosomal pathway. The latter were able to induce caspase3-mediated cell killing.

**Summary/conclusion**: We improved EXO-DEPT EV engineering by increasing their HchrR mRNA copy number without using plasmids and transfection reagents. Work is in progress to further enhance mRNA loading in the EXO-DEPTs using Cholesterol-Teg oligos complementary to the 3' and 5' mRNA regions. These measures can also stabilize mRNA expression in the recipient cells. Whether the zipcode sequence (believed to facilitate mRNA loading into EVs) we have so far used is essential, and whether stable expression of the mRNA can be enhanced by incorporation of the 3'UTR of Beta-globin mRNA are under investigation.

PT07.07

Extracellular vesicles as a drug delivery platform – post-production physico-chemical modification and *in vitro* internalization

Sarah Le Saux ^1^; Ellie Barlow Myers^1^; Josephine Lai Kee Him^2^; Patrick Bron^2^; Jean-Marie Devoisselle^1^; Philippe Legrand^1^; Joel Chopineau^1^; Marie Morille^1^

^1^Institut Charles Gerhardt de Montpellier (ICGM) - UMR 5253 CNRS-ENSCM-UM, MACS (Matériaux Avancés pour La Catalyse et La Santé) team, Montpellier, France; ^2^Centre de Biochimie Structurale (CBS) - CNRS UMR 5048 - UM - INSERM U 1054, Montpellier, France

**Background**: Despite the proof of concept of their efficiency as drug delivery systems (DDS) compared to synthetic nanoparticles, the rationale of using extracellular vesicles (EVs) still requires numerous improvements (yield of production, drug loading, pharmacokinetics). In this context, our team aims at overcoming these hurdles by using its pharmaceutical/physico-chemical skills to perform post-production modifications of EVs, in order to create a potent DDS.

**Methods**: We proved ablility to produce, isolate (differential ultracentrifugation) and characterize (dynamic light scattering, nanoparticle tracking analysis (NTA), protein dosage, western blot, proteomics, cryoTEM) EVs from murine MSC with yields coherent with their use in this project. Importantly, we developed a freeze-drying protocol for their long-term storage, with no impact on vesicle numbers, structure (cryoTEM) and content (proteomic). After labelling with a lypophilic dye, EVs were incubated with the parent cells or foreign cells (NIH3T3), in the presence of endocytosis inhibitors, and tracked by flow cytometry. All experiments were also performed on liposomal commercial standards (PC/Chol) as a comparison.

**Results**: EVs were 94 ± 11 nm (NTA, *n* = 9) with a production yield of 3.41 µg protein and 9.48.108 particles/10^6^ cells (*n* = 9). The western blot and proteomics analysis evidenced the presence of EV-specific markers such as TSG101, CD81 and ADAM10. The EVs were internalized to a greater extent than their liposomal counterparts in both target cells (*n* = 3). Our preliminary data suggest that they could follow different endocytic routes. Among the processes evaluated for drug loading, EVs were extruded through 50 nm membranes without damage. We are currently investigating whether the performed modifications impact their internalization rate and pathway.

**Summary/conclusion**: Our team has been able to reproducibly isolate, characterize and label mMSC-derived EVs. The EVs show increased internalization *in vitro* compared to liposomes currently used as DDS, whatever the target cell type, and EVs may follow a different endocytic route than liposomes. We propose here to present our latest results regarding the rationale of using EVs as vectors for drug delivery.

**Funding**: The PhD project is funded by MESR (Ministère de l’Enseignement Supérieur et de la Recherche) funding.

PT07.08

A systematic review and meta-analysis of parameters affecting the therapeutic potential of mesenchymal stem cell-derived extracellular vesicles in pre-clinical studies

Faezeh Shekari ^1^; Sara Assar Kashani^2^; Abdo Reza Nazari^2^; Ensiyeh Haji Zadeh^2^; Hossein Baharvand^1^

^1^Department of Stem Cells and Developmental Biology, Cell Science Research Center, Royan Institute for Stem Cell Biology and Technology, Tehran, Iran, Tehran, Iran; ^2^Royan institute, Tehran, Iran

**Background**: Mesenchymal stem cells (MSC) therapy is one of the most commonly employed cellular therapy in human clinical trials. Since MSCs secrete extracellular vesicles (EVs) to mediate in regeneration, EVs are undergoing extensive evaluation as a replacement or adjutant to cells in cellular therapy in pre-clinical studies. To date, there has been no meta-analysis of studies using MSC-EV therapy in animal studies. 

**Methods**: By searching systematically in PubMed and Scopus databases, more than 1000 reports were identified. After screening for eligibility, a total of approximately 100 studies are found to report MSC-EV therapy in animal disease models.

**Results**: All the found pre-clinical studies reporting the therapeutic potential of MSC-derived EVs underwent comprehensive review, quality assessment and data extraction. Most of these studies employed animal models for kidney, heart, skin and lung disease as well as cancer. Although culture conditions of the EV-producing cells have overlapping characteristics, we discussed many different technical aspects, the methods of EV isolation and downstream characterizations that can make a significant difference in the overall results of animal studies. In addition, we explored the effect of clinically relevant *in vivo* animal administration parameters on functional outcome to better design future clinical studies utilizing MSC-EVs.

**Summary/conclusion**: Our study provides new insight regarding parameters affecting the therapeutic potential of MSC-derived EVs in pre-clinical studies. Although there is significant improvement in animal disease model after MSC-derived EVs therapy, most of reports are still far from transparency required for designing a clinical trial.

**Funding**: This study was funded by grants provided from Royan Institute and Iranian Proteomics Society.

PT07.09

Nanoparticles, liposomes and exosomes as microRNA delivery systems for neurodegenerative disease: remyelination inductors in multiple sclerosis

Iñaki Osorio-Querejeta ^1^; Ana Ayerdi^2^; Susana Carregal-Romero^3^; Leslie Nash^4^; Ainhoa Alberro^1^; Leire Iparraguirre^1^; Imer Mäger^5^; Matthew JA Wood^5^; Pedro Ramos-Cabrer^6^; Maider Muñoz-Culla^1^; David Otaegui^1^

^1^Multiple Sclerosis Unit, Biodonostia Health Institute, Paseo Doctor Beguiristain S/N, 20014, San Sebastián, Spain., Donostia-san Sebastián, Spain; ^2^TECNALIA. División Salud. Área Biomateriales.Parque Tecnológico de San Sebastián Mikeletegi Pasealekua, 2 E-20009 Donostia-San Sebastián - Gipuzkoa (Spain), Donostia-San Sebastián, Spain; ^3^CIC biomaGUNE, Paseo de Miramón 182, Donostia-San Sebastián, Spain; ^4^Regenerative Medicine Program, Ottawa Hospital Research Institute, Ottawa, Canada; ^5^Department of Physiology, Anatomy and Genetics, University of Oxford, Oxford, United Kingdom., Oxford, UK; ^6^Magnetic Resonance Imaging. Molecular Imaging Unit, CIC BiomaGUNE, Paseo de Miramón 182, Donostia-San Sebastian, Spain

**Background**: Multiple sclerosis is an autoimmune and demyelinating disease of the central nervous system. Commercially available treatments are focused in the attenuation of the immune response, but up to now, none of them are focused in improving remyelination. Nevertheless, alternative strategies are being studied. MicroRNAs have been shown to work as remyelination inductors. More concretely, miR-219 has been shown to be involved in the differentiation of oligodendrocyte precursor cells (OPCs) and therefore remyelination.

In this work, nanoparticles (NPs), liposomes (LPs) and exosomes (EXs) were compared as miR-219 delivery systems in an OPC culture where the ability of miR-219 to induce OPC differentiation was also analysed.

**Methods**: OPCs were obtained from P-1 mice brains and cultured in laminin-coated P24 plates prior to the treatment.

PLGA nanoparticles and DSPC liposomes were loaded with miRIDIAN microRNA mmu-miR-219a-5p. Exosomes were obtained from HEK-293T cells infected with pLKO-mmu-miR219a-5p plasmid. All the respective controls were done.

For uptake experiments, NPs were loaded with coumarin and LP with DiOC18. In addition, miRIDIAN microRNA mimic transfection control with Dy547 was used for NP and LP. Exosomes were labelled with Celltracker CM-Dil.

In order to quantify the differentiation degree of OPCs and the levels of miR-219 in the vehicles, qPCR was carried out.

**Results**: Preliminary results showed higher levels of miR-219 and a better uptake for LPs and NPs in comparison with EXs. However, LPs and NPs were not able to induce OPCs differentiation whereas EXs did.

**Summary/conclusion**: EX, which showed the lowest miR-219 and uptake levels, were able to induce OPCs differentiation. These results might indicate that EX are extremely efficient as microRNA delivery systems. In addition, we hypothesized that the additional cargo that EXs may carry could favour the shown effects.

**Funding**: Basque Government PhD students program supports IOQ, AA, LI.

EMBO STF # 7109

DTS15/00069

FIS 14/00939

PT07.10

Chemotherapeutic-loaded extracellular nano-vesicles produced via sulfhydryl blocking

Dominique Ingato; Julius A. Edson; Michael Zakharian; Young Jik Kwon

University of California, Irvine, Irvine, USA

**Background**: Extracellular vesicles (EVs) have great potential as therapeutic carriers as they are expected to exhibit high levels of biocompatibility and to allow for targeted delivery to specific tissues. Exosomes, naturally occurring EVs ranging in size from 30–100 nm, have been widely studied for transport of biomacromolecules and small molecule drugs. However, limited scalability of production associated with naturally occurring EVs has been a key challenge in the field. Here, we demonstrate use of sulfhydryl blocking for increasing cellular production of nano-sized vesicles by more than 10-fold. We show the utility of using nano-vesicles produced via sulfhydryl blocking for delivery of chemotherapeutic doxorubicin *in vivo*.

**Methods**: Nano-vesicles were produced from murine lymphoma (EL4) cells by culturing the cells in DPBS with paraformaldehyde for sulfhydryl-blocking. Nano-vesicles were isolated by a series of centrifugation and centrifugal filtration steps and characterized by transmission electron microscopy (TEM) and dynamic light scattering (DLS). Nano-vesicles were loaded with doxorubicin by incubation with the drug followed by centrifugal filtration to remove free drug.

**Results**: Nano-vesicles produced from murine lymphoma cells were similar in size to exosomes from the same cell line at approximately 100 nm in diameter. Treatment of murine lymphoma with drug-loaded nano-vesicles resulted in improved survival outcomes and diminished tumour growth in addition to more favourable biodistribution.

**Summary/conclusion**: We have shown that this improved method of nano-sized EV production both enhances yield and has relevance to the field of chemotherapeutic delivery. However, nano-vesicles have a range of potential applications beyond the scope of this study and the method of production we describe could be used to generate nano-vesicles for a variety of other applications.

**Funding**: This research did not receive any specific grants from funding agencies in the public, commercial, or not-for-profit sectors. The National Science Foundation Graduate Research Fellowship supported Dominique Ingato and Julius A. Edson.

PT07.11

Milk exosomes – intranasal versus oral delivery of exosomes and exosomal-curcumin

Farrukh Aqil ^1^; Radha Munagala^1^; Jeyaprakash Jeyabalan^2^; Ashish kumar Agrawal^3^; Ramesh C. Gupta^4^

^1^Department of Medicine and JG Brown Cancer Center, University of Louisville, Louisville, USA; ^2^JG Brown Cancer Center, University of Louisville, Louisville, USA; ^3^James Graham Brown Cancer Center, University of Louisville, Louisville, USA; ^4^Department of Pharmacology and Toxicology and JG Brown Cancer Center, University of Louisville, Louisvilleq, USA

**Background**: Curcumin (CUR), a plant polyphenolic, represents the most widely investigated compound in pre-clinical and clinical studies. However, this agent suffers from poor oral bioavailability due to very limited aqueous solubility and rapid hepatic metabolism. Numerous nano-curcumin formulations have been developed in the past decade to overcome these issues; however, none have reached clinical translatability due to concerns related to scalability, costs and/or toxicity related to the nanomaterials used. Bovine milk exosomes discovered in this laboratory seem to overcome these limitations. Here, we report that route of delivery can profoundly affect tissue accumulation of exosomes and exosomal-curcumin (ExoCUR) using murine models.

**Methods**: Exosomes were isolated from bovine milk and loaded with CUR using procedures described previously. To determine the route of delivery-dependent biodistribution of exosomes, the exosomes were labelled with DiR, administered to nude mice intranasally and by oral gavage, and different organs were imaged ex vivo. To measure CUR distribution, ExoCUR and CUR were administered daily for 7 days to wild-type mice intranasally and by oral gavage (2.4 mg/kg, bwt) and lung CUR levels were measured by UPLC. Finally, to assess efficacy of exosomal delivery, we determined growth inhibition of cervical tumour xenograft in nude mice by oral delivery of ExoCUR and CUR (20 mg/kg on alternate days for 7 weeks).

**Results**: Oral delivery of DiR-labelled exosomes showed similar tissue distribution; however, intranasal delivery led to predominant (50%) accumulation of the exosomes in the lung. Intranasal delivery of ExoCUR also showed substantially (>20-fold) higher lung CUR level compared with oral route. In the tumour model, CUR delivered orally failed to achieve any inhibition of the cervical tumour xenograft while ExoCUR showed significant (>60%) tumour inhibition.

**Summary/conclusion**: Our data suggest that route of administration can significantly influence the biodistribution of exosomes as well as ExoCUR. Furthermore, exosomal formulation of poorly bioavailable compounds such as CUR can achieve significant biological effects presumably by enhancing its bioavailability and sustained release.

**Funding**: From Duggan Endowment and Helmsley Trust Fund.

PT07.12

Generation of engineered exosomes for targeted delivery of therapeutic microRNAs in CAP cells

Nikola Strempel ^1^; Nikolas Zeh^2^; Sabine Hertel^1^; Benjamin Weis^2^; Silke Wissing^1^; Nicole Faust^1^; Kerstin Otte^2^

^1^CEVEC Pharmaceuticals GmbH, Koeln, Germany; ^2^University of Applied Sciences Biberach, Biberach, Germany

**Background**: miRNAs are small non-coding RNA molecules which mediate biological function due to their key role in gene regulation. Various studies indicate the presence of miRNAs in exosomes. Since deregulation of miRNAs is a common feature in cancer, they could serve as targets for therapeutic intervention. However, various biological barriers including *in vivo* nuclease degradation and miRNA-induced immune response drastically hinder their bioavailability. Hence, targeted delivery of RNA therapeutics by exosomes may display a promising strategy.

The CAP cell line is a fully characterized human suspension cell line which has been developed for industrial production of biotherapeutics including gene therapy vectors and difficult-to-express proteins. CAP cells grow to high cell densities of >2 × 10^7^/ml in serum-free medium in a wide range of bioreactors, allowing for an easy scale-up of production processes. However, there are currently no reports as to whether they may as well produce exosomes suitable for targeted delivery of therapeutics.

In the present study, we evaluated the concept to enable targeted delivery of therapeutic miRNAs using exosomes derived from CAP cells as vehicles.

**Methods**: In order to evaluate CAP cells as production hosts for exosomes, exosomal preparations were examined for vesicle identity, size, morphology and concentration using dynamic light scattering, flow cytometry, western blotting and electron microscopy.

**Results**: The stable expression of a GFP fusion protein enabled the tracing of produced exosomes using flow cytometry. To functionally analsze isolated exosomes regarding their potential to deliver small therapeutic agents, these fluorescently labelled exosomes were further engineered to overexpress therapeutic, pro-apoptotic and control miRNAs by stable genome integration into CAP cells. qPCR analysis confirmed the enrichment of specific miRNAs in exosomes derived from those stable cell pools. The cells have been further engineered to overexpress modified surface receptors to facilitate targeted uptake by tumour cells.

**Summary/conclusion**: The current study reveals human CAP cells to be a highly suitable host for the serum-free production of exosomes and pursues the therapeutic concept of using CAP-derived exosomes as delivery vehicle for miRNAs.

PT07.13

Characterization of bovine milk-derived extracellular vesicles as delivery system for therapeutics

Akiko Kogure ^1^; Masaharu Somiya^2^; Yusuke Yoshioka^1^; Takahiro Ochiya^1^

^1^Division of Molecular and Cellular Medicine, National Cancer Center Research Institute, Chu-ou, Japan; ^2^The Institue of Scientific and Industrial Reseach, Osaka University, Ibaraki-shi, Japan

**Background**: Extracellular vesicles (EVs) are nano-sized vesicles that are related to cell-cell communication via the functionally active cargo. As EVs naturally carry proteins, lipids, DNA and various forms of RNA, they are explored as a means of drug discovery. Several reports showed that bovine milk is ideal raw material for the drug delivery application of EVs, since bovine milk contains numerous EVs and are widely available. However, the character including toxicity of bovine milk-derived EVs (mEVs) are not fully evaluated. In this study, we determined the bioavailability of mEVs upon systemic administration into mice. In addition, we investigated the potential of mEVs for use as a biologically active drug delivery vehicle in treating cancer.

**Methods**: The cytotoxicity of mEVs was evaluated using the WST-8 in HEK293 cells and mouse macrophage cell line Raw264.7 cells. After the several intravenous administrations of mEVs into mice, toxicity, immunogenicity and anaphylactic reaction were examined. The cellular uptake was observed using a confocal laser scanning microscope with PKH-labelled mannose-conjugated mEVs.

**Results**: In the animal experiments, we did not observe any systemic toxicity upon intravenous administration. Some types of cytokines in blood were slightly increased; however, anaphylactic reaction was not observed, suggesting that mEVs can be used as safe drug delivery system. Moreover, mEVs were efficiently taken up by Raw264.7 cells *in vitro* without affecting cell viability. The cellular uptake rate of mEVs was markedly increased by mannose conjugate.

**Summary/conclusion**: These results suggested that mEVs could be used for the delivery of therapeutic molecules which target macrophage.

**Funding**: This study was supported by Grant in Aid for the Japan Agency for Medical Research and Development (A-MED) through the Basic Science and Platform Technology Program for Innovative Biological Medicine (JP17am0301013).

PT07.14

Endogenous and exogenous loading of extracellular vesicles for therapeutic delivery of the renin-angiotensin system peptide angiotensin-(1–7) in cardiomyocyte hypertrophy

Laura S. Downie; Lorraine M. Work; Stuart A. Nicklin

University of Glasgow, Glasgow, United Kingdom

**Background**: The renin-angiotensin system (RAS) peptide angiotensin II (AngII) mediates cardiac hypertrophy. The counter-regulatory RAS axis peptide Angiotensin 1–7 [Ang-(1–7)] inhibits cardiomyocyte hypertrophy. Extracellular vesicles (EVs) were purified from cardiomyocytes +/- treatment with AngII or Ang-(1–7) to assess cardiomyocyte hypertrophy. EVs were loaded with Ang-(1–7) via electroporation for therapeutic delivery.

**Methods**: H9c2 cardiomyocytes were untreated (control) or treated with AngII or Ang-(1–7). EVs were isolated from conditioned media by differential ultracentrifugation, characterized by BCA, western immunoblot, Nanosight and TEM and incubated with recipient cardiomyocytes. Next, cells were stained with F-Phalloidin actin and area measured. Gene expression of hypertrophy marker brain natriuretic peptide (BNP) was assessed by qRT-PCR. Control EVs were electroporated in the presence of Ang-(1–7) and levels determined by ELISA.

**Results**: H9c2 cardiomyocyte-derived EV size was 101.0 ± 2.4 nm and EV markers CD63 and TSG-101 were consistently detected. EVs from AngII-treated cardiomyocytes significantly increased recipient cardiomyocyte area compared to control EVs [control: 3291.1 ± 90.1 µm^2^ vs. AngII :5252.3 ± 125.4 µm^2^; *p* < 0.001] and significantly increased BNP expression [*p* < 0.017]. EVs isolated from Ang-(1–7)-treated H9c2 cardiomyocytes significantly reduced AngII-induced hypertrophy in recipient cardiomyocytes [AngII + control EVs: 5566.3 ± 139.0 µm^2^ vs. AngII+Ang-(1–7) EVs: 4212.7 ± 132.1 µm^2^; *p* < 0.01]. Electroporation-loaded EVs with Ang-(1–7) [naïve EVs: 0.0 pg/ml vs. Ang-(1–7) EVs: 342.3 ± 9.1 pg/ml; *p* < 0.001]. Ang-(1–7)-loaded EVs significantly reduced AngII-induced hypertrophy in recipient cardiomyocytes [naive EVs: 4641.2 ± 35.3 µm^2^ vs. Ang-(1–7) EVs: 2758.4 ± 20.1 µm^2^; *p* < 0.001].

**Summary/conclusion**: EVs isolated from AngII-treated H9c2 cardiomyocytes stimulate recipient cardiomyocyte hypertrophy. EVs isolated from Ang-(1–7)-treated cardiomyocytes inhibit hypertrophy. Furthermore, EVs exogenously loaded with Ang-(1–7) inhibit cardiomyocyte hypertrophy. These findings have implications for understanding the role of the RAS and EV function in cardiomyocytes.

**Funding**: Biotechnology and Biological Sciences Research Council (BBSRC, RCUK).

PT08: Cardiovascular Insult Chairs: J. Brian Byrd; Navneet Dogra Location: Exhibit Hall 17:15–18:30

PT08.01

EVs from Wistar Kyoto and spontaneously hypertensive rats have differential vasodilatory effects on resistance arteries

Miranda Good^1^; Luca Musante^2^; Robert M. Carey^3^; Nancy Howell^3^; Thu H H. Le^4^; Brant E. Isakson^1^; Uta Erdbrügger ^4^

^1^University of Virginia Health System, Robert M Berne Cardiovascular Research Center, Charlottesville, USA; ^2^University of Virginia Health System, Department of Medicine, Division of Nephrology, Charlottesville, USA; ^3^University of Virginia Health System, Department of Medicine, Divison of Endocrinology and Metabolism, Charlottesville, USA; ^4^Department of Medicine, Division of Nephrology, University of Virginia, Charlottesville, VA, United State, Charlottesville, USA

**Background**: Extracellular vesicles (EVs) have been described as novel bio-markers and bio-activators in vascular dysfunction in HTN. However, the exact mechanisms how EVs affect vascular function is not known. To examine the functional effects of EVs on acetylcholine (ACh)-mediated vasodilation, we freshly isolated 3^rd^/4^th^-order mesenteric arteries and circulating EVs from 12-week-old normotensive control Wistar-Kyoto (WKY) and spontaneously hypertensive (SHR) rats.

**Methods**: Circulating EVs were collected from WKY and SHR rats from citrated blood through a carotid catheter withdrawal. Differential centrifugation was applied to generate an EV pellet. EV size and concentration were determined by tunable resistive pulse sensing. Arteries were cannulated on a pressure myograph, pressurized to 80 mmHg. EVs (~6 × 10^7^ EV/ml) were added to the vessel lumen and circulating bath solutions and equilibrated for 10 min. Inner diameter was measured as cumulative concentrations of ACh were applied to the bath following a 10 µM phenylephrine (PE) pre-constriction.

**Results**: Mean EV size was similar for WKY (196 nm) and SHR (213 nm), as was the particle concentration. No significant difference in ACh vasodilation was observed in control arteries from WKY and SHR rats (no EVs), although SHR arteries were more vasoconstrictive to PE. Interestingly, WKY arteries treated with SHR EVs demonstrated enhanced vasodilation compared to arteries treated with WKY EVs. This difference was not present in arteries from SHR rats treated with WKY or SHR EVs. WKY arteries pretreated with 100 µM LNAME, a nitric oxide synthase inhibitor, had similar ACh-mediated vasodilation with both WKY and SHR EV treatment. The enhanced ACh-mediated vasodilation was lost when WKY arteries were treated with EVs from 6-week-old pre-hypertensive SHR or delipidated EVs (by lipid organic extraction) from 12-week-old hypertensive SHR.

**Summary/conclusion**: Together, these data suggest that upon development of HTN, SHR rats produce EVs that can enhance ACh-mediated vasodilation in normotensive arteries, but this effect is lost in arteries from hypertensive rats. Additionally, this effect requires intact vesicles and may be nitric oxide synthase-dependent. This data supports the functional role of EVs in vascular regulation in HTN.

**Funding**: National Lung, Heart and Blood Institute, USA.

PT08.02

High yield mechanically induced extracellular vesicles display as efficient regenerative effect as their parental mesenchymal stem cells or starvation-induced EVs *in vitro* and *in vivo* in a model of chronic heart failure

Iris Marangon ^1^; Max Piffoux^2^; Nathalie Mougenot^3^; Claire Wilhelm^2^; Florence Gazeau^2^; Onnik Agbulut^4^; Amanda K A silva^2^

^1^Université Sorbonne Paris Cité, Laboratoire Matière et Systèmes Complexes, CNRS UMR 7047 Université Paris Diderot, France, paris, France; ^2^Laboratoire Matière et Systèmes Complexes, Paris, France; ^3^Sorbonne Universités, Université Pierre et Marie Curie Paris 6, Plateforme PECMV, UMS28, Paris, France, paris, France; ^4^Sorbonne Universités, Université Pierre et Marie Curie Paris 6, Adaptation biologique et vieillissement, UMR8256, CNRS, France, paris, France

**Background**: On the road towards the use of extracellular vesicles (EVs) for regenerative medicine, technological hurdles remain unsolved: high-yield, high purity and cost-effective production of EVs. 

**Methods**: Pursuing the analogy with shear-stress induced EV release in blood, we are developing a mechanical stress EV triggering cell culture approach in scalable and GMP-compliant bioreactors for cost-effective and high yield EV production.

The third-generation set-up allows the production of up to 300,000 EVs per mesenchymal stem cell, a 100-fold increase compared to classical methods, i.e. physiological spontaneous release in depleted media (around 2000 EVs/cell), with a high purity ratio 1 × 10^10^ p/µg.

**Results**: We investigated *in vitro* the regenerative potential of high-yield mechanically induced MSC-EVs by demonstrating an equal or increased efficiency compared to classical EVs. The regenerative properties of mechanically induced MSC-EVs were confirmed *in vivo* in a murine model of chronic heart failure. Mice underwent surgery to induce a permanent myocardial infarction and were treated 1 month postinfarction by percutaneous EV injection of 3 × 10^11^ EVs (high and medium shear stress EVs, serum starvation EVs) or MSCs (1 × 10^6^). Heart functional parameters were analysed by ultrasound 2 months postinfarction. Interestingly, shear-induced EVs had the same effect compared to EVs produced by starvation or to parental cells, with an increase in the left ventricular ejection fraction of 10–20% compared to pretreatment values, whereas PBS injected controls lost 18%. Biomolecular analysis and histology are ongoing.

**Summary/conclusion**: We demonstrated an equal or superior regenerative effect of high-yield mechanically produced EVs compared to spontaneously released EVs or parental cells *in vitro* and *in vivo*. This unique technology for EV production combines decisive assets for clinical translation of EV-based regenerative medicine: a GMP-compliant set-up, high-density cell culture, high-yield release of EVs per cell and high-purity EVs.

PT08.03

Coronary artery bypass graft (CABG) surgery - Does the surgery procedure affect the EV status?

Christina Schlingschroeder ^1^; Sebastian Borosch^2^; Christian Stoppe^3^; Eva Miriam Buhl^4^; Christian Beckers^1^; Ruediger Autschbach^1^; Sandra Kraemer^1^

^1^Department of Thoracic and Cardiovascular Surgery, University Hospital RWTH Aachen, Aachen, Germany; ^2^Department of Thoracic and Cardiovascular Surgery, Uniklinik RWTH Aachen, Aachen, Germany; ^3^Department of Intensive Care and Intermediate Care, University Hospital, RWTH Aachen, Aachen, Germany; ^4^Electron Microscopic Facility, Medical Faculty, University Hospital RWTH, Aachen, Aachen, Germany

**Background**: Patients with high-grade stenosis of coronary arteries are frequently scheduled for coronary artery bypass graft (CABG) surgery, which can be performed either with (on pump) or without (off pump) the use of cardiopulmonary bypass (CPB). Recent studies indicated that the concentration of extracellular vesicles (EVs) increases in patients undergoing an on-pump CABG surgery and those EVs can be used as an early biomarker for myocardial ischemia. In this context, it has come into focus if using the CPB influences the post-operative EV status or if the increased vesicles depend only on the surgical trauma itself. We therefore analysed the EV composition, size and concentration in patients undergoing off pump CABG surgery.

**Methods**: EDTA-plasma from off pump CABG-patients at four time points (preoperative, immediately after surgery, 24 h and 48 h post-operative) was centrifuged at 2000 g for 10 min and additionally at 10,000 g for 20 min. The resulting supernatant was used to isolate EVs by size-exclusion chromatography (SEC). Particle size and concentration were analysed by nanoparticle tracking analysis (NTA), their composition was determined by western blot. Electron microscopy was performed to ensure successful EV isolation.

**Results**: EVs from plasma were successfully isolated by SEC. Electron microscopy images indicated the presence of EVs at all time points. The EV concentration decreased during the surgery, and again elevated 24 h post-surgery. Fourty eight hour after surgery, the EV concentration decreased again. Similar effects were observed by analysing the EV composition. Flotillin levels for example were strongly reduced immediately after surgery. The size of the vesicles decreased from baseline to 24 h post-surgery and increased again 48 h after surgery.

**Summary/Conclusion**: Performing CAGB surgery without use of CPB had a significant effect on EV concentration, size and cargo. In contrast to current literature, which demonstrated an increased vesicular concentration after on pump CABG surgery, our results showed that the CPB is an important stimulus of the surgery and influenced the EV status. Yet, it is still necessary to evaluate the clinical significance of the perioperative EV release in cardiac surgery patients.

PT08.04

microRNAs enriched in lymphocytic microparticles exhibit antiangiogenic effect

Chun Yang ^1^; Chenrongrong Cai^2^; Lijuan Wang^3^; Muqing Gu^3^; Carmen Gagnon^4^; Pierre Hardy^4^

^1^Research center of CHU sainte-justine, University of Montreal, Montreal, QC, Canada; ^2^Department of Pharmacology, University of Montreal, Montreal, QC, Canada; ^3^Department of Gynecological Endocrinology, Beijing Obstetrics and Gynecology Hospital, Capital Medical University, Bejing, China (People’s Republic); ^4^Departments of Pediatrics and Pharmacology, University of Montréal, Montreal, QC, Canada

**Background**: Excess angiogenesis or neovascularization plays a key role in the pathophysiology of many diseases such as cancer, diabetic blindness, exudative age-related macular degeneration, retinopathy of prematurity, rheumatoid arthritis, psoriasis, etc. We have previously demonstrated that microparticles derived for apoptotic lymphocytes (LMPs) possess strong anti-angiogenic effect. This study is designed to investigate the effects of microRNAs that enriched in LMPs.

**Methods**: We isolated the total RNAs from LMPs and performed the RNA sequence to identify the microRNAs components of anti-angiogenic LMPs. The specific inhibitory experiments were performed to verify the activity of miRNA-181a (miR-181a) in mediating the effect of LMPs on endothelial cell proliferation. The effect of miR-181a on human umbilical vein endothelial cells proliferation was assessed *in vitro*. The impact of miR-181a on angiogenesis was confirmed using *in vitro* angiogenesis assay. The gene expression of angiogenic factor VEGF and MARPK1 (a miR-181a direct target gene) assessed by real-time qPCR.

**Results**: RNA sequence revealed that miRNAs were selectively enriched in LMPs, among them the miR-181a is one of the most abundant components. Importantly, the inhibitor of miR-181a significantly abrogated the effect of LMPs on endothelial cell proliferation, but overexpression of miR-181a reduced endothelial cell growth in a dose-dependent manner. MiR-181a strongly inhibited the tube formation in *in vitro* angiogenesis assay. In addition, the expressions of MARPK1 and VEGF were found downregulated in the miR-181a overexpressed endothelial cells.

**Summary/Conclusion**: These data strongly suggested the development of miR-181a as a novel therapeutic strategy for the treatment of angiogenesis-related diseases.

**Funding**: This work was supported by an operating grant to P. Hardy from the Canadian Institutes of Health Research [362383].

PT08.05

Circulating extracellular vesicles released by blood flow restricted exercise have an altered miRNA profile and induce proliferation of skeletal muscle precursor cells

Jesper Just ^1^; Jean Farup^2^; Mette Sloth^1^; Yan Yan^3^; Jørgen Kjems^3^; Frank Vincenzo de Paoli^4^; Kristian Vissing^5^; Kim Ryun. Drasbek^1^

^1^Center of Functionally Integrative Neuroscience, Department of Clinical Medicine, Aarhus University, Århus C, Denmark; ^2^Department of Clinical Medicine - The Research Laboratory for Biochemical Pathology, Aarhus University, Aarhus, Denmark; ^3^Interdisciplinary Nanoscience Center (iNANO), Aarhus University, Aarhus, Denmark; ^4^Department of Biomedicine - Forskning og uddannelse, Vest, Aarhus University, Aarhus, Denmark; ^5^Section of Sport Science, Department of Public Health, Aarhus University, Aarhus, Denmark

**Background**: Both ischemic conditioning and high intensity training (HIT) have the potential of activating endogenous organ protection against prolonged damaging ischemia. Blood flow restricted exercise (BFRE) offers an attractive alternative low intensity training regime, as it combines ischemic conditioning and exercise, and thus, could give a more effective protection while improving overall fitness. In BFRE, external pressure that occludes venous outflow but maintain arterial inflow is applied to arms or legs during exercise. Interestingly, BFRE promotes similar effects on skeletal muscle when compared to HIT. Thus, patients with chronic conditions for whom it is not physically possible to perform HIT, low-intensity BFRE offers an alternative prophylactic training regime. This study investigates if blood-borne extracellular vesicles (EVs) and their microRNA (miRNA) content could be possible effectors of BFRE.

**Methods**: EVs were isolated from plasma of six healthy human subjects before and 1 h after completing BFRE. EVs were then characterized by NTA and TRPS. Small EV RNAs were isolated and sequenced by NGS. Differential expression of EV miRNAs was analysed by DESeq2 followed by functional enrichment analysis. Furthermore, isolated EVs were applied in an *in vitro* proliferation assay on primary muscle derived cells to test the functional effect of BFRE primed EVs.

**Results**: No difference in EV quantity or size was observed after BFRE. However, the expression of 16 miRNAs was significantly altered after BFRE. Target prediction and functional enrichment analysis of these miRNAs revealed several interesting target genes and biological pathways involved in skeletal muscle cell turnover. Interestingly, BFRE conditioned EVs significantly increased the proliferation of muscle precursor cells.

**Summary/Conclusion**: EVs from BFRE plasma have an altered miRNA profile that might be responsible for the increased cell proliferation, indicating that EVs in part carry the functional response of BFRE.

**Funding**: The study was supported by the Novo Nordisk Foundation and Riisfort Foundation.

PT08.06

Unveiling the role of EV-mediated vascular cell: cell communication in human pulmonary hypertension

Fernando De la Cuesta; Raghu Bhushan; Julie Rodor; Andrew H Baker

Centre for Cardiovascular Science, University of Edinburgh, Edinburgh, UK

**Background**: Although existing drug interventions successfully relief the symptoms of pulmonary arterial hypertension (PAH), most patients eventually become resistant to therapy and succumb to the disease. Excessive TGFβ signalling is a common cause of PAH and stimulation of this pathway is a well-known strategy to mimic the disease *in vitro*.

Previous results from our groupshow transport of non-coding RNAs (ncRNAs) enclosed in extracellular vesicles (EVs) from human pulmonary smooth muscle cells (PASMC) to endothelial cells (PAEC) plays a crucial role in PAH progression. The aim of this work is to monitor transport of EVs within vascular cells in an *in vitro* model of PAH and to characterize the impact of ncRNA cargoes in this communication.

**Methods**: To visualize local transfer of EVs from PASMCs (donor) to PAECs (recipient) in co-culture, PASMC were transduced with a lentivirus (MOI = 10) conferring the ability to transcribe Cre recombinase and store its mRNA in EVs (PASMC Cre+). Recipient PAECs had been transduced with a reporter lentivirus (MOI = 0.2) to exhibit red fluorescence on basal conditions and switch into green upon receipt of EVs carrying Cre mRNA (PAECs Rep+). PAECs Rep+ were FACS sorted and co-cultured together with PASMC Cre+.

**Results**: Expression of Cre mRNA in PASMC EVs was confirmed by qPCR. Co-cultures showed significantly increased ratio of eGFP+/DsRed+ cells with greater proportion of PASMC Cre+: PAECs Rep+ (1:1 = 9.7-fold; 2:1 = 24.5-fold and 3:1 = 44.9-fold), which supports reporter switch being caused by Cre transferred by EVs from donor cells. Modulation of TGFβ signalling in PASMCs using TGFβ1 and BMP4 did not alter release of EVs (Control = 1.06×10^9^ EVs/ml) or uptake by recipient PAECs (Control = 1.53±0.26%). For this reason we hypothesize differentially transported cargoes may account for a potential phenotypic switch in recipient PAECs.

**Summary/Conclusion**: Using a modified Cre-loxP method, we have been able for the first time to visualize local transfer of EVs within primary vascular cells in co-culture (from PASMC to PAEC). PAH induction *in vitro* through modulation of TGFβ signalling does not affect release of EVs by donor nor uptake by recipient cells. Therefore transport mediated by EVs is not enhanced during PAH development *in vitro*.

**Funding**: This work was funded by MSCA-Individual Fellowship and British Heart Foundation (BHF).

PT08.07

Cardiac dysfunction after myocardial infarction: role of pro-inflammatory extracellular vesicles

Vanessa Biemmi ^1^; Giuseppina Milano^1^; Stefano Panella^1^; Alessandra Ciullo^1^; Francesco Muoio^1^; Elisabetta Cervio^1^; Tiziano Tallone^1^; Tiziano Moccetti^2^; Giuseppe Vassalli^3^; Lucio Barile^1^

^1^Laboratory of Cellular and Molecular Cardiology, Fondazione Cardiocentro Ticino, Lugano, Switzerland. Swiss institute for Regenerative Medicine (SIRM), Lugano, Switzerland; ^2^Fondazione Cardiocentro Ticino, Lugano, Switzerland; ^3^Laboratory of Cellular and Molecular Cardiology, Fondazione Cardiocentro Ticino, Lugano, Switzerland

**Background**: Cardiac repair after myocardial infarction (MI) is a complex series of events initiated by an important inflammatory phase followed by a reparative step. A prolonged or incorrect inflammatory phase can lead to cell death and infarct expansion. Extracellular vesicles (EVs), including exosomes (40–150 nm, Exo) and microvesicles (200–400 nm, MV), are secreted by cells as mediator of cell-cell communication due to their ability to shuttle nucleic acids and proteins. Secreted EVs play a crucial role in the acute and chronic phases of MI, in terms of inflammatory progression and myocardial remodeling. GW4869 is a sphingomyelinase inhibitor, able to inhibit the release of mature Exo from MVBs. In this study we examined whether blocking the generation of inflammatory Exo was protective against ventricular dysfunction after MI.

**Methods**: To study cytotoxic effect of pro-inflammatory EVs, GW4869 or vehicle were injected IP in rats 1 h before the LAD ligation. Twenty four hours after injection rats underwent blood sampling and echocardiography. The total number of EVs in rat plasma was assessed by NanoSight. To assess heart function progression, echocardiography and hemodynamic analysis was performed at 7, 14 and 28 days after MI

**Results**: The concentration of EVs significantly decreases in GW4869 treated group as compared to vehicle injected animals. Moreover the number of infiltrated monocyte, CD68+ cells, in hearts was significantly reduced after injection of GW4869. Left ventricular ejection fraction (EF%) was comparably reduced in both groups at 24 h post-MI but recovered to a greater extent in the GW4869-treated group than in control rats at 28 days post-MI. Moreover scar size was reduced in GW4869 treated group compared to vehicle one. Animals treated with GW4869 display a higher velocity of left ventricle relaxation and an improved contractility capacity, both in the contraction phases and in the relaxation one.

**Summary/Conclusion**: The inhibition of release of pro-inflammatory EVs production *in vivo* by injection of GW is able to reduce the inflammatory process and leads to a greater improve of cardiac function after MI.

PT08.08

Atherosclerotic patients have lower levels of BLTR1 expressing microvesicles compared to healthy individuals

Mathilde Sanden ^1^; Jaco Botha^2^; Michael René Skjelbo Nielsen^3^; Morten Hjuler Nielsen^1^; Erik Berg Schmidt^3^; Aase Handberg^1^

^1^Department of Clinical Biochemistry, Aalborg University Hospital, Aalborg, Denmark; ^2^Department of Clinical Biochemistry, Aalborg University Hospital, Aalborg, Denmark, Dronninglund, Denmark; ^3^Department of Cardiology, Aalborg University Hospital, Aalborg, Denmark

**Background**: Monocytes/macrophages play a crucial role in the development, progression, and complication of atherosclerosis. In particular, foam cell formation driven by CD36 mediated internalisation of oxLDL leads to activation of monocytes and subsequent release of monocyte-derived microvesicles (MMVs). Further, pro-inflammatory leukotriene B4 derived from arachidonic acid (AA) promotes atherosclerosis through the high-affinity receptor BLTR1. Thus, we aimed to investigate the correlation between different MMV phenotypes on the one hand, and AA and eicosapentaenoic acid (EPA) contents in different compartments including atherosclerotic plaques, plasma and granulocytes on the other. This might elucidate the potential of CD36 and BLTR1 bearing MMV phenotypes as novel biomarkers in predicting atherosclerosis.

**Methods**: Plasma samples from 48 subjects with femoral atherosclerosis and 24 healthy controls were analysed on an Apogee A60 Micro-PLUS flow cytometer. Platelet-poor plasma was labelled with lactadherin-FITC, anti-CD14-APC, anti-CD36-PE and anti-BLTR1-AF700. MVs were defined as phosphatidylserine-exposing (PS+) events ≤1000 nm in size. EPA and AA content in granulocytes, plasma phospholipids and atherosclerotic plaques were analysed using gas chromatography.

**Results**: Patients with atherosclerosis had lower levels of BLTR1+ MVs (*p* = 0.007), CD14+BLTR1+ MVs (*p* = 0.007) and CD14+BLTR1+CD36+ MVs (*p* = 0.001) compared to healthy controls. Further, CD14+ MVs and CD14+CD36+ MVs correlated negatively with AA in granulocytes (*r* = -0.302, *p* = 0.039 and *r* = -0.322, *p* = 0.028, respectively). CD14+CD36+ MVs correlated negatively with AA in plasma phospholipids (*r* = -0.315, *p* = 0.029). Lastly, CD14+ MVs and CD14+BLTR1+ MVs tended to correlate inversely with AA in plasma phospholipids and AA in atherosclerotic plaques, respectively (*r* = -0.284, *p* = 0.050 and r = -0.291, *p* = 0.058).

**Summary/Conclusion**: We demonstrated that atherosclerotic patients had lower levels of BLTR1+ MV phenotypes compared to healthy individuals. This is possibly caused by higher levels of LTB4 and inflammatory cytokines in patients which leads to receptor down-regulation. Further investigations into the origin and phenotype may support the potential of circulating MVs in early diagnosis of atherosclerosis.

PT08.09

Microvesicles from T cells overexpress miR-146b-5p in HIV-1 infection and repress endothelial activation

Estelle Balducci; Aurelie S. Leroyer; Romaric Lacroix; Stéphane Robert; Dilyana Todorova; Stéphanie Simoncini; Luc Lyonnet; Corinne Chareyre; Olivia Zaegel-Faucher; Joelle Micallef; Isabelle Poizot-Martin; Patrice Roll; Françoise Dignat-George

Aix-Marseille Université, Marseille, France

**Background**: Human immunodeficiency virus type 1 (HIV-1) promotes a generalized activation of host responses that involves not only CD4 T cells, but also cells of the microenvironment, which are not directly infected, such as endothelial cells. The mechanisms triggering HIV-1-associated vascular alterations remain poorly understood. Microvesicles (MVs), implicated in cell-to-cell communication, have been recently described as carriers of microRNAs (miRNAs). We hypothesized that HIV-1 infection induce cellular miRNA expression in CD4 T cells which may be vectorized by MVs and transferred in a paracrine manner to endothelial cells to regulate vascular homeostasis.

**Methods**: Using a miRNome quantitative RT-PCR analysis, we showed that HIV-1 infection leads to a dysregulation of several miRNAs and identified miR-146b-5p as upregulated in both CD4 T cells, CD4 T cells derived-MVs and circulating MV obtained from antiretroviral therapy-naive HIV-1-infected patients, compared to age- and sex-matched healthy subjects. We further used a CEM T cell line transfected with miR-146b-5p mimic to study the effects of MVs from CEM overexpressing miR-146b-5p mimic (miR-146b-MVs) on the endothelium.

**Results**: Here, we show that MVs from T cell line overexpressing miR-146b-5p mimics (miR-146b-MVs): (1) protect their miRNA cargo from RNase A degradation, (2) transfer miR-146b-5p mimic into human umbilical vein endothelial cell and (3) reduce endothelial inflammatory responses *in vitro* and *in vivo* in lungs from mice injected with miR-146b-MVs. This paracrine control of endothelial inflammatory responses mediated by MVs involved decreased expression of nuclear factor-κB (NF-κB) responsive molecules, ICAM-1 and VCAM-1, through down-regulation of IRAK1 and TRAF6.

**Summary/Conclusion**: These data advance our understanding on chronic inflammatory responses affecting endothelial homeostasis, in infectious and non-infectious diseases and pave the way for potential new anti-inflammatory strategies.

**Funding**: Grants were received from the French National Agency for Research on AIDS and Viral Hepatitis (ANRS) (http://www.anrs.fr/).

PT08.10

Extracellular vesicles mediate a targeted translocation of functional inflammatory modulators between vascular endothelial cells and monocytes

Baharak Hosseinkhani ^1^; Nynke M.S. Van den Akker^2^; Sören Kuypers^3^; Daniel G. M. Molin^2^; Luc Michiels^3^

^1^Bionanotechnology group, Biomedical Research Institute (BIOMED), Hasselt University, Hasselt, Belgium; ^2^Maastricht University, Department of Physiology, Cardiovascular Research Institute Maastricht (CARIM), Maastricht, The Netherlands; ^3^Hasselt University, Biomedical Research Institute (BIOMED), Hasselt, Belgium

**Background**: EV-mediated intercellular networking between monocytes (MC) and endothelial cells (EC) plays an active role at the crossing of inflammation and development of vascular disease. So far, the mode of action of released EV during an inflammatory stress response is poorly understood. Therefore, we aim to unravel the immunomodulatory content of EV derived from inflammatory- triggered EC and their pathological and functional impact on the cellular fitness of their recipients.

**Methods**: Transmission electron microscopy, National Testing Agency and western blot were used to confirm the presence of EV in the culture supernatant of a human umbilical vein endothelial cell (HUVEC), either untreated (uEV) or treated with TNF-α to induce an inflammatory stress (tEV). In addition, protein arrays were used to discover the immunomodulatory content of both uEV and tEV. Thereafter, HUVEC and THP-1 were exposed to the uEV and tEV. Relevant pro/anti-inflammatory markers (IL-1β, IL-4, IL-6, IL6-R, IL-8, IL-10, IL-13, TNF-α, ICAM-1, VCAM-1, CCL2, CD-40, HSP-70, CXCL-10, CCL-4, CCL-5, TIMP-2) were evaluated on RNA level (qPCR) and protein level (ELISA, protein arrays) in both cell types. Functionality of uEV and tEV were assessed using migration and adhesion cell-based assays.

**Results**: EV derived from inflamed vascular EC (tEV) was readily taken up by HUVEC and THP-1 in culture. tEV were verified as packages of pro-inflammatory and chemotactic mediators containing GM- CSF, ICAM-1, VCAM-1, IL-6, IL-8, CXCL-10, CCL4 and CCL-5. In addition, tEV were able to translocate various functional inflammatory mediators to their target cells and modulate them towards either pro-inflammatory (HUVEC) or anti -inflammatory (THP-1) mode. Accordingly, *de novo* synthesis of pro-inflammatory markers (IL-6, IL8, ICAM-1 and VCAM-1) in HUVEC treated with tEV were significantly increased. In the case of THP-1, both tEV and uEV do induce anti-inflammatory responses as indicated by the increased level of IL-10, CCL-4 and CCL-5. Moreover, tEV promoted the adhesion and migration of MC and EC respectively.

**Summary/Conclusion**: Taken together, our current findings proved that an inflammatory cross-talk between inflamed EC and their neighboring EC and circulating MC is established via EV-carrying corresponding mediators.

**Funding**: This work was co-financed by the EU through the Interreg IV Flanders-The Netherlands project Interreg V Flanders-The Netherlands project Trans Tech Diagnostics (TTD).

PT08.11

Investigation of monocyte-dependent activation of endothelial cells by microvesicles *in vitro*

Ying Ying Tan; Kieran P. O’Dea; Florence Beckett; Sanooj Soni; Masao Takata

Section of Anaesthetics, Pain Medicine and Intensive Care, Imperial College London, Chelsea and Westminster Hospital, London, UK

**Background**: Vascular retention of circulating microvesicles (MVs) may have a critical role in endothelial dysfunction during systemic inflammation. We recently demonstrated that during subclinical endotoxaemia in mice, MV uptake within the lungs increased through enhanced internalisation by “marginated” monocytes, but found no evidence for uptake by pulmonary vascular endothelial cells. Here, using *in vitro* human and mouse models of endothelial-monocyte co-culture, we investigated the hypothesis that MVs induce endothelial inflammation indirectly via MV-mediated monocyte activation.

**Methods**: MVs were generated from primary human monocytes or J774A.1 mouse macrophages by sequential LPS and ATP treatments. DiD-fluorescence labelled or unlabelled MVs were incubated with human lung microvascular endothelial cells (HLMVECs) or mouse b.End5 cells, alone or in co-culture with human monocytes or mouse lung-marginated monocytes obtained by perfusion. DiD-labelled MV uptake, endothelial activation (VCAM-1 and E-selectin expression) and monocyte activation (CD86 and ICAM-1 expression) were quantified by flow cytometry.

**Results**: MVs were taken up by human and mouse monocytes, but contrasting with our previous *in vivo* findings, HLMVEC and b.End5 cells also showed significant uptake. MVs induced direct activation of endothelial cells, as represented by upregulation of VCAM-1 (HLMVEC: Control 189±65 vs. MV 365±33 MFI, *p* < 0.05; b.End5: Control 26±5 vs. MV 156±72, *p* <0.05.) and E-selectin (HLMVEC: Control 4.8±0.8 vs. MV 24.4±9.2, *p* <0.05, b.End5: Control 7.0±1.5 vs. MV 17.4±3.5, *p* < 0.01.) in monoculture. Endothelial activation was not augmented by monocytes in co-culture model, despite evidence of monocyte activation (CD86 and ICAM-1 upregulation).

**Summary/Conclusion**: Contrary to our hypothesis and *in vivo* results, we found that MVs can directly activate endothelial cells under *in vitro* conditions, with no evidence found for indirect, monocyte-dependent activation. This fundamental discrepancy between *in vitro* and *in vivo* findings provides a caution for the relevance of conventional *in vitro* “static” culture studies for MV uptake, and points to a critical role for vascular capture of circulating MVs by monocytes under *in vivo* physiological “flow” conditions.

**Funding**: This work was funded by the Chelsea & Westminster Health Charity.

PT08.12

Microvesicle release during exercise-induced cardiac stress in young adult hypertension

Lisa Ayers ^1^; Adam Lewandowski^2^; Odaro Huckstep^2^; Wilby Williamson^2^; Berne Ferry^1^; Paul Leeson^2^

^1^Oxford University Hospitals NHS Trust, Oxford, UK; ^2^University of Oxford, Oxford, UK

**Background**: Microvesicles are released into the circulation during cardiac stress. Little is known about microvesicle release in those with hypertension. Microvesicles have both activating and regulatory roles in the pathogenesis of hypertension and may be useful in the diagnosis, prognosis and monitoring of this condition. Therefore, we aim to determine if microvesicle release during cardiac stress differs in young adults with and without hypertensive disease.

**Methods**: Microvesicle release was measured in 23 non-hypertensive and 16 hypertensive young adult participants. Blood samples were obtained during exercise testing at three time-points; before, immediately post and following 20 min of recovery. Platelet, endothelial, leucocyte, granulocyte and monocyte derived microvesicles were measured by flow cytometry.

**Results**: Cardiac stress was associated with a significant elevation in platelet, endothelial, leucocyte, granulocyte and monocyte-derived microvesicles, which returned to baseline within 20 min for endothelial and leucocyte microvesicles. The significant elevation in platelet, granulocyte and monocyte-derived microvesicles was only seen in the non-hypertensive participants, not in those with hypertension.

In addition, in the non-hypertensive group, those with a blunted release of platelet microvesicles had significantly higher diastolic blood pressures during exercise, higher indicators of arterial stiffness and lower respiratory capacity.

**Summary/Conclusion**: In young adult hypertension there is a blunted release of microvesicles during exercise-induced cardiac stress. Furthermore, non-hypertensive participants with a blunted platelet microvesicle release were found to have greater peripheral resistance and arterial stiffness, potential early markers of hypertension risk. Therefore, microvesicle release is hypothesised to be a protective mechanism during cardiac stress, and this mechanism seems to be lost at an early stage of disease.

**Funding**: Dr Lisa Ayers is funded by a Postdoctoral Healthcare Scientist Research Fellowship supported by the National Institute for Health Research and Health Education England.

PT08.13

Specific blood EV phenotype in angiotensin induced HTN in Mice

Sabrina La Salvia ^1^; Luca Musante^2^; Joanne Lannigan^3^; Sylvia Cechova^3^; Thu H H. Le^3^; Uta Erdbrügger^3^

^1^Genomic and post-Genomic Center, C. Mondino National Institute of Neurology Foundation, IRCCS, Pavia, Italy; ^2^University of Virginia Health System, Department of Medicine, Division of Nephrology, Charlottesville, VA, USA; ^3^Department of Medicine, Division of Nephrology, University of Virginia, Charlottesville, VA, USA

**Background**: Hypertension (HTN) affects over 50 million Americans and is a major risk factor for a wide range of cardiovascular diseases. There is emerging evidence that extracellular vesicles (EVs) might be novel bio-markers and bio-activators in the pathogenesis of HTN. EVs are derived from parental cells, including endothelial cells, platelets, and immune cells. The aim of our study is to define the exact parental cell origin of circulating EVs in Angiotensin II (A II) induced HTN before and after antihypertensive treatment (Rx)

**Methods**: Mice were treated with A II (400 ng/kg/min) via mini-osmotic pumps for 2 and 4weeks. A group of mice was treated with Candesartan (ARB), and another group with Hydralazine hydrochloride, Hydrochlorothiazide, Reserpine (HHR). Systolic blood pressure (SBP) was measured using tail-cuff manometry. Enumeration and phenotyping of EVs were determined using imaging flow cytometry and the following surface markers: E-Selectin (CD62E), Endoglin (CD105), VE cadherin (CD144), PECAM (CD31), platelets (CD41), p-Selectin (CD62P), leukocytes (CD45), monocyte/macrophage (Ly6g-/CD11B+), T-cell (CD3) and B-cells (CD19)

**Results**: Compared to untreated controls (*n* = 7), A II treated mice (*n* = 11) had an increase in SBP by 30 mmHG (*p* = 0.02) and SBP was significantly and equally reduced with ARB (*n* = 5, *p* = 0.0026 vs. A II alone) or HHR (*n* = 5, *p* = 0.0340, vs. A II alone). Endothelial derived EVs (CD62E, CD105, CD144 and CD31) were significantly elevated after 2 weeks of Angiotensin II Rx only, but decreased after 4 weeks. In contrast, leukocyte derived EVs (CD45+) were significantly increased after 2 weeks (A II Rx) and remained elevated after 4weeks. The average numbers of Monocyte/Macrophage derived EVs), were numerically reduced with HHR-treated mice, and increased with ARB treated-mice, whereas T- and B-cell derived EVs were numerically lower after antihypertensive Rx in both HHR and ARB treated groups

**Summary/Conclusion**: Specific populations of endothelial and leukocyte derived EVs are elevated at different time points during A II induced HTN. Subgroups of myeloid and lymphoid derived EVs may be differently affected with AT1 receptor dependent and independent antihypertensive treatment. The changing profiles of the EVs may reflect an evolving (patho-) physiologic response of the vasculature before and after antihypertensive treatment

PT08.14

The protein cargo of endothelial cell-derived extracellular vesicles released in response to IL-3 identifies the Wnt signaling pathway as a relevant mediator of inflammation

Giusy Lombardo ^1^; Patrizia Dentelli^1^; Gabriele Togliatto^1^; Arturo Rosso^1^; Kari Espolin Fladmark^2^; Giovanni Camussi^1^; Maria Felice Brizzi^1^

^1^Department of Medical Sciences University of Turin, Turin, Italy; ^2^Department of Molecular Biology, University of Bergen, Bergen, Norway

**Background**: The proangiogenic cytokine Interleukin 3 (IL-3) is released by inflammatory cells in physiological and pathological conditions. We have previously shown that IL-3-treated endothelial cells (ECs) release extracellular vesicles (EVs), which serve as a paracrine mechanism for neighboring ECs, by transferring active molecules. However, the real impact of EC-derived IL-3-EV protein cargo in inflammatory settings has been poorly investigated.

**Methods**: In this study, we focused on the EC-IL-3-EV protein content using label free mass spectrometry based analysis to identify the differentially expressed proteins in EC-IL-3-EVs vs. EC-EVs. Moreover, siRNA technology was applied to validate proteomic analysis.

**Results**: Among the 563 identified proteins, 445 proteins are upregulated and 67 proteins are downregulated (±1.5-fold variations) in EC-IL3-EVs compared to the EC-EVs. Proteins enriched in EC-IL-3-EVs are mostly linked to molecular functions involved in translation, catalytic, transferase, glucosidase, peroxidase mRNA and RNA binding activity. Down-regulated proteins are mostly nuclear proteins, like proteins involved in nucleotide binding and RNA splicing. Analyzing the biological pathways, we found that EC-IL-3-EVs-mediated signaling events are mainly related to the angiogenic pathways (e.g. PDGFR beta, VEGF, VEGFR, ALK1, TGF beta or Wnt signaling pathways). In particular, the Wnt signaling pathway seems to be a key mediator of EC-IL-3-EVs in inflammatory settings, as demonstrated by functional *in vitro* validation.

**Summary/Conclusion**: Taken together these results show for the first time a proteomic profile of EC-derived EVs in an inflammatory setting containing IL-3, and identifies the Wnt signaling pathway as a potential therapeutic target.

PT08.16

MicroRNA-19 in human adipose-derived stem cells exosomes rescuing acute liver failure rats models through anti-inflammatory effect

Yinpeng Jin; Xi Wang; Hongchao Li; Qingchun Fu

Shanghai Public Health Clinical Center, Fudan University, Shanghai, China (People’s Republic)

**Background**: Multiple studies have shown that human adipose-derived stem cells (hASCs) are used to treat multiple diseases by secreting exosomes. Exosomes contain a variety of substances, including multiple microRNA involved in inflammatory response, which can enhance or inhibit the inflammatory response by promoting or inhibiting inflammatory cytokines. Early experiment confirmed the theory in our group, rats with liver failure were treated with hASCs exosomes, a variety of inflammatory pathways fall in liver tissue.

**Methods**: The lymphocytes were obtained from the spleen of mice respectively using hASCs, overexpressing/silencing microRNA - 19 hASCs exosomes deal with LPS activated lymphocyte secretion, and observe the inflammatory factor, active oxygen change,P47phox and lymphocyte apoptosis. The model of hepatic failure was constructed by using hASCs exosomes, overexpression/silencing microrna-19 hASCs exosomes, to observe the survival rate of rats, inflammatory markers of liver tissue and pathological changes of liver tissues.

**Results**: The expression levels of il-10, il-1, il-6 and TNF- were the lowest, and the silent group was the highest *in vitro* cell experiments.The lymphocyte apoptosis was the lightest and the silent group was the most serious in the expression of microRNA-19 exosomes. Active oxygen and P47phox change with inflammatory factors. In the animal experiment, the survival rate of the overexpressing microRNA-19 hASCs exosomes group was the highest, the liver tissue pathology, active oxygen and P47phox were the lowest, while the silent group was the opposite.

**Summary/Conclusion**: MicroRNA-19 in the hASCs exosomes can inhibit liver tissue inflammation of the liver failure rat model induced by D - gal.The treatment mechanism of exosomes is further explored, for the future clinical use of hASCs exosomes to provide theoretical basis for treatment of hepatic failure patients.

PT08.17 = OWP3.02

Origin of extracellular vesicles released during exhaustive exercise

PT09: EVs in Autoimmunity and Sepsis Chairs: Lola Fernandez Messina; Fabiana Geraci Location: Exhibit Hall17:15-18:30

PT09.01= OWP1.02

Role of CD4 in therapeutic mesenchymal stem cell-derived vesicles for joint diseases

PT09.02

Anti-inflammatory activity of exosome-mimetic nanovesicles from mesenchymal stem cells in septic mice

Kyong-Su Park ^1^; Ganesh V. Shelke^2^; Kristina Svennerholm^3^; Elga Bandeira^1^; Cecilia Lässer^2^; Su Chul Jang^4^; Rakesh Chandode^5^; Inta Gribonika^5^; Jan Lötvall^2^

^1^University of Gothenburg, Gothenburg, Sweden; ^2^Krefting Research Centre, Institute of Medicine, University of Gothenburg, Gothenburg, Sweden; ^3^Anesthesiology and Intensive Care Medicine, Institute of Clinical Science, Sahlgrenska Academy, University of Gothenburg, Gothenburg, Sweden; ^4^Krefting Research Centre, Institute of Medicine, University of Gothenburg, Boston, USA; ^5^Department of Microbiology and Immunology, Institute of Biomedicine, University of Gothenburg, Gothenburg, Sweden

**Background**: Sepsis remains a source of high mortality in hospitalized patients despite proper antibiotics approaches. Treatment with exosomes from mesenchymal stem cells (MSCs) is an evolving field in sepsis due to their immunosuppressive properties. However, exosomes are naturally produced at low quantities, and the isolation method is demanding. Recently, artificially generated nanovesicles (NVs) from cells have been applied to various disease models to overcome the disadvantages of exosomes. The aim of this study to determine whether MSCs-derived NVs can suppress local and systemic inflammation in septic mice, and to elucidate the mechanism involved.

**Methods**: NVs were produced from bone marrow-derived MSCs by the breakdown of cells through serial extrusions through filters. Isolated NVs were analysed by transmission electron microscopy. Mice (C57BL/6) were intraperitoneally injected with E. coli-derived outer membrane vesicles (OMVs) to establish sepsis, and then injected with 2000 million NVs. Eye exudates and body temperature were evaluated at 6 h. Innate inflammation was assessed in peritoneal fluid and blood through investigation of infiltration of cells and cytokine production. The biodistribution of NVs labelled with Cy7 dye was analysed using near-infrared imaging.

**Results**: The NVs were characterized by spherical shape and diameters of 100–200 nm. NVs inhibited OMVs-induced eye exudates and hypothermia, representing septic signs. Moreover, NVs significantly suppressed neutrophil infiltration in peritoneum and various chemokines and cytokines production in blood, notably TNF-α and IL-6. In biodistribution study, NVs spread to the whole mouse body and localized in the lung, liver and kidney at 6 h.

**Summary/Conclusion**: This study shows that MSCs-derived NVs have beneficial effects in mice model with sepsis through immunomodulation of cells and cytokines, suggesting that artificial NVs may be novel exosome-mimetics to clinically applicable to septic patients.

**Funding**: This work was supported by grants from Göteborgs Läkarsällskap and CODIAK Biosciences Inc.

PT09.03

Rheumatoid factor is detected on circulating extracellular vesicles in a subpopulation of rheumatoid arthritis patients with a more severe disease phenotype

Onno Arntz ^1^; Bartijn Pieters^1^; Rogier Thurlings^2^; Peter van de Kraan^1^; Fons van de Loo^1^

^1^Experimental Rheumatology, Radboudumc, Nijmegen, The Netherlands; ^2^Rheumatology, Radboudumc, Nijmegen, The Netherlands

**Background**: Rheumatoid arthritis (RA) is a systemic disease characterized by polyarticular joint inflammation. In 65%–80% of RA patients rheumatoid factor (RF), autoantibodies of immunoglobulin -M, -A or -G classes directed against the Fc portion of IgG, is detectable in their circulation. High RF levels predict a more severe disease and comorbidities, probably due to their involvement in immune complex formation and activation of complement (crucial mediators of the effector phase of inflammation in the pathogenesis of RA). Extracellular vesicles (EVs) play an important role in cell-cell communication and are produced by all cells including B-cells that express membrane-bound antibodies (B-cell receptor). In this study we investigate whether RF + EVs are detectable in the circulation of RA patients and if this relates to parameters of disease activity.

**Methods**: EVs were isolated from platelet-free plasma of 38 RA patients and from age and sex-matched 24 healthy controls (HC) by size exclusion chromatography. EV markers (tetraspanins) were detected by western blot and miRNA content by RT-qPCR. Particle size and concentration were measured by electron microscopy and nanosight tracking analysis. Protein concentration was determined by micro-BCA. RF levels were measured using a commercial ELISA. The percentage of RF + EVs was determined by measuring bound and unbound PHK labeled EVs to protein L magnetic beads in a fluorometer.

**Results**: Mean EV particle size, concentration and protein content were not different between RA patients and HC. Twenty seven of the 38 RA patients were classified as RF + (>10 IU/ml) and of the clinical parameters studied only their erythrocyte sedimentation rate (ESR) was higher (31 vs. 14 mm/hr). In 14 RF + patients, RF was detectable on a small portion of EVs not exceeding 4% of the total number of circulating EVs. Interestingly, RA patients with RF + EVs showed higher disease activity as assessed by patient global health assessment using a visual analog scale (63 vs. 31), blood C-reactive protein (22 vs. 9 mg/l) and ESR (43 vs. 19 mm/hr) levels, than RA patients with undetectable RF + EVs.

**Summary/Conclusion**: This study shows for the first time that in a subpopulation of RA patients RF is present on EVs, which might originate from their B-cells. The higher disease activity in RA patients expressing RF on their EVs suggests that RF + EVs are involved in RA pathogenesis.

PT09.04

Extracellular vesicles (EVs) secreted by mesenchymal stem cells (MSCs) exert opposite effects with respect to their cells of origin in mice with DSS-induced colitis

Michele Grassi ^1^; Martina Piccoli^1^; Anna Maria Tolomeo^1^; Chiara Franzin^2^; Marcin Jurga^3^; Barbara Lukomska^4^; Chiara Marchiori^1^; Andrea Porzionato^1^; Ignazio Castagliuolo^1^; Michela Pozzobon^2^; Maurizio Muraca^1^

^1^University of Padova, Italy, Padova, Italy; ^2^Stem Cells and Regenerative Medicine Lab, Fondazione Istituto di Ricerca Pediatrica Città della Speranza, Padova, Italy; ^3^The Cell Factory BVBA (Esperite NV), Niel, Belgium; ^4^NeuroRepair Department, MMRC, PAS, Warsaw, Poland

**Background**: Several reports have described a beneficial effect of MSC administration in mice with experimental colitis. However, worsening of colitis following MSC treatment was also reported.

**Methods**: We compared the effects of MSCs or MSC-EV administration in dextran sulfate sodium (DSS) colitis model. Since cytokine conditioning was reported to enhance MSC immune modulatory activity, the cells were kept either under standard culture conditions (naïve, nMSCs) or primed by IL1b, IL6 and TNFalfa (induced, iMSCs)

Colitis was induced in C57BL/6 mice with DSS in drinking water for 5 days followed by 2 days on plain water. Healthy controls received plain water. Colitic animals were assigned to one of the following treatments on days 3, 5 and 7: vehicle only (controls), 1 × 10^7^ nMSCs, 1 × 10^7^ iMSCs, 3 × 10^10^ nMSC-EVs and 3 × 10^10^ iMSC-EVs. Animals were sacrificed on day 8. To assess colitis severity we determined: changes in body wt, Disease Activity Index, colon length, histomorphometric analysis of the whole colon, cytokine expression in intestinal mucosa.

**Results**: nMSCs and iMSCs administration was associated with clinical and histological worsening with respect to controls. However, mice treated with both nMSC-EVs and iMSC-EVs showed clinical improvement, even if no significant difference was found in histological/morphometric score with respect to controls. These opposite effects were particularly evident with iMSCs. Cytokine expression in colon mucosa showed reduced TNFalpha and increased IL-10 in mice treated with iMSC-EVs.

**Summary/Conclusion**: In conclusion, both nMSCs and iMSCs worsened DSS-induced colitis, confirming that these cells can behave as pro-inflammatory agents depending on the environment. In contrast, both nMSC-EVs and iMSC-EVs showed a partially beneficial effect, suggesting a more predictable behaviour and a safer therapeutic profile with respect to their cells of origin.

**Funding**: The BROAD MEDICAL RESEARCH PROGRAM AT CCFA supported this work

PT09.05

Neutrophil-derived exosome drives the autoinflammatory responses of generalized pustular psoriasis via activating NOD2 in keratinocytes

Shuai Shao; Hui Fang; Gang Wang

Department of Dermatology, Xijing Hospital, Fourth Military Medical University, Xi’an, China (People’s Republic)

**Background**: Generalized pustular psoriasis (GPP) is a rare, recurrent and life-threatening disease, characterized by the infiltration of neutrophils into the epidermis to form generalized pustules. Neutrophils are the most abundant leukocytes present in human blood and in the lesional skin of GPP patients. Though short-lived, neutrophils can immediately secrete cytokines, chemokines and vesicles. Our study aimed to illustrate the functions of neutrophils in the immune disorder of GPP.

**Methods**: Clinical data analysis, real time PCR, western bot, co-culture cells, electron microscope, flow cytometry, mass spectrometry, ELISA and siRNA.

**Results**: Herein, we demonstrated that the neutrophil to lymphocyte ratio (NLR) was correlated with the severity of GPP, and decreased dramatically after effective treatment, which indicated that the NLR score could be a marker for the severity and prognosis of GPP, and neutrophil might play a critical role in the pathogenesis of GPP. Besides, keratinocytes co-cultured with GPP neutrophils indirectly produced more CXCL1, CXCL2, CXCL8, CCL20, IL36G and TNFα than those in the direct co-culturing system. Further, exosomes derived from GPP neutrophils could enter and activate keratinocytes to secrete the above-mentioned mediators. The proteome profiling of GPP neutrophil exosomes identified olfactomedin 4 (OLFM4) as a critical distinct protein. And neutrophil exosomes with OLFM4 cargo activated keratinocytes to highly produce these chemokines and cytokines via NOD2 and the downstream NFκb and MAPK signaling pathways. Importantly, the flow cytometry results found that the proportion of OLFM4-positive neutrophils was shown to be higher in patients with progressive GPP than that in controls.

**Summary/Conclusion**: Taken together, these data suggest that neutrophil-derived exosomes enter keratinocytes to stimulate the expression and secretion of CXCLs, IL36G and TNFα, resulting in the chemotaxis of more neutrophils, which promotes the autoinflammatory responses in generalized pustular psoriasis.

**Funding**: This work was supported by National Natural Science Foundation of China [nos. 81430073 and 81502716]

PT09.06

Pro-inflammatory role of blister fluid-derived exosomes in bullous pemphigoid

Hui Fang; Shuai Shao; Man Jiang; Gang Wang

Department of Dermatology, Xijing Hospital, Fourth Military Medical University, Xi’an, China (People’s Republic)

**Background**: Bullous pemphigoid is an autoimmune inflammatory disorder characterized by the presence of autoantibodies against bullous pemphigoid autoantigens, leading to dermal-epidermal separation with consequent blister formation. However, whether and how the components of blister fluid exacerbate the progression of bullous pemphigoid is unclear. Exosomes are nanometre-sized vesicles released from cells into the body fluid, where they can transmit signals throughout the body.

**Methods**: Blister fluid exosomes from patients with BP were characterized by electron microscopy, western blot analysis and Nanosight. Blister fluid exosomes were incubated with primary human keratinocytes *in vitro*. Cytokines were measured by RT-PCR and ELISA. The protein content of blister fluid exosomes was analysed by mass spectrometry.

**Results**: We found that exosomes isolated from the blister fluids of patients with bullous pemphigoid exhibited the expected size and expressed marker proteins CD63, CD81 and CD9. In addition, blister fluid-derived exosomes were internalised by human primary keratinocytes, inducing the production of critical inflammatory cytokines and chemokines. Western blotting analysis showed robust and rapid activation of ERK1/2 and STAT3 signalling pathways in human primary keratinocytes after stimulation with blister fluid-derived exosomes. We also found that the blister fluid-derived exosomes indirectly induced neutrophil trafficking through up-regulating CXCL8 *in vitro*. Furthermore, CD63 was localised mostly to keratinocytes and infiltrated granulocytes in skin lesions, suggesting that these cells are the possible sources of exosomes in blister fluid. Using mass spectrometry, we analysed the proteomes of blister fluid-derived exosomes and identified a variety of proteins implicated in inflammatory and immune responses.

**Summary/Conclusion**: Our findings provide strong evidence that blister fluid-derived exosomes are involved in the local autoinflammatory responses of the skin associated with bullous pemphigoid.

**Funding**: This work was supported by grants from the National Natural Science Foundation of China [81220108016 and 81703125].

PT09.07

T-cell-derived exosomes are potential biomarkers or therapeutic targets for autoimmune diseases

Huai-Chia Chuang; Tse-Hua Tan

Immunology Research Center, National Health Research Institutes, Zhunan, Taiwan (Republic of China)

**Background**: Systemic lupus erythematosus (SLE) and rheumatoid arthritis (RA) are chronic, debilitating, incurable, and life-threatening diseases; patients need to receive treatments throughout their life. Identification of novel therapeutic targets will help development of effective treatments for SLE or RA. The number of exosomes in sera of SLE patients is correlated with the disease severity of SLE patients. To date, the properties (specific surface markers and intra-exosomal molecules) of exosomes in SLE or RA patients, as well as regulatory mechanisms of exosome-mediated autoimmune responses remain unclear. In addition, T cells play critical roles in the pathogenesis of SLE or RA. Thus, it is important to identify and characterize T-cell-derived exosomes in SLE and RA patients as novel biomarkers or therapeutic targets for SLE and RA.

**Methods**: To study the properties of T-cell-derived exosomes from autoimmune patients, T-cell-derived exosomes isolated from SLE and RA patients were subjected to proteomics and MACSPlex assays. The identified intra-exosomal molecules or surface molecules were further characterized using clinical samples and animal models for autoimmune diseases. (Written informed consent, approved by the IRB at either Taichung Veterans General Hospital, Taiwan (#C10130B) or Taipei Veterans General Hospital, Taiwan (#2017-06-003BC), was obtained from all patients.)

**Results**: The flow cytometry data showed that numbers of T-cell-derived exosomes were drastically enhanced in supernatants of T cells from SLE and RA patients compared to those from HC. Sixteen and 14 exosomal surface proteins were increased in SLE patients and RA patients, respectively. The proteomics data showed that multiple proteins were specifically expressed in T-cell-derived exosomes of all SLE patients but not in HC. The identified SLE-specific exosomal proteins included surface proteins, protein kinases, protein phosphatases and metabolic enzymes. Notably, several SLE-specific exosomal proteins in T-cell-derived exosomes were overexpressed in autoimmune disease animal models. The potential pathogenic roles of these identified molecules will be presented in the meeting.

**Summary/Conclusion**: The identified intra-exosomal proteins and surface proteins of T-cell-derived exosomes are potential biomarkers or therapeutic targets for SLE or RA.

PT09.08

Decoding the role of circulating extracellular vesicles in chronic fatigue syndrome/myalgic wncephalomyelitis: an exploratory pilot study

Jesus Castro-Marrero^1^; Esther Serrano-Pertierra^2^; Myriam Oliveira-Rodríguez^2^; Maria Cleofé Zaragozá^1^; Jose Alegre^1^; Maria del Carmen Blanco-López ^2^

^1^CFS/ME Unit, Vall d’Hebron University Hospital Research Institute (VHIR), Universitat Autònoma de Barcelona, Barcelona, Spain; ^2^Department of Physical and Analytical Chemistry, Faculty of Chemistry, University of Oviedo, Oviedo, Spain

**Background**: Chronic fatigue syndrome (CFS), also known as myalgic encephalomyelitis (ME) is a complex, heterogeneous and multisystem neuroimmune condition of unknown specific cause, and for which no clinically established diagnostic tests, and no universally FDA-approved drugs for treatment are available. CFS/ME is characterized by an extreme disabling fatigue and other core symptoms that do not improve with rest; it persists for more than 6 months, and cannot be explained by any underlying medical condition. Circulating EVs could be an emerging tool for biomedical research in CFS/ME. To date, no data on EV biology in CFS/ME are as yet available. This study aimed to isolate and characterize the blood-derived EVs from CFS/ME patients and to address its utility as effective non-invasive biomarkers in the clinical setting.

**Methods**: Serum-derived EVs were isolated from 10 Spanish CFS/ME patients and 5 age- and sex-matched healthy controls (HCs) using a polymer-based precipitation method. Their protein cargo, size distribution and concentration were analyszed by western blot and nanoparticle tracking analysis. Furthermore, EVs were detected using a lateral flow immunoassay based on exosomal tetraspanins markers CD9 and CD63 as targets.

**Results**: We found that the EV-enriched fraction amount was significantly higher in CFS/ME than in HCs (*p* = 0.007). EVs were significantly smaller than in CFS/ME compared with HCs (*p* = 0.014). No significant differences were found regarding the CD9 signal intensity on EV-enriched fractions between CFS/ME and HCs. However, CD63 levels were somewhat higher in CFS/ME than in HCs, although this trend did not reach statistical significance.

**Summary/Conclusion**: This is the first study to show preliminary evidence of changes in the number and size of circulating EVs in CFS/ME, indicating their possible potential involvement in illness pathogenesis. Further studies focusing on critical role that EVs may play in CFS/ME are now urgently warranted.

**Funding**: This work was partially supported by the Consejería de Economía y Empleo del Principado de Asturias (Plan de Ciencias, Tecnología e Innovación 2013–2017) under [grant number GRUPIN14-022]. The authors are gratefully acknowledged for the support from the European Regional Development Fund (ERDF).

PT09.09

Systemic lupus erythematosus (SLE) and rheumatoid arthritis (RA) have increased concentration of serum microvesicles compared to healthy patients

Fernando Vianna Cabral Pucci; Amanda S. Rosa; Arthur V. Sacramento; Rinaldo W. Pereira

Catholic University of Brasilia, Brasilia, Brazil

**Background**: LE is a systemic disease of diffuse presenting clinical characteristics that challenge its diagnosis and prognosis. Rheumatoid arthritis (RA) is a very incident autoimmune disease which affects joints, leading to bone deformities and its diagnosis is still more restricted than that for systemic lupus erythematosus (SLE). The pathogenesis of such diseases is still unclear. Extracellular vesicles, such as exosomes, have an important role in the organism and may be involved in immune response modulation by their carried components. This study proposes the isolation and purification of plasma EV from the healthy controls, SLE and RA patients, comparing the vesicle profiles between them.

**Methods**: The study was approved by the Ethics of Research Committee of UCB. The plasma samples from the participants who signed the informed consent file and were assigned to one of three groups: G1, active SLE patients; G2, active RA patients; and G3, healthy control. EVs were isolated by a series of centrifugations, filtrations and ultracentrifugations. The tunable resistive pulse sensing (qNano) method, NP100 pore (50–330 nm), was the chosen one for quantification and measurement of the vesicles.

**Results**: All three groups were composed of 23 individuals (n = 23). The G1 group obtained a mean concentration of 3.18x10^10^ (±2.06 × 10^10^) particles/ml, an average mode diameter of 91.07 (±7.12) nm and a mean diameter of 117 (±6.41) nm. The G2 group presented a mean concentration of 2.85 × 10^10^ (±2.90 × 10^10^) particles/ml, an average mode diameter of 88.84 (±3.29) nm and meant diameter of 108.76 (±4.2) nm; and G3 showed a mean concentration of 9.65 × 10^09^ (±6.61 × 10^09^) particles/ml, an average mode diameter of 91.44 (±12.33) nm and meant size of 107.8 (±7.56) nm.

**Summary/Conclusion**: We observed the increase of one order of magnitude in the mean concentration of EVS in the SLE and AR patients groups compared to healthy controls. If these increases play some role in the pathogenesis or prognosis is the ongoing investigation.

**Funding**: This work was funded by Fundação de Apoio à Pesquisa do Distrito Federal, CNPq, CAPES and Universidade Católica de Brasília.

PT09.10

Exosome-type vesicular pool of phospholipases A2 in bronchoalveolar lavage fluid of patients with acute respiratory distress syndrome. A new role in the dissemination of inflammation?

Elefteria Kazepidou^1^; Marilena E. Lekka ^1^; George Leondaritis^1^; Marianna Antonelou^2^; Alexia Tsapinou^1^; Apostolos Angeropoulos^1^; Vasilios Koulouras^1^; George Nakos^1^

^1^University of Loannina, Loannina, Greece; ^2^National Kapodistrian of Athens, Athens, Greece

**Background**: Inflammation triggers the release of secretory phospholipase A_2_ (PLA_2_) from a variety of cells, including alveolar epithelial, polymorphonuclear cells and macrophages. The presence of PLA_2_ in the bronchoalveolar lavage (BAL) fluid of patients with acute lung injury/acute respiratory distress syndrome (ALI/ARDS) has been associated with the severity of the syndrome; however, its secretion mechanism is still obscured. During the last years, extracellular vesicles (EVs) of endosomal origin with the physical characteristics of exosomes have been emerged as organelles performing intercellular communication. EVs/exosomes may alter the immune status or even the physiological function of recipient target cells through shuttling of their cargo molecules.

**Methods**: In this work we have characterized EVs/exosomes isolated from BAL fluid of patients with and without ALI/ARDS, using physical, morphological and biochemical approaches. Furthermore, we provide biochemical and morphological evidence for the presence of an EV pool of sPLA2-IIA in the BAL fluid of ARDS patients.

**Results**: Exosomal type extracellular vesicles were isolated from BAL fluid of patients with and without ARDS and characterized on the basis of their density, diameter, the presence of tetraspanins CD63 and CD81 and the absence of GRP78. In the EVs of exosomal type from ARDS patients we identified secretory phospholipase A2 type II (sPLA2-IIA) and in sporadic samples pcPLA2. by immunofluorescence and immunogold TEM.

**Summary/Conclusion**: To our knowledge, this is the first description of exosomal localization of a secreted PLA2 isoform in human samples. Exosomal sPLA2-IIA might be involved in the responsiveness of recipient cells in the lung during the development of ARDS, in a functionally distinct manner from soluble sPLA2 present in BAL fluid, which is presumably implicated in lung surfactant hydrolysis during the course of the disease. The presence of PLA2 isoenzymes on EVs may reveal new insight into the development and propagation of lung inflammation, but can also help adopt appropriate management approaches and thus, new ways for patients’ treatment.

PT09.11

Different anti-inflammatory effects of extracellular vesicles from adipose-derived mesenchymal stem cell or keratinocyte cell line on osteoarthritic cartilage

Miguel Tofiño-Vian ^1^; Isabel Guillén^2^; María Dolores Pérez del Caz^3^; Miguel Ángel Castejón^4^; María José Alcaraz^1^

^1^Departamento de Farmacología e IDM, Universidad de Valencia, Valencia, Spain; ^2^Departamento de Farmacia, CEU-Cardenal Herrera, Valencia, Spain; ^3^Departamento de Quemados y Cirugía Plástica, Hospital La Fe, Valencia, Spain; ^4^Departamento de Cirugía Ortopédica y Traumatología, Hospital de La Ribera, Valencia, Spain

**Background**: Osteoarthritis (OA) is a joint condition associated with articular cartilage loss, low-grade synovitis and alterations in subchondral bone and periarticular tissues. In OA, the interest for mesenchymal stem cell (MSC)-EV therapeutic applications has increased. However, there is an increasing concern about the reproducibility of recent EV publications.

We have assessed the immunomodulary properties of adipose-derived MSCs (AD-MSCs) microvesicles (MV) and exosomes (EX) in OA chondrocytes and compared their effects with EVs from a different biological source. 

**Methods**: AD-MSCs from abdominoplasty fat and immortalized keratinocytes (HaCaT) were cultured with appropriate media supplemented with EV free human serum. EVs were isolated from conditioned media by differential centrifugation and characterized by resistive pulse sensing.

Cartilage explants and primary chondrocytes were obtained from knee specimens of advanced OA. Both were stimulated with interleukin (IL)-1β (10 ng/mL) and treated with MSC- or HaCaT-MV (3.6 × 10^7^ particles (p)/mL) or EX (7.2 × 10^7^ p/mL) for 24 h. Then, levels of IL-6, IL-10 and TNFα were measured.

**Results**: RPS revealed distinct size and concentration EV signatures from AD-MSCs (MV: 317 ± 54 nm and 8 × 10^9^ p/mL; EX: 151 ± 27 nm and 4 × 10^10^ p/mL) or HaCaT (MV: 281 ± 2 nm and 7 × 10^10^ p/mL; EX: 105 ± 1 nm and 1.1 × 10^12^ p/mL).

MSC-EV treatment of OA cartilage explants and chondrocyte cultures reduced the inflammatory cytokines IL-6 and TNFα with respect to those solely stimulated IL-1β. The anti-inflammatory cytokine IL-10 increased with respect to explants or cells solely stimulated with IL-1β. On the contrary, the levels of the same cytokines were not affected by treatment with HaCaT-EVs.

**Summary/Conclusion**: Administration of EV may have potential pharmacological applications in OA. However, experimental procedures to avoid data artefacts are currently lacking; in this regard, the use of non-related EVs as negative controls has proven useful. Interestingly, cell line HaCaT EVs had less deviation in size, and were obtained in higher concentrations, compared to EVs from primary cell cultures. Further studies on EV properties may lead to new and more specific therapeutic targets based on the interaction between AD-MSC-EVs and cells.

**Funding**: This work was funded by MINECO, ISCIII, and FEDER [SAF2013-48724-R] and Generalitat Valenciana [PROMETEOII/2014/071].

PT09.12

Tiotropium inhibits the release of pro-inflammatory extracellular vesicles by acetylcholine-stimulated lung epithelial cells

Tommaso Neri; Valentina Scalise; Ilaria Passalacqua; Roberto Pedrinelli; Pierluigi Paggiaro; Alessandro Celi

University of Pisa, Pisa, Italy

**Background**: Tiotropium is a long-acting muscarinic antagonist routinely used as a bronchodilator in chronic obstructive pulmonary disease (COPD). Based on its role in preventing acute exacerbations of COPD, it has been speculated that besides its known bronchodilator properties tiotropium also exerts anti-inflammatory effects. We have shown that extracellular vesicles (EV) generated by mononuclear cells induce a pro-inflammatory phenotype in human lung epithelial cells. The aim of this study was to investigate whether muscarinic stimulation induces the generation of pro-inflammatory EV by alveolar (A549) and bronchial (16HBE) epithelial cells and whether tiotropium modulates such effect.

**Methods**: The generation of A549- and 16HBE-derived EV induced by acetylcholine (Ach; 1 mM; 1 h) in the presence or in the absence of tiotropium was investigated through a prothrombinase assay. Ach-induced A549-EV and 16HBE-EV were incubated overnight with A549 and 16HBE cells, respectively, and the concentrations of IL-8 and MCP-1 in the conditioned medium assessed by ELISA.

**Results**: Ach stimulation of A549 cells caused an increase in EV from 0.225±0.088 to 0.381±0.087 mM PS (*p* < 0.05; paired t-test). EV generated by Ach-stimulated A549 cells caused an autocrine stimulation of the synthesis of IL-8 (487±242 pg/mL vs. 1896±211 pg/mL for unstimulated and EV-stimulated A549 cells, respectively) and MCP-1 (1299±237 pg/mL vs. 5973±924 pg/mL for unstimulated and EV-stimulated A549 cells); *p* < 0.05 for both comparisons; paired t-test. Preincubation of cells with tiotropium prior to Ach stimulation caused a dose-dependent inhibition of EV generation that reached maximum at 50 pg/mL (0.225±0.101 nM PS). Similar results were obtained with 16HBE cells.

**Summary/Conclusion**: Muscarinic stimulation causes the generation of pro-inflammatory EV by human lung epithelial cells that is inhibited by tiotropium. This observation could contribute to explain the effect of tiotropium in the reduction of acute exacerbations of COPD.

PT09.13

Endothelial Progenitor Cell Exosomes Improve the Outcome of a Murine Model of Sepsis

Yue Zhou; Pengfei Li; Andrew Goodwin; James Cook; Perry Halushka; Hongkuan Fan

Department of Neuroscience, Medical University of South Carolina, Charleston, SC, USA

**Background**: Microvascular dysfunction leads to multi-organ failure and mortality in sepsis. Our previous studies demonstrated that administration of exogenous endothelial progenitor cells (EPCs) confers protection in sepsis as evidenced by reduced vascular leakage, improved organ function and increased survival. We hypothesized that EPC-exosomes protect the microvasculature through the transfer of miRNAs.

**Methods**: Mice were rendered septic by cecal ligation and puncture (CLP), and EPC-exosomes were administered intravenously at 4 h post-CLP. Mice survival, organ dysfunction, plasma cytokines and chemokines, and lung and kidney vascular leakage were determined. We determined the miRNA contents of EPC exosomes with next generation sequencing and examined the potential role of microRNA-126 in the observed benefits of EPC-exosomes.

**Results**: EPC-exosomes treatment improved survival, while suppressing lung and renal vascular leakage, and reducing liver and kidney dysfunction in septic mice. EPC-exosome administration attenuated sepsis-induced increases in plasma levels of IL-6, INFγ, TNFα, IL-10 and MCP-1. Moreover, we found that microRNA-126-3p and 5p were highly abundant in EPC-exosomes. We demonstrated that exosomal miR-126-5p and 3p suppressed LPS-induced HMGB1 and VCAM1 levels, respectively, in human microvascular endothelial cells (HMVECs). Inhibition of microRNA-126-5p and 3p through transfection with microRNA-126-5p and 3p inhibitors abrogated the beneficial effect of EPC-exosomes. The inhibition of exosomal microRNA-126 failed to block LPS-induced increase in HMGB1 and VCAM1 protein levels in HMVECs and negated the protective effect of exosomes on sepsis survival.

**Summary/Conclusion**: EPC-exosomes prevent microvascular dysfunction and improve sepsis outcomes potentially through the delivery of miR-126.

**Funding**: This work was funded by NIH [1R01GM113995].

PT09.14

Exosomes with different surface markers present various exosomal content and function

Ching-Hua Hsieh

Department of Plastic Surgery, Kaohsiung Chang Gung Memorial Hospital, Kaohsiung, Taiwan (Republic of China)

**Background**: The specific surface markers of exosomes secreted during illness are deemed to function as recognition of the target cells for cell-to-cell communication, indicating the host cells may transfer different exosomal content to different cells to execute various function. This study aimed to investigate whether the secreted exosomes during sepsis could be grouped according to their surface markers with different cargo content and functions.

**Methods**: The blood was drawn from C57BL/6 mice in an animal model of sepsis at 16 h in the presence or absence of cecal ligation and puncture (CLP). The exosomes were isolated and grouped with Exo-Flow flowcytometry detecting their surface markers (CD9, CD31, CD44 and Rab5b) into six different subpopulations: (1) Control-exo; (2) CLP-exo; (3) CLP-exoCD9; (4) CLP-exoCD31; (5) CLP-exoCD44; (6)CLP-exoRab5b. The exosomal miRNAs of each subpopulation were detected with next-generation sequencing with validation by subsequent real-time polymerase chain reaction to identify the composition of predominant miRNAs inside the exosomes. Angiogenesis-related growth factors were quantified by multiplex ELISA. Angiogenesis as tube formation and cell migration were measured after the transfection of exosomes from different subpopulations into the primarily-cultured endothelial cells isolated from C57BL/6 aorta.

**Results**: The most predominant five exosomal miRNAs after CLP (mmu-miR-486-5p, mmu-miR-3107-5p, mmu-miR-10a-5p, mmu-miR-143-3p, mmu-miR-25-3p) and the angiogenesis-related growth factors (Angiopoietin-2, Follistatin, EGF, IL-8 and VEGF-A) were differently expressed among the CLP-exo, CLP-exoCD9, CLP-exoCD31, CLP-exoCD44 and CLP-exoRab5b. The exosomes secreted during sepsis enhanced the tube formation and cell migration of the primarily-cultured endothelial cells. However, the increased tube formation and cell migration were various among the endothelial cells transfected with exosomes as CLP-exoCD9, CLP-exoCD31, CLP-exoCD44 and CLP-exoRab5b.

**Summary/Conclusion**: The secreted exosomes with different surface markers during sepsis contain different microRNAs as well as protein content and present various ability to increase the angiogenesis of the transfected endothelial cells.

**Funding**: This study was supported by the grants [CMRPG8F1841 & CMRPG8F1842] from the Chang Gung Memorial Hospital

LBT01: Late Breaking Poster Session – Methodology Chairs: Muthuvel Jayachandran; Theresa WhitesideLocation: Exhibit Hall 17:15 - 18:30

LBT01.01

Single vesicle counting enabled by DNA nanostructures

Wenwan Zhong ^1^; Kaizhu Guo^2^; Wen Shen^1^

^1^University of California, Riverside, Riverside, USA; ^2^University, Riverside, USA

**Background**: Extracellular vesicles (EVs) could be useful for sensitive and specific cancer diagnosis and prognosis, but their identification requires detailed molecular analysis of the EVs from different sources.

**Methods**: Single vesicle counting can overcome the noise limitation in batch analysis and reveal the presence of the EVs carrying unique molecular signatures highly indicative to their specific cell of origin. Herein, we propose a simple strategy to enable single vesicle counting and detect multiple exosome cargos in individual vesicles. Our central hypothesis is that DNA nanostructures can be established upon recognition of the molecular signatures on exosomes, and enable single EV counting and EV cargo profiling.

**Results**: We have proved that DNA nanostructure (DNS) can be grown on exosome surface and enable detection of single vesicles using conventional microscope or flow cytometer. DNS is established by Hybridization Chain Reaction (HCR) upon recognition of CD63. An initiator that contains the aptamer sequence for CD63 and a stem-loop structure was designed so that binding to CD63 opened the stem for hybridization with Hairpin 1 (H1) and initiated the growth of a long dsDNA through continuous hybridization between H1 and Hairpin 2 (H2). Only CD63 or exosomes could initiate growth of long DNA products from HCR as proved by gel electrophoresis. TEM also detected particles ~500 nm in diameter after the reaction, and the mode diameter of the vesicles detected by Nanosight NS300 increased by ~50 nm. DNS enabled detection of exosomes in the conventional flow cytometer, while exosomes labelled with anti-CD63-conjugated QDs were not observed. More interestingly, the EVs carrying both CD63 and HER2 on its surface could be recognized by dual-labelling using two initiators. The exosomes produced by the breast cancer cell carry high content of HER2 and CD63, but those from the non-tumour cell line MCF-10A exhibit low HER2 and high CD63 expression. When these exosome populations were mixed at a 2 (SKBR3):1 (MCF-10A) ratio (particle concentration measured by NTA before mixing), dual TIC-DNS could clearly differentiate the presence of two groups of exosomes.

**Summary/Conclusion**: We believe our technique can help with identification of exosomes in clinical setting rapidly with low sample consumption.

**Funding**: This study was funded by NIH R01CA188991.

LBT01.02

Pilot scale and GMP compliant production in STR bioreactor and purification process of wild-type and engineered extracellular vesicles by TFF

Nasser Nassiri Koopaei^1^; Steven Pomeroy^1^; Mitch Phelps^2^; Thomas D. Schmittgen ^1^

^1^College of Pharmacy, University of Florida, Gainesville, FL, USA; ^2^College of Pharmacy, Ohio State University, Columbus, OH, USA

**Background**: Production and scale up of therapeutic extracellular vesicles (EVs) is currently limited by a lack of validated, reproducible and good manufacturing practice (GMP) compliant manufacturing processes. Culturing EV producing cells on plastic and EV purification using ultracentrifugation are inefficient processes that are not scalable.

**Methods**: We propose EVs production in stirred tank bioreactor pursued by the tangential flow filtration (TFF) method (100 KDa cut-off cassette membranes) to purify the EVs. Wild type EVs produced by HEK293T cells were cultured in suspension and on Corning enhanced attachment, Cytodex 1 and Cytodex 3 microcarriers and were purified by ultracentrifugation or TFF. The bioreactor experiments were conducted in an Eppendorf BioFlo320 in 1 and 3 l vessels equipped with a pitched blade impeller. The culture inoculums were grown and expanded in T25, T75 and then, spinner flasks. Cytodex 1 microcarriers were used to grow HEK 293T adherent cells. The suspension experiments were performed in serum free medium (SFM II), Glutamax 1X, 8% CO2 and 37°C, and for adherent cells 5% exosome depleted DMEM, 5% CO2 and 37°C. The bioreactor process parameters were adjusted and controlled during the process. Dissolved oxygen (30%), pH (7.2), temperature (37°C) and mixing rate (50 rpm) were controlled. The process continued for 5 days and then, the supernatant was harvested and processed to purify the EVs.

**Results**: Cytodex 3 showed the best cell adhesion and EV yield, while the Corning one resulted in high total protein in the sample. We have successfully cultured up to 0.5 l of the suspension cultures on Cytodex 3 microcarriers. TFF resulted in EVs with a reduced particle size and showed less particle loss relative to ultracentrifugation. Typical assays for EV validation confirmed the presence of EVs purified by TFF. The suspension process yielded 8.3×1013 EVs/L (80 nm) and continued with a fed-batch approach. The adherent cells yielded 4.1×1013 EVs/L (104 nm) following TFF process with 2.26 µg/µL protein.

**Summary/Conclusion**: Future studies include continued development and scale-up of TFF purified and bioreactor culture of both wild-type and genetically engineered EVs.

LBT01.03

Engineered Extracellular Vesicles as Therapeutic Decoys

Oscar PB Wiklander ^1^; Joel Z. Nordin^1^; Dhanu Gupta^1^; Yiqi Seow^2^; Sririam Balusu^3^; Xiu-Ming Liang^1^; Giulia Corso^1^; Ulrika Felldin^1^; Mariana Conceicao^4^; Manuela Gustafsson^1^; Roosmarijn E. Vandenbroucke^3^; Matthew JA Wood^4^; André Görgens^5^; Samir El-Andaloussi^5^

^1^Clinical Research Center, Department for Laboratory Medicine, Karolinska Institutet, Stockholm, Sweden; ^2^Molecular Engineering Laboratory, Proteos, Agency for Science, Technology and Research (A*STAR), Singapore, Singapore; ^3^VIB-UGent Center for Inflammation Research, Ghent, Belgium; ^4^Department of Physiology, Anatomy and Genetics, University of Oxford, Oxford, UK; ^5^Clinical Research Center, Department for Laboratory Medicine, Karolinska Institutet, Stockholm, Sweden, Hälsovägen, Sweden

**Background**: Extracellular vesicles (EVs) are being increasingly explored as therapeutic entities, both as natural delivery vectors and in their own right, as improved cell-based therapies. Here we introduce a novel concept of using mesenchymal stromal cell (MSCs)-derived EVs that are engineered to display sequestering receptors against inflammatory cytokines, here referred to as decoy EVs. The aim was to exploit EVs to convey the immunomodulatory effects of MSCs combined with their capacity as natural macromolecular messengers. Furthermore, by displaying functionalized receptors, being devoid of intracellular signaling domains, decoy EVs can target the inflammatory cytokine axis without affecting pathways important for homeostasis.

**Methods**: Cells were engineered to express optimized constructs encoding for either IL-6ST, a receptor for IL-6-IL-6R complexes targeting IL-6 trans-signaling, or TNFR1, which targets TNF-alpha, both fused to an EV-sorting moiety. The engineered decoy EVs, subsequently isolated from conditioned media, were evaluated using reporter cell lines, stimulated by either IL-6-IL-6R complexes or TNF-alpha with a luminescent or fluorescent reporter read-out for the respective cytokine. The therapeutic potential of decoy EVs were further evaluated *in vivo*, in three different inflammatory mouse models.

**Results**: *In vitro*, the results demonstrated dose-dependent inhibition of decoy EVs on respective cytokine pathways. Next, the effects of decoy EVs *in vivo* were evaluated in a TNBS-colitis model and a LPS-induced systemic inflammation mouse model, showing protective effects with increased survival and decreased weight loss. To further assess the potential of decoy EVs on inhibiting pro-inflammatory pathways, decoy EVs were evaluated in a multiple sclerosis model, experimental autoimmune encephalomyelitis (EAE). Mice induced with EAE and treated with decoy EVs displayed significantly milder symptoms when compared to mock control treatment.

**Summary/Conclusion**: In conclusion, by combining the beneficial effects of stem cell therapies and protein therapeutics, engineered decoy EVs may have great potential to be the next generation of biotherapeutics.

LBT01.04

Development of a standardized exosome production process for clinical use

Sílvia C. Rodrigues; Renato Cardoso; Filipe Duarte; Cláudia O. Gomes; Joana Simões Correia

Exogenus Therapeutics, SA, Cantanhede, Portugal

**Background**: Exogenus Therapeutics is developing new therapeutic tools for the treatment of skin diseases, based on exosomes secreted by umbilical cord blood (UCB) cells. Ensuring manufacturing of clinical-grade vesicles under GMP, while increase homogeneity and scalability of the product batches, is a major challenge in the field of EV-inspired therapeutics.

**Methods**: We have implemented several changes to the manufacturing workflow of our lead product Exo-101 namely integration of Automatic UCB Processing (AP), implementation of an exosome purification strategy based on Ultrafiltration and Chromatography (UF-SEC), combined with pooling from different donors. We evaluated the impact of these alterations by validating the biophysical and biomolecular characteristics of Exo-101 (by NTA, TEM, flow cytometry and qPCR). The therapeutic potential was confirmed on a delayed wound healing mice model.

**Results**: We demonstrate that independently of using manual (MP) or automatic (AP) UCB processing, the purified exosomes are very similar in size (MP~150.2±4.0 nm and AP~152.4±6.5 nm), particularly enriched in particles with 50–200 nm (>75%), and express classical and non-classical markers such as CD9, CD63 and CD15. The UF-SEC based manufacturing method, combined with donors’ pooling, leads to higher Exo-101 yield. Importantly, this GMP-compliant version of Exo-101 has improved regenerative potential, enhancing the acceleration of wound closure as from day 3, leading to 20% improvement at day 10.

**Summary/Conclusion**: We have been successful in optimizing a standardized GMP-compliant process for the production of clinical-grade exosomes. With this expertise, Exogenus Therapeutics is in a privileged position to support other companies and research groups in transforming R&D-based purification processes into controlled manufacturing of exosomes for clinical use.

**Funding**: This work was co-funded by Centro 2020 - Regional Operational Program, Portugal 2020 and European Union through FEDER.

LBT01.05

Influence of storage condition on size and concentration of exosomes derived from canine oviduct cells and their uptake in cumulus cells in a time dependent manner

Seok Hee Lee; Hyun Ju Oh; Min Jung Kim; Ki Hae Ra; Dimas Arya Abdillah; Jin Wook Kim; Byeong Chun Lee

Seoul National University, Seoul, Republic of Korea

**Background**: It has been suggested that communication between oviduct cells and oocytes existed in co-culture system, and cumulus oocyte complexes (COCs) exposed to the oviductal secretions effectively improved oocyte competence. In particular, exosomes, a smallest extracellular vesicle, can easily undergo endocytosis by mediating cellular communication. However, no studies have been performed to evaluate the influence of storage condition on oviductal exosome to broaden its application. We investigate whether oviductal exosomes exhibit a stability following the storage condition, and whether cumulus cells exhibit an exosome uptake

**Methods**: The oviduct cells were collected by flushing in estrus stage dog and exosomes were obtained from oviduct cell culture medium using exosome extraction kit. To evaluate the stability of exosomes with different storage condition, exosomes were divided into three groups (Fresh, -4°C, and -20°C). The exosomes from -4°C, and -20°C have been stored for 1 week. The nanoparticle tracking analysis was performed to evaluate the size/concentration of exosomes. For exosome uptake analysis, cumulus cells from COCs were cultured and labeled with PKH26 was seeded in medium. After 24 h, oviductal exosomes labeled with PKH67 were added. The exosome uptake was observed by microscopy in different time (4, 12 and 24 h).

**Results**: There are no significant differences with regard to size/concentration of exosomes in different storage conditions (Fresh: 189.0 ± 2.1 mm, 4.9 ± 0.1 × 10^8^/ml, -4 °C: 186.3 ± 3.0 mm, 5.6 ± 0.2 × 10^8^/ml and -20 °C: 185.7 ± 0.7, 5.1 ± 0.5 × 10^8^/ml). The uptake of labeled oviductal exosomes by cumulus cells was significantly increased in 24 h incubation compared with 4 and 12 h groups. However, there was no significant difference in exosome uptake between 4 and 12 h incubation groups.

**Summary/Conclusion**: The storage conditions of oviductal exosomes don’t give any influence on exosome size/concentration. Also, regardless of different storage condition, all groups of exosomes are effectively uptaken by cumulus cells after 24 h incubation, which suggest that functional studies in intercellular communication by oviductal exosomes would provide conclusive evidence in canine oocyte and embryo development.

**Funding**: This work was funded by NRF [20142A1021187], Korea IPET [#316002-05-3-SB010], Research Institute for Veterinary Science and the BK21 plus program.

LBT01.06

Quantification and phenotyping of EVs by HPLC-size exclusion chromatography with on-line fluorescence detection

Diana Kitka; Veronika Kudar Hegedusne; Robert Deak; Judith Mihaly; Zoltan Varga

Biological Nanochemistry Research Group, Institute of Materials and Environmental Chemistry, Research Centre for Natural Sciences, Hungarian Academy of Sciences, Budapest, Hungary

**Background**: Size exclusion chromatography (SEC) is a powerful tool for the isolation of extracellular vesicles (EVs) from various body fluids. However the advantages of HPLC-SEC in the quantification and phenotyping of EVs is not yet utilized. The objective of this study is to demonstrate for the first time that HPLC-SEC with on-line fluorescence detection can be used for the quantitative analysis of various EV preparations.

**Methods**: EV samples were prepared from platelet free plasma (PFP EVs) and from red blood cell concentrate (REVs), and were thoroughly characterized by flow cytometry, TEM, DLS and infrared spectroscopy. Wheat germ agglutinin (WGA), Alexa Fluor 647 Conjugate, was used as a general glycoprotein/membrane label, and FITC conjugated anti-human CD235A was used for labeling REVs. HPLC-SEC measurements were performed using a 200 mm x 5 mm glass column filled with Sepharose CL-2B cross linked agarose gel and with a JASCO PU-2089 pump supplemented with an FP-4020 fluorescence detector.

**Results**: Sepharose CL-2B gel is capable of separating EVs from soluble proteins and lipoprotein particles, which is also demonstrated in our HPLC-SEC measurements on PFP EVs and REVs. Due to these characteristics, removing the unbound WGA and anti-CD235a markers prior to the HPLC-SEC measurement was not necessary. With other words, the fluorescence chromatograms directly provide the labeling efficiency of the used markers. This enabled the quantification of EV bound markers by taking into account the initial concentration of the labels. EV concentrations corresponding to as low as 1 ng of WGA and 10 ng of CD235a were measured by the proposed technique.

**Summary/Conclusion**: This study provides the proof-of-concept of using online fluorescence detection in HPLC-SEC, which serves as a fast, sensitive and specific technique for the characterization of EV preparations. The use of WGA as a general membrane marker provides a sensitive way for the detection of EVs, whereas specific fluorescent antibody conjugates - such as CD235a in our case - can be used for phenotyping of EVs from different origin.

**Funding**: This work was supported by the National Research, Development and Innovation Office (Hungary) under grant numbers [PD 121326 and NVKP_16-1-2016-0007]. ZV was supported by the Janos Bolyai Research Fellowship.

LBT01.07

Phenotyping of EVs by multiwavelength fluorescence nanoparticle tracking Analysis

Clemens Helmbrecht

Particle Metrix GmbH, Inning, Germany

**Background**: Nanoparticle tracking analysis (NTA) of bionanoparticles, such as EVs, vesicles or liposomes, is an efficient technique for quantification of size and total concentration. With fluorescence detection option, F-NTA allows the specific quantification of subpopulations of biomarkers on single particle level. Traditionally, samples are analysed applying only one laser wavelength. For the first time, we show phenotyping of EVs by a NTA instrument equipped with two laser sources, 405 nm and 488 nm, allowing rapid analysis of biomarker concentration or ratios.

**Methods**: EVs were derived from cell line and plasma respectively and isolated and purified by ultracentrifugation, tangential flow filtration or size exclusion chromatography. For the determination of vesicle content, protocols for several plasma membrane dyes were developed and optimized for NTA detection. Several antibodies were evaluated for EV characterization and protocols were optimized for NTA detection.

**Results**: Switching between scatter and fluorescence mode allows quantification of vesicle content. The efficiency depending on protocol and dye such as PKH67, DiO and CMG are compared. Effect of bleaching was minimized due to fast acquisition. Several fluorescently labeled antibodies for detection of CD63, CD81 and CD9 have been evaluated. Total concentration as well as biomarker ratios are presented as function of origin and purification of EVs.

**Summary/Conclusion**: Phenotyping of EVs derived from cell line and plasma was performed by multiwavelength NTA applying 405 nm and 488 nm for excitation. Alignment-free switching between excitation wavelengths allows quantification of biomarker ratios on the same sample within minutes reducing measurement time and precious sample amount.

LBT01.08

Low-density lipoprotein associates with extracellular vesicles via apolipoprotein B

Barbara W Sodar ^1^; Krisztina Pálóczi^1^; Tamás Visnovitz^1^; Krisztina V Vukman^1^; Éva Pállinger^1^; Árpád Kovács^1^; Eszter Á Tóth^1^; Hargita Hegyesi^1^; Ágnes Kittel^2^; Sára Tóth^1^; Edit Buzas^1^

^1^Department of Genetics, Cell and Immunobiology, Semmelweis University, Budapest, Hungary; ^2^Institute of Experimental Medicine, Hungarian Academy of Sciences, Budapest, Hungary

**Background**: We have shown recently that low-density lipoprotein (LDL) co-isolates with extracellular vesicles (EVs) derived from blood plasma and the supernatant of platelet concentrates. Furthermore, we found that with current isolation protocols, EVs and LDL cannot be separated. By transmission electron microscopy we also demonstrated the association of EVs with LDL *in vitro*.

**Methods**: We labeled THP-1 human monocytic leukemia cells with the lipophilic dyes PKH67 and DiI. After labeling, small (d < 200 nm) and medium sized (d: ~ 200–800 nm) EVs were isolated by differential centrifugation and gravity-driven filtration from the supernatant. To exclude the possible effect of bovine lipoproteins, we used a 24 h serum free incubation for EV production. Sulfate-aldehyde latex beads were coated with native, oxidized and acetylated LDLs as well as with purified native apolipoproteins (apoA1, apoB, apoC2 and apoE). After blocking with BSA and glycin, fluorescently labeled EVs were incubated with the beads. Fluorescence of the beads resulting from that of the attached EVs, was analysed by flow cytometry. EV adhesion to different coatings was compared both to the bare and to the blocked-only beads.

**Results**: Both small and medium sized EVs showed significant adhesion to apoB (*p* < 0.05). There was no difference between the signals of small and medium EVs. We also observed adhesion to native, oxidized and acetylated LDLs, apoA1 and apoC2. However, in the case of apoE, no binding was detected.

**Summary/Conclusion**: The interaction between LDL and EVs might be mediated by the apolipoprotein B component of LDL.

**Funding**: This work was supported by: National Research, Development and Innovation Office NKFIH, Hungary [OTKA11958, OTKA120237, NVKP_16-1-2016-0017], Ministry for National Economy of Hungary [VEKOP-2.3.2-16-2016-00002 and VEKOP-2.3.3¬15¬2016¬00016].

LBT01.10

Comparative analyses of exosome isolation methods from distinct biofluids

Tânia Soares Martins ^1^; José Catita^2^; Ilka Martins Rosa^1^; Odete A. B. da Cruz e Silva^1^; Ana Gabriela Henriques^1^

^1^iBiMED - Institute of Biomedicine, Aveiro, Portugal; ^2^Paralab SA, Gondomar, Gondomar, Portugal

**Background**: Exosomes are present in various body fluids and can cross blood-brain barrier, which enhances their potential as drug-delivery targets but also as diagnostic tools. Indeed, these nanovesicles can be a resource for proteomic, lipidomic and genetic biomarkers. However, exosome isolation from different biofluids is a challenge. Differential ultracentrifugation is the most commonly used method although it is laborious and not adequate for large-scale clinical studies; thus alternative methods are urgently needed. Other methodologies have been addressed, but additional studies should be carried out to identify the best approach in terms of exosome recovery and purity, especially for cerebrospinal fluid (CSF).

**Methods**: Herein, two commercial precipitation-based methods and one column-based method were compared for exosome isolation from human serum, plasma and CSF. Characterization included morphological analysis by transmission electron microscopy (TEM), exosome yield determination by nanoparticle tracking analysis (NTA) and colorimetric assay, exosome stability by eletrophoretic light scattering, proteomic and purity analysis.

**Results**: In general, the three methodologies isolated vesicles within the expected size range (30–150 nm) with spherical shape, as confirmed by NTA and TEM analysis, although the highest exosome yield and purity were obtained using the column-based method. Regarding exosome stability no significant differences were observed for the biofluids using the different extraction methods, but in comparative terms CSF-derived exosomes were more stable in solution.

**Summary/Conclusion**: The work herein presented aids in the characterization of exosome isolation methods, suggesting that these can be applied as quick and reliable alternatives for exosome purification from distinct and reduced biofluids volumes. This will be of significance in particular to advance clinical research on exosomal biomarker discovery and therapeutics fields.

**Funding**: This work was funded by PTDC/DTPPIC/5587/2014, Instituto de Biomedicina (iBiMED)-UID/BIM/04501/2013, Fundação para a Ciência e Tecnologia (FCT) of the Ministério da Educação e Ciência, COMPETE program, QREN, European Union (Fundo Europeu de Desenvolvimento Regional).

LBT01.11

Evaluation of usefulness of the mini-SEC method for purification of exosomes for mass spectrometry proteomic studies

Mateusz Smolarz ^1^; Agata Włosowicz^1^; Agata Abramowicz^1^; Lukasz Marczak^2^; Piotr Widlak^1^; Monika Pietrowska^1^

^1^Maria Sklodowska-Curie Institute - Oncology Center, Gliwice Branch, Gliwice, Poland; ^2^Institute of Bioorganic Chemistry, Polish Academy of Sciences, Poznan, Poland

**Background**: Biological properties of exosomes in the context of cancer development and progression are the subject of numerous scientific studies. Exosomes can be isolated from various types of biological material, e.g. blood and its derivatives, urine, saliva, cerebrospinal fluid, as well as from a culture medium for different cell lines. An important issue in conducting research on exosomes is their isolation from a research material. Techniques of exosome isolation and purification are the basis for a good sample preparation for mass spectrometry analyses. Mini-SEC technique separates solution components in terms of their mass. Therefore, exosomes get purified from proteins derived from the material they are isolated from.

**Methods**: We used four isolation variants and two types of research material: (1) healthy donor serum and (2) medium from a cell culture (FaDu cell line). In addition, as a negative control, commercial exosome-free serum was used. The prepared material (serum or concentrated medium) was loaded onto columns and fractionated in terms of size from high to low mass component. The presence of exosomes was evaluated using transmission electron microscopy (TEM) and western blot. For all fractions, MS analysis was performed for each of the conducted isolations.

**Results**: In the fractionated mini-SEC preparations we detected the presence of exosomes using frequently used exosome markers. However, we did not detect proteins that constitute their content (e.g. GAPDH, actin) in fractions containing exosomes. We determined that some proteins present in databases (e.g. ExoCarta) as of exosomal origin are in fact derived from the material the exosomes are isolated from.

**Summary/Conclusion**: The use of mini-SEC technique removed contamination with high-abundant proteins present in a sample (serum, plasma or cell culture medium), and also increased the number of exosomal protein identifications in a sample.

**Funding**: This work was supported by National Science Centre, grants no. [2013/11/B/NZ7/01512 and 2016/22/M/NZ5/00667].

LBT01.13 = OWP2.09

Isolation of extracellular vesicle-associated small RNA from canine mitral valve interstitial cells using ultracentrifugation and tangential flow filtration with size exclusion chromatography

Vicky Yang; Dawn Meola; Kristen Thane; Andrew Hoffman

Tufts University Cummings School of Veterinary Medicine, North Grafton, MA, USA

**Background**: Myxomatous mitral valve disease is a highly prevalent canine cardiac disease that can lead to congestive heart failure. Histologic changes in the valves include greater prevalence of valvular interstitial cells (VIC) with myofibroblastic phenotype. These changes and the functional consequences are virtually identical to mitral valve prolapse in people. Our previously published work shows that, compared to VIC harvested from normal mitral valves, VIC from diseased valves had decreased cellular expression of let-7c, miR-17, miR-20a, and miR-30d. However, the miRNA content of extracellular vesicles (EVs) from normal and diseased VIC have not yet been analyzed.

**Methods**: VIC were isolated by enzymatic digestion from normal and diseased valves (n = 5/group). Passage 2 VIC were cultured in defined chemical media, and the conditioned media was collected every 24 hrs for 3 days. EV were then isolated using ultracentrifugation (UC) (300 ×*g*, 10 min; 2000 ×*g*, 10 min; 10,000 ×*g*, 30 min; 100,000 ×*g*, 70 min) followed by size exclusion chromatography (HPLC), or using tangential flow filtration (TFF) (100,000Da MWCO PES filters) followed by HPLC. EV was further characterized using nanoparticle tracking analysis, TEM, and western blot for CD9 and TSG101. RNA from VIC were isolated using the mirVana miRNA isolation kit and from EV using the Qiagen miReasy kit. Isolated RNA concentrations were determined by the Agilent Bioanalyzer.

**Results**: HPLC showed a single peak corresponding to the EV fraction for samples first processed by UC, whereas those first processed by TFF showed two distinct peaks (F1 and F2 fractions). Average total particle yield was higher by TFF + HPLC vs. UC+HPLC (7.8×10^9^ ± 7.3×10^9^ vs. 1.5×10^9^ ± 6.0×10^8^), with 74% of the TFF + HPLC particles residing in the F1 vs. F2 fraction. TFF + HPLC yielded on average more small RNA than UC+HPLC (9.4 ± 7.4 ug/ul vs. 6.3 ± 10.1 ug/ul), with 59% of the total RNA residing in the F1 fraction. Western blot showed that F1 EV was positive for TSG101 while F2 EV was not.

**Summary/Conclusion**: Compared to UC+HPLC, TFF + HPLC yielded higher RNA concentrations and was able to separate two different EV populations. The miRNA content of the two EV fractions and of the VICs will be further analysed by RNA sequencing to better understand the miRNA expression differences between the cellular and EV populations.

**Funding**: The work was funded by Shipley Foundation

LBT01.14

Characterization of EVs isolated from differently processed bovine milk

Marije Kleinjan ^1^; Sten F.H.M. Libregts^1^; Anouk Feitsma^2^; Joost van Neerven^2^; Marca H.M. Wauben^1^

^1^Department of Biochemistry and Cell Biology, Faculty of Veterinary Medicine, Utrecht University, Utrecht, The Netherlands, Utrecht

**Background**: We investigated how processing of bovine milk affected the EV quantity and composition by isolating EVs from homogenized, pasteurized or ultra-heat-treated (UHT) milk and comparing these EVs to raw bovine-milk-derived EVs.

**Methods**: EVs from differently processed bovine milk were isolated using differential centrifugation followed by sucrose density gradient centrifugation. Density gradient fractions 4–6, 7–9 and 10–12 were pooled and analysed using high-resolution flow cytometry, cryo EM and western blot. Small RNA from EV containing fractions was isolated and concentrations small RNA were determined by Bioanalyzer.

**Results**: The quantity of EVs as measured by high-resolution flow cytometry is not affected in pasteurized milk when compared to raw milk. However, homogenization and pasteurization resulted in a strong reduction of EVs in fraction 7–9. In UHT milk, the amount of EVs was drastically reduced. These results were confirmed by cryo EM. Western blotting showed that the general EV markers CD9 and CD63 were most prominent in fraction 7–9 of all kinds of milk, except for UHT-treated milk where no protein signals could be detected by western blotting. Remarkably, in raw milk, MHCI and MHCII were detected in fraction 7–9, whereas these markers were detected mostly in fraction 4–6 after pasteurization. This could indicate that MHCI/II-positive EV populations were lost or damaged during milk processing. After pasteurization, a clear loss of small RNA cargo was seen in fraction 7–9, but not in fraction 4–6. Furthermore, homogenization of milk clearly affected the distribution of MFG-E8 through the gradient.

**Summary/conclusion**: Processing of milk affects the EV population. Depending on the type of processing, different effects on the total EV population or on EV subsets were observed. Although no clear effects on total EV numbers were observed after pasteurization, the total RNA yield was reduced and the EV integrity was probably affected (shift in buoyant density based on distribution of MHCI/II and miRNAs). Homogenization most likely affected mainly the MFG membranes in milk while UHT treatment had the most detrimental effect on EVs.

**Funding**: The research is performed under a CRA between FrieslandCampina and Utrecht University.

LPT01.15 = OWP2.08

Free flow electrophoresis allows preparation of extracellular vesicles fractions with high recovery and purity rates

Gerhard Weber^1^; Simon Staubach^2^; Christian Reiter ^1^; Bernd Giebel^2^

^1^FFE Service GmbH, Feldkirchen, Germany; ^2^Institute for Transfusion Medicine, University Hospital Essen, Essen, Germany

**Background**: Free flow electrophoresis (FFE) is a well established (micro)preparative method to separate analytes with inherent difference of charge density and/or difference of pI-value. Run with media of different pH-values (pH = 8 – pH = 4.8), FFE has classically been optimized to effectively separate amphoteric analytes, like proteins and peptides, from non-amphoteric analytes, like lipid vesicles, DNA and RNA.

**Methods**: According to the need to isolate pure extracellular vesicles (EVs) especially from plasma samples, we took the challenge and optimized the FFE for the EV purification, either as a stand alone method or in combination with a second separation method, the size exclusion chromatography (SEC), being performed before FFE. Obtained FFE-fractions (48 per run) were analysed for their protein content (fluorescence 280–350 nm), the sizes of their proteins (SDS PAGE, Coomassie and silver staining) and for the presence of EV markers in dot blot and Western blot analyses.

**Results**: Initially, EV markers were recovered in FFE fractions which also contained high concentrations of non-EV associated proteins such as albumin. By changing several parameters, we have optimized FFE and maximized the sample throughput at a minimum dilution, both in a continuous and in an interval mode.

Now, the largest content of negatively charged EVs from plasma and serum samples can be enriched in 1–3 fractions. These fractions are diluted 1:3 only and contain less than 1% of total sample protein. Coomassie staining of SDS PAGEs confirmed that their protein profile differ from that of EV-free FFE fractions. Particles within the EV fractions could be quantified by nanoparticle tracking analysis (NTA) without prior concentration. The true EV-nature of the harvested particles was confirmed by western blot analysis. Of note, maybe due to the high heterogeneity of EVs in given samples, a minor proportion of vesicles have been detected in other FFE fractions, which will be characterized in the future. Now, with the improved continuous separation protocol two plasma or serum samples can be processed in parallel at a throughput of 5 mL per hour each

**Summary/Conclusion**: In summary, FFE provides a powerful method, to purify and fractionize EVs from plasma and serum samples as well as from other liquids. If required it can be combined with other EV processing technologies like SEC.

LBT02: Late Breaking Poster Session - Cancer I Chairs: Chiara Ciardiello; Pilar SepulvedaLocation: Exhibit Hall 17:15 - 18:30

LBT02.01

Role of exosomal Connexin43 in melanoma progression

Adrián Varela. Vázquez ^1^; Amanda Guitián-Caamaño^2^; Alejandro Castro-Iglesias^2^; Tamara Camino-Martínez^3^; Susana Belén Bravo-López^4^; María Pardo^5^; Eduardo Fonseca^2^; María D. Mayán^2^

^1^Translational Research in Cell Communication and Signaling (CellCOM). Instituto de Investigación Biomédica de A Coruña (INIBIC). CH-Universitario A Coruña (XXIAC). Servizo Galego de Saúde (SERGAS). Universidade da Coruña. Xubias de Arriba, 84 15006 A Coruña, Spain., A Coruña, Spain; ^2^CellCOM research group. Instituto de Investigación Biomédica de A Coruña (INIBIC). Servizo Galego de Saúde (SERGAS). Universidade da Coruña (UDC). A Coruña, Spain, A Coruña, Spain; ^3^Obedisomic group, Instituto de Investigación Sanitaria de Santiago de Compostela (IDIS), Complexo Hospitalario Universitario de Santiago de Compostela (CHUS). Universidade de Santiago de Compostela (USC). Santiago de Compostela, Spain, Santiago de Compostela, Spain; ^4^Proteomics laboratory. Instituto de Investigación Sanitaria de Santiago de Compostela (IDIS), Complexo Hospitalario Universitario de Santiago de Compostela (CHUS). Universidade de Santiago de Compostela (USC). Santiago de Compostela, Spain, Santiago de Compostela, Spain; ^5^Grupo Obesidómica. Instituto de Investigación Sanitaria de Santiago de Compostela (IDIS). Complejo Hospitalario Universitario de Santiago. Travesía da Choupana s/n. 15706, Santiago de Compostela, Spain, Santiago de Compostela, Spain

**Background**: Loss of gap junction (GJ) intercellular communication (GJIC) and/or downregulation of connexins (Cxs) have been reported in different cancer cell lines as well as in tissues of many tumour types including melanoma. Cxs have been described as tumour suppressors in earlier stages of melanoma. However during tumour cell invasion and metastasis their role is a matter of some controversy. Extracellular vesicles (EVs) and exosomes released by cells participate in cell communication and can be involved in tumour progression. The transmembrane protein connexin43 (Cx43) was found in exosomas and participate in the transfer of information to the target cell though GJs.

**Methods**: Ectopic expresión of Cx43 was performed using vectors and electroporation. Protein levels and cellular sublocalization were studied by western blot and immunofluorescence. Exchange of lucifer yellow was used to check GJIC. Exosomes were isolated by ultracentrifugation and analysed using the NanoSight instrument and electron microscopy. The protein content was analysed by LC-MS/MS using a 6600 triple Tof.

**Results**: Exosomes were eficiently isolated from human melanoma cells lines, however Cx43 was only present in exosomes derived from the melanoma cells that overexpressed Cx43 (A375Ma2-Cx43). When different melanoma cell lines were exposed to exosomal Cx43, these vesicles decreased cell proliferation and blocked colonies grown. The analysis of the protein content revealed 464 proteins exclusively present in exosomes positive for Cx43 compared to exosomes without Cx43, isolated from melanoma cell lines. Several of identified proteins are related with regulation of apoptosis such as APAF-1. We also identified proteins that regulate p53 expression, the CDKN2A anti-proliferative activity and the EGFR signaling pathway.

**Summary/Conclusion**: Our results indicate that exosomal Cx43 through its scaffolding function could be involved in the recruitment of proteins and other compounds to the exosomes switching the role of these EVs in melanoma. Further understanding of the role of Cx43 in the exosomes will have implications for the development of new therapeutic strategies as drug carries and delivery vehicles to combat metastasis in melanoma.

LBT02.02

Ha-RasV12 induced augmented secretion of Wnt5a and EpCAM containing exosome in MDCK cells

Hsi Hui Lin; Ming Jer Tang

Department of Physiology, National Cheng Kung University Medical College, Tainan, Taiwan (Republic of China)

**Background**: We have previous demonstrated that Ha-Ras V12 overexpressing cells develop a specific mechanical phenotype which includes cell softening and loss of stiffness sensing. However, the molecular mechanism whereby Ha-Ras V12 overexpression induces cell transformation and the mechanical phenotype has not been explored before.

**Methods**: We employed MK4 cells, MDCK cells harboring inducible Ha-RasV12 expression to test whether exosome isolated from conditioned media of Ha-RasV12-overexpressed MK4 cells induced cell softening, loss of stiffness sensing, and increase in migration and invasion ability. Using atomic force microscope and nanoparticle tracking analysis, we investigate if Ha-RasV12 overexpression induces augmentation of exosome secretion.

**Results**: We demonstrated that exosome isolated from conditioned media of Ha-RasV12-overexpressed MK4 cells induced cell softening, loss of stiffness sensing, and increase in migration and invasion ability only in Cav1-knockdown MDCK cells. Using atomic force microscope and nanoparticle tracking analysis, here we demonstrated that Ha-RasV12 overexpression induced significant augmentation of exosome secretion, which can be blocked by U0126, a MAPK inhibitor. In addition, the levels of Wnt5a and EpCAM were markedly enhanced in conditioned media of Ha-RasV12 overexpressing cells. Both Wnt5a siRNA and C59 (a porcupine (O-acyltransferase) inhibitor) inhibited Ha-RasV12-induced cell softening and transformation. Cav1 downregulation either by Ha-RasV12 or by targeted shRNA, increased Fzd2 protein levels without affecting its mRNA levels, suggesting a novel role of Cav1 in inversely regulating Fzd2 expression. Therefore, the anti-transformation of Cav1-overexpressing MK4 cells is probably due to the Cav1-dependent repression of Fzd2, which hindered Ha-RasV12-Wnt5a-Stat3 pathway.

**Summary/Conclusion**: In summary, our results showed that enhanced secretion of Wnt5a containing exosome is indispensible for Ha-RasV12-driven cellular and mechanical transformation. However, the function of EpCAM in exosome remains to be investigated.

LBT02.03

Tumourigenic capacity of exosomes isolated from TNBC cells

Patricia M. M. Ozawa ^1^; Faris Alkhilaiwi^2^; Danielle M. Ferreira^1^; Enilze M. S. F. Ribeiro^1^; Luciane R. Cavalli^2^

^1^Department of Genetics, Universidade Federal do Paraná, Curitiba, Brazil; ^2^Lombardi Comprehensive Cancer Center, Georgetown University, Washington, DC, USA

**Background**: Exosomes are extracellular vesicles of endocytic origin that are present in body fluids and known to play key roles in intercellular signaling communication. Several studies showed the importance of exosomes in cancer processes, like angiogenesis, cell migration, invasion and drug resistance. Triple negative breast cancer (TNBC) is a clinically aggressive subtype of breast cancer, associated with treatment resistance, recurrence and high mortality rates. Therefore, studies that aim to elucidate the TNBC pathogenic mechanisms’ are critical to increase the knowledge of their biology and future clinical translation. In this study we accessed the tumourigenic capacity of exosomes isolated from TNBC cells in cell proliferation.

**Methods**: Exosomes isolated from HCC1806 cell line (from culture media containing exosome-free FBS) were co-cultured with a non-tumourigenic epithelial cell line (MCF-10A), with cell proliferation measured by MTS assay. Western blotting for CD9 and CD63 were performed to confirm exosome isolation and an uptake labeled-based assay confirmed the exosomes uptake.

**Results**: A significant increase in cell proliferation was observed when MCF-10A cells were treated with different concentrations of HCC1806 exosomes (HCC-exo), but interestingly, not when treated with exosomes from MCF-10A and MCF-7 cell lines. To determine the potential genes and mechanisms that may be affected in the HCC-exo cells, we conducted a multiplexed cancer progression analysis, using the nCounter PanCancer Progression Panel. A number of 262 genes (out of 770 genes) were significantly differentially expressed among the parental HCC1806 and the HCC-exo cells; these included 123 genes associated with tumour growth, 100 with angiogenesis, 91 with the EMT pathway, 87 with invasion and 20 with metastasis. Some of the genes overexpressed on the HCC-exo cells were the PIK3R2, SRC and MMP9 genes.

**Summary/Conclusion**: These preliminary results showed that exosomes from a highly tumourigenic TNBC cell can significantly induce proliferation in non-tumoural cells *in vitro*, possibly by the regulation of key cancer driver genes. Further functional studies, in exosomes isolated from other TNBC cell lines are required to validate our initial findings and to understand the full tumourigenic potential of exosomes in cancer.

**Funding**: This work was funded by CAPES: PDSE - 88881.136048/2017-01

LBT02.04

Plasma exosome count is correlated with grade of lung cancer stages; comparison between pulmonary vein versus peripheral vein

Byeong Hyeon Choi ^1^; Yu Hua Quan^2^; Jiyun Rho^2^; Sunghoi Hong^3^; Yong Park^4^; Yeonho Choi^5^; Ji-Ho Park^6^; Hwan Seok Young^7^; Kook Nam Han^2^; Young Ho Choi^2^; Hyun Koo Kim^2^

^1^Department of Biomedical Sciences, College of Medicine, Korea University, Seoul, Republic of Korea; ^2^Department of Thoracic and Cardiovascular Surgery, Korea University Guro Hospital, College of Medicine, Korea University, Seoul, Republic of Korea; ^3^School of Biosystem and Biomedical Science, College of Health Science, Korea University, Seoul, Republic of Korea; ^4^Korea University School of Medicine, Seoul, Republic of Korea; ^5^School of Biomedical Engineering, Korea University, Seoul, Republic of Korea; ^6^Department of Bio and Brain Engineering, Korea Advanced Institute of Science and Technology, Daejeon, Republic of Korea; ^7^Department of Radiology, Korea University Guro Hospital, College of Medicine, Korea University, Seoul, Republic of Korea

**Background**: Lung cancer-derived exosomes are spill out of primary cancer and circulate in the peripheral blood after passing through the pulmonary drainage vein. But, there are no studies that performed quantitative comparisons of exosome using peripheral venous (PP) blood and pulmonary venous (PV) in each pathological stage of lung cancer patients. We explored the relationship between stage of patients undergoing surgery and exosomes according to the blood sampling site.

**Methods**: Five rabbits were used for both normal and lung cancer model. The cancer model was constructed using in a non-invasive manner using real-time CT fluoroscopy-guided VX2 injection in rabbit lung. Five normal and 35 lung cancer patients were included, who underwent lung cancer surgery. Preclinical blood was collected from a PP in ear vein and PV. For patient, 3 ml of blood was collected from the lobar PV of the primary lung cancer site, and PP-blood from median cubital vein during surgery. Quantitative analysis was performed by nanoparticle tracking assay, CD63 enzyme-linked immunosorbent assay, and western blotting.

**Results**: In preclinical, lung cancer lesion was confirmed by PET/CT image 2 weeks after injection, and solitary nodule was well formed. Exosome-count was no significant difference between PP and PV exosomes in normal (*p* = 0.8), However, it was increased in PP of lung cancer compared to normal (*p* = 0.012), and more increased in PV of cancer model (*p* = 0.0012). In patients, exosome counts and CD63 in PP were increased than control (*p* < 0.0001), and more significantly increased in PV than PP of patients (*p* < 0.0001). We investigated whether the increase of exosomes is related to stage and tumour size, and statistically that PV is more closely related than PP (*p* < 0.0001).

**Summary/Conclusion**: We first compared PV and PP-derived exosomes in lung cancer patients and found that they were correlated with the stage. These results suggest that PV exosomes may provide more accurate results than conventional pathologic tests in patients undergoing lung cancer surgery.

**Funding**: This research was supported by a Korea Health Technology R&D Project through the Korea Health Industry Development Institute (KHIDI), funded by the Ministry of Health & Welfare, Republic of Korea [HR14C0007].

LBT02.06

Plasma EV phenotype modulation in metastatic renal cell cancer patients receiving tyrosine kinase inhibitor

Eriomina Shahaj^1^; Chiara Camisaschi^2^; Elena Verzoni^3^; Luca Lalli^2^; Agata Cova^2^; Paola Squarcina^2^; Simona Ferro^2^; Luana Lugini^4^; Veronica Huber ^2^

^1^Fondazione IRCCS Istituto Nazionale dei Tumori, Milan, Italy; ^2^Unit of Immunotherapy of Human Tumors, Fondazione IRCCS Istituto Nazionale dei Tumori, Milan, Italy; ^3^Medical Oncology, Fondazione IRCCS Istituto Nazionale dei Tumori, Milan, Italy; ^4^Department of Oncology and Molecular Medicine, National Institute of Health, Rome, Italy

**Background**: Plasma EVs, a heterogeneous population of vesicles with different origins, have attracted major interest as biomarker source, especially in cancer patients. Besides containing those deriving from tumour cells, the composition and phenotype of plasma EVs might reflect immune status and its modulation in relation to anti-cancer agents. Here we investigated if the EV phenotype associated with changes in routine blood tests and peripheral blood immunophenotype in metastatic renal cell cancer patients (mRCC) undergoing tyrosine kinase inhibitor (TKI) therapy.

**Methods**: After approval by the internal ethical committee, PBMCs and plasma samples were collected from consenting patients at baseline, 3 and 6 months during therapy and stored in liquid N2 and -80°C, respectively. EVs, isolated by two-step differential centrifugation, were evaluated by flow cytometry and western blot. PBMC immunomonitoring was performed by 10-colour cytofluorimetry.

**Results**: EVs contained in F1 (16,500 g) and F2 (118,000 g) expressed CD9 and VLA-2 and both proteins decreased in expression after 3 months TKI administration. The amount of CD9 and VLA-2 in F1 correlated significantly with a decrease of immunosuppressive CD14+HLA-DRneg myeloid-derived suppressor cells as well as monocyte and platelet counts in samples obtained at 3 months with respect to baseline, detected by flow cytometry of PBMCs and routine blood tests. CD9 and VLA-2 in F1 EVs also correlated inversely with CD3negCD56hi16neg cells, a subset of natural killer cells.

This indicates an association of circulating EV phenotype with changes occurring at peripheral blood level in RCC patients receiving TKI.

**Summary/Conclusion**: These preliminary data suggest that plasma EVs may reflect the immune status and the immunomodulating effects occurring during cancer therapies. Additionally, they encourage the rapid development of reliable techniques for the systematic application of body fluid EVs as immune biomarkers of liquid biopsy in cancer.

**Funding**: This work was funded by Italian Ministry of Health grant [GR-2011-02351400].

LBT02.07

Molecular profiling plasma extracellular vesicle unveils features associated with breast cancer aggression, metastasis and invasion

Zhenyu Zhong; Matthew Rosenow; Janet Duncan; David Spetzler

Caris Life Sciences, Phoenix, AZ, USA

**Background**: Extracellular vesicle (EV) based liquid biopsies have been proposed to be a readily obtainable biological substrate recently for both profiling and diagnostics purposes. Development of a fast and reliable preparation protocol to enrich such small particles could accelerate the discovery of informative, disease-related biomarkers. Though multiple EV enrichment protocols are available, in terms of efficiency, reproducibility and simplicity, precipitation based methods are most amenable to studies with large numbers of subjects. However, the selectivity of the precipitation becomes critical.

**Methods**: Here we present a simple plasma EV enrichment protocol based on pluronic block copolymer. The enriched plasma EV was able to be verified by multiple platforms, including DLS, ELISA, western blot, TEM, NGS and semi-quantitative mass spectrometry. Also, plasma EVs from 20 advanced cancer and non-cancer patients were enriched and proteomic profiles were compared. Feature selection and cancer/non-cancer predictive performance were evaluated on a random-forest based cross-validation model.

**Results**: Our results showed that the particles enriched from plasma by the copolymer were EV size vesicles with membrane structure; proteomic profiling showed that EV related proteins were significantly enriched, while high abundant plasma proteins were significantly reduced in comparison to other precipitation based enrichment methods. Next generation sequencing confirmed the existence of various RNA species that was found in EVs from previous studies. Small RNA sequencing showed enriched species compared to the corresponding plasma. Moreover, plasma EVs enriched from 20 advanced breast cancer patients and 20 age-matched non-cancer controls were profiled by semi-quantitative mass spectrometry. Total 60 protein features were identified in classifying advanced breast cancer patients from controls. Interestingly, a large portion of these features were associated with breast cancer aggression, metastasis as well as invasion, consistent to the advanced clinical stage of the patients.

**Summary/Conclusion**: We have developed a plasma EV enrichment method with improved precipitation selectivity compared to other precipitation based methods and it may suitable for large scale plasma EV study

LBT02.08

Detection of lung cancer-specific membrane proteins in plasma exosomes

Taiyoun Rhim; Jisu Lee; Sol Kim; Soohwan Kim; Minhyung Lee

Hanyang University, Seoul, Republic of Korea

**Background**: Recently, we identified four membrane proteins which showed lung cancer specificity. In this study, we tried todetermine whether the cancer specific membrane proteins can be detected on exosomes in the blood of cancer patients or not.

**Methods**: A mouse xenograft model of human lung cancer carcinoma was constructed by injecting lung cancer cells subcutaneously into nude mice. The ELISA condition was optimized using blood samples of xenograft mice

**Results**: The protein G was coated on ELISA plate to ensure the antigen binding domain of the CD63 antibody is orientated away from the plate. The lung cancer specific expressed membrane proteins were detected by sandwich exosome ELISA method in plasma samples of xenograft mice. There was a significant correlation between the size of the xenografted tumour and the amount of protein detected in the exosomes.

**Summary/Conclusion**: In this study, we succeeded to detect lung cancer -specific membrane proteins in plasma exosomes. This success shows the possibility of novel lung cancer diagnostic methods in the future.

LBT02.09

Proteomic analysis of tumour tissue resident EVs in breast cancer

Aleksander Cvjetkovic ^1^; Cecilia Lässer^2^; Rossella Crescitelli^2^; Hafsteinn Petursson^3^; Roger Olofsson^3^; Jan Lötvall^2^

^1^Gothenburg University, Gothenburg, Sweden; ^2^Krefting Research Centre, Institute of Medicine, University of Gothenburg, Gothenburg, Sweden; ^3^Sahlgrenska Academy at the University of Gothenburg, Gothenburg, Sweden

**Background**: Tumours have a protein expression profile that to a degree distinguishes itself from the tissue of origin. This property is extended to the vesicles that the cancerous cells secrete into the tumour microenvironment. Eventually these vesicles could reach the blood circulation and would thus be of interest as biomarkers for disease detection. The aim of this study was to characterize and determine the proteome of tumour-tissue derived extracellular vesicles from breast cancer.

**Methods**: Breast cancer tumour tissues from six patients were cut into smaller pieces (approximately 1 × 1 × 1 mm) and partially enzymatically digested with DNase and Collagenase in cell culture medium for 30 min at 37°C. The digested tissue was filtered through a 70 µm filter to remove pieces of tissue. Vesicles were isolated from the media with an isolation process consisting of differential ultracentrifugation and density gradient floatation aimed at isolating extracellular vesicles. Isolated vesicles were then lysed and trypsin digested before being analysed with mass spectrometry and subsequent label free quantification.

**Results**: In total, approximately 1400 proteins were identified, of which several were found to be related to the tumour. Amongst these were EGFR and HER2, both molecules important in breast cancer biology. More than 300 proteins were detected in tumour vesicles of at least five out of six patients and further experiments are determining whether these are viable biomarker candidates.

**Summary/Conclusion**: The protein expression profiles between tumour tissue-derived vesicles are overall similar, but specific proteins seem to reflect on tumour phenotype, and may be further explored for biological function or biomarker discovery.

The study was approved by the Regional Ethical Approval Committee in Gothenburg, Sweden with informed consent given by all participants.

LBT02.10

Identification of serum microRNAs as diagnostic and prognostic biomarkers for breast cancer

Wen-Hong Kuo ^1^; Ko-Chien Chen^2^; Takahiro Ochiya^3^; Chen-An Tsai^4^; Tang-Long Shen^5^; King-Jen Chang^6^

^1^National Taiwan University Medical School, Taipei, Taiwan (Republic of China); ^2^Department of Life Sciences, National Taiwan University, Taipei, Taiwan (Republic of China); ^3^Division of Molecular and Cellular Medicine, National Cancer Center Research Institute, Chuo-ku, Japan; ^4^National Taiwan University, Taipei, Taiwan (Republic of China); ^5^Department of Plant Pathology and Microbiology, National Taiwan University, Taipei, Taiwan (Republic of China); ^6^Taiwan Adventist Hospital, Taipei, Taiwan (Republic of China)

**Background**: In an era of precision medicine, biomarker discovery is indispensable for novel therapeutics to optimize treatment efficacy. MicroRNAs within patient serum have emerged as novel diagnostic biomarkers for several diseases. They are essential regulators of global mRNA expression in cells. Aberrant regulation of miRNA can result in tumour initiation, drug resistance and metastasis in cancer. miRNA assays are convenient for large-scale studies covering multiple miRNA targets and realistic in screening across diverse breast cancer types for early detection or factors that drive cancer progression.

**Methods**: In this study, we collected patient serum samples from four major molecular subtypes: luminal A, luminal B, triple negative and HER2 type, and breast cancer patients with benign tumour and ductal carcinoma *in situ* (DCIS). Microarray analysis of miRNA expression was utilized and unique serum miRNA signatures between non-cancer and breast cancer patients were identified.

**Results**: While early diagnosis aids in effective management of breast cancer, prognosis is also important to patients during the course of treatment. Thusly, we observed specific miRNA profiles across breast cancer subtypes, suggesting that secreted miRNA coincide with the secreting cancer cell. Moreover, specific clusters of miRNAs demonstrated changes in expression levels over the course of time and varies across subtypes. These trend differences suggest diverse roles taken up by the cancer cell during specific time-points of cancer progression.

**Summary/Conclusion**: Through classifying these heterogeneous compositions of the cancer cell, molecular mechanisms underlying these identified biomarkers can be essential in developing effective treatments and translational research is needed.

LBT02.11

Finding the needle in the Haystack - prostate cancer diagnostics by liquid biopsy

Stefanie Monika Ende; Stefanie Binder; Michael Reuter; Dennis Löffler; Sven-Holger Puppel; Conny Blumert; Kristin Reiche; Friedemann Horn

Fraunhofer IZI Leipzig, Leipzig, Germany

**Background**: Extracellular vesicles (EVs) harbour great potential when applied in innovative liquid biopsy approaches for the diagnosis of various diseases. They could outperform conventional procedures by avoiding risks and disadvantages of regular biopsies e.g. pain, fever, bleeding, infection and various lasting damages. Their immense diagnostic value in discriminating between healthy and cancer patients was already shown in several studies but the use of vesicle-based tests in clinical settings is still very limited. This is at least partially due to the fact that vesicles relevant for diagnosis are massively outnumbered by vesicles produced by multiple, divergent other sources, and hence the informative biomarker patterns are often concealed by irrelevant ones. We aim at developing a specific and sensitive diagnostic test for prostate cancer (PCa) based on plasma vesicles that can be identified by tissue specific surface markers. Based on these surface markers, we will establish methods to specifically enrich vesicles depending on their tissue of origin by antibody- or aptamer-mediated pulldown, and subsequently use these to identify disease-associated biomarkers. The enrichment will allow a highly sensitive detection of cancer-relevant biomarkers, yielding a better statistical power for the resulting diagnostic test.

**Methods**: We used next-generation sequencing to elucidate the composition of exosomal RNA Content and performed mass spectrometry to find surface protein markers specific for their cells or tissue of origin.

**Results**: We found that exosomes from different cancer cell lines can be distinguished by their RNA cargo of which the majority is protein coding. Thereby, we were able to identify a variety of highly specific RNA biomarker candidates specifically enriched in exosomes of the PCa cell lines.

**Summary/Conclusion**: This combinatory approach will enable us to isolate and enrich cell-specific EVs and to identify RNA tumour markers present in tumour-derived vesicles. Subsequently, our findings will be used to establish a test system for the identification of highly specific diagnostic and prognostic biomarkers in blood of PCa patients. If this approach is successful, the established protocols can be transferred and adapted to various malignancies as well as other complex diseases.

LBT03: Late Breaking Poster Session 3 – OMICS Chairs: Emma Guns; Elisa Lázaro-IbáñezLocation: Exhibit Hall 17:15 - 18:30

LBT03.01

Plasma extracellular vesicles in patients with HIV and type 2 diabetes; a proteomic approach to search for HIV comorbidity biomarkers

Beate Vestad ^1^; Tuula A. Nyman^2^; Malene Hove-Skovsgaard^3^; Maria Stensland^2^; Lilly Alice Steffensen^4^; Trude Aspelin^4^; Kari Bente Foss Haug^4^; Ole Kristoffer Olstad^4^; Johannes R. Hov^1^; Susanne Dam Nielsen^3^; Marius Trøseid^5^; Reidun Øvstebø^4^

^1^Research Institute of Internal Medicine, Oslo University Hospital Rikshospitalet, Oslo, Norway; ^2^Department of Immunology, Institute of Clinical Medicine, University of Oslo and Oslo University Hospital Rikshospitalet, Oslo, Norway; ^3^Department of Infectious Diseases, University Hospital of Copenhagen Rigshospitalet, Copenhagen, Denmark; ^4^The Blood Cell Research Group, Department of Medical Biochemistry, Oslo University Hospital, Ullevål, Norway, Oslo, Norway; ^5^Section of Clinical Immunology and Infectious diseases, Oslo University Hospital Rikshospitalet, Oslo, Norway

**Background**: Despite viral suppression by antiretroviral drugs in people living with HIV (PLWH), inflammation persists, and PLWH are at higher risk for non-AIDS comorbidities compared to the general population. The combination of HIV and type 2 diabetes (T2D) has been associated with further increased inflammation and cardiovascular risk. Here, we have analysed proteome profiles of extracellular vesicles (EVs) from plasma to search for biomarkers of HIV comorbidity development.

**Methods**: Fasting plasma (400 µL, EDTA) was sampled from a cross-sectional cohort of virally suppressed individuals (HIV+T2D, HIV alone and controls; n = 12 for each group). Plasma EVs were isolated by size exclusion chromatography (SEC), size and concentration were estimated using nanoparticle tracking analysis (NTA). Label-free quantitative proteomics was performed by liquid chromatography mass spectrometry followed by protein identification, quantification and statistical analysis with MaxQuant and Perseus softwares. Bioinformatic analyses were done using Ingenuity Pathway Analysis and Funrich.

**Results**: Individuals with HIV+T2D had higher particle levels than controls (*p* = 0.02), and slightly higher than “HIV alone” (*p* = 0.07). Collectively, 712 human proteins were identified; 95 differentially abundant proteins were found in “HIV+T2D” and 38 proteins in “HIV alone” compared to controls (*p* < 0.05 for both comparisons). Proteins from “HIV+T2D” were found to be mostly involved in acute phase response signaling and nuclear receptor signaling (FXR/RXR and LXR/RXR activation), while proteins from “HIV alone” were involved in the production of nitric oxide and reactive oxygen species in macrophages. Comparing “HIV+T2D” to “HIV alone”, 79 differentially abundant proteins were detected (*p* < 0.05), mainly involved in inflammatory response and cell-to-cell signaling.

**Summary/Conclusion**: The proteomic profile of plasma EVs from individuals with both HIV and T2D revealed several candidate HIV comorbidity biomarkers, which should further be assessed in cohorts powered for clinical endpoints.

**Funding**: This work was funded by the South-Eastern Norway Regional Health Authority (Helse Sør-Øst) and Sonneborn Stiftelse.

LBT03.02

Proteome of exosomes released by HPV(+) and HPV(-) head and neck cancer cells

Monika Pietrowska ^1^; Lukasz Marczak^2^; Agata Abramowicz^1^; Marta Gawin^1^; Sonja Funk^3^; Priyanka Sharma^4^; Piotr Widlak^1^; Theresa L. Whiteside^4^

^1^Maria Sklodowska-Curie Institute - Oncology Center, Gliwice Branch, Gliwice, Poland; ^2^Institute of Bioorganic Chemistry, Polish Academy of Sciences, Poznan, Poland; ^3^Department of Otorhinolaryngology, University of Duisburg-Essen, Essen, Germany; ^4^Department of Pathology, University of Pittsburgh School of Medicine and University of Pittsburgh Cancer Institute, Pittsburgh, PA, USA

**Background**: Infection with human papilloma virus (HPV) is an important pathological factor in head and neck squamous cell carcinoma (HNSCC). Two categories of HNSCC can be distinguished in terms of HPV status: HPV(+) and HPV(-). HNSCCs differ from each other in respect to their biology and response to therapy. Exosomes are produced by all living cells and mediate intercellular communication. Their protein profiles resemble those of parent cells. Exosomes interact with and reprogram functions of human immune cells. The aim of this study was to examine protein profiles of tumour cell-derived exosomes by mass spectrometry for the presence of proteins which could interact with immune cells and modulate their functions.

**Methods**: We studied protein profiles of exosomes released by cells of three HNSCC HPV(+) cell lines: SCC-2, SCC-47, SCC-90 and two HNSCC HPV(-) cell lines: PCI-13 and PCI-30. Exosomes were isolated from tumour cell supernatants by min-size exclusion chromatography (mini-SEC). The isolated exosomes were assessed for: (1) morphology and size by transmission electron microscopy; (2) number of vesicles by q-Nano; and (3) the protein content. Molecular profiles were determined using high-resolution tandem mass spectrometry (LC-MS/MS) technique. The results were confirmed using on-bead flow cytometry technique.

**Results**: Exosomes originating from HPV(+) and HPV(-) cancer cells had the same size (30–150 nm) and morphology. However, only HPV(+) exosomes contained the following proteins: E6/E7, Rb and survivin, while HPV(-) exosomes were negative for cyclin D1 and had low levels of p53. Application of high-resolution mass spectrometry enabled detection of CD47 and CD276 receptor proteins detected only in exosomes originating from HPV(+) cells.

**Summary/Conclusion**: As both of these proteins play key roles in exosome interactions with immune cells, the data suggest that HPV(+) cancers modulate the host immune system differently than HPV(-) cancers.

**Funding**: The research was financed in part by the Polish National Science Centre project no. [2013/11/B/NZ7/01512].

LBT03.03

Proteomic analysis of exosomes released from irradiated head and neck cancer cells

Agata Abramowicz ^1^; Mateusz Smolarz^1^; Lukasz Marczak^2^; Piotr Widlak^1^; Monika Pietrowska^1^

^1^Maria Sklodowska-Curie Institute - Oncology Center, Gliwice Branch, Gliwice, Poland; ^2^Institute of Bioorganic Chemistry, Polish Academy of Sciences, Poznan, Poland

**Background**: Head and neck squamous cell carcinoma is the sixth most common cancer worldwide with a poor prognosis. Deeper understanding of resistance mechanisms induced in cancer cells during radiotherapy may contribute to improvement of HNSCC curability. We believe that exosomes reported as important players in intercellular communication may play a significant role in response to radiation and other genotoxic agents used in cancer treatment.

**Methods**: UM-SCC6 cells were irradiated with doses of 2, 4, and 8 Gy and cell culture supernatant was collected after 24 h. Exosome samples were purified by differential centrifugation and filtration (0.22 µm), then supernatant was concentrated and finally separated with SEC. Collected fractions were assessed by immunodetection of tetraspanin markers, DLS and TEM techniques. Exosome-enriched fractions were prepared for mass spectrometry analysis according to FASP protocol. Functional enrichment analysis was performed with FunRich software.

**Results**: Mass spectrometry analysis revealed that exosomes from control and irradiated cells contained 801 common and 56 non-overlapping proteins; 54 of them were specific for exosomes from irradiated cells. Quantitative analysis showed that among exosome proteins strongly induced by irradiation there were proteins directly involved in DNA repair like Ku70, Ku80 and E3 ubiquitin-protein ligase HUWE1. Secretion of chaperones like HSP27 and CCT was also significantly increased after irradiation.

**Summary/Conclusion**: Qualitative and quantitative analysis of proteins identified in exosomes released from UM-SCC6 cells revealed effects of radiation on composition of exosome proteome. Among components whose enhanced packing to exosomes was induced by ionizing radiation there were proteins associated with cellular response to stress, DNA repair and intercellular interactions.

**Funding**: This work was supported by National Science Centre, [grant no. 2013/11/B/NZ7/01512].

LBT03.04

Comparative lipidomics platform reveals distinct lipid profiles in extracellular vesicles

Xabier Osteikoetxea ^1^; Jan Schick^1^; Martin Bachman^2^; Ian Sinclair^2^; Nikki Heath^1^; Elisa Lázaro-Ibáñez^3^; Olga Shatnyeva^3^; Niek Dekker^3^; Filippos Michopoulos^4^; Rachel Rowlinson^5^; Ross Overman^1^

^1^Discovery Biology, Discovery Sciences, IMED Biotech Unit, AstraZeneca, Alderley Park, United Kingdom, Macclesfield, United Kingdom; ^2^Sample Management, Discovery Sciences, IMED Biotech Unit, AstraZeneca, Alderley Park, United Kingdom, Alderley Edge, United Kingdom; ^3^Discovery Biology, Discovery Sciences, IMED Biotech Unit, AstraZeneca, Gothenburg, Sweden, Mölndal, Sweden; ^4^Bioscience, IMED Oncology, IMED Biotech Unit, AstraZeneca, Alderley Park, United Kingdom, Alderley Edge, United Kingdom; ^5^Discovery Biology, Discovery Sciences, IMED Biotech Unit, AstraZeneca, Alderley Park, United Kingdom, Alderley Edge, United Kingdom

**Background**: Recently, extracellular vesicles (EVs) have been shown to play important roles in various physiological and pathological processes. One area that has attracted much attention is oncology, with different studies pointing towards the potential of utilizing EVs for diagnostics and therapy. To date, there are many reports on the protein and nucleic acid composition of EVs and their respective roles in the biological functions of these particles. Less is known about EV lipid composition and how it may contribute to various biological roles of EVs or be useful for diagnostics. We developed a high throughput acoustic mist ionisation mass spectrometry (ACMS) platform to investigate the lipid composition of EVs secreted by a panel of non-tumoural, tumoural and metastatic cell lines.

**Methods**: A range of EV subpopulations with differences in size and protein markers were isolated from conditioned media of cell lines by differential centrifugation and filtration. EVs were characterized by nanoparticle tracking analysis, transmission electron microscopy and western blot. Finally, EV preparations were directly subjected to ACMS for analysis of lipid composition. Principle-component analysis was used to analyse and visualize spectral differences.

**Results**: Using 1 μL per EV sample hundreds of features were detected in both positive and negative ion modes in the mass range of 400–1000 Da. Most features belonged to glycerophosphocholines, phosphorylethanolamines, phosphatidylinositols, phosphatidylserines and sphingomyelins among other lipid classes. EV subpopulations and cells were found to differ in lipid composition with some lipid classes such as phosphorylethanolamines overrepresented in EVs as compared to cells. Other differences in lipid composition, such as side chain length and degree of saturation, were observed especially when comparing metastatic to non-metastatic tumoural as well as non-tumoural cell lines.

**Summary/Conclusion**: Cells and distinct EVs subpopulations differ in their lipid composition. Additionally, comparisons of EVs released by non-tumoural, tumoural or metastatic cell lines reveal important differences in their lipid composition which could potentially be useful for diagnostics and to better understand the biological effects of lipids transferred by EVs.

LBT03.05

Comprehensive small RNA sequencing and analysis of extracellular vesicles

D. Michiel Pegtel^1^; Nils Groenewegen ^2^; Michael Hackenberg^3^; Danijela Koppers-Lalic^2^

^1^Exosome Research Group, Department of Pathology, Cancer Center Amsterdam, VU University Medical Center, Amsterdam, The Netherlands., Amsterdam, The Netherlands; ^2^ExBiome BV, Amsterdam, The Netherlands; ^3^ExBiome BV, Granada, Spain

**Background**: Cancer diagnosis is dependent on invasive tissue biopsies and/or expensive imaging techniques, both with their limitations. The detection of cancer biomarkers in body fluids is a promsing approach to complement cancer detection, diagnosis and response monitoring. Exbiome BV offers a next-gen sequencing-based platform for the identification and detection of small (micro) RNA cancer biomarkers in liquid biopsy sources such as urine and blood. MicroRNAs are small gene regulators that are altered in cancer and robustly detected in body-fluids in part due to their association with extracellaulr vesicles (EVs). MiRNAs incorporated into cancer EVs are direct indicator of disease process but circulting miRNAs may also serve as also indicators of ongoing immune responses or metabolic (systemic) chances. One limitation is the high abudnance of certain small RNAs in circulation, overwelming potentially relevant miRNAs, hampering discovery and valdiation of robust biomarkers as indicators of disease. 

**Methods**: Extracellular vesicles (EVs) in bio-fluids contain disease-associated small RNA signatures consisting in part of 21–22 nucleotide miRNAs. Exbiome’s technology platform offers a complete pipeline for full characterization of extracellular small RNAome from patients samples, including EV purification (with standardized size exclusion chromatography), RNA extraction, library preparation, illumina sequencing and a state-of art comprehensive bioinformatics data analysis, quality control and data interpretation.

**Results**: Using our pipeline we analysed 100+ small RNA libraries from circulating plasma EVs. We detected an unprecedented number of miRNAs in healthy individuals and cancer patient plasma samples. We offer a comprehensive analysis of circulating small RNAs with unique quality controls to ensure reliable outcome of the downstream analysis.

**Summary/Conclusion**: Our data shows that a limited amount of high quality plasma (1 ml) is sufficient for a comprehensive next-gen analysis of the EV small RNA transcriptome which is applicable for the discovery of non-invasive cancer biomarkers.

LBT03.06

Radio-detoxified endotoxin alters the protein profile of bone-marrow derived exosomes and enhances the release of endothelial progenitor cells in local chest-irradiated mice

Hargita Hegyesi ^1^; Nikolett Sándor^2^; Violetta Léner^3^; Géza Sáfrány^3^; Virág Lovas^1^; Tamás Visnovitz^1^; Krisztina Pálóczi^4^; Lilla Turiak^5^; Lóránd Bertók^3^; Edit I. Buzás^6^

^1^Semmelweis University Department of Genetics, Cell- and Immunobiology, Budapest, Hungary; ^2^National Public Health Center National Research Directorate for Radiobiology and Radiohygiene, Budapest, Hungary; ^3^National Public Health Center National Research Directorate for Radiobiology and Radiohygiene, Anna st 5, Hungary; ^4^Department of Genetics, Cell- and Immunobiology, Semmelweis University, Budapest, Hungary; ^5^Research Centre for Natural Sciences, Hungarian Academy of Sciences, Budapest, Hungary; ^6^MTA-SE Immune-Proteogenomics Extracellular Vesicle Research Group, Budapest, Hungary

**Background**: As the incidence of breast cancer continues to rise, the use of radiotherapy (RT) has emerged as a leading treatment modality. However, RT also increases the risk of coronary heart disease and cardiac mortality. Several studies have demonstrated the protective effects of radio-detoxified endotoxin (RD-LPS) in reducing chemotherapy- and radiation-induced cardiac damages. Bone-marrow (BM) derived endothelial progenitor cells (EPCs) have been shown to have regenerative potential in endothelial injuries. In our chest-irradiated mouse model here we investigated if exosomes (EXOs) could play a role in RD-LPS induced EPC activation.

**Methods**: Hearts of C57BL/6 mice received a 16 Gy single dose of X-ray radiation. In this mouse model of RT-induced cardiac injury, we quantified RD-LPS treated BM derived EXOs, analysed their proteomic composition by MS, measured IFITM3 protein levels in BM derived EXOs released after RD-LPS treatment by an ELISA. EPCs (CD31+ or FLK-1+) and CD34+ hematopoietic stem cells (HCS) were immunophenotyped both in blood and BM samples by flow cytometry.

**Results**: Mice showed increased lethality after 16 Gy local chest irradiation, while RD-LPS treatment prolonged their median survival significantly. Both in BM and circulation of the exposed and RD-LPS treated groups, the number of EPCs and HCS were higher than in the non-irradiated mice. MS results demonstrated that BM EXO proteins in RD-LPS treated mice included both a common set of EXO proteins and specific subsets of treatment-related proteins such as interferon-induced transmembrane protein-3 (IFITM3), which correlated with treatment-associated functions. Flow cytometry and ELISA assessment of EXOs secreted by BM cells of RD-LPS treated mice, revealed a difference in the expression of IFITM3 between EXOs released in the presence or absence of RD-LPS.

**Summary/Conclusion**: This is the first study to demonstrate that RD-LPS treatment induces migration of EPCs into the circulation, which leads to an attenuated RT mortality. EPC activation is dependent on RD-LPS treatment, which leads to IFITM3 upregulation in the BM derived EXOs. Our data suggest that EXO IFITM3 may play a role and serve as a potential biomarker in cardiac regeneration.

**Funding**: This work was supported by National Research, Development and Innovation Fund of Hungary; with the following grants [NVKP_16-1-2016-0017].

LBT03.07 = OWP2.07

Immunofluorescence flow cytometry of extracellular vesicle surface proteins

John Nolan; Erika Duggan

Scintillon Institute, San Diego, CA, USA

**Background**: Like the cells that produce them, extracellular vesicles (EVs) bear surface molecules that can give clues to identity and function. Unlike cells, surface proteins on EVs are present in numbers that challenge the sensitivity of conventional flow cytometers, which presents challenges to quantitative and reproducible measurements. We have adapted calibration and standardization approaches from quantitative IF of cells to enable quantitative and reproducible measurement of EV surface proteins.

**Methods**: Erythrocytes and platelets (RBCs, PLTs) were washed, treated with ionophore (A23187) in the presence of Ca+2, and centrifuged (2 ×, 2500 ×*g*, 15 min) to remove cells and large debris. Cell lines were cultured for 48 h in EV-free media and the media collected, centrifuged to remove cells and large debris, and concentrated ~100-fold by centrifugal ultrafiltration and stored at -80C. Vesicle flow cytometry (VFC) was performed using a vesicle measurement kit comprised of a vesicle staining solution and a synthetic vesicle size standard. EV samples were stained with fluorescent antibodies (FL-Abs) to various surface markers and measured by flow cytometry using a fluorescence trigger. Fluorescence intensity was calibrated using commercial MESF intensity standards, custom intensity standards and antibody-capture standards.

**Results**: VFC measures the number, size, and FL-*Ab* staining of individual EVs, to ~70 nm in diameter and ~30–50 PE-Abs. We performed VFC with IF on RBC and PLT EVs using antibodies to abundant cell surface proteins, with antigen-free vesicles and non-specific IgGs serving as controls. RBC EVs were 75–300 nm in diameter (median 160 nm) and bound 90–6000 PE-Abs (median 2200 MESF) to CD235ab. PLT EVs were 75–500 nm in diameter (median 175 nm) and bound 90–9000 PE-Abs to CD41 (median 900 MESF), 50–6000 CD61 (median 480 MESF) and 50–3000 PE-Abs (median 625 MESF) to CD9. Antibody capture beads with calibrated numbers of *Ab* binding sites allow quantitative assessment of different fluorescent conjugates for suitability in EV IF.

**Summary/Conclusion**: By observing the basic tenets of quantitative FC, including using appropriate controls, standards, calibration protocols and experimental design, EV IF can be performed quantitatively and reproducibly.

Scientific Program ISEV2018Friday, 04 May 2018 Symposium Session 10 - EV Biogenesis and Uptake Chairs: Isabel Guerrero; Guillaume van Niel Location: Auditorium 08:30 - 10:00

OF10.01

Following the trafficking of extracellular vesicles markers to understand the biogenesis of different extracellular vesicles subtypes

Mathilde Mathieu ^1^; José Ignacio Valenzuela^2^; Mathieu Maurin^3^; Gaëlle Boncompain^2^; Franck Perez^2^; Clotilde Thery^1^

^1^Institut Curie / PSL Research University / INSERM U932 / Université Paris Descartes, Paris, France; ^2^Institut Curie / PSL Research University / INSERM Umr144, Paris, France

**Background**: Different studies reported apparently contradictory roles of vesicles secreted by tumour cells. These discrepant observations might be due to differences in the types of vesicles analysed. Defining better the various kinds of EVs secreted by tumour cells would help to elucidate these divergent roles. We focused on understanding how the different types of EVs are generated by following the trafficking of proteins differently associated with EV subtypes, in particular tetraspanins.

**Methods**: We used the RUSH system, an innovative technique developed at the Institut Curie, to synchronize and follow the trafficking of tetraspanins. Synchronized trafficking enables to quantify the extent of transport, to identify the intermediate stations of trafficking, to carry out experiments in real-time and in living cells and to screen for specific inhibitors or enhancers of transport for a protein of interest. We chose to study in HeLa and MCF7 cells the trafficking of different markers secreted in several types of EVs, especially CD63 and CD9. By combining the RUSH system with 4D live-cell imaging, kinetic and co-localization analyses we analysed some aspects of their intracellular trafficking and arrival to the plasma membrane.

**Results**: We showed by immunoprecipitation that some small EVs carry both CD63 and CD9 while some others carry only CD9. CD63 and CD9 do not traffic to the same compartments and are found only transiently into common intracellular compartments, despite their presence in similar EVs. While CD63 is addressed to endosomal compartments, CD9 traffics to the plasma membrane. This observation suggests that some CD9-bearing small EVs form at the plasma membrane rather than in endosomal compartments, and thus do not correspond to exosomes.

**Summary/Conclusion**: Understanding how CD63 and CD9 are sorted into similar or different EVs while trafficking differently will provide new insights on the biogenesis mechanisms of the different types of EVs.

OF10.02

A novel conserved exosome biogenesis pathway mediates adaptive response to microenvironmental stress in cancer cells

Shih-Jung Fan; Benjamin Kroeger; Kristie McCormick; John Mason; Helen Sheldon; Mark Wainwright; John Morris; Adrian Harris; Clive Wilson; Deborah CI. Goberdhan

University of Oxford, Oxford, UK

**Background**: In the classical exosome secretory pathway, intraluminal vesicles (ILVs) formed in late endosomal multivesicular bodies (MVBs) are released as exosomes when these compartments fuse to the plasma membrane. We test the hypothesis that recycling endosomes form other types of exosome.

**Methods**: We have combined mechanistic studies from human cancer cell lines with a Drosophila model of exosome biogenesis that we have developed. Our *in vitro* human cell culture models have enabled us to analyse the effects of microenvironmental stress mediated by reduced signalling through mechanistic Target of Rapamycin Complex 1 (mTORC1) on exosome protein content, using western analysis, and on exosome function, using an IncuCyte live cell imager to analyse target cell response. This analysis has been complemented by our *in vivo* fly model, which has enabled us to visualise different types of multivesicular endosome, using super-resolution 3D-structured illumination microscopy.

**Results**: We demonstrate that vesicles carrying distinct cargos, including the small GTPase Rab11, are formed inside Rab11-positive recycling endosomal compartments in flies and human cancer cell lines. Decreasing mTORC1 activity in cancer cells by reducing extracellular glutamine in glutamine-dependent tumour cells or by pharmacological inhibition stimulates secretion of these alternative exosomes. This effect is mediated by increased membrane flux through Rab11a-compartments, increasing secretion of exosomes that preferentially maintain endothelial networks and drive ERK-MAPK-dependent cancer cell growth. This activity that is suppressed by blocking ILV biogenesis or Rab11a-dependent trafficking.

**Summary/Conclusion**: We conclude that exosome heterogeneity is partly generated by biogenesis in different endosomal compartments and that a metabolically regulated switch in secreting different classes of exosome may mediate adaptive responses of tumours to microenvironmental stresses and anti-cancer therapies.

**Funding**: This paper was funded by Cancer Research UK [C19591/A19076], the Cancer Research UK Oxford Centre Development Fund [C38302/A12278], the BBSRC [BB/K017462/1, BB/L007096/1, BB/N016300/1], John Fell Fund, Oxford, Wellcome Trust, Royal College of Surgeons.

OF10.03

HA-EVs are a unique species of EV with diverse properties and widespread biological relevance

Uma Thanigai Arasu^1^; Kai Härkönen^1^; Sanna Oikari^1^; Arto Koistinen^2^; Kirsi Rilla ^1^

^1^Institute of Biomedicine, University of Eastern Finland, Kuopio, Finland; ^2^SIB Labs, University of Eastern Finland, Kuopio, Finland

**Background**: Extracellular vesicles (EVs) are small plasma membrane-derived particles released into the extracellular matrix (ECM) by virtually all cell types. Recently, EVs have received increased interest because of their capability to carry nucleic acids, proteins, lipids and signaling molecules and to transfer their cargo into the target cells. Less attention has been paid to the carbohydrates carried on the surfaces of EVs and their impact on EV biogenesis and targeting. One of those carbohydrates ishyaluronan (HA), one of the main building materials of the ECM with an overwhelming ability to bind water. A typical feature of active cells is a thick pericellular HA-rich matrix. EVs that are generated by these cells carry a similar HA coat on their surface and are thus called HA-EVs.

**Methods**: Interestingly, based on our recent results, HA synthesis on the plasma membrane and filopodia accelerates biogenesis of HA-EVs. To obtain more details on their structure and functions, we analysed HA-EV biogenesis, kinetics and their binding to target cells by live cell and superresolution microscopy, electron microscopy and their combinations, NTA and ELISA assays.

**Results**: We found that HA-EVs are generated by diverse mechanisms, such as shedding from filopodia, retraction fibers, fractionation of protrusions, and they have variable size and morphology. Additionally, binding assays showed that they have specific effects on target cells, such as induction of HA synthesis and EMT.

**Summary/Conclusion**: We suggest that shedding of HA-EVs is a general mechanism for all active cell types, such as by cancer (1, 2), stem (3) and injured (4) cells that produce HA on their filopodia and other plasma membrane protrusions. HA coating on the surface of EVs acts as potential prognostic and therapeutic factor and mediates tissue regeneration. It also regulates homing and targeting of EVs and has potential as a tool for drug delivery.

**References**

1. Rilla et al. Exp Cell Res. 2013;319:2006–2018.

2. Rilla et al. Adv. Cancer Res. 2014;123:121–148.

3. Arasu et al. Matrix Biol. 2017;64:54–68.

4. Koistinen et al. Matrix Biol. 2016;63:38–54.

**Funding**: This work was funded by Academy of Finland.

OF10.04

The integrin Mac1/CR3 plays central role in production and cargo editing of EVs issued from neutrophilic granulocytes

Erzsébet Ligeti ^1^; Balázs Bartos^1^; Dávid Szombath^1^; Lilla Turiak^2^; László Drahos^3^; Dániel Veres^4^; Ágnes Kittel^5^; Attila Mócsai^1^; Ákos Lőrincz^1^

^1^Department of Physiology, Semmelweis University, Budapest, Hungary; ^2^Research Centre for Natural Sciences, Hungarian Academy of Sciences, Budapest, Hungary; ^3^Hungarian Academy of Sciences, Budapest, Hungary; ^4^Department of Biophysics, Semmelweis University, Budapest, Hungary; ^5^Institute of Experimental Medicine, Hungarian Academy of Sciences, Budapest, Hungary

**Background**: In previous works we characterized three physiologically occurring types of EVs released from granulocytes spontaneously (sEV), during apoptosis (apoEV) or upon activation with opsonized particles (aEV). The latter EVs are specifically enriched in granule proteins and possess antibacterial effect. Our goal was to identify receptor(s) and signaling pathway(s) responsible for specific aEV formation.

**Methods**: Medium-size EVs were obtained from isolated neutrophils (PMN) by two-step centrifugation and characterized by dynamic light scattering and electron microscopy. EV generation was assessed on the basis of protein content and of EV count determined by flow cytometry. Protein identification was carried out by mass spectrometry and proteomic analysis.

**Results**: On human PMNs Ig-binding Fc receptors (FcR), complement-binding CR3 (Mac1 integrin) and pattern recognition receptors (PRR) were stimulated separately or in combination and EV generation was determined. Stimulation of PRR had weak effect whereas activation of CR3/Mac1 resulted in significant aEV generation. FcRs did not seem to be involved in EV production. These results were supported by experiments carried out on PMN issued from genetically deficient animals. Both in the human and in the murine systems tyrosine kinases, calcium signaling and phospholipase C were required for aEV production. Specific enrichment of proteins of azurophil and specific granule origin was detected only in aEVs initiated by Mac1/CR3 stimulation, and inhibition of tyrosine kinases prevented the cargo editing. Importantly, all these interventions did not influence sEV production.

**Summary/Conclusion**: We have identified a specific receptor and part of the initiated signaling pathway which are responsible for generation of aEVs with special cargo content, but are not involved in constitutive release of EVs. We thus provide evidence for the existence of two separate molecular mechanisms of EV generation in PMN.

**Funding**: This work was funded by NKFIH K119236 and VEKOP-2.3.2-16-2016-00002, Hungary.

OF10.05

Extracellular vesicle budding is inhibited by redundant regulators of TAT-5 flippase localization and phospholipid asymmetry

Katharina Beer; Ann M. Wehman

Rudolf Virchow Center at the University of Würzburg, Würzburg, Germany

**Background**: Cells release extracellular vesicles (EV) that mediate intercellular communication and repair damaged membranes. Despite the pleiotropic functions of EVs *in vitro*, their *in vivo* function is debated, largely because it is unclear how to specifically induce or inhibit their formation. In particular, the mechanisms of microvesicle release by plasma membrane budding or ectocytosis are poorly understood. We previously showed that TAT-5 phospholipid flippase activity inhibits microvesicle budding by ESCRT-mediated ectocytosis in *C. elegans*. TAT-5 maintains the asymmetric localization of the lipid phosphatidylethanolamine (PE) in the plasma membrane, but no proteins were known that regulate TAT-5 activity to inhibit ectocytosis.

**Methods**: We used the C. elegans embryo as a genetic model system for EV budding. We generated degron reporter strains that make plasma membrane-derived EVs visible by light microscopy and screened for new regulators of microvesicle budding using RNAi and mutant strains.

**Results**: We identified new TAT-5 regulators associated with retrograde endosomal recycling, specifically the PI3Kinase VPS-34, the Beclin1 homolog BEC-1, the DnaJ protein RME-8, and the uncharacterized Dopey homolog PAD-1. PI3Kinase, RME-8 and semi-redundant sorting nexins are required for the plasma membrane localization of TAT-5, which is important to maintain PE asymmetry and inhibit EV release. The GEF-like protein MON-2 also has roles in endosomal trafficking that regulate EV release, albeit redundantly with sorting nexins independent of the core retromer. In contrast, PAD-1 is required for the lipid flipping activity of TAT-5, without directly regulating TAT-5 localization.

**Summary/Conclusion**: This study identified new proteins that regulate EV release and uncovered redundant intracellular trafficking pathways important for TAT-5 lipid flippase activity. This work pinpoints TAT-5 and PE as key regulators of plasma membrane budding, further supporting the model that PE externalization drives ectocytosis.

OF10.06

Uptake of Extracellular Vesicles in a cell free extract

Jeff Coleman^1^; Clotilde Thery^2^; Gregory Lavieu ^3^

^1^Yale University, New Haven, CT, USA; ^2^Institut Curie / PSL Research University/INSERM U932, Paris, France; ^3^Institut Curie/INSERM, Paris, France

**Background**: Tremendous progresses have been made in understanding the physiology and physiopathology of EVs. However, our knowledge of the cell biology of EVs remains far behind, especially the delivery process within the acceptor cell. This is not satisfying when considering the high translation impact that EVs could offer.

**Methods**: To gain insight in the EVs uptake process, we used a classical *in vitro* cell free approach. We developed a content mixing assay: briefly, purified EVs containing a tagged cargo were mixed with purified plasma membrane sheets. After incubation, samples were submitted to protease digestion.

**Resultss**: EVs cargo that is normally protected from protease digestion became degraded only when PM sheets and EVs were exposed at pH5.5 suggests that EVs content release requires PM-derived membranes and an endosome-like environment. Importantly, pretreatment with protease that stripped off proteins from the surface of EV/PM sheets, prevented content release. On the same line, purified EVs were unable to mix with protein free/cholesterol enriched liposomes, regardless of the pH. As a positive control, we observed membrane mixing at pH5.5 between those very same liposomes and EVs harbouring VSV-G, a viral fusogenic protein known to fuse with cholesterol containing membranes at acidic pH.

**Summary/Conclusion**: Altogether our results suggest that EVs content release requires proteins present at the surface of the acceptor cell, followed by endocytosis/acidification that triggers the content release. Analogy with certain viruses suggests that the delivery process could correspond to a membrane fusion event.

With this assay in hand, we are now in a position to further characterize the content release mechanism (fusion?) to ultimately identify the core machinery required for this process.

**Funding**: This work was funded by INSERM and ARC.

Symposium Session 11 - EVs and Metastatic Niches Chairs: Vincenza Dolo; Jacky Goetz Location: Room 5 08:30 - 10:00

OF11.01

Breast cancer-derived extracellular vesicles modulate the activity of signaling pathways in the brain microenvironment

Golnaz Morad; Marsha Moses

Vascular Biology Program, Boston Children’s Hospital, Boston, MA 02115, Boston, MA, USA

**Background**: Breast cancer (BCa) metastasis to the brain is associated with high mortality and remains a major medical challenge. With the goal of elucidating the early mechanisms leading to brain metastasis, we have focused on the role of extracellular vesicles (EVs) in this process. Tumour-derived EVs have been implicated as potential regulators of metastatic microenvironments. We hypothesized that BCa-derived EVs can facilitate brain metastasis by inducing alterations in key signaling pathways in the brain microenvironment.

**Methods**: EVs were isolated from the parental MDA-MB-231 BCa cell line (P-EVs) and its brain-seeking variant (Br-EVs). Female nude mice received retro-orbital injection of EVs or PBS three times per week for 3 weeks. Following EV treatment, one group of mice was sacrificed and brain samples were collected for protein expression analysis. The relative activity of 26 signaling pathways were analysed in brain tissues collected from control and Br-EV-treated mice, using the ActivSignal Immuno-Paired Antibody Detection platform. A second group of mice received an intracardiac injection of the brain-seeking MDA-MB-231 cells. Metastasis formation was evaluated by histological analysis after 4 weeks.

**Results**: Treatment with Br-EVs induced a 2.5-fold increase in the frequency of brain metastasis compared to the control and the P-EV-treated groups. Evaluation of the effect of Br-EV treatment on the activity of signaling pathways in the brain microenvironment demonstrated an increase in the heat shock response, as supported by increased phosphorylation of HSP70 and HSP27. The JAK/STAT and PI3K/AKT pathways, both known to be involved in brain metastases, were also downregulated by Br-EVs. To our knowledge, this is the first report that EVs modulate these signaling pathways in the pre-metastatic brain microenvironment.

**Summary/Conclusion**: EVs derived from brain-seeking BCa cells increase the frequency of brain metastasis. Our signaling pathway analyses suggest that this facilitation of metastasis formation involves an EV-derived increase in the heat shock response and a decrease in the activation of JAK/STAT and PI3K/AKT pathways in the brain microenvironment. These novel findings may have significant clinical potential.

**Funding**: This work was supported by the Breast Cancer Research Foundation and the Advanced Medical Research Foundation.

OF11.02

Zebrabow as *in vivo* model system to monitor vesicles mediated transfer in cancer

Martin E. van Royen ^1^; Wilma Teubel^2^; Thomas A. Hartjes^3^; Tjakko van Ham^4^; Guido W. Jenster^3^

^1^Department of Pathology, Erasmus Optical Imaging Centre, Erasmus MC, Rotterdam, The Netherlands; ^2^Department of Urology, Erasmus MC, Rotterdam, The Netherlands; ^3^Erasmus Medical Center, Rotterdam, The Netherlands; ^4^Department of Clinical genetics, Erasmus MC, Rotterdam, The Netherlands

**Background**: Tumour cells influence their microenvironment, enhancing tumour progression and metastasis via extracellular vesicle (EV) mediated transfer of proteins and RNAs. Zebrafish are ideal *in vivo* model system because of their simple manipulation and natural transparency for fluorescent imaging. Using non-invasive imaging and the Cre-LoxP switch-reporter system we explored the potential of this model system to visualize *in vivo* spreading and uptake of cancer cell-derived EVs.

**Methods**: Vesicles were isolated from two prostate cell lines expressing high levels of Cre-recombinase. Four nL of vesicle isolate, or synthetic Cre-recombinase mRNA was injected the yolk of embryos in early development (1–8 cells). The Zebrabow fish contains a Cre-LoxP -reporter that switches in fluorescence in cells expressing Cre-recombinase, mediated through injected mRNA or via uptake of EVs isolated from Cre-expressing cell lines. After 4–6 day of further development, fish were, immobilized in agarose and positioned in a microplate for microscopic inspection of fluorescence in the complete zebrafish using a high content screening system. Using qPCR, absolute amounts of Cre mRNA in the EVs were determined. The EV concentrations were determined using EVQuant.

**Results**: Injected synthetic Cre-recombinase mRNA was able to efficiently switch the Cre-LoxP reporter system in injected embryos, resulting in a large variation of fluorescent cells distributed in the complete zebrafish in a mosaic pattern. In contrast, injected EVs derived from cells with high Cre expression were able to colour switch cells in only 1 out of 60 injected zebrafish. The low efficiency in EV-mediated Cre-protein or RNA transfer is correlated with small quantity of Cre-mRNA present in the 4 nL EV isolate that contained approximately 30 × 10^−14^ pg compared to the 50 pg present in the 4 nL synthetic Cre-mRNA solution.

**Summary/Conclusion**: The Zebrabow Cre-LoxP reporter system is an efficient reporter for Cre activity and could therefore be an ideal model system to study EV-transfer *in vivo*. However, the amount of EV-mediated transfer of Cre-mRNA is too low with a single injection of 4 nL of purified EVs from Cre-expressing cell lines. This very low efficacy can well be explained by the relative low Cre-mRNA quantity in EVs and the small volume that can maximally be injected in the yolk of zebrafish embryos.

OF11.03

Pre-metastatic cancer exosomes induce immune surveillance by patrolling monocytes

Michael P. Plebanek^1^; Nicholas Angeloni^1^; Elena Vinokour^1^; Anna Henkin^2^; Dalia Martinez-Marin^3^; Stephanie Filleur^3^; Reshma Bhowmick^4^; Jack Henkin^5^; Stephen Miller^1^; Igal Ifergan^1^; Yesung Lee^6^; Iman Osman^6^; Shad Thaxton^1^; Olga Volpert ^7^

^1^Northwestern University, Chicago, IL, USA; ^2^Massachusetts Institute of Technology, Boston, MA, USA; ^3^Texas Tech University, Lubbock, TX, USA; ^4^University of Texas MD Anderson Cancer Center, Houston, TX, USA; ^5^Northwestern University, Evanston, IL, USA; ^6^New York University, New York, NY, USA; ^7^MD Anderson Cancer Center, Houston, TX, USA

**Background**: Cancer exosomes are often involved in the suppression of innate immune responses. Monocytes and macrophages are essential in the metastatic microenvironments, in tumour-promoting or tumour-suppressive capacities. Non-classical or patrolling Ly6C low monocytes (PMo) were identified for the ability to remove damaged cells and rely on nuclear receptor Nr4a1 for survival. Recently, Nr4a1-positive PMo were implicated in scavenging metastatic tumour cells in the lungs. However, the events that control PMo at the metastatic niche remain unknown.

**Methods**: We isolated and tested exosomes from spontaneously occurring and artificially generated metastatic/ non-metastatic melanoma cells and tested them *in vivo* for altering metastatic capacity of human and mouse cells. The effect on bone marrow myeloid cells was examined by FACS and dependence on specific cell types was determined using clodronate liposomes and neutralizing antibodies. The effects on macrophages were examined in functional and biochemical assays. The relevance of the findings was assessed by a functional and biomarker analysis of patient exosomes.

**Results**: Exosomes from non-metastatic melanoma cells (ExoNM) are taken up by myeloid cells in the bone marrow and cause an expansion of Ly6C low monocytes, which display elevated levels of integrin-β2, CX3CR1, and Nr4a1, which define patrolling monocytes. Pigment epithelium-derived factor (PEDF) is known for its anti-angiogenic, anticancer effects. In melanoma, PEDF suppresses ameboid invasion and metastasis. PEDF is also implicated in control of macrophage polarization via unknown mechanisms. Here, we demonstrate that PEDF is present at high levels on the surface of exosomes from non-metastatic melanoma cells and its presence is critical for the activation of an innate immune response and elimination of melanoma metastasis. The resultant events induce Nr4a1 in myeloid cells, cause PMo expansion, recruitment, and expansion of TRAIL-positive macrophages, which kill and engulf tumour cells. Together, PMo and NK cells eliminate metastasis as is shown by depletion experiments.

**Summary/Conclusion**: Our results suggest that non-metastatic tumours generate triggers of innate immune response(s) such as PEDF, which are delivered to the cells of the immune system by exosomes and maintain tumour clearance.

OF11.04

Melanoma-derived exosomes reinforce metastasis by inducing lymphangiogenesis and impairing dendritic cell function

Susana Garcia-Silva^1^; Alberto Benito-Martin^2^; Laura Nogues-Vera^3^; Alberto Hernandez-Barranco^1^; Raghu Kataru^4^; Cristina Merino^1^; Marina Mazariegos^1^; Ana Isabel Amor^1^; Marta Hergueta-Redondo^5^; Irina Matei^3^; Babak Mehrara^4^; David Lyden^6^; Héctor Peinado ^5^

^1^Microenvironment and Metastasis Group, Molecular Oncology Program, Spanish National Cancer Research Centre (CNIO), Madrid, Spain; ^2^Department of Pediatrics, Drukier Institute for Children’s Health and Meyer Cancer Center, Weill Cornell Medical College, New York, NY, USA; ^3^Department of Pediatrics, Drukier Institute for Children’s Health and Meyer Cancer Center, Weill Cornell Medical College, New York, NY, USA; ^4^Memorial Sloan Kettering Cancer Center, New York, NY, USA; ^5^Microenvironment and Metastasis Group, Molecular Oncology Programme, Spanish National Cancer Research Centre (CNIO), Madrid, Spain; ^6^Departments of Pediatrics, Cell and Developmental Biology, Drukier Institute for Children’s Health and Meyer Cancer Center, Weill Cornell Medical College, New York, NY, USA

**Background**: Lymph nodes (LNs) adjacent to the primary tumour are often the first site of metastasis. It has been demonstrated that tumour cells induce early changes in the architecture and physiology of tumour draining LNs, even before tumour colonization in a process known as pre-metastatic niche formation. Melanoma-secreted exosomes have been shown to home to LNs supporting metastatic progression although the mechanism is unknown. 

**Methods**: Exosomes from human and mouse melanoma cell lines were purified by ultracentrifugation and labeled with fluorescence dyes. Exosomes were injected intrafootpad for studying retention and changes in LN populations. Those changes were anaysed by FACS. RNA sequencing was performed on lymphatic endothelial cells (LECs). C-MS/MS analysis of melanoma-secreted exosomes was executed and further data analysis was developed using Perseus and Panther.

**Results**: Tumour-secreted exosomes derived from highly metastatic models and lymph-tropic models have a faster and wider distribution through the lymphatic system as compared with exosomes derived from low or non-metastatic models. We found that LECs and subcapsular and medullary macrophages are the main cell types incorporating exosomes. We performed transcriptional profiling of human LECs after treatment with melanoma-derived exosomes. We identified a subset of genes related to the neural origin of melanocytes. This group includes several members of the neurotrophin family that could be crucial for the pre-metastatic niche formation. In addition, exosome treatment elicits upregulation of lymphangiogenesis-related genes and triggers neo-vessel formation and increased lymphangiogenesis *in vivo*. Blocking of specific cargo in exosomes led to a decrease of melanoma metastasis, lymphangiogenesis and reactivation of dendritic cell function.

**Summary/Conclusion**: Our data support that melanoma-secreted exosomes reinforce metastasis by targeting lymphatic endothelial cells in the LN inducing a lymphangiogenesis and impairing dendritic cell function.

**Funding**: This work is supported by grants from National Institutes of Health (National Cancer Institute), United States Department of Defense, Ministerio de Economía y Competitividad [SAF2014-54541-R], Fundación FERO and Asociación Española Contra el Cáncer.

OF11.05

Paracrine mechanisms induced by large oncosomes spontaneously shed by aggressive cells to promote adhesion and invasion of prostate cancer via αv integrin-dependent activation of FAK-AKT pathway

Chiara Ciardiello ^1^; Alessandra Leone^1^; Maria Serena Roca^1^; Tania Moccia^1^; Michele Minopoli^2^; Carlo Vitagliano^1^; Biagio Pucci^3^; Susan Costantini^3^; Francesca Capone^4^; Maria Rita Milone^4^; Rita Lombardi^4^; Francesca Bruzzese^1^; Maria Vincenza Carriero^2^; Dolores Di Vizio^5^; Alfredo Budillon^1^

^1^Experimental Pharmacology Unit, Istituto Nazionale per lo Studio e La Cura dei Tumori Fondazione Giovanni Pascale - IRCCS, Napoli, Italy; ^2^Neoplastic Progression Unit, Istituto Nazionale per lo Studio e La Cura dei Tumori Fondazione Giovanni Pascale - IRCCS, Napoli, Italy; ^3^Mercogliano Oncology Research Center (CROM) Istituto Nazionale per lo Studio e La Cura dei Tumori Fondazione Giovanni Pascale IRCCS, Mercogliano, Italy; ^4^Mercogliano Oncology Research Center (CROM) Istituto Nazionale per lo Studio e La Cura dei Tumori Fondazione Giovanni Pascale - IRCCS, Mercogliano, Italy; ^5^Departments of Surgery, Biomedical Sciences, and Pathology and Laboratory Medicine, Cedars-Sinai Medical Center, Los Angeles, USA

**Background**: The identification of new molecular prognostic markers for Prostate Cancer (PCa) is needed to optimize both therapeutic options and follow-up strategies. Thereafter, we characterized a syngeneic model consisting of parental DU145 PCa cells and their derived more aggressive subline DU145R80. A proteomic approach highlighted a small number of differentially expressed proteins between the two cell lines, including key-molecules in cancer progression such as αv-integrin. In the present study, by using the DU145/DUR14580 system, we defined a novel αv-integrin-dependent paracrine effect exerted by large EVs previously described as large oncosomes (LO) to promote tumour cell aggressiveness.

**Methods**: FITC-conjugated cholera toxin B subunit labeled cells highlighted blebbing. Flow cytometry using standard beads identify EVs >1 µM. LO were isolated from cell media by discontinuous 5%–60% OptiPrep™ density gradient ultra-centrifugations. Adherent cells were counted manually. Invasion was evaluated by Boyden chambers. Proteolytic activity was measured by gel zimography. Xenografts of LO-treated/untreated DU145 cells were employed in nude mice.

**Results**: In the present study we found spontaneous blebbing and increased LO shedding from DU145R80 compared to parental DU145 cells. LO from DU145R80 carried increased amount of active metalloproteinase 2 and αv-integrin, compared to LO from DU145. DU145R80-derived LO increased adhesion and invasion in recipient DU145 cells, activating FAK-AKT pathway and increasing proteolytic activity of recipient cells. By blocking αV-integrin on LO surface, using an anti-αv antibody, we reverted the LO-induced effect on adhesion, invasion and MMPs activity in DU145 recipient cells. DU145R80-derived LO promote DU145 tumorogenesis *in vivo*.

**Summary/Conclusion**: Overall, these findings highlighted αv-integrin as a critical molecule in the mechanisms by which LO promote PCa cells aggressiveness.

OF11.06

Circulating large EVs in plasma of patients with metastatic prostate cancer contain chromosomal DNA and report cancer-specific genomic alterations

Tatyana Vagner^1^; Cristiana Spinelli^2^; Valentina R. Minciacchi^3^; Mandana Zandian^4^; Andries Zijlstra^5^; Michael R Freeman^4^; Francesca Demichelis^6^; Edwin M. Posadas^7^; Hisashi Tanaka^8^; Dolores Di Vizio ^9^

^1^Department of Surgery, Cedars-Sinai Medical Center, Los Angeles, CA, USA; ^2^McGill University, Montreal, Canada; ^3^Georg-Speyer Haus, Institute for Tumor Biology and Experimental Therapy, Frankfurt, Germany; ^4^Cedars-Sinai Medical Center, Los Angeles, CA, USA; ^5^Department of Pathology, Microbiology and Immunology, Vanderbilt University Medical Center, Nashville, TN, USA; ^6^Institute for Precision Medicine, Weill Cornell Medical College-New York Presbyterian Hospital, New York, NY, USA; Centre of Integrative Biology, University of Trento, Trento, Italy; ^7^Cedars Sinai Medical Center, Los Angeles, CA, USA; ^8^Division of Cancer Biology and Therapeutics, Departments of Surgery, Biomedical Sciences and Pathology and Laboratory Medicine, Samuel Oschin Comprehensive Cancer Institute, Cedars-Sinai Medical Center, Los Angeles, CA, USA; ^9^Departments of Surgery, Biomedical Sciences, and Pathology and Laboratory Medicine, Cedars-Sinai Medical Center, Los Angeles, CA, USA

**Background**: Cancer-derived extracellular vesicles (EVs) are heterogeneous membrane-enclosed structures of highly variable size and content. Atypically large bioactive EVs termed large oncosomes (LO) are released by highly migratory tumour cells as a consequence of DIAPH3 reduced expression, which results in an amoeboid phenotype. LO have been identified in tumour tissue and plasma of patients with metastatic prostate cancer. LO provide an attractive reservoir of circulating biomarkers due to their large volume and tumour specificity. Advancements in sequencing technologies have allowed the analysis of genomic landscape of cancer using circulating cell-free DNA obtained from blood. However, one of the main challenges that remain is that this DNA does not derive only from tumour cells. Since LO are specifically released by tumour cells, we aimed to characterize DNA packaged in LO and explore its potential to report cancer-specific genomic alterations.

**Methods**: Differential and density gradient ultracentrifugation; whole genome sequencing, tunable resistive pulse sensing, western blot, pulse-field gel electrophoresis, digital PCR.

**Results**: In this study, we demonstrate that LO represent the EV population that is exquisitely enriched in chromosomal DNA up to 2 Mbp in size. Using controlled experimental conditions, we confirm reproducible recovery of known concentrations of tumour-derived DNA from circulating LO. We show that LO DNA obtained from plasma of patients with metastatic prostate cancer reaches levels of up to 100 ng/ml, while negligible amounts of DNA are present in large EV preparations obtained with the same protocol from cancer-free individuals. In addition, we develop a digital PCR assay that allows detection of copy number imbalance between MYC and PTEN, which are the most frequently amplified and deleted in metastatic prostate cancer. Using this assay, we show that LO DNA obtained from as little as 1 ml of patient plasma can report cancer-specific copy number alterations.

**Summary/Conclusion**: Our results demonstrate that circulating LO contain high molecular weight, chromatinized DNA and indicate that LO-derived DNA reflects genomic makeup of the tumour, suggesting that LO may be a valuable source of tumour-derived DNA in plasma.

**Funding**: This work was funded by National Institutes of Health NIH UCLA SPORE in Prostate Cancer award [P50 CA092131; DoD PCRP Award PC150836] (to DDV).

Symposium Session 12 - EV Characterization: State-of-the-art Approaches Chairs: Irinka Nazarenko; Rienk Nieuwland Location: Room 608:30 - 10:00

OF12.01

Gold nanoparticle ring and hole structures-based platforms for capture and label-free detection of exosomes

Duraichelvan Raju ^1^; Muthukumaran Packirisamy ^1^; Srinivas Bathini^1^; Simona Badilescu^1^; Anirban Ghosh^2^; Rodney J. Ouellette^3^

^1^Concordia University, Montreal, Canada; ^2^Department of chemistry and biochemistry, Université de Moncton, Moncton, Canada; ^3^Atlantic Cancer research Institute, Moncton, Canada

**Background**: Exosomes are considered as potential biomarkers for cancer and other pathological conditions, as well as could be used for minimal-invasive liquid-biopsy. For this reason, it is extremely important to develop methods for their capture and detection, which can be used under clinical settings. Herein, we present a simple label-free platform of gold nanoparticle ring and hole structure, using the plasmonic band of gold by using a synthetic polypeptide, called Vn96, that has strong affinity for EVs.

**Methods**: In the past, a localized surface plasmon resonance (LSPR) platform, based on gold nano-islands was developed for the capture and detection of exosomes by our group. In the present work in order to enhance the sensitivity of the detection, a new LSPR platform is investigated. The new platform is based on gold nanoparticles that form a ring structure surrounding the nanoholes with diameters in the range of 200–800 nm. The nanoring-nanohole structures fabricated by using a simple nanospheres lithography method based on polystyrene (PS) microspheres. Freshly prepared colloidal gold particles were mixed with PS suspended in DI water, an Au-PS nanocomposite, formed by self-assembly of polystyrene microspheres and gold colloids deposited on glass substrate, using vertical thermal convection technique. After annealing, the PS microspheres were removed by dissolution in an appropriate solvent. The fabrication process was optimized in terms of annealing temperature and time of immersion in the solvent.

**Results**: The ring-hole structures were imaged by scanning electron microscopy, the size distribution and the density of holes were determined. The refractive index sensitivity of the optimized platforms has been found around 300 nm/RIU and the sensing protocol for the capture and detection of exosomes has been carried out on substrates. It has been found that the ring-hole platforms, fabricated are more sensitive for the detection of exosomes. The sensitivity of structures containing small holes has been found higher and accounted for higher density of holes.

**Summary/Conclusion**: The enhanced sensitivity of the ring and holes nanostructures is explained by the preferential adsorption of exosomes on the ring-hole because of a diminished steric hindrance.

**Funding**: This work was funded by New Brunswick Innovation Foundation (NBIF), Canada and Natural Sciences and Engineering Research Council (NSERC), Canada.

OF12.02

Vibrational spectroscopy as a tool for fingerprinting tumour exosomes

Randy Carney; Kit Lam

UC Davis Medical Center University of California, Davis, Sacramento, CA, USA

**Background**: Distinguishing compositionally-unique exosome subpopulations in circulation is challenging, yet could be very useful for clinical or basic biology studies, for example, discriminating tumour-associated exosomes from healthy ones. The objective of our study is to develop tumour-specific ligands as spectral markers prior to vibrational spectroscopy analysis of exosomes and related extracellular vesicles (EVs) isolated from human plasma.

**Methods**: Using a combinatorial library-based screening methodology, our lab has recently discovered several unique peptide ligands capable of binding specific tumour cells through their overexpressed integrins (e.g. LXY30 peptide binding to a3B1 integrin). To investigate whether these ligands are capable of specific binding to the exosomes derived from those tumour cells, we have employed a combination of characterization schemes for both bulk exosomes, including flow cytometry and proteomic profiling, and also for single exosomes, including laser tweezers Raman spectroscopy and nanoparticle tracking analysis. We further expand our analyses with a custom multispectral optical tweezers platform, capable of simultaneous measurement of fluorescence and Raman spectra for single trapped vesicles. Next, surface-enhanced Raman spectroscopy (SERS) was used to detect and profile surface-bound exosomes specifically interacting with gold nanoparticle probes decorated with tumour or exosome-specific markers (e.g. LXY30, anti-CD9).

**Results**: We have measured strong binding of the peptide ligand LXY30 to integrins present on single exosomes derived from ovarian, brain, and lung tumour cells. Moreover, LXY30 shows little affinity to other types of normal cell-derived exosomes or to tumour exosomes with varying integrin profiles. With LXY30 decorated SERS-active gold nanoprobes, ovarian cancer exosomes can be accurately detected in human plasma.

**Summary/Conclusion**: We demonstrate the potential of a targeted SERS-based approach for sensing small numbers of tumour-associated exosomes amongst the normal secretome background. This methodology has the potential to transform both the understanding of compositional differences amongst circulating exosomes and also the ease in which cancer could be diagnosed.

**Funding**: This work was funded by Ovarian Cancer Education and Research Network (OCERN) Research Grant

OF12.03

“None of us is the same as all of us”: nanoscale probing of heterogeneity of stem-cell derived extracellular vesicles by resonance enhanced atomic force microscope infrared spectroscopy

Sally Yunsun Kim^1^; Dipesh Khanal^1^; Bill Kalionis^2^; Wojciech Chrzanowski ^3^

^1^The University of Sydney, Sydney, Australia; ^2^The Royal Women’s Hospital, Parkville, Australia, Melbourne, Australia; ^3^The University of Sydney, Camperdown, Australia

**Background**: Extracellular vesicles (EVs) are specialized, nanoscale messengers that deliver biological signals. The evidence shows that within populations of EVs, important properties including morphology, composition and content vary substantially. Thus, measuring EV heterogeneity is paramount to our understanding of how EVs influence physiological and pathological functions of their target cells. Thus far, devising effective methods for measuring EV heterogeneity remains a global challenge.

**Methods**: We present, for the first time, a study of the molecular and structural composition of individual EVs, subpopulations of EVs and whole populations of EVs using resonance enhanced atomic force microscope infrared spectroscopy (AFM-IR). This approach is label-free, has ultra-high sensitivity and has the power to measure EV heterogeneity. EVs were isolated from placenta stem cells using ultrafiltration and after further purification using the additional size-exclusion chromatography column and both methods were compared.

**Results**: We demonstrated for the first time the possibility to characterise individual EV at nanoscale, EV populations and showed the critical differences in their composition depending on extraction protocols - heterogeneity.

Ultra-high resolution of AFM-IR that allows probing of multiple points on individual EVs is key to develop new extraction and separation protocols for EVs and to unlock their full therapeutic and diagnostic potential. Our approach outperforms other methods for vesicles characterization providing unmatched resolution (single vesicle) and is “probe free”, thus it avoids bias and resolution limitations of molecular probes.

**Summary/Conclusion**: The AFM-IR is advancing the EV field forward by revealing their molecular constituents and structures, as well as enabling purity assessment of EV preparations. The data presented in this study suggest AFM-IR can transform existing protocols for interrogating EV composition and structures, and assessing EV purity. This nanoscale technique can be developed into a powerful screening tool for detecting specific EV “fingerprints” that are associated with pathology by correlating the structural differences to biomarkers, addressing unmet clinical needs in diseases where early diagnosis is critical, for example multiple sclerosis or cancer.

OF12.04

Membrane protein quantification on extracellular vesicles by surface plasmon resonance imaging and time-resolved fluorescence immunoassay

Elmar Gool^1^; Frank A.W Coumans ^2^; Janne Leivo^3^; Mirella Vredenbregt - van den Berg^4^; Auguste Sturk^5^; Ton G. van Leeuwen^2^; Rienk Nieuwland^5^; Guido W. Jenster^4^

^1^Department of Biomedical Physics and Engineering (BMEP) & Department of Clinical Chemistry (LEKC) Academic Medical Center, Amsterdam, The Netherlands; ^2^Department of Biomedical Engineering and Physics, and Vesicle Observation Center, Academic Medical Centre of the University of Amsterdam, Amsterdam, The Netherlands; ^3^Department of Biochemistry and Food Chemistry University of Turku, Turku, Finland; ^4^Department of Urology Erasmus Medical Center, Rotterdam, The Netherlands; ^5^Laboratory of Experimental Clinical Chemistry, and Vesicle Observation Center, Academic Medical Center, University of Amsterdam, Amsterdam, The Netherlands

**Background**: Detection of transmembrane proteins on extracellular vesicles (EVs) is typically performed using Western blot or enzyme-linked immunosorbent assay. However, both techniques have limited analytical sensitivity and quantification abilities. Recently, more sensitive and quantitative methods have become available, including surface plasmon resonance imaging (SPRi) and time-resolved fluorecence immunoassay (TRFIA).

**Methods**: Both SPRi and TRFIA capture target-exposing EVs at an antibody-coated surface. SPRi detects a change in refractive index upon capture of EVs, whereas TRFIA detects captured EVs through labeling with an europium-conjugated antibody. CD9 exposure was determined qualitatively and quantitatively for 16 culture-derived EV samples by SPRi and TRFIA.

**Results**: For 11 EV samples (69%), qualitative detection of CD9 with SPRi and TRFIA was in agreement. The quantitative signal amplitudes of all EV samples showed, however, a R2-correlation of 0.09. A cause of discrepancy is the 80%–95% reduction in labeling intensity, when capture and labeling are performed in TRFIA with the same antibody (CD9, CD63 and EpCAM), which was confirmed with fluorescence microscopy for EpCAM. Another cause of discrepancy occurs during labeling of captured EVs by TRFIA. This labeling depends on the antigen density whereas detection by SPRi does not. Thus, samples containing a subpopulation of EVs with high numbers of antigens were positive in TRFIA but not in SPRi.

**Summary/Conclusion**: To conclude, SPRi and TRFIA gave comparable qualitative phenotyping results, but incomparable quantitative results due to (1) competition between capture and labeling antibody in TRFIA when the same antibody is used, and (2) a non-linear relationship between refractive index-based and labeling-based detection. Our results indicate that results of different quantitative phenotyping techniques need to be addressed with care. Therefore, we recommend to translate the results into average antigen density on detected EVs to enable the comparison of results.

**Funding**: This work was supported by the Cancer-ID perspectief program of NWO Applied and Engineering Sciences [Project #14197].

OF12.05

Proximity assays for detection and characterization of exosomes

Ehsan Manouchehri; Alireza Azimi; Qiujin Shen; Masood Kamali-Moghaddam

Department of Immunology, Genetics and Pathology, IGP Uppsala University, Uppsala, Sweden

**Background**: Despite the large number of technologies currently used to detect and characterize exosomes in biofluids, the need remains for improved methods. The flow cytometry-based methods for quantitative and qualitative characterization of exosomes, for instance, meet challenges such as the small size of the exosomes, paucity of antigen molecules present on the surface of the exosomes, making it difficult or impossible to distinguish individual exosomes from background by conventional flow cytometry.

**Methods**: We have applied the proximity ligation assay in combination with flow cytometry readout for sensitive and specific detection of individual exosomes. Here, the exosomes are first enriched on a solid support using a capture antibody - immobilized via a cleavable DNA molecule. Subsequently, the exosomes are probed with a set of proximity probes, each consisting of an affinity binder conjugated to a ssDNA molecule. Prior to the signal amplification via rolling signal amplification, the exosomes are released from the solid support by DNA cleavage, allowing multicolour detection and measurement of individual exosomes in a flow cytometer.

**Results**: The use of up to seven antibodies in combination with signal amplification allows detection of exosomes with high specificity and sensitivity. By using different reporting fluorophores for each pair of probes, a specific exosome population may be distinguished from all other exosomes in complex matrices such as in blood plasma.

**Summary/Conclusion**: Here, we discuss the application of proximity assays to analyse individual exosomes and the potential of such approach to be used to identify exosomes as disease biomarkers.

OF12.06

Individual EV visualization using TIRF microscopy for EV subpopulation study

Chungmin Han ^1^; Siwoo Cho^2^; Wonju Jo^2^; Jaesung Park^2^

^1^Pohang University of Science and Technology, Pohang, Republic of Korea; ^2^POSTECH, Pohang, Republic of Korea

**Background**: Extarcellular vesicles are cell-secreted particles that contain various biological substances such as phospholipids, proteins and nucleic acids. Based on previous findings, EVs showed heterogeneity not only in physical properties but also in their contents and biogenesis processes. However, current characterization methods could not elucidate the diversity of individual EVs and subpopulations. In this research, we developed an individual EV visualization method and revealed subpopulations exist in heterogeneous plasma EVs.

**Methods**: We established the method based on the knowledge that EVs contain both proteins and lipids. To visualize them, surface proteins of EVs were biotinylated for the immobilization on a quartz surface and lipophilic tracers were adopted to label the lipid components. To improve the quality of the image, we PEGylated the imaging surface. Due to this PEGylated surface, we can also efficiently immune-label the immobilized EVs without time-consuming washing steps.

**Results**: Using this method, we can successfully observe well-isolated signals from individual EVs. Among the various labeling options, immune and lipid labeling showed superior image qualities. From dual labeling (immune & lipid labeling) signal analysis, we confirmed that only a portion of EVs express the tetraspanin markers (CD9, 63, 81) and a substantial amounts of lipoprotein markers were also detected. From the dual immune labeling signal analysis, we also observed the correlations between various marker expressions.

**Summary/Conclusion**: We developed an individual EV visualization method using TIRF based single molecule co-localization technique. We immobilized EVs by surface protein biotinylation and fluorescently visualized EVs by lipid or immune labeling. As a result, we can obtain clear and well-isolated signals from individual EV particles. Further analysis of the obtained signals provides us with information about EV subpopulations.

**Funding**: This research was funded by the Ministry of Health and Welfare, Republic of Korea [grant no. HI16C2221 and grand no. HI16C0665].

Plenary Session 2: Advances in Exosomes Biology Chairs: Antonio Marcilla; Marca Wauben Location: Auditorium 10:30 - 11:45

PL 3

Sorting of small RNAs into EVs secreted by human cells

R. Schekman^1^; M. Shurtleff^1^; M. Temoche-Diaz^1^; J. Yao^2^; Y. Qin^2^; A. Lambowitz^2^

^1^Department of Molecular and Cell Biology and Howard Hughes Medical Institute University of California, Berkeley, Berkeley, CA. 94720 USA; ^2^Department of Molecular Biosciences University of Texas at Austin Austin, TX 78712 USA

Highly purified EVs isolated from human cell lines display a small number of substantially (~ 1000 fold) enriched miRNAs that differ from one cell line to another. In spite of the small number of such species, no single RNA sorting sequence is evident. In order to explore the mechanism of RNA sorting, we established a cell-free reaction that reproduces the selective incorporation of synthetic, mature miRNAs (miR223 and miR122) into vesicles formed in a reaction containing membranes and cytosol from mechanically disrupted HEK293 cells. The sorting reaction requires both membrane and cytosol and is stimulated by hydrolysable ATP and incubation at a physiologic temperature. Using biotinlyated derivatives of two different miRNAs, we found different sets of RNA binding proteins incorporated along with each species, among which the proteins Ybx1 and Lupus La are required to sort mir223 and miR122, respectively. EVs also contain more abundant major species of small RNA including full-length tRNA, Y-RNA and vault RNA, and each requires the Ybx1 protein for selective sorting into exosomes secreted by cells and into vesicles in the cell-free reaction. tRNAs in EVs appear to have a distinct chemical modification that is much less abundant in tRNAs in HEK293 cells. This modification may be involved in tRNA sorting or in the function of tRNA transported to a target cell.

Shurtleff MJ, Temoche-Diaz MM, Karfilis KV, Ri S, Schekman R (2016). Y-box protein 1 is required to sort microRNAs into exosomes in cells and in a cell-free reaction. Elife. Aug 25;5. pii: e19276. doi: 10.7554/eLife.19276. PMID: 27559612

Shurtleff, M., Yao, J., Qin, Y, Nottingham, R., Temoche-Diaz, M., Schekman, R and Lambowitz, A. (2017). A broad role for YBX1 in defining the small non-coding RNA composition of exosomes PNAS 2017 October, 114 (43) E8987-E8995. https://doi.org/10.1073/pnas.1712108114

Shurtleff, M., Temoche-Diaz, M. and Schekman, R. (2018). Extracellular Vesicles and Cancer: Caveat Lector. Ann. Rev. Cancer Biology https://doi.org/10.1146/annurev-cancerbio-030617-050519

PL 4

Advances on the cell biology of extracellular vesicles

Graca Raposo

Institut Curie, Paris, France

Extracellular vesicles (EVs) comprise a heterogeneous group of cell-derived membrane structures, so called exosomes and microvesicles. They originate from the endosomal system and are secreted after fusion of endosomes with the plasma membrane (exosomes) or are shed from the plasma membrane (Microvesicles). EVs are present in biological fluids and are involved in multiple physiological and pathological processes. EVs constitute a mechanism for intercellular communication, allowing cells to exchange proteins, lipids and genetic material influencing on cellular functions. Knowledge of the cellular processes that govern extracellular vesicle biology is essential to shed light on the physiological and pathological functions of these vesicles as well as on clinical applications involving their use and/or analysis. Over the past years knowledge have been acquired regarding the origin, biogenesis, secretion, targeting and fate of these vesicles which would be an asset to modulate their function in vivo. However there are still many unknowns that deserve future investigations.

Featured Abstracts - Session 1 Chair: Clotilde Thery Location: Auditorium 11:45 - 12:20

FA1.01

Effect of tetraspanin-blocking peptides in the biogenesis of exosomes in melanoma cells

Carla Mazzeo^1^; Zoraida Andreu^1^; Soraya López^1^; Victor Toribio^1^; Susana García Silva^2^; Begoña Hurtado^2^; Héctor Peinado^2^; María Yañez^1^

^1^Centro de Biología Molecular “Severo Ochoa” CSIC/UAM, Madrid, Spain; ^2^Spanish National Cancer Research Centre (CNIO), Madrid, Spain

**Background**: To address the role of tetraspanins in exosome biogenesis overcoming compensation mechanisms that occur in tetraspanin-deficient animals, we have analysed the effect of previously characterized blocking peptides that functionally mimic the effects of tetraspanin knockdown combined with genetic deletion by the CRIPSR/Cas9 system in melanoma cells.

**Methods**: A metastasizing melanoma cell line was treated for 7d with cytopermeablepeptides that functionally mimic the effects of CD9orCD63 tetraspanin knockdown. In addition, CD9gene was deleted from this cell line using the CRISPR/cas9 system. A detailed quantification of exosome secretion was performed by combining flow cytometry with NTAanalyses. Exosome morphology and the different maturation steps of MVBwere analysed by electron microscopy and immunofluorescence of appropriated markers. The composition of exovesicles obtained from cell cultures subjected to the different treatments was determined by a proteomic approach using iTRAQ. To study the metabolic phenotype (respiration capacity as well as the levels of glycolysis) we employed the Seahorse XF CellMitoStressTest. Finally, we analysed the therapeutic potential of the blocking peptides in a xenograph model of melanoma in mice.

**Results**: Our data reveal that blocking either tetraspanin CD63orCD9 or deleting CD9gene by the CRISPR/Cas9system results in a clear reduction in exosome secretion. The remnant EVs obtained in the supernatant of treated cells are of bigger size and different composition (enriched in ECM components). Characterization of the MBV maturation in treated cells revealed different alterations in the endolysosomal system. Blocking CD9 resulted in a depletion of MVB and an increase in lysosomes. Unexpectedly, these alterations in the endolysosomal system are accompanied by a clear reduction in cell proliferation reduction of the glycolytic capacity and an increase in the number of mitochondria in the cell. *In vivo*, intratumour injection of the blocking peptides reduces tumour burden and the size of metastasis.

**Summary/Conclusion**: Our data suggest that blocking tetraspanin function alters the maturation of MVB inducing a metabolic shift in tumour cells with a promising therapeutic potential.

**Funding**: This work was supported bygrants from Fundación BBVA, FundaciónRamónAreces and BFU2014-55478-R and Network of Excellence in the Research and Innovation on Exosomes REDIEX. MEyC [SAF2015-71231-REDT].

FA1.02

Mechanisms for the exosomal secretion and transmission of α-synuclein in the brain

Jasn Howitt ^1^; Ley-Hian Low^2^; Ulrich Sterzenbach^3^; Seong-Seng Tan^3^

^1^Department of Health and Medical Sciences Swinburne University, Melbourne, Australia; ^2^Department of Neurology University California, San Francisco, CA, USA; ^3^Florey Institute of Neuroscience and Mental Health, Melbourne, Australia

**Background**: Recent evidence implicates the transmission of α-synuclein within the brain as a pathway involved in the pathogenesis of Parkinson’s disease. However, little is known about the initial cellular events that result in the propagation of pathology associated with Parkinson’s disease.

**Methods**: Cell culture was used to identify the mechanism involved in the exosomal release of α-synuclein. *In vivo* studies were conducted with; (1) wild type, (2) M83 α-synuclein over-expressing mice and (3) α-synuclein knockout mice. Exosomes with or without α-synuclein were nasally delivered to mice and after four months the animals underwent behavioural testing before analysis of brain tissue.

**Results**: We have identified a mechanistic pathway involving ubiquitination of α-synuclein that results in exosomal packaging and release from cells. *In vivo*, we administered exosomes via nasal delivery, a system we have previously identified to deliver functional exosomes to the brain. In both wild type and α-synuclein transgenic mouse brains we observed Lewy body-like aggregates after delivery of exosomes containing α-synuclein. Delivery of control exosomes did not result in brain aggregates, similarly, delivery of α-synuclein containing exosomes to α-synuclein knockout mice did not result in brain aggregates. Behavioural testing showed that animals given α-synuclein containing exosomes had movement deficits in their hind limbs, whereas animals given control exosomes or α-synuclein exosomes to knockout mice did not display any behavioural deficits.

**Summary/Conclusion**: Here we identified a mechanistic pathway for the packaging of α-synuclein into exosomes and show that these exosomes are able to propagate aggregated forms of the protein to the brains of rodents. These findings show how exosomes can transmit α-synuclein in the brain resulting in Lewy body-like aggregates and movement deficits that are found in Parkinson’s disease.

**Funding**: This work was funded by NHMRC project grants awarded to J Howitt.

Symposium Session 13 - Role of Tumour EVs in Cell-Cell Communication Chairs: Antonella Bongiovanni; Hector Peinado Location: Auditorium 13:45 - 15:15

OF13.01

Computer guided image analysis of nuclear membrane instability in tissues reveals clinical relevance for nucleus-derived EVs

Tatiana Novitskya^1^; Adel Eskaros^1^; Mariana Reis-Sobreiro^2^; Michael R Freeman^2^; Dolores Di Vizio^2^; Andries Zijlstra^1^

^1^Department of Pathology, Microbiology and Immunology, Vanderbilt University Medical Center, Nashville, TN, USA; ^2^Departments of Surgery, Biomedical Sciences, and Pathology and Laboratory Medicine, Cedars-Sinai Medical Center, Los Angeles, CA, USA

**Background**: Although it is well established that oncogenic transformation causes cells to shed a heterogeneous population extracellular vesicles (EV), reliable methods for evaluating and quantifying the biogenesis of EV in patient tissue have been lacking. In prior studies of prostate cancer, we observed extensive EV shedding and enhanced malignant behaviour in cancer cells that exhibit nuclear instability. Nuclear blebbing and shedding of EV containing genomic material could be detected in tumour tissue from experimental models of nuclear membrane instability generated by depletion of the cytoskeletal regulator DIAPH3 or nuclear lamin A/C. To determine the clinical significance of this mechanism in prostate cancer, we developed a novel approach to the quantitative analysis of EV production in formalin-fixed paraffin-embedded clinical tissues. 

**Methods**: To visualize release of nucleus-derived particles, multiplex immunofluorescent detection of nuclear histone, DNA and nuclear envelope (Emerin) together with the epithelial cytokeratin (CK18) was performed on a tissue microarray containing tumour, adjacent benign and metastatic LN tissue (*n* = 80). Machine learning was leveraged, for the first time, to develop an image analysis pipeline that enabled single-cell segmentation and quantitation of nucleus-derived EV associated with nuclear membrane instability.

**Results**: Nucleus-derived EV was evident in ≥50% of prostate cancer patients and ≥80% of tumour-involved lymph nodes. Intra-patient differences in particle size, location and enumeration suggest that significant variation in the mechanisms of biogenesis may exist. Most importantly the number of nucleus-derived particles corresponded with disease recurrence as detected by elevation of prostate-specific antigen after surgery. Their relevance in disease progression was supported by experimental xenograft models of nuclear membrane instability that exhibit increased tumour cell motility and metastasis.

**Summary/Conclusion**: Taken together, these results not only reveal a correlation between EV biogenesis and patient outcome in prostate cancer but also provide the first proof-of-principle for the ability of computer-assisted image processing to visualize and investigate EV biogenesis in human tissue.

OF13.02

3D culture modelling illustrates a role for EVs in mediating the biomechanics of the tumour microenvironment

Roslyn Williams^1^; Nicola Wright^2^; Stuart Hunt^1^; Robin Delaine-Smith^3^; Martin Knight^3^; Bhome Rahul^4^; Alex Mirnezami^4^; Paul Hughes^5^; Nicholas Peake^2^

^1^University of Sheffield, Sheffield, UK; ^2^Sheffield Hallam University, Sheffield, UK; ^3^Queen Mary, University of London, Sheffield, UK; ^4^University of Southampton, Southampton, UK; ^5^University of Durham, Durham, UK

**Background**: It is now well established that the biomechanical properties of cancer tissue have an important role in determining the progression of disease. Enhanced synthesis, remodelling and cross-linking of extracellular matrix, mediated primarily by stromal fibroblasts, leads to tissue stiffening which drives pro-oncogenic biomechanical signalling in invading cancer cells. Extracellular vesicles (EVs) play an important role in mediating cross-talk between cancer cells and fibroblasts. In this work, we used 3D culture models to assess the effect of biomechanical culture conditions on EV synthesis, and the impact of cancer-derived EVs on biomechanical conditions within an organotypic *in vitro* environment.

**Methods**: Primary colonic fibroblasts in monoculture or co-culture with the colorectal cancer cell line SW480 were established in a collagen gels, and mechanical properties defined by unconfined compressive testing. In addition, SW480 cells were cultured in mechanically tailored alginate beads, and EVs collected by ultracentrifugation. EV properties were characterized by nanoparticle tracking analysis. Protein analysis was performed by Western blot and RNA analysis by qRT-PCR.

**Results**: Our results showed that colon fibroblasts mediate the biomechanical properties of collagen cultures through contractile and remodelling processes, and co-culture with SW480 cells drives this activity. A role for the protein cross-linking enzyme transglutaminase-2 (TG2) in mediating the biomechanical environment was identified using siRNA. Fibroblast-derived TG2 inhibited cancer spheroid growth, and loss of TG2 was observed in cancer-associated fibroblasts compared to normal fibroblasts. Using mechanically tailored alginate, we found that biomechanical conditions determined SW480 EV properties, with 3D culture leading to significantly enhanced release and altered size profile. Culture conditions also impacted on levels of a key regulator of TG2, miR-19. Finally, we observed that SW480 EVs significantly altered the contractile function of fibroblasts and the biomechanical properties of collagen cultures, reflecting miR-mediated targeting of TG2 by colorectal cancer-derived EVs.

**Summary/Conclusion**: EVs are responsive to, and mediators of, the biomechanical tumour microenvironment.

**Funding**: This work was funded by Bowel & Cancer Research, UK.

OF13.03

Melanoma-derived microvesicles are taken up by lymph node resident macrophages and lymphatic endothelial cells and induce lymph node remodeling

Alessandro Gallo^1^; Noelle Leary^1^; Héctor Peinado^2^; Lothar Dieterich ^1^

^1^Institute of Pharmaceutical Sciences, ETH Zürich, Switzerland; ^2^Microenvironment and Metastasis Group, Molecular Oncology Programme, Spanish National Cancer Research Centre (CNIO), Madrid, Spain

**Background**: Tumour-derived extracellular microvesicles (EVs) released into the interstitium are taken up by associated lymphatic vessels (LVs) and transported to draining lymph nodes (LNs). However, only few studies have addressed the fate and function of tumour EVs in the lymphatic system so far. Our aim was to track EV transport to, and cellular uptake within, draining LNs, and to determine their effects on LN expansion and remodeling that are typical for tumour-draining LNs.

**Methods**: EVs were purified from B16F10 melanoma supernatants by size exclusion chromatography (SEC), characterized and labeled fluorescently. For *in vivo* uptake studies in mice, EVs were injected into the hind paw, and draining LNs (popliteal, sacral and inguinal) were analysed by FACS. EV uptake by relevant cell populations was also confirmed *in vitro*. LN remodeling was evaluated macro- and microscopically after three consecutive daily EV injections.

**Results**: SEC yielded highly pure EVs with a mean diameter of 100–120 nm. Interstitial injection of EVs into the hind paw of mice resulted in rapid transport to popliteal LNs, where EV-associated fluorescence could be detected within lymphatic sinuses as early as 2 h after injection. EVs were predominantly taken up by CD169+ LN macrophages as well as by LECs, but not by dendritic cells or lymphocytes. EV uptake by bone marrow-derived macrophages and cultured LECs could also be confirmed *in vitro*. Furthermore, EVs remained in the first draining (popliteal) LNs, and were never detected in secondary (sacral) LNs. Interestingly, repeated EV injection resulted in a massive expansion and remodeling of popliteal LNs.

**Summary/Conclusion**: Our data show that melanoma-derived EVs are efficiently drained from the interstitium by LVs, transported to draining LNs, and are taken up by LN macrophages and LECs, leading to LN swelling and remodeling. This suggests that melanoma-associated LN remodeling is at least partly caused by EVs drained from the primary tumour.

**Funding**: This work was funded by ETH Zürich Career Seed Grant and Krebsliga Zürich Research Grant

OF13.04

GD3 ganglioside-enriched extracellular vesicles stimulate melanocyte migration

Andreia H. Otake ^1^; Ana Paula Marques Duarte^2^; Renata de Freitas. Saito^1^; Alexandre Ferreira Ramos^2^; Roger Chammas^1^

^1^Institute of Cancer of the University of Sao Paulo (ICESP); ^2^Faculty of Medicine (FMUSP), USP, Brazil, Sao Paulo, Brazil

**Background**: Gangliosides are a family of sialic acid-containing glycosphingolipids that are located mainly in the outer leaflet of plasma membranes. Melanocytes express mainly the monosialoganglioside G_M3_ and upon their transformation into melanomas, they accumulate more complex gangliosides - such as the disialoganglioside G_D3_ and its derivatives. G_D3_-synthase is the sialyltransferase responsible for the conversion of G_M3_ into G_D3_ and its expression or activity are altered in several tumours, including melanomas.

**Methods**: We have transfected the GD3 synthase gene (ST8Sia I) in a normal melanocyte cell line in order to evaluate changes in the biological behaviour of non-transformed cells.

**Results**: GD3-synthase expressing cells converted GM3 into GD3 and accumulated both GD3 and its acetylated form, 9-O-acetyl-GD3. Melanocytes were rendered more migratory on laminin-1 surfaces. Cell migration studies using the different transfectants, either treated or not with the glucosylceramide synthase inhibitor D-1-threo-1-phenyl-2-palmitoylamino-3-pyrrolidino-1-propanol (PPPP), allowed us to show that while GM3 is a negative regulator of melanocyte migration, GD3 increases it. Removal of cell surface cholesterol abrogated the inhibitory effects of GM3. GD3 and 9-O-acetyl-GD3 gangliosides co-localized with integrins in cell lamellipodia, but not in uropods. We showed that gangliosides were shed to the matrix by migrating cells and that GD3 synthase transfected cells shed extracellular vesicles (EVs) enriched in GD3. EVs enriched in GD3 stimulated cell migration of GD3 negative cells, as observed in time lapse microscopy studies. Otherwise, EVs shed by GM3+ veGD3-ve cells impaired migration and diminished cell velocity in cells overexpressing GD3.

**Summary/Conclusion**: The balance of antimigratory GM3 and promigratory GD3 gangliosides in melanocytes could be altered by horizontal transfer of ganglioside enriched extracellular vesicles. This study highlights that extracellular vesicles transfer biological information not only through their cargo, but also through their membrane components, which include a variety of glycosphingolipids remodeled in disease states such as cancer.

**Funding**: This work was supported by Fundação de Amparo à Pesquisa do Estado de São Paulo [FAPESP, 1998/14247-6, 2001/01416-9, 2014/03742-0].

OF13.05

Exosomal heparan sulphate proteoglycans (HSPGs) drive induction of a pro-angiogenic stromal cell phenotype

Alex P. Shephard; Zsuzsanna Tabi; Rachel Errington; Aled Clayton; Jason P. Webber

Tissue Microenvironment Group, Division of Cancer and Genetics, School of Medicine, Cardiff University, Cardiff, UK

**Background**: We have previously demonstrated that prostate cancer exosomes drive TGFβ-dependent differentiation of stromal fibroblasts to a pro-angiogenic disease supporting phenotype. Furthermore, these studies implicated a role for heparan sulphate glycosaminoglycans in exosome mediated TGFβ delivery. Here we explore the role of specific exosome-associated heparan sulphate proteoglycans (HSPGs) in activation of TGFβ signalling and the regulation of both fibroblast differentiation and angiogenic function.

**Methods**: HSPG-deficient prostate cancer cells (Du145) were generated using shRNAs to target specific HSPGs. Fibroblasts were stimulated with either control or HSPG-deficient exosomes, prior to culture with human endothelial cells (HUVECs). Formation of vessel like structures was visualized by CD31 staining. Conditioned media and mRNA from exosome treated fibroblasts were analysed for growth factors including HGF, VEGF and TGFβ. Luciferase reporter assays were used to analyse the signalling pathways involved, with fibroblasts transfected with a SMAD reporter plasmid prior to stimulation with control or HSPG-deficient exosomes.

**Results**: We have successfully generated stable prostate cancer cell lines that secrete exosomes lacking specific HSPGs. Exosomes deficient in syndecan 3, syndecan 4, glypican 1, glypican 6 or betaglycan were unable to induce SMAD-dependent TGFβ signalling and showed attenuated ability to drive stromal cell differentiation. Secretion of angiogenic factors by stromal cells was also reduced, resulting in an attenuated ability of fibroblasts to support the formation of vessel-like endothelial structures.

**Summary/Conclusion**: Exosome-induced differentiation of fibroblasts to a pro-angiogenic phenotype is dependent on specific HSPGs present on the exosome surface. HSPGs are required for exosome activation of SMAD-dependent TGF-β signalling. Exosomal-HSPGs may therefore represent novel targets for attenuation of fibroblast-assisted tumour growth.

**Funding**: This work was funded by Prostate Cancer UK - Career Development Fellowship (held by Dr J Webber)

OF13.06

CD44 is a novel homing receptor for extracellular vesicles

Kai Härkönen ^1^; Silja Pyysalo^1^; Sini Hakkola^1^; Kirsi Ketola^1^; Carla Oliveira^2^; Sanna Oikari^1^; Kirsi Rilla^1^

^1^Institute of Biomedicine, University of Eastern Finland, Kuopio, Finland; ^2^i3S - Instituto de Investigação e Inovação em Saúde, Universidade do Porto, Porto, Portugal

**Background**: The surface molecular composition of extracellular vesicles (EV) is the most important feature regulating EV adhesion and receptor-ligand interactions with the target cells. The multifunctional adhesion molecule and principal hyaluronan (HA) ligand CD44 is one of these surface receptors binding also to other extracellular matrix components including collagen, fibronectin, and laminin. HA-CD44 interactions mediate the recruitment of activated leucocytes stem cells and tumour cells from the circulation which makes CD44 known as a “homing receptor”. The bonds between HA and CD44 are remarkably strong, which provides resistance to shear during adhesion of lymphocytes on endothelial cells.

**Methods**: Here, we hypothesized that these same mechanisms of HA-CD44 interactions regulate the homing of EV to reprogram other cells and to prepare a favourable niche for metastasis of cancer cells. To answer this hypothesis, we utilized a CD44-negative human gastric cancer line MKN74 stably expressing CD44 standard form and compared them to cells expressing empty vector pIRES-EGFP2 (MOCK). First, we confirmed the CD44 expression of these cell lines by CD44 immunostainings, western blotting, ELISA and QPCR. Next, the secretion and size distribution of EV secreted by both cell lines was analysed by NTA analysis, and the potential of EV binding to target cells was studied by superresolution microscopy.

**Results**: The results indicated that the MOCK cells have low HA binding capacity compared to the CD44 overexpressing cells. In addition, the NTA results showed no differences in EV secretion of CD44-negative and overexpressing cells. These results suggest that CD44 regulates EV interactions with their target cells.

**Summary/Conclusion**: Further studies will show the more detailed mechanisms of these interactions. Furthermore, CD44 and HA are potential multipurpose EV biomarkers, because they are upregulated in inflammatory, injured and cancer cells and accumulate on the surface of EV secreted in these situations.

**Funding**: This study is funded by Academy of Finland.

Symposium Session 14 - Tissue Injury and Repair Chairs: Bernd Giebel; Mariko Ikuo Location: Room 5 13:45 - 15:15

OF14.01

Human neural stem cell extracellular vesicles improve recovery in a porcine model of ischemic stroke

Robin Webb ^1^; Erin E. Kaiser^2^; Brian J. Jurgielewicz^2^; Samantha Spellicy^2^; Shelley Scoville^1^; Tyler Thompson^1^; Raymond L. Swetenburg^1^; Franklin West^2^; Steven Stice^2^

^1^ArunA Biomedical, Athens, GA, USA; ^2^Regenerative Bioscience Center, University of Georgia, Athens, GA, USA

**Background**: Recent work from our group suggests that human neural stem cell-derived extracellular vesicle (NSC EV) treatment improves both tissue and sensorimotor function in a preclinical thromboembolic mouse model of stroke. The objective of the current study was to evaluate the therapeutic potential of NSC EVs in the stroked porcine brain which, like the human brain, is gyrencephalic and contains more than 60% white matter. Preclinical efficacy of NSC EV was evaluated through both magnetic resonance imaging (MRI) and longitudinal assessment of behaviour and motor function.

**Methods**: Ischemic stroke was induced by permanent middle cerebral artery occlusion (MCAO), followed by intravenous treatment with either NSC EV or PBS at 2, 14, and 24 h post-stroke. Tissue level recovery was evaluated via MRI at 1 and 84 days post-stroke. Functional and behavioural recovery was assessed longitudinally using open field testing and gait analysis.

**Results**: NSC EV treatment was neuroprotective and led to significant improvements over PBS treatment at the tissue and functional levels. Twenty four hours post-stroke, intracranial hemorrhage was eliminated in ischemic lesions in NSC-EV treated pigs (0/7) vs. PBS-treatment (7/8). Both cerebral infarct volume and brain swelling were decreased after NSC EV treatment relative to PBS treatment. Apparent diffusion coefficient maps indicated that edema was significantly reduced after NSC EV treatment compared to PBS. Fractional anisotropy of the corpus callosum at 84 days post-MCAO demonstrated improved white matter integrity in NSC EV-treated animals. Behaviour and mobility improvements paralleled structural changes, as NSC EV-treated pigs exhibited improved outcomes including increased exploratory behaviour and faster restoration of spatiotemporal gait parameters.

**Summary/Conclusion**: NSC EV treatment led to significant improvements over PBS treatment at both tissue and functional levels. NSC EV efficacy in a large animal stoke model suggests unique therapeutic tropism may be derived from use of unaltered tissue specific EVs.

**Funding**: This work was supported by ArunA Biomedical, Inc., NINDS [grant R43NS103596], Science and Technology Center Emergent Behaviors of Integrated Cellular Systems (EBICS) [Grant No. CBET-0939511], and the Georgia Research Alliance.

OF14.02

Hypoxia modifies the release of extracellular vesicles by mesenchymal cells improving renal recovery after ischemia-reperfusion injury

Federica Collino^1^; Teby da Silva^1^; Jarlene Lopes^1^; Stephany Corrêa^2^; Camila Wendt^1^; Kildare Miranda^1^; Eliana Abdelhay^2^; Christina Takiya^1^; Adalberto Vieyra^1^; Rafael S. Lindoso ^1^

^1^Carlos Chagas Filho Biophysics Institute (IBCCF) Federal University of Rio de Janeiro, Rio de Janeiro, Brazil; ^2^Cancer National Institute - INCA, Rio de Janeiro, Brazil

**Background**: Extracellular vesicles (EVs) composition depends on the cell of origin and its state. Hypoxia has been described to alter the paracrine profile of mesenchymal cells and may alter EVs composition and their effects. In this work, we investigated the role of EVs secreted by human-adipose-derived-stem-cells (hADMSC) submitted to hypoxia in renal recovery.

**Methods**: Cell culture: hRPTC (HK-2) (ATCC) were cultivated with K-SFM. hADMSC were cultivated with ADSC™ Growth Medium (both from Lonza).

EV isolation: Supernatant of hADMSC culture maintained for 72 h in normoxia or hypoxia (1% O2) condition was centrifuged at 3000 g, followed by an ultracentrifugation of 100,000 g for 2h. EVs were characterized by Nanoparticle tracking (NanoSight LM10), flow cytometry and electron microscopy.

Injury model: Male Wistar rats were submitted to bilateral ischemia for 45 min, followed by renal subcapsular administration of EVs during reperfusion period (72 h). Histological and functional analyses were performed. *In vitro* model: hRPTC were incubated with antimycin A, leading to ATP depletion. EVs were then incubated with hRPTC for 24 h.

Proteomic analysis: Qualitative and quantitative nano-ultra-high pressure chromatography (nanoUPLC) tandem nanoESI-HDMSE experiments were conducted with a nanoACQUITY UPLC system.

**Results**: hADMSC submitted to hypoxia presented an increase in the secretion of EVs. In addition, hypoxic-EVs promoted better renoprotective effects such as reduction of apoptosis, and inflammation and protection of tissue architecture when compare with normoxic-EVs. Proteomic analysis revealed that hypoxic-EVs triggered different responses in renal cells associated with energy metabolism and cell survival.

**Summary/Conclusion**: The present data showed that hypoxia can alter EVs secretion and such modifications resulted not only in a better outcome but also triggered different pathways in the renal recovery process. These results indicate that hypoxia may be an interesting strategy for kidney diseases treatment.

**Funding**: This work was funded by National Institute of Science and Technology for Regenerative Medicine REGENERA; Brazilian National Research Council; Carlos Filho Rio de Janeiro State Research Foundation.

OF14.03

Human induced pluripotent stem cell extracellular vesicles trigger a miRNA-dependent anti-inflammatory mechanism to tackle ischemia

Mario Barilani ^1^; Francesca Polveraccio^2^; Francesca Pischiutta^3^; Elisa Zanier^4^; Valentina Bollati^1^; Vincenza Dolo^5^; Lorenza Lazzari^2^

^1^EPIGET LAB, Department of Clinical Sciences and Community Health, Università degli Studi di Milano, Milan, Italy; ^2^Cell Factory, Laboratory of Regenerative Medicine, Department of Services & Preventive Medicine, Fondazione IRCCS Ca’ Granda Ospedale Maggiore Policlinico, Milan, Italy, Milan, Italy; ^3^IRCCS Mario Negri, Milan, Italy, Milan, Italy; ^4^IRCCS Mario Negri, Milan. Italy, Milan, Italy; ^5^Department of Life, Health and Environmental Sciences, University of L’Aquila, L’Aquila, Italy

**Background**: Human induced pluripotent stem cells (hiPSC) are considered cell therapy candidates for their unlimited differentiation capacity and lifespan. Currently, mesenchymal stem cells (MSC) are the short-lived cell type most used in regenerative medicine for their paracrine properties mediated by extracellular vesicles (EV). Thus, an unlimited stem cell EV source retaining this regenerative potential is still not defined. Herein, we aimed at defining (1) whether MSC-derived hiPSC secrete EV (2) able to induce tissue repair in a model of ischemia (3) with a specific molecular mechanism that could account for such functionality.

**Methods**: EV were isolated from hiPSC or MSC 24 h-conditioned medium by ultracentrifugation and characterized by nanoparticle tracking analysis, scanning and transmission electron microscopy and flow cytometry. Brain ischemia was induced by oxygen and glucose deprivation in an *ex vivo* organotypic mouse model. Damaged tissues received EV for 48 h, after which cell tissue viability by PI incorporation, cell population survival by pPCR and inflammation by multiplex protein array were evaluated. EV miRNome content was defined by high-throughput PCR-array.

**Results**: hiPSC produced intact cytoplasm-containing EV (137±12 nm size; 361±27 10^6^/cm^2^). In our ischemia model, both MSC-EV and hiPSC-EV were able to significantly reduce necrosis compared to damaged untreated control. Intriguingly, astrocytes were the neural population more efficiently protected by EV. This protective/reparative effect correlated with significant tissue level reduction of apoptotic/inflammatory TNFα (four-fold) and INFγ (three-fold). To investigate the link between EV and this anti-inflammatory modulation, we defined their whole miRNome content. Interestingly, a subset of miRNA targeting TNFα and INFγ was identified.

**Summary/Conclusion**: For the first time, we showed that hiPSC-EV possess the potential to regenerate damaged tissues via a miRNA-dependent anti-inflammatory mechanism.

**Funding**: This project was funded by Fondazione Grigioni per il Morbo di Parkinson.

OF14.04

Exosomal miR-29 mediates the therapeutic effects of placenta-derived mesenchymal stromal cells in Duchenne muscular dystrophy

Peter Berenstein^1^; Ariel Bier^1^; Darya Margoulis^1^; Hodaya Goldstein^1^; Simona Cazacu^2^; Amir Dori^3^; Chaya Brodie ^1^

^1^Bar-Ilan University, Ramat-Gan, Israel; ^2^Henry Ford Health Systems, Detroit, USA; ^3^Sheba Medical Center, Ramat-Gan, Israel

**Background**: Duchenne muscular dystrophy (DMD) is a degenerative lethal, X-linked disease of skeletal and cardiac muscles caused by mutations in the dystrophin gene. Cell therapy using different cell types, including mesenchymal stem cells (MSCs), has been considered as a potential approach for the treatment of DMD. The safety and therapeutic impact of these cells have been demonstrated in preclinical and clinical studies and their functions are attributed to paracrine effects that are mediated by secreted cytokines and extracellular vesicles.

**Methods**: Here, we studied the therapeutic effects of MSCs derived from bone marrow, adipose tissue, umbilical cord and placenta on the differentiation of human myoblasts from healthy controls and Duchenne patients and on mdx mice using novel quantitative miRNA reporters, ImageStreamX and confocal microscopy for exosome delivery and *in vivo* imaging techniques.

**Results**: Treatment of myoblasts with the different MSCs or exosomes secreted by these cells demonstrated that placenta derived MSCs (PL-MSCs) and their exosomes (PL-exosomes) exerted a preferential differentiation effects on mouse and human myoblasts compared to cells/exosomes derived from bone marrow and adipose tissues. Similarly, PL-MSCs and PL-exosomes decreased the expression of fibrogenic genes in DMD patient myoblasts and increased the expression of utrophin in these cells. The PL-MSC effects were mediated by the transfer of exosomal miR-29 to the myoblasts. Intramuscular transplantation of MSCs or exosomes in mdx mice resulted in decreased creatine kinase level, decreased inflammatory cytokine expression and increased utrophin expression. In addition, the PL-MSCs and PL-exosomes significantly decreased the level of fibrosis in the diaphragm and cardiac muscles and the expression of TGF-beta. Imaging analyses using MSCs or exosomes labeled with fluorescent dyes demonstrated localization and engraftment of the cells and exosomes in the muscle tissues up to 4 weeks post-treatment.

**Summary/Conclusion**: These results demonstrate that PL-MSCs and their secreted exosomes have important clinical applications in cell therapy of DMD partly via the delivery of exosomal miR-29 and targeting of multiples pathways including tissue fibrosis, inflammation and utrophin expression

**Funding**: This work was funded by Israel Science Foundation, Adi, Science in Action and ExoSTem Biotec

OF14.05

Molecular content and regenerative potential of EVs from native and genetically modified induced pluripotent stem cells in heart repair *in vivo*

Ewa K. Zuba-Surma ^1^; Katarzyna Kmiotek-Wasylewska^1^; Sylwia Bobis-Wozowicz^1^; Marta Adamiak^1^; Anna Labedz-Maslowska^1^; Guangming Cheng^2^; Sylwia Kedracka-Krok^3^; Magdy Girgis^3^; Elzbieta Karnas^1^; Malgorzata Sekula^4^; Zbigniew Madeja^1^; Buddhadeb Dawn^3^

^1^Department of Cell Biology, Faculty of Biochemistry, Biophysics and Biotechnology, Jagiellonian University, Krakow, Poland; ^2^Division of Cardiovascular Diseases, Cardiovascular Research Institute, University of Kansas Medical Center, Kansas City, KS, USA; ^3^Malopolska Centre of Biotechnology, Jagiellonian University, Krakow, Poland; ^4^Malopolska Centre of Biotechnology, Krakow, Poland

**Background**: Extracellular vesicles (EVs) from stem cells (SCs) participate in tissue repair by transferring bioactive cargo. Although, EVs from different SCs were studied, the molecular profile and regenerative capacity of induced pluripotent SCs (iPS)- derived EVs (iPS-EVs) were not well investigated.

The aim was to examine (1) phenotype and molecular content of iPS-EVs, (2) their functional impact on mature target cells (cardiac and endothelial cells) *in vitro*, and (3) regenerative capacity in tissue injury models including murine acute myocardial infarction (AMI) *in vivo*; and (4) biological properties of EVs form iPS cells overexpressing procardio- and proangiogenic miRNAs (miR-1, miR-199a and miR-126).

**Methods**: iPS cells were cultured in serum- and feeder-free conditions. miRNAs were overexpressed by lentiviral transduction.

iPS-EVs were harvested from conditioned media by sequential centrifugation including ultracentrifugation (100,000g). iPS-EV morphology and size were examined by AFM, NTA (Nanosight) and DLS (Izon), the antigen presence- by high-sensitivity FC (Apogee M-50) and WB, the mRNAs/miRNAs content- by real-time RT-PCR, the global proteom -by mass spectrometry.

Functional assays in target cells after iPS-EV treatment *in vitro* include: proliferation, migration, differentiation, metabolic activity and cell viability analyses. Regenerative potential of iPS-EVs was examined in murine AMI model *in vivo*.

**Results**: We confirmed that iPS-EVs (1) contain iPS and exosomal markers; (2) are enriched in mRNAs, miRNAs and proteins from iPS cells regulating e.g. cell proliferation and differentiation; (3) transfer the cargo to target cells impacting on their functions *in vitro*; (4) exhibit regenerative potential by improving heart function after iPS-EV injection (at 35d). Importantly, no teratoma formation was found in iPS-EV- treated animals.

**Summary/Conclusion**: We showed that iPS-EVs: (1) carry and transfer bioactive content of iPS cells to heart cells improving their functions *in vitro*; (2) may be enriched by genetic modifications of parental iPS cells, which enforce their activity; (3) enhance heart repair *in vivo*.

We conclude that iPS-EVs may represent new safe therapeutic tool in tissue regepair, alternative to whole iPS cells.

**Funding**: This study was supported by TEAM-2012/9-6 (FNP) to EZS and UMO-2013/10/E/NZ3/00750 (NCN) grants to EZS.

OF14.06

Opioid-mediated extracellular vesicle production and NLRP3 inflammasome activation cause vascular damage

Stephen R. Thom; Veena Bhopale; Kevin Yu; Ming Yang

University of Maryland School of Medicine, Baltimore, MD, USA

**Background**: Whether opioids alter circulating extracellular vesicles (EVs) is unknown. Interleukin (IL)-1β plays a major role in opioid addiction by poorly understood effects within and outside the CNS. Because IL-1β is packaged within EVs, we hypothesized opioids stimulate EVs production.

**Methods**: In response to morphine and hydromorphone human and murine neutrophil microparticle (MPs) production *ex vivo* was assessed by flow cytometry, exosome formation by tunable resistive pulse sensing. Mice were injected IP.

**Results**: Based on protein depletion using small inhibitory RNA and specific inhibitors, human and murine neutrophils generate MPs high in IL-1β by an oxidative stress response involving mitochondria, NADPH oxidase and nitric oxide synthase-2. After 1 h incubation at 37 C with 0, 50, 100 and 200 nM morphine, suspensions of 550 murine neutrophils generated, respectively, (mean + SE, *n* = 3, *p < 0.05 ANOVA), 62+5, 296+34*, 1351+179*, and 2560+413* MPs, and responses were inhibited by 1 µM naloxone (opioid-receptor antagonist). IL-1β content in control MPs was 1.6 + 0.6 pg/million MPs, but after 100 nM morphine IL-1β was 92.8+8.1 (*p* < 0.01) pg/million MPs. Exosome production was also doubled. Whereas control mice had 625+80 MPs/µl plasma with IL-1β concentration of 38+9 pg /million MPs; after 2 h those injected with 20 mg/kg morphine had 6329+289 MPs/µl with IL-1β concentration of 678+49 pg /million MPs, (*n* = 4, *p* < 0.05). Morphine induced MPs had surface proteins indicative of production by neutrophils (Ly6G+), microglia (P2Y12 and CD45+) and endothelium (CD31+/CD41-dim). Time-course and dose-responses demonstrated diffuse capillary leak in brain and colon that was abrogated by treating mice with IV polyethylene glycol telomere B to lyse EVs.

**Summary/Conclusion**: Opioid-receptor stimulation triggers oxidative stress, leukocyte EVs production and NLRP3 inflammasome activation. Morphine-induced EVs cause vascular injuries.

**Funding**: This study was funded by Office of Naval Research [Grant N00014-16-1-2868].

Symposium Session 15 - EVs and the Nervous system Chairs: Andrew Hill; David Otaegui Location: Room 6 13:45 - 15:15

OF15.01

Study of exosomal microRNAs from microglia involved in neuroprotection in Hirudo medicinalis

Quentin Lemaire; Christophe Lefebvre; Michel Salzet; Antonella Raffo-Romero; Tanina Arab; Christelle Van Camp; Françcoise LeMarrec-Crocq; Jacopo Vizioli; Pierre-Eric Sautière

Université de Lille, INSERM, Villeneuve D’ascq, France

**Background**: Unlike vertebrates, the medicinal leech (*Hirudo medicinalis*) can be lesioned only on axons without any contact on neuronal cell bodies due to the tubular structure of its nerve cord. At this time, the microglial cells migrate to the site of lesion in close contact with damaged axons. Those cells are able to release extracellular vesicles (EVs) to dialogue with neurons. We showed that microglial EVs are massively present in lesioned connectives and in ganglion around the neuronal cell bodies following injury. Taking into account that EVs contain proteins, lipids and nucleic acids (mRNAs and microRNAs) we focused on microRNA populations mediating the microglia-neurons crosstalk for a better understanding of neuroprotection.

**Methods**: The methodology is based on (1) the collection of activated microglia from injured leech nerve cord, (2) the isolation of microglial EVs by a differential centrifugation with a density gradient, (3) the characterization of vesicular microRNAs by NGS (RNAseq) and (4) quantification of microRNAs representation in microglial EVs.

**Results**: The first results show that EVs from microglia contain many identified microRNAs. We start the quantitative study to explore their differential representation in EVs from a primary culture of microglia under early vs. late activated state. The preliminary results show that some microRNAs are more represented in EVs in an early activated state compare to a late activated state. Taking into account these results, we study target mRNAs which could be under the influence of these microRNAs using bio-informatics and we show that numerous mRNAs involved in neuroinflammation pathways (wnt or TGF-) could be regulated by this microRNAs.

**Summary/Conclusion**: The further studies (1) will use fluorescent molecular beacons specific for each miRNA to determine the percentage of positive EV subpopulations and (2) will measure the impact of miRNAs on neuronal survival (neurite outgrowth and neuronal protein signatures) by using synthetics miRNAs (mimics or inhibitors).

OF15.02

Extracellular vesicle associated microRNA-29a elicits microglial inflammation and synaptodendritic injury during chronic methamphetamine abuse

Dalia Moore^1^; Alexander Clark^1^; Benjamin Lamberty^1^; Howard Fox^1^; Gurudutt Pendyala^2^; Sowmya V. Yelamanchili ^1^

^1^Department of Pharmacology and Experimental Neuroscience, UNMC, Omaha, NE, USA; ^2^Department of Anesthesiology, UNMC, Omaha, NE, USA

**Background**: Methamphetamine (Meth) and related amphetamine compounds, which are potent psychostimulants, are among the most commonly used illicit drugs. Though numerous studies have shown that Meth abuse causes both short-term and long-term damage to the brain, the specific role of non-neuronal cells, such as glial cells, in directly affecting neuronal heath is not well understood. Recent studies on extracellular vesicles (EV) and their role in cell-cell communication have opened new avenues of research in this direction. In the present study, we isolated and characterized EV associated microRNA cargo from the brains of chronically administered rhesus macaques and self-administered rats.

**Methods**: Density gradient EV isolations from brain tissue, nanoparticle tracking analysis, transmission electron microscopy, Taqman RT-PCR, *in situ* hybridization, *in vitro* primary neuronal and microglial cultures

**Results**: Chronic Meth administration changed EV dynamics in the brain. Our investigation revealed that the genes involved in the endosomal sorting complexes required for transport are responsible are significantly increased upon Meth treatment. Small RNA sequencing revealed increased the levels of miR-29a. *In situ* hybridization in monkey brain sections reveal that miR-29a is exclusively presents in microglia and neurons but absent from astrocytes. *In vitro* culture of microglia revealed that miR-29a is released into EVs upon Meth treatment. MiR-29a packed into artificial EV-like particles elicits synaptodendritic damage to the primary hippocampal neurons. Furthermore, we also show that miR-29a starts a chronic inflammatory cycle by also activating microglia and releasing pro-inflammatory factors such as interleukin-1β, interleukin-6 and tumour necrosis factor-α in a time-dependent manner. Finally, we also show that ibudilast, an anti-inflammatory phosphodiesterase inhibitor, to reduce the release of EV and miR-29a thereby alleviating its toxic affects.

**Summary/Conclusion**: We conclude that chronic Meth abuse interferes with EV biogenesis. Increased expression of miR-29a in EV is further responsible for chronic inflammation and synaptic injury in neurons. These affects can be ameliorated by the use of an anti-inflammatory drug ibudilast.

**Funding**: This work was supported by NIH/NIDA R01DA042379

OF15.03

Apolipoprotein E4 compromises brain exosome production and secretion

Katherine Y. Peng ^1^; Rocio Perez-Gonzalez^1^; Melissa J. Alldred^1^; Jose Morales-Corraliza^1^; Stephen D. Ginsberg^1^; Mariko Saito^2^; Mitsuo Saito^3^; Paul M. Mathews^1^; Efrat Levy^4^

^1^Center for Dementia Research, Nathan S. Kline Institute for Psychiatric Research, Orangeburg, USA; ^2^Department of Neurochemistry, Nathan S. Kline Institute for Psychiatric Research, Orangeburg, USA; ^3^Department of Analytical Psychopharmacology, Nathan S. Kline Institute for Psychiatric Research, Orangeburg, USA; ^4^Departments of Psychiatry, Biochemistry & Molecular Pharmacology, and the Neuroscience Institute, NYU Langone Medical Center, Orangeburg, USA

**Background**: The apolipoprotein E (*APOE)* gene codes for the brain’s primary cholesterol carrier protein. In both humans and humanized *APOE* mice the Alzheimer’s disease-risk *APOE* ɛ4 allele (*APOE4*) alters the number and size of neuronal endosomes, a pathology common to several neurodegenerative disorders, including Alzheimer’s disease. Given that exosomes derive from the endosomal system, we investigated the impact of *APOE4* on brain-derived exosomes.

**Methods**: Extracellular vesicles (EV) were isolated from brain tissue of neuropathologically normal humans and of APOE targeted-replacement mice at 6, 12 and 18 months of age. Antibodies against TSG101 and ALIX were used to identify the exosome population within these samples. Protein, mRNA and lipid analyses were performed on both EV and whole-brain samples.

**Results**: We found lower exosome levels in the brains of neuropathologically normal human APOE4 carriers compared to individuals homozygous for the risk-neutral ɛ3 allele (APOE3). In APOE4 compared with APOE3 mice, brain exosome levels were lower in an age-dependent manner: lower levels were observed at 12 and 18 but not at 6 months of age. Protein and mRNA expressions of the exosome pathway regulators TSG101 and Rab35 were also lower in APOE4 compared with APOE3 mouse brains at 12 months of age, arguing for decreased exosome biosynthesis and secretion, respectively, from the endosomal pathway. Cholesterol and ganglioside levels were higher in brain exosomes isolated from 12-month-old APOE4 compared with APOE3 mice.

**Summary/Conclusion**: Our findings show an APOE4-driven downregulation of brain exosome biosynthesis and release that is associated with altered lipid homeostasis. Failure to maintain proper functioning of the interdependent endosomal-exosomal pathways during aging, which is essential for diverse homeostatic and catabolic cellular processes, is likely to contribute to neuronal vulnerability in neurodegenerative disorders, including Alzheimer’s disease.

**Funding**: This work was supported by the NIH (P01 AG017617 and R01 AG057517 to PMM and EL; R01 AG043375 and P01 AG014449 to SDG; T32-GM066704 and T32-AG052909 to KYP). KYP was additionally supported by the Sackler Institute of Graduate Biomedical Sciences, New York University School of Medicine.

OF15.04

Conditional deletion of Rab35 and Alix in mice to study exosomes in neuron-glia interaction *in vivo*

Kerstin Miebach ^1^; Christina Müller^2^; Anja Schneider^3^; Wiebke Möbius^4^; Anja Scheller^5^; Laura Stopper^5^; Frank Kirchhoff^5^; Remy Sadoul^6^; Eva-Maria Krämer-Albers^2^

^1^University of Mainz, IDN, Molecular Cell Biology, Mainz, Germany, Mainz, Germany; ^2^IDN, Molecular Cell Biology, Johannes Gutenberg University Mainz, Mainz, Germany; ^3^German Center for Neurodegenerative Diseases (DZNE), Bonn, Bonn, Germany; ^4^MPI for Experimental Medicine, Göttingen, Göttingen, Germany; ^5^Molecular Physiology, CIPMM, University of Saarland, Homburg, Germany; ^6^Institute of Neurosciences, Grenoble, Grenoble, France

**Background**: In the CNS, myelinating oligodendrocytes (OLs) provide trophic support and mediate long-term neuronal integrity. We showed that neuronal activity triggers the release of oligodendroglial exosomes from multi-vesicular bodies (MVB) that are subsequently internalized by neurons. Oligodendroglial exosomes promote neuronal metabolic activity and transport of cargo along axons, indicating their importance in glial support. To examine the role of exosomes in neuron-glia communication, we are studying transgenic mouse models with a potential defect in OL exosome secretion due to conditional deletion of Rab35 and Alix.

**Methods**: We are analysing transgenic mice floxed in the gene locus of Rab35- and Alix and crossed to oligodendroglial Cre-drivers mediating deletion (KO) specifically in oligodendroglial precursor cells and mature OLs. To confirm impaired exosome release by OLs we quantified isolated exosomes by western blotting (WB) using different markers and nanoparticle tracking analysis (NTA). Furthermore, we determined the ultrastructure and number of MVBs in optic nerves by electron microscopy (EM). We currently apply stress paradigms to neurons and examine the potential of KO-derived exosomes to enhance metabolic activity of neurons. To determine exosome transfer to neurons *in vivo*, we are utilizing CreERT2-mediated reporter gene recombination subjected to a distinct tamoxifen injection-protocol to visualize and quantify exosome delivery from OLs to neurons in different brain areas.

**Results**: NTA and WB of exosomes derived from wild-type versus KO-mice provide evidence that exosome secretion is affected by Rab35- and Alix-deletion in OLs. EM analyses of optical nerve cross sections demonstrate a compartment specific increase of MVBs in Rab35-KO OLs. Functional analysis elucidating exosome delivery to neurons and their ability to mediate metabolic support is ongoing and will give insight into the roles of Rab35 and Alix to produce functionally competent exosomes.

**Summary/Conclusion**: Conditional deletion of Rab35 and Alix provides a useful means to examine the precise role of oligodendroglia-derived exosomes in neuron-glia interaction and glial support *in vivo* and may also be utilized to interfere with exosome transfer in other tissues.

**Funding**: This work was funded by DFG.

OF15.05

Interrelationships between endosomal pathology and exosomal generation and release in neurodegenerative disorders

Efrat Levy ^1^; Rocio Perez-Gonzalez^2^; Katherine Y. Peng^2^; Paul M. Mathews^2^

^1^Departments of Psychiatry, Biochemistry & Molecular Pharmacology, and the Neuroscience Institute, NYU Langone Medical Center, Orangeburg, USA; ^2^Center for Dementia Research, Nathan S. Kline Institute for Psychiatric Research, Orangeburg, USA

**Background**: Dysfunction of the neuronal endosomal pathway is a characteristic of down syndrome (DS) and Alzheimer’s disease (AD) and of carriers of the AD-risk apolipoprotein E ɛ4 allele (*APOE4*). We hypothesized that the efficient release of endosomal material via exosomes into the extracellular space, as observed in the brains of DS patients and a mouse model of the disease and by DS fibroblasts, is necessary for a neuron to prevent accumulation of endosomal contents. Conversely, *APOE4-*driven downregulation of exosome release in the brains of *APOE4* human carriers and *APOE4* targeted-replacement mice appears to contribute to endosomal pathology. We investigated *in vitro* the interrelationship between the endosomal and exosomal pathways.

**Methods**: Fibroblasts from DS patients and age-matched controls were transfected with CD63 siRNA or negative control siRNA. Level of exosomal secretion was studied by western blot analysis, and number and area of endosomes by immunohistochemistry.

**Results**: Knockdown of the tetraspanin CD63, a regulator of exosome biogenesis, diminished exosome release by DS fibroblasts but not by control cells. CD63 knockdown did not affect endosomal morphology in control cells, but the number and total area occupied by endosomes was greater in DS fibroblasts in which CD63 expression was reduced.

**Summary/Conclusion**: In neurodegenerative disorders with endosomal-lysosomal dysfunction, exosome secretion serves as a disposal mechanism for potentially toxic materials that are abnormally accumulated in endosomal compartments. Conversely, APOE4-driven downregulation of brain exosome biosynthesis and release contributes to endosomal pathology. Failure to maintain proper functioning of the interdependent endosomal-exosomal pathways during aging likely contributes to neuron degeneration and our findings argue that exosome production plays a central role maintaining homeostatic function of the endosomal-lysosomal system.

**Funding**: This work was supported by the NIH (P01 AG017617 and R01 AG057517) and the Alzheimer’s Association (NIRG-14-316622).

OF15.06

Immunomodulatory treatment approach to CNS injury: role of mesenchymal stem cell derived extracellular vesicles

Amit K. Srivastava; Katherine A. Ruppert; Tin T. Nguyen; Karthik S. Prabhakara; Siqin Zhaorigetu; Naama Toledano Furman; Charles S. Cox; Matthew T. Harting; Scott Olson

Department of Pediatric Surgery, The University of Texas Health Science Center at Houston, Houston, USA

**Background**: Extracellular vesicles (EVs) secreted by mesenchymal stem cells (MSCs) have been proposed to be a key mechanistic link in the efficacy of cell therapies in response to injuries through paracrine effects. We hypothesize that EVs derived from inflammation stimulated MSCs would possess enhanced immunomodulatory effects and have a greater therapeutic effect in CNS injury.

**Methods**: We derived EVs from inflammation-stimulated and naïve MSCs (MSCEv+ and MSCEv respectively) using a cGMP-compliant tangential flow filtration system. Both EVs were characterized for size, surface marker expression, cytokine expression and RNA content. We further evaluated the immunomodulatory properties of both EVs by *in vitro* primary splenocyte inhibition/activation assay and peripheral blood mononuclear cell-EV interaction assay. We then used both EVs in a T10 contusion spinal cord injury (SCI) rat model to evaluate their therapeutic effects.

**Results**: MSCEv+ attenuated the *in vitro* release of pro-inflammatory cytokines to a greater extent as compared to MSCEv, with a distinctly different pattern of uptake by activated primary leukocyte subpopulations. The efficacy of EVs was partially attributed to COX2/PGE2 expression. We found that both EVs were therapeutically beneficial in experimental SCI in improving clinical outcome measures. However, MSCEv treatment had a greater efficacy in improving locomotor recovery and MSCEv+ treatment in improving mechanical sensitivity threshold. Flow cytometric analysis of cells from SCI epicenter revealed a decrease in pro-inflammatory M1 microglia and an increase in anti-inflammatory M2 microglia by both EVs. Spleen analysis showed increased myeloid cells, with a decrease in NK cells and leukocytes in EV treated SCI rats.

**Summary/Conclusion**: Our findings demonstrate that MSC-EVs isolated in clinically relevant cGMP-compliant manner can be used to effectively attenuate inflammation in CNS injury and perhaps in other conditions. Additionally, EVs derived from inflammation stimulated MSCs demonstrate a modified therapeutic profile with increased efficacy in some outcome measures.

**Funding**: This work was funded by TIRR Foundation award (AKS); Center for Clinical and Translational Sciences Training Award, University of Texas McGovern Medical School (M.T.H.); Ladybug (C.S.C. and M.T.H.); Glassell Family Stem Cell Research Fund (SDO);

Symposium Session 16 - Strategies for Studying Blood-related EVs Chairs: Edit Buzas; Chris Gardiner Location: Auditorium 15:45 - 16:45

OF16.01

Rapid isolation of artificial liposomes and exosomes extracted from plasma of healthy donors utilizing a novel insulator-based dielectrophoretic device

Leilei Shi; Leyla Esfandiari

University of Cincinnati, Cincinnati, USA

**Background**: Exosomes are small membrane vesicles, 30–100 nm in size which acts as vehicles for molecular cargo in cell-cell communication. They have a promising potential as biomarkers for diagnosis and new semi-synthetic drug delivery vehicles for personalized therapeutics. Currently, the conventional method to separate exosomes from biofluids is differential ultracentrifugation, which is very time-consuming and labour-intensive. The phenomenon of dielectrophoresis (DEP) that involves the movement of a particle resulting from the interaction between the induced polarization and the spatially non-uniform electric field provides a promising mean for isolation of nano-vesicles.

In this work, we demonstrated an insulator-based dielectrophoretic (iDEP) device that is capable of rapid entrapment of nano-vesicles from solutions under the low applied DC field across a nanopipette.

**Methods**: A glass nanopipette was fabricated, backfilled with PBS solution, and mounted between two PDMS chambers. Fluorescently labeled liposomes of 100 nm were re-suspended into PBS solution at 10^11^/mL. The mixture of liposomes and negatively charged nanoparticles was re-suspending in PBS at 10^11^/mL and 10^7^/mL. Lyophilized exosomes from plasma of healthy donors were reconstructed in PBS at 10^8^/mL. The prepared solution of 50 uL was injected in the chamber facing the tip. Potential of 10 V/Cm was applied across the pipette while the pipette’s conductance was measured along with the optical recording.

**Results**: The unique conductance changes across the pipette and the microscopic image of the accumulated nano-vesicles at the tip indicated the isolation of the liposomes as the negative potential bias was applied at the base. Selective entrapment of liposomes from the solution containing nanoparticles was achieved as the voltage polarity was reversed. Furthermore, unlabeled exosomes were successfully trapped after 100 s similar to the liposomes isolation.

**Summary/Conclusion**: A novel and rapid iDEP approach to trap nano-vesicles from solution was presented. The device has a spatiotemporal resolution and can further be optimized to selectively isolate extracellular vesicles from cell culture medium or biofluids and thus, facilitate liquid biopsies in future.

**Funding**: This work was funded by University of Cincinnati start-up funds to Dr L. Esfandiari.

OF16.02

Release of mitophagosomes from TRAP-activated platelets

Silvia H. De Paoli ^1^; Mehulkumar Patel^1^; Oumsalama K. Elhelu^1^; Tseday Z. Tegegn^1^; Michael B. Strader^1^; Lukas L. Diduch^2^; Abdu Alayash^1^; Jan Simak^1^

^1^CBER FDA, Silver Spring, USA; ^2^Dakota Consulting, Inc., Silver Spring, USA

**Background**: Platelet (PLT) extracellular vesicles (PEVs) exhibit several activities with pathophysiological importance and may serve as diagnostic biomarkers. PLTs contain active autophagy/mitophagy pathways, however, association of PEV release with these processes have not been elucidated.

**Methods**: The release of mitochondria (MITO) and autophagy associated PEVs from human PLTs activated by thrombin receptor-activating peptide (TRAP) was investigated by flow cytometry (FC), MS-proteomics, transmission electron microscopy (TEM), LASER-scanning confocal microscopy (LSCM) and nanoparticle tracking analysis.

**Results**: Despite marked differences in proteomic profiles of large/dense PEVs (L-PEVs, obtained by 20,000 *g* spin; mean diameter 350 nm) and small PEVs (S-PEVs, obtained by 100,000 *g* spin of 20,000 *g* sup.; mean diameter 103 nm), both L-PEVs an S-PEVs carried proteasome complex and autophagy related proteins. MITO proteins including superoxide dismutase and ATP synthase were identified in both PEV fractions, though with lower abundance in the S-PEVs. TEM of PEVs showed damaged MITO, MITO fusing with cytoplasmic vacuoles and MITO-containing vesicles. About 50% of FC-detectable PEVs (DHPE labeled) exposed the MITO marker TOM20; 16±1% of PEVs expressed the autophagosomal protein LC3 (LC3+ PEVs) and 12±3% of PEVs (75% of LC3+ PEVs) were TOM20+ LC3+ PEVs.

**Summary/Conclusion**: Our results suggest that a portion of TOM20+ PEVs are mitophagosomes - products of PLT mitophagy. The formation of PEVs decorated with LC3 is indicative of a type-2 mitophagy in which LC3 bearing vesicles attach to damaged MITO, explaining the observed population of TOM20+ LC3+ PEVs. PLT mitophagosomes, their structure, mechanism of release and potential biologic effects need further investigation.

**Funding**: These findings and conclusions have not been formally disseminated by FDA and should not be construed to represent any Agency determination or policy.

OF16.03

What are we looking at? Extracellular vesicles, lipoproteins or both?

Jens B. Simonsen

DTU Nanotech - Technical University of Denmark, Kgs. Lyngby, Denmark

**Background**: The EV research field is facing two major challenges: (1) Isolation of non-lipoprotein contaminated plasma-derived EVs and (2) Accurate fluorescence-based tracking of EVs mediated by post-inserted fluorophores.

**Methods: (**1) Here, I present the fundamental physical properties of EVs and lipoproteins (size and density) that underline the inherent problems in isolating pure EVs from lipoproteins using physical-based purification methods. (2) Size-exclusion chromatography (SEC)-isolated plasma EVs (fluorophore-labeled post SEC-isolation) were incubated for 2 h with blood plasma. Postincubation, the sample was separated by SEC and the fluorescence intensity of the different SEC-fractions was measured.

**Results: (**1) A literature-based survey of the density and size distributions of EVs and lipoproteins highlights the significant overlap in size and/or density between different sub populations of lipoproteins and EVs. (2) The preliminary SEC-data show that a significant amount of the fluorophore-label was associated to SEC-fractions not related to EVs, but probably to lipoproteins. These results question the notion that the fluorescence readout from cells and tissues in *in vitro* and *in vivo* studies can be solely correlated to the uptake of fluorophore-labeled EVs.

**Summary/Conclusion**: The similar physical properties of EVs and lipoproteins in terms of density, size and capability to host labile amphiphilic fluorophores challenges our statements about the biological fate and functions of EVs because it questions what we are actually looking at.

**Funding**: This work was funded by Novo Nordisk Foundation.

OF16.04

Acetylcholinesterase activity co-isolates minimally with small EVs and does not correlate with particle count

Dillon C. Muth ^1^; Zhaohao Liao^1^; Tine H. Schøyen^1^; Tessa Seale^2^; Lorena Martin-Jaular^3^; Matias Ostrowski^4^; Clotilde Thery^5^; Kenneth Witwer^1^

^1^The Johns Hopkins University School of Medicine, Baltimore, MD, USA; ^2^Johns Hopkins University, Dept of Molecular and Comparative Pathobiology, Baltimore, USA; ^3^Institut Curie, Inserm U932- Centre d’immunothérapies Des cancer, Paris, France; ^4^INBIRS Institute, School of Medicine, University of Buenos Aires, Buenos Aires, Argentina, Buenos Aires, Argentina; ^5^Institut Curie / PSL Research University / INSERM U932, Paris, France

**Background**: Acetylcholinesterase (AChE) activity has been proposed and used as a measure of EV abundance. AChE activity is easily, quickly and cheaply assayed, making it a potentially attractive option for EV quantitation. To evaluate this use of AChE activity, we examined data from different EV isolation methods using multiple cell lines grown in cell culture conditions varying by amounts of serum and serum EVs.

**Methods**: Cell lines were grown in media differing by serum status: EV-replete serum, commercial EV-depleted serum, or serum-free formulations. Cell culture conditioned medium (CCM) was harvested from various leukocyte cell lines, including T-lymphocytic lines H9 and PM1 and the promonocytic line U937. Following a slow spin to remove cells, EVs were isolated from CCM by differential ultracentrifugation (2000, 10,000 and 100,000 ×*g*) with or without subsequent iodixanol velocity density gradients. Pellets and fractions were assayed for AChE activity by standard colorimetric test; the presence of EV markers (CD63, CD81 and syntenin), and a negative marker (GM130, Golgi) by western blot; and particle count by single particle tracking (ParticleMetrix, NanoSight).

**Results**: AchE activity was highest in replete serum medium. During differential centrifugation, most AChE activity was depleted in the 2000 ×*g* and 10,000k ×*g* steps, with little remaining activity in the 100,000 ×*g* pellets. When 100,000 ×*g* pellets were further separated by iodixanol gradient, early AChE activity-enriched fractions overlapped only minimally with tetraspanin-positive EV fractions. AChE activity did not correlate significantly (*p* < 0.05) with measured particle count in any examined condition.

**Summary/Conclusion**: These findings indicate that AChE activity may be mostly associated with debris and/or large particles and is particularly abundant in medium containing undepleted serum. At least for small EVs, high AChE activity may betray contamination, not EV abundance. Additional experiments may be merited to validate these results for primary cells or in biological fluids, but, overall, AChE activity appears to be a poor indicator of EV abundance, echoing a cautionary note sounded in the MISEV2014 guidelines and other publications.

**Funding**: This research was supported in part by the US National Institutes of Health through DA040385 and AG057430 (to KWW).

## 

Symposium Session 17 – Alterations in EV Stability and Function Chairs: Carmen Fernandez; Ana Claudia Torrecilhas Location: Room 5 15:45–16:45

OF17.01

Overexpression of miR-504 in glioma stem cells inhibits the oncogenic potential and the crosstalk of these cells with microglia via exosomal delivery

Danie Rand^1^; Simona Cazacu^2^; Xin Hong^3^; Cunli Xiang^3^; Ruicong She^3^; Indrani Datta^3^; Laila Poisson^3^; Chaya Brodie ^1^

^1^Bar-Ilan University, Ramat-Gan, Israel; ^2^Henry Ford Health Systems, Detroit, USA; ^3^Henry Ford Hospital, Detroit, USA

**Background**: Glioblastoma (GBM) is a highly aggressive tumour that exhibits resistance to therapy and poor prognosis. A small subpopulation of glioma stem cells (GSCs) has been implicated in radio-resistance and tumour recurrence. Mesenchymal transformation of GBM and GSCs is associated with aggressive phenotypes, radiation resistance and positive regulatory interaction with microglia.

**Methods**: Here, we analysed miRNAs associated with the stemness and mesenchymal transformation of GSCs using miRNA microarray analysis of these cells compared with human neural stem cells (NSCs) and mesenchymal stromal cells (MSCs). Self-renewal, stemness microglia activation and exosomal delivery were studied. Data were analysed using ANOVA or a Student’s t-test with correction for data sets with unequal variances.

**Results**: We identified gene clusters associated with glioma cell invasiveness, axonal guidance and TGF-beta signaling. miR-504 was significantly downregulated in GSCs; its expression was decreased in GBM compared with normal brain specimens and was further lower in the mesenchymal subtype. The effects of miR-504 on the stemness, mesenchymal transformation of GSCs and their interaction with microglial cells were studied. Overexpression of miR-504 inhibited the self-renewal, migration and the expression of mesenchymal markers in GSCs. The inhibitory effect of miR-504 was partly mediated by upregulating the tumour suppressor miR-145. In addition, miR-504 targeted Grb10 and EGFR2, which act as oncogenes in GSCs and GBM. Using novel reporters and imaging methods we demonstrated that overexpression of miR-504 in GSCs resulted in its delivery by GSC-secreted exosomes to microglia and in the abrogation of the GSC-induced polarization of microglia to M2 phenotype. Finally, miR-504 overexpression inhibited xenograft growth and prolonged the survival of mice harbouring GSC-derived xenografts. miR-504 was detected in high levels in circulating serum exosomes of xenografted mice.

**Summary/Conclusion**: We identified the miR-504/miR145/CTGF and miR-504/Grb10/Egr1 pathways as important regulators of the mesenchymal transformation of GBM. Overexpression of miR-504 exerts anti-tumour effects in GSCs as well as bystander effects on the polarization of microglia, and possibly also on peripheral immune responses, via exosomal delivery.

OF17.02

Pathogen-derived extracellular vesicles mediate “Division of Labour” virulence in the fatal human fungus Cryptococcus gattii

Ewa Bielska ^1^; Marta Arch Sisquella^2^; Maha Aldeieg^3^; Charlotte Birch^1^; Eloise O’Donoghue^1^; Robin C. May^1^

^1^Institute of Microbiology and Infection, School of Biosciences, University of Birmingham, UK, Birmingham, United Kingdom; ^2^Fundación Instituto de Investigacion Germans Trias i Pujol, Barcelona, Spain, Barcelona, Spain; ^3^School of Biological Sciences, University of Reading, UK, Reading, United Kingdom

**Background**: *Cryptococcus gattii* is a fungal pathogen that can cause fatal infections in both immunocompromised and immunocompetent humans and other animals. The Pacific Northwest outbreak of cryptococcosis, which started in 1999, was caused by a hypervirulent *C. gattii* lineage and led to over 500 life-threatening infections. Recent data has shown that the hypervirulence of this lineage results from a unique “Division of Labour” mechanism within the fungal population which drives tremendously fast growth of the pathogen within phagocytic cells. In this process, non-replicating “guardian” cells protect the pathogens from the hostile environment of the macrophages, but the mechanism by which this occurs remains enigmatic.

**Methods**: To study “Division of Labour” we used *in vitro* co-infection assays with fluorescently labeled cryptococcal strains and transwell systems to test the hypothesis that the presence of secreted effectors may enhance fungal proliferation. Extracellular vesicles (EVs) from the outbreak C. gattii strain R265 and non-pathogenic ICB180 were isolated using ultracentrifugation, followed by characterization using electron microscopy and NanoSight NTA and visualized by fluorescence microscope using antibodies specific for cryptococcal capsule localized in the EVs.

**Results**: We found that the mechanism of cooperation between individual cryptococci relies on the exchange of EVs between the fungal strains and can be triggered over large cellular distances. The mechanism is specific for the outbreak strain and not for non-pathogenic counterparts.

EVs isolated from the outbreak strain of C. gattii are internalized promptly by white blood cells via endocytosis and taken up to phagosomes where they induce rapid proliferation of non-pathogenic cells *in vitro*. The effect is mediated by exosomal proteins and RNA, and not by exosomal DNA or capsular materials.

**Summary/Conclusion**: To our knowledge, we demonstrate for the first time that fungal EVs are involved in virulence mechanisms that can be conferred over large distances within fungal populations.

**Funding**: This work was funded by European Research Council under the European Union’s Seventh Framework Programme (FP/2007-2013): ERC Grant Agreement No. 614562; Biotechnology and Biological Sciences Research Council: BB/R008485/1

OF17.03

The role of extracellular vesicles in mediating radiation-induced bystander effects in the haematopoietic system

Katalin Lumniczky ^1^; Tünde Szatmári^1^; Dávid Kis^1^; Eszter Persa^1^; Rita Hargitai^1^; Enikő Kis^1^; Géza Sáfrány^2^

^1^National Public Health Institute, Division of Radiobiology and Radiohygiene, Department of Radiation Medicine, Budapest, Hungary; ^2^National Public Health Center National Research Directorate for Radiobiology and Radiohygiene, Anna st 5, Hungary

**Background**: Radiation-induced bystander effects are the manifestation of radiation effects in cells not directly hit by radiation. The mechanisms governing radiation-induced bystander effects are still not entirely clarified. Due to their complex RNA, microRNA and protein cargo extracellular vesicles (EVs) are potential mediators of radiation-induced bystander effects.

An *in vivo* study was designed to investigate the role of EVs in radiation-induced bystander effects in the haematopoietic system.

**Methods**: C57Bl/6 mice were irradiated with different doses of ionizing radiation, EVs were isolated from the bone marrow and injected into the tail vein of unirradiated mice. The effect of EV transfer was studied by comparing molecular and phenotypic changes of bone marrow cells, splenocytes and plasma of EV-recipient, bystander mice to the directly irradiated animals.

**Results**: Activation of the DNA damage response pathway in the bone marrow and spleen of the bystander animals was comparable to the directly irradiated animals. Phenotypical changes in both the bone marrow and spleen of bystander animals were present, however they were restricted to certain cellular subpopulations. Inflammation- and stress-related soluble factors investigated in the plasma of directly irradiated and bystander animals showed a substantial overlap and mainly chemokines and chemokine ligands were affected.

A panel of differentially expressed miRNAs were identified in the EVs isolated from the bone marrow of irradiated mice with predicted involvement in pathways related to DNA damage repair, hematopoietic and immune system regulation, suggesting their participation in mediating radiation-induced bystander effects.

**Summary/Conclusion**: In conclusion, we proved that EVs mediated certain radiation effects in the haematopoietic system of bystander mice and identified potential miRNAs carried by EVs which might be responsible for these effects.

**Funding**: This work was funded by DoReMi FP7 project (grant agreement number: 249689), the Euratom research and training programme 2014–2018 (CONCERT, grant agreement number: 662287) and National Research, Development and Innovation Office, Hungary (grant agreement number: VKSZ_14-1-2015-0021)

OF17.04

The RNA-binding protein hnRNPA2B1 inhibits the export of miR-503 into exosomes

Jennifer Perez Boza ^1^; Amandine Boeckx^1^; Michelle Lion^2^; Ingrid Struman^3^

^1^Laboratory of Molecular Angiogenesis, GIGA-R, Liege University, Liege, Belgium; ^2^Laboratory of Protein Signaling and Interactions, GIGA-R (Molecular Biology of Diseases), University of Liège, Liege, Belgium; ^3^Laboratory of Molecular Angiogenesis, GIGA-R (Cancer), University of Liège, Liege, Belgium

**Background**: The exosomal export of the anti-tumoural miR-503 is positively regulated by the chemotherapeutic agent Epirubicin (Epi). The aim of this study is to determine the mechanism underlying this process.

**Methods**: To determine the partners of miR-503, serial crosslinkings (CLs) were performed in HUVECs prior pulling down a synthetic miR-503-biotin. The proteins associated to this microRNA were then identified by mass spectrometry and validated by western blotting (WB). Then, the effect of Epi on these putative partners was studied at gene and protein levels and the affinity of these proteins with miR-503 was determined using immunoprecipitation techniques. The role of these proteins in the export of miR-503 was assessed by a series of silencing experiments.

**Results**: Nine different proteins were identified by mass spectrometry analysis and only five putative partners were validated by WB. While the treatment with Epi induced the expression of FN1, both the levels of hnRNPA2B1 and TSP1 were significantly reduced. Moreover, the treatment with the chemotherapeutic drug induced a significant increase in the export of ANXA2 into exosomes. Immunoprecipitation studies showed that hnRNPA2B1 has an important affinity for miR-503 and that the interaction of these partners with miR-503 is substantially reduced after treatment. Finally we found that the knock down of hnRNPA2B1 alone was able to reproduce the increase of exosomal miR-503 induced by the treatment with Epirubicin.

**Summary/Conclusion**: Our data suggests that Epi mediates the export of miR-503 into exosomes via hnRNPA2B1 downregulation and subsequent complex destabilization. This study shows that hnRNPA2B1 has an active role in maintaining miR-503 inside the cell and thus inhibits its export into exosomes. Of note, for the first time, this work provides evidence that a RNA binding protein can play a negative role in the export of microRNAs into exosomes.

**Funding**: This study was supported by the University of Liège (ULg), the Fonds National de la Recherche Scientifique (FNRS), Télévie and the fonds Léon Frédéricq. The authors declare that they have no competing interests.

Symposium Session 18 – EV-inspired Therapeutics in Veterinary Medicine Chairs: Hanne Winther-Larsen; Marca Wauben Location: Room 6 15:45–16:45

OF18.01

Targeted-pig trial on safety and immunogenicity of serum-derived exosomes obtained from Porcine Respiratory and Reproductive virus infections

Sergio R. Montaner Tarbes ^1^; Elena Novell^2^; Vicens Tarancón^2^; Francesc E. Borràs^3^; Maria Montoya^4^; Lorenzo Fraile^5^; Hernando A. del Portillo^6^

^1^Universitat de Lleida and Innovex Therapeutics SL., Barcelona, Spain; ^2^Grup de Sanejament Porci de Lleida, Lleida, Spain; ^3^REMAR-IVECAT Group, “Germans Trias i Pujol” Health Science Research Institute, Can Ruti Campus, Badalona, Spain, Badalona, Spain; ^4^The Pirbright Institute, Madrid, Spain; ^5^Universitat de Lleida - Department of Animal Science, Lleida, Spain; ^6^ISGlobal, Hospital Clínic - Universitat de Barcelona. Institute for Health Sciences Trias I Pujol (IGTP), Badalona, Spain. Catalan Institution for Research and Advanced Studies (ICREA), Barcelona, Spain

**Background**: The porcine reproductive and respiratory syndrome virus (PRRSV) is one of the most important diseases of veterinary interest. Available vaccines have serious limitations such as little protective immunity, possible reversion to virulence, inability to induce long lasting and heterologous protection. As previously reported by us, exosomes from PRRSV convalescent swine sera contain immunogenic viral proteins. The aim of this study was to perform a targeted-pig trial to test the safety and immunogenicity of such exosomes.

**Methods**: PRRSV convalescent sera were obtained from pigs that overcome PRRSV acute infection. Exosomes were obtained by a combination of ultracentrifugation and size exclusion chromatography and characterized by BCA, Flow cytometry, nanosight, Cryo-TEM and proteomic analyses. Animals were vaccinated with exosomes and/or viral peptides identified by proteomics in combination with Montanide. Immune responses were measured by a commercial ELISA (IDEXX X3 PRRSV), by an indirect in-house ELISA and by IFN-γ ELISPOT.

**Results**: No clinical symptoms or adverse effects were observed in animals infected with up-to 2 mg of exosomes, unequivocally demonstrating that this vaccine formulation is free of virus and safe. ELISA analysis demonstrated that immunizations elicited specific humoral IgG immune responses, albeit variably. Yet, sera from these same vaccinated animals was diagnosed free of virus using a commercial test; thus, indicating that this vaccine approach is able to differentiate vaccinated from infected animals. Last, priming the animals with exosomes from convalescence animals and boosting them with synthetic peptides identified by MS associated with them, elicited distinctive and high IFN-γ immune response when stimulated with viral peptides (around 400 SFCx106 PBMCS).

**Summary/Conclusion**: Altogether, our data support further development of plasma-derived exosomes from convalescence animals as a novel antigen discovery and vaccine strategy against PRRSV.

**Funding**: SMT have an Industrial PhD fellow by Government of Catalonia (AGAUR) as part of a collaborative agreement between INNOVEX THERAPEUTICS SL and the University of Lleida (Id No 2014 DI 044).

OF18.02

ARMMs as a versatile platform for intracellular delivery of macromolecules

Qiyu Wang; Quan Lu

Harvard University, Boston, MA, USA

**Background**: Majority of disease-modifying therapeutic targets are restricted to the intracellular space and are therefore not druggable using existing biologic modalities. The ability to efficiently deliver macromolecules inside target cells or tissues would greatly expand the current landscape of therapeutic targets for future generations of biologic drugs, but remains challenging.

**Methods**: Here we report the use of extracellular vesicles, known as ARMMs (arrestin domain containing protein 1 [ARRDC1]-mediated microvesicles), for packaging and intracellular delivery of a myriad of macromolecules, including the tumour suppressor p53 protein, RNAs, and the genome-editing CRISPR-Cas9/guide RNA Fcomplex.

**Results**: We demonstrate selective recruitment of these macromolecules into ARMMs. When delivered intracellularly via ARMMs, these macromolecules are biologically active in recipient cells. P53 delivered via ARMMs induced DNA damage-dependent apoptosis in multiple tissues in mice.

**Summary/Conclusion**: Together, our results provide proof-of-principle demonstration that ARMMs represent a highly versatile platform for packaging and intracellular delivery of therapeutic macromolecules.

OF18.03

Chitosan coated extracellular vesicles as an adjuvant for immunization against salmonid rickettsial septicemia in an adult zebrafish model

Julia Tandberg ^1^; Leidy Lagos^2^; Erik Ropstad^3^; Gro Smistad^1^; Marianne Hiorth^1^; Hanne Cecilie Winther-Larsen^1^

^1^University of Oslo, Oslo, Norway; ^2^Norwegian University of life science, Moss, Norway; ^3^Norwegian University of Life Sciences, Oslo, Norway

**Background**: Extracellular bacterial vesicles (EVs) are 50–250 nm spherical structures secreted from the surface of many bacteria. Proteomic and biochemical characterization has revealed that the vesicles contain a variety of bacterial components, including proteins, lipopolysaccharides, DNA and RNA. This makes MVs interesting as potential vaccine candidates, as they represent several aspects of the bacteria, but in a non-replicative form. EV-based vaccines have, furthermore, been successfully used for epidemic control in against serogroup B meningococcal disease, but there are still little known regarding the use of EV-based vaccines in other animals. The present study focused on evaluating extracellular vesicles coated with chitosan as a potential vaccine candidate against the intracellular pathogen *Piscirickettsia salmonis* using an adult zebrafish infection model.

**Methods**: For the dose-response experiment 25 fish per group were injected with 10, 20 or 40 µg of chitosan coated EVs (cEVs) or 20 µL phosphate buffer (control group) by i.p. injections, using a 27 g needle. For the immunization experiment 65 fish per group were injected with of either 20 µg cEVs, 20 µL of 0.15% free chitosan or 20 µL phosphate buffer (control group) by i.p. injections. The fish were then challenge by i.p. injection after an immunization period of 28 days with a challenge dose of 10^8^ CFU P. salmonis. Organ sampling was performed at the end of the dose-response experiment, and after 1, 14 and 28 days’ post-immunization (dpi) and 1, 3, 7 and 28 days’ post-challenge (dpc) for the immunization experiment. Fish for histology was sampled at 28 days’ post-immunization and 3 and 7 days’ post-challenge in the immunization experiment.

**Results**: The cMVs provided a significant protection, while a small but non-significant reduction in mortalities were registered for fish injected with only chitosan. Both free chitosan and cMVs were shown to induce an increased immune gene expression of cd4-1, cd8a, mhc1zja, mpeg1.1, tnfa, il1b, il10 and il6, but to a higher degree in the cMV group.

**Summary/Conclusion**: Taken together the results indicate a potential use of chitosan coated EVs as a vaccine against intracellular fish pathogens.

**Funding**: The work was financially supported by the University of Oslo and The Research Council of Norway; Biotek2021 Program Grant no# 233849

OF18.04

Level of extracellular vesicles, carrying the fibrinolytic activator tPA, is reduced in coronary venous blood during stimulation of cardiac sympathetic nerves in pigs

Trude Aspelin ^1^; Morten Eriksen^2^; Lilly Alice Steffensen^1^; Anne Marie Siebke. Trøseid^3^; Tonje Bjørnetrø^4^; Kari Bente Foss Haug^1^; Torstein Lyberg^1^; Reidun Øvstebø^1^

^1^The Blood Cell Research Group, Department of Medical Biochemistry, Oslo University Hospital, Ullevål, Norway, Oslo, Norway; ^2^Institute for Experimental Medical Research, Oslo University Hospital and University of Oslo, Norway, Oslo, Norway; ^3^The Blood Cell Research Group, Department of Medical Biochemistry, Oslo University Hospital, Norway, Oslo, Norway; ^4^Department of Oncology, Akershus University Hospital, Norway, Oslo, Norway

**Background**: Extracellular vesicles (EVs) carrying membrane-anchored proteins and cytoplasmic constituents of a variety of maternal cells, play important roles in intercellular communication and in various biological processes. Exercise, mental stress and myocardial ischemia are associated with increased sympathetic activity. Catecholamines, e.g. norepinephrine (NE), activate adrenergic receptors on endothelial cells, leukocytes, platelets i.e. leading to initiation of both coagulation and fibrinolysis. The main fibrinolytic activator, tissue plasminogen activator (tPA), has been demonstrated on microparticles. Accordingly, we aimed to investigate the release of EVs into coronary venous blood during sympathetic nerve stimulation (SS), and the EVs characteristics.

**Methods**: In an *in vivo* pig model (n = 3), the sympathetic nerves to the heart were electrically stimulated for 3 min. Blood samples were collected simultaneously from a coronary vein and a femoral artery at baseline, during stimulation (3 min), and 30 min after stimulation. EVs were isolated from citrate plasma using size exclusion chromatography, quantified using nanoparticle tracking analysis and confirmed by electron microscopy. EVs captured with anti-CD63-coated magnetic beads were analysed using western blot (CD81, TSG101, tPA and calnexin). NE in plasma was measured and coronary blood flow was monitored to facilitate estimation of cardiac EV and NE release.

**Results**: At baseline, increased mean concentrations of EVs in venous vs. arterial blood, indicated coronary net release of EVs. During SS, the mean arterial EV concentration increased 12% whereas venous EV concentration decreased 29% resulting in a “negative coronary release”, implying EV removal from circulation. Simultaneously, a massive coronary release of NE was observed. After 30 min of recovery, EV and NE levels had returned to nearly baseline values. Interestingly, tPA+ EVs were detected among the CD63+ EVs.

**Summary/Conclusion**: In the present study, we found decrease in coronary venous EV concentration during SS, indicating a local EV uptake or trapping of EVs with tPA at the coronary vessel wall. This may suggest a new principle to secure local fibrinolysis. The mechanisms are uncertain; however, simultaneously released NE may be involved.

**Funding**: This work was funded by Oslo University Hospital

Industry SessionsLocation: Auditorium 16:45–17:15

Meet the Expert Session: *in vivo* Imaging on EVsLocation: Auditorium 18:30–20:00

Meet the Expert Session: EVs on Immunology and VaccinesLocation: Room 5 18:30–20:00

Meet the Expert Session: Biobanks for EVsLocation: Room 6 18:30–20:00

Poster Session PF01: Analysis of EVs in Liquid Biopsy (Storage, Preparative Studies, Spike-ins, etc) Chairs: Esperanza Gonzalez; Jaesung ParkLocation: Exhibit Hall 17:15–18:30

PF01.01 = OWP3.05

Comparison of generic fluorescent dyes for detection of extracellular vesicles by flow cytometry

Leonie de Rond ^1^; Edwin van der Pol^2^; Chi M. Hau^3^; Zoltan Varga^4^; Auguste Sturk^5^; Ton G. van Leeuwen^2^; Rienk Nieuwland^5^; Frank A.W Coumans^6^

^1^Academic Medical Center, University of Amsterdam, Amsterdam, The Netherlands; ^2^Biomedical Engineering & Physics, Academic Medical Center, University of Amsterdam, Amsterdam, The Netherlands; ^3^Laboratory Experimental Clinical Chemistry, Academic Medical Center, University of Amsterdam, Amsterdam, The Netherlands; ^4^Biological Nanochemistry Research Group, Institute of Materials and Environmental Chemistry, Research Centre for Natural Sciences, Hungarian Academy of Sciences, Budapest, Hungary; ^5^Laboratory of Experimental Clinical Chemistry, and Vesicle Observation Center, Academic Medical Center, University of Amsterdam, Amsterdam, The Netherlands; ^6^Department of Biomedical Engineering and Physics, and Vesicle Observation Center, Academic Medical Centre of the University of Amsterdam, Amsterdam, The Netherlands

**Background**: Because extracellular vesicles (EVs) in plasma are potential biomarkers of disease, a generic fluorescent dye specifically staining EVs is desirable. Here we evaluated five commonly used generic dyes for flow cytometry.

**Methods**: EVs from MCF7-conditioned culture medium and human plasma were stained with calcein AM, calcein violet, CFSE, di-8-ANEPPS or lactadherin. The concentration of EVs detected by generic dyes was measured by flow cytometry (A60-Micro, Apogee). EVs were identified by immunostaining EpCAM for MCF7-EVs, and CD61 for platelet EVs. Scatter triggering was applied as a reference, and the influence of non-EV components was evaluated.

**Results**: Di-8-ANEPPS, lactadherin and side scatter detected 100% of EpCAM+ MCF7-EVs. In plasma, di-8-ANEPPS inefficiently stained EVs due to protein binding, which improved by protein removal. Lactadherin and side scatter detected 33% and 61% of CD61+ EVs, respectively. Because all generic dyes stained proteins, the overall sensitivity to detect platelet EVs in plasma was 33% at best. Calcein AM, calcein violet and CFSE were either inefficient at detection of EVs in both samples, or suffered from swarm detection and/or insufficient event rates.

**Summary/Conclusion**: None of the generic dyes detected all and only EVs in plasma. Side scatter triggering detected the highest concentration of plasma EVs on our flow cytometer, followed by lactadherin. The choice between scatter or lactadherin primarily depends on the sensitivity of the flow cytometer used.

**Funding**: This work was funded by The Netherlands Organisation for Scientific Research - Domain Applied and Engineering Sciences (NWO-TTW), research programs VENI 13681 (FC) and Perspectief CANCER-ID 14195 (LR).

PF01.02

Biomarker development and validation for nanoscale flow cytometry of liquid Biopsies

Desmond Pink ^1^; Robert J. Paproski^1^; Renjith Pillai^2^; Deborah Sosnowski^1^; Catalina Vasquez^2^; APCARI Consortium^1^; John D. Lewis^1^

^1^University of Alberta, Edmonton, Canada; ^2^Nanostics Inc, Edmonton, Canada

**Background**: Detection of biomarkers in liquid biopsy samples is a rapidly expanding field, yet standardized protocols have still not been set. Levels of extracellular vesicle (EV) biomarkers in liquid biopsy samples often constitute a very small fraction of the total EVs (10’s – 100s of available antibody binding sites for biomarker on each EV).

**Methods**: In order to establish parameters for maximal sensitivity and quantitative stability of biomarker signal, we have utilised the optical reporter palmitoylated-EGFP to label membrane EVs in cancer cells as a surrogate for fluorescent labeled biomarkers. Plasma and serum was obtained from healthy volunteers. Conditioned media from healthy LNCaP cells (PALMGFP) was used as a positive signal spike in plasma, serum and urine from healthy volunteers. To mimic the variability in patient EV concentration, PALMGFP was spiked into increasing concentrations of EVs from different liquid biopsies.

**Results**: Optimal sample concentration, based on events per second analysis was determined along with minimal sample analysis time. Sample stability was assessed at the collection, processing and during the total period of analysis. The dynamic range of positive signal spike was determined in plasma and serum and confirmed to be linear down to ~1 in 450,000 background events. Carryover of background or positive biomarker events was confirmed to be less than 5% over 96 consecutive samples.

**Summary/Conclusion**: We have confirmed that using nanoscale flow cytometry, we can detect rare positive signal events that match the expected biomarker levels on EVs in liquid biopsies. Using the Apogee A50 platform EV analysis in complex fluids is fast, yet sensitive, reproducible and can be used to assess disease biomarkers both in the lab and in clinic.

**Funding**: This work was funded by Alberta Cancer Foundation Motorcycle Ride for Dad, Prostate Cancer Canada

PF01.03

An accessible method of flow cytometer scatter standardization for EV analysis

Joshua A. Welsh ^1^; Peter Horak^2^; Wilkinson James^2^; Jennifer Jones^3^; Verity Ford^4^; David Smith^4^; Judith Holloway^4^; Nicola Englyst^4^

^1^Molecular Immunogenetics and Vaccine Research Section, Vaccine Branch, CCR, NCI, NIH, Bethesda, MD, USA; ^2^Faculty of Physical Sciences and Engineering, University of Southampton, Southampton, UK; ^3^National Cancer Institute, Bethesda, MD, USA; ^4^University of Southampton, Southampton, UK

**Background**: Extracellular vesicle flow cytometry (EV-FC) results remain an area lacking standardization. Despite methods of scatter standardization being proposed, implementation is complex and requires proprietary or specialized information. Here we demonstrate an automated method of standardizing conventional flow cytometer scatter data by acquiring nanobeads of known diameter and refractive index, combined with freely available software we have developed. This method allows for accessible standardization of the scatter parameters for EV analysis and provides the ability to convert arbitrary axes to units of diameter or refractive index with the correct controls.

**Methods**: Polystyrene & Silica NIST beads (ThermoFisher Scientific, Paisley, UK) ranging in diameter from 200 nm to 1600 nm were acquired using flow cytometry. Data was obtained on a Fortessa X-20, LSR Fortessa, Canto I, Attune NxT. SSC-H median of each bead population were compared to a precalculated database of predicted scatter for each of the beads from collection half-angles from 0.1 to 90 degrees in 0.1 degree increments. Analysed bead diameters were derived using the above modelling technique and were compared with manufacturer’s bead specifications to determine the model accuracy to predict flow cytometer collection optics. This method was further applied to a jet-in-air sorter, the MoFlo Astrios-EQ, to evaluate its performance in systems with variable alignment and optical geometries.

**Results**: Standardised flow cytometer scatter measurements predicting bead diameters and comparing them to bead specifications showed a median variation (25th percentile, 75th percentile) of 2.59% (0.55%, 5.28%).

**Summary/Conclusion**: This work demonstrates that flow cytometer scatter measurements can be obtained using a user-friendly methodology without the requirement of specialised flow cytometer components. This method also further allows extrapolation to determine particle diameter or refractive index offering potentially new methods of EV and submicron biomaterial analysis.

**Funding**: This work was funded by Faculty of Medicine Doctoral Training Award scheme, University of Southampton for a PhD studentship

PF01.04

Urinary extracellular vesicles (uEVs) have unique characteristics as demonstrated by imaging and spectral cytometry

Luca Musante ^1^; Sabrina La Salvia^2^; Uta Erdbrügger^3^; Joanne Lannigan^3^

^1^University of Virginia Health System, Department of Medicine, Division of Nephrology, Charlottesville, USA; ^2^Genomic and post-Genomic Center, C. Mondino National Institute of Neurology Foundation, IRCCS, Pavia, Italy; ^3^Department of Medicine, Division of Nephrology, University of Virginia, Charlottesville, VA, USA

**Background**: Urinary extracellular vesicles (uEVs) provide a source of valuable biomarkers for kidney and urogenital diseases. Imaging flow cytometry (iFC) allows detection of particles that are < 200 nm in size and has a high level of sensitivity for small particle fluorescence. In addition, spectral flow cytometry (sFC), which is based on whole spectrum analysis, can be used to further characterize the findings of the iFC analysis.

**Methods**: Urine, blood and saliva (internal auto fluorescent control) EVs were isolated by differential centrifugation. uEVS were stained with different annexin V conjugates: FITC, PE, PerCPCy5.5, Pacific Blue™, Brilliant Violet 421, Brilliant Violet 510, APC, Alexa Fluor® 647 respectively. Gating strategy was based on the low scatter of the unstained uEVs and the negative control was all fluorescent probes alone in buffer.

**Results**: Acquisition of uEVs alone in iFC showed auto-fluorescence emission in channel 5 (λex 660 nm; λem 740 nm) for camera 1 and channel 11 (λex 660 nm; λem 740 nm) for camera 2. Auto-fluorescence emission in channel 11 was caused by excitation from the violet laser (λex 405 nm) and red laser (λex 642 nm). Auto-fluorescence in Channel 5 was caused by excitation of both blue (λex 488 nm) and yellow laser (λex 561 nm). Spectral analysis of unlabelled uEVS, plasma EVs (pEVs), and saliva EVs (sEVs) showed that this auto-fluorescence was unique and specific for uEVs. Spectrum plots showed a distinct signature across the 488, 405, and 640nm lasers, with a dominant emission peak in the red regions of each laser for the uEVS, but not for the others. These results confirmed what was seen using iFC. Finally, conjugated Annexin V, used at the same concentration, showed different affinity for phosphatidylserine depending on the conjugated fluorescent dye. AV-APC, AV-PE and AV-FITC showed greater binding to uEVs, than the other fluorochromes used.

**Summary/Conclusion**: While iFC represents a major advancement in the identification of uEVs, our results suggest that unexpected additional complication of the analysis originated from the auto-fluorescence with a peculiar spectral emission that needs to be taken into account when multicolour antibodies panels are planned. Likewise, choice of AV fluorochrome conjugate should be carefully considered.

PF01.05

Molecular drivers and markers of pancreatic cancer initiation and progression

Claire Gourzones ^1^; Patrick Jacquemin^2^; Ingrid Struman^3^

^1^Laboratory of Molecular Angiogenesis, GIGA-R, University of Liège, Belgium, Liege, Belgium; ^2^de Duve Institute, Université catholique de Louvain, Belgium, Brussels, Belgium; ^3^Laboratory of Molecular Angiogenesis, GIGA-R (Cancer), University of Liège, Liege, Belgium

**Background**: Pancreatic ductal adenocarcinoma (PDAC) is the fourth cause of cancer-related death worldwide with a 5-year survival below 6% due to lack of early diagnostic markers and the poor efficiency of the treatment, especially for advanced stages of the disease.

Exosomes carry proteins and nucleic acids that can be transferred into recipient cells and could be good biomarkers either for diagnostic or follow-up purposes.

The aim of our project is to identify new exosomal molecular markers and drivers of PDAC initiation, progression and metastasis. The first step is the identification of exosomal proteins and RNAs in the plasma of mouse models and patients associated with the early development of the disease. The second step aims to understand the role of these molecular markers on the development of PDACs.

**Methods**: To achieve this project, we have access to mouse models in which conditional expression of KRASG12D in pancreatic cells in association with a cerulein-induced pancreatitis induces the early preneoplastic stage of the disease (PanIN), which then leads to the development of PDAC and metastasis upon further activation of other proto-oncogenes such as p53 or the loss of tumour suppressors.

We have isolated exosomes in the plasma of mice taken at different stages of the disease and in the plasma of 16 human PDAC patients and healthy controls. Exosomal RNA has been purified and we have performed exosomal smallRNA profiling by RNA sequencing.

**Results**: Preliminary results show that several miRNAs such as miR-335, 1290, 1246 and 210 are enriched in the plasma of PDAC patients. Some of these miRNAs (miR-1290, miR-1246 and miR-210) are known oncomirs and miR-355 and miR-210 are also abundant in exosomes isolated from the PDAC-derived cell line PANC1.

**Summary/Conclusion**: Next, we will study if the export of these identified miRNAs plays a role in the disease by treating tumour cells and cells from the tumour microenvironment with exosomes produced by PDAC cell lines in which the expression of these miRNAs will be modulated.

**Funding**: This work was supported by the FNRS (Fonds National de la Recherche Scientifique, Belgium) and the University of Liège. CG holds a Télévie Fellowship (Belgium).

PF01.06

Characterization of EXOsomes of the Spanish POpulation of REference (EXOSPORE)

María García-Flores^1^; Verónica Fernández-Pascual^2^; María Luisa Hortas^3^; Raquel Romero^4^; Rocío Aguilar-Quesada^5^; Maria Isabel García Sánchez^6^; Rosa Pinto Labajo^7^; María Pérez Caro^7^; Francisco Javier García-Palomo^7^; Ainara Egia Bizkarralegorra^8^; Oihana Belar^8^; Jorge Gallego de la Fuente^9^; Montserrat Tora^10^; María Antonia Fortuño^11^; Aurora Astudillo^12^; María Ruiz^13^; Manuel M. Morente^14^; Maria Jesus Artiga^14^; Susana Teijeira^15^; Jose Antonio Lopez-Guerrero ^1^

^1^Fundación Instituto Valenciano de Oncología, Valencia, Spain; ^2^Institut d’Investigacions Biomèdiques August Pi i Sunyer, Barcelona, Spain; ^3^Servicio de Salud Público de Andalucia, Malaga, Spain; ^4^Servicio de Salud Público de Andalucía, Cadiz, Spain; ^5^Servicio de Salud Público de Andalucia, Granada, Spain; ^6^Servicio de Salud Público de Andalucía, Sevilla, Spain; ^7^Banco Nacional de ADN, Salamanca, Spain; ^8^Biobanco Vasco, Bilbao, Spain; ^9^Biobanco VIH HGM del Hospital Gregorio Marañón, Madrid, Spain; ^10^Mar Biobanc-IMIM- Hospital del Mar, Barcelona, Spain; ^11^Clínica Universitaria de Navarra (CIMA), Pamplona, Spain; ^12^Hospital Central de Asturias, Oviedo, Spain; ^13^Biobanco IRBLleida, Lleida, Spain; ^14^Centro Nacional de Investigaciones Oncológicas, Madrid, Spain; ^15^Instituto de Investigación Sanitaria Galicia Sur (IISGS), Complejo Hospitalario Universitario de Vigo, Vigo, Spain

**Background**: Extracellular vesicles (EVs) are of great interest given their involvement in multiple biological functions and in therapeutic use: biomarkers for diagnosis or monitoring of certain diseases, vectors for the transport and administration of certain drugs or as therapeutic agents per se.

Most researchers working with EVs need controls/references for their studies. Nowadays, there is not any consensus regarding the type and number of controls to be studied.

**Methods**: In order to know better the needs of EVs researchers in the field of controls, an online survey about the convenience for researchers of having controls for their studies was distributed, the responses being highly favourable.

**Results**: A total of 40 answers were received from researchers focused on pathologies such as cancer, cardiovascular and infectious diseases. Most of these studies were focused on fields such as the identification of biomarkers, diagnosis or prognosis of diseases.

An average of about 60 cases/study (range: 10–300) are included, the main source of obtaining VEs being plasma, cell culture media and urine. The method preferred for obtaining exosomes was ultracentrifugation, although there were already a variety of procedures for its isolation (ultrafiltration, commercial isolation kits).

The number of controls used in each study, was also very variable: same number than cases, or 20% of the analysed cases, with a range between 10 and 100 controls per study. All EVs researchers would be interested in having a collection of controls because they considered it very useful and they all would be potential users, especially if this collection was aimed at adults and both sexes. Regarding the type of characterization in which the researchers would be interested, most of them opted for proteomics and transcriptomics and they considered the association of information regarding sample donors very useful.

**Summary/Conclusion**: In light of these needs, we decided to propose the EXOSPORE, a coordinated action between Biobanks and other institutions to constitute a representative collection of EVs isolated from plasma and urine that can be used as controls in the field of biomedical research.

**Funding**: This study is partially supported by grants PT17/0015/0051 from the ISCIII and PROMETEO/2016/103 from the Conselleria d’Educacó i Investigació de la Generalitat Valenciana.

PF01.07

Extracellular vesicle isolation and characterization in the follow up of HCV-infected patients treated with directly-acting antiviral agents (DAAs)

Meritxell Llorens. Revull ^1^; Marta Bes^2^; Celia Perales^1^; Josep Gregori^3^; Carmen de la Torre^2^; Elena Vargas^1^; Damir Garcia^1^; Maria Eugenia Soria^1^; Qian Chen^1^; Sofia Píriz^1^; Irati Fernandez^1^; Francisco Rodriguez-Frias^4^; Silvia Sauleda^2^; Josep Quer^1^; Juan Ignacio Esteban^1^

^1^Viral Hepatitis, Liver Unit, Vall d’Hebron Institute of Research (VHIR)-Hospital Universitari Vall d’Hebron (HUVH), Barcelona, Spain; ^2^Laboratori de Seguretat Transfusional, Banc de Sang i Teixits (BST), Barcelona, Spain; ^3^Roche Diagnostics S.L., Sant Cugat del Vallès., Barcelona, Spain; ^4^Liver Pathology Unit, Departments of Biochemistry and Microbiology, HUVH, Barcelona, Spain

**Background**: Extracellular vesicles (EVs) are present in human biofluids and as such, they can potentially serve as a source of novel biomarkers in disease diagnosis such as cancer or infectious diseases. Exosomes are a type of EVs, with a lipid bilayer, that carries a broad repertoire of cellular components. Exosome content generally reflects the nature and status of the cell of origin. The aim of this preliminary study is to explore the most suitable method for exosome isolation to characterize and study the exosome content as a diagnostic tool for liver diseases.

**Methods**: Exosomes were isolated by an affinity-based method (WAKO kit) which uses T-cell immunoglobulin domain and mucin domain-containing protein 4 (Tim4). Isolated EVs were quantified and visualized by the nanosight NS300 and transmission electron microscopy (TEM), respectively. CD63, CD9, HSP70 and CD81 proteins were quantified by western blot. The total RNA was extracted by miRNeasy Mini Kit (Qiagen), and the microRNA (miRNA) integrity was measured by the RNA 6000 Pico mRNA (Agilent Technologies) using Bioanalyzer 2100.

**Results**: The technique was able to reproducibly obtain 10^9^ EVs/ml of serum as quantified by nanosight tracking analysis in both fresh and frozen samples.

The preparation showed close-to-spherical shape particles of 50–150 nm, according to TEM analysis. At present, only the CD63 protein has been identified by western blot, therefore additional tests are needed for EV characterization. Around 100 pg/µl of total RNA was obtained from the samples.

**Summary/Conclusion**: Exosome isolation using magnetic beads is optimal in order to obtain a highly pure EV preparation. Improvements in extraction methodology are ongoing.

PF01.08

Physical activity as pre-analytical parameter for liquid biopsies

Daniel Enderle ^1^; Alexandra Spiel^1^; Johan Skog^2^; Mikkel Noerholm^1^

^1^Exosome Diagnostics GmbH, Martinsried, Germany; ^2^Exosome Diagnostics Inc., Waltham, USA

**Background**: Liquid biopsies often aim to compare the amount of nucleic acids of tumour origin with nucleic acids that are shed from healthy cells. While it is understood that the amount of tumour material in circulation strongly depends on tumour stage, the conditions influencing the amount of wildtype nucleic acids are less well described. The amount of RNA contained in exosomes and other extracellular vesicles (exoRNA), as well as the amount of cell-free DNA (cfDNA), may be influenced by pre-analytical parameters - potentially changing the interpretation of a liquid biopsy result. Here, we analyse the release of exoRNA and cfDNA by physical activity.

**Methods**: We sampled the blood of four healthy individuals by venipuncture before and after 30 min of continuous physical exercise and used a combined co-extraction of exoRNA and cfDNA to analyse their relative abundance in plasma. Size distribution of the total nucleic acids was accessed using a BioAnalyzer 6000 Pico Kit. The content of exoRNA was quantified using RT-qPCR for specific mRNAs (GAPDH and RPL0) and the content of cfDNA was quantified using primers for the loci of often used oncogenes (KRAS, BRAF and PIK3CA).

**Results**: All four individuals showed increased amount of total nucleic acids after physical exercise, mainly corresponding to the size of mono- and dinucleosomal DNA. Comparing the pre- and post-exercise datapoints of each patient, the increase in cfDNA was measured consistently between 10 and 30-fold, while the increase in exosomal mRNAs was only two-fold on average.

**Summary/Conclusion**: While both exoRNA and cfDNA increased already after 30 min of exercise, the increase in cfDNA consistently exceeded the increase of exosomal RNA by an order of magnitude. This effect of physical activity has to be taken into account when interpreting datasets that use the absolute or relative amount of nucleic acids in liquid biopsies.

PF01.09

Optimization of flow cytometer settings with fluorescently-tagged retrovirus for the analysis of extracellular vesicles by nanoscale flow cytometry

Dylan Burger ^1^; Vera A Tang^2^; Fengxia Xiao^3^; Anna K Fritzsche^2^; Marc-André Langlois^2^

^1^Kidney Research Centre, Ottawa Hospital Research Institute, University of Ottawa, Ottawa, Canada; ^2^University of Ottawa, Ottawa, Canada; ^3^Ottawa Hospital Research Institute, Ottawa, Canada

**Background**: Nanoscale flow cytometry (NFC) is becoming a method of choice for the phenotypic analysis of extracellular vesicles (EVs). Small EVs range in size from approximately 50–250 nm in diameter, which places them at the limit of detection for commercial flow cytometers. Optimization of flow cytometer settings for the analysis of EVs can therefore be challenging. 

**Methods**: Reference materials such as fluorescently labeled polystyrene or silica beads are often used in the optimization of flow cytometer settings, however, synthetic beads have a higher refractive index are often much more fluorescent than biological samples of equivalent size. Here we demonstrate that an eGFP-tagged retrovirus is more effective than synthetic beads as a reference material for NFC instrument set-up. An eGFP-tagged murine leukemia virus (124 nm) was compared to Apogee Bead Mix - a mix of silica (not fluorescent) and polystyrene (fluorescent) beads (110–585 nm) to optimize and calibrate detector gain and threshold for side-scatter (SSC) and fluorescence. EVs isolated from urine (80 nm median size by NTA) and cell-culture media (HUVEC, 67 nm median by NTA) were labeled with DiO and analysed by NFC. EV counts and MFI were evaluated for instrument set-up performed using either synthetic beads or fluorescently-tagged virus.

**Results**: We report that instrument set-up performed with virus resulted in eight times more DiO+ events acquired in urine EVs, and close to 10-fold more events in HUVEC EVs when compared to instrument set-up with beads.

**Summary/Conclusion**: These findings suggest that fluorescently-tagged virus should be considered for use as a reference material for optimal analysis of EVs by NFC.

**Funding**: This study was supported by grants from the Canadian Institutes of Health Research and the Canada Foundation for Innovation (to DB and MAL)

PF01.10

Urinary exosomal and cell-free DNA detects somatic mutation and copy number alteration in urothelial carcinoma of bladder

Kwang Hyun Kim

Department of Urology, Ewha Womans University College of Medicine, Seoul, Republic of Korea

**Background**: Urothelial bladder canrcinoma (UBC) is characterized by a large number of genetic alteration. Urinary DNA is promising resources for liquid biopsy in urological malignancies. In this study, we performed genomic profiling of UBC and matched urinary cell free DNA (cfDNA) and exosomal DNA (exoDNA).

**Methods**: We included nine patients who underwent surgery for UBC. Fresh frozen tumour sample and normal blood sample was used for genomic profiling of UBC. We also performed genomic profiling of matched urinary DNA to investigate whether genomic alteration in tumour samples are echoed in urinary DNA. Urinary exoDNA was extracted from urinary exosome which was isolated by ExoQuick and urinary cfDNA was extracted by commercial kit using magnetic bead. We performed nine gene target sequencing for somatic mutation analysis and low depth whole genome sequencing (ldWGS) for copy number analysis.

**Results**: In this analysis, we found 17 somatic mutations in six patients, and 17 included six nonsynonymous SNVs, three stopgain SNVs, two frameshift deletion and six synonymous SNVs. Of 17 somatic mutations, 12 were identified in cfDNA and exoDNA with the mean allele frequency of 54.5% and 65.6%, respectively. Mean depth of cfDNA and exoDNA was 1721X and 1627X, respectively. In copy number analysis, mean 20.4% of whole genome region was covered by >1X. Copy number plots of cfDNA and exoDNA showed similar pattern with those of tumour samples. When we compare the log2 ratio of 100,000 bin size in whole genome regions, Pearson correlation coefficients of tumour vs. cfDNA (0.481) and tumour vs. exoDNA (0.455) were higher than that of tumour vs. normal (0.086).

**Summary/Conclusion**: In conclusion, both urinary cfDNA and exoDNA were representative of the entire human genome and allowed genomic profiling of UBC. Specifically, copy number analysis using ldWGS has potential to be used as tools developing biomarker with low cost and whole genome coverage.

PF01.11

Water intake depletes concentration of extracellular vesicles in peripheral blood

Ljubisa Paden; Tina Vogrinec; Roman Stukelj; Manca Pajnic; Mitja Drab; Veronika Kralj-Iglic

Laboratory of Clinical Biophysics, Faculty of Health Sciences, University of Ljubljana, Ljubljana, Slovenia

**Background**: Extracellular vesicles (EVs) have been identified as promising in diagnosis and treatment of different diseases and in assessment of the state of the organism. The advantage of EV-based methods is that EVs can be isolated from body fluids, which are obtained by minimally invasive procedures. However, methods for EV harvesting and assessment have not yet reached sufficient repeatability and accuracy to be introduced in clinical practice. Also it is not yet clear how a person should be prepared for sampling of the body fluid. Here we considered the effect of water intake before the sampling on the concentration of EVs in isolates from peripheral blood.

**Methods**: Twenty-six healthy human adult volunteers (average age 39.8 years) were included in the study. After recording the drinking habits, the intake of water was increased for 30% before the first blood sampling and then decreased for 30% before the second sampling. The volunteers did not eat at least 12 h before blood sampling. EVs were isolated from 3 mL samples of blood by repetitive centrifugation (up to 17570 g) and washing by phosphate buffered and citrated saline. EVs were counted by flow cytometry. Standard parameters of blood and urine were assessed in a routine clinical laboratory.

**Results**: The samples taken after increased water intake yielded considerably (for 26%) lower concentration of EVs in isolates comparing to samples taken after decreased water intake (*p* = 0.05).

Concentration of EVs in isolates correlated with most urine parameters (decrease of the freezing temperature, potassium ions, chloride ions urea, creatinine and urate) and only two blood parameters (sodium ions and chloride ions) (*p* < 0.05).

**Summary/Conclusion**: Water intake is important in preparing for blood sampling for assessment of EVs. Mild and short-term over-hydration and dehydration did not cause notable change in most of the blood parameters; it however notably affected urine parameters, in correlation with concentration of EVs.

**Funding**: This work was funded by Slovenian Research Agency Grants (P3-0388, J5-7098 and J2-8166)

PF01.12

Flow cytometry analysis of platelet microparticles in cord blood: the effect of delay in sample preparation

Andrea Hujacova^1^; Tereza Brozova^2^; Tibor Mosko^1^; Zbynek Stranak^2^; Karel Holada ^1^

^1^Institute of Immunology and Microbiology, First Faculty of Medicine, Charles University Prague, Prague, Czech Republic; ^2^Department of Neonatology/Pediatrics, Institute for the Care of Mother and Child, Prague, Czech Republic

**Background**: Plasma levels of circulating platelet microparticles (PMPs) are an emerging marker of platelet activation, thrombosis, inflammation and endothelial dysfunction with a possible prognostic value. Cord blood represents an attractive alternative to venous blood especially in extremely preterm birth infants. Flow cytometry is capable to provide data both about the number of detected PMPs and the levels of their markers expression. However, preparation of cord blood samples in clinical settings may be complicated by numerous issues. Our study was aimed on identification of possible confounding factors of PMPs analysis in clinical settings. The study was approved by ethical committee of ICMC and signed informed consents were obtained.

**Methods**: Two approaches to sample preparation were compared. In the first, aliquots of whole citrate anticoagulated cord blood were centrifuged and analysed immediately or stored for 6 h at 24 C before the processing. In the second, the platelet free plasma (PFP) was prepared immediately after the blood collection and PFP aliquots were analysed either directly or after 6 h at 4 C. Separate study compared the effect of PFP flash freezing on the results of PMPs analysis. PMPs were double labeled by mAbs CD36FITC/CD41PE or CD41FITC/CD62PE, washed and their counts and fluorescence analysed by flow cytometer.

**Results**: Delay in the whole blood processing led to ~three-fold increase of PMPs counts (*p* < 0.01, *n* = 6) which was accompanied by ~25% increase of their CD41 signal (*p* < 0.05, *n* = 6). In comparison the storage of PFP in the fridge did not have significant effect on PMPs counts or their CD36, CD41 and CD62 signal (*n* = 6). Freezing of PFP before the labeling led to ~40% decrease of PMPs counts (*p* < 0.05, *n* = 6) and surprisingly also of their CD36, CD41 and CD62 signal by 24%, 29% and 21%, respectively (*p* < 0.01).

**Summary/Conclusion**: Both the delay in preparation of PFP from whole cord blood or freezing of PFP before the analysis had significant effect on detected PMPs counts and the level of their mAb signals. In contrast, the storage of PFP at 4 C gave results comparable to fresh, immediately analysed PFP.

**Funding**: The study was funded by grants of the Czech health research council NV15-32961A and NV17-31403A.

PF01.13

Hydrogel microparticle based multiplex qRT-PCR profiling microRNAs in urinary extracellular vesicle for renal transplantation rejection

Won Jin Kim ^1^; Seungwon Jung^1^; Jung-Woo Seo^2^; Kwang Pyo Kim^1^; Sang Ho Lee^2^; Sang Kyung Kim^3^

^1^Kyung-Hee University, Yongin, Republic of Korea; ^2^Kyung-Hee University, Seoul, Republic of Korea; ^3^Korea Institute of Science and Technology, Seoul, Republic of Korea

**Background**: MicroRNAs (miRNAs) are small non-coding RNAs which are emerged as biomarkers of several diseases. Since miRNAs are known to be enriched in extracellular vesicles (EVs), miRNAs in EV have been highlighted as non-invasive promising diagnostic and prognostic biomarkers. Recently, miRNAs in urinary EVs have been used for observing acute immune rejection after renal transplantation, and several miRNAs have been screened as a biomarker. To diagnose acute immune rejection via miRNAs, rapid and accurate analysis tool is needed.

**Methods**: To satisfy these needs, we developed high-throughput and uncomplicated method using hydrogel microparticles which have target specific primers immobilized in their porous structure. At the same time, one-step qRT-PCR can be conducted for rapid and uncomplicated running process.

**Results**: With this particle, the same dynamic range was shown as the conventional method with 97% PCR efficiency using artificially synthetic miRNAs. Also, we confirmed that miRNA-negative total RNA (E.coli total RNA) did not affect the target detection and PCR efficiency. In terms of specificity, precursor form and mature form of the miRNA were distinguished, and the single-nucleotide variated miRNAs were discriminated. Using these particles, six miRNA biomarkers were analysed successfully in a single one-step qRT-PCR with urine samples from immune rejection patients.

**Summary/Conclusion**: Briefly, we developed high-throughput, straightforward and rapid method using hydrogel microparticles for profiling miRNA in urinary EVs. This technique is expected to be applicable to non-invasive disease diagnosis and prognosis through profiling miRNAs of liquid biopsies other than urine EVs, and diagnosis efficiency can be improved through quick analysis time.

**Funding**: This work was supported by the National Research Foundation of Korea (NRF) grant funded by the Korea government (MSIP) (NRF-2015R1A2A1A10055994).

PF01.14

Co-isolation and analysis of extracellular vesicle (EV)-associated DNA and cell free DNA (cfDNA) improves the diagnostic and prognostic value of circulating BRAF V600E in metastatic melanoma patients

Davide Zocco ^1^; Simona Bernardi^2^; Mauro Novelli^3^; Natasa Zarovni^1^; Francesco Carpi^4^; Ottavia Malavenda^3^; Pietro Quaglino^3^; Domenico Russo^2^; Antonio Chiesi^1^; Maria Teresa Fierro^3^

^1^Exosomics Siena, Siena, Italy; ^2^CREA Lab- Blood Diseases and Bone Marrow Transplantation, University of Brescia, ASST Spedali Civili Brescia, Brescia, Italy; ^3^Dermatologic Clinic, Department of Medical Sciences, Turin University, Torino, Italy; ^4^Dept. Life Sciences, Imperial College London, UK, London, United Kingdom

**Background**: Detection of *BRAF* V600E on cell free tumour DNA (cfDNA) is emerging as a promising means to improve patient’s stratification or enable BRAFi therapy monitoring in a minimally invasive manner. Here, we evaluate extracellular vesicle-(EV)-associated-DNA (EV-DNA) as an alternative and valuable source of circulating *BRAF* V600E. To do so, we identified a clinically compatible protocol for the isolation of EV-DNA and assessed *BRAF* gene status on plasma samples from metastatic melanoma (MM) patients at the beginning and during BRAFi therapy.

**Methods**: EV isolation by peptide-mediated affinity-(PA) was selected after protocol benchmarking and compared to the reference protocol for cfDNA isolation (CF). DNA was extracted from plasma samples of MM patients (*n* = 50) at baseline and during BRAFi treatment with both protocols and BRAF gene status was assessed by digital PCR (dPCR).

**Results**: PA isolation captured more BRAF V600E-positive EVs than ultracentrifugation in a spike-in model. Most of the isolated DNA was found to be associated to the outer side of the EV membrane as EV-DNA or co-purified as cfDNA. PA isolation improved the detection of BRAF V600E gene copies from plasma samples of mutation-positive MM patients in comparison to CF, increasing the diagnostic and prognostic value of this circulating mutation (AUC PA = 0.72; CF = 0.66; Log-rank test, OS: *p* valuePA = 0.0046; *p* valueCF = 0.04; PFS: *p* valuePA = 0.091; *p* valueCF = 0.25).

**Summary/Conclusion**: Co-isolation of EV-DNA and cfDNA improves the clinical value of circulating BRAF V600E in comparison to the current reference protocol for liquid biopsy.

**Funding**: This work was funded by Exosomics Siena SpA.

PF01.15

Systematic study of exRNA isolation reveals presence of distinct exRNA carriers

Srimeenakshi Srinivasan ^1^; Pike See Cheah^2^; Kirsty Danielson^2^; Peter De Hoff^1^; Justyna Filant^3^; Clara Laurent^4^; Lucie Laurent^4^; Parham Nejad^5^; Anu Paul^5^; Ravi Shah^6^; Bridget Simonson^2^; Cuong To^1^; Irene Yan^7^; Xuan Zhang^2^; Leonora Balaj^8^; Xandra O. Breakefield^9^; Saumya Das^2^; Roopali Gandhi^5^; Jodi Lapidus^10^; Tushar Patel^7^; Anil Sood^3^; Louise C. Laurent^1^

^1^University of California, San Diego, CA, USA; ^2^Massachusetts General Hospital, Boston, MA, USA; ^3^The University of Texas MD Anderson Cancer Center, Houston, TX, USA; ^4^University of California San Diego, San Diego, CA, USA; ^5^Brigham and Women’s Hospital, Boston, MA, USA; ^6^Partners HealthCare, Boston, MA, USA; ^7^Mayo Clinic, Jacksonvilee, Fl, USA; ^8^Mass General Hospital/Harvard Medical School, Boston, MA, USA; ^9^Department of Neurology and Radiology and Program in Neuroscience, Massachusetts General Hospital and Harvard Medical School, Boston, MA, USA; ^10^Oregon Health & Science University, Portland, OR, USA

**Background**: Extracellular RNAs (exRNAs) have lately spawned a lot of interest as potential biomarkers, mediators of intercellular communication and therapeutic agents. ExRNA study is challenging due to the impact of biological variables on exRNA levels and technical concerns, such as low abundance and biases in methods used for isolation and analysis. Here, we systematically investigated a variety of isolation methods on standardized biofluids across multiple sites.

**Methods**: Total and carrier enriched exRNA was isolated from five biofluids. Precipitation, membrane filtration, ultracentrifugation or affinity purification was used for exRNA carrier enrichment. exRNA was then extracted from total biofluid and the exRNA carrier enriched fractions. Small RNA libraries were prepared from selected samples using NEBNext Small RNA Library Preparation kit and sequenced on a HiSeq4000, and the data was analysed, focusing on miRNA and coding RNA reads.

**Results**: Our data suggests distinct sources of variation in each biofluid. The cell type of origin was the strongest source of variability in cell culture supernatants, followed by RNA isolation method. Inn plasma/serum, RNA isolation method contributed the most to variability, suggesting enrichment of certain subsets of miRNAs and mRNAs by each method. In bile, the rather small number of miRNAs detected were reproducibly measured in samples isolated using the miRCury Biofluids kit, while fragments of many coding RNAs were efficiently isolated using all the tested methods.

**Summary/Conclusion**: Our results demonstrate that reproducibility within and agreement between methods vary significantly across exRNA isolation methods and biofluids. Notably, none of the tested RNA isolation methods provided complete isolation of all exRNAs. Each method has a specific bias for specific exRNA carriers. These findings suggest that the selection of the method used for exRNA isolation is a critical consideration for studies in this field.

PF01.16

The somewhat forgotten role of isotype control antibodies in selecting and validating phenotype markers and antibody panels for EV characterisation

Jaco Botha; Mathilde Sanden; Morten Mørk; Søren R. Kristensen; Aase Handberg

Department of Clinical Biochemistry, Aalborg University Hospital, Aalborg, Denmark

**Background**: Since the dawning of the field of research into extracellular vesicles (EVs), flow cytometry has been a widely used method for characterisation of EVs due to its ability to detect multiple parameters on single particles in a high-throughput manner. Selection of phenotype markers and set-up of antibody panels for characterisation is often an arduous task laden with pitfalls that can potentially lead to inaccurate conclusions being drawn if proper controls are not employed. Here, we report on how potential pitfalls in selecting and validating phenotypic markers for the characterisation of EVs can be avoided by correct interpretation of adequately matched isotype control antibodies.

**Methods**: Flow cytometry was performed on an Apogee A60 Micro-PLUS. Platelet-poor plasma from healthy individuals was stained with lactadherin-FITC and one or a combination of commonly used antibodies against platelet (CD41), leukocyte (CD45), endothelium (CD144, CD146, CD62E, VEGFR2, CD31) and activated tissue factor (TF, CD142) or matching isotype control antibodies for each panel. All antibodies were centrifuged at 17000g for 10 min prior to staining to remove potential aggregates. EVs were defined as lactadherin-binding events ≤ 1000 nm.

**Results**: In platelet-poor plasma, it was possible to see distinct differences in number of positive events and mean positive channel fluorescence between CD41, CD45 and CD31 and their respective isotype controls. However, this was not the case for antibodies against CD146, CD144, CD62E, VEGFR2 or TF. Similar results were observed when comparing the commonly used CD31+/CD41- endothelial phenotype with its isotype control as well as for several antibody clones against CD146, CD144, CD62E and TF and with different fluorophore combinations.

**Summary/Conclusion**: Some doubt has to be cast on the routine usage of the aforementioned markers for EV characterization, as several commonly used antibodies against endothelium and TF did not provide results that could not be attributed to non-specific binding of antibodies. Although these markers are present on cells, the biological basis for their presence on EVs might be dubious due to cellular asymmetry or brief windows of expression. Careful interpretation of isotype controls in setting up and validating antibody panels might provide a safeguard against this pitfall.

PF01.17

Effect of hemodialysis on circulating submicron particle levels

Dylan Burger ^1^; Fengxia Xiao^2^; Hussein Abujrad^2^; Yasamin Al-Rewashdy^2^; Marcel Ruzicka^2^; Alexander Sorisky^2^; Teik Chye Ooi^2^

^1^Kidney Research Centre, Ottawa Hospital Research Institute, University of Ottawa, Ottawa, Canada; ^2^Ottawa Hospital Research Institute, Ottawa, Canada

**Background**: Hemodialysis is a renal replacement therapy that is used to filter the blood in the setting of kidney failure. During hemodialysis, blood is passed through an extracorporeal circuit and over a semipermeable membrane filter which removes fluids and waste products but retains cellular material and most proteins. The purpose of this study was to examine the impact of hemodialysis on circulating levels of membrane microparticles and other submicron particles.

**Methods**: Fifty six patients (age 61±2 years, 30M/26F) were studied. Non-fasting plasma samples from patients with stage 5 chronic kidney disease were collected immediately before and within 30 min after hemodialysis at the mid-week hemodialysis session. An FX 800 hemodiafilter (Fresenius Medical Care) with a mean pore size of ~3.3 nm was used. Total submicron particles were assessed by nanoparticle tracking analysis. In addition, levels of endothelial (CD144+), platelet (CD41+), leukocyte (CD45+), and total (Annexin V +) microparticles were assessed by flow cytometry.

**Results**: Total submicron particle number, as assessed by nanoparticle tracking analysis, was significantly lower post-dialysis (5.3 × 10^10^ pre-dialysis vs. 1.4 × 10^10^ particles/ml post-dialysis, *p* < 0.001). When examined as a function of size, reductions were seen after hemodialysis in particles <40 nm (pre: 7.1 × 10^9^ vs. post: 1.2 × 10^9^, *p* < 0.001), particles 40–100 nm (2.6 × 10^10^ vs. 5.0 × 10^9^, *p* < 0.001), and 100–1000 nm (1.0 × 10^10^ vs. 6.6 × 10^9^, *p* < 0.001). By flow cytometry, significant reductions after hemodialysis were seen in circulating annexin V+ve microparticles (3 × 10^7^ vs. 1.6 × 10^7^, *p* < 0.01), platelet microparticles (1.8 × 10^7^ vs. 1.0 × 10^7^, *p* < 0.001), leukocyte microparticles (2.4 × 10^6^ vs. 1.8 × 10^6^, *p* < 0.05), and endothelial microparticles (8.4 × 10^5^ vs. 5.9 × 10^5^, *p* < 0.01).

**Summary/Conclusion**: Hemodialysis is associated with reductions in circulating submicron particles including membrane microparticles. Accordingly, there may be significant interdialytic variation in circulating submicron particle levels. Investigators interested in measuring extracellular vesicles should consider the timing of biosampling in patients undergoing hemodialysis.

**Funding**: This study was supported by grants (to DB) from the Canadian Institutes of Health Research, Diabetes Canada, and the Canada Foundation for Innovation.

PF01.18

Specific capture and analysis of tumour exosomes in plasma is a promising liquid biopsy approach for comprehensive detection of actionable genomic alterations in prostate cancer patients

Laura Bianciardi ^1^; Chiara Foroni^2^; Simona Bernardi^3^; Davide Zocco^1^; Francesca Valcamonico^4^; Alfredo Berruti^4^; Antonio Chiesi^1^; Natasa Zarovni^1^

^1^Exosomics Siena, Siena, Italy; ^2^CREA Lab-Oncology Unit, University of Brescia, ASST Spedali Civili Brescia, Brescia, Italy; ^3^CREA Lab- Blood Diseases and Bone Marrow Transplantation, University of Brescia, ASST Spedali Civili Brescia, Brescia, Italy; ^4^Oncology Unit, University of Brescia, ASST Spedali Civili Brescia, Brescia, Italy

**Background**: Castration-resistant prostate cancer (CRPC) is characterized by disease progression following surgical or pharmaceutical androgen deprivation. CRPC has been associated to poor prognosis and linked to multiple genomic instability events affecting androgen receptor (AR) gene. Tumour heterogeneity and lack of effective strategies for risk stratification and monitoring that timely predict disease evolution and drug resistance contribute to poor therapy efficacy. Our aim was to assess the feasibility of different liquid biopsy protocols for (co)detection of actionable AR alterations in plasma of CRPC patients and their prognostic and predictive relevance.

**Methods**: Proprietary antibody and peptide based affinity isolation platform (AI) was evaluated for enrichment of tumour EVs from CRPC patients’ plasma collected upon written patient’s consent. DNA and RNA were extracted using kit-based flow and analysed by digital PCR (Thermo Fischer) to determine the presence of AR-V7, AR T878A and AR gene copy number variation. AR status for each patient was compared to results obtained with benchmark isolation kits (Qiagen exoRNeasy MAXI and Qiagen QIAamp circulating nucleic acid).

**Results**: Tumour EVs focused approach enabled sensitive and, in some samples concomitant, detection of DNA (T878A, AR WT, CNV) and RNA targets (AR-V7, AR-FL) in line with their published frequencies. Our AI was superior to exoRNeasy in detection of AR-V7 (100% vs. 57% samples) and AR-V7 enrichment ratio (2.3 vs. 0.31 respectively). Comprehensive AR genomic status correlated well with patient classification to therapy responders or not, otherwise not discriminated by standard clinical parameters.

**Summary/Conclusion**: We have identified the protocol for reliable detection of actionable AR alterations related to treatment response/resistance in CRPC. Its further validation and implementation in clinical setting holds promise for accurate treatment eligibility tests and optimization and improved long-term outcomes.

**Funding**: This work was funded by Exosomics Siena SpA.

PF01.19

Designing nanoscale flow cytometry assays: Fixing, Blocking and Permeabilizing Samples

Desmond Pink ^1^; Renjith Pillai^2^; Robert J. Paproski^1^; Deborah Sosnowski^1^; Catalina Vasquez^2^; APCARI Consortium^1^; John D. Lewis^1^

^1^University of Alberta, Edmonton, Canada; ^2^Nanostics Inc, Edmonton, Canada

**Background**: Identification of biomarkers in tissue culture media followed by detection in liquid biopsy samples is a rapidly expanding field, yet standardized protocols have still not been agreed upon. Designing experiments to characterize populations of EVs in liquid biopsy must incorporate significant measures to minimize the signal to noise ratio. The use of blocking agents, fixatives and washing media to minimize non-specific binding is understood to be important but has not necessarily been optimized. In these experiments we examined these procedures on nanoscale flow cytometry experiments.

**Methods**: Nanoscale flow cytometry was performed on the Apogee A50. PC3 palmitoylated GFP and cytosolic GFP expressing cell lines were used to generate conditioned media. Samples were treated with detergent, with or without fixing to determine the potential for permeabilization without dissolution. Blocking agents and washing in either PBS or PBS-Tween20 were used to determine if non-specific binding could be decreased.

**Results**: Blocking with ≤5% BSA or FBS provided ~10% of the optimal sample concentration but neither agent improved resolution of FL signal. Membrane palm-GFP samples only showed a loss of GFP signal when incubated with Tween20 at 37 C, but cytosolic GFP samples showed a minimal loss of 20% even at 4 C. Fixing samples did not alter cytosolic GFP concentration, however fixation did not prevent Tween20 induced loss of cytosolicGFP. Similarly, cytosolicGFP was decreased significantly when samples were diluted in detergent.

**Summary/Conclusion**: Our current experiments demonstrate that the use of blocking, washing and permeabilization procedures for nanoscale EV flow cytometry is complicated. The use of blocking agents can be used, but at the consequence of total sample concentration analysed. EV sample fixation is possible, does not affect fluorescent signal, but does not prevent the loss of interior EV components like cytosolic GFP when gentle detergents are used. We are now applying these data to clinical plasma samples to improve the resolution of certain biomarkers but much work remains in the field to design, optimize and standardise procedures for nanoscale flow cytometry of EVs.

**Funding**: This work was funded by Alberta Cancer Foundation, Motorcycle Ride for Dad, Prostate Cancer Canada

PF02: EVs in Cancer: Surrogate Marker Chairs: Cecilia Lasser; Sonia Melo Location: Exhibit Hall 17:15–18:30

PF02.01

Probing the role of myofibroblast-derived extracellular vesicles in cancer

Samuel J. Higginbotham; Stuart Hunt; Daniel W. Lambert

The University of Sheffield, Sheffield, UK

**Background**: The presence of cancer-associated fibroblasts (CAF) with a myofibroblastic phenotype is associated with poor prognosis in many solid tumours. A key factor in the differentiation of fibroblasts into myofibroblasts is cancer cell-derived extracellular vesicles (EV). Little, however, is known of the influence of fibroblast-derived EV on cancer cell behaviour, or whether the abundance, size or cargo of fibroblast-derived EV is altered on differentiation to a myofibroblastic CAF phenotype. Myofibroblasts show differential gene expression from resting fibroblasts and thus it was hypothesised the nature and/or cargo of extracellular vesicles secreted on differentiation are altered and that this influences the behaviour of neighbouring cancer cells. The aims of the project were to characterise the differentiation of NOFs to myofibroblasts and to assess the size, number and molecular markers of the extracellular vesicles secreted. Furthermore, the miRNA cargo of fibroblast and myofibroblast-derived EVs was analysed.

**Methods**: Primary human normal oral fibroblasts (NOF) were differentiated into myofibroblasts by incubation with TGFβ-1, as assessed by qPCR, immunofluorescence and immunoblotting of myofibroblast markers. The size and number of secreted vesicles were assessed via nanoparticle tracking analysis (ZetaView). EV purity was assessed by western blotting with vesicle markers after isolation by size exclusion chromatography and ultracentrifugation. Finally, microRNA content of the vesicles was assessed using tiling low density qPCR arrays.

**Results**: In response to TGFβ-1 treatment, fibroblasts showed increased expression of myofibroblast markers α-SMA and fibronectin EDA-1. This was associated with the appearance of α-SMA-rich stress fibres, indicative of myofibroblast differentiation. Analysis of EV-miRNA content is ongoing.

**Summary/Conclusion**: This work provides insight and a framework for further study into how the miRNA content of myofibroblast-derived vesicles may alter the TME and affect cancer progression.

PF02.02 = OWP1.05

Investigating the roles of macrophage colony-stimulating factor (CSF-1) and carbonic anhydrase 9 (CAIX) in neratinib resistant HER2+ breast cancer cell lines and extracellular vesicles

PF02.03

Cancer-testis antigens MAGE-A proteins are incorporated into extracellular vesicles released by cells

Anneli Kuldkepp^1^; Magda Karakai^1^; Olavi Reinsalu^1^; Jasper August Tootsi^1^; Reet Kurg ^2^

^1^University of Tartu, Tartu, Estonia; ^2^Institute of Technology, University of Tartu, Tartu, Estonia

**Background**: Melanoma antigens (MAGE-A) represent a unique class of tumour antigens which are expressed in a wide variety of malignant tumours, while their expression in healthy normal tissues is restricted to germ cells of testis, fetal ovary and placenta. Their restricted expression and immunogenicity make them ideal targets for immunotherapy in human cancers. MAGE-A expression is observed mainly in cancers that have acquired malignant phenotypes, invasiveness or metastasis, and the expression of MAGE-A family proteins has been linked to a poor prognosis in cancer patients.

**Methods**: Expression plasmids encoding for MAGE-A proteins were electroporated into cells and EVs were isolated from the media by differential ultracentrifugation. EVs were analysed by immunoblotting, flow cytometry and confocal microscopy using antibodies specific for MAGE-A proteins.

**Results**: We have previously shown that MAGEA4 and MAGEA10 proteins are expressed on the surface of retrovirus virus-like particles (VLP-s) induced by over-expression of MLV Gag-protein. In the current study, we have analysed the expression of MAGE-A proteins in extracellular vesicles (EVs) released by mammalian cells. We show that ectopically expressed MAGE-A proteins are incorporated into extracellular vesicles using different mammalian cell lines. MAGE-A proteins are expressed on the surface of EVs and are resistant to the treatment with salt and non-ionic detergents. MAGE-A proteins can also be used to guide recombinant proteins, e.g. EGFP and Cherry, onto the surface of EVs.

**Summary/Conclusion**: This study shows that some MAGE-A proteins are directed to the surface of EVs released by cells and they can be used to generate EVs with desired properties.

**Funding**: This work was supported by Estonian Research Council [grant IUT20-27] and by the European Regional Development Fund through the Center of Excellence in Molecular Cell Engineering.

PF02.04

Characterisation of large extracellular vesicles in paediatric medulloblastoma

Suzanne M. Johnson; Antonia Banyard; Martin G. McCabe

University of Manchester, Manchester, UK

**Background**: Medulloblastoma is the most common malignant brain tumour of childhood. Despite aggressive surgery, craniospinal radiotherapy and multi-agent chemotherapy, approximately one third of patients succumb to treatment-resistant metastatic disease within 5 years of diagnosis. Currently, time to diagnosis varies and is reliant on vigilant observation of generic symptoms coupled with the availability of imaging facilities to observe tumour mass in the brain. Early detection and improved treatment strategies are therefore urgently needed.

We have previously shown that tumour derived EVs, distinct from exosomes, could be detected in the peripheral blood. This observation led us to hypothesise that these larger EVs could serve as biomarker reservoirs with potential value to the clinic.

**Methods**: EV isolation was performed using a combination of filtration, differential centrifugation and size exclusion chromatography to preserve the integrity of EV membranes and collect all EVs for investigation. We characterized a sub-group of larger EVs (>300 nm) using a panel of fluorescent markers including PKH26, CD133 and CD15 by fluorescence microscopy and imaging flow cytometry. Transmission electron microscopy provided verification of membrane integrity and intra-vesicle content.

**Results**: We found that EVs produced by medulloblastoma cell lines express filamentous actin and a range of surface markers which may help to identify the cell of origin in clinical samples. MB-EVs contain cell adhesion molecules and medulloblastoma sub-group specific markers such as β-Catenin. Correlative analysis is on-going with a focus on EV sub-group specific expression patterns.

**Summary/Conclusion**: Our observations suggest that larger EVs may have the potential to carry multiple markers which could identify their cell of origin and therefore have some use as a malignancy indicator in a clinical setting. Future work will extend these investigations to primary tumours and clinical samples.

**Funding**: This work was funded by Teenage Cancer Trust, Christie Hospital Research Fund

PF02.05

Rhabdomyosarcoma exosome proteomics yield functional role for extracellular vesicles

Sandra E. Ghayad ^1^; Ghina Rammal^2^; Farah Ghamloush^3^; Mona Diab-Assf^4^; Firas Kobeissy^5^; Yehia Mechref^6^; Raya Saab^7^

^1^Department of Biology, Faculty of Science II, Lebanese University, Fanar, Lebanon, Bsalim, Lebanon; ^2^Department of Biology, Faculty of Science II, Lebanese University, Fanar, Lebanon, Beirut, Lebanon; ^3^Children’s Cancer Institute, American University of Beirut, Beirut, Lebanon, Beirut, Lebanon; ^4^Department of Chemistry and Biochemistry, Faculty of Sciences II/EDST, Lebanese University, Lebanon, Fanar, Lebanon; ^5^Department of Biochemistry and Molecular Genetics, Faculty of Medicine, American University of Beirut, Beirut, Lebanon; ^6^Department of Chemistry and Biochemistry, Texas Tech University, Lubbock, TX, USA; ^7^Department of Anatomy, Cell Biology and Physiology, American University of Beirut, Beirut, Lebanon; and, Children’s Cancer Institute, American University of Beirut, Beirut, Lebanon

**Background**: Rhabdomyosarcoma (RMS) is an aggressive childhood soft tissue tumour with two distinct subtypes, embryonal (ERMS) and alveolar (ARMS) histologies. Exosomes (Exo) are important intercellular communication vehicles secreted into body fluids by multiple cell types, including tumour cells. They have been demonstrated to contribute to the metastatic progression of tumour cells through paracrine signaling. Tumour Exo contain intact and functional protein, mRNA and miRNA that may alter the cellular environment to favour tumour growth. Thus we evaluated the protein cargo of RMS-derived Exo and the molecular pathways they are implicated in to decipher their role in the progression of this aggressive disease

**Methods**: We isolated and characterized Exo from three ERMS and two ARMS cell lines. In order to determine the protein content within these Exo, we conducted a mass spectrometry analysis validated by multiple reaction monitoring (MRM) and western blot. We determined the biological processes and pathways in which the different exosomal protein cargos are involved using Panther classification system software.

**Results**: Results revealed the expression of 161 common proteins in all three ERMS-derived Exo and 122 common proteins in both ARMS-derived Exo among which 81 proteins were common to both subtypes. These commonly expressed proteins include, not only exosomal markers, but also proteins involved in “cell signaling”, “cell movement” and “cancer”. The pathways engaging the identified proteins revealed 37 common pathways including “integrin signaling pathway”, “inflammation mediated by chemokine and cytokine signaling pathway” and “angiogenesis”. These pathways may contribute to the paracrine signaling in tumour progression. Finally, the comparison of exosomal proteins of RMS cells with the datasets of exosomal proteins from other cancer cells available in Exocarta revealed that 62 proteins were specific to RMS Exo and were not found in other cancer Exo.

**Summary/Conclusion**: Through identifying the protein cargos of RMS-derived Exo and the pathways involving these proteins, we were able to highlight the important role played by Exo in cancer progression. Our results revealed that RMS-derived Exo may carry cell/tissue specific proteins, which may provide some new biomarkers for diagnosis and indicate Exo functions in RMS.

**Funding**: This work was funded by Lebanese University grant and MPP grant from AUB

PF02.06

Exosomes derived from hypoxic GBM cells deliver miR-25 to normoxic cells to elicit chemoresistance

Jiwei Wang ^1^; Taral Lunavat^2^; Wenjing Zhou^2^; Mingzhi Han^2^; Krister Stokke^2^; Frits Thorsen^2^; Rolf Bjerkvig^2^; Jian Wang^2^; Xingang Li^3^

^1^University of Bergen, Bergen, Norway; ^2^Department of Biomedicine, University of Bergen, Bergen, Norway; ^3^Department of Neurosurgery, Qilu hospital, Shandong University, Jinan, China (People’s Republic)

**Background**: Hypoxia is one of the crucial microenvironments to promote chemotherapy resistance in glioblastoma multiforme (GBM). Exosomes, initially considered to be cellular “garbage dumpsters”, are now implicated in mediating interactions with cellular environment. However, mechanisms underlying the association between exosomes and hypoxia-induced chemoresistance during cancer progression remain poorly understood.

**Methods**: The role of hypoxic and normoxic exosomes from GBM cells was investigated using *in vitro* and *in vivo* assays to assess cell proliferation/survival and invasion. MiRNA sequencing of normoxic and hypoxic GBM-derived exosomes was performed. Real-time PCR was used to quantify miR-25 expression. Luciferase reporter assays were conducted to confirm target gene associations.

**Results**: In this study, we found that exosomes derived from hypoxic GBM cells increased the proliferation and invasion of normoxic GBM cells by enhancing the glycolytic capacity. These alterations endowed normoxic GBM chemoresistance by activating PI3K-AKT signaling pathway. Of the 302 miRNAs that were differentially expressed in miRNA sequencing, miR-25 stood out as one of the most significantly upregulated miRNAs under hypoxic conditions. miR-25 depletion in hypoxic GBM cells led to decreased miR-25 levels in exosomes and significantly reduced hypoxic exosomes induced chemoresistance. Finally, we found that miR-25-mediated regulation of cellular chemoresistance involved downregulated expression of the target protein, PHLPP2 that is crucial for activating PI3K-AKT signaling patyway.

**Summary/Conclusion**: In conclusion, our findings suggest that hypoxic microenvironment may stimulate GBM cells to generate miR-25-rich exosomes that are delivered to normoxic cells to promote chemoresistance.

PF02.07

The effect of hypoxia on extracellular vesicles secretion from renal carcinoma and normal embryonic kidney cells

Anatoliy Samoylenko ^1^; Artem Zhyvolozhnyi^2^; Naveed Ahmad^1^; Qi Xu^1^; Fabienne Wagner^3^; Khem Giri^4^; Genevieve Bart^1^; Seppo Vainio^1^

^1^Oulu University, Oulu, Finland; ^2^Palladin Institute of Biochemistry, National Academy of Sciences of Ukraine, Kiev, Ukraine; ^3^Wurzburg University, Wurzburg, Germany; ^4^IECM, ONIRIS, USC1383 INRA, Nantes, France, Nantes, France

**Background**: Intratumoural hypoxia is believed to be one of the key factors involved in cancer progression. Though a number of recent studies indicated that hypoxia promotes secretion of EV by tumour cells, the molecular mechanisms of EV action in carcinogenesis under hypoxia remain unclear.

**Methods**: We purified EV from culture media from Renca renal carcinoma and embryonic kidney UBtip cells, kept for 24 h under normoxia or hypoxia (1% oxygen), by sequential centrifugation and Exo-spin™ columns. The EV was characterized by electron and confocal microscopy, nanoparticle tracking analysis (NTA) and western blotting using EV-specific markers. Cells proliferation and viability were assayed by live cell imaging using IncuCyte ZOOM (Essen BioScience), mRNA expression by qPCR. EV proteins were sequenced by ultra-performance liquid chromatography-mass spectrometry (UPLC-MS) and total RNA on NextSeq550 (Illumina).

**Results**: Microscopy and NTA showed that hypoxia treatment significantly increased the EV concentration in culture media from Renca cells but not from UBtip cells. We also found that EV isolated from cancer-derived but not embryonic kidney-derived cells influence cellular motility in culture. These EV also differentially regulated expression of several genes in target cells. Hypoxia changed protein and RNA content of Renca-derived and UB-derived exosomes. Among the pathways found to be influenced by hypoxia in cancer-derived EV was caveolar-mediated endocytosis signaling. We have recently showed the involvement of the same pathway in the renal carcinogenesis by using a novel renal cancer/normal kidney cells co-culture system.

**Summary/Conclusion**: Hypoxia induces production of EV by renal carcinoma cells and changes EV’s protein and RNA content. The role of identified potential hypoxia cancer markers will be further analysed using our recently developed renal cancer/normal kidney cells co-culture system.

**Funding**: The study was supported by CIMO and Finnish Cancer Foundation grants.

PF02.08

New and improved methods for characterization of tumour-specific markers in exosomes

Carmen Campos Silva ^1^; Sheila López Cobo^1^; Henar Suárez Montero^2^; Amanda Moyano Artime^3^; Annette Paschen^4^; Maria del Carmen Blanco-López^3^; María Yáñez-Mó^5^; Mar Valés^6^

^1^Immunology and Oncology Department, Spanish National Centre for Biotechnology (CNB-CSIC), Madrid, Spain, Madrid, Spain; ^2^Molecular Biology Center Severo Ochoa (CBM), Madrid, Spain., Madrid, Spain; ^3^Department of Physical and Analytical Chemistry, Faculty of Chemistry, University of Oviedo, Oviedo, Spain, Oviedo, Spain; ^4^Department of Dermatology, University Hospital Essen, University Duisburg-Essen, Essen, Germany., Essen, Germany; ^5^Departamento de Biología Molecular. UAM, Madrid, Spain; ^6^Immunology and Oncology Department, Biotechnology National Center (CNB-CSIC), Madrid, Spain, Madrid, Spain

**Background**: The activation of the immune system mediated by engagement of NKG2D with its ligands is a crucial step in the regulation of both innate and specific immune responses, in particular, in immune recognition of cancer. NKG2D-ligand expression is upregulated when the cells suffer different types of stress, notably tumoural transformation. However, the NKG2D-mediated response can be modulated by the release of these molecules to the extracellular milieu, leading to immune evasion.

**Methods**: To generate tools that facilitate investigation of the role of the presence of the NKG2D-ligand MICA in tumour derived-exosomes, we have analysed different methods for the detection and characterization of tumour-derived exosomes including newly developed lateral flow devices and bead capture-based flow cytometry tests.

**Results**: Comparison of the different techniques; Western blot, ELISA, flow cytometry and lateral flow, demonstrates that the use of the same combination of tetraspanins and tumour markers antibodies can result in very different outcomes when using different techniques. In fact, when optimising the combinations and concentrations of antibodies for use in each technique, special care had to be taken due to the risk of multimeric aggregate formation.

**Summary/Conclusion**: Translating methods originally established for detection of soluble molecules into the detection of vesicles needs a careful optimization of each technique. The implications of these data for the detection of tumour markers in exosomes of biological samples will be discussed.

**Funding**: This work has been supported by grants from the Spanish Ministry of Economy (MINECO/FEDER) [SAF2015-69169-R and the Network of Excellence for Research in Exosomes, Rediex. CCS was a recipient of a master’s fellowship from “Fundación Ramón Areces-UAM” and later of a postgraduate fellowship “JAE Intro” from the CSIC; SLC was a recipient of a travel fellowship from Geivex. The authors and Immunostep collaborate in an R&D project (CSIC-UAM).

PF02.09

Oncogenic role of B7-H3 from tumour cell-derived extracellular vesicles

Caroline E. Nunes-Xavier; Karine Flem-Karlsen; Øystein Fodstad

Department of Tumor Biology, Institute for Cancer Research, Oslo University Hospital Radiumhospitalet, Oslo, Norway

**Background**: The human B7-H3 molecule is an immunoregulatory protein which consists of 534 amino acids in its predominant longer form, but it is also present as a shorter isoform, as well as soluble isoforms. B7-H3 protein is not expressed, or is expressed at low levels, in most normal cells or tissues. In contrast, B7-H3 protein is overexpressed in many types of malignancies, which is linked to poor prognosis, increased tumour grade and decrease in overall survival. However, the molecular basis for the functional roles of B7-H3 in cancer is poorly known. We have identified an oncogenic non-immunological role of B7-H3 in melanoma and breast cancer cells, which promotes metastasis and resistance to therapy.

**Methods**: Extracellular vesicle purification and analysis

Tumour cells were grown in appropriate cell culture media with exosome depleted FBS for 3 days. Extracellular vesicles were purified by sequential centrifugation of cell culture supernatant, first by centrifugation at 1000 g for 5 min, then 2500 g for 10 min. To remove larger cell debris and possible apoptotic bodies, supernatant was centrifuged at 20,000g for 20 min (Beckman coulter, JA-25.50 rotor). Finally, extracellular vesicles were collected by centrifugation at 100,000g for 70 min (Beckman coulter, 70Ti rotor), and washed in 10 ml PBS. Extracellular vesicle fraction was verified by electron microscopy, used for *in vitro* functional studies, lysed and analysed by immunoblot.

**Results**: We detect B7-H3 in the membranes of tumour cells, in intracellular vesicles, as well as in the secretome, and will present preliminary data on the oncogenic role of B7-H3 from tumour cell-derived extracellular vesicles.

**Summary/Conclusion**: As extracellular vesicles can travel in the body and fuse to recipient cells at a distance, cancer-derived extracellular B7-H3 could be transferred to recipient cells thereby inducing proliferation and metastasis or resistance to therapy. In this manner, the extracellular isoforms could also contribute to the increased invasion and metastatic capacity driven by B7-H3.

**Funding**: This work was supported by the following grants: KFK, ØF: The Norwegian Cancer Society, and CENX: The Research Council of Norway [grant number: 239813]. We also thank Arne E. Ingels` legacy for financial support. This communication was funded by diseases (MDPI) 2017 travel award.

PF02.10

Analysis of nuclear and mitochondrial DNA ratio in human melanoma cell line derived vesicles

Jörg P. Burgstaller^1^; Sabine Brandt^2^; Gottfried Brem^3^; Daniela M. Brodesser ^4^

^1^Biotechnology in Animal Production, Department for Agrobiotechnology, IFA Tulln, Tulln, Austria;, Tulln, Austria; ^2^Equine Clinic; University of Veterinary Medicine Vienna, Vienna, Austria; ^3^Institute of animal breeding and genetics; University of Veterinary Medicine Vienna, Vienna, Austria; ^4^Biotechnology in Animal Production, Department for Agrobiotechnology, Tulln, Austria

**Background**: It is now established that extracellular vesicles (EVs) have a significant role in cancer signaling. EVs are frequently reported to contain nucleic acids in varying amounts, covering both the nuclear (nDNA) and mitochondrial (mtDNA) genome. EVs are routinely extracted by differential centrifugation of cell culture supernatant, thereby separating defined EV fractions (apoptotic bodies; microvesicles; exosomes). In this study, we analysed mtDNA: nDNA ratio within these fractions in three human melanoma cell lines and human fibroblasts as control.

**Methods**: Melanoma Cell lines (A375, SKMel28, 518A2) and human fibroblasts were cultured in exosome depleted media for 24 h; EVs were isolated with differential centrifugation of cell culture supernatant. DNA of each respective pellet enriched for apoptotic bodies, microvesicles and exosomes was extracted. MtDNA to nDNA ratio was quantified by qPCR (nuclear DNA: single copy gene MIA GenBank [NC_000019.10]; mtDNA: MT-ND4 GenBank [NC_012920]).

**Results**: Preliminary data indicate that the ratio of nDNA to mtDNA increases with each step in differential centrifugation. This suggests that in exosomes the relative amount of mtDNA is reduced compared to microvesicles, and even more in apoptotic bodies, irrespective of analysed cellular origin.

In an ongoing study the ratio of extravesicular vs intravesicular nucleic acids in the respective centrifugation pellets is analysed.

**Summary/Conclusion**: The ratio of nDNA to mtDNA increases with each step in differential centrifugation, both in melanoma and fibroblast cell lines. Further research is needed to discriminate further between extravesicular vs. intravesicular nucleic acids in the respective centrifugation pellets, and to elucidate the underlying mechanisms of nucleic acid distribution in EVs.

**Funding**: This work was supported by [grant LS15-020] (to J.P.B. and S.B.) from Life Science Calls, Niederösterreichische Forschungs-und Bildungsge-sellschaft (NFB; http://www.nfb.at/)

PF02.11

A comparison of extracellular vesicle subtypes released by triple-negative breast cancer and non-tumorigenic breast epithelial cells

Joana Cabral ^1^; Shirley Hanley^2^; Susan Logue^3^; Rhodri Ceredig^1^; Afshin Samali^1^; Manus Biggs^1^; Matthew D. Griffin^1^

^1^Centre for Research in Medical Devices (CÚRAM) at National University of Ireland, Galway, Galway, Ireland; ^2^Flow Cytometry Core Facility at National University of Ireland, Galway, Ireland; ^3^Apoptosis Research Centre at National University of Ireland, Galway, Ireland

**Background**: Triple-negative breast cancer (TNBC) poses a serious therapeutic challenge. Neoplastic transformation affects extracellular vesicle (EV) biogenesis and cancer cell-derived EVs may mediate immune modulation, chemo-resistance and metastasis. Cancer-associated mechanisms that regulate EV production and function require further investigation. This study aimed to enumerate and characterize the major EV subtypes released by a human TNBC cell line compared to a non-malignant breast epithelial cell line (nmBEC).

**Methods**: Three EV sub-fractions were isolated from supernatants of TNBC cells (MDA-MB-231) and nmBECs (MCF/10A) by sequential centrifugation steps: 2000 ×*g* (apoptotic body [*AB*]-enriched), 12000 ×*g* (ectosome-enriched) and 120000 ×*g* (exosome-enriched). EV marker expression was characterized by immunogold TEM and latex-bead-based flow cytometry. Quantification of EVs was performed by BCA protein assay and nanoparticle tracking analysis (NTA).

**Results**: Mid-to-high-level expression of CD9, CD63 and CD81 was confined, as expected, to the exosome-enriched fractions of both cell types. Protein assays correlated poorly with NTA-based particle counts with the greatest discrepancies seen for *AB*- and ectosome-enriched fractions from both cell types. The release rate (Particles/cell/72 h) of ectosome- and exosome-enriched fractions from TNBC cells was several-fold greater than from nmBECs. Mean and mode particle sizes of ectosome- and exosome-enriched fractions were similar for TNBC cells compared to nmBECs.

**Summary/Conclusion: (**1). A TNBC-derived cell line demonstrated higher release rate and similar size of ectosomes and exosomes compared to a non-cancer-derived breast epithelial cell line. (2). Careful quantitative and qualitative analysis of EV sub-fraction release from cultured TNBC cell lines and primary tumour cells has potential as a screening approach for novel anti-cancer agents and for discovery of EV-associated mechanisms of TNBC progression.

**Funding**: This work was funded by Science Foundation Ireland (SFI) and the European Regional Development Fund (Grant Number 13/RC/2073), Manus Biggs is funded by a joint SFI/BBSRC grant [Grant number 16/BBSRC/3317] and Susan Logue is funded by SFI Starting Investigator Research Grant [Grant Number 15/SIRG/3528]

PF02.12

Characterizing the extracellular vesicle (EV) production of colorectal cancer cell lines

Zsuzsanna Szvicsek ^1^; Adam Oszvald^1^; Istvan Kovacs^1^; Gyongyver Orsolya Sandor^1^; Aniko Zeold^1^; Andrea Kelemen^1^; Edit Buzas^1^; Zoltan Wiener^1^

^1^Semmelweis University, Department of Genetics, Cell and Immunobiology, Budapest, Budapest, Hungary

**Background**: Colorectal cancer (CRC) is one of the most frequent causes of cancer-related death in the Western countries. CRC is a heterogeneous disease with different molecular background and clinical manifestations. Interestingly, colorectal cancer cell lines (CCCLs) can be classified into categories similarly to CRC patients. The potential use of EVs in the early diagnosis of tumours is based on the assumptions that (1) EV production increases during tumorigenesis and (2) tumour-derived EVs carry a specific molecular pattern. Here we studied the EV production of CCCLs from different CRC groups and the effect of external factors on EV production.

**Methods**: We analysed CCCL-derived EVs by qNano and measured their EV production by bead-based methods and FACSCalibur. We also used publicly available gene expression data and we measured gene expression by RT-qPCR.

**Results**: We observed a large heterogeneity in the EV production among CCCLs, although all of them secreted both CD81+ and CD63+ EVs. We could not detect a correlation between the EV production and the subtype or mutations of CCCLs. Furthermore, selected external factors, such as HGF, IL-11, IL-22 or TNF alpha had no major influence on the EV production. This suggests that these stroma-derived factors are not central in the elevated EV release from CRC tumour cells.

**Summary/Conclusion**: All studied CCCLs produced EVs, however, the analysed stromal factors did not have a major influence on the EV secretion of CRC cells.

**Funding**: This work was supported by the OTKA-NN [118018] and the National Competitiveness and Excellence Program Hungary [NVKP_16-1-2016-0007, NVKP_16-1-2016-0017] by the National Research, Development and Innovation Office (Hungary), by the Semmelweis University Starting Grant and by the [ICGEB-CRP_ HUN16-04_EC] (International Centre for Genetic Engineering and Biotechnology, Italy). Z.W. and A.Z. are supported by the János Bolyai Fellowship (Hungarian Academy of Sciences).

PF02.13

Increased amounts of cancer-related membrane molecules in extracellular vesicles secreted from ganglioside-enriched cancer cell lines

Koichi Furukawa; Iori Kobayashi; Yoshiteru Kodama; Yuhsuke Ohmi; Satoko Yamamoto; Rika Takeuchi; Keiko Furukawa

Chubu University College of Life and Health Sciences, Kasugai, Japan

**Background**: Cancer-associated glycosphingolipids have been considered to be tumour markers, and used as targets of cancer treatment. We have analysed functions of gangliosides in malignant melanomas and gliomas etc, and reported that cancer-associated gangliosides enhance malignant properties of cancer cells by forming complexes with various cancer-related membrane molecules, such as growth factor receptors and integrins. In this study, we have tried to examine the contents of extracellular vesicles (ECVs) secreted from ganglioside-enriched cancer cells in order to clarify roles of ECVs in the regulation of cancer microenvironments by individual cancer cells.

**Methods**: Ganglioside GD3 synthase (ST8SIA1) cDNA was introduced into GD3-negative cell lines of melanoma, glioma and small cell lung cancer, leading to establishment of GD3-positive cells as well as GD3-negative control cells. ECVs were collected from culture supernatants by repeated ultra-centifugation (100,000 g pellets from 20,000 g supernatants) combined with filtration. Contents in ECVs were analysed by Western blotting.

**Results**: In ECVs from GD3-positive melanoma cells, GD3 was detected in thin-layer chromatography. They also contained GD3 synthase mRNA as analysed by RT-qPCR. In Western blotting, increased levels of integrin family members in ECVs were shown in GD3-positive cells compared with those in GD3-negative cells, while integrin levels in cell lysates were almost equivalent between GD3-positive and GD3-negative cells. Particularly in melanoma cells, levels of integrin α2, β1 and β2 were markedly increased in GD3-positive cell-derived ECVs. Levels of epidermal growth factor receptors in ECVs also showed similar increases compared with ECVs from GD3-negative cells.

**Summary/Conclusion**: Effects of GD3 expression on the conposition of ECVs of cancer cells suggested that these increased membrane molecules play roles in the invasion, migration and metastasis cooperating with gangliosides in ECVs.

**Funding**: This study was supported by a Grant-in-Aid from the Ministry of Education, Culture, Sports and Technology (MEXT) of Japan.

PF02.14

Role of microRNA-335-5p in gastric cancer derived extracellular vesicles

Iva Polakovicova ^1^; Edison Salas-Huenuleo^2^; Lorena Lobos-González^3^; Manuel Varas-Godoy^4^; Nicolas Carrasco-Veliz^5^; Alejandro Corvalán^6^

^1^Laboratory of Oncology, Faculty of Medicine, Pontifícia Universidad Católica de Chile; Center for Chronic Diseases, Pontifícia Universidad Católica de Chile, Santiago, Chile; ^2^Laboratory of Nanobiotechnology and Nanotoxicology, Facultad de Ciencias Químicas y Farmacéuticas, Universidad de Chile, Santiago, Chile; ^3^Fundación Ciencia y Vida, Andes Biotechnologies, Santiago, Chile; ^4^Centro de Investigación Biomédica, Faculty of Medicine, Universidad de Los Andes, Santiago, Chile; ^5^Departament of Chemistry, Faculty of Science, Pontifícia Universidad Católica de Valparaíso, Santiago, Chile; ^6^Laboratory of Oncology, Faculty of Medicine, Pontifícia Universidad Católica de Chile; Advanced Center for Chronic Diseases, Pontifícia Universidad Católica de Chile, Santiago, Chile

**Background**: MicroRNA-335-5p (miR-335) has been reported to be dysregulated in various types of cancer, including gastric cancer (GC). Recently, we have shown the downregulation of miR-335 in GC tissues relative to their paired adjacent non-tumour tissues. We have also demonstrated that miR-335 is downregulated in plasma samples from GC patients. Here, we aimed to investigate the expression of miR-335 in GC derived extracellular vesicles (EVs) in plasma. Moreover, we focused on exosomes loaded with miR-335, their effect after incorporation in GC cells, and their biodistribution *in vivo.*

**Methods**: EVs were isolated from a cohort of 12 plasma patients’ samples, from supernatants from two GC cell lines, a primary tumour derived cell line AGS and metastatic derived cell line HS746T, and from cells transfected with miR-335 mimics and characterized by western blot and nanosight. MiR-335 expression levels in plasma EVs, cell lines, transfected cells and their EVs, as well as expression of target genes of miR-335, were analysed by qPCR. Incorporation of EVs into cells was quantified by flow cytometry. In biodistribution studies, EVs were labeled with fluorescent dye DiR, injected intravenously in the tail of mice (3 per condition) and their distribution in time was evaluated using *in vivo* visualizing machine. Brain, heart, lung, spleen, kidney, liver, stomach, colon, intestine and bone marrow were evaluated. Plasma samples were obtained with written informed consent from patients. Animal studies were approved by ethical committee.

**Results**: Our cohort of patients show a tendency that plasma EVs isolated from GC patients contain less miR-335 when compared to healthy donors. *In vitro* data demonstrate that upon uptake of miR-335-enriched EVs by GC cells, the expression of CDH11 and PLAUR is altered in a similar manner as these genes are regulated in GC cells transfected with miR-335. *In vivo* studies in mice shows, that after intravenous injection of these EVs labeled with DiR, EVs enriched in miR-335 show different distribution in time in several organs, including stomach, in comparison to control EVs.

**Summary/Conclusion**: MiR-335 is present in EVs isolated from both plasma and GC cell culture supernatants. EVs enriched in miR-335 show functional properties after cell uptake and different biodistribution in mice.

**Funding**: This work was funded by FONDECYTs [3160592, 11140204, 11150624, 1151411], FONDAP [15130011]

PF02.15

Initiation of leukemia phenotype via extracellular vesicles

Theo Borgovan ^1^; Peter Quesenberry^2^; Laura Goldberg^1^; Pam Egan^1^; Chibuikem Nwizu^3^

^1^Brown University / Rhode Island Hospital Hematology Oncology Department, Providence, USA; ^2^Brown University, Providence, USA; ^3^Brown University Medical School, Providence, USA

**Background**: We have shown that extracellular vesicles (EVs) from explant prostate cancer induce a neoplastic phenotype in normal prostate cell lines. We have also shown EVs from mesenchymal stem cells (MSC) can have a healing effect, reversing the malignant phenotype in prostate and colorectal cancer; as well as mitigating radiation damage to marrow. The role of EVs in leukemia and its microenvironment remains to be studied, and may provide insight for therapeutic advances. We hypothesize that EVs derived from normal MSC can have a healing effect, inhibiting the growth of myelogenous leukemia.

**Methods**: Kasumi AML cells lines were seeded in a 96 well plate with various concentrations of MSC-derived EVs. Vesicles were isolated using an established differential centrifugation technique, and were co-cultured with Kasumi. To study cellular proliferation we employed the CyQUANT Assay, a fluorescence-based method for quantifying viable cells. Fluorescence was measured after 60 min. Fluorescence intensities were normalized to control wells containing non-EV treated cells alone.

**Results**: Proliferation of AML cells after one day of co-culture with 2.6^8^ &1.3^10^ MSC-EVs respectively was inhibited in a dose dependent manner: with 2.6E8 EVs leading to ~ 15% reduction in growth, and 1.3^10^ EVs leading to ~60& reduction when normalized to non-EV treated controls.

Three days of co-culture with similar doses resulted in ~40% and ~80% reduction in proliferation when normalized to control.

At day 6 of co-culture growth was inhibited by ~80% at both EV concentrations when normalized to control.

**Summary/Conclusion**: MSC-derived EVs inhibits the growth of the AML cell line *in vitro*. This effect is seen as early as 1 day of co-culture and persists out to 3, and 6 days implicating an miRNA-mediated mechanism that has been discussed in previous works. We feel this is perhaps a model of how a normal marrow works to suppress early cancer.

As leukemia develops the cross-talk between AML and its microenvironment alters the MSCs to promote a survival signal favouring AML growth. Future work involves the capacity of AML-derived EVs to alter the phenotype of normal marrow towards a pro-leuekmic phenotype. Employing mathematical models to quantify and ultimately predict these changes allows for precise therapeutic intervention.

**Funding**: This work was funded by NIH [T32 Grant].

PF02.16

Endocytosis and intracellular trafficking of prostate cancer exosomes

Alex Cocks ^1^; Hope Roberts-Dalton^1^; Philip Lewis^1^; Jason P. Webber^2^; Rachel Errington^2^; Peter Watson^1^; Arwyn Jones^1^; Aled Clayton^2^

^1^Cardiff University, Cardiff, UK; ^2^Tissue Microenvironment Group, Division of Cancer and Genetics, School of Medicine, Cardiff University, Cardiff, UK

**Background**: Prostate cancer exosomes interact with fibroblasts in the tumour microenvironment to stimulate myofibroblast differentiation, generating a stroma that supports tumour growth. We propose that uptake of prostate cancer exosomes and delivery of their cargo to the fibroblast is required to generate this disease promoting phenotype. The microscopy techniques available enable us to determine the fate of the exosome following uptake. Understanding the uptake kinetics of exosomes and their intracellular trafficking may provide insights into how exosomes induce myofibroblast differentiation, and how they could be manipulated therapeutically.

**Methods**: A novel thiol based labelling technique was carried out to allow visualization and quantification of exosomes taken up by fibroblasts, by fluorescence microscopy and flow cytometry respectively. The endocytic routes used by exosomes to gain entry to fibroblasts was determined utilising siRNA mediated knockdowns of endocytic regulators, and intracellular trafficking of the exosomes was monitored by time-lapse microscopy.

**Results**: Fluorescent thiol labelling allows visualization of exosomes, but does not affect the exosome function with respect to myofibroblast differentiation. Exosomes are taken up by fibroblasts through Clathrin mediated endocytosis and traffic towards lysosomes. Modulation of exosome uptake through interference with the exosome surface is ongoing.

**Summary/Conclusion**: Endocytosis of exosomes can be perturbed by targeting regulators of endocytosis, as well as proteins on the exosome surface revealing that uptake of exosomes by fibroblasts can be modulated. Utilising diverse microscopy techniques clarifies the fate of the exosome within the fibroblast. The impact of uptake inhibition on the ability for fibroblasts to differentiate into pro-tumoural myofibroblasts is currently being examined.

**Funding**: This project is funded by Tenovus Cancer Care

PF02.17

Lysosomal inhibition in triple-negative breast cancer cells alters exosome composition and function

Jing Xu ^1^; Shane Colborne^1^; Elham Hosseini-Beheshti^2^; Emma Guns^3^; Gregg Morin^4^; Sharon Gorski^4^

^1^Canada’s Michael Smith Genome Sciences Centre, Vancouver, Canada; ^2^Vancouver Prostate Centre, Sydney, Australia; ^3^Vancouver Prostate Centre, Vancouver, Canada; ^4^Canada’s Michael Smith Genome Sciences Centre, Vancouver, Canada

**Background**: Triple-negative breast cancer (TNBC) is a subtype of aggressive breast cancer that lacks estrogen, progesterone and HER2 receptors. Consequently, chemotherapy is one of the main treatment options of TNBC. Macroautophagy (hereafter autophagy) is a catabolic pathway where lysosomal degradation of organelles and proteins provides nutrients that support cellular functions. Previous work demonstrated pro-survival roles of autophagy in TNBC and precipitated investigations to evaluate whether concurrent lysosomal inhibition improves chemotherapy efficacy. As lysosome and autophagy machinery interact extensively with the endocytic pathway that gives rise to exosomes, a type of extracellular vesicle of relevance in cancer, we investigated the effect of lysosomal inhibition on the content and function of TNBC-derived exosomes.

**Methods**: TNBC cell line conditioned media was pre-cleared using differential centrifugation and concentrated using centrifugal filtration. The ExoQuick reagent was used to precipitate exosomes. western blotting, NanoSight, and transmission electron microscopy were used to characterize the exosomes isolated. Mass spectrometry was used to identify exosomal proteins. Proliferation and tube-formation of endothelial (HMEC-1) cells treated with TNBC-derived exosomes were measured as surrogate markers of angiogenesis.

**Results**: Treatment of TNBC cell lines with lysosomal inhibitor chloroquine (CQ) blocked autophagy turnover and altered exosome biogenesis. CQ-treated TNBC produced fewer but more protein-rich exosomes compared to the controls. CQ treatment also altered the level of exosomal autophagy-related proteins. Exosomes derived from control and CQ-treated TNBC cells had different effects on HMEC-1 growth and tube formation.

**Summary/Conclusion**: Perturbation of lysosomal physiology can impact both macroautophagy and exosomal cargo and function. Presence of autophagy-related proteins suggests the potential involvement of autophagy machinery in exosome biogenesis.

**Funding**: This work is supported by CIHR.

PF02.18

Extracellular vesicle cargo is indicative of HPV status in oropharyngeal carcinoma

Ben Peacock; Daniel W. Lambert; Keith Hunter; Stuart Hunt

The University of Sheffield, Sheffield, UK

**Background**: Viruses are capable of manipulating host endosomal-exosomal pathways which can aid in tumourigenesis. Human papilloma virus (HPV) encoded proteins can alter the production and cargo of extracellular vesicles (EVs) secreted by cervical cancer cells. However, the extent of HPV’s oncogenic properties relating to EV release in oropharyngeal carcinoma (OPC) is not well understood. Here, we aimed to evaluate differences in size, quantity and molecular contents of EVs released by HPV positive (HPV+) and HPV negative (HPV-) OPC cell lines.

**Methods**: EVs were purified from the conditioned medium of OPC cell lines by size exclusion chromatography. EV size and concentration was measured by tuneable resistive pulse sensing (TRPS). Transmission electron microscopy was used to validate size measurements made by TRPS. Vesicular protein and RNA were extracted for subsequent mass spectrometry and small RNA sequencing, respectively.

**Results**: There was no significant difference in the modal diameter of vesicles released by HPV+ compared to HPV- cell lines (*n* = 9). However, HPV- cells produced significantly more EVs (up to two-fold) than HPV+ cells (*n* = 9, *p* value < 0.05). A total of 90 proteins were identified that showed a significantly different abundance based on HPV status (*p* value < 0.05). EGFR was only detected in EVs from HPV- cells. Bioinformatics analysis of miRNA abundance data revealed that samples clustered based on the HPV status of the producing cell.

**Summary/Conclusion**: The current study highlights that the molecular EV cargo (protein and miRNA) is correlated with the HPV status of the cell of origin, suggesting a differing role in the tumour microenvironment and their potential use as a source of circulating biomarkers in OPC.

PF02.19

Pancreatic cancer stem cell exosomes orchestrate an organized intra-tumour communication network that contributes to tumour dynamics and plasticity

Sonia A. Melo ^1^; Carolina Ruivo^1^; Nuno Bastos^1^; Barbara Adem^1^; Carlos Melo^2^; Pedro Moutinho^3^; Guilherme Macedo^4^; Jose Carlos Machado^1^; Raghu Kalluri^5^

^1^i3S - Instituto de Investigação e Inovação da Universidade do Porto, Porto, Portugal; ^2^The Gurdon Institute - University of Cambridge, Cambridge, United Kingdom; ^3^Serviço de Gastrenterologia do Hospital de São João, Porto, Portugal; ^4^Gastroenterology Service of Centro Hospitalar de São João (CHSJ), Porto, Portugal; ^5^MD Anderson Cancer Center, Houston, USA

**Background**: Tumours are heterogeneous, composed of different subpopulations of cancer cells with phenotypic, genetic and epigenetic differences. Phenotypic differences such as in metabolism, gene expression, migration, proliferation, angiogenesis and metastatic potential are characteristic of different subpopulations of cancer cells. Recent studies indicate that cancer cell subpopulations interact with each other in a cooperative effort to promote invasion, tumour growth, and metastasis. The evidence that cancer cell subpopulations behave as a community, establishing a cooperative process adds an extra challenge to therapeutic intervention. This illustrates the importance of understanding the mechanism(s) behind the cooperation of subpopulations of cancer cells, and explore their potential as novel therapy targets in cancer. Exchange of genetic and molecular information through exosomes is one of the preferred routes for intercellular communication.

**Methods**: We use co-cultures of subpopulations of pancreatic-cancer cell lines that secrete color-coded exosomes, genetically engineered mouse models, and pancreatic cancer patient derived xenografts to show that cancer stem cells exosomes mediate an organized intra-tumour communication network involved in tumour progression.

**Results**: Our results show that the flow of exosomes between subpopulations of pancreatic cancer cells occurs in a non-random way, and that preferential routes of communication are established from cancer stem cells to non-cancer stem cell subpopulations. This network of communication is dynamic and re-shapes its communication routes in different conditions.

**Summary/Conclusion**: Our preliminary results indicate that the intra-tumour network of communication mediated by exosomes (ExoNet) confers the tumour the dynamic ability to efficiently adapt to the environment, in a reversible manner.

**Funding**: This work was funded by FEDER and COMPETE 2020 (POCI) and by FCT “Institute for Research and Innovation in Health Sciences” (POCI-01-0145-FEDER-007274), and “The Role of Exosomes in Tumor Heterogeneity: More than Just Bubbling” (PTDC/BIM-ONC/2754/2014); NORTE-01-0145-FEDER-000029. S.A.Melo is supported by FCT IF/00543/2013. C. Ruivo SFRH/BD/131461/2017, and N. Bastos FCT SFRH/BD/130801/2017.

PF02.20

Highly sensitive detection of IDH1 R132H mutations in plasma of glioma patients

Elena Castellanos-Rizaldos ^1^; Leonora Balaj^2^; Pier Paolo Peruzzi^3^; Dalin Chan^1^; Xuan Zhang^4^; Seth Yu^1^; Johan Skog^5^; Bob Carter^6^

^1^Exosome Diagnostics, Inc., Waltham, MA, USA; ^2^Mass General Hospital/Harvard Medical School, Boston, MA, USA; ^3^Department of Neurosurgery, Brigham and Women’s Hospital, Boston, MA, USA; ^4^Massachusetts General Hospital, Boston, MA, USA; ^5^Exosome Diagnostics Inc., Waltham, MA, USA; ^6^Department of Neurosurgery, Massachusetts General Hospital, Boston, MA, USA

**Background**: Gliomas are the most common primary malignant brain tumours in adults. Recurrent isocitrate dehydrogenase (IDH) gene mutations are found in up to 80% of low grade gliomas and 20% of secondary glioblastomas, identifying tumours with distinct etiology, associated genetic alterations and overall natural history. Detection of the mutation prior to surgery would aid in the management of care, including the extent of surgical resection and treatment regimen which has been shown to correlate with improved survival.

Cell-free DNA is released into biofluids from dying cellular processes and has been used to detect mutations in plasma from cancer patients. However, cfDNA mutation analysis has been challenging for brain cancers. On the other hand, exosomes and other extracellular vesicles are actively released from living processes into plasma, and combining these two sources of nucleic acids can increase sensitivity. The goal of this study was to develop a droplet digital PCR -based assay to detect the R132H mutation from plasma samples of glioma patients using cfDNA and exosomal nucleic acids (exoNA) as input material.

**Methods**: We developed a plasma-based assay that combines exoNA and cell free DNA to detect R132H plasma samples with 1–2 mL of input material using ddPCR. The test was optimized to detect single copies of the mutation and then, its clinical performance was assessed on an interim cohort of 17 clinical samples for which matched tissue data was available as well as two healthy control samples.

**Results**: In this study, we detected R132H in the patient samples analysed with 53% sensitivity and 100% specificity. This result is similar to the sensitivity we previously published using exosomes from cerebrospinal fluid, however plasma is a much more accessible biofluid for diagnostics. In addition, we postulate that the ratios of DNA to RNA mutations may potentially indicate the disease status where a high level of the mutation at the DNA level indicates tumour cell death, while a high level of mutation at the RNA, may indicate tumour growth and progression.

**Summary/Conclusion**: We report a novel platform to capture both exoNA and cfDNA in circulation in plasma to detect rare copies of R132H from patients with low grade gliomas. Combining these two sources of information may enable a more sensitive diagnosis and tracking of the course of the disease.

PF03: EVs and Stem Cells (Including Cancer) Chairs: Alena Ivanova; Marta MonguióLocation: Exhibit Hall 17:15–18:30

PF03.01

Mesoangioblast derived extracellular vesicles have paracrine effects on different cell types

Maria Magdalena Barreca; Fabiana Geraci

Department STEBICEF, University of Palermo, Palermo, Italy

**Background**: Mouse mesoangioblasts are vessel associated progenitor stem cells endowed with the ability of multipotent mesoderm differentiation. We have already demonstrated that these stem cells, as all the other stem cells, are able to release in the extracellular milieu membrane vesicles (EV) containing biological active molecules, such as FGF2, MMP2/9 and Hsp70. Moreover, we have already demonstrated that EV released by mesoangioblasts contains Hsp70 as transmembrane protein that is involved in an autocrine signalling, through transmembrane receptors, responsible for increased cell migration.

Today takes hold the idea that the vesicles can replace stem cells opening a new scenario in regenerative medicine. To this aim, we investigated the possible paracrine interaction of mesoangioblast EV on different cell types and their effects.

**Methods**: Mesoangioblast (A6) EV were collected from conditioned medium by ultracentrifugation. Human Jurkat lymphocytes were cultured with or without A6 EV to investigate their effect on cell activation and proliferation. Jurkat activation was also evaluated after incubation with murine macrophages (Raw 264.7) conditioned medium treated with or without A6 EV. All these analysis were performed by FACS. Enzymatic removing of N-lynked glycans was performed by treating EV with either PNGase F or EndoH.

**Results**: We have analysed the immunomodulatory effect of mesoangioblast EV on human lymphocytes. We have demonstrated that EV is able to inhibit both lymphocyte activation and proliferation. We also started to investigate the mechanisms of interaction between EV and target cells. In particular, we have observed the involvement of EV saccharidic residues in cell targeting. The enzymatic removal of EV saccharidic residues by PNGase F induces a substantial reduction in EV-target cell interaction. Conversely, Endo H increases this interaction.

**Summary/Conclusion**: In conclusion, we demonstrated that mesoangioblast EV interacts with lymphocytes influencing their behaviour. Furthermore, we showed that EV saccharidic residues exert a role in EV-cell interplay.

**Funding**: This research was supported by grants from the University of Palermo.

PF03.02

Establishment of *in vitro* assays to monitor the immune-modulatory capacity of MSC-derived EVs towards cytokine and T cell response for clinical application

Esther Schwich ^1^; Lambros Kordelas^2^; Robin Dittrich^1^; Verena Börger^1^; Peter A. Horn^1^; Bernd Giebel^3^; Vera Rebmann^1^

^1^Institute for Transfusion Medicine, University Hospital Essen, Essen, Germany; ^2^Department of Bone Marrow Transplantation, University Hospital Essen, Essen, Germany; ^3^Institute for Transfusion Medicine, University Hospital Essen, Essen, Germany

**Background**: Graft-versus-host disease (GvHD) is a severe complication after hematopoietic stem cell transplantation, in which donor T-cells attack the patient’s tissue. GvHD is mediated by escalated secretion of pro-inflammatory cytokines such as TNFa and imbalance of effector and regulatory T-cells. Further, CCR7-mediated migration of naïve and regulatory donor T-cells into secondary lymphoid organs is crucial in the pathogenesis of GvHD. Although mesenchymal stem cells and their extracellular vesicles (MSC-EVs) contain immune-modulatory capabilities, the strength of the immune-modulatory effects and thus the efficacy of corresponding clinical products may vary between individual preparations. To warrant a certain quality, it is the aim of our study to establish a functional *in vitro* assay allowing testing for the immune-modulatory capacities of MSC-EV preparations considered as GvHD therapeutics.

**Methods**: Peripheral blood lymphocytes in presence/absence of two different MSC-EV preparations (MSC-EV1 and MSC-EV2) were either stimulated with PMA/Ionomycin for 4 h or with CD3/CD28 for 48 h to monitor cytokine response and T-cell subsets, respectively. GvHD relevant MSC-EV modulations were evaluated by 12-colour (CD45RA, CCR7, CCR4, FOXP3, CD25, CD38, CD39, Ki67, TNFa, IFNg, IL-10 and live/dead) flow cytometric analysis.

**Results**: Upon PMA/Ionomycin stimulation, MSC-EV1 increased the frequencies of IFNg and TNFa secretion of different T-cell subsets, whereas MSC-EV2 decreased the frequencies. Upon CD3/CD28 stimulation, MSC-EV1 decreased the frequency of Ki67- naïve T-cells (CD3+CD45RA+CCR7+) while the frequency of Ki67- effector memory cells (CD3+CD45RA-CCR7-) was increased. Interestingly, the effect of MSC-EV2 was vice versa.

**Summary/Conclusion**: This demonstrates that we are able to determine differences in the immune-modulatory capacity of different MSC-EVs towards T-cell cytokine response and towards composition and activation/regulatory status of T-cell subsets.

**Funding**: This research was funded by European Regional Development Fund 2014-2020 (EFRE) and European Union.

PF03.03

Osteosarcoma derived extracellular vesicles mediated epigenetic alterations in mesenchymal stem cells

Roman Kornilov; Iftekhar Chowdhury; Bettina Mannerström; Riitta Seppänen-Kaijansinkko; Sippy Kaur

Department of Oral and Maxillofacial Diseases, University of Helsinki and Helsinki University Hospital, Helsinki, Finland

**Background**: Osteosarcoma (OS) is the most common primary heterogeneous malignant bone tumour of long bones affecting children and adolescents. While relatively rare, it is still the third-highest cause of cancer-related death in pediatric patients. Cell of origin and tumour driving genetic alterations associated with OS development remains unclear. For last three decades 5 year survival rates for metastatic and recurrent OS is below 20% and has remained unchanged. Novel treatment approaches are urgently needed, thus understanding the cellular origin of OS will have direct implication in improving the treatment approaches through identification of new therapeutic targets.

We examined the epigenetic influence of OS-extracellular vesicles (EVs) on mesenchymal stem cell (MSC) and pre-osteoblast, and the consequences of OS-EVs treatment on the epigenetic reprograming of MSCs.

**Methods**: Three MSC and pre-osteoblast donor lines were treated with EVs extracted from commercially available human osteosarcoma cell line at four different time points (day 0, 3, 7 and 14). To examine the epigenetic influence of OS-EVs on MSCs and pre-osteoblast, LINE-1 methylation analysis and methylation status of 24 tumour suppressor genes were assessed on MSCs using commercially available MS-MLPA kit.

**Results**: Our data indicated that internalized OS-EVs by both MSC and pre-osteoblast mediated a strong epigenetic response. Interestingly treatment with OS-EVs mediated LINE-1 hypomethylation and induced the hypermethylation of TIMP3, only in MSCs whereas opposite effect was seen in pre-osteoblast, which indicated that MSCs but not pre-osteoblast were more susceptible to epigenetic transformation. Thus, OS-EVs dictated the fate of MSC via modulating its epigenetics status.

**Summary/Conclusion**: Overall, this study provided an evidence that epigenetic regulation may appear to be an early event which play a major role in transformation of MSCs.

**Funding**: This work is funded by Helsinki University Hospital and University of Helsinki project funding

PF03.04

MicroRNA-21 over expression in umbilical cord blood hematopoietic stem cells by leukemia microvesicles

Farnaz Razmkhah ^1^; Sedigheh Amini kafi-abad^2^; Sorayya Ghasemi^3^; Masoud Soleimani^4^

^1^Hematology Research Center, Shiraz University of Medical Sciences, Shiraz, Iran; ^2^Department of pathology, Blood Transfusion Research Center, High institute for Research and Education in Transfusion Medicine, Tehran, Iran; ^3^Genetic Department, Faculty of Medicine, Shahrekord University of Medical Sciences, Shahrekord, Iran; ^4^Department of Hematology, Faculty of Medicine, Tarbiat Modares University, Tehran, Iran

**Background**: Microvesicles are able to induce the cell of origin’s phenotype in a target cell. Leukema is known by uncontrolled proliferation of blast cells in the bone marrow. MicroRNA-21, as an oncomir, is up-regulated in almost all cancer types such as leukemia which results in cell proliferation. In this study, we examine the ability of leukemia microvesicles to induce hematopoietic stem cells (HSCs) proliferationvia microRNA-21 dysregulation.

**Methods**: Leukemia microvesicles were isolated from HL-60 and NB-4 cell lines by ultracentrifuge and then their protein was measured by Bradford method. Normal HSCs were isolatedfrom umbilical cord blood samples by CD-34 antibody. These cells were treated with 20 and 40 µg/ml leukemia microvesicles for 5 and 10 days, respectively. Cell count, CD-34 analysis and microRNA-21gene expression assay were done at day 5 and 10.

**Results**: HSCs showed a significant increase in microRNA-21 gene expression and cell count after treatingwith leukemia microvesicles comparing with control groups. CD-34 analysis did not show any difference in studied groups.

**Summary/Conclusion**: This data suggests that HSCs proliferation followed by microRNA-21 gene over expressioncan be another evidence of leukemia like phenotype induction in a healthy target cell by leukemia microvesicles.

PF03.05

The combination of cell and gene therapy as tool to design a new generation therapy based on MSC’s derived exosomes

Marta Gómez; Akaitz Dorronsoro González; Rafael Sánchez; Hernan González-King; Pilar Sepulveda

Instituto de Investigación Sanitaria La Fe., Valencia, Spain

**Background**: Mesenchymal stem cells (MSCs) have been tested in multiple preclinical models obtaining excellent results in tissue regeneration and autoimmune diseases. The therapeutic effect in preclinical models is not due to the differentiation of the cells but to paracrine mechanisms mediated by secreted factors, including exosomes. However, the results obtained in the majority of the clinical trials using MSCs are not as good as expected. Our proposal is to use cutting-edge hypothesis fusing gen and cell therapy to boost the therapeutic properties of MSCs and their exosomes.

**Methods**: Human MSCs were obtained from Inbiobank and cultured in proliferation media (DMEM supplemented with 10% FBS). Exosomes were isolated by ultracentrifugation from exosome harvesting media (DMEM supplemented with 10% exosomes-depleted FBS) conditioned by different MSC lines during 48 h. For isolation of exosomes derived from licensed MSCs, harvesting media was supplemented with TNF-α, IFN-γ and IL-1β. The overexpression of Hypoxia Inducible Factor (HIF) in MSC was done by lentiviral transduction of the cells. The immunosuppressive capacity of different MSC lines and the exosomes derived from them was studied by measuring activated T cell proliferation co-cultured with cells or with exosomes for 5 days.

**Results**: Overexpression of HIF increases immunosuppressive features of MSC. Immunomodulation by MSC is a paracrine process and different authors published that exosomes have immunomodulatory capacity. In previous experiments, we observed that MSC-HIF cells secreted more exosomes than regular MSCs but have been able to show now that those exosomes are not more suppressive than their wild type counterparts are. It is remarkable that even though immunomodulation has to be activated in MSCs by pro-inflamatory molecules, exosomes secreted by none-licensed MSC already showed regulatory features. However, the suppressive capacity of these vesicles is very limited and *in vivo* therapy requires very high doses of exosomes. In this piece of work, we show that licensing MSC increases the immusuppressive capacity of the exosomes dramatically.

**Summary/Conclusion**: Taking all together, we believe that a cell-free therapy strategy based on exosomes derived from MSCs could be a safe treatment for autoimmune and inflammatory diseases

**Funding**: This work was funded by ISCIII [PI16/00107, RD16/0011/0004].

PF03.06

Stem cell-derived exosomes as a biomaterial source for immune modulating therapy

Seulbee Lee ^1^; Hyesun Jung^2^; Insik Hwang^1^; Ah-Young Jang^1^; Kyung-Ah Choi^1^; Hang-Soo Park^1^; Sunghoi Hong^1^

^1^School of Biosystem and Biomedical Science, College of Health Science, Korea University, Seoul, Republic of Korea; ^2^School of Biosystem and Biomedical Science, Department of Public Health Sciences, Korea University, Seoul, Republic of Korea

**Background**: Exosomes, membranous vesicles in the 30~150 nm diameter which secreted by most cell types, are involved in cell-to-cell communications. The vesicles have been reported that they can modulate inflammatory responses in immune cells. Since stem cell derived exosomes has emerged as a new strategy for treating immune disorders accompanying acute inflammatory reactions, we examined their possibility as a biomaterial source for immune modulating therapy using stem cell-derived exosomes.

**Methods**: The immunomodulatory abilities of stem cell-derived conditioned media (SC-CM) were verified by real-time qPCR, western blotting and NO assay. Exosomes from SC-CM were isolated using column chromatographic separation. We analysed the exosomes using dynamic light scattering (DLS), transmission electron microscope (TEM), western blotting (CD9 and CD63 positive extracellular vesicles) and flow cytometry (counting PKH67 labeled vesicles). To determine the anti-inflammatory effects of the NSC derived exosomes, they were incubated with human keratinocyte cell line, HaCaT. Then, proteins within the exosome were identified by tandem mass tagging (TMT) labeling mass spectrophotometry.

**Results**: SC-CM reduced the expression levels of a variety of pro-inflammatory cytokine and chemokine genes and proteins induced by TNFα and IFNγ, and inhibited the phosphorylation of NF-κB and STAT1. Similarly, when the stem cell-derived exosomes were treated to HaCaT cells, they also decreased the pro-inflammatory cytokine and chemokine genes and proteins. We identified several potential factors through proteomics analysis of the exosomes, which may modulate the inflammatory reactions.

**Summary/Conclusion**: Our results show that exosomes can act as potential mediators to inhibit the inflammatory reactions, suggesting that the stem cell-derived exosomes could be used as a new therapeutic biomaterial for the immune-mediated inflammatory diseases, such as autoimmune disorders, cerebrovascular diseases and tumours.

**Funding**: This research was supported by a grant of the Ministry of Health & Welfare, Republic of Korea [HR14C0007] and from the Ministry of Science, ICT and Future Planning [2017M3A9C6026996] of the government of the Republic of Korea.

PF03.07

Leukemia microvesicles induce LSC specific genes over expression in umbilical cord blood hematopoietic stem cells

Farnaz Razmkhah ^1^; Sedigheh Amini kafi-abad^2^; Sorayya Ghasemi^3^; Masoud Soleimani^4^

^1^Hematology Research Center, Shiraz University of Medical Sciences, Shiraz, Iran; ^2^Department of pathology, Blood Transfusion Research Center, High institute for Research and Education in Transfusion Medicine, Tehran, Iran; ^3^Genetic Department, Faculty of Medicine, Shahrekord University of Medical Sciences, Shahrekord, Iran; ^4^Department of Hematology, Faculty of Medicine, Tarbiat Modares University, Tehran, Iran

**Background**: Microvesicles as a new device of cell-cell communication are potentially able to induce some phenotypes and genotypes of an origin cell in a target cell. In the current study, we evaluate the role of leukemia microvesicles in the expression of leulemia stem cells (LSCs) specific genes in healthy hematopoietic stem cells (HSCs).

**Methods**: HL-60 and NB-4 cell lines (acute promyelocytic leukemia cell lines) were selected for microvesicles isolation by ultracentrifugation. Then, the level of microvesicles’ protein was assessed by Bradford method to be used as microvesicle dose. Healthy HSCs were obtained by magnetic association cell sorting (MACS) and CD-34 micro-beads from umbilical cord blood samples and then, were treated with 20 and 40 µg/ml leukemia microvesicles for 5 and 10 days, respectively. LY-86, LRG-1 and PDE9A genes expression as LSC specific genes were analysed by quantitative real time polymerase chain reaction (QRT-PCR).

**Results**: Healthy HSCs showed a significant increase in LY-86, LRG-1 AND PDE9A genes expression after treatment with both 20 and 40 µg/ml HL-60 and NB-4 microvesicles at day 10.

**Summary/Conclusion**: Our results suggest that healthy HSCs can be transformed genetically by leukemia microvesicles to over express LSC specific genes. This may be another evidence of leukemia-like transformation by leukemia microvesicles.

PF03.08

Extracellular vesicles derived from aged mesenchymal stem cells improve the regeneration capacity of mesenchymal stem cells

Xiaoqin Wang; Chrysoula Tsirigoti; Forugh Vazirisani; Peter Thomsen; Karin Ekström

Department of Biomaterials, Institute of Clinical Sciences, Sahlgrenska Academy, University of Gothenburg, Gothenburg, Sweden

**Background**: Mesenchymal stem cells (MSCs) secret extracellular vesicles (EVs) which contribute to the repair of various tissues. Studies have shown that *in vitro* ageing (passage number of cells in culture) altered the characteristics of MSCs including reduced proliferation and differentiation capacities. However, it is not yet known if ageing affects the secretion and the biological effects of MSC-derived EVs.

**Methods**: Conditioned media were collected from 3 days serum free culture of human adipose-derived MSCs at P5 and P6 (low passages, LP), and P15 and P16 (high passages, HP). EVs were isolated by Exospin isolation kit and characterized by western blot and nanoparticle tracking analysis. MSCs were treated with both EVs_LP and EVs_HP with two different doses for 6 days and the proliferation capacity was evaluated by Cell Counting kit 8. Moreover, the effect of EVs on osteogenic differentiation capacity was investigated by ALP assay after 2 weeks of EVs treatment.

**Results**: Both MSC_LP and MSC_HP secreted EVs that were positive for CD63 and Flotillin 1, and negative for Grp94. Particle quantification showed that MSC_HP secreted more EVs than MSC_LP. Both EVs_LP and EVs_HP promoted MSC proliferation in comparison with non-treated group. In the low-dose treatment, EVs_LP and EVs_HP increased the proliferation of MSC_LP to a similar degree. However, in the high-dose treatment EVs_HP stimulated more proliferation of both MSC_LP and MSC_HP than EVs_LP. Furthermore, treatment with EVs_HP enhanced the ALP activity of MSC_LP under osteogenic condition.

**Summary/Conclusion**: Taken together, our preliminary data showed that *in vitro* ageing of MSCs promotes the secretion of EVs. Both EVs_LP and EVs_HP promote proliferation of MSCs in a dose-dependent and origin-associated manner. However, only EVs_HP showed to increase ALP activity of MSC_LP, indicating stimulation of osteogenic differentiation. In conclusion, it is suggested that ageing alters the secretion and the biological effects of EVs derived from MSCs.

**Funding**: This work was funded by Swedish Research Council [K2015-52X-09495-28-4], Handlanden Hjalmar Svensson Foundation, the Felix Neuburg Research Fund, the Adlerbertska Foundation and the Area of Advance Materials of Chalmers and GU Biomaterials within the Strategic Research Area initiative launched by the Swedish Government

PF03.09

Extracellular vesicles from human dental pulp stem cells as proangiogenic strategy in tooth regeneration

Greet Merckx ^1^; Baharak Hosseinkhani^2^; Sören Kuypers^2^; Lore Vanspringel^1^; Joy Irobi^3^; Luc Michiels^2^; Ivo Lambrichts^1^; Annelies Bronckaers^1^

^1^Morphology Research Group, Biomedical Research Institute (BIOMED), Hasselt University, Diepenbeek, Belgium; ^2^Bionanotechnology group, Biomedical Research Institute (BIOMED), Hasselt University, Hasselt, Belgium; ^3^Neurofunctional genomics Group, Biomedical Research Institute (BIOMED), Hasselt University, Diepenbeek, Belgium

**Background**: Tooth loss remains a major health issue since current therapies cannot regenerate damaged dental tissues such as pulp and enamel. Successful pulp regeneration depends on angiogenesis, which is key for oxygen and nutrient supply. Proangiogenic features have already been assigned to mesenchymal stem cells (MSCs) within the dental pulp. So far, paracrine factors, including VEGF, have been identified as responsible angiogenic mediators. However, more recent studies indicate that extracellular vesicles (EVs) produced by bone marrow-derived MSCs (BMMSCs) also have the potential to induce neovascularisation. Therefore, we compared the angiogenic properties of EVs from dental pulp stem cells (DPSCs) with those of BMMSCs.

**Methods**: EVs were isolated from serum-free conditioned medium of DPSCs and BMMSCs after 48 h by differential ultracentrifugation. EV size and concentration were measured by nanoparticle tracking analysis (NTA) and purity was confirmed by western blot with enrichment of classical EV markers CD9, CD63, CD81 and TSG101 and absence of non-EV marker mitochondrial complex V. The functional effect of EVs on the migration of human umbilical vein endothelial cells (HUVECs), as a key step in angiogenesis, was studied in a transwell system.

**Results**: Preliminary data suggest that EVs from DPSCs induce HUVEC migration (*n* = 4). However, this effect was less compared to BMMSC EVs (*n* = 2), which might be caused by the lower EV yield from DPSCs as measured by NTA. Uptake of DPSC EVs by HUVECs was confirmed with confocal microscopy.

**Summary/Conclusion**: Our preliminary data show promising *in vitro* proangiogenic effects of DPSC EVs. In the future, we will compare the angiogenic factors present in DPSC and BMMSC EVs and analyse their potential to induce blood vessel growth *in ovo*. Ultrastructural analysis of both EV types will be performed. Acquired insights have positive implications for pulp regeneration and diseases associated with insufficient angiogenesis, such as stroke.

PF03.10

Stem cell-derived extracellular vesicles as a cell-free therapy for tissue repair/regeneration: a systematic review and meta-analysis of preclinical studies

Pengbin Yin; Yi Li; Yuan Deng; Houchen Lv; Licheng Zhang; Peifu Tang

Chinese PLA General Hospital, Beijing, China (People’s Republic)

**Background**: Encouraging results indicate that stem cells derived EVs could benefit tissue repair, thereby representing a promising alternative for stem cells. Nonetheless, preclinical studies of EVs therapy have yielded inconsistent results. Therefore, we conducted a systemic review and meta-analysis in order to establish the overall effects of stem cells derived EVs in preclinical studies and explore the factors may cause the controversy.

**Methods**: PubMed, Embase, Web of Science and the Cochrane library were systematically searched for studies from inception to October 2017 without any language restriction. The inclusion criteria were published studies investigating the EVs’ effects on tissue repair/regeneration. Studies with randomized controlled groups were included while review, editorial, case report, case series, protocol paper, study without any control group and study with only *in vitro* comparison were excluded. Outcomes for repair/regeneration effects should be reported and compared across intervention groups. Two authors independently screened the retrieved articles in accordance with the predefined inclusion criteria. The overall effects of EVs on repair/regeneration were calculated by pooling estimate from the results obtained.

**Results**: In total, 23 studies were included ultimately. Tissues involved in these studies include heart, liver, kidney, lung, bone and wound. Five studies regarding myocardial infarction repair were included. Pooling estimated result showed that left ventricular ejection fraction in the intervention group significantly as compared to that in the control group (pooled difference: 14.17 95%CI 12.94–15.40, *p* < 0.001). Research into liver repair was conducted in various disease models, therefore not suitable for a meta-analysis. Neither is wound healing for the same reason. At last, regarding kidney, bone, lung, the numbers of included studies are also insufficient for meta-analyses.

**Summary/Conclusion**: Stem cell-derived EVs treatment resulted in significant improvement in the repair of myocardial infarction compared with placebo. For tissue like kidney, liver, lung, bone and wound, further studies with consistent disease model and outcome are required to evaluate the overall effect of EVs.

**Funding**: The research was funded by National Natural Science Foundation of China [81702176].

PF03.11

Pattern of secretion *in vitro* of microvesicles and exosomes in equine mesenchymal stem cells derived from the same animals but from different tissues

Navarrete Felipe^1^; Cabezas Joel^1^; Daniela Rojas^2^; Andrea Navarro^1^; Pedro Pablo Silva^2^; José Manríquez^1^; Fernando Saravia^2^; Lleretny Rodríguez-Alvarez^1^; Fidel Ovidio Castro ^3^

^1^Department of Animal Science, Faculty of Veterinary Sciences, Universidad de Concepcion, Chillan, Chile; ^2^Department of Pathology, Faculty of Veterinary Sciences, Universidad de Concepcion, Chillán, Chile; ^3^Universidad de Concepción, Chillán, Chile

**Background**: Mesenchymal stem cells (MSC) have been postulated as responsible for cell and tissue renewal, they play an important immunomodulatory role and exert their action mainly via paracrine signaling. Microvesicles (MVs) and exosomes (Exo) had been identified as key player in regulation of biological action. Depending on their niche, the regenerative and immunomodulatory properties of MSC may vary as well as their MVs/Exo pattern. Here we compared the MV/Exo pattern of MSC derived from different tissues of the same animals

**Methods**: Six primary cell cultures were derived and expanded from the adipose and endometrial tissue of three different mares. Population doubling time (PDT), colony formation (CF) and surface expression of MSC markers (FACS) were studied. At P2, cells were subjected to differentiation and staining at 0,7,14 and 21 days. Migration experiments using “scratch method” were performed. Each experiment was replicated 3X, controls were included. For MV/Exo analysis, MSC were cultured in DMEM+10% FCS. At 50%, 75% and 90% of confluence, medium was changed and cells were further cultured for 48 h in same medium but depleted of MV/Exo by serial ultracentrifugation. The supernatant was collected and subjected to NTA (Nanosight NS300). Results were analysed according to tissue of origin and confluence using multiple comparison

**Results**: Cells derived from both origins displayed similar PDT, CF, migration and tri-lineage differentiation consistent with a MSCs phenotype. Regarding MVs/Exo analysis, the mean average particle size detected was 119 nm for endometrial MSCs and 118 nm for adipose MSC what matches to exosomes. The kinetics of accumulation of Exo secreted by the MSC varied during confluence, being higher at 75% for both origins, it was significantly higher in adipose MSC (*p* < 0.05). Specific staining with anti CD9 and CD63 unequivocally ascribed the particles as Exo undistinguishably among the origins or confluence status

**Summary/Conclusion**: Mare endometrial cells display main biological properties of adipose MSCs, the MV/EXo secretion pattern was also similar in terms of average particle size and kinetics. At 75% confluence, there is a higher amount of Exo secreted by cells from both origins. The characteristics of this exosomes remain to be tested and deep sequencing is being carried at present

**Funding**: This work was funded by Grant Fondecyt [1150757], Government of Chile

PF03.12 = OWP.1.03

Osteoblast-secreted extracellular vesicles stimulate the expansion of CD34+ human umbilical cord blood cells

PF03.13

Mesenchymal stromal cell derived extracellular vesicles show distinct chondrogenesis microRNA expression profiles from their parental cells

Rachel E. Crossland ^1^; Monica Reis^2^; Matthew J. Barter^3^; Lindsay Nicholson^1^; David A. Young^3^; Anne M. Dickinson^1^; Xiao-nong Wang^1^

^1^Institute of Cellular Medicine, Newcastle University, Newcastle upon Tyne, UK; ^2^Department of Pediatrics, Harvard Medical School, Boston, MA, USA; ^3^Institute of Genetic Medicine, Newcastle University, Newcastle upon Tyne, UK

**Background**: Mesenchymal stromal cells (MSCs) are frequently used in clinical trials for wide-ranging immunological and degenerative diseases. MSC-secreted extracellular vesicles (MSC-EVs) are increasingly reported as the key paracrine factors responsible for MSC clinical benefit, indicating their potential as a cell-free therapy for regenerative medicine. However, the role of MSC-EVs in MSC biology is largely unknown and their molecular composition has not been fully characterized. Here we report the microRNA expression profiles of MSC-EV, including chondrogenesis microRNAs.

**Methods**: Primary BM-MSCs (*n* = 3) identity was determined by phenotypic profiles, morphology and tri-lineage differentiation. MSC-EVS (*n* = 3) were isolated from cell-conditioned medium by differential ultracentrifugation, and characterized by flow cytometry (CD83/CD63/CD9), western blot (Alix&Flotillin), NTA and electron microscopy. Global microRNA expression profiling was performed using NanoString Human MicroRNA V3 (*n* = 799) and selected microRNAs were assessed by qRT-PCR.

**Results**: Comparing matched MSC and MSC-EV samples, 50 microRNAs were differentially expressed (fold change (FC) -49.04–85.93, *p*-value < 0.001–0.049). Of these, 39 were downregulated (FC -1.96–-49.04, p =< 0.001–0.049) and 11 were upregulated (FC 1.71–85.97, *p* = 0.001–0.047) in MSC-EVs. The top 5 highly expressed microRNAs comprised >50% of total expression counts (MSCs = 51.8%; miR-125b = 18.5%, let-7a = 15.0%, let-7b = 8.3%, let-7i = 5.3%, miR-145-5p = 4.7%) (MSC-EVs = 71.3%; miR-4454/7975 = 60.5%, miR-125b = 3.3%, miR-4286 = 3.0%, miR-21-5p = 2.3%, let-7a = 2.2%). qRT-PCR validation in an independent cohort (*n* = 7) confirmed four chondrogenesis microRNAs which were over expressed in MSC-EV vs. MSC (miR-29b *p* = 0.01, miR-142-3p *p* < 0.001, miR-21-5p *p* = 0.004, miR-140 *p* = 0.02), and miR-145-5p which was under-expressed in MSC-EV vs. MSC (*p* = 0.04).

**Summary/Conclusion**: MSC-EV microRNA expression can be successfully profiled using NanoString technology. MSC-EVs show differential expression of specific microRNAs, including chondrogenesis-related microRNAs from parental MSCs, which may contribute to their clinical benefit. This has implications for cell-free therapies for degenerative cartilage diseases, including osteoarthritis.

**Funding**: This work was funded by the EC [FP7-People-2012-ITN] and Arthritis Research UK.

PF03.14

TGF-β1 silencing adipose stem cell-derived exosomes as a new therapeutic strategy for liver fibrosis

Yinpeng Jin ^1^; Hongchao Li^2^; Xi Wang^1^; Qingchun Fu^2^

^1^Shanghai Public Health Clinical Center, Fudan University, Shanghai, China (People’s Republic); ^2^Public health clinic center affiliated to fudan university, Shanghai, China (People’s Republic)

**Background**: At present, exosomes of adipose stem cells were widely applied in scientific and research field, and many studies suggested that the transplantation of exosomes can be used for liver fibrosis.

**Methods**: Separating and purifying the adipose stem cells from human adipose tissue .Detecting the immunophenotype of adipose stem cells by flow cytometry. Adipose-derived stem cells were induced to differentiate into adipocytes and osteocytes using cell inductors. Exosomes was isolated by ultrafiltration method from cell culture medium. Morphology of exosomes was acquired by Nanosight and electron microscope. TGF-β1 gene knockdown exosomes was constructed. CCK8 was used to detect the effect of exosomes and TGF-β1 knockdown exosomes to the proliferation of activated hepatic stellate cells.To acquire the liver fibrosis model by Intraperitoneal injection of carbon tetrachloride and the transplantation of exosomes and TGF-β1 knockdown exosomes was perfomed.Liver tissue slice staining and serologic detection were used to evaluate the improvement of fibrosis in rats.

**Results**: TGF-β1 knockdown exosomes can inhibit the proliferation of activated hepatic stellate cells *in vitro*. Animal experiments showed that the level of liver fibrosis of TGF-β1 knockdown exosomes transplantation rats is significant than other groups. Liver tissue slice staining and serologic detection showed the improvement of fibrosis in rats.

**Summary/Conclusion**: It is difficult to improve the level of liver fibrosis for transplanting adipose stem cells exosomes. However, TGF-β1 gene knockdown exosomes have a significant improvement in the liver fibrosis of rats.

PF03.15

Monocyte-derived extracellular vesicles involve mesenchymal stem/stromal cells into tissue remodeling

Arjen Gebraad; Sippy Kaur; Roman Kornilov; Riitta Seppänen-Kaijansinkko; Bettina Mannerström

Department of Oral and Maxillofacial Diseases, University of Helsinki and Helsinki University Hospital, Helsinki, Finland

**Background**: Monocytes and osteoclasts share precursors and both provide pro-osteogenic signals to mesenchymal stem/stromal cells (MSCs) and osteoblastic progenitors. It is not known whether EVs from these cells play a role in controlling bone remodeling and regeneration.

**Methods**: Human peripheral blood monocytes were activated by lipopolysaccharide or differentiated towards osteoclasts. Osteoclasts resorbed hydroxyapatite coatings when cultured on these surfaces. EVs isolated from the conditioned medium were characterized by transmission electron microscopy and nanoparticle tracking analysis. The presence of EV-specific proteins was confirmed by western blotting. We studied the uptake of the EVs by adipose tissue-derived MSCs (AT-MSCs) using confocal microscopy and flow cytometry. We evaluated gene expression using microarrays and assessed osteogenic differentiation markers in AT-MSCs after culture with the EVs for 18 days.

**Results**: AT-MSCs interacted with all three cell types through internalization of their EVs. Only monocyte-EVs however, affected AT-MSC gene expression. Monocyte-EVs upregulated the expression of various cytokines involved in the chemotaxis of leukocytes. In addition, monocyte-EVs upregulated the expression of matrix metalloproteinases (MMPs) and ICAM1 (CD54) compared to controls. These molecules are markers of bone lining cells, an osteoblast subtype that requires MMPs during the clean-up phase between bone resorption and bone formation.

**Summary/Conclusion**: The signals carried by monocyte-EVs involve MSCs into remodeling of the microenvironment, a crucial step in tissue repair. Currently, we are verifying our results at protein level while we control for possible contaminations of lipopolysaccharide among the monocyte-EVs. In addition, we are assessing the differences in effects of the EVs between AT-MSCs and bone marrow-derived MSCs.

**Funding**: This research was supported by University of Helsinki project funding [WBS490302, WBS73714112], the Jouko Pentikäinen fund from the Finnish Cultural Foundation and Helsinki University Hospital State funding for university-level health research [Y1014SUL05, TYH2016130].

PF03.16

Biological and regenerative properties of extracellular vesicles from mesenchymal stem cells of various origin in cardiovascular regeneration

Ewa K. Zuba-Surma ^1^; Anna Labedz-Maslowska^1^; Guangming Cheng^2^; Katarzyna Kmiotek-Wasylewska^1^; Sylwia Bobis-Wozowicz^1^; Malgorzata Sekula^3^; Magdy Girgis^2^; Elzbieta Karnas^4^; Sylwia Kedracka-Krok^5^; Robert Vincent^2^; Michal Sarna^3^; Zbigniew Madeja^1^; Buddhadeb Dawn^2^

^1^Department of Cell Biology, Faculty of Biochemistry, Biophysics and Biotechnology, Jagiellonian University, Krakow, Poland; ^2^Division of Cardiovascular Diseases, Cardiovascular Research Institute, University of Kansas Medical Center, Kansas City, KS, USA; ^3^Malopolska Centre of Biotechnology, Krakow, Poland; ^4^Laboratory of Stem Cell Biotechnology; Malopolska Centre of Biotechnology; Jagiellonian University; Krakow; Poland; ^5^Malopolska Centre of Biotechnology, Jagiellonian University, Krakow, Poland

**Background**: Growing evidence indicates that mesenchymal stem cell - derived extracellular vesicles (MSC-EVs) play a pivotal role in several organ repairs. However, their role in cardiovascular regeneration was not well studied.

The aim was to examine a detailed bioactive content and functional properties of MSC-EVs of different origin *in vitro* and their regenerative capacity in murine model of acute myocardial infarction (AMI) *in vivo*.

**Methods**: Murine and human MSCs form bone marrow and umbilical cord tissues, respectively, were cultured in different conditions including serum-free media.

MSC-EVs were harvested from conditioned media by sequential centrifugation including ultracentrifugation (100,000 g). MSC-EV morphology and size were examined by AFM, NTA (Nanosight) and DLS (Izon), the antigen presence- by high-sensitivity FC (Apogee M-50) and WB, the mRNAs/miRNAs content- by real-time RT-PCR, the global proteom -by mass spectrometry.

Functional assays in target cardiac and endothelial cells after iPS-EV treatment *in vitro* include: proliferation, migration, differentiation, metabolic activity and cell viability analyses. Immunological properties of MSC-EVs were investigated via blood MNC activation *in vitro*, while regenerative capacity- in murine AMI model *in vivo*.

**Results**: We found MSC-EVs to carry several proteins and mRNA/ microRNA transcripts regulating cardiac and angiogenic differentiation processes. Significant impact of MSC culture conditions on the molecular and functional properties of MSC-EVs was also confirmed in multiple assays *in vitro*. Our data also (1) indicated a great impact of MSC-EVs on proangiogenic capacity of heart endothelial cells *in vitro* and (2) confirmed their regenerative potential *in vivo* by showing improved heart histology, anatomy and function in murine AMI model.

**Summary/Conclusion**: Our data showed that MSC-EVs of different origin represent important carriers transferring bioactive content to mature target cells playing an effective role in heart regeneration *in vivo*.

We conclude that MSC-EVs may represent safe therapeutic tool, alternative or supporting to whole cell-based therapy in cardiovascular repair.

**Funding**: This study was supported by UMO-2013/10/E/NZ3/007500 (NCN) and UMO-2015/16/W/NZ4/00071 (NCN) [grants to EZS]. FBBB JU is a partner of the Leading National Research Center (KNOW) supported by the MSHE.

PF03.17

Regulation of therapeutic compounds in extracellular vesicles by 3D-organizing different physical interactions between mesenchymal stem cells and culture matrices

Sunyoung Jung ^1^; Taehee Kim^2^; Jinseok Kim^1^; Hojae Bae^3^; Oh Young Bang^4^; Jae Min Cha^2^

^1^Center for Bionics of Biomedical Research Institute, Korea Institute of Science and Technology, Seoul, Republic of Korea; ^2^Medical Device Research Center, Research Institute for Future Medicine, Samsung Medical Center, Seoul, Republic of Korea; ^3^KU Convergence Science and Technology Institute, Department of Stem Cell and Regenerative Biology, Konkuk University, Seoul, Republic of Korea; ^4^Department of Neurology, Samsung Medical Center, School of Medicine, Sungkyunkwan University, Seoul, Republic of Korea

**Background**: As lipid-shielded and nano-sized vesicles retaining an equivalent medicinal potency to live mesenchymal stem cells (MSCs), MSC-derived extracellular vesicles (EVs) are in focus as a promising therapeutic strategy in regenerative medicine. However, current MSC culture methods only deliver an arbitrary cocktail of therapeutic molecules to collected EVs. Therefore, as primed for a targeted disease, desired recruitment of the multifaceted therapeutic compounds in EVs should be addressed. In this study, we regulated cytokine inclusions packaging into EVs by 3D-organizing different physical interactions between MSCs and culture matrices.

**Methods**: MSCs were encapsulated in gelatin methacryloyl (GelMA) hydrogel with different mechanical stiffness mimicking brain (~1 kPa), muscle (~15 kPa) and collagenous bone tissues (~100 kPa). 3D-cultured MSCs and collected EVs were comprehensively characterized and analysed by various biological assays for imaging, growth kinetics, qPCR array, NTA, cytokine arrays and western blot. The driven therapeutic efficacies of EVs were evaluated by different culture models of angiogenic, osteogenic and neurogenic stimulation.

**Results**: MSC’s characteristics were influenced by encapsulation conditions with varying matrices’ stiffness. MSCs were likely to show neural-like attributes in lower rigidity of matrices, whereas demonstrating osteogenic characteristics as rigidity increased. EVs collected from each condition contained distinguished cytokine compositions such that larger amounts of angiogenic and neurotrophic factors were found in the softer hydrogel, whereas cytokines related to osteo/chondrogenic stimulation were abundantly presented as rigidity increased.

**Summary/Conclusion**: Our study showed an efficient and scalable method to manipulate EV compositions. To practically employ EVs to clinics, this research could provide the valuable information needed to custom-engineer therapeutic properties of EVs.

**Funding**: This study was supported by the grants from National Research Foundation of Korea (NRF) funded by Ministry of Science, ICT & Future Planning [NRF-2017R1C1B2002624], and Convergence Technology Development Program for Bionic Arm through the NRF funded by the Ministry of Science, ICT & Future Planning [No. 2017M3C1B2085292].

PF03.18

Proteomic characterization and anti-inflammatory effect of primed canine adipose mesenchymal stem cell conditioned medium

Pauline Cajon ^1^; Florence Poirier^2^; Georges Uzan^3^; Didier Lutomski^4^; Philippe Mauduit^3^; Jean-Jacques Lataillade^5^; Tewfik Kadri^1^

^1^StemT, Elancourt, 78990 France, Bobigny, France; ^2^Laboratoire de protéomique, CSPBAT, UFR SMBH Léonard de Vinci, Bobigny, France; ^3^UMRMD5 Inserm/SSA 1197, Institut de Recherche Biomédicale Des Armées, CTSA HIA Percy, Villejuif, France; ^4^Laboratoire de protéomique, CSPBAT, UFR SMBH Léonard de Vinci, Bobigny, Bobigny, France; ^5^UMRMD5 Inserm/SSA 1197, Institut de Recherche Biomédicale Des Armées, CTSA HIA Percy, Clamart, France

**Background**: In the past 15 years, mesenchymal stromal cells (MSCs) have emerged as a therapeutic innovative tool for regeneration of injured and inflamed tissues. In veterinary medicine, those cells are raising an increasing interest. Some years ago, the main action of MSC was described as tissue integration after differentiation. However, paracrine secretion has been proposed as the principal mechanism involved in tissue repair. Many pre-conditioning approaches have been explored in order to modify the secretory pattern of MSC. In the present study, we wanted to define canine adipose mesenchymal stem cell secretome after different priming conditions that would mimic an inflammatory environment. In particular, we wondered whether conditioned medium (CM) would have a beneficial effect on inflammation.

**Methods**: The first step of this investigation was to determine a proteomic profile of the MSC CM, to find the presence of specific cytokines and characterize the population of secreted extracellular vesicles (EV). Proteomic profiling of the MSC secretome was made by electrophoresis coupled with mass spectrometry and were confirmed by ELISA.

Then, to assess the CM effect on inflammation, a canine macrophage cell line DH82 was activated by LPS and treated with concentrated canine adipose MSC-derived CM. The level of TNFα, IL1β, IL10, IL6 and IL8 cytokines were quantified by ELISA.

**Results**: The first results showed that MSC secreted more proteins and EV after different priming conditions.

Moreover, CM down-regulates macrophage secretion of TNFα and IL1β pointing that MSC-derived CM exhibits an anti-inflammatory effect.

**Summary/Conclusion**: These data indicate that CM containing EV delivered by canine adipose MSC could be a good alternative for the treatment of canine inflammatory diseases. Finally, the priming optimization of MSC secretome could potentially lead to optimize the anti-inflammatory effect of CM.

PF04: EVs and the Immune System Chairs: Martin van Herwijnen; Mar Vales-Gomez Location: Exhibit Hall 17:15–18:30

PF04.01 = OWP1.01

Immunomodulatory function of human mesenchymal stromal cells-derived extracellular vesicles on type-I interferon response in human plasmacytoid dendritic cells and lupus murine pDCs

Lin Kui ^1^; Godfrey CF Chan^2^; Pamela PW Lee^3^

^1^Department of Paediatrics and Adolescent Medicine, The University of Hong Kong, Hong Kong, Hong Kong; ^2^Department of Paediatrics & Adolescent Medicine, LKS Faculty of Medicine, The University of Hong Kong, Hong Kong, Hong Kong; ^3^Department of Paediatrics and Adolescent Medicine, LKS Faculty of Medicine, The University of Hong Kong, Hong Kong, Hong Kong

**Background:** Immunoregulatory effect of Mesenchymal stem cell (MSC) is attributed to Extracellular vesicles (EVs) secretion. Given its effectiveness in preclinical studies of autoimmune disease, no one has examined its effect on SLE pathogenesis, signify by excessive type-I IFN production by pDCs and animal models. We found that TSG-6, a key anti-inflammatory protein secreted by activated MSC, downregulates TLR7 and TLR9 activation in human pDC. Herein, we investigate the effect of MSC and MSC-EVs on regulating cytokines production in pDCs, and whether such effect is mediated by TSG-6.

**Methods:** htMSC (immortalized human MSCs), was cultured in CDPF medium for 48 hours. EV were isolated by ultracentrifugation at 100,000g, 3hr, at 4°C and were characterized by Transmission electron microscopy, Nanosight, and western-blot. Comparison of immunosuppressive function between htMSC-EV and TSG-6 knockdown htMSC on TLR9-mediated cytokine production in pDC was determined with GEN2.2, a human pDC cell-line, following activation by CpG-A, and analysis by qPCR and ELISA. Finally, we compared the IFN-α and TNF-α intracellular expression in pDCs of htMSC-EV treated NZB W/F1 mice with PBS control-group.

**Results:** Upon activation of TLR9 by CpG-A, IL-1ß, TNF-α and IFN-α transcription was upregulated in GEN2.2. Such response was reduced when CpG-A-primed GEN2.2 were co-cultured with htMSC. Knockdown of TSG-6 in htMSC dampened its capacity to suppress IL-1ß, TNF-α, IFN-α and IRF7 transcription in GEN2.2. To find out whether MSC exert its immunosuppressive effect by means of EV, we isolated EVs from hTERT MSCs and found htMSC-EV contained TSG-6 protein. Coculture of htMSC-EV with CpG-A-primed GEN2.2 resulted in downregulation of IFN-α transcription and protein expression, mediated via reduction in total and phospho-IRF7. htMSC-EV treatment of NZB/W F1 mice resulted in augmentation of splenic and bone marrow pDCs, with a reduction in bone marrow pDCs TNF-α and splenic pDC IFN-α expression.

**Summary/Conclusion:** For the first time, we showed that MSC downregulated TLR9 activation in human pDCs, in a TSG-6 dependent manner. Furthermore, htMSC-EV contain TSG-6 and suppress IFN-α response in CpG-A-activated pDCs through reducing total and phospho-IRF7. Finally, htMSC-EV treatment modulated pDCs in NZB/W F1 mice.

**Funding:** Edward and Yolanda Wong Foundation.

PF04.02

Correlation of exosomal miRNA- and anthropometric profile of an active lifestyle

Kitti Garai ^1^; Adam Gyebrovszki^2^; Emese Katai^3^; Tamas Nagy^3^; Judit E. Pongracz^1^; Krisztian Kvell^1^; Marta Wilhelm^2^

^1^Institute of Pharmaceutical Biotechnology, Faculty of Pharmacy, University of Pecs, Pecs, Hungary; ^2^Institute of Physical Education and Sport Sciences, Faculty of Science, University of Pecs, Hungary; ^3^Szentagothai Research Center, University of Pecs, Hungary

**Background**: Sedentary lifestyle may contribute to the process of immunosenescence, yet lifestyle-related immune alterations are not well described.

Extracellular vesicles are present in all body fluids in significant quantities. Their amount and molecular profile reflects the physiological condition of the body. Some articles suggest that exosomes may be involved in exercise-mediated adaptation processes. However, the long-term impact of regular physical activity on exosome miRNA profile has not been addressed thoroughly.

**Methods**: Our study aimed to examine the effects of a 6-month long lifestyle intervention program on physiological parameters and exosome miRNA, cytokine and hTREC profile. Physically inactive, but otherwise healthy young individuals were subjected to 1 h training three times a week. Body weight, body mass index, body fat %, skeletal muscle % was calculated using bioimpedance analyser. Exosomal miRNA was isolated from serum samples before lifestyle intervention, after 3 months and 6 months of regular physical activity. Exosome miRNA samples were evaluated with NanoString miRNA platform.

**Results**: Six-month training improved the aerobic capacity of each participating individual, and body composition changes were also measurable. Both systolic and diastolic blood pressures changed throughout the study period. We could detect significant changes in several blood parameters including glucose, insuline, cholesterol and cortisol levels. Improvement in the health status of individuals was also supported by the decrease of C reactive protein (CRP) levels. Exosome miRNA profiling showed a significant number of microRNAs being up and down regulated in response to 6 months of regular physical activity.

**Summary/Conclusion**: Exosome miRNA, cytokine, hTREC profiles and physiological changes show that a longer timeframe is even more effective for strong physical fitness and health.

**Funding**: Scientific research support was provided by PTE-TTK, GINOP-2.3.2-15-2016-00047, PTE AOK KA-2016-16, PTE Pharmaceutical Talent Center program and the PTE Viral Pathogenesis Talent Center program via KK. The Janos Bolyai Scholarship of the Hungarian Academy of Sciences also supported KK.

PF04.03

LL-37 impacts extracellular microvesicles production by murine dendritic cells

Adriana Hanai Cieslinski. Tavares^1^; Brenda Louyse Olimpia Souza Teixeira^2^; Lara R. Quadrado^2^; Aldo Tavares^1^; Anamelia Bocca^1^; Felipe Saldanha-araujo^1^; Octavio Franco^2^; Rinaldo W. Pereira ^2^

^1^University of Brasilia, Brasilia, Brazil; ^2^Catholic University of Brasilia, Brasilia, Brazil

**Background**: Extracellular vesicles (EV) can serve as carries of cellular information. EVs derived from dendritic cells (DC) have been shown to target other immune cells and modulate their function. EVs production by DC is induced by a diverse array of signals including cytokines, LPS, and antigens but the role of antimicrobial peptides, such as the human cathelicidin LL37, in this process is largely unknown. In this context, we investigate whether LL-37 induces and alters DC-derived EVs profile.

**Methods**: Murine bone marrow-derived DCs were stimulated with LPS (as a positive control) and different concentrations of LL-37. EVs were obtained from cultured cell supernatants and purified by ultracentrifugation. Particle size distribution and concentration of EVs was measured by tunable resistive pulse sensing, and transmission electron microscopy was performed to characterize their morphology.

**Results**: Our preliminary results show that LL-37 increases the concentration of and decreases the average size of EVs when compared with LPS. EV morphology from our samples was in accordance with the literature.

**Summary/Conclusion**: The next ongoing step is the investigation about the role of LL37 induced EVs in the immunomodulation well described to be carried out by cathelicidin.

**Funding**: This work was funded by Fundação de Apoio à Pesquisa do Distrito Federal, CNPq, CAPES and Universidade Católica de Brasilia.

PF04.04

Immunoproteomic characterization of outer membrane vesicles from high-producing actinobacillus pleuropneumoniae

Fabio Antenucci ^1^; Zofia Magnowska^2^; Manfred Nimtz^3^; Camille Roesch^4^; Lothar Jänsch^3^; Anders Miki Bojesen^2^

^1^University of Copenhagen, København S, Denmark; ^2^University of Copenhagen, Copenhagen, Denmark; ^3^Helmholtz Centre for Infection Research, Braunschweig, Germany; ^4^Izon Science Ltd, Lyon, France

**Background**: Outer membrane veiscles (OMVs) are produced by the majority of Gram-negative bacteria. Thanks to the antigenic similarity between OMVs and the bacterial outer membrane, OMVs have proven to be promising for the development of novel vaccines against bacterial pathogens. In this work we describe the immunoproteomic characterization of OMVs from *Actinobacillus pleuropneumoniae* (App), a Gram-negative pathogen of great veterinary interest, in the context of vaccine development.

**Methods**: OMVs were isolated from App MIDG2331 serotype eight wild type and an isogenic ΔnlpI mutant using a modified version of the hydrostatic filtration protocol described by Musante et al.. OMVs proteins were purified by Wessel-Flüge extraction and resolved by 2D PAGE. Protein staining and 2D western blotting were then used to identify relevant protein spots, which were excised and subjected to protein identification by MALDI peptide mapping.

**Results**: Our analysis led to the identification of several virulence factors in App OMVs, including all three Apx toxins produced by App MIDG2331 (Apx II, III and IV) and proteins involved in nutrient acquisition. Some of the proteins were also shown for the first time to be highly immune-reactive.

**Summary/Conclusion**: Our data suggest that OMVs may play a central role in App pathogenicity and that they represent promising immunogens, due to the presence of several highly immunogenic determinants in the OMVs. The identification of Apx toxins and factors involved in nutrient acquisition support the hypothesis that App may use OMVs to satisfy its nutritional requirements and at the same time hamper the host immune response, thanks to the ability of Apx toxins to target lymphocytes.

**Funding**: This work was funded by Center for research in pig production and health (CPH PIG), University of Copenhagen Research Center for Control of Antimicrobial Resistance (UC-CARE) and SEGES Pig Research Center.

PF04.05

miRNA profiling of circulating EVs in Myalgic Encephalomyelitis/Chronic Fatigue Syndrome (ME/CFS)

Eloy Almenar-Pérez ^1^; Lubov Nathanson^2^; Teresa Sánchez-Fito^1^; Leonor Sarria^2^; Germán Cerdá-Olmedo^1^; Elisa Oltra^1^

^1^School of Medicine, Catholic University of Valencia, Valencia, Spain; ^2^Kiran C Patel College of Osteopathic Medicine, Nova Southeastern University, Ft Lauderdale, FL, USA

**Background**: ME/CFS (ICD-10; G93.3) is a complex multisystem disease of unknown origin with characteristic clinical features that include post-exertional malaise, cognitive dysfunction, orthostatic intolerance, on-going flu-like symptoms and unrefreshing sleep in conjunction with other. Its worldwide prevalence is 0.4%–1% with a female to male ratio of 6:1. Current treatments rely on the management of symptoms due to a lack of understanding of the underlying mechanisms of disease onset and progression. The aim of this work was to identify biomarkers of ME/CFS by analysing miRNA profiles of patient plasma EVs and comparing them to those of their PBMCs. This information should improve our knowledge of ME/CFS and allow the development of unbiased quantitative diagnostic methods.

**Methods**: miRNA profiles of PBMCs or EVs isolated from plasma (Invitrogen cat.4484450) of ME/CFS patients and population, sex, age and BMI-matched healthy participants (N = 15 per group) from the ME UK Biobank (London, UK) were determined using Nanostring technology (nCounter Human v3 miRNA Expression Assay Kit). Gene ontology (GO) and the Kyoto encyclopedia of genes and genomes (KEGG) were used to determine disrupted cellular functions in ME/CFS. The study was approved by the DGSP-CSISP CEIC (ref. UCV201701), Spain. Signed informed consent was required for inclusion of samples.

**Results**: miRNA profiles evidenced a global trend for miRNA downregulation in patients with respect to healthy controls (76% and 64% of the miRNAs presented inhibition, by at least 50%, in PBMCs and EVs respectively; while only one miRNA in PBMCs and 6% of them in EVs showed upregulation to this level). Qualitatively, miRNA profiles in PBMCs did not match those obtained from EVs indicating active packaging of miRNAs in EVs. The functions to be affected by the deregulated miRNAs support a model of immune, mitochondrial and neural defects for this disorder.

**Summary/Conclusion**: This is the first report of paired PBMCs and EV miRNA profiles of ME/CFS patients by enzyme-free array technology. The results confirm previous proposals that this epigenetic mechanism is linked to the pathophysiology of ME/CFS. Validation studies with expanded cohorts are needed before particular miRNA profiles can be used as biomarkers of ME/CFS in a clinical setting.

**Funding**: The study was funded by the ME Association’s Ramsay Research Fund (RRF) (UK).

PF04.06

Characterization of human plasma extracellular vesicles and their role in aging-related immunosenescence and immune response

Ainhoa Alberro ^1^; Matías Sáenz-Cuesta^2^; Lucía Sepúlveda^2^; Iñaki Osorio-Querejeta^1^; Leire Iparraguirre^1^; Irantzu Llarena^3^; Itziar Vergara^2^; Adolfo López de Munain^4^; David Otaegui^1^

^1^Multiple Sclerosis Unit, Biodonostia Health Institute, Paseo Doctor Beguiristain S/N, Donostia - San Sebastián, Spain; ^2^Biodonostia Health Research Institute, Donostia - San Sebastián, Spain; ^3^CIC biomaGUNE, Donostia - San Sebastián, Spain; ^4^Donostia University Hospital and Biodonostia Health Research Institute, Donostia - San Sebastián, Spain

**Background**: Aging is a universal and heterogeneous process that leads to reduced adaptation and increased vulnerability. Cellular senescence is one of its hallmarks and results in major changes in gene expression, chronic inflammation and altered intercellular communication.

The age-associated dysfunction and accumulation of senescent cells of the immune system is called immunosenescence. A decrease of naïve and increase of terminally differentiated T cells has been reported - loss of the co-stimulatory CD28 and a partially compensatory expression of CD56 or CD57. Regarding intercellular communication, inflammaging has been broadly studied, while the role of extracellular vesicles (EVs) remains unclear.

We previously reported that the total concentration of plasma EVs does not increase with age. In the present work, we investigated the senescent features of EVs and their role in T cell activation.

**Methods**: All participants (21–104 years) gave informed consent and the study was approved by the pertinent ethics committee. PBMCs were isolated with Ficoll-Hypaque method and EVs as described before by our group.

Flow cytometry was applied for T cell characterization and to assess activation (FACSCantoII), as well as for EV characterization (CytoFLEX).

**Results**: Senescent T cells (CD28-/CD56- and CD28-/CD56+) gradually accumulate with age (21–89), and CD8 cells are more affected than CD4 cells. Non-agenarians and centenarians do not show an increased senescence of CD8 cells, while it is reduced in CD4 cells when compared to octogenarians. T cell markers, CD3/CD4/CD8/CD28/CD56, are present in plasma EVs, but no accumulation of EVs with senescent features was reported with age.

Co-culture of PBMCs and non-autologous plasma EVs of different age ranges do not induce T cell activation. In contrast, in the presence of PHA, EVs boost activation of PBMCs from adults and non-agenarians, but not in octogenarians and centenarians.

**Summary/Conclusion**: EVs secreted by T cells in plasma are detected by flow cytometry, but they do not resemble the senescence phenotype of cells, at least in their membrane. Besides, non-autologous EVs of different donors can promote T cell activation and this process is influenced by the age of receptor cells.

**Funding**: The Dept. of Education of the Basque Govt. support AA, IOQ and LI

PF04.07

Cdc42 mediates CpG DNA-increased cellular uptake of extracellular vesicles

Ying Zhang; Hang Hubert Yin

School of Pharmaceutical Sciences, Tsinghua University, Beijing, China (People’s Republic)

**Background**: Toll-like receptors (TLR) are activated upon the microbial infection and play important roles in innate immunity. TLR9 is one of the TLR family members that locates inside cells and specifically responds to CpG DNA from bacteria. Extracellular vesicles (EV) have been suggested to serve as a delivery system that carries proteins, nucleic acids and lipids, which is critical for cell-cell communication in the immune system. In particular, EV have been implicated as a transporter for immune potentiators to access the intracellular receptor; however, the role of EV in the TLR9-regulated immunity has not been characterized yet. In this study, we aimed to investigate the effect of CpG DNA on the composition, function and transfer of EV and the underlying mechanism.

**Methods**: The protein composition of EV was investigated by proteomics and western blot analyses. Enzyme-linked immunosorbent assay was used to detect the level of cytokines such as TNF-a. To study the transfer of EV, we utilized a Cre/LoxP cell system in which EV exchange induces a specific color switch in reporter-expressing cells. Moreover, we used siRNA to knock down the level of protein such as Cdc42 in receptor cells and observed the internalization of EV in the target cells by immunofluorescence staining.

**Results**: We showed that CpG DNA increased the transfer of EV between immune cells, as well as modulated the protein composition. In addition, comparing to vehicles, EV isolated from CpG DNA-stimulated cells induced an elevated level of TNF-a. Moreover, the level of Cdc42 protein was increased in EV and the receptor cells in presence of CpG DNA. In cells which Cdc42 was knocked down, the uptake of CpG DNA-stimulated EV was markedly reduced.

**Summary/Conclusion**: We elucidated a novel mechanism which is important for the internalization of EV in the context of TLR9 activation. Our findings may provide insight into the development of novel therapeutic strategies for diseases by modulating the uptake of EV.

PF04.08

Biologically counteractive extracellular vesicles are produced by neutrophilic granulocytes under different conditions

Ferenc Kolonics ^1^; Ákos Lőrincz^1^; Veronika Farkas^2^; Ádám Farkas^2^; Erzsébet Ligeti^1^

^1^Department of Physiology, Semmelweis University, Budapest, Hungary; ^2^Department of Medical Biochemistry, Semmelweis University, Budapest, Hungary

**Background**: Extracellular vesicles (EVs) are known for their capability of transferring biologically active molecules from their cell of origin. Our previous results show that neutrophilic granulocytes (polymorphonuclear neutrophils, PMN) can release EVs with or without antibacterial properties depending on their activation state. Several groups reported both pro- and anti-inflammatory effects of PMN-derived EVs produced upon different stimuli. In this study, we investigated under comparative conditions the thrombo- and immunomodulatory effects of three different well-characterized PMN-derived EV populations.

**Methods**: Human PMN were stimulated with opsonized particles or left non-activated for 20 min. Other PMN were incubated in unstimulated conditions for 24 h. Cells were eliminated and the medium-sized EV fraction was pelleted via differential centrifugation and filtration. EVs derived from these three different conditions (from activated cells – aEV, spontaneously produced cells – sEV, from apoptotic cells – apoEV) were co-incubated with PMN, monocytes, lymphocytes or pooled human plasma. We evaluated the uptake of the vesicles and their effect on phagocytosis, cell migration, superoxide production and coagulation.

**Results**: Both sEVs and aEVs were taken up by all three investigated cell types. Neither the kinetics nor the maximal capacity of PMN phagocytosis was affected by the EVs. aEVs seem to slightly enhance the migratory potential of PMN as opposed to sEVs. Superoxide production of PMN was enhanced by aEVs and decreased by sEVs. apoEVs showed a strong procoagulant effect in recalcified plasma both in the presence and absence of thromboplastin (TP), while sEVs only enhanced coagulation in the absence of TP and aEVs did not have any effect on coagulation.

**Summary/Conclusion**: Our data show that human PMN release different EV populations, which have selective and specific – occasionally even opposing – effects on various physiological processes depending on the conditions during their release from the cells.

**Funding**: This work was funded by NKFIH K119236, VEKOP-2.3.2-16-2016-00002, Hungary.

PF04.09

Extracellular vesicles mediate innate immune activation in hepatitis virus infections

Stephanie Jung; Ulrike Protzer

Helmholtz Center Munich, Munich, Germany

**Background**: Hepatitis B virus (HBV) infection is a major health problem with 257 million chronical carriers worldwide, who are at high risk to develop cirrhosis or liver cancer. Therapeutic options exist but only prevent disease progression; they do not lead to viral clearance. Coinfection of HBV patients with hepatitis D virus (HDV), which is a defective virus requiring HBV surface proteins for productive virus release and propagation, can cause fulminant hepatitis with massive liver damage and high mortality rates.

The innate immune response is mediated by a subset of evolutionary inherited pattern recognition receptors (PRRs) which recognize characteristic pathogen-associated molecular patterns (PAMPs) such as viral nucleic acids, leading to cytokine production. However, as a virus that is well adapted to the human species, HBV has extensive immune evasive mechanisms. HDV as a sattelite virus profits from HBV-mediated immunosuppression. Consequently, neither an HBV nor an HDV detecting PRR has been identified so far.

**Methods**: We hypothesize that exosomes released by HBV-/HDV-infected cells shuttle virus-derived RNAs or specific non-coding RNAs to non-infected immune cells, which act to shape the immune response. Appropriate systems for the purification of hepatitis infection-derived extracellular vesicles (EVs) as well as for specific immunostimulations were established.

**Results**: Notably, HDV infection of hepatoma cell lines leads to the release of immunostimulatory EVs specifically leading to the upregulation of proinflammatory cytokine release from human macrophages. Next-generation sequencing of exosomal RNA content will lead to the identification of the recognized PAMP and subsequent identification of their corresponding PRR.

Infection with ultraviolet-inactivated HBV likewise enhances immunostimulatory potential of EVs, whereas HBV protein expression depending on active viral genomes seems to inhibit cytokine expression. Immunorecognition of inactive HBV components will allow to dissect HBV life cycle for potential interactions with the innate immune system.

**Summary/Conclusion**: Resulting data shall elucidate not only fundamental pathways of innate immunity but clarify the role of extracellular vesicles during hepatitis virus infections.

**Funding**: SJ was funded by the Helmholtz Association’s Initiative and Networking Fund.

PF04.10

Exploring the role of extracellular vesicles (EVs) in immune response

Simone Ritz; Maria Meira; Nadège Lagarde; Claudia Sievers; Tobias Derfuss; Raija Lindberg

Department of Neurology and Biomedicine, University of Basel, University Hospital Basel, Basel, Switzerland

**Background**: Extracellular vesicles (EVs) play an important role in intercellular communication in physiological (e.g. communication in brain, regulation of immune responses) and in pathological conditions (e.g. cancer, autoimmune diseases). Nearly all cell types, including immune cells, produce exosomes, microparticles and apoptotic bodies, collectively termed EVs.

Our goal is to study the functional importance of exosomes in the immunopathogenesis of multiple sclerosis (MS). We specifically aim at characterizing serum-derived exosomes from patients with MS and healthy volunteers (HV) and studying their effects on various immune cells.

**Methods**: Exosomes were isolated from platelet-free serum of HV and MS patients with various disease courses by iodixanol gradient centrifugation (OptiPrep) followed by size-exclusion chromatography (SEC). Nanoparticle Tracking Analysis was used for enumeration and size determination. Peripheral blood mononuclear cells were isolated by Ficoll density gradient centrifugation. Immune cells were separated by MACS technology and stimulated *in vitro*. Exosomes were added and their interaction with immune cells was determined by ImageStream X. Expression of activation markers was analysed by flow cytometry (Attune NxT). Total RNA was extracted from immune cells, and transcriptional expression was analysed using real-time RT-PCR-based assays.

**Results**: OptiPrep gradient centrifugation, followed by SEC, resulted in a homogenous exosome population. Levels of exosomes in sera from relapsing-remitting (RR) MS patients were significantly higher than in those from HV. Analysis of the interaction between exosomes and immune cells revealed a strong association of exosomes with monocytes, followed by CD4+ T, CD8+ T and B cells. Moreover, application of exosomes impacted on the activation and transcriptional regulation of primary immune cells *in vitro*.

**Summary/Conclusion**: Increased levels of exosomes in RRMS patients suggest their potential role in the immunopathogenesis of MS. However, further experiments are needed to confirm the functional importance of exosomes in immune regulation of MS. Characterization of exosomes from various disease courses of MS and evaluation of the effects of current treatments will be performed.

**Funding**: This work was funded by Swiss MS Society, Swiss National Science Foundation.

PF04.11

Analysing leukemia-derived extracellular vesicle modulation of immune activity in lymphocytes

Alejandro Pando ^1^; John Reagan^2^; Patrycja Dubielecka-Szczerba^1^; Loren Fast^1^

^1^Division of Hematology/Oncology, Rhode Island Hospital, Warren Alpert School of Medicine, Brown University, Providence, RI, USA; ^2^Hematology at Lifespan Cancer Institute/Medicine, Brown University, USA

**Background**: In patients with haematologic malignancies, the microenvironment created by cancer cells contributes to immune response inhibition. Extracellular vesicles are heterogeneous membrane particles involved in the exchange of a broad amount of bioactive particles between various cellular populations and have emerged as important intercellular communicators. Cancer-derived extracellular vesicles (CEVs) play a major role in cancer cell communication with their surroundings and recent findings point to their role in inhibition of anti-leukemic immune responses. The detailed mechanisms by which CEVs play their immunomodulatory role are unknown. To better understand the effects of CEVs on immune cells, we examined the effect of extracellular vesicles (EVs) derived from the acute myeloid leukemia (AML) cell line, MOLM-14, on normal donor T cells.

**Methods**: T cell subsets CD4+, CD8+ and CD4+CD39+ Tregs were isolated using Miltenyi isolation kits from the peripheral blood of healthy donors. Thymidine incorporation assays were performed 5 days after co-incubation of T cells with EVs or T cells with phosphate-buffered saline (PBS). EV-exposed T cell and non-EV-exposed T cell cytotoxicity of leukemia cells was measured via chromium release assays.

**Results**: T cells incubated with AML-EVs demonstrated an increase in proliferation but did not translate into increased cytotoxic killing of leukemia cells. T cells incubated with AML-EV resulted in underrepresentation of activation markers (CD69) on CD4+ and CD8+ T cells. We are currently investigating the changes in gene expression that occur in T cell subsets when incubated with AML cell line-derived EVs, syngeneic plasma-derived EVs and PBS.

**Summary/Conclusion**: Our results suggest that AML-EV alter T cell proliferative responses leading to an aberrant response. We are currently investigating the gene expression altered by these EVs.

PF04.12

A mixed lymphocyte reaction as a functional assay for extracellular vesicles of different origins

Michel Bremer; Verena Börger; Peter A. Horn; Bernd Giebel

Institute for Transfusion Medicine, University Hospital Essen, University of Duisburg-Essen, Essen, Germany

**Background**: Extracellular vesicles (EVs), such as exosomes and microvesicles, are shed by all cell types and found in all body fluids. EVs transmit specific information from their cells of origin to specific target cells and are key factors in a novel form of intercellular communication. Depending on their origin, EVs can modulate immune responses and either act pro-inflammatory (e.g. mature dentric cells-EVs) or anti-inflammatory (e.g. mesenchymal stem cell (MSC) and many tumour cell-derived EVs). Aiming to analyse immune-modulating properties of EVs from different sources, *in vitro*, we established a novel form of a mixed lymphocyte reaction (MLR) assay.

**Methods**: Here, human peripheral blood-derived mononuclear cells (MNCs) were pooled from up to 12 different healthy donors warranting high cross-reactivity, even following an optimized freezing and thawing procedure. After thawing, mixed MNCs are cultured for 5 days in the absence or presence of EVs. Thereafter, cell morphologies are documented and cells are phenotypically characterized by flow cytometry. By analysing the expression of a collection of different lineage and activation markers, we selected a panel of antigens apparently being regulated by therapeutically active MSC-EVs.

**Results**: For example we observed that in the presence of active MSC-EVs, more CD14+ (monocytes) and CD56+ (natural killer cells) are recovered from the MLR than in corresponding control samples. In contrast, in the presence of active MSC-EVs, contents of CD4+ and CD8+ T cells got slightly decreased. Focusing on T cells, we learned that active MSC-EVs reduced the content of CD4 and CD8 T cells expressing T cell activation markers like CD54 and CD25.

**Summary/Conclusion**: Currently, we compare the immunomodulatory capabilities of EVs of different cell types. Furthermore, we proceed in optimizing the marker panel to distinguish immune cell subtypes such as the different types of CD4+ cell types (TH1, TH2, TH17 and TRegs).

**Funding**: This research was funded by European Regional Development Fund 2014–2020 (EFRE) and European Union.

PF04.13

Natural killer (NK) cells and NK-exosomes in chronic bacterial lung infection

Neha D. Patil; Maud Thérésine; Olivia Domingues; Jacques Zimmer

Innate Cellular Immunity and Chronic Inflammation, Department of Infection and Immunity, Luxembourg Institute of Health, Esch-Sur-Alzette, Luxembourg

**Background**: Bare lymphocyte syndrome (BLS), a genetic disorder that affects the human leukocyte antigen (HLA) gene complex, causes partial loss of the major histocompatibility complex (MHC). Categorized on the basis of the phenotypic HLA class I expression levels, BLS type I is often associated with the mutation/deletion in the transporter associated with antigen processing (TAP) molecule. Patients suffering from TAP deficiency exhibit recurring bacterial infections of the upper respiratory tract, and baseline natural killer (NK) cells that lack cytotoxicity towards class MHC class I deficient targets. These non-functional NK cells might contribute to the impaired antibacterial defence.

**Methods**: To study the mechanism, we will conduct experiments on wild-type (wt) and TAP-KO mice with C57BL/6 background along with the human samples obtained from TAP-deficient patients. To establish a mouse model with chronic lung infection, the bacterial species *Pseudomonas aeruginosa* will be introduced through intra-tracheal intubation. Mucosal-associated invariant T (MAIT) cells are another cell type implicated in antibacterial defence. NK cells and MAIT cells will be studied in various functional assays and for deriving exosomes, which will be used to understand intercellular communication.

**Results**: It has been reported that exosomes derived from normal NK cells are cytotoxic to various cells and we would like to study this phenomenon with regards to TAP-KO NK cells. Our focus is on their release from and internalization in the cells and the cargo. We have been successful in isolating exosomes from NK cells by differential centrifugation and analysing them by ImageStreamX. We have also established the cytotoxicity of wt mouse-derived NK-exosomes using cytotoxicity assays.

**Summary/Conclusion**: Further functional assays and genomic and proteomic analysis of both types of exosomes are necessary to recognize their role in TAP deficiency and to fully understand the mechanism of action of NK cells and their communication with MAIT cells with regards to antibacterial defence.

**Funding**: This work was funded by Luxembourg National Research Fund (FNR), Luxembourg.

PF04.14

Inflammatory molecules are cell-to-cell transported by exosomes in the anti-inflammatory human autologous conditioned serum

Maria Weisshaar ^1^; Jamal Ghanam^1^; Stephan Irsen^2^; Julio Reinecke^3^; Peter Wehling^3^

^1^Bonn-Rhein-Sieg University of Applied Sciences, Rheinbach, Germany; ^2^Caesar Institute, Bonn, Germany; ^3^Orthogen AG, Duesseldorf, Germany

**Background**: Local injection of autologous conditioned serum (ACS) is a well-known therapy for inflammatory diseases (IDs). While patients’ blood is incubated to generate ACS (with subsequent centrifugation), immune cells produce high amounts of growth factors and cytokines. This include, amongst others, interleukin-1 receptor antagonist (IL-1ra), interleukins 6 and 10, tumour necrosis factor alpha (TNF-α) and transforming growth factor beta 1 (TGF-β1). The aim of this study was to analyse exosomes release into ACS as well as their cytokine cargo.

**Methods**: Whole blood was left at 37°C for 3, 6, 9 and 24 h in a specialized CE marked medical device to obtain ACS. Polyethylene glycol precipitation method was used to isolate exosomes from ACS. The characteristics of exosomes were determined using transmission electron microscopy (TEM). Exosomes’ protein pattern was determined by sodium dodecyl sulfate polyacrylamide gel electrophoresis (SDS-PAGE) and Western blot. ELISA was used to quantify IL-10, IL-1ra, IL-6 and TNF-α carried by isolated exosomes.

**Results**: SDS-PAGE analysis reveals the presence of time-dependent intensity bands (regarding ASC incubation time) in the range of 25 and 58 KDa, corresponding to the main markers of exosomes, CD9 and CD63 (CD81). TEM analysis shows that the 2S3 ACS-fraction (6 h at 37°C) contains the highest amount of exosomes (8.77 × 10^7^ exosome/mL), with a diameter range of 25–58 nm. Western blot results confirmed the presence of the CD63 and HSP70 exosomes markers with the highest intensity bands in the 2S3 fraction. Exosomes’ cargo of IL-1ra and IL-6 increases over time (up to 24 h) to a value of 1626.5 ± 377.1 and 105.2 ± 13.7 pg mL^−1^, respectively, while the exosomal content of TNF-α decreases in time from a value of 92.1 pg.mL-1 (t0) to 0.4 pg mL^−1^ (t24). However, the concentration of IL-10 reaches a maximum (95.8 ± 26.2 pg mL^−1^) in the 2S3 fraction before decreasing by time.

**Summary/Conclusion**: Incubation time affects exosomes release into ACS and their protein cargo. Among the quantified cytokines in ACS-derived exosomes, the anti-inflammatory IL-1ra was the most abundant molecule. This suggests a plausible role of these nanoparticles as anti-inflammatory agents during IDs. However, more researches are required to shed light on the involvement of ACS’ exosomes in inflammation management during IDs.

PF04.15

Characterization of the immune potential of human islet extracellular vesicles

Alissa K. Rutman; Sarita Negi; Marco Gasparrini; Craig Hasilo; Jean Tchervenkov; Steven Paraskevas

Research Institute, McGill University Health Centre, Montreal, Canada

**Background**: The cellular events involved in the generation of an autoreactive immune response in type 1 diabetes (T1D) are not well understood. In both physiological and pathological conditions, cells release a variety of signals, including extracellular vesicles (EV). These nanosized membrane vesicles are known to present antigen in other inflammatory conditions. Previous work in our laboratory has identified that human islets produce EV containing islet autoantigens. This raises the question of whether human islet EV are capable of eliciting an immune response similar to that which causes T1D.

**Methods**: Human islets were isolated from multiorgan donor pancreases. Islets were cultured for 24 h; islet-conditioned media (ICM) was collected and analysed by nanoparticle tracking analysis, electron microscopy and/or flow cytometry. EV were purified from ICM by sequential centrifugation. Peripheral blood mononuclear cells (PBMC) were isolated from healthy volunteers and diabetic patients (DP) by Ficoll. Purified EV were labelled and co-cultured with PBMC. EV internalization, cytokine production, proliferation, memory B and T cell activation were analysed by flow cytometry and/or ImageStream. GAD65 antibody ELISAs were run on EV-PBMC culture supernatants. Analysis of variance or paired *t*-tests were used to compare controls and EV-exposed samples.

**Results**: We demonstrate that the majority of EV are 100–300 nm in size. EV are selectively internalized by monocytes and B cells in a time-dependent manner. EV stimulation triggers an increase in pro-inflammatory cytokine (IL-6, TNFα, IFNγ) expression. Additionally, EV exposure leads to an augmentation in CD4, CD8 and CD19 cell proliferation. Interestingly, EV induce differential activation of memory B cells (CD19+IgD-CD27+CD69 expression) and T cells (CD4+CD62L-CCR7-CD45RO+CD69 expression) in DP as compared to nanovesicle as well as GAD65 antibody production, suggesting antigen-specific stimulation. *In vitro* Ibrutinib treatment dampens EV-induced memory B cell activation.

**Summary/Conclusion**: Islet EV are immunogenic, trigger immune cell activation and autoantibody production. Advances in this field may help to better characterize the mechanisms by which islets and immune cells cross talk to generate islet specific responses. This will help identify targets to be blocked to inhibit EV-mediated immunity and T1D pathogenesis.

PF04.16

HuR-driven extracellular export of miRNA in mammalian hepatic and immune cells

Suvendra N. Bhattacharyya; Kamalika Mukherjee

Molecular Genetics Division, CSIR-Indian Institute of Chemical Biology, Kolkata, India

**Background**: miRNAs, the 22 nucleotide long non-coding RNAs, form miRNP complexes with Argonaute proteins and regulate gene expression by imperfect base pairing to the 3′UTR of target messages. Human ELAV protein HuR is an RNA-binding protein which has strong affinity for AU-rich elements in the 3′UTR of target mRNAs. It binds with target mRNAs by replacing the miRNPs, stabilizes the mRNAs and facilitates their translation. Therefore, HuR is a negative regulator of miRNA function as it relieves the mRNAs from miRNA-mediated repression

Exosomes are extracellular vesicles (EVs) of 30–90 nm and are secreted by a wide range of cells and contain proteins, RNAs and miRNAs. They help in cell-to-cell communication, by transporting various proteins, mRNAs and miRNAs.

**Methods**: We use human hepatoma cell Huh7 and murine macrophage cell RAW264.7 to test the EV-mediated export of miRNAs. We have observed a reduction in cellular miR-122 content in amino acid-starved human hepatic cells, due to their accelerated extracellular export. HuR accelerates this EVs-mediated export of miRNAs. In stressed cells, HuR replaces miRNPs from target messages and is both necessary and sufficient for the export of corresponding miRNAs.

**Results**: HuR reversibly binds miRNAs and replaces them from Ago2 on endoplasmic reticulum and subsequently, itself gets freed from respective miRNAs upon ubiquitination on multivesicular bodies. HuR-unloaded miRNAs get exported out via EVs, thereby delimiting cellular miR-122 level during amino acid starvation.

**Summary/Conclusion**: Therefore, by modulating extracellular export of miR-122, HuR controls stress response and autophagy in amino acid-starved human hepatic cells. I discuss the mechanism of HuR-driven export of miRNAs in immune cells and show how the miRNA export by HuR control immune response in macrophages cells to control proinflammatory response in macrophages.

**Funding**: This work was sponsored by the Department of Science and Technology (DST), Government of India Swarnajayanti Fellowship fund.

PF04.17

Differential interaction of platelet-derived extracellular vesicles with leukocyte subsets in human whole blood

René Weiss^1^; Marion Gröger^2^; Sabine Rauscher^2^; Birgit Fendl^1^; Tanja Eichhorn^1^; Michael Bernhard Fischer^1^; Andreas Spittler^2^; Viktoria Weber ^1^

^1^Department for Health Science and Biomedicine, Danube University Krems, Krems, Austria; ^2^Medical University of Vienna, Vienna, Austria

**Background**: There is evidence that extracellular vesicles (EVs) are primarily associated with granulocytes and monocytes, but scarcely with lymphocytes. In this context, we studied the association of EVs with innate immune cells, particularly with monocyte subsets.

**Methods**: Association of EVs with immune cells was visualized by imaging flow cytometry and confocal microscopy. Monocyte subsets were identified directly in whole blood based on their CD14 and CD16 expression as classical (CM; predominantly phagocytic; CD14++CD16-), intermediate (IM; phagocytic and pro-inflammatory; CD14++CD16+) and non-classical monocytes (NCM; mainly pro-inflammatory; CD14-CD16++) using flow cytometry. The association of monocyte subsets with platelet EVs was detected using lactadherin (LA) as marker of phosphatidylserine and CD41 as platelet marker. In addition to the characterization in whole blood, we studied the association of platelet EVs with monocytes isolated from PBMCs by negative depletion of non-monocytes.

**Results**: Imaging flow cytometry and confocal microscopy confirmed the preferential interaction of platelet EVs with monocytes and granulocytes. The distribution of monocyte subsets in freshly drawn whole blood was 86.1 ± 2.1%, 4.9 ± 1.1% and 9.0 ± 2.6% for CM, IM and NCM, respectively, and freshly isolated monocytes exhibited an almost identical distribution. Overnight resting, however, induced a significant shift towards IM (4.9 ± 1.1% vs. 59.1 ± 24.0%). We found that 5.5 ± 3.6% of all CM, 16.6 ± 6.1% of all IM and 3.5 ± 2.1% of all NCM were CD41+LA+, indicating their association with platelet EVs. Storage of whole blood induced an increase in monocyte-EV aggregates to 66.3 ± 12.1% for CM, to 80.1 ± 8.7% for IM and to 28.4 ± 11.1% for NCM, indicating the preferential association of EVs with CM and IM.

**Summary/Conclusion**: Monocyte isolation and storage induce a shift towards IM. EVs exhibit differential interaction with monocyte subsets and are preferentially associated with CM and IM.

**Funding**: This work was funded by the Christian Doppler Society (Christian Doppler Laboratory for Innovative Therapy Approaches in Sepsis).

PF04.18

Immunomodulatory activity of clinical grade mesenchymal stem cell-derived extracellular vesicles on human NK cell activities

Valeria La Marca^1^; Raffaele Simeoli^1^; Marcin Jurga^2^; Kelly Van Wemmel^2^; Marijke Buvé^2^; Maurizio Muraca^3^; Federico Vigevano^4^; Alessandra Fierabracci ^1^

^1^Infectivology and Clinical Trials Area, Type 1 Diabetes Centre, Children’s Hospital Bambino Gesù, Rome, Italy; ^2^The Cell Factory BVBA (Esperite NV), Niel, Belgium; ^3^Department of Women’s and Children’s Health SDB University of Padova, Padova, Italy; ^4^Department of Neuroscience, Children’ s Hospital Bambino Gesù, Rome, Italy

**Background**: Mesenchymal stem cells (MSCs) exert their biological effects through secretion of extracellular vesicles (EVs). We previously showed that MSC-EVs have immunomodulatory properties on both human T and B cells. Natural killer (NK) cells are essential effectors within our innate immunity but are able to influence the adaptive system as well. They perform the elimination of target cells through secretion of molecules such as perforine/granzyme, cytokines and chemokines. However, to date, our knowledge about the immunomodulatory activity and the control of MSCs over NK cell function is limited. In this study, we aim to investigate the immunomodulatory activity of clinical grade (CG) MSC-EVs on NK activities compared to parent MSCs.

**Methods**: Human umbilical cord-derived MSCs (0.05 × 10^6^) or CG MSC-EVs (CF-MEV-117 at concentration of 5 × 10^9^ EV/mL) were co-cultured alongside human peripheral blood mononuclear cells (PBMC) (0.5 × 10^6^) from normal donators in presence of IL-2 (200 IU/mL) for 4 days. On the fifth day, PBMC were exposed to K562 cells as effector and target, respectively. Degranulation assay and IFN-γ levels were evaluated by flow cytometry following 3 and 24 h incubation with K562, respectively.

**Results**: Percentage of degranulating NK cells (CD56+ CD107a+) was significantly reduced following incubation with both MSCs and MSC-EVs but at higher extent when only EVs isolated from MSCs were used. Similarly, exposure of K562-stimulated PBMC to MSC-EVs induced a significant reduction of CD56+ IFN-γ+ cells compared to co-culture with parental MSCs.

**Summary/Conclusion**: We have developed and standardized a reproducible method for the production, quantification and immunophenotyping of CG EVs with similar immunomodulatory properties of UC MSC progenitors. Our data indicate that the use of MSC-EVs could be effective in the treatment of a wide range of immunological diseases and provide a more accessible alternative for allogenic MSCs.

**Funding**: This work was funded by Esperite NV, Niel, Belgium.

PF04.19

Impact of human blood plasma-derived protein corona on extracellular vesicle uptake

Petter Somersalo; Heikki Saari; Marjo Yliperttula

University of Helsinki, Division of Pharmaceutical Biosciences, Helsinki, Finland

**Background**: The introduction of a nanoparticle into blood plasma results in plasma proteins being adsorbed to its surface, forming a protein corona. The formation of the corona is a dynamic process, governed by individual protein concentrations as well as their respective affinities for the surface. Proteins of the corona interact with surrounding cells, thus being able to influence the cellular uptake of the nanoparticle.

In this study, said phenomenon was investigated with regard to extracellular vesicles (EVs) isolated from the conditioned media of PC-3 prostate cancer cells. Moreover, the impact of a human blood plasma-derived protein corona on EV uptake into PC-3 cells was assessed.

**Methods**: EVs were isolated from collected PC-3 cell culture medium using differential centrifugation. Experiments were performed separately for a 20000 x*g* EV-fraction (here referred to as microvesicles) and an 110000 x*g* EV-fraction (here referred to as exosomes). Size distributions and concentrations of EVs were determined by nanoparticle tracking analysis. Analysis of plasma proteins adsorbed to the EV surface was done by incubating a dialysis unit containing EVs in plasma, followed by sodium dodecyl sulfate polyacrylamide gel electrophoresis. Cellular uptake of EVs was assessed using confocal microscopy.

**Results**: The protein band patterns of plasma-exposed EVs indicated adsorption of plasma proteins to their surface. Furthermore, plasma exposure resulted in a slightly reduced or unchanged uptake of DiO-labelled EVs, probably at least partly mediated by the protein corona formed.

**Summary/Conclusion**: The impact of a protein corona on EV distribution seems to be an aspect poorly evaluated, even though its implications might be of major importance, e.g. in a drug delivery context. It is worth noting that the current study limits itself to the use of PC-3-derived EVs and PC-3 cells as recipient cells. Indeed, it would be essential to investigate this issue more widely, using a large array of cell lines both in EV production and as target cells.

**Funding**: This project was funded by the Finnish Funding Agency for Innovation (TEKES, now part of the Business Finland organization) and Academy of Finland.

PF04.20

TNF-α and opsonized particles stimulate different type of extracellular vesicle production from neutrophilic granulocytes

Viktória Szeifert; Ákos Lőrincz; Balázs Bartos; Erzsébet Ligeti

Department of Physiology, Semmelweis University, Budapest, Hungary

**Background**: Previously, our group characterized three distinct extracellular vesicle (EV) populations released from human neutrophilic granulocytes (polymorphonuclear neutrophils, PMN): EVs formed spontaneously (sEVs), upon activation with opsonized particles (aEVs) and during apoptosis (apoEVs). Our aim was to examine and compare the TNF-α-induced EV production with our previously described EV populations.

**Methods**: Medium-sized EVs were separated by a two-step centrifugation from PMNs isolated from the peripheral blood of healthy volunteers under different conditions (sterile/non-sterile) and at different time points (immediately/delayed). We evaluated the EV release based on their count determined by flow cytometry, their protein amount determined by Bradford assay and their protein cargo determined by proteomic analysis. Viability of cells during activation was also followed to evaluate apoptosis and apoEV production.

**Results**: Primed PMN produced proportionally more EV under all circumstances than PMN prepared under sterile conditions did. Surprisingly, this priming effect could not be replaced by TNF-α treatment. TNF-α treatment increased EV production by naive PMN; however, it could not raise EV release induced by opsonized particles. Both sterile preparation and TNF-α treatment increased apoptosis during opsonized Zymosan activation. Older neutrophils showed the lowest EV production in each group and the worst EV answer upon opsonized particles.

**Summary/Conclusion**: Our data suggest that TNF-α-induced EV production is independent from EV generation triggered by opsonized particles. TNF-α-induced EVs may represent a fourth distinctive type of EVs derived from human PMN.

**Funding**: This work was funded by NKFIH K119236, VEKOP-2.3.2-16-2016-00002, Hungary.

PF04.21

Differentiating C2C12 myocytes release exosomes and shedding microvesicles that trigger different inflammatory responses in RAW264.7 macrophages

Michele Guescini; Serena Maggio; Paola Ceccaroli; Emanuela Polidori; Michela Battistelli; Giosuè Annibalini; Vilberto Stocchi

Dipartimento di Scienze Biomolecolari (DISB), University of Urbino, Urbino, Italy

**Background**: Skeletal muscle is a highly plastic tissue capable of adapting to different stresses. This feature is largely attributable to the presence of satellite cells. Within the satellite cell niche, muscle stem cells exchange signals with other cell types, and among these, complex interactions between skeletal muscle and the immune system have been reported. It has been shown that during myogenic differentiation, myotubes release extracellular vesicles (EVs) which participate in the signalling pattern of the microenvironment. Here we investigated whether EVs released by differentiating myocytes can mediate cell communication between muscle cells and macrophages.

**Methods**: RAW264.7 cells and C2C12 mouse adherent myoblasts were cultured in DMEM supplemented with 10% heat-inactivated foetal bovine serum, 2 mM glutamine, penicillin (100 U/mL) and streptomycin (100 μg/mL), and maintained in a 5% CO_2_ atmosphere at 37°C. EVs were purified by serial centrifugations and finally pelleted by ultracentrifugation at 110,000 *g*. The EVs collected during myogenic differentiation process were characterized using transmission electron microscopy, Western blot and density gradient.

**Results**: To evaluate whether EVs released by differentiating myocytes could mediate muscle-macrophage communication, exosomes and shedding microvesicles isolated from C2C12 cells were used to treat RAW264.7 cells, a suitable cell line model of macrophages. The mRNA expression analysis of key macrophage markers showed that after treatments, IL-6 and IL-1β were mainly upregulated in response to shedding microvesicles, whereas IL-10 stimulation was obtained using exosomes.

**Summary/Conclusion**: Exosomes and shedding microvesicles released from differentiating myocytes show a tendency to differentially modulate the IL-6 and IL-10 expression levels in RAW267.4 macrophages. These new findings will help to shed light on the mechanisms underlining intercellular communication during muscle regeneration and repair.

**Funding**: MG was supported by Italian Ministry of Health (GR-2011-02350264)

PF05: EV-based Non-cancer Biomarkers Chairs: Anabela Cordeiro; Melissa Gualdron Location: Exhibit Hall 17:15–18:30

PF05.01

MicroRNA signature from plasma-derived EVs for dementia with Lewy bodies as promising non-invasive biomarkers

Ana Gamez-Valero^1^; Francesc E. Borràs^2^; Katrin Beyer^3^

^1^HUGTiP and IGTP Institute with the Universitat Autónoma de Barcelona, Badalona, Spain; ^2^REMAR-IVECAT Group, “Germans Trias i Pujol” Health Science Research Institute, Can Ruti Campus, Badalona, Spain; ^3^Institut d’Investigació en Ciéncies de La Salut Germans Trias i Pujol, Badalona, Spain

**Background**: Dementia with Lewy bodies (DLB) shows overlapping features with Alzheimer disease (AD) leading to its misdiagnosis and hindering its adequate treatment. It is well established that microRNAs play an important role in neurodegeneration and they can be found in brain and the central nervous system. Most cell types, from reticulocytes to neurons, secrete extracellular vesicles (EVs) which specific composition depends on the secreting cell-type and cellular status, thus making them attractive for biomarker discovery. EVs’ size allows them to pass across the blood–brain barrier being able to obtain brain-derived EVs and central nervous system-related vesicles in blood circulation.

Thus, we hypothezied that changes in the molecular composition of vesicles from DLB/AD patients may be indicative of disorders affecting the brain. Our main objective was to identify disease-specific microRNA biosignatures through the analysis of plasma-derived EVs from DLB, AD patients and age-matched control individuals.

**Methods**: EVs were isolated using size exclusion chromatography and characterized by nanoparticle tracking analysis, cryogenic electron microscopy and flow cytometry against the vesicular markers CD9, CD81 and CD63. After lyophilization, small RNA was extracted using a smallRNA purification kit following manufacturer’s instructions. By next-generation sequencing, we obtained a profile of more than 300 microRNAs present in both DLB and healthy control cohorts.

**Results**: A panel of 22 miRNAs differentially expressed between the groups and identified as possibly disease-related was selected for validation by quantitative PCR. From those, a smaller group of miRNAs were considered as potential biomarkers for DLB being evaluated in a group of AD patients and also in addittional independent groups of control and DLB individuals by qPCR.

**Summary/Conclusion**: Although preliminary, these results represent an integrated miRNA profile in plasma-EVs that is likely to provide non-invasive biomarkers for the differential diagnosis of DLB versus AD. Moreover, we confirmed that changes related to neurodegeneration could be reflected in blood circulation which represents an unvaluable information available under minimally invasive procedures.

**Funding**: This work was supported by Spain’s Ministry of Health FIS grants [PI12/1702 and PI15/216] and the MaratóTV3 grant [1405/10].

PF05.02 = OWP2.04

Normalization of urinary extracellular vesicles

Charles J. Blijdorp_1_; Thomas A. Hartjes_1_; Martin E. van Royen_2_; Guido W. Jenster_1_; Robert Zietse_1_; Ewout J. Hoorn_1_

^1^Erasmus Medical Center, Rotterdam, The Netherlands; ^1^Department of Pathology, Erasmus Optical Imaging Centre, Erasmus MC, Rotterdam, The Netherlands

**Background:** Urinary extracellular vesicles (uEVs) have emerged as a powerful non-invasive tool to study renal epithelial transport in humans. However, the optimal method to quantify and normalize uEVs remains unclear, especially for spot urines.

**Methods:** Four healthy subjects were subjected to overnight thirsting (10 pm-noon) followed by water loading (20 ml/kg in 30 min). Spot urines were collected during thirsting (T1-2) and after water loading (WL1-4, noon-7 pm). Subsequently, 4 uEV quantification techniques were compared: (1) nanoparticle tracking analysis (NTA), (2) uEV isolation by ultracentrifugation followed by immunoblotting of CD9, CD63, CD81, ALIX, and TSG101, (3) a timeresolved fluorescence immunoassay (TRFIA) that captures CD9+ uEVs, and (4) EVQuant, a novel technique which counts individual fluorescently labeled EVs after immobilization in a matrix. A Bland-Altman analysis was used to compare methods using NTA as reference.

**Methods:** As expected, urine osmolality was near-maximal during thirsting, decreased after water loading and then increased again. The results of the 4 uEV quantification methods showed similar dynamics as urine osmolality suggesting that uEV number changes in proportion to urinary concentration. Of interest, EVQuant identified 2.4 ± 0.5 times more uEVs than NTA. Using NTA as reference, the Bland-Altman analysis showed that EVQuant had the best agreement (SD of bias 16%) followed by TRFIA (SD of bias 22%). Of the uEV-markers, CD9 agreed best with NTA (SD of bias 28%). uEV number correlated strongly with urine creatinine (R2 0.9, P<0.0001).

**Summary/Conclusion:** uEV number is proportional to urinary concentration and urine creatinine can be used to normalize spot urines for uEV number. EVQuant is a promising alternative to NTA and appears more sensitive for uEV detection. These uEV quantification methods can also be used to analyze if changes in a uEV protein of interest are the result of more protein per uEV or the excretion of more uEVs containing this protein.

**Funding:** Dutch Kidney Foundation.

PF05.03

Urinary exosomes and the packing CCL-2 mRNA as biomarkers of IgA nephropathy

Ye Feng; Linli Lv; Weijun Wu; Zuolin Li; Leting Zhou; Bicheng Liu

Zhong Da hospital, Nanjing, China (People’s Republic)

**Background**: Immunoglobulin A nephropathy (IgAN) is characterized by variable histological changes and clinical course; thus, non-invasive biomarkers reflecting the histological injury and progression of renal function are needed. Here we reported that urinary exosomes and the packing CCL2 mRNA could serve as novel biomarkers of IgAN.

**Methods**: A screening (6 IgAN, 6 healthy controls) and a validation cohort (55 IgAN, 24 healthy controls) of patients with biopsy-proven IgAN and healthy controls were enrolled in our study. We isolated exosomes from urine samples at the time of renal biopsy. Thirty-seven patients were followed up during the study. Kidney histological damage of IgAN patients was scored according to the Oxford classification. Urinary exosome protein and profile of the packing inflammatory response-related genes were assessed and their correlation with clinic and histological injury parameters were analysed.

**Results**: Urinary exosome release was increased remarkably in IgAN patients compared to controls and strongly correlated with levels of proteinuria and tubular injury. Furthermore, exosome production is associated with higher histological activity (mesangial hypercellularity, cellular crescent and endocapillary hypercellularity). Profile of the packing inflammatory-related mRNA revealed CCL-2 was remarkably upregulated in IgAN patients. Validation study confirmed the findings and found its correlation with the levels of estimated glomerular filtration rate. Furthermore, CCL-2 was positively correlated with tubulointerstitial inflammation and fibrosis, and C3 deposition. Impressively, CCL-2 showed good performance in discriminating patients with different levels of tubulointersitial inflammation. Besides, in the follow-up population, high CCL-2 levels at the time of renal biopsy are associated with progressive renal function deterioration.

**Summary/Conclusion**: In summary, urinary exosomes and the packing CCL-2 mRNA may be promising non-invasive biomarkers of IgAN reflecting renal histological injury and renal function deterioration.

**Funding**: This study was supported by the National Natural Scientific Foundation (No. 81470922, 31671194, 81720108007, 81670696) and Clinical Research Center of Jiangsu Province (No. BL2014080) and Jiangsu Province Medical Youth Talent (QNRC2016818).

PF05.04

Characterization and proteomic profile of extracellular vesicles from peritoneal dialysis efflux

Laura Carreras-Planella; Marta Monguió-Tortajada; Jordi Soler-Majoral; Cristina Rubio-Esteve; Marcella Franquesa; Josep Bonet; Maria Isabel Troya-Saborido; Francesc E. Borràs

REMAR-IVECAT Group, “Germans Trias i Pujol” Health Science Research Institute, Can Ruti Campus, Badalona, Spain

**Background**: Peritoneal dialysis (PD) is considered the best option for a cost-effective mid-term dialysis in patients with chronic renal failure. However, functional failure of the peritoneal membrane (PM) forces many patients to stop PD treatment and start haemodialysis. Currently, PM functionality is monitored by the peritoneal equilibration test, a tedious technique that often shows changes when the membrane damage is advanced. As in other pathologies, the identification and characterization of extracellular vesicles (EVs) in the peritoneal dialysis efflux (PDE) may represent a non-invasive alternative to identify early biomarkers of PM failure.

**Methods**: Using size-exclusion chromatography, we isolated EVs from PDE of newly enrolled and longer-treated PD patients. EVs were characterized by the presence of tetraspanin markers, nanoparticle tracking analysis profile, cryo-electron microscopy and their content proteomic profile was analysed by mass spectrometry.

**Results**: We report the isolation and characterization of PDE-EVs. Based on mass spectrometry, we found a set of well-conserved EV protein markers among patients. Interestingly, the proteomic profile also revealed remarkable changes between the two groups of patients.

**Summary/Conclusion**: These results are the first step to the identification of PDE-EVs-based new markers of PM damage, which could support clinicians in their decision-making in a non-invasive manner.

**Funding**: This work was supported by grants from Instituto de Salud Carlos III (FIS PI16/00072), “Suport Grups de Recerca” programme of Generalitat de Catalunya (2014SGR804, Group REMAR), Instituto de Salud Carlos III-Red de Investigación Renal (REDinREN) (RD16/0009 Feder Funds), and Fundació Cellex. MF was sponsored by the Beatriu de Pinós-B contract (2014BP B00118) from Agència de Gestió d’Ajuts Universitaris i de Recerca (AGAUR) – Generalitat de Catalunya. FEB was sponsored by the “Researchers Stabilization Program” from the Spanish “Sistema Nacional de Salud” (SNS- ISCIII) and “Direcció d’Estratègia i Coordinació” Catalan Health Department (CES07/015). The funders had no role in study design, data collection and analysis, decision to publish,or preparation of the manuscript.

PF05.05

Sputum exosomes: promising biomarkers for idiopathic pulmonary fibrosis

Makon-Sébastien Njock ^1^; Julien Guiot^2^; Monique Henket^2^; Olivier Nivelles^1^; Renaud Louis^2^; Ingrid Struman^1^

^1^Laboratory of Molecular Angiogenesis, GIGA-R, University of Liège, Liège, Belgium; ^2^Department of Pneumology, CHU Liège, Liège, Belgium;

**Background**: Idiopathic pulmonary fibrosis (IPF) is a progressive fibrosing interstitial lung disease of unknown etiology which leads rapidly to death. As diagnosis of IPF is complex, the development of novel molecular biomarkers is a central challenge for the future of translational research. Consequently, we sought to characterize microRNA (miR) content of exosomes from sputum of IPF patients compared to healthy donors in order to identify novel biomarkers of the disease.

**Methods**: Exosomes were isolated from induced sputum samples of 14 IPF patients diagnosed following American Thoracic Society (ATS) /European Respiratory Society (ERS) recommendations and 11 healthy donors with standard ultracentrifugation protocol. Exosomal miR content was analysed by miR qPCR arrays, and diseases/biological processes associated to altered miRs were determined by bioinformatic analysis.

**Results**: The presence of exosomes was confirmed in sputum from both IPF patients and healthy donors. The profiling of exosomal miRs revealed 21 differentially expressed miRs in the sputum of IPF patients compared to healthy donors. Further validation of miRs presenting an aberrant expression allowed us to identify for the first time an IPF-specific miR signature from sputum exosomes, among which miR-142-3p and miR-33a-5p present an upregulation (fold change (FC)>3, *p* < 0.01), whereas let-7d-5p a downregulation (FC < 0.5, *p* < 0.01). The bioinformatic analysis revealed that altered miRs are associated to inflammatory diseases, among which IPF is the most relevant one (*p* = 3.78E-10). Interestingly, most of the biological processes highlighted in this analysis are in agreement with IPF etiology, which confers to our candidates an evident role as IPF biomarkers. Based on these findings, functional tests with IPF-sputum exosomes and mimics of altered miRs are underway to test their impact on IPF progression.

**Summary/Conclusion**: For the first time, we identified potential biomarkers for IPF from sputum exosomes. Our findings may thus lead to a better understanding about the roles of these miRs in the pathogenesis of IPF and thus open new avenues for therapeutic approaches. This study reinforced the concept that sputum exosomes might be a novel source of biomarkers for the diagnosis of pulmonary diseases.

**Funding**: This work was supported by University of Liège; Fonds National de la Recherche Scientifique; and Fonds d’Investissement de Recherché Scientifique du Centre Hospitalier Universitaire de Liège.

PF05.06

Use of *Leishmania* promastigote EVs in serological diagnosis of leishmaniasis

Sofia S. Esteves; Inês Costa; Nuno Santarém; Anabela Cordeiro-da-Silva

Parasite Disease Group, Instituto de Biologia Molecular e Celular, Instituto de Investigação e Inovação em Saúde, Universidade do Porto, Portugal

**Background**: Digenetic protozoan parasites of the genus *Leishmania* are the etiological agents of vector-borne human-neglected tropical diseases known as leishmaniasis. This disease affects between 0.7 and 1 million being responsible for 20–30,0000 deaths/year. These parasites can also infect dogs (the most common reservoir) originating a widespread veterinary problem, Canine leishmaniasis (CanL). In the absence of vaccines (for humans), disease control requires the capacity to detect not only diseased individuals (symptomatic) but also infected (asymptomatic). More so this is essential as these asymptomatic individuals contribute to the perpetuation of the disease by acting as reservoirs. The available tools to detect infection are suboptimal with an ongoing effort by the community to find new markers for asymptomatic cases. In this context, the promastigote EVs are a possibility to exploit as they contain many of the proteins that are abundant in the promastigote form of the parasite (the infective form). In this context, these EVs should be efficiently recognized in the early stages of infection.

**Methods**: To address this, EVs were recovered from promastigote culture in a defined media. The EVs were recovered by a combination of filtration and ultracentrifugation, characterized by transmission electron microscopy, dynamic light scattering and nanoparticle tracking analysis. The evaluation of recognition was performed using ELISA and cut-off determination by receiver operating characteristic curve analysis using sera from healthy, symptomatic and asymptomatic cases for both human and CanL. To better understand the EVs potential, the same samples were also evaluated using soluble promastigote Leishmania antigens (SPLA), total exogenous antigens and vesicle-depleted exogenous antigens.

**Results**: The results obtained for the human and canine samples were distinct. The EVs in the context of CanL presented very high specificity and sensitivity, being also seropositive in 90% of the asymptomatic cohort, while SPLA was only seropositive for 50% of the samples. For the human disease, the serological performance of the EVs was not inferior to SPLA in symptomatic and asymptomatic groups.

**Summary/Conclusion**: Overall, the aforementioned data suggest that *Leishmania* EVs contain antigens that might be used to improve serological evaluation of infection in CanL and also as a possible alternative to conventional serology in symptomatic cases.

**Funding**: This work was funded by NORTE-01-0145-FEDER-000012.

PF05.07

An increased level of CD41-positive extracellular vesicles recovered by 100,000 ×*g* centrifugation from stimulated platelets

Chun-yi Chiang; Chihchen Chen

Institute of Nanoengineering and Microsystems, National Tsing Hua University, Hsinchu, Taiwan (Republic of China)

**Background**: Circulating extracellular vesicles (EVs) have been implicated in various (pro)inflammatory and metabolic conditions, including cardiovascular diseases, pregnancy, cancers and diabetes. Platelet-derived EVs have been reported to be the most abundant EVs in human blood. However, there are major inconsistencies in the numbers of circulating EVs reported in the literature, and many variables that may affect quantification of EVs. It is often presumed that circulating EVs bare the same membrane lineage markers as their originating cells, which may not be the case. In addition, the indiscrimination of large and small EVs in some studies further introduces confusion.

**Methods**: The aim of this study was to characterize the number, size profile and compositions of circulating EVs derived from normal or calcium ionophore, A23871, stimulated platelets or red blood cells (RBCs). To obtain RBCs and platelets of high purity, an iodixanol barrier isolation method was optimized to minimize both the contamination by other cell types and platelet preactivation. EVs were collected after platelets and RBCs were incubated in Tyrode-HEPES buffer at 37°C for 30 min. EVs were enumerated using nanoparticle tracking analysis (NTA), and recovered by centrifugation at 10,000 ×*g* (10K pellet) or 100,000 ×*g* (100K pellet) to examine the presence of surface markers by Western blot.

**Results**: We prepared EVs from above 99.92% pure platelets and RBCs and collected EVs *in vitro*. NTA showed that more than 70% of EVs derived from RBCs were smaller than 100 nm, while only 16% and 5% of EVs were derived from unstimulated and stimulated platelets, respectively. Each platelet secreted 64 ± 3 EVs per hour on average with the mean diameter of 146 ± 6 nm. Importantly, the expression level of CD41, a platelet-specific marker, was highly expressed in the 10K EV pellet, but not detected in the 100K EV pellet. Upon calcium ionophore stimulation, platelets secreted 3.7 times more EVs compared to normal controls. In addition, CD41 expression in the 10K pellet decreased from 1.20 to 0.94 relative to that in the platelet cell lysate.

**Summary/Conclusion**: Among 100K blood cell-derived EV pellets, CD41 was only detected in EVs released from stimulated platelets, suggesting the expression of CD41 in the 100K pellet may be utilized as a biomarker indicating the activation of platelets.

**Funding**: This work was funded by MOST [106-2221-E-007-003, 106-2628-E-007 -010 -MY3].

PF05.08

Circulating exosomal microRNAs in obstructive sleep apnea

David Sanz-Rubio ^1^; Inmaculada Martin-Burriel^2^; Victoria Gil^1^; Marta Forner^3^; J Pablo Cubero^1^; José Mª Marin^1^

^1^HCU Miguel Servet/IIS Aragón, Zaragoza, Spain; ^2^Departamento de Anatomía, Embriología y Genética Animal, Universidad de Zaragoza, Zaragoza, Spain; ^3^HCU Miguel Servet/CIBERES, Zaragoza, Spain

**Background**: Obstructive sleep apnea (OSA) is a prevalent respiratory sleep disorder. Epidemiological studies indicate that there may be an association between OSA and cardiovascular and metabolic diseases. Some microRNAs (miRNAs) overexpressed in atherosclerosis or related to inflammation and hypoxia could have a main role in OSA and their comorbidities.

**Methods**: OSA patients were recruited from a Sleep Unit in the frame of a long-term longitudinal cohort study. We selected 88 OSA patients who were non-smokers (apnea–hypopnea index (AHI) ≥30 events/hour) and 25 matched controls (AHI < 5). They did not display comorbidities other than OSA at baseline (GovTrials, NCT014575421). At recruitment and yearly, carotid ultrasound was performed and subclinical atherosclerosis (SA) was defined as by the presence of carotid plaques or by an intima-media thickness >0.85 mm. Plasma-derived exosomes were isolated by precipitation using miRCURY™ Exosome Isolation Kit. Exosomes were characterized by transmission electron microscopy, dynamic light scattering assay and Western blot. Exosome total RNA was obtained using miRCURY™ RNA isolation kit. miRNAs were analysed by real-time quantitative PCR (RT-qPCR) using miRCURY LNA™ technology.

**Results**: At baseline, the expressions of miR-21-5p were increased in patients with OSA compared to controls (fold change (FC): 1,74 (*p* < 0.05)), being higher in patients with SA (*n* = 38; FC:1,85). miRNA-320a-3p showed a significantly increased (*p* < 0.05) expression in OSA patients with SA (FC: 1,59). At 1-year follow-up, the expression of miR-320a-3p kept significantly elevated in OSA patients with SA not treated with continuous positive airway pressure (CPAP) (*n* = 13; FC:1,88) and showed an increased expression in OSA patients without SA treated with CPAP (*n* = 28; FC:1,48). miR-21-5p displayed a persistent overexpression among non-treated OSA patients without SA (FC:2,51) and a decreased in patients treated with CPAP (FC: 1,64).

**Summary/Conclusion**: Circulating exosomes cargo of miR-21-5p and miR-320a-3p are increased in patients with OSA and SA. After 1 year of effective treatment with CPAP in OSA patients, circulating exosomal miR-21-5p seems to be more sensible to CPAP treatment. This study suggests that those miRNAs may play a role as an intermediary mechanism in cardiovascular morbidity in OSA.

**Funding**: This work was supported by Instituto Carlos III, Ministry of Health (PI/2175 and PI/1940).

PF05.09

Extracellular vesicle analysis for biomarker identification in cerebral spinal fluid and blood from patients with Parkinson’s disease

Miles Trupp; Anna Gharibyan; Shaochun Zhu; Lars Forsgren

Pharmacology and Clinical Neuroscience, Umeå University, Umea, Sweden

**Background**: Parkinson’s disease is a progressive neurodegeneration that can begin in olfactory and vagal neurons and may spread via misfolded and aggregated alpha-synuclein in extracellular vesicles. The development of disease-modifying medications can be improved by the discovery of early biomarkers of disease and the characterization of the molecular mechanisms of transfer of aggregated proteins between neurons. We are attempting to identify molecular markers of toxic vesicles as candidate biomarkers for disease progression and therapeutic targets.

**Methods**: We have isolated and characterized exosomes from neuronal and glial cells as well as from cerebrospinal fluid and blood. We have used electron and atomic force microscopy to analyse their physical properties, cell-based assays for functional studies and mass spectrometry-based proteomics to characterize their molecular composition.

**Results**: In cell culture systems, pathological conditions such as mitochondrial stress can affect both physical properties and protein composition of exosomes. In particular, stress-induced exosomes appeared to be smaller and more homogeneous in size than those produced by the cells growing in normal conditions. We have identified proteins altered in exosomes from stressed neuronal and glial cells using mass spectrometry-based proteomic profiling. These candidate biomarkers for toxic exosomes are being used for targeted multiple reaction monitoring assays using extracellular vesicles isolated from cerebral spinal fluid and plasma from patients with Parkinson’s disease.

**Summary/Conclusion**: Our goal is to identify robust biomarkers for diagnosis of parkinsonian disorders based on isolation of exosomes from patient samples collected at Umeå University Hospital. These biobanks include longitudinal cerebral spinal fluid samples collected from patients during progression of Parkinson’s disease and pre-symptomatic blood samples collected many years prior to diagnosis of disease. The fractionation of biofluids based on extracellular vesicle extraction increases the number of detectable proteins and allows the identification of additional candidate biomarkers for neurodegenerative diseases.

**Funding**: This work was funded by the Swedish Parkinson Foundation, Kempe Foundation, Hjärnfonden, Erling Persson Family Foundation, and Swedish Research Council.

PF05.10

Gestational age prediction using maternal serum and placental miRNA expression

Peter De Hoff; Srimeenakshi Srinivasan; Louise C. Laurent; Cuong To; Clara Laurent; Antoinette Mason; Kajal Verma; Aileen Fernando; Katharine Knepper; Jennifer Tasarz; Aishwarya Vuppala; Rachael Overcash; Richelle Olsen; Hilary Roeder; Gladys Ramos; Mana Parast

University of California, San Diego, CA, USA

**Background**: Extracellular RNAs (exRNAs) have potential utility as predictive, diagnostic and prognostic biomarkers for a variety of conditions. In any given biofluid, exRNAs originate from different tissues, potentially obscuring relevant tissue/disease-associated patterns. Pregnancy is a unique condition in which an entirely new organ, the placenta, is formed. The goal of this study is to identify miRNAs that display gestational age-specific expression in the human placenta and in maternal serum, and to compare these expression patterns.

**Methods**: Small RNAseq was performed on 64 placentas and 72 maternal serum samples collected across gestation. Total RNA was extracted from the placental tissues, and both total RNA and EV-enriched RNA was isolated from the serum samples. NEBNext Small RNA NGS libraries were prepared with using a miniaturized protocol adapted for use on the Mosquito HTS robotics platform. The Illumina HS4000 1×50 deep sequenced libraries were processed through the ExceRpt pipeline. The resultant data were filtered and normalized, and co-expressed miRNA clusters were identified using affinity propagation.

**Results**: Distinct clusters of miRNAs displaying gestational age-specific expression patterns were identified in both the placental tissue and maternal serum datasets. However, for many miRNAs, including known placenta-specific miRNAs, the expression patterns differed markedly between the two datasets, perhaps due to selective secretion of placental small RNAs from different placental cell types.

**Summary/Conclusion**: Nevertheless, the fact that both placental and maternal serums contain miRNAs that display strong gestational age-specific patterns of expression is promising for development of a pregnancy “clock” that can aid in dating of pregnancies where standard clinical criteria are unavailable.

PF05.11

Circulating microRNAs as potential biomarkers of pediatric acute lymphoblastic leukemia

Andrea Rzepiel ^1^; Nóra Kutszegi^1^; Bálint Egyed^1^; András Gézsi^2^; Judit C. Csányi^1^; Ágnes F. Semsei^1^; Gábor Nyírő^1^; Judit Müller^1^; Csaba Szalai^1^; Dániel J. Erdélyi^1^

^1^Second Department of Pediatrics, Semmelweis University, Budapest, Hungary; ^2^MTA-SE Immune-Proteogenomics Extracellular Vesicle Research Group, Budapest, Hungary

**Background**: Acute lymphoblastic leukemia (ALL) is the most common pediatric malignancy. There are approximately 60 new ALL cases per year in Hungary. Extracellular vesicles containing microRNAs are in great interest of scientific research. Their role is not fully understood, especially not in pediatric leukemia. Altered microRNA expression pattern is established in many malignant conditions. The aim of this study was to identify a set of microRNAs associated with pediatric ALL and its genetic subgroups.

**Methods**: Platelet-free plasma samples were obtained from 16 newly diagnosed *de novo* and 5 relapsed pediatric ALL patients and 10 healthy controls. RNA isolation was carried out using Qiagen miRNeasy Serum/Plasma Kit. Quantification of 46 candidate miRNAs was performed using Custom TaqMan Advanced Low Density miRNA Array Card.

**Results**: The expression of 19 microRNAs showed significant difference when comparing ALL and healthy control platelet-free plasma samples (*p* < 0.05). miR-128-3p, miR-181b-5p and miR-222-3p elevated most significantly in ALL samples.

No difference was found in microRNA levels of hyperdiploid, ETV6/RUNX1 fusion-positive and normal karyotype ALL patients.

**Summary/Conclusion**: Based on the literature, the role of miR-128 and miR-181 family members is known in normal lymphopoiesis, which can explain the background of our findings. Tumour suppressor gene *TP53* as a target of certain microRNAs such as miR-222 might take a part of the development of leukemia. Circulating microRNAs might serve as biomarkers for pediatric ALL.

**Funding**: This study was supported by the National Research, Development and Innovation Office (NKFIH) K115861.

PF06: Novel Developments in EV Isolation Chairs: Carmen Fernandez; Felix RoyoLocation: Exhibit Hall 17:15–18:30

PF06.01

Characterization of RNA contained in highly purified exosomes from foetal bovine serum

Filiberto A. Bautista-Moreno; Mariana Flores-Torres; Selma Eréndira Avendaño-Vazquez; C. Fabián Flores-Jasso^2^

Instituto Nacional de Medicina Genómica, Ciudad de México, Mexico

**Background**: Recently, Krichevsky’s lab described the classes of RNA contained in a foetal bovine serum (FBS) by separating vesicular and non-vesicular fractions and concluded that extracellular vesicles (EVs) could mediate microRNA transfer into cell cultures potentially biasing the results of small RNA detection. Before deep-sequencing, the RNA was isolated from exosome pellets obtained by ultracentrifugation. However, it is been widely shown that ultracentrifugation pellets are highly contaminated by non-vesicular proteins, some of which potentially include members of Argonaute family and cognate microRNAs. The drawback of using ultracentrifugation as sole procedure to isolate exosomes is that it may drag down non-vesicular proteins, difficulting results interpretation. We aimed to obtain a highly pure EVs fraction in order to characterize the RNAs present in that fraction and compare it with the RNA contained in the whole FBS.

**Methods**: To cope with this problem, we isolated the EVs using a combination of pre-existing techniques. First, we used a size-exclusion chromatography, followed by an ultrafiltration with 100-KDa filters, which concentrate the samples and achieve to eliminate the proteins smaller than 100 KDa. Then, we performed a precipitation of the EVs using the Vn96 peptide, which has affinity for the heat-shock proteins, particularly the family 70 (Hsp70). We evaluated the EVs purity by Western blot, and isolated and deep sequenced the small RNA from whole FBS and the Hsp70-positive EVs containing fraction.

**Results**: By using a mix of three pre-existing techniques, we could obtain a highly pure EVs fraction from FBS. We obtained the RNA profile, where we observed a different pattern in the expression profiles of the RNAs from EVs and the whole FBS.

**Summary/Conclusion**: The differences between the EVs and whole FBS classes of RNAs might be important when similar results are found in others biofluids that are used or proposed as biomarkers, due to some of the RNAs present in the non-vesicular fraction which could potentially interfere with diagnosis. The functionality of the miRNAs found in the bovine EVs fraction remains to be defined and tested in non-bovine cell cultures.

PF06.02

Comparison of foetal calf serum EV-depletion protocols indicates differences in depletion efficiency of miRNAs and other RNA classes

Tom Driedonks; Maarten Nijen Twilhaar; Marca H.M. Wauben; Esther N.M Nolte-’t-Hoen

Department of Biochemistry & Cell Biology, Faculty of Veterinary Medicine, Utrecht University, Utrecht, The Netherlands

**Background**: Foetal calf serum is a common supplement of cell culture medium and a known source of contaminating extracellular vesicles (EV) that contain RNA. To prevent unwanted interference in (functional) characterization of cell culture EV, FCS-EV are commonly depleted by overnight ultracentrifugation. It was recently shown that only part of the FCS-RNA could be depleted by ultracentrifugation, and that remaining FCS-RNA may confound EV-RNA analyses. Since different methods to deplete bovine EV-RNA from FCS are being used, we compared their efficiency to deplete bovine RNA, and determined the contribution of remaining bovine RNA to EV-RNA purified from cell cultures.

**Methods**: We tested the effects of (1) FCS dilution factor and (2) decanting versus pipetting off EV-depleted supernatant on FCS-RNA depletion. The depletion efficiency of specific bovine miRNAs and various other non-coding RNAs was determined by RT-qPCR. Murine cell lines releasing high or low numbers of EV were cultured in EV-depleted media, for which the contribution of bovine RNA to EV-RNA isolates was assessed.

**Results**: Depletion of FCS-RNA after overnight ultracentrifugation was most efficient in diluted FCS and when avoiding decanting of supernatant. Dilution of FCS from 100% to 30% increased the percentage of pelleted RNA from 16% to 39% (average *n* = 3). Interestingly, bovine small non-coding RNAs 7SL and Y4-RNA were depleted more efficiently than miRNAs. Moreover, EV from murine cell lines cultured in EV-depleted medium showed strong enrichment of RNAs conserved in murine/bovine genomes, whereas bovine-specific RNAs were not enriched.

**Summary/Conclusion**: Optimization of FCS-EV depletion protocols reduces the levels of contaminating bovine RNAs in culture medium, but the depletion efficiencies for different RNA classes are variable. Accurate reporting of EV-depletion methods and inclusion of medium control samples will therefore increase experimental reproducibility in EV-RNA studies.

**Funding**: This work was supported by European Research Council under the European Union’s Seventh Framework Programme [FP/2007–2013]/ERC Grant Agreement number [337581].

PF06.03

How good is the gold standard – the impact of methods on exosome function study

Hridika Barua; Snehal Midge; Yumei Zhang; Phuong Tran; Wang Yin; Wei Duan

School of Medicine in Melbourne, Deakin University, Australia

**Background**: Approximately half of the published extracellular vesicle (EV)/exosome papers used cell culture-based system to generate EV for both biochemical and cell biological studies. Majority of those studies on human EV/exosomes used various percentage of “exosome-depleted serum” (EDS), serum of bovine origin that has been processed to “deplete” bovine EV/exosomes. Many researchers in the EV field, especially those newly entered the EV field, are under the impression that “EDS” is devoid of EV/exosomes of bovine origin, as its name implied.

Recently, however, growing number of EV/exosome researchers start to appreciate the potential impact of bovine-derived EV/exosome in the preparations of human EV/exosome using cell culture.

Herein, we examined if the “EDS” is really depleted of bovine EV/exosome.

**Methods**: EDS was prepared from foetal bovine serum (Bovogen, USA) as described in the 2006 method article. Foetal bovine serum (FBS) was diluted 1:5 using phosphate-buffered saline. The diluted 20% FBS was centrifuged at 100,000 ×*g* using TLA-110 fixed angle rotor for 18 h at 4°C. The number of particles present in serum was measured using Nanosight (NS300).

**Results**: FBS contains ~1 × 10^10^ to 1.0 × 10^12^ EV particles/mL. After centrifugation, total EV counts was reduced from ~2.24 × 10^11^/mL in FBS to ~6.67 × 10^9^/mL in EDS. While exosome (30–100 nm) counts was reduced from ~1.1 × 10^11^/mL in FBS to ~5.2 × 10^9^/mL in EDS and the microvesicle (100–1000 nm) counts was reduced from ~1.1 × 10^11^/mL in FBS to ~5.2 × 10^9^/mL in EDS. Interestingly, the percentage of exosome in total EV was increased from the ~49.17% in serum to ~83.21% in EDS; while that for microvesicles in was reduced from the 50.17% in serum to 16.96% in EDS.

**Summary/Conclusion**: The EDS prepared using the gold standard method is not depleted with EV, in fact it contains ~6.67 × 10^9^/mL bovine EV. Furthermore, EDS has distorted ratio of bovine exosomes vs. microvesicles compared with FBS. Thus, the “human” EV preparation contains ~5–15% EV of bovine origin in some human EV prepared using EDS. Given that bovine EV can be non-specifically uptaken by human cells and affects cellular functions, caution should be exercised when using EDS.

**Funding**: This work was supported by Deakin University.

PF06.04

Precipitation-based EV purification from rat plasma co-precipitates part of protein-bound miRNAs

Jenni Karttunen ^1^; Mette Heiskanen^1^; Vicente Navarro-Ferrandis^1^; Kirsi Rilla^2^; Arto Koistinen^3^; Asla Pitkänen^1^

^1^AIV Institute for Molecular Sciences, University of Eastern Finland, Kuopio, Finland; ^2^Institute of Biomedicine, University of Eastern Finland, Kuopio, Finland; ^3^SIB Labs, University of Eastern Finland, Kuopio, Finland

**Background**: Plasma extracellular vesicles (EVs) and their miRNA cargo offer a source for non-invasive biomarker discovery. However, methods to isolate pure EVs from plasma are still developing, and it is important to ensure that protein-bound miRNAs, accounting 66% of plasma miRNAs, are removed during purification. Membrane particle precipitation-based EV purification is an appealing choice: the protocol is simple, the yield is high and there are compatible RNA isolation kits available. Here, we evaluated the capability of precipitation-based method to enrich EV-specific miRNAs from a small volume of rat plasma.

**Methods**: We compared the original plasma, purified EVs and remaining supernatant. Then, we performed size-exclusion chromatography (SEC) analysis on both original plasma and EV pellet. SEC-fractions were analysed using protein and miRNA concentration, droplet digital PCR for four miRNAs, and nanoparticle tracking analysis (NTA). Selected SEC fractions and precipitation purified EVs were also analysed with transmission electron microscopy (TEM).

**Results**: Precipitation-based EV purification co-precipitated 18–22% of plasma proteins and 21–99% of protein-bound miRNAs with EVs, depending on the individual miRNA. In addition, the amount of miR-142-3p, found mainly in EV-fractions, was decreased after the purification, indicating that part of it is lost during purification. Western blot and TEM showed both protein and lipoprotein contamination in the precipitation purified EVs.

**Summary/Conclusion**: Our data demonstrate that precipitation-based method is not sufficient for purification of EV-related miRNA cargo. The particle number measured with NTA is high, but mostly coming from contaminating lipoproteins. Even though a part of the protein-bound miRNA is removed, co-precipitated miRNAs together with lipoprotein-bound miRNAs still dominate the miRNA content of precipitation-based EV purification.

PF06.05

Serum-free media supplements carry miRNAs that co-purify with extracellular vesicles

Martin Auber ^1^; Hannah Mende^1^; Oliver Drechsel^2^; Eva-Maria Krämer-Albers^1^

^1^Institute of Developmental Biology and Neurobiology – Molecular Cell Biology, Johannes Gutenberg University of Mainz, Mainz, Germany; ^2^Institute of Molecular Biology gGmbH, Johannes Gutenberg University of Mainz, Mainz, Germany

**Background**: Numerous studies report the association of miRNAs with extracellular vesicles (EVs). In most cases, EVs were harvested from cell culture-conditioned media containing serum or a defined media supplement as nutrient. We are analysing miRNAs associated with EVs derived from oligodendrocytes, which deliver EV-associated cargo to neurons. Since a recent study revealed miRNAs in vesicle-depleted-foetal bovine serum medium co-isolating with EVs, we controlled media supplements routinely used for neural cell culture for the presence of miRNAs that had been identified in a RNA-Seq data set of oligodendrocyte-derived EVs.

**Methods**: We characterized the topology of RNAs associated with oligodendroglial EVs by enzymatic digests. We furthermore performed RNA-Seq of small RNAs purified from EVs isolated from primary cultured oligodendrocytes by differential centrifugation to reveal EV-enriched miRNAs. Validation of miRNAs was performed by qPCR of miRNAs isolated from purified EVs as well as un-conditioned media or the media supplements NB21 and B27 subjected to the EV-isolation protocol. To reveal potential sources of miRNAs, individual media components were assessed by qPCR.

**Results**: Enzymatic digestion of isolated EVs using RNAse and protease indicated that oligodendroglia-derived EVs contain a distinct population of small RNAs. Intriguingly, validation of miRNAs identified by RNA-Seq revealed that most EV-associated miRNAs were robustly detected in un-conditioned media and the media supplements NB21 and B27. By screening individual supplement components, we were able to exclude bovine serum albumin as major source of miRNA contamination and identified a single component as carrier of miRNAs.

**Summary/Conclusion**: Our study shows that a single component of defined media supplements may carry major contaminating miRNAs into EV samples. Hence, EV RNA-Seq data should be carefully controlled. Our study identifies the major contaminating source and may help to formulate miRNA-free media supplements in the future.

**Funding**: This work was funded by DFG.

PF06.06

Development of poreless filter for extracellular vesicles isolation and staining for prostate cancer diagnosis

Hyunwoo Shin ^1^; Hwapyeong Jeong^2^; Siwoo Cho^1^; Jingeol Lee^1^; Jaesung Park^1^

^1^POSTECH, Pohang, Republic of Korea; ^2^Pohang University of Science and Technology, Pohang, Republic of Korea

**Background**: Prostate-specific antigen (PSA) is commonly used to diagnose prostate cancer (PCa). However, PSA shows low specificity, such that benign hyperplasic conditions can also be associated with a PSA increase. To overcome this limitation of PSA, a new approach that detects cancer extracellular vesicles (EVs) has been introduced. However, clinical diagnosis using EVs to date has been limited by the lack of effective purification strategies, and time-consuming marker detecting processes. To overcome the limitations, we have developed a simple strategy employing poreless filter (PF) to isolate and detect PCa-derived vesicles. In this study, we have isolated purified EVs from patients’ plasma without a loss, and have detected prostate markers after staining the EVs using PF.

**Methods**: With the aid of the simulation, we have developed PF for an EVs isolation and staining system, which provides us with high-performance and less staining process time. After PF optimization, 10 benign hyperplasia (BPH) and 20 PCa patients were recruited, and 200 µl of each patient’s plasma was collected. The experiment was approved by the Ethics Committee of South Korea (IRB number: KC14SISI0213). EVs were isolated using existing methods (ultracentrifugation and commercial kits) and PF. PSMA (PCa protein marker) antibody staining and purification was based on PF, and we have measured the resulting expression level of PSMA.

**Results**: The PF have recovered ~100% of EVs from the plasma, whereas ultracentrifugation, precipitation-based commercial kit and filter-based commercial kit have recovered 40%, 70% and 50%, respectively. Relative impurity (EV recovery efficiency/protein recovery efficiency) of PF was lower than the others were. Antibody staining and purification based on PF have recovered almost all the stained EVs and have reduced process time to 20%. After isolating and staining EVs from the patients’ plasma by PF, we measured an expression level of PSMA. As a result, significant differences between BPH and PCa in expression levels of PSMA have been identified (*p* < 0.01).

**Summary/Conclusion**: We have developed a new EVs isolation and staining method, which is easily accessible by clinical personnel.

**Funding**: This work was supported by Korea Health Industry Development Institute grant (KHIDI) funded by the Korea government (No. HI16C0665).

PF06.07

Data-driven identification of robust extracellular vesicle subpopulation *in vitro* models from patient blood

Catherine Planey^1^; Chi-Chih Kang ^2^

^1^Mantra Bio, Inc., San Francisco, CA, USA; ^2^Mantra Bio, Inc., Berkeley, CA, USA

**Background**: Given exosomes are present in high concentrations in human blood, it is natural to use human blood samples to inform what therapeutic *in vitro* models ensure the best chance for downstream clinical translation of novel extracellular vesicle (EV) therapeutics. We compared lung cancer blood samples against *in vitro* cell culture lung cancer samples and analysed the shared RNA signalling between these two different models to characterize shared EV subpopulations.

**Methods**: We purchased retrospective samples of 1 mL of blood each from three early-stage non-small-cell lung carcinoma (NSCLC) and four non-cancer patients through a private biobank. We also prepared two replicates each from an A549 NSCLC and a HEK293 (non-cancer) epithelial human cell line culture.

We isolated EVs from the seven human blood and four cell culture samples using the ExoQuick and ExoQuick-TC systems, respectively. We then lysed the EVs and measured their internal RNA expression using RNA-seq.

Using the DESeq R package, we identified an intersecting list of shared genes that were both differentially expressed between the non-cancer and cancer human blood, and the non-cancer and cancer cell culture samples. We then evaluated the level of the proteins produced by these shared gene(s) in a publicly available EV NCI-60 cancer cell culture mass spectrometry data set.

**Results**: One gene, *IQGAP1*, was significantly underexpressed in NSCLC vs. non-cancer samples in both the human blood and cell culture data sets. When inspecting the level of the IQGAP1 protein product in the public mass spectrometry data set, a metastatic lung cancer cell line, HCI H226, had higher levels than those in A549, while other non-metastatic lung cancer cell lines such as NCI H640 and HOP 92 had lower levels, highlighting the variance of biomarkers across different lung cancer subtype and stage models.

**Summary/Conclusion**: Our work provides a preliminary framework for identifying EV *in vitro* models that mimic human disease signalling. More refined EV isolation techniques, in particular those targeting specific disease-related subpopulations, will elucidate even more concordant signal between human and *in vitro* models.

**Funding**: This research was funded by Mantra Bio, Inc.

PF06.08

Purification of extracellular vesicles from plasma by heparin-coated magnetic beads

Yiyao Huang ^1^; Dillon C. Muth^2^; Lei Zheng^3^; Kenneth W. Witwer^1^

^1^Department of Molecular and Comparative Pathobiology, Johns Hopkins University School of Medicine, Baltimore, MD, USA; ^2^The Johns Hopkins University School of Medicine, Baltimore, MD, USA; ^3^Department of Laboratory Medicine, Nanfang Hospital, Southern Medical University, Guangzhou, China (People’s Republic)

**Background**: To promote clinical and particularly biomarker applications of EVs, isolation methods are needed to obtain EVs with high quality and concentration and with a minimum of specialized equipment and hands-on time. Previously, Balaj et al. reported efficient isolation of EVs from cell culture medium using heparin-coated magnetic beads. Reasoning that this technology could be easily parallelized, we evaluated application of the method to human plasma samples.

**Methods**: Plasma from healthy human donors was concentrated and partially purified by three rounds of dilution and filtration through a 100-kDa filter. The retentate of this “pre-washed” plasma was incubated with heparin-coated magnetic beads overnight. Unbound material was removed by magnetic separation and, in some experiments, incubated with fresh beads in a second reaction round. In separate experiments, different elution buffers (high salt, Tris buffer and a commercial elution buffer) were separately added to elute EVs. Protein and particle concentrations and ratios were measured by protein assay and single particle tracking (ParticleMetrix). Morphology and specific markers of EVs were examined by transmission electron microscopy and Western blotting.

**Results**: Plasma EVs were successfully obtained through a published heparin-coated bead method. However, efficiency of capture was much lower from plasma than previously reported for cell culture-conditioned medium. Among different elution buffers to remove EVs from heparin beads, a commercial elution buffer achieved higher particle counts as compared with home-made high salt and Tris buffers. Interestingly, a second heparin bead incubation with the “unbound” plasma fraction produced a higher particle concentration and particle-to-protein ratio (purity) than the first incubation.

**Summary/Conclusion**: Heparin beads can be used for separating EVs from plasma, but only with low efficiency. We observed that a secondary incubation of unbound plasma with heparin beads led to higher EV recovery. This phenomenon may be explained by different affinities of heparin for EVs versus other biological components of plasma, which are “precleared” in the first incubation. In addition, some EVs in plasma do not seem to bind heparin.

**Funding**: The research was supported in part by the US National Institutes of Health through DA040385 and AG057430 (to KWW).

PF06.09

Optimization of a size-exclusion chromatography protocol to isolate plasma-derived extracellular vesicles for transcriptional biomarkers research

Laetitia Gaspar^1^; Magda M. Santana^1^; Rita Perfeito^1^; Patrícia Albuquerque^1^; Teresa M. Ribeiro-Rodrigues^2^; Henrique Girão^2^; Rui Nobre ^1^; Luís Pereira de Almeida^1^

^1^Center for Neuroscience and Cell Biology (CNC), University of Coimbra, Coimbra, Portugal; ^2^Institute for Biomedical Imaging and Life Sciences (IBILI), Faculty of Medicine, University of Coimbra, Coimbra, Portugal

**Background**: Size-exclusion chromatography (SEC) has been reported as an advantageous method to isolate extracellular vesicles (EVs) from plasma. When compared to other methods, SEC is faster, has a relatively low cost and requires a small amount of starting material. Here, we optimized a SEC protocol to isolate EVs from plasma for subsequent RNA transcriptional analysis of biomarker candidates.

**Methods**: EVs were isolated from human plasma using a commercially available SEC column. Sequential fractions were collected and characterized. Purity was evaluated by Ponceau and Western blot analysis; concentration and size distribution by nanoparticle tracking analysis (NTA); and total RNA profile by automated electrophoresis.

**Results**: EVs were eluted in fractions (F) 7, 8, 9 and 10, as evidenced by the presence of the EV marker Flotilin-1 and the absence of the cellular marker Calnexin, in Western blot. Plasma proteins started to elute from F11. The RNA profile of the obtained EV populations showed to be enriched in small RNAs. Based on these results, two EVs populations were characterized: one composed of EVs eluted from F7 to F9 and other with EVs eluted between F7 and F10. Both of these EV populations (F7–F9 and F7–F10) showed to be enriched in EVs with no signs of cellular contamination, as demonstrated by the presence of Flotilin-1 and the absence of Calnexin. NTA revealed higher EV concentration in F7–F10, with a bigger average size, in comparison to F7–F9. High reproducibility of the method was observed, as comparable EV purity, concentrations, sizes and RNA profiles were obtained along 12 runs.

**Summary/Conclusion**: The EVs-associated RNA profile obtained with this protocol is mainly constituted by small RNA species which along with data from Western analysis demonstrates the purity of the EVs populations and its applicability for downstream transcriptional applications. The method was fast, easy and very reproducible, showing its potential for biomarker research and for rapid translation into clinics.

**Funding**: This work was co-supported by EU-JPND project (JPCOFUND/0001/2015) and FCT (Portugal). It was also supported by FEDER through COMPETE 2020 and by FCT (CENTRO-07-ST24-FEDER-002006, POCI-01-0145-FEDER-007440, SFRH/BD/90730/2012, SFRH/BPD/66705/2009, UID/NEU/04539/2013 and 01/BIM-ESMI/2016).

PF06.10

Evaluation of the preanalytical conditions on the size, concentration and characteristics of extracellular vesicles isolated from serum, EDTA- and citrated plasmas

Anne Marie Siebke. Trøseid ^1^; Trude Aspelin^1^; Lilly Alice. Steffensen^1^; Tonje Bjørnetrø^2^; Beate Vestad^3^; Eduarda M Guerreiro^4^; Kari Bente Foss Haug^1^; Reidun Øvstebø^1^

^1^The Blood Cell Research Group, Department of Medical Biochemistry, Oslo University Hospital, Norway, Oslo, Norway; ^2^Department of Oncology, Akershus University Hospital, Oslo, Norway; ^3^Research Institute of Internal Medicine, Oslo University Hospital Rikshospitalet, Oslo, Norway; ^4^Department of Oral Biology, University of Oslo, Oslo, Norway

**Background**: Blood contains huge amounts of membrane-embedded extracellular vesicles (EVs) released from different cells. Depending on their biogenesis, EVs comprise a heterogeneous group of vesicles. They can be seen as mini-maps of their cells of origin with both physiologic and pathologic relevance. EV size, concentration and composition may give important clinical information, and the potential of EVs from blood for diagnosis and treatment is being investigated. However, the effects of using plasma or serum as well as preanalytical conditions, such as choice of anticoagulant and centrifugation procedures, need to be settled.

**Methods**: Blood samples from consenting, fasting, healthy donors (*n* = 3) were sampled into tubes containing K2-EDTA, Na-citrate, barrier gel or no additive. Tubes were centrifuged at 2500 x*g*, 15 min after 45 min respite. Plasma and serum were immediately pipetted off and either stored in aliquotes at −80°C or re-centrifuged at 2500 x*g*, 15 min and stored at −80°C.

EVs were isolated from 500 µl plasma/serum using size-exclusion chromatography (SEC), collected in pooled joint fractions (F7–10) and concentrated 2:1 (centrifugal evaporation), before the size and concentration were analysed using nanoparticle tracking analysis. CD9+ and CD61+ EVs were captured by specific antibody-coated magnetic beads and analysed by flow cytometry (CD9 and CD61) and Western blot (CD9 and TSG101). The presence of EVs was confirmed by transmission electron microscopy.

**Results**: Overall, the mean sizes of vesicles ranged from 101 to 106 nm and the mean concentrations varied from 1.54 10E11 to 1.94 × 10E11/mL in joint fraction with no significant differences between serum and plasmas centrifuged ones. The concentration of EVs isolated from EDTA plasma centrifuged once differed significantly (*p* = 0.036) from plasma centrifuged twice. All samples analysed contained CD9-, CD61- and CD63-positive EVs. Serum levels of CD9+ and CD9+/CD61+ EVs (flow cytometry) and CD63+/CD9+ (Western blot) showed a tendency to be higher than equivalent EVs isolated from plasmas.

**Summary/Conclusion**: Despite the small sample size, our NTA-based results so far indicate that EVs isolated from serum or plasma by SEC contain similar levels of EVs, whereas the yield of isolated CD9+EVs isolated from serum is higher than in isolates from plasma.

**Funding**: This work was funded by Oslo University Hospital.

PF06.11

Isolation of extracellular vesicles from human plasma using a novel three-step protocol

Xiaogang Zhang; Ellen Borg; Willem Stoorvogel

Department of Biochemistry & Cell Biology, Faculty of Veterinary Medicine, Utrecht University, Utrecht, The Netherlands

**Background**: Several methods have been applied to isolate extracellular vesicles (EVs) from human plasma, including differential (ultra)centrifugation, density gradient centrifugation, ultrafiltration, size-exclusion chromatography (SEC) and polymer-based precipitation. In plasma, however, the abundance of extracellular vesicles is very low relative to other particulate constituents with comparable size and/or buoyant densities, including lipoprotein particles and protein complexes. Until now, EV isolation to homogeneity remains a problem. We here describe a novel three-step isolation method to purify EVs from human plasma.

**Methods**: Fresh blood was collected using citrate carrying anticoagulant tubes. Cells, platelets and large microvesicles were removed from human blood by differential centrifugation. EVs were then precipitated using polyethylene glycol (PEG). Pelleted EVs were resuspended and separated from co-precipitated lipoprotein particles and protein complexes by upward displacement into a linear Nycodenz density gradient. Finally, EV carrying fractions were applied onto a Sepharose CL-2B column for SEC.

**Results**: As compared to ultracentrifugation, EVs were more efficiently precipitated from human plasma using PEG. However, PEG-precipitated EVs were highly contaminated with low density lipoprotein particles, high density lipoprotein particles (HDL), and non-EV-associated protein (complexes). EVs were efficiently separated from these contaminants by subsequent fractionation on Nycodenz density gradients. However, some HDL contaminants remained, which could be removed in the third step using SEC.

**Summary/Conclusion**: These data indicate that subsequent isolation steps are required to isolate EVs to homogeneity from plasma. Single-step isolation methods may result in gross overestimation in the amount of EV-associated protein or misinterpretation of EV molecular compositions.

**Funding**: Xiaogang Zhang is the recipient of a doctoral scholarship from China Scholarship Council.

PF06.12

Efficient isolation of extracellular vesicles from blood plasma based on iodixanol density gradient ultracentrifugation combined with bind-elute chromatography

Gábor Brenner^1^; Zsófia Onódi ^1^; Csilla Terézia Nagy^1^; Ágnes Kittel^2^; Mateja Manček Keber^3^; Zoltán Giricz^1^

^1^Department of Pharmacology, Semmelweis University, Budapest, Hungary; ^2^Institute of Experimental Medicine, Hungarian Academy of Sciences, Budapest, Hungary; ^3^National Insitute of Chemistry, Ljubljana, Slovenia

**Background**: Blood-derived extracellular vesicles (EVs) are extensively investigated both as biomarkers and therapeutics. However, efficient isolation of EVs from a limited amount of sample is a great challenge. Thus, the aim of this study was to identify a method to isolate the majority of EVs from blood plasma, while eliminating impurities such as lipoprotein particles and soluble proteins.

**Methods**: Rat and human blood samples underwent low-speed centrifugations to remove cells, debris and large particles without prior filtration. Density gradient ultracentrifugation (DGUC) was performed by layering 50%, 30% and 10% iodixanol solutions on top of which sample was loaded and centrifuged at 120,000 ×*g* for 24 h. Fractions were collected from top to bottom. Fractions with the highest EV content were further purified by ultracentrifugation or size exclusion chromatography. Efficiency and purity were assessed by Western blot. Morphology and size distribution of particles were examined by dynamic light scattering (DLS) and electron microscopy (EM).

**Results**: Highest band intensities of EV markers Alix and Tsg101 were detected (60% and 59%, respectively) at a density of 1.13–1.17 g/mL. The presence of EVs was confirmed by EM and DLS, showing particles with a mean diameter of 38 ± 2 nm. By DGUC, 95% of lipoprotein- and 84% of albumin contamination were separated from EV-containing fractions. However, 67% of the total fibrinogen content was present in EV-rich fractions, indicating the need for further purification. After loading 1.3-mL EV-rich fractions of DGUC on HiScreen Capto Core 700 column, the majority of Tsg101 signal was observed in 2 mL eluate in which albumin was not detectable, while the amount of fibrinogen decreased but was not completely removed from EV-rich eluate.

**Summary/Conclusion**: DGUC with iodixanol shows higher efficiency than generally used methods for the isolation of blood-derived exosomes. It separates EVs from the majority of vesicle-like lipoproteins, and reduces the amount of contaminating soluble proteins. Further purification of EV-rich DGUC fractions by chromatography on Capto Core 700 column yields amounts of EVs significantly higher than currently described methods with less contamination by non-EV plasma components.

**Funding**: The project was funded by NKFIH NVKP 16-1-2016-0017. ZG Holds a Bolyai Fellowship from the Hungarian Academy of Sciences.

PF06.13

A novel two-step EV isolation from plasma using size-exclusion chromatography and antibody-mediated removal of lipoproteins

Anders Askeland ^1^; Jonas E. Nielsen^1^; Gunna Christiansen^2^; Aase Handberg^1^; Søren R. Kristensen^1^; Shona Pedersen^1^

^1^Department of Clinical Biochemistry, Aalborg University Hospital, Aalborg, Denmark; ^2^Department of Biomedicine, University of Aarhus, Aarhus, Denmark

**Background**: Owing to extracellular vesicles (EVs) ubiquitous distribution within tissues and bio-fluids, EV isolation is an essential part of all EV research. Unfortunately, EV isolation remains a challenging task, especially when isolating EVs from complex bio-fluids such as plasma. The biggest challenge is the co-isolation of non-EV proteins and lipoproteins, both of which are abundantly present in plasma. In an attempt to understand these challenges, our group has previously examined several commonly used EV isolation methods for plasma, where we demonstrated that EV isolates obtained by size-exclusion chromatography (SEC) contained minimal levels of non-EV proteins, however, high levels of lipoproteins. Recently, our research group has also showed that lipoproteins can be removed from plasma by antibody-mediated removal. Based on these findings, the aim of this study was to evaluate a novel two-step EV isolation by SEC and subsequent lipoprotein removal, for an ultra-pure EV isolate.

**Methods**: EV isolation will be performed in five replicates from a single plasma pool collected from healthy donors. Briefly explained, EVs are first isolated from platelet poor plasma using commercially available qEV Original SEC columns. The resulting EV fractions are incubated with magnetic beads conjugated with antibodies against apolipoprotein B-48 and B-100 (ApoB). After incubation, the magnetic beads and lipoproteins are removed, leaving a final EV isolate. For comparison, the procedure is performed both with and without lipoprotein removal. The isolated EVs will be characterized using transmission electron microscopy with CD9 immunoblotting, nanoparticle tracking analysis and Western blotting against CD9 and ApoB.

**Results**: This two-step EV isolation should mitigate the current limitation of SEC when used on plasma, where we previously found that EV isolates produced by SEC have a significantly higher lipoprotein- and lower non-EV protein content compared to conventional ultracentrifugation (unpublished). Potentially, this novel method could result in the generation of an ultra-pure EV isolation with minimal co-isolation of non-EV components.

**Summary/Conclusion**: If successful, this EV isolate would allow for greatly improved plasma EV characterization, a process that has previously been difficult due to varying degrees of non-EV contamination.

PF06.14

Isolation of bone marrow extracellular vesicles for *in vivo* studies in mice

Eszter Persa ^1^; Tunde Szatmari^1^; Katalin Lumniczky^2^; Livia N. Naszalyi^3^; Munira Kadhim^4^; Géza Sáfrány^5^

^1^National Public Health Institute, Budapest, Hungary; ^2^Division of Radiation Medicine, National Public Health Center – National Research Directorate for Radiobiology and Radiohygiene, Budapest, Hungary; ^3^Hungarian Academy of Sciences, Budapest, Hungary; ^4^Oxford Brookes University, Oxford, UK; ^5^Division of Molecular Radiobiology, National Public Health Center National Research Directorate for Radiobiology and Radiohygiene, Budapest, Hungary

**Background**: Extracellular vesicles (EVs) are membrane-derived particles actively released by cells. Due to their complex cargo, consisting of proteins, lipids, RNAs and miRNAs, EVs play important roles in intercellular communication even between distant cells. *In vivo* approaches using animal models can help to better understand the exact mechanism of EV release, distribution between donor and recipient cells and the signalling processes regulated EVs and their cargo. Our goal was to work out a good method for isolation of bone marrow (BM)-derived EVs from mice.

**Methods**: C57Bl/6 and CBA/H mice of different age were used. BM was flushed and cell supernatant was used for further EV isolation. Four different methods were tried: ultracentrifugation (UC) and three kits for EV isolation, Exoquick TC (EQ), miRCURY and qEV columns. The amount of EVs was determined based on protein content and measured by Coomassie assay. Dynamic light scattering was used to determine size distribution of the samples. EVs were visualized by electronmicroscopy (EM) and characterized by Western blotting with EV-specific (TSG101 and CD9) and non-EV-specific (calnexin) proteins and by flow cytometry. EV samples isolated with EQ were further purified using G-25 spin column.

**Results**: There was no difference regarding EV amount and phenotype between young and older animals. EVs isolated by UC were more homogenous in size compared to the other methods. EQ-prepared EVs rendered EVs in a size range comparable to those isolated by UC, but later fractions rendered EVs with increasing diameters. EQ and UC offered the largest amount of EVs. EV samples isolated by MiRCURY and qEV contained more calnexin than EVs isolated by EQ.

**Summary/Conclusion**: BM-derived EVs could be isolated using any of the above-mentioned methods. Based on adequate amount and purity of samples, UC and EQ kit resulted in comparable EV parameters both in terms of purity and amount. Thus, both methods are suitable for isolating BM-derived EVs directly from mice. However, one should take into account the fact that UC isolation needs much more work than EQ method.

**Funding**: This work was funded by the DoReMi FP7 project (249689), the Euratom research and training programme 2014–2018 (CONCERT, 662287) and a Hungarian research grant funded by the National Research, Development and Innovation Office (VKSZ_14-1-2015-0021).

PF06.15

Isolation of blood-derived exosomes by dual size-exclusion chromatography

Jik Han Jung ^1^; Junyong Yoon^2^; Ji-Ho Park^1^

^1^Department of Bio and Brain Engineering, Korea Advanced Institute of Science and Technology, Daejeon, Republic of Korea; ^2^Korea Advanced Institute of Science and Technology, Daejeon, Republic of Korea

**Background**: Recently, various exosome isolation methods have been developed for studying of exosomes. However, phygiological sources such as serum and plasma are still challenging, in the aspect of purity. This is because these blood samples contain large quantities of lipoproteins and soluble proteins. Although numerous methods of eliminating these contaminants have been developed, they are time-consuming and require complexible steps for isolation. Thus, we introduce a rapid and simple method which is composed of dual size-exclusion chromatography (SEC).

**Methods**: Human blood samples were kindly provided by “Korea University Anam Hospital”. Column was packed with a total volume of 10 ml; the compositions included one resin which interacts with molecules lower than 5000 kDa, and the other which interacts with molecules lower than 500 kDa in order to prepare SEC column. Then, 0.5 ml of the sample was loaded on the top of the column, and each 0. 5 ml eluate was collected. All samples were analysed by absorbance at 280 nm, bicinchoninic acid assay, dynamic lighting scattering (DLS), sodium dodecyl sulfate polyacrylamide gel electrophoresis, Western blot, transmission electron microscopy and nanoparticle tracking analysis.

**Results**: In the case of the developed dual SEC, CD63 was detected in fractions 10–15. Apolipoprotein B (ApoB) was detected in fractions 9–11 and soluble proteins were intensively detected in fractions 13–15. The collected fractions of 10–12 of the dual column showed ≥50 times higher density of CD63 and ApoB, when compared to the commercially available kits.

**Summary/Conclusion**: In this work, we studied the size distribution of exosomes, lipoproteins and soluble proteins using dual SEC. Based on the principle of SEC, we designed a dual column system for eliminating lipoproteins and soluble protein in one step. Also, the purified exosomes showed higher purity compared to those purified with commecialized kits, by focusing on removing of lipoproteins and soluble proteins.

**Funding**: This research was supported by a grant of the Korea Health Technology R&D Project through the Korea Health Industry Development Institute (KHIDI), funded by the Ministry of Health and Welfare, Republic of Korea (HI14C3477).

PF06.16

Plasma nanostructuring of the tools increases the yield of extracellular vesicles in blood isolates

Roman Stukelj ^1^; Matic Resnik^2^; Manca Pajnic^1^; Vid Šuštar^3^; Henry Hägerstrand^4^; Ita Junkar^2^; Miran Mozetič^2^; Veronika Kralj-Iglic^1^

^1^Laboratory of Clinical Biophysics, Faculty of Health Sciences, University of Ljubljana, Slovenia, Semic, Slovenia; ^2^Jozef Stefan Institute, Ljubljana, Slovenia; ^3^Lymphocyte Cytoskeleton Group, Institute of Biomedicine/Pathology, BioCity, University of Turku, Turku, Finland; ^4^Faculty of Science and Engineering, BioCity, Åbo Akademi University, Turku, Finland

**Background**: Isolation of extracellular vesicles (EVs) still represents a major drawback due to a simple fact that EVs are likely to interact with surfaces of medical tools, especially surfaces of the polypropylene tubes used in the isolation process. In order to diminish or prevent the adsorption of the EVs on the surface of polypropylene tubes, we elaborated the surface with gaseous plasma. We expected to obtain higher concentration of EVs in the isolates as less material was expected to adhere to the surface.

**Methods**: For the preparation of samples, the atmospheric pressure plasma jet with a single electrode and argon as feed gas was used for treatment of inner tube walls. We used standardized polypropylene 1.5-mL conical transparent tubes with a snap-cap from three different manufacturers with differences in chemical composition, morphology and water contact angle. EVs were isolated from 3 ml of whole blood from healthy fasting donors, by repetitive centrifugation (up to 17570 *g*) and washing by phosphate buffered and citrated saline. EVs were counted by flow cytometry.

**Results**: The concentration of EVs in plasma-treated tubes was on the average higher (for 36%, *p* = 0.003) compared to the untreated ones. Significant differences in EVs yield were observed at the same gaseous plasma conditions between tubes from different manufacturers (*p* corresponding to all differences <0.05). The increase was 24%, 35% and 48%.

**Summary/Conclusion**: Results from flow cytometry indicate that the isolation yields of EVs are higher when gaseous plasma-treated tubes are used, mainly due to altered surface nano-topography and chemistry.

**Funding**: Authors would like to acknowledge the Slovenian Research Agency (ARRS) grant P3-0388, J5-7098, J2-8166, L7-7566 and for funding of the Young researcher grant PR-06154.

PF06.17

Extraction and analysis of intact EVs collected from dried blood spots

Malene M. Jørgensen; Rikke Baek; Kim Varming

Department of Clinical Immunology, Aalborg University Hospital, Aalborg, Denmark

**Background**: Venous blood is a convenient source of circulating extracellular vesicles (EVs). However, blood sampling requires authorized personnel and immediate purification of the vesicles. The present study demonstrate that intact EVs can in fact be obtained from dried blood card samples, which can be prepared by unauthorized personnel, or even at home by the user and shipped by regular mail. Intact EVs can be detected in extracts from dried blood spot samples even after prolonged storage.

**Methods**: In the first experiment, venous peripheral blood (EDTA, CPDA, heparin and serum) was drawn from three healthy donors and compared with blood obtained using a lancet from their fingers. The blood drops thoroughly saturated the paper (blood card specially designed for whole cells) and was allowed to air-dry before storage and analysis. The extraction procedures were optimized and eight different buffer compositions were tested.

In the second experiment, blood samples from 20 healthy donors were used to test the effect of prolonged storage of the dried blood cards. The most optimal extraction procedure found in the first experiment was used to compare the EV contents after 1 h, 7, 14 and 21 days after collection.

To evaluate the EV concentration and composition, the samples were analysed by the EV Array using 15 selected surface-markers.

**Results**: Elution of EVs from dried blood spots was found to be possible when a soaking procedure was used and followed by a short centrifugation. During the centrifugation, the EVs were collected in a specific sample buffer. The composition of the buffers was found to be very important for the outcome of the extraction.

The qualitative tests revealed that, for most of the markers (11 out of 15), the samples from dried blood spots showed similar results as for blood drawn using EDTA or CPDA. After 21 days of storage at room temperature, a higher degree of haemolysis was observed in the extracted samples. The increase in free haemoglobin generated a higher background signal, but the samples were still acceptable for analysis with the EV Array.

**Summary/Conclusion**: The direct use of EVs for disease diagnosis has been limited by the current lack of methods to purify, measure and characterize these. This study has shown that dried blood cards can be used to collect EVs prior to analysis using a protein microarray-based technology.

PF06.18

Comparing extracellular vesicle enrichment methods for use on small sample volumes: how low can we go?

Bianca Paris; David R F. Carter; Ryan C. Pink

Oxford Brookes University, Oxford, UK

**Background**: Extracellular vesicles (EVs) are abundant in body fluids and can be obtained by minimally invasive biopsy as useful diagnostic biomarkers. In many clinical and research settings, initial sample volume is limited, especially when biobank storage is concerned. Therefore, to facilitate precise discovery or diagnosis, EVs should be isolated with high yield and purity, and incur minimal damage in the process. Achieving this is heavily influenced by the experimental conditions and methodology used; therefore, the present study aims to compare EV yield and purity when performing several common enrichment methods on small volumes of human plasma.

**Methods**: Human whole blood samples were processed by differential centrifugation to obtain platelet-free plasma. EVs were enriched from decreasingly small aliquots of platelet-free plasma (1 mL–100 µL) by size-exclusion chromatography (SEC), ultracentrifugation and polymer precipitation. Following each technique, EV number was measured by nanoparticle tracking analysis and co-isolation of contaminant particles was assessed by bicinchoninic acid assay, transmission electron microscopy and sodium dodecyl sulfate polyacrylamide gel electrophoresis. Total protein was extracted and quantified as an additional measure of yield for downstream proteomic applications.

**Results**: SEC achieved a high EV recovery efficiency compared to ultracentrifugation, and resulted in high numbers of EVs even from very small volumes of plasma. Minimal co-isolation of contaminant particles was observed in SEC-enriched EVs compared to both ultracentrifugation and polymer precipitation techniques.

**Summary/Conclusion**: These findings suggest that SEC is the preferred method to reduce co-isolation of contaminants when enriching EVs from complex substrates such as body fluids. SEC is also a good candidate for obtaining sufficient EVs for practical downstream applications when sample volumes are limited, as is the case in many clinical and research contexts.

PF06.19

An optimized workflow for the isolation and purification of extracellular vesicles from small serum volumes

Kieran Brennan ^1^; Margaret McGee ^1^; Kenneth Martin^2^; Ciaran Richardson^2^

^1^University College Dublin, Dublin, Ireland; ^2^Randox Teoranta, Dungloe, Ireland

**Background**: Extracellular vesicles (EVs) are nanometre-scale, membrane-enclosed vesicles that are released from a multitude of cell types and mediate intercellular communication via the transfer of proteins, small RNAs and mRNAs to recipient cells. EVs have gained a lot of interest in the past few years as a source of cancer biomarkers with both diagnostic and prognostic value. A blood-based cancer screening test is appealing because the specimens can be obtained readily in a non-invasive manner, and poses minimal risk to patients. EVs as a source of blood-based biomarkers present a considerable challenge due to a combination of small sample size, serum viscosity and difficulties in separating EVs from serum proteins and lipoproteins.

**Methods**: In this study, we evaluate particle yield and purity using four isolation methods: differential ultracentrifugation, polymer-based precipitation, size-exclusion chromatography and iodixanol density gradient centrifugation, on their own and in combination, for the isolation of EVs from 100 to 250 µl of human serum. Comprehensive characterization of EV yield and protein content was performed by nanoparticle tracking analysis and Bradford assay following TCA protein precipitation respectively. Furthermore, the relative abundance of EV markers, CD63 and TSG101, and lipoprotein markers, APOB, APOA1 and APOE, was determined by Western blot analysis for each method.

**Results**: Our results demonstrate that polymer-based precipitation recovered the highest number of EVs, while providing the least pure preparations of exosomes. Iodixanol density gradient centrifugation and size-exclusion chromatography provided the best EV/protein ratio by nanoparticle tracking analysis and Bradford assay. Based on Western blotting, we found that the size-exclusion chromatography was superior in isolating EVs devoid of high density lipoprotein.

**Summary/Conclusion**: Our data reveal that a combination of isolation methods is necessary for adequate separation of soluble proteins and lipoproteins from serum EVs.

**Funding**: This work was supported by the Irish Research Council in partnership with Randox Teoranta [grant number EPSPD/2015/45].

PF06.20

microRNA expression profile in microvesicles released from genistein-treated immune cells

Lucia Gimeno-Mallench ^1^; Cristina Mas-Bargues^1^; Jorge Sanz-Ros^1^; Marta Inglés^2^; Eva Serna^1^; Mar Dromant^1^; Consuelo Borrás^1^; Juan Gambini^1^; Jose Viña^1^

^1^Freshage Research Group – Department of Physiology-University of Valencia, CIBERFES, INCLIVA, Valencia, Spain; ^2^Freshage Research Group – Department of physiotherapy-University of Valencia, CIBERFES, INCLIVA, Valencia, Spain

**Background**: Intercellular communication is an essential hallmark of multicellular organisms. The nutrients we ingest from food are in contact with immune cells in the bloodstream and can promote the formation of microvesicles (MVs). Some foods contain molecules with regulatory activity, such as genistein, a natural polyphenol found in soy. We aimed to study the microRNA expression profile of MVs released from genistein-treated immune cells.

**Methods**: For this purpose, we collected blood samples from five women (aged 18–25 years) in vacutainers, and obtained peripheral blood mononuclear cells (PBMCs) by centrifugation. The cells were further cultured and treated with 0.5 μM genistein and 0.01% dimethyl sulfoxide as a control. After 48 h, the MVs were isolated by ultracentrifugation. Total RNA containing small RNAs was isolated using a total exosome RNA and protein isolation kit (Invitrogen) according to manufacturer’s directions. MicroRNA expression profile was determined by using the Genechip miRNA 4.0 Array, and subsequently analysed by principal component analysis.

**Results**: We observed that microRNA expression profile of MVs isolated from genistein-treated PBMCs was different to that of the MVs isolated from control PBMCs.

**Summary/Conclusion**: We suggest that this particular microRNA expression profile induced by genistein may be involved in the systemic beneficial effects of this molecule.

**Funding**: This work was supported by the following grants: SAF2010-19498, SAF2013-44663-R, ISCIII2006-RED13-027, ISCIII2012-RED-43-029, CIBERFES (ISCIII2016-CIBER); PROMETEO2010/074, PROMETEOII2014/056, ACIF2014/165, RS2012-609; CM1001 and FRAILOMIC-HEALTH.2012.2.1.

EVs in Diseases of the Nervous System Chairs: Eva Maria Albers; Tine Hiorth Schøyen Location: Exhibit Hall 17:15–18:30

PF07.01

Extracellular vesicles as part of the search for Alzheimer’s disease blood-based biomarkers

Jessica Wahlgren; Kina Höglund; Henrik Zetterberg; Kaj Blennow

Department of Psychiatry and Neurochemistry, Institute of Neuroscience and Physiology, University of Gothenburg, Mölndal, Sweden

**Background**: To support the clinical diagnosis of Alzheimer’s disease (AD), there is a need for blood-based biomarkers to facilitate sampling and analysis. Several obstacles need to be overcome including development of sensitive methods and evaluation of pre-analytical factors. Here we investigate the potential use of extracellular vesicles from blood as biomarkers to improve the diagnostic utility of already established cerebrospinal fluid (CSF) AD biomarkers in blood and to thereby improve the diagnosis of AD at an early stage.

**Methods**: Extracellular vesicles were isolated from paired plasma and serum samples using an established immunoprecipitation method enriching for neural cell adhesion molecules (L1CAM) by capturing positive vesicles on L1CAM-coated beads. Quantification and size determination of extracellular vesicles was performed using nanoparticle tracking analysis (NTA). Detection of exosome and AD marker proteins was done using Western blot and ELISA. Comparative studies between AD and controls using exosomes isolated from paired serum and plasma samples were performed using ELISA kit for total tau, phosphorylated tau and amyloid beta protein.

**Results**: L1CAM-positive vesicles from both serum and plasma were positive for amyloid beta and tau, including phosphorylated tau protein. There were no significant differences between AD and control in serum for any of the AD markers. However, in plasma a small difference was detected for total and phosphorylated tau. Negative control beads, i.e. not coated with antibody yielded no positive signal. Interestingly, NTA showed particles of considerable amounts present in these isolates.

**Summary/Conclusion**: There is an L1CAM-positive subpopulation of extracellular vesicles in the blood from AD as well as healthy control subjects. Unspecific binding of extracellular vesicles that are not L1CAM positive to the streptavidin-coated resin beads seems to occur of similar count as beads incubated with EVs stained with L1CAM antibody. All three established CSF biomarkers in AD were detectable with ELISA, but no differences between AD and controls were seen in exosome isolates from serum. However, a modest difference was observed in exosome isolates from plasma for total tau and phosphorylated tau.

PF07.02

Processing of the amyloid precursor protein in the exosomal pathway: propagation of Alzheimer’s disease pathology

Rocio Perez-Gonzalez ^1^; Efrat Levy^2^

^1^Center for Dementia Research, Nathan S. Kline Institute for Psychiatric Research, Orangeburg, NY, USA; ^2^Departments of Psychiatry, Biochemistry & Molecular Pharmacology, and the Neuroscience Institute, NYU Langone Medical Center, New York, NY, USA

**Background**: The main component of the amyloid deposited in the brain of Alzheimer’s disease patients is β-amyloid (Aβ), a proteolytic product of the amyloid β precursor protein (APP). Mature APP undergoes proteolytic cleavage by α- and β-secretases to produce C-terminal fragments (APP-CTFs). β-APP-CTF is a neurotoxic protein that is also the source of Aβ following cleavage by γ-secretase. It was previously shown that amyloidogenic APP processing mainly occurs in endosomes and that exosomes contain APP, APP-CTFs, a minute fraction of Aβ, and the secretases involved in APP metabolism, but the exosomal contribution to amyloid pathology remains unknown. We have investigated whether APP processing occurs in the exosomal pathway.

**Methods**: Exosomes were isolated from postmortem human and mouse brains, and from the culture media of human fibroblasts and of the neuroblastoma cell line SH-SY5Y. The content of APP, APP metabolites and APP secretases in exosomes was analysed by Western blot and compared with the content in the brain or cell homogenates.

**Results**: We found that exosomes isolated from human and mouse brains as well as exosomes secreted by cells *in vitro* are enriched in APP-CTFs. All three APP secretases were detected in the exosome preparations and interestingly, β-secretase 1 (BACE1) and the mature form of the -secretase ADAM10 were also enriched in exosomes, whereas the γ-secretase subunit Nicastrin was not. Our data also show that exosomal β- and α- secretases are active, based on the observation of continuous generation of APP-CTFs in isolated exosomes.

**Summary/Conclusion**: Our data show that APP processing continues in exosomes following their release into the extracellular space from the endosomal multivesicular bodies, implicating exosomes as carriers and generation sites of the neurotoxic β-APP-CTF and an extracellular source of Aβ. Given the stability of exosomes, this may propagate amyloid pathogenicity throughout the brain.

**Funding**: This work was supported by the NIH (P01 AG017617 and R01 AG057517) and the Alzheimer’s Association (NIRG-14-316622).

PF07.03

To study anti-tau antibody loading and neuronal uptake efficiency of human bone marrow mesenchymal stem cells-derived extracellular vesicles

Azadeh Amini ^1^; Hamid Akbari Javar^2^; Faezeh Shekari^3^; Koorosh Shahpasand^3^; Hossein Baharvand^3^

^1^Department of Pharmaceutical Biomaterials and Medical Biomaterial Research Center, Faculty of Pharmacy, Tehran University of Medical Sciences, Tehran, Iran; ^2^Department of Pharmacutics, Faculty of Pharmacy, Tehran University of Medical Sciences, Tehran, Iran; ^3^Department of Stem Cells and Developmental Biology, Cell Science Research Center, Royan Institute for Stem Cell Biology and Technology, Tehran, Iran

**Background**: Despite significant progress in drug delivery issue, efficient central nervous system (CNS) delivery of neuro therapeutics remains challenging. Extracellular vesicles (EVs), part of normal cell-to-cell communication, were introduced recently as a transporter that can overcome biological barriers against CNS delivery. So these natural nanoliposomes are promising tools for delivery systems design, especially for CNS. In present study, we examine the efficiency of mesenchymal stem cell (MSC)-derived EVs for drug loading and neuronal uptake.

**Methods**: We isolated human bone marrow-derived mesenchymal stem cells (hBMSCs)-EVs by differential ultracentrifugation coupled to density gradient technique. The protein content of harvested vesicles was measured using a BCA Protein Assay Kit. Then vesicles were characterized by performing dynamic light scattering, transmission electron microscopy and Western blotting. We examined different drug loading methods (incubation, freeze and thaw and sonication) and comprise the loading efficiency using an ELISA procedure. Neuronal uptake of vesicles also was studied using PKH-26-labeled vesicles.

**Results**: We isolated the 114-nm size vesicles from the hBMSCs condition media that presented EV marker protein. Quantification using BCA Protein Assay revealed 30 × 10^6^ hBMSCs could produce approximately 4000 µg extracellular vesicles. The results disclosed EVs loaded a significant amount of anti-tau antibody and neurons can uptake this loaded vesicles.

**Summary/Conclusion**: In our study, we designed a drug delivery method that can be used as a brain delivery system. So we loaded anti-tau antibody into hBMSC-EVs and then studied the neuronal uptake of these systems efficiently. The results disclosed EVs loaded a significant amount of anti-tau antibody and neurons can uptake this loaded vesicles.

PF07.04

*In vitro* and *in vivo* effects of plant ceramide to increase exosomes capable of eliminating Alzheimer’s amyloid-ß

Kohei Yuyama ^1^; Kaori Takahashi^2^; Katsuyuki Mukai^3^; Yasuyuki Igarashi^1^

^1^Hokkaido University, Sapporo, Japan; ^2^Daicel Corporation, Sapporo, Japan; ^3^Daicel Corporation, Minato-ku, Japan

**Background**: Accumulation of amyloid-ß protein (Aß) in human brain is early pathogenesis of Alzheimer’s disease (AD). We have previously reported the function of neuron-derived exosomes to promote Aß clearance. Neuronal exosomes trap Aß through their surface glycolipids and transport Aß into microglia to degrade. It is known that in certain group of cells, exosomes are produced in ceramide (Cer)-dependent mechanism. In this study, we found exogenous treatment with Cer, which is extracted from plant (*Amorphophallus konjac*), can increase exosome production in neurons and decrease Aß in cell culture systems and AD model animals.

**Methods**: Neuronal SH-SY5Y cells were treated with konjac Cer (mainly constituted of d18:2 sphingoid bases) for 24 h and then the exosomes in the medium were measured. To study the effect of Cer on Aß clearance, we regulated exosome secretion by Cer treatment in transwell cultures, which consists of SH-SY5Y and microglial BV-2 cells, and then measured extracellular Aß human APP transgenic mice were used as AD model animals. Konjac glucosylceramide (GluCer) of 1 mg/day was orally administered into the mice for 14 days. After the treatment, NCAM1, a neuronal marker, -positive exosomes in serum and Aß levels in brain were measured.

**Results**: We found that secretion of neuronal exosomes was promoted by Cer addition. In transwell study, upregulation of exosome production by Cer enhanced Aß uptake into microglia and significantly decreased extracellular Aß. Oral administration of GluCer into the mice resulted in marked reductions in Aß levels and amyloid depositions in the hippocampus. Additionally, after the GluCer treatment, NCAM1 and Aß in serum exosomes were increased.

**Summary/Conclusion**: Our present data suggest that plant Cer decreases extracellular Aß through promoting exosome-dependent Aß clearance *in vitro* and *in vivo*. Plant Cer may be used as a functional food material to alleviate AD pathology.

PF07.05

Raman spectroscopy for the molecular profiling of extracellular vesicles in Parkison’s disease

Alice Gualerzi ^1^; Silvia Picciolini^1^; Andrea Sguassero^1^; Federica Terenzi^2^; Furio Gramatica^1^; Sandro Sorbi^3^; Marzia Bedoni^1^

^1^Laboratory of Nanomedicine and Clinical Biophotonics (LABION), IRCCS Fondazione Don Carlo Gnocchi, Milan, Italy; ^2^Dipartimento di Neuroscienze, Psicologia, Area de Farmaco e Salute del Bambino, Università di Firenze, Florence, Italy; ^3^IRCCS Don Carlo Gnocchi, Fondazione Don Carlo Gnocchi, Florence, Italy

**Background**: Parkinson’s disease (PD) is an age-related neurodegenerative pathology characterized by progressive movement disorders and by intraneuronal accumulation of misfolded α-synuclein. As for most neurodegenerative diseases in the early diagnosis, the monitoring and the evaluation of the rehabilitation outcome are difficult to be objectively assessed, but mainly rely on clinical scaling. Circulating extracellular vesicles (EVs) deriving from all body organs can provide an overview of the patient’s clinical status and of the disease progression that might be related to the prognosis and the rehabilitation outcome.

**Methods**: Serum EVs were isolated by size-exclusion chromatography and ultracentrifugation. Then, Raman analysis was performed in order to obtain a snapshot of the EV biochemical profile. Following the label-free biophotonic procedure previously optimized in our laboratory, spectra were obtained taking advantage of a Raman microspectroscope (Aramis, Horiba) operating with a 532-nm laser beam in the spectral ranges 600–1800 and 2600–3200 cm^−1^. Multivariate statistical analysis was applied for the comparison of the Raman fingerprints from healthy subjects and PD patients.

**Results**: The preliminary results of our pilot study demonstrated the ability of the proposed method to highlight the differences in the biochemical composition of EVs from human serum. In particular, we demonstrated the presence in the serum of PD patients of EVs associated or loaded with atypical cargoes compared to age- and sex-matched healthy controls.

**Summary/Conclusion**: Although preliminary, our data provide support to the already proposed prion hypothesis of PD pathogenesis. Moreover, our data suggest the possibility to evaluate the spectrum of circulating EV populations as a whole, using the Raman fingerprint as a biomarker, complementary to specific molecular markers.

**Funding**: The present study was supported by the Italian Ministry of Health (Ricerca Corrente 2017, IRCCS Fondazione DOn Carlo Gnocchi).

PF07.06

Neutral Sphingomyelinase 2 influences transfer of oligomeric alpha-synuclein through exosomal pathway and its relation to oxidative stress

Valerie Sackmann; Maitrayee Sardar Sinha; Livia Civitelli; Christopher Sackmann; Martin Hallbeck

Linköping University, Linköping, Sweden

**Background**: Progressive accumulation and cell-to-cell transfer of α-synuclein (α-syn) aggregates is a characteristic feature of Parkinson’s disease (PD). Recently, exosomes have been proposed to play an influential role in cellular transmission of neurodegenerative-related proteins such as oligomeric α-syn (oα-syn). Neutral Sphingomyelinase 2 (nSMase2; encoded by *SMPD3*) hydrolyzes sphingomyelin (SM) to produce ceramide, which generates exosomes through an ubiquitin-independent mechanism. nSMase2 is sensitive to oxidative stress, but there is little information available about how SM metabolism contributes to the pathogenesis of PD.

**Methods**: nSMase2 was downregulated in SH-SY5Y cells by multiple avenues, such as SMPD3 siRNA and a CRISPR/Cas9-targeted cell line. Using flow cytometry, we analysed whether the transfer of oα-syn between neurons was inhibited by blocking nSMase2-related exosome generation. Oxidative stress was induced by keeping the cells in a hypoxic (1% oxygen) incubator for 48 h. Exosomes were isolated by step-gradient ultracentrifugation and characterized by qNano, EXOCET and immunoblot. Analysis of the SM-pathway was performed by real-time-PCR, immunoblotting, confocal microscopy, enzymatic activity and toxicity assays.

**Results**: oα-syn was found in the exosomal fraction, and by inhibiting SMPD3 with siRNA or CRISPR/Cas9, cell-to-cell transfer of oα-syn between neuron-like cells was significantly reduced. Exosome size and concentration were also altered from hypoxia and SMPD3 inhibition. oα-syn became very toxic to cells during hypoxia, while also causing α-syn aggregation, but these effects were nullified with SMPD3 inhibition. Furthermore, nSMase2 enzyme activity, but not protein and gene levels, was significantly increased in response to hypoxia and was negated by inhibiting SMPD3.

**Summary/Conclusion**: Inhibiting SMPD3 may hinder the progression of PD by reducing the amount of oα-syn that is transferred between neurons via exosomes. Increased nSMase2 enzyme activity correlated with cellular toxicity of oα-syn in the presence of oxidative stress, possibly by causing α-syn aggregation, which was negated by downregulating SMPD3. This provides evidence that altering the SM pathway may provide a new avenue to halt PD pathogenesis.

PF07.07

Increased size of extracellular vesicles in amyotrophic lateral sclerosis

Daisy Sproviero^1^; Sabrina La Salvia^1^; Marta Giannini^1^; Valeria Crippa^2^; Stella Gagliardi^1^; Orietta Pansarasa^1^; Mauro Ceroni^3^; Angelo Poletti^2^; Cristina Cereda ^1^

^1^Genomic and Post-Genomic Center, C. Mondino National Institute of Neurology Foundation, IRCCS, Pavia, Italy; ^2^Department of Scienze Farmacologiche e Biomolecolari (DiSFeB), Centro di Eccellenza sulle Malattie Neurodegenerative, Università degli Studi di Milano, Milan, Italy; ^3^Neurology Department, National Institute of Neurology Foundation, Pavia, Italy

**Background**: Amyothrophic lateral sclerosis (ALS) is a progressive adult-onset neurodegenerative disease that affects cortical and spinal motor neurons. The disease is a proteinopathy, in which misfolded proteins (SOD1, TDP-43 and FUS) are templates for the formation of protein oligomers that accumulate and interfere with neuronal function, eventually leading to cell death. These proteins can be transported by extracellular vesicles (EVs), spherical vesicles heterogeneous in size (30 nm–1 µm in diameter), which are classified mainly, on their biogenesis, dimension and superficial markers, in exosomes (EXOs) and microvesicles (MVs). The aim of the present study was to characterize MVs and EXOs in plasma of ALS patients.

**Methods**: MVs and EXOs were isolated from plasma of 30 sporadic ALS patients and 30 healthy volunteers (CTRLs) by ultracentrifugation. Concentration and dimension of MVs and EXOs were analysed by Nanosight NS300. Transmission electron microscopy (TEM) was used to study the morphology of MVs and EXOs. Protein loading of SOD1, TDP-43 and FUS was studied by Western blot analysis.

**Results**: The mean dimension both for MVs (*t*-test, *p* < 0.01) and for EXOs (*t*-test, *p* < 0.001) resulted in increased in ALS patients compared to controls by nanoparticle tracking analysis and TEM. No variation was found in the number of EVs. Misfolded SOD1 was more concentrated in EXOs than in MVs (*p* < 0.001), while TDP-43 and FUS protein levels were slightly higher in MVs than in EXOs (*p* < 0.001). However, misfolded SOD1, TDP-43, p-TDP-43, FUS protein levels were higher in MVs derived from ALS patients than CTRLs (ANOVA test, *p* < 0.05, *p* < 0.05, *p* < 0.01, *p* < 0.05).

**Summary/Conclusion**: In this study, we demonstrated that MVs of ALS patients are enriched with toxic proteins compared to CTRLs while EXOs do not show any protein changes. MVs might act as “postmen” to aberrantly deliver toxic proteins to different tissue and cells affected in ALS disease.

**Funding**: This work was supported by Italian Ministry of Health (grant number: RC13-1603C); AriSLA foundation for funding (Granulopathy-VCP and autophagolysosomal pathway: guardians 449 of proteostasis and stress granule dynamics. Unraveling their implication in ALS); Fondazione Regionale 450 per la Ricerca Biomedica for TRANS-ALS (Translating molecular mechanisms into ALS risk and patient’s 451 well-being).

PF07.08

Plasma-derived extracellular vesicles contain mutant SOD1 in hSOD1G93A transgenic swine

Elena Berrone ^1^; Paola Crociara^1^; Monica Lo Faro^1^; Elena Vallino Costassa^1^; Alessandra Favole^1^; Maria Chiara Deregibus^2^; Giovanni Camussi^3^; Cesare Galli^4^; Roberto Duchi^4^; Adriano Chiò^5^; Andrea Calvo^5^; Federico Casale^5^; Giuseppe Fuda^5^; Giovanni De Marco^5^; Cristina Casalone^1^; Cristiano Corona^1^

^1^Istituto Zooprofilattico Sperimentale del Piemonte Liguria e Valle d’Aosta, Turin, Italy; ^2^University of Turin, Turin, Italy; ^3^Department of Medical Sciences, University of Turin, Turin, Italy; ^4^Avantea srl, Laboratory of Reproductive Technologies, Cremona, Italy; ^5^CRESLA, Regional ALS Reference Centre for Piemonte Region, Turin, Italy

**Background**: Two goals of amyotrophic lateral sclerosis (ALS) research are (a) validation of new experimental models and (b) identification of diagnostic biomarkers, in order to speed up the diagnosis, to monitor its progression and to assess whether a new therapy may be effective. Extracellular vesicles (EVs) and their content may be a reliable clinical biomarker for ALS, as they have yet been used for the diagnosis and prognosis of various diseases. In this context, we developed an hSOD1G93A transgenic swine characterized by a long preclinical and clinical phase in order to clarify certain ALS etiopathogenetic aspects. In particular, EVs characterization in this animal model could elucidate their role in relation to key elements of the disease process. Therefore, this study aimed at evaluating hSOD1 protein into EVs isolated from hSOD1G93A transgenic swine plasma.

**Methods**: EVs were isolated from plasma of hSOD1G93A and wild-type (WT) pigs by a modified precipitation method. EVs were characterized by Tunable Resistive Pulse Sensing, flow cytometry (Cytoflex Beckman) and Western blotting (WB). After immunoprecipitation of lysed EVs, WT and hSOD1 protein detection was performed by WB.

**Results**: Phenotype characterization confirmed that the majority of EVs were exosomes (particle diameter mean: 119 nm, %: 52,29) expressing the typical exosome markers (CD63, TSG101, Flotillin 1, Alix). As regard SOD1 analysis, the hSOD1 protein with the G93A mutation was found only in plasma-derived exosomes of the transgenic swine model, but not in WT.

**Summary/Conclusion**: These results showed that the majority of EVs derived from hSOD1G93A swine model are exosomes able to carry hSOD1G93A protein. Therefore, parallel investigations of exosomes on ALS patients and hSOD1G93A swine model could clarify their role in the pathogenesis of the disease and could represent a new diagnostic and therapeutic strategy.

**Funding**: This work was supported by funding from Italian Ministry of Health and Compagnia di San Paolo Foundation.

PF07.09

Muskelin regulates PrP^C^ exosome packaging and membrane levels and influences prion disease incubation time

Susanne Krasemann ^1^; Frank Heisler^2^; Leonhard Veenendaal^1^; Yvonne Pechmann^2^; Hermann Altmeppen^1^; Mary Muhia^2^; Michaela Schweizer^2^; Matthias Kneussel^2^; Markus Glatzel^1^

^1^University Medical Center Hamburg Eppendorf UKE, Institute for Neuropathology, Hamburg, Germany; ^2^University Medical Center Hamburg Eppendorf UKE, ZNMH, Hamburg, Germany

**Background**: Conformational conversion and spreading of the cellular prion protein (PrP^C^) is key to prion disease pathophysiology. PrP^C^ is a GPI-anchored cell surface protein, has a rapid turnover and is finally degraded in acidic lysosomes. Alternatively, PrP^C^ may be either recycled back to the cell surface or secreted to the extracellular space via exosomes. Regulation of PrP^C^ turnover and sorting into exosomes is not fully understood. Since both PrP^C^ membrane as well as exosome levels affect conversion to and spreading of the misfolded protein isoform PrP^Sc^, PrP turnover may critically influence prion disease progression.

Neuronal PrP^C^ vesicle transport depends on kinesin-1 and cytoplasmic dynein, but regulatory mechanisms that specify and control PrP intracellular trafficking are still unknown. Since muskelin associates with motor protein complexes, we wanted to address whether muskelin might influence the regulation of PrP trafficking.

**Methods**: We transfected culture cells with PrP- and muskelin-reporter constructs to determine interaction and co-localization of both proteins. Muskelin-knockout (KO) mice and primary neurons of these mice were used to confirm our findings *in vivo* and to determine the impact of muskelin on prion disease pathophysiology.

**Results**: Primary neurons from muskelin-KO mice display impaired transport of PrPC vesicles, PrPC lysosomal targeting and degradation. As a consequence, muskelin-KO leads to elevated levels of PrPC at the plasma membrane and increased packaging of PrPC into exosomes. In contrast, overexpression of muskelin led to reduction of exosomal PrP levels. Interestingly, overall exosome secretion remains unchanged. Infection of muskelin-KO mice with prions leads to significantly accelerated prion disease.

**Summary/Conclusion**: We could identify muskelin as a regulator of PrP sorting that is affecting its levels at the plasma membrane and on exosomes, thereby significantly influencing prion disease pathophysiology.

**Funding**: This work was supported by Werner-Otto-Stiftung.

PF07.10

Study of retinal-extracellular vesicles in a model of retinitis pigmentosa: the rd10 mouse

Lorena Vidal ^1^; Maria Oltra^1^; Ayse Sahaboglu^2^; Jorge Barcia^1^; Sancho Javier^1^

^1^Catholic University of Valencia, Valencia, Spain; ^2^University of Tuebingen Institute for Ophthalmic Research, Thuringen, Germany

**Background**: Several degenerative eye diseases, such a *retinitis pigmentosa* (RP), age-related macular degeneration and diabetic retinopathy, are associated with impaired photoreceptor cells and retinal pigment epithelium (RPE) function. RPE and photoreceptor cells release extracellular vesicles (EVs), which might introduce their cargo in neighbouring cells, thus influencing their fate. It has been confirmed that retinal EVs change in number and in cargo when the retina is under stress. The aim of this study is to obtain, isolate and investigate EVs released from the retina under physiological and pathological conditions. We also check EVs after applying a treatment that prolongs the retinal surviving time.

**Methods**: For our first approach, we used an animal model of RP: the rd10 mouse. EVs from retinal organotypical explants (of rd10 and wild-type (wt)) were isolated using isolating columns. EVs identity was confirmed by electron microscopy and nanoparticle tracking analysis. Subsequently, retinal EVs were counted using EV markers (CD9) and specific retinal markers (RPE65, rhodopsin) by fluorescence-activated cell sorting and Western blot.

**Results**: We successfully isolated EVs from retinal explants. Identity of EVs was confirmed in both cases and typical morphology was observed under the electron microscope. Higher amount of retinal EVs are observed in pathological conditions. Nevertheless, the population of EVs presenting RPE65 and rhodopsin was reduced in the rd10, when compared to wt. When treatment was applied, EVs levels returned to wt conditions.

**Summary/Conclusion**: Apparently, the RPE and photoreceptors modify their EVs release rate and cargo depending on their physiological state. That fact may help us in the future to develop an early diagnostic for eyes conditions, such as RP.

**Funding**: This work was financially supported by from Catholic University of Valencia “San Vicente Martir”.

PF07.11

The effect of aspirin daily dose change on platelet-derived microvesicles in patients after ischaemic stroke

Justyna Rosinska; Maria Lukasik; Joanna Maciejewska; Robert Narozny; Wojciech Kozubski

Department of Neurology, Poznan University of Medical Sciences, Poznan, Poland

**Background**: Platelet-derived microvesicles (pMV) are increased in ischaemic stroke and possess prothrombotic and proinflammatory potential. Reports on responsiveness of pMV to treatment with aspirin are contradictory; however, the vast majority suggest limited impact of this treatment on pMV which might encourage searching for novel therapeutic strategies. The aim of this pilot study was to evaluate the effect of aspirin daily dose change on pMV in patients after ischaemic stroke.

**Methods**: We recruited patients with a history of ischaemic stroke from 3 to 12 months prior to study enrolment. Blood samples were collected at baseline, while aspirin was taken in daily doses of 75 mg in accordance with previous recommendations, and after a 3-day period of taking aspirin in increased doses (150 mg/day). pMV were isolated from citrated blood by centrifugation, incubated with the following antibodies: CD61/PerCP (platelet gating Ab), Annexin V/PE (Ab against phosphatidylserine), CD62P/PE-Cy5 (Ab against P selectin), PAC-1/FITC (Ab against active form of GPIIb/IIIa) and CD154/APC (Ab against CD40L) then analysed with an Apogee A50-Micro flow cytometer. Thromboxane B2 (TXB2) serum level by enzyme-linked immunosorbent assay was also measured to confirm compliance with aspirin therapy.

**Results**: We included 35 patients with a history of ischaemic stroke. The increase of aspirin daily dose did not cause a statistically significant difference in pMV concentration or their subtypes defined by expression of superficial markers such as phosphatidylserine, CD 40L and selectin P. Only reduced concentration of PAC-1+ CD61+ was observed [16 (13–26) n/µl vs. 9 (5–12) n/µl; *p* = 0.04].

**Summary/Conclusion**: The increase of aspirin daily dose did not affect pMV (CD61+) concentration in patients after ischaemic stroke. However, it did decrease the concentration of PAC-1+ CD61+. Further larger population-based studies are needed to establish the relationship between aspirin daily dose and concentration of pMV as well as their subpopulation in patients after ischaemic stroke.

**Funding**: This study was financially supported by the governmental research grant National Science Centre 2014/15/B/NZ4/00736.

PF07.12

The effect of remote ischaemic conditioning on the physicochemical properties of extracellular vesicles

Jesper Just^1^; Thomas Ravn Lassen^2^; Tingting Gu ^1^; Hans Erik Boetker^2^; Kim Ryun Drasbek^1^

^1^Center of Functionally Integrative Neuroscience, Department of Clinical Medicine, Aarhus University, Denmark, Århus C, Denmark; ^2^Department of Clinical Medicine, Aarhus University, Aarhus N, Denmark;

**Background**: Remote ischaemic conditioning (RIC) is a non-invasive treatment procedure that has been shown to exert powerful protection against ischaemia-reperfusion injury in acute myocardial infarction and stroke. Currently, RIC is being evaluated in treating a number of other diseases. RIC is performed by inducing repeated short cycles of controlled limb ischaemia and reperfusion with a blood pressure cuff. Blood-borne extracellular vesicles (EVs) released by the RIC intervention are considered to, in part, mediate the protective effects of RIC through biological interaction with target cells. However, the effect of RIC on the physicochemical properties of EVs remains unknown, which is of utmost importance to understand the functional biological properties of the EVs after RIC intervention.

**Methods**: Blood plasma was collected from control rats (Sprague Dawley) and rats subjected to RIC (5 min post RIC). EVs were then isolated from plasma by size-exclusion chromatography and characterized by tunable resistive pulse sensing (TRPS) to measure concentration, size and zeta potential (surface charge) on a particle-by-particle basis.

**Results**: We did not observe any changes in concentration or size distribution between RIC and control EVs. Successful measurements of RIC EV zeta potential on a particle-by-particle basis were achieved. However, no difference in the zeta potential mean or EV subpopulations (zeta potential frequency distribution) between RIC and control EVs was observed.

**Summary/Conclusion**: Using the TRPS measuring technique, we did not find differences in the physicochemical properties of EVs isolated from RIC or control rat plasma in regards to EV concentration, size distribution or surface charge.

**Funding**: The study was supported by the Novo Nordisk Foundation and Riisfort Foundation.

PF07.13

Ischaemia-related conditions induce secretion of miR-21-5p-containing extracellular vesicles that alter microglial activation

Nea Bister ^1^; Shaila Eamen^1^; Benjamin Huremagic^2^; Paula Korhonen^1^; Sanna Loppi^1^; Flavia Scoyni^1^; Henna Konttinen^1^; Lesley Cheng^3^; Laura J. Vella^4^; Maria Bouvy-Liivrand^5^; Simone Caligola^2^; Andrew F. Hill^3^; Katja M. Kanninen^1^; Rashid Giniatullin^1^; Merja Heinäniemi^5^; Rosalba Giugno^2^; Tarja Malm^1^

^1^A.I. Virtanen Institute for Molecular Sciences, University of Eastern Finland, Kuopio, Finland; ^2^Department of Computer Science, University of Verona, Verona, Italy; ^3^Department of Biochemistry and Genetics, La Trobe Institute for Molecular Science, Melbourne, Australia; ^4^The Florey Institute of Neuroscience and Mental Health, Melbourne, Australia; ^5^School of Medicine, University of Eastern Finland, Kuopio, Finland

**Background**: Ischaemic stroke is a prevalent cause of mortality and morbidity worldwide. Despite several clinical trials, there is no effective treatment for motor and cognitive deficits induced by stroke, suggesting poorly understood pathology. Recent studies show a crucial role of microRNAs (miRNAs) in cellular adaptation to various stress conditions. MiRNAs can be trafficked in extracellular vesicles (EVs), providing a compelling mechanism for cell-to-cell communication. However, the effect of ischaemia on the release of EVs is largely unknown. This study was carried out to investigate whether vesicular miRNAs mediate cell-to-cell communication in ischaemic stroke.

**Methods**: To screen for stroke-induced miRNA changes, small RNA sequencing was performed on brain tissue collected from healthy mice and after stroke surgery. Effects of cellular stress on the first stages of transcriptional regulation were obtained from nascent RNA sequencing (GRO-seq) performed on neurons exposed to glutamate. EVs were isolated by ultracentrifugation-based method from Neuro 2A (N2A) cell-conditioned medium from normal or ischaemia-related conditions. EV preparations were characterized by nanoparticle tracking analysis, electron microscopy and Western blot. RNAs from EVs and N2A cells were extracted, and miRNAs were analysed by qPCR. EVs were administered to microglial cells to analyse the effects of EVs on cytokine secretion.

**Results**: The expression of miR-21-5p was upregulated in ischaemic brain tissue and N2A cells. Neuronal transcription of the miR-21 locus was increased after exposure to glutamate. N2A cell-derived EVs exhibited vesicular morphology and size distribution typical for exosomes, and EVs contained miR-21-5p. Administration of EVs from N2A cells altered microglial responses to lipopolysaccharide, suggesting immunomodulatory effects.

**Summary/Conclusion**: Based on the current knowledge, we propose miR-21-5p as a promising candidate for further studies to investigate its functions in EVs and stroke-induced injury.

**Funding**: The University of Eastern Finland funded doctoral student position. This work was also funded by Academy of Finland, Emil Aaltonen Foundation, and Paavo Nurmi Foundation.

PF07.14

Exosomes and neuroinflammatory microRNAs: cytokine-specific profiles

Ashley Russell ^1^; Sujung Jun^2^; Sara Lewis^1^; Stephanie Rellick^1^; James Simpkins^1^

^1^West Virginia University, Morgantown, WV, USA; ^2^Johns Hopkins University School of Medicine, Baltimore, MD, USA

**Background**: Evidence suggests that exosomes participate in the spread of pathology by transferring misfolded proteins and aberrant microRNAs (miR) from diseased to healthy cells. Many neurodegenerative diseases are associated with chronic neuroinflammation, characterized by increased levels of immunomodulatory molecules, such as tumour necrosis factor-alpha (TNF-α) and interferon-gamma (IFN-γ).

**Methods**: We investigated the effects of these cytokines on exosome secretion, miR expression and mitochondrial function. We exposed a neuronal cell line to varying concentrations of TNF-α or IFN-γ for 24 h, isolated exosomes and used the NanoSight NS300 to determine if enriched vesicles were consistent in size with exosomes after cytokine exposure. Using qRT-PCR we profiled the exosomal and intracellular levels of three miRs associated with neuroinflammation (miR-34a, -146a and -155), and also assessed mitochondrial function after exposure to these cytokines directly or after exposing naïve cells to exosomes isolated from the conditioned media of cytokine exposed cells. Finally, we performed Western blot analyses to determine changes in miR-34a mRNA target protein expression.

**Results**: Exposure to either cytokine significantly increased exosome secretion compared to control. Exposure to TNF-α induced a dose-dependent increase in all three miRs, with differences in intracellular profiles (miR-34a unchanged, miR-145a and -155 significantly upregulated). Data suggest IFN-γ exposure induces different miR expression patterns than does TNF-α. Exposure to either cytokine does not appear to induce mitochondrial dysfunction. Interestingly, exposing naïve cells to isolated exosomes from the media of cytokine-exposed cells increases the respiratory capacity of mitochondria. Imaging studies confirm naïve cells take up the exosomes.

**Summary/Conclusion**: These data suggest that various cytokines can induce exosome secretion, leading to differential miR profiles due to miR-specific exosomes packaging and secretion mechanisms. All three miRs were dose-dependently increased in exosomes, while the intracellular levels differed. These cytokines do not directly impact mitochondria; however, naive cells respond to cytokine-induced exosomes.

**Funding**: This work was funded by the NIH grants PO1AG022550, P01AG027956, P20GM109098, T32GA052375, U54GM104942.

PF07.15

Exploring the neuroprotective function of extracellular vesicles containing small heat-shock proteins (HSPB1 and HSPB8) upon neuroinflammation

Bram Van den Broek ^1^; Sam Vanherle^1^; Vicky De Winter^2^; Sören Kuypers^3^; Vincent Timmerman^2^; Veerle Somers^3^; Luc Michiels^4^; Joy Irobi^5^

^1^Neurofunctional genomics Group, Biomedical Research Institute (BIOMED), Hasselt University, Hasselt, Belgium; ^2^Peripheral Neuropathy Group, VIB-Department of Molecular Genetics, University of Antwerp, Antwerpen, Belgium; ^3^Biomedical research institute (BIOMED), Hasselt University, Hasselt, Belgium; ^4^Bionanotechnology group, Biomedical research institute (BIOMED), Hasselt University, Hasselt, Belgium; ^5^Neurofunctional genomics Group, Biomedical Research Institute (BIOMED), Hasselt University, Diepenbeek, Belgium

**Background**: Currently, the repair mechanisms of multiple sclerosis (MS) are still unknown. However, it is known that small heat-shock proteins (HSPBs), which have protective functions, are upregulated in MS lesions. During MS lesion development, HSPB1 and 8 are upregulated in astrocytes but downregulated in oligodendrocytes and microglia cells. Furthermore, it is shown that mutations in HSPB1 and 8 cause peripheral neurodegeneration. Although the protective intracellular functions of HSPBs are known, the extracellular functions are unclear. One way cells secrete HSPBs is by releasing extracellular vesicles (EV). We hypothesize that extracellular HSPBs exhibit neuroprotective roles which are altered upon inflammation in oligodendrocytes.

**Methods**: To determine the protective activity of intracellular and extracellular HSPBs in oligodendrocytes, we establish HSPB overexpressing cell-lines for the production and characterization of HSPB-positive EV under normal and TNF-α-inflamed conditions. These purified HSPB-EVs are further applied to measure their role in cell survival and their chaperone activity by looking at the amount of monomeric HSPB (active form) in comparison with the dimeric fraction.

**Results**: Pilot data show that in oligodendrocytes, upon heat shock, there is a slight increase in early apoptosis. Striking when oligodendrocytes were stimulated with inflamed EV, they exhibited a similar level of apoptosis comparable to known inflammatory mediators. In addition, immunoblot analysis of oligodendrocytes showed low expression of monomeric HSPB1 and 8 in non-stressed cells.

**Summary/Conclusion**: Reduction in endogenous expression of HSPB1 and 8 in oligodendrocytes might impair their cytoprotective activity during early inflammation.

**Funding**: This work was financed by Hasselt University and by EFRO through the Interreg V Grensregio Vlaanderen Nederland project Trans Tech Diagnostics.

PF07.16

Characterization of the CSPα-extracellular vesicle export pathway

Desmond Pink ^1^; Donnelier Julien^2^; Janice Braun^2^; John D. Lewis^1^

^1^University of Alberta, Edmonton, Canada; ^2^Cumming School of Medicine, University of Calgary, Calgary, Canada

**Background**: Extracellular vesicles (EVs) are a collection of secreted vesicles of diverse size and cargo and have been implicated in the physiological removal of nonfunctional proteins as well as the cell-to-cell transmission of disease-causing proteins in several neurodegenerative diseases. We have previously shown that cysteine string protein (CSPα; DnaJC5), a molecular co-chaperone that is critical for proteostasis at the synapse, is responsible for the export of disease-causing-misfolded proteins from neurons in EVs. This export is resveratrol-sensitive but the CSPα-EV and resveratrol-sensitive EV pools have never been explored and compared.

**Methods**: To perform the initial characterization experiments, conditioned media, from CAD cells treated under different conditions, was analysed using both nanoparticle tracking analysis and nanoscale flow cytometry (Apogee A50). EVs were also imaged using electron microscopy.

**Results**: A subpopulation of EVs released from CAD cells contains the disease-associated protein GFP-tagged 72Q huntingtin^exon1^. Export of the GFP-tagged EVs increased in the presence of CSPα and decreased in the presence of resveratrol. Further examination revealed stimulation of EV export by mutant CSPαs was also inhibited by resveratrol. In addition to CSPα, we also identified another J protein co-chaperone, DnaJB2 (HSJ1), that targets GFP-tagged 72Q huntingtin^exon1^ cargo for EV export. These data identify the involvement of a specific subset of EVs that remove misfolded proteins that can be pharmacologically targeted.

**Summary/Conclusion**: Our data highlight the parallels between proteostasis and EV export, as two J proteins known to contribute to neuronal proteostasis are the same as those that mediate export of misfolded proteins.

**Funding**: The work was supported by a grant from the Alzheimer Society of Alberta and Northwest Territories and the Alberta Prion Research Institute. The authors would like to express their gratitude to Dr Frank Visser for technical support.

EVs in Cardiovascular Diseases and Coagulation Chairs: Ramaroson Andriantsitohaina; Costanza Emanueli Location: Exhibit Hall 17:15–18:30

PF08.01

Plasma- and urine-derived exosomes reveal a microRNA signature in hypertensive patients with albuminuria

Javier Perez-Hernandez ^1^; Dolores Olivares^2^; Daniel Perez-Gil^2^; Angela Riffo-Campos^1^; Elena Solaz^1^; Fernando Martinez^1^; Gernot Pichler^1^; F. Javier Chaves^2^; Josep Redon^1^; Raquel Cortes^1^

^1^Cardiometabolic and Renal Risk Research Group, INCLIVA Biomedical Research Institute, Valencia, Spain; ^2^Genomic and Genetic Diagnosis Unit, INCLIVA Biomedical Research Institute, Valencia, Spain

**Background**: Urinary albumin excretion (UAE) is an indicator of early renal damage and cardiovascular risk. MicroRNAs (miRNAs) regulate gene expression and changes in urinary and plasma miRNAs have been reported in the progression of kidney diseases. The aim of this work was to establish an exosomal miRNA profile associated to the presence of albuminuria in hypertension.

**Methods**: We have analysed 52 hypertensive patients, 24 microalbuminuric (UAE = 162.8 ± 168.2 mg/g Cr; age 52.7 ± 8.4 years) and 28 normoalbuminuric (age 54.5 ± 5.6 years). Exosomes were isolated by differential ultracentrifugation from urine and plasma. Individual exosomal RNA samples were profiled using small RNA sequencing. MiRNA over-representation and enrichment analysis were performed in order to identify the most regulated biological processes (KEGG pathway). MiRNA candidates were validated by RT-qPCR and their levels were correlated to UAE.

**Results**: We identified a signature of 29 exosomal miRNAs differentially expressed in response to microalbuminuria. The most regulated biological processes were cell signalling (MAPK, calcium, p53), TGF-β and VEGF pathways, regulation of actin cytoskeleton, cell cycle and apoptosis as well as cell adhesion and extracellular matrix interaction. We validated a decrease in the amount of miR-26a-5p and miR-222-3p in plasma exosomes of microalbuminuric patients compared to normoalbuminuric (*p* < 0.05), whereas miR-126-3p and miR-191-5p were significantly augmented (*p* < 0.01). In addition, miRNA levels were correlated with albumin excretion inversely, miR-26a-5p and miR-222, or directly, miR-126-3p and miR-191-5p (*p* < 0.01).

**Summary/Conclusion**: Our results show an exosomal miRNA signature associated to microalbuminuria in hypertension. Interestingly, most of the deregulated miRNAs are involved in the maintenance of the glomerular filtration barrier and altered in glomerular pathologies. These results would remark the importance of exosomal miRNAs in the intraglomerular crosstalk.

**Funding**: This work was supported by the funding for research in health sciences of the Carlos III Health Institute (PI16/01402 and PI12/02615) and with ERDF funds.

PF08.02

Comparative analysis of extracellular vesicles from arterial and venous blood reveals only minor differences in vesicle composition

Stefanie Hermann ^1^; Dominik Buschmann^1^; Benedikt Kirchner^1^; Melanie Märte^2^; Florian Brandes^2^; Marlene Reithmair^3^; Gustav Schelling^2^; Michael W. Pfaffl^1^

^1^Division of Animal Physiology and Immunology, School of Life Sciences Weihenstephan, Technical University of Munich, Freising, Germany; ^2^Department of Anesthesiology, University Hospital, Ludwig-Maximilians-University Munich, Munich, Germany; ^3^Institute of Human Genetics, Ludwig-Maximilians-University Munich, Munich, Germany

**Background**: The circulatory system entails arterial blood delivering nutritive substances to tissues, and venous blood removing metabolic waste. Although circulating extracellular vesicles (EVs) are crucial vehicles for intercellular communication, arteriovenous differences in EV composition still have to be revealed. Various biomarker studies use vesicular microRNA (miRNA) as target, and depending on the patient’s state of health and the routinely used type of vascular access, blood samples may be either derived from arterial or venous origin. We compared EV-specific miRNA expression profiles of arterial and venous serum samples from cardiac surgical patients (CSPs) and further characterized the vesicles.

**Methods**: We included 19 CSPs in our study. For each patient, arterial blood was drawn from the radial artery, while venous blood was sampled from the superior vena cava via indwelling vascular catheters. We isolated EVs from sera using a polymer-based precipitation method and extracted total vesicular RNA. EVs were characterized using nanoparticle tracking analysis, Western blot analysis and transmission electron microscopy. Based on small RNA sequencing, differential expression analysis was performed using DESeq2.

**Results**: EV size, concentration and morphology from arterial and venous blood samples were highly similar, and typical EV protein markers were present in all samples. The obtained next-generation sequencing data revealed no significantly regulated miRNAs between arterial and venous blood EVs (baseMean 50, log2 fold change ≥I1I, *p*-adj ≤0.05). High patient-specific intra-sample diversity was shown, while arteriovenous inter-sample variations were minimal (principal component analysis).

**Summary/Conclusion**: Our data show that EVs from arterial and venous blood specimens of CSPs don’t differ in size, morphology and miRNA content. It is likely that these results could be extended to other patient populations as well. Thus, it is probably feasible to use arterial or venous serum samples for EV biomarker studies with comparable results regarding miRNA expression profiles. This may not apply to all individuals and all disorders, however, and additional arteriovenous comparisons may have to be performed under different pathophysiologic situations (e.g. newborns or cardiogenic shock).

PF08.03

Can vesicular microRNAs predict negative perioperative outcomes in cardiac surgery?

Dominik Buschmann ^1^; Marlene Reithmair^2^; Benedikt Kirchner^1^; Stefanie Hermann^1^; Melanie Märte^3^; Florian Brandes^3^; Ortrud Steinlein^2^; Michael W. Pfaffl^1^; Gustav Schelling^3^

^1^Division of Animal Physiology and Immunology, School of Life Sciences Weihenstephan, Technical University of Munich, Freising, Germany; ^2^Institute of Human Genetics, Ludwig-Maximilians-University Munich, Munich, Germany; ^3^Department of Anesthesiology, University Hospital, Ludwig-Maximilians-University Munich, Munich, Germany

**Background**: Open-heart surgery is one of the most commonly performed surgical procedures worldwide, but carries a substantial risk for adverse outcomes such as postoperative organ failure. Extracellular vesicle (EV)-based biomarkers for outcome prediction and risk-stratification may be useful to identify patients at risk for negative outcomes including mortality.

**Methods**: We isolated serum EVs from patients (*n* = 19) prior to open-heart surgery and from healthy volunteers (*n* = 20) by precipitation. EVs were characterized by nanoparticle tracking analysis, transmission electron microscopy and immunoblotting. Next-generation sequencing (NGS) was utilized to profile EV-associated miRNAs. Differential expression of miRNAs between patients and volunteers was assessed using DESeq2. Expression levels of dysregulated miRNAs were correlated to prospectively recorded outcome-relevant variables registered during and after surgery.

**Results**: There were no significant differences in morphology or marker proteins in EV populations from patients and volunteers. In NGS data, however, a total of 86 and 77 miRNAs were significantly up- or downregulated, respectively, in patient EVs prior to surgery. Expression patterns of miRNAs separated cardiovascular disease patients from volunteers in principal component analysis. For a set of differentially regulated miRNAs, expression levels were found to correlate with intraoperative epinephrine dosing requirements (*r* = 0.52, *p* = 0.02), serum lactate levels (*r* = 0.47, *p* = 0.04) and low urine excretion (*r* = −0.48, *p* = 0.03), indicating a systemic low-perfusion state due to perioperative heart failure. These miRNAs include miR-125a-5p for epinephrine requirements (log2 fold change (FC) = 1.83, *p*-adj = 2.06E-06), let-7d-3p (log2FC = 1.07, *p*-adj = 4.01E-08) for serum lactate and miR-30a-5p (log2FC = 1.04, padj = 2.06E-06) for low perioperative urine excretion. Clinical parameters more closely related to the surgical procedure than to organ dysfunction (e.g. duration of surgery and ICU therapy, inflammation) or demographic variables (e.g. age, sex or body mass index) did not significantly correlate with miRNA expression.

**Summary/Conclusion**: Analysing EV-miRNAs prior to heart surgery might help to identify patients at risk for perioperative cardiovascular instability and adverse outcomes.

PF08.04

Extracellular vesicles released by tumour microenvironment and clonal monocytes induce a procoagulant climate within chronic myelomonocytic leukemia tumour niche

Natacha Mauz ^1^; Park Sophie ^1^; Mathieu Meunier^1^; Landry Seyve^2^; Julie Brault^3^; Jean-Yves Cahn^1^; Cognasse Fabrice^4^; Benoît Polack^5^

^1^Clinique Universitaire d’Hématologie, CHU Grenoble Alpes, La Tronche, France; ^2^CHU Grenoble Alpes, Laboratoire d’hémostase, La Tronche, France; ^3^CHU Grenoble Alpes, Centre de Diagnostic et de Recherche sur La CGD, La Tronche, France; ^4^GIMAP-EA3064, Université de Lyon, Saint-Etienne, France; ^5^CHU Grenoble Alpes, Laboratoire d’Hémostase, La Tronche, France

**Background**: Chronic myelomonocytic leukemia (CMML) is a haematological malignancy close to myelodysplastic syndromes (MDS). The role of tumour microenvironment (TME) in MDS pathogenesis is increasingly described, especially the interactions between mesenchymal stromal cells (MSC) and haematopoietic stem cells (HSC). Cancer is associated with a procoagulant state participating in tumour development and metastatic process. Monocytes release procoagulant microparticles (MP) carrying tissue factor (TF). In this project, we show that extracellular vesicles (EV) released by CMML MSC and monocytes induce a procoagulant climate within the tumour niche.

**Methods**: MSC were isolated from five CMML patients and five healthy donors (HD) bone marrows, cultured in a classic medium containing foetal bovine serum (FBS) and incubated in a medium deprived of FBS EV 72 h before collecting EV. Monocytes were isolated from the blood of five CMML patients and four reactive monocytosis and deprived of FBS for 40 h to induce vesiculation.

EV were extracted by sequential centrifugations of medium supernatant. Pellets obtained after 10,000 and 100,000 *g* ultracentrifugations respectively correspond to medium EV (mEV) containing mostly MP and small EV (sEV) containing exosomes and small MP. EV concentration and size were analysed by nanoparticle tracking analysis. Coagulation was explored by thrombin generation assay (TGA) and fibrinolysis by fibrinography, a new assay currently developed.

**Results**: Vesiculation rate (number of EV shed per cell) is higher for MSC than monocytes (*p* < 0.01). Mean size is higher for mEV (226 nm) than sEV (188 nm, *p* = 0.01).

TGA experiments performed with normal poor platelet plasma show that CMML MSC bear a higher procoagulant activity (PCA) than HD MSC and 0.5 pM of TF. sEV issued from CMML MSC and monocytes carry a PCA unlike sEV from HD MSC and monocytes. The same experiments repeated with a factor VII-deficient plasma or adding an anti-TF antibody show an abolition of TF-pathway-mediated thrombin generation, suggesting that sEV PCA is linked to the presence of TF. Fibrinolysis analyses show a longer clot lysis time when adding CMML MSC sEV compared to HD (*p* = 0.02), revealing a higher clot resistance to lysis.

**Summary/Conclusion**: EV issued from clonal monocytes and TME induce a procoagulant climate within CMML tumour niche. This procoagulant environment coud impact haematopoietic stem cell homoeostasis.

PF08.05

Differential contribution of extracellular vesicles from different settings to thrombin generation

Carla Tripisciano ^1^; René Weiss^1^; Tanja Eichhorn^1^; Andreas Spittler^2^; Michael Bernhard Fischer^1^; Viktoria Weber^1^

^1^Danube University Krems, Krems, Austria; ^2^Medical University of Vienna, Vienna, Austria

**Background**: We aimed to assess the pro-coagulant activity of extracellular vesicles (EVs) enriched from different physiological matrices and compare EVs from blood products and cell culture environment.

**Methods**: Platelet and erythrocyte concentrates were obtained from whole blood apheresis using an automated blood collection system (Gambro BCT). Following the removal of blood cells (2500 *g*), two EV fractions were enriched by sequential centrifugation at 20,000 *g* (EV I) and 100,000 *g* (EV II). Vesicle fractions were normalized to protein content and characterized by a combination of nanoparticle tracking analysis, cryo-electron microscopy, imaging flow cytometry and flow cytometry, using lactadherin as a marker for phosphatidylserine (PS)-exposing vesicles, CD41 for platelet-, CD235a for erythrocyte- and CD14 for monocyte-derived EVs.

**Results**: EVs derived from physiological platelet and erythrocyte units supported thrombin generation in a dose-dependent and PS-dependent manner, due to the activation of the contact pathway. By inhibition of factor XII using corn trypsin inhibitor, thrombin formation was inhibited in blood-derived EVs, but not in EVs from lipopolysaccharide-stimulated monocytic cells, which triggered the extrinsic pathway due to their exposure of tissue factor (TF). EVs enriched from unstimulated monocytic cells, in contrast, supported neither TF- nor FXII-triggered thrombin generation. Removal of residual protein contaminants from blood-derived EV fractions using size-exclusion chromatography did not result in reduced thrombogenicity, therefore excluding the co-enrichment of soluble clotting factors.

**Summary/Conclusion**: Our findings indicate the potential of EVs derived from blood products, but not from cell culture environments, to support thrombin generation triggered via the contact pathway, while the exposure of functionally active TF on EVs, and therefore their ability to initiate coagulation, seems to be restricted to pathological settings. The differences in the thrombogenic ability of EVs isolated from supernatants vs. blood products suggest that EVs isolated from blood might support coagulation due to their association with blood-derived coagulation factors.

**Funding**: This work was funded by the Christian Doppler Society; Christian Doppler Laboratory for Innovative Therapy Approaches in Sepsis.

PF08.06

Increased venous and intra-atrial appendicular blood plasma levels of tissue factor-exposing extracellular vesicles in atrial fibrillation patients

Morten Mørk ^1^; Jan J. Andreasen^2^; Lars H. Rasmussen^3^; Gregory Y.H. Lip^4^; Shona Pedersen^1^; Rikke Baek^3^; Malene M. Jørgensen^3^; Søren R. Kristensen^1^

^1^Department of Clinical Biochemistry, Aalborg University Hospital, Aalborg, Denmark; ^2^Department of Cardiothoracic Surgery, Aalborg University Hospital, Aalborg, Denmark; ^3^Department of Clinical Medicine, Aalborg University, Aalborg, Denmark; ^4^Institute of Cardiovascular Sciences, University of Birmingham, Birmingham, UK

**Background**: Atrial fibrillation (AF) is the most common sustained cardiac arrhythmia. AF is associated with a markedly increased risk of stroke caused by thrombi formed in the left atrial appendage (LAA) of the heart. In a previous study, elevated venous blood levels of tissue factor (TF) antigen in AF patients were demonstrated. TF is the principal initiator of blood clotting *in vivo*. TF-bearing extracellular vesicles (EVs) may be released from activated cells in the LAA in AF patients. We aimed to study if venous and intra-LAA blood concentrations of TF-bearing EVs and other procoagulant biomarkers are elevated in AF patients.

**Methods**: From 13 patients with AF and 12 controls without AF, venous blood (Vpre) was sampled prior to cardiac surgery. Intraoperatively, venous blood (Vint) and blood sampled directly from the LAA were collected. A protein microarray-based method (EV Array) was used for evaluation of blood plasma levels of EVs, including subtypes exposing TF. In addition, plasma levels of TF antigen, von Willebrand factor (vWF) antigen, cell-free deoxyribonucleic acid (cf-DNA), procoagulant phospholipids (PPLs) and total submicron particles as measured by nanoparticle tracking analysis were evaluated.

**Results**: Median Vpre TF antigen concentration was significantly higher in the AF patient group (335 pg/mL) than in the control group (232 pg/mL) (*p* < 0.05), with a similar significant difference (*p* < 0.05) in the Vint, and insignificant trend (*p* = 0.07) in the LAA samples. Median Vpre vWF antigen level was significantly higher (1.54 kIU/L) in the AF patient group than in the control group (1.19 kIU/L) (*p* < 0.05) with a similar significant difference in the Vint and LAA samples. Median Vpre level of TF-bearing EVs was significantly higher (3.2 arbitrary units) in AF patients than in controls (0.0 arbitrary units) (*p* < 0.05) with a similar significant difference in the Vint and LAA samples. No significant differences in levels of cf-DNA, PPLs or total submicron particles were found between the AF patient group and the control group. When comparing Vint and LAA samples, no significant differences in levels of any of the measured analytes were observed.

**Summary/Conclusion**: Elevated blood plasma concentrations of TF in AF patients may be partly explained by increased levels of TF-bearing EVs. TF-bearing EVs may play a role in AF-related thrombogenicity.

PF08.07

Involvement of platelet αIIbβ3 integrin and downstream signalling pathways in release of extracellular vesicles, CXCL4 and CCL5

Alexandra C.A. Heinzmann ^1^; Tanja Vajen^1^; Nicole M.M. Meulendijks^2^; Dennis P.L. Suylen^1^; Judith M.E.M. Cosemans^1^; Johan W.M. Heemskerk^1^; Tilman M. Hackeng^1^; Rory R. Koenen^1^

^1^Department of Biochemistry, Cardiovascular Research Institute Maastricht (CARIM), Maastricht University, Maastricht, The Netherlands; ^2^The Netherlands Organisation for Applied Scientific Research (TNO), Material Solutions, Eindhoven, The Netherlands

**Background**: Platelets play essential roles in haemostasis and thrombosis, and are important in inflammation and immunity. These functions are mediated by the presence of bioactive molecules in platelet interior, which are secreted upon activation. Chemokines CCL5 and CXCL4 are stored in platelet α-granules, and become released by stimulation of thrombin or collagen receptors. During prolonged storage and after activation, platelets can also shed extracellular vesicles (EVs), which modulate haemostatic and inflammatory processes. The aim of this study was to compare the release mechanisms of EVs and chemokines in activated platelets.

**Methods**: Isolated platelets were activated with convulxin or thrombin for 30 min at 37°C. Isolation of EVs was performed with ultracentrifugation at 20,000 *g* for 1 h at 4°C. Chemokines were found in the supernatant and EVs were present in the pellet. Release of chemokines was measured by immunoassays, while release of EVs was quantified by measuring their phosphotidylserine content (prothrombinase assay) and nanoparticle tracking analysis. Investigation of different aspects of αIIbβ3 integrin and associated outside-in signalling was performed by treatment of platelets prior to activation with different inhibitors.

**Results**: Stimulation of collagen and/or thrombin receptors with convulxin and thrombin resulted in a robust release of EVs and CCL5 and CXCL4. Release of EVs, but not of chemokines, was abrogated by inhibiting cytoskeletal rearrangement and blocking integrin αIIbβ3 with eptifibatide. Whereas blockade of c-Src only weakly affected EV release, it could be inhibited by blockade of Gα13. Neither blockade of c-Src nor of Gα13 influenced release of chemokines. To further investigate αIIbβ3-associated signalling, calpain and PTPN1 were blocked upon platelet activation. Inhibition of calpain significantly reduced EV release, yet increased chemokine secretion. In addition, PTPN1 inhibitor also resulted in decreased EV release, yet showed only minor effects on chemokine release.

**Summary/Conclusion**: This study set out to examine the involvement of αIIbβ3 integrin and outside-in signalling events in platelet EV and chemokine release. The current data highlight the importance of αIIbβ3 integrin in EV release by activated platelets, while chemokine secretion appears to be governed by the inside-out signalling pathway.

PF08.08

Explosive versus penetrating mechanisms of combat injury in the generation of prothrombotic microvesicles

Anna E. Sharrock ^1^; Paul Harrison^2^; Rory Rickard^1^; Sara Rankin^3^; Tom Woolley^4^

^1^Academic Department of Military Surgery and Trauma, Birmingham, UK; ^2^Institute of Inflammation and Ageing, Birmingham University, Birmingham, UK; ^3^National Heart and Lung Institute, Imperial College, London, UK; ^4^Academic Department of Military Anaesthesia and Critical Care, Birmingham, UK

**Background**: Combat casualties with explosive injuries are postulated to have a higher risk of coagulopathy and death compared to those injured by penetrating mechanisms. The role of microvesicles (MVs) in this process has yet to be established.

**Methods**: Blood was retrieved from UK combat casualties during combat operations in Afghanistan on emergency department (ED) admission, 45 and 90 mins, during intensive therapy unit admission to aeromedical repatriation. Calibrated automated thrombography (CAT) and flow cytometry (FC) was used to assess tissue factor (TF) and phosphatidylserine (PS) activity and MV lineage (CD11b, CD146, CD235a, CD61, TF, PS). Results were compared to healthy volunteers (HV) and analysed by mechanism of injury (explosive vs. penetrating).

**Results**: Analysis comprised 39 time point samples from 9 patients (FC) and 14 from 5 patients (CAT). At ED admission, all FC CD11b+, CD146+, CD235a+ and CD61+ events rose vs. HV (*p* < 0.05). At ED admission and 45 min later, the time to peak TF and PS activity was faster versus HV (TF mean 8.78 (*p* = 0.003) and 9.09 (*p* = 0.001) vs. 19.58 min, PS mean 7.89 (*p* < 0.000) and 7.44 (*p* < 0.000) vs. 20.92 min), and the endogenous thrombin potential of PS was greater (mean 1054 (*p* = 0.003) and 1360 (*p* < 0.000) vs. 282.2).

**Summary/Conclusion**: MV may have a role in the development and propagation of coagulopathy in combat casualties. The differential increase in MV in patients with explosive injuries may contribute to poorer outcomes in this group

**Funding**: Higher degree research funding was provided for A Sharrock by The Drummond Foundation, Surrey, UK, and the Royal Centre for Defence Medicine, Birmingham, UK.

PF08.09

VEGFR2 shed from human umbilical vein endothelial cells on inside-out extracellular vesicles

Sukhbir Kaur ^1^; Abdel G. Elkahloun^2^; Satya P. Singh^3^; David D. Roberts1^1^

^1^Laboratory of Pathology and Laboratory of Experimental Carcinogenesis, Center for Cancer Research, National Cancer Institute, National Institutes of Health, Bethesda, MD, USA; ^2^Cancer Genetics Branch, National Human Genome Research Institute, National Institutes of Health, Bethesda, MD, USA; ^3^Inflammation Biology Section, National Institute of Allergy and Infectious Diseases, National Institutes of Health, Bethesda, MD, USA

**Background**: Extracellular vesicles (EVs) are mediators of intercellular communication that exhibit diversity in their biomarkers, macromolecular contents, function and origin. Exosomes and ectosomes are the best-characterized EVs and share a membrane topology with their cells of origin. Here we report a class of inside-out vesicles that express vascular endothelial growth factor receptor-2 (VEGFR2).

**Methods**: EVs expressing VEGFR2 released by endothelial cells and present in human plasma were characterized using Western blotting, flow cytometry analysis and electron microscopy. The RNA content of VEGFR2+ and VEGFR2− EVs was analysed using microarray analysis.

**Results**: Human umbilical vein endothelial cells release 100–200-nm vesicles that are recognized by an antibody specific for the cytoplasmic domain but not the extracellular domain of VEGFR2 and are distinct from CD63+ EVs. The results suggest that these EVs are inside out. VEGFR2+ also contains HSP-90 and flotillin-1. Their non-coding and messenger RNA contents differ from that of conventional EVs released from the same cells.

**Summary/Conclusion**: Most VEGFR2 is present on a subset of inside-out vesicles released by endothelial cells that can also be found in human plasma. c-VEGFR2 terminal antibodies can be useful for identification of EV-associated VGEFR2 in pathological blood or liquid biopsy specimens.

**Funding**: This work was funded by the NIH Intramural Research Program ZIA SC 009172 (DDR).

PF08.10

hAFS-EV cardioactive potential for myocardial regeneration

Carolina Balbi ^1^; Kristen Lodder^2^; Lucio Barile^3^; Luisa Pascucci^4^; Marie Josè Goumans^2^; Anke M Smits^2^; Sveva Bollini^1^

^1^Department of Experimental Medicine, University of Genova, Genova, Italy; ^2^Department of Molecular Cell Biology, Leiden University Medical Center, Leiden, The Netherlands; ^3^Laboratory of Cellular and Molecular Cardiology, Fondazione Cardiocentro Ticino, Lugano. Swiss institute for Regenerative Medicine (SIRM),, Lugano, Switzerland; ^4^Veterinary Medicine Department, University of Perugia, Perugia, Italy

**Background**: Growing interest has been driven to stem cell-derived extracellular vesicles (EV) as mediators of paracrine effects. Human amniotic fluid stem cells (hAFS) are immature progenitors with significant cardioprotective potential in promoting cardiomyocyte survival under ischaemic or drug-induced cardiotoxic injury. We recently reported first characterization of hAFS-EV as biological mediators of pro-survival, proliferative and anti-inflammatory effects. Here, we address functional reactivation of resident cardiac progenitor cells and cardiomyocyte proliferation by hAFS-EV.

**Methods**: hAFS were isolated from leftover samples of amniotic fluid from prenatal diagnosis, according to Helsinki declaration. hAFS were cultured for 24 h in hypoxic and serum-free condition to improve EV secretion. EV were isolated by ultracentrifugation from hAFS-conditioned medium (hAFS-CM) and their microRNA (miRNA) content characterized. To pinpoint EV therapeutic role, a myocardial infarction (MI) mouse model was treated with hAFS-CM, with hAFS-CM depleted by EV (hAFS-DM) and with hAFS-EV.

**Results**: hAFS secrete EV of 100–1000 nm in size expressing CD81, CD63, TSG101 and ALIX. Their intra-myocardial delivery soon after MI resulted in the increase of pro-regenerative miR-146a (*p < 0.05), miRNA-210 and miR-199a in cardiac host cells, 3 h after injection. Seven days post MI mice receiving hAFS-EV showed improvement of left ventricle ejection fraction by 88% (*p < 0.05), compared to controls. EV alone recapitulated beneficial effects of the whole hAFS secretome (hAFS-CM vs. hAFS-EV: *p* > 0.05). hAFS-EV and hAFS-CM, but not hAFS-DM, stimulated resident cardiomyocyte proliferation by twofold compared to controls (*p < 0.05). WT1+ epicardial progenitor cells (EPDC) may play a pivotal role in sustaining cardiac regeneration; however, they are usually quiescent in the adult tissue; following either hAFS-CM or hAFS-EV stimulation, endogenous WT1+ GFP+ EPDC significantly increased (*p < 0.05 and ***p < 0.001 respectively) compared to controls, in WT1 epicardial lineage trace mice 7 days after MI.

**Summary/Conclusion**: These results substantiate delivery of EV bioactive factors to trigger endogenous cardiac regeneration. hAFS-EV recapitulate most of the whole hAFS secretome cardioactive potential, thus representing an appealing tool for future translational cardiovascular therapy.

**Funding**: The research was funded by Programma Giovani Ricercatori “Rita Levi Montalcini” 2012 from the Italian Ministry of Education and Research.

PF08.11

Distinct anti-fibrotic effects of exosomes derived from normoxia and hypoxia cultured-endothelial colony-forming cells

Wenhao Liu ^1^; Zhiteng Chen^1^; Haifeng Zhang^2^; Yanxin Chen^1^; Jingfeng Wang^1^

^1^Sun Yat-sen Memorial Hospital, Sun Yat-sen University, Guangzhou, People’s Republic of China; ^2^The University of British Columbia, Vancouver, Canada

**Background**: The therapeutic potential of endothelial colony-forming cells (ECFCs) could be impaired during ischaemic environment which constrain its reparative ability. Exosomes are accepted to be important in intercellular communications and promising to be may be an important therapeutic tool. However, the differences between exosomes derived from hypoxia and normoxia ECFCs were unknown. The purpose of this study was to investigate the alterations of anti-fibrotic effects of hypoxia-treated ECFC-derived exosomes and the underline mechanism.

**Methods**: ECFCs were isolated from peripheral blood and conditioned mediums were collected after 72 h incubation in normoxia or hypoxia chamber, respectively. Exosomes were derived from both normoxia- (nor-exo) and hypoxia (hyp-exo)-treated ECFCs. Isolated exosomes were injected from caudal vein of myocardial infarction rats and left ventricular function and fibrosis were assessed. Effects of exosomes on cardiac fibroblasts (CFs) activations were also evaluated. microRNAs (miRNAs) inside exosome were extracted and compared using next-generation RNA sequencing, which were confirmed by PCR. Targets of identified miRNA were validated using dual-luciferase reporter gene assay.

**Results**: Nor-exo significantly improves cardiac function, released cardiac fibrosis *in vivo* and ameliorated CFs activation *in vitro*. All of these effects were dramatically attenuated in hyp-exo-treated group. Next-generation RNA sequencing identified a total of 1861 miRNA expression differences between the two exosomes populations. PCR confirmed that miR-10b-5p, which is abundantly expressed in nor-exo, was suppressed to the most extent in hyp-exo. miR-10b-5p significantly attenuated activation of CFs and downregulated fibrosis-related gene *Smurf1* and *HDAC4*. Dual-luciferase reporter gene assay validated that miR-10b-5p binded to 3′UTR of *Smurf1* and *HDAC4* and thus inhibited their expressions.

**Summary/Conclusion**: Anti-fibrotic effects of exosomes from hypoxia ECFCs were dismissed, at least because of decreased miR-10b-5p inside them. This study deepens our understanding of the response of ECFCs to hypoxia and tries to give a novel explanation of stem cells dysfunction in ischaemic organ.

**Funding**: This work was supported by the National Natural Science Foundation for Jing-feng Wang (Grant No. 81570213).

PF08.12

Von Willebrand factor and thrombospondin-1 in exosomes derived from blood outgrowth endothelial cells in ischaemic heart disease

Arief Wibowo ^1^; Stefan Janssens^1^; Jozef Bartunek^2^

^1^KU Leuven, Leuven, Belgium; ^2^KU Leuven, Aalst, Belgium

**Background**: Blood outgrowth endothelial cells (BOECs) mediate therapeutic neovascularization in experimental models. We hypothesized that BOECs promote angiogenesis via secretion of exosomes.

**Methods**: BOECs were isolated from the peripheral blood of patients with severe ischaemic heart disease and were exposed to hypoxia (1% O_2_) or normoxia for 12 h. Exosomes were isolated from the medium by differential ultracentrifugation. Size and the number of exosomes were determined by nanoparticle tracking analysis (NTA) and immunoblot analysis of the surface markers of exosomes. Matrigel 2D-tube formation assay was performed to explore the angiogenic potential of HUVECs in the presence or absence of BOEC-derived exosomes. qPCR analysis was performed to investigate transcript levels of angiogenic factors both in BOECs and in BOEC-derived exosomes. Proteomics analysis was performed to investigate protein level of angiogenic factors in BOECs in both patients and controls. We validated proteomics results with ELISA.

**Results**: Quantification of exosomes by NTA showed higher concentrations of exosomes in the medium after hypoxia compared to normoxia (14.38 ± 0.23 × 10E8 vs. 12.05 ± 0.23 ×10E8 particles/ml, *n* = 7, *p* = 0.0004). Immunoblot analysis confirmed robust expression of the exosome markers TSG101 and Flotilin-1. 2D-tube formation assay indicated an increased mature vascular network after 4 h exposure to BOEC-derived exosomes when compared to negative control conditions (*p* < 0.001). qPCR analysis indicated that angiogenic transcripts for VEGFA, PLGF, MCP-1 and ANG2 were uniformly present in all exosome. Proteomics study of BOEC-derived exosomes reveals von Willebrand factor (vWF) and thrombospondin-1 (THBS1) as the most abundantly identified proteins in ischaemic cardiomyopathy patients and control, respectively, and increase during hypoxia. ELISA results confirmed the exosomal content of vWF and THBS1.

**Summary/Conclusion**: Our results suggest that BOEC-derived exosomes can be effectively taken up by neighbouring cells, and induce vascular network formation. Further research including *in vivo* studies is needed to investigate the underlying mechanism of the pro-angiogenic effects.

**Funding**: This work was funded by Initial Training Networks, Marie Skłodowska–Curie Actions – Research Fellowship Programme and KBS, the King Boudain Foundation, VZW Cardiovascular Research Center, Aalst.

PF08.13

Exosomes from adipose-derived stem cells induce angiogenesis

Xin Dai^1^; LIna Zhao^2^; Dong Liu ^1^

^1^Morehouse School of Medicine, Atlanta, GA, USA; ^2^Zhejiang University, The Second Affiliated Hospital, Hangzhou, People’s Republic of China

**Background**: The worldwide epidemic of ischaemic heart diseases urgently requires innovative treatments in spite of the significant advances in medical, interventional and surgical therapy for these diseases. The emergence of stem cell-based therapeutic strategies may represent a promising outlook for patients with cardiovascular disease, particularly in the setting of myocardial infarction. Cell secretion is an important mechanism for stem cell-based therapeutic angiogenesis along with cell differentiation to vascular endothelial cell or smooth muscle cell. Cell-released exosomes have been recently implicated to play an essential role in intercellular communication. The purpose of this study is to explore the potential effects of stem cell-released exosomes in angiogenesis.

**Methods**: Adipose-derived stem cells (ASCs)-secreted exosomes were collected with an exosome precipitation solution. Exosomes were identified with transmission electron microscopy, nanosight analysis and immunoblotting for an exosome marker, Alix. We observed that labelled exosomes were efficiently delivered into target cells using fluorescent microscopy and flow cytometry.

**Results**: Exosomes increased the proliferation, migration and tube formation of human microvascular endothelial cells (HMVECs). Cell migration was determined by a combined use of membrane-based Boyden chamber, propidium iodide-staining and software-assisted counting of nuclei of migrated cells. Tube formation assay was performed by counting the tube length of migrated HMVECs on matrigel. Hypoxia-preconditioning of ASCs upregulated the secretion of exosomes and enhanced the angiogenic effect of the released exosomes.

**Summary/Conclusion**: Our findings provide the first evidence that exosomes from ASCs, particularly from hypoxia-preconditioned ASCs, promote angiogenesis *in vitro*.

**Funding**: This work was supported by NIH grants SC1HL134212, P50HL117929, G12MD007602 and SC2GM099629.

PF09: EVs, Pathogens and Cross Organism Communication Chairs: Anush Arakelyan; Joanne Lannigan Location: Exhibit Hall 17:15–18:30

PF09.01

Outer membrane vesicles of *E. coli*-mediated resistance to ampicillin by carrying resistant genes and proteins

Nader Kameli; Erik Beuken; Paul Savelkoul; Frank Stassen

Maastricht University, Maastricht, The Netherlands

**Background**: Antibiotic-resistant bacteria are one of the biggest threats in modern medicine. Understanding the mechanisms of resistance as well as the transmission of resistance genes is crucial for the development of new classes of antibiotics. Outer membrane vesicles (OMVs), which are released by Gram-negative and -positive bacteria, have been found to play crucial roles in bacterial pathogenicity. Recent studies suggested that MVs are involved in the protection against antibiotic-mediated killing.

**Objectives**: Here we hypothesize that OMVs contribute to antibiotic resistance. First, we aim to demonstrate the presence of resistant genes and functional enzymes within outer membrane vesicles. Then we will investigate whether OMVs can protect susceptible *E. coli* from antibiotics-meditated killing.

**Methods**: One *E. coli* strain with plasmid encoding the beta-lactamase CTX-M-15 resistance gene and susceptible *E. coli* strain were used. Antibiotic susceptibility profiles of the strains were determined using a VITEK®2 system. OMVs were isolated from bacterial cultures by a combination of ultrafiltration and size-exclusion chromatography. The presence of OMVs was confirmed by tunable resistive pulse sensing in addition to the Bradford assay to determine the protein content. PCR and nitrocefin assays were used to detect resistance gene and active beta-lactamase, respectively. Disc diffusion test and microtiter plate test were used to investigate the efficacy of antibiotics and protection respectively when ampicillin was pre-incubated with OMVs derived from resistant or susceptible bacteria.

**Results**: Our data show that *E. coli* releases a significant amount of OMVs during 18 h of culturing. Also, we could demonstrate the presence of DNA and most importantly resistance genes and functional beta-lactamase protein inside the MVs.

**Summary/Conclusion**: Interestingly, OMVs derived from resistant bacteria decrease the efficacy of ampicillin and enhance the growth of susceptible *E. coli* when exposed to ampicillin comparing with OMVs derived from wild-type *E. coli* or phosphate-buffered saline. This finding emphasizes the contribution of OMVs in antibiotics resistance as an important virulence factor for bacterial surviving.

PF09.02

Biofilm-related sRNAs are differentially encapsulated in membrane vesicles from *Pseudomonas aeruginosa* PAO1

Carla Perez-Cruz ^1^; Ferran Brianso^2^; Elena Mercade^1^

^1^Department of Biology, Health and Environment, University of Barcelona, Barcelona, Spain; ^2^Statistics and Bioinformatics Unit (UEB), Vall d’Hebron Research Institute (VHIR), Barcelona, Spain

**Background**: Membrane vesicles (MVs) are spherical structures (20–200 nm) that are secreted from the outer membrane of Gram-negative bacteria to deliver bacterial effectors to distant cells. They are implicated in several functions such as pathogenesis and horizontal gene transfer. Moreover, MVs play an important role in biofilm development through the secretion and delivery of quorum sensing signals. Recent publications describe the presence of regulatory small RNA (sRNA) in MVs, although their role is still unknown. The aim of the current work is to identify and determine the function of sRNAs associated with MVs from *Pseudomonas aeruginosa* PAO1, related to their possible implication in biofilm formation.

**Methods**: RNA and MVs isolation, Bioanalyzer, Qubit RNA HS, RT-qPCR, high-resolution flow cytometry, RNA-seq, transfer, Cryogenic transmission electron microscopy.

**Results**: To date, we have determined that MVs can package sRNAs (25–100 nt). Then, we confirmed that sRNAs are encapsulated inside MVs through RNase protection assay. Moreover, we have proved that these RNAs are stable inside MVs after an overnight incubation at 37°C. By high-resolution flow cytometry, we have enumerated the number of MVs during bacterial growth, finding that MVs concentration increases during the transition to stationary phase, while it decreases in later stationary phase. Then, we have monitored the expression of three PAO1 sRNAs implicated in biofilm formation (PhrS, CrcZ and RsmZ) and we found that they were differentially packaged inside MVs depending on the growth phase. To directly identify the sRNAs in MVs, sequencing of total RNA extracted from MVs obtained at different growth points have been performed. The final step is to demonstrate that functional sRNA can be delivered to PAO1 cells by MVs.

**Summary/Conclusion**: Differential encapsulation of sRNA inside *Pseudomonas aeruginosa* PAO1 MVs has been proved and opens up to study whether MV-associated sRNAs could play a role in cell-to-cell communication.

**Funding**: This work was funded by the grant CTQ2014-59632-R from the Ministerio de Economia y Competitividad, Spain, and CPC was a recipient of the fellowship APIF2015 from the UB. The funders have no role in study design, data collection and analysis, decision to publish, or preparation of the abstract.

PF09.03

Iron restriction is central to nutritional immunity, but does it affect the extracellular vesicles of bacterial pathogens?

Simon Swift ^1^; Priscila Dauros-Singorenko^2^; Jiwon Hong^3^; Alana Whitcombe^3^; Denis Simonov^3^; Peter Tsai^3^; Cristin Print^3^; Matthew Kang^4^; Anthony Phillips^2^

^1^University of Auckland, Grafton, New Zealand; ^2^School of Biological Sciences, University of Auckland, Auckland, New Zealand; ^3^University of Auckland, Auckland, New Zealand; ^4^Department of Obstetrics and Gynaecology, University of Auckland, Auckland, New Zealand

**Background**: Bacterial pathogens produce extracellular vesicles (EVs) that carry a cargo of potential virulence factors deployed in an infection. We hypothesize that the production of EVs and their cargo change depending upon environmental conditions. Iron restriction represents one host parameter that is an important barrier to infection, a process termed nutritional immunity. Many known effectors of bacterial virulence are upregulated during growth under iron restriction.

**Methods**: We investigated the EVs produced by uropathogenic *Escherichia coli* cultured in iron-restricted and iron-replete conditions. EVs were purified by density gradient centrifugation and analysed by transmission electron microscopy, nanoparticle tracking analysis and for LPS, DNA, RNA and protein content. RNA sequencing and proteomic approaches were applied to obtain a more detailed view of the RNA and protein content. The effect of EV RNA on cultured bladder epithelium cells was determined at the transcriptional level by the application of Clariom S Microarrays (Affymetrix) after lipofectamine transfection of the EV RNA.

**Results**: We did not observe any striking differences in the quantity or size of EVs produced, or the gross amounts of EV-associated LPS, DNA, RNA or protein for the different culture conditions. Analysis of the RNA and protein cargoes of EVs identified some components that were consistently enriched in samples grown in the presence or absence of iron. Differential transcriptional signatures were observed from cultured bladder cells depending upon whether they were challenged with EV RNA prepared from iron-replete or iron-restricted cultures.

**Summary/Conclusion**: We conclude that iron restriction influences the EVs produced by bacteria, and that this may have functional implications during the progression of an infection.

**Funding**: This work was funded by Health Research Council of New Zealand Explorer Grant, Lottery Health Research of New Zealand Project Grant, and a New Zealand Ministry of Business, Innovation and Employment Smart Ideas Grant.

PF09.04

The effect of extracellular vesicles from *Staphylococcus aureus* and *Staphylococcus epidermidis* on RAW264.7 macrophages

Forugh Vazirisani; Karin Ekström; Peter Thomsen

Department of Biomaterials, Institute of Clinical Sciences, Sahlgrenska Academy, University of Gothenburg, Gothenburg, Sweden

**Background**: The majority of biomaterial-associated infections (BAI) are caused by the Gram-positive bacteria *Staphylococcus aureus* (*S. aureus*) and *Staphylococcus epidermidis* (*S. epidermidis*). Lately, it has been reported that extracellular vesicles (EVs) are secreted from Gram-positive bacteria for multiple purposes such as delivery of toxins and bacterial components to the host cells. Osteoclasts are responhsible for bone resorption. It has been shown that *S. aureus* protein A (*SpA*) mediates bone loss in osteomyelitis. The aim of the present study was to study the effects of these EVs on the viability of RAW264.7 macrophages and the differentiation of these cells to osteoclasts.

**Methods**: EVs were isolated from *S. aureus*, and *S. epidermidis* cultures (109 CFU/ml) and characterized by Western blot, electron microscopy and nanoparticle tracking analysis. RAW264.7 cells were seeded in 96-well plates (10,000 cells/well) and stimulated further in medium with or without RANKL (5 ng/ml). Different doses of EVs (0, 5, 20 and 50 µg/ml) were added to cells and cell viability was evaluated using propidium iodide in a NucleoCounter® or by Neutral red (NR) staining. The effect of EVs on differentiation of RAW264.7 cells to osteoclasts was evaluated by TRAP staining after 7 days.

**Results**: The size of secreted vesicles was ~100 nm. Protein A, SCP-A, α- and δ-toxins were detected in *S. aureus* EVs while *S. epidermidis* EVs contained only δ-toxin. Staphylococcal EVs (5–50 µg/ml) decreased the viability of RAW264.7 cells as analysed by both NR uptake and NucleoCounter®. However, EVs did not affect the differentiation of viable cells into osteoclasts.

**Summary/Conclusion**: The size of secreted vesicles was ~100 nm. Protein A, SCP-A, α- and δ-toxins were detected in *S. aureus* EVs while *S. epidermidis* EVs contained only δ-toxin. Staphylococcal EVs (5–50 µg/ml) decreased the viability of RAW264.7 cells as analysed by both NR uptake and NucleoCounter®. However, EVs did not affect the differentiation of viable cells into osteoclasts.

PF09.05

Shiga toxin interactions with microvesicles

Annie Villysson ^1^; Anne-Lie Ståhl^1^; Ludger Johannes^2^; Daniel Gillet^3^; Diana Karpman^1^

^1^Department of Pediatrics, Clinical Sciences Lund, Lund, Sweden, Lund, Sweden; ^2^Institut Curie, PSL Research University, U1143 INSERM, UMR3666 CNRS, Paris, France; ^3^SIMOPRO, CEA, Université Paris-Saclay, France, Paris, France

**Background**: Shiga toxin (Stx)-stimulated blood cells are activated and shed microvesicles that may carry the toxin to other cells, thereby evading the host response. Toxin can be taken up by target cells, such as renal cells, within microvesicles, wherein the toxin is released, ultimately leading to cell death.

**Methods**: This study examined shedding of toxin-positive microvesicles from toxin-stimulated cells. Furthermore, as toxin circulates in blood cell-derived microvesicles, the capacity of the toxin to bind to microvesicles in plasma, in the absence of cells, was investigated.

**Results**: HeLa cells stimulated with Stx1B released toxin-positive microvesicles within 5–10 min, detected by flow cytometry and live cell imaging. In the presence of Retro 2.1, that blocks retrograde trafficking of the toxin to the Golgi, toxin-positive microvesicles increased over time, suggesting that toxin incorporation in microvesicles can occur before transfer to the Golgi. The presence of the Gb3 receptor on microvesicles from HeLa cells and blood cells were demonstrated by thin layer chromatography and Stx overlay. Stx1B was shown to bind directly to blood cell-derived microvesicles, even in the presence of plasma, demonstrated by electron microscopy and flow cytometry.

**Summary/Conclusion**: The results indicate that Stx is instantly shed in microvesicles from toxin-stimulated cells and thereafter continuously shed, presumably in order to rid cells of toxin. Moreover, circulating blood cell-derived microvesicles may bind toxin directly. These mechanisms may explain how toxin is transferred to target organs.

PF09.06

Characterization of extracellular vesicles produced by vaginal microorganisms

Anastasiia Artuyants; Anthony Phillips; Augusto Simoes-Barbosa

School of Biological Sciences, University of Auckland, Auckland, New Zealand

**Background**: The human vagina is known to host a vast number of bacteria, both commensals and pathogens. It is accepted that the microbiota of healthy woman is generally represented by lactobacilli. A more diverse group is mostly formed by anaerobic microorganisms that cause bacterial vaginosis (BV). *In vivo* co-existence of these microorganisms suggests that they might engage in some type of communication between themselves and likely with the host using extracellular vesicles (EVs) as mediators. In this study, we focused on the evaluation of EVs production from representatives of normal and BV conditions – *Lactobacillus gasseri* ATCC 9857 and *Gardnerella vaginalis* ATCC 14018.

**Methods**: “Crude” preparations from bacterial cultures were used for further purification and fractionation by density gradient centrifugation (DGC) or size-exclusion chromatography (SEC). Particles, protein and RNA were quantified. Nanoparticle tracking analysis, polyacrylamide gel electrophoresis and transmission electron microscopy (TEM) were used to characterize vesicles in purified fractions.

**Results**: Both bacteria released EVs with a size of ~100 nm. *G. vaginalis* produced a higher number of vesicles than *L. gasseri* (1.5 × 10^12^/ml and 2.4 × 10^11^/ml, respectively). Higher protein concentration was also found in *G. vaginalis* vesicles. RNA was detected in EVs from both bacteria, although *G. vaginalis* contained mainly small RNA, whereas *L. gasseri* vesicles had rRNA peaks.

When comparing purification methods, DGC consistently resulted in five (*L. gasseri*) or four (*G. vaginalis*) fractions. For *L. gasseri*, the third fraction contained most of the particles and protein. While for *G. vaginalis* there was no correlation between particles and protein enrichment, the recovery of protein remained high. On the other hand, SEC method demonstrated the opposite trend with protein–particles correlation only for *G. vaginalis*-derived vesicles. TEM showed properly structured membrane vesicles in particle-rich fractions regardless of species or purification method.

**Summary/Conclusion**: We established the ability of two vaginal bacteria to release EVs, which could indicate their active involvement in association with host organism. We also characterized these vesicles and showed that purification methods are species dependent and should be optimized for each type of microorganism.

PF09.07

Isolation and characterization of serum extracellular vesicles (EVs) from Atlantic salmon infected with *Piscirickettsia salmonis*

Leidy Lagos ^1^; Julia Tandberg^2^; Alexander Kashulin-Bekkelund^3^; Duncan Colquhoun^4^; Henning Sørum^3^; Hanne Cecilie Winther-Larsen^5^

^1^Norwegian University of Life Sciences, Ås, Norway; ^2^University of Oslo, Oslo, Norway; ^3^Norwegian University of Life Sciences, Oslo, Norway; ^4^Veterinary Institute, Oslo, Norway; ^5^University of Oslo, OSLO, Norway

**Background**: Secretion of extracellular vesicles (EVs) is a common feature of both eukaryotic and prokaryotic cells. Isolated EVs have been shown to contain different types of molecules, including proteins and nucleic acids and reported to be key players in intercellular communication. Little is known, however, of EV secretion in fish, and the effect of infection on EV release and content. In the present study, EVs were isolated from the serum of healthy and *Piscirickettsia salmonis-*infected Atlantic salmon in order to evaluate the effect of infection on EV secretion. *P. salmonis* is facultative intracellular bacterium that causes a systemic infection disease in farmed salmonids.

**Methods**: EVs isolated from both infected and non-infected fish had and average diameter of 230–300 nm as confirmed by transmission electron microscopy, nanoparticle tracking analysis and flow cytometry.

**Results**: Mass spectrometry identified 167 proteins in serum EVs from both groups of fish. Interestingly, 35 unique proteins were identified in serum EVs isolated from the fish infected with *P. salmonis*. These unique proteins included proteasomes subunits, granulins and major histocompatibility class I and II.

**Summary/Conclusion**: Our results suggest that EV release could be part of a mechanism in which host stimulatory molecules are released from infected cells to promote an immune response.

PF09.08

Circulating miRNAs in plasma extracellular vesicles are potentials biomarkers in resistance to HIV-1 infection

Luanda Mara da Silva Oliveira ^1^; Josenilson Feitosa de Lima^1^; Fabio Seiti Yamada Yoshikawa^1^; Liã Barbara Arruda^1^; Bosco Christiano Maciel da Silva^1^; Fernanda de Mello Malta^2^; Alberto Jose da Silva Duarte^1^; Maria Notomi Sato^1^

^1^Laboratory of Dermatology and Immunodeficiencies (LIM-56), Dermatology Department, Tropical Medicine Institute, Medicine School of University of São Paulo, São Paulo, Brazil; ^2^Laboratory of Gastroenterology and Tropical Hepatology, Department of Gastroenterology, Tropical Medicine Institute, Medicine School of University of São Paulo, São Paulo, Brazil

**Background**: Extracellular vesicles (EVs) can mediate communication and information exchange between cells by carrying genetic materials. MicroRNAs (miRNAs) are small non-coding RNAs involved in post-transcriptional regulation of gene expression and they can play important roles in viral infections. Alterations of specific miRNAs were described in HIV infection. Since EVs are abundantly found in plasma, their miRNA profile could be a biomarker in HIV infection. Here, we investigated whether a particular miRNA signature could be associated to natural resistance to HIV-1.

**Methods**: We isolated EVs from 800 µL of plasma of five HIV-1 exposed uninfected individuals (EU), five HIV typical progressors (TP), seven HIV elite controllers (EC) and four healthy controls by using exoRNeasy serum/plasma kit. The expression of 84 miRNAs associated to inflammatory response and autoimmunity was analysed by RT-qPCR (miScript miRNA PCR Array). Expression levels were calculated from Ct values by the Livak method.

**Results**: We observed that infection and/or exposure to HIV-1 alters the expression profile of miRNAs in circulating EVs. An increased expression of miR-125b-5b in EC group compared to TP was detected, suggesting that this miRNA may be associated to the higher resistance of EC to the virus. In addition, levels of miR-372 and miR-449b-5p expression were reduced in EU compared with HD individuals, indicating that HIV-1 may leave a molecular imprint in exposed individuals even without establishing an infection.

**Summary/Conclusion**: Our study suggests that miRNA content in EVs can be altered HIV-1 infection. Monitoring of miRNAs in circulating EVs could track the exposure to HIV-1 in uninfected individuals and these miRNAs are also a potential biomarker for prediction of resistance to HIV-1 infection.

**Funding**: This work was supported by São Paulo Research Foundation (FAPESP, Grants 2016/10552-2 and 2015/00263-7) and National Council for Scientific and Technological Development (CNPq, Grant 435262/2016-5).

PF09.09

Oncogenic retroviral protein is conveyed by extracellular vesicles in ovine lung cancer

Fabienne Archer ^1^; Alexandra Erny^1^; Kathy Gallay^2^; Maryline Gomes^2^

^1^Univ Lyon1 -INRA- EPHE, Lyon Cedex 07, France; ^2^UMR754, Lyon Cedex 07, France

**Background**: Retroviruses exploit cellular machinery to propagate and modulate the immune response. Striking similarities have been observed in the generation and dissemination of retroviruses and small extracellular vesicles (EVs) by the host cells. EVs are thought to facilitate intercellular communication processes and transfer RNA, DNA, retrotransposon and protein. They can mediate immune functions or mask virus components from immune surveillance. They can also contribute to the horizontal transfer of oncogenes or pathogenic elements such as virus elements or prion, inducing deregulation of the recipient cell and propagation of the disease. JSRV is an oncogenic retrovirus that transform lung epithelial cell via the oncogenic properties of its envelope protein. It causes an adenocarcinoma in small ruminants. Among the cells isolated from the virus-induced tumours, we have isolated a population of uninfected but transformed cells, suggesting a transformation by a mechanism different from the retroviral infectious cycle. We hypothesize that EVs may recruit the JSRV envelope protein and participate in the deregulation of lung cells.

**Methods**: We have characterized EVs released by cells expressing the envelope protein and evidenced the presence of the protein and its mRNA (Western Blot, RT-PCR, electron microscopy).

**Results**: We are currently studying the impact of this cargo on cell proliferation and transformation, on Akt/p70S6K signalling pathway activation and cytokines production by the recipient cells. Retrovirus and exosomes have the same size and densities, and express very similar contents which complicated their separation. In order to better understand their respective role in the infectious/tumoural process, we are currently characterizing these different populations.

**Summary/Conclusion**: These results should provide more insight into the retrovirus propagation strategies.

**Funding**: This work was funded by FINOVI.

PF09.10

Human cytomegalovirus-infected cells release extracellular vesicles that carry viral surface proteins

Anush Arakelyan ^1^; Soina Zicari^1^; Wendy Fitzgerald^1^; Christophe Vanpouille^1^; Anna Lebedeva^2^; Alain Schmitt^3^; Morgane Bomsel^4^; William Britt^5^; Leonid Margolis^6^

^1^Section of Intercellular Interactions, Eunice-Kennedy National Institute of Child Health and Human Development, Bethesda, MD, USA; ^2^Evdokimov University of Medicine and Dentistry, Moscow, Russia; ^3^EM Facility,U1016INSERM,UMR 8104 CNRS Cochin Institute, Paris Descartes University, Paris, France; ^4^Mucosal entry of HIV and mucosal immunity, Cochin Institute, Paris Descartes University, Paris, France; ^5^Departments of Pediatrics, Microbiology, and Neurobiology, University of Alabama School of Medicine, Birmingham, AL, USA; ^6^Eunice-Kennedy National Institute of Child Health and Human Development, Bethesda, MD, USA

**Background**: Extracellular vesicles (EVs) are released by many if not by all cells in the human body. These EVs incorporate, from the cell of origin, various cellular molecules and proteins. If a cell is infected with a virus, EVs can incorporate viral proteins as well. Upon interactions with cells, EVs carrying viral proteins may trigger various physiological responses. Here, we show that EVs carry human cytomegalovirus (HCMV) envelope proteins that are essential for HCMV infectivity.

**Methods**: We isolated EVs from UL32-EGFP-HCMV viral suspension, produced by MRC-5 cells, using an OptiPrep step-gradient. We analysed EVs that were concentrated between 10% and 15% of the OptiPrep gradient for carrying HCMV proteins by staining them with antibodies specific for gB and gH, two viral envelope glycoproteins present on the surface of HCMV. All lipidic particles were labelled with a fluorescent dye (DiI) to distinguish HCMV virions that were GFP-positive/DiI-positive from EVs that were GFP-negative/DiI-positive.

**Results**: Flow analysis demonstrated that EVs constituted 99.7 ± 0.1% (*n* = 3) of the total events, while 0.3 ± 0.1% (*n* = 3) were UL32-EGFP-HCMV. Next, we analysed DiI-labelled EVs for the presence of HCMV surface proteins by staining with anti-gB AF647 antibodies and with anti-gH PB antibodies or with their isotype controls IgG AF647 and IgG PB. Labelled EVs were analysed with flow cytometer, triggering on DiI fluorescence. On average, 15 ± 3.7% (*n* = 3) of EVs were positive for gB and 5.3 ± 2.3% (*n* = 3) were positive for gH HCMV surface proteins and 3.74 ± 1.5% (*n* = 3) were positive for both gB and gH.

**Summary/Conclusion**: EVs released from HCMV-infected cells carry viral surface proteins. Production by infected cells of EVs carrying various viral proteins is a general phenomenon for various viruses. Understanding of the exact details and molecular mechanisms of this contribution may reveal new therapeutic targets.

**Funding**: The work of AA, SZ, WF, CV, AL and LM was supported by the NICHD/NIH Intramural Program. The work of AL was also supported by the Russian Federation Government grant #14.B25.31.0016 and RFBR grant #16-04-017/16. The work of AS and MB was supported by ANRS (AO2015-2-17046). The work of WB was supported by NIH (1RO1AI089956-01A1).

PF09.11

Modulation of exosome content through metabolic inhibitor treatment for antiviral outcomes

Rhea C. Alonzi; Roxana Filip; Tyler Shaw; Jordan Nhan; John P. Pezacki

University of Ottawa, Ottawa, Canada

**Background**: Viruses from the Flaviviridae family such as hepatitis C virus (HCV) and dengue virus (DENV) affect millions worldwide, causing significant medical and economic burden. Through modulation of host factors, such as hepatic microRNAs, and proteins involved in lipid metabolism, host resources are diverted to achieve cellular entry, viral particle formation and propagation. Interestingly, few studies have examined changes in exosome-derived miRNA populations which may affect lipid biosynthetic pathways central to viral life cycle. This study herein assessed whether treatment of cells with small-molecule metabolic inhibitors could increase the population of exosomal miRNAs which regulate fatty acid (FA) oxidation and sterol homoeostasis, effectively limiting flavivirus replication.

**Methods**: Huh7 cells were grown in the presence of either 5-µM compound A or B, 75-µM compound C, and/or 20-µM GW4869, dimethyl sulfoxide or methanol for 24 h. Exosomes were isolated from conditioned media using standard ultracentrifugation or commercial precipitation methods and assessed using immunoblotting and nanoparticle tracking analysis. Exosomal miRNAs were profiled and compared to host-cell populations. To determine the effects of the modified exosomes on viral RNA levels and lipid metabolism, Huh7.5 cells were infected with DENV (multiplicities of infection (MOI) = 2) or HCV (MOI = 0.1) for 4–48 h prior to a 24—48-h treatment with 5–100 µg/ml of exosomes.

**Results**: Small-molecule treatments produced differential miRNA profiles in exosomes, with enhanced roles in FA biosynthesis pathways, tumour suppression and viral carcinogenesis amongst others. Subsequent treatment of infected cells with 50–100 µg/ml Compound A- and C-exosomes resulted in a marked decrease in expression of genes mediating lipid metabolism, such as *FADS1, FASN, SCD1, SREBP2*, with a corresponding decrease in intracellular HCV and DENV levels.

**Summary/Conclusion**: Preliminary results have indicated that small-molecule treatments with metabolic inhibitors, such as Compound A and C, may alter the miRNA profile of exosomes *in vitro*, generating bioactive exosomes with enhanced antiviral potential.

**Funding**: We acknowledge the financial support provided from the Natural Sciences and Engineering Research Council and Ontario Graduate Scholarship funding agencies.

PF09.12

Isolation of fungal extracellular vesicles and their potential role in cell-cell communication

Tilen Konte ^1^; Špela Petelin^1^; Simona Sitar^2^; Samo Hudoklin^3^; Ema Žagar^2^; Peter Veranic^3^; Ana Plemenitaš^1^; Metka Lenassi^1^

^1^University of Ljubljana, Faculty of Medicine, Institute of Biochemistry, Ljubljana, Slovenia; ^2^Department of Polymer Chemistry and Technology, National Institute of Chemistry, Ljubljana, Slovenia; ^3^University of Ljubljana, Faculty of Medicine, Institute of Cell Biology, Ljubljana, Slovenia

**Background**: The extracellular vesicles (EVs) released from microorganisms are gaining a lot of attention due to their potential role in intra- and inter-species communication. It has been proposed that fungal EVs secreted during human infection could even mediate immune-response modulation, host cell damage and pass over the blood–brain barrier. However, the literature on isolation and characterization of fungal EVs is still limited. In our study, we optimized the isolation of EVs from two fungal species and studied their potential role in cell-cell communication.

**Methods**: *Saccharomyces cerevisiae* and *Hortaea werneckii* cultures were inoculated at different optical densities (ODs) and grown overnight to collect EVs. Cells were removed from the media with sequential centrifugations or filtration, and supernatant was concentrated using ultrafiltration spin columns. The EVs were pelleted with ultracentrifugation and analysed with transmission electron microscopy (TEM). Asymmetric-flow field-flow fractionation (AF4) and nanoparticle tracking analysis (NTA) were used to determine the particle concentration and size distribution. EVs from osmoadapted cultures were used to test the potential induction of adaptive response in osmosensitive cells.

**Results**: No measurable amounts of EVs were detected in cultures with OD < 1.5, which were grown for <18 h. Sufficient amount of EVs was detected only after the cultures were grown for 18 h to OD >> 1.5. On TEM images, clear structures of spherical cup-shaped particles were observed. Based on AF4-MALS and NTA data, the isolated EVs had geometric radii of 62–71 nm and concentration range of 10^9^–10^12^ particles/mL.

**Summary/Conclusion**: With the optimized isolation protocol, we were able to harvest comparable amounts and morphologies of fungal EVs as in isolations from human cell lines. But did the EVs from osmoadapted fungal cells induce the adaptive response in osmosensitive cells? To learn about that, you are kindly invited to visit our poster.

**Funding**: This work was supported by Slovenian Research Agency (P1-0170)

LBF04: Late Breaking Poster Session – Pathogens Chairs: Dolores Bernal; Peter Nejsum Location: Exhibit Hall 17:15–18:30

LBF04.01

Malaria parasite-derived vesicles associate with the NF-kB signalling pathway

Mirit Biton^1^; Yifat Ofir-Birin^1^; Sefi Zargarian^2^; Neta Regev-Rudzki ^1^; Motti Gerlic^2^

^1^Weizmann Institute of Science, Rehovot, Israel; ^2^Tel Aviv University, Tel Aviv, Israel

**Summary/Conclusion**: Host human red blood cells are parasites that can exchange active cargo intercellularly among them via secreted extracellular vesicles (EVs). These EVs contain parasite and host proteins and RNA and parasite gDNA. It has been shown that the host monocyte uptake of early stage (ring)-derived parasite vesicles triggers the activation of the DNA-sensing pathway within these immune cells. Here, we provide the evidence that internalization of late-stage (trophozoite) *Plasmodium falciparum*-derived EVs by monocytes prompts the activation of a known master regulator transcription factor, nuclear factor kappa B (NF-kB). The activated NF-kB is then translocated to the nucleus to induce transcription of a target gene. As NF-kB is a coordinator of innate and adaptive immune responses, and is involved in cellular signalling of several RNA sensors, such as RIG-I and TLR3, our finding opens a new line of investigation concerning the function of the vesicle RNA cargo. Our newly discovered crosstalk mechanism strongly supports the existence of a “manipulation strategy” of the host immune environment by the *P. falciparum* parasite.

LBF04.02

Extracellular vesicles from Kaposi’s sarcoma-associated herpesvirus infected-human endothelial cells stimulate the type 1 interferon response

Hyungtaek Jeon; Seung-Min Yoo; Myung-Shin Lee

Department of Microbiology and Immunology, School of Medicine, Eulji University, Daejeon, Republic of Korea

**Background**: Kaposi’s sarcoma-associated herpesvirus (KSHV) is the etiologic agent of Kaposi’s sarcoma (KS), which is the most common cancer in AIDS patients. KSHV encodes various immune modulatory viral proteins to escape from host immune defence mechanisms. Especially, KSHV viral proteins including vIRF1, ORF45, ORF52, etc. strongly modulate the host type I interferon (IFN) response. However, IFN response is very weak during primary KSHV infection to human endothelial cells even before viral gene expression. Currently, it is not known the reason why KSHV has to evolve to manipulate type I IFN response with various viral proteins. For the first time, we demonstrated here that the extracellular vesicles (EVs) released from KSHV-infected cells is a strong stimulator for type I IFN in human endothelial cells, which would be a host defence mechanism for KSHV infection.

**Methods**: Human umbilical vein endothelial cells (HUVECs) were infected with recombinant KSHV, BAC16. EVs were isolated from the conditioned media of KSHV-infected HUVECs (KEVs) by differential ultracentrifugation methods. After KEVs were treated on uninfected HUVECs for 24 h, gene expressions were analysed by various molecular biologic techniques.

**Results**: We have developed procedures to isolate EVs in the supernatant from *de novo* KSHV-infected HUVECs without the contamination of KSHV virions. mRNA microarray showed that type I IFN signalling pathway was prominently activated in KEVs-treated uninfected HUVECs, which was validated by RT-qPCR and ELISA. We also found KSHV infection stimulates the production of the EVs up to 30-fold compared to uninfected cells, which would be a factor for type I IFN response in the microenvironment. Mechanistically, type I IFN response by KEVs would be mediated by mitochondrial DNA through cGAS-STING pathway.

**Summary/Conclusion**: EVs from KSHV-infected cells stimulate the type I IFN response through cGAS-STING pathway, which is a firstly described immune defence mechanism in KSHV-infected cells. Our results reveal that EVs from KSHV-infected cells induce antiviral immune response using intertwined mechanisms, which might be a reason for KSHV to evolve to have evasion strategies against IFN response.

**Funding**: This work was supported by a grant from the NRF of Korea (NRF-2017R1A2B4002405, NRF-2017R1A2B1006373).

LBF04.03

The response of the cells to toxin listeriolysin O and its mutants

Apolonija Bedina Zavec ^1^; Ana Špilak^1^; Matic Kisovec^1^; Maja Jamnik^1^; Veronika Kralj-Iglič^2^; Gregor Anderluh^1^; Marjetka Podobnik^1^

^1^National Institute of Chemistry, Ljubljana, Slovenia; ^2^Faculty of Health Sciences, University of Ljubljana, Ljubljana, Slovenia

**Background**: Listeriolysin O (LLO) is a toxin from the intracellular pathogen *Listeria monocytogenes*, which forms pores in cholesterol-rich lipid membranes of host cells. Large β-barrel pores formed by LLO indicate significant plasticity, from arc- or slit-shaped pores to supramolecular assemblies generating large defects in membranes. This plasticity is modulated by protein concentration, pH and temperature; therefore, LLO is interesting for the applications in medicine and biotechnology. The release of extracellular vesicles (EVs) was used to examine the response of the cells to LLO.

**Methods**: The effects of LLO and its mutants were tested on myelogenous leukemia cell line K562, which is highly sensitive *in vitro* target for the natural killer cells.

**Results**: Two mutant proteins were generated by our group, Y406A and A318C+L334C. The single mutant Y406A is able to bound to membranes and oligomerized similarly to the wild-type LLO (wtLLO), but the final membrane insertion step requires acidic pH. The double cysteine mutant A318C+L334C is, when it is in the oxidized state, locked in a certain position that pore formation cannot be complete. This mutant does not exhibit haemolytic activity in the oxidized state, but it bound to the lipid membranes to the same extent in reduced or oxidized state. Mutant Y406A was not cytotoxic at neutral pH, while at pH6 it got almost the same citotoxicity as wtLLO at pH7.4. Mutant A318C+L334C in the oxidized state was about 100-fold less citotoxic than the wtLLO, while it got about 10-fold less cytotoxicity than wtLLO, when it was in the reduced state. The level of EV secretion was significantly increased at cytolethal concentrations. Vesiculation level was also increased at about 10-fold lower concentrations than cytolethal. However, at about 100-fold lower concentrations than cytolethal, the effect was reversed and cells shedding less EVs than control cells.

**Summary/Conclusion**: Mutants are significantly less toxic than wtLLO under physiological conditions and become toxic under acidic conditions or oxidation; therefore, mutants are highly appropriate for stimuli responsive applications. EV shedding acts as the main clearance mechanism for LLO at cytolethal and subcytolethal concentrations in cell line K562; whereas at lower concentrations, the endocytosis is probably the main mechanism to prevent pore formation.

LBF04.04

*Lactobacillus plantarum*-derived extracellular vesicles improved the quality and safety of tuna meat

Wei-Hsuan Hsu ^1^; Ko-Chien Chen^2^; Tang-Long Shen^3^

^1^Industrial Technology Research Institute, New Taipei City, Taiwan (Republic of China); ^2^Department of Life Sciences, National Taiwan University, Taipei, Taiwan (Republic of China); ^3^Department of Plant Pathology and Microbiology, National Taiwan University, Taipei, Taiwan (Republic of China)

**Background**: Lactic acid bacteria such as *Lactobacillus* species are probiotics and have been widely used in dairy food with long history. Extracellular vesicles (EVs) derived from probiotics have been found to suppress the growth of poisoned bacteria, revealing that *Lactobacillus*-derived EVs may have potentials on the application of food technology. High-value crustacean with tuna fish is highly perishable due to the microbiological, biochemical or physical changes during post-mortem storage and lead to the shelf life limitation of seafood.

**Methods**: This study is to investigate the protective effect of EVs isolated from different *Lactobacillus* strains on tuna spoil, histamine production and quality lose.

**Results**: The results showed that *L. plantarum*-derived EVs protected tuna sashimi meat from rotten to against the production of total volatile basic nitrogen and total microbial numbers.

**Summary/Conclusion**: Taken together, *Lactobacillus*-derived EVs have potential on the application of seafood storage.

LBF04.05

Identification the exosomes from adipose-derived stem and progenitor cells for hypoxic brain injury

Chia-Wei Huang; Chia Ching Wu

National Cheng Kung University, Tainan, Taiwan (Republic of China)

**Background**: Perinatal cerebral hypoxic-ischaemic (HI) injury is the major cause of neonatal mortality during childbirth and resulted in severe neurological deficits in survivors. The neurovascular unit composes the main architecture of brain which is severely damaged to trigger the pathogenesis after injury. Adipose-derived stem cells (ASCs) are an ideal source for cell-based therapy with similar characteristic to the bone marrow mesenchymal stem cells. Transplantation of endothelial lineage cells (ELCs) can prevent the vascular damage and blood–brain barrier disruption. Neural differentiation of stem cell provides alternative source for neural lineage cells (NLCs).

**Methods**: ASCs can sense the microenvironmental cues for differentiating into ELCs using laminar shear stress and towards NLCs on chitosan-coated surface. Microenvironments cause cells to modulate its microRNAs (miRs) for signal transduction and differentiation.

**Results**: We recently discovered the synergic of ELCs and NLCs combination to prevent neonatal rat pups from HI brain injury. In this study, we further investigated the mechanism of miRs in ASCs differentiation and ELC-NLC interactions for the neurovascular regeneration. The miR expressions in ASCs, ELCs and NLCs were profiled to identify new miRs and their direct target genes that regulate cell differentiation in response to microenvironments. The properties of secreted exosome were characterized by nanoparticle tracking analysis and transmission electron microscopy. When treating the conditional medium to the pro-inflamed cells, different medium from stem or progenitor cells showed various therapeutic outcomes. The exosomes isolated from the combination of ELC-NLC showed best inhibition of inflammation responses and prevention of cell death in damaged endothelial cells.

**Summary/Conclusion**: Thus, the exosomes from therapeutic cells is an important mediator to prevent brain injury.

LBF04.06

Role of extracellular vesicles released by vascular endothelium on its own damage during dengue virus infection

Pedro Pablo Martínez Rojas; Verónica Monroy Martínez; Blanca Haydé Ruiz Ordaz

Biomedical Research Institute/National Autonomous University of Mexico, Mexico City, Mexico

**Background**: Dengue fever presents a broad clinical spectrum ranging from the self-limited form to severe dengue (SD) that includes the dengue shock syndrome (DSS). SD pathogenesis is characterized with high levels of cellular activation and cytokines production with plasma extravasation due to vascular endothelium damage. The endothelial cells (ECs) role is to maintain vascular homoeostasis. During dengue virus (DENV) infection, ECs may increase the release of extracellular vesicles (EVs). EVs may have important implications in vasculopathy during DSS. We propose to evaluate the role of EVs (microvesicles [MV]/exosomes) derived from DENV-infected ECs on vascular barrier (permeability).

**Methods**: DENV amplification and viral titration by lytic plate assay. Kinetics of DENV infection in human ECs (HMEC-1) at different multiplicities of infection (MOI): E protein detection by flow cytometry assay (FC). Evaluation of ECs surface markers [PECAM-1, ICAM-1, P-selectin, tissue factor (TF, CD142), CD63/CD81 and PAR-1] was performed by FC. Isolation of EVs was performed by ultracentrifugation, characterization by nanoparticle tracking analysis and transmission electron microscopy, and detection of Annexin V or CD63/CD81 by FC. Co-culture assays of EVs with EC-naïve cells were used to determine the presence of TF/PAR-1 surface receptors by FC and TNF-α/IL-8 gene expression by RT-PCR. We also evaluate the EVs effect on ECs monolayer disruption by vascular permeability assay.

**Results**: DENV cytopathic effect was shown at 7–10 days post-infection (PI). The viral titer was 1.0 × 10^7^ PFU/mL. MOI 5 at 96 h PI was the best condition achieved in DENV-infection kinetics, with 3.1% viral E protein+ cells. DENV induced ICAM-1/TF/PAR-1 overexpression at 24 h PI. No changes in PECAM-1/P-selectin expression were observed. Likewise, Annexin V+ MVs were detected. The rest of the experiments are being developed.

**Summary/Conclusion**: DENV-infected human ECs presented activation or damage markers overexpression (TF+ and PAR-1+) with MVs release.

**Funding**: This study has been supported by funding from DGAPA-PAPIIT Grant IN212014. The authors wish to acknowledge CONACYT Mexico.

LBF04.07 = OWP1.08

Impact of pathogenic microbes and healthy microbiota by *Lactobacillus*-derived extracellular vesicles

Bao-Hong Lee ^1^; Wei-Hsuan Hsu^2^; Tang-Long Shen^3^

^1^Division of Hematology and Oncology, Department of Internal Medicine, Taipei Medical University Hospital, Taipei, Taiwan (Republic of China); ^2^Industrial Technology Research Institute, New Taipei City, Taiwan (Republic of China); ^3^Department of Plant Pathology and Microbiology, National Taiwan University, Taipei, Taiwan (Republic of China)

**Background**: The potential of *Lactobacillus* strains against pathogenic microbial infection has been investigated over past decades. Extracellular vesicles (EVs) are membrane-based structure secreted from various microbes, including lactic acid bacteria. EVs serve as vehicles to carry different types of cellular cargo, such as lipids, proteins, receptors and effector molecules to the recipient cells. Recent studies have demonstrated that *Lactobacillus*-derived EVs modulate microbiota, suppress cancer cells and regulate dendritic cells, whereas their detail mechanisms remain unclear.

**Methods**: We have attempted to investigate the characteristic and anti-microbe activity of Lactobacillus-derived EVs.

**Results**: Our data showed that under a similar growth condition, the EVs production and size distribution among these three *Lactobacillus* strains, including *L. acidoplilus, L. plantarum* and *L. reuteri*, were clearly distinct. Nevertheless, these EVs were prominently capable of suppressing the growth of pathogenic microbe *Escherichia coli, Staphylococcus aureus* and *Vibrio parahaemolyticus*.

**Summary/Conclusion**: These results indicated that *Lactobacillus*-derived EVs may be applied as novel agents for maintaining or regulating healthy microbiota.

LBF05: Late Breaking Poster Session – RNA Chairs: Louise Laurent; Lorraine O’Driscoll Location: Exhibit Hall 17:15–18:30

LBF05.02

HBV-derived exosome is sysemically functional

Ai Kotani

Tokai University, Isehara, Japan

**Background**: Hepatitis B virus (HBV) induced chronic hapatitis which often causes liver chirosis and hepatocellular carcinoma. The exosome secreted from the hepatocytes which are infected by HBV seems to have a systemic roles.

**Methods**: The distribution of the exosome secreted from HBV-infected liver cells was investigated.

**Results**: Brain, lung, LN and others took the exosome.

**Summary/Conclusion**: These results suggest that the exosome secreted from the HBV-infected hepatocytes systemically works.

LBF05.03

Urinary exosomes microRNAs: a future biomarkers in lupus patients with renal involvement

Eloi Garcia Vives^1^; Cristina Solé Marcé ^1^; Marta Vidal^2^; Josefina Cortés-Hernández^1^; Josep Ordi-Ros^1^

^1^Fundacio Universitaria Institut de Recerca Vall d’Hebron (VHIR), Barcelona, Spain; ^2^Hospital Parc Taulí, Barcelona, Spain

**Background**: Kidney involvement is the most frequent manifestation of systemic lupus erythematosus. Despite an improvement in the therapeutic field, still 30% of patient’s progress to chronic renal insufficiency. Renal biopsy is still the gold standard to diagnosis and monitoring the lupus nephritis. In the recent years, different urinary biomarkers were studied but none seems to be sufficient effective to replace renal biopsies. For this reason, microRNAs in urinary exosomes could be an alternative source to find new biomarkers.

**Objective**: To study the expression of microRNAs in urinary exosomes in patients with active lupus nephritis pre- and post-treatment.

**Methods**: Urinary exosomes from urine samples were isolated using miRCURY exosome isolation kit urine in a cohort of 14 active lupus nephritis patients (pre- and post-treatment). They were characterized with Cryo-transmission electron microscopy, NanoSight and WesternBlot. MicroRNAs were extracted using miRCURY RNA Isolation Kit Cell and Plant. MicroRNA screening was carried out in a predesigned array and the patients were classified depending on their response to the treatment (remission or non-remission). Validation of differentially expressed (DE) microRNAs in urinary exosomes by qPCR-RT was done in a new cohort of patients (*N* = 43; 21 in remission and 22 in non-remission). DE microRNAs were also evaluated in serum exosomes.

**Results**: Twenty-five miRNAs showed significant differences between remission and non-remission group in the screening cohort. Validation in the new cohort and in serum exosomes samples confirmed only eight DE miRNAs. Correlation with clinical parameters showed that proteinuria has good correlation with six of them. MiR-31 (*p* = 0.041), miR-532 (*p* = 0.021), miR-107 (*p* = 0.004) and miR-135b (*p* < 0.001) were highly expressed on those patients who achieve complete remission.

**Summary/Conclusion**: This is the first screening of microRNAs in urinary exosomes from lupus nephritis patients and we have demonstrated that they could be used as predictive biomarker of clinical response.

**Funding**: This work was supported by a grant for Spain Government “Instituto de Salud Carlos III” (PI15/02117).

LBF05.04 = OWP3.09

Alterations in the miRNA cargo of HIV-infected macrophage-derived extracellular vesicles promote pulmonary smooth muscle proliferation

Himanshu Sharma; Navneet K. Dhillon; Mahendran Chinnappan; Stuti Agarwal; Pranjali Dalvi

University of Kansas Medical Center, Kansas City, Mo, USA

**Background**: Our previous studies consistently demonstrate enhanced pulmonary vascular remodelling in HIV-1 infected individuals, simian immunodeficiency virus-infected macaques and in HIV-transgenic rats exposed to illicit drugs. We reported significant perivascular inflammation around the remodeled vessels; however, the exact role of these inflammatory cells in the development of pulmonary vascular remodelling remains unknown. Our recent *in vitro* findings revealed that HIV-1-infected and cocaine (H + C)-treated human monocyte-derived macrophages (MDMs) secrete higher number of extracellular vesicles (EVs) compared to mono-treatments. We now hypothesize that dual hit of HIV-1 and cocaine may alter miRNA cargo of macrophage-derived EVs in a way that promotes smooth muscle proliferation.

**Methods**: EVs were isolated by ultracentrifugation from supernatants collected from HIV-1Bal-infected and cocaine (H + C)-treated MDMs at 4 days post-infection and used for analysis of miRNA expression. We selected five PI3/AKT signalling-associated miRNAs for analysis based on small RNA seq findings. Human primary pulmonary arterial smooth muscle cells (HPASMCs) were treated with EVs or MDM supernatants followed by proliferation assay.

**Results**: We observed a significant increase in the expression of miR130a and 27a in EVs derived from H + C-treated MDMs compared to untreated group with significantly elevated miR130a levels in H + C EVs when compared to only HIV-1 or only cocaine mono-treatments. Examining the effect of EVs on HPASMCs showed that both mRNA and protein expression of PTEN, TSC-1 and TSC-2 were significantly reduced in cells exposed to H + C EVs and this corresponded to increased activation of PI3K-AKT signalling and proliferation of smooth muscle cells. Furthermore, inhibition of miRNA130a in HPASMCs with antagomir-130a blocked the EV-mediated decrease in PTEN mRNA expression, thus confirming direct role of miR130a in modulating PTEN expression and therefore potentiating the PI3/AKT signalling-mediated cell proliferation.

**Summary/Conclusion**: In summary, our findings suggest a pivotal role of EVs derived from HIV-1-infected and cocaine-treated macrophages in modulating pulmonary smooth proliferation and this may play a crucial role in development of HIV-associated pulmonary arterial hypertension.

**Funding**: This work was supported by NIH grants R01DA034542, R01DA042715 and R01HL129875.

LBF06: Late Breaking Poster Session – Neurobiology Chairs: Chaya Brodie; Lesley Cheng Location: Exhibit Hall 17:15–18:30

LBF06.01

Progress in separation of extracellular vesicles from brain tissue of human, macaque and mouse

Yiyao Huang ^1^; Laura J. Vella^2^; Vasiliki Machairaki^3^; Linzhao Cheng^4^; Andrew F. Hill^5^; Lei Zheng^6^; Kenneth Witwer^7^

^1^Department of Molecular and Comparative Pathobiology, Johns Hopkins University School of Medicine, Baltimore, MD, USA; ^2^The Florey Institute of Neuroscience and Mental Health, Australia, Melbourne, Australia; ^3^Department of Neurology, Johns Hopkins University School of Medicine, Baltimore, MD, USA; ^4^Division of Hematology and Institute for Cell Engineering, Johns Hopkins University School of Medicine, Baltimore, MD, USA; ^5^Department of Biochemistry and Genetics, La Trobe Institute for Molecular Science, Australia, Melbourne, Australia; ^6^Department of Laboratory Medicine, Nanfang Hospital, Southern Medical University, Guangzhou, China (People’s Republic); ^7^The Johns Hopkins University School of Medicine, Baltimore, MD, USA, Baltimore

**Background**: Efficient and specific isolation of extracellular vesicles (EVs) from brain tissue will greatly facilitate both mechanistic and biomarker studies of central nervous system diseases. Recently, Vella et al. reported efficient enrichment of EVs from human brain tissue by differential and gradient density ultracentrifugation. We have now validated and modified the method, applying it to variously processed brain tissues of human, macaque and mouse.

**Methods**: Post-mortem tissues were obtained from separate studies: human (Johns Hopkins Alzheimer’s Disease Research Center, frozen), macaque (perfused, fresh) and mouse (perfused and non-perfused, frozen and fresh). Tissue was processed as previously described (300 and 10k x*g* centrifugation) and subsequent fractions 1–3 defined by sucrose gradient of 10k x*g* supernatant. Additionally, for mouse brain, size-exclusion chromatography (SEC) was applied to the 10k x*g* supernatant before applying the sample on the gradient. Protein and particle concentrations and ratios, morphology and protein markers were examined by single particle tracking (ParticleMetrix), electron microscopy, Western blotting and flow cytometry.

**Results**: As previously published, most EVs were found in gradient fraction 2 for all samples, and human EVs of acceptable purity were obtained. However, EVs from mouse and macaque brain tissues contained high amounts of negative (cellular) markers calnexin, GM130 and Bip. Adding SEC of 10k x*g* supernatants prior to gradient depleted negative markers. Perfusion resulted in no significant difference in particle numbers or total protein, but expression levels of some markers (e.g. Golgi marker GM130) were reduced.

**Summary/Conclusion**: We have validated a recently published method and observed that protocol modifications enhance separation of EVs from brain tissues including those from mice. Since several factors, both intrinsic and extrinsic, could explain the apparent differences in results between species, additional studies are needed to understand the influence of these variables. Further progress will facilitate mechanistic and applied EV studies of central nervous system disease.

**Funding**: This work was supported by the US National Institutes of Health through DA040385 and AG057430 (to KWW).

LBF06.02

Plasma-based detection of gliomas

Sabrina Roy; Julia Small; Elizabeth Lansbury; Leonora Balaj; Noah Sadik

Mass General Hospital/Harvard Medical School, Boston, MA, USA

**Background**: Glioblastomas (GBM) are among the most common and aggressive form of primary brain tumours in adults. The epidermal growth factor receptor (EGFR) is often amplified in GBM with a subset characterized by a mutation known as variant III (EGFRvIII). Prior studies have demonstrated that GBM patients release tumour-derived extracellular vesicles (EVs) into biofluids such as cerebrospinal fluid. This has paved the way for EV-associated protein and RNA-based analytics that can be used to assess tumour molecular phenotype via liquid biopsy approach. However, once a vesicle leaves a cancer cell and enters the circulation, it becomes diluted among all of the EVs originating from normal cells. Identifying enrichment protocols for cancer EVs will be essential to develop useful EV-based markers for cancer detection. Currently, no circulating biomarkers are available to diagnose GBM.

**Methods**: EVs were isolated from Gli36 glioma cells engineered to express EGFRvIII. These EVs were spiked into pooled healthy control plasma (HCP) in order to pull down EVs using magnetic beads. After capturing the bead-bound EVs, we performed droplet digital PCR to determine EGFRvIII copy number in (1) the control (mock samples), (2) the supernatant (unbound EVs) and (3) the pulled-down EVs (bound EVs).

**Results**: In pilot studies, we found that glioma EVs can be pulled down and analysed by their expression of EGFR and EGFRvIII using magnetic beads. Moreover, dilution curves and analyses of cancer EVs allowed us to establish a limit of detection (LOD) of ~10 EVs in clean PBS background – this lower LOD was also reproducible in HCP background. In GBM patients, we have shown that glioma-specific markers such as the EGFRwt and EGFRvIII, as well as Podoplanin, have been detected on the surface of tumour EVs and can be reliably used in pull-down assays. We also reliably detect putative EV markers (i.e. CD63, CD81 and CD9) which can be used to normalize input and pull down efficiency.

**Summary/Conclusion**: We show that the mutational profile of EGFRvIII brain tumours can be measured in blood without a biopsy. This method may be useful as a non-invasive tool to verify and subtype brain tumours in cases where its location makes biopsies risky or impossible, for drug clinical trial enrollment, to facilitate early surgical planning, and to change practice paradigms for GBM.

**Funding**: This work was supported by the NIH grants UH3 TR000931 (BSC, LB) and P01 CA069246 (BSC).

LBF06.03

Neural-derived peripheral biomarkers for antidepressant response from plasma exosomes

Corina Nagy; Saumeh Saeedi-Tabar; Jean-Francois Theroux; Gustavo Turecki

DMHUI, McGill University, Montreal, Canada

**Background**: Major depressive disorder (MDD) affects millions of people worldwide; however, response to treatment is highly variable, with only one-third of patients responding to the first antidepressant they are prescribed. Consequently, there has been a surge in research to discover biomarkers of MDD treatment response. To date, most research in the field has been conducted in peripheral tissues, which, although useful for biomarker discovery, limits the relevance of these findings to the biology of psychiatric disease. Given that exosomes can freely cross the blood–brain barrier, neural-derived exosomes (NDE) found in plasma can act as biomarkers, as well as provide information regarding central changes resulting from antidepressant drug response. MicroRNAs (miRNA) are an important class of exosomal cargo, which likely influence the functioning of recipient cells. As such, differential NDE miRNA profiles can act as predictive biomarkers, as well as provide mechanistic insight into changes which occur during antidepressant response.

**Methods**: For our pilot study, exosomes were isolated from 2 ml of plasma from 10 controls and 10 MDD patients (5 responders, 5 non-responders) using a size-exclusion column from Izon Science (Christchurch, NZ). Each sample was divided to produce a “whole exosomes” fraction and a “neural-derived (NDE)” fraction, immunoprecipitated using the neural marker L1CAM. Fractions were quantified and sized using tunable resistive pulse sensing on the gNano gold, and RNA was extracted from L1CAM+ fraction and its depleted supernatant for library preparation using the 4N-small RNA-Seq (Galas) protocol. A known plant miRNA was spiked-in to all samples for normalization and sequenced on the Illumina HiSeq platform.

**Results**: We found that NDE are smaller than the full pool of plasma exosomes. Exosomes from patients, regardless of antidepressant response, are significantly smaller than controls in both the full and NDE fractions. We have also identified a group of miRNAs that are highly enriched in the NDE fraction, and that overlap with miRNAs found in brain. Differential analyses show a number of potential targets for follow-up investigation.

**Summary/Conclusion**: Isolating NDE from plasma provides a very valuable resource for biomarker discovery in MDD. We aim to use exosomes to provide neural miRNA profiles of MDD drug response.

**Funding**: This work was funded by CIHR.

LBF06.04

Delivery of ribosomes from glia to neurons

Andrea Schnatz ^1^; Kerstin Müller^2^; Christina Müller^1^; Christina F Vogelaar^3^; Eva-Maria Krämer-Albers^1^

^1^IDN, Molecular Cell Biology, Johannes Gutenberg University Mainz, Mainz, Germany; ^2^IMAN, University Medical Center, Johannes Gutenberg University Maniz, Mainz, Germany; ^3^Department of Neurology, Section Neuroimmunology, University Medical Center, Mainz, Germany

**Background**: The capacity to regenerate following axonal injury greatly varies amongst the different neuronal subtypes. While central neurons are generally assumed to be incapable of spontaneous regeneration, neurons of the peripheral nervous system encounter a growth-permissive milieu. Simultaneously, several studies have demonstrated *de novo* protein synthesis in injured peripheral axons locally providing the components necessary for an immediate regenerative response. Whereas the required mRNAs were shown to originate from the neuron’s soma, the source of axonal ribosomes remained obscure. We generated the so-called “RiboTracker” mouse line expressing ribosomal protein L4 tagged with tdTomato (L4-tdTomato) in distinct cells when crossed to specific Cre mice.

**Methods**: Quantitative immunohistochemistry and immuno electron microscopy of *in vivo* transected sciatic nerves of neuronal and glial RiboTracker-Cre lines; immunocytochemistry of co-cultured glial RiboTracker-Cre cells with wild-type peripheral nervous system (PNS) or central nervous system(CNS) tissues; Western blotting of L4-tdTomato+ Schwann cell-derived microvesicles and exosomes isolated via centrifugation.

**Results**: We found that ribosomes are predominantly transferred from Schwann cells to peripheral axons following injury *in vivo*. In co-culture approaches using RiboTracker glial cells and wild-type PNS or CNS tissues, we were also able to demonstrate a glia-to-axon transfer from L4-tdTomato+ ribosomes. Moreover, our observations strongly suggest vesicle-mediated transfer mechanisms of glial ribosomes to axons upon injury.

**Summary/Conclusion**: Ribosomes are transferred from glia to axons in a vesicle-mediated process potentially providing new targets and therapeutic strategies to improve central axonal regeneration.

**Funding**: This work was financially supported by Deutsche Forschungsgemeinschaft (DRG) (Grant/Award Number: CRC TRR128); Focus Program Translational Neuroscience (FTN), Mainz; and Intramural funding program from the JGU, Mainz.

LBF06.05

Modulation of microglia responses via mesenchymal stromal cells derived-extracellular vesicles

Dorota Kaniowska^1^; Kerstin Wenk^2^; Frank Emmrich^1^; Yarua Jaimes ^1^

^1^Fraunhofer Institute for Cellular Therapy and Immunology, Leipzig, Germany; ^2^Institute for Clinical Immunology, University of Leipzig, Leipzig, Germany

**Background**: Microglia cells are the central nervous system immune cells and have been pointed out as the main mediators of the inflammation leading to neurodegenerative disorders. Mesenchymal stromal cells (MSCs) are a heterogeneous population of cells with very high self-renewal properties and uncomplicated *in vitro* culture. Research has shown that MSCs have the capacity to induce tissue regeneration and reduce inflammation. Studies demonstrated that MSCs have complex paracrine machineries involving shedding of cell-extracellular vesicles (EVs), which entail part of the regulatory and regenerative activity of MSCs, as observed in animal models. We proposed MSC-derived EVs as regulators of microglia activation.

**Methods**: We have used an *in vitro* model for stimulation of the BV-2 microglia cell line and primary cells with lipopolysaccharides (LPS) during 6 and 24 h. Real-time PCR methods were used to assessed the transcripts upregulation of tumour necrosis factor (TNF)‐α, interleukin (IL)‐1β, IL‐6, nitric oxide synthases (iNOS), prostaglandinendoperoxide synthase 2 (PTGS2) and chemokine ligand (CCL)‐22 . Protein levels of TNF‐α, IL‐1β and IL‐6 were evaluated by ELISA and cytometric bead arrays. Expression of the microglia activation cell surface markers were measured by flow cytometry. Western Blot methods were used to detect protein phosphorylation.

**Results**: We demonstrated that the presence of MSC‐EVs prevents TNF‐α, IL‐1β and IL‐6 upregulation by microglia cells towards LPS. Also, inducible isoform of nitric oxide synthases (iNOS) and prostaglandinendoperoxide synthase 2 (PTGS2) upregulation were hampered in the presence of MSC‐EVs. Higher levels of the M2 microglia marker chemokine ligand (CCL)‐22 were detectable in microglia cells after co‐culture with MSC-EVs in the presence and absence of LPS. Moreover, upregulation of the activation markers CD45 and CD11b by microglia cells was prevented when co-cultured with MSC‐MVs. Furthermore, MSC‐EVs suppressed the phosphorylation of the extracellular signal kinases 1/2 (ERK1/2), c‐Jun N-terminal kinases (JNK) and the p38 MAP kinase (p38) molecules.

**Summary/Conclusion**: MSC‐EVs are strong modulators of microglia activation. Further investigation of these vesicles could open new avenues for future cell-free therapies to treat neuroinflammatory diseases.

LBF06.06

Analysis of tau in neuron-derived extracellular vesicles

Francesc Xavier Guix Rafols ^1^; Grant T. Corbett^2^; Diana J. Cha^2^; Maja Mustapic^3^; Wen Liu^2^; David Mengel^2^; Zhicheng Chen^2^; Elena Aikawa^4^; Tracy Young-Pearse^2^; Dimitrios Kapogiannis^5^; Dennis J. Selkoe^2^; Dominic M. Walsh^2^

^1^Laboratory for Neurodegenerative Disease Research, Ann Romney Center for Neurologic Diseases, Brigham & Women’s Hospital and Harvard Medical School, Boston, MA, USA, San Sebastian de Los Reyes, Spain; ^2^Laboratory for Neurodegenerative Disease Research, Ann Romney Center for Neurologic Diseases, Brigham & Women’s Hospital and Harvard Medical School, Boston, MA, USA; ^3^Laboratory of Neurosciences, National Institute on Aging, NIH, Baltimore, MD, USA; ^4^Center for Interdisciplinary Cardiovascular Sciences, Division of Cardiovascular Medicine, Brigham & Women’s Hospital and Harvard Medical School, Boston, MA, USA; ^5^National Institute on Aging/National Institutes of Health (NIA/NIH), Baltimore, USA

**Background**: Progressive cerebral accumulation of tau aggregates is a defining feature of Alzheimer’s disease (AD). The “pathogenic spread model” proposes that aggregated tau is passed from neuron to neuron. Such a templated seeding process requires that the transferred tau contains the microtubule binding repeat (MTBR) domains that are necessary for aggregation. While it is not clear how a protein such as tau can move from cell to cell, previous reports have suggested that this may involve extracellular vesicles (EVs). Thus, measurement of tau in EVs may both provide insights on the molecular pathology of AD and facilitate biomarker development.

**Methods**: We used differential centrifugation to isolate and characterize exosomes from cultured primary and iPSC-derived neurons (iNs), as well as from human cerebrospinal fluid (CSF) and plasma. Since MTBR domain of tau is known to drive aggregation, we set out to determine whether MTBR-containing forms of tau are present in neural EVs.

**Results**: In medium from two different iN lines, we detected MTBR-containing tau in exosomes at very low levels. Analysis of the exosome pellet from CSF revealed low levels of tau, equivalent to ~0.1 pg/ml of CSF. As was evident with EVs from cultured neurons and CSF, neurally derived exosomes from human plasma also contained aggregation-competent tau.

**Summary/Conclusion**: Exosomes contain aggregating-competent tau, but further studies will be required to examine the potential for tau-containing exosomes to seed aggregation in recipient cells.

**Funding**: This work was supported in part by the Intramural Research Program of the National Institute on Aging, NIH (Dimitrios Kapogiannis, Maja Mustapic), and by grants to Dominic M. Walsh from the Alzheimer’s Drug Discovery Foundation and the Harvard NeuroDiscovery Center through a major gift from Rick and Nancy Moskovitz.

LBF06.08

Development of MSC-NTF cell exosomes for the treatment of neurodegenerative diseases

Haggai Kaspi; Jonathan Semo; Natalie Abramov; Revital Aricha

Brainstorm Cell Theraputics, Petach Tikva, Israel

**Background**: MSC-NTF cells are autologous bone marrow derived mesenchymal stem cells (MSC) induced under culture conditions to produce high levels of neurotrophic factors and miRNAs that support neuronal growth and survival, leveraging the potential therapeutic benefits of MSCs that includes anti-inflammatory and immunomodulatory properties. MSC-NTF derived exosomes may possess a unique features for the enhanced cell to cell delivery of therapeutics to the brain, including proteins, microRNA, RNA and other signalling molecules, due to their ability to cross the blood–brain barrier and distribute widely within the brain and spinal cord.

**Methods**: We developed a high scale process to isolate high-purity product of exosomes secreted by MSC-NTF cells using tangential flow filtration. Quantification, size and integrity were verified by zetaview and transmission electron microscopy. Immunomodulatory and neuro-regenerative properties were studied *in vitro* on peripheral blood mononuclear cells and neural precursor cells.

**Results**: MSC-NTF derived exosomes inhibited T cells proliferation, decreased secretion of inflammatory cytokines and increased the proportion of Treg cells in a dose-dependent manner. Culture of neural precursor cells with exosomes accelerated neuronal differentiation and neurite outgrowth in a dose-dependent manner.

**Summary/Conclusion**: MSC-NTF cells-derived exosomes may leverage the benefits of the cells of origin by providing an option to deliver bio-active molecules to the brain in a non-invasive administration, thus creating a novel and promising therapeutic strategy for neurodegenerative diseases.

Scientific Program ISEV2018Saturday, 05 May 2018Symposium Session 19 – Cross-organism Communication in Bacterial Infections Chairs: Igor Almeida; Cherie Blenkiron Location: Auditorium 08:30–10:00

OS19.01

Transcytosis of extracellular vesicles produced by *Bacillus subtilis* 168 in human intestinal Caco-2 cell monolayers

Ana Paula Domínguez Rubio; Jimena Hebe. Martínez; Marcos Palavecino; Mariana Piuri; Oscar Edgardo Pérez

Departamento de Química Biológica, Universidad de Buenos Aires, Ciudad Autónoma de Buenos Aires, Argentina

**Background**: *Bacillus subtilis* 168 is a regular resident of the mammalian gastrointestinal (GI) microbiome and has been used in food fermentations, being awarded the status of “Generally Recognized As Safe” (GRAS). Extracellular vesicles (EVs) have been proposed to be involved in signalling between probiotic bacteria and their mammalian hosts. *B. subtilis* 168 produces EVs which were on the nanometric size range (50–300 nm). EVs carried cytoplasmic components, such as specific proteins, which suggest a role for the EVs in the bacteria–GI cells interface. We hypothesize that transcytosis of EVs across intestinal epithelial cells is a crucial step in the host-probiotic communication. To test this, the ability of EVs produced by the probiotic strain *B. subtilis* 168 to cross intestinal epithelial cell barrier was investigated in an *in vitro* model of human Caco-2 cells.

**Methods**: *B. subtilis* 168 was grown in BHI medium at 37°C under agitation for 18 h. Cells were removed from the culture by centrifugation. Supernatant was then concentrated using a 100-kDa filter membrane. The concentrated supernatant was spun at 110000 *g* for 2 h to pellet EVs. Isolated EVs were stained with carboxyfluorescein succinimidyl ester. Human colon carcinoma Caco-2 cells were differentiated for 14 days (100% conﬂuence). EVs’ uptake was analysed as the number of EVs labelled inside the cell by confocal laser scanning microscopy. Transcytosis was studied as the fluorescence measured in the collected medium from the transwell lower chamber and EVs were also observed. The cytotoxicity of the EVs was evaluated using MTT assay.

**Results**: Intact EVs uptake in Caco-2 cells was linear for up to 30 min: *y* = 1.02 × −1.25 and *R* ^2^ = 0.97 (*p* < 0.05). In transcytosis studies, fluorescence was recorded after 120 min elapsed and increased 50% at 240 min (*n* = 3). We also found intact EVs in the collected medium from the lower chamber of the transwell. EVs did not significantly reduce cell viability (*p* > 0.05).

**Summary/Conclusion**: EVs produced by the probiotic strain *B. subtilis* 168 crossed intestinal epithelial cell barrier of human Caco-2 cells. This evidence suggests that EVs could play a key role in signalling between GI bacteria and mammalian hosts. The expression and further encapsulation of proteins into EVs of GRAS bacteria could represent a scientiﬁc novelty, with applications in food and clinical therapies.

OS19.02

Explosive cell lysis is required for membrane vesicle biogenesis in *Pseudomonas aeruginosa* biofilms

Amelia L. Hynen; James J. Lazenby; Lynne Turnbull; Cynthia B. Whitchurch

The ithree Institute, University of Technology Sydney, Sydney, Australia

**Background**: We have recently determined that explosive cell lysis events account for the biogenesis of membrane vesicles (MVs) in biofilms by the Gram-negative bacterium *Pseudomonas aeruginosa*. Live-cell super-resolution microscopy (OMX 3D-SIM) revealed that explosive cell lysis liberates shattered membrane fragments that rapidly vesicularize into MVs. This vesicularization process also captures cellular content that has been released into the extracellular milieu, thereby packaging it as MV cargo. We have determined that explosive cell lysis is mediated by the endolysin Lys that degrades the peptidoglycan of the bacterial cell wall. As *Lys*-deficient mutants are severely abrogated in the formation of MVs, explosive cell lysis appears to be the major mechanism for MV biogenesis, at least in *P. aeruginosa* biofilms.

The endolysin Lys is encoded within the highly conserved R- and F-pyocin gene cluster. The R- and F-pyocins resemble headless bacteriophage tails and are related to lytic bacteriophage. Endolysins of lytic bacteriophage are transported from the cytoplasm to the periplasm via holins that form pores in the inner membrane. *P. aeruginosa* possesses three putative holins encoded by hol, *alpB* and *cidA*. Hol is likely to be the cognate holin for Lys as it is also encoded in the R- and F-pyocin gene cluster and has been previously shown to mediate Lys translocation. However, both AlpB and CidA have also been previously implicated in lytic processes, but an endolysin associated with these systems has not been described.

**Methods**: Isogenic single, double and triple deletion mutants were generated in hol, alpB and cidA by allelic exchange.

**Results**: We found that all three holin systems contribute to explosive cell lysis in *P. aeruginosa* biofilms. However, each holin appears to have a unique contribution to explosive cell lysis as complementation of a single holin deletion with another of the holins was not always sufficient to restore explosive cell lysis to wild-type levels.

**Summary/Conclusion**: Our findings have revealed that explosive cell lysis is a novel mechanism for the production of MVs and other cell-derived public goods in *P. aeruginosa* biofilms. Furthermore, we have found that three holin systems contribute to explosive cell lysis in *P. aeruginosa*.

OS19.03

Extracellular vesicles secreted by bacteria induce host cell apoptosis

Pankaj Deo; Seong Chow; Thomas Naderer

Monash University, Melbourne, Australia

**Background**: Outer membrane vesicles (OMVs) secreted by Gram-negative bacteria contribute to the pathogenesis of infectious diseases by eliciting immune responses. Cytosolic inflammatory caspases sense OMV-derived lipopolysaccharide to induce inflammatory cell death, termed pyroptosis. OMVs, however, can also cause apoptotic cell death, but the host factors involved remain elusive.

**Methods**: OMVs isolated from *Neisseria gonorrhoeae* were co-incubated with bone marrow-derived macrophages from wild-type or genetically deleted host factor mice.

**Results**: OMVs enabled the trafficking of bacterial outer membrane localized virulence factors to mitochondria. Consequently, OMV treatment resulted in the loss of mitochondrial membrane potential, cytochrome c release, apoptotic caspase activation and cell death in a time-dependent manner, whereby caspase inhibition prevented OMV-induced apoptosis. Unexpectedly, genetic deletion of the BCL-2 family member, MCL-1, completely abrogated the ability of OMVs to induce apoptosis, whereas loss of related BCL-XL increased apoptotic cell death. OMV exposure resulted in the upregulation of the pro-apoptotic MCL-1 isoform, MCL-1S, at the expense of pro-survival MCL-1L. Consequently, expression of a stabilized form of pro-survival MCL-1L prevented OMV-induced apoptosis.

**Summary/Conclusion**: These results demonstrate that OMVs activate intrinsic and extrinsic apoptotic pathways, which may dampen innate immune responses and thereby impact disease outcome.

OS19.04

Nasal microbiota modifies the effects of particulate air pollution on plasma extracellular vesicles

Jacopo Mariani ^1^; Chiara Favero^1^; Laura Pergoli^1^; Laura Cantone^1^; Mirjam Hoxha^1^; Michele Carugno^1^; Matteo Bonzini^1^; Andrea Cattaneo^2^; Angela Cecilia. Pesatori^1^; Valentina Bollati^1^

^1^EPIGET LAB, Department of Clinical Sciences and Community Health, Università degli Studi di Milano, Milan, Italy; ^2^Department of Science and High Technology, University of Insubria, Como, Italy, Como, Italy

**Background**: Extracellular vesicle (EV) production is a powerful and not yet fully understood biological mechanism, probably involved in systemic responses to particulate matter (PM) exposure. As PM enters the human body through inhalation, and locally modifies the composition of the nasal microbiota (NMB), it is possible to hypothesize that NMB modifies the effect of PM exposure on EV release. In a previous study, we identified two clear NMB profiles characterized by a different relative abundance of the *Moraxella* genus (≤25% or >25%) which we defined respectively as Mor− and Mor+. We thus examined 40 healthy volunteers, classified as either Mor− or Mor+, and compared the effects of PM on EV in the two groups, representative of a homogenous and an unbalanced bacterial community.

**Methods**: Individual PM exposure was estimated by a personal sampler (worn for 24 h before blood drawing). Size and cellular origin of plasma EVs were characterized by nanoparticle-tracking and flow-cytometry analysis. NMB was examined through metabarcoding analysis of V3–V4 of the 16S rRNA gene regions.

**Results**: In the Mor− group, PM10 measured the day before enrolment was positively associated with EV release (defined as geometric mean ratio [GMR]): CD14+/monocytes, GMR 5.42 (*p* = 0.048); CD105+/endothelium, GMR 5.38 (p = 0.011). On the contrary, the Mor+ group showed a negative effect of PM10 on EV release: CD14+/monocytes, GMR 0.02 (*p* = 0.008); CD66+/neutrophils: GMR 0.002 (p = 0.006)). The associations were confirmed also for PM2.5 exposure.

**Summary/Conclusion**: Our data show that an unbalanced NMB modifies the effect of PM on EV production. Further studies are needed to explore the underlying molecular mechanisms responsible for such effect and to explore the role of NMB as a possible factor of susceptibility to inhaled pollutants.

**Funding**: This project received support from the EU Programme “Ideas” (ERC-2011-StG 282413 to Prof. Valentina Bollati, principal investigator).

OS19.05

Amplifying host innate immunity through modulation of p38, Jak2 and ALK activities and induction of macrophage differentiation and IL-6 dependent bacterial clearance by exosomes released from *Yersinia pestis*-infected monocytes

Adam Fleming^1^; Heather Hobbs^1^; Valentin Giroux^2^; Weidong Zhou^1^; Valerie Calvert^1^; Carolina Salvador-Morales^2^; Nitin Agrawal^2^; Emanuel Petricoin^1^; Ramin M. Hakami ^1^

^1^George Mason University, Manassas, VA, USA; ^2^George Mason University, Fairfax, VA, USA

**Background**: Our laboratory studies exosome (EX) effects during infection with highly pathogenic agents such as *Yersinia pestis* (Yp), the causative agent of plague. Plague is a re-emerging disease, and Yp is classified as a pathogen of highest concern (Category A) that serves as an excellent model for studies of Gram-negative pathogens. There are no approved vaccines or therapeutics. We have identified the molecular mechanisms by which exosomes released from Yp-infected monocytes (EXi) modulate innate immune response to assist the host in clearing the infection.

**Methods**: EX were purified from naïve U937 monocytes (EXu) and Yp-infected U937 (EXi) by serial centrifugation followed by sucrose density gradient purification, and characterized by transmission electron microscopy and CD63 and TSG101 markers. Immune responses of naïve U937 cells and response mechanisms were analysed following treatment with equivalent amounts of EXi or EXu (as control). Immune response studies included macrophage differentiation assays, multiplex measurements of inflammatory cytokines, and bacterial uptake and clearance assays. Mechanistic studies included quantitative protein microarray analysis of 173 host signalling proteins, siRNA knockdown of EXi-induced cytokines in recipient cells and mass spectrometry analysis of exosome contents. For all assays, at least four biological replicates were performed.

**Results**: EXi induce monocyte differentiation to macrophages and dramatic release of IL-6, IL-8 and IL-10 from among 10 inflammatory cytokines analysed. All these effects are also seen when monocytes are infected with Yp. The EXi also induce a substantial increase in the capacity of the recipient monocytes to clear bacteria in an IL-6-dependent manner. Specific host signalling molecules are strongly modulated by the EXi, including p38, Jak2 and ALK, all of which influence some or all of the observed phenotypes. Mass spectrometry analysis showed that Urease, GroEL and elongation factor Tu of Yp are packaged into the EXi, all of which are antigenic in other bacteria.

**Summary/Conclusion**: EXi prime distant naïve monocytes through modulation of distinct pathways such as p38 and Jak2 to mount immune responses similar to when they become infected with Yp. These include differentiation to macrophages and migration to infection site for increased IL-6-dependent bacterial clearance.

**Funding**: U.S. MRMC grant was awarded to RMH (W81WH-15-T-0003).

OS19.06

P2X1 purinergic blockade protects cells from microvesicle release and Shiga toxin-mediated toxicity

Karl Johansson ^1^; Anne-Lie Ståhl^1^; Ludger Johannes^2^; Diana Karpman^1^

^1^Department of Pediatrics, Clinical Sciences Lund, Lund University, Lund, Sweden; ^2^Institut Curie, PSL Research University, U1143 INSERM, UMR3666 CNRS, Paris, France

**Background**: Shiga toxin (Stx) may circulate within blood cell-derived microvesicles, thus reaching its target organ, the kidney. Our group has previously shown that a non-selective purinergic receptor inhibitor, suramin, decreased microvesicle release from red blood cells stimulated with Stx. The aim of this study was to investigate if specific P2X1 blockade could protect cells from the effects of Stx.

**Methods**: A highly selective P2X1 receptor antagonist, NF449, was used in *in vitro* studies to investigate its effects on Stx-mediated calcium influx, retrograde transport, microvesicle release and toxicity in HeLa cells.

**Results**: Stx1 caused rapid calcium influx in HeLa cells loaded with fluo-4 calcium indicator as detected by fluorescence microscopy. NF449 pretreatment abolished Stx-induced calcium influx completely. Once internalized, Stx1B-subunit predominantly localized with a Golgi marker. Pretreatment with NF449 did not prevent Stx1B transport to the endoplasmic reticulum. Stx1 and Stx2 induced microvesicle release from HeLa cells as measured by flow cytometry, an effect abolished by preincubation with NF449. Cell death, induced by exposure to Stx for 24 h, was reduced in the presence of NF449, exhibiting 56% and 64% increased viability after exposure to Stx1 and Stx2, respectively, in comparison to untreated cells.

**Summary/Conclusion**: Taken together, P2X1 receptor blockade inhibited Stx-mediated calcium influx, microvesicle release and cytotoxicity and thus protected HeLa cells. These data imply that P2X1 blockade should be explored as a treatment for Stx-mediated disease as well as microvesicle-associated diseases in general.

Symposium Session 20 – EVs as Shuttles of Genetic Material Chairs: Kwang Pyo Kim; Kenneth Witwer Location: Room 5 08:30–10:00

OS20.01

Systematic evaluation of techniques for the isolation and detection of small non-coding RNA from urine-derived extracellular vesicles

Elena S. Martens-Uzunova; Natasja Dits; Mirella Vredenbregt - van den Berg; Guido W. Jenster

Erasmus Medical Center, Rotterdam, The Netherlands

**Background**: The ability to stratify prostate cancer (PCa) patients in a non-invasive manner, into these who benefit from radical treatment versus these who can be enrolled in an active surveillance or watchful waiting program, would answer a currently unmet clinical need. A promising solution to this clinical problem is the use of the minimally invasive “liquid biopsy” approach that aims at the detection of tumour biomarkers in blood or urine.

Over the last years, extracellular vesicles (EVs) emerged as a novel promising source of cancer-related biomarkers. Tumour cell originating EVs can be used as a source of protein and RNA biomarkers.

**Methods**: We evaluated available methods for the extraction and quantitation of small RNAs present in urinary EVs in order to examine their use as minimally invasive PCa biomarkers. We tested 11 different combinations of direct and stepwise methods for EV isolation and RNA extraction and quantitated the content of previously established by us small RNAs with high biomarker potential in PCa by two different qPCR techniques.

**Results**: To obtain high amounts of uniform quality starting material, urine samples from healthy donors were depleted from native EVs by ultracentrifugation protocol and spiked in with known amount of EVs isolated from PCa cells. The amount of spiked EVs was equivalent to the amount of removed vesicles. Subsequently, EVs were captured by four different techniques, i.e. ultrafiltration, precipitation, size-exclusion chromatography and affinity capture. Total RNA was isolated either directly from the captured EVs or after EV recovery using two different kits, with or without phenol–chloroform extraction. The amounts of small RNAs (miRNAs, isoMiRs, tRNA fragments, snoRNA and snoRNA fragments) were measured by quantitative real-time PCR (qPCR) either with a SyBR Green technique and LNA-based primers or with a probe-based Taq-Man technique.

**Summary/Conclusion**: Direct, non-organic RNA extraction proved superior to stepwise, phenol–chloroform based techniques in terms of small RNA quantitation. All tested types of small RNAs were successfully detected by qPCR.

**Funding**: This work was supported by IMMPROVE consortium (Innovative Measurements and Markers for Prostate Cancer Diagnosis and Prognosis using Extracellular Vesicles) sponsored by Dutch Cancer Society, Alpe d’HuZes grant: EMCR2015-8022.

OS20.02

Extracellular vesicles mediate the horizontal transfer of an active LINE-1 retrotransposon

Yumi Kawamura ^1^; Yusuke Yamamoto^2^; Taka-Aki Sato^3^; Takahiro Ochiya^2^

^1^National Cancer Center Research Institute, Tokyo, Japan; ^2^Division of Molecular and Cellular Medicine, National Cancer Center Research Institute, Chuo-ku, Japan; ^3^School of Integrative and Global Majors, University of Tsukuba, Tsukuba, Japan

**Background**: Long interspersed element-1 (LINE-1 or L1) retrotransposons replicate through a copy-and-paste mechanism using an RNA intermediate. Previous reports have shown that extracellular vesicles (EVs) from cancer cells contain retrotransposon RNA, including HERV, L1 and Alu sequences. However, the effects of EVs carrying retrotransposon RNA and their ability to retrotranspose in EV-recipient cells have not been reported. In this study, we used a cancer cell model to determine the functional transfer and activity of an active human L1 retrotransposon in EV-recipient cells.

**Methods**: To detect *de novo* L1 retrotransposition events, human cancer cell lines MDA-MB-231-D3H2LN (MM231) and HCT116 cells were transfected with a retrotransposition-competent human L1 tagged with a reporter gene. EVs were prepared from the culture medium of transfected cells by a series of filtration and ultracentrifugation steps. EVs were characterized by nanoparticle tracking analysis, transmission electron microscopy, Western blots, and EV RNA was analysed to detect the presence of L1-derived RNA transcripts. The EV-mediated delivery of L1 RNA was investigated using a co-culture system. L1 retrotransposition events in EV-recipient cells were detected by reporter gene expression and performing PCR from genomic DNA with primers flanking the reporter gene.

**Results**: The enrichment of L1 RNA and RNA-transcripts derived from the reporter construct was confirmed in EVs isolated from the culture supernatant of MM231 and HCT116 cells. Continuous exposure to L1 EVs in the co-culture system revealed that reporter genes were introduced into the genome of EV-recipient cells by L1 retrotransposition. In addition, host-encoded factors were activated in response to increased L1 RNA exposure in EV-recipient cells.

**Summary/Conclusion**: Using an *in vitro* L1 retrotransposition model, we show that EVs can mediate L1 retrotransposition across cells without direct cell-to-cell contact. Our results suggest that an active L1 element can be transmitted horizontally to neighbouring cells through secreted EVs. The results presented here suggest a novel mechanism of L1 mobilization mediated by EVs and may have important implications in the pathogenesis of genetically influenced diseases.

OS20.03

Diverse long-RNAs are differentially sorted into exosomes secreted by mutant KRAS colorectal cancer cells

Jessica J. Abner; Scott A. Hinger; Jeffrey L. Franklin; Qi Liu; Jie Ping; Alissa Weaver; Robert J. Coffey; James G. Patton

Vanderbilt University, Nashville, TN, USA

**Background**: Although much is known about extracellular miRNA, the identity and functional roles of secreted coding and long non-coding RNAs (> 200nt) are largely unknown. We have previously shown that mutant *KRAS* colorectal cancer (CRC) cells release exosomes containing distinct proteomes, miRNAs and circular RNAs. Here, we comprehensively identify the broad and diverse classes of CRC extracellular long RNAs secreted in exosomes and we demonstrate that export of specific RNAs is regulated by *KRAS* status.

**Methods**: We performed RNA sequencing to compare exosomes to their cognate cells. We then used Transwell® culture assays to monitor functional transfer of miRNAs from donor to recipient cells. To assay functional transfer of lncRNAs, we implemented a novel CRISPR/Cas9-based RNA-tracking system to monitor delivery to recipient cells.

**Results**: We show that distinct coding and noncoding RNAs are enriched in exosomes compared to cellular profiles. We detected strong enrichment of Rab13 in mutant KRAS exosomes and demonstrate functional delivery of Rab13 mRNA to recipient cells in Transwell culture assays. We show that gRNAs containing export signals from secreted RNAs can be transferred from donor to recipient cells.

**Summary/Conclusion**: Our data support the existence of cellular mechanisms to selectively export diverse classes of RNA. Current work seeks to discover regulatory proteins that mediate sorting of RNA into exosomes.

OS20.04

Extracellular vesicles limit invasive potential of fibroblasts in an autocrine mechanism mediated by EVs microRNA

Paulina Podszywalow-Bartnicka; Agnieszka Wesolowska; Anna Cmoch; Lukasz Bugajski; Katarzyna Piwocka

Nencki Institute of Experimental Biology, Polish Academy of Sciences, Warsaw, Poland

**Background**: Fibroblasts represent one of the major components of the bone marrow stroma, which supports haematopoietic cells. Extracellular vesicles (EVs) play a role in the communication between both mono- and heterotypic cells. We have showed that extracellular signals delivered from leukemia cells increased invasiveness of human HS-5 bone marrow fibroblasts. Here we investigated the impact of autocrine regulation of fibroblasts by secreted vesicles and EVs miRNA on their invasive potential, because this could possibly counteract the effect of leukemia secreted factors stimulating invasion.

**Methods**: Experiments were performed on HS-5 cells incubated with or without EVs obtained from HS-5 cells conditioned medium by ultracentrifugation. Adhesion, cells morphology and cytoskeleton dynamics were studied using fluorescent microscopy or fluorescence-activated cell sorting. Invasive potential was determined by matrigel invasion, gelatin degradation and formation of invasive protrusions. The profile of miRNA in EVs fraction was assessed by microarrays and real-time PCR, then the activity was verified by luciferase assay. Protein level of miRNA targets was checked by Western blotting.

**Results**: We observed that the addition of fibroblasts-derived EVs increased cells adhesion, stimulated formation of filopodia and β-actin filaments. Based on the miRNA profile, we found that some of the miRNAs in the EVs displayed high activity in the cells and some had very little. Addition of EVs increased their cellular activity. The EVs miRNA inhibited invasive potential and enhanced adhesion of the cells due to targeting of proteins involved in regulation of actin dynamics and formation of invasive protrusions.

**Summary/Conclusion**: Autocrine role of EVs and miRNA secreted by fibroblasts might serve as a self-regulating loop which limits the invasive potential of stromal fibroblasts.

**Funding**: This work was supported by grant 2013/10/E/NZ3/00673 from National Science Center.

OS20.05

The impact of 3D cellular architecture in the microRNA and protein content of extracellular vesicles

Sara Rocha ^1^; Joana Carvalho^1^; Patrícia Oliveira^1^; Maren Voglstaetter^2^; Domitille Schvartz^3^; Andreas Thomsen^4^; Nadia Walter^3^; Richa Khanduri^2^; Jean-Charles Sanchez^3^; Andreas Keller^5^; Carla Oliveira^1^; Irina Nazarenko^2^

^1^i3S - Instituto de Investigação e Inovação em Saúde, Universidade do Porto, Porto, Portugal, Porto, Portugal; ^2^Institute for Infection Prevention and Hospital Epidemiology; Medical Center - University of Freiburg, Faculty of Medicine, University of Freiburg, Freiburg, Germany, Freiburg, Germany; ^3^Department of Human Protein Sciences, Centre Médical Universitaire, Geneva, Switzerland, Geneva, Switzerland; ^4^Department of Radiation Oncology, Medical Center - University of Freiburg, Freiburg, Germany, Freiburg, Germany; ^5^Clinical Bioinformatics, Saarland University, University Hospital, Saabruecken, Germany

**Background**: The success of malignant tumours is conditioned by the intercellular communication between tumour cells and their microenvironment. *In vivo* models have been used to study the role of extracellular vesicles (EVs) as shuttles of information between cells; however, in most cases, EVs are collected from 2D *in vitro* cultures that poorly resemble the *in vivo* context. Knowing that 3D *in vitro* models recapitulate better the *in vivo* features of tumours, we hypothesized that EVs secreted by 3D cultures mimic better the signals used for intercellular communication than EVs secreted in 2D conditions.

**Methods**: We performed a comparative analysis of biochemical features, small RNA and proteomic profiles of EVs secreted by 2D and 3D cultures of gastric cancer (GC) cells. We established a 3D *in vitro* model for culture and isolation of EVs from GC spheroids. Cellular organization, polarization and viability were assessed by H&E, Ki-67, E-cadherin, Mucin-1 and AnV/PI staining. EVs, isolated from conditioned media of 2D and 3D cultures by differential ultracentrifugation, were characterized by transmission electron microscopy, nanoparticle tracking analysis and imaging flow cytometry. EVs’ small RNA and proteomic profiles were analysed by next-generation sequencing and liquid chromatography-tandem mass spectrometry, and validated by qRT-PCR and Western blot, respectively. Omics data were integrated using bioinformatics tools.

**Results**: Our 3D cultures recapitulated the histological properties of tumours and their *in vivo* polarization, and were more cost-effective in producing EVs than 2D cultures. EVs secreted by 2D and 3D cultures had similar small RNA profiles, enriched in microRNAs, and 8–13% of the microRNA content of EVs was dependent on the cell culture condition. The proteomic profile was different for EVs collected from 2D and 3D cultures, and proteins identified as differentially regulated were downregulated in 3D. Integrative network analysis of microRNA and protein data revealed that proteins associated with ARF6 signalling, known to be involved in endocytosis and receptor recycling, were significantly decreased in EVs released by 3D cultured cells.

**Summary/Conclusion**: Our study suggests that 3D cell organization influences the EV cargo towards enrichment in microRNAs and decrease in certain proteins. Further studies are now requested to understand the impact of 3D cellular architecture on EVs’ function.

S Rocha & J Carvalho: co-first authors

C Oliveira & I Nazarenko: co-senior authors

OS20.06

Integrative pathway analysis on protein and miRNA allows investigation of the role of extracellular vesicles in mediating the adaptive response of prostate cancer cells to androgen deprivation

Carolina Soekmadji ^1^; Jiyuan An^1^; Anja Rockstroh^2^; James E Riches^2^; Grant A Ramm^1^; Colleen C Nelson^2^; Pamela J Russell^2^

^1^QIMR Berghofer, Herston, Australia; ^2^Queensland University of Technology, Brisbane, Australia

**Background**: Androgens, including testosterone, are known to modulate prostate cancer proliferation by activating the androgen receptor, the primary driver in prostate cancer cell proliferation and survival. Androgen deprivation therapy (ADT) is aimed at blocking the production and function of male hormone androgens responsible for cancer development and growth. We recently described the role of androgen in influencing the secretion of extracellular vesicles (EVs) in an advanced prostate cancer model (1–3). We sought to investigate the complexity of EV proteins and miRNAs to gain deeper insight into the role of EVs in mediating prostate cancer drug resistance. We utilized a high-throughput paired protein and miRNA analysis from isolated EV in comparison with parental cells.

**Methods**: To identify which EV candidates were affected by androgens, prostate cancer cells were cultured in the presence of foetal bovine serum or androgen-depleted charcoal stripped serum. Analysis was performed to investigate the effect of 10 nM physiological androgen dihydrotestosterone or 10 µM anti-androgen enzalutamide. EVs were isolated by differential ultracentrifugation and were characterized by Western blot, time-resolved fluoroimmunoassay, tunable resistive pulse sensing and transmission electron microscopy. The protein and miRNA cargos of EVs were assessed by mass spectrometry and next-generation sequencing, respectively. Pathways were evaluated by Ingenuity Pathway Analysis. Changes in expression of genes were confirmed by Western and/or quantitative reverse transcription PCR.

**Results**: Using this approach, we have discovered novel candidate EV-derived proteins and miRNAs which could mediate prostate cancer proliferation and survival. Changes in expressions of several EV candidate molecules promote the survival of prostate cancer cells under androgen deprivation. In ongoing experiments, we sought to validate the EV subpopulations involved in this adaptive process, leading to resistance towards androgen deprivation.

**Summary/Conclusion**: Our data bring us a step closer to understand the role of EVs in the progression of advanced prostate cancer, where cancer has become unresponsive towards ADT.

**References**: (1) Soekmadji C, et al. (2017) *Proteomics* 1600427-n/a. (2) Soekmadji C, et al. (2017) *Prostate* 77, 1416–1423. (3) Soekmadji C, et al. (2016) *Oncotarget* 8, 52237–52255.

**Funding**: This work was supported by Movember Foundation GAP1 Exosomes, US DoD No. W81XWH-12-1-0047 and W81XWH-16-1-0736.

Symposium Session 21 – Role of EVs in Regulating Tumour Cell Behaviour Chairs: Susanne Gabrielsson; Jason Webber Location: Room 6 08:30–10:00

OS21.01

lncRNA HOTAIR affects EMT and extracellular vesicle content and function

Irena Nowak^1^; Claudia Berrondo^2^; Jonathan Flax^1^; Thomas Osinski^1^; Carla J. Beckham ^1^

^1^University of Rochester, Rochester, NY, USA; ^2^Seattle Children’s Hospital UW, Seattle, WA, USA

**Background**: Previously, we showed the long non-coding RNA Hox antisense intergenic transcript (HOTAIR) enriched in urothelial bladder cancer (UBC) cell lines, extracellular vesicles (EVs), patient tumours and urinary EVs. HOTAIR affects genes involved in epithelial-to-mesenchymal transition (EMT) pathways like canonical Wnt and EGF signalling. Loss of HOTAIR correlates with reduced migration and invasion. Wnt and EGF pathways transactivate one another via common signalling molecules and this may contribute to monotherapy failure, tumour heterogeniety and progression. Given the importance of HOTAIR in regulation of these pathways, we explored the possibility that HOTAIR may affect EMT by regulating transactivaion and EV content and function.

**Methods**: UBC and breast cancer (BCa) cell lines were treated with rWnt3a or EGF; EMT was evaluated by qRTPCR (target genes [TG]), immunoblotting, *in vitro* migration and invasion assays. HOTAIR was knocked down (KD) with siRNA or shRNA in UBC and BCa cells treated with rWnt3a or EGF; EMT was evaluated by qRTPCR [TG], immunoblotting, *in vitro* migration and invasion assays. LC/LC-mass spectrometry was performed on EVs from control or HOTAIR KD cells. Immunoblotting, immunohistochemitry and qRTPCR were used to confirm the presence of Rab5, 7,11, EGFR in cells and EVs.

**Results**: rWnt3a and EGF treatment increases TG expression, *in vitro* migration and invasion and transactivation in UBC and BCa cells. Loss of HOTAIR increases Wnt antagonist genes, reduces EGF and Wnt gene expression and loss of *in vitro* migration and invasion with rWnt3a treatment. Loss of HOTAIR increases Rab5, reduces Rabs 7, 11 and alters sub-cellular EGFR in cells. HOTAIR KD cell EVs have reduced EGFR, fewer EVs with altered content and do not facilitate migration or invasion.

**Summary/Conclusion**: We show HOTAIR is necessary for Wnt-responsiveness and may mediate Wnt/EGF transactivation. EMT is also regulated through intercellular communication by EVs. HOTAIR regulates hundreds of genes and our data implicate HOTAIR in the regulation of several genes in the early endocytic and recycling pathways. We find HOTAIR KD cells produce fewer EVs with altered protein cargo and these EVs do not facilitate migration or invasion. HOTAIR appears to affect EMT through Wnt and EGF pathways, EV biogenesis, content and function.

**Funding**: This work was supported by Wilmot Cancer Foundation Fellowship.

OS21.02

RASSF1C oncogene promotes amoeboid phenotype and invasiveness via extracellular vesicle transfer in breast cancer

Nikola Vlahov^1^; Maria Laura Tognoli ^2^; Daniela Pankova^2^; Sander Steenbeek^3^; Michael Eyres^2^; Jacco van Rheenen^3^; Eric O’ Neill^2^

^1^Beatson Institute, Glasgow, UK; ^2^Department of Oncology, University of Oxford, Oxford, UK; ^3^Hubrecht Institute-KNAW & University Medical Center Utrecht, Utrecht, The Netherlands

**Background**: *RASSF1* (RAS association domain family 1) is one of the most frequently epigenetically inactivated tumour suppressor genes in cancer. High promoter methylation of the *RASSF1* gene has been associated with poor prognosis in breast, lung and colon cancers. The *RASSF1* isoform RASSF1A is considered a bona fide tumour suppressor. Interestingly, in tumours with RASSF1 promoter methylation, an alternative oncogenic isoform, RASSF1C, is expressed and it directly affects cell–cell junctions and epithelial cell polarity leading to epithelial-to-mesenchymal transition.

Our aim was to characterise RASSF1C oncogenic functions in breast cancer cells. Furthermore, we sought to understand how RASSF1C cells transfer their aggressive phenotype to recipient cells.

**Methods**: Extracellular vesicles (EVs) were isolated from RASSF1C cells using differential centrifugations and processed for cargo (Western blot, mass spectrometry) and morphological analysis (transmission electron microscoscopy, nanoparticle tracking analysis).

A Cre-LoxP model for *in vitro* and *in vivo* EV transfer was employed to assess the ability of RASSF1C cells to alter the fate of recipient cells. MDA-231 donor cells expressing CFP+Cre+ (CTRL or RASSF1C) were cultured (*in vitro*) or co-injected (*in vivo*) with T47D reporter cells harbouring a dsRed-LoxP-eGFP allele. Unrecombined reporter cells thus express dsRed, whereas recombined reporter cells switch to eGFP, after having taken up Cre+ve EVs from the donor cells.

**Results**: We describe a new role for RASSF1C where its expression in breast cancer cells drives an amoeboid phenotype through direct activation of the Rho/ROCK axis. As a result, RASSF1C cells adopt high contractility, invasive properties and a stemlike transcriptional profile.

Moreover, RASSF1C cells release high numbers of EVs that are internalised by recipient cells. These EVs are responsible for transferring the invasive ability to less aggressive cells *in vitro*.

Additionally, *in vitro* and *in vivo* application of the Cre-LoxP system showed that MDA-231 RASSF1C donor cells elicit an aggressive phenotype in T47D reporter cells, increasing their metastatic potential both through local and distant EV communication.

**Summary/Conclusion**: The work proposes a new mechanistic insight of RASSF1C oncogenic potential and could partially explain the molecular mechanisms underlying the poor prognosis observed in breast cancer patients bearing RASSF1 promoter methylation.

**Funding**: This work was supported by CRUK/MRC.

OS21.03

Role of exosomes in controlling directional migration of cancer cells

Alissa Weaver; Bong Hwan Sung

Vanderbilt University, Nashville, TN, USA

**Background**: Directional migration of cancer cells is promoted by exosome secretion, but the underlying mechanisms are not well understood. Directional migration of cells could occur by responding to directional cues such as chemical or matrix gradients, or the topology of the environment. Here we examine the role of exosome secretion in controlling direction migration of fibrosarcoma cells in the presence of either chemical or topological cues.

**Methods**: Exosome secretion was inhibited by knocking down Rab27a. We also performed rescue experiments by coating purified exosomes or larger microvesicles onto various migration environments. Migration was tracked in time-lapse movies and speed and directional persistence was quantified from the tracks of the migrating cells.

**Results**: Migration of human fibrosarcoma cells towards a gradient of exosome-depleted serum was diminished by knocking down expression of Rab27a, an exosome secretion regulator. Rescue experiments, performed by coating extracellular vesicles and purified fibronectin onto the cell migration area of chemotaxis chambers, revealed that exosomes but not microvesicles affect both the speed and directionality of migrating cancer cells. In addition, fibronectin promoted only speed and not directionality of migrating cells. We also tested whether exosome secretion affects migration of cells in diverse topologies, using ECM-coated synthetic nanofibres to mimic *in vivo* environments. In these environments as well, we find that exosome secretion promotes persistent migration of cancer cells. Compared to control cells that migrate relatively persistently on aligned nanofibres, exosome secretion-inhibited cells continuously change their direction of migration in a bidirectional mode of migration. In random fibre meshworks, exosome-inhibited cells exhibit excessive pausing at the junctions of random nanofibres.

**Summary/Conclusion**: Overall, we find that exosomes promote multiple aspects of cell motility including speed, directionality and persistence, which are needed to navigate complex environments.

**Funding**: This work was funded by the National Institutes of Health, grants R01GM117916, R01CA206458 and R01CA163592.

OS21.04

Hypoxia-driven changes in EV composition are autophagy-dependent

Marijke I. Zonneveld ^1^; Tom G.H. Keulers^1^; Hanneke Peeters^1^; Sten F.H.M. Libregts^2^; Marca H.M. Wauben^2^; Kasper M.A. Rouschop^1^

^1^Autophagy Lab, Department of Radiotherapy, GROW - School for Oncology & Developmental Biology, Maastricht University, Maastricht, The Netherlands; ^2^Department of Biochemistry and Cell Biology Faculty of Veterinary Medicine, Utrecht University, Utrecht, The Netherlands

**Background**: Hypoxia is an important component of the tumour microenvironment (TME), correlating with increased angiogenesis, migration and treatment resistance. Tumour cells respond to changes in their TME through the secretion of extracellular vesicles (EV). How hypoxia influences EV communication with the TME remains unclear. Here, we investigated the impact of hypoxia on EV secretion, composition and function. Hypoxia is a potent trigger for autophagy and recently, endosomal proteins involved in exosome biogenesis were shown to interact with members of the autophagy machinery. Therefore, we investigated the role of autophagy in the secretion of EV.

**Methods**: HT-29, U87 and MDA-MD-231 cells were exposed to moderate (0.2% O_2_) or severe (0.02% O_2_) hypoxia. The involvement of the autophagy machinery was assessed by inducible knockdown cell lines for ATG7, ATG12 and LC3B. EV were isolated using a density gradient or size-exclusion chromatography. EV were analysed by high resolution flow cytometry, Western blot and next-generation sequencing. To assess angiogenic potential, endothelial cells were exposed to EV and monitored for tubule formation.

**Results**: Using high resolution flow cytometry, we observed that the total number of secreted EV was not altered during hypoxia. Nevertheless, depending on the severity of hypoxia, we observed that gross protein content was either increased (0.2% O_2_) or decreased (0.02% O_2_) compared to controls. In contrast, the gross RNA content did not seem to be affected. Immunoblot analysis revealed distinct patterns for CD9, CD63 and Flotillin-1, suggesting composition changes dependent on oxygen level. Strikingly, an emphatic decrease in EV markers was observed by Western blot, as well as in total EV numbers in autophagy-deficient cells under hypoxia. Functionally, EV from hypoxic cells increased endothelial tubule formation.

**Summary/Conclusion**: Collectively, these results indicate that hypoxia alters EV composition and that autophagy is required for EV secretion during hypoxia.

**Funding**: This research was supported by Worldwide Cancer Research as part of project WCR 16-0265.

OS21.05

Extracellular vesicles from fibroblasts undergoing oncogene-induced senescence are enriched in lysophosphatidic acid

Krizia Sagini ^1^; Lorena Urbanelli^1^; Sandra Buratta^1^; Stefano Giovagnoli^2^; Silvia Caponi^3^; Daniele Fioretto^4^; Nico Mitro^5^; Donatella Caruso^5^; Carla Emiliani^6^

^1^Department of Chemistry, Biology and Biotechnology, University of Perugia, Perugia, Italy, Perugia, Italy; ^2^Department of Pharmaceutical Sciences, University of Perugia, Perugia, Italy, Perugia, Italy; ^3^Istituto Officina dei Materiali del CNR (CNR-IOM) - Unità di Perugia, c/o Department of Physics and Geology, University of Perugia, Perugia, Italy, Perugia, Italy; ^4^Department of Physics and Geology, University of Perugia, Perugia, Italy, Perugia, Italy; ^5^Department of Pharmacological and Biomolecular Sciences, University of Milan, Milan, Italy, Milan, Italy; ^6^Department of Chemistry, Biology and Biotechnology, University of Perugia, Perugia, Italy

**Background**: The identification of biomarkers to monitor ageing process and prevent chronical disease should be of primary importance in societies where the population has become increasingly old. Senescent cells, which are characterized by permanent proliferation arrest in response to DNA damages, accumulate in aged tissues. Oncogene activation, which finally affects genomic integrity, leads to a peculiar senescence process termed Oncogene-Induced Senescence (OIS). Senescent cells release the so-called senescence-associated secretory phenotype, which includes not only soluble factors but also extracellular vesicles (EVs). EVs contain complex cargo and appeared as an attracting source of biomarkers. Cellular lipids play an important role in EV formation, secretion and uptake and EV transferred lipids impact on recipient cell signalling.

**Methods**: To identify lipid signatures associated with OIS in EVs, we analysed the lipidome of human fibroblasts upon the expression of oncogenic H-Ras (HRasV12) and their released EVs. EVs were isolated from the medium of transfected cells after selection using Exoquick™. Serum-free medium was used to avoid contaminations by foetal bovine serum lipoproteins or bovine EVs. The quality of EV preparation was checked by nanoparticle tracking analysis, electron microscopy and immunoblotting (IB). Total lipids were extracted from cells and EVs and analysed with liquid chromatography-tandem mass spectrometry method.

**Results**: Fibroblasts undergoing OIS upon H-RasV12 expression released a greater amount of EVs carrying higher level of tetraspanin proteins and ESCRT components. When the lipidomic profiles of fibroblasts were compared to that of released EVs, it results that EVs had a higher lipid/protein ratio and a different glycerophospholipid and sphingolipid distributions. Moreover, results revealed a specific H-Ras-induced signatures in EVs, namely the enrichment in sphingomyelin, lysophosphatidic acid (LPA) and sulfatides. Of interest, LPA is also a ubiquitous bioactive molecule able to influence various biological processes by binding to cognate G protein-coupled receptors.

**Summary/Conclusion**: In conclusion, the lipid profile of fibroblasts and their released EVs allowed the identification of specific OIS-associated signature. Specifically, the showed enrichment of LPA in H-RasV12 EVs appeared interesting not only as potential biomarker but also as signalling mediator towards neighbour cells.

OS21.06

Extracellular vesicles transfer of the myddosome as the proinflammatory signal from the Waldenström macroglobulinaemia lymphoma cells carrying MyD88L265P mutation

Mateja Manček Keber ^1^; Duško Lainšček^1^; Mojca Benčina^1^; Jiaji G. Chen^2^; Rok Romih^3^; Zachary R. Hunter^2^; Steven P. Treon^2^; Roman Jerala^1^

^1^National Insitute of Chemistry, Ljubljana, Slovenia; ^2^Dana Farber Cancer Institute, Harvard Medical School, Boston, MA< USA; ^3^Faculty of Medicine, Ljubljana, Slovenia

**Background**: Link between activation of inflammatory signalling pathways and cancer is particularly evident in Waldenström macroglobulinaemia (WM), where more than 90% of patients harbour a mutant of MyD88. MyD88 is a signalling adapter protein that plays a pivotal role in innate immunity. MyD88 is recruited to the Toll/interleukin-1 receptor domains of activated Toll-like receptors, leading to formation of the myddosome and NF-κB activation. MyD88^L265P^ constitutively activates the signalling pathway and provides a survival signal to cancer cells, thus chronic inflammation may contribute to the tumour microenvironment.

**Methods**: EVs containing MyD88L265P were isolated from overexpressed HEK293 cells and WM lymphoma cell lines by ultracentrifugation. bone marrow-derived macrophages and bone marrow-derived cultured mast cells were stimulated and cytokine expression was analysed by qPCR. MyD88L265P and MyD88wt signalling complexes were detected by confocal microscopy. EVs were injected intramedullary into femur or i.v. to assess the pathological relevance by immunohistochemistry and luminescence.

**Results**: Here we identified an alternative mechanism to the transmission of inflammatory mediators by transfer of the myddosome via EVs. Constitutively active MyD88L265P was transferred to other recipient cells mainly through endocytosis, where mutated MyD88 recruited the endogenous MyD88wt to trigger cell activation without receptor activation. *In vivo* internalization of EVs containing MyD88 occurred and the changes to the bone marrow microenvironment were observed. MyD88-containing EVs were detected in the bone marrow aspirates of WM patients.

**Summary/Conclusion**: We report that the transfer of constitutively active signalling mediators via EVs represents a new mechanism of intercellular transfer of inflammatory signals that is amplified in the recipient cells by the recruitment of the endogenous signalling mediators. This process may play an important role in WM cancer development by triggering inflammation in the nontransformed cells independently of the membrane receptors.

Plenary Session 3: EVs in Environment, Diet and Health Chairs: Hernando del Portillo; Kenneth Witwer Location: Auditorium 10:30–11:00

PL 5

Parasitic Worm EVs: From Vaccines to Anti-inflammatories

Alex Loukas

Centre for Biodiscovery and Molecular Development of Therapeutics, Australian Institute of Tropical Health and Medicine, James Cook University, Australia, Cairns, Australia

Parasitic helminths (worms) infect more than 2 billion people and many more livestock and companion animals. Infections with worms presents a double-edged sword for humans. On the one hand they are pathogens that exact an enormous toll on the health of infected hosts in developing countries, causing hundreds of thousands of deaths annually. On the other hand, worms are potent regulators of inflammation and developed sophisticated strategies to modulate and skew the host’s immune response to benefit their own survival. A bystander effect of helminth-driven immunoregulation is protection against the onslaught in developed countries of non-infectious diseases that result from a dysregulated immune system, including inflammatory bowel diseases, asthma and diabetes. Both immunoepidemiological studies and clinical trials using iatrogenic helminth infection have proven that some worms are least can protect against autoimmune diseases. Using animal models of autoimmunity, allergy and metabolic syndrome, worm secreted proteins have been shown to suppress inflammation via distinct mechanisms of action. We and others have recently shown that parasitic worms secrete EVs in addition to soluble proteins, small molecules and nucleic acids, and those EVs are internalised by host cells whereupon they regulate inflammatory processes. Worm EVs have hallmark features of exosomes but also possess specific families of proteins that are unique to these parasites. Moreover, worm EVs have an abundance of miRNAs that are predicted to target host cytokine and immune signalling genes, implying yet another strategy by which helminths regulate host inflammation. We showed that administration of hookworm EVs (but not whipworm or grape EVs) to mice protects against inducible colitis and suppresses inflammatory cytokine production but induces regulatory (IL-10) cytokine production. In an attempt to block worm EV-mediated host immunoregulation as an anti-parasite vaccination strategy, we vaccinated animals with EV recombinant surface tetraspanins (TSPs). Vaccination reduced worm burdens in parasite challenged animals and anti-TSP antibodies block EV uptake by host cells and suppress downstream phenotypic effects. Parasitic worm EVs therefore represent a goldmine of anti-parasite vaccine targets as well as novel anti-inflammatory therapeutics inspired by host-parasite co-evolution.

Featured Abstracts – Session 2 Chair: Ramaroson Andriantsitohaina Location: Auditorium 11:00–11:35

FA2.01

Live tracking of endogenous exosomes *in vivo*

Frederik Verweij ^1^; Celine Revenu^2^; Guillaume Arras^2^; Damarys Loew^2^; Philippe Herbomel^3^; Guillaume Allio^4^; Jacky Goetz^5^; Filippo del Bene^2^; Graca Raposo^2^; Guillaume van Niel^6^

^1^Center for Psychiatry and Neuroscience / Institut Curie, Paris, France; ^2^Institut Curie, Paris, France; ^3^Institut Pasteur, Paris, France; ^4^Institut Immunologie-Hématologie, Strasbourg, France; ^5^INSERM U1109, Strasbourg, France; ^6^CNRS, Paris, France

**Background**: Extracellular vesicles (EVs) such as exosomes are released by a wide variety of cell types and found in all organism tested so far. Even though EVs are implicated in many important physio- and pathological processes, our understanding of their relevance *in vivo* remains poorly understood, mainly due to the lack of model organisms that allow the accurate spatiotemporal assessment of EV biogenesis, transfer and fate at single-vesicle level.

**Methods**: We developed an animal model to study endogenous exosomes *in vivo* by expressing CD63-pHluorin, a fluorescent reporter for exosome secretion, in zebrafish embryos. Using a combination of light- and electron microscopy techniques and proteomic ex vivo analysis, we explored the physiology of exosomes, including their biogenesis, transfer, uptake and fate.

**Results**: We observed exosome release *in vivo* and tracked a massive pool of endogenous EVs in the blood-flow and interstitial fluid of zebrafish embryos. We identified the yolk syncytial layer (YSL), a cell layer with essential nutrient transport functions, as a major contributor to this pool of EVs by cell layer-specific expression. We further identified YSL-derived EVs as exosomes generated in a syntenin-dependent manner and enriched, among others, in solute carriers. These exosomes were directly released into the vascular system, and, by following the blood flow, went through the whole organism to finally reach and accumulate in the caudal vein plexus. Here, exosomes were specifically captured and endocytosed by scavenging early macrophages and endothelial cells. While endocytosis of exosomes was dynamin-dependent in both cell types, inhibition of scavenger receptors interfered mostly with uptake in endothelial cells. Interestingly, we could not observe functional transfer of Cre-protein. In fact, selective inhibition of vATPases showed massive accumulation of YSL-derived exosomes in late endo-/lysosomal compartments destined for degradation. Functionally, the origin, distribution and fate of these EVs are compatible with a role in trophic support for the developing embryo.

**Summary/Conclusion**: Altogether, these data reveal for the first time the release, journey, targets and fate of endogenous exosomes *in vivo* and support a role in nutrient delivery.

**Funding**: This work was supported by EMBO-ALTF-1383-2014, ARC-PJA-20161204808, FRM.

FA2.02

Spatio-temporal analysis of glia to neuron exosome transfer *in vivo* using CreERT2-reporter transgenic mouse models

Christina Müller^1^; Wen-Ping Kuo-Elsner^2^; Anja Scheller^3^; Laura Stopper^3^; Frank Kirchhoff^3^; Eva-Maria Krämer-Albers ^1^

^1^IDN, Molecular Cell Biology, Johannes Gutenberg University Mainz, Mainz, Germany; ^2^IDN, Molecular Cell Biology, University of Mainz, Mainz, Germany; ^3^Molecular Physiology, CIPMM, University of Saarland, Homburg, Germany

**Background**: Extracellular vesicles (EVs) emerged as key players in cell–cell communication in the healthy and diseased nervous system. However, tools to visualize EV-transfer and to study its impact on brain physiology *in vivo* are lacking. Our previous work demonstrated that exosomes derived from oligodendrocytes (OL) are involved in neuron–glia communication mediating glial support and long-term neuronal maintenance, e.g. by promoting axonal transport. Here, we introduce a CreERT2-reporter mouse model to visualize exosome transfer from glia to neurons *in vivo* and to determine its prevalence across different brain regions.

**Methods**: PLP-CreERT2 and NG2-CreERT2 mice-driving CreERT2 expression in mature OL and OL precursors, respectively, were crossed to Rosa26-tdTomato reporter mice (Ai14) and subjected to consecutive Tamoxifen injections promoting reporter gene recombination in exosome target neurons in addition to donor OL. Recombined neurons were quantified in brain sections using an ImageJ plugin and allocated to brain regions. We further studied the influence of neuronal electrical activity on exosome transfer by subjecting CreERT2-reporter mice to monocular deprivation and quantifying reporter gene recombination in the ipsilateral and contralateral cortex.

**Results**: Recombined neurons indicating glia to neuron exosome transfer were detected in several brain areas of PLP-CreERT2-reporter as well as NG2-CreERT2-reporter mice with highest numbers observed in the striatum, amygdala and the cortex. With increasing age we detected a higher number of recombined neurons providing evidence that exosome transfer is ongoing with ageing. Monocular deprivation resulted in a reduced number of recombined neurons selectively in the contralateral versus the ipsilateral cortex (optic chiasm) while other brain regions remained unaffected, indicating that lack of electrical activity along the optic tract diminishes exosome transfer.

**Summary/Conclusion**: Spatio-temporal analysis of double transgenic OL-specific CreERT2-reporter mice demonstrates that OL to neuron exosome transfer occurs throughout the brain with highest prevalence in the striatum and amygdala. CreERT2-reporter mice provide a useful means to determine EV-transfer *in vivo* under different physiological conditions.

**Funding**: This work was funded by DFG.

Symposium Session 22 – Parasitic EVs: From Basics to Translation Chairs: Amy Buck; Neta Regev-Rudzki Location: Auditorium 13:45–15:15

OS22.01

Understanding host: pathogen interactions mediated by exosomes produced by the parasite *Trichomonas vaginalis*

Anand Rai; Olivia Twu; Patricia J. Johnson

UCLA, Los Angeles, CA, USA

**Background**: The parasite *Trichomonas vaginalis *is the causative pathogen of the most prevalent, non-viral sexually transmitted infection worldwide. Depending on the parasite strain and host, infections can vary from asymptomatic to highly inflammatory. We previously reported that *T. vaginalis* generates and secretes microvesicles with physical and biochemical properties similar to mammalian exosomes. *T. vaginalis* exosomes fuse with and deliver cargo to the host cell, assisting in parasite colonization and eliciting immune responses that may combat parasite clearance.

**Methods**: We are currently studying the mechanisms underlying the delivery of *T. vaginalis* exosomal cargo to mammalian host cells.

**Results**: This time-dependent process is likely mediated by carbohydrate:protein interactions. Vesicle fusion varies among *T. vaginalis* strains; exosomes from strains that are highly adherent and cytolytic to host cells exhibit a greater efficiency in delivering cargo to cells.

**Summary/Conclusion**: Our work on the identification of molecules present on the surface of both the parasite exosomes and the host cell that play critical roles in host:pathogen interaction will be discussed.

**Funding**: This work was funded by the National Institutes of Health, USA.

OS22.02

The role of extracellular vesicles in the modulation of endothelial junctions in an *in vitro* model of cerebral malaria

Valery Combes; Benjamin Sealy; Iris Cheng

The University of Technology Sydney, Sydney, Australia

**Background**: Malaria resulted in 438,000 deaths in 2015, with 90% due to cerebral malaria (CM). CM occurs when *Plasmodium falciparum*-infected red blood cells (PRBCs) sequestrate within the cerebral microvasculature causing neurological lesions associated with alteration of the blood–brain barrier (BBB). Using *in vitro* c-culture systems and murine models, recent studies by our group and others have suggested that extracellular vesicles (EVs) participate to the development of the vascular lesion during CM.

**Methods**: Using an *in vitro* BBB model, we aim to investigate the effect that EVs have on the modulation of endothelial integrity by measuring the expression of VE-cadherin and the activation status of the endothelial monolayer. EVs released by both normal RBCs (NRBCs) and PRBCs after 48h culture were purified from the supernatant by sequential centrifugation.

Primary human brain endothelial cells (HBECs) were incubated with NRBCs, packed RBCs (PRBCs), NRBC- and PRBCs-EVs (nEVs, pEVs), or a combination of them. PRBCs were added to HBEC at a ratio of 50:1 while 3 μg of EVs were added per 100,000 cells. VE-cadherin expression was assessed by a combination of high content, high resolution and OMX super-resolution microscopy to measure the overall changes in VE-cadherin expression as well as the local changes due to the presence of PRBCs and/or EVs. Expression of ICAM-1 and VCAM-1 were measured by flow cytometry.

**Results**: Quantitative image analysis showed that NRBC and both nEVs and pEVs triggered modulation of VE-cadherin expression, whereas PRBC and PRBC-Mix conditions resulted in a significant down-regulation. We also observed that p-EVs were taken up by HBEC at twice the rate of nEVs. Expression of eCAMs, was increased in the presence of PRBCs and further increased with PRBC-Mix.

**Summary/Conclusion**: These results suggest that interactions between EVs and their cells of origin do not always trigger the same cellular response in their target cell. Therefore, the combined presence of both EVs and cells may either potentiate or compensate each other effects. Further studies are needed to determine which molecular pathways are involved in the changes observed.

**Funding**: This work was funded by the University of Technology Sydney (internal funds) and the Australian National Health & Medical Research Council Project Grant.

OS22.03

Exploration of extracellular vesicles from Ascaris suum provides evidence of parasite-host cross talk

Eline P Hansen^1^; Bastian Fromm^2^; Sidsel D Andersen^3^; Antonio Marcilla^4^; Kasper L Andersen^1^; Andrew R Williams^1^; Aaron R Jex^5^; Robin B Gasser6; Neil D Young^6^; Ross S Hall^6^; Allan Stensballe^7^; Yan Yan^8^; Merete Fredholm^1^; Stig M Thamsborg^9^; Peter Nejsum ^10^

^1^Department of Veterinary and Animal Sciences, Faculty of Health and Medical Sciences, University of Copenhagen, Denmark, Copenhagen, Denmark; ^2^Department of Tumor Peter Nejsum Biology, Institute for Cancer Research, The Norwegian Radium Hospital, Oslo University Hospital, Norway, Oslo, Norway; ^3^Department of Clinical Medicine, Faculty of Health, Aarhus University, Denmark, Aarhus, Denmark; ^4^Departament de Farmàcia I Tecnologia Farmacéutica i Parasitologia, Universitat de Valéncia, Spain, BURJASSOT (VALENCIA), Spain; ^5^Population Health and Immunity Division, The Walter and Eliza Hall Institute, Australia, Melbourne, Australia; ^6^Faculty of Veterinary and Agricultural Sciences, The University of Melbourne, Australia, Melbourne, Australia; ^7^Department of Health Science and Technology, Aalborg University, Denmark, Aalborg, Denmark; ^8^Interdisciplinary Nanoscience Center (iNANO), Aarhus University, Denmark, Aarhus, Denmark; ^9^Department of Veterinary and Animal Sciences, Faculty of Health and Medical Sciences, University of Copenhagen, Denmark, Melbourne, Australia; ^10^Aarhus University, Denmark, Aarhus N, Denmark

**Background**: The highly prevalent porcine helminth, *Ascaris suum*, compromise pig health and reduce farm productivity worldwide. The closely related human parasite, *A. lumbricoides*,infects more than 800 million people and causes approximately 1.31 million disability-adjusted life years. These parasites infections are often chronic by nature and have a profound ability to modulate their hosts immune responses. This study provides the first in-depth characterization of extracellular vesicles (EVs) from different developmental stages and body parts of *A. suum* and their potential role in the host-parasite interplay.

**Methods**: EVs were isolated by ultracentrifugation and visualised by Transmission Electron Microscopy and NanoSight. Next Generation Sequencing and proteomics were used to characterise the content of EVs and their functional properties tested on dendritic cells in vitro.

**Results**: The release of EVs during the third larval stage (L3), L4 and adults was demonstrated by Transmission Electron Microscopy, and the uptake of EVs from adult A. suum in intestinal epithelial cells followed by accumulation of RNA in the nucleus by confocal microscopy. Next Generation Sequencing of EV-derived RNA identified a number of micro(mi)RNAs from the different A. suum life stages and body parts and potential transcripts of potential host immune targets, such as IL-13, IL-25 and IL-33, were identified. Proteomics of EVs identified several proteins with immunomodulatory properties and other proteins previously shown to be associated with parasite EVs. Furthermore, EVs from A. suum body fluid stimulated the production of the pro-inflammatory cytokines IL-6 and TNF-α in dendritic cells in vitro.

**Summary/conclusion**: The release of EVs during the third larval stage (L3), L4 and adults was demonstrated by Transmission Electron Microscopy, and the uptake of EVs from adult A. suum in intestinal epithelial cells followed by accumulation of RNA in the nucleus by confocal microscopy. Next Generation Sequencing of EV-derived RNA identified a number of micro(mi)RNAs from the different A. suum life stages and body parts and potential transcripts of potential host immune targets, such as IL-13, IL-25 and IL-33, were identified. Proteomics of EVs identified several proteins with immunomodulatory properties and other proteins previously shown to be associated with parasite EVs. Furthermore, EVs from A. suum body fluid stimulated the production of the pro-inflammatory cytokines IL-6 and TNF-α in dendritic cells in vitro.

**Funding**: The release of EVs during the third larval stage (L3), L4 and adults was demonstrated by Transmission Electron Microscopy, and the uptake of EVs from adult A. suum in intestinal epithelial cells followed by accumulation of RNA in the nucleus by confocal microscopy. Next Generation Sequencing of EV-derived RNA identified a number of micro(mi)RNAs from the different A. suum life stages and body parts and potential transcripts of potential host immune targets, such as IL-13, IL-25 and IL-33, were identified. Proteomics of EVs identified several proteins with immunomodulatory properties and other proteins previously shown to be associated with parasite EVs. Furthermore, EVs from A. suum body fluid stimulated the production of the pro-inflammatory cytokines IL-6 and TNF-α in dendritic cells in vitro.

OS22.04

Discovery of *P. vivax* proteins in plasma-derived exosomes from malaria-infected liver-chimeric (huHep) FRG mice

Melisa Gualdrón-López ^1^; Erika L. Flannery^2^; Niwat Kangwanrangsan^3^; Dietmar Fernandez-Orth^4^; Joan Segui-Barber^5^; Félix Royo^6^; Juan M. Falcón-Pérez^6^; Carmen Fernandez-Becerra^7^; Stefan H.I. Kappe^2^; Jetsumon Sattabongkot^8^; Juan Ramón Gonzalez^4^; Sebastian A. Mikolajczak^2^; Hernando A. del Portillo^9^

^1^ISGlobal, Hospital Clínic - Universitat de Barcelona, Institut d’Investigació Germans Trias i Pujol (IGTP), Badalona, Spain; ^2^Center for Infectious Disease Research, Seattle, WA, USA; ^3^Mahidol Vivax Research Unit, Rachathewi Bangkok, Thailand; ^4^ISGlobal, Barcelona, Spain; ^5^ISGlobal, Hospital Clínic - Universitat de Barcelona, Barcelona, Spain; ^6^CIC bioGUNE, CIBERehd, Bizkaia Science and Technology Park, Derio, Bizkaia, Spain, Derio, Spain; ^7^ISGlobal, Hospital Clínic - Universitat de Barcelona, Institute for Health Sciences Trias I Pujol (IGTP), Badalona, Spain; ^8^Mahidol Vivax Research Unit, Thailand, Rachathewi Bangkok, Thailand; ^9^ISGlobal, Hospital Clínic - Universitat de Barcelona. Institute for Health Sciences Trias I Pujol (IGTP), Badalona, Spain. Catalan Institution for Research and Advanced Studies (ICREA), Barcelona, Spain

**Background**: Exosomes contain molecular signatures implying the cell of origin; thus, they offer a unique opportunity to discover non-invasive biomarkers of disease. *Plasmodium vivax* is a major obstacle in the goal of malaria elimination due to the presence of undetectable dormant liver stages (hypnozoites) that reactivate after the initial infection causing disease. The human liver-chimeric FRG (huHep) mouse is a robust *P. vivax* infection model for development of liver stages, including hypnozoites. We studied the proteome of plasma-derived exosomes isolated from *P. vivax*-infected FRG huHep mice with the objective of identifying *P. vivax* liver-stage biomarkers.

**Methods**: Exosomes were purified from plasma of FRG huHep mice infected with *P. vivax* by size-exclusion chromatography (SEC). Exosomes were characterized by nanoparticle tracking analysis, cryo-electronmicroscopy and bead-based flow cytometry. SEC fractions containing exosome-like vesicles were analysed by mass spectrometry-based proteomics. Validation of identified parasite and human proteins was done by Western blot.

**Results**: Molecular and morphological characterization showed that purified exosomes, from the plasma of FRG huHep mice, were enriched in SEC fractions (7–10) showing a mean size of 121.21 nm and 2.29 × 10^11^ particles/ml. Proteomic analysis of these fractions showed the presence of 290 and 234 proteins from mouse and human origin, respectively, including canonical exosome markers and proteins previously detected in liver-derived exosomes. Remarkably, we identified *P. vivax* proteins including enzymes, surface proteins, components of the endocytic pathway, translation machinery and uncharacterized proteins. Western blot analysis validated the presence of a human liver protein and of an uncharacterized *P. vivax* protein.

**Summary/Conclusion**: This study represents a proof-of-principle that plasma-derived exosomes from *P. vivax* FRG-huHep mice contain human hepatocyte and *P. vivax* proteins with the potential to unveil biological features of liver infection and identify biomarkers of hypnozoite infection. We are currently assessing immunoprecipitation techniques to enrich liver-derived exosomes from plasma to increase the sensitivity to detect *P. vivax* proteins.

**Funding**: This work was supported by MINECO, REDiEX, Gencat (PERIS), ISGlobal.

OS22.05

Development of human reticulocyte-derived exosomes as a new vaccine delivery platform against *Plasmodium vivax* malaria

Miriam Diaz-Varela ^1^; Melisa Gualdrón-López^2^; Armando de Menezes-Neto^3^; Daniel Perez-Zsolt^4^; Ana Gámez-Valero^3^; Joan Segui-Barber^5^; Nuria Izquierdo-Useros^4^; Javier Martinez-Picado^4^; Ricardo Lauzurica-Valdemoros^6^; Carmen Fernandez-Becerra^7^; Hernando A. del Portillo^8^

^1^ISGlobal, Hospital Clínic - Universitat de Barcelona, Barcelona, Spain, Barcelona, Spain; ^2^ISGlobal, Hospital Clínic - Universitat de Barcelona. Institut d’Investigació Germans Trias i Pujol (IGTP), Badalona, Spain; ^3^ISGlobal, Hospital Clínic - Universitat de Barcelona, Barcelona, Spain; ^4^IrsiCaixa AIDS Research Institute, Badalona, Spain; ^5^ISGlobal, Hospital Clínic - Universitat de Barcelona, Barcelona, Spain; ^6^Nephrology Service, Hospital Universitari Germans Trias i Pujol, Badalona, Spain; ^7^ISGlobal, Hospital Clínic - Universitat de Barcelona. Institute for Health Sciences Trias I Pujol (IGTP), Badalona, Spain; ^8^ISGlobal, Hospital Clínic - Universitat de Barcelona. Institute for Health Sciences Trias I Pujol (IGTP), Badalona, Spain. Catalan Institution for Research and Advanced Studies (ICREA), Barcelona, Spain

**Background**: *Plasmodium vivax* is the most widely distributed human malaria parasite. This parasite preferentially invades reticulocytes, cells that selectively remove obsolete proteins through exosome release in their maturation to erythrocytes. Apart from their essential role in erythropoiesis, reticulocyte-derived exosomes (Rex) were shown to be involved in the modulation of the immune response in a murine reticulocyte-prone malaria model resembling *P. vivax*. Rex from this murine malaria infection carried parasite antigens and when used in immunizations adjuvanted with CpG, elicited a spleen-dependent long-lasting protective immune response, thus, suggesting the use of Rex from infections as a potential approach for vaccination against *P. vivax.*

**Methods**: To extrapolate these findings to *P. vivax*, we initially determined the protein composition of human Rex (HuRex). HuRex were isolated from *in vitro* cultures of human cord blood reticulocytes and subjected to mass spectrometry-based proteomics. To avoid technological confounding, we used two different isolation methodologies, ultracentrifugation and size-exclusion chromatography (SEC). Next, we studied the capture of HuRex by monocyte-derived dendritic cells (mDCs). In parallel, plasma-derived exosomes isolated by SEC from naturally *P. vivax*-infected patients (PvEx), which we have shown to contain parasite proteins, were used to study their *in vitro* interaction with sorted immune cell populations from human spleens.

**Results**: HuRex proteomics rendered a list of 418 proteins, where MHC class I molecules and adhesins were identified among others. The presence of MHC class I molecules in HuRex along with their capacity to be captured by mDCs suggests a role of HuRex in antigen presentation. Furthermore, we observed an active uptake of PvEx by human spleen T cells, a population whose distribution was altered by Rex immunization during the protective antimalarial immune response in the murine model.

**Summary/Conclusion**: Further experimentation is guaranteed to determine the role of Rex in antigen presentation and protection against *P. vivax* infections as well as their potential as a new vaccine delivery platform against *P. vivax*.

**Funding**: This worked was funded by Generalitat de Catalunya, MINECO, REDiEX and Fundación Ramón Areces.

OS22.06

Secreted extracellular vesicles from the hookworm-like nematode *Nippostrongylus brasiliensis* prevents inducible colitis in mice

Ramon M. Eichenberger ^1^; Javier Sotillo^1^; Paul R. Giacomin^1^; Matthew A. Field^2^; Alex Loukas^1^

^1^Centre for Biodiscovery and Molecular Development of Therapeutics, Australian Institute of Tropical Health and Medicine, James Cook University, Australia, Cairns, Australia; ^2^Australian Institute of Tropical Health and Medicine, James Cook University, Cairns, Australia,

**Background**: Gastrointestinal (GI) parasites, hookworms in particular, have evolved to cause minimal harm to their hosts, allowing them to establish chronic infections. This is mediated by creating an immunoregulatory environment. Indeed, hookworms are such potent suppressors of inflammation that they have been used in clinical trials to treat inflammatory bowel diseases (IBD) and coeliac disease. Since the recent description of helminths (worms) secreting extracellular vesicles (EVs), vesicles from different helminths have been characterised and their salient roles in parasite–host interactions have been highlighted.

**Methods**: Here, we analyse EVs from the rodent parasite *Nippostrongylus brasiliensis*, which has been used as a model for human hookworm infection. *N. brasiliensis* EVs are actively internalised by mouse gut organoids, indicating a role in driving parasitism. We used proteomics and RNA Seq to profile the molecular composition of *N. brasiliensis* EVs and have begun to evaluate the mechanisms by which these vesicles aid the parasite in evading host immune attack. To determine whether GI nematode EVs had immunomodulatory properties that could protect against IBD, we assessed their potential to suppress GI inflammation in a mouse model of inducible chemical colitis.

**Results**: We identified numerous proteins with potential and known immunoregulatory functions, and 52 miRNA species, many of which putatively map to mouse genes involved in regulation of inflammation. EVs from *N. brasiliensis* but not those from the whipworm *Trichuris muris* or control vesicles from grapes protected against colitic inflammation in the gut of mice that received a single intra-peritoneal injection of EVs. Key cytokines associated with colitic pathology (IL-6, IL-1b, IFNg, IL-17a) were significantly suppressed in colon tissues from EV-treated mice. In contrast, high levels of the anti-inflammatory cytokine IL-10 were detected in *N. brasiliensis* EV-treated mice.

**Summary/Conclusion**: Proteins and miRNAs contained within helminth EVs hold great potential application in development of drugs and vaccines to treat helminth infections as well as chronic non-infectious diseases resulting from a dysregulated immune system, such as IBD.

Symposium Session 23 – Mechanisms of EV Uptake and Biodistribution Chairs: Dave Carter; Maria Yañez-Mó Location: Room 5 13:45–15:15

OS23.01

Casting a line to trailing cells: a simple mechanism for polarizing signalling in the posterior lateral line primordium

Damian E. Dalle Nogare; Ajay B. Chitnis

Eunice Kennedy Shriver National Institute of Child Health and Human Development, National Institutes of Health, Bethesda, USA

**Background**: The zebrafish posterior lateral line primordium (PLLp) is a group of ~150 cells which spearheads the development of the lateral line by migrating along the length of the embryo, periodically depositing epithelial rosettes which serve as sense organ precursors. The PLLp is patterned by juxtaposed and mutually inhibitory Wnt and FGF signalling systems. Wnt in leading cells drives the expression of both FGF ligands and FGF signalling inhibitors. FGF ligand therefore activates receptors in more trailing cells, promoting rosette formation. However, the mechanisms by which this polarity is established and then maintained are incompletely understood.

**Methods**: We used high resolution imaging in live zebrafish embryos mosaically labelled with a membrane GFP to characterize the formation and release of extracellular vesicles during the development of the PLLp.

**Results**: Using high resolution timelapse imaging, we show that leading cells extend long vesicle-bearing fillopodial protrusions, similar to cytonemes, towards trailing cells. Small extracellular vesicles released by these protrusions are taken up by trailing cells and rapidly transported apically, where FGF is known to accumulate in a microlumenal compartment of the epithelial rosette. The extension of these protrusions is sensitive to inhibition of HSPG sulfation, a manipulation also known to prevent an effective FGF response in trailing cells. Furthermore, we show that the direction of extension of these protrusions is highly correlated with the direction and speed of cell migration.

**Summary/Conclusion**: We propose that extracellular-vesicle mediated signalling is, at least in part, responsible for delivering signals from leading cells to trailing cells to in a manner intrinsically tied to the directionality of PLLp movement.

**Funding**: This work was supported by Intramural program of the Eunice Kennedy Shriver National Institute of Child Health and Human Development, National Institutes of Health.

OS23.02

Exploring the role of exosomal surface glycans in cellular uptake

Charles Williams ^1^; Raquel Pazos^2^; Felix Royo^3^; Niels-Christian Reichardt^2^; Juan M Falcon-Perez^3^

^1^CIC bioGUNE - CIC biomaGUNE, Derio, Spain; ^2^CIC biomaGUNE, Donostia, Spain; ^3^CIC bioGUNE, Derio, Spain

**Background**: Exosomes are known to mediate intercellular communication through bioactive protein, RNA and lipid cargoes. However, the functional roles of exosomal glycans are less certain. Herein we investigate the role of these glycans in cell binding and capture, using extracellular vesicles (EVs) from two hepatic murine cell lines, AML12 and MLP29.

**Methods**: We first characterised the surface glycosylation profiles of our EVs models using lectin microarray technology and subsequently modified these native profiles through the application of either PNGaseF or neuraminidase glycosidases. We incubated fluorescently labelled EVs with a panel of cell lines representative of different organ types. The uptake of the EVs was then assayed via flow cytometry and confocal microscopy.

**Results**: EVs derived from AML12 and MLP29 show a glycan profiles in broad agreement with the conserved glycan signature previously reported for mammalian EVs, with strong signals observed from the lectins indicative of high mannose and complex type glycans. We also observed the presence of fucosylated glycans and, contrary to other reports, our EVs exhibited low signals for sialic-binding lectins. Physical characterisation revealed a small but significant alteration in vesicle size and charge for AML12 exosomes upon neuraminidase treatment but no change for MLP29 exosomes. Incubation of cells with glycoengineered EVs revealed a variety of responses depending on the EV treatment and the recipient cells.

**Summary/Conclusion**: Key differences were observed in the cell affinities for glycoengineered exosomes. Our work contributes to a growing body of evidence that exosomal glycans play a functional role in cell binding and uptake, whilst exact effects appear to change between cell types and EV models.

**Funding**: This work was funded by the Ramón Areces Foundation to JMF and is co-supported by CIC bioGUNE and CIC biomaGUNE.

OS23.03

α5β1 integrin regulates cell adhesion to tumoural exosomes

Beatriz Cardeñes ^1^; Raquel Reyes^1^; Yesenia Machado^1^; Soraya López-Martín^2^; María Yáñez-Mó^3^; Carlos Cabañas^1^

^1^CBM-SO, CSIC, Madrid, Spain; ^2^Molecular Biology Center Severo Ochoa (CBM), Instituto de Investigación Sanitaria Princesa (IIS-IP), Madrid, Spain; ^3^Departamento de Biología Molecular. UAM, Madrid, Spain

**Background**: Exosomes are small vesicles which have been identified as vehicles of intercellular communication, since these vesicles are capable to transfer nucleic acids, proteins and lipids to other cells.

**Objective**: To analyse the role of α_5_β_1_ integrin in the processes of binding/fusion of tumour-derived exosomes to recipient cells.

**Methods**: Exosomes were purified by tangential flow filtration and size-exclusion chromatography. We studied α5β1 integrin on the exosomal surface of tumour-exosomes. In addition, we used blocking anti-β1 and anti-α5 monoclonal antibodies (mAbs Lia 1/2 and P1D6, respectively) to study the effects in the binding/fusion of Colo-320 (colocarcinoma cells) and SKOV-3 (ovarian carcinoma)-derived exosomes to recipient cells.

**Results**: Our results show that the integrin α5β1 is in the surface of tumour-exosomes and that anti-β1 and anti-α5 mAbs have inhibitory effects on the adhesion of tumour cells to tumour-derived exosomes.

**Summary/Conclusion**: These results suggest that α5β1integrin regulates the binding/fusion of tumour-derived exosomes to target cells.

**Funding**: This work was supported by grants from Fundación Ramón Areces and Ref.SAF2016-77096 from Ministerio Economía y Competitividad.

OS23.04

A system of cytokines encapsuated in extracellular vesicles

Wendy Fitzgerald^1^; Michael R. Freeman^2^; Michael M. Ledrman^3^; Elena Vasilieva^4^; Roberto Romero^5^; Leonid Margolis ^1^

^1^Section of Intercellular Interactions, Eunice-Kennedy Shriver National Institute of Child Health and Human Development, Bethesda, MD, USA; ^2^Cedars Sinai Medical Center, Los Angeles, CA, USA; ^3^Case Western University, Cleveland, OH, USA; ^4^Moscow University for Medicine and Dentistry, Moscow, Russia; ^5^National Institute of Child Health and Human Development, Detroit, MI, USA

**Background**: Cytokines are classical soluble molecules mediating cell–cell communications in multicellular organisms. Recently, another system of cell–cell communication was discovered, which is mediated by extracellular vesicles (EVs). Here we undertook a first systematic study to investigate the possible role of EVs as carriers of cytokines in eight *in vitro, ex vivo* and *in vivo* biological systems.

**Methods**: We collected samples from (a) cultured T cells, (b) cultured monocytes, (c) explants of tonsillar tissue, (d) explants of cervix, (e) placental villi, (f) amnion tissues, (g) amniotic fluid and (h) blood plasma of healthy volunteers. For each of the systems, we measured 33 cytokines released either in a free (soluble) form, or attached to and/or encapsulated within EVs.

**Results**: (a) In all the *in vitro, ex vivo* and *in vivo* systems, we found EV-associated cytokines; (b) although some cytokines are preferentially released in EVs and others in a free form, any given cytokine can be encapsulated into EVs; (c) the same cytokine in one biological system can be released in association with EVs, while in another system as free (soluble) molecules; (d) in the same biological system, the pattern of cytokine encapsulation into EVs is dramatically changed by system activation; (e) EVs that encapsulate cytokines can deliver them to sensitive cells and trigger their physiological response; (f) EV-encapsulated cytokines were not revealed by standard cytokine assays

**Summary/Conclusion**: The release of cytokines either in a free or in an EV-associated form is tightly regulated and may reflect system adaptation to specific physiological needs, in particular whether these cytokines are needed to act near the secreting cell or at a distance. EV-encapsulated cytokines that have been missed in regular cytokine measurements are a significant part of a general system of cell–cell communication. A better understanding of this system may lead to new therapeutic strategies.

**Funding**: WF, LM and RR were supported by NICHD Intramural Program. MF and ML were supported by The Center for AIDS Research at CWRU [grant AI 36219]. Funding for EV was provided by Russian Federation Government [grant #14.B25.31.0016].

OS23.05

Plug-and-play decoration of isolated EVs with nanobodies improves their cell-specific interactions

Sander A.A. Kooijmans; Jerney J.J.M. Gitz-Francois; Raymond M. Schiffelers; Pieter Vader

Department of Clinical Chemistry and Haematology, UMC Utrecht, The Netherlands

**Background**: Extracellular vesicles (EVs) hold great potential as biocompatible and efficient delivery systems for biological therapeutics. However, the “pre-programmed” tropism of EVs may interfere with their intended pharmaceutical application. We therefore developed a novel method to confer tumour-targeting properties to isolated phosphatidylserine (PS)-exposing EVs in a biocompatible “plug-and-play” fashion.

**Methods**: Anti-EGFR nanobodies (EGa1) or control nanobodies (R2) were fused to PS-binding C1C2 domains of lactadherin and expressed in HEK293 cells. Fusion proteins were purified using affinity chromatography and gel filtration. Protein binding to phospholipids and EGFR was tested using protein-lipid overlay assays and ELISAs. EVs isolated from erythrocytes and Neuro2A cells were mixed with C1C2-nanobodies and purified with SEC. Decorated EVs were characterized by NTA, Western blotting and immuno-electron microscopy. Cellular EV uptake was measured by flow cytometry and fluorescence microscopy.

**Results**: C1C2-nanobodies were obtained at high purity and stored in a stabilizing buffer. The proteins bound specifically to PS and showed no affinity for other EV membrane lipids. In addition, EGa1-C1C2 showed high affinity for EGFR (which is overexpressed in a wide variety of tumours) and inhibited binding of the receptor’s natural ligand EGF, whereas R2-C1C2 did not associate with this receptor. Both proteins spontaneously docked onto membranes of EVs from primary erythrocytes and cultured Neuro2A cells without affecting EV size and integrity. Decoration with EGa1-C1C2 dose-dependently improved EV association with and uptake by EGFR-positive tumour cells, even in presence of an excess of EGFR-negative cells. In contrast, decoration with R2-C1C2 slightly reduced cellular EV uptake.

**Summary/Conclusion**: PS-positive EVs can be decorated with C1C2-fusion proteins in a plug-and-play fashion, circumventing the need to engineer EV secreting cells. We employed this method to introduce tumour-targeting nanobodies onto the surface of isolated EVs, which dramatically improved their cell-specific interactions. This could promote EV cargo delivery in tumours and reduce off-target effects.

**Funding**: This work was supported by SAAK, JJJMG, RMS: ERC-STG #260627; PV: NWO VENI #13667.

OS23.06

TGF beta-1 on extracellular vesicle surface: scratching the surface for orientation, origin and function

Ganesh V. Shelke ^1^; Yin Yanan^2^; Su Chul Jang^3^; Cecilia Lässer^4^; Stefan Wennmalm^5^; Hans Jürgen Hoffmann^6^; Jonas A. Nilsson^7^; Li Li^2^; Yong Song Gho^8^; Jan Lötvall^4^

^1^Krefting Research Centre, Institute of Medicine, University of Gothenburg, Gothenburg, Sweden; ^2^Department of Laboratory Medicine, Shanghai General Hospital, Shanghai JiaoTong University, Shanghai, China, Shanghai, China (People’s Republic); ^3^Krefting Research Centre, Institute of Medicine, University of Gothenburg, Boston, MA, USA; ^4^Krefting Research Centre, Institute of Medicine, University of Gothenburg, Gothenburg, Sweden; ^5^SciLife Laboratory, Royal Institute of Technology, Solna, Sweden; ^6^Department of Respiratory Diseases and Allergy, Aarhus University Hospital, Aarhus, Denmark; ^7^Department of Surgery, Institute of Clinical Sciences, University of Gothenburg, Gothenburg, Sweden; ^8^Department of Life Sciences, Pohang University of Science and Technology, Pohang, Republic of Korea

**Background**: Transforming growth factor1 (TGFb1) has been shown to be associated with extracellular vesicles (EVs) and is shuttled to recipient cells. However, it is not known how TGFb1 associates itself with EVs. This study investigates the “form and topology” of TGFb1 released from human mast cells and how it induces phenotypic changes in human mesenchymal stem cells (MSC).

**Methods**: Primary human mast cells and a human mast cell line HMC1 were used to obtain EVs, using ultracentrifugation and floatation, which was used to determine the distribution of TGFb1 and the coexistence of other EV markers (identifies using membrane proteomics). Antibody-bead based capturing and fluorescence correlation spectroscopy analyses were performed to validate the co-localization of CD63 and TGFb1. TGFb1 signalling was evaluated in MSC upon EV treatment. We also physically traced the localization of EV in recipient MSCs by a novel organelle separation method. Acidification of EVs was performed to determine the presence of the active and inactive forms of TGFb1. Moreover, glycan dependency of TGFb1 was tested by eliminating the surface glycan with Heparinase-II or inhibiting heparan sulphate glycoproteins synthesis in the HMC1 cells.

**Results**: TGFb1 was localized to an EV population that was also positive for tetraspanins (CD63, CD81 and CD9) and flotillin-1. EVs induce the activation of MSCs via phosphorylation of SMAD2/3, which results in enhancing the migratory MSC phenotype. EVs were taken up by MSC, and were retained in the endosomal compartment at a time of activation of the recipient cell, associated with prolonged signalling. EV-associated TGFb1 is more potent than free TGFb1 in inducing recipient cell activation. Both active and inactive form of TGFb1 is associated with HMC1 EVs, but only the inactive form of TGFb1 was depended on heparan sulphate glycoproteins for its binding to EVs.

**Summary/Conclusion**: This study illustrates how TGFb1 is decorated on EVs from mast cells, and delivers its biological function to human MSC in an enhanced manner.

**Funding**: This work was supported by VBG Group Herman Krefting Foundation for Allergy and Asthma Research, Swedish Cancer Foundation, Swedish Research Council and Swedish Heart and Lung Foundation to support this work. GS is supported by EAACI, Assar Gabrielssons, Lundgren, Sahlgrenska University Hospital and Sahlgrenska Academy.

Symposium Session 24 – EV-inspired Therapeutics Chairs: Nobuyoshi Kosaka; Hubert Yin Location: Room 6 13:45–15:15

OS24.01

Dynamic bioreactor systems for clinical-scale production of human amnion epithelial cells-derived extracellular vesicles

Gina D. Kusuma; Dandan Zhu; Jean L. Tan; Mirja Krause; Rebecca Lim

Hudson Institute of Medical Research, Clayton, Australia

**Background**: Human amnion epithelial cells (hAECs) are currently used as cell therapy products in preclinical studies and clinical trials for chronic lung diseases, stroke and liver cirrhosis. These promising regenerative effects are largely attributed to hAECs’ paracrine effect through their secretome. We further investigated the therapeutic potential of extracellular vesicles (EVs) which are released by hAECs in large numbers. To translate EVs therapies to the clinic, development of large-scale clinical manufacturing for EVs isolation and purification is of critical importance. Dynamic bioreactors are routinely used to manufacture cells and cell-derived products. We evaluated commercially available bioreactor systems for scalable hAEC-EV production.

**Methods**: hAECs were cultured under serum-free conditions in traditional 2D culture system, biaxial agitation bioreactor, and fixed bed bioreactor. Cell viability, pH, glucose and lactic acid levels were monitored daily. Conditioned media were sampled daily and potency assessed for immunomodulatory and pro-angiogenic activity, as has been shown in hAECs. EVs were isolated by serial ultracentrifugation; EVs concentration and particle size distribution were measured by nanoparticle tracking analysis.

**Results**: Protein yield and particle numbers were significantly higher in hAECs-EVs cultured in both bioreactors compared to 2D culture after 7 days. However, only hAEC-conditioned medium from biaxial agitation bioreactor showed comparable immunomodulatory properties on T cell proliferation, human umbilical vein endothelial cells angiogenesis and macrophage phagocytosis as expected from 2D culture.

**Summary/Conclusion**: The microenvironment in bioreactor systems altered EV biogenesis in hAECs. The biaxial agitation bioreactor produces higher mass transfer due to its unique mixing pattern and also demonstrates better cell viability for cell suspension systems. Biaxial agitation bioreactor represents a robust and effective method for large-scale clinical grade hAECs-EVs production.

OS24.02

Development of intracellular delivery system based on extracellular vesicles derived from cells in acidic environments

Natsumi Ueno^1^; Mie Matsuzawa^1^; Kosuke Noguchi^1^; Tomoya Takenaka^1^; Tomoka Takatani-Nakase^2^; Tetsuhiko Yoshida^3^; Ikuo Fujii^4^; Ikuhiko Nakase ^1^

^1^NanoSquare Research Institute, Osaka Prefecture University, Sakai-shi, Japan; ^2^School of Pharmacy and Pharmaceutical Sciences, Mukogawa Women’s University, Nishinomiya, Japan; ^3^Keio University School of Medicine, Tsukuba, Japan; ^4^Graduate School of Science, Osaka Prefecture University, Sakai-shi, Japan

**Background**: Extracellular vesicles (exosomes, EVs), secreted by various cell types, contain bioactive molecules (e.g. microRNAs). EVs have been shown to participate in cell-to-cell communications including cancer and other diseases. Environmental conditions of the related cells have been shown to affect the EV-based cell-to-cell communications; however, detailed mechanisms are still unknown. In this research, we studied the effects of environmental pH conditions on secretion and cellular uptake efficacy of EVs. We here also demonstrate modification of arginine-rich cell-penetrating peptides on the isolated EVs for development of intracellular delivery system based on EVs.

**Methods**: Secreted EVs were isolated via ultracentrifugation of HeLa cells stably expressing GFP-fused CD63 (an EV (exosome) membrane marker protein) cultured in different pH conditions. All peptides were prepared by Fmoc solid-phase synthesis.

**Results**: Even though pH reduction in cell culture condition decreases the cellular proliferation speed, we found that the pH condition significantly enhanced secretion efficacy of EVs with increased zeta potential. Expression level and location of GFP-fused CD63 in the original cells (HeLa cells stably expressing GFP-fused CD63) were also intensively affected by the environmental pH condition analysed using a confocal laser microscope. In addition, increased cellular uptake efficacy of EVs, which were isolated from the cells cultured in low pH condition, was observed, and the efficacy was influenced by addition of serum in the cell culture medium. Modification of arginine-rich cell-penetration peptides on the isolated EVs also resulted in further enhanced cellular uptake efficacy, suggesting useful techniques for intracellular delivery of therapeutic molecules based on EVs.

**Summary/Conclusion**: Our findings may contribute to understanding the mechanisms of EV-based cell-to-cell communications affected by environmental conditions and to developing EV-based intracellular delivery system.

OS24.03

Towards extracellular vesicles as versatile biogenic drug delivery system: loading method by facilitated fusion with liposomes of tunable membrane and inner composition

Max Piffoux ^1^; Amandine Pinto^2^; Alba Nicolas boluda^3^; Claire Wilhelm^3^; Marc Pocard^2^; Florence Gazeau^3^; Amanda K A Silva^3^; David Tareste^4^

^1^Laboratoire Matière et Systèmes Complexes, Paris, France; ^2^Unité mixte de recherche 965 - ART : Carcinose angiogenèse et recherche translationnelle, Paris, France; ^3^laboratoire Matière et Systèmes Complexes, Paris, France; ^4^U894 Centre de Psychiatrie et de Neuroscience, Paris, France

**Background**: On the road towards the clinical use of extracellular vesicles (EVs) as natural drug delivery system, one major challenge remains to load EVs with various drugs of interest and/or to engineer EV membrane to make biogenic EVs as versatile as synthetic liposomes.

**Methods**: We designed a new EV/liposome fusion technology as a tool to solve the EV loading challenge. The concept relies on the use of polyethylene glycol (PEG) to induce fusion of EVs with drug-loaded liposomes of different compositions, allowing the production of hybrids EVs with new properties like PEGylated EVs and/or drug-loaded EVs. This method is combined to a new high yield method for production and loading of neutral precursor liposomes.

**Results**: The liposome production method allows encapsulation of up to >80% of virtually any hydrophilic or lipophilic compounds such as sulforhodamine B, inorganic 5–20-nm nanoparticles, siRNA or fluorescent lipids into 50–100-nm neutral liposomes. Depending on fusion parameters and liposome composition, PEG-facilitated fusion of EVs with liposomes allows the transfer to mesenchymal stem cells-derived EVs of up to >95% from different liposomal lipophilic drugs or functionalized lipids and >40% from hydrophilic inner compounds (rhodamine/siRNA). The resulting hybrid EVs keep their endogenous biological activity and display additional tunable functionalities coming from liposomes.

Hybrid EVs display a three- to fourfold increase in tumour cell internalization compared to precursor liposomes *in vitro* with related increase in the therapeutic effect using an FDA-approved photosensitizer agent (Foscan) as light-activated therapeutic cargo. *In vivo* biodistribution patterns show increased accumulation in tumours compared to healthy tissues in an orthotopic peritoneal carcinomatosis mouse model. Therapeutic studies are ongoing.

**Summary/Conclusion**: EV/liposome fusion, coupled to high yield production of drug-loaded neutral liposomes, allows to boost the EV loading efficiency even for hydrophilic drugs, rendering feasible the democratization and standardization of EV-based drug delivery systems.

OS24.04

Allogenicity boosts exosome-induced antigen-specific humoral and cellular immunity and mediate long-term memory *in vivo*

Susanne Gabrielsson; Pia Larssen; Rosanne Veerman; Gözde Gucluler; Stefanie Hiltbrunner; Mikael Karlsson

Karolinska Institutet, Stockholm, Sweden

**Background**: Exosomes are interesting as potential cancer immunotherapy vehicles due to their capacity to stimulate tumour-specific activity in mice. However, clinical trials using peptide-loaded autologous exosomes showed only moderate T cell responses, suggesting a need for optimization of exosome-induced therapy in humans. We previously demonstrated that the presence of antigen-specific CD8^+^ T cells and anti-tumour responses to whole antigen were independent of major histocompatibility complex on exosomes and hypothesized that repeated injections of allogeneic exosomes would potentiate antigen-specific responses.

**Methods**: Allogeneic or syngeneic exoxomes derived from bone-marrow-derived dendritic cells were injected once or twice into C57BL/6 mice, and immune responses were measured by flow cytometry, ELISA and ELISPOT. Exosomes were analysed by electron microscopy, NanoSight, fluorescence-activated cell sorting, Western blot and ELISA. Exosomes were also given as treatment in the B16 melanoma model.

**Results**: Two injections of allogeneic exosomes enhanced antigen-specific CD8+ T cell, germinal center B cell and follicular helper T cell and antigen-specific antibody responses compared to syngeneic exosomes. Exosome-injected mice demonstrated antigen-specific memory after 4 months, with highest antibody avidity in mice receiving double allogeneic exosome injections. Furthermore, allogeneic exosomes were more potent than syngeneic to delay cancer progression in a melanoma mouse model.

**Summary/Conclusion**: Our findings support the use of allogeneic exosomes over syngeneic for therapeutic use in clinical studies where an adaptive immune response is desired.

**Funding**: This work was supported by Swedish Medical Research Council, the Cancer and Allergy Foundation, the Swedish Cancer Foundation, and the Radiumhemmets Research Foundations.

OS24.05

RNA nanoparticle orientation to control ligand display on exosomes for cancer regression

Daniel W. Binzel ^1^; Fengmei Pi^1^; Tae Jin Lee^2^; Zhefeng Li^1^; Meiyan Sun^3^; Piotr Rychahou^4^; Hui Li^1^; Farzin Haque^1^; Shaoying Wang^1^; Carlo Croce^2^; Bin Guo^3^; Mark Evers^4^; Peixuan Guo^5^

^1^College of Pharmacy; Center for RNA Nanobiotechnology and Nanomedicine; Comprehensive Cancer Center, Dorothy M. Davis Heart and Lung Research Institute, College of Medicine; The Ohio State University, Columbus, USA; ^2^Comprehensive Cancer Center, College of Medicine; The Ohio State University, Columbus, USA; ^3^Department of Pharmacological and Pharmaceutical Sciences, College of Pharmacy; University of Houston, Houston, USA; ^4^Markey Cancer Center; Department of Surgery; University of Kentucky, Lexington, USA; ^5^College of Pharmacy; Center for RNA Nanobiotechnology and Nanomedicine; Comprehensive Cancer Center, Dorothy M. Davis Heart and Lung Research Institute, Department of Cancer Biology and Genetics, College of Medicine; The Ohio State University, Columbus, USA

**Background**: Exosomes show promise for the delivery of therapeutics due to their ability to deliver high levels of payloads by fusion with cells, yet lack specific targeting to diseased cells leading to toxicities. RNA nanoparticles can specifically target cancer cells but undergo endosome entrapment limiting their therapeutic impact. Here benefits of the two technologies are combined to specifically delivery small interfering RNAs (siRNAs) at a high payload.

**Methods**: Exosomes isolated from HEK293T cells were purified by centrifugation with addition of a high density cushion to prevent destruction from centrifugation forces. Arrow-shaped RNA nanoparticles containing cancer-targeting moieties were decorated on exosome surfaces by hydrophobic cholesterol labels. siRNA was loaded into exosomes as payloads. Decorated exosomes were then tested against three cancer lines for therapeutic assessment.

**Results**: It was shown that arrow shape of the RNA nanoparticles led to either internalization or surface display on exosomes. Placing the anchoring cholesterol on the arrow-tail results in display of RNA aptamer or folate on the exosome surface. Placing the cholesterol at the arrow-head results in partial loading of RNA nanoparticles into the exosome. Resulting exosomes were competent for specific delivery of siRNA, and efficiently blocked tumour growth in prostate cancer xenograft, orthotopic breast cancer and patient-derived colorectal cancer *in vivo* models. Results show knockdown of survivin gene by siRNA delivery and no signs of toxicity.

**Summary/Conclusion**: Here we combine the targeting advantages of RNA nanotechnology with the delivery efficiency of exosomes overcoming roadblocks of both technologies, and provide an efficient method for ligand display to exosome for specific *in vivo* cell targeting.

**Reference**: F Pi, et al, P Guo. Nanoparticle orientation to control RNA loading and ligand display on extracellular vesicles for cancer regression. Nat Nanotechnol. 2018 Jan;13(1):82–89.

**Funding**: The research was supported mainly by National Institutes of Health grants UH3TR000875 and U01CA207946 (to PG), and partially by R01CA186100 (to BG),

R35CA197706 (to C.M.C.), P30CA177558 and R01CA195573 (to B.M.E.).

OS24.06

Mesenchymal stem cell-derived extracellular vesicles delivered in a thermosensitive gel are effective healing mediators in porcine and murine models of digestive fistula

Gabriel Rahmi^1^; Max Piffoux ^2^; Jeanne Volatron^3^; Guillaume Perrod^1^; Laetitia Pidial^4^; Claire Wilhelm^5^; Olivier clément^1^; Florence Gazeau^5^; Amanda K A Silva^5^

^1^Hopital Européen Georges Pompidou, APHP and PARCC, INSERM U970, Université Sorbonne Paris Cité (USPC), Université Paris Descartes, Paris, France; ^2^Laboratoire Matière et Systèmes Complexes, Paris, France; ^3^Laboratoire Matière et Systèmes Complexes, CNRS UMR 7047 Université Paris Diderot, 10 rue Alice Domon et Léonie Duquet, France, France; ^4^INSERM U970 - PARCC, PARIS, France; ^5^Laboratoire Matière et Systèmes Complexes, Paris, France

**Background**: Digestive fistulas are disabling and challenging conditions. We explored, in a porcine fistula model and in a murine fistula model, the healing potential of allogenic extracellular vesicles (EVs) derived from porcine adipose tissue stromal cells (ADSCs) and from mechanically stressed murine mesenchymal stem cells (MSC), respectively, both administered at the fistula site through a thermoresponsive pluronic F-127 gel.

**Methods**: Esophageal fistulas were surgically created in pigs by placing two plastic stents during 30 days into the neck of pigs. Colon-skin fistulae were surgically created in rats. Animals were randomized into a control group, a group treated with gel and a group treated with gel containing 1.3 × 10^11^ EVs/ml. Clinical, endoscopic and radiological evaluation of pig fistula healing was performed at day 30 and day 45, before histological assessment.

**Results**: ADSC EVs displayed pro-angiogenic and pro-survival properties *in vitro*. In pigs *in vivo*, complete fistula healing was reported to be 100% for the gel + EVs group, 67% for the gel group and 0% for the control. Only the combination of gel and EVs resulted in a statistically significant (i) reduction of fibrosis, (ii) decline of inflammatory response, (iii) decrease in the density of myofibroblasts and (iv) increase of angiogenesis. In rats, similar results were obtained with the diminution of fistula diameter and diminution of output in the gel + EV group compared to the control.

**Summary/Conclusion**: This study provides evidences that ADSC-EVs and MSC-EVs delivered into a thermosensitive gel can induce a therapeutic effect in both preclinical swine and murine fistula model. It opens up new prospects for local minimally invasive EV delivery based on a thermo-actuated administration strategy in fistula therapy and beyond. The combination of gel with EVs may represent the next-generation therapeutic options for fistula management deserving to reach further pharmaceutical development steps towards clinical investigation.

Symposium Session 25 – EVs and Viral Infections Chairs: Leonid Margolis; Alissa Weaver Location: Auditorium 15:45–17:15

OS25.01

Human immunodeficiency virus Tat-mediated synaptic alterations – platelet-derived growth factor as a therapeutic strategy

Shilpa Buch; Guoku Hu; Fang Niu; Ke Liao

University of Nebraska Medical Center, Omaha, USA

**Background**: While combination antiretroviral therapy (cART) has resulted in a dramatic drop in viremia, persistence of HIV protein Tat results in a neuroinflammatory milieu, leading to neurocognitive impairment – NeuroHIV. Reversible synaptodendritic injury is a hallmark feature of NeuroHIV. Our findings demonstrate Tat-mediated induction of astrocyte-extracellular vesicle (EV)-miR-7, that upon uptake by the neurons, leads to synaptic impairment with downregulation of neuroligin (NLGN)-2. NLGNs comprise of cell adhesion proteins that regulate synaptic architecture and remodelling. PDGF-CC is a neuroprotective agent that has proven efficacy in various preclinical models of neurodegeneration. Current study was aimed at identifying the role of NLGNs in Tat-astrocyte-EV-miR-7-mediated neuronal injury and the neuroprotective role of PDGF-CC in reversing this process.

**Methods**: EVs were isolated from Tat-stimulated mouse/human primary astrocytes using the standard differential ultracentrifugation method and characterized by transmission electron microscopy, NanoSight and Western blot analyses. miR-7 levels in EVs were determined using real-time PCR. Uptake of astrocytic EVs by neurons was assessed by confocal microscopy. Rodent hippocampal neurons were exposed to EVs from Tat-stimulated astrocytes and assessed for inhibitory (GAD65 and gephyrin) and excitatory (vGlut1 and PSD95) synapses by immunostaining and confocal microscopy.

**Results**: miR-7 was increased in the astrocytes from SIV+/HIV+ brains. Tat-stimulated astrocytes upregulated induction and release of miR-7 in EVs that were taken up by neurons, resulting in synaptic injury. EV-miR-7 targeted neuronal NLGN2 and PDGF-CC pretreatment restored EV-miR-7-mediated synaptic injury.

**Summary/Conclusion**: EVs released from HIV Tat-stimulated astrocytes demonstrated upregulation of miR-7, which in turn, was shown to target neuronal NLGN2, leading to synaptic loss. PDGF-CC restored Tat-astrocyte EV-miR-7-mediated downregulation of NLGN2 and associated synaptic loss.

**Funding**: This work was supported by grants MH112848, DA040397, MH106425 (to SB), and DA042704 (to GH) from the National Institutes of Health. The support by Nebraska Center for Substance Abuse Research is acknowledged.

OS25.02

Exosomal release of the human cytomegalovirus-encoded chemokine receptor US28

Maarten P. Bebelman ^1^; Jeffrey van Senten^1^; René J.P. Musters^2^; D. Michiel Pegtel^3^; Martine J. Smit^1^

^1^Division of Medicinal Chemistry, Amsterdam Institute for Molecules Medicines and Systems, VU University Amsterdam, Amsterdam, The Netherlands., Amsterdam, The Netherlands; ^2^Departement of Physiology and Advanced Optical Microscopy Core in O|2, VU University Medical Center, HV Amsterdam, The Netherlands; ^3^Exosome Research Group, Dept. Pathology, Cancer Center Amsterdam, VU University Medical Center, Amsterdam, The Netherlands

**Background**: The human cytomegalovirus (HCMV) is a widespread human herpesvirus that causes a lifelong latent infection. Although this infection is generally asymptomatic in healthy individuals, HCMV has been associated with the development of various types of cancer, including glioblastoma. One of the key proteins responsible for the oncomodulatory effect of HCMV is the viral chemokine receptor US28, which is expressed during both latent and lytic stages of HCMV infection. This viral receptor localizes to multivesicular bodies (MVBs) and constitutively activates proliferative and pro-angiogenic signalling pathways. We hypothesize that exosomal release of US28 might contribute to HCMV pathology.

**Methods**: We developed an optical reporter based on US28 and a pH-sensitive GFP (pHluorin) that enables live cell imaging of the fusion of US28-containing MVBs with the plasma membrane. Furthermore, we generated an HCMV strain containing US28-pHluorin to study exosomal release of US28 in HCMV-infected cells.

**Results**: Live cell total internal reflection fluorescence microscopy on HCMV-infected cells revealed that US28-pHluorin-containing MVBs fuse with the plasma membrane. In line with this, extracellular vesicles (EVs) isolated from the culture supernatant of infected cells contain US28. Moreover, analysis of the EV-fraction by super-resolution stimulated emission depletion microscopy confirmed the presence of US28-pHluorin-positive EVs with a diameter of 50–100 nm, corresponding to the size of exosomes.

**Summary/Conclusion**: Together, these results suggest that HCMV-infected cells release US28 via exosomes. In future studies, the US28-pHluorin system can be used to study the functional consequences of US28 exosome release and to identify potential strategies to block exosomal communication by HCMV.

**Funding**: This research was funded by a Dutch Organization for Scientific Research – Amsterdam Institute for Molecules, Medicines and Systems STAR Graduate Program grant (022.005.031) to M.P. Bebelman.

OS25.03

JC polyomavirus uses extracellular vesicles to infect target cells

Jenna Morris-Love ^1^; Bethany O’Hara^1^; Aisling S. Dugan^2^; Walter J. Atwood^1^; Gretchen V. Gee^1^

^1^Brown University, Providence, USA; ^2^Assumption College, Worcester, USA

**Background**: JC polyomavirus (JCPyV) is a non-enveloped virus that establishes persistent infection in humans and causes the neurodegenerative disease progressive multifocal leukoencephalopathy (PML) in immunocompromised patients. JCPyV uses a two-step entry mechanism involving attachment to the sialic acid moiety of lactoseries tetrasaccharide C (LSTc), followed by interaction with type 2 serotonin receptors that facilitate entry. Virus from PML patients contains mutations that disrupt LSTc binding, indicating that JCPyV may use an alternative pathway to infect cells. Here, we investigate the role of extracellular vesicles in JCPyV infection.

**Methods**: Extracellular vesicles (EVs) were purified from supernatants of infected glial cells (SVGA) or HEK293FT cells by differential centrifugation. Purified EVs were characterized by transmission electron microscopy and Western blot for EV and viral markers. Purified EVs were used to infect SVGA cells and infection was scored by indirect immunofluorescence analysis using antibodies against JCPyV VP1 at 3 days post infection. EVs containing wild-type and PML-mutant pseudoviruses (L54F and S268F) were assayed by measuring luciferase activity at 2 and 5 days post transduction.

**Results**: JCPyV-infected cells produce EVs containing infectious virus and the EVs efficiently transmit the infection to target cells. EVs produced from JCPyV-infected cells have virus inside the vesicles and bound to the outside of the vesicles. EV-mediated infection is not dependent on sialic acid, and EVs can uniquely transmit PML-mutant viruses to SVG-A cells.

**Summary/Conclusion**: JCPyV can use extracellular vesicles to infect target cells.

OS25.04

Extracellular vesicles released by Zika-infected cells carry viral components – implications for viral dissemination and pathogenesis

Armando Menezes-Neto; Conceição E. A. Silva; Rafael F. O. França

Instituto Aggeu Magalhães - FIOCRUZ, Recife, Brazil

**Background**: The recent epidemics of Zika have revealed that infection can lead to devastating outcomes, prompting WHO to declare it a global public health emergency. Although usually associated to mild symptoms, Zika has neuroinvasive properties, and can cause neurological complications in adults and severe congenital malformations. ZIKV is an enveloped positive-strand RNA Flavivirus. There are pending questions regarding how the virus disseminates from its point of entry to new host cells and which strategies it uses to gain access to restricted sites such as the central nervous system of the foetus. extracellular vesicles (EVs) are implicated in viral dissemination as carriers of infectious viral components and as mediators of receptor-independent viral transmission. Thus, we hypothesize that EVs might be involved in the spread of Zika to and among neural cells and may also act as a vehicle for the crossing of the placental barrier. Therefore, we aimed to characterize the EVs released from ZIKV-infected cells by surveying for the presence of viral antigens or genomic material, and determine whether these EVs can contribute to the establishment of infection or to the development of the distinctive pathogenicity of Zika.

**Methods**: Two human cell lines, glioblastoma and neuroblastoma-derived, were infected with an Asian strain of ZIKV at a MOI of 1 and kept in culture in EV-depleted media for 72 h. Supernatants were submitted to EV enrichment by ultracentrifugation (UC). Preparations were further processed by density gradient and magnetic-based selection of vesicles, and were characterized by transmission electron microscopy (TEM), Western blotting (WB) and RT-qPCR.

**Results**: Zika-infected cells release a mixture of viral particles and EVs that are co-enriched by UC, as revealed by TEM. Viral genomic material and non-structural proteins can still be detected by RT-qPCR and WB after EVs are further isolated by positive selection in magnetic columns.

**Summary/Conclusion**: In addition to virions, Zika-infected cells release EVs that carry viral components. These EVs could contribute to viral dissemination.

**Funding**: This work was funded by Fundação de Amparo à Ciência e Tecnologia do Estado de Pernambuco – FACEPE; Conselho Nacional de Desenvolvimento Científico e Tecnológico – CNPq; and Fundação Instituto Oswaldo Cruz – FIOCRUZ.

OS25.05

Microglia respond to HIV-1 protein Nef expression by releasing distinct extracellular vesicle population

Pia Pužar Dominkuš^1^; Matjaž Stenovec^2^; Jana Ferdin^1^; Simona Sitar^3^; Saša Trkov Bobnar^2^; Eva Lasič^2^; Ana Plemenitaš^1^; Boris Matija Peterlin^4^; Ema Žagar^3^; Marko Kreft^2^; Metka Lenassi ^1^

^1^University of Ljubljana, Faculty of Medicine, Institute of Biochemistry, Ljubljana, Slovenia; ^2^University of Ljubljana, Faculty of Medicine, Institute of Pathophysiology, Laboratory of Neuroendocrinology-Molecular Cell Physiology, Ljubljana, Slovenia; ^3^National Institute of Chemistry, Department of Polymer Chemistry and Technology, Ljubljana, Slovenia; ^4^University of California San Francisco, Department of Medicine, San Francisco, USA

**Background**: Microglia not only protect the central nervous system against injury or infection but also promote neurodegeneration when activated improperly or serve as HIV-1 cellular reservoirs. We here examined the effect of HIV-1 protein Nef expression on intracellular biogenesis and extracellular release of vesicles (extracellular vesicles, EVs) from human microglia.

**Methods**: We have studied intracellular and extracellular vesicles in Nef-expressing (transfected or HIV-1 infected) immortalized human microglia by live confocal and electron microscopy, asymmetric-flow field-flow fractionation connected to detectors, flow cytometry, nanoparticle tracking analysis and immunoblotting of subcellular fractions and EVs.

**Results**: Nef-particles in Nef-expressing microglia comprise large, intracellular Ca^2+^ concentration-independent, non-directional mobile population, which differs in mobility to dextran-laden or Lysotracker-laden endo-/lysosomes. Nef-particles differ from late endosomes/lysosomes also in terms of abundance, size (area) and protein markers. Importantly, Nef-particles significantly co-localize with organelles immunopositive for tetraspanins CD9 and CD81, likely representing the plasma membrane-derived compartments previously connected to HIV-1 assembly. After release, EVs are in higher concentrations (up to 30×), smaller in size (average root mean square roughness (*R*_rms_) 172 nm), float on sucrose gradient in exosome fractions (positive for flotillin, Tsg101, annexin) and some contain Nef (≥2%), when compared to constitutively released EVs (around 5 × 10E7 EVs/10E6 cells; average *R*_rms_ 365 nm). Nef is released with flotillin-positive EVs also from HIV-1 infected microglia.

**Summary/Conclusion**: Microglia respond to Nef expression by releasing distinct EV population, likely promoting HIV-1 pathogenesis. This is also the first report to propose that microglial CD9- and CD81-positive plasma membrane-derived compartments are associated with EV biogenesis and Nef release.

**Funding**: This work was supported by the Slovenian Research Agency (ARRS) [research grants J3-5499, P1-170, P3-310].

OS25.06

Identifying novel cellular components specifically incorporated into HIV versus exosomes and other small EVs

Lorena Martin-Jaular ^1^; Zhaohao Liao^2^; Pehuen Gerber^3^; Matias Ostrowski^4^; Kenneth Witwer^2^; Georg Borner^5^; Clotilde Thery^6^

^1^Institut Curie, Inserm U932- Centre d’immunothérapies des Cancer, Paris, France; ^2^The Johns Hopkins University School of Medicine, Baltimore, MD, USA; ^3^INBIRS Insitute, School of Medicine, University of Buenos Aires, Buenos Aires, Argentina; ^4^INBIRS Institute, School of Medicine, University of Buenos Aires, Buenos Aires, Argentina; ^5^Department of Proteomics and Signal Transduction, Max Planck Institute of Biochemistry, Martinsried, Germany; ^6^Institut Curie / PSL Research University / INSERM U932, Paris, France

**Background**: HIV buds from infected cells by a mechanism that shares many aspects with the biogenesis of small extracellular vesicles (sEVs). Consequently, sEVs and HIV share many physical and chemical characteristics, which make their separation difficult. For this reason, the function of sEVs during HIV infection remains unclear. Here, we used a novel un-biased approach to identify the cellular components specifically incorporated into either HIV or sEVs

**Methods**: Jurkat cells were infected with VSV-G-pseudotyped NL4-3 virus. EVs were obtained by differential centrifugation of medium conditioned by non-infected and HIV-infected cells. Velocity OptiPrep gradient was used to further separate sEVs from virus. EVs were analysed by Western blotting (WB) for the presence of different markers previously described in sEVs and/or HIV. Fractionation profiling was performed from quantitative proteomic analyses of EVs from Jurkat cells labelled with SILAC amino acids.

**Results**: OptiPrep gradients revealed different types of sEVs in the non-infected and in the HIV-infected cells, with insufficient discrimination achieved by the presence of AChE or CD45, markers that putatively discriminate EVs from HIV. In addition, separation of different particles was not possible due to overlap of markers between fractions. We used a global proteomic approach to identify novel specific markers of the virus or sEV subtypes. Two biological replicates of infected and non-infected samples were analysed. Principal component analysis reveals that the HIV proteins form a cluster very close to several sEV markers. Comparison of fractions from non-infected and HIV-infected cells led us to identify candidate proteins that changed location within the different types of vesicles after infection, either moving towards or away from the HIV cluster. Validation of a short list of candidates was done by WB after differential centrifugation of conditioned medium.

**Summary/Conclusion**: OptiPrep gradients showed imperfect association of classical protein markers to sEVs or virus. Using a quantitative proteomic approach, we have defined a short list of novel marker candidates that have been validated by WB. Our results will allow obtaining HIV-free sEVs and assessing their function during the course of HIV infection

Symposium Session 26 – EV-based Non-cancer Biomarkers Chairs: Carolina Soekmadji; Hidetoshi Tahara Location: Room 5 15:45–17:15

OS26.01

Extracellular vesicle biomarkers predict response to experimental treatment in a clinical trial in Parkinson’s disease

Dimitrios Kapogiannis ^1^; Dilan Athauda^2^; Seema Gulyani^1^; Hanuma Karnati^1^; Nigel Greig^1^; Thomas Foltynie^2^

^1^National Institute on Aging/National Institutes of Health (NIA/NIH), Baltimore, MD, USA; ^2^University College London, London, UK

**Background**: Brain insulin resistance (IR) is implicated in Parkinson’s disease (PD) pathogenesis. Exenatide, a GLP-1 analogue that in animal models reverses IR, generated positive results in a recent clinical trial. We previously detected insulin pathway markers in neuronal origin-enriched plasma/serum extracellular vesicles (EVs) including pY-IRS1, pSer-IRS1, AKT and mTOR. We analysed samples from the exenatide trial to assess whether EV biomarkers change with exenatide and predict clinical benefits.

**Methods**: We isolated neuronal origin-enriched EVs using Exoquick followed by L1CAM immunoprecipitation from serum of 60 participants with PD, at baseline, 24 and 48 weeks post-randomization (exenatide 2 mg or placebo once weekly), and after a 12-week washout (60 weeks). Using repeated measures models, we analysed differences in biomarkers covarying EV concentration and size to normalize for differential EV yield. To determine whether changes in EV biomarkers were related to the primary clinical motor outcome, we used linear regression against MDS-UPDRS part 3.

**Results**: Compared to placebo, exenatide promoted activating phosphorylations on IRS-1 tyrosine residues and downstream substrates including Akt and mTOR at 24, 48 and 60 weeks. Furthermore, the beneficial clinical effects of exenatide on motor function (MDS-UPDRS part 3 changes) were associated with EV biomarker changes suggesting reduction in neuronal IR and concomitant activation of mTOR signalling.

**Summary/Conclusion**: The results suggest target engagement of insulin/Akt/mTOR signalling pathways in neurons by exenatide and provide a mechanistic context for the clinical findings of the trial. EV biomarkers may be used to follow molecular target engagement, thereby revolutionizing clinical trials in neurodegenerative diseases and beyond.

**Funding**: This research was supported in part by the Intramural Research Program of the NIH, National Institute on Aging.

**Reference**: 1. Athauda D, et al. Exenatide once weekly versus placebo in Parkinson’s disease: a randomised, double-blind, placebo-controlled trial. Lancet. 2017.

OS26.02

Validation of human cerebrospinal fluid microRNAs as biomarkers for Alzheimer’s disease

Julie Saugstad ^1^; Jack Wiedrick^1^; Jodi Lapidus^1^; Ursula Sandau^1^; Theresa Lusardi^1^; Christina Harrington^1^; Trevor McFarland^1^; Babett Lind^1^; Douglas Galasko^2^; Joseph Quinn^1^

^1^Oregon Health & Science University, Portland, USA; ^2^The University of California, San Diego, San Diego, USA

**Background**: The discovery of extracellular RNAs in cerebrospinal fluid (CSF) raised the possibility that miRNAs may serve as biomarkers of Alzheimer’s disease (AD). Our discovery studies identified a set of miRNAs that can discriminate AD from controls. Here we analyse the expression of AD-specific miRNAs in a new and independent cohort of CSF donors, in order to validate their performance as biomarkers for AD.

**Methods**: CSF from 47 AD and 71 control donors were obtained from the Shiley Marcos AD Research Center at UC, San Diego. The expression of 36 candidate miRNA biomarkers was analysed using TaqMan® Low Density Custom miRNA Arrays. Stringent data analysis included seven different classifying methods (LogRank, ROC, CART, CFOREST, CHAID, BOOST, UH2 discovery assessment), each used to independently rank the candidate markers in order (1 = best, 26 = worst). The total score for each miRNA provided a ranking for each candidate biomarker. Multimarker modelling and covariate analysis were performed on the top-ranking miRNAs. Classification performance of miRNA biomarkers were compared to that of ApoE4 genotype, and incremental improvement adding miRNA biomarkers to ApoE4 was assessed.

**Results**: Data analysis validated that the candidate miRNAs discriminate AD from controls in a new and independent cohort of donors. Cluster analysis revealed 26 miRNAs in three rank groups. Analysis of the contribution of individual miRNAs to multimarker performance revealed 14 best miRNAs. Top-performing linear combinations of six and seven miRNAs have area under the curve (AUC) of 0.775–0.796, relative to ApoE4+ AUC of 0.637 in this sample set. Addition of ApoE4 genotype to the model also improved performance, i.e. AUC of 7 miRNA plus ApoE4 improves to 0.82.

**Summary/Conclusion**: We have validated that CSF miRNAs discriminate AD from controls. Combining the top 14 miRNAs improves sensitivity and specificity of biomarker performance, and adding ApoE4 genotype improves classification.

**Funding**: This work was funded by NIH NCATS UH3TR000903 (to JAS and JFQ), and NIA AG08017 (to JFQ).

OS26.03

Identification of microRNAs from extracellular vesicles as potential biomarkers for frontotemporal dementia

Laura Cervera-Carles ^1^; Ignacio Illán-Gala^1^; Daniel Alcolea^1^; Isabel Sala^1^; Belén Sánchez-Saudinós^1^; Olivia Belbin^1^; Estrella Morenas-Rodríguez^1^; María Carmona-Iragui^1^; Oriol Dols-Icardo^1^; Laia Muñoz-Llahuna^1^; Ana Gamez-valero^2^; Katrin Beyer^3^; Rafael Blesa^1^; Juan Fortea^1^; Alberto Lleó^1^; Jordi Clarimón^1^

^1^Memory Unit, Neurology Department, IIB Sant Pau, Hospital de la Santa Creu i Sant Pau, Universitat Autonoma de Barcelona, Barcelona, Spain; ^2^HUGTiP and IGTP Institute with the Universitat Autónoma de Barcelona, BADALONA, Spain; ^3^Department of Pathology, Hospital Universitari and Health Science Research Institute Germans Trias i Pujol, Universitat Autonoma de Barcelona, Badalona, Spain

**Background**: Frontotemporal dementia (FTD) is a heterogeneous entity with several known causal genes, mainly related to RNA regulation. Recent studies have revealed the important role of microRNAs, involved in the modulation of gene expression, in the physiopathology of FTD. Extracellular vesicles (EVs), containing microRNAs and being present in all biofluids, could act as intermediates in intercellular communication and target signalling pathways related to this disease. This study aims at identifying microRNAs contained in cerebrospinal fluid (CSF) EVs that could be useful as diagnostic biomarkers for FTD.

**Methods**: EV-associated microRNA levels were determined in 72 CSF samples from patients within the FTD spectrum and neurologically healthy controls. EVs were characterized by bead-based flow cytometry, using three exosome markers: tetraspanins CD9, CD63 and CD81. MicroRNA levels were quantified by qPCR, using oligonucleotides with locked nucleic acids. The study comprised a screening (752-microRNA panels) in a subset of samples and a subsequent analysis of potential candidates (26-microRNA panels) in the whole study group.

**Results**: All three tetraspanins were present in the EV-enriched fraction isolated from 250 µL CSF. The amount of RNA extracted from the EV-enriched fraction proved to be enough to obtain a consistent signal for microRNA quantification by qPCR. Up to 130 EV-associated microRNAs (17.3%) were detected in CSF. A total of 26 microRNAs from the screening were selected for further analysis, including previously described microRNAs related to FTD proteins, such as miR-9, miR-34c, miR-107 and miR-124. Few candidate microRNAs appeared to be differently expressed in healthy controls and FTD patients.

**Summary/Conclusion**: The use of highly sensitive techniques allows the detection of EV-associated microRNAs in small volumes of biofluids. Differences in the microRNA profile between healthy controls and FTD patients show their potential as diagnostic biomarkers. Further studies are warranted to assess their possible role as biomarkers and to disentangle the mechanisms involved in the etiology of FTD.

**Funding**: This study was supported by grants from Instituto de Salud Carlos III (PI15/00026) to J Clarimon.

OS26.04

On-chip detection, sizing and proteomics of extracellular vesicles

Sameh Obeid^1^; Géraldine Lucchi^2^; Thierry Burnouf^3^; Wilfrid Boireau^4^; Celine Elie-caille ^4^

^1^French National Institute for Agricultural Research | INRA, Rennes, France; ^2^French National Institute for Agricultural Research | INRA, Dijon, France; ^3^College of Biomedical Engineering Taipei Medical University, Tapei, Taiwan (Republic of China); ^4^FEMTO-ST Institute, UBFC, Besancon, France

**Background**: Microparticles are small extracellular vesicles (EVs) (from ~100 to 1000 nm) produced by different cell types, through the budding of the plasma membrane, while exosomes (from ~30 to 120 nm) originate from the endolysosomal pathway before fusing with the plasma membrane to be released. Increased platelet-derived microparticles (PMPs) formation has been reported to contribute to the inflammatory role of blood components used for transfusion. When PMPs formation results from thrombin activation, they are able to aggregate monocyte cells *in vitro*. Nevertheless, the reason(s) for this EVs functionality/effect on target cells still need to be clarified, due to their high variety in size, protein composition and the potential concomitant presence of exosomes and small MPs in the analysed samples.

**Methods**: Our project consists in proposing a nano-bio-analytical (NBA) platform combining several biophysical techniques including surface plasmon resonance (SPR), mass spectrometry and atomic force microscopy (AFM) enabling EVs phenotyping, proteomic profiling and nanometrology.

**Results**: This NBA platform already gave a new introspection of PMP samples, showing that more than 95% of the vesicles were below 300 nm in diameter, over a wide concentration range (10^7^–10^12^ particles/ml), with thrombin-activated platelets-derived PMP CD41+ vesicles (TPMPs) slightly smaller than normal resting platelets-derived PMP CD41+ (NPMPs) ones. An on-chip nano-liquid chromatography-tandem mass spectrometry analysis revealed more than 200 proteins (from 500 ng of on-chip captured EVs) and a differential proteome between NPMPs and TPMPs, with at least 30 specific proteins for each PMPs sample. Moreover, a correlation has been demonstrated between the nature of identified proteins and the signalization pathways involved in neutrophil aggregation.

**Summary/Conclusion**: The NBA platform stands as a versatile and upgradable analytical solution for EVs analysis; thus, one of our developments focuses on a real introspection of EVs subsets that are probably co-captured by the same spot, thanks especially to the use of secondary antibodies, in order to achieve an, as high as possible, ultra-specific EVs subsets signature.

**Funding**: This work was funded by CNRS interdisciplinary call (Défi Instrumentations aux limites) and Franche-Comté region.

OS26.05

Circulating macrophage-derived extracellular vesicles predict post-operative myocardial infarction

Wade T. Rogers ^1^; Maggie Schmierer^1^; Scott Damrauer^2^; Emile Mohler^2^; Jonni Moore^2^

^1^CytoVas, LLC, Philadelphia, PA, USA; ^2^University of Pennsylvania, Philadelphia, PA, USA

**Background**: Post-operative cardiovascular complications cause significant morbidity and mortality. Identifying individuals at highest risk is a challenge. Previous work has demonstrated that circulating extracellular vesicles (EVs) associate with increased cardiovascular disease. 

**Methods**: We conducted a prospective multisite study of cardiovascular events in individuals undergoing major vascular surgery to test the hypothesis that cell- and vesicle-derived biomarkers predict post-operative cardiovascular outcomes. The primary endpoint was major adverse cardiovascular events and myocardial injury after non-cardiac surgery within 30 days. Panels enumerating cell subsets including progenitor cells, Th17 and Tang were developed. EV subsets were enumerated using antibodies to CD3, CD31, CD41a, CD105, CD64, CD144 and CD47. Assays were performed on a pair of modified FACSCanto Plus flow cytometers (BD Biosciences). Instrument modifications were aimed at improving detection sensitivity for small particles. Mie Theory calculations confirmed detection of EVs down to 106 nm in diameter, with a large majority smaller than 300 nm.

**Results**: No cellular subset significantly associated with post-operative events. Of the 128 EV subsets enumerated, only CD31+CD105+CD64+ macrophage-derived EVs (MEVs) associated with events after adjusting for multiple comparisons (Padj = 5.3 × 10^−3^). MEVs, controlled for history and demographics, resulted in a logistic regression model with area under the receiver operating characteristic (AUROC) curve of 0.921 (95% confidence level [0.860–0.975]; *P* = 1.6 × 10^–9^) and a diagnostic odds ratio of 32.8. An existing standard-of-care algorithm (RCRI) was less informative (AUROC = 0.774 [0.666, 0.868]).

**Summary/Conclusion**: MEVs are a novel biomarker for post-operative cardiovascular events. The association of these inflammatory vesicles with cardiovascular events provides new insights into heart disease and suggests a novel approach to preoperative risk assessment.

**Funding**: This work was funded by Becton Dickinson Biosciences.

OS26.06

Mass spectrometry analysis of urinary extracellular vesicles recovered in the low centrifugation pellet after elimination of uromodulin by tris(2-carboxyethyl) phosphine hydrochloride

Luca Musante ^1^; Sabrina La Salvia^2^; Uta Erdbrüegger^3^

^1^University of Virginia Health System, Department of Medicine, Division of Nephrology, Charlottesville, USA; ^2^University of Virginia, Charlottesville, USA; ^3^Department of Medicine/Nephrology Division, University of Virginia, Charlottesville, USA

**Background**: Urinary extracellular vesicles (uEVs) provide a source of valuable biomarkers for kidney and urogenital diseases. Analysis of uEVs in mass spectrometry (MS) is challenged by Tamm–Horsfall Protein (THP) or uromodulin. In this study, we used tris(2-carboxyethyl) phosphine hydrochloride (TCEP-HCl) as reducing reagent to deplete uEVs from THP before MS analysis. This is the first proteomic characterization of uEVS recovered in the second step of the differential centrifugation protocol.

**Methods**: Urine was centrifuged at relative centrifugation force of 4600*g* and EVs were collected by centrifugation at 20,000 *g*. EVs were resolubilized in buffer with and without protease inhibitors. Elimination of THP-HCl was performed by reduction with 10 mM TCEP-HCl followed by a second centrifugation step at 20,000 *g* per 30 min. The protein content of the depleted uEVs pellet-THP was established in MS. Gene ontology (GO) term annotations of the identified EV proteins were compared to the entries in the vesiclepedia repository. Several of the identified vesicles markers were further confirmed with Western blot analysis.

**Results**: Here we demonstrate that disruption of the 24 disulfide bonds by TCEP-HCl successfully released THP in the supernatant of the second centrifugation step. Liquid chromatography-tandem mass spectrometry of EVs-THP free identified 1053 proteins with at least two unique peptides and a 99% confidence level for protein identification. Out of these, 1018 proteins were present in the vesiclepedia repository. Using GO term annotation it was shown that the majority of these proteins were most significantly associated with “extracellular exosome”. Specific nephron markers like podocin, and collectrin along with vesicle markers like TSG101 and CD9 were confirmed by immunoblotting.

**Summary/Conclusion**: THP-HCl showed to be a powerful reducing agent which allowed the removal of the bulk of THP rapidly and independently from the amount of THP in the sample. Overall, these results show a very significant overrepresentation of protein-encoding genes for exosomes suggesting that the low centrifugation pellet includes all EV types. Inclusion of this pellet in the exosomes analysis should be considered and not discarded.

Symposium Session 27 – OMICS Applied to EVs Chairs: Alicia Llorente; Suresh Mathivanan Location: Room 6 15:45–17:15

OS27.01

A systems biology approach on pericardial fluid exosomes unravels relationships between miRs, proteins and metabolites that may play a potential role in promoting heart ischaemia in diabetic patients

Maryam Anwar^1^; Parul Dixit^1^; Jaimy Saif^2^; Sezin Aday^2^; Antonis Myridakis^1^; Andrea Martinez^1^; Marc Dumas^1^; Giovanni Biglino^2^; Enrico Petretto^3^; Costanza Emanueli ^4^

^1^Imperial College London, London, UK; ^2^University of Bristol, Bristol, UK; ^3^Imperial College London & Duke-NUS Medical School, Singapore, Singapore; ^4^Bristol Heart Institute, London, UK

**Background**: Exosomes are powerful vehicles for efficient cell-to-cell communication with significant relevance in cardiovascular homoeostatic and pathogenic processes. Working on clinical samples from non-diabetic cardiac surgery patients and in cell and mouse models, we recently showed that exosomes released by the myocardium accumulate into the human pericardial fluid (PF) and can exert important vascular actions. Type 2 diabetes mellitus (T2DM) induces microangiopathy and impairs endogenous reparative angiogenesis, thus contributing to ischaemic heart disease (IHD).

**Methods**: This project investigated novel mechanisms underpinning T2DM-associated IHD. To study this systematically, we collected PF samples from three groups of patients (IHD with/out T2DM and non-ischaemic, non-diabetic controls) and we produced and bioinformatically integrated various omics (high-throughput transcriptomic, proteomic and metabolomics) on the PF and the PF exosomes. Using R package Limma, metabolites and proteins that are differentially expressed (DE) under T2DM conditions were identified. Moreover, hierarchical clustering and gene set enrichment analysis identified groups of miRs that are DE under T2DM. Employing a network approach, we integrated miR, protein and metabolic data by using our newly developed R package Metabosignal, integrating interaction information from various databases.

**Results**: To understand relationships in the network, we derived shortest paths connecting PF and PF exosome DE proteins and metabolites and found an interaction circuit connecting insulin-like growth factor (IGF) protein to clusterin (complement system) and the metabolite mannosamine. Interestingly, IGF and mannosamine are highly expressed in the PF of diabetic patients whereas clusterin is poorly expressed. Experiments in HUVECs in our lab revealed that clusterin levels are indeed reduced under conditions of high glucose (diabetes) and hypoxia (mimicking ischaemia).

**Summary/Conclusion**: To conclude, this approach gives an initial insight into some of the relationships between metabolites and proteins from which plausible hypothesis can be generated to test in the lab.

**Funding**: This project is funded by BHF.

OS27.02

Quantitative proteomics of transforming growth factor beta receptor type 2-primed exosomes derived from DNA mismatch repair-deficient colorectal tumour cells

Fabia Fricke ^1^; Jürgen Kopitz^2^; Johannes Gebert^2^

^1^German Cancer Research Center (DKFZ), Clinical Cooperation Unit Applied Tumor Biology, Heidelberg, Germany; ^2^Applied Tumor Biology, University Hospital Heidelberg, Germany

**Background**: Microsatellite unstable (MSI) colorectal cancers (CRC) that lack DNA mismatch repair function show a high frequency of inactivating mutations in the tumour suppressor transforming growth factor beta receptor type 2 (TGFBR2) leading to abrogated downstream signalling and MSI tumour progression. Previously, we found that TGFBR2 can cause general changes in the protein content of MSI CRC-derived exosomes. Here, we analysed these proteomic alterations at the quantitative level using stable isotope labelling of amino acids in cell culture (SILAC)-based mass spectrometry and also assessed the TGFBR2-dependent exosomal phosphoproteome.

**Methods**: We used an MSI CRC model cell line (HCT116-TGFBR2) enabling doxycycline-inducible TGFBR2 expression and downstream signalling in an isogenic background. Exosomes were isolated by differential centrifugation and precipitation and characterized by electron microscopy, nanoparticle tracking, and Western blot analysis. Quantitative differences of the exosomal protein profile were identified by SILAC and subsequent mass spectrometry. Exosomal phosphopeptides were enriched by immobilized metal affinity chromatography.

**Results**: Proteomic profiling of exosomes revealed that TGFBR2 expression of MSI tumour cells modulates the protein cargo of secreted exosomes. Reconstituted expression/signalling of TGFBR2 revealed quantitative differences in exosomal protein subsets originating from TGFBR2-deficient or TGFBR2-proficient MSI tumour cells. In particular, we observed TGFBR2-dependent variations in phosphoserine, -threonine, and -tyrosine peptides indicating that the TGFBR2 expression status not only influences the selection of exosomal proteins but also influences the biological activity of these cargo proteins.

**Summary/Conclusion**: Our results highlight the pathological relevance of the MSI tumour driver TGFBR2 on the (phospho-) protein signature of MSI tumour-derived exosomes. This tumour driver-linked cargo profile enables exosome-mediated crosstalk between cancer cells and recipient cells with powerful effects on MSI tumour progression.

**Funding**: This work was supported by Intramural funding from the University Hospital Heidelberg to Dr Johannes Gebert and Prof. Jürgen Kopitz.

OS27.03

Proteomic signature of circulating extracellular vesicles in dilated cardiomyopathy

Ana Gamez-Valero ^1^; Santiago Roura^2^; Josep Lupón^3^; Carolina Gálvez-Montón^2^; Antoni Bayes-Genis^3^; Francesc E. Borràs^4^

^1^HUGTiP and IGTP Institute with the Universitat Autónoma de Barcelona, BADALONA, Spain; ^2^ICREC Research Programme, IGTP, Badalona, Spain; ^3^Cardiology Service, HUGTiP, Badalona, Spain; ^4^REMAR-IVECAT Group, “Germans Trias i Pujol” Health Science Research Institute, Can Ruti Campus, Badalona, Spain

**Background**: Dilated cardiomyopathy (DCM) remains a major cause of heart failure. Better disease characterization using novel molecular techniques is needed to refine disease progression. This study explored the proteomic signature of plasma-derived extracellular vesicles (EVs) obtained from DCM patients and healthy controls using size-exclusion chromatography (SEC).

**Methods**: Purified EV fractions were analysed by liquid chromatography-mass spectrometry (LC-MS/MS). Raw data obtained from LC-MS/MS were analysed against the Uniprot human database using MaxQuant software. Additional analyses using Perseus software were based on the Intensity-Based Absolute Quantification values from MaxQuant analyses.

**Results**: A total of 90.07 ± 21 proteins were identified (183 different proteins) in the DCM group and 96.52 ± 17.91 proteins (227 different proteins) in the control group. A total of 176 proteins (74.6%) were shared by controls and DCM patients, whereas 51 proteins were exclusive for the DCM group and 7 proteins were exclusive for the control group. Fibrinogen, serotransferrin, alpha-1-antitrypsin and a variety of apolipoprotein family members were clustered in SEC-EVs derived from DCM patients relative to controls (*p* < 0.05). Regarding gene ontology analysis, response to stress and protein activation-related proteins was enriched in DCM-EVs compared to controls.

**Summary/Conclusion**: We here report the distinct proteomic signature of circulating EVs from DCM patients compared to those from healthy subjects. We also reveal that SEC obtains highly purified EV fractions from peripheral blood samples for subsequent use in determining disease-specific proteomic signatures.

**Funding**: This work was supported by grants from the Ministerio de Economía, Industria y Competitividad (SAF2014-59892-R), Fundació La MARATÓ de TV3 (201405/10, 201502, 201516), Societat Catalana de Cardiologia, Generalitat de Catalunya (SGR 2014, CERCA Programme), and the Fundació Bancària La Caixa. This work was also funded by the Red de Terapia Celular – TerCel (RD16/0011/0006), CIBER Cardiovascular (CB16/11/00403), and Fondo de Investigación Sanitaria, Instituto de Salud Carlos III (FIS PI14/01682) as part of the Plan Nacional de I+D+I cofounded by ISCIII-Sudirección General de Evaluación y el Fondo Europeo de Desarrollo Regional (FEDER).

OS27.04

Tracking and capturing of bioorthogonal labelled RNA carried by extracellular vesicles during maternal–embryo communication

Masoumeh Es-haghi ^1^; Annika Häling^2^; Freddy Lattekivi^3^; Stoyan Tankov^3^; Victoria James^4^; Tamer Nafee^5^; Sulev Koks^3^; Alireza Fazeli^3^

^1^Institute of Biomedicine and Translational Medicine Department of Pathophysiology, Tartu, Estonia; ^2^Institute of Biomedicine and Translational Medicine, Tartu, Estonia; ^3^Institute of Biomedicine and Translational Medicine, Department of Pathophysiology, University of Tartu, Tartu, Estonia; ^4^School of Veterinary Medicine and Science, University of Nottingham, Nottingham, UK; ^5^Academic Unit of Reproductive and Developmental Medicine, Department of Oncology and Metabolism, The Medical School, Sheffield, UK

**Background**: During implantation window, the uterine epithelium acquires a receptive phenotype and is being prepared for the initial blastocyst attachment. This unique phenomenon may stem from embryonic–maternal crosstalk utilizing an intricate language. Extracellular vesicles (EV) could be a logical mean for maternal–embryo communication. The current investigation was aimed at deciphering the main signals exchanged between the mother and the baby.

**Methods**: The 5-ethynyl uridine (EU)-labelled trophoblast spheroids were cultivated with an endometrial cell line in a non-contact co-culture system. The trophoblast EU-labelled RNA was tracked and captured in endometrial cells. The transferred labelled RNA was affinity-precipitated and purified using biotin-azide click chemistry. Total RNA-sequencing was conducted with synthesized cDNA from captured labelled and non-EU labelled RNA (background) (*n* = 4). Differential expression analysis of RNA-seq data was performed using edgeR and limma packages to identify the transferred transcripts using differential enrichment as a proxy. The Integrative Genomics Viewer was used to validate the coverage of differentially enriched transcripts. The results were confirmed by quantitative PCR (qPCR). To establish the route of candidate RNA transfer, EVs were isolated from co-culture media using size-exclusion chromatography. Total RNA was extracted from EVs, EU-labelled RNA was affinity-precipitated and the absolute copy number of putatively transferred RNA sequences was quantified.

**Results**: Differential enrichment analysis demonstrated that the majority of putatively transferred transcripts were non-coding RNAs derived from the mir99alet7c cluster (Chromosome 21: LINC00478). The presence of non-coding sequences from this chromosomal region in the RNA extracted from EVs was confirmed by qPCR. This suggests that these sequences are carried by throphoblast EVs.

**Summary/Conclusion**: In this study, we showed that bioorthogonal RNA labelling chemistry can be used for the deciphering trophoblast–endometrial communications. These are the initial steps towards decoding the earliest stages of the mother–offspring language/crosstalk.

OS27.05

*M. tuberculosis* immunomodulatory lipids, beyond the wall. Global lipidomic analysis of vesicles released by the bacillus and by infected host cells

Emilie Layre; Jérôme Nigou

IPBS-CNRS, Toulouse, France

**Background**: *M. tuberculosis* (Mtb) produces a wide diversity of lipids that modulate host immune responses as pathogen-associated molecular patterns, T-cell antigens or virulence factors. This unique repertoire has been essentially deciphered by characterizing the structure and properties of the lipids that constitute the bacillus envelope. However, yet uncharacterized mycobacterial lipids are released from the envelope within vesicles produced by the bacillus itself and within exosome-like vesicles released by host cells during infection. Although the production of vesicles might be a key path by which bacterial lipids interfere with immune effectors beyond the site of infection, the content of these vesicles in immunomodulatory mycobacterial lipids remains poorly characterized. Whether vesicles shuttle specific lipid families including uncharacterized ones, if their composition depends on mycobacterial strains virulence or if they differentially regulate immune responses, remains an open question. In this context, we have undertaken to characterize the nature and properties of mycobacterial lipids shuttled within mycobacterial and host vesicles.

**Methods**: Using virulent and attenuated strains, we performed the global analysis of the lipid content of bacterial and host exosome-like vesicles, thanks to a sensitive Mtb-dedicated high-performance liquid chromatography-mass spectrometry approach allowing the targeted screening of known mycobacterial lipids as well as unbiased identification of new molecules. In addition, using reporter cell lines we have analysed the capacity of these vesicles to activate pathogen recognition receptors (PRR) known to recognize Mtb lipids, such as TLR2 and C-type lectins.

**Results**: Focusing on known lipid families, we highlight that many of the major immunomodulatory mycobacterial lipids (including strain-specific lipids) are present within vesicles but nevertheless show a selective distribution compared to their relative abundance in the bacillus envelope. These differences in mycobacterial lipid profiles are accompanied by a differential activation of tested PRR.

**Summary/Conclusion**: Our study provides important insights into the biological function of mycobacterial lipids, through their trafficking within extracellular vesicles, in host–pathogen interactions of the tuberculosis infection.

**Funding**: This work was funded by CNRS, Fondation pour la Recherche Médicale.

OS27.06

ExRNA Atlas analysis provides an exRNA census and reveals six types of vesicular and non-vesicular exRNA carrier profiles detectable across human body fluids

Oscar D. Murillo ^1^; William Thistlethwaite^1^; Rocco Lucero^1^; Sai Lakshmi Subramanian^1^; Neethu Shah^1^; Andrew R. Jackson^1^; Joel Rozowsky^2^; Robert R. Kitchen^3^; James Diao^4^; Timur Galeev^4^; Jonathan Warrell^4^; Kristina Hanspers^5^; Anders Riutta^5^; Alexander Pico^5^; Roger P. Alexander^6^; David Galas^6^; Andrew I. Su^7^; Louise C. Laurent^8^; Kendall Jensen^9^; Matthew Roth^1^; Mark B. Gerstein^10^; Aleksandar Milosavljevic^1^

^1^Department of Molecular & Human Genetics, Baylor College of Medicine, Houston, USA; ^2^Yale University, New Haven, USA; ^3^Exosome Diagnostics, Boston, USA; ^4^Department of Molecular Biophysics & Biochemistry, Yale University, New Haven, USA; ^5^Gladstone Institutes, San Francisco, USA; ^6^Pacific Northwest Research Institute, Seattle, USA; ^7^Department of Integrative, Structural and Computational Biology, The Scripps Research Institute, La Jolla, USA; ^8^University of California, San Diego, San Diego, USA; ^9^Neurogenomics, Translational Genomics Research Institute, Phoenix, USA; ^10^Department of Molecular Biophysics & Biochemistry, Yale University, New Haven, USA

**Background**: To gain insights into exRNA communication, the NIH Extracellular RNA Communication Consortium created the Extracellular RNA Atlas including 5309 exRNA-seq and qPCR profiles, most obtained from five body fluids (cerebrospinal fluid, saliva, serum, plasma, urine).

**Methods**: Extensive metadata, uniform processing and standardized data quality assessments facilitated integrative analysis of miRNA, tRNA, Y RNA, piRNA, snRNA, snoRNA and lincRNA abundance across 21 data sets represented in the Atlas. A computational deconvolution method was applied to infer ncRNA profiles of specific exRNA carriers (vesicular or not) and to estimate relative amounts of exRNA contributed to each Atlas sample by the carriers.

**Results**: We obtain a census of ncRNAs that includes, among others, 96 miRNAs abundantly detected (>10 RPM) in CSF, saliva, serum, and plasma, of those, 46 are detected in all five fluids, including urine. Deconvolution of ncRNA profiles reveals six major carrier types and a striking amount of their sample-to-sample abundance variability. In contrast, highly concordant exRNA profiles of all six carrier types can be detected across different studies and biofluids. Three (LD and HD exosomes and HDL particles) of the six were previously purified and profiled. We define three new carrier profiles, ABF, CP and XSA, that are yet to be profiled in isolation and carry miRNAs in higher abundance than the LD, HD and HDL. All six carrier profiles are detected across body fluids, with ABF and HD exosome profiles detected in all five body fluids; XSA and LD exosome profiles in all except saliva; CP in CSF and plasma; and HDL particle profiles in plasma and saliva. We demonstrate the potential of this knowledge and methodology to improve interpretation of individual case–control studies by reducing variance due to sample-to-sample variation in carrier abundance and by assigning differential (cases vs. controls) abundance of specific small ncRNAs to specific carrier types.

**Summary/Conclusion**: ExRNA Atlas analysis yields global insights into vesicular and non-vesicular exRNA communication by combining and deconvoluting data across multiple studies.

**Funding**: This work was funded by National Institutes of Health, National Institute on Drug Abuse (U54 DA036134).

Meet the Expert Session: Biomarkers on EVsLocation: AuditoriumSession Chair: Andrew Hill 18:30–20:00

Meet the Expert Session: EVs in Neglected Tropical DiseasesSession Chairs: Igor C. Almeida; Carmen Fernandez-BecerraLocation: Room 518:30–20:00

Meet the Expert Session: Can Research on EVs AccelerateSession Chairs: Evaristo Feliu Frasnedo; Theresa Whiteside Clinical Impact in Leukemia? (Supported by the Fundacio Josep Carreras)Location: Room 618:30–20:00

Poster Session PS01: EVs in Tissue Injury and Repair Chairs: Elizebet Ligeti; Magdalena LorenowiczLocation: Exhibit Hall 17:15–18:30

PS01.01

Validation of engineered cardiac grafts for the local delivery of multifunctional extracellular vesicles for myocardial repair

Marta Monguió-Tortajada ^1^; Cristina Prat-Vidal^2^; Isaac Perea-Gil^2^; Carolina Gálvez-Montón^2^; Santiago Roura^2^; Antoni Bayes-Genis^3^; Francesc E. Borràs^4^

^1^REMAR-IVECAT Group, IGTP, Badalona, Spain; ^2^ICREC research program, IGTP, Badalona, Spain; ^3^Cardiology Service, HUGTiP, Badalona, Spain; ^4^REMAR-IVECAT Group, “Germans Trias i Pujol” Health Science Research Institute, Can Ruti Campus, Badalona, Spain

**Background**: The administration of extracellular vesicles (EVs) derived from mesenchymal stem cells (MSCs) is a promising alternative treatment for several pathologies, including cardiac repair after myocardial infarction (MI). MSC-EVs have immunomodulatory, regenerative and pro-angiogenic capabilities both autologous and allogeneically. However, the optimal delivery strategy for EV therapy remains a challenge. Thus, the purpose was to validate novel bioengineered 3D scaffolds as an efficient support for the local delivery of bioactive, multifunctional EVs.

**Methods**: We purified EVs from porcine cardiac adipose tissue MSCs by size-exclusion chromatography and characterized them morphologically and phenotypically. We then developed two decellularized cardiac scaffolds from myocardial and pericardial tissues and embedded them with fluorescently labelled MSC-EVs for tracking and retention assessment.

**Results**: The regenerative, alloreactivity and immunomodulatory properties of porcine MSC-EVs were assessed *in vitro* to validate their potential for myocardial repair. The structure of the two acellular scaffolds was preserved upon the decellularization process and their proteome characterization showed enrichment of matrisome proteins and major cardiac extracellular matrix components. Both engineered cardiac scaffolds retained MSC-EVs even after thorough washing and a week-long culture, as shown by whole-tissue fluorometric scanning, confocal and scanning electron microscopy imaging.

**Summary/Conclusion**: Collectively, our data indicate that both engineered cardiac scaffolds may be suited for effective EV local administration and will be further evaluated in preclinical MI swine models on restoring cardiac function post-MI. The confined administration of multifunctional EVs within a scaffold may potentiate cardiac repair by increasing the local dose of MSC-EVs, constitute a bioactive niche for regeneration and could be used as a cell-free, off-the-shelf product to regenerate post-infarcted myocardium.

**Funding**: This work was funded by Fundació La Marató TV3 (201516), Societat Catalana de Cardiologia, PERIS (SLT002/16/00234), and Generalitat de Catalunya (2014SGR804 and 2014SGR699).

PS01.02

Extracellular vesicles-mediated epithelial cell senescence by fibroblasts in IPF pathogenesis

Tsukasa Kadota ^1^; Yusuke Yoshioka^1^; Yu Fujita^2^; Jun Araya^2^; Kazuyoshi Kuwano^2^; Takahiro Ochiya^1^

^1^Division of Molecular and Cellular Medicine, National Cancer Center Research Institute, Chuo-ku, Japan; ^2^Division of Respiratory Diseases, Department of Internal Medicine, The Jikei University School of Medicine, Minato-ku, Japan

**Background**: Idiopathic pulmonary fibrosis (IPF) is a progressive and lethal fibrosing interstitial lung disease of unknown etiology. Aberrant phenotypic alterations of alveolar epithelial cell, including accelerated cellular senescence, have been proposed to be responsible for regulating fibrosis development. However, the detailed mechanisms for modulating cellular senescence are poorly understood. Here, we investigated the involvement of extracellular vesicles (EVs)-mediated intercellular communication between lung fibroblasts (LFs) and primary human bronchial epithelial cells (HBECs) in regulating epithelial cell senescence during IPF pathogenesis.

**Methods**: LFs were obtained from IPF and non-IPF patients who underwent lobectomy. EVs from LFs were isolated by ultracentrifugation. The profiles of EV-associated microRNAs (miRNAs) were examined by microarray and real-time PCR. Cellular senescence was evaluated with senescence-associated β-galactosidase staining and expression levels of p21Waf1/Cip1 and p16INK4A. The immunohistochemistry was used to detect expression in IPF and non-IPF lungs.

**Results**: LF-derived EVs were characterized by the presence of EV marker proteins and electron microscope. Confocal microscopic examination elucidated uptake of labelled EVs derived from LFs by HBECs. Intriguingly, EVs derived from IPF-LFs promoted cellular senescence in HBECs. EV microarray analysis elucidated that several miRNAs were upregulated in EVs derived from IPF-LFs, which regulate cellular senescence. Importantly, expression of the target gene of the miRNAs was downregulated in lungs of IPF patients and silencing the gene in HBEC led to cellular senescence. Moreover, fibroblast-derived extracellular vesicle-mediated cellular senescence in HBEC was abrogated by inhibiting the miRNAs.

**Summary/Conclusion**: EV-associated miRNAs from IPF-LF accelerate epithelial cell senescence as a part of aberrant mesenchymal–epithelial interactions in IPF pathogenesis.

PS01.03

Mesenchymal stromal cell-derived extracellular vesicles (EVs) as mediators of anti-inflammatory effects: endorsement of macrophage polarization

Claudia Lo Sicco^1^; Daniele Reverberi^2^; Chiara Franzin^3^; Michela Pozzobon^3^; Luisa Pascucci^4^; Ranieri Cancedda^1^; Roberta Tasso ^2^

^1^University of Genova, Genova, Italy; ^2^Ospedale Policlinico San Martino-IRCCS per l’Oncologia, Genova, Italy; ^3^Stem Cells and Regenerative Medicine Lab, Fondazione Istituto di Ricerca Pediatrica Città della Speranza, Padova, Italy; ^4^University of Perugia,Veterinary Medicine Department, Perugia, Italy

**Background**: Mesenchymal stromal cells (MSCs) are effective therapeutic agents enhancing the repair of injured tissues. Preliminary results indicate that in an inflammatory environment as the one generated during the early phases of the wound healing process, MSC paracrine activity is significantly modulated promoting a functional switch of macrophages from a pro- (M1) to an anti-inflammatory (M2) state. Extracellular vesicles (EVs) are players in cell-to-cell communication by serving as vehicles for transfer between cells of membrane and cytosolic proteins, lipids and genetic information. The aim of the present study was to carry out a detailed characterization of EVs released by human adipose derived-MSCs to investigate their involvement as modulators of MSC anti-inflammatory effects inducing macrophage polarization.

**Methods**: The EV-isolation method was based on repeated ultracentrifugations of the medium conditioned by MSC exposed to either normoxic or hypoxic conditions (EV-Normo and EV-Hypo). Both types of EVs were efficiently internalized by responding bone marrow-derived macrophages, eliciting their switch from an M1 to an M2 phenotype. Observations that different macrophage subsets are associated with different stages of muscle regeneration led us to investigate whether EV treatment could influence macrophage polarization from M1 to M2 phenotype *in vivo*. We opted for a cardiotoxin (CTX) injury in the mouse tibialis anterior (TA) muscle. Muscles subjected to CTX-damage followed by injection of either EV-Normo or EV-Hypo were examined at different times.

**Results**: EV-Normo and EV-Hypo interacted with macrophages recruited during the initial inflammatory response. In injured and EV-treated muscles, a down-regulation of IL6 and the early marker of innate and classical activation Nos2 was concurrent to a significant up-regulation of Arg1 and Ym1, late markers of alternative activation. These effects, accompanied by an accelerated expression of the myogenic markers Pax7, MyoD and eMyhc, were even greater following EV-Hypo administration.

**Summary/Conclusion**: These data indicate that MSC-EVs possess effective anti-inflammatory properties, making them potential therapeutic agents more handy and safe than MSCs.

**Funding**: This work was supported by the Italian Ministry of Health ("Young Investigator Grant” – GR-2013-02357519).

PS01.04

Mesenchymal stromal/stem cell-derived extracellular vesicles promote human cartilage regeneration

Lucienne Vonk^1^; Sanne van Dooremalen^2^; Nalan Liv^3^; Judith Klumperman^3^; Paul Coffer^2^; Daniël Saris^1^; Magdalena Lorenowicz ^2^

^1^Department of Orthopedics, University Medical Center Utrecht, Utrecht University, Utrecht, The Netherlands; ^2^Center for Molecular Medicine& Regenerative Medicine Center University Medical Center Utrecht, Utrecht University, Utrecht, The Netherlands; ^3^Center for Molecular Medicine, University Medical Center Utrecht, Utrecht University, Utrecht, The Netherlands

**Background**: Osteoarthritis (OA) is a rheumatic disease leading to chronic pain and disability with no effective treatment available. Recently, allogeneic human mesenchymal stromal/stem cells (MSC) entered clinical trials as a novel therapy for OA. Increasing evidence suggests that therapeutic efficacy of MSC depends on paracrine signalling. Here we investigated the role of bone marrow MSC-derived extracellular vesicles (BMMSC-EVs) in cartilage repair.

**Methods**: To test the effect of BMMSC-EVs on OA cartilage inflammation, the tumour necrosis factor alpha (TNF-alpha)-stimulated OA chondrocyte monolayer cultures were treated with BMMSC-EVs and inflamatory gene expression was measured by qRT-PCR after 48 h. To access the impact of BMMSC-EVs on cartilage regeneration, the BMMSC-EVs were added to the regeneration cultures of human OA chondrocytes, which were analysed after 4 weeks for glycosaminoglycan content by DMMB and qRT-PCR. Furthermore, paraffin sections of the regenerated tissue were stained for proteoglycans (safranin-O) and type II collagen (immunostaining).

**Results**: We show that BMMSC-EVs promote cartilage regeneration *in vitro*. Treatment of OA chondrocytes with BMMSC-EVs induces production of proteoglycans and type II collagen and promotes proliferation of these cells. MSC-EVs also inhibit the adverse effects of inflammatory mediators on cartilage homoeostasis. Our data show that BMMSC-EVs downregulate TNF-alpha-induced expression of pro-inflammatory cyclooxygenase-2, pro-inflammatory interleukins and collagenase activity in OA chondrocytes. The anti-inflammatory effect of BMMSC-EVs involves the inhibition of NF-κB signalling, activation of which is an important component of OA pathology. Thus, our findings indicate that BMMSC-EVs have the ability to promote human OA cartilage repair by reducing the inflammatory response and stimulation of OA chondrocytes to produce extracellular matrix, the essential processes for restoring and maintaining cartilage homoeostasis.

**Summary/Conclusion**: Taken together, our data demonstrate that MSC-EVs can be important mediators of cartilage repair and hold great promise as a novel therapeutic for cartilage regeneration and osteoarthritis.

**Funding**: This work was funded by Dutch Arthritis Foundation, WKZ Foundation, ZonMW.

PS01.05

Excretion of urinary extracellular vesicles does not differ between apparently healthy postmenopausal women without and with histories of pre-eclampsia

Muthuvel Jayachandran; John Lieske; Vesna Garovic

Mayo Clinic Rochester, Rochester, USA

**Background**: Several studies confirmed the association of previous pre-eclampsia (PE) and cardiovascular disease. However, little is known about the relationship between PE and future kidney health and disease. Our previous studies confirm that populations of urinary extracellular vesicles (EVs) can reflect kidney health and disease above and beyond traditional biomarkers. Here we examined whether populations of renally derived urinary EVs differ in postmenopausal women without and with a history of PE.

**Methods**: This study was approved by the Mayo Clinic Institutional Review Board. Bio-banked cell-free random urine from postmenopausal age- and parity-matched apparently healthy (no prior disease and events) women with (*n* = 40) and without (*n* = 40) a history of PE was studied. Urinary EVs >0.2 µm were analysed by digital flow cytometry using fluorophore conjugated antibodies. Raw EV counts (EV/µL urine) were normalized to urinary creatinine (EV/mg creatinine). Ratios of EV/CD63 (exosome) or EV/annexin-V (microvesicle) were also calculated.

**Results**: Median age (60 years), serum creatinine, estimated glomerular filtration rate, urinary protein, albumin and creatinine excretion were similar between women with and without prior PE. The total number of urinary EVs positive for annexin-V, CD63, inflammatory markers (ICAM-1, VCAM-1, tissue factor and MCP-1), angiotensin receptor 1 and 2 and renal cell injury markers (beta-2 microglobulin, cystatin C, clusterin, kidney injury molecule-1, laminin alpha-5 and neutrophil gelatinase-associated lipocalin (NGAL)) also did not differ between groups. Similarly, the number of urinary EVs derived from glomerular cells (juxtaglomerular cells, mesangial cells, podocytes, and parietal cells), specific nephron segments and stem/progenitor cells also did not differ based upon prior history of PE.

**Summary/Conclusion**: This study suggests that long-term renal health of postmenopausal women is not affected by a history of PE in younger life.

**Funding**: This work was funded by NIH AG44170; U54DK083908; Mayo Clinic O’Brien Urology Research Center (U54 DK100227); R25-DK101405.

PS01.06

Harnessing the human mesenchymal stem cells (hMSCs) secretome to couple the RV/PA during pulmonary fibrosis (PF)

Luis A. Ortiz ^1^; Joel Njah^2^; Jadranka Milosevic^2^; Ariana Detwiler^2^; Lai Ruen^3^; Andre Choo^3^; Sai Lim^4^

^1^Division of Environemntal and Occupational Health University of Pittsburgh, Pittsburgh, USA; ^2^University of Pittsburgh, Pittsburgh, USA; ^3^SOCRATES, Singapore, Singapore; ^4^SOCRATES, Singapore, Singapore

**Background**: In a large cohort of patients undergoing treatment for pulmonary fibrosis (PF) at the University of Pittsburgh, we demonstrated that right ventricular (RV) failure is the proximate cause of death. Subsequently, we developed an experimental model in which bleomycin induces lung fibrosis, pulmonary arterial hypertension (PAH), and RV dysfunction in C57BL/6 mice. Human mesenchymal stem cells (hMSCs) retained in the lung release exosomes and ameliorate the myocardial ischaemia following coronary ligation. Here we hypothesize that hMSCs or their secretory activity (exosomes) can be used to protect the RV/pulmonary arterial (PA) coupling during PAH in bleomycin-exposed mice.

**Methods**: We intravenously administer hMSCs (500,000 cells), exosomes (20 µg/mouse) 30 and 35 days after the recurrent (12 doses) instillation of bleomycin (20 mg/kg) into C57BL/6 mice. Subsequently, we performed haemodynamic evaluations (in spontaneously breathing mice) to assess the effect of these interventions on the RV function and PA pressure of bleomycin-treated mice. We also evaluated the effects of MSCs and exosomes, on the RV adenosine triphosphate (ATP) generation, and ROS production.

**Results**: Compared to control, bleomycin treatment induced significant increases in RV systolic pressure (RVSP) (20 ± 3 vs. 32 ± 1 mmHg) and RV diastolic pressure (RVDP) (3 ± 1 vs. 8 ± 1 mmHg), and depressed RV ejection fraction (EF) (60% vs. 30%) 60 days after bleomycin injection. These changes were significantly (*p* < 0.05) attenuated by the intravenous administration of hMSCs (RVSP 20 ± 3, RVDP 2 ± 1?mmHg, EF 60%) or their exosomes (RVSP 20 ± 3, RVDP 5 ± 1 mmHg, EF 60%). Bleomycin effects on RV were associated with significant (*p* < 0.05) increases in mitochondria RV H_2_O_2_ generation (1 ± 1 vs. 5.5 ± 1 mmol/min/mg) and reduction in ATP production (20 ± 3 controls vs. 5 and 10 ± 4 pmol/min/mg) after bleomycin administration. These changes were significantly (*p* < 0.05) modulated by administration of hMSCs (RV H_2_O_2_ generation 3 ± 1 and 3 ± 1?mmol/min/mg, ATP production 8 ± 3 and 12 ± 4 pmol/min/mg) or exosomes (RV H_2_O_2_ generation 2 ± 0.5 and 2 ± 1 mmol/min/mg, ATP production 11 ± 3 and 12 ± 1 pmol/min/mg).

**Summary/Conclusion**: Similar to humans with severe IPF, bleomycin exposure induces significant RV dysfunction in C57BL/6 mice. This RV dysfunction is associated with significant mortality, increase in RV mitochondrial ROS and reduced ATP generation. These haemodynamic and metabolic responses in the RV of bleomycin-treated mice are ameliorated by the IV administration of hMSCs or exosomes.

**Funding**: This work was supported by NIH grants R01HL110344 and R01HL114795.

PS01.07

Exosomes derived from mesenchymal stem cells as a possible therapy for osteoarthritis

Maria Elisabetta Federica Palamà ^1^; Simonetta Carluccio^1^; Daniele Reverberi^2^; Georgina Shaw^3^; Frank Barry^3^; Mary Murphy^3^; Chiara Gentili^1^

^1^University of Genoa, Genova, Italy; ^2^Ospedale Policlinico San Martino-IRCCS per l’Oncologia, Genova, Italy; ^3^NUIG Galway, Galway, Ireland

**Background**: Osteoarthritis is a pathological condition that affects a large part of the elderly population in the world. The main cause seems to be the establishment of an inflammatory process that brings to the degradation of the articular cartilage, degeneration of ligaments and thickening of the subchondral bone. In recent years, human mesenchymal stem cells (hMSCs) are emerging as promising cell therapy candidate for the treatment of this clinical condition. Many studies demonstrate that MSCs attend to tissue repair through secretion of trophic factors or extracellular vesicles. We developed a “donor-to-patient” closed, scalable and automated system for aseptic therapeutic cell manufacturing using a xeno-free medium. We validated the potential therapeutics benefits of secreted factors, conditioned medium and exosomes isolated from MSC culture in this innovative culture system, for cartilage and bone repair.

**Methods**: We isolated hMSCs from iliac crest marrow aspirates of healthy donors and human articular chondrocytes (HACs) from cartilage biopsies, after informed consent. MSCs-derived exosomes or secretome were given to HAC cultured under both physiological and inflammatory conditions, to evaluate their role in cartilage homoeostasis maintenance.

**Results**: In a damaged tissue, the initial inflammatory response plays a key role triggering tissue repair and homoeostasis, but can be detrimental in the long term, causing fibrosis. We observed that under inflammatory condition, HAC are able to internalize and recruit more MSC-derived exosomes, compare the control chondrocytes. We will focus on the characterization of MSC-conditioned media and exosomes and we will investigate their effects in maintenance of cartilage commitment and in the activation of different regeneration pathways (IL6, IL8, COX2 and PGE-2). The effect of MSCs-derived exosomes could be protective for the articular cartilage and we will evaluate *in vitro* and *in vivo* if they may be a possible therapy for osteoarthritis.

**Summary/Conclusion**: Our study suggests that MSC exosome may exert protective effects in degenerative joint conditions and provide support for further studies of this innovative approach in joint disease.

PS01.09

Endothelial colony-forming cell-derived exosomes attenuate pulmonary hypertension and hypoplasia in neonatal rats

Flore Lesage ^1^; Joanne Joseph^1^; Rajesh A Alphonse^2^; Arul Vadivel^1^; Chanèle Cyr-Depauw^1^; Shumei Zhong^1^; Dylan Burger^3^; Mervin C Yoder^4^; Bernard Thébaud^1^

^1^Sinclair Centre for Regenerative Medicine, Ottawa Hospital Research Institute, Ottawa, Ottawa, Canada; ^2^Faculty of Medicine and Dentistry, University of Alberta, Edmonton, AB, Edmonton, Canada; ^3^Kidney Research Centre, Ottawa Hospital Research Institute, University of Ottawa, Ottawa, Canada; ^4^Wells Center for Pediatric Research, Indiana University, Indianapolis, IN, US, Indianapolis, USA

**Background**: Pulmonary hypertension (PH) complicates the course of more than 10% of neonates with respiratory failure. In these patients, PH interferes with the postnatal vascular and alveolar lung development, which is critical to establish a functional gas-exchanging unit. To date, no effective therapy for neonatal PH is available, leading to lifelong morbidities. Evidence suggests that angiogenic growth factors drive lung development. Endothelial colony-forming cells (ECFCs) represent a subset of vascular progenitors capable of self-renewal and *de novo* vessel formation. We hypothesized that exogenous supplementation of ECFCs will restore the disrupted lung vascular growth in PH lungs and that this effect is mediated via exosomal signalling.

**Methods**: Rats were injected subcutaneously with the pulmonary endothelial toxin monocrotaline (MCT) at postnatal day (PN) 6. Human umbilical cord blood (UCB)-derived ECFCs or their exosomes were injected intravenously at PN7 (prophylaxis) or PN14 (rescue). Rats were analysed at PN28 for lung function (Flexivent), vascular function (Doppler ultrasound and right heart hypertrophy), and alveolar and vascular structure (histology).

**Results**: Injection of neonatal rats with MCT at PN6 resulted in disrupted alveolar and lung vascular growth, and PH. This was associated with an impaired function of lung-resident ECFCs. Therapy with UCB-derived ECFCs or their exosomes resulted in a significant improvement of pulmonary and vascular function and structure, and attenuated PH.

**Summary/Conclusion**: The impaired function of lung-resident ECFCs in growing rats resulting from MCT injection contributes to PH and arrested alveolar development. Exogenous ECFCs or their exosomes may offer new treatment strategies for patients suffering from PH – both neonates and adults. The use of ECFC-derived exosomes is particularly exciting as they may not generate an immune response, enabling allogenic administration and thus the production of an off-the-shelf therapy.

**Funding**: This work was funded by Heart and Stroke Foundation Canada and Ontario Institute for Regenerative Medicine.

PS01.10

Human liver stem cell-derived EVs abrogate fibrotic markers in TGF-β1-activated fibroblasts

Sharad Kholia ^1^; Maria Beatriz Herrera Sanchez^2^; Federica Collino^3^; Giovanni Camussi^4^

^1^University of Torino, Torino, Italy; ^2^2i3T, Torino, Italy; ^3^Federal University of Rio de Janeiro, Rio de Janeiro, Brazil; ^4^Department of Medical Sciences University of Turin, Turin, Italy

**Background**: Kidney fibrosis is the progressive pathological accumulation of extracellular matrix on the kidney parenchyma initiated during injury. It is a harmful process mainly mediated by the profibrotic cytokine TGFβ1, inevitably leading to the loss of renal function. Recently, stem cell derived extracellular vesicles (EVs) have been shown to exhibit regenerative properties. For instance, mesenchymal stem cell EVs have been shown to aid in cardiac repair and human liver stem cell EVs (HLSC EVs) in the recovery of acute kidney injury. Here, we investigated whether HLSC EVs had any effect on TGFβ1-mediated activation of fibroblasts.

**Methods**: Mouse kidney fibroblasts were treated with TGF-β1 cytokine in the presence or absence of various concentrations of HLSC EVs for 4 days. Post incubation, the cells were subjected to quantitative real-time PCR or immunofluorescence microscopy to analyse the expression levels of the fibroblast activation markers: αSMA, collagen 1a1 and TGF-β both at a molecular and protein level.

**Results**: On treating fibroblast with TGF-β1, there was a significant upregulation of the fibroblast activation markers. However, on treating the cells with various concentrations of HLSC EVs, the activation markers were significantly downregulated both at a molecular and protein level. For instance, all doses of EVs significantly prevented the upregulation of αSMA; however, only the higher dose significantly prevented the upregulation of TGFβ and collagen 1a1.

**Summary/Conclusion**: On the basis of this data, we conclude that the profibrotic cytokine TGFβ1 induces the activation of fibroblasts through the upregulation of profibrotic markers. This activation is abrogated on treating with HLSC EVs. Hence, as one of the key factors of fibrosis is TGF-β1-mediated activation of fibroblasts and as HLSC EVs downregulated this activation in our model, we speculate that HLSC EVs may act as potential therapeutic agents in the treatment and prevention of kidney fibrosis.

**Funding**: This work was funded by Marie Curie Industry-Academia Partnerships and Pathways (IAPP) FP7-PEOPLE-2013 grant: EVStemInjury Project 612224: Extracellular Vesicles and exosomes from adult stem cells in the regeneration of organ injury.

PS01.11

Intratracheal mesenchymal stem/stromal cells (MSCs)-derived extracellular vesicles (EVs) significantly improve morphological and biochemical parameters in an animal model of bronchopulmonary dysplasia

Patrizia Zaramella^1^; Andrea Porzionato^1^; Arben Dedja^1^; Chiara Franzin^2^; Diego Guidolin^1^; Veronica Macchi^1^; Raffaele De Caro^1^; Marcin Jurga^3^; Eugenio Baraldi^1^; Maurizio Muraca ^1^

^1^University of Padova, Padova, Italy; ^2^Stem Cells and Regenerative Medicine Lab, Fondazione Istituto di Ricerca Pediatrica Città della Speranza, Padova, Italy; ^3^The Cell Factory BVBA (Esperite NV), Niel, Belgium

**Background**: Intravenous administration of mesenchymal stromal cells (MSCs)-derived extracellular vesicles (EVs) can reverse the development of bronchopulmonary dysplasia (BPD) in rodent models. However, systemic administration of EVs could cause concern in a fragile patient population such as preterm neonates. Thus, we suggest that intratracheal (IT) administration of MSC-EVs, if proven effective in a reliable animal model, could represent a safer and more convenient tool for future clinical studies on patients with BPD.

**Methods**: The study was conducted on Sprague Dawley rat pups exposed to normobaric oxygen concentration set at FiO_2_ 0.6 until postnatal day (P) 14. Experimental groups (*n* = 10) included healthy controls (room air), hyperoxia-exposed pups receiving IT vehicle only and hyperoxia exposed pups receiving IT either human Wharton Jelly-derived MSCs (2 × 10E6) or MSC-EVs (1.3 × 10E10) on days P3, P7 and P10. Animals were euthanized on P14. Alveolarization was stereologically assessed as described previously. The thickness of the medial layer of small pulmonary arteries was also morphometrically evaluated. Cytokine expression was analysed in lung lysate.

**Results**: Untreated hyperoxia-exposed animals showed lower total surface of air spaces, lower total alveoli number (Nalv) and higher mean alveolar volume (Valv) than normoxia-exposed animals. Treatment with both MSCs and MSC-EVs produced significant increase in Nalv and significant decrease in Valv compared to sham-treated animals. The medial layers of small pulmonary arteries were unchanged, probably due to the relatively short follow-up time. Reduced IL-10 and TGFb1 concentrations were found in the lungs of hyperoxic animals. Both parameters were significantly increased following both treatments.

**Summary/Conclusion**: Similarly to their cells of origin, MSC-EVs significantly improved both morphological and biochemical parameters in an animal model of BPD, suggesting that IT EVs administration could represent a convenient and effective approach to reverse the development of BPD treatment in preterm neonates.

**Funding**: This work was supported by the Department of Women’s and Children’s Health of the University of Padova.

PS01.12

Microvesicles induced in hyperglycaemic conditions regulate endothelial cell stiffness and cell shape fluctuations

Anna Elżbieta Drożdż ^1^; Tomasz Kołodziej^1^; Marta Targosz-Korecka^1^; Robert Jach^2^; Hubert Huras^3^; Ewa Stępień^1^

^1^Faculty of Physics, Astronomy and Applied Computer Science of the Jagiellonian University, Kraków, Poland; ^2^Department of Gynecological Endocrinology, Faculty of Medicine of the Jagiellonian University Medical College, Krakow, Poland; ^1^Department of Obstetrics and Perinatology, Faculty of Medicine of the Jagiellonian University Medical, Krakow, Poland

**Background**: Cell mechanical properties and shape fluctuations are related with cell local and transitional motility. Both control cell migration processes in wound healing. Hyperglycaemic conditions impair endothelial cell migration and elastic cell properties.

**Objectives**: Microvesicles (MVs) induced in hyperglycaemia can regulate endothelial cell mechanical properties and local motions.

**Methods**: Human umbilical vein endothelial cells (HUVECs) were cultured in preconditioned media (differential centrifugation) with MVs induced in (a) normoglycaemic – MV NGC and (b) hyperglycaemic – MV HG (25 mM/ml glucose) conditions. Cell shape fluctuations as cell local motions (CLM) were recorded and cell stiffness as elastic moduli (EM) was analysed. For CLM, HUVECs were cultured in density 1640 cells/cm^2^, recorded for 14 h and images were taken every 10 min. For EM, cells were incubated for 14 h in density 77,000 cells/cm^2^ and analysed with an atomic force microscope (AFM) in a contact mode. Average cell area (ACA) and shape parameters were calculated. MV density was in range between 4 and 8 mln per well (flow cytometry tested).

**Results**: ACA of HUVECs in NGC conditions was significantly lower than in HGC (1989 ± 811 vs. 2755 ± 1627 µm^2^; *p* = 0.05). In the presence of MV, ACA and shape were altered. MV NGC caused the area increase in HGC (2616 ± 35 vs. 2974 ± 1401 µm^2^; *p* = 0.05), incubation with MV HGC – no changes observed. Differences in solidity and circularity were also observed. Additionally, the MV (NGC and HGC) induced the stiffness increase (EM), both at the cell surface (1.86 ± 0.16 vs. 2.44 ± 0.87 kPa; *p* = 0.5) and in deeper cell layers (2.76 ± 1.01 vs. 4.68 ± 0.85 kPa; *p* = 0.05), when compared to non-conditioned medium.

**Summary/Conclusion**: Observed differences in ACA, stiffness and shape show that MVs regulate HUVEC local motility and mechanical properties in hyperglycaemic conditions. These findings suggest that impaired wound healing is regulated on a single cell level and brings a new insight to understand the underlying biophysical mechanisms.

**Funding**: This study was funded by the NCN grant (2012/07B/NZ5/02510).

PS01.13

Myoblast-exosome is a mediator of protective signal of remote ischaemic conditioning

Yan Yan ^1^; Morten Venø^2^; Susanne Venø^3^; Andrea Toth^3^; Morten Nielsen^3^; Jørgen Kjems^1^

^1^Interdisciplinary Nanoscience Center (iNANO), Aarhus University, Denmark, Aarhus, Denmark; ^2^Department of Molecular Biology and Genetics, Aarhus University, Aarhus, Denmark; ^3^Department of Biomedicine, Aarhus University, Aarhus, Denmark, Aarhus, Denmark

**Background**: Remote ischaemic conditioning (RIC) is a medical procedure that can attenuate ischaemic–reperfusion injury and can be executed by brief cycles of ischaemia and reperfusion in the arm or leg. Exosomes secreted from host cells can circulate in the blood stream and thereby transfer their content into recipient cells to impose new functions. Some studies also showed that exosomes could traverse through the blood–brain barrier. Our hypothesis is that the RIC procedure stimulates myoblast to secrete exosomes with a characteristic content of small RNA that can target remote organs and alleviate the acute ischaemia–reperfusion injury on remote organ.

**Methods**: C2C12 cells were cultured in 100 mm dishes and the media was changed to exosome collection media before hypoxia-reoxygenation (HR) treatment. The HR protocol consisted of five cycles of 1% O_2_ at 37°C for 10 min in hypoxia chamber, followed by 5% CO_2_/95% air incubator for 10 min at 37°C. Exosomes were collected by ultracentrifugation and characterized using nanoparticle tracking analysis and TEM. Exosome function was validated by *in vitro* angiogenesis assay and cell viability assay. Exosome RNA was purified. Small RNA libraries were prepared and used for deep sequencing.

**Results**: The size of exosomes secreted from C2C12 is approximately 120 nm and the yield is approximately 4000 exosomes/cell. In an angiogenesis assay test, HR-exosomes significantly increased the number of cell junctions and tubes, as well as the total length of tubes compared to normal cultured C2C12-exosomes and negative control (no exosomes). In the cell viability test, culturing C2C12 cells in hypoxia chamber for 5 h significantly slowed cell proliferation compared to normal cultured C2C12. Exosomes purified from both HR and normal cultured C2C12 significantly promoted cell proliferation of hypoxia treated C2C12 with HR exosomes exhibiting the strongest effect. Agilent small RNA assay showed that small RNAs (<100 nt) were enriched in C2C12-exosomes and NGS profiling of microRNAs revealed significant changes specifically when the C2C12 cells were HR treated.

**Summary/Conclusion**: In summary, HR treatment of C2C12 cells promoted the function of the secreted exosomes in angiogenesis and cell viability, which indicates that HR myoblast-exosomes can be a mediator of the protective function of RIC on remote damaged organs.

PS01.14

Stem cell exosomes as a biochemical cue for recovery from skin photo-ageing

Youn Jae Jung ^1^; Ji Suk Choi^1^; Jae Dong Kim^2^; Yong Woo Cho^1^

^1^Hanyang University, Ansan, Republic of Korea; ^2^Exostemtech Inc., Ansan, Republic of Korea

**Background**: Ultraviolet (UV) radiation is one of the most harmful environmental factors that accelerate skin **ageing**. Repeated exposures to UV radiation, in particular UVB, cause imbalance between dermal matrix synthesis or degradation by aberrant upregulation of matrix metalloproteinases (MMPs), which leads to overall skin photo-**ageing**. In this study, we investigated the effects of exosomes derived from human adipose-derived stem cells (HASCs) on photo-damaged human dermal fibroblasts (HDFs).

**Methods**: Exosomes were isolated from conditioned media (CM) during HASCs proliferation through prefiltration in 0.22 µm, followed by tangential flow filtration (TFF) with 500-kDa MWCO ultrafiltration membrane filter capsule. The collected exosomes were characterized by transmission electron microscopy (TEM), nanoparticle tracking analysis (NTA) and Western blot analysis. Total RNAs were extracted from HASC-exosomes and exosomal miRNAs were profiled using miRNA arrays. Cytokines in HASC-exosomes were analysed using human 80 cytokine array kit. The effects of HASC-exosomes were evaluated by monitoring of the cellular behaviours and expression of MMPs in UVB-exposed dermal fibroblasts.

**Results**: HASC-exosomes displayed a round shape and approximately 30–200 nm in diameter. HASC-exosomes were positive for exosomal surface markers, including CD9, CD63 and CD81. Various miRNAs and cytokines related to dermal matrix synthesis were identified in HASC-exosomes. We found that HASC-exosomes improve the migration ability of HDFs reduced by UVB irradiation. In addition, HASC-exosomes attenuate UVB-induced MMP expression and promote dermal matrix synthesis by regulating TIMP-1 and TGF-β1 expression.

**Summary/Conclusion**: We propose that HASC-exosomes could contribute to the restoration of UVB-irradiated dermal fibroblasts and are highly promising as an anti-photo**ageing** agent.

PS01.15

Improving cell viability by extracellular vesicles from amniotic fluid cells

Annalisa Radeghieri; Serena Ducoli; Lucia Paolini; Andrea Zendrini; Sara Busatto; Giulia Savio; Paolo Bergese; Giovanna Piovani

Department of Molecular and Translational Medicine, Brescia, Italy

**Background**: A growing number of studies suggest that stem cells (SCs) exert their therapeutic effect primarily by a paracrine regulation through extracellular vesicles (EVs) by delivering growth factors, proteins, nucleic acids and lipids. SC EVs have demonstrated the ability to regenerate tissues and neovascularization in models of myocardial infarction, muscle and kidney injury. Thus it is feasible that SC EVs could substitute SC to treat various diseases, circumventing issues associated with cell-based strategies, such as stress-induced necrosis or aberrant differentiation.

Amniotic fluid has been recently recognized as an important yet underutilized source of multipotent stem like cells, showing high plasticity and capacity to differentiate into the three types of germ layer cells. We have demonstrated that amniotic fluid cells (AFC) secrete a population of small (nanosized) EVs, which enclose the catalytic subunit of telomerase, the hTERT protein, suggesting a possible new activity for this protein. In this contribution, we will report on our last experiments and results in exploration of the functional properties of AFC EVs to improve cell viability and growth rate in different cell lines.

**Methods**: EVs from AFC culture medium were purified using sequential centrifugation steps. A biochemical (Western blot analysis) and biophysical (atomic force microscopy, AFM) characterizations were performed. EVs were then graded for purity and quantified by CONAN (COlorimetric NANoplasmonic) assay. Finally EV-based wound/healing and vitality assays were performed on different cell lines.

**Results**: The CONAN assay allowed us to assess purity and determine the molar concentration of the EV formulations while AFM imaging confirmed the sample to be composed of nanosized EV populations (50–100 nm). Incubation with EVs experiments gave promising results in terms of the possibility to use AFC EVs as additives to improve cell culture viability.

**Summary/Conclusion**: The contribution will present and discuss original results on EV mediated mechanisms by which AFC cells exert a positive effect towards slow growing cell cultures, with an interest on fundamental understanding of EV paracrine signalling and potential application of EVs as therapeutic agents in regenerative medicine.

**Funding**: This work was supported by University of Brescia research fund (ex 60%) to A.R., P.B. and G.P.

PS01.16

Extracellular vesicles secreted by dendritic cells can recruit mesenchymal stem/stromal cells: *in vitro* and *ex vivo* evidence

Andreia M. Silva ^1^; José H. Teixeira^1^; Ana R. Ferreira^2^; Maria I. Almeida^2^; Carla Cunha^2^; Daniela P. Vasconcelos^1^; Nuno Neves^3^; Mário A. Barbosa^1^; Susana G. Santos^2^

^1^i3S – Instituto de Investigação e Inovação em Saúde, Universidade do Porto, Portugal; INEB – Instituto de Engenharia Biomédica, Universidade do Porto, Portugal; ICBAS – Instituto de Ciências Biomédicas Abel Salazar, Universidade do Porto, Portugal., Porto, Portugal; ^2^i3S – Instituto de Investigação e Inovação em Saúde, Universidade do Porto, Portugal; INEB – Instituto de Engenharia Biomédica, Universidade do Porto, Portugal., Porto, Portugal; ^3^i3S – Instituto de Investigação e Inovação em Saúde, Universidade do Porto, Portugal; FMUP – Faculdade de Medicina da Universidade do Porto, Departamento de Cirurgia, Serviço de Ortopedia, Porto, Portugal., Porto, Portugal

**Background**: Mesenchymal stem/stromal cells (MSC) are being studied for bone regenerative therapies (1). They are likely recruited to lesion sites by activated immune cells, which secrete chemoattractants and extracellular vesicles (EV). We previously showed that human monocyte-derived dendritic cells (hDC) could recruit MSC via paracrine action (2). Here, we hypothesize that EV secreted by DC would be main effectors of MSC recruitment and that bone injury would influence DC-EV functionality.

**Methods**: EV were isolated from cultures of hDC (hDC-EV) and rat bone marrow (BM)-derived DC (rDC-EV). BM was isolated from an *in vivo* femoral bone defect model, at 3 and 14 days post-injury. EV effect on primary BM-MSC migration was tested by transwell migration assay. EV chemokine profile was analysed by membrane array.

**Results**: The results obtained show that hDC-EV significantly and dose-dependently promoted human MSC recruitment. EV content analysis revealed the presence of chemotactic mediators, with osteopontin and matrix metalloproteinase-9 being confirmed inside EV. Also, rDC-EV from 14 days post-injury significantly promoted rat MSC migration, compared to those from non-operated animals, whereas rDC-EV from 3 days post-injury significantly decreased it. Of note, rDC-EV from 14 days post-injury were enriched in MCP-1, whereas rDC-EV from 3 days post-injury were enriched in TIMP-1.

**Summary/Conclusion**: In conclusion, DC-EV can contribute to MSC recruitment, in a dose and time post-injury dependent fashion. These results will be further exploited for development of new bone tissue regenerative strategies.

**Funding**: Work funded by project NORTE-01-0145-FEDER-000012, Norte Portugal Regional Operational Programme (NORTE 2020), under PORTUGAL 2020 Partnership Agreement, through European Regional Development Fund (ERDF); Portuguese funds through FCT, PhD (AMS, JHT, DMV, DPV) and Post-Doc (MIA, CC) fellowships.

**References**: (1) Marcacci et al. (2007), Tissue Eng. 13; (2) Silva et al. (2014), PLoS ONE 9.

PS01.17

Human platelet derived exosomes induce chondrogenic differentiation

Miquel Antich-Rosselló; Maria Antonia Forteza-Genestra; Marc Blasco-Ferrer; Maria del Mar Ferrà-Cañellas; Antoni Gayà; Javier Calvo; Marta Monjo; Joana Maria Ramis

Group of Cell Therapy and Tissue Engineering Group, Research Institute on Health Sciences (IUNICS), University of the Balearic Islands, Palma de Mallorca, Spain

**Background**: Osteoarthritis (OA) affects more than 40 million people across Europe, thus becoming the fastest growing cause of disability worldwide. Although numerous treatments for various forms of arthritis have been identified, such therapies are restricted by considerable side effects and limited efficacy.

Tissue engineering approaches have emerged in recent years as a novel opportunity, and the use of platelet-rich plasma (PRP) constitutes an appealing biological approach to favour the healing of tissues otherwise doomed by a low healing potential, such as cartilage. Platelets constitute a reservoir of growth factors that promote cellular recruitment, growth and morphogenesis, and modulate inflammation. However, the need of autologous PL for an effective treatment limits its use. Here we propose the direct use of exosomes platelet derived as an alternative to PL. Exosomes are known to be subcellular vesicles between 30 and 100 nm which contain protein and nucleic acids capable to stimulate cell proliferation.

**Methods**: Exosomes derived from PL were isolated by ultracentrifugation (UC). The obtained exosomes were characterized by TEM (transmission electron microscopy), DLS (dynamic light scattering), AFM (atomic force microscopy) and for the presence of exosome markers by Western blot. Exosomes and PL were both tested on the chondrogenic ATDC-5 cell line. Metabolic activity and glycosaminoglycans staining (Alcian Blue Staining) were checked out. Moreover, gene expression assays were performed.

**Results**: Treatment with exosomes or with PL gave similar results as for metabolic activity, alcian blue staining or gene expression of marker genes.

**Summary/Conclusion**: In conclusion, exosomes platelet derived can be used as an alternative to platelet lysates for chondrogenic differentiation.

**Funding**: This work was supported by the Instituto de Salud Carlos III (contracts to J.M.R. and M.A.F.G.; CP16/00124), the Vicepresidència I Conselleria d’Innovació, Recerca I Turisme del Govern de les Illes Balears (contract to M.A.R.; FPI/2046/2017) and the Ministerio de Empleo y Seguridad Social with the Sistema de Garantía Juvenil (contracts to M.M.F.C. and M.B.F.). The authors thank Dr. F. Hierro and Dr. J. Cifre (UIB) for their technical contribution with TEM and AFM respectively.

PS01.18

Cell-free regenerative medicine: use of human platelet-derived extracellular vesicles to induce pre-osteoblast differentiation

Maria Antonia Forteza-Genestra; Miquel Antich-Rosselló; Miguel Artigues; Marc Blasco-Ferrer; Antoni Gayà; Javier Calvo; Marta Monjo; Joana Maria Ramis

Group of Cell Therapy and Tissue Engineering Group, Research Institute on Health Sciences (IUNICS), University of the Balearic Islands, Palma de Mallorca, Spain

**Background**: Platelet concentrated is used in regenerative medicine for its high content in growth factors and proteins. However, the need of autologous blood and the lack of standard protocols limits its clinical use. Using platelet derived-extracellular vesicles (EVs), such as exosomes (30–100 nm) or microvesicles (100–1000 nm), are an alternative to platelet concentrated due to their advantages since no autologous blood is needed and can be sterilized by filtration and stored until use.

Our aim was to test if platelet lysate and platelet-derived EVs extracted by different methods exerted the same effect on the differentiation of the pre-osteoblastic cell line MC3T3-E1.

**Methods**: Platelet-derived EVs were isolated by different methodologies: polyethylene glycol (PEG) precipitation, ultracentrifugation or the commercial kit Exo-Spin™. The obtained EVs were characterized in terms of size by TEM (transmission electron microscopy), DLS (dynamic light scattering), AFM (atomic force microscopy) and for the presence of EVs markers by Western blot. Five micrograms of isolated EVs or platelet lysate were used to treat MC3T3-E1 cells for 48 h and the effect in metabolic activity was studied by resazurin reduction.

**Results**: Exosomes isolation by PEG precipitation allows the obtaining of smaller size particles with a higher protein concentration compared to the other evaluated methods. In addition, platelet lysate and exosomes obtained by PEG precipitation lead to a similar metabolic activity on mouse pre-osteoblasts.

**Summary/Conclusion**: Thus, the platelet lysate effect on the cells could be due to the EVs present, suggesting that platelet-derived EVs could be used as alternative to platelet concentrates.

**Funding**: This work was supported by the Instituto de Salud Carlos III (contracts to J.M.R and M.A.F.G.; CP16/00124) and the Ministerio de Empleo y Seguridad Social with the Sistema de Garantía Juvenil (contracts to M.A.R. and M.B.F.). The authors thank Dr. F. Hierro and Dr. J. Cifre (UIB) for their technical contribution with TEM and AFM respectively.

PS02: EV Engineering and Sorting of Cargo in EVs Chairs: Dave Carter; Gregory Lavieu Location: Exhibit Hall 17:15–18:30

PS02.01

Engineering exosomes as refined drug delivery vehicles

Stefania Zuppone; Andrea Salonia; Riccardo Vago

Urological Research Institute, IRCCS San Raffaele Scientific Institute, Milan, 20132, Italy, Milan, Italy

**Background**: Exosomes are naturally secreted nanosized vesicles that recently emerged as suitable vehicles for the delivery of therapeutic molecules in cancer treatment. They have several advantages compared to current synthetic nanoparticles systems, which comprise their natural origin, controlled immunogenicity and absence of cytotoxicity. However, successful exosomes exploitation as drug carrier system still requires further investigation.

**Methods**: HEK293 cells were used for exosomes production. Exosomes isolation was performed by sequential centrifugations and specific exosomal markers and cargo encapsulation were detected by Western blot. Permeabilization with detergents and pH altering buffers, freeze-thaw cycles or sonication were used to incorporate exogenous therapeutic proteins into purified exosomes. Genetically engineering exosomes were obtained by transfecting cells with a construct encoding tetraspannins (CD9, CD63 and CD81) fused to a reporter gene.

**Results**: We compared different physical and chemical methods for exosome loading with therapeutic molecules to the genetic engineering of the donor cells. All methods for direct loading perturbed the integrity of vesicles and determined a limited incorporation of exogenous proteins. Instead, the expression of a fluorescent reporter gene fused to tetraspannins in donor cells resulted in a massive incorporation of fusion proteins in exosomes and structural preservation. To induce the selective release of exosome-carried, tetraspannin-fused therapeutic proteins in target tumour cells, we inserted a cleavage site, which was selectively processed by proteases over-expressed in model cancer cells.

**Summary/Conclusion**: We found genetic engineering as the most promising approach to produce exosomes carrying therapeutic molecules, due to structural preservation and increased encapsulation efficiency compared to other methods. Furthermore, we demonstrated that the introduction of a protease specific cleavage site conferred target selectivity to these therapeutic nanocarriers.

**Funding**: The project was funded by the Italian Ministry of Health.

PS02.02

EV-encapsulated small molecule inhibitor decreases viability in cancer cell lines

Eleana Hatzidaki; Ioanna Vlachou; Maria Papadimitriou; Ioannis Papasotiriou

Research Genetic Cancer Centre, Florina, Greece

**Background**: Here in Research Genetic Cancer Centre (RGCC) we are in the process of developing a novel small molecule ERK inhibitor. We have currently synthesized an intermediate molecule – RGCC169 – which needed to be tested in order to confirm we are using the appropriate tools. The limited solubility that this compound exhibits makes it difficult to enter the cell membrane and exert its effects.

**Methods**: EVs were isolated from human serum by PEG precipitation and their presence was confirmed by CD63 expression. EV intracellular fate was determined by fluorescence microscopy during various time points. RGCC169 was EV encapsulated and loading was determined by HPLC using both AcN and MeOH. RGCC169 cell sensitivity was determined using both a Her2 negative, PIK3CA mutated (MCF7) and a Her2 positive, PIK3CA/KRas mutated (HCT-116) cell line. EV-encapsulated RGCC169 cytotoxicity was evaluated by MTT viability assay on MCF7 cell line.

**Results**: EVs are delivered intracellularly by endocytosis within 30 min. We have successfully loaded our compound into EVs. AcN vs MeOH mobile phases give different loading efficiencies. Sensitivity to RGCC169 was greater in PIK3CA mutated cell lines. Encapsulated RGCC169 was shown to have increased cytotoxicity over RGCC169 alone.

**Summary/Conclusion**: MeOH gives higher encapsulation efficiency compared to AcN. This could either be due to the greater ability of MeOH to break apart EV pellets, or due to great variability of loading. EVs are delivered by endocytosis. Her2 positive, PIK3CA/KRas mutated cell lines are less sensitive to RGCC169 possibly due to the higher levels of activated ERK. EV encapsulation increased significantly cell sensitivity to RGCC169. The above findings confirm that we have successfully devised a delivery system for our novel molecule’s intracellular transport and that are indeed using the appropriate synthesis methods for the achievement of our final goal; that is the synthesis of a novel cytotoxic drug.

PS02.03

Fabrication of an EV sorting and sensing device using nanostructures

Sung-Wook Nam ^1^; Moon-Chang Baek^2^

^1^Department of Molecular Medicine, Kyungpook National University School of Medicine, Daegu, Republic of Korea; ^2^Department of Molecular Medicine, Kyungpook National University School of Medicine, Jung-Gu, Republic of Korea

**Background**: Extracellular vesicle (EV) sorting and sensing via nanostructures are important to achieve a size-dependent analysis of protein, miRNA and chemical cargo inside the vesicles. In this regard, an implementation of a lab-on-a-chip device having the EV sorting functionality has been pursued by utilizing the flow dynamics and physical properties of the particles.

**Methods**: To develop a sorting and sensing device for EV, we employed semiconductor processes. Nanostructures were fabricated upon a large-scale silicon wafer through a combined method of electron beam lithography (EBL) and photolithography. In this presentation, we introduce two approaches of the device fabrications: (1) to utilize the silicon nanostructure as an EV sorting device and (2) to produce polydimethylsiloxane (PDMS) microfluidics replicated from the given silicon wafer as a mould structure.

**Results**: Upon 200 mm silicon wafer, we demonstrate 200 nm features to build EV sorting and sensing structures. We use a positive-type electron-beam resist material to construct nanopillar-based fluidic channels. By connecting the nanopillar-based channels to the bigger structures spanning across the silicon wafer, we prepare a lab-on-a-chip device coupled with the EV sorting element. Also, we develop a PDMS fabrication system using the silicon wafer as a mould structure. The EV sorting features are fabricated in the PDMS fluidic chamber.

**Summary/Conclusion**: We demonstrate an EV sorting and sensing device by implementing nanostructures in a lab-on-a-chip. Our method may offer a manufacturing system of biochips that have versatile functions including EV sorting, sensing and chemical analysis.

**Funding**: This research was supported by the Bio&Medical Technology Development Program of the National Research Foundation (NRF) funded by the Ministry of Science & ICT (2017M3A9G8083382).

PS02.04

Novel antibody-mediated drug delivery system for targeting exosomal microRNA

Asako Yamayoshi ^1^; Ryo Konishi^2^; Akio Kobori^2^; Naoto Yamashita^3^; Eishi Ashihara^3^; Akira Murakami^3^; Hiroshi Sugiyama^4^

^1^The Hakubi Cemter for Advanced Research, Kyoto Univiersity, Kyoto, Japan; ^2^Department of Biomolecular Engineering, Kyoto Institute of Technology, Kyoto, Japan; ^3^Department of Clinical and Translational Physiology, Kyoto Pharmaceutical University, Kyoto, Japan; ^4^Graduate School of Science, Kyoto University, Kyoto, Japan

**Background**: Recently, microRNAs (miRNAs) have been identified in exosomes, which can be taken up by neighbouring or distant cells. It has also been reported that such miRNAs (exosomal-miRNAs) regulates gene expression in the recipient cells. According to recent reports, the aberrant expression of miRNAs is associated with most pathological disease processes, including carcinogenesis. Therefore, exosomal-miRNAs are considered as significant therapeutic targets for cancer therapy. However, there is no report to regulate the function of miRNAs in exosomes. In this study, we try to develop novel drug delivery system using anti-exosome antibody–oligonucleotide conjugates (ExomiR-Tracker) for functional inhibition of exosomal-miRNAs.

**Methods**: Cellular uptakes and localization of ExomiR-Trackers were evaluated by confocal microscopy. We evaluate the inhibitory effects of ExomiR-Tracker according to previous report (Ariyoshi et al., Bioconj Chem. 2015).

**Results**: First, we evaluated cellular uptakes and localization of anti-miR-conjugated antibody by confocal microscopy. In this experiment, Alexa647-labelled anti-miR and cationized anti-exosome antibody were used for ExomiR-Tracker. Fluorescence signals were successfully observed within HeLa cells. In contrast, in the case of control molecules, no fluorescent signals were observed.

Next, we evaluated inhibitory effects of ExomiR-Tracker against miRNA functions. It was found that luminescence intensity of ExomiR-Tracker-treated cells was recovered compared to the case of control ExomiR-Tracker. This result suggests that ExomiR-Tracker successfully inhibit the function of miR-Luc in HeLa cells. We also confirmed these effects *in vivo*.

**Summary/Conclusion**: We successfully demonstrated that ExomiR-Tracker is incorporated into the recipient cells and inhibits miRNA function *in vitro* and *in vivo*. To the best of our knowledge, this is the first example of regulating exosomal-miRNA in the recipient cells.

**Funding**: This study was partly supported by a Grant-in-Aid for Scientific Research from the Ministry of Education, Science, Sports and Culture of Japan（Grant No. 15K05564）and JST, PRESTO (Grant No. JPMJPR178A), Japan.

PS02.05

Designer RNA binding proteins for loading exogenous RNA into extracellular vesicles

Olga Shatnyeva ^1^; Anders Gunnarsson^2^; Euan Gordon^3^; Elisa Lázaro-Ibáñez^1^; Lavaniya Kunalingam^2^; Nikki Heath^4^; Xabier Osteikoetxea^5^; Ross Overman^6^; Marcello Maresca^7^; Niek Dekker^1^

^1^Discovery Biology, Discovery Sciences, IMED Biotech Unit, AstraZeneca, Gothenburg, Sweden, Mölndal, Sweden; ^2^AstraZeneca R&D, Innovative Medicines, Discovery Sciences, Mölndal, Sweden; ^3^AstraZeneca R&D, Innovative Medicines, Discovery Science, Mölndal, Sweden; ^4^Discovery Biology, Discovery Sciences, IMED Biotech Unit, AstraZeneca, Alderley Park, Macclesfield, UK; ^5^Discovery Biology, Discovery Sciences, IMED Biotech Unit, AstraZeneca, Alderley Park, Macclesfield, UK; ^6^Discovery Biology, Discovery Sciences, IMED Biotech Unit, AstraZeneca, Alderley Park, Macclesfield, UK; ^7^AstraZeneca R&D, Innovative Medicines, Discovery Sciences, Mölndal, Sweden

**Background**: Recently extracellular vesicles (EVs) have gained tremendous attention as a delivery vehicle for effective targeted drug delivery. RNA-based therapeutics has great potential to target a large part of the currently undruggable genes and gene products and to generate entirely new therapeutic paradigms in disease. However, deliverability and stability of RNA-based drugs is still limited, which is primarily due to the lack of appropriate delivery systems. Recent studies have shown that EVs are natural carriers of miRNA and this intrinsic property could be exploited as a gene delivery system. Current approaches for loading of EVs with RNA are *in vitro* electroporation, transfection, co-incubation, co-expression of target RNA and zipcoding but all of these suffer from poor efficiency. Our study aims to use RNA binding proteins (RBP) fused to EV marker proteins for *in vitro* loading of EVs with cargo RNA tagged with the cognate RNA recognition elements.

**Methods**: A RNA library of target RNA fused to a specific RNA binding sequence was generated where the position of the recognition site was varied. We used surface plasmon resonance analysis to characterize a library of modified sgRNAs for its ability to form the complex between the RNA binding protein and sgRNA *in vitro*. Next, Expi293 cells were co-transfected with the set of modified sgRNAs and RBP fused to EV markers following EV purification by differential ultracentrifugation. EVs were then characterized by nanoparticle tracking analysis (NTA), Western blot and single molecule microscopy and efficiency of sgRNA loading to exosomes was determined using qPCR.

**Results**: We found that introduction of RNA recognition elements to the tetraloop, loop 2 and 3ʹ end of sgRNA did not interfere with binding to RBP. Fusion proteins between RBP and EV proteins incorporate RBP into EVs efficiently and results in selective targeting to EVs of sgRNA containing the RNA recognition binding elements. Additionally, we found that EV from cells expressing sgRNA together with RBP contained 10-fold more sgRNA compared to EV from cells expressing sgRNA only.

**Summary/Conclusion**: Overall, in this study, we have developed novel approach for RNA loading into EVs using cell engineering and demonstrated a proof of principle with Expi293 EVs. We envision this approach will be useful for loading of RNA a variety of therapeutic applications.

PS02.06

A comparative study of methodologies to encapsulate gold nanoparticles into exosomes for theragnostics

María Sancho ^1^; Manuel Beltrán-Visiedo^1^; Marimar Encabo-Berzosa^1^; Victor Sebastian^1^; Manuel Arruebo^1^; Jesús Santamaría^1^; Pilar Martín-Duque^2^

^1^Department of Chemical Engineering, Aragon Nanoscience Institute (INA), University of Zaragoza, Zaragoza, Spain; ^2^Fundación Araid-IACS, Zaragoza, Spain, Zaragoza, Spain

**Background**: Apart from the role of exosomes as intercellular communication vehicles, they have been recognized as excellent disease biomarkers and good evaluators of the prognosis of different pathologies. Hollow gold nanoparticles (HGNs) have attracted the interest of recent research because of their biomedical potential as drug carriers, gene vectors, imaging tools and therapeutic agents. HGNs are able to reach the tumours eliminating malignant cells when applying optical hyperthermia. Moreover, HGNs could be used for molecular imaging (by microscopy or CT to track the vector that may carry them. Thus, the encapsulation of these nanoparticles with biomimetic moieties such as exosomes would maximize the extravasation, would prevent their recognition by the immune system and would increase their steric stabilization, all resulting in a more efficient accumulation of nanoparticles in the pathological area.

**Methods**: We combined theragnostics potential of exosomes carrying products derived from nanotechnology such as HGNs. We used different methods of encapsulation of HGNs in exosomes derived from B16F10 cells (electroporation, passive loading at room temperature, thermal-shock, sonication or saponin-assisted loading). Furthermore, exosomes derived from B16F10 cells loaded with HGNs were also directly purified from the supernatants of cells preincubated with the HGNs, achieving a high yield of exosomes loaded with NPs. The obtained vectors were characterized by TEM and DLS.

**Results**: We show that HGNs internalization into B16F10 exosomes was achieved almost by all the physicochemical methods tested. However, only about 15% of the exosomes were loaded with nanoparticles. Nevertheless, incubation of B16F19 cells with HGNs and subsequent purification of the loaded exosomes allowed us to obtain up to 50% of internalization rates.

**Summary/Conclusion**: Therefore, as HGNs could be used for therapy (by using optical hyperthermia) or imaging in a CT scanner, the results obtained in this work open the possibility of using exosomes as vectors for delivering AuNPs to different pathologies, including tumours. The possibility of the tumours to be treated by hyperthermia in the case of cancer or the imaging of the exosomes migrating in real time to different pathological areas would be feasible, showing a great potential and diversity on the diseases to be monitored.

PS02.07

Remote loading of ester-based prodrugs and fluorescent labels using intravesicular hydrolases

Linglei Jiang ^1^; Pieter Vader^2^; Wim Hennink^3^; Raymond M. Schiffelers^2^

^1^UMC Utrecht, Utrecht, The Netherlands; ^2^Department of Clinical Chemistry and Haematology, UMC Utrecht, The Netherlands; ^3^Utrecht Institute for Pharmaceutical Sciences, Utrecht, The Netherlands

**Background**: Extracellular vesicles (EVs) are promising drug carriers due to their attractive biocompatibility and inherent targeting ability. It has been proven to be challenging to incorporate molecules selectively into the interior of EVs. For instance, electroporation can induce EV membrane pore formation, through which hydrophilic molecules can be loaded. Disappointingly, it has been shown to result in significant EV aggregation, which obscures actual loading efficiency. Surfactant-facilitated loading compromises EV membrane integrity allowing the passage of hydrophilic compounds into the EV interior. However, due to the low intravesicular volume, the absolute loading of compounds is usually limited.

Here we investigated the enzyme gradient across the EV membrane as a driving force for intravesicular compound accumulation. Membrane-permeable compounds that are converted by intravesicular enzymes into membrane-impermeable molecules were used in an attempt to promote efficient remote loading. By profiling hydrolase activity we identified EVs of various types and sources that were most likely to benefit for remote loading.

**Methods**: A431, skov3 and HEK293 cell lines were cultured in serum-free media. EVs were isolated by size exclusion chromatography. The total hydrolase activity was profiled by ActivX TAMRA-FP Serine Hydrolase Probes after proteins were separated by gel electrophoresis. The activity of two specific hydrolase subsets, i.e. acetylcholinesterase and carboxylesterase, were investigated using a colorimetric assay and fluorescent assay respectively.

**Results**: The EVs from different sources show different hydrolase patterns. The hydrolase profile of the EVs is different from its parental cell. Sensitive colorimetric and fluorescent assay was validated by using donor cell lysate, there is a good correlation between OD412 nm (or Ex/Em 490/526) and the protein amount of cell lysate within the range of 1.6–100 µg. Results on acetylcholinesterase and carboxylesterase comparison among EV types is still under investigation. Loading of prodrug gallate ester and mycophenolate mofetil is also under study now.

**Summary/Conclusion**: The hydrolase cargo differs between different EVs and between parental cells. The presence of hydrolase inside EV presents a novel promising strategy for hydrophilic drug loading.

PS02.08

Preparation and function of CD9-integrated proteoliposomes

Mitsuru Ando ^1^; Shuheng Yan^1^; Yoshihiro Sasaki^1^; Kazunari Akiyoshi^2^

^1^Department of Polymer Chemistry, Graduate School of Engineering, Kyoto University, Kyoto, Japan; ^2^Kyoto University, Kyoto, Japan

**Background**: Tetraspanins are well-known as the representative exosomal membrane proteins. However, their biological functions on exosomes have not been well elucidated. Relation of CD9, one of tetraspaninin, in sperm–egg fusion process and interaction of recombinant ECL2 domain of CD9 with integrin were reported. We developed the effective preparation method of proteoliposomes by using cell-free membrane protein synthesis/liposomes system (so-called artificial cell system). In this study, we prepared full-length CD9-integrated liposomes using our artificial cell system and investigated functions of CD9 liposome.

**Methods**: Plasmid DNA construction: pURE-CD9 was constructed by human CD9 cDNA into the pURE1 vector.

**Preparation**: Liposomes were prepared using natural swelling method. Cell-free synthesis of CD9 was performed with liposomes. The proteoliposomes were purified by density gradient ultracentrifugation.

**Cellular uptake**: Cellular binding and uptake of CD9-proteoliposomes was evaluated by using flow cytometer after incubation proteoliposomes with HCT116 cells. Competitive uptake inhibition was performed by co-incubation of proteoliposomes and integrin alphaVbeta3 ligand, vitronectin.

**Results**: In the presence of liposomes, more than half of cell-free synthesized CD9 was directly reconstituted to liposome. The immunoprecipitation assay showed that ECL2 domain of CD9 was protruded to outside of the liposomes, indicating that, at least a part of, synthesized CD9 showed similar orientation to that in the cellular membrane. Next, we investigated the cellular uptake of CD9-proteoliposomes in integrin alphaVbeta3-overexpressing HCT116 cells. The CD9 proteoliposomes was strongly interacted with cells in comparison of control proteoliposomes. The interaction was probably integrin-mediated process due to the inhibition of the uptake by vitronectin.

**Summary/Conclusion**: We successfully constructed bioactive full-length CD9-integrated proteoliposomes. Such artificial exosomes containing exosomal membrane proteins such as tetraspanins by using cell-free membrane protein synthesis/liposome system should be useful for understanding of biological functions of membrane proteins in exosomes.

PS03: EV Biogenesis and Uptake Chairs: Ana Gradilla; Frederick Verweij Location: Exhibit Hall 17:15–18:30

PS03.01 = OWP3.01

Sarco/endoplasmic reticulum ATPase inhibition activates calcium signalling pathways for microvesicle biogenesis

Jack D. Taylor^1^; Michael Johnson^2^; Gregory Monteith^3^; Mary Bebawy^4^

^1^University of Technology Sydney, Sydney, Australia; ^2^School of Life Sciences, University of Technology Sydney, NSW, Sydney, Australia; ^3^The School of Pharmacy, The University of Queensland, Brisbane, Australia; ^4^The Graduate School of Health, The University of Technology Sydney, Sydney, Australia

**Background**: An increase in intracellular Ca2+ is a key initiator of microvesicle (MV) biogenesis. The Ca2+-signalling pathway(s) implicated in this are currently unknown. This study aims to elucidate the Ca2+ pathways involved in MV biogenesis in malignant and non-malignant cells in an attempt to identify selective drug targets for vesicle inhibition.

**Methods**: Interrogation of the Ca2+ signalling pathway was done using the SERCA inhibitor, thapsigargin (TG), the Calpain inhibitor II (ALLM) and the inhibitor of Store Operated Ca2+ entry (YM58483). AFM was used to study cell surface topography in response to inhibitors in HBEC-D3, MCF-7, and MCF-7/Dx cells (see Taylor et al., 2017). MV isolation and flow cytometric quantification were done as per Roseblade et al. (2015). Real-time deconvolution (DeltaVision personalVD, Elite) and super resolution (DeltaVision OMX Blaze) microscopy were used for live cell imaging using CellLight Plasma Membrane-RFP, Bacmam 2.0®.

**Results**: ALLM selectively inhibited vesiculation in malignant cells confirming a basal Ca2+-calpain dominant pathway. This was not observed for non-maligant cells confirming an alternative vesiculation pathway independent of calpain (Taylor et. al., 2017). Depletion of endoplasmic reticulum (ER) stores by TG alone resulted in slight and significant increases in vesiculation in malignant and non-malignant cells respectively, suggesting a maintained level of Ca2+ through a SOCE pathway. In the presence of YM58483 alone we saw no significant effect above basal levels in both cell types. In the presence of TG and YM58483 we observed inhibition of vesiculation, consistent with a SERCA/SOCE mediated regulation of vesiculation. Consequently, only differentiator in vesiculation in malignant vs non-malignant cells appears to be the involvement of calpain rather than Ca2+ signalling through SECRA/SOCE. In visualising the morphology of the cells using both AFM and live cell imaging we observed vesiculation to be perinuclear, clustered and polarised in MCF-7 cells at rest and upon activation in both cell types

**Summary/Conclusion**: We show for the first time the involvement of SERCA/SOCE Ca2+ signalling in MV vesiculation. Differences in basal vesiculation in malignant and non-malignant cells are at the level of calpain rather than the SERCA/SOCE pathway.

PS03.02

Antibiotic-induced release of small extracellular vesicles with surface-associated DNA

Andrea Németh^1^; Norbert Orgovan^2^; Barbara W Sodar ^1^; Xabier Osteikoetxea^3^; Krisztina Pálóczi^1^; Ágnes Kittel^4^; Lilla Turiak^5^; Zoltan Wiener^6^; Sára Tóth^1^; Robert Horvath^2^; Edit Buzas^7^

^1^Department of Genetics, Cell- and Immunobiology, Semmelweis University, Budapest, Hungary; ^2^Institute of Technical Physics and Materials Science, Hungarian Academy of Sciences, Budapest, Hungary; ^3^Discovery Biology, Discovery Sciences, IMED Biotech Unit, AstraZeneca, Alderley Park, Macclesfield, UK; ^4^Institute of Experimental Medicine, Hungarian Academy of Sciences, Budapest, Hungary; ^5^Research Centre for Natural Sciences, Hungarian Academy of Sciences, Budapest, Hungary; ^6^Semmelweis University, Department of Genetics, Cell and Immunobiology, Budapest, Hungary; ^7^Semmelweis University, Department of Genetics, Cell and Immunobiology, Budapest, Budapest, Hungary

**Background**: Ciprofloxacin, an antibiotic widely used both in cell cultures and human therapy, is known to induce genotoxic stress in Jurkat cells. Here we investigated the impact of sustained Ciprofloxacin exposure on Jurkat cell extracellular vesicle release.

**Methods**: Extracellular vesicles (large, intermediate and small ones) released by antibiotic-treated and control Jurkat cells were characterized by flow cytometry, tunable resistive pulse sensing and transmission electron microscopy. PCR was performed to detect mitochondrial DNA and genomial DNA sequences associated with extracellular vesicles. Binding of extracellular vesicles to fibronectin was assessed with a label-free optical biosensor. The protein content of the different vesicle populations was analysed by mass spectrometry.

**Results**: We demonstrated that extracellular vesicles released upon sustained Ciprofloxacin treatment carry substantial amounts of DNA. As verified by DNase I treatment, vesicles smaller than 200 nm carried surface-associated DNA. Using density gradient ultracentrifugation we identified two populations of small vesicles. Only one of them carried DNA on their surface. In addition, we demonstrated that exofacial DNA on small extracellular vesicles increased vesicle binding to fibronectin.

**Summary/Conclusion**: Our data demonstrate that a substantial amount of DNA is detectable on the surface of small extracellular vesicles upon sustained exposure of cells to Ciprofloxacin. This is in contrast to the earlier assumption that DNA is an internal cargo molecule of extracellular vesicles.

**Funding**: This work was supported by National Scientific Research Program of Hungary (OTKA) #11958 and #120237; #PD104369, #PD112085; #PD 109051, NVKP_16-1-2016-0017 and NVKP_16-1-2016-0007, MEDINPROT Program, BMBS COST Action BM1202 ME HAD, FP7-PEOPLE-2011-ITN-PITN-GA-2011-289033 DYNANO, Lendület program of the Hungarian Academy of Sciences, Starting Grant by the Semmelweis University (Z.W.) and by the ERC_HU grant of NKFIH. Z.W. is supported by the János Bolyai Research Fellowship (Hungarian Academy of Sciences).

PS03.03

S-palmitoylation is a post-translational modification of Alix that regulates its interaction with the CD9 tetraspanin

Daniele P. Romancino^1^; Valentina Buffa^1^; Stefano Caruso^2^; Antonella Bongiovanni ^1^

^1^Institute of Biomedicine and Molecular Immunology (IBIM), National Research Council (CNR), Palermo, Italy; ^2^UMR-1162, Functional Genomics of Solid Tumors, Inserm, Paris, France

**Background**: The multifunctional protein Alix is a *bona fide* extracellular vesicle (EV) regulator. Skeletal muscle (SkM) cells can release Alix-positive nano-sized EVs directly from their plasma membrane, offering a new paradigm for understanding how myofibres communicate within skeletal muscle and other organs. S-palmitoylation is a reversible lipid post-translational modification (PTM) that is involved in different biological processes, such as the trafficking of membrane proteins and stabilization of protein interaction.

**Methods**: Here, we have evaluated the extent to which S-palmitoylation is functionally linked to Alix and EVs by: (i) a comparative analysis of publicly available palmitoyl- and exosome-proteome data sets and (ii) altering protein palmitoylation, using a specific inhibitor (2-Br-Palmitate; 2BP) and evaluating S-palmitoylation of Alix as well as its subcellular distribution and interaction in SkM cells.

**Results**: We found a higher percentage of S-palmitoylated proteins in exosomes, compared to all the other cellular compartments. This finding suggests that this PTM could be a distinctive signature for exosomal proteins. By coupling bioinformatic observation with biochemical analyses, we have also determined that endogenous Alix undergoes S-palmitoylation. In particular, exosomal Alix is palmitoylated to a larger extent than cellular Alix, and the inhibition of palmitoylation altered its subcellular localization. Furthermore, endogenous Alix interacts with CD9, and S-palmitoylation supports this interaction, as it also does for tetraspanin complexes in the tetraspanin enriched microdomains.

**Summary/Conclusion**: Thus, we propose that S-palmitoylation might regulate the proper function of Alix in facilitating interactions among exosome-specific regulators in SkM-derived exosome biogenesis. Essential discoveries related to SkM-derived EVs may help in designing engineered exosomes which can be employed in the tissue regeneration field, e.g. to help in recovery from muscle atrophy and/or injury.

**Funding**: The research leading to these results has been funded by the Italian Ministry for Education, University, and Research in the framework of the Flagship Project NanoMAX.

PS03.04

An *in vivo Drosophila* RNAi screen for identification of secretory multivesicular body trafficking factors

Leonie Witte; Karen Linnemannstoens; Julia Christina Gross

University Medical Center Göttingen, Goettingen, Germany

**Background**: During endosomal maturation, intraluminal vesicles bud into multivesicular bodies (MVB). MVBs can then be trafficked towards the cell membrane and release these vesicles into the extracellular space as exosomes. However, not all MVBs will secrete their content as exosomes. Instead, only a subset of MVBs will travel towards the plasma membrane for the secretion of exosomes, while the other subset will fuse with the lysosome to induce content degradation. The mechanism of how MVBs are divided into either subset and how secretory MVBs are targeted towards the cell membrane remains elusive.

**Methods**: The interaction of specific trafficking factors with cytoplasmic surface proteins could destine MVBs towards either direction by directly mediating their trafficking properties. In order to identify trafficking factors involved in the secretion of exosomes we are conducting an RNAi screen in *Drosophila* wing imaginal discs.

In this model system, Wg is expressed in a specific cell population and exosome secretion of Wg from this cell stripe is involved in wing development. Co-expression of the exosomal marker Tsp96F-mCherry and monitoring of Wg and Tsp96F-mCherry secretion by immunofluorescence microscopy serves as a readout for exosome secretion *in vivo*, thus allowing us to screen for secretion defects upon knockdown of potential trafficking factors.

**Results**: Using this model, we are screening multiple trafficking-related candidate proteins by analysing and quantifying the central vs. peripheral as well as the apical vs. basal distribution of Wg and Tsp96F-mCherry. Indeed, knockdown of specific *Drosophila* trafficking factors leads to visible changes in Tsp96F-mCherry and Wg distribution in wing imaginal discs, thus implying a role in their secretion.

Further investigation of human orthologues of motor proteins potentially involved in MVB trafficking in human colorectal cancer cells reveals a connection between a candidate kinesin and EV secretion. We are currently looking into its influence on the intracellular trafficking of MVBs and exosomal markers and on Wnt trafficking as an exemplary cargo travelling on exosomes.

**Summary/Conclusion**: Taken together, we are using a *Drosophila in vivo* model system and human cell culture to identify and validate evolutionary conserved trafficking factors mediating intracellular transport of MVBs and the release of EV.

PS03.05

Kinase modifiers of exosome secretion in PDAC cells

Sandra Polaschek; Rebecca Schmid; Tim Eiseler; Thomas Seufferlein

Universitätsklinikum Ulm, Ulm, Germany

**Background**: Pancreatic ductal adenocarcinoma (PDAC) are characterized by poor prognosis due to late stage diagnosis and early metastasis in the majority of cases. It is therefore vital to understand the factors that determine the evolution of tumours and define strategies that allow to prevent distant metastasis. Kinases are important regulators of PDAC tumour growth, progression and metastasis. Certain kinases involved in PDAC progression were further shown to modulate exosome secretion, e.g. pyruvate kinase M2 (PKM2). Secretion of exosomes has emerged as an important feature to determine and shape the premetastatic niche of PDACs. In particular, exosomal microRNA cargo is known to enhance invasiveness, drug resistance, modulate immune response and cross-talk of PDACs to pancreatic stellate cells.

**Methods**: We will perform a flow cytometry-based screening with immuno-purified exosomes to identify novel kinase regulators of exosome secretion in PDAC cells.

**Results**: For an initial screening, stable Panc1-CD81-mcherry and cells are transduced with lentiviruses against single kinase isoforms. To this end we will utilize a whole kinome shRNA library present in our lab. Following knockdown of individual kinases fluorescent CD81-positive exosomes will be adsorbed to anti-CD81-Dynamag beats and subjected to flow cytometry analysis. Positive hits will be re-screened using Panc1-CD63-EGFP and Panc1-TSG101-mcherry cells. Subsequently, PDAC relevant re-screen targets will be analysed by performing a full characterization according to MISEV criteria. In addition, we aim to identify changes of cargo content, in particular microRNAs by running a miR microarrays analysis (Agilent).

**Summary/Conclusion**: By completing this kinome-wide screening for kinase regulators of exosome secretion in PDAC, we hope to identify novel hits that will affect PDAC carcinogenesis, tumour progression and metastasis.

**Funding**: This study was funded by Deutsche Forschungsgemeinschaft GRK 2254 HEIST.

PS03.06

Modifications of the glycome of extracellular vesicles affect their biodistribution in mice

Félix Royo ^1^; Unai Cossio^2^; Jordi Llop^2^; Juan M. Falcón-Pérez^1^

^1^CIC bioGUNE, CIBERehd, Bizkaia Science and Technology Park, Derio, Bizkaia, Spain, Derio, Spain; ^2^CIC biomaGUNE, Donostia, Spain

**Background**: One of the most exciting objectives in the field of extracellular vesicles (EVs) is to be able to target them specifically against certain tissues. Recent data point towards the influence of surface proteins in the biodistribution of EVs in a living organism. It is our hypothesis that glycosylation, an important protein modification that plays a key role in ligand-binding recognition, could influence the affinity of EVs for different tissues.

**Methods**: Purified EVs derived from hepatic cells were treated with a neuraminidase, an enzyme that digests the terminal sialic acid residues from glycoproteins. Afterwards, EVs were labelled with ^[124I]^NaI and injected in mice intravenously or in the hook (the lateral tarsal region just above the ankle). The amount of radioactivity in major organs was measured at different time points after administration both *in vivo* using positron emission tomography and *ex vivo* (after animal sacrifice) using dissection and gamma counting.

**Results**: As expected, intravenous injection leads to rapid accumulation of EVs in the liver, contrary to ^[124I]^NaI (no EVs, used as the control). After some hours, the distribution leads to the presence of EVs in different organs, and interestingly, also in brain. Glycosidase-treated EVs showed an important accumulation in the lungs compared with intact EVs. This pattern was also confirmed in the animals injected through the hook.

**Summary/Conclusion**: The EVs derived from hepatic cell lines are systemically distributed in several organs, although the main accumulation occurs in the liver. The modification of the glycome that decorates the EVs surface affects the distribution of these vesicles, allowing the transformed EVs to reach more abundantly the lungs. Further studies will help to determine different protocols to target a variety of organs.

**Funding**: This work was supported by RAMON ARECES FUNDATION and The Spanish Ministry of Economy and Competitiveness MINECO (PLAN NACIONAL).

PS03.07

A quantitative method to measure EV uptake

Victor Toribio ^1^; Beatriz Cardeñes^2^; Sara Morales-Lopez^3^; Soraya López-Martín^4^; Carlos Cabañas^2^; María Yáñez-Mó^5^

^1^Centro de Biología Molecular “Severo Ochoa” CSIC/UAM, Madrid, Spain; ^2^CBM-SO, CSIC, Madrid, Spain; ^3^Instituto de Investigación Sanitaria Princesa (IIS-IP), Madrid, Spain; ^4^Molecular Biology Center Severo Ochoa (CBM), Instituto de Investigación Sanitaria Princesa (IIS-IP), Madrid, Spain; ^5^Departamento de Biología Molecular, UAM, Madrid, Spain

**Background**: Because EV size lies below the limit of resolution of optical techniques, discrimination between EV binding to the target cell and uptake is usually not feasible with microscopy or cytometry techniques, leading to artefactual results. Our aim was to construct a suitable and quantitative method to analyse and explore the molecular mechanisms of EV uptake by the target cells, based on tetraspanins, classical EV-markers.

**Methods**: Human tetraspanins CD9 and CD63 were fused to a dual GFP-Luciferase-split vector tag. Incorporation of fusion proteins into EVs was assessed by bead-based flow cytometry and Western blot. Measurement of binding and uptake was performed by a combination of classical Renilla substrates and Enduren.

**Results**: Dual GFP-Luciferase-split constructs of tetraspanins were shown to present the same subcellular localization than endogenous proteins. In addition, by both bead-based flow cytometry and Western blot they could be correctly detected at EVs after lentiviral infection of producing cells. Incubation of target cells that expressed the complementary domains of the dual GFP-Luciferase-split construct with transfected exosomes could not recover the fluorescence or the luciferase function. However, when EVs carried the fully reconstituted Dual-GFP-Luciferase protein, we could distinguish the binding from active uptake by target cells by the combination of different Renilla Luciferase substrates. This method was shown to be highly sensitive and quantitative, making possible a high-throughput screen to unravel the molecular mechanisms of EV uptake in different biological systems.

**Summary/Conclusion**: We here describe a highly sensitive and quantitative method to analyse the molecular mechanisms of EV uptake in different biological systems.

**Funding**: This work was supported by grants from Fundación Ramón Areces, grants BFU2014-55478-R and REDIEX, and SAF2015-71231-REDT from Ministerio de Economía y Competitividad.

PS03.08

Ligand–receptor Interactions in exosome targeting in ischemic acute kidney injury

Jose Luis Vinas ^1^; Matthew Spence^1^; Alex Gutsol^1^; William Knoll^1^; David Allan^2^; Burns Kevin^1^

^1^Kidney Research Centre, Ottawa Hospital Research Institute, University of Ottawa, Ottawa, Canada; ^2^Ottawa Hospital Research Institute, University of Ottawa, Ottawa, Canada

**Background**: Infusion of human cord blood endothelial colony forming cell (ECFC)-derived exosomes protects mice against ischaemia/reperfusion acute kidney injury (AKI), via transfer of exosomal microRNA-(miR)-486-5p. Mechanisms mediating recruitment and retention of exosomes to injured tissues are unclear.

The interaction of CXC chemokine receptor type 4 (CXCR4) with stromal cell-derived factor (SDF)-1α has been shown to promote ECFC adhesion and migration in hypoxic endothelial cells. We therefore hypothesized that ECFC exosomes specifically target ischaemic kidneys, via the CXCR4/SDF-1α interaction.

**Methods**: Exosomes were isolated from ECFC conditioned media by serial centrifugation and characterized by nanoparticle tracking, immunoblots and electron microscopy. Ischaemia–reperfusion kidney injury was induced in mice by renal vascular clamp, with intravenous infusion of DiR- or PKH-26-labelled exosomes. Optical imaging determined the biodistribution of exosomes, and tissue miR-486-5p levels were measured by qPCR. Human umbilical vein endothelial cells (HUVECs) were cultured to study the role of CXCR4/SDF1-α interaction in normoxic and hypoxic conditions.

**Results**: Infusion of exosomes increased miR-486-5p levels only in kidneys after 30 min, 4 h and 24 h of reperfusion. Biodistribution showed selective targeting of exosomes to the kidneys. By histology, PKH-labelled exosomes localized to proximal tubule and glomeruli 30 min after injection. Exosomes expressed CXCR4 by immunoblot, and SDF-1-α secretion was upregulated in hypoxic HUVECs. In HUVECs, incubation with blocking antibody against SDF-1α or the CXCR4 inhibitor Plerixafor significantly inhibited uptake of PKH-26 labelled exosomes. In hypoxic endothelial cells, PKH-26 labelled exosome uptake increased by 35%, and this was significantly inhibited by Plerixafor.

**Summary/Conclusion**: ECFC exosomes selectively target the kidneys after ischaemia–reperfusion AKI, with rapid transfer of miR-486-5p that persists at 24 h. Our results suggest that targeting of exosomes in AKI may be mediated by interaction of exosomal CXCR4 with endothelial cell SDF-1α. These data provide further support for the promising therapeutic potential of ECFC exosomes in human AKI.

**Funding**: This study was funded by Kidney Foundation of Canada and Canadian Institutes of Health Research.

PS03.09

A simple method to label vesicles for visualization and *in vivo* tracking

Edison Salas-Huenuleo ^1^; Iva Polakovicova^2^; Manuel Varas-Godoy^3^; Lorena Lobos-González^4^; Julian Bejarano^1^; Alejandro Corvalán^5^; Marcelo J. Kogan^1^

^1^Laboratory of Nanobiotechnology and Nanotoxicology, Facultad de Ciencias Químicas y Farmacéuticas, Universidad de Chile, Santiago, Chile; ^2^Laboratory of Oncology, Faculty of Medicine, Pontifícia Universidad Católica de Chile; Center for Chronic Diseases, Pontifícia Universidad Católica de Chile, Santiago, Chile; ^3^Centro de Investigación Biomédica, Faculty of Medicine, Universidad de Los Andes, Santiago, Chile; ^4^Fundación Ciencia y Vida, Andes Biotechnologies, Santiago, Chile; ^5^Laboratory of Oncology, Faculty of Medicine, Pontifícia Universidad Católica de Chile; Advanced Center for Chronic Diseases, Pontifícia Universidad Católica de Chile, Santiago, Chile

**Background**: Labelling of vesicles for their visualization *in vitro* or *in vivo*, involves the use of fluorescent dyes. To obtain labelled vesicles free of unincorporated dye, purification steps are necessary. The standard method is density gradient ultracentrifugation which is not only time consuming, but counts with high sample loss and requires expensive equipment. Here, we established a simple and fast method to acquire labelled vesicles for *in vivo* tracking and visualization.

**Methods**: Extracellular vesicles (EVs) from cell culture supernatant, synthetic exoliposomes (ELIP) and thermosensitive liposomes (TLIP) were obtained and characterized by nanosight, transmission electron microscopy and zeta potential determinations. Subsequently, the nanostructures were incubated with DiR fluorophore. DiR-labelled vesicles were purified by two different methods, using optiprep density gradient ultracentrifugation or commercial exo-spin columns. The eluates obtained from columns and density gradient fractions were characterized by nanosight, dynamic light scattering, zeta potential, protein content, fluorescence spectroscopy and imaging. Obtained yields of labelled vesicles were compared. Next, purified labelled EVs, ELIP and TLIP were administrated via tail vein injection in mice with an equivalent number of particles and visualized at 48 h using In Vivo imaging system. Organs were extracted, visualized and fluorescence intensity was measured. All animal procedures and care were approved by implicated ethic committees.

**Results**: Using exo-spin column, DiR labelled EVs, ELIP and TLIP were obtained. Profound characterization of every step, column and eluate during the process showed that free DiR was not present in labelled samples. Next, we established that the use of column provides reproducible results with low sample loss. The working time is less than 10 min, significantly less than up to 24 h of the density gradient method. Finally, we used these labelled vesicles to determine and compare their biodistribution in organs of mice.

**Summary/Conclusion**: We compared two methods and established the use of exo-spin column as a tool to obtain labelled vesicles in a reproducible, simple and faster manner, with no need of expensive equipment.

**Funding**: This study was funded by FONDECYT 3160592, 11140204, 11150624, 3160323, 1151411, 11140204, and FONDAP 15130011.

PS03.10

Circulating exosomes as delivery mechanism of free fatty acids (cFFA)

Elena Grueso^1^; Nahuel Aquiles. García^2^; Akaitz Dorronsoro González^1^; Hernan González-King^1^; Rafael Sánchez^1^; Alicia Martínez^3^; Beatriz Jávega^4^; Enrique O’Connor^3^; Jose Anastasio Montero^1^; Pilar Sepúlveda ^1^

^1^Instituto de Investigación Sanitaria La Fe., Valencia, Spain; ^2^Cedars-Sinai, La Jolla, USA; ^3^Centro de Investigación Príncipe Felipe, Valencia, Spain; ^4^Universidad de Valencia., Valencia, Spain

**Background**: Circulating free fatty acids (cFFA) are involved in different human diseases such as diabetes, atherosclerosis and metabolic syndrome, although the exact role of cFFA in each disease needs to be clarified. In this context, we studied how circulating exosomes function as cargo vesicles for the transportation of cFFA from blood to target tissues. Exosomes are small membrane vesicles (30–100 nm) formed by reverse budding in the cytoplasm and secreted by a large variety of cells. These nanovesicles participate in the intercellular communication by delivering a large variety of bioactive molecules among tissues.

**Methods**: Serum from healthy donors was obtained before (PRE) and 20 min after (POST) a high caloric breakfast. Circulating exosomes (cExo) were purified by ultracentrifugation and characterized by Nanosight, SEM and detection of tetraspanins. Using Western blot we studied the levels of platelet glycoprotein 4 (CD36) in the isolated cExo. The content of lipids and the ability of cExo to uptake cFFA were measured using Red Nile dye and BODIPY® 500/510.

**Results**: POST cExo showed higher levels of CD36 compare to PRE cExo. Using Nile Red we demonstrated that POST cExo have higher levels of lipids compare to PRE cExo, correlating with CD36 levels. CD36 has an important role in cFFA uptake by cells. Using BODIPY® 500/510 we demonstrate that cExo are capable of incorporating cFFA and that CD36 has an active role in this process. Furthermore, we also observed that cExo are able to deliver cFFA to human cardiac microvascular endothelial cells and cardiomyocytes.

**Summary/Conclusion**: Taken together, our results shed light on the role of cFFA in metabolic pathologies. Our results indicate that circulating exosomes are able to actively incorporate free fatty acids by CD36 and deliver them to target tissues.

**Funding**: This study was funded by ISCIII: PI16/00107, RD16/0011/0004.

PS03.11

New role of alpha-2-macroglobulin into the shedding of microvesicles

Alexandra Laberge^1^; Akram Ayoub^1^; Syrine Arif^1^; Sebastien Larochelle^1^; Alain Garnier^2^; Veronique J. Moulin ^2^

^1^Centre de recherche d’Organogénèse Expérimental de l’Université Laval/LOEX, Québec, Canada; ^2^Université Laval, Quebec, Canada

**Background**: Cells release membranous structures known as microvesicles (MVs) that play an important role in tissue morphogenesis and wound healing. Myofibroblasts are cells present in healing tissue that produce new extracellular matrix, stimulate angiogenesis and contract wound edges. They have been shown to shed MVs upon stimulation with serum or plasma. However, the exact molecule that induces MV production is unknown.

**Methods**: A succession of chromatography, electrophoresis and mass spectrometry methods was performed on serum to identify the molecule that stimulates MV formation. Production of MVs by myofibroblasts was measured after each step of the purification sequence and after stimulation with two potent molecules.

**Results**: Among the numerous proteins present in serum, alpha-2-macroglobulin (A2M) was found to stimulate the production of MVs in a dose-dependent manner. We showed that low-density lipoprotein receptor-related protein 1 (LRP1), an A2M receptor, is expressed on the surface of myofibroblasts. Addition of inhibitors of A2M-LRP1 binding decreased the production of MVs by myofibroblasts.

**Summary/Conclusion**: Stimulation of the shedding of MVs from myofibroblasts during wound healing is a novel function of A2M.

**Funding**: This study was funded by Natural Sciences and Engineering Research Council.

PS03.12

Bioavailability of bovine milk extracellular vesicles

Maria S. Hansen; Kristine I. M. Blans; Jan T. Rasmussen

Molecular Biology and Genetics, Aarhus University, Aarhus, Denmark

**Background**: Milk extracellular vesicles (MEVs) are a novel class of milk bioactives, which most likely are resistant to the digestive system after consumption. Around the world, people drink milk from many animals such as cow, camel, goat and sheep, with cow’s milk being the most consumed type. As bovine milk is a rich source of MEVs, it is in our interest to investigate the biological potential of bovine MEVs. We have developed a protocol to obtain a pure MEV fraction from raw, untreated milk, validated, e.g. by the presence of well-described EV markers and absence of major milk contaminants such as casein and milk fat globules. In order to obtain more knowledge about the bioavailability of MEVs, we have investigated factors affecting *in vitro* uptake of MEVs in intestinal epithelium. Additionally, MEVs from processed milk have been isolated and compared to MEVs from unprocessed milk.

**Methods**: MEVs from bovine milk were gently purified by size exclusion chromatography after an initial centrifugation step to remove milk fat and milk cells. For *in vitro* cell studies, isolated MEVs were specifically labelled with lactadherin marked with a fluorophore. Cellular uptake of MEVs was evaluated quantitatively by measuring total fluorescence on lysed cells.

**Results**: Bovine MEVs were successfully labelled with fluorescent lactadherin. Quantitative measurements of cellular uptake of MEVs after incubation confirmed that MEVs are definitely internalized. Moreover, the investigations revealed that this uptake is time and temperature dependent. Several interventions were tested and evaluated in regard to cellular uptake. These include MEV concentration, temperature, simulated intestinal digestion conditions and the employment of different intestinal epithelial cell lines.

**Summary/Conclusion**: A specific and non-invasive fluorescent labelling method was proven suitable to investigate bovine MEV uptake by different intestinal epithelial cell lines. *In vitro* cellular internalization of MEVs was confirmed, which indicates that MEVs definitely have the potential to convey bioactivity.

**Funding**: This study was funded by Aarhus University and Arla Foods.

PS04: Novel Developments in EV Isolation Chairs: Tom Driedonks; Louise Laurent Location: Exhibit Hall 17:15–18:30

PS04.01

Systematic evaluation of techniques for the isolation and detection of small non-coding RNA from urine-derived extracellular vesicles

Elena S. Martens-Uzunova; Natasja Dits; Mirella Vredenbregt-van den Berg; Guido W. Jenster

Erasmus Medical Center, Rotterdam, The Netherlands

**Background**: The ability to stratify prostate cancer patients in a non-invasive manner, into these who benefit from radical treatment versus these who can be enrolled in an active surveillance or watchful waiting programme, would answer a currently unmet clinical need. A promising solution to this clinical problem is the use of the minimally invasive “liquid biopsy” approach that aims at the detection of tumour biomarkers in blood or urine.

Over the last years, extracellular vesicles (EVs) emerged as a novel promising source of cancer-related biomarkers. Tumour cell originating EVs can be used as a source of protein and RNA biomarkers.

**Methods**: We evaluated available methods for the extraction and quantitation of small RNAs present in urinary EVs in order to examine their use as minimally invasive PCa biomarkers. We tested 11 different combinations of direct and stepwise methods for EV isolation and RNA extraction and quantitated the content of previously established by using small RNAs with high biomarker potential in PCa by two different qPCR techniques.

**Results**: To obtain high amounts of uniform quality starting material, urine samples from healthy donors were depleted from native EVs by ultracentrifugation protocol and spiked in with known amount of EVs isolated from prostate cancer cells. The amount of spiked EVs was equivalent to the amount of removed vesicles. Subsequently, EVs were captured by four different techniques, i.e. ultrafiltration, precipitation, size exclusion chromatography and affinity capture. Total RNA was isolated either directly from the captured EVs or after EV recovery using two different kits, with or without phenol–chloroform extraction. The amounts of small RNAs (miRNAs, isoMiRs, tRNA fragments, snoRNA and snoRNA fragments) were measured by quantitative real-time PCR (qPCR) either with a SyBR Green technique and LNA-based primers or with a probe-based Taq-Man technique.

**Summary/Conclusion**: Direct, non-organic RNA extraction proved superior to stepwise, phenol–chloroform based techniques in terms of small RNA quantitation. All tested types of small RNAs were successfully detected by qPCR.

**Funding**: This study was funded by IMMPROVE consortium (Innovative Measurements and Markers for Prostate Cancer Diagnosis and Prognosis using Extracellular Vesicles) sponsored by Dutch Cancer Society, Alpe d’HuZes grant: EMCR2015-8022.

PS04.02

Repeatable, high-purity isolation of urinary extracellular vesicles for uro-oncological biomarker studies

Bert Dhondt ^1^; Glenn Vergauwen^2^; Jan Van Deun^2^; Edward Geeurickx^1^; Joeri Tulkens^1^; Lien Lippens^1^; Ikka Miinalainen^3^; Pekka Rappu^4^; Jyrki Heino^3^; Nicolaas Lumen^4^; Olivier De Wever^5^; An Hendrix^2^

^1^Laboratory of Experimental Cancer Research, Department of Radiation Oncology and Experimental Cancer Research, Faculty of medicine and health sciences, Ghent University, Ghent, Belgium; ^2^Laboratory of Experimental Cancer Research, Department of Radiation Oncology and Experimental Cancer Research, Cancer Research Institute Ghent (CRIG), Ghent University, Ghent, Belgium, Ghent, Belgium; ^3^Biocenter Oulu, Department of Pathology, Oulu University Hospital, University of Oulu, Oulu, Finland; ^4^University of Turku, Department of Biochemistry, Turku, Finland; ^7^Department of Urology, Ghent University Hospital, Ghent, Belgium; ^5^Laboratory of Experimental Cancer Research, Department of Radiation Oncology and Experimental Cancer Research, Cancer Research Institute Ghent (CRIG), Ghent University, Ghent, Belgium

**Background**: Urinary extracellular vesicles (uEV) have raised interest as a potential source of biomarker discovery. Contaminants such as Tamm-Horsfall protein (THP) polymers hinder accurate downstream analysis by masking low abundance proteins or by entrapping non-EV associated extracellular RNA molecules.

**Methods**: Cell-free urine samples from prostate cancer patients were concentrated by ultrafiltration. uEV were isolated using a bottom-up discontinuous Optiprep™ density gradient (ODG) in six technical replicates and characterized by nanoparticle tracking analysis (NTA), transmission electron Microscopy (TEM) and unbiased proteomic analysis (LC–MS/MS).

**Results**: NTA and TEM confirmed the enrichment of 100 nm uEV in density fractions of approximately 1.1 g/ml (EV-rich fractions) and THP contaminants in the high density fractions. Unbiased mass spectrometry-based proteomics identified consistent and biologically relevant EV-associated proteins with high repeatability as analysed by principle component analysis and hierarchical clustering. Volcano plot analysis showed a clear differential protein enrichment between EV rich density fractions and THP high density fractions and gene set enrichment analysis of these proteins demonstrated differential biological functions and cellular origin.

**Summary/Conclusion**: Bottom-up ODG results in an efficient and reproducible separation of uEV from THP, a first step towards biomarker discovery in genitourinary cancer patients.

**Funding**: This study was funded by Kom op tegen Kanker (Stand up to Cancer), the Flemisch Cancer Society.

PS04.03 = OWP2.03

Microscale electrophoretic separations of exosomes

PS04.04

Urinary extracellular vesicles in diabetic kidney disease: validation of preferable preparative techniques

Karina A. Barreiro ^1^; Om P. Dwivedi^1^; Maija Puhka^1^; Carol Forsblom^2^; Leif Groop^1^; Tobias Huber^3^; Harry Holthöfer ^1^

^1^Institute for Molecular Medicine Finland FIMM, University of Helsinki, Finland, Helsinki, Finland; ^2^Folkhälsan Institute of Genetics, Folkhälsan Research Center, Helsinki, Finland, Helsinki, Finland; ^3^III. Medizinische Klinik, Universitätsklinikum Hamburg-Eppendorf, Hamburg, Germany, Hamburg, Germany

**Background**: We compared performance of different methods for urinary extracellular vesicle (uEV) harvest and the respective transcriptome yields for biomarker identification in diabetic kidney disease (DKD).

**Methods**: Type 1 diabetic (T1D) patients and normal controls were included in the study. uEVs were isolated from 20 to 40 ml of 24 h urine collection by ultracentrifugation (UC), hydrostatic filtration dialysis (HFD) or kit-based isolation (KI). Quality of uEV yield was analysed with EM and Western blotting (WB). Isolated RNAs were profiled with Bioanalyzer Pico kit and subjected to RNAseq using HiSeq 2000 (Illumina) pair-end (2 × 100) protocol. Output reads were aligned to human reference genome and counted using GENCODE annotations. We used gene length normalized values FKPM (fragments per kilobase of exon per million) as expression measurement for genes.

**Results**: The isolated uEVs appeared typical at EM and were positive for CD9 and kidney-derived podocalyxin in WB. The size distribution of uEVs (by NTA) was similar in HFD and UC while KI samples were enriched in smaller vesicles (up to 300 nm).The RNA yield was slightly higher in UC and KI samples while sufficient for RNAseq in all. The number of reads for KI samples was lower and the intron content higher than in UC or HFD. For UC samples, we detected (FKPM >1) average of 13,161 genes and high expression (FPKM ≥5) of kidney specific genes (SLC12A3, SLC12A1, LGALS1, ATP6V1B1, NPHS2, AQP3, AQP2, SLC22A12). Full analysis of 182 kidney specific genes showed >70% (total 132) of the genes in uEVs. Principal component analysis of these distinguished macro-albuminuric from normoalbuminuric T1D patients. Six genes were differentially expressed in DKD (Puncorrected <0.001 and fold change >1.5 or <0.66). The highest expressed genes in EVs (*N* = 5153, FKPM ≥10) were enriched (*P* > 10–11) in pathways of cellular metabolism (oxidative phosphorylation and TCA cycle), mitochondrial, vesicle trafficking and ribosome functions. Pathway and gene enrichment analyses (*P* < 0.05, *N* = 956) differentially expressed genes implicated (*P* < 0.002) TGF-beta and PI3K-Akt signalling as well as immune pathways in DKD.

**Summary/Conclusion**: We show that uEV transcriptome captures the kidney specific transcriptome and differentiates T1D patients from controls while full method standardization is needed.

PS04.05

Isolation of intact extracellular vesicles (EVs) and comparison of EVs isolated from urine and plasma

Hyun-Kyung Woo ^1^; Juhee Park^2^; Vijaya Sunkara^1^; Yoon-Kyoung Cho^2^

^1^Ulsan National Institute of Science and Technology (UNIST), Ulsan, Republic of Korea; ^2^Center for Soft and Living Matter, Institute for Basic Science (IBS), Ulsan, Republic of Korea

**Background**: Extracellular vesicles (EVs) are cell-derived vesicles in the range of 40–1000 nm, and potential source of cancer diagnostic biomarkers and therapeutic agents [1]. It could be found in almost all types of body fluids such as blood, urine, cerebrospinal fluid, ascites and so on. Despite the increasing importance of EVs as an important clinical biomarker, the isolation and analysis method remains the main impediment to be adapted as a routine clinical test [2]. We developed a facile method, “Exodisc”, to isolate intact extracellular vesicles from urine using a centrifugal microfluidic device [3]. Here, we would like to discuss the correlation of urinary EVs prepared on a disc with blood-derived EVs.

**Methods**: The device is consisted of three polycarbonate (PC) layers and laminated with two pressure-sensitive, double-sided adhesives. On the device, two types of membranes are inserted; track-etched PC membrane (600 nm pore size) and AAO membrane (20 nm pore size) as filter I and II respectively. 1 mL of raw urine sample is injected in the sample chamber and large debris are precipitated (~300×*g*). By controlling valves, clear supernatant flow through two filters by concentrating EVs on the filter II. Finally, EVs are eluted in PBS after two times of washing steps. To isolate plasma EVs, ultracentrifugation (150,000×*g*, 90 min) is used with subsequent washing step (150,000×*g*, 90 min).

**Results**: Isolation of intact EVs could be achieved within 30 min starting from raw urine samples of prostate cancer patients and healthy donors, which results ~4 times higher number of EVs compared to that prepared by ultracentrifugation (UC) method. Compared to plasma-driven EVs prepared by UC, the urinary EVs were smaller in number of particles, however, bigger in size and higher in the amounts of RNAs and miRNAs.

**Summary/Conclusion**: The “Exodisc” provides rapid isolation of intact EVs from urine samples with higher recovery compared to conventional UC methods. The characterization and comparison of EVs isolated from other types of body fluids may synergistically contribute to liquid biopsy of cancer.

PS04.06

A path to ultra-low input microRNA sequencing from urinary extracellular vesicles after acoustic trap enrichment

Anson T. Ku ^1^; Mikael Evander^2^; Margareta Persson^1^; Hans Lilja^3^; Thomas Laurell^4^; Yvonne Ceder^1^

^1^Lund University, Lund, Sweden; ^2^Acousort, Lund University, Lund, Sweden; ^3^Lund University, Memorial Sloan Kettering, Oxford University, Lund, Sweden; ^4^Lund University, University of Tokyo, Dongguk University, Lund, Sweden

**Background**: There are increasing recognition that microRNA (miRNA) contained in extracellular vesicles (EVs) play a pivotal role in disease progression. The challenge to use miRNA in EVs as a biomarker has been hampered by a lack of a robust method to enrich and sequence miRNA from minute quantities of initial samples. Utilizing the acoustic trap, which is a novel microfluidic technology that utilizes ultrasonic waves to enrich extracellular vesicles, we enriched urinary EVs in a contact-free and automated manner. Next, we compared the performance of two different small RNA library preparations using 130 pg of input RNA derived from urinary EVs. In addition, we compared the miRNA obtained from acoustic trap to ultracentrifugation to determine the performance of the acoustic trap method.

**Methods**: Urinary extracellular vesicles were enriched from approximately 2.5 mL of urine by acoustic trap and ultracentrifugation follow by RNase A treatment. Total RNA was extracted using Single Cell RNA extraction kit (Norgen) and approximately 130 pg of RNA was used for library construction using the small RNA library preparation kits, NEXTFlex (Perkin Elmers) and CATs (Diagenode). Specifically, two library replicates were constructed from acoustic trapped sample and one from the ultracentrifugation enriched sample. The library profiles were confirmed by Bioanalyzer and Qubit DNA assay and sequenced on an Illumina NextSeq platform. The miRNA expression of three miRNAs, has-miR-16, 21, and 24, was validated using qRT-PCR.

**Results**: Small RNA libraries were successfully constructed from 130 pg of RNA derived from acoustic trap and ultracentrifugation method using both NEXTFlex and CATS small RNA library preparation kits. Three different miRNAs were used to validate the finding by qRT-PCR.

**Summary/Conclusion**: Acoustic trap enrichment of urinary EVs can produce sufficient quantities of RNA for miRNA sequencing using either NEXTFlex or CATS small RNA library preparation.

**Funding**: This study was funded by Swedish Foundation for Strategic Research, Swedish Research Council (2014-03413, 621-2014-6273 and VR-MH 2016-02974), Knut and Alice Wallenberg Foundation (621-2014-6273), Cancerfonden (14-0722 and 2016/779), NIH (P30 CA008748), Prostate Cancer Foundation, and NIHR Oxford Biomedical Research Centre Program in UK. Stefan Scheding is a fellow of the Swedish Cancer Foundation.

PS04.07

EV-TRACK: evaluation, updates and future plans

Jan Van Deun; Olivier De Wever; An Hendrix

^1^Laboratory of Experimental Cancer Research, Department of Radiation Oncology and Experimental Cancer Research, Cancer Research Institute Ghent (CRIG), Ghent University, Ghent, Belgium

**Background**: Transparent reporting is a prerequisite to facilitate interpretation and replication of extracellular vesicle (EV) experiments. In March 2017, the EV-TRACK consortium launched a resource to improve the rigour and interpretation of experiments, record the evolution of EV research and create a dialogue with researchers about experimental parameters.

**Methods**: The EV-TRACK database is accessible at http://evtrack.org, allowing online deposition of EV experiments by authors pre- or post-publication of their manuscripts. Submitted data are checked by EV-TRACK admins and an EV-METRIC is calculated, which is a measure for the completeness of reporting of information necessary to interpret and repeat an EV experiment. When the EV-METRIC is obtained at the preprint stage, it can be implemented by authors, reviewers and editors to help evaluate scientific rigour of the manuscript.

**Results**: Between March 2017 and January 2018, data on 150 experiments (unpublished: 49%; published: 51%) were submitted by 74 unique users. The average EV-METRIC for all experiments was 43% (unpublished: 57%; published: 31%).

For seven experiments, authors added additional data that were not included in the original article specifically to the corresponding EV-TRACK entry, as a way to improve the transparency of their experiments. These data included isolation protocol details such as ultracentrifugation rotor type, and antibody clone and dilution that were used in immunoassays.

Based on user comments, a new submission system was developed. The new system is more complete, including additional questions on vesicular DNA analysis, size-exclusion chromatography and flow cytometry. It has also been modified to make it more intuitive and user-friendly.

The usability of the EV-TRACK platform as a database is being improved, allowing more versatile search options and easier download of data. Additionally, the database will be completed by adding data of EV-related papers published in 2015, 2016 and 2017 that are not yet included.

**Summary/Conclusion**: The EV-TRACK knowledgebase is a unique resource devoted to EV research, with a focus on transparent reporting of experimental data. Through constant evaluation and interaction with its users, new developments will ensure that the platform remains up-to-date, user-friendly and relevant.

PS04.08

Antibody aggregates: a potential pitfall in the search of rare EV-populations

Rikke W. Rasmussen ^1^; Frederik Prip^2^; Mathilde Sanden^3^; Morten Hjuler Nielsen^4^; Jaco Botha^5^; Aase Handberg^6^

^1^Department of Clinical Biochemistry, Aalborg University Hospital, Aalborg, Denmark, Hornslet, Denmark; ^2^Department of Clinical Biochemistry, Aalborg University Hospital, Aalborg, Denmark, Aarhus, Denmark; ^3^Department of Clinical Biochemistry, Aalborg University Hospital, Aalborg, Denmark; ^4^Department of Clinical Biochemistry, Aalborg University Hospital, Aalborg, Denmark, Lystrup, Denmark; ^5^Department of Clinical Biochemistry, Aalborg University Hospital, Aalborg, Denmark, Dronninglund, Denmark; ^6^Department of Clinical Biochemistry, Aalborg University Hospital, Aalborg, Denmark, Risskov, Denmark

**Background**: Recently, small-particle flow cytometers have become available with sufficient sensitivity and resolution for characterization of extracellular vesicles (EVs). Although increased sensitivity holds great potential for characterization of the bulk of EVs, it has also led to introduction of new artefacts. Commercially available protein-based labels including antibodies have previously been demonstrated to contain varying amounts of aggregates, which could potentially interfere with interpretation of results. We present data demonstrating the extent of this issue, and that its extent can be limited by high-speed centrifugation prior to use.

**Methods**: Flow cytometry was performed on an Apogee A60 Micro-PLUS flow cytometer. Platelet-poor plasma (PPP) and PBS were labelled with lactadherin-FITC, anti-CD36-PE, anti-CD62E-APC and anti-ICAM-AF700, or with lactadherin-FITC and corresponding isotype controls. All labels were either untreated or centrifuged at 17,000 g for either 5 or 30 min before labelling. EVs were defined as phosphatidylserine-exposing (PS+) events ≤1000 nm.

**Results**: Initially, we compared the concentrations of different labels in PBS in order to study the presence of aggregates. Aggregates were present for all of the investigated protein labels/antibodies, and large variability was observed between different labels and antibodies and their respective isotype controls (0.44–391 events/µl). By comparing PPP and PBS, aggregates constituted differing proportions of measured positive events using a light scatter threshold (5.0–60.7%). Interestingly, larger proportions were observed when applying a combined light scatter and fluorescence threshold (7.0–112%). High-speed centrifugation for 5 min effectively reduced the amount of aggregates in PBS (0–138 events/µl), while 30 min of centrifugation reduced aggregates to an even greater extent (0–41.8 events/µl).

**Summary/Conclusion**: Antibody aggregates generating false positive results in the characterization of EVs is an issue that the EV community should be aware of. Aggregates may potentially lead to false conclusions and could in particular have an impact on the characterization of rare EV phenotypes. Thus, the authors recommend employing high-speed centrifugation of protein-based labels before use and implementing routines to control for aggregates.

PS04.09

Isolation of clinical grade exosomes by a two-step FPLC purification method

Kristina Lang; Robert Steinfeld

Department for Child and Adolescent Health, University Medical Center Goettingen, Goettingen, Germany

**Background**: Exosomes are due to their unique characteristics in size, stability and functionality predestined to be applied as drug delivery vehicles. Their cargo is protected by a double membrane and their transport is directed depending on their membrane composition. Unfortunately most methods for exosome preparations are based on simple separation by size and density. Since other extracellular vesicles have similar properties and are copurified. To improve the quality of exosomal preparations to enable their clinical application we developed a two-step FPLC-method to prepare biologically active, highly pure exosomes in a large scale.

**Methods**: This innovative purification method is based on the specific tagging of any exosome surface-protein. The material is first purified by a size exclusion chromatography removing smaller particles. Followed by immobilized metal affinity chromatography which is specifically retaining exosomes with the tagged protein. These vesicle preparations were fully characterized in size, density, by their protein markers and their morphologic properties.

**Results**: In Western blot analyses we could show a reduction of extracellular and intercellular contaminations below 10% in comparison to the raw material, while exosome-enriched markers as flotillin-2 and alix were retained with 26% and 30%. In contrast, preparations from a standard ultracentrifugation protocol had markers of contaminating proteins twice as high. The particle-per-1 µg-protein ratio of our exosome preparations is higher than 4e109, being an indicator for a high purity. With scanning electron microscopy using gold-coating a cup-shaped morphology of the vesicles could be shown. A mean size of the particles of 166.3 ± 17.5 nm was determined by nanoparticle tracking analyses.

**Summary/Conclusion**: Using our method biologically active exosomes with a high purity can be purified in a large scale. This way the therapeutic application of exosomes as drug delivery vehicles is becoming more realistic.

**Funding**: This study was funded by Ministry of Science and Culture of Lower Saxony and the VW foundation.

PS04.10

Sequencing and reproducibility analyses of small RNA extracted from prostate cancer exosomes isolated using nanoDLD chip technology

Navneet Dogra ^1^; Gustavo Stolovitzky^2^; Stacey Gifford^3^; Carlos Cordon^4^; Ashutosh Tewari^4^

^1^Icahn School of Medicine at Mount Sinai, New York City, USA; ^2^IBM/Icahn School of Medicine at Mt. Sinai, New York, USA; ^3^IBM, New York, USA; ^4^Icahn School of Medicine at Mt. Sinai, New York, USA

**Background**: Exosomes are an exciting target for liquid biopsy-based cancer diagnostics. However, isolation of pure exosomes is an ongoing challenge for the extracellular vesicle community. Multiple studies have shown that exosomes and their nucleic acid and protein content are dependent upon the specific method used for isolation. Hence, there is a need to establish methods and tools for reproducible isolation of exosomes.

**Methods**: We have developed a nanoscale Deterministic Lateral Displacement (nanoDLD) lab-on-a-chip technology for size based separation of exosomes. The chips are fabricated using CMOS compatible – and thus manufacturable – technology and consist of pillar arrays where nanofluidics flow patterns sort exosomes from larger and smaller components. We have isolated prostate cancer cell culture supernatant and prostate cancer patient urine samples and used the nanoDLD chip and ultracentrifugation to extract exosomes from these samples.

Furthermore, we have used SMARTer smRNA-Seq Kit for library preparation and Hiseq2500 at New York Genome Center (NYGC) for small RNA sequencing.

**Results**: We demonstrate size-based separation of exosomes from cell culture and urine samples, and sequencing of their small RNA cargo. We performed reproducibility studies of RNA transcripts isolated through nanoDLD chip and with traditional exosome isolation methods (UC). We compare smRNAseq studies of exosomes isolated from human prostate cancer tissues and patient samples.

**Summary/Conclusion**: These preliminary results indicate the potential of our nanoDLD chip technology for isolating exosomes for the detection of exosome biomarkers from cell culture media and patient samples.

PS04.11

Novel AC electrokinetic platform for rapid isolation and characterization of extracellular vesicles from NSCLC patients

Juan P. Hinestrosa ^1^; David Searson^1^; Delia Ye^1^; Robert Kovelman^1^; James Madsen^1^; Robert Turner^1^; David Bodkin^2^; Rajaram Krishnan^1^

^1^Biological Dynamics, San Diego, USA; ^2^Cancer Center Medical Oncology Group, La Mesa, USA

**Background**: Extracellular vesicles (EVs) contain proteomic and genomic information that can be used for cancer diagnosis and treatment response monitoring. Currently the time and equipment needed for EV isolation and characterization limit their use as diagnostic targets. In this work, a novel AC electrokinetic (ACE) platform for the isolation and characterization of membrane-bound programmed death ligand-1 (PD-L1) positive EVs from NSCLC patients.

**Methods**: The ACE platform consists of a microelectrode array that selectively isolates nanoparticles with diameters of 40–800 nm directly from physiological fluids. EV isolation and antibody staining using the platform ACE required less than 2 h to complete. EVs isolated by ultracentrifugation from the pancreatic cancer cell line ASPC-1 were used to validate the ACE platform’s performance. Subsequently, EVs were isolated and PD-L1 levels analysed from 10 NSCLC patient and 10 healthy donor plasma samples. These samples were collected via approved IRB protocols.

**Results**: EVs from pancreatic cancer ASPC-1 cells were detected using an anti-CD63 antibody and an antibody to the pancreatic cancer specific Glypican-1, confirming that the ACE platform could isolate EVs and identify membrane-bound proteins. Recovered EVs were isolated and EV-associated RNA was amplified by RT-PCR. EVs from isolated from NSCLC patient plasma on the ACE platform contained higher levels of PD-L1 compared to healthy individuals (*p* < 0.001). In parallel on the ACE platform, circulating cell-free DNA (ccf-DNA) was isolated and quantified from the same samples, with increased levels measured in cancer patients.

**Summary/Conclusion**: ACE is a novel platform for the isolation and detection of membrane-bound protein markers on EVs in less than 2 h. Positive identification of PD-L1 was achieved on the isolated EVs from NSCLC patients. This same platform can be used to isolate and characterize RNA that is carried by exosomes and measure ccfDNA using a single patient sample. Thus, the ACE platform has the potential to become a multimodal analytics tool for cancer diagnostics and treatment monitoring.

**Funding**: This study was funded by Biological Dynamics.

PS04.12

EVs isolation by SMART-SEC: analysis of isolated contaminants and fluorescent labelled EVs

Esperanza Gonzalez ^1^; Juan M. Falcón-Pérez^2^

^1^CIC bioGUNE, Derio, Spain; ^2^CIC bioGUNE, CIBERehd, Bizkaia Science and Technology Park, Derio, Bizkaia, Spain, Derio, Spain

**Background**: Size exclusion chromatography or SEC has become the gold standard for EVs purification, even unseating the traditional differential ultracentrifugation (UC). Many works have already proved that this approach preserves shape, integrity and functionality of EVs and is applicable to any sample, ranging from conditioned media to body fluids. In this study, we aimed to develop an easy going and cost effective hand-made size exclusion chromatography (SEC) that we have named SMART (**S**killed **M**inimalist **ART**ificer). This system has been used to analyse typical EVs contaminants, purified EVs and conditioned media, and fluorescent labelling.

**Methods**: EVs previously purified by UC from hepatic carcinoma SK-Hep1 cells and concentrated conditioned media from fluorescence-labelled SK-Hep1 cells were separated in SMART columns. Additionally, EVs contaminants represented in two cocktails comprising Apoferritin, Albumin, Cytochrome C and IgG or HDL, LDL and VLDL were also separated. The SEC performance was examined and evaluated by means of Western and Dot blots, protein quantification, Nanoparticle Tracking Analysis and Electron Microscopy.

**Results**: In SMART-SEC fractioning, CD81 marks two separated EVs populations. However, others EVs markers such as CD63, TSG101, Flotilin-1 or Hsp70 show a peak corresponding with the biggest CD81 positive EVs, whereas contaminants overlap to a greater degree with the smallest CD81 population. In particular, lipoproteins cannot be perfectly separated from EVs. Finally, fluorescent EVs coming from labelled SK-Hep1 cells map overlapping the smallest CD81 positive EVs population.

**Summary/Conclusion**: SMART-SEC brings comparable separation respect to previous formats in terms of fractioning of EVs markers and contaminants, as well as reproducibility. SMART columns separate at least two different populations of CD81 positive EVs, being the one containing the biggest EVs better fractionated from contaminants, whereas the one covering the smallest size greatly overlap with contaminants and fluorescent species. SMART consist of a SEC mini-format that allows for improved management of a certain number of samples in the same set and is highly valuable for diary use and general applications.

**Funding**: This study was funded by Ramón Areces, MINECO and CIBER Instituto de Salud Carlos III.

PS04.13

Capture and label-free detection extracellular vesicles on gold-nano-island based microfluidic Lab-on-a-CHIP device using synthetic-peptide Vn96

Anirban Ghosh ^1^; Srinivas Bathini^2^; Duraichelvan Raju^2^; Simona Badilescu^2^; Awanit Kumar^3^; Muthukumaran Packirisamy^2^; Rodney J. Ouellette^4^

^1^Adjunct professor at the Department of chemistry and biochemistry at Université de Moncton, Moncton, Canada; ^2^Concordia University, Montreal, Canada; ^3^Atlantic Cancer Research Institute, Moncton, Canada; ^4^Atlantic Cancer research Institute, Moncton, Canada

**Background**: Given the tremendous potential of circulating extracellular vesicles (EVs) for liquid-biopsy, there is great demand for simple, robust and clinically adaptable EV isolation and characterization Lab-on-a-CHIP (LOC) platforms. Towards this, LOCs have been developed for capture, quantification and characterization of circulating EVs using EV-surface specific antibodies. The detection was performed either using fluorescent or label-free surface plasmon-resonance (SPR) sensors. The antibody-based isolation faces many challenges of quality control and shelf-life. To address the need for better affinity-based EV isolation method, we used a next generation affinity-based EV capture technology that uses a synthetic peptide (Vn96). Our group developed a LOC to capture EVs using Vn96, grafted onto gold nano-island (GNI) based on LSPR (localized SPR) sensing platform, and thus contributing to the emerging field of plasmofluidics.

**Methods**: The LOC was built as: deposition of gold-nano-particle (GNP) on the glass surface and annealing of those deposited GNP to form GNI, bonding of PDMS onto the GNI and simultaneous LSPR in each spectrum. We have used scanning electron microscopy, atomic force microscopy, tunable resistive pulse sensing to count enriched EVs on LOC and relevant molecular analysis.

**Results**: We designed, simulated and fabricated LOCs to identify the best microfluidic channel design on PDMS which were bonded on to a glass surface containing GNI grafted with Vn96-peptide using chemistry to covalently attach streptavidin onto the GNI followed by attachment biotinylated Vn96. At each steps of tagging streptavidin to affinity attachment of EV onto Vn96 was quantitated using LSPR to identify parameters for the best efficiency. Our results demonstrated that Vn96-grafted LOC enriched EVs as a function of red-shift in the pick-LSPR spectra and was further characterized by eluting the attached EV from LOC for counting, imaging and molecular characterization.

**Summary/Conclusion**: Our results demonstrate that Vn96-based affinity enrichment of EVs can be adapted on plasmofluidic platform using label-free quantification. We are advancing our current results to integrated LOC to perform complete hand-free protocol: from EV enrichment to multi-parametric molecular analysis.

**Funding**: This study was funded by New Brunswick Innovation Foundation, Canada.

PS04.14

Novel label-free method for extracellular-vesicle enrichment from biological fluids and cell culture medium

Prateek Singh ^1^; Jonne Ukkola^2^; Sry D. Hujaya^2^; Henrikki Liimatainen^3^; Seppo Vainio^1^

^1^University of Oulu, Oulu, Finland; ^2^Fibre and Particle Engineering, University of Oulu, Oulu, Finland; ^3^Lignocellulose Research Team, Fibre and Particle Engineering, University of Oulu, Oulu, Finland

**Background**: Plant cellulose is the most abundant biopolymeric raw material on Earth. It is a biodegradable and biocompatible material providing new bio-based platforms and chemicals for green technologies. We have developed cellulose nanofibres which allow capturing of extracellular vesicles (EVs) from aqueous solutions. In this study, cellulose nanofibrils (CNFs) from wood fibres were used as a platform for EV purification. CNFs are based on long, polymeric cellulose chains consisting of hundreds to several thousand repeating glucopyranose units each containing three hydroxyl groups which can be easily, chemically modified to have versatile functions.

**Methods**: EVs from RENCA cell lines and bovine milk were used to assess the performance of the nanocellulose promoted EV isolation method. To obtain CNF, the pretreated wood fibres were fibrillated to nanoscale with a microfluidizer. CNF was further oxidized to dialdehyde and dicarboxyl acid cellulose (DAC and DCC respectively). Ethylenediamine cellulose (EDAC) was prepared via reductive amination by first oxidizing fibres with sodium periodate, reacted with EDA and then reduction with sodium borohydride. BCA protein assay and transmission electron microscopy were utilized to verify EV removal.

**Results**: Four different CNF qualities were prepared and used to pull down EVs from dilute aqueous solutions. Our preliminary tests showed that intact, non-functionalized CNF and DCC were inactive towards EVs. DAC on the other hand, showed slightly more preferred binding to the EVs. The best binding to EVs was observed with amino-modified EDAC, indicating that electrostatic interactions between protonated amines in EDAC and negatively charged EV membrane play an important role in facilitating EV pulldown. Compared to ultracentrifugation, EDA functionalized nanocellulose pulls down 70% of the EVs, in a total processing time of 1.5 h.

**Summary/Conclusion**: The CNFs were rapid alternatives to EV purifications as compared to lengthy ultracentrifugation. Antibody functionalization of these nanocellulose fibres can further increase purification efficiency of EVs from solutions.

PS04.15

Chitosan, a non-toxic polysaccharide based extracellular vesicle isolation technology: potential for therapy and liquid biopsy applications

Awanit Kumar^1^; Surendar Reddy Dhadi^1^; Sebastien R. Fournier^1^; Catherine R. Taylor^1^; Sheena R. Fry^1^; Rodney J. Ouellette^1^; Aniran Ghosh ^2^

^1^Atlantic Cancer Research Institute, Moncton, Canada; ^2^Adjunct professor at the Department of chemistry and biochemistry at Université de Moncton, Moncton, Canada

**Introduction**: Most currently available extracellular vesicles (EVs) isolation methods have specific limitations in various applications due to purity or complexity of the methods or clinical adaptability. Thus, there is a great demand for simple, robust and clinically adaptable and applicable EVs isolation methods. The present work demonstrates the EVs capture efficacy of chitosan, a non-animal and non-toxic polysaccharide for potential human applications. Chitosan is FDA-approved for various clinical applications and thus may provide opportunities for EV-based cellular delivery vehicle.

**Methods**: Purified chitosan of various molecular sizes from non-animal origin were used for this study. We tested the different formulations of the above chitosan based on their pH and effective concentration. Chitosan-isolated EVs (CH-EVs) were characterized using nanoparticle tracking analysis (NTA), transmission electron microscopy (TEM), atomic force microscopy (AFM), Western blot and polymerase chain reaction. CH-EVs were also tested for their potential as cellular delivery vehicles.

**Results**: We determined optimal formulation (pH) and concentrations ranges of chitosan for their ability to isolate EVs from different source materials using previously mentioned physical and molecular techniques. We found that chitosan functions in a wide range of conditions which are suitable for EVs isolation using acidic as well as pH-neutralized solutions. Our preliminary data also indicates that chitosan-isolated EVs are internalized into cells, which suggests their potential as a therapeutic delivery means.

**Summary/Conclusion**: As a non-toxic FDA approved molecule, chitosan may represent a superior matrix for future therapeutic manipulations and applications that require CH-EVs complex. This method of EV isolation may also be used for liquid biopsy assays to identify disease markers and actionable mutations.

**Funding**: This study was funded by Atlantic Canada Opportunities Agency (ACOA).

PS04.16

A functional assessment of affinity isolated inflammatory extracellular vesicle subpopulations

Sören Kuypers ^1^; Revathy Munuswamy^2^; Nynke M.S. Van den Akker^3^; Daniel G.M. Molin^3^; Luc Michiels^4^; Baharak Hosseinkhani^2^

^1^Hasselt University, Biomedical research institute (BIOMED), Hasselt, Belgium; ^2^Bionanotechnology group, Biomedical Research Institute (BIOMED), Hasselt University, Belgium, Hasselt, Belgium; ^3^Maastricht University, Department of Physiology, Cardiovascular Research Institute Maastricht (CARIM), Maastricht, The Netherlands; ^4^Hasselt University, Bionanotechnology group, Biomedical research institute (BIOMED), Hasselt, Belgium

**Background**: Characterization of extracellular vesicles (EV) is currently impeded by co-isolation of impurities. Affinity based methods yield high purity EV; however, it is still difficult to isolate intact and therefore biologically active EV subgroups. We have demonstrated that we could successfully isolate functionally intact inflammatory EV (iEV) subgroups using an affinity based method. This method enables us to study the biological activity of specific EV subgroups in functional *in vitro* cell based assays as well as *in vivo* studies.

**Methods**: EV bulk was isolated using size-exclusion chromatography (SEC) from vascular endothelial cells (EC) supernatants, either from EC untreated or treated with TNF-α to induce inflammatory stress. An in-house developed affinity based method was applied to select EV subgroups. Transmission electron microscopy (TEM), nanoparticle tracking analysis (NTA) and Western blot (WB) were used to characterize EV. The biological activity of intact EV subgroups was tested by analysing their effect on expression of inflammatory associated biomarkers in and functional activity of target cells.

**Results**: TEM, NTA and WB confirmed that intact subgroups were successfully purified using this affinity-based method. ICAM-1 expression was upregulated in EC upon treatment with iEV subgroups. Additionally, monocyte adhesion on EC, a widely used functional inflammatory *in vitro* assay, was increased after treatment of these EC with iEV subgroups. These cell experiments prove that the EV subgroups maintain their functional biological integrity.

**Summary/Conclusion**: These results prove that we isolate biologically active EV subgroups associated with inflammation. Moreover, their functionality was confirmed in a monocyte adhesion assay. This approach provides a powerful tool to select diagnostically relevant, functionally intact EV subgroups from liquid biopsies.

**Funding**: This work was financed by Hasselt University and by the European Regional Development Fund (ERDF), European Commission and Province of Belgium Limburg through the Interreg V Grensregio Vlaanderen Nederland project Trans Tech Diagnostics (TTD).

PS04.17

From bench to bedside: a systematic approach to increased laboratory exosome production

Christina M.A.P. Schuh; Rafael Tapia; Maroun Khoury

Cells for Cells, Santiago, Chile

**Background**: Over the last years, interest for microvesicles and exosomes has significantly increased as they revealed a high therapeutical potential for several clinical conditions, such as haemorrhagic shock, cancer, among others. The bottleneck for preclinical and clinical testing remains the reliable production of exosomes with consistent quality, as existing processes not only are unreliable concerning purity and scaling (<500 ml), but also are unreproducible due to batch-differences. The aim of our study was to design a process and evaluation system for optimized laboratory scale production of exosomes that can be transferred to a GMP environment.

**Methods**: Mesenchymal stem cells derived from menstrual fluid were cultivated under classic cell culture conditions or using microcarrier support, chosen under the prerequisite to be transferrable into GMP: BioNoc, Cytodex 3 and Capex. Culture conditions were evaluated assessing the exosome yield (NanoSight), exosome composition (Western blot), as well as cell viability (MTT assay) and onset of cell senescence (X-Gal assay). Ultracentrifugation of supernatants and its variations (gradient centrifugations, centricon prepurification) is the most abundantly used method for exosome isolation. Tangential flow filtration represents a GMP-compliable alternative to purify exosomes from small (500 ml) to large (10 l) volumes and through defined kDa cut-offs-modulate the composition. Following purification, exosomes can be stored in native or lyophilized state.

**Results**: We will present results on how microcarrier implementation improves exosome yield and cell viability, as well as data on tangential flow filtration compared to ultracentrifugation.

**Summary/Conclusion**: Our process offers a systematic approach to step-by step optimize exosome production regarding yield and purity, and-due to its GMP-compliable techniques – facilitating the translation of exosome therapies into the clinics.

**Funding**: Financial support from CORFO Chile Project “Capital Humano Para La Innovacion” 17CH-83954 is gratefully acknowledged.

PS04.18

Isolation of extracellular vesicles by gel filtration: a comparison of two commonly used protocols

Sebastian Borosch ^1^; Christina Schlingschroeder^2^; Christian Stoppe^3^; Eva Miriam Buhl^4^; Christian Beckers^2^; Ruediger Autschbach^2^; Sandra Kraemer^5^

^1^Department of Thoracic and Cardiovascular Surgery, Uniklinik RWTH Aachen, Aachen, Germany; ^2^Department of Thoracic and Cardiovascular Surgery, University Hospital RWTH Aachen, Aachen, Germany; ^3^Department of Intensive Care and Intermediate Care, University Hospital, RWTH Aachen, Aachen, Germany; ^4^Electron Microscopic Facility, Medical Faculty, University Hospital RWTH, Aachen, Germany; ^5^Department of Thoracic and Cardiovascular Surgery, University Hospital RWTH Aachen, Aachen, Germany

**Background**: The purification of extracellular vesicles (EVs) is still a subject of constant debate in the current literature. The most common protocol is differential centrifugation followed by ultracentrifugation. Nevertheless, other purification methods are emerging and offer high purities and high yields in less time. One of these methods is gel filtration. Several companies provide ready-made columns and promise a fast and reliable purification procedure. We compared two of these columns and evaluated their efficiency.

**Methods**: We used cell culture supernatant from primary cardiac cells as well as plasma from coronary artery bypass graft (CABG) surgery patients. The cell culture supernatant and plasma were differentially centrifuged to eliminate impurities. Cell culture supernatant was additionally ultrafiltrated. 0.5–1 ml were applied on the gel filtration columns. We compared the qEV columns from iZON with the Exo-Spin midi columns from Cell Guidance Systems. Fractions of 0.5 ml were collected. Size and concentration were analysed by nanoparticle tracking analysis (NTA). Additionally, electron microscopy was performed and the EV composition was characterized by Western blot. Stain free images and micro-BCA assays provided information about the purity of the isolated EVs.

**Results**: The different systems provided EVs in different qualities, depending on the starting material. For cell culture supernatants, both columns resulted in comparable yields and purity of vesicles. In contrast, there were drastic differences between the columns when plasma was applied. The Cell Guidance Systems columns resulted in an elevated protein to particle ratio compared to the qEV columns, indicating more protein contaminations. Electron microscopy further confirmed this finding and impurities were detected in the designated EV-rich fractions. The qEV columns on the other hand provided EVs with less protein contaminations and were more reliable and consistent.

**Summary/Conclusion**: Isolation of EVs from cell culture supernatant was possible with both systems in comparable yield and purity. However, EV isolation from human plasma resulted in significant differences between the columns. qEV columns provided sufficient EVs and showed an acceptable balance between yield, purity and effort whereas cell guidance columns provided insufficient plasma EVs.

PS04.19

Semi-quantitation and characterization of serum-derived exosomes in coronary artery disease by glycan recognition bead, EXÖBead

Dapi Meng Lin. Chiang ^1^; Dominik Buschmann^2^; Benedikt Kirchner^2^; Florian Brandes^3^; Gustav Schelling^3^; Michael W. Pfaffl^2^; Chin-Sheng Lin^4^

^1^Biovesicle Inc., Taipei, Taiwan (Republic of China); ^2^Division of Animal Physiology and Immunology, School of Life Sciences Weihenstephan, Technical University of Munich, Germany, Freising, Germany; ^3^Department of Anesthesiology, University Hospital, Ludwig-Maximilians-University Munich, Germany, München, Germany; ^4^Division of Cardiology, Department of Medicine, Tri-Service General Hospital, National Defense Medical Center, Taipei, Taiwan, Taipei, Taiwan (Republic of China)

**Background**: Exosomes are extracellular vesicles released by various cells into a variety of biofluids such as serum. Glycoproteins are a type of cargo loaded into exosomes by the endosomal sorting complex required for transport (ESCRT) machinery. Previous studies using ultrastructural analysis showed that the number of exosomes is increased in the atherosclerotic human aorta compared to healthy donors. The concentration of exosomes in coronary artery disease (CAD) patients, however, has not yet been analysed. We have previously demonstrated that beads based on glycan recognition beads (EXÖBead) capture exosomes from small volumes of serum. In this study, we assessed the concentration and molecular composition of circulating exosomes in CAD patients and healthy volunteers.

**Methods**: We used EXÖBead to capture and analyse exosomes by semi-quantitative flow cytometry (FACS). Serum was collected in patients undergoing percutaneous coronary intervention. We incubated EXÖBead with 250 µl precleared serum from healthy donors (*n* = 14) and CAD patients (*n* = 18). The exosome marker CD63 was detected in exosome-EXÖBead complexes by FACS. We also incorporated 10% exosome-free foetal bovine serum (FBS) in PBS as an anti-human CD63 antibody staining negative control. Additionally, expression patterns of CD63 and ESCRT components in exosomes isolated by EXÖBead were analysed by Western blot (WB). The amount of exosomes is measured by Nanoparticle Tracking Analysis (NTA) by elution from the EXÖBead.

**Results**: Median fluorescence intensity of CD63 in exosome-beads complexes from CAD patients was higher than for healthy donors. EXÖBead isolation captured more exosomal protein from CAD serum, and CD63 was found to be enriched in CAD exosomes compared to healthy volunteers. The number of exosomes is also increased in CAD serum.

**Summary/Conclusion**: As evidenced in samples isolated by EXÖBead, CAD patients may secrete more exosomes into the circulation. In addition, CAD exosomes may carry more cargo proteins. This study is still ongoing for demonstrating these finding in a larger cohort and also discovering more potential biomarkers in CAD patients.

PS04.20

Scalable xeno-free manufacturing of extracellular vesicles derived from human mesenchymal stem cells

Lye Theng Lock; Kelvin S. Ng; Prarthana Ravishankar; Robert D. Kirian; Jon Rowley

RoosterBio Inc., Frederick, USA

**Background**: Having been investigated in >800 clinical trials without significant adverse events, human mesenchymal stem cells (hMSCs) are a safe and clinically relevant cell source for producing extracellular vesicles (EVs) such as exosomes. Not only can hMSC-EVs deliver exogenous agents including RNA and proteins, hMSC-EVs also inherit therapeutic potential of hMSCs and have been applied in >20 disease models. However, based on the current state-of-the-art, a single hMSC-EV dose would require an equivalent of >10 hMSC doses to generate, rendering this technology cost-prohibitive.

Typical EV generation and isolation procedures utilized today involve (1) an initial expansion phase lasting 14–30 days where hMSCs are cultured in serum-containing medium; (2) buffer exchange where exogenous EVs in the serum-containing medium are rinsed off and an EV-free collection medium is added; and (3) an EV collection phase where hMSC-EVs accumulate in the EV-free medium. We hypothesize that the cost and yield of producing hMSC-EVs can be optimized in parallel with a scalable hMSC manufacturing process to make these technologies commercially viable.

**Methods**: To this end, we utilized high-volume xeno-free (XF) hMSCs and streamlined batch culture process to expand hMSCs within 5 days, minimizing time and cost to obtain a high volume of high-quality hMSCs. Cells were characterized for their cell surface marker expression, trilineage differentiation potential, angiogenic cytokine secretion and immunomodulatory activity. We further investigated the productivity of hMSC-EVs in 2D versus 3D culture.

**Results**: Our preliminary data demonstrate that hMSC-EV yield is >8× higher in 3D than in 2D, resulting in a more efficient EV scale-up process. The cells in 3D maintained their potency markers and functionality as in a 2D culture, indicating the maintenance of secreted EVs quality. The cost comparison for initial cell expansion, and subsequently EV production, will be presented.

**Summary/Conclusion**: This efficient, robust and clinically relevant XF bioreactor process for producing EVs from high-quality hMSCs will improve the cost profile towards manufacturing clinical-grade EVs.

PS05: EVs in the Nervous System (Neuronal Network, Blood-Brain-Barrier) Chairs: Dimitrios Kapogiannis; Javier RomeroLocation: Exhibit Hall 17:15–18:30

PS05.01 - OWP2.02

Detection and characterization of different neuronal and glial populations of exosomes by surface plasmon resonance imaging

PS05.02 = OWP3.03

Extracellular vesicles as mediators of periphery-to-brain communication in inflammation-associated brain disorders

PS05.03

Pathological spread of TAR DNA binding protein 43 (TDP-43) in an induced pluripotent stem cell model of dementia

David Hicks; Alys Jones; Stuart Pickering-Brown; Nigel Hooper

University of Manchester, Manchester, UK

**Background**: Intracellular inclusions of TAR DNA binding protein 43 (TDP-43) have been recognized as pathological hallmarks of frontotemporal dementia (FTD) and motor neuron disease (MND) for a decade. Recent studies have revealed the presence of TDP-43 inclusions in 20–50% of Alzheimer’s disease (AD) cases.

**Methods**: TDP-43 has been shown to be able to seed aggregation of TDP-43 in healthy cells via a mechanism which has been suggested to be exosome-dependent. In this study, we have isolated exosomes using differential ultracentrifugation and size exclusion chromatography from SH-SY5Y and NSC-34 neuroblastoma cell lines and also from human neurons derived from induced pluripotent stem cells (iPSCs).

**Results**: TDP-43 was found to co-sediment at 100,000×*g* with exosome markers Tsg101, CD9 and CD63. Furthermore, TDP-43 was not isolated in the same fractions as the non-vesicle marker Grp78 and the non-exosome extracellular vesicle marker mitofilin. The isolated exosome population had a mean vesicle diameter of approximately 50 nm as indicated by dynamic light scattering and electron microscopy, which correlates with the defined diameter of an exosome. Thus endogenous TDP-43 has been shown to be present in exosomes isolated from unstimulated neuroblastoma cells and human cortical neurons. Exosomes derived from stressed cells were able to reduce expression of nuclear TDP-43 in iPSC-derived neurons.

**Summary/Conclusion**: Future work will focus on the ability of neurons derived from patients with TDP-43, GRN and C9ORF72 mutations to seed aggregation of TDP-43 in control neurons derived from healthy individuals in a co-culture system. A mechanistic dissection of this process may reveal novel therapeutic targets in FTD, MND and AD.

**Funding**: This study was funded by Alzheimer’s Society, MRC and Dr Donald Dean Fund for Dementia Research.

PS05.04

A novel method for identification of extracellular vesicles derived from the blood–brain barrier and their role in multiple sclerosis pathogenesis

Jennifer R. Linden; Samantha Shetty; Timothy Vartanian

Weill Cornell Medical School, New York, NY, USA

**Background**: The blood–brain barrier (BBB) plays a key role in MS pathogenesis; however, the molecular mechanisms involved are still poorly understood. Our ability to study the molecular and cellular changes occurring at the BBB in living subjects is necessarily hampered by the inaccessibility of CNS endothelial cells to direct experimentation. A technique to study BBB dysfunction on the cellular level in real time in human subjects is needed. We propose to isolate CNS derived extracellular vesicles (CNS-EV) from MS patients and compare their molecular contents to EV isolated from healthy controls. We hypothesize that circulating CNS-EV will be higher in MS patients compared to healthy controls and will contain alerted molecular contents.

**Methods**: The myelin and lymphocyte protein MAL is specifically expressed by CNS microvasculature. By using a ligand specific for MAL, we have developed a flow cytometry reagent that specifically identifies CNS-EV. EV isolated from peripheral blood are identified using antibodies against known endothelial cell markers.

**Results**: Relapsing remitting multiple sclerosis (RRMS) patients in relapse and secondary progressive multiple sclerosis (SPMS) patients have significantly higher circulating CNS-EV compared to healthy controls. Interestingly, CNS-EV from RRMS patients are phenotypically different from CNS-EV from SPMS patients. This indicates that the mechanisms of BBB permeability in RRMS patients may be different from that of SPMS patients. Extracellular vesicles from MS patients also significantly increased BBB permeability in an *in vitro* model of the human BBB compared to HC. In addition, extracellular vesicles from MS patients significantly upregulated monocyte and lymphocyte activation and increased adherence to human brain endothelial cells compared to HC. This indicates that EMP may play an important role in propagating MS pathogenesis by influencing BBB permeability and immune activation.

**Summary/Conclusion**: Current studies are underway to evaluate the molecular contents of EV from healthy controls versus MS patients to determine the mechanisms involved in this process. Identifications of these mechanisms may assist in the development of treatments that would prevent new MS lesion formation.

**Funding**: This study was funded by National Multiple Sclerosis Society.

PS05.05

Intranasal delivery of lncRNA-Cox2 siRNA loaded exosomes as a therapeutic strategy for restoring lipopolysaccharide and morphine mediated functional impairment of microglia

Guoku Hu; Ke Liao; Fang Niu; Shilpa Buch

University of Nebraska Medical Center, Omaha, USA

**Background**: Impairment of microglial functioning is a hallmark of neuroinflammation. In this study, we demonstrated that LPS and morphine independently induced impairment of microglial functioning (proliferation/activation and phagocytosis) via induction of long-noncoding RNA (lncRNA)-Cox2. Knockdown of lncRNA-Cox2 could thus be envisioned as a therapeutic strategy to restore microglial functioning in the CNS. Herein we propose intranasal administration of EVs loaded with lncRNA-Cox2 siRNA as a noninvasive method for restoring LPS and morphine mediated impairment of microglial functions.

**Methods**: EVs were isolated from normal human primary astrocytes using the standard differential ultracentrifugation method and were characterized using transmission electron microscopy, NanoSight, atomic force microscopy and Western blot analyses. EVs were transfected with lncRNA-Cox2 siRNA using Exo-Fect Exosome Transfection Reagent and were labelled with PKH26. Groups of mice were intranasally administered labelled EVs dropwise with a micropipette and assessed for biodistribution using Xenogen IVIS 200 imager. Separate group of mice were administered intranasally either control siRNA or lncRNA-Cox2 siRNA loaded EVs following intraperitoneal injections of either LPS or morphine. Brains of these mice were harvested for assessment of microglial functions by qPCR and immunostaining.

**Results**: IVIS imaging results demonstrated that labelled EVs localized primarily in the lungs, liver, brain, gut and heart 4 h post-EV administration. Interestingly, 24 h-post-EV administration mice, labelled EVs had disappeared in the lungs, but continued to be present in the brain and heart. Furthermore, there was a significant uptake of labelled EVs by the microglia in the brain with lincRNA-Cox2 siRNA EVs ameliorating microglial phagocytic activity in morphine-administrated mice, and dampening LPS-mediated microglial proliferation/activation.

**Summary/Conclusion**: Intranasal delivery of lncRNA-Cox2 siRNA loaded EVs into mice resulted in restoration of LPS/morphine-mediated impairment of microglial functioning.

**Funding**: This work was supported by grants MH112848, DA040397 (SB) and DA042704 (GH) from the National Institutes of Health. The support by Nebraska Center for Substance Abuse Research is acknowledged.

PS05.06

Investigating the mechanisms of molecular exchange in between retinal neurons

Aikaterini Kalargyrou; Robin Ali; Rachael Pearson

UCL Institute of Ophthalmology, London, UK

**Background**: Retinal degeneration due to the loss of photoreceptors (PRs) is the leading cause of untreatable blindness. Repair by transplantation of healthy PRs is a promising therapeutic tool. Previous studies have shown that transplantation of PR precursors can rescue visual function in some models of retinae dystrophy. Previously, this was thought to arise from donor PRs integrating within the host retina. However, we have recently shown that, where some host PRs remain, many reporter-labelled cells previously interpreted as integrated donor cells, were actually host PRs that acquired the label through molecular exchange or material transfer, between donor and host cells. This exchange is robust and permits acquisition by the host cell of many proteins expressed only by the donor. Since extracellular vesicles (EVs) are increasingly recognized as key players of molecular communication, we hypothesized that material transfer is mediated by the exchange of molecular information packaged in EVs.

**Methods**: Rod PRs were isolated from postnatal day (P)4 wildtype mouse retinae using MACS and cultured for 14–21 days. EVs were isolated from culture medium using differential ultracentrifugation. Large, medium and small EVs retrieved by 2K, 10K and 100K spins were analysed with DLS, TEM, Western blot, dot-blot and RTqPCR. MVB analysis in whole eyes was performed using TEM. TNTs were analysed with confocal imaging. Functional exchange was assessed using with a Cre-loxP recombination read-out system.

**Results**: Cultured PRs release a variety of EVs in a developmentally dependent manner. Small EVs (sEVs) bear proteins typical of PRs and of endocytic origin. When separated in a transwell co-culture system, Cre+ photoreceptors can mediate recombination of underlying reporter retinal cells through a mechanism that does not require sustained cell–cell contact. In culture, primary PRs extend filamentous actin+ protrusions within the first 24 h. These changes over time, and immunofluorescence analysis reveals the presence of vesicular like forms within them.

**Summary/Conclusion**: Primary PRs release sEVs with morphological and molecular profiles typical of neuronal EVs in a developmentally dependent manner. These sEVs appear capable of mediating horizontal signalling with other retinal cells *in vitro*.

PS06: EVs in Metabolic Diseases and Aging Chairs: Nicole Noreen Hooten; Ryou-u TakahashiLocation: Exhibit Hall 17:15–18:30

PS06.01

MIF adipocytokine uses extracellular vesicles as secretion pathway

Jérémy Amosse^1^; Maeva Durcin^2^; Marine Malloci^3^; Luisa Vergori^1^; Séverine Dubois^4^; Gilles Simard^5^; Olivier Hue^6^; M. Carmen Martinez^1^; Ramaroson Andriantsitohaina^1^; Soazig Le Lay ^1^

^1^INSERM UMR1063, Angers, France; ^2^INSERM U1063/University of the French West Indies, Angers, France; ^3^UMR INSERM 1063, Angers, France; ^4^Service EDN, Angers, France; ^5^Biochimie-CHU Angers, Angers, France; ^6^UPRES EA 35-96 UFR-STAPS Université Des Antilles et de La Guyane, Pointe À Pitre, French Southern and Antarctic Lands

**Background**: Obesity-associated metabolic dysfunctions have been linked to dysregulated production of secreted factors from adipose tissue, known as adipocytokines. Besides, accumulating evidences suggest a role for fat-derived extracellular vesicles (EV) in the development of metabolic disturbances. Since EV convey numerous proteins and metabolites, we aimed to evaluate their contribution in the secretion of adipocytokines.

**Methods**: Plasma samples collected from patients suffering of metabolic syndrome were used to isolate EV subtypes, namely microvesicles (MV) and exosomes (EXO) by differential centrifugations. Patients were classified according to their body mass index (BMI): Control (BMI < 27), overweight (2730). 22 adipocytokines circulating concentrations were successively measured on total, MV- and EV-depleted plasma samples by multiplex immunoassays.

**Results**: MV and EXO populations, respectively, quantified by flow cytometry and NTA, were significantly increased with BMI supporting a role of these vesicles as metabolic relays in the context of obesity. Multiplex analysis of plasma adipocytokines confirms dysregulated production of these factors with increased BMI. Sequential depletion of MV and EXO from all plasma patients did not modify adipocytokine plasma levels, at the exception of MIF (macrophage migration inhibitory factor). Importantly, MIF plasma concentration was decreased by half following MV depletion and this MV-associated proportion was unchanged with obesity. Specific association of MIF with MV was observed in purified MV from different cell sources, including adipocytes and lymphocytes, demonstrating that this adipocytokine uses MV as a constitutive secretory pathway. Finally, we demonstrated that MV-associated MIF was able to mediate functional responses such as ERK activation in macrophages.

**Summary/Conclusion**: All together, our study emphasizes the importance to consider the MV secretory pathway in the metabolic actions of MIF adipocytokine.

**Funding**: This study was approved by Angers University hospital ethical committee (NCT: 00997165) and received written consent from patients. This work was funded by a research national grant (ANR MilkChEST no. ANR-12-BSV6-0013-04), by GIS APIS-GENE and the Francophone Society of Diabetes.

PS06.02

Critical role of Rap1 in triggering the effects of microparticles from metabolic syndrome patients on vascular smooth muscle cell functions

Liliana Perdomo^1^; Luisa Vergori^1^; Lucie Duluc^1^; Maggy Chwastyniak^2^; Marion Laudette^3^; Xavier Vidal-Gomez^1^; Raffaella Soleti^1^; Florence Pinet^2^; Frank Lezoualc’h^3^; Séverine Dubois^4^; Samir Henni^5^; Jérôme Boursier^5^; Frédéric Gagnadoux^1^; Ramaroson Andriantsitohaina ^1^; M. Carmen Martinez^1^

^1^INSERM UMR1063, Angers, France; ^2^INSERM U1167 – Université de Lille Nord de France – Institut Pasteur de Lille, Lille, France; ^3^Institut of Cardiovascular and Metabolism Diseases, Toulouse, France; ^4^Service EDN, Angers, France; ^5^CHU Angers, Angers, France

**Background**: The metabolic syndrome (MetS) is a cluster of interrelated risk factors for cardiovascular disease and atherosclerosis including hyperglycaemia, dyslipidaemia, hypertension and obesity. We have previously shown that circulating levels of microparticles (MPs), small vesicles released from plasma membrane, from MetS patients induce endothelial dysfunction. Here, we analyse whether MPs from MetS patients may participate to the alteration of smooth muscle cells (SMC) function described during the atherosclerosis development.

**Methods**: Circulating MPs of non-MetS subjects and MetS patients have been isolated from plasma and characterized by proteomic analysis. Then, the involvement of Rap1 in the effects of MPs on human aortic SMC (HASMC) proliferation, migration and cytokine secretion was analysed.

**Results**: Differential proteomic analysis of MPs from both types of individuals identifies Rap1, a small GTPase, as twofold overexpressed in MPs from MetS compared with non-MetS subjects. In addition, Rap1 is in active state, that is, GTP-associated, in both types of MPs. When HASMC are incubated with MPs for 24 h, both types of MPs significantly promote proliferation and migration. Even more, MetS MPs are able to increase the expression of the pro-inflammatory molecules MCP-1 and IL-6. Neutralization of Rap-1 by specific antibody or pharmacological inhibition of Rap-1 with GGTI-298 either partially or completely prevents the effects of MPs from MetS patients but not those from non-MetS MPs. These effects include HASMC proliferation, migration, inflammation and increase of p38 and ERK5 phosphorylation.

**Summary/Conclusion**: These data suggest that overexpression of Rap1 in MetS MPs might participate in the enhanced SMC proliferation, migration and activation of MAPK/ERK pathway leading to atherosclerosis.

PS06.03

Functional characterization of mucosal extracellular vesicles of rodents following metabolic surgery

Bailey Peck ^1^; Mingrui An^2^; Aleksander Kupe^2^; David Lubman^2^; Randy Seeley^2^

^1^Department of Surgery, University of Michigan Medical School, Arborann, USA; ^2^Department of Surgery, University of Michigan Medical School, Ann Arbor, USA

**Background**: Metabolic surgeries, in addition to promoting weight loss, reduce appetite and induce disease remission in patients with type 2 diabetes. While it is clear that this effect does not result from mechanical restriction or nutrient malabsorption, the molecular underpinnings of metabolic surgery are still under rigorous investigation. In addition to changes in metabolic signalling peptides, humans and rodents experience changes in gut microbiota composition following surgery. When transplanted to germ-free animals, microbiota from metabolic surgery animals induce reduced weight gain and adiposity.

**Methods**: To evaluate the potential role of extracellular vesicles (EVs) in promoting these beneficial effects, we isolated EVs from the small intestinal mucus of rodents following a sham or vertical sleeve gastrectomy (VSG) surgeries using differential centrifugation. We used molecular biology, genetics, microscopy and mass spectrometry-based technologies to characterize the EVs from each source.

**Results**: We found that the mucus layer of VSG animals was enriched with high-density nanoparticles relative to sham-operated animals. Broadly, we found that mucus-based EV proteins largely originate from the host and that VSG EVs have more protein diversity, despite originating from fewer external species relative to sham animals. Treatment of small intestinal enteroids with mucosal EVs from sham-operated animals resulted in increased survival relative to untreated enteroids. Surprisingly, treatment with EVs from VSG-operated animals was highly cytotoxic. When we repeated treatment in enteroids originating from Tlr4−/− mice, the cytotoxicity of VSG EVs remained, indicating that microbial EV-based LPS did not induce the observed cytotoxicity through established Tlr4 pathways.

**Summary/Conclusion**: These preliminary results demonstrate that the mucus layer of obese animals are significantly altered by metabolic surgery and that mucosal EVs may play an important role in regulating intestinal epithelial physiology, particularly during the weeks following metabolic surgery.

**Funding**: This work was supported by grants from NIH/NIDDK (R01DK107652, T32DK101357).

PS06.04

Human bone marrow mesenchymal stem cells derived extracellular vesicles *in vitro* characterization and cytotoxicity on rat islet cells

Sara Assar Kashani ^1^; Faezeh Shekari^2^; Ensiyeh Haji Zadeh^1^

^1^Royan institute, Tehran, Iran; ^2^Department of Stem Cells and Developmental Biology, Cell Science Research Center, Royan Institute for Stem Cell Biology and Technology, Tehran, Iran

**Background**: Mesenchymal stem cells (MSCs) have been increasingly used in treatment of type 1 diabetes (T1D). In attention to disadvantages of cell therapy versus cell-free therapy, extracellular vesicles (EVs) released from MSCs have drawn wide attention as a promising alternative in cell therapy.

In this study, we investigated the effect of human bone marrow mesenchymal stem cells derived EVs (hBMSC-EVs) on the function of dispersed rat islet cells *in vitro*.

**Methods**: we used supernatant derived from the dynamic expansion of hBMSCs to isolate EVs through gradient ultracentrifugation. EVs were measured for their protein content using a BCA Protein Assay Kit and then characterized by electron microscopy and the size distribution of EVs was measured by dynamic light scattering (DLS) in order to measure the cytotoxicity of the dose-dependent manner of EVs, MTS assay was tested.

Also, we tested if cells could uptake hBMSC-EVs labelled with red fluorescent PKH-26 to follow their functional assay on dispersed rat pancreatic islet cells.

To evaluate the effect of hBMSC-derived EVs on single cell viability we assayed dispersed islet cells using fluorescein diacetate (FDA) and propidium iodide (PI) staining.

**Results**: We quantified that according to the amount of 36 × 10^6^ hBMSC could produce approximately 1218 µg exosomes and 1190 µg microvesicle.

DLS and electron microscopy also have been done for the collected EVs.

Cells were plated at 30,000 cells/well and incubated with exosomes at different concentration (0, 10,100 µg/ml) and the control (PBS) for 48 h. The nanoparticle have been shown to interact with MTS reagent and caused false positive results against the bright field microscopy images after co-culture of islet cells with PKH-26 labelled EVs at different time points (2, 24 and 48 h), the results showed that EVs could be internalized by islet cells.

FDA-PI staining also showed the effect of hBMSC-EVs on the viability of dispersed rat islet cell.

**Summary/Conclusion: I**n this study, we have worked on the characterization of hBMSC-EVs and a cytotoxicity assays on dispersed rat islet cells *in vitro*.

PS06.05

The ageing process alters catalase activity in circulating extracellular vesicles of Wistar rats

Laura Cechinel; Karine Bertoldi; Ionara Rodrigues Siqueira

Universidade Federal do Rio Grande do Sul, Porto Alegre, Brazil

**Background**: Exosomes are able to transfer antioxidant enzymes, namely catalase and superoxide dismutase. Besides, circulating exosomes can produce reactive species during pathological conditions. Recently it was demonstrated that healthy ageing process induces a disruption on circulating exosomes, specifically CD63 content, and an oxidative profile with increased reactive species levels. Our aim was to investigate the effect of ageing process on catalase activity in circulating extracellular vesicles.

**Methods**: The Local Ethics Committee (CEUA – Comissão de Ética no Uso de Animais – UFRGS; nr. 29.818) approved all animal procedures and experimental conditions. Male Wistar rats of 3- and 21-month-old were used. Extracellular vesicles were obtained using a commercial kit based on vesicles precipitation (ExoQuick, System Biosciences). The samples were incubated with ethanol (10%) and Triton (10%). The catalase activity was evaluated at 25°C in order to determine the rate of degradation of H_2_O_2_ in 10 mM phosphate buffer (pH 7.0) at 240 nm. The catalase activity was normalized for total protein content.

**Results**: Circulating extracellular vesicles of aged rats showed reduced catalase activity compared to young adult groups.

**Summary/Conclusion**: Our results suggest that the normal ageing process can modify the ability of circulating extracellular vesicles for molecules exchange, such as antioxidant enzymes.

**Funding**: This work received financial support from Conselho Nacional de Desenvolvimento Científico e Tecnológico-CNPq (grant# 476634/2013-01). CNPq fellowships (Dr. I.R. Siqueira; K. Bertoldi; L.R. Cechinel).

PS06.06

Plasmatic extracellular vesicles released in days during high particulate matter levels are able to activate endothelial cells

Federica Rota ^1^; Chiara Favero^2^; Rita Antonioli^2^; Laura Pergoli^2^; Laura Cantone^2^; Mario Barilani^2^; Lorenza Lazzari^3^; Valentina Bollati^1^

^1^EPIGET LAB, Department of Clinical Sciences and Community Health, Università degli Studi di Milano, Milan, Italy; ^2^EPIGET LAB, Department of Clinical Sciences and Community Health, Università degli Studi di Milano, Milan, Italy; ^3^Cell Factory, Laboratory of Regenerative Medicine, Department of Services & Preventive Medicine, Fondazione IRCCS Ca’ Granda Ospedale Maggiore Policlinico, Milan, Italy

**Background**: Exposure to particulate matter (PM) has been consistently associated with respiratory and cardiovascular (CV) risks. Recent findings propose that in lungs PM produces a strong inflammatory reaction which triggers the release of specific extracellular vesicles (EVs). EVs might reach the systemic circulation, playing a key role in PM-induced health risk. We aim to determine whether EVs isolated from the blood of healthy subjects in a day characterized by low exposure (LE day) or high exposure (HE day) to PM are able to induce a different activation of endothelial cells *in vitro*. Since obesity is a strong CV risk factor, we will further consider if the subject’s body mass index (BMI) can modify this effect.

**Methods**: We isolated EVs from the blood of three overweight (OW) and three normal weight (NW) subjects at two time points (LE day and HE day). EVs were purified and characterized by nanoparticle-tracking analysis and flow cytometry, and used to stimulate primary endothelial cells for 24 h. EVs derived from endothelium (CD105+) and activated endothelium (CD62e+) were successively quantified.

**Results**: First of all plasma EV concentration was higher in HE day than in LE day (23,498 × 10^6^/mL versus 5835 × 10^6^/mL; *p* = 0.01) for all the subjects. In endothelial cells exposed to subjects’ EVs, we analysed the ratio between CD105+ and CD62e+ produced EVs. We observed an increased CD62E+/CD105+ ratio, suggestive of an increased endothelial activation, in cells treated with HE day-EVs. After BMI stratification, we observed that the effect was due to NW subjects (CD62e+/CD105+ = 3.38 vs 1.39; *p* < 0.0001) whereas EVs produced from OW subjects were not able to induce this activation.

**Summary/Conclusion**: EVs seem to have the potential to act as marker of PM susceptibility and as molecular mechanism in the chain of events connecting PM exposure to endothelial alterations, frequently linked to exposure and health risk.

**Funding**: This project received support from the EU Programme “Ideas” (ERC-2011-StG 282413), principal investigator Prof. Valentina Bollati.

PS06.07

Exosomes from high glucose-treated mesangial cells trigger dysfunction of podocytes

Antonio S. Novaes ^1^; Raphael Felizardo^2^; Niels OS Camara^2^; Mirian Boim^1^

^1^Federal University of São Paulo, São Paulo, Brazil; ^2^University of São Paulo, São Paulo, Brazil

**Background**: Understanding of how mesangial cells communicate with podocytes in the diabetic environment is important for the development of new targets for the prevention and treatment of diabetic nephropathy (DN). The aim of this study was to investigate whether exosomes secreted by high glucose-treated (HG-Exos) mouse mesangial cells (MMC) are able to induce dysfunction of normal podocytes.

**Methods**: MMC were cultured under standard (5 mM) or high glucose concentration (30 mM) for 24 h. Exos secreted to the culture medium were purified by ultracentrifugation. The vesicles size/concentration ratio was determined by the particle tracking (NanoSigth) and their characterization was performed by the presence of markers CD63 and CD81 by Western blot. Podocytes in culture were stimulated by HG-Exos for 24 h. Podocytes makers (actinin IV, p-cadherin and synaptopodin) and profibrotic markers (desmin, TGF-β1 and collagen IV) were analysed by qPCR. HG stimulus induced a change in the amount, but not in the size of Exos released by MMC.

**Results**: HG-Exos induced phenotypic transition of podocytes that underwent epithelial mesenchymal transition, demonstrated by a downregulation of actinin 4, p-cadherin, synaptopodin together with an upregulation of desmin and TGF-β1.

**Summary/Conclusion**: These results demonstrated the paracrine communication via exosomes between MMC and podocytes, and suggest that high glucose stimulus in MMC can modified podocytes function contributing to DN.

**Funding**: This study was funded by FAPESP – Fundação de Amparo à Pesquisa do Estado de São Paulo.

PS06.08

Microvesicles as novel biomarkers of frailty

Marta Giannini^1^; Daisy Sproviero^2^; Orietta Pansarasa^2^; Stella Gagliardi^3^; Maria Chiara Mimmi^2^; Tino Emanuele Poloni^4^; Antonio Guaita^4^; Cristina Cereda ^2^

^1^Genomic and Post-Genomic Center, IRCCS, C. Mondino National Institute of Neurology Foundation,Pavia,Italy, Pavia, Italy; ^2^Genomic and post-Genomic Center, C. Mondino National Institute of Neurology Foundation, IRCCS, Pavia, Italy; ^3^Genomic and post-Genomic Center, C. Mondino National Institute of Neurology Foundation, IRCCS, Pavia, Italy; ^4^Golgi Cenci Foundation, Abbiategrasso (MI), Italy, Milano, Italy

**Background**: Frailty is a geriatric syndrome characterized by loss of biological functions across multiple organ systems. Different pathways, linked to cellular senescence and inflammation, are involved in frailty and the identification of biomarkers is still needed. Microvesicles (MVs) represent a promising source of biofluid biomarkers, considering their functions in intercellular communication as carrier of proteins and genomic material.

**Methods**: MVs were isolated from blood of non-frail, prefrail and frail elderly people (*N* = 14 for each group), classified by evaluating functional status, the presence of diseases, physical and cognitive deficits. MVs were stained with CD3 (T Cells), CD4 (T helper), CD8 (T cytotoxic), CD163 (macrophage), CD197 (activated B and T cells), CD221 (insulin-like growth factor receptor IGFR) and CD182 antibodies (IL8), Annexin V (vesicular marker) and calcein (MVs membrane fluorescent dye). Samples were analysed by flow cytometer FACS Canto II (BD Biosciences, USA) using calibration beads (Submicron Bead Calibration Kit, 0.2–1 μm).

**Results**: MVs’ concentration did not show significant difference among the three groups. CD3, CD4 and CD197 derived MVs were slightly increased in MVs of prefrail and frail patients compared to non-frail individuals. CD163 derived MVs slightly increased in non-frail individuals in comparison to the other two groups, while CD221 derived MVs are overrepresented in prefrail and frail subjects compared to non-frail (ANOVA test, **p < 0.01).

**Summary/Conclusion**: The increase in CD3, CD4 and CD197 derived MVs in prefrail and frail individuals could be related to the chronic low-grade state of inflammation. The significant presence of CD221+ derived MVs in prefrail and frail patients could be linked to IGFR, which is already recognized as a common biomarker of senescence. MVs derived from target cells can provide not only possible biomarkers, but also potential mechanisms linked to senescence development.

**Funding**: This project was supported by Cariplo 2018: Association between frailty trajectories and biological markers of aging; FrailBioTrack.

PS06.09

miR-296-5p and PDGF-BB in CD31EV cargo: novel biomarkers of vascular smooth muscle cell dysfunction in diabetes

Claudia Cavallari ^1^; Gabriele Togliatto^1^; Patrizia Dentelli^1^; Arturo Rosso^1^; Giusy Lombardo^1^; Maddalena Gili^1^; Chiara Gai^1^; Anna Solini^2^; Giovanni Camussi^1^; Maria Felice Brizzi^1^

^1^Department of Medical Sciences University of Turin, Turin, Italy; ^2^Department of Surgical, Medical, Molecular and Critical Area Pathology, University of Pisa, Pisa, Italy

**Background**: Endothelial cell-derived extracellular vesicles (CD31EVs) are a new entity for therapeutic/diagnostic purposes. The roles of CD31EVs as biomarkers and mediators of smooth muscle cell (VSMC) dysfunction in type 2 diabetes (T2D) are investigated herein.

**Methods**: Human atherosclerotic plaque specimens from 11 T2D and six non-diabetic individuals undergoing carotid endoarteriectomy surgery were analysed. siRNA technology was performed on vascular smooth muscle cells (VSMCs). The CD31 microbead kit was used to isolate CD31EVs from the sera of T2D (D-CD31EVs) and non-diabetic individuals (ND-CD31EVs). In selected experiments, VSMCs were cultured in HG and then treated with ND-CD31EVs, D-CD31EVs or stimulated with PDGF-BB. CD31EVs were processed for transmission electron microscopy (TEM), biological effects. miR analysis was also performed. PDGF-BB concentration in D-CD31EVs was measured using an ELISA kit.

**Results**: We discovered that VSMCs, from human atherosclerotic arteries of T2D individuals, express low bak/bax and high bcl-2 levels. These effects were recapitulated in VSMCs subjected to HG and boosted by diabetic-sera-derived-EVs (D-CD31EVs). Moreover, unlike non-diabetic serum-derived EVs, D-CD31EVs increased HG-cultured VSMC resistance to apoptosis. We also found an increased expression of miR-296-5p in both T2D-derived atherosclerotic specimens and HG-cultured VSMCs treated with D-CD31EVs. D-CD31EVs were found almost depleted of miR-296-5p, while enriched in membrane-bound-platelet-derived-growth-factor-BB (mbPDGF-BB). Thus, we postulated that mbPDGF-BB transfer by D-CD31EVs could account for VSMC-miR-296-5p content. By depleting CD31EVs of PDGF-BB or blocking the PDGF-BB receptor-β, we demonstrated that PDGF-BB contributes to D-CD31EV-mediated miR-296-5p expression and downstream events. In fact, while PDGF-BB-treatment recapitulated the D-CD31EV-mediated anti-apoptotic programme and VSMC resistance to apoptosis, PDGF-BB-depleted CD31EVs failed. Finally, D-CD31EVs also increased VSMC migration and recruitment to neovessels, by means of mbPDGF-BB.

**Summary/Conclusion**: This study identifies the mbPDGF-BB in D-CD31EVs as a relevant mediator of diabetes-associated VSMC dysfunction, and recognizes CD31EV-miR-296-5p-mbPDGF-BB content as novel diabetes-associated biomarkers.

PS06.10

Role of vascular smooth muscle cell derived-exosomes in age-related vascular amyloidosis

Meredith Whitehead; Sadia Ahmad; Catherine Shanahan

King’s College London, London, UK

**Background**: Exosomes have recently been recognized as key mediators of age-related formation of amyloid, particularly in the brain. The age-related accumulation of amyloid is commonly associated with degenerative diseases, such as Alzheimer’s disease. Exosomes have also been implicated in vascular smooth muscle cell (VSMC) calcification, a manifestation of ageing.

MFGE8 is an age-associated protein expressed by VSMCs and secreted by exosomes. MFGE8 is an amyloid precursor and can be cleaved into a 50-amino acid peptide called medin, which forms aortic medial amyloid (AMA) in ageing vessel walls. The mechanism of AMA formation and deposition is unknown.

The aims are to study: (1) changes in exosome secretion and content with age, (2) amyloid protein loading in exosomes and (3) if MFGE8 and/or AMA can promote calcification.

**Methods**: Western blotting, qPCR and immunostaining were used to study medin and MFGE8 expression in VSMCs, at different ages and in calcifying conditions. FACS analysis was used for quantification of exosome secretion. Exosomes were isolated by differential ultracentrifugation. Extracellular matrix (ECM) was synthesized *in vitro* for immunofluorescent staining and Western blotting. Cresolphthalein assays were used to quantify calcification of VMSCs.

**Results**: MFGE8 and medin were present in the aortas of old, but not young subjects. MFGE8 was expressed by VSMCs and secreted by exosomes. Medin is deposited in the ECM and blocking exosome release decreased its deposition. The expression and secretion of MFGE8 increased in calcifying conditions and recombinant MFGE8 increases calcification while siRNA knockdown of MFGE8 decreased calcification.

**Summary/Conclusion**: Medin and MFGE8 are abundant in aged subjects and are secreted by exosomes into the ECM. Exosome release is increased with age, which could contribute to the deposition of medin in the ECM and the formation of amyloid. MFGE8 may play a role in accelerating calcification by inducing an osteogenic phenotype via the ERK pathway. Both MFGE8 and medin secretion by exosomes could contribute to the age-related development of vascular calcification.

**Funding**: This work is funded by the British Heart Foundation.

PS06.11 = OWP1.07

Role of Wnt4 exosomes in thymic ageing

Krisztina Banfai ^1^; Kitti Garai^1^; David Ernszt^2^; Judit E. Pongracz^1^; Krisztian Kvell^1^

^1^Institute of Pharmaceutical Biotechnology, Faculty of Pharmacy, University of Pecs, Pecs, Hungary, Pécs, Hungary; ^2^Institute of Physiology, Faculty of Medicine, University of Pecs, Pecs, Hungary, Pécs, Hungary

**Background**: Wnt4 plays a crucial role in promoting the development and halting the ageing of the thymus. During ageing Wnt4 is down-regulated, while PPARγ is up-regulated and triggers adipose involution. However, miR27b was described to suppress PPARγ. Our goal was to prove the presence of Wnt4 in exosomes, to detect its effect and follow its path both *in vitro* and *in vivo*.

**Methods**: Exosomes were harvested from control and Wnt4 over-expressing TECs (thymic epithelial cells) for further experiments. Exosomes were visualized by transmission electron microscopy. Exosomal miR27b levels were measured by TaqMan qPCR, while Wnt4 protein content was assayed by ELISA. DiI-labelled exosomes were applied on mouse and human thymus sections and also iv-injected into mouse for *in vivo* tracking.

**Results**: Transmission electron microscopy showed exosomes ranging 50–100 nm in size. TaqMan miRNA assay measured elevated miR27b levels, while ELISA showed high Wnt4-content in Wnt4-exosomes compared to control exosomes. For functional studies steroid (Dx)-induced TECs were used as cellular ageing model. Dx accelerated ageing, but Wnt4-containing exosomes could efficiently counteract Dx-induced senescence. We have obtained diverse staining patterns using DiI-labelled Wn4-exosomes on sections of young and aged samples. Finally, *in vivo* injected DiI-labelled Wnt4-exosomes showed detectable homing to the thymus.

**Summary/Conclusion**: According to our results Wnt4 and miR27b are present in TEC exosomes. Our findings indicate that Wnt4 is a key inhibitor thymic involution potentially via miR27b. However, further experiments are required for possible applications.

**Funding**: Scientific research support was provided by PTE AOK KA-2016-16, PTE Pharmaceutical Talent Center program and the PTE Viral Pathogenesis Talent Center program via KK. The Janos Bolyai Scholarship of the Hungarian Academy of Sciences also supported KK.

PS06.12

Extracellular vesicles and their miRNA cargo in ageing and age-associated diseases

Lucia Terlecki-Zaniewicz^1^; Vera Pils^1^; Ingo Lämmermann^1^; Regina Weinmüllner^1^; Madhusudan Bobbili Reddy^1^; Markus Schosserer^1^; Florian Gruber^2^; Matthias Hackl^3^; Johannes Grillari ^1^

^1^CDL for Biotechnology of Skin Aging BOKU – Department of Biotechnology, Vienna, Austria; ^2^Department of Dermatology, Medical University of Vienna, Austria; Christian Doppler Laboratory for the Biotechnology of Skin Aging, Vienna, Austria, Vienna, Austria; ^3^TAmiRNA GmbH Vienna, Vienna, Austria

**Background**: Cellular senescence has evolved from an *in vitro* model system to study ageing to a multifaceted phenomenon of *in vivo* importance as senescent cell removal delays the onset of a variety of age-associated diseases and chemotherapy induced premature ageing.

**Methods**: In order to understand how senescent cells that accumulate within organisms with age negatively impact on organ and tissue function, we have characterized senescent cell derived extracellular vesicles (EVs) and their miRNA cargo and their functional role in the context of cellular and organismal ageing, especially on how EV derived miRNAs influence differentiation and proliferation of skin keratinocytes and mesenchymal stem cells.

**Results**: We identified EVs and circulating miRNAs as *bona fide* members of the senescence associated secretory phenotype (SASP) that are transferred from senescent cells to their microenvironment or even the systemic environment. Upon uptake, recipient cells alter their behaviour, including changes in osteogenic differentiation of mesenchymal stem cells, in wound healing of skin keratinocytes or apoptotic behaviour of skin fibroblasts. Especially in the context of osteogenic differentiation, we were further able to show that circulating miRNAs are prognostic biomarkers of osteoporotic fracture risk.

**Summary/Conclusion**: In summary, we present evidence of the importance of specific miRNAs and highlight their potential use as biomarkers of ageing and age-associated diseases, or even as therapeutic tools and targets to prevent age-associated diseases.

**Funding**: This study was funded by Christian Doppler Gesellschaft, FWF, EU FP7 SYBIL, EU FP7 Frailomic.

PS06.13 = OWP1.04

Prostate cancer-derived extracellular vesicles facilitate osteoclast fusion and differentiation via enhancing filopodia formation in osteoclast precursors

PS07: EVs in Tumor Metastasis Chairs: Takahiro Ochiya; Carla Oliveira Location: Exhibit Hall 17:15–18:30

PS07.01

Extracellular vesicles in an *in vivo* system for macrophage migration

Karen Linnemannstoens; Leonie Witte; Julia Christina Gross

University Medical Center Goettingen, Goettingen, Germany

**Background**: Cell migration is a polarized cellular process in which the protruding leading edge opposes a retracting trailing edge. EVs control directionally persistent cell migration by creating an autocrine local gradient. This requires polarized delivery of MVBs to the plasma membrane and subsequent polarized secretion. While most of the studies in cell culture focus on migratory phenotypes, EV studies in developmental/physiological signalling have mostly been conducted in polarized epithelia like imaginal discs. The question is whether the same cellular machineries direct the selective sorting of cargo into different vesicles which are then secreted apically vs. basally or from the leading vs. trailing edge respectively.

**Methods**: To understand this complex process in a multicellular organism, we study EV biogenesis and polarized secretion in the model of migrating *Drosophila* pupal haemocytes, which are components of the haemolymph and constitute blood cells and macrophages in flies.

Whereas isolation of EVs from cell culture supernatants and human serum are established, neither the presence nor function of secreted EVs in haemolymph has been studied so far. We established a technique to purify EVs from haemolymph by differential centrifugation and analysed the resulting pellets by several techniques.

We generated several EV reporter fly lines expressing fluorescently labelled haemocytes and EV marker. These lines allow to visualize both haemocytes and vesicles within the cells and characterize their dynamics by live imaging. Besides, RNAi constructs targeting specific genes can be expressed exclusively in haemocytes to analyse the effect on EV localization.

**Results**: EVs were purified by differential centrifugation and the pellets corresponding to microvesicles and exosomes were analysed by Western blot, nanoparticle tracking analysis and mass spectrometry. Combining RNAi, confocal microscopy and automated image analysis, we identified new factors required for EV localization in isolated pupal haemocytes. These factors have evolutionary conserved function in human tumour cells and we are currently characterizing their function both for EV release as well as in cell migration.

**Summary/Conclusion**: Taken together, our approach allows for a rapid screening of potentially interesting candidate genes in an *in vivo* setting of EV release and cell migration.

PS07.02

Analysing novel mechanisms involved in tumour-adipose tissue crosstalk during melanoma metastasis: role of secreted exosomes and soluble factors

Lucía Robado de Lope ^1^; Alberto Benito-Martin^2^; Sara Sánchez-Redondo^1^; Diego Megias^3^; Marta Hergueta-Redondo^1^; Héctor Peinado^1^

^1^Microenvironment and Metastasis Group, Molecular Oncology Programme, Spanish National Cancer Research Centre (CNIO), Madrid, Spain; ^2^Department of Pediatrics, Drukier Institute for Children’s Health and Meyer Cancer Center, Weill Cornell Medical College, New York, USA; ^3^Confocal Microscopy Unit, Biotechnology Programme, Spanish National Cancer Research Centre (CNIO), Madrid, Spain

**Background**: Increasing evidences reveal a link between obesity and the development and progression of certain types of cancer. However, the implication of obesity in melanoma metastasis is not well known. Recent data support a role for secreted factors [e.g. soluble factors and extracellular vesicles] in the communication between tumour cells and adipose tissue during metastasis. Still, the specific factors reinforcing the metastatic behaviour have not been defined yet.

**Methods**: Mice under regular and high fat diet (HFD) were intravenously injected with melanoma cells to analyse their metastatic behaviour in both conditions. In addition, we isolated adipose tissue from control and HFD mice to analyse the secretome of different fat depots. We also performed *in vitro* and *in vivo* approaches to determine the uptake of exosomes by adipose tissue. Flow cytometry analysis was done after the *in vivo* injection of tumour-derived exosomes in control and HFD mice. *In vitro* analysis was performed using the Opera High Content Screening System. We analysed the phenotypic changes promoted by tumour-derived exosomes in adipose tissue-derived mesenchymal stem cells (AD-MSCs).

**Results**: We found that HFD-fed mice had increased metastatic burden in specific anatomical locations of adipose tissue (e.g. inguinal, retroperitoneal) compared to controls. To decipher the factors involved, we analysed adipose tissue-secreted exosomes and soluble factors and found that some cytokines were highly secreted in the HFD group, which may be involved in metastatic cell homing. In addition, we found that tumour-secreted exosomes home to adipose tissue depots and are uptaken by AD-MSCs. Particularly, AD-MSCs from HFD mice increased their ability to uptake exosomes *in vivo. In vitro* analysis suggests that tumour-derived exosomes from highly malignant models impair lipid accumulation in AD-MSCs.

**Summary/Conclusion**: Our data show that chemokines secreted by different adipose tissue depots may favour metastatic seeding. Moreover, we propose that tumour-secreted exosomes are a novel mechanism of communication between tumour and AD-MSCs impairing their function and reinforcing metastatic behaviour.

**Funding**: This work is supported by grants from the National Institutes of Health, Worldwide Cancer Research, WHRI Academy and “La Caixa – Severo Ochoa International PhD program”.

PS07.03

Use of tumour-secreted exosomes to define new biomarkers and targets to prevent malignant peripheral nerve-sheath tumour progression

Teresa González Muñoz ^1^; Angela Di Giannatale^2^; Claudia Savini^1^; Susana Garcia-Silva^1^; Alberto Benito-Martin^3^; Cristina Merino^1^; Héctor Peinado^1,3^

^1^Microenvironment and Metastasis Group, Molecular Oncology Program, Spanish National Cancer Research Centre (CNIO), Madrid, Spain; ^2^Department of Oncohematology, Bambino Gesù Children’s Hospital, IRCCS, Rome, Italy; ^3^Department of Pediatrics, Drukier Institute for Children’s Health and Meyer Cancer Center, Weill Cornell Medical College, New York, USA

**Background**: Malignant peripheral nerve sheath tumours (MPNSTs) are highly aggressive and metastatic sarcomas with poor prognosis commonly related to neurofibromatosis type 1 (NF1). Recent data demonstrate that tumour-microenvironment communication plays a crucial role in the progression of these tumours. While soluble factors have been described as the main communication mechanism in this crosstalk, the role of secreted exosomes in this scenario is completely unknown.

**Methods**: Exosomes from MPNST cell lines and from plasma of NF1 patients in different stages were isolated by ultracentrifugation methods. Exosome protein concentration was measured by BCA. Molecular signature from MPNST-derived exosomes was analysed by mass spectrometry. Endoglin levels were tested in plasma circulating exosomes by ELISA and in human NF1-related tumours by immunohistochemistry. A knockdown of endoglin was performed in the STS26T MPNST cell line and its influence on gene expression and signalling pathways was analysed by RNA-Seq and validated by qRT-PCR and Western blot. The effect of human anti-endoglin antibodies in tumour growth and metastasis was examined *in vivo*.

**Results**: The protein content of exosomes secreted by MPNST cell lines and circulating exosomes from NF1 patients was significantly increased compared to controls. Mass spectrometry analysis showed endoglin, a TGF-β co-receptor with an important function in angiogenesis, as one of the top candidates secreted by MPNST cells. Endoglin levels were significantly increased in circulating exosomes and in NF1-related tumours along the progression of the disease. Mechanistically, endoglin knockdown resulted in the downregulation of the BMP and MAPK/ERK signalling pathways in MPNST-derived cell lines. Endoglin knockdown also led to the downregulation of angiogenesis-related factors. Finally, human anti-endoglin antibodies significantly reduced MPNST tumour growth and lymph node metastasis *in vivo*.

**Summary/Conclusion**: Our data suggest that analysis of circulating exosomes in NF1 patients could be useful for early detection of the progression of the disease and support the use of endoglin as a new MPNST biomarker and a potential therapeutic target to block the progression of these tumours.

**Funding**: This work is supported by grants from U.S. Department of Defense and Asociación de Afectados de Neurofibromatosis de España.

PS07.04

Contribution of Ral GTPases dependent extracellular vesicles to lung metastasis

Shima Ghoroghi ^1^; Olivier Lefebvre^1^; Annick Klein^1^; François Delalande^2^; Christine Carapito^2^; Vincent Hyenne^3^; Jacky Goetz^1^

^1^INSERM U1109, Strasbourg, France; ^2^LSMBO, Institut Pluridisciplinaire Hubert Curien, Strasbourg, France; ^3^INSERM U1109/CNRS SNC5055, Strasbourg, France

**Background**: Extracellular vesicles (EVs) contribute to tumour progression and metastasis by mediating the communication between tumour and stromal cells. Notably, tumour EVs have been shown to modify the microenvironment at distance from the primary tumour, creating a premetastatic niche where tumour cells can more easily settle. We have recently shown that two related GTPases, RalA and RalB contribute to EVs secretion in nematodes as well as in mouse mammary tumour cells (4T1 cells) (Hyenne et al., 2015).

**Methods**: Here, we investigate the implication of the Ral-dependent EVs in tumour progression and metastasis formation in mice.

**Results**: We first observed that both RalA and RalB are present in EVs secreted by 4T1 cells. 4T1 cells depleted for RalA or RalB have defects in late endosomal compartments (visualized by electron microscopy) and display distinct EV protein content (analysed by mass spectrometry), which suggest a role for RalA/B in EV biogenesis. To investigate the role of these GTPases in tumour progression and metastasis formation, we realized orthotopic injections in immuno-competent mice. We show that although cells depleted for RalA or RalB have different effects on primary mammary tumour growth, they both have reduced capacity of triggering lung metastasis. Preliminary observations suggest that neither RalA, nor RalB, affect tumour cell migration. Thus, to assess whether this phenotype could be related to differences in EV secretion or content, we investigated the functional properties of RalA/B dependent EVs. We observed that 4T1 EVs promote permeability of endothelial cells monolayer *in vitro*, and that this phenotype is impaired when 4T1 cells are depleted for RalA or RalB. We then used non-invasive whole-animal imaging to validate this observation *in vivo* where tumours depleted of RalA/B display a reduced vascular permeability.

**Summary/Conclusion**: Altogether, our results show that RalA and RalB GTPases contribute to metastasis formation, possibly through the release of EVs, which promote local vascular permeability.

PS07.05

Extracellular vesicles from metastatic medulloblastoma cell lines carry mRNAs known to correlate with metastatic disease

Hannah K. Jackson ^1^; Franziska Linke^1^; Ian Kerr^2^; Beth Coyle^1^

^1^The University of Nottingham School of Medicine, Nottinghamshire, UK; ^2^The University of Nottingham School of Life Sciences, Nottingham, UK

**Background**: Medulloblastoma is the most common malignant paediatric brain tumour. Over one-third of tumours are metastatic at diagnosis and almost all patients have metastases at relapse. Currently, there is no curative treatment for patients with metastatic medulloblastoma, which is underdiagnosed by current techniques. To improve diagnosis and treatment a greater understanding of the mechanisms of metastasis is required, as is the development of newer diagnostic methods. We hypothesized that extracellular vesicles (EVs) may play a role a medulloblastoma metastasis.

**Methods**: EVs were isolated from medulloblastoma cell lines using an optimized ultracentrifugation isolation method. Validation was by nanoparticle tracking analysis, transmission electron microscopy and Western blotting. The RNA cargo of the isolated EVs was analysed for the expression of medulloblastoma metastasis-associated genes c-Met, ABCB1, MMP 2, EMMPRIN and ITG-A9 by qRT-PCR. Immunofluorescence was also used to analyse the distribution of the corresponding proteins.

**Results**: Multistep centrifugation, filtration and ultracentrifugation results in highly purified EV preparations. Our data demonstrate that medulloblastoma cells secrete two distinct populations of exosomes and microvesicles, with unique size, morphology and cargo. We have also shown that more aggressive, metastatic cell lines produce significantly higher quantities of exosomes compared with less aggressive, non-metastatic cell lines. Finally, we have identified that candidate genes of medulloblastoma metastasis; c-Met, ABCB1, ITGβ1, MMP2 and EMMPRIN are present in both exosomes and microvesicles.

**Summary/Conclusion**: This study provides new insights on medulloblastoma extracellular vesicles. Our results indicate that mRNA of metastasis-associated genes is passed from the parent cells to exosomes and microvesicles. Thus, extracellular vesicles are potential diagnostic biomarkers for medulloblastoma patients.

**Funding**: This study was funded by Children’s Brain Tumour Research Centre – Life Cycle; James Tudor Foundation; School of Life Sciences, University of Nottingham.

PS07.06

Snail modulates extracellular vesicles-mediated interleukin release by cells constituting premetastatic niche in human colorectal cancer

Izabela Papiewska-Pajak ^1^; Patrycja Przygodzka^1^; Sylwia Michlewska^2^; Damian Krzyżanowski^1^; Joanna Boncela^1^; M. Anna Kowalska^1^

^1^Institute of Medical Biology of Polish Academy of Sciences, Lodz, Poland; ^2^University of Lodz, Lodz, Poland

**Background**: Extracellular vesicles (EVs), that include microvesicles (MV) and exosomes, from tumour cells has been considered messengers in intercellular communication, mediate the formation of metastatic niches and affect cancer progression. Colorectal cancer (CRC) is the third most common cancer worldwide. Also, involved in cancer progression is Snail, a key transcription factor of the epithelial–mesenchymal transition (EMT). We established the clones of human CRC HT29 cell line that express Snail and investigated the Snail effect on the pro-metastatic function of EVs released by those clones.

**Methods**: We isolated EVs from conditioned media using differential centrifugation and ultracentrifugation and characterized them by transmission electron microscopy (TEM) and Western blotting (WB). The exosomes and MVs were labelled using PKH67 dye to examine their uptake into human endothelial cells (HUVECs) and monocyte/macrophage cell line (THP-1). In order to quantify the amount of cytokines secreted by HUVECs and THP-1 cytometric bead array (CBA) kit was used.

**Results**: We confirmed the identity of exosome and MV fractions of EVs by TEM. CD63 marker but not cytochrome c was present on EVs as judged by WB that confirms the purity of vesicles. EVs released by control HT29 and Snail-overexpressing HT29 clones were incorporated into HUVECs and THP-1. Secretion of interleukin (IL)-8, from those cells was augmented in the presence of Snail-overexpressing HT29 EVs as compared to EVs form control HT29 clone.

**Summary/Conclusion**: We found that the EVs from Snail-overexpressing HT29 cells that were incorporated into the cells constituting premetastatic niche, significantly increased release of IL-8, a chemokine that has pro-angiogenic and pro-inflammatory properties. It confirms the role of Snail and provides inside into the mechanism by which Snail and EVs contribute to modification of premetastatic niches.

**Funding**: This study was supported by the project DEC-2011/02/A/NZ3/00068 from the National Science Center, Poland.

PS07.07

Quantitative proteomics of extracellular vesicles derived from isogenic metastatic and non-metastatic breast cancer in mice models

Jae Won Oh^1^; Hye Won Jung^2^; Yi Rang Na^2^; Seung Hyeok Seok^2^; Kwang Pyo Kim ^3^

^1^Department of Applied Chemistry, The Institute of Natural Science, College of Applied Science, Kyung Hee University, Yongin, Republic of Korea, Seoul, Republic of Korea; ^2^Department of Microbiology and Immunology, and Institute of Endemic Disease, Seoul National University College of Medicine, Seoul, South Korea, seoul, Republic of Korea; ^3^Kyung-Hee University, Yongin, Republic of Korea

**Background**: Metastasis, a major cause of breast cancer-related mortality, is a complicate process that is a series of cascade requiring a lot of soluble factors as well as tumour-promoting stromal cells. Among these soluble factors, containing proteins and nucleic acids, are important determinants in intercellular communication and subsequent formation of microenvironment favourable to tumour. There has been much efforts to find key metastatic factors secreted in extracellular vesicles for elucidation of underlying mechanism as well as identification of effective therapeutic targets. Considering that cancer cells injected into mice along with extracellular vesicles become more aggressive due to interaction with other cells in tumour microenvironment, it is necessary to analyse the exosome from tumour cells *in vivo* rather than *in vitro* cell line.

**Methods**: In this study, we hypothesized that cancer-derived extracellular vesicles have a potential role in metastasis and thus cancer cells secrete extracellular vesicles differently depending on their metastatic potentials. Using fluorescent-labelled cancer cell of non-metastatic (67NR)/metastatic (4T1) mouse breast cancer, we selectively isolated cancer cells from primary tumour mass and analysed the proteomic profiling of primary cancer cell-derived extracellular vesicles. We performed quantitative proteomic analysis of prepared extracellular vesicles derived from breast cancer in mouse models using isobaric tag based tandem mass tag (TMT) and liquid chromatography coupled with tandem mass spectrometry (LC–MS/MS).

**Results**: We identified more than 3000 extracellular proteins and 154 significantly up-regulated proteins and 114 significantly down-regulated proteins in extracellular vesicles from 4T1 (*p* < 0.05). Interestingly, migration related pathways and factors are specifically up regulated in extracellular vesicles from 4T1. These results suggest that migration factors from extracellular vesicles play critical roles in intravasation through specific migration pathways.

**Summary/Conclusion**: Taken together, proteomic profiling of extracellular vesicles from non-metastatic/metastatic breast cancer cells leads to identification of possible non-invasive biomarkers and suggest the novel driving factors responsible for the macrophage polarization to facilitate metastasis.

PS07.08

Omental fat extracellular vesicles promote gastrointestinal cancer aggressiveness: a potential novel mechanism of peritoneal metastasis

Shelly Loewenstein ^1^; Anat Aharon^2^; Joseph M. Klausner^1^; Guy Lahat^1^

^1^Surgery Division, Tel Aviv Sourasky Medical Center, Tel Aviv, Israel; ^2^Rambam Health Care Campus, Haifa, Israel

**Background**: The peritoneal cavity and the omentum specifically is a common site for gastrointestinal (GI) cancer metastasis. To date, conventional systemic therapy is ineffective for the treatment of peritoneal metastasis originating from the pancreas, stomach or colon; therefore, peritoneal spread is considered an ominous event in the course of these diseases. The omentum is composed of adipose tissue bands that contain mainly adipocytes, but also consists of fibroblasts, vascular cells and immune cells. Extracellular vesicles (EVs) are nano-sized spherical vesicles that include exosomes and microparticles that are released from many cell types into the extracellular space. EVs play a major role in intercellular communication within the tumour microenvironment. Our aim was to study the effects of human omental fat EVs on GI cancer progression and omental metastases.

**Methods**: Adipose tissue explants were prepared from human omental fat of GI cancer patients and EVs were isolated from the conditioned medium using ultracentrifugation. EVs were characterized using nanoparticle tracking analysis (NTA) and transmission electron microscopy (TEM). The cell origin of the omental fat derived EVs was characterized by FACS analyses and their uptake by pancreatic and gastric cancer cells was determined by confocal microscopy and FACS analysis. EVs effects on on GI cancer growth and motility were evaluated.

**Results**: NTA and TEM demonstrated a homogeneous population of EVs. EV expression of adipocyte (Perlipin1), macrophage (CD14) and endothelial (CD62E) markers were observed. Interestingly, tumour markers such as EpCAM were also detected on omental fat EVs. Omental fat EVs were taken up by pancreatic and gastric cancer cells enhancing their proliferation, migration and invasion.

**Summary/Conclusion**: We have isolated and characterized for the first time EVs from human omental fat of GI cancer patients. Furthermore, we have identified the cell origin of these EVs within the omental fat demonstrating that adipocytes and macrophages are the main source of omental fat EVs. In addition, we have shown that omental fat secreted EVs enhance GI cancer proliferation and motility. Deciphering the mRNA, miRNA and protein profiles of omental fat, EVs is needed in order to further characterize molecular mechanisms involved in this unique crosstalk between fat and cancer cells.

PS07.09

CAF-derived exosomes remodel ECM by targeting lung fibroblasts via integrin α2β1 at the pre-metastatic niche

Tingjiao Liu ^1^; Jing Kong^2^

^1^College of Stomatology, Dalian Medical University, Dalian, China (People’s Republic); ^2^Dalian Medical University, Dalian, China (People’s Republic)

**Background**: Carcinoma-associated fibroblasts (CAFs) contribute to metastasis by modifying the primary tumour microenvironment. It remains to be determined whether CAFs can promote metastasis through remodelling of the microenvironment in distant organs. We hypothesized that intercellular communication between CAFs and secondary organs is critical for metastatic progression. Salivary adenoid cystic carcinoma (SACC) is an ideal tumour model to study lung metastasis, which constitutes about 75% of the total metastases.

**Methods**: CAF cells were isolated from the SACC tumour tissue. A SACC cell line with high lung metastatic ability, SACC-LM, was also used in this study. Exosomes were isolated and their morphology was confirmed by TEM, Western blot and NTA analysis. BALB/c nude mice and C57BL/6J mice were used in this study.

**Results**: Here, we show that CAF-derived exosomes (CAF-Exo) induced lung pre-metastatic niche formation in mice and consequently increased lung metastatic colonization. CAF-Exo presented a greater ability for extracellular matrix remodelling than tumour exosomes. POSTN is a potential biomarker characterizing the CAF-Exo-induced pre-metastatic niche. We found that integrin α2β1 mediated CAF-Exo uptake by lung fibroblasts, and its blockage prevented lung pre-metastatic niche formation. Integrin β1 expression was considerably higher in plasmal exosomes from mice transplanted with CAFs. In human SACC tumour stroma, integrin β1 showed higher expression in patients with lung metastasis than those without lung metastasis. These data suggest that exosomal integrin β1 may be a prognostic marker of SACC lung metastasis.

**Summary/Conclusion**: Our study provides new insight into pre-metastatic niche formation and prompts the development of an integrated strategy targeting both tumour and stromal cells to prevent metastasis at an early stage.

**Funding**: This study was funded by National Natural Science Foundation of China (No. 81171425).

PS07.10

The crosstalk between human osteosarcoma and endothelial cells *in vitro* through tumour-derived extracellular vesicles

Alekhya Mazumdar; Ana Gvozdenovic

Department of Orthopaedics, Balgrist University Hospital, Zürich, Zürich, Switzerland

**Background**: Osteosarcoma (OS) is the most common primary malignant bone tumour in children and adolescents with a high propensity for pulmonary metastases, the major cause of death in patients. Hence, understanding the metastatic process is critical for combating patient mortality. It has been demonstrated in several carcinoma models that tumour-derived extracellular vesicles (EVs) mediate metastasis by educating distant sites towards a supportive metastatic microenvironment. Interaction between the cancer cells with the endothelium is crucial for metastasis. It is well established that an activated endothelium in metastatic prone sites positively correlates with increased tumour cell homing, adhesion and metastasis. Here, we hypothesized that OS cells communicate with endothelial cell via EVs. We therefore investigated the *in vitro* uptake and subsequent effects of OS-derived EVs on the activation of endothelial cells and tumour cell-endothelium adhesion.

**Methods**: EVs were purified from cell culture supernatants of highly metastatic 143-B human OS cells using standard differential centrifugation. HUVEC monolayers were treated with PKH67 labelled EV and EV internalization was assessed by flow cytometry. mRNA expression of endothelial activation markers was examined using RT-qPCR. *In vitro* adhesion assay was employed to study tumour cell adhesion to EV-treated HUVECs.

**Results**: HUVECs efficiently internalized 143-B-derived EVs in a time and concentration-dependent manner. EV uptake HUVECs lead to upregulation of adhesion molecules E-selectin and ICAM-1 when compared to their mRNA levels in untreated control. Additionally, pretreatment of HUVECs with EVs resulted in a concentration-dependent increase of tumour cell adhesion to HUVEC.

**Summary/Conclusion**: Our *in vitro* experiments showed that 143-B OS-originating EVs activate HUVEC cells and enhance tumour cell–endothelial cell adhesion. These results underscore the importance of EVs in facilitating the intercellular communication between OS cells and endothelial cells thus fostering pre-metastatic vasculature and promoting tumour cell binding to vessel walls, a critical step required for disseminated tumour cells to form distant metastatic colonies. Identification of EV factors contributing to the pre-metastatic niche foundation could open new avenues in OS management.

PS07.11

The effect of chemotherapy induced intercellular communication on breast cancer metastasis

Paschalia Pantazi; David R F. Carter; John Runions; Susan A Brooks

Oxford Brookes University, Oxford, UK

**Background**: Breast cancer is among the most common types of cancer for women and the primary cause of cancer related death. The high mortality rates are due to the metastatic spread of cancer cells and tumour recurrence after therapy. Transferring their cargo from one cell to another, EVs (extracellular vesicles) are involved in maintaining homeostasis in normal physiology, but are deregulated in cancer. EVs have been shown to play particular roles in all hallmarks of cancer with great focus given on the various steps of the metastatic cascade. The aim of this project is to investigate the effect of chemotherapy induced intercellular communication via EVs on breast cancer metastasis.

**Methods**: Two chemotherapeutic agents commonly used in breast cancer therapy regimens, docetaxel and mitomycin C were employed in this study. Metastatic potential after incubation with EVs derived from drug treated cells has been assessed by labelling with the glycosylation marker HPA (helix pomatia agglutinin), expression analysis of EMT (epithelial to mesenchymal transition) markers, motility and invasion assays.

**Results**: EVs from drug treated cells altered the glycosylation patterns of recipient cells as revealed by HPA labelling, while EVs from non-treated cells showed no effect. EVs from docetaxel treated cells enhanced invasiveness and motility of recipient cells and lowered the expression of the epithelial marker CDH1.

**Summary/Conclusion**: These results suggest that cells that have survived chemotherapy release EVs that are able to enhance the metastatic capacity of intact cells.

PS07.12

Role of exosomes in liver cancer metastasis

Sze Keong Tey; Xiaowen Mao; Wai Ping Yam

The University of Hong Kong, Pokfulam, Hong Kong

**Background**: Hepatocellular carcinoma (HCC) is a primary malignancy of liver. HCC is often diagnosed at an advanced stage accompanied by extrahepatic metastasis. Despite the studies on extrahepatic metastasis in HCC carried out over the years, the precise mechanistic basis of HCC metastasis has not been fully explained. Emerging evidences have demonstrated cancer cells derived exosomes play an important role in influencing the local tumour microenvironment and forming pre-metastatic niche in distant organ sites. Thus, exosome research may bring new hope to solve the mystery of metastatic organotropism in HCC.

**Methods**: Exosomes were isolated from the conditioned medium of different cell lines by ultracentrifugation and validated by transmission electron microscopy, nanoparticle tracking analysis and immunoblotting of exosome markers. The biological effects of exosomes were studied using transwell and matrigel invasion assays. The *in vivo* effect of exosomes in promoting liver tumour growth and distant metastasis were analysed in mice “educated” with repeated intravenous injection of exosomes. In lung metastatic site, the pulmonary vasculature and vascular leakiness were revealed by FITC-lectin stain and presence of Texas Red-dextran respectively.

**Results**: Exosomes were isolated and a higher amount of exosome protein was obtained from metastatic MHCC97L and MHCCLM3 cells when compared to normal liver cell MIHA and non-metastatic BEL7402 cells. Functionally, exosomes of metastatic cells significantly augmented both the migratory and invasive properties of naïve normal liver cells LO2 and non-metastatic SMMC7721 and BEL7402 cells. Exosome “education” of mice by MHCC97L-exosomes enhanced the growth of primary tumour in liver and distant metastasis to lungs. Analysis of exosome organotropism showed that fluorescently labelled MHCC97L-exosomes were frequently observed in the lung as compared to other organ sites. Further study of vascular permeability in the lung revealed MHCC97L-exosomes destabilized the pulmonary vasculature and enhanced the endothelial permeability of lung. Proteomic profiling of MIHA- and MHCC97L-exosomes revealed distinct expression profiles.

**Summary/Conclusion**: Exosomes plays a crucial role in the interplay between HCC cells and microenvironment of the distant organ that enhances liver cancer metastasis.

PS07.13

Extracellular vesicles contained in malignant ascites contribute to progression of high grade serous ovarian carcinoma

Vendula Pospíchalová ^1^; Anna Kotrbová^1^; Zankruti Dave^1^; Eva Jandáková^2^; Markéta Bednaříková^3^; Luboš Minář^2^; Vít Weinberger^2^; Vítězslav Bryja^1^

^1^Institute of Experimental Biology, Faculty of Science, Masaryk University, Brno, Czech Republic, Brno, Czech Republic; ^2^Department of Obstetrics and Gynecology, University Hospital Brno, Czech Republic, Brno, Czech Republic; ^3^Department of Internal Medicine – Hematology & Oncology, University Hospital Brno and Medical Faculty, Masaryk University, Czech Republic, Brno, Czech Republic

**Background**: High-grade serous ovarian carcinoma (HGSOC) is the most frequent form of ovarian cancer and the deadliest gynaecologic malignancy worldwide. HGSOC is often associated with ascites (a pathologically accumulated fluid in the peritoneum), so far an undervalued source of primary tumour tissue as well as complex tumour microenvironment. Ascites contains numerous types of cells, extracellular vesicles (EVs) and proteins that in combined fashion regulate tumour growth and spreading. However, the molecular details on how EVs regulate HGSOC progression remain largely unknown.

**Methods**: We generated a model of “negative approach” by using ascitic fluids differentially depleted from none, one or both types of EVs (exosomes and microvesicles) by ultracentrifugation and filtration. This approach yields more precious patient material to be available for experiments.

**Results**: HGSOC cells treated with ascites had increased (cancer) stem cells characteristics and migratory/invasive potential. These effects were diminished or completely lost, if the ascitic fluid had been depleted from exosomes and/or microvesicles. Thus we isolated and thoroughly characterized ascitic extracellular vesicles and we aim to investigate how they alter crucial cancer cell behaviours.

**Summary/Conclusion**: Our pilot data indicate that EVs contained in malignant ascites may play important role in the acquisition of metastatic stem cell-like characteristics of HGSOC cells, yet EVs are differentially required for various aspects of the complex metastatic stem cell like behaviour. We believe this project will deepen our knowledge about molecular mechanisms of HGSOC progression, which is an imperative for better management of this devastating disease in future.

**Funding**: This study was funded by Czech Science Foundation under Grant. No. 16-16508Y.

PS07.14

Heat-shock factor 2 associates with cancer-derived extracellular vesicles

Eva Henriksson; Jens Luoto; Lea Sistonen

Faculty of Science and Engineering, Åbo Akademi University, Finland, Turku, Finland

**Background**: The heat-shock factors (HSF1–4) are transcription factors essential for cellular stress responses and mammalian development. They have also roles in human pathologies, as HSF1 supports malignant growth and HSF2 suppresses cancer invasion. We have previously reported that in the prostate cancer cell line PC3, HSF2 protein levels decline in three-dimensional (3D) organotypic cultures as the organoids become invasive. Silencing of HSF2 accelerates the invasive transformation. The underlying molecular mechanisms are however unclear. We hypothesize that HSF2 is released from cancer cells in extracellular vesicles (EVs) in a cancer-invasion dependent manner.

**Methods**: EVs were isolated from the growth media of PC3 and human U2OS osteosarcoma cells by differential ultracentrifugation or iodixanol density gradient fractionation and analysed by Western blotting. The same cell lines, as well as HSF2-knockout U2OS cells generated using CRISPR-Cas9 technique, were cultivated in Matrigel to produce 3D organotypic cultures. The extracellular proteins were visualized with immunofluorescence microscopy.

**Results**: We found PC3 and U2OS cells to release HSF2 in 2D and 3D cell cultures. The extracellular HSF2 (eHSF2) was present in CD63 and CD81 positive density fractions prepared from 2D culture medium and in 3D cultures the eHSF2 co-localized with CD81 outside the organoids. The characterization of HSF2-carrying EVs is currently being conducted.

**Summary/Conclusion**: Our results show that HSF2 is secreted by cancer cells and the secretion could lead to low intracellular HSF2 levels, enabling an invasive organoid development. This might also apply to cancer patients, as low HSF2 levels in tumours correlate with poor survival.

PS07.15

Glioblastoma stem cells induce an invasive phenotype in normal astrocytes via extracellular vesicles

Susannah Hallal ^1^; Duthika Mallawaaratchy^1^; Heng Wei^2^; Michael Buckland^2^; Kimberley Kaufman^1^

^1^The University of Sydney, Sydney, Australia; ^2^Royal Prince Alfred Hospital, Sydney, Australia

**Background**: Glioblastoma (GBM) carries an exceedingly poor prognosis due to its highly invasive and recurrent nature. Astrocytes, non-malignant counterparts of GBM cells, become reactive around GBM tumours, with changes to their morphology, proliferation rates and motility. While interactions between tumour cells and astrocytes are important in GBM biology, the contribution of extracellular vesicle (EV) signalling is unknown. We aimed to understand how GBM-derived EVs affect normal primary astrocytes in order to better understand GBM intercellular communication and how this could support tumour progression.

**Methods**: EVs were isolated from culture supernatants of WK1, JK2, RN1 primary GBM “Stem” cells (NES+/CD133+) and differentiated (“Diff”) progeny cells (NES-/CD133-). EVs were characterized by transmission electron microscopy, nanosight tracking analysis and mass spectrometry (MS)-based protein profiling. The internalization of GBM-EVs by normal astrocytes was observed by DiI-labelling and fluorescence microscopy. A Cy3-gelatin podosome/invadopodia assay was used to observe changes to the migration and invasion patterns of normal astrocytes after exposure of the astrocytes to a range of GBM-EVs for 24 h. To understand the changes observed in astrocyte migration, we performed comprehensive quantitative MS-based proteomics. Using Ingenuity Pathway Analysis, an interaction network was generated from differentially abundant proteins and upstream regulators. Predicted changes in activation states were tested by qPCR.

**Results**: We observed a significant increase in podosome/invadopodia formation and Cy3-gelatin degradation by the normal astrocytes in response to the GBM stem- and GBM differentiated-EVs, with GBM stem-EVs eliciting a greater effect on the astrocytes. More than 1650 proteins were identified and quantified by mass spectrometry and bioinformatics predicted an upstream activation of FN1 and TGFB1 and inhibition of p53 in normal astrocytes exposed to GBM-EVs. qPCR studies confirmed predicted increases in RNA levels of FN1 and TGFB1 and a decrease in TP53 in GBM-EV exposed astrocytes.

**Summary/Conclusion**: The inhibition of TP53 signalling and the activation of FN1 and TGFB1 in normal astrocytes may be a mechanism by which GBM manipulates normal astrocytes to acquire a cancerous phenotype and aid GBM malignancy.

PS07.16

Role of exosomes in inducing neuroendocrine differentiation in advanced prostate cancer

Sharanjot Saini; Divya Bhagirath; Thao Yang; Shahana Majid; Rajvir Dahiya; Yuichiro Tanaka

SFVAMC and UCSF, San Francisco, USA

**Background**: Neuroendocrine prostate cancer (NEPC) is an aggressive variant of advanced prostate cancer (PCa) present in ~30% of metastatic castration-resistant tumours, often emerging as a result of AR-targeted therapies such as enzalutamide, via neuroendocrine differentiation (NED). Owing to NED, tumours show neuroendocrine (NE) features with the expression of neuronal markers such as enolase 2 (ENO2), chromogranin A (CHGA) and synaptophysin (SYP). Clinically, NEPC manifests as the presence of visceral metastatic disease, low serum PSA levels relative to disease burden and limited response to AR signalling inhibitors. The molecular basis of NED/NEPC is poorly understood. We propose that in addition to cell intrinsic genetic determinants of NED, tumour exosomes are important to facilitate neuroendocrine differentiation of prostate tumours via horizontal transfer of functional NE factors and regulatory microRNAs (miRNAs) to recipient cells.

**Methods**: Exosomes were isolated from cell culture models of PCa NED followed by (i) small RNA-next generation sequencing (NGS) and (ii) Western blot analyses for oncogenic factors to identify novel regulators that play a role in exosome-mediated intercellular communication underlying NED. Exosome isolation reagent was used for exosome isolation as per manufacturer’s instructions. The integrity of exosomal preparations was confirmed by nanoparticle tracking analysis (NTA). cDNA libraries were generated from purified small RNA (100 ng), index libraries were equally pooled and sequenced. Further, *in vitro* “uptake experiments” were performed with labelled exosomes isolated from neuroendocrine cell line models and incubation with parental LNCaP cells followed by gene expression profiling by real time PCR and immunoblot analyses.

**Results**: Key oncogenic NE factors and oncogenic miRNAs are released in exosomes by cancer cells induced to undergo neuroendocrine differentiation. These oncogenic factors, via their exosomal uptake, act in a paracrine manner on neighbouring non-NE cancer cells leading to initiation of NE-like alterations.

**Summary/Conclusion**: Exosome-mediated intercellular communication is crucial to induction of neuroendocrine differentiation states in prostate cancer.

**Funding**: This study was supported by Grant Number RO1CA177984, U01CA184966.

PS07.18 = OWP1.04

Cancer-derived extracellular vesicles facilitate osteoclast fusion and differentiation via enhancing filopodia formation in osteoclast precursors

PS07.19

Different expression patterns of exosomal miRNAs under Cyclosporin A and Rapamycin treatment in distinct aggressiveness colorectal carcinomas

Valeria Tubita ^1^; Maria Jose Ramirez-Bajo^2^; Juan Jose Lozano^3^; Daniel Moya Rull^4^; Jordi Rovira^1^; Elisenda Banon-Maneus^2^; Josep M Campistol^5^; Fritz Diekmann^5^; Ignacio Revuelta^5^

^1^IDIBAPS, Barcelona, Spain; ^2^Fundació Clínic per a La Recerca, Barcelona, Spain; ^3^CIBEREHD, Barcelona, Spain; ^4^Laboratori Experimental de Nefrologia i Trasplantament (LENIT), Barcelona, Spain; ^5^Hospital Clínic de Barcelona, Barcelona, Spain

**Background**: The aggressiveness of colorectal cancer in kidney transplantation is much more significant than the increase in its incidence compared with the general population. The molecular mechanism of the different immunosuppressive drugs with antagonistic antineoplasic properties is not well characterized. Exosomes (Ex) are involved in tumour immunity, angiogenesis and metastasis. Exosomal miRNAs can affect expression of a variety of target genes. Our aim is to investigate if distinct colon cancer cells treated with Cyclosporin A (CsA) and Rapamycin (RAPA) can induce the production of different Ex content that could explain the cancer progression in kidney transplantation.

**Methods**: Metastatic (HCT116) and non-metastatic (SW480) cells were treated with CsA and RAPA. Ex isolation was made by differential ultracentrifugation and Ex characterization by nanoparticle tracking analysis, flow cytometry and electron microscopy. A comparative miRNAs profile by Affymetrix miRNA 4.1 Array Strips was made in miRNAs isolated from Ex released from conditioned cells under CsA and RAPA.

**Results**: Bioinformatics analysis indicated that miR6787-5p, miR6746-5p and miR6127 were common and highly differentially expressed in Ex from HCT116 after both drugs. These miRNAs were down-regulated by CsA and up-regulated by RAPA in Ex from HCT116. These data were not shown in SW480 derived Ex. We identified putative target genes related to the miRNAs implicated in the NOTCH signalling and in immunoregulatory/inflammatory processes.

**Summary/Conclusion**: In conclusion, CsA and RAPA induce a different exosomal miRNAs expression pattern in metastatic cancer cell line, not evidenced in non-metastatic cells. Exosomal miRNAs could be a potential biomarker in cancer progression and metastasis.

**Funding**: FP7-PEOPLE: BIOTRACK (229673). PROFESSIONAL TRAINING AND CAREER DEVELOPMENT IN BIOMEDICINE. Marie Curie Action. Redes Tematicas De Investigacion Cooperativa En Salud, REDINREN (RD12/0021/0028 and RD16/0009/0023) both co-funded by ISCIII-Subdirección General de Evaluación and Fondo Europeo de Desarrollo Regional (FEDER) “Una manera de hacer Europa”.

PS08: EVs as Cancer Biomarkers Chairs: Yiyao Huang; Irina Nazarenko Location: Exhibit Hall 17:15–18:30

PS08.01

Comparing the efficiency of exosomes isolation from prostate and pancreatic cancer cell lines using size exclusion chromatography and ExoQuick-TC

Mei Yieng Chin ^1^; Emma Guns^1^; Jessica Kalra^2^

^1^Vancouver Prostate Centre, Vancouver, Canada; ^2^BC Cancer Research Cetre, Vancouver, Canada

**Background**: Both prostate cancer (PCa) and pancreatic ductal adenocarcinoma (PDAC) continue to demonstrate poor outcomes due to late stage diagnosis. Research has concentrated on finding biomarkers for early detection while the cancer is still localized and amenable to therapy; however, these markers remain elusive. Exosomes are quickly becoming a prominent tool in biomarker research and show promise in the development of liquid biopsies for early screening programmes. We believe that different subtypes of PCa and PDAC, particularly more aggressive forms of the disease, produce unique exosome subtypes that can be characterized by exosomal protein and nucleic acid profiles. In order to develop a robust understanding of the nature of exosome subtyping, an optimized exosome isolation method is required. The studies described focus on characterizing exosomes collected from the conditioned media of PCa (PC3, 22RV1 and LNCAP), normal pancreatic exocrine and PDAC (PANC-1, BxPC3 and MIAPaCa-2) cell lines.

**Methods**: We compare the efficiency of two methods of isolation: size exclusion chromatography (SEC) and ExoQuick-TC (EQ). The morphology and size of exosomes was characterized by transmission electron microscopy (TEM). The size and relative abundance of exosomes collected using each method was quantified by NanoTracking Analysis (NTA). Protein was purified from exosomes and Western blots were performed to assess the level of expression of exosome markers.

**Results**: The size of exosomes isolated by each method and across cell lines was similar (60–130 nm); however, the quality of exosomes isolated was better when using SEC compared to EC. Standardized protein markers for exosome isolation (CD81, CD9, CD63, ALIX and HSP70) showed significant variability across cell lines indicating that cancer subtypes produce exosomes with unique protein profiles.

**Summary/Conclusion**: Future research will translate these results to clinical samples from urine and serum for comparison.

**Funding**: This study was funded by Pancreas Centre British Columbia Seed Funding and Canadian Institution for Health Research.

PS08.02

Identifying potential biomarkers for lung cancer from the cancer derived exosomes using the nano-gap-mode surface-enhanced Raman scattering (SERS)

Wei-Lun Huang ^1^; Kundan Sivashnamugan^2^; Ten-Chin Wen^2^; Wu-Chou Su^1^

^1^Department of Internal medicine, National Cheng Kung University Hospital, College of Medicine, National Cheng Kung University, Tainan, Taiwan (Republic of China); ^2^Department of Chemical Engineering, National Cheng Kung University,, Tainan, Taiwan (Republic of China)

**Background**: Exosomes have been shown to play important roles in many diseases including lung cancer. Thus, the exosomes could be good targets for identifying potential biomarkers for the related disease. In this study, we tried to find out the lung cancer biomarkers using a novel nano-gap-mode surface-enhanced Raman scattering (SERS) from lung cancer derived exosomes.

**Methods**: EVs were isolated from culture supernatants using ultra-centrifugation and ultra-filtration and then evaluated by TEM, Western blot analysis and Nanosight. The biomarkers identification was done using SERS.

**Results**: Here, we used an Ag nanocubes (NCs) on an Au nanorod (NR) array substrate with a large density of hot-ring area to construct the nano-gap-mode SERS which is suitable for the size of exosomes. Using this system, a strong plasmonic cavity effect was obtained and the SERS signals of the exosomal biomolecule composition could be sensitively detect at concentrations 10e4–10e5 times lower than that of normal blood samples. In addition, the sample requirement is much less than the traditional characterization techniques (5 µl of diluted exosome samples), which makes it suitable for clinical applications. In this study, we found that the exosomes derived from non-malignant cell lines showed stronger SERS signals of nucleic acid and lipids, whereas exosomes derived from lung cancer cell lines exhibited stronger SERS signals of protein.

**Summary/Conclusion**: The nano-gap-mode constructed by the attachment of Ag NCs on the HR area of Au NRs which is suitable for the size of exosomes should be the key for enhancing the electromagnetic effect and thus the SERS signal of exosomes. In this study, our preliminary data in lung cancer showed that the novel nano-gap-mode SERS based method with high sensitivity and minimal sample requirement make it suitable for identifying exosomal biomarkers.

**Funding**: This work was supported by DOH 102-TD-PB-111-NSC101 and MOHW 105-TDU-PB-211-000006 from the Ministry of Health and Welfare, Taiwan, NSC 103-2120-M-006-006 and MOST 104-2314-B-006-046-MY3 from the Ministry of Science and Technology, Taiwan.

PS08.03

Characterization of extracellular vesicles using Raman spectroscopy for label-free cancer detection

Wooje Lee ^1^; Afroditi Nanou^1^; Linda Rikkert^2^; Frank A.W. Coumans^3^; Cees Otto^1^; Leon Terstappen^4^; Herman Offerhaus^1^

^1^University of Twente, Enschede, The Netherlands; ^2^Department of Medical Cell Biophysics, University of Twente, Enschede, The Netherlands, Amsterdam, The Netherlands; ^3^Department of Biomedical Engineering and Physics, and Vesicle Observation Center, Academic Medical Centre of the University of Amsterdam, Amsterdam, The Netherlands; ^4^Department of Medical Cell BioPhysics, University of Twente, Enschede, The Netherlands, Enschede, The Netherlands

**Background**: Extracellular vesicles (EVs) enable intercellular communication by transporting a wide range of biomolecules. The transported biomolecules vary depending on the origin of the EVs. This implies that the EVs derived from different origins have a distinct chemical composition and signature. This signature might in turn be used as a biomarker to detect diseases. Raman spectroscopy is a type of vibrational spectroscopy that is based on inelastic scattering by molecules. It allows us to investigate spectral fingerprint of chemicals. In this work, we demonstrated the potential of EVs as a cancer biomarker using Raman spectroscopy.

**Methods**: Four EV subtypes were prepared; two subtypes were derived from blood products of healthy donors (red blood cell and platelet) and two others were derived from prostate cancer cell lines (LNCaP and PC3). Raman optical tweezer allows the capturing of vesicles at the waist of the focused laser beam. Excitation beam (*λ* = 647 nm) was focused onto the sample to capture EVs and to obtain Raman fingerprint of EVs. The power of the beam was 50 mW under the objective. The exposure time per spectrum was 10 s and 16 spectra were obtained at the fixed position.

**Results**: Since the spectral differences among EV subtypes are small, a multivariate analysis method called principal component analysis (PCA) was conducted on the spectral fingerprints of the samples. The Raman spectra in the range of 400–1800/cm (654 data points) were selected for the analysis. PCA scores separate about 98% of the prostate cancer-EVs from the healthy group.

**Summary/Conclusion**: We have explored spectral differences between cancer- and healthy cell-derived EVs to figure out the potential EV as a cancer biomarker. Raman spectroscopy was employed to obtain the spectral fingerprint of EV subtypes. The result of multivariate analysis shows the spectral differences between healthy cells derived EVs and prostate cancer cell-derived EVs. The result shows that more than 90% of EVs can be separated into the two categories. This result shows the clear discrimination of these two groups based on their spectral fingerprints and the potential of EVs as a cancer biomarker.

**Funding**: This work is financed by The Netherlands Organization for Scientific Research (NWO).

PS08.04

Single cancer cell detection using microflow cytometry and ultrasound-mediated extracellular vesicle release

Robert J. Paproski; Roger J. Zemp; John D. Lewis

University of Alberta, Edmonton, Canada

**Background**: Circulating tumour cells (CTC) have significant prognostic value for various cancers. Extracellular vesicles (EVs) have also shown prognostic value for some cancers although estimating CTC burden using circulating EVs can be difficult since it is unknown if detected EVs originated from CTCs or tumours. Since ultrasound can stimulate EV release ~100-fold (Cancer Res. 2017;77:3–13), we hypothesize that CTCs could be estimated by determining the increase in cancer-related EVs in post-sonicated samples using microflow cytometry. This would allow normalization of EVs for each patient using pre-ultrasound samples as well as provide high sensitivity since a single CTC could generate hundreds EVs compared to a single event with cell-based flow cytometry.

**Methods**: In PCR tubes, 1,000,000 HT1080 cells (representing background cells) and approximately 1000, 100, 10, 5 and 1 PC3 prostate cancer cell(s) expressing palmitoylated green fluorescent protein (PALM-GFP) were mixed in 200 µL culture growth medium. Cells were centrifuged and 75 µL supernatant pre-ultrasound samples were taken followed by cell resuspension with 2% (v/v) albumin microbubbles. Cells were exposed to 60 s of high pressure ultrasound, centrifuged, 75 µL supernatant post-ultrasound samples were taken, and samples were analysed with an Apogee A50 cytometer.

**Results**: Mean PALM-GFP+ particles increased 4-, 40-, 80-, 490- and 2300-fold in samples containing 1, 5, 10, 100 and 1000 PC3 PALM-GFP cells respectively (*p* < 0.05 for all groups). Log transformed data showed a linear correlation between the number of PC3 PALM-GFP cells and PALM-GFP+ particles (*r* ^2^ = 0.93).

**Summary/Conclusion**: Our technique demonstrated single cancer cell detection sensitivity even when only analysing 6% of the post-ultrasound sample volume. This technique could be added to conventional cancer EV-based assays for a more comprehensive analysis of patient biofluids using the same microflow cytometry platform.

PS08.05

Characterization of subpopulations of circulating extracellular vesicles by imaging flow cytometry

Franz Lennard. Ricklefs ^1^; Cecile Maire^1^; Katharina Kolbe^1^; Mareike Holz^1^; Rudolph Reimer^2^; Markus Glatzel^3^; Ennio Chiocca^4^; Eva Tolosa^1^; Manfred Westphal^1^; Katrin Lamszus^1^

^1^University Medical Center Hamburg-Eppendorf, Hamburg, Germany; ^2^Heinrich-Pette-institut, Hamburg, Germany; ^3^University Medical Center Hamburg Eppendorf UKE, Institute for Neuropathology, Hamburg, Germany; ^4^Harvard Medical School, Brigham and Women’s Hospital, Boston, USA

**Background**: EVs are commonly characterized by nanoparticle analysis (NTA), electron microscopy and immunoblot detection of vesicle markers (i.e. CD9, CD81, CD63, Annexin V). It is unclear, however, to what extent marker profiles overlap and how useful they are for distinguishing different cell types of origin. With the goal of defining markers that allow enrichment of cancer EVs from patient blood, we utilized imaging flow cytometry (IFC) to discriminate single EVs via multiple surface markers.

**Methods**: EVs were isolated from blood of cancer patients (*n* = 25), healthy controls (*n* = 20), PALM-GFP-GL261 and PALM-GFP-CT2A brain tumour-bearing mice (*n* = 5), cancer cell cultures (*n* = 12), neural stem cells (NSC), cerebral endothelial cells (cEC) and T-cells (*n* = 4). EVs were analysed by IFC, immunoblotting, electron microscopy and NTA.

**Results**: IFC allows the detection of up to four different markers on single EVs sized <200 nm, including CD9, CD81, CD63 and Annexin V, and allows the discrimination of different EV subpopulations present in human and murine plasma and in cell culture supernatants. Circulating plasma EVs in patients and controls as well as in mice are primarily CD9 positive, whereas CD81 and CD63 distinguish different subpopulations. Interestingly, cancer patients exhibit increased levels of circulating EV compared to aged-matched healthy controls (*p* < 0.001), as measured by NTA and IFC. In particular, double-positive EVs (i.e. CD9+/CD81+) are elevated in cancer patients (*p* = 0.018) vs healthy controls, whereas single-positive EVs are not. In accordance with these findings, cancer cell lines excrete increased levels of double positive EVs *in vitro*, whereas NSCs and cECs primarily produce CD9+ EVs, and T-cells predominantly release CD81+ EVs.

**Summary/Conclusion**: EVs can be characterized by IFC, a unique technique that facilitates the discrimination of different EV subpopulations. The identification and classification of different circulating EV populations is an essential step towards capitalizing the potential of tumour-derived EVs as biomarkers which are easily accessible by liquid biopsy.

PS08.06

Detection and characterization of apoptotic tumour cell-derived extracellular vesicles using Raman and surface enhanced Raman spectroscopy

Catherine Lynch ^1^; Karen Faulds^2^; Christopher D. Gregory^1^

^1^MRC Centre for Inflammation Research, University of Edinburgh, Edinburgh, UK; ^2^Centre for Molecular Nanometrology, University of Strathclyde, Glasgow, UK

**Background**: In certain cancer types, such as non-Hodgkin lymphoma, a high rate of apoptosis is a marker of poor prognosis due to the accumulation and proliferation of tumour-associated macrophages (TAMs). These TAMs can promote tumour cell proliferation, angiogenesis and tissue remodelling, and are activated to this phenotype by the apoptotic cells and, potentially, extracellular vesicles released from apoptotic cells (Apo-EVs).

Raman spectroscopy is a label-free, non-destructive vibrational spectroscopy technique in which laser light is inelastic scattered from a sample. This signal can be increased using roughened metal surfaces, such as gold or silver nanoparticles, and is known as surface enhanced Raman spectroscopy (SERS).

EVs from both apoptotic and non-apoptotic tumour cells were analysed by Raman and SERS with a view to developing a method to detect Apo-EVs as a diagnostic and prognostic marker of disease, as well as having potential to monitor treatment response.

**Methods**: Cancer cell lines were irradiated with UVB radiation to induce apoptosis. The EVs were isolated using a combination of low-speed centrifugation and filtration. EVs were characterized by Raman spectroscopy using a 532 nm laser and the spectra analysed by multivariate analysis. Gold nanoparticles conjugated with antibodies against plasma membrane proteins present on the EVs were used to detect the EVs using SERS.

**Results**: The EV spectra show characteristic bands for proteins, lipids and nucleic acids. Multivariate analysis shows a detectable difference between EV populations. SERS enables more targeted detection based on specific proteins present on the EV surface, allowing phenotyping analysis of the population.

**Summary/Conclusion**: This study shows that Raman spectroscopy is a useful technique for characterization of EVs in a label-free manner. Further, SERS can be used for targeted analysis of the EVs by conjugating antibodies to the metal nanoparticles, with facile multiplexing.

**Funding**: This study was funded by the EPSRC and MRC under grant number EP/L019559/1 (OPTIMA) and by the University of Edinburgh College of Medicine and Veterinary Medicine.

PS08.07

Electrochemical and optical biosensing for the detection of cancer exosomes from breast cancer cells

Silio Lima Moura; Mercè Martì; Maria Isabel Pividori

Grup de Sensors i Biosensors, Departament de Química, Universitat Autònoma de Barcelona, Barcelona, Spain

**Background**: The identification of novel biomarkers represents a worldwide challenge not only for the improvement of early diagnostics, but also for patient monitoring and for the evaluation of the efficiency of a therapeutic strategy. Exosomes are nano-sized and cup-shaped vesicles, which are currently under intensive study as potential diagnostic biomarkers for many health disorders, including cancer. This is a growing need for sensitive methods capable of accurately and specifically determining exosome concentration. This work addresses the study of different receptor by flow cytometry as well as the design of a quantitative and rapid method for total exosome counting based on magneto-actuated platforms with electrochemical and optical readout.

**Methods**: Two different strategies were explored for the magnetic separation of exosomes: (i) direct covalent immobilization on tosyl-activated magnetic particles or (ii) immunomagnetic separation by anti-CD9, -CD24, -CD63, -CD81 antibody-modified magnetic beads.

**Results**: Exosome counting by the magneto-actuated immunoassay with optical readout and magneto electrochemical biosensor was successfully achieved in human serum and offers outstanding results in analytical performance.

**Summary/Conclusion**: This proof-of-concept study as a rapid, cost-effective and high-sample-throughput detection of exosome can potentially establish for promising applications in cancer diagnostics.

**Funding**: Ministry of Economy and Competitiveness (MINECO), Madrid (Under grant BIO2016-75751-R) and Conselho Nacional de Desenvolvimento Científico e Tecnológico of the Ministry of Science, Technology and Innovation of Brazil (under grant 233595/2014-7) funded this study.

PS08.08

Droplet microfluidics enabled single-exosome-counting immunoassays for cancer diagnostics

Chunchen Liu^1^; Xiaonan Xu^2^; Bo Li^1^; Bo Situ^1^; Weilun Pan^1^; Taixue An^1^; Shuhuai Yao^2^; Lei Zheng ^1^

^1^Department of Laboratory Medicine, Nanfang Hospital, Southern Medical University, Guangzhou, China (People’s Republic); ^2^Department of Mechanical and Aerospace Engineering The Hong Kong University of Science and Technology, Hong Kong, Hong Kong, Hong Kong

**Background**: Exosomes shed by tumour cells have been recognized as promising biomarkers for cancer diagnostics due to their unique composition and functions. Quantification of low concentrations of specific exosomes present in very small volumes of clinical samples may lead to non-invasive cancer diagnosis and prognosis.

**Methods**: Using droplet microfluidics, we encapsulated single exosome complexes tagged with an enzymatic reporter that produces fluorescent signal for detection.

**Results**: Our droplet based single exosome counting immunoassays (droplet digital ExoELISA) approach enables absolute counting of cancer-specific exosomes to achieve unprecedented accuracy. Using a plasma sample of 10 µL, we were able to detect as few as ~5 enzyme-labelled exosome complexes (~10−17 M). We demonstrated the application of the droplet digital ExoELISA platform in quantitative detection of exosomes directly in plasma samples from breast cancer patients.

**Summary/Conclusion**: We believe our approach may have the potential for cancer early diagnostics and accelerate the discovery of clinical diagnostic cancer exosomal biomarkers.

PS08.09

Virtual Biorepository (VBR): a web-based service for sharing biofluid-, tissue-, cell- and other bio-samples

Neethu Shah^1^; Sameer Paithankar^1^; William Thistlethwaite^1^; Jorge Arango^2^; Yashar Kalani^3^; Julie Saugstad^4^; Theresa Lusardi^4^; Joseph Quinn^4^; Lori Chase^5^; Tushar Patel^5^; Andrew R. Jackson^6^; Sai Lakshmi Subramanian^6^; Matthew Roth^6^; Bob Carter^7^; Fred Hochberg^8^; Aleksandar Milosavljevic ^6^

^1^Baylor College of Medicine, Houston, USA; ^2^Phoenix Children’s Hospital, Phoenix, USA; ^3^Barrow Neurological Institute, Phoenix, USA; ^4^Oregon Health & Science University, Portland, USA; ^5^Mayo Clinic, Jacksonville, USA; ^6^Department of Molecular & Human Genetics, Baylor College of Medicine, Houston, USA; ^7^Department of Neurosurgery, Massachusetts General Hospital, Boston, MA, Boston, USA; ^8^UC San Diego, San Diego, USA

**Background**: Virtual biorepository (VBR) arose from the need of investigators within the NIH exRNA Communication Consortium (ERCC) to share biofluid samples across institutions for the purpose of collaborative protocol development and biomarker discovery. The initial goal was to enable the sharing of cerebrospinal fluid (CSF) samples between members of the ERCC-based CSF consortium. VBR has since been extended to accommodate biosamples from a variety of research projects.

**Methods**: VBR is a distributed system consisting of a VBR hub and a set of local or cloud-hosted VBR nodes. The hub supports sample queries based on publicly shared metadata about deidentified biosamples. Participant institutions can restrict access to samples or specific metadata fields to authorized users. Sample lists that satisfy search criteria are placed in a shopping cart for ordering from sample providers. The VBR shopping cart allows end-to-end tracking of biosample exchange between investigators from different institutions. Researchers communicate directly with each other to make specific biosample sharing arrangements. VBR nodes are under control of the sample providers and managed independently of the hub.

**Results**: The VBR hub (beta) is available at (https://genboree.org/vbr-hub/), for use by the global extracellular RNA research community. VBR hub currently provides access to metadata for 56,397 CSF and liver disease samples from six institutions. The biofluid samples are suitable for the study of exRNAs and exmiRs from biofluids, and assessment of biomarker sensitivity and specificity. To facilitate biosample exchange, all participant institutions minimally agree to a common IRB language and uniform MTAs, available on the VBR hub. The ERCC data coordination centre provides assistance regarding maintenance of data within individual VBR nodes using pre-defined metadata templates.

**Summary/Conclusion**: VBR addressed the needs of investigators within the ERCC to share biofluid samples, and has now been extended to include liver disease samples, and various other tissues, cells and sample slides. These resources will be particularly useful for catalysing collaborations, protocol development and biomarker discovery.

**Funding**: This study was funded by NIH Common Fund Extracellular RNA Communication Consortium (ERCC) grant U54 DA036134.

PS08.10

Monitoring the potential role of circulating miR-181b-5p in minimal residual disease in paediatric acute lymphoblastic leukaemia

Nóra Kutszegi ^1^; Andrea Rzepiel^1^; András Gézsi^2^; Mónika Papp^1^; Bálint Egyed^1^; Henriett Butz^1^; Judit C. Csányi^1^; Ágnes F. Semsei^1^; Gábor T. Kovács^1^; György Péter^3^; Csaba Szalai^1^; Dániel J. Erdélyi^1^

^1^Semmelweis University, Budapest, Hungary; ^2^MTA-SE Immune-Proteogenomics Extracellular Vesicle Research Group, Budapest, Hungary; ^3^Heim Pál Children’s Hospital, Budapest, Hungary

**Background**: Circulating microRNAs are promising biomarkers as they can be found in a variety of body fluids and can be non-invasively or minimally invasively obtained. The profile of circulating microRNAs reflects the presence of malignant and non-malignant diseases. Recently, plasma miR-181b-5p was found to be upregulated in acute myeloid leukaemia patients. In addition, it was associated with shorter overall survival. The aim of our study was to determine the relative expression pattern of plasma miR-181b-5p through paediatric acute lymphoblastic leukaemia (ALL) treatment to evaluate its possible role in minimal residual disease (MRD) detection.

**Methods**: Peripheral blood was obtained from 11 paediatric pre-B ALL patients with normal karyotype at four different time points of their treatment: on day 1 at diagnosis, and on days 8, 15 and 33. The preparation of platelet-free plasma from blood samples was carried out within 2 h of sampling. Cell-free total RNA was purified using the miRNeasy Serum/Plasma Kit (Qiagen). Quantitative RT-PCR was performed to detect the relative expression of miR-181b-5p using the Taqman Advanced miRNA assays.

**Results**: The relative expression level of miR-181b-5p was significantly reduced on days 8, 15 and 33 compared to that on day 1 (*p* = 0.006, *p* = 0.047 and *p* = 0.009 respectively). The fold change between day 1 and day 8 and between day 1 and day 15 correlated significantly with the flow cytometry-based MRD values of ALL patients on day 15 (r = 0.982, *p* = 0.00009 and r = 0.956, *p* = 0.003 (Pearson)).

**Summary/Conclusion**: Growing evidence suggests that miR-181b affects several types of malignancies, including leukaemia. Based on our results, the measurement of plasma miR-181b-5p expression may have relevance in the development of a less invasive MRD monitoring in paediatric ALL.

**Funding**: This study was supported by National Research, Development and Innovation Office (NKFIH) K115861.

PS08.11

Serum exosomal microRNAs as non-invasive biomarkers for human hepatocellular carcinoma

Gyeonghwa Kim ^1^; Se Young Jang^2^; Yu Rim Lee^2^; Jung Gil Park^3^; Hye Won Lee^4^; Soo Young Park^2^; Won Young Tak^2^; Young Oh Kweon^2^; Keun Hur^1^

^1^Department of Biochemistry and Cell Biology, School of Medicine, Kyungpook National University, Daegu, Korea, Daegu, Republic of Korea; ^2^Department of Internal Medicine, Kyungpook National University Hospital, Daegu, Korea, Daegu, Republic of Korea; ^3^Department of Internal Medicine, College of Medicine, Yeungnam University, Daegu, Korea, Daegu, Republic of Korea; ^4^Department of Pathology, Dongsan Medical Center, School of Medicine, Keimyung University, Daegu, Korea, Daegu, Republic of Korea

**Background**: Although considerable progress has been made in the treatment of hepatocellular carcinoma (HCC), early detection is still highly considered the key to improved survival. Recently, cancer cell-derived extracellular vesicles have been known to contain various intracellular biomolecules including microRNAs (miRNAs). The aim of this study was to evaluate whether exosomal miRNAs can serve as a serum-based biomarker in HCC.

**Methods**: Expression of six miRNAs (miRNA-24, -130a, -182, -203, -373 and -423) was analysed in the exosome samples. We also investigated expression status of the six miRNAs in matched HCC tissues and corresponding normal liver tissues.

**Results**: We observed that serum exosomal miRNA-203 (*P* < 0.05) and miRNA-373 (*P* < 0.05) were significantly up-regulated in advanced HCC patients. More interestingly, high serum exosomal miRNA-203 and miRNA-373 was associated with HCC progression (*P* < 0.01) as well as prognosis (*P* < 0.05) of HCC patients.

**Summary/Conclusion**: We provided the novel evidence for usefulness of serum circulating exosomal miR-203 and miR-373 expressions as strong potential biomarkers for predicting prognosis and metastasis of HCC patients.

PS08.12

Extracellular small non-coding RNAs as promising biomarkers for early cancer detection

Yukie Nishiyama ^1^; Yumiko Koi^2^; Genki Nishimura^1^; Eri Kojima^1^; Morihito Okada^2^; Hidetoshi Tahara^1^

^1^Cellular and Molecular Biology, Graduate School of Biomedical Sciences, Hiroshima University, Hiroshima, Japan; ^2^Department of Surgical Oncology, Hiroshima University, Hiroshima, Japan

**Background**: Extracellular small non-coding RNAs, such as microRNAs, isoforms of microRNAs (isomiRs) and tRNA-derived fragments (tRFs) are known to regulate expression of genes involved in cell metabolism, and are released into body fluid from various cells with extracellular vesicles including exosomes. In this study, we focused on isomiRs and tRFs as novel cancer biomarkers and characterized their expression profiles to find those expressed specifically in serum from cancer patients.

**Methods**: Serum samples were collected from the patients who provided written informed consent to participate in the study (approved by IRB of Hiroshima University). Cells were cultured in DMEM with FBS and the supernatant were collected after 1-day culture without FBS. Small RNAs were purified from serum and cell culture supernatant by using miRNeasy Mini Kit (Qiagen). Extracellular vesicles (EVs), including exosomes, were isolated by using Total Exosome Isolation kit (Thermo Fisher Scientific). Size and amounts of EVs were measured by qNano (IZON). Next generation sequencing (NGS) was performed by using Ion S5 (Thermo Fisher Scientific). The data were analysed by using CLC Genomics and JMP, and sequences of small RNAs (15–55 nt) found to differ between cancer patients and healthy individuals were considered candidate biomarkers.

**Results**: We identified several isomiRs and tRFs expressed specifically in serum from cancer patients. Some of them were expressed at higher levels and performed better as biomarkers than microRNAs. The expression profiles of some isomiRs and tRFs in cancer serum samples were demonstrated to correlate with extracellular RNA profiles in EVs released from cultured cancer cell lines. The combined use of isomiR and tRF levels allowed us to detect early-stage pancreatic cancer.

**Summary/Conclusion**: Our results suggest that isomiR and tRF forms of extracellular small non-coding RNAs in serum are useful biomarkers for NGS-based detection of early cancers.

PS08.13

Exosomal miR-486-5p, miR-181a-5p and miR-30d-5p from hypoxic tumour cells are candidate circulating markers of high-risk rectal cancer

Tonje Bjørnetrø ^1^; Kathrine Røe Redalen^2^; Nirujah S. Thusyanthan^3^; Sebastian Meltzer^3^; Rampradeep Samiappan^4^; Caroline Jegerschöld^4^; Karianne Risberg Handeland^3^; Anne Hansen Ree^3^

^1^Department of Oncology, Akershus University Hospital, Norway, Oslo, Norway; ^2^Department of Oncology, Akershus University Hospital, Lørenskog, Norway, Trondheim, Norway; ^3^Department of Oncology, Akershus University Hospital, Lørenskog, Norway, Oslo, Norway; ^4^Department of Bioscience and Nutrition, Karolinska Institute, Stockholm, Sweden

**Background**: Tumour hypoxia (oxygenation deficiency) contributes significantly to treatment resistance and metastasis in locally advanced rectal cancer (LARC). Exosomes play a central role in the aggravated biology caused by hypoxia through their cargo. We aimed to characterize exosomal miRNAs from hypoxic colorectal cancer (CRC) cell lines and investigate these miRNAs in circulating exosomes of LARC patients (approved by medical ethics committee).

**Methods**: Five CRC cell lines were cultured in medium supplemented with 1% bovine serum albumin under normoxia (21% oxygen) or hypoxia (0.2% oxygen) for 24 h. Exosomes were isolated from conditioned media by differential ultracentrifugation, size-determined by cryo-electron microscopy and nanoparticle tracking analysis, and characterized by Western blot and flow cytometry. Plasma samples from 24 patients (informed consent given) were collected at the time of diagnosis and exosomes were isolated using the miRCURY™ Exosome Isolation Kit (Exiqon). Expression profiling of exosomal miRNAs was conducted using the miRCURY LNA™ Universal RT microRNA PCR Human panel I (Exiqon). Data normalization was performed based on global array mean and differentially expressed miRNAs were determined by Student’s *t*-test.

**Results**: Extracellular vesicles from the CRC model were confirmed as exosomes and harboured strong cell line-specific miRNA profiles with 35 unique miRNAs differentially expressed between hypoxic and normoxic cells. These miRNAs were considered candidate circulating markers of tumour hypoxia in patients if they either were similarly regulated in two cell lines and detected in at least 75% of patient samples, or were more than twofold changed in a cell line and detected in all patient samples. Using these criteria, decreased exosomal levels of miR-486-5p and miR-181a-5p were associated with cell line hypoxia as well as organ-invasive primary tumour (*p* = 0.032) and lymph node metastases (*p* = 0.024) in the LARC patients respectively. Moreover, exosomal miR-30d-5p level was increased in cell line hypoxia and plasma from patients with metastatic progression (*p* = 0.036).

**Summary/Conclusion**: Exosomal miR-486-5p, miR-181a-5p and miR-30d-5p were regulated by hypoxia in CRC cell lines and retrieved as circulating markers in high-risk LARC patients.

PS08.14

The biology of exosome derived from senescent cells

Ryo Okada; Akiko Takahashi

Project for Cellular Senescence, Cancer Institute, Japanese Foundation for Cancer Research, Koto-ku, Japan

**Background**: Cellular senescence, a state of irreversible cell cycle arrest, prevents the proliferation of cells at risk for neoplastic transformation. Additionally, senescent cells increase the secretion of various pro-inflammatory proteins, such as inflammatory cytokines, chemokines or growth factors, into the surrounding extracellular space. These novel senescent phenotypes, termed the senescence-associated secretory phenotype (SASP), reportedly contributes to tumour suppression, wound healing, embryonic development or tumourigenesis promotion depending on the biological context. On the other hand, emerging evidence is revealing that exosomes contribute to many aspects of physiology and disease through intercellular communication. Recently we have reported that exosome secretion was significantly increased in senescent cells (Takahashi et al., Nat Commun. 2017). However, the biological roles of exosome secretion in exosome-secreting cells and exosomes from senescent cells have remained largely unexplored. Therefore, we tried to analyse the biological function of exosome derived from senescent cells.

**Methods**: To enhance our understanding of exosome biology, we examined the mechanism of exosome secretion in senescent cells. Firstly, pre-senescent normal human diploid fibroblasts (HDFs) were rendered senescent by either serial passage or ectopic expression of oncogenic Ras, then we performed a cell proliferation analysis using cancer cells incubated with condition medium or exosomes from pre-senescent and senescent HDFs. Secondly, to investigate the molecular mechanisms for increasing exosome secretion in senescent cells, we knocked down and overexpressed several components, which are essential for exosome biogenesis.

**Results**: We found that some factors which are required for exosome biogenesis are specifically activated in senescent cells. Moreover, exosome from senescent cells promotes cell proliferation and chromosomal instability in cancer cells.

**Summary/Conclusion**: We have revealed a new role of exosomes derived from senescent cells as one of the SASP factors.

PS08.15

Extracellular vesicles derived from senescent cells repress tumour growth via miRNAs

Mariko Ikuo; Megumi Okada; Shigeyuki Teranishi; Masaki Kinehara; Akira Shimamoto; Hidetoshi Tahara

Cellular and Molecular Biology, Graduate School of Biomedical Sciences, Hiroshima University, Hiroshima, Japan

**Background**: Cellular senescence is a mechanism to arrest growth of DNA damaged or oncogenic stress exposed cells and avoid their tumourigenesis. Our previous studies revealed the important roles of microRNAs in cellular senescence induction. The microRNAs are small non-coding RNAs that repress target mRNAs’ functions. Extracellular vesicles (EVs) convey various molecules including microRNAs and act as cell–cell communication tools to regulate biological events. However, their roles in cellular senescence are still unclear. In this study, we examined whether EVs secreted from senescent cells regulate cancer cell’s activities.

**Methods**: Senescent cells were established by continuous culture of normal human fibroblast cell TIG-3. Ultracentrifugation was used for EV collection. Particle numbers and size distributions were analysed by a nanopore-based particle analyser, qNano. Exosomal marker protein expressions were analysed by Western blot. MicroRNA expression profiles were analysed by next generation sequencing. MicroRNA and mRNA expressions were quantified by quantitative reverse transcription polymerase chain reaction. Luciferase expressing MDA-MB-231 derivative cell line MDA-MB-231-D3H2LN was used for mice xenograft model to assess *in vivo* tumour growth.

**Results**: S-EV sample consisted of particles around 110 nm and expressed exosomal marker proteins. S-EVs treatment repressed *in vitro* cell growth and invasion activity of breast cancer cell line MDA-MB-231. The expression of miR-127-3p and miR-134-5p were enriched in S-EVs. Mir-127-3p and miR-134-5p expressions were increased in S-EVs treated cancer cell. Growth arrest activity of S-EVs was inhibited by pretreatment of LNA-miRNA inhibitor for miR-127-3p and miR-134-5p. S-EVs inhibited tumour growth in mice xenograft model.

**Summary/Conclusion**: Senescence cell-derived extracellular vesicles have tumour inhibitory activities mediated by miRNAs.

PS08.16

UVA induced plasma membrane damage promotes shedding of EVs from melanocytes and activates cell proliferation

Petra Wäster; Ida Eriksson; Inger Rosdahl; Karin Öllinger

IKE, Linköping University, Sweden, Linköping, Sweden

**Background**: Ultraviolet radiation (UV) causes transfer of melanin from melanocytes to keratinocytes. In addition, we have made the novel finding that exposing melanocytes to UVA, but not UVB, induces immediate shedding of extracellular vesicles (EVs) from the cells. EV-shedding is preceded by UVA-induced plasma membrane damage, which is rapidly repaired by lysosomal exocytosis. The EVs, containing marker proteins from lysosomes as well as flotillin-1 and CD63, are taken up by keratinocytes. We found the transfer and uptake mechanisms of melanin and EVs to be mechanistically unrelated. The aim of the present study was to characterize the effect induced by melanocyte-produced EVs on keratinocytes.

**Methods**: We have performed gene expression analysis of keratinocytes, exposed to purified EVs produced by melanocytes after UV irradiation. The results are compared with public databases and correlated to proliferation and melanoma progression. The function of candidate genes and miRNAs in UV-induced intercellular communication and in melanoma progression are verified.

**Results**: Exposure to melanocyte-derived EVs enhances keratinocyte proliferation. Data analysis shows up-regulation of 127 genes (FC > 1.5) and in-depth bioinformatic analysis identifies TGF-β/SMAD signalling and associated microRNAs (mir21, mir24-2 and mir200c) as candidate signalling molecules. In accordance, transfection with mir21-mimic induces proliferation in keratinocytes, as well as in melanocytes. Interestingly, melanoma cells spontaneously release EVs and mir21 is upregulated during melanoma progression.

**Summary/Conclusion**: We discern the melanocytes as important players in the protection against UV, not only by distribution of melanin, but through rapid generation of EVs that enhances proliferation, which might promote sun-induced thickening of epidermis. Moreover, we provide new insight on UVA induced alterations of skin homeostasis. The knowledge could be applied on melanoma initiation and progression.

PS08.17

Insights into the role of extracellular vesicles in lenalidomide-resistance multiple myeloma

Tomofumi Yamamoto ^1^; Nobuyoshi Kosaka^1^; Yutaka Hattori^2^; Takahiro Ochiya^1^

^1^Division of Molecular and Cellular Medicine, National Cancer Center Research Institute, Chuo-ku, Japan; ^2^Clinical Physiology and Therapeutics, Keio University Faculty of Pharmacy, Minato-ku, Japan

**Background**: Multiple myeloma (MM) is a malignancy of terminally differentiated plasma cells. Although the prognosis of MM has dramatically improved with new therapeutic drugs, such as lenalidomide, MM is still incurable because of the acquisition of drug resistance in MM. The mechanisms of its drug-resistance acquisition have been proposed, but the detail mechanisms are not fully explained; however, contribution of extracellular vesicles (EVs) for drug resistance in MM has not been clarified yet. It has been shown that cisplatin induced the release of EVs from ovarian cancer cells, and those EVs promoted the invasiveness and drug resistance in their bystander cells, indicating that EVs are involved in drug resistance during cancer cell progression. In this study, we will investigate the role of EVs in lenalidomide-resistance multiple myeloma.

**Methods**: In order to obtain lenalidomide resistant cell lines, low concentration of lenalidomide was exposed to three different types of multiple myeloma cell lines (KMS 21, KMS 27, KMS 34) for a long period. Acquisition of lenalidomide resistant in MM was assessed by MTS assay for cellular proliferation and apoptosis assay, which was evaluated by caspase activity. The amount of EVs was measured by ExoScreen, which is ultra-sensitive detection method of EVs by measuring surface protein of EVs, such as CD9 and CD63, and by the nanoparticle tracking analysis.

**Results**: Three different lenalidomide resistant cell lines, which were established by exposing the low concentration of lenalidomide, were established. The amount of EV secretion was significantly higher in resistant cell lines compared with non-resistant cell lines. The amount of EV from lenalidomide resistant cell lines was not changed drastically along with the increasing concentration of lenalidomide to lenalidomide resistant cell lines.

**Summary/Conclusion**: These results suggest that continuous stress, such as long-term exposure of anti-cancer agent in MM, change the phenotype of MM, leading to the increased production of EVs from lenalidomide resistant cell lines. We are seeking the roles and mechanism of EVs from lenalidomide resistant cell lines. This study will propose the novel therapy for treating the lenalidomide resistant MM cells.

PS08.18

Exosomes increase SH-SY5Y neuroblastoma cells radioresistance by activating the AKT survival pathway

Flavia Tortolici ^1^; Anna Giovanetti^2^; Giulia Carrozzo^3^; Francesca Mastrostefano^4^; Stefano Rufini^4^

^1^Department of Biology University of Rome “Tor Vergata”, Rome, Italy; ^2^Technical Unit for Radiation Biology and Human Health ENEA CR Casaccia, Roma, Italy; ^3^Department of Biology University of Rome “Tor Vergata, Rome, Italy; ^4^Department of Biology University of Rome “Tor Vergata”, Rome, Italy

**Background**: Ionizing radiation is the main approach for eradicating cancer. However, in numerous cases radiotherapy may induce resistance to radiation leading to therapeutic failure. Therefore, more effective strategies against radioresistance are urgently needed. As we have described in a previous work, following irradiation SH-SY5Y neuroblastoma cells release exosomes with different physicochemical characteristics, able to increase cell survival. The aim of this study is to investigate the intracellular mechanism by which exosomes induce an increase of cell survival after irradiation.

**Methods**: To study their autocrine effects, exosomes were purified from the culture media of unirradiated and irradiated SH-SY5Y cells maintained in an exosomes deprived medium for 2 h. The purified exosomes were then added to new SH-SY5Y, which were finally irradiated. MTT assay, BrdU incorporation, motility and clonogenicity were applied to evaluate cell radioresistance, while survival signalling pathways were analysed by Western blotting.

**Results**: After irradiation, all the analysed parameters of cell vitality are modified in exosome treated respect to untreated cells. The radioresistance observed in exosomes treated cells is correlated to the activation of the PI3K/AKT survival signalling pathway that involves the FoxO1 phosphorylation. Intriguingly, the Western blot analysis of the microvesicles purified from SH-SY5Y culture medium shows the presence of activated AKT kinase, i.e. phosphorylated on both serine 473 and threonine 308 residues.

**Summary/Conclusion**: These observations indicate that exosomes may induce radiation resistance in SH-SY5Y cells by mechanisms involving FoxO1 phosphorylation, thus blocking the apoptotic process triggered by radiation. Our hypothesis is that this pathway is activated or reinforced by the uptake of exosomes carrying phosphorylated AKT.

**Funding**: This study was funded by Italian Ministry of Foreign Affairs and international Cooperation (grant: PGR00782).

PS08.19

Extracellular vesicles shedding in response to chemotherapy in melanoma promotes tumour growth after temozolomide treatment

Luciana Andrade ^1^; Andreia H. Otake^2^; Silvia Cardim^1^; Mariana Ikoma^1^; Felipe Silva^1^; Roger Chammas^3^

^1^Instituto do Cancer do Estado de Sao Paulo-ICESP, Sao Paulo, Brazil; ^2^ICESP – FMUSP, Sao Paulo, Brazil; ^3^ICESP – FMUSP, Sao Paulo, Brazil

**Background**: Extracellular vesicles (EVs) are emerging as a key players in intercellular communication. It has been shown that tumour cells secrete large amounts of EVS that can be taken up by malignant and stromal cells. Several groups have demonstrated that EVs shed by tumour cells can induce resistance to therapy promoting tumour growth. Based on that, our goal is to investigate if EVs secreted by melanoma cells in response to chemotherapy can modulate tumour growth and progression.

**Methods**: Human melanoma cell lines were treated with temozolomide (TMZ) and EVs secreted under these conditions were purified from cell media after ultracentrifugation. EVs quantification was determined using Nanosight NT LM10. The presence of Annexin V, CD9 and CD63 were determined using a flow cytometry. For macrophage polarization studies, murine macrophages were incubated with LPS and interferon gamma or IL4 in the presence of EVs derived from TMZ or vehicle melanoma treated cells to induce M1 and M2 polarization respectively. After 24 h, M1 and M2 gene expression were determined by qPCR. For *in vivo* studies, human melanoma cells admixed with EVs derived from TMZ or vehicle treated cells were injected s.c. in nude mice. Tumour growth was measured with a caliper. Statistical analysis was performed using GraphPad Prism.

**Results**: Our findings showed a significant increase in EVs secreted by human melanoma cell lines in response to TMZ treatment. Nanotracking analysis revealed that the majority of EVs range from 100 to 200 nm in size, comprising both exosome and microvesicles which were positive for CD9, CD63 and Annexin V. We observed that EVs shed by melanoma cells after TMZ treatment modulate macrophage phenotype by skewing macrophage activation towards the M2 phenotype as demonstrated by the significant increase in M2 gene expression (Arg-1, IL10 and MRC1). In addition, these vesicles promoted tumour growth *in vivo*, indicating a pro-tumoural effect of EVs secreted in response to chemotherapy.

**Summary/Conclusion**: Our results showed an increase in the amount of EVs released by melanoma cells in response to chemotherapy which were able to induce macrophage polarization towards M2 phenotype favouring tumour growth *in vivo*, indicating that EVs could constitute a route for tumour repopulation after chemotherapy in melanoma.

**Funding**: This work was supported by Fapesp and CNPq.

PS09: Novel Developments in EV Characterization Chairs: Miriam Diaz; Wojciech ChrzanowskiLocation: Exhibit Hall 17:15–18:30

PS09.01 = OWP3.04

Extracellular vesicles deformation on surface: some tracks to limit it

PS09.02

Aggregation-Induced Emission Probe/Graphene Oxide Aptasensor for Label-free and “turn-on” fluorescent detection of cancerous exosomes

Bo Li; Chunchen Liu; Weilun Pan; Lei Zheng

Department of Laboratory Medicine, Nanfang Hospital, Southern Medical University, Guang Zhou, China (People’s Republic

**Background**: Exosomes are emerging as non-invasive diagnostic biomarkers of cancer because they carry biomolecules that include proteins and nucleic acids for intercellular communication. Assessing special surface proteins provides a powerful means of identifying the origins of parent cells.

**Methods**: Herein, we combined the strengths of prostate-specific membrane antigen (PSMA) aptamers, the aggregation-induced emission (AIE) probe for nucleic acid and the integration of AIE probe and graphene oxide (GO) to develop a label-free and “turn-on” fluorescent sensor platform for prostate cancer exosomes. In the presence of prostate cancer exosomes, the non-specific and weaker binding between aptamers dyed by AIE probes and GO with high quenching ability is broken, and the specific and stronger binding between aptamers and exosome surface protein displaces aptamers from GO surface. Then aptamers binding with exosomes appear “turn-on” fluorescent property because the interaction of aptamers with the AIE probes.

**Results**: Under optimal conditions, the linear range of detection for prostate cancer exosomes is estimated to be 1.1 × 10^5^ to 5.8 × 10^6^ exosomes/μL with a detection of limit (LOD) of 7.3 × 10^4^ exosomes/μL. We further successfully applied it for exosomes quantification in serum samples from prostate cancer patients.

**Summary/Conclusion**: The AIE/GO aptasensor is expected to become a powerful tool for comprehensive exosomes studies.

**Funding**: This study was funded by National Natural Science Foundation of China (81702100).

PS09.03

Development of lateral flow test for detection of exosomes biomarkers in urine samples

Jesus Berganza ^1^; Zoraida Rosé^1^; Garbiñe Olabarria^1^; Juan M. Falcón-Pérez^2^

^1^GAIKER Technology Center, Zamudio, Spain; ^2^CIC bioGUNE, CIBERehd, Bizkaia Science and Technology Park, Derio, Bizkaia, Spain, Derio, Spain

**Background**: The main objective of the work is the development of a portable system for immunodetection of exosomes biomarkers in urine samples. Lateral flow or immunochromatographic assays are cheap, easy to use and point-of-care diagnostic tests widely used in diagnostic applications. As proof of concept, a quantitative lateral flow test based on fluorescent beads that detects the exosomal marker CD63 has been developed and its performance was assayed directly on urine samples or preparations obtained by different concentration methods.

**Methods**: *Antibody*: mouse anti-human CD63 from BD. *Antigen*: CD63 recombinant antigen from Novus Biologicals. COOH-Fluorescent Latex Beads. Isolation of exosomes from urine samples with centrifugal ultrafiltration and ultracentrifugation. Manufacturing of lateral flow assay half-strip with anti-CD63 antibody conjugated fluorescent beads and CD63 antigen sprayed on nitrocellulose membrane. Fluorescence strip reader (ESE Quantitative Lateral Flow Reader) from QIAGEN.

**Results**: The main parameters for the manufacturing of lateral flow strips have been developed: membrane pore size, antigen concentration in line test, antibody in line control and conjugation of antibody to beads. 25 μl of different fractions obtained by ultracentrifugation from the same urine sample were tested in the lateral flow strips. For the fractions where the exosomes are concentrated (pellet after ultracentrifugation) the fluorescence signal decreases from 3000 to 0 units. While in the fractions without exosomes (supernatant after ultracentrifugation) the fluorescence signal does not be different to the negative control.

**Summary/Conclusion**: These results are a promising proof of concept for the development of a portable detection system of urinary exosomes biomarkers that could be associated with pathological profiles of urinary system. The lateral flow test developed in this work is specific for detection of CD63 biomarker, but the method can be adjusted to detect other exosomal markers.

**Funding**: This study was funded by ELKARTEK Program 2017, Economic Development and Infrastructures Department, Basque Government.

PS09.04

Development of 3-hexanoyl-NBD cholesterol (3NBDC) as a biochemical tool to detect extracellular vesicle cholesterol by flow cytometry

Shuaishuai Hu; Steve Meaney; Claire Wynne

Dublin Institute of technology, Dublin, Ireland

**Background**: It is well established that extracellular vesicles (EVs) contain cholesterol; however, there is a lack of information around the biological roles and metabolic fate of this cholesterol. Studies in this area have been hampered by the availability of accessible methods to visualize and track EV cholesterol. Cholesterol labelled at the C22 position with nitrobenzoxadiazole (NDB) has been described in the literature as a viable cholesterol tracer; however, addition of a bulky NDB moiety at the C22 position within the membrane is expected to perturb normal membrane structure. Instead, cholesterol analogues labelled at the C3 position represent alternative sensor molecules expected to display membrane orientation similar to that of cholesterol, with minimal disturbance of internal membrane organization.

**Methods**: Cholesterol exchange between erythrocytes and plasma was studied by incubating plasma with 3NBDC labelled erythrocytes for different time points over a 12 h period, before detecting the fluorescence intensity of the plasma by spectrophotometry and of the erythrocytes by flow cytometry. HeLa and THP-1 cells were also treated with 3NBDC for various time points before fluorescence intensity was measured by flow cytometry. EV from macrophage cells and plasma were treated with 3NBDC for 1 h before fluorescence intensity was measured by flow cytometry. Written informed consent was obtained from donors under DIT ethics application.

**Results**: Exchange studies from 3NBDC labelled erythrocyte to lipoproteins revealed behaviour similar to cholesterol. Incubation of differentiated THP-1 cells with 3NBDC labelled EVs revealed a time-dependent uptake of the NBDC label. Labelled cells and EVs could be readily detected by flow cytometry, and uptake of labelled EVs could also be directly followed by flow cytometry.

**Summary/Conclusion**: These data indicate that 3NBDC is a viable cholesterol tracer that can be used to further investigate EV biology. We are currently expanding these studies to trace the intracellular itinerary of 3NBDC following uptake of labelled EVs.

**Funding**: This study was funded by Dublin Institute of technology Fiosraigh – Research Scholarships.

PS09.05

Quantitative analysis of nucleic acids in extracellular vesicles at the single-particle level via an ultrasensitive flow cytometer

Ye Tian ^1^; Haisheng Liu^1^; Manfei Gong^1^; Wenqiang Zhang^1^; Ling Ma^2^; Shaobin Zhu^2^; Xiaomei Yan^1^

^1^Department of Chemical Biology, Xiamen University, Xiamen, China, Xiamen, China (People’s Republic); ^2^NanoFCM Inc., Xiamen, China, Xiamen, China (People’s Republic)

**Background**: Quantitative analysis of EVs at the single-vesicle level is indispensable for the biological study of EVs. However, the nanoscale size and the minute quantity of molecular content render it technically quite challenging. Building upon a laboratory-built high-sensitivity flow cytometer (HSFCM), we recently developed a rapid approach for protein profiling and sizing of individual EVs down to 40 nm. Here we report the progress in the quantitative analysis of nucleic acids in single EVs.

**Methods**: EVs were isolated from cultured medium of human colorectal cancer HCT15 cell line using differential ultracentrifugation. DNase and RNase were used to enzymatically digest the nucleic acids adsorbed onto the surface of the EVs whereas the counterparts enclosed within vesicles are protected by lipid membranes and remain intact. Membrane transmissible nucleic acid stains such as SYTO 9 and SYTO RNASelect were used to selectively stain DNA and RNA respectively. The samples were then analysed on the HSFCM before and after the enzymatic treatment.

**Results**: Upon SYTO 9 staining, besides individual EVs with concurrent peaks on both the side scattering and fluorescence channels, we also observed numerous fluorescent peaks with no correlated side scattering signals. Because these uncorrelated fluorescent peaks disappeared upon DNase treatment, we ascribe them to the DNA fragments in suspension and not associated with EVs. It is interesting to find out that after being treated with DNase, the subpopulation of EVs lightened by SYTO 9 decreased from 40% to less than 10%. These results suggest that most DNA were not encapsulated inside EVs and therefore can be digested by the enzyme. When the EV isolate was stained by SYTO RNASelect (a RNA selective dye), we found that only around 10–20% of isolated EVs (~90% purity) can be detected with fluorescent peaks concurrently with side scattering. Correlation analysis with side scattering signals indicates that this subpopulation of EVs is large size vesicles.

**Summary/Conclusion**: The ultrasensitive flow cytometer enables quantitatively analysis of the nucleic acids in individual EVs, which can be helpful in the illustration of EV-mediated, RNA-based intercellular communication.

PS09.06

Differential fluorescence nanoparticle tracking analysis for enumeration of the extracellular vesicle content in mixed particulate solutions

Karin Pachler ^1^; Alexandre Desgeorges^1^; Christina Folie^1^; Magdalena Mayr^1^; Heide-Marie Binder^1^; Eva Rohde^2^; Mario Gimona^2^

^1^GMP Unit, Spinal Cord Injury and Tissue Regeneration Center Salzburg (SCI-TReCS), Paracelsus Medical University Salzburg, Salzburg, Austria; ^2^GMP Unit, Spinal Cord Injury and Tissue Regeneration Center Salzburg (SCI-TReCS) and University Institute for Transfusion Medicine, Paracelsus Medical University Salzburg, Salzburg, Austria

**Background**: A major concern for the extracellular vesicle (EV) field is the current lack of accurate methods for EV quantification. Due to the structure and the size range of EVs, current technologies are inadequate: Total protein measurement is unsuitable to quantify EVs from serum-containing conditioned media, ELISA kits suffer from technical difficulties, and classical nanoparticle tracking analysis (NTA) allows quantification and size determination of particles, but fails to discriminate between EVs, lipids and protein aggregates. Fluorescence-based NTA (FL-NTA) is an emerging method for counting and phenotyping of EVs. EVs can be fluorescently labelled with non-specific membrane markers or with antibodies specifically recognizing EV surface marker proteins. We are currently establishing a differential FL-NTA method using specific antibodies against surface markers in analogy to cell flow cytometric analysis.

**Methods**: EVs from umbilical cord mesenchymal stromal cells (UC-MSCs) were isolated by a tangential flow filtration/ultracentrifugation protocol with or without subsequent size exclusion chromatography. EV preparations were stained with AlexaFluor 488-conjugated specific antibodies or corresponding isotype controls. Amount and size of particles in normal scattering light mode (N mode) versus fluorescence mode (FL mode, laser wavelength 488 nm) was measured using ZetaView Nanoparticle Tracking Analyzer (Particle Metrix).

**Results**: All UC-MSC-EV preparations were found positive for typical EV marker proteins and negative for MHC I. Additional purification of EV preparations by size exclusion chromatography led to a greater percentage of EV marker protein-positive nanoparticles.

**Summary/Conclusion**: Differential FL-NTA facilitates determination of the percentage of EV marker protein-positive nanoparticles within a mixed particulate solution. We aim to expand our set of markers to other MSC-EV positive and negative surface marker proteins in order to establish FL-NTA-based surface marker profiling as an additional method for quantifying EVs.

**Funding**: This work was supported by project EXOTHERA (funded by the European Regional Development Fund and Interreg V-A Italia–Austria 2014-2020).

PS09.07

Imaging flow cytometry: a potent method to identify distinct subpopulations of small extracellular vesicles

Michel Bremer ^1^; Rita Ferrer-Tur^1^; André Görgens^2^; Verena Börger^3^; Peter A. Horn^3^; Bernd Giebel^3^

^1^Institute for Transfusion Medicine, University Hospital Essen, University of Duisburg-Essen, Essen, Germany; ^2^Clinical Research Center, Department for Laboratory Medicine, Karolinska Institutet, Stockholm, Sweden, Hälsovägen, Sweden; ^3^Institute for Transfusion Medicine, University Hospital Essen, Essen, Germany

**Background**: Although different extracellular vesicle types have been defined regarding their cellular origin, for now, exosomes can hardly been discriminated from small microvesicles or other small EV types. There are hardly any methods available, now, allowing to discriminate different EV-types of comparable sizes. Recently, we have optimized imaging flow cytometry for the single EV detection and characterization of small EVs (70–150 nm) [1]. Upon extending our imaging flow cytometric analyses on EVs, we have established EV antibody-labelling protocols, now, to discriminate distinct EV-subpopulations.

**Methods**: As starting material we have used conditioned media of mesenchymal stem/stromal cells (MSCs), which have been cultured in human platelet-lysate (hPL) supplemented media. Since hPL contains a high concentration of vesicles not being removed in our protocol, conditioned MSC-media provide a collection of MSC-EVs released as well as non-metabolized hPL vesicles. To unravel the EV subpopulations of the conditioned media, different antigen combinations were used and analysed on an imaging flow cytometer.

**Results**: Upon introducing different antigens to characterize EVs, such as the tetraspanins CD9, CD63 and CD81, MSC-EVs were found to express CD81, but not CD9. In contrast, hPL vesicles lacked any CD81 expression, but were highly positive for CD9. Thus, by using anti-CD81 and anti-CD9 antibodies MSC-EVs can effectively be discriminated from residual hPL vesicles.

**Summary/Conclusion**: Overall, our analyses demonstrate, imaging flow cytometry is a powerful technique for the characterization of sEVs at a single vesicle level. Very likely, it will help us to efficiently dissect the heterogeneity of EV containing samples in the future.

**Funding**: This research was funded by European Regional Development Fund 2014-2020 (EFRE) and European Union.

**Reference**: [1] Webinar GA. Analysis of extracellular vesicles including exosomes by imaging flow cytometry. Science. 2016;352:1238–1238.

PS09.08

Analysis of surface glycans on extracellular vesicles using lectin array and roles of their glycans in cellular recognition

Asako Shimoda ^1^; Shin-ichi Sawada^1^; Yoshihiro Sasaki^2^; Kazunari Akiyoshi^1^

^1^Kyoto University, Kyoto, Japan; ^2^Department of Polymer Chemistry, Graduate School of Engineering, Kyoto University, Kyoto, Japan

**Background**: Extracellular vesicles (EVs) are known as biologically derived carriers for the delivery of various functional molecules including proteins, lipids and nucleic acids. Recent studies showed that the population of EVs isolated from the same cell is heterogeneous in size and components; however, evaluation methods for their diversity are not established yet. Glycans on cell surfaces play critical roles in biological processes. Although a lot of proteomics or genomics studies of EVs have been reported so far, little is known about the details of surface glycans on EVs. Here, we analysed glycans on EVs using an evanescent-field fluorescence-assisted lectin array system which can directly detect weak glycan–lectin interactions without the destruction of EVs. Roles of the surfaces glycans in cellular recognition were investigated.

**Methods**: EVs were isolated from mesenchymal stem cells by differential ultracentrifugation. These EVs were characterized by immunoblotting, transmission electron microscopy, nanoparticle tracking analysis and lectin array analysis.

**Results**: Typical exosomal marker (CD81)-positive nano-sized (150–200 nm in diameter) vesicles were collected from all cell lines used in this study. In glycan analysis, intact EVs or cell membranes were added to glass slides spotted with 45 lectins and their glycan profiles were compared with each other. In particular, we found that EVs showed high affinity to sialic acid-binding lectins and the cellular uptake of EVs was mediated by sialic acid-binding immunoglobulin-type lectins *in vitro*. Experiments of subcutaneous injection of the fluorescently labelled EVs into mice showed their transport into lymph nodes and internalization by antigen-presenting cells, particularly those expressing CD11b.

**Summary/Conclusion**: In conclusion, glycan analysis of EVs using a lectin array system is a simple and valid tool for the EV standardization and EV-cell interaction.

**Reference**: [1] Shimoda A, et al. Biochem Biophys Res Commun. 2017;491:701–707.

PS09.10

TEM and Cryo-TEM microscopy as a tool to elucidate prokaryotic membrane vesicle structure

Carla Perez-Cruz^1^; Nicolas Baeza^1^; Carmen Lopez-Iglesias^2^; Elena Mercade ^1^

^1^Department of Biology, Health and Environment, University of Barcelona, Barcelona, Spain; ^2^The Maastricht Multimodal Molecular Imaging institute, Maastricht University, Maastricht, The Netherlands

**Background**: There is a need to characterize the structure of membrane vesicles (MVs). In most published studies, MVs morphology and integrity is revealed by transmission electron microscopy (TEM) micrographs from negatively stained MVs, but the resolution of this technique is not enough. TEM observation of specimens cryoimmobilized by high pressure freezing (HPF) followed by freeze substitution (FS) and sectioning, together with cryo-TEM observation of frozen-hydrated specimens, allow the visualization of biological samples close to their native state, enabling us to refine our knowledge of bacterial structures such us MVs.

**Methods**: Cryo-immobilization of bacteria and MVs by HPF-FS and TEM; cryo-TEM of plunge-frozen whole bacteria and MVs; encapsulation of DNA inside the MVs by TEM after gold DNA immunolabelling.

**Results**: The use of these techniques revealed some interesting findings. First, the structural analysis of the extracellular matter produced by many Gram-negative Antarctic bacteria after HPF-FS TEM allowed us to establish its complexity, appearing as a netlike mesh containing large numbers of MVs. The release of MVs through bulging and “pinching off” from the outer membrane was confirmed. Additionally, we demonstrated a new model of vesiculation in both environmental and pathogenic bacteria that leads to the formation of a different type of outer membrane vesicle with a double-bilayer structure, which encapsulates DNA and thus could be involved in DNA transfer. Furthermore, we detected that the introduction of mutations in bacterial strains to induce hypervesiculating phenotypes leads to alterations in MV composition and in their ability to interact with host cells, which can be explained by significant modifications in MVs structure and this may have a major impact on MV functionality.

**Summary/Conclusion**: This study exposes the need for conducting a detailed structural analysis by high-resolution TEM techniques when working with MVs. This analysis should be mandatory in order to guarantee the good research practice in MV research field, especially if they are intended to be used for therapeutic purposes.

**Funding**: This study was funded by Government of Spain (CTQ2014-59632-R). CPC received the fellowship APIF2015 from the UB, and NB BES2015-074582 from the Government of Spain.

PS09.11

**Enhancing accuracy of clinical predictions on shifted microflow cytometry data with signal standardization**

Robert J. Paproski ^1^; Desmond Pink^1^; Renjith Pillai^2^; Catalina Vasquez^2^; John D. Lewis^1^

^1^University of Alberta, Edmonton, Canada; ^2^Nanostics Inc, Edmonton, Canada

**Background**: We have developed a state-of-the-art XGBoost-based algorithm for predicting clinical outcomes from microflow cytometry data which significantly outperformed CITRUS for predicting prostate cancer aggressiveness in 215 patients (AUCs 0.75 vs 0.59). However, our algorithm, like many others, is sensitive to data shifting which requires correction.

**Methods**: To correct microflow cytometry data shifting, we have developed two separate algorithms. The first identifies the marker status of particles using density-based information. A 281 patient cohort had prostate-specific membrane antigen signals multiplied by 0.125, 0.25, 0.5, 1, 2, 4, 8, 16, 32, 64, 128 or 256 followed by prediction of prostate cancer aggressiveness using our previous and new algorithms. The second algorithm standardized light scatter between samples using a standard bead sample which was compared to the same beads run with different voltages (300–400 V). Histograms of beads with and without light scatter correction were compared to a histogram of standard beads run at 350 V with mean absolute error calculated.

**Results**: Our fluorescence correction algorithm provided similar AUCs to our previous algorithm on the unaltered 281 patient data set. However, our previous algorithm had AUCs of 0.5 for all shifted data sets, suggesting that relatively small changes in fluorescence levels greatly compromised test scores. The fluorescence correction algorithm maintained stable AUCs for all shifted data sets with a coefficient of variation of 1.2%. When analysing the light scatter from bead samples run at different voltages, our light scatter correcting algorithm could re-align the non-linearly shifted light scatter histograms with up to 83% less error than the non-corrected samples.

**Summary/Conclusion**: Correcting microflow cytometry light scatter and fluorescence signals increased clinical test score reproducibility which should improve the reliability of our microflow cytometry-based clinical assay if deployed at various remote clinical laboratories.

PS09.12

High-visibility detection of exosomes by interferometric reflectance imaging

Selim Unlu^1^; Celalettin Yurdakul^1^; Ayca Yalcin-Ozkumur^1^; Marcella Chiari ^2^; Fulya Ekiz-Kanik^1^; Nese Lortlar Ünlü^1^

^1^Boston University, Boston, USA; ^2^CNR ICRM, Milan, Italy

**Background**: Optical characterization of exosomes in liquid media has proven extremely difficult due to their very small size and refractive index similarity to the solution. We have developed Interferometric Reflectance Imaging Sensor (IRIS) for multiplexed phenotyping and digital counting of individual exosomes (>50 nm) captured on a microarray-based solid phase chip. These earlier experiments were limited to dry sensor chips. In this work, we present our novel technology in exosome detection and characterization.

**Methods**: We present advances of IRIS technique to improve the visibility of low-index contrast biological nanoparticles such as exosomes in a highly multiplexed format. IRIS chips are functionalized with probe proteins and exosomes are captured from a complex solution. We have recently demonstrated the integration of pupil function engineering into IRIS technique. By tailoring the illumination and collection paths through physical aperture masks we achieved significant contrast enhancement. For in-liquid detection of exosomes, we have also developed disposable cartridges amenable to high quality optical imaging. Furthermore, we have refined the acquisition and analysis of IRIS images to enable accurate size determination of exosomes.

**Results**: We have shown that IRIS can enumerate, estimate particle size and phenotype exosomes from purified samples from cell culture, or directly from a small of volume clinical sample. We have conducted preliminary experiments utilizing silica nanoparticles. The results demonstrated a nearly 10-fold signal enhancement for 50 nm silica nanoparticles. Given that the nanoparticle signal in an interferometric measurement scales with particle polarizability, and hence particle volume, we expect to be able to detect low-index nanoparticles down to 30 nm with better than 1% contrast. In liquid exosome detection and characterization experiments are currently ongoing.

**Summary/Conclusion**: IRIS technique represents a unique capability to count and characterize individual exosomes directly captured from a complex solution in a multiplexed format. With this unprecedented capability, we foresee revolutionary implications in the clinical field with improvements in diagnosis and stratification of patients affected by different disorders.

**Funding**: This study was funded by EU Horizon 2020 programme under grant agreement No 766466.

PS09.13

Small-particle flow cytometry: a new frontier in detection and characterization of extracellular vesicles in liquid biopsies

Jaco Botha ^1^; Mathilde Sanden^2^; Aase Handberg^3^

^1^Department of Clinical Biochemistry, Aalborg University Hospital, Aalborg, Denmark, Dronninglund, Denmark; ^2^Department of Clinical Biochemistry, Aalborg University Hospital, Aalborg, Denmark, Aalborg, Denmark; ^3^Department of Clinical Biochemistry, Aalborg University Hospital, Aalborg, Denmark, Risskov, Denmark

**Background**: Flow cytometry has been a widely used method for characterization of extracellular vesicles (EVs). However, the applicability of flow cytometry has been somewhat limited due to the inability of conventional flow cytometers (FCM) to detect smaller EVs and discriminate between single events and so-called swarms of EVs. To overcome these issues, recent advances in flow cytometry have led to the development of FCMs dedicated to the analysis of small particles (spFCM). Thus, the aim of this study is to benchmark a novel FCM platform against a conventional FCM with regard to sensitivity, resolution and reproducibility in characterizing EVs directly in plasma.

**Methods**: Flow cytometry is performed on FACSAria III high-speed cell sorter (BD) and Apogee A60 Micro-PLUS (Apogee Flow Systems) platforms. Sensitivity and resolution are assessed using 100 nm fluorescent silica beads and a cocktail of non-fluorescent silica beads ranging from 180 to 1300 nm respectively. Reproducibility of concentration determinations and fluorescence signals are assessed by measuring platelet-poor plasma (PPP) from a pool of healthy donors both in a single day (*n* = 20) and spread out over a whole week (*n* = 4 × 5). PPP is labelled with lactadherin-FITC, anti-CD41-APC and anti-CD36-PE. EVs are defined as phosphatidylserine-exposing (PS+) events ≤1000 nm.

**Results**: Initial results demonstrate that spFCM is able to measure EVs down to 100 nm. We additionally demonstrated that the bulk of EVs detected with spFCM are within the 100–300 nm range, which is in accordance with observations from previous studies. Additionally, concentration determination of EVs on spFCM was reproducible (CV = 3.68–7.32%), as was median positive channel fluorescence (MPCF) of EV phenotypes (CV = 1.44–6.63%). However, experiments are currently still ongoing and final results pending.

**Summary/Conclusion**: Although spFCM has been around for several years, few research groups have access to this platform due to its expensive and specialized nature. Thus, little is known about its applicability in the field of EV research, and to the authors’ knowledge, this study is the first to provide a direct benchmark against a more commonly used conventional FCM.

PS09.14 = OWP2.01

Isolation and phenotype characterization of microvesicle subpopulations from mixed cells in an *in vitro* model of lung microvascular injury

PS09.15

Nanoarray for single exosome-like extracellular vesicle proteomics

Philippe DeCorwin-Martin ^1^; Rosalie Martel^2^; Eun Hae Oh^1^; David Juncker^1^

^1^Biomedical Engineering Department, McGill University, Montreal, Quebec, Canada, Montreal, Canada; ^2^Biological & Biomedical Engineering Program, McGill University, Montreal, Quebec, Canada, Montreal, Canada

**Background**: The heterogeneity of extracellular vesicles (EVs) requires new tools to characterize subpopulations and elucidate the effects and mechanisms by which they shape cellular processes. Recently, significant progress has been achieved in flow cytometry and fluorescence microscopy for high-throughput analysis of high-abundance markers in single EVs but none have yet been validated for single proteins on single vesicles. Here, we identify exosome-like extracellular vesicle (ELEV) subpopulations from breast cancer cell lines enriched on nanoarrays with single-ELEV resolution and single-molecule sensitivity.

**Methods**: A nanoarray of anti-mouse IgGs was printed onto a glass slide using lift-off nanocontact printing, and the surface was passivated before incubation with mouse monoclonal capture antibodies. The nanoarray consists of 100 nm capture spots spaced 2 m apart that capture single ELEVs by virtue of their small size. ELEV samples, purified from cell supernatant using size exclusion columns, were incubated on the nanoarray overnight and detected using fluorescently tagged detection antibodies.

**Results**: Single ELEV capture was demonstrated on the nanoarray using AFM correlated with fluorescence microscopy. ELEVs could by detected with a single antibody as shown by single molecule photobleaching traces. Known exosome markers, integrins and general cancer markers were probed on exosomes derived from breast cancer cell lines, defining initial subpopulations.

**Summary/Conclusion**: The heterogeneity of EVs calls for methods that can measure single vesicles to allow for an accurate description of vesicle composition. With the nanoarray’s ability to enrich single ELEVs of interest in a high-throughput manner, ELEV subpopulations with unique co-expression patterns can now be studied for their distinct effects.

**Funding**: This study was funded by Genome Canada Disruptive Innovation in Genomics and NSERC.

PS09.16

Immunophenotyping extracellular vesicles by flow cytometry using CCD-based imaging technology

Sherree L. Friend; Haley R. Pugsley; Bryan Davidson; Phil Morrissey

Merck KGaA/MilliporeSigma, Seattle, USA

**Background**: Extracellular vesicles are membrane derived structures that include exosomes, microvesicles and apoptotic bodies. The importance of extracellular vesicles as key mediators of intercellular communication is not well understood. Exosomes have been shown to transfer molecules between cells, potentially transmitting signals. Exosomes are released under normal physiological conditions; however, they are also believed to serve as mediators in the pathogenesis of neurological, vascular, haematological and autoimmune diseases as well as cancer. Quantifying and characterizing extracellular vesicles in a reproducible and reliable manner is challenging due to their small size (exosomes range from 30 to 100 nm in diameter). Extracellular vesicle analysis can be done using high-magnification microscopy; however, this technique has a very low throughput. Attempts to analyse extracellular vesicles using traditional PMT based flow cytometers has been hampered by the limit of detection of such small particles and their low refractive index. To overcome these limitations, we have employed the Amnis imaging technology that has the advantage of high throughput flow cytometry with higher sensitivity to small particles due to the CCD based, time-delay-integration image capturing system.

**Methods**: Exosomes were purchased or obtained from different sources, stained with multiple labelled monoclonal antibodies and quantified by CCD-based flow cytometry. Sensitivity is calculated by standardizing each instrument to MESF standards.

**Results**: Data will be presented using the Amnis imaging technology to immunophenotype extracellular vesicles derived from different sources. Strategies to optimize detection of extracellular vesicles will also be discussed.

**Summary/Conclusion**: Amnis imaging technology is able to detect and phenotype exosomes with very high sensitivity.

PS09.17 = OWP2.07

Development of high sensitivity flow cytometry for sizing and molecular profiling of individual extracellular vesicles down to 40 nm

PS09.18

Characterization of extracellular vesicles by transmission electron microscopy: comparison of negative staining protocols

Linda Rikkert ^1^; Leon Terstappen^2^; Rienk Nieuwland^3^; Frank A.W. Coumans^4^

^1^Department of Medical Cell Biophysics, University of Twente, Enschede, The Netherlands, Amsterdam, The Netherlands; ^2^Department of Medical Cell BioPhysics, University of Twente, Enschede, The Netherlands, Enschede, The Netherlands; ^3^Laboratory of Experimental Clinical Chemistry, and Vesicle Observation Center, Academic Medical Center, University of Amsterdam, Amsterdam, The Netherlands, Amsterdam, The Netherlands; ^4^Department of Biomedical Engineering and Physics, and Vesicle Observation Center, Academic Medical Centre of the University of Amsterdam, Amsterdam, The Netherlands

**Background**: Transmission electron microscopy (TEM) is a high-resolution imaging technique capable to distinguish extracellular vesicles (EVs) from similar-sized non-EV particles. However, TEM sample preparation protocols are diverse and have never been compared directly to each other. In this study, we compare commonly applied negative staining protocols for their efficacy to detect EVs.

**Methods**: Four negative staining protocols were selected from literature, which differ in fixation of the EV sample and mounting of EVs to a TEM grid. These protocols were applied to a single sample of cell-free human urine. Images were taken at one selected image location and five predefined locations of the grids. The obtained images were compared for their qualitative and quantitative usefulness with respect to: morphology, EV count and quality of the obtained TEM images.

**Results**: EVs were detectable by all four protocols. However, at predefined locations, the EV recovery varied by twofold between protocols. Evaluation of image quality by four different researchers active in the EV field demonstrated a difference in image quality and suitability for EV research.

**Summary/Conclusion**: EV sample preparation protocols have a large influence on the TEM image quality. The sample protocol without fixation, carbon coated grids, blotting after sample mounting and short incubation with uranyl acetate was preferable over the other evaluated protocols based on numerical evaluation and overall image quality.

**Funding**: This work is supported by The Netherlands Organisation for Scientific Research – Domain Applied and Engineering Sciences (NOW-TTW), research programs VENI 13681 (Frank Coumans) and Perspectief CANCER-ID 14198 (Linda Rikkert).

PS09.19

Co-localization, counting and size characterization of single exosomes using a direct from sample surface capture based imaging technique

George Daaboul; Gabriel Reznik; Aditya Dhande; Amit Deliwala; David Freedman

nanoView Biosciences, Boston, USA

**Background**: One of the major barriers in EV research is the current limitations of analytical tools for the characterization of EVs due to their small size and heterogeneity. EVs span a range as small as 50 nm to few microns in diameter. Recently, flow cytometers have been adapted to combine light scatter measurements from nanoparticles with fluorescent detection of exosome markers. However, the small-size of exosomes makes specific detection above background levels difficult because large populations of small diameter vesicles (50–200 nm) are too small for traditional visualization technologies. Also, fluorescent surface marker detection is limited because of the reduced number of epitopes available to detect on a single particle.

**Methods**: To better characterize these small vesicles, we have developed a label-free visible-light microarray imaging technique termed Single Particle Interferometric Reflectance Imaging Sensor (SP-IRIS) that allows enumeration and sizing of individual nanovesicles captured on the sensor that has been functionalized with an array of membrane protein specific capture probes. Furthermore, we combined fluorescence detection with light scatter readout to co-localize multiple markers on individual EVs captured on the sensor surface. The fluorescence sensitivity was measured using fluorescent polystyrene nanoparticles with diameters of 20–200 nm, corresponding to 180–110,000 fluorescein equivalent units. The calculated fluorescence detection limit approaches single fluorescence sensitivity. SP-IRIS technology requires a sample volume of 5–100 µL with a detection limit of 5 × 10^5^ particles/mL.

**Results**: To demonstrate the utility of the SP-IRIS detection method we studied EV heterogeneity from three different pancreatic cancer cell lines (Panc1, Panc 10.05 and BxPC3) by arraying the surface with antibodies against CD81, CD63, CD9, Epcam, EGFR, Tissue Factor, Epcam, MHC-1, MHC-2 and Mucin-1. Furthermore, to demonstrate the applicability of the SP-IRIS technology for liquid biopsy we demonstrated detection of pancreatic cancer derived exosome spiked-in into human plasma.

**Summary/Conclusion**: The SP-IRIS direct-from-sample high-throughput technique could improve standardization of exosome preparations and facilitate translation of exosome-based liquid biopsies.

LBS07: Late Breaking Poster Session – Repair and Signalling Chairs: Costanza Emanueli; Geoffrey DeCoutoLocation: Exhibit Hall 17:15–18:30

LBS07.01

Exercise-induced muscle damage, extracellular vesicles and microRNA

Jason Lovett; Peter Durcan; Kathy Myburgh

Stellenbosch University, Stellenbosch, South Africa

**Background**: Extracellular vesicles (EVs) are nano-sized (30–1000 nm) mediators of intercellular communication. EVs are stable and abundantly present in biofluids such as blood. Circulating EVs are known to contain microRNA (miR). Divergent circulating EV miR profiles are present in healthy and pathological states. The miR profile of EVs may therefore provide useful information with regard to the physiological state of internal tissues. Skeletal muscle (SkM) is frequently injured during exercise or performance of other physical activities. It is difficult, however, to quantify the extent of injury or regeneration present in injured muscle. A reliable indicator of the muscle injury/regenerative status would therefore be useful.

**Methods**: An exercise intervention consisting of plyometric jumping and downhill running, previously verified as inducing mild SkM damage (mild z-line streaming), was performed by nine adult male subjects. Serum creatine kinase (CK) and plasma EVs were analysed at baseline, 2 and 24 h post-exercise. Perceived muscle pain (PMP) was assessed at 2, 24 and 48 h post-exercise. EVs were isolated using size exclusion columns and visualized with transmission electron microscopy (TEM). EV size and numbers were quantified by nanoparticle tracking analysis (NTA), and expression profiles of miR-1, 133a, 133b, 206 (myomiRs) and miR-31 were quantified with qPCR.

**Results**: PMP and CK were significantly elevated post-exercise (up to *p* < 0.001), providing indirect evidence for SkM damage. TEM revealed an abundant and heterogeneously sized pool of intact EVs. A concomitant abundance of EVs was seen with NTA (mean = 9 × 10^10^ particles/ml). Mean EV diameters were 127 ± 15 nm across all time points. No change in EV size or number was seen over time. The four myomiRs did not change following the exercise intervention. However, EV miR-31 decreased at 24 h post-exercise when compared to baseline (*p* < 0.05).

**Summary/Conclusion**: Rather than a change in circulating EV size, number or myomiR cargo, EV miR-31 decreased post-exercise-induced muscle damage. These data suggest that the miR profile of circulating EVs is altered in response to SkM injury, and selected EV miR profiles may be a useful tool in better understanding SkM injury severity.

**Funding**: This study was funded by The National Research Foundation of South Africa.

LBS07.03

Human mesenchymal stromal cell-derived exosomes promote wound healing in a mouse model of radiation-induced injury

Alexandre Ribault ^1^; Céline Loinard^1^; Stephane Flamant^2^; Sai Kiang Lim^3^; Radia Tamarat^1^

^1^IRSN, Fontenay-aux-Roses, France; ^2^IRSN, Fontenay-aux-roses, France; ^3^IMB A*STAR, Singapore, Singapore

**Background**: Mesenchymal stromal cells (MSCs) have been reported to promote tissue regeneration in numerous pre-clinical animal models, including radiation burns. MSC-derived exosomes (MSC-EXO) might be a main paracrine mechanism for these cells to mediate their therapeutic effect. Recent studies have shown that MSC-EXO could exert regenerative functions in several tissues, including skin and skeletal muscle. We hypothesized that MSC-EXO could participate to the wound healing process of radio-induced injury in mice.

**Methods**: Mice lower limb was exposed to 80 Gy X-ray irradiation to induce radiation injury. After 14 days, mice received an intramuscular injection of 106 human MSCs, 400 µg MSC-EXO or PBS. Animals were monitored weekly to establish an injury score based on the assessment of wound extent, ulceration, moist desquamation and limb retraction. Skin perfusion was evaluated by laser Doppler imaging. Mice were sacrificed at several time points, and tissues of both irradiated and contralateral limbs were harvested for histological and biochemical analyses. Bone marrow, spleen and blood were collected for analysis of inflammatory cells and circulating factors.

**Results**: MSC-EXO decreased the injury score at 7 and 14 days post-injection, compared to MSC and PBS groups, suggesting that MSC-EXO promote wound healing in a preventive manner. Irradiation increased skin perfusion in PBS-injected animals, while MSC-EXO and MSCs restored skin perfusion to levels similar to non-irradiated legs. Moreover, we found that MSC-EXO increased blood concentration of VEGF at day 3 post-injection, while MSCs tended to increase SDF-1α blood levels at 3 and 7 days post-injection. MSC-EXO enhanced the migration of irradiated endothelial cells *in vitro*, as compared to PBS. This effect was abrogated by TGF-β and PI3K inhibitors. Flow cytometry analysis revealed a significant decrease in monocyte population in spleen and blood by day 3 post-injection in MSC-EXO and MSC groups compared to PBS, suggesting a larger recruitment of monocytes to the injured site in these groups.

**Summary/Conclusion**: These results suggest that MSC-EXO exert a beneficial effect on the wound healing process in irradiation condition. In particular, MSC-EXO contributes to restore irradiated skin perfusion to normal levels. Further analyses are ongoing in order to determine MSC-EXO mechanisms of action.

LBS07.04

Extracellular vesicles from human iPS-derived cardiovascular progenitor cells stimulate the proliferation of cardiomyocytes in the injured heart

Bruna Lima Correa ^1^; Nadia El Harane^1^; Philippe Menasché^1^; Maria L. Perotto^2^; Laetitia Pidial^1^; Ivana Zlatanova^1^; Hany Nematalla^1^; Eliwabeth Woolaver^1^; Gabriel Ifergan^1^; Anaïs L. Kervadec^1^; Valérie L. Bellamy^1^; Nisa L. Renault^2^; Jean-Sébastien Silvestre^1^

^1^INSERM U970 – PARCC, Paris, France; ^2^INSERM U970, Paris, France

**Background**: Extracellular vesicles (EV) seem to mediate the benefits of cell therapy for ischaemic heart failure but their mechanism of action remains poorly understood. The doubly transgenic fate-mapping MerCreMer/ZEG mice model allows to distinguish whether new cardiomyocytes originate from the division of preexisting ones (GFP^+^, Troponin [TnT^+^]) or have differentiated from endogenous progenitors, in which case they stain positive for Lac Z and TnT but negative for GFP.

**Methods**: Myocardial infarction was induced in 12 MerCreMer/ZEG mice by permanent occlusion of the left anterior descending coronary artery.

Three weeks later, the surviving mice with a left ventricular ejection fraction (LVEF) ≤45% received transcutaneous echo-guided injections in the peri-infarct myocardium of EV from 1.4 × 10^6^ human iPS-derived cardiovascular progenitor cells (hiPS-CPg-derived EV) (hiPS-Pg; 10 × 109, *n* = 6) or PBS (*n* = 6). Seven days later, four mice (two in each group) were sacrificed for histological assessment. The remaining mice were blindly evaluated by echocardiography 6 weeks after injections, and their hearts were also processed for histology. In parallel, *in vitro* assays were developed to determine if fluorescently labelled EV were internalized in cultured rat cardiomyocytes.

**Results**: Seven days after EV injection, there was an average of 35 ± 10 cardiomyocytes in the infarcted area of the two treated hearts, which stained positive for TnT and LacZ but negative for GFP, suggesting that they differentiated from endogenous progenitors. Conversely, no TnT+ cardiomyocytes were identified in the scar of PBS-injected hearts. Six weeks after injections, cardiac function was only improved in the EV-treated hearts (*n* = 4), as evidenced by decreased LV volumes and increased LVEF (+16%) relative to baseline. *In vitro*, EV were internalized by CM and the transfer of their miRNA and protein payload was demonstrated by a positive intracellular staining for labels specific for these moieties.

**Summary/Conclusion**: hiPS-CPg-derived EV improve the function of chronically infarcted hearts, possibly, in parts, through EV-mediated miR and protein transfer fostering generation of new cardiomyocytes from endogenous sources.

**Funding**: This study was funded by INSERM; Universite Paris Descartes; The LabEx REVIVE; The FRM, AFM Telethon; The LeDucq Foundation.

LBS07.05

Comparison of non-coding RNA content of extracellular vesicles derived from Wharton’s Jelly, amniotic and chorionic mesenchymal stem cells

Andrew Hoffman ^1^; Airiel Davis^2^; Kristen Thane^1^; Dawn Meola^1^; Sally Robi nson^1^; Vicky Yang^1^

^1^Tufts University Cummings School of Veterinary Medicine, North Grafton, USA; ^2^Tufts University Cummings School of Veterinary Medicine, Grafton, USA

**Background**: Placental tissues as a source of mesenchymal stem cells (pMSC) have several advantages over other sources: they are generally discarded, non-controversial, harvest is non-invasive, and pMSC derived are highly replicative. Also, pMSC as extraembryonic, foetal cells exhibit “youthful” properties which may have therapeutic advantages. Extracellular vesicles are a principal component of the pMSC secretome that exert a similar range of biological activities as parent cells, yet the mechanisms are poorly understood. In particular it is unknown how pMSC and respective EV isolated from placental sites (Wharton’s Jelly, Amnion, Chorion) differ with respect to non-coding RNA including important functional biotypes (miRNA, Y RNA). 

**Methods**: The study was performed using placental tissues from littermate (*n* = 5) puppies. Stepwise ultracentrifugation was employed to isolate EV from serum-free culture supernatants of pMSC cultured from Wharton’s Jelly (WJ-MSC), amniotic (AM-MSC) and chorionic (C-MSC) tissues. RNA was isolated using miRNeasy, Truseq small RNA library prepared and RNAseq (HiSeq2500, 50 nt reads) performed. We analysed RNA biotypes and quantified miRNA from each type of pMSC and respective EV.

**Results**: All pMSC types substantially over-expressed miRNA compared to EV. Conversely, EV content was significantly higher than parent pMSC for rRNA, Y RNA (RNY-4,-3, and -1) fragments, lincRNA, anti-sense RNA, RN7SKP and snRNA. The rank of miRNA prevalence was not different between pMSC and EV, with highest reads for miR-21 > miR-22 > miR-199. Significant differences in miRNA read counts were noted only for miRNA with low read counts (C-MSC lower in miR-23a, miR-107 and miR-130a).

**Summary/Conclusion**: pMSC are a rich source of EV with substantial Y RNA content (~25% total small RNA) and miRNA (1–2% total small RNA) but differences between subtypes (WJ-, AM-, C-MSC) were subtle, suggesting convergence of pMSC small RNA packaging into EV from these placental sources.

**Funding**: This study was funded by Shipley Foundation.

LBS07.06

Identification of exosomes in the infective stage of entomopathogenic nematodes

Duarte Toubarro ^1^; Jorge Frias^1^; Antonio Marcilla^2^; Alicia Galiano^2^; Nelson Simões^1^

^1^Biotechnology Center of Azores, University of Azores, Portugal, Ponta Delgada, Portugal; ^2^Departament de Farmàcia I Tecnologia Farmacéutica i Parasitologia, Universitat de Valéncia, Spain, BURJASSOT (VALENCIA), Spain

**Background**: Entomopathogenic nematodes are insect parasitic nematodes able to kill the host short after the contact. Currently, the pathogenicity of these organisms is ascribed to excretory/secretory products (ESP), released by the infective nematode. In an attempt to identify the molecular effectors, we noticed the presence of exosome-like vesicles for the first time in *Steinernema carpocapsae*.

**Methods**: Exosomes were isolated from the ESP by size-exclusion chromatography (Sepharose CL-2B) and particle size was determined by nanotracking analysis (NTA). Proteomic profile of exosomes was determined by MS/MS analysis. Exosomes in induced nematodes were detected by TEM analysis and size estimated.

**Results**: Although almost 90% of the exosomes had a predicted size determined by TEM between 30 and 130 nm, the global size distribution determined by NTA ranged from 90.6 nm to 201.6 nm being the mean 146.1 nm and the mode 161.2 nm. The majority of exosomes detected by TEM were localized near to nematode lateral fields or alae and a few crossing the cuticle. MS/MS analysis of exosome vesicles allow to the identification of filament disassembly proteins (e.g. unc-78, dynamin, dystonin, titin), several cytoskeletal-related proteins (actin, tubulin, α-actinin and myosin) and vesicle transport-related proteins (clathrin, transthyretin), which are proteins known to be released by cells through a vesiculation process. However, the majority of proteins identified in S. carpocapsae exosomes belong to molecular binding and catalytic activity categories. In the former category, protein binding (GO:0005515) and carbohydrate binding (GO:0030246) were the most represented, and in the second category metalloendopeptidases and serine peptidases were the most relevant.

**Summary/Conclusion**: Our findings reveal that exosomes are another mechanism by which EPNs interact with the host providing a mechanical way for the delivery of molecular effectors.

**Funding**: This research was supported by FP7 project (BIOCOMES). DT gratefully acknowledges for the FCT research grant (SFRH/PBD/77483/2011) and FRCT grant (M3.1.a/F/050/2016) and JF for the Biocomes research grant (FP7 Grant Agreement no. 612713) and for the FCT studentship (SFRH/BD/131698/2017).

LBS07.07

Vesicular release of Interleukin-36γ is Toll-like receptor dependent

Christopher J. Papayannakos ^1^; Daniel Zhu^1^; Ali Rana^1^; James DeVoti^2^; Lionel Blanc^3^; Vincent Bonagura^2^; Bettie Steinberg^1^

^1^Oncology Center, The Feinstein Institute for Medical Research, Manhasset, New York, United States, Manhasset, USA; ^2^Center for Immunology and Inflammation, The Feinstein Institute for Medical Research, Manhasset, New York, United States, Manhasset, USA; ^3^Center for Autoimmune, Musculoskeletal and Hematopoietic Diseases, The Feinstein Institute for Medical Research, Manhasset, New York, United States, Manhasset, USA

**Background**: Interleukin-36γ (IL36γ) is a cytokine central to epithelial immunology that can promote both inflammatory and wound healing responses. Induction and release will occur from primary human foreskin keratinocytes (HFK) stimulated with poly-I:C (pIC), a TLR3 agonist or flagellin, a TLR5 agonist, in the absence of necrotic cell death. There is evidence that IL36γ can be actively secreted as a cargo of extracellular vesicles (EV) post-pIC exposure. Specific packaging and non-classical release mechanisms of IL36γ are not understood.

**Methods**: Signalling studies were performed with small molecule inhibitors to NF-kB, Nrf2 and autophagy. Soluble IL36γ secretion was measured by ELISA. EV isolation from conditioned media and subcellular fractionation were performed by differential centrifugation through density gradients. Immunoblot was used for protein analysis.

**Results**: Inhibition of Nrf2 under pIC but not flagellin-stimulation results in a significant decrease in IL36γ expression. NF-kB does not play a significant role in regulating IL36γ. Soluble secretion kinetics reveal an earlier accumulation of full-length IL36γ with flagellin over that of pIC. IL36γ is released in association with extracellular vesicles (EVs) only during pIC stimulation. Characterization of markers from EVs pelleted from pIC- and flagellin-treated HFK conditioned media is positive for ALIX, TSG101, Hsc70 and Flotillin-1. The levels of these markers are elevated in the pellets following treatment with either agonist compared to untreated controls, indicating similar levels of EVs released during stimulation. Released EVs from pIC treatment float between 1.09 and 1.11 g/mL consistent with the density of exosomes. Subcellular fractionation indicates that post-pIC exposure, IL36γ tracks with intracellular vesicles positive for Hsc70 more so than TSG101. This provides evidence that IL36γ is present in multiple populations of small EVs. Finally, we have made the novel observation that the previously described post-translational processing of IL36γ may be taking place within an Hsc70+ compartment.

**Summary/Conclusion**: These data support a pIC-mediated vesicular release mechanism for IL36γ and a novel example of the selective packaging of a cytokine as a small EV cargo.

**Funding**: This research was supported in parts by R01 DE017227-06A1.

LBS07.08

T-cell synaptic ectosomes relay signals through microcluster transfer

Stefan Balint; David G. Saliba; Pablo F. Cespedes; Ewaldus B. Compeer; Salvatore Valvo; Michael L. Dustin

The Kennedy Institute of Rheumatology, University of Oxford, Roosevelt drive, Headington, Oxford OX3 7FY, Oxford, United Kingdom

**Background**: Extracellular vesicles (EV) are proposed to transfer information between cells. In the immunological synapse T cell receptor (TCR) interaction with pMHC drives microcluster formation and signalling that is terminated in parts through sorting of TCR into EVs that bud into the synapse, synaptic ectosomes (SE). Previously, we used correlative light and electron microscopy to characterize SEs. However, this approach has some limitations such as the poor resolution of fluorescent signals and the lack of information on receptor organization in individual SE.

**Methods**: SE released by CD4 T cells were captured on planar supported lipid bilayer (PSLB) containing either ICAM1, ICAM1 and aCD3 or ICAM1, aCD3, CD40 and ICOSL. SEs were stained with WGA to visualize the membrane and with directly conjugated antibodies against TCR, CD40L, ICOS, BST2 and imaged by multicolour dSTORM. To assess functionality of released SEs, DCs maturation after incubation with SEs was measured by cytokine array.

**Results**: SEs released onto PSLB containing ICAM1, CD40, ICOSL and aCD3 were ~80 nm in size and about 40 SEs were released per cell. Three subsets of SEs were observed, one having only TCR, another having only CD40L, ICOS or BST2, and the majority double positive for TCR and CD40L, ICOS or BST2. TCR microclusters colocalized with ICOS and BST2, but segregated from CD40L within single SEs. This is consistent with TCR, BST2 and ICOS occupying overlapping microclusters, whereas TCR and CD40L occupy spatially distinct microclusters. Distinct TCR and CD40L microclusters on SEs transferred to PSLB can stimulate DCs in an antigen independent manner. Our data suggest that T cells release different subsets of SEs with different protein composition that constitute functional units to activate DCs.

**Summary/Conclusion**: We propose that SE transfer perpetuates the biological impact of receptors and ligands coclustering in interfaces beyond the separation of the interacting cells. This enables T cells to distribute information in cellular networks within tissues. Linkage of TCR and CD40L clusters in single SE offers additional opportunities for specificity and synergy. SEs provide a general strategy to perpetuate signals initiated in cell–cell interfaces beyond the period of synapsis.

**Funding**: This study was funded by ERC AdG 670930, Wellcome Trust 100262, Kennedy Trust, NIH AI043542, NIH tetramer core facility, EMBO ALTF 1420-2015.

LBS07.09

MicroRNA-containing microvesicles of healthy origins: a potential tool for the therapy of atherosclerosis

Adriana Georgescu; Nicoleta Alexandru; Florentina Safciuc; Alina Constantin; Miruna Nemecz; Gabriela Tanko; Alexandru Filippi; Emanuel Dragan; Maya Simionescu

Institute of Cellular Biology and Pathology ‘Nicolae Simionescu’ of Romanian Academy, Bucharest, Romania

**Background**: Microvesicles, endothelial progenitor cells (EPC) and circulating microRNAs (miRNAs) are considered as regulators in atherosclerosis but their direct effect in the vascular repair process is controversial. We questioned whether blood cell-derived microvesicles (bMV) and EPC-derived microvesicles (eMV) isolated from healthy hamsters influence atherosclerosis development in hypertensive-hyperlipidaemic hamsters and investigated the mechanisms underlying their repair capacity.

**Methods**: Forty hamsters were divided into: (1) simultaneously hypertensive-hyperlipidaemic (HH); (2) HH receiving bMV or eMV (by retro-orbital injection) from healthy hamsters and (3) normal healthy animals used as controls (C). All experimental procedures were conducted in accordance with the Helsinki Declaration.

**Results**: We found that (1) bMV/eMV transplantation suppressed development of atherosclerosis by (i) alleviation of dyslipidaemia, hypertension, increased circulating EPC levels and reduced cytokine/chemokine profiles (VEGF, IL-6, IL-8); (ii) structural and functional remodelling of the vessel wall and heart; (2) bMV/eMV operate as protective and delivery system for miRNAs in the circulation and serve as intercellular carriers of functional Ago2-miRNA, Stau1-miRNA and Stau2-miRNA complexes; (3) bMV/eMV protect against atherosclerotic vascular disease via miR-10a, miR-21, miR-126, miR-146a transfer to circulating EPC. The favourable effects were similar for eMV and bMV.

**Summary/Conclusion**: Transplantation of healthy bMV/eMV counteracts HH diet-induced detrimental effects by transferring miRNAs they carry to circulating EPC. The findings indicate that bMV/eMV could be employed as therapeutic tools for transferring miRNAs in atherosclerosis suggesting that they may become a novel remedy for cardiovascular diseases.

**Funding**: Work was supported by grants of the Romanian National Authority for Scientific Research, CNCS-UEFISCDI, project no. PN-III-P1-1.2-PCCDI-2017-0527 and Romanian Academy.

LBS07.10

Pancreatic islet-secreted exosomal microRNA-29 family members travel to liver and promote hepatic insulin resistance

Jing Li; Yujing Zhang; Xiaohong Jiang

Nanjing University, Nanjing, China (People’s Republic)

**Background**: Secreted microRNAs are novel endocrine factors that play roles in diseases. We found that miR-29 family member (miR-29a/b/c) in plasma and pancreatic islet was remarkably increased. Further analyses suggested that the increased circulating miR-29s in obese subjects were likely released by pancreatic islets under conditions of high plasma FFAs. Intravenous injection of secreted miR-29s causes mouse insulin resistance.

**Methods**: In order to investigate the possibility and function of pancreatic islet-derived secreted miR-29s in circulation, three types of transgenic mice were generated. First, to mimic the FFA-induced expression of miR-29s in obese subjects, we generated transgenic mice expressing miR-29s under control of the insulin promote. Second, to directly identify the recipient tissue of secreted miR-29s, we synthesized a heterogeneous miRNA (mutant miR-29a). Using this distinguishable miRNA, we generated another transgenic mouse model (miR-29a mut mice) that specifically expressed mutant miR-29a in the pancreatic islet. To critically test the pathological role of pancreatic islet-secreted miR-29s in obesity-associated IR, we generated a third type of transgenic mouse model-miR-29s-deficient (miR-29s def) mice. This animal model expresses a sponge target construct, which is designed to complement with miR-29s and adsorb the endogenous miR-29s.

**Results**: In miR-29s TG mice, the secretion of miR-29s increased and insulin suppression of hepatic gluconeogenesis was inhibited. Tracking a distinguishable mutant miR-29a that *in vivo* selectively overexpressed in β-cell revealed that pancreatic islet-derived miR-29s were predominantly delivered to liver, and caused IR via supressing PI3K-pathway. Moreover, *in vivo* disruption of miR-29s expression in β-cell restored HFD-induced IR. *In vitro* studies demonstrated that isolated exosomes containing high levels of miR-29s inhibited PI3K-pathway and increase hepatic glucose production. These results elucidate a new β-cell function and its possible relation to type 2 diabetes.

**Summary/Conclusion**: Pancreatic islet can secrete miR-29s into circulation under chronic high-level FFAs condition; secreted miR-29s can travel to liver and promote hepatic insulin resistance through inhibiting PI3K-pathway.

LBS07.11

Walnuts supplementation alter exosomal miRNA in elderly subjects

María-Carmen López de las Hazas ^1^; Judit Gil-Zamorano^1^; Montserrat Cofán^2^; Diana C. Mantilla-Escalante^1^; María Yáñez-Mó^3^; Joan Sabaté^4^; Emilio Ros^5^; Alberto Dávalos^1^; Aleix Sala-Vila^5^

^1^Laboratory of Epigenetics of Lipid Metabolism, Instituto Madrileño de Estudios Avanzados (IMDEA)-Alimentación, CEI UAM+CSIC, Madrid 28049, Spain., Madrid, Spain; ^2^Lipid Clinic, Endocrinology and Nutrition Service, Institut d´Investigacions Biomèdiques August Pi i Sunyer, Barcelona 08036, Spain. CIBER Fisiopatología de La Obesidad y Nutrición (CIBEROBN), Instituto de Salud Carlos III, 28029 Madrid, Spain., Barcelona, Spain; ^3^Departamento de Biología Molecular. UAM, Madrid, Spain; ^4^Center for Nutrition, Healthy lifestyle and Disease prevention, School of Public Health, Loma Linda University, Loma Linda, CA, 92350, USA, loma Linda, USA; ^5^Lipid Clinic, Endocrinology and Nutrition Service, Institut d´Investigacions Biomèdiques August Pi i Sunyer, Barcelona 08036, Spain. CIBER Fisiopatología de La Obesidad y Nutrición (CIBEROBN), Instituto de Salud Carlos III, 28029 Madrid, Spain., Barcelona, Spain

**Background**: Regular consumption of walnuts has been associated with many health benefits. However, the underlying mechanism of these effects remains understudied. Recent studies suggest that dietary compounds may modify the secretion of exosomes. Exosomes are nanovesicles secreted to the extracellular space and are involved in intercellular communication and associated with different physiological and pathological processes. It is widely known that exosomes transport in their cargo different molecules including microRNAs (miRNAs). miRNAs are post-transcriptional small non-coding RNAs regulators and are present in both, tissues and biological fluids. miRNAs are thought to mediates intercellular communication and their identification are valuable biomarkers of diseases.

**Methods**: Therefore, we aimed to determine whether 1-year walnuts intake providing 15% of daily energetic requirements in elderly people might modulate the secretion of exosomes and/or modulate their cargo and miRNAs content to promote body health.

**Results**: Although no significative changes in exosome particle size in response to dietary supplementation with walnuts were found, our results showed a differential secretion of miRNAs which are transported in exosomes. Functional analysis and possible tissue of origin suggest that modulated miRNAs participate in diverse biological pathways.

**Summary/Conclusion**: Overall, our data suggest the modulation of the expression of miRNAs through a pharma-nutrition approach might be a viable alternative or adjunct to current pharmacologic therapy targeting circulating miRNAs.

**Funding**: This research was supported by a grant from the California Walnut Commission, Sacramento, CA, USA. The funding agency had no involvement in any stage of the study. Also supported by the ISCIII and the European FEDER Funds through Fondo de Investigación Sanitaria (PI15/01014), by the Spanish Agencia Estatal de Investigación and European Feder Funds (AGL2016-78922-R), and Fundación Ramón Areces (Madrid, Spain). AS-V is a recipient of the ISCIII Miguel Servet fellowship (CP12/03299). MCLH was supported by a postdoctoral research contract funded by the community of Madrid and the European Union (PEJD-2016/BIO-2781). DCM-E is a fellow of “Centro de Estudios Interdisciplinarios Básicos y Aplicados” (CEIBA), Colombia, through the program “Bolivar Gana con Ciencia”.

LBS07.12

Mesenchymal stem cell-derived exosomes for treating liver fibrosis

Soyoung Son; Hwa Seung Han; Hansang Lee; Sol Shin; Jae Hyung Park

Sungkyunkwan University, Suwon-si, Republic of Korea

**Background**: Currently, treatment for liver fibrosis is slowing the progress of symptoms and deterioration of liver function. The unique property of Mesenchymal stem cells (MSCs) has made them a promising strategy for the treatment of liver fibrosis since MSC can reduce liver inflammation, promote hepatic regeneration, secrete protective cytokines and differentiate into hepatocytes. However, there is a limitation such as a tumour formation or cellular rejection. To solve those limitations, we suggested exosome-based therapy as a new therapeutic approach.

**Methods**: Human adipose-derived MSCs-derived exosome (A-Exo) was purified using a Tangential Flow Filtration System. The physicochemical characteristics of A-Exo were confirmed by electron microscopy and nanoparticle tracking analysis. Immunofluorescence analysis was conducted in order to examine the effect of A-Exo on the expression level of α-Smooth Muscle Actin (α-SMA). Mice model of liver fibrosis was prepared by intraperitoneally administering Thioacetamide. The exosome sample was administered intravenously and the effect of alleviating hepatic fibrosis was verified. Blood was collected and the level of ALT, ALP, TBIL (total bilirubin) and TP (total protein) were measured. The therapeutic efficacy was also evaluated by measuring the weight ratio of the liver to the total weight.

**Results**: The expression level of α-SMA was increased in activated hepatic stellate cells treated with TGF-β1, while the expression level was decreased depending on the treatment concentration of A-Exo. The amount of RNA of the fibrosis-related factors was decreased when the activated hepatic stellate cells were treated with exosomes. In *in vivo* experiments, a substantial accumulation of A-Exo was observed in liver, and liver function was improved by administration of A-Exo.

**Summary/Conclusion**: A-Exo was developed to overcome the problems of the conventional chemotherapy or stem cell therapy. The effects of A-Exo were confirmed by *in vitro* cell experiment and *in vivo* mice model. In mice model of liver fibrosis, A-Exo effectively inhibited the formation of fibrous septa as well as maintained the structural morphology of hepatocytes, thereby suppressing the fibrosis of liver tissue. Overall, A-Exo exhibited potential for a new therapeutic strategy for liver fibrosis.

LBS07.13

The use of exosomes as an important tool to kidney recellularization

Eliezer Francisco. De Santana ^1^; Fernanda Rocha. De Souza^1^; Aline Da Silva^1^; Antonio S. Novaes^2^; Nádia K Guimarães-Souza^1^

^1^Institute of Education and Research of The Brazilian Jewish Beneficent Society Albert Einstein, São Paulo, Brazil; ^2^Federal University of São Paulo, São Paulo, Brazil

**Background**: Chronic kidney disease is a worldwide growing problem. The kidney has ability for nearly complete regenerate itself after ischaemia/reperfusion or toxic injury. However, in some injuries the kidney develops fibrosis with loss of function. In recent years, the progression mechanisms for kidney disease and possible interventions have been on focus of studies. Some progression factors, such as growth factors (GF) that can lead to regeneration have been described like the hepatocyte growth factor (HGF). Recently, cell communication between mesenchymal stem cells (MSC) and renal epithelial cells has been recognized. HGF presence in exosomes (EXO) produced by MSC may be a source for regeneration stimulus. The main purpose of this project is to evaluate the effect of EXO from umbilical cord MSC on the adherence and proliferation of primary renal cell growing in a decellularizated porcine matrix.

**Methods**: The methodology consisted of three main steps: characterization of human renal cells in culture; obtaining the EXO from umbilical cord MSC and its characterization by Western blot (Cd63 and Cd81); and decellularization of porcine kidney by the sodium dodecyl sulphate (SDS) decellularization method for 24 h. The products of these three steps were mixed together for the recellularization experiment.

**Results**: Human primary kidney cells growth in cultures. Porcine decellularized kidney tissue preserved the architecture. EXO from MSC were positive for Cd63 and Cd81 in Western blot. While there were no cells before the recellularization, the decellularized renal tissue presented cells after the process of recellularization with direct influence of the EXO and GF. The presence, adherence and proliferation of renal cells were confirmed by histological analysis with haematoxylin by microscopy.

**Summary/Conclusion**: EXO from MSC showed an influence in the adherence and proliferation of human renal cells growth in a porcine kidney scaffold.

**Funding**: This research project was funded by FAPESP.

LBS07.14

Profiling extracellular vesicles derived from equine mesenchymal stem cells and tendon derived cells for tendon regeneration

Giulia Sivelli; Roger K. Smith; Isé François; Jayesh Dudhia

Royal Veterinary College, North Mymms, United Kingdom

**Background**: Tendon injuries represent a clinical challenge for treatment in human and horses. EVs secreted by mesenchymal stem cells (MSCs) are known to be involved in repair and inflammation resolution processes in different tissues and animal species. The main aim of this study is to investigate the role of EVs derived from MSCs and tendon derived cells (TDCs) in promoting tendon regeneration and inflammation pro-resolution pathways via paracrine mediated cellular communication.

**Methods**: An equine *in vitro* model of tendon inflammation was used to characterize EVs released by IL-1β stimulated equine MSCs and TDCs at 24 and 48 h. The amount of EVs harvested from the media was assessed by FACS. The chosen parameters were optimal to detect microspheres from 0.1 to 1 μm diameter simultaneously on the FSC-PMT and Annexin V conjugated with PE was used to portray the positive fluorescent events in a SSC/FSC-PMT graph. EVs were acquired at medium flow rate for 1 min. Aliquots of fresh media were tested in the same conditions to establish EVs background presence.

**Results**: FACS analysis conducted on media (*n* = 3 horses) showed a basal expression of EVs in control conditions. There is no significant difference in EVs numbers produced by either cell types under IL-1β stimulation vs control conditions (no IL-1β) at 24 h (*p* = 0.089) and 48 h (*p* = 0.768).

**Summary/Conclusion**: Although, the IL-1β stimulus does not induce a change in the quantity of EVs, it may trigger a qualitative change in the EV cargo. We are currently investigating the potential effect of IL-1 activated EVs to modulate the expression of inflammation pro-resolution markers.

**Funding**: Giulia Sivelli is funded by the European Union Horizon 2020 Programme (H2020-MSCAITN-2015) under the Marie Skłodowska-Curie Grant Agreement No. 676338.

LBS07.15 = OWP1.06

Extracellular vesicles isolated from cardiosphere-derived cells and mesenchymal stem cells elicit distinct immunomodulatory properties *in vitro* and *in vivo*

Ann-Sophie Walravens; Sasha Smolgovsky; Lauren Kelly; Kiel Peck; Linda Marbán; Geoffrey de Couto; Luis R.-Borlado

Capricor Therapeutics, Inc., Beverly Hills, USA

**Background**: Cardiosphere-derived cells (CDCs) possess cardioprotective, regenerative and immunomodulatory characteristics when delivered to the heart post-myocardial infarction (MI). These effects are recapitulated by CDC extracellular vesicles (EVs; CDC-EVs) in acute and chronic models of MI. It has been reported that mesenchymal stem cell (MSC) extracellular vesicles (MSC-EVs) confer some immunomodulatory effects in different indications. Thus, here we compared CDC-EVs to MSC-EVs by examining their RNA cargo and testing their ability to modulate macrophage function *in vitro* and *in vivo*.

**Methods**: CDCs and MSCs were cultured for 15 days in serum-free media and then conditioned media collected, filtered and concentrated by ultrafiltration (10 kDa MWCO) to isolate EVs. Differences in CDC-EV (*n* = 12) and MSC-EV (*n* = 4) RNA cargo was determined by small RNA-seq (NextSeq 500, Illumina). The functional effect of EVs was tested on macrophages both *in vitro* and *in vivo*. For our *in vitro* assays, activated peritoneal macrophage were treated with vehicle, CDC-EVs or MSC-EVs and then assessed for proinflammatory gene expression by qPCR. For our *in vivo* assays, mice were stimulated with zymosan (intraperitoneal injection) and then treated with vehicle, CDC-EVs or MSC-EVs (intravenous injection). Forty-eight hours later, peritoneal macrophages were isolated and analysed by flow cytometry.

**Results**: RNA-seq analysis revealed a greater overall abundance of Y RNA fragments and distinct miR composition in CDC-EVs compared to MSC-EVs. When examining the origin of EV-derived Y RNA fragments, a greater proportion of Y4-derived (*p* < 0.05), but lower amount of Y5-derived (*p* < 0.05), Y RNA were observed in CDC-EVs. *In vitro*, macrophages treated with CDC-EVs (*n* = 5), in contrast to MSC-EVs (*n* = 4), induced a dose-dependent increase in anti-inflammatory genes (*p* < 0.01). *In vivo*, CDC-EVs (*n* = 6) significantly reduced (*p* < 0.05) the accumulation of CD11b+F4/80+ peritoneal macrophages compared to MSC-EVs (*n* = 4).

**Summary/Conclusion**: Here, we show that CDCs and MSCs produce intrinsically different EV populations. We demonstrate that both the RNA composition and the functional effects exerted on macrophages are distinct. Together, these data support the therapeutic utility of CDC-EVs in a range of inflammatory diseases.

LBS08: Late Breaking Poster Session – Biogenesis Chairs: Susanne Gabrielsson; Malene Joergensen Location: Exhibit Hall 17:15–18:30

LBS08.01

Systems biology analysis reveals that several common diseases are associated with genes involved in the biogenesis of extracellular vesicles

András Gézsi; Anita Varga; Edit I. Buzás

MTA-SE Immune-Proteogenomics Extracellular Vesicle Research Group, Budapest, Hungary

**Background**: Extracellular vesicles (EVs) have received considerable attention in recent years because of mediating cell-to-cell communication in a wide variety of physiological and pathological processes. However, research on whether certain diseases are associated with genes that participate in the biogenesis of EVs remains less studied. The aim of our study was to determine the relationships between key genes in EV biogenesis and diseases using systems biology approaches.

**Methods**: We recently developed a Quantitative Semantic Fusion System, which allows efficient prioritization of diverse biological entities such as genes, taxa, diseases, phenotypes and pathways. By (1) constructing computation graphs over the entities and their pairwise relations and (2) setting evidences on certain entities, the system prioritizes all other entities by propagating the evidences through the network. We selected genes that participate in EV biogenesis by prior expert knowledge, and prioritized diseases and disease categories based on different computation networks. *p*-Values of prioritization results were computed by permutation tests.

**Results**: EV biogenesis genes are significantly associated with several diseases, including cardiovascular diseases (*p* = 0.01) such as heart failure (*p* = 0.02) and myocardial reperfusion injury (*p* < 0.01); pathologic functions (*p* = 0.01) such as neoplasm invasiveness (*p* < 0.01) and gliosis (*p* = 0.03). Pathway-mediated analysis (i.e. which diseases are associated with genes that participate in the same pathway as EV biogenesis genes) raises the possible association of many common diseases such as diabetes (*p* = 0.02), Alzheimer’s disease (*p* < 0.01) and obesity (*p* = 0.05).

**Summary/Conclusion**: Genes that participate in the biogenesis of EVs are significantly associated with numerous common diseases, including different types of tumours and cardiovascular diseases, which further emphasizes the key role of EVs in human health and disease.

**Funding**: This work was supported by the National Scientific Research Program of Hungary (OTKA) grant nos. 112872, 111958 and 120237.

LBS08.02

Lipid-modulated exosomal miRNAs

Diana Carolina Mantilla-Escalante; María-Carmen López de las Hazas; Judit Gil-Zamorano; Maria del Carmen Crespo; Andrea Del Saz-Lara; Almudena García-Ruiz; Alberto Dávalos

Laboratory of Epigenetics of Lipid Metabolism, Instituto Madrileño de Estudios Avanzados (IMDEA)-Alimentación, CEI UAM+CSIC, Madrid 28049, Spain., Madrid, Spain

**Background**: Excessive consumption of fat and lack of physical activity promotes lipid metabolism dysregulation such as dyslipidaemias. Increasing evidence suggest that cells are able to communicate through the secretion of nanovesicles called exosomes. Exosomes are small vesicles (30–150 nm) capable of carrying RNAs (including microRNAs) and other types of molecules. microRNAs are small non-coding RNAs that post-transcriptionally regulate gene expression and can be used as biomarkers of different diseases.

**Methods**: The aim of this study is to characterize the exosome miRNA content after the acute intake of dietary fats. For this, miRNAs were isolated from plasma samples of mouse fed with dietary lipids and analysed by RT-qPCR. An initial screening of more than 700 microRNAs was carried out in plasma samples.

**Results**: Of the total number of microRNAs analysed, only around 400 were detected, of which 32 potential candidates were validated in a second cohort of mice plasma samples. Dietary modulated miRNAs were searched in exosomes and only one miRNA candidate was consistently found to be modulated by dietary fats.

**Summary/Conclusion**: In conclusion, our study shows that microRNAs may change their expression in exosomes due to lipid dietary intake. Although more studies are needed, microRNAs could be considered as possible targets for the therapeutic treatments for diseases associated to lipid metabolism.

LBS08.03

Role of exosomes in chemotherapy-induced bystander effect

Arinzechukwu Ude; Michael Ladomery; Ruth Morse

University of the West of England Bristol, Bristol, United Kingdom

**Background**: A recent phenomenon is donor cell leukaemia (DCL) where transplanted haematopoietic stem cells become malignant in the recipient while the donor remains healthy. We hypothesized that chemotherapy treated mesenchymal stem cells (MSC) of the bone marrow produce a bystander effect during transplantation via exosomes trafficking microRNAs. Bystander effect occurs when treatment signatures or biological effects are induced in unexposed cells which are in close proximity to the directly exposed cells, via intercellular communication. Exosomes are small 50–100 nm vesicles that play an integral role in intercellular communication via uptake of lipids, microRNAs, mRNAs and proteins by recipient cells.

**Methods**: The MSC cell line, HS-5, was treated with and without the known exosome inhibitor, GW4869 (5, 10 and 20 µM) for an hour and then clinically relevant doses of chlorambucil (40 µM), carmustine (10 µg/ml), etoposide (10 µM) and mitoxantrone (500 ng/ml) for 24 h. The drugs were washed off and HS-5 cells were then co-cultured with the TK6 lymphoblast cell line (bystander). HS-5 cells were subjected to scanning electron microscopy (SEM) to confirm release of exosomes post-treatment. Bystander damage in the TK6 cells was initially assessed by cytotoxicity using trypan blue exclusion.

**Results**: SEM demonstrated increased release of microvesicles from treated HS-5 without GW4869. However there was no evidence that cytotoxicity of any chemotherapeutic drug was reduced by GW4869 treatment, irrespective of the concentration used.

**Summary/Conclusion**: Our current data suggests that exosome trafficking plays a role in cellular communication in the BM, but does not affect cytotoxicity of bystander cells. This may be important if bystander cells survive in a genotoxic environment, which remains to be assessed.

**Funding**: This study was funded by University of the West of England (UWE) Bristol, UK and Petroleum Development Trust Fund (PTDF), Nigeria.

LBS08.04 = OWP3.08

Evidence for selective mRNA sorting into cancer exosomes

Mohammad Arshad Aziz ^1^; Fatima Qadir^2^; Ahmad Waseem^2^; Muy-Teck Teh^2^

^1^University of Otago, Dunedin, New Zealand; ^2^Centre for Oral Immunobiology & Regenerative Medicine, Institute of Dentistry, Barts & The London School of Medicine and Dentistry, Queen Mary University of London, England, United Kingdom., London, United Kingdom

**Background**: Exosomes are membrane bound vesicles released by cells into their extracellular environment. It has been shown that cancer cells exploit this mechanism for local and/or distant oncogenic modulation. As it is not clear if oncogenic mRNA molecules are sorted selectively or randomly into exosomes, this study investigated using a cell culture model.

**Methods**: Exosomes were isolated using an established ultracentrifugation method from cell culture supernatant of a premalignant buccal keratinocyte (SVpgC2a) and a malignant (SVFN10) cell lines. Exosome and cell debris pellets were then subjected to RNase A and proteinase K protection assays prior to extraction of total RNA for reverse transcription quantitative PCR (RT-qPCR) to quantify mRNA of 15 expressed genes.

**Results**: RNA in cell debris pellet were sensitive to RNase A treatment but exosomal RNA were resistant to RNase A. Pre-incubation of exosome pellet with Triton-X to solubilize membranes rendered exosomal RNA sensitive to RNase A, indicating that exosomal RNA was protected within exosomal membranes. RT-qPCR showed that mRNA were present within exosomes. Of the 15 genes selected for RT-qPCR in this study, two (FOXM1 and HOXA7) were found to be more abundant in exosomes secreted from the malignant SVFN10 cells compared to the premalignant SVpgC2a cells. RNase A pretreatment on exosomal pellet did not degrade FOXM1 and HOXA7 mRNA suggesting that these mRNA were protected within exosomes. Interestingly, one gene (ITGB1), although abundantly expressed in parental cell, was not resistant to RNase A pretreatment indicating that not all mRNA purified from the exosomal pellet were sorted into the vesicles.

**Summary/Conclusion**: In conclusion, this study presented the first evidence that mRNA molecules were found to be protected within exosomes secreted by human buccal keratinocytes. Furthermore, we presented evidence for selective sorting of specific mRNA molecules into exosomes which is independent of parental cell mRNA concentration. This suggests that tumour cells preferentially package certain oncogenes in their exosomes as a potential intercellular vehicle for reprograming target cells. Signature of mRNA contents within cancer exosomes may have clinical applications for diagnostic and therapeutic purposes.

LBS08.05 = OWP3.07

Unravelling the distribution of extracellular vesicles *in vivo* using recombinant tetraspanins

Stefan Vogt ^1^; Madhusudhan Reddy Bobbili^1^; Carolina Patrioli^1^; Samir Barbaria^1^; Markus Schosserer^2^; Lucia Terlecki-Zaniewicz^2^; Elsa Arcalis^3^; Dietmar Pum^3^; Severin Muehleder^4^; Wolfgang Holnthoner^4^; Christopher Kremslehner^5^; Florian Gruber^6^; Johannes Grillari^2^

^1^Department of Biotechnology, University of Natural Resources and Life Sciences, Vienna, Austria., Vienna, Austria; ^2^CDL for Biotechnology of Skin Aging BOKU – Department of Biotechnology, Vienna, Austria; ^3^Department of Applied Genetics and Cell Biology, University of Natural Resources and Life Sciences, Vienna, Austria., Vienna, Austria; ^4^Ludwig Boltzmann Institute for Experimental and Clinical Traumatology, AUVA Research Centre, Endothelial Cell Croup, Vienna, Austria., Vienna, Austria; ^5^Department of Dermatology, Medical University of Vienna, Austria; Christian Doppler Laboratory for Biotechnology of Skin Aging, Austria., Vienna, Austria; ^6^CDL for Biotechnology of Skin Aging Medical University of Vienna, Vienna, Austria

**Background**: Extracellular vesicles (EVs) which were considered as garbage bags of cells came into view only a decade ago and are now increasingly recognized for their importance in cell-to-cell communication. It is their apparent natural ability to transfer cargo from donor cell to recipient cell thereby conferring messages in paracrine or endocrine manner. Over a decade, lot of research has been done to understand the omics, mode of secretion and uptake mechanisms. However, especially the trafficking of EVs *in vivo* is still poorly understood.

**Methods**: We here generated the tetraspanins CD63 and CD81 C-terminally fused to a snorkel tag that adds an additional transmembrane domain to the four existing ones to be able to attach further tags facing the extracellular space. Due to their extravesicular orientation, these tags can be used as a future tool to understand trafficking of EVs *in vivo*. As a first step we aimed to give proof of principle that our constructs allow to track and isolate functional recombinant EVs from cultured cells. We therefore established a method to isolate functional EVs carrying our recombinant tetraspanins using a combination of anti-haemagglutinin affinity matrix and Precission protease cleavage to isolate EVs without damaging the EV membrane and without losing the CLIP and FLAG tags which are preceding to Precission protease site and HA tag.

**Results**: Indeed, we were able to purify the EVs by this strategy. To further proof that these EVs are able to transfer intact and active cargo to recipient cells, we additionally loaded the EVs with Cre recombinase mRNA. Therefore, we stably expressed recombinant tetraspanins and Cre recombinase in donor HeLa cells and fluorescent colour switch LoxP system in recipient HEK293 cells. Indeed, snorkel tagged EVs were taken up in this experiment. Using an *in vivo* mimicking 3D cell culture model, we also observed a crosstalk from human dermal fibroblasts to keratinocytes with snorkel tag containing EVs.

**Summary/Conclusion**: Finally, we are currently testing if snorkel tag containing EVs from the stable HeLa cell line introduced into a xenograft mouse model can be isolated from plasma and tissues to understand the distribution of tumour derived EVs in different tissues. We therefore pave the ground for using snorkel-tagged EVs as a valuable tool to understand EV trafficking *in vivo*.

LBS08.06 = OWP3.06

Role of calcium signalling in the biogenesis of different types of extracellular vesicles derived from the same cell

Ákos Lőrincz ^1^; Balázs Bartos^1^; Dávid Szombath^1^; Dániel Veres^1^; Ágnes Kittel^2^; Erzsébet Ligeti^1^

^1^Department of Physiology, Semmelweis University, Budapest, Hungary; ^2^Institute of Experimental Medicine, Hungarian Academy of Sciences, Budapest, Hungary

**Background**: It has been reported for several cell types that initiation of a sharp calcium signal by application of artificial means such as calcium ionophores induces generation of extracellular vesicles (EVs). However, the role and requirement of calcium signals triggered by natural stimuli in production of different types of EVs released from the same cell is largely unknown.

**Methods**: Medium-sized EVs were obtained in two centrifugation and filtration steps from neutrophils (PMN) isolated from human peripheral blood or murine bone marrow. Murine PMN-EVs were characterized in detail using dynamic light scattering and electron microscopy. EVs were quantitated by flow cytometry and protein measurements.

**Results**: EV production from human neutrophilic granulocytes occurring spontaneously (sEV) and upon stimulation with opsonized particles (aEV) was compared in the absence and presence of extracellular calcium. Generation of aEV was seriously impaired by calcium deficiency whereas release of sEV was not affected. These results were supported in similar experiments carried out on neutrophils isolated from murine bone marrow. Murine neutrophils deficient in phospholipase γ2, the key enzyme for intracellular calcium signalling, were also impeded in release of aEVs whereas sEV production proceeded undisturbed.

**Summary/Conclusion**: Requirement for extracellular calcium supply and intracellular calcium signalling strongly diverges in generation of different types of EVs from the same cell. These findings supply molecular data on the existence of distinguishable cellular pathways of EV production.

**Funding**: This study was funded by NKFIH K119236, Hungary.

LBS08.07 = OWP1.09

Catching the Hedgehog: unravelling Hedgehog secretion during filopodia-mediated transport

Gustavo Aguilar ^1^; Markus Affolter^2^; Isabel Guerrero^3^

^1^Biozentrum, University of Basel, Madrid, Spain; ^2^Biozentrum, University of Basel, Basel, Switzerland; ^3^Centro de Biología Molecular Severo Ochoa (CSIC-UAM), Madrid, Spain

**Background**: During embryonic development cells acquire different fates, proliferate and die in a tightly controlled manner. To orchestrate these processes, cell-to-cell communication occurs via signalling molecules that instruct cell behaviour at a distance. Among these secreted molecules, signalling by morphogens is thought to be able to subdivide a developing tissue in a concentration-dependent fashion. Therefore, the dispersal of morphogens is a key event in the formation of the concentration gradients during “patterning” processes. The lipid-modified Hedgehog (Hh) is one of these morphogens; proposed to disperse via exovesicles presented by filopodia-like structures (called signalling filopodia or cytonemes) that protrude from producing towards receiving cells. The receiving cells also extend filopodia towards presenting cells, exposing the receptor to the Hh morphogen.

**Methods**: We have analysed the mechanisms for receptor and ligand exchange and also the trafficking machinery implicated. To do so, we are implementing new contact-dependent exocytosis sensors to visualize ligand and receptor secretion. We have also developed synthetic binders to membrane-trap these molecules upon presentation for reception. We are combining these tools to elucidate the basis for morphogen transport and contact-dependent cell signalling using the *in vivo* model of *Drosophila* epithelial morphogenesis.

**Results**: Our results support the model of basolateral long distance presentation of the membrane anchored Hh by signalling filopodia in a polarized epithelium, in opposition to the apical diffusion model. We also suggest that these filopodia are the active sites for receptor presentation and ligand exchange.

**Summary/Conclusion**: The use of novel tools in a multicellular organism provides a unique information to resolve the cellular basis of paracrine signalling events during tissue patterning. Our data support a model of filopodia mediated cell–cell signalling, discarding previous models of free diffusion of morphogens during epithelial development.

LBS08.09

Biodistribution, safety and toxicity profile of engineered extracellular vesicles

Elisa Lázaro-Ibáñez ^1^; Amer Saleh^2^; Maelle Mairesse^2^; Jonathan Rose^3^; Jayne Harris^2^; Neil Henderson^4^; Olga Shatnyeva^1^; Xabier Osteikoetxea^5^; Nikki Heath^5^; Ross Overman^5^; Nicholas Edmunds^2^; Niek Dekker^1^

^1^Discovery Biology, Discovery Sciences, IMED Biotech Unit, AstraZeneca, Gothenburg, Sweden, Mölndal, Sweden; ^2^AstraZeneca R&D, Innovative Medicines, Drug Safety & Metabolism, Cambridge, United Kingdom; ^3^AstraZeneca R&D, Innovative Medicines, Laboratory Animal Science, Cambridge, United Kingdom; ^4^AstraZeneca R&D, Innovative Medicines, Biomarkers & Bioanalysis, Mölndal, Sweden; ^5^Discovery Biology, Discovery Sciences, IMED Biotech Unit, AstraZeneca, Alderley Park, United Kingdom, Macclesfield, United Kingdom

**Background**: The potential use of extracellular vesicles (EVs) as therapeutic carriers has attracted much interest with positive results in preclinical studies. Future development of EVs as delivery vectors requires in depth understanding of their general toxicity and biodistribution following *in vivo* administration, particularly if EVs are derived from a xenogeneic source. Using human embryonic kidney cells EVs, we evaluated the general toxicity and compared different tracking methods to understand *in vivo* biodistribution of EVs in mice.

**Methods**: EVs were generated from human wild type or transiently transfected Expi293F engineered cells to express reporter proteins, and isolated by differential centrifugation at 100K after removal of cell debris and larger EVs. Next, EVs were characterized by Western blotting, nanoparticle tracking analysis, transmission electron microscopy and fluorescent microscopy. To study EV-safety and toxicity, BALB/c mice were dosed with EVs by single intravenous (i.v.) injection, blood was collected to evaluate cytokine levels and haematology, and tissues were examined for histopathological changes. For biodistribution studies, red fluorescent protein and DiR-labelled EVs, or luminescent NanoLuc-labelled EVs were i.v. injected in mice, and the tissue distribution and pharmacokinetics of EVs were evaluated using an *in vivo* imaging system (IVIS).

**Results**: Administration of EVs in mice did not induce any significant toxicity with no gross or histopathological effects in the examined tissues 24 h after EV dosing. Moreover, there was no evidence of marked inflammatory cytokine induction. *In vivo* imaging showed that DiR-EV fluorescence signal was mainly detected in the liver and spleen with a relatively long retention time in the body (24 h), while red fluorescent protein-EVs produced a very weak signal mainly associated with the liver and spleen. On the other hand, luminescence signal derived from NanoLuc-labelled EVs was detected primarily in the lung with short retention time (1 h).

**Summary/Conclusion**: This study shows that Expi293F-derived EVs do not induce significant toxicity or immunogenicity following single i.v. injection. These results also demonstrate that the use of engineered fluorescent/luminescent EVs is highly suitable to assess the *in vivo* EV biodistribution.

LBS09: Late Breaking Poster Session – Cancer II Chairs: Valentina Minciacchi; Javier SotilloLocation: Exhibit Hall 17:15–18:30

LBS09.01

Bystander effect of exosomes derived from cervical adenocarcinoma cells in response to irradiation

Sachiko Inubushi ^1^; Yoshiko Fujita^1^; Ryohei Sasaki^2^

^1^Kobe Unibersity Guraduate School of Medicine, Kobe, Japan; ^2^Kobe University Hospital, Kobe, Japan

**Background**: Cervical cancer is the second leading cause of cancer deaths among female cancer worldwide. In recent years, cervical cancer is the most prevalent cancer in women in their late 20s to 30s in Japan. We focused on cervical adenocarcinoma which is one of cervical cancers. Cervical adenocarcinoma is reported to be poor prognosis because of difficulty of early detection and of resistance to standard to radiotherapy or chemotherapy. In this study, we investigated the role of extracellular vesicles (EVs) secreted from cervical adenocarcinoma cells in response to irradiation.

**Methods**: Human cervical cancer (HCA-1) cells were cultured by MEM medium. For the preparation of conditioned media, the culture media was replaced with fresh media supplemented with 10% FBS (depleted of bovine EVs) immediately before treatment with irradiation. Irradiated EVs (IR-EVs) isolation was conducted from the conditioned medium collected 48 h after irradiation (5 Gy). HCA-1 cells were treated with the IR-EVs, to assess the cell viability using WST-1 assay. EVs labelled with PKH26 uptake by HCA-1 cells were analysed by fluorescence microscopy.

**Results**: HCA-1 cells derived EVs were characterized by the presence of EV marker proteins such as CD9 and CD63. The recipient HCA-1 cells exhibited higher uptake efficiency of the exosomes from the IR (5 Gy)-EVs than the IR (0 Gy)-EVs. We revealed that IR-EVs (5 Gy) reduce cell viability for HCA-1 cells.

**Summary/Conclusion**: Our data indicated that the bystander effect of exosomes derived from cells in response to irradiation might be existed. Now, we are also investigating characteristics of miRNAs encapsulated in exosomes involved in cell survival.

**Funding**: This study was partly supported by Society for Women’s Health Science Research of Japan.

LBS09.02

Metabolomic profiling of exosomes isolated from serum of head and neck cancer patients after radiotherapy – a preliminary study

Agata Włosowicz ^1^; Lukasz Marczak^2^; Tomasz Rutkowski^1^; Piotr Widlak^1^; Monika Pietrowska^1^

^1^Maria Sklodowska-Curie Institute – Oncology Center, Gliwice Branch, Gliwice, Poland; ^2^Institute of Bioorganic Chemistry, Polish Academy of Sciences, Poznan, Poland

**Background**: Growing interest in exosomes has resulted in numerous “omics” approaches in search of distinctive molecules. Proteome and transcriptome of exosomes have been widely described and discussed; however, metabolites carried by these vesicles have not been given as much attention. Metabolome analysis of cancer-derived exosomes could provide valuable insight into metabolism changes of cancer cells and tumour microenvironment.

**Methods**: Exosomes were isolated from serum of healthy volunteers and patients with squamous cell carcinoma. Serum samples from patients were taken before (A) and after (B) radiotherapy. Exosomes were isolated using size exclusion chromatography. A mixture of MeOH/H_2_O was used for extraction of low-molecular metabolites. Next, samples were analysed by gas chromatography-mass spectrometry (GC-MS).

**Results**: Comparison of metabolomic profiles of exosomes taken from patients and healthy volunteers revealed metabolite classes common for both groups. The most abundant metabolites were sugars and carboxylic acid derivatives. In addition, amino acids, alcohols, nucleic acids components, amines, carbohydrates and steroid derivatives were found. Nevertheless, most of compounds identified in exosomes were also found in serum itself which was analysed as a control (background). However, changes in the level of several compounds were observed in quantitative assay of exosome metabolome before and after radiotherapy.

**Summary/Conclusion**: Quantitative and qualitative changes in exosomal metabolome were found after radiotherapy. However, it is worth noting that determination of specific exosomal metabolites is difficult due to overlapping effect of contaminating metabolites of non-exosomal origin present in serum.

**Funding**: This work was supported by National Science Centre, Poland, Grant no. 2013/11/B/NZ7/01512 and no. 2016/22/M/NZ5/00667.

LBS09.03

Analysing factors resulting in the elevated EV release of pancreatic cancer cells

Gyongyver Orsolya Sandor ^1^; Andras Aron Soos^1^; Lili Szabo^2^; Edit Buzas^1^; Aniko Zeold; Zoltan Wiener

Semmelweis University, Department of Genetics, Cell and Immunobiology, Budapest, Budapest, Hungary

**Background**: Pancreatic ductal adenocarcinoma has a low survival rate due to the late diagnosis and not well-known disease mechanism. The EV-based early detection of pancreatic cancer is based on the idea that cancer cell-derived EVs carry a characteristic molecular pattern and they release EVs at a highly elevated level. Despite intensive efforts, factors increasing EV production from pancreatic cancer cells are not well known yet. By using bioinformatical and experimental approaches, we asked whether gene expression profiles can predict the increased EV release.

**Methods**: Genes influencing EV production were manually selected. Publicly available gene expression sets were analysed by Gene Set Enrichment Analysis (GSEA). EVs derived from cell lines representing normal pancreatic duct and primary pancreatic ductal adenocarcinoma were studied by antibody-coated magnetic beads and flow cytometry.

**Results**: KRas or p53 mutations did not result in either the positive or negative enrichment of genes responsible for EV production. Similarly, we found no enrichment of these genes when comparing samples from normal pancreas and pancreatic cancer or pancreatic cancers with different cancer-promoting niche types. Interestingly, pancreatic cancer cell lines produced EVs at a highly elevated level compared to normal pancreatic cells in both 2D and 3D cultures. All four cancer cell lines carried the same mutation in KRas, however, we observed a large heterogeneity in their CD81+ and CD63+ EV production.

**Summary/Conclusion**: Collectively, our data suggest that the increased EV release of pancreatic ductal adenocarcinoma cells cannot be explained by the general upregulation of genes influencing EV production.

**Funding**: This work was supported by the János Bolyai Fellowship (A.Z. and Z.W., Hungarian Academy of Sciences; BO/01012/16/8 and BO/00068/15/8), by the National Competitiveness and Excellence Program Hungary (NVKP_16-1-2016-0007) and by the National Research, Development and Innovation Office (Hungary).

LBS09.04

Exosomes derived from hypoxic glioma cells deliver miR-25 to normoxic cells to elicit chemoresistance

Jiwei Wang ^1^; Taral Lunavat^2^; Jian Wang^2^; Rolf Bjerkvig^2^; Frits Thorsen^2^; Xingang Li^3^

^1^University of Bergen, Bergen, Norway; ^2^Department of Biomedicine, University of Bergen, Bergen, Norway; ^3^Department of Neurosurgery, Qilu hospital, Shandong University, Jinan, China (People’s Republic)

**Background**: Hypoxia is one of the crucial microenvironments to promote chemotherapy resistance in glioblastoma multiforme (GBM). Exosomes, initially considered to be cellular “garbage dumpsters”, are now implicated in mediating interactions with cellular environment. However, mechanisms underlying the association between exosomes and hypoxia-induced chemoresistance during cancer progression remain poorly understood.

**Methods**: Human glioma cells pretreated with exosomes derived from normoxic or hypoxic glioma cells were assessed for their proliferation, invasion and apoptosis. miRNA array analysis was performed and the roles of miR-25-3p in proliferation and apoptosis were validated both *in vivo* and *in vitro*. The downstream target of miR-25-3p was determined using a dual luciferase reporter assay.

**Results**: In this study, we found that exosomes derived from hypoxic glioma cells increased the proliferation and invasion of normoxic GBM cells by enhancing the glycolytic capacity. These alterations endowed normoxic GBM chemoresistance by activating PI3K-AKT signalling pathway. Given that exosomes have been shown to transport miRNAs to alter cellular functions, we performed miRNA sequencing of normoxic and hypoxic GBM-derived exosomes. Of the 302 miRNAs that were differentially expressed, miR-25 stood out as one of the most significantly upregulated miRNAs under hypoxic conditions. miR-25 depletion in hypoxic GBM cells led to decreased miR-25 levels in exosomes and significantly reduced hypoxic exosomes induced chemoresistance. Finally, we found that miR-25-mediated regulation of cellular chemoresistance involved downregulated expression of the target protein, PHLPP2 that is crucial for activating PI3K-AKT signalling pathway.

**Summary/Conclusion**: In conclusion, our findings suggest that hypoxic microenvironment may stimulate glioma cells to generate miR-25-rich exosomes that are delivered to normoxic glioma cells to promote chemoresistance.

LBS09.05

Membrane metalloproteinases regulation by insertion into microdomains and extracellular vesicles

Henar Suárez Montero ^1^; Soraya López-Martín^2^; Kate E. Hebron^3^; Andries Zijlstra^4^; María Yáñez-Mó^5^

^1^Molecular Biology Center Severo Ochoa (CBM), Madrid, Spain., Madrid, Spain; ^2^Molecular Biology Center Severo Ochoa (CBM), Instituto de Investigación Sanitaria Princesa (IIS-IP), Madrid, Spain, Madrid, Spain; ^3^Vanderbilt University, NASHVILLE, USA; ^4^Department of Pathology, Microbiology and Immunology, Vanderbilt University Medical Center, Nashville, TN, USA, Nashville, USA; ^5^Departamento de Biología Molecular. UAM, Madrid, Spain

**Background**: Membrane-bound proteases degrade matrix proteins or shed adhesion receptors. Recent reports suggest that TEM, supramolecular complexes organized by tetraspanin membrane proteins, regulate proteolytic events by the association of tetraspanins with proteases from the ADAMs, MT-MMP and γ-secretase families. MT1-MMP metalloproteinase is critical for matrix degradation during cancer invasion, angiogenesis and development. Invasive cells form specialized actin rich protrusions called invadopodia where MT1-MMP is translocated. Recently, it has been demonstrated that these areas can actively secrete extracellular vesicles (EVs) enriched in MT1-MMP.

MT1-MMP has a cytoplasmic domain with positively charged amino acids, which makes it a good candidate to stablish interactions with ERM. ERM link transmembrane proteins with the cytoskeleton, and they are abundant in EVs. MT1-MMP is inserted into TEMs via its association with tetraspanin CD151; and TEMs have also been involved in protein sorting into EVs.

**Methods**: To study the relation between MT1-MMP, ERM proteins and tetraspanins we have generated different mutations of MT1-MMP cytoplasmic domain and deleted the gene of CD151 by CRIPR/cas9 technology.

**Results**: Our data suggest that the juxtamembrane positive cluster is responsible for MT1-MMP/ERM interaction and regulates MT1-MMP activity, auto-processing and stability at the plasma membrane. The modifications in the cytoplasmic region of MT1-MMP do not impair its association with the tetraspanin CD151, but impaired accumulation and coalescence of CD151/MT1-MMP complexes at actin rich structures. Regarding EVs, blocking the interaction of MT1-MMP and ERMs show little effect on the sorting of MT1-MMP into EVs, while deleting CD151 gene revealed a role for tetraspanin in the rate of EVs secretion and in the incorporation of MT1-MMP to extracellular vesicles.

**Summary/Conclusion**: MT1-MMP proper distribution and processing on the cell surface is controlled by ERM through their interaction with the metalloproteinase cytoplasmic domain. Besides, tetraspanin CD151 controls MT1MMP inclusion in EVs and the rate of secretion of these vesicles in cancer cells.

**Funding**: This work was supported by grants from Fundación Ramón Areces and BFU2014-55478-R and REDIEX SAF2015-71231-REDT from Ministerio de Economía y Competitividad.

LBS09.06

Oncogenes systematically reprogramme the exosome biogenesis pathway to promote tumourigenesis

Haifeng Zhang ^1^; Gian Luca Negri^2^; Tianqing Yang^2^; Christopher Hughes^2^; Shane Colborne^3^; Gregg Morin^3^; Poul Sorensen^2^

^1^The University of British Columbia, Vancouver, Canada; ^2^University of British Columbia, Vancouver, Canada; ^3^Canada’s Michael Smith Genome Sciences Centre, Vancouver, Canada

**Background**: Tumour cells have unique survival capacities in the GILA (growth in low attachment) condition, a stressful 3D culture condition in which non-tumour cells undergo apoptosis. However, the underlying molecular mechanism remains elusive. Since acute changes in mRNA translation represent a major component of stress adaptation, we hypothesized that tumour cells adapt to the GILA adversity via altering the translatome or the acute synthesis of specific proteins.

**Methods**: Using Click technology coupled with pulsed-SILAC and mass spectrometry (Click-pSILAC), the global nascent protein translation profiles were compared between non-transformed and oncogene-transformed cells in the GILA condition. Moreover, the impact of acute translatome rewiring on the global proteome was also evaluated by Tandem Mass Tag labelling. The impact of Myo1b depletion on the global secretome of tumour cells was determined using the Click-pSILAC approach. Ultracentrifugation was used for exosome isolation, and NanoSight tracking analysis and electron microscopy were performed to quantify and visualize exosomes.

**Results**: These systematic analyses uncovered that numerous key components of the membrane trafficking and exosome biogenesis pathway are upregulated in oncogene-transformed cells, such as Rabgef1, Sec23b, Myo1b and multiple Rab family proteins. In support of the role of this pathway in exosome biogenesis, transformed cells acquired increased exosome formation capacity compared with non-transformed cells. Importantly, exosomes derived from transformed cells could confer non-transformed cells with acquired adaptability in the GILA condition. Moreover, blocking Rab27b, one of the key regulators of exosome formation, diminished the stress adaptability and tumourigenicity of transformed cells. Furthermore, global secretome analyses uncovered that Myo1b depletion in tumour cells markedly decreased the abundance of exosome markers in the secretome, which has enabled us to identify Myo1b as a novel regulator of exosome biogenesis.

**Summary/Conclusion**: Collectively, our findings suggest that oncogenes systematically reprogramme the membrane trafficking and exosome biogenesis pathway to promote stress adaptation and drive tumourigenicity in tumour cells.

**Funding**: This study is funded by the Stand Up To Cancer (SU2C) grant awarded to Dr. Poul Sorensen, MD, PhD.

LBS09.07

Effects of extracellular vesicles deriving from BRAF inhibitor resistant melanoma cells on immune cells: role of immune checkpoint expression

Eriomina Shahaj ^1^; Elisabetta Vergani^1^; Viviana Vallacchi^1^; Licia Rivoltini^1^; Monica Rodolfo^1^; Veronica Huber^2^

^1^Fondazione IRCCS Istituto Nazionale dei Tumori, Milan, Italy; ^2^Unit of Immunotherapy of Human Tumors, Fondazione IRCCS Istituto Nazionale dei Tumori, Milan, Italy

**Background**: Melanoma extracellular vesicles (EVs) are endowed with protumourigenic features and can condition the immune system favouring immune escape. Immune checkpoints (IC) are key molecules involved in the regulation of immune responses and can be expressed also by tumour cells. We investigated the expression of IC in melanoma cells with acquired resistance to BRAF kinase inhibitor and in cognate EVs and their modulation in EV-interacting immune and tumour cells.

**Methods**: EVs were isolated by differential ultracentrifugation. IC expression was evaluated in sensitive/resistant cell line pairs and EVs by qRT-PCR, Western blot and flow cytometry. The IC modulation in target cells via EVs was studied with melanoma cells transfected with CD81GFP fusion protein. Interaction and IC modulation were assessed by flow cytometry of and CD81-GFP exosome/target cell co-cultures.

**Results**: The IC PDL1, PDL2, HVEM, Galectin9, CD155 and VISTA were expressed in melanoma cells with higher levels of PDL1, PDL2 and CD155 in the resistant variants. EVs reflected the IC expression of originating cells and contained IC transcripts. CD81GFP positive exosomes interacted with melanoma cells, both BRAF inhibitor resistant and sensitive. In PBMC co-cultures they preferentially targeted monocytes, inducing the up-regulation of PDL1 and Galectin9, and impaired T cell proliferation.

**Summary/Conclusion**: IC expression by melanoma cells can be influenced by BRAF inhibitor treatment. EVs reflect IC expression of originating cells and may represent a surrogate of melanoma resistance status. The activity of IC-carrying EVs on interacting cells suggests their involvement in immunomodulation and immune escape.

**Funding**: This study was funded by 12162 AIRC 5X1000 Rivoltini and 2015-0911 Cariplo Vallacchi.

LBS09.08

Induction of structural and functional effects of myeloma cells after Daratumumab treatment

Yuliya Yakymiv ^1^; Angelo Corso Faini^1^; Barbara Castella^2^; Alberto L. Horenstein^2^; Cristiano Bracci^2^; Fabio Morandi^3^; Alessandra Larocca^4^; Stefania Oliva^4^; Massimo Massaia^5^; Mario Boccadoro^4^; Fabio Malavasi^3^

^1^Laboratory of Immunogenetics, Department of Medical Sciences, Torino, Italy, Torino, Italy; ^2^Laboratory of Immunogenetics, Department of Medical Sciences, and CeRMS, University of Torino, Italy, Torino, Italy; ^3^Stem Cell Laboratory and Cell Therapy Center, Istituto Giannina Gaslini, Genova, Italy, Torino, Italy; ^4^AOU Città della Salute e della Scienza di Torino, Italy, Torino, Italy; ^5^SC Ematologia AO S. Croce Carle, Cuneo, Italy, Torino, Italy

**Background**: A pleiotropic cell surface glycoprotein with receptorial and enzymatic functions, CD38 is prevalently expressed by haematological tissues, where it serves as a target for therapeutic antibodies in multiple myeloma (MM). Daratumumab (Dara) is approved as monotherapy or in combination with other anti-MM agents and yields good results. Dara is currently being appraised for its immunomodulatory potential.

**Methods**: Microvesicles (MV) were isolated from the culture supernatant through differential centrifugation steps. The phenotype of MV was analysed by flow cytometry, using appropriate conjugated mAbs. MV internalization was evaluated by confocal microscopy. NK proliferation, viability and cytotoxicity after MV exposure were assessed by flow cytometry using conventional assays (CFSE, Annexin V/PI). Analyses of gene modulation were performed with NGS.

**Results**: CD38 engagement at 37°C by Dara on MM cells is followed by polar aggregation of the target molecule in the membranes, with release of MV measuring 100–1000 nm in diameter. MV obtained after Dara treatment may be internalized by NK cells, myeloid-derived suppressor cells and monocytes, all FcR+. NK which are significantly reduced *in vivo* during Dara treatment, were selected for testing functional MV-mediated effects. Analysis of the genes modulated in NK cells exposed to the MV/Dara complex was followed by functional *in vitro* experiments. In both conditions, the results confirmed reduced proliferative ability and enhanced killing of MM cells mediated by NK cells.

Further evidence of Dara’s immunomodulatory effects is that, when located on the MV surface, the CD38/Dara complex is surrounded by a set of ectoenzymes (CD38, CD39, CD73 and CD203a) involved in the generation of ADO. Furthermore, the MV phenotype was integrated by the presence of CD55 and CD59, complement inhibitory receptors. The picture is completed by the finding that PD-L1 accumulates in the same raft that harbours CD38. A reasonable inference is that the Dara-driven MV may play a role in the modulation of immune checkpoint pathways.

**Summary/Conclusion**: The present work establishes that membrane domains containing CD38 in MM patients treated with Dara may interfere with a particulate signalling communication system adopted by the neoplasia to reshape the environment and escape defence mechanisms.

**Funding**: This study was funded by Janssen Pharmaceutica.

Scientific Program ISEV2018 Sunday, 06 May 2018 Symposium Session 28 – Late Breaking Abstracts Chair: Dolores DiVizio Location: Auditorium 09:00–10:00

LBO1.01

Blood microvesicles derived from neurovascular network correlate with amyloid-β deposition in the brains of postmenopausal women

Muthuvel Jayachandran; Brian Lahr; Kent Bailey; Val Lowe; Kejal Kantarci; Virginia Miller

Mayo Clinic Rochester, Rochester, USA

**Background**: The deposition of neurotoxic aggregates of amyloid-β (Aβ) fibrils in the cells of brain is a key feature of Alzheimer’s disease (AD). Experimental studies demonstrated the role of extracellular vesicles in Aβ deposition in the brain. This study aims to characterize blood microvesicles (MV) from activated cells of neurovascular network and to determine whether blood levels of MV from the cells of neurovascular network associate with Aβ deposition in the brains of postmenopausal women.

**Methods**: This study was approved by Mayo Clinic IRB. Venous blood was collected from postmenopausal women (*n* = 67; median age 60) who participated in the brain imaging study of Mayo Clinic Specialized Center of Research on Sex differences. The blood MV positive for the markers of blood–brain barrier (BBB)–endothelium (low density lipoprotein receptor-related receptor), astrocytes (GFAP, glial fibrillar acidic protein), microglia (Iba1, ionized calcium adaptor molecule 1), neuron (Tuj-1, neuron specific class III beta-tubulin) and AD (Tau or amyloid β1–42) were quantified by digital flow cytometer. Aβ accumulation (PiB standard unit value ratio) in the brain was measured by 11C Pittsburgh compound B (PiB) PET imaging. Two principal components (PC) based on the six markers were analysed for joint association with Aβ using multivariable ordinal logistic regression (MVOLR). Also, each MV variable was analysed for an association with Aβ using Spearman correlation.

**Results**: Number of MV positive for BBB-endothelium, astrocytes, neurons and Aβ1–42 markers (range of median values: 3.4–5.5/µL plasma) tended to be higher than MV positive for microglia and Tau markers (0.16–0.42/µL plasma). In MVOLR, PC 1 (contrast between MV-lab-1 and MV-GFAP/MV-Tuj1) had a significant association (*p* = 0.033) with Aβ. A significant Spearman correlation of *ρ* = 0.27 (*p* = 0.026) was found only between Aβ deposition and astrocyte-derived MV.

**Summary/Conclusion**: MV derived from cells of the neurovascular network can be detected in peripheral blood and seem to be associated jointly with Aβ. In analyses of each biomarker, only astrocyte-derived MV was associated with deposition of Aβ in the brain of postmenopausal women. Confirmation of this result in larger cohort may help to identify deposition of Aβ in the brain by blood test.

**Funding**: This study was funded by NIH AG44170 and NIH AG16574.

LB01.02

Graphene-oxide quenching-based molecular beacon imaging for exosome-mediated transfer of neurogenic miRNA on microfluidic platform

Hyun Jeong Oh ^1^; Hyejin Park^2^; Dayoung Yoon^3^; Seok Chung^2^; Do Won Hwang^4^; Dong Soo Lee^4^

^1^Division of Mechanical Engineering, Korea University, Seoul, Republic of Korea; ^2^School of Mechanical Engineering, Korea University, Seoul, Korea, Seoul, Republic of Korea; ^3^KU-KIST Graduate School of Converging Science and Technology, Korea University, Seoul, Korea, Seoul, Republic of Korea; ^4^Department of Nuclear Medicine, Seoul National University College of Medicine, Seoul, Republic of Korea

**Background**: Exosomes are cell-derived vesicles that shuttle biological components such as miRNAs related to regulation of cell proliferation and differentiation. Neurogenic microRNA (miRNA) such as miR-124 or miR-9 plays essential roles in neurogenesis of neural stem cells (NSCs) and neural progenitor cells (NPs) and can be transferred via exosomes. Graphene-oxide (GO) quenching-based molecular beacon was developed to detect miRNAs in living cells quickly and sensitively. In this study, we applied GO quencher-based molecular beacon sensor to visualize neurogenic miR-193a levels delivered via exosome during cell-non-autonomous neurogenesis of neural progenitor cells on microfluidic platform.

**Methods**: Exosomal transport was visualized using the RFP-tagged CD63 plasmid vector and RFP-tagged exosomes were monitored by confocal microscopy on microfluidic device. F11, neural progenitor cells were used to examine exosomes. FAM-labelled peptide nucleic acid (PNA) 193a probe had the complementary sequence to miR-193a. Fluorescence signal of FAM-PNA193a was quenched by GO nanosheet attachment. FAM-PNA193a-GO complex was used to visualize miR-193a levels during exosome-mediated neurogenesis in F11 cells.

**Results**: The miR-193a induced neurogenesis in neural progenitor cells by regulating proliferation-related target genes. The miR-193a was highly expressed in exosomes secreted from differentiated cells after neurogenesis. Fluorescence of FAM-PNA193a was completely quenched by GO and FAM-PNA193a-GO complex recovered fluorescence by miR-193a but not by miR-scr. Exosomes containing miR-193a from differentiated (D)-donor cells were delivered to undifferentiated (UD)-recipient cells and lead them to neurogenesis.

Fluorescence signals of FAM-PNA193a-GO were recovered in UD-recipient F11 cells differentiated to the neuronal lineage by exosome-mediated neurogenesis 3 days after co-culture with D-donor F11 cells.

**Summary/Conclusion**: In this study, we propose that exosome-tracing microfluidic platform and molecular beacon imaging using fluorescent dye-PNA (against a specific miRNA)-GO complex can be applied to visualize exosomes and individual cellular expression of mature microRNAs revealing their precise spatial localization by the intercellular exosome delivery to undergo processes such as cell-non-autonomous neurogenesis.

LB01.03

Menadione driven oxidative stress related microvesicle shedding associates with resilience to hypoxia in a neuronal ischaemia model

Emma Buzzard ^1^; Michelle Potter^1^; Wei Zhang^1^; Manjot Gill^1^; Dong Hyeok Park^2^; Manu Vatish^3^; Jeung Sang Go^2^; Rebecca Dragovic^1^; Karl J. Morten^1^

^1^Nuffield Department of Womens and Reproductive Health, University of Oxford, Oxford, United Kingdom; ^2^Pusan National University South Korea, Busan, Republic of Korea; ^3^Nuffield Department of Obstetrics & Gynaecology, University of Oxford, John Radcliffe Hospital, Oxford, OX3 9DU, United Kingdom, Oxford, United Kingdom

**Background**: Extracellular vesicle (EV)-mediated transfer of cargo is a key element of cell–cell communication that can be beneficial (e.g. restorative properties of stem cell EVs) or injurious, as represented in pre-eclampsia (caused by placental vesicle shedding) and acute traumatic brain injury (exacerbated by EV release into circulation). Here, a subpopulation of EVs – microvesicles (MVs) – were generated in a model which exposes a GFP expressing human glioblastoma line to the superoxide generating redox cycler Menadione under physiological concentrations of glucose and oxygen.

**Methods**: The cellular effects of menadione under standard tissue culture glucose conditions (25 mM) and physiological levels of 5 mM and 1 mM glucose were assessed using the Clariostar plate reader (BMG Labtech). Experiments were also carried out under normoxia (20%), 8% O_2_ and 1% O_2_.

To correlate MV shedding with superoxide production we used an intracellular O_2_ probe (MitoXpress intra, Luxcel Biosciences) as an indirect indicator of mitochondrial superoxide production. Multiplexing the O_2_ probe and GFP using the atmospheric control unit on the Clariostar we are able to measure vesicle release following menadione treatment and under different O_2_ conditions.

Flow cytometry (LSRII BD) and Nanoparticle tracking analysis (NTA) NS500 were used to quantitate MV levels in the culture media.

**Results**: When the human glioblastoma line U87MG was exposed to menadione a cell killing/proliferation dose–response curve was observed. Menadione’s effects were more significant under low glucose conditions. Under hypoxia menadione was no longer toxic appearing to actually increase cell growth. In addition to causing cell death menadione also increased the GFP signal in the media. NTA confirmed the presence of high levels of MVs. When menadione experiments were carried out at 8% oxygen a significant drop in intracellular oxygen was observed with an associated increased in GFP secretion presumably due to MV release. When switched to 1% O_2_ a significant increase in intracellular O_2_ was observed potentially linking to the increased viability observed in long-term menadione experiments at low O_2_.

**Summary/Conclusion**: Redox cycler generated mitochondrial ROS associates with MV shedding in a neuronal model. Under low O_2_ a neuronal protective effect is observed.

**Funding**: This study was funded by Horizon 2020 grant MetaCELL.

LB01.04

Unravelling the role of extracellular small heat-shock proteins in neuroinflammation

Sam Vanherle^1^; Bram Van den Broek^2^; Vicky De Winter^3^; Revathy Munuswamy^4^; Baharak Hosseinkhani^4^; Vincent Timmerman^3^; Luc Michiels^5^; Joy Irobi ^6^

^1^Neurofunctional genomics Group, Biomedical Research Institute (BIOMED), Hasselt University, Diepenbeek, Belgium, Diepenbeek, Hasselt, Belgium; ^2^Neurofunctional genomics Group, Biomedical Research Institute (BIOMED), Hasselt University, Diepenbeek, Belgium, Hasselt, Belgium; ^3^Peripheral Neuropathy Group, VIB-Department of Molecular Genetics, University of Antwerp, Antwerpen, Belgium, Antwerp, Belgium; ^4^Bionanotechnology group, Biomedical Research Institute (BIOMED), Hasselt University, Martelarenlaan 42, B-3500 Hasselt, Belgium, Hasselt, Belgium; ^5^Hasselt University, Bionanotechnology group, Biomedical research institute (BIOMED), Martelarenlaan 42, 3500 Hasselt, Belgium, Hasselt, Belgium; ^6^Neurofunctional genomics Group, Biomedical Research Institute (BIOMED), Hasselt University, Diepenbeek, Belgium, Diepenbeek, Belgium

**Background**: Multiple sclerosis (MS) is a chronic demyelinating disease of the central nervous system (CNS). During lesion development in MS, there is increased expression of small heat-shock proteins (HSPBs). The protective roles of HSPBs in regulating neural cell survival, inhibiting protein aggregation and regulating inflammation in the CNS is well known. Nonetheless, the extracellular role of HSPBs in neuroinflammation is unclear.

One way that HSPBs are released into the extracellular space is through extracellular vesicles (EV). During neuroinflammation, neural cells (microglia and oligodendrocyte) release EVs either carrying beneficial or detrimental biomarkers into the environment. Here, we characterize the neural cells derived-EV and hypothesize that impaired EV expression can disrupt cell survival communications during neuroinflammation.

**Methods**: The role of HSPB-EV in inflammation is investigated in two steps. (1) Establish HSPBs expressing neural cell lines for the production of HSPB-EV. (2) Isolation and characterization of EV using different techniques.

**Results**: NTA measurement of microglial derived-EV showed an increased EV secretion upon inflammation. Stable HSPBs expressing cell derived-EV revealed a decrease in EV release during inflammation. SPR analysis of oligodendrocyte derived-EV showed interactions with ICAM1 and HSP70. In addition, immunoblot analysis of oligodendrocyte derived-EV showed a downregulation of monomeric HSPB8 and phosphorylated HSPB1 during inflammation.

**Summary/Conclusion**: Neural cells derived-EVs constitutively express HSPB1 and HSPB8. However upon inflammation, there is a downregulation of both the monomeric forms as well as the phosphorylated HSPB1. This study shows that reduced expression in the extracellular HSPB1/B8-EV upon neuroinflammation can impair neural cell survival signalling.

**Funding**: This work was financed by Hasselt University and by EFRO through the Interreg V Grensregio Vlaanderen Nederland project Trans Tech Diagnostics.

Symposium Session 29 – Late Breaking Abstracts Chair: Tang Long Shen Location: Room 5 09:00–10:00

LB02.01

On-chip liquid biopsy: progress in isolation of exosomes and their RNA sequencing for prognosis of prostate cancer

Navneet Dogra ^1^; Gustavo Stolovitzky^2^; Carlos Cordon^3^; Ashutosh Tewari^3^; Kamlesh Yadav^1^; Russell McBride^4^; Eren Ahsen Mehmet^5^; Stacey Gifford^6^; Benjamin Wunsch^7^; Joshua Smith^7^; Sungcheol Kim^7^

^1^Icahn School of Medicine at Mount Sinai, New York City, USA; ^2^IBM/Icahn School of Medicine at Mt. Sinai, New York, USA; ^3^Icahn School of Medicine at Mt. Sinai, New York, USA; ^4^Icahn School of Medicine at Mount Sinai & IBM, NY, USA; ^5^Icahn School of Medicine at Mount Sinai, NY, USA; ^6^IBM, New York, USA; ^7^IBM, NY, USA

**Background**: Exosomes are an exciting target for “liquid biopsies”. However, isolation of exosomes and reproducible detection of their biomarkers remains an ongoing challenge. We have developed a microfluidic nanoscale DLD (deterministic lateral displacement) device that brings capabilities with size based sorting of colloidal particles at the tens of nanometres scale (Wunsch et al., Nat Nanotechnol. 2016).

**Methods**: Using our chip technology, we have isolated exosomes from prostate cancer cell lines and patient tissue, blood and urine samples. After exosome isolation, small RNA libraries were prepared and sequencing is carried out at New York Genome Center using illumine sequencer HISeq2500. Our nanofluidic pillar array is manufactured in SiO_2_ mask using optical contact lithography and deep ultra violet lithography.

**Results**: We demonstrate microfluidic on-chip size-based separation of exosomes. We showed that our microfluidic device is capable of sorting exosomes population from different bodily fluids and cell culture medium. Once exosomes are isolated via the chip, we performed RNA sequencing study for biomarker discovery. These results are important and exciting for the following reasons: (1) we were able to separate smaller particles (below 30 nm) from larger (30–100 nm) vesicle population. (2) Exosomal RNA content from the prostate cancer patient urine and blood samples were compared with prostate tumour RNA to identify molecular transport. (3) Combined with fluorescence microscopy, our technology can sort and identify multiple epitopes simultaneously on single exosomes surface.

**Summary/Conclusion**: These exciting preliminary results indicate the potential of this technology for sorting exosomes and detection of certain disease related biomarkers from plasma, urine, serum or circulating tumour-derived exosomes.

**Funding**: This study was funded by Icahn School of Medicine at Mt. Sinai and IBM.

LB02.02

Tissue-tropism, parasite cargo and signalling of spleen fibroblasts by plasma-derived exosomes from vivax malaria infections

Haruka Toda ^1^; Joan Segui-Barber^1^; Miriam Diaz-Varela^1^; Susana Garcia-Silva^2^; Alicia Galiano^3^; Melisa Gualdrón-López^4^; Barbara Baro^5^; Anne Almeida^6^; Marcelo Brito^6^; Wuelton Monteiro^6^; Marcus V.G. de Lacerda^7^; Maria del Pilar Armengol Barnils^8^; Antonio Marcilla^3^; Héctor Peinado^9^; Carmen Fernandez-Becerra^10^; Hernando A. del Portillo^11^

^1^ISGlobal, Hospital Clínic – Universitat de Barcelona, Barcelona, Spain, Barcelona, Spain; ^2^Microenvironment and Metastasis Group, Molecular Oncology Program, Spanish National Cancer Research Centre (CNIO), Madrid, Spain, Madrid, Spain; ^3^Departament de Farmàcia I Tecnologia Farmacéutica i Parasitologia, Universitat de Valéncia, Spain, BURJASSOT (VALENCIA), Spain; ^4^ISGlobal, Hospital Clínic – Universitat de Barcelona. Institut d’Investigació Germans Trias i Pujol (IGTP), Badalona, Spain, Badalona (barcelone), Spain; ^5^Fundação de Medicina Tropical Dr. Heitor Vieira Dourado (FMT-HVD), Manaus, Amazonas, Brazil., Manaus, Brazil; ^6^Fundação de Medicina Tropical Dr. Heitor Vieira Dourado (FMT-HVD), Manaus, Amazonas, Brazil. Escola Superior de Ciências da Saúde, Universidade do Estado do Amazonas, Manaus, Brazil, Manaus, Brazil; ^7^Fundação de Medicina Tropical Dr. Heitor Vieira Dourado (FMT-HVD), Manaus, Amazonas, Brazil. Instituto de Pesquisas Leônidas & Maria Deane, FIOCRUZ, Manaus, Brazil, Manaus, Brazil; ^8^Institut d’Investigació Germans Trias i Pujol (IGTP), Badalona, Spain, Barcelona, Spain; ^9^Microenvironment and Metastasis Group, Molecular Oncology Programme, Spanish National Cancer Research Centre (CNIO), Madrid, Spain, Madrid, Spain; ^10^ISGlobal, Hospital Clínic – Universitat de Barcelona. Institute for Health Sciences Trias I Pujol (IGTP), Badalona, Spain, Barcelone, Spain; ^11^ISGlobal, Hospital Clínic – Universitat de Barcelona. Institute for Health Sciences Trias I Pujol (IGTP), Badalona, Spain. Catalan Institution for Research and Advanced Studies (ICREA), Barcelona, Spain

**Background**: *In vivo* imaging of the spleen in experimental infections of BALB/c mice with a reticulocyte-prone malaria parasite showed the formation of fibroblast barrier cells where infected reticulocytes actively adhere avoiding macrophage destruction (Martin-Jaular et al., Cell Microbiol. 2011). These studies thus suggested that reticulocyte-prone malarial parasites could remain attached in parasite-induced spleen niches where there is abundance of reticulocytes. As exosomes in malaria act in inter-cellular communication, we hypothesized that they could play a role in this phenomenon in natural infections of *P. vivax*, a reticulocyte-prone human malaria parasite.

**Methods**: Plasma-derived exosomes from vivax patients (PvEX) and healthy donors (hEX) were characterized by NTA and FACS analysis. *In vivo* distribution was determined in C57BL/6 mice. Moreover, PKH67-labelled PvEX and PKH26-labelled hEX were incubated with human splenic fibroblasts (hSF) and uptake activity was observed by confocal microscopy. Phenotypical changes of recipient hSF cells were analysed by qRT-PCR using several specific genes. Last, mass spectrometry of was performed to identify parasite cargo associated with exosomes from infection.

**Results**: NTA analysis of plasma-derived exosomes revealed twice as much exosomes in patients as opposed to healthy donors. Moreover, *in vivo* distribution experiments in mice revealed spleen-specific tropism of exosomes from vivax infections when compared to exosomes from worm infections or controls. Furthermore, a significant higher uptake by of exosomes from infections was observed in human spleen fibroblasts when compared to exosomes from healthy individuals. Noticeably, ICAM-1, a known receptor for binding of *P. vivax* (Bernabeu et al., Cell Microbiol. 2015), was specifically upregulated by exosomes from infections in a dose-dependent manner. Last proteomic analysis of exosomes from infections revealed the presence of parasite proteins associated with them.

**Summary/Conclusion**: These findings suggest that exosomes from natural vivax infections signal human spleen fibroblasts facilitating cytoadherence of the parasite.

**Funding**: This study was funded by Generalitat de Catalunya, Ministerio Español de Economía y Competitividad, REDiEX and Fundación Ramón Areces. HT is recipient of an AGAUR PhD fellowship.

LB02.03

Alterations in platelet membranes and formation of lipid mediators during storage

Sami Valkonen ^1^; Minna Holopainen^2^; Jesmond Dalli^3^; Reijo Käkelä^4^; Pia R-M. Siljander^1^; Saara Laitinen^2^

^1^EV group, Faculty of Bio-and Environmental Sciences and Faculty of Pharmacy, University of Helsinki, Finland, Helsinki, Finland; ^2^Finnish Red Cross Blood Service, Helsinki, Finland, Helsinki, Finland; ^3^William Harvey Research Institute, Barts and the London School of Medicine, Queen Mary University of London, London, United Kingdom, London, United Kingdom; ^4^Division of Physiology and Neuroscience, Department of Biosciences, University of Helsinki,Helsinki, Finland

**Background**: The time-dependent variation in glycerophospholipid (GPL) composition of platelets has been studied previously, potentially explaining the proposed link between the concentrate storage time and incidence of adverse transfusion reactions (ATR). The lipid composition and more specifically the lipid mediator (LM) profile of platelet concentrates is functionally important and may help to understand the mechanisms of ATR. This knowledge could be further exploited to tailor transfusion treatments to different types of patients: as the LM composition modulates immune responses, different patients groups would benefit from platelet concentrates with different LM profiles.

**Methods**: Studied samples were isolated on days 1, 2, 5 and 8 from clinical grade platelet concentrates. The GPL profile of the whole concentrate, platelets and extracellular vesicles (EVs) was analysed with electrospray-ionization mass spectrometry and the content of LMs in the whole product and EVs was determined using liquid chromatography–tandem mass spectrometry. Western blot was used to prove the presence of enzymes producing LMs.

**Results**: The GPL profile of the whole concentrate and platelets differed notably from each other whereas the EVs resembled the whole product. The GPL profile of platelets was more prone to time-dependent alterations than that of the whole product and EVs.

Clear time-dependent differences in the pro-resolving and pro-inflammatory LMs of the whole product and EVs were observed. The enzymes needed for the production of LMs were also present in the concentrate.

**Summary/Conclusion**: During platelet storage time-dependent changes in LM and GPL profiles alter platelet functionality. Further analyses will be needed to enable tailoring of concentrate use to avoid ATR and to optimize patient suitability.

**Funding**: Part of this work was funded by Tekes programs SalWe GID and NanoSkin, the Academy of Finland and Magnus Ehrnrooth Foundation.

LB02.04

Validation of purification methods for extracellular vesicles

Jacopo Zini^1^; Heikki Saari^2^; Marjo Yliperttula^2^; Olli-Petteri Nivaro^1^

^1^University of Helsinki, Helsinki, Finland; ^1^University of Helsinki, Division of Pharmaceutical Biosciences, Helsinki, Finland

**Background**: EVs are involved in cellular communication and they can serve as efficient carriers to deliver chemotherapeutic drugs to tumor cells, but the biodistribution of exogenous EVs is determined by cell source, purification methods and artificial targeting. Anyhow purification and characterization remain challenging for many reasons such as i) lack in standardization methods, ii) high variability of EVs production, moreover the composition of EVs can change based on the time and conservation method The aim of the present study was to find an univocal and reproducible method to obtain pure EVs.

**Methods**: We compared and combined different purification methods such as: ultra-filtration (UF), differential centrifugation (DC), density gradient centrifugation (G), size exclusion chromatography (SEC) and salting out (SO). Then we analyze the resulting suspension focusing mainly in tow aspect: particle size distribution and Protein- Lipid ratio. Pericles size distribution has been analyzed with the NTA and the asymmetrical-flow field-flow fractionation (AF4) coupled to a multi-angle light-scattering detector (MALS) that is considered the golden standard regarding the EVs purifications. Protein- lipids ratio has been studied with a bio-photonic approach, we used the as Infrared and Raman spectroscopy, both are vibrational spectroscopy technique and they are complementary each other’s.

**Results**: The combination of different purification methods (DC + G) allows to get samples with a low concentrations of free proteins and aggregate. The particle size distribution seems not affected by different protocols that means we are able to recovery both small exosomes and large microvesicles after each purification steps. The spectra obtained with Raman and IR spectroscopy shown peak related to different chemical entities as amide and alkyl group. The protein- lipids ratio is determinate by the comparison of the intensity of this 2 peak. Our results underline that the multi steps purification protocols is able to reach a lower P/L that means the samples contains a lower quantity of free proteins.

**Summary/Conclusion**: According to our data the combinations of two different methods, such as UF or DC with the density gradient gentrification leads to a more pure samples that shown ales amount of aggregates, more uniform particle size distribution and a lower protein-lipids ratio.

SS 30 LB: Symposium Session 30 – Late Breaking Abstracts Chair: Lei Zheng Location: Room 6 09:00–10:00

LB03.01

Regulation of exosome secretion via the intricate tuning of multivesicular endosome transport towards the plasma membrane

Maarten P. Bebelman^1^; Frederik Verweij^2^; Roberta Palmulli^3^; Xavier Heiligenstein^4^; Graca Raposo^5^; D. Michiel Pegtel^6^; Guillaume van Niel ^7^

^1^Division of Medicinal Chemistry, Amsterdam Institute for Molecules Medicines and Systems, VU University Amsterdam, de Boelelaan 1108, 1081 HZ Amsterdam, The Netherlands., Amsterdam, The Netherlands; ^2^INSERM U894 Centre de Psychiatrie et Neurosciences, Paris, France; ^3^Institut Curie, PSL Research University, CNRS, UMR144, Paris, France., Paris, France; ^4^Institut Curie, PSL Research University, CNRS, UMR144, Paris, France., paris, France; ^5^Institut Curie, Paris, France; ^6^Exosome Research Group, Dept. Pathology, Cancer Center Amsterdam, VU University Medical Center, de Boelelaan 1118, 1081 HV Amsterdam, The Netherlands; ^7^CNRS, Paris, France

**Background**: Exosomes correspond to intraluminal vesicles of multivesicular endosomes (MVE) that are released after fusion of MVEs with the plasma membrane. Despite the growing interest in exosome functions, especially in disease, the mechanisms responsible for their secretion are far from being fully understood. This knowledge is yet capital as it is the first step that controls this intercellular mode of communication. MVEs are very dynamic endosomal organelles that can be transported by various molecular motors and interact with other intracellular organelles during their maturation process. In this study, we investigated the impact of tuning MVE-transport and their interactions with other organelles, notably the ER and lysosomes, on exosome release.

**Methods**: To study exosome release, we profited from CD63-pHluorin, a pH-sensitive reporter of MVE-plasma membrane fusion that can be imaged by live-cell TIRF microscopy. We combined this live imaging approach with correlative light electron microscopy (CLEM) and conventional EV analysis methods. Using these approaches, we investigated the role of MVE-associated Rab-GTPases, molecular motors and inter-organelle contacts in the regulation of MVE targeting and fusion with the plasma membrane.

**Results**: Live imaging of MVE-plasma membrane fusion revealed subpopulations of MVEs that have distinct abilities to release exosomes. Combined with conventional EV analysis methods this approach identified endosomal molecular motors involved in the targeting of MVEs towards the plasma membrane for fusion. Furthermore, manipulating the interactions of MVEs with the Endoplasmic reticulum affects their ability to fuse not only with lysosomes but also with the plasma membrane.

**Summary/Conclusion**: Our data show the interdependency of several key mechanisms that modulate MVE homeostasis, inter-organelle contacts and motility, and subsequent exosome release. An increased understanding of the processes involved in MVE exocytosis might contribute to the development of novel approaches to target and manipulate exosomal communication in disease.

**Funding**: This study was funded by Fondation pour la Recherche Medicale (AJE20160635884) to G.v.N., the EMBO ALTF 1383-2014 to F.V., the Fondation ARC fellowship (PJA 20161204808) to F.V., LabEx celthisphybio to G.v.N. and F.V., the CCA travel grant to M.B. and the curie International PhD program to R.P.

LB03.02

Naturally and targeted engineered DNA cargo in bacterial extracellular vesicles control rates of interspecies horizontal gene exchange and can be regulated by environmental cues

Frances Tran; James Boedicker

University of Southern California, Los Angeles, USA

**Background**: Most bacteria release extracellular vesicles (EVs). Recent studies have found these vesicles are capable of gene delivery; however, the consequences of vesicle-mediated transfer on the patterns and rates of gene flow within microbial communities remains unclear. Previous studies have not determined the impact of both the genetic cargo and the donor and recipient species on the rate of vesicle-mediated gene exchange. Therefore, we look at the genetic and biophysical controls of EV production, DNA loading and vesicle mediated uptake. We dissect plasmid dynamics, including plasmid origin, size and copy number, and their regulation on vesicle-mediated gene transfer. Additionally, we demonstrate that antimicrobial peptides released by bacteria can control the production, loading and uptake of DNA loaded vesicles. Our work examines the potential for EVs as a mechanism of gene transfer within heterogeneous microbial populations.

**Methods**: Different plasmids were genetically engineered to have different characteristics. EVs were harvested from different species of Gram-negative microbes carrying these different plasmids. The rates of gene transfer into recipient species were measured. A synthetic system was also engineered in bacterial cells to target and load plasmid DNA into EVs.

**Results**: We demonstrated that vesicles enable gene exchange between diverse species of Gram-negative bacteria, and that the identity of the genetic cargo, donor strain and recipient strain all influence gene transfer rates. Each species released and acquired vesicles containing genetic material to a variable degree, and the transfer rate did not correlate with the relatedness of the donor and recipient species. Our synthetic system increased the amount of DNA being loading by tethering plasmids to the membrane. This subsequently controlled the rate of gene exchange. We also show that vesicle production and uptake can be regulated by antimicrobial peptides.

**Summary/Conclusion**: Our results suggest that EVs may be a general mechanism to exchange non-specialized genetic cargo between bacterial species. Taken together, we can create a framework for how horizontal gene transfer by EVs occurs in the environment as an adaptive tool to other bacterial species and/or environmental cues. With this we can engineer systems to load DNA into EVs and to increase targeted uptake.

LB03.03

Harnessing extracellular vesicles from human red blood cells for gene therapies against cancer

Minh TN. Le; Muhammad Waqas Usman; Tin Pham; Luyen Vu; Boya Peng; Jiahai Shi

City University of Hong Kong, Kowloon, Hong Kong

**Background**: Extracellular vesicles (EVs) are natural RNA carriers that may act as biocompatible delivery vehicles for gene therapies. Billions of cells are often required to obtain sufficient EVs for therapies as the yield of EV purification is low when using stringent methods to ensure high purity and good quality of the EVs. Immortalized cells are often used for EV purification but they are not suitable for clinical purposes due to the risk of oncogenesis. Hence, we sought to harness EVs from the most abundant primary cell type, the red blood cells (RBCs) which make up 84% all cells in the human body. Human RBCEVs are ideal for clinical application because RBCs are readily available from blood bank and even from patients’ own blood; and RBCs have no DNA hence there is no risk of horizontal gene transfer.

**Methods**: EVs were purified from Red-Cross donated blood samples using ultracentrifugation with sucrose cushion and electroporated with antisense oligonucleotides (ASO) or Cas9 mRNA and gRNAs for miRNA inhibition or genome editing, respectively, in leukaemia cells and breast cancer cells *in vitro* and in xenograft mouse models.

**Results**: We obtained 10^13^–10^14^ RBCEVs per blood unit, with a homogenous population of ~100–200 nm EVs. RBCEVs contained EV markers and abundant haemoglobin A. RBCEVs were taken up robustly by leukaemia cells and provided better delivery of ASOs than commercial transfection reagents with no observable cytotoxicity. Local or systemic delivery of ASO-loaded RBCEVs provided efficient knockdown of the oncogenic miR-125b and suppression of breast cancer or leukaemia growth in xenograft mouse models. Moreover, RBCEVs were able to deliver Cas9 mRNA and gRNA to leukaemia cells for genome editing.

**Summary/Conclusion**: This study demonstrates a novel approach for delivery of RNAs to cancer cells using RBCEVs that is scalable, safe and efficient.

**Funding**: This project is funded by the Hong Kong Health and Medical Research Fund (9211101), the Hong Kong Research Grants Council (9048069), the National Natural Science Foundation of China (81602514, 81773246 and 81770099) and Shenzhen Science and Technology Innovation Fund (JCYJ20170413115637100).

LB03.04

Exosomes as drug delivery vehicles for therapeutic proteins to the brain

Elena V. Batrakova; Matthew Haney; Natalia Klyachko; Yuling Zhao; Alexander Kabanov

University of North Carolina at Chapel Hill, Chapel Hill, USA

**Background**: The successful systemic delivery of therapeutic proteins to the brain has been hampered by poor penetration across the blood–brain barrier. The use of exosomes as “natural nanoparticles” to deliver therapeutic proteins to the brain offers crucial advantages compared to other nanoparticulate drug delivery systems. Comprised of natural lipid bilayers with the abundance of adhesive proteins, exosomes readily interact with cellular membranes of target cells, and pass through biological barriers. We posit that exosomes secreted by monocytes and macrophages can provide an unprecedented opportunity to avoid entrapment in mononuclear phagocytes (as a part of a host immune system), and at the same time enhance delivery of incorporated therapeutic proteins to target cells ultimately increasing drug therapeutic efficacy. In light of this, we developed a new exosome-based delivery system for various potent therapeutic proteins, including neurotrophic factors, antioxidants and lysosomal enzymes.

**Methods**: Therapeutic proteins were loaded into exosomes *ex vivo* using two approaches: (i) transfection of exosome-producing cells with therapeutic protein-encoding plasmid DNA or (ii) incorporation the drug into naive exosomes released by macrophages. The second approach utilized various methods, including permeabilization of exosomal membranes with saponin, sonication, extrusion or freeze-thaw cycles to achieve high loading efficiency.

**Results**: A reformation of exosomes upon sonication and extrusion, or permeabilization with saponin resulted in high-loading efficiency, sustained release, and preservation of the therapeutic protein against proteases degradation. Exosomes were readily taken up by neuronal cells *in vitro*. A considerable amount of exosomes was detected in the inflamed brain in mice following intranasal administration. Exosomal formulations provided significant neuroprotective effects in *in vitro* and *in vivo* models of neurodegeneration.

**Summary/Conclusion**: Overall, exosome-based formulations have a potential to be a versatile strategy to treat different devastating neurodegenerative disorders.

Plenary Session 4: Tissue Environment and Architecture Chairs: Eva Maria Kramer-Albers; Hector Peinado Location: Auditorium 10:30–11:00

PL 6

How does a linear sequence of DNA become a 3-dimensional tissue and why cancer cells forget!

Mina Bissell

University of California – Berkeley, Berkeley, USA

Sunday, 6 May 2018

10:30 Mina J. Bissell

**How does a linear sequence of DNA become a 3-dimensional tissue and why cancer cells forget!**

Mina Bissell

ISEV2018 Annual Meeting

“If there is one generalization that can be made from all tissue and cell culture studies with regards to the differentiated state, it is this: Since most, if not all, functions are changed in culture qualitatively and/or quantitatively, there is no constitutive gene expression in higher organisms; i.e. the differentiated state is unstable and the (micro)environment regulates gene expression.”

(Mina Bissell, International Review of Cytology, 1981)

Then I spent the next 4 decades to examine if the above statement is correct and searched for mechanisms. I will discuss the significance of interactions between the extracellular matrix and the cytoskeleton with nucleus and chromatin, and why it needs to be dynamic and reciprocal. I will also discuss the mechanism of how the normal cells maintain homeostasis and what may go wrong when they forget and become cancerous.

FA 03: Featured Abstracts – Session 3 Chairs: Rienk Nieuwland Location: Auditorium 11:00–12:30

FA3.01

Recombinant extracellular vesicles: biological reference material to standardize extracellular vesicle research

Edward Geeurickx ^1^; Elisa Heyrman^1^; Jan Van Deun^2^; Lien Lippens^3^; Joeri Tulkens^3^; Bert Dhondt^4^; Glenn Vergauwen^2^; Kris Gevaert^5^; Francis Impens^6^; Ikka Miinalainen^7^; Pieter-Jan Van Bockstal^8^; Thomas De Beer^9^; Marca H.M. Wauben^10^; Esther N.M Nolte-’t-Hoen^11^; Johan Swinnen^12^; Sven Eyckerman^13^; Pieter Mestdagh^14^; Jo Vandesompele^14^; Geert Braems^15^; Hannelore Denys^16^; Olivier De Wever^2^; An Hendrix^2^

^1^Laboratory of Experimental Cancer Research, Department of Radiation Oncology and Experimental Cancer Research, Faculty of medicine and health sciences, Ghent University, Ghent, Belgium, Gent, Belgium; ^2^Laboratory of Experimental Cancer Research, Department of Radiation Oncology and Experimental Cancer Research, Cancer Research Institute Ghent (CRIG), Ghent University, Ghent, Belgium, Ghent, Belgium; ^3^Laboratory of Experimental Cancer Research, Department of Radiation Oncology and Experimental Cancer Research, Faculty of medicine and health sciences, Ghent University, Gent, Belgium; ^4^Laboratory of Experimental Cancer Research, Department of Radiation Oncology and Experimental Cancer Research, Faculty of medicine and health sciences, Ghent University, Ghent, Belgium, Ghent, Belgium; ^5^Department of Biochemistry, Ghent University, VIB Medical Biotechnology Center, Ghent, Belgium., Ghent, Belgium; ^6^Department of Biochemistry, Ghent University, VIB Medical Biotechnology Center, Ghent, Belgium., Gent, Belgium; ^7^Biocenter Oulu, Department of Pathology, Oulu University Hospital, University of Oulu, Oulu, Finland, Oulu, Finland; ^8^Laboratory of Pharmaceutical Process Analytical Technology, Department of Pharmaceutical Analysis, Faculty of Pharmaceutical Sciences, Ghent University, Ghent, Belgium, Gent, Belgium; ^9^Laboratory of Pharmaceutical Process Analytical Technology, Department of Pharmaceutical Analysis, Faculty of Pharmaceutical Sciences, Ghent University, Ghent, Belgium., Gent, Belgium; ^10^Department of Biochemistry and Cell Biology Faculty of Veterinary Medicine, Utrecht University, Utrecht, The Netherlands; ^11^Department of Biochemistry & Cell Biology, Faculty of Veterinary Medicine, Utrecht University, Utrecht, The Netherlands, Leuven, Belgium, Leuven, Belgium; ^13^Department of Biochemistry, Ghent University, VIB Medical Biotechnology Center, Ghent, Belgium, Gent, Belgium; ^14^Center for Medical Genetics, Faculty of medicine and health sciences, Ghent University Hospital, Ghent University, Ghent, Belgium, Gent, Belgium; ^15^Department of Gynaecology, Faculty of Medicine and Health Sciences, Ghent University Hospital, Ghent University, Ghent, Belgium, Ghent, Belgium; ^16^Department of Medical Oncology, Ghent University Hospital, Ghent, Belgium

**Background**: Extracellular vesicles (EV) derived from liquid biopsies are emerging as potent biomarkers in health and disease. However, the complexity of liquid biopsies and the plethora of isolation and detection methods introduce variability that impedes interlaboratory concordance and clinical application. To evaluate and mitigate this variability, we developed recombinant EV (rEV) as a biological reference material with unique traceability, and physical and biochemical similarity to endogenous EV (eEV).

**Methods**: rEV are purified by density gradient (DG) from cell culture supernatant of HEK293T cells expressing an eGFP-tagged self-assembling protein that directs its own release. We studied the similarity of rEV and eEV using electron microscopy, zeta potential analysis, nanoparticle tracking analysis (NTA), lipidomics and proteomics. We assessed the traceability, stability and commutability of rEV using fluorescent NTA (fNTA), flow cytometry (FC), fluorescent microplate reader, quantitative real time PCR (qRT-PCR) and ELISA. rEV was spiked in plasma to calculate the recovery efficiency of EV isolation methods and to normalize eEV numbers in plasma using fNTA and ELISA.

**Results**: rEV shows biophysical and biochemical similarity to eEV such as morphology, zeta potential, size distribution, density and protein/lipid content. rEV can be accurately quantified by fNTA and FC in eEV-comprising samples. In addition, rEV behaves linearly with fluorescent intensity levels (*R* ^2^ = 0.969) and ELISA concentrations (*R* ^2^ = 0.978), and semi-logarithmic with qRT-PCR for eGFP mRNA (*R* ^2^ = 0.938). rEV is stable during multiple freeze-thaw cycles at −80°C and can be lyophilized without changes in morphology, concentration and aggregation. EV recoveries from plasma for size-exclusion chromatography, differential ultracentrifugation, DG and ExoQuick were respectively 100%, 10%, 30% and 100%. For the first time, we could calculate the normalized EV concentration for breast cancer patients, which was significantly higher than healthy individuals (1.77E11 vs 6.51E10 particles/mL plasma).

**Summary/Conclusion**: We developed rEV, a biological reference material for EV research which can be used as positive control, spike-in material or calibrator to ensure standardized EV measurements in various applications.

**Funding**: This study was funded by FWO-SB.

FA3.02

A genome-wide CRISPR screen using barcoded-microRNAs enables systematic interrogation of extracellular vesicle biology

Albert Lu; Suzanne Pfeffer

Stanford University, Stanford, USA

**Background**: Extracellular vesicles, including exosomes, mediate transfer of biologically active molecules such as microRNAs between neighbouring or distant cells. Many recent reports suggest that these vesicles may play important roles in both normal physiology and the pathogenesis of multiple disease states, including cancer. However, their biological significance and the underlying molecular mechanisms of their biogenesis and release remain largely unknown.

**Methods**: Taking advantage of current knowledge of exosome-mediated microRNA export, we designed artificially barcoded-exosomal microRNAs (bEXOmiRs) to study this process. bEXOmiR reporters contain a 15 nt-random sequence that can be detected quantitatively by next generation sequencing. Pooled cell cultures expressing single bEXOmiRs in each cell were used to monitor bEXOmiR abundance in exosome preparations. We could then express pairs of unique CRISPR guide (sg)RNAs associated with individual bEXOmiRs and determine how knockout of every gene influenced the release of microRNAs in isolated exosomes from large-scale suspension cultures of Cas9-edited cells. In order to perform a genome-wide screen, we have used this technology in conjunction with ~250,000 sgRNA-bEXOmiR pairs.

**Results**: As expected, next generation sequencing revealed a gene signature consistent with previous studies in the field, including known regulator positive controls. A large number of new genes were identified with previously unrecognized roles in extracellular microRNA export and their involvement has been validated by orthogonal assays.

**Summary/Conclusion**: Altogether, next generation sequencing analysis of barcoded microRNA abundance coupled with CRISPR-Cas9 screening represents a powerful and unbiased means for the systematic discovery of genes involved in microRNA packaging and extracellular vesicle release.

**Funding**: This research was funded by the U.S. National Institutes of Health grant DK37332.

FA3.03

Systematic methodological evaluation of a multiplex bead-based flow cytometry assay for detection of extracellular vesicle surface signatures

Oscar PB Wiklander^1^; Beklem Bostancioglu^1^; Ulrika Felldin^1^; Antje Zickler^2^; Florian Murke^3^; Joshua A. Welsh^4^; Björn Evertsson^5^; Xiu-Ming Liang^1^; Giulia Corso^1^; Manuela Gustafsson^1^; Dara Mohammad^1^; Constanze Wiek^6^; Helmut Hanenberg^6^; Michel Bremer^3^; Dhanu Gupta^1^; Mikael Björnstedt^2^; Jennifer Jones^7^; Bernd Giebel^8^; Joel Z. Nordin^1^; Samir El-Andaloussi^9^; André Görgens ^9^

^1^Clinical Research Center, Department for Laboratory Medicine, Karolinska Institutet, Stockholm, Sweden, Stockholm, Sweden; ^2^Division of Pathology F56, Department of Laboratory Medicine, Karolinska Institutet, Karolinska University Hospital Huddinge, Stockholm, Sweden, Stockholm, Sweden; ^3^Institute for Transfusion Medicine, University Hospital Essen, University of Duisburg-Essen, Essen, Germany, Essen, Germany; ^4^Molecular Immunogenetics and Vaccine Research Section, Vaccine Branch, CCR, NCI, NIH, Bethesda, MD, USA, Bethesda, USA; ^5^Department of Clinical Neuroscience, Karolinska Institutet, Karolinska University Hospital, Stockholm, Sweden, Stockholm, Sweden; ^6^Department of Hematology, University Hospital Essen, University of Duisburg-Essen, Essen, Germany, Essen, Germany; ^7^National Cancer Institute, Bethesda, USA; ^8^Institute for Transfusion Medicine, University Hospital Essen,, Essen, Germany; ^9^Clinical Research Center, Department for Laboratory Medicine, Karolinska Institutet, Stockholm, Hälsovägen, Sweden

**Background**: Extracellular vesicles (EVs) can be harvested from cell culture supernatants and from all body fluids and can be roughly classified as exosomes and microvesicles. Nowadays, it is commonly accepted in the field that there is a much higher degree of EV heterogeneity within these two subgroups than previously thought. Moreover, surface marker signatures of EVs are likely to be dependent on the cell type source and other multiple parameters. To date, no specific markers to even discriminate exosomes from microvesicles have been identified yet, and only few EV surface markers have been related to specific cell sources.

**Methods**: In the last few years, improved flow-cytometric assays have been developed. However, most assays are limited to dedicated instruments and require extensive operator expertise. In this study, we systematically evaluated the use of a multiplex bead-based assay which can be used with most standard flow cytometers for detection of human EV surface signatures.

**Results**: First, we assessed assay variability, sample stability over time, dynamic range and the limitations of this assay in terms of EV input quantity and EV concentration on different instruments. Next, we compared how EV sample quality affects assay results and found that this assay was fit for purpose in detecting, quantifying and comparing EV surface signatures in various sample types, including unprocessed cell culture supernatants, cell culture-derived EVs isolated by different isolation methods (differential ultracentrifugation, tangential flow filtration and size-exclusion chromatography), as well as biological fluids. Furthermore, we explored the use and limitations of this assay to assess heterogeneities in EV surface signatures by combining different sets of detection antibodies in EV samples derived from different immortalized cell lines. Finally, we demonstrate differential detection of EV surface markers in supernatants of primary haematopoietic progenitor cell subsets after short-term culture in low cell doses of 25,000 cells/well.

**Summary/Conclusion**: Taken together, we show that this multiplex bead-based flow cytometric assay allows robust, sensitive and reproducible detection and quantification of EV surface marker expression in various sample types, which will be extremely valuable for many researchers working in the EV field.

FA3.04

miRNAs expressed in brain and serum extracellular vesicles act as indicators of pre-clinical and clinical prion disease

Lesley Cheng ^1^; Camelia Quek^2^; Shayne A. Bellingham^3^; Laura J. Ellett^4^; Cathryn L. Ugalde^1^; Arun Khadka^1^; Amirmohammad N. Kenari^1^; Laura J. Vella^5^; Benjamin J. Scicluna^1^; Mitch Shambrook^1^; David I. Finkelstein^5^; Victoria Lawson^4^; Andrew F. Hill^1^

^1^Department of Biochemistry and Genetics, La Trobe Institute for Molecular Science, Australia, Melbourne, Australia; ^2^Department of Biochemistry and Genetics, La Trobe Institute for Molecular Science, La Trobe University, VIC, Australia, Melbourne, Australia; ^3^Department of Biochemistry and Molecular Biology, The University of Melbourne, Melbourne, VIC, Australia, Melbourne, Australia; ^4^Department of Pathology, The University of Melbourne, Melbourne, Victoria 3010, Australia, Melbourne, Australia; ^5^The Florey Institute of Neuroscience and Mental Health, Australia, Melbourne, Australia

**Background**: Prion diseases are transmissible neurodegenerative disorders distinguished by long pre-clinical incubation periods during which the infectious prion actively propagates in the brain and other tissues. We previously demonstrated that exosomes assist in the propagation of prions and that the exosomal RNA contents can be used as biomarkers of prion infection. As miRNA dictate phenotypic changes post-transcriptionally, this study aims to determine whether there is clinical utility of miRNA biomarkers during pre-clinical and clinical disease.

**Methods**: To define miRNA profile changes over the weeks of infection, a time-course using a mouse-adapted model of human prion disease and next-generation sequencing were employed to profile miRNA associated with prion infection. Thalamus brain sections and serum samples were collected at 3 and 13 weeks post-inoculation, representing the early and late pre-clinical stages of the disease. Tissues at the terminal, clinical stage were also collected upon persistent signs consistent with terminal prion disease.

**Results**: Profiling of miRNA expression revealed a collection of miRNAs that are differentially expressed during the development of prion disease in this model. Prion associated miRNAs identified in the thalamus tissue were also present in extracellular vesicles isolated from serum across each time-point demonstrating potential clinical utility. The differentially expressed miRNAs were also validated in extracellular vesicles isolated from brain tissue of the mice and in an organotypic brain slice model infected with the same prion strain.

**Summary/Conclusion**: The presence of these miRNAs may assist in identifying pathways involved in the pathogenesis of prion disease. This study has discovered clinically relevant miRNAs that may benefit the progress of diagnostic development to detect prion-related diseases such as Creutzfeldt-Jakob disease.

**Funding**: This study was funded by CJD Support Group Network (CJDSGN) and grants from the Australian National Health and Medical Research Council (N.H.M.R.C).

FA3.05

Non-invasive brain delivery with hybrid extracellular vesicles (EVs) for therapy of Machado-Joseph disease (MJD)

Patrícia Albuquerque ^1^; Magda Santana^1^; Rui J. Nobre^1^; Catarina Miranda^1^; Sara Lopes^1^; Teresa M. Ribeiro-Rodrigues^2^; Henrique Girão^2^; Célia Gomes^2^; Luis Almeida^1^

^1^Center for Neuroscience and Cell Biology (CNC), University of Coimbra, Coimbra, Portugal, Coimbra, Portugal; ^2^Institute for Biomedical Imaging and Life Sciences (IBILI), Faculty of Medicine, University of Coimbra, Coimbra, Portugal, Coimbra, Portugal

**Background**: Machado-Joseph disease (MJD) is a neurodegenerative disorder that associates with an expansion of a CAG tract in the *ATXN3 *gene, translating into a polyglutamine repeat expansion in the ataxin-3 protein. This leads to neuronal dysfunction in several regions of the CNS, resulting into diverse clinical manifestations and in premature death. Unfortunately, MJD still remains incurable. Extracellular vesicles (EVs), namely exosomes, have emerged as promising tools for efficient delivery of therapeutic strategies due to their stability, stealth capacity in bloodstream and the ability to overcome natural barriers in particular the blood–brain barrier (BBB). Association of EVs with adeno-associated virus (AAV) may take advantage of the best characteristics of the two systems. Therefore, the aim of this work was to develop an EV-AAV-based hybrid vector system that expresses on its surface a fusion protein including a transmembrane EV domain and a brain targeting peptide.

**Methods**: EVs were characterized regarding size, morphology, typical protein markers and AAV capsid protein content. To assess brain-targeting capacity, EVs were loaded with luciferase and biodistribution was evaluated by bioluminescence imaging. Hybrid EVs-AAV vectors encoding silencing sequences targeting mutant ataxin-3 mRNA were produced and intravenously injected in a transgenic mouse model of MJD. Controls were injected with EVs containing scramble sequences. Motor behaviour performance was evaluated, followed by neuropathological analysis for mutant ataxin-3 protein aggregates (IHC) and thickness of cerebellar layers.

**Results**: Hybrid EV-AAVs delivered genetic material to mice brains in a specific and efficient manner, as confirmed by bioluminescence imaging. Importantly, transgenic mice IV-administered with therapeutic EVs displayed better performance in behavioural assessment in comparison with controls. They also exhibited reduced mutant ataxin-3 levels and attenuation of cerebellar-associated neuropathology.

**Summary/Conclusion**: We have developed an original brain-targeted EV-AAV hybrid gene delivery system for the treatment of MJD, with the ability to cross the BBB through minimally invasive administration. This is the first EV-based gene delivery system for MJD treatment, constituting a promising delivery tool for other brain-related disorders.

ISEV2018 Wrap up SessionBasic Science Chair: Alissa WeaverClinical Chair: J. Brian Byrd12:30–12:50

Awards Ceremony and Closing Remarks12:50– 13:30

Industry Poster SessionLocation: Exhibit Hall03–05 May 201817:15–18:30

IP 01

A nano- and microparticle mix for CytoFLEX size standardization

George Brittain; Sergei Gulnik

Beckman Coulter Life Sciences, Miami, USA

**Background**: The CytoFLEX platform is distinguished by its exquisite sensitivity for size- and fluorescence-based detection. Utilizing VSSC, the CytoFLEX Flow Cytometer is capable of resolving 80 nm-latex and 100 nm-silica nanoparticles. Since most size-based microparticle mixes were not designed to assess nanoparticle detection, their size range is insufficient to adequately standardize the CytoFLEX. In addition, these mixes tend to contain a lot of contaminating particulate on the lower end, making more sensitive instruments appear to be noisier.

**Methods**: In order to address these issues, we prepared a better nano- and microparticle mix specifically for the CytoFLEX. The CytoFLEX Sizing Bead mix contains a mixture of fluorescent and non-fluorescent latex and silica NIST-traceable size standards between 80 nm and 2 m in size. In this poster, we demonstrate the performance of our sizing bead mix using a CytoFLEX-S B-R-V-N, and compare it with the commercially available ApogeeMix beads.

**Results**: The CytoFLEX-S was able to effectively detect and resolve all beads within the CytoFLEX Sizing Bead mix, with the 80 nm-latex beads perfectly resolved above the noise threshold. The ApogeeMix beads were noisier than the CytoFLEX beads, and the smallest bead (110 nm-latex) was resolved over half a decade higher than the noise threshold of the CytoFLEX.

**Summary/Conclusion**: Ultimately, our CytoFLEX Sizing Beads proved to better address the size-standardization requirements of the CytoFLEX than the ApogeeMix beads. These sizing beads can be used with any flow cytometer, allowing the user to extend their size standardization into the nanoparticle range (≤100 nm).

The CytoFLEX Sizing Bead mix and the CytoFLEX are for Research Use Only. The Beckman Coulter product and service marks mentioned herein are trademarks or registered trademarks of Beckman Coulter, Inc. in the United States and other countries. All other trademarks are the property of their respective owners.

IP 02

A prototype CytoFLEX for high-sensitivity, multiparametric nanoparticle analysis

George Brittain; Sergei Gulnik; Yong Chen

Beckman Coulter Life Sciences, Miami, USA

**Background**: Flow cytometry may be uniquely suited to address the needs of the EV field. It has the potential to provide for quantitative, particle-by-particle, multiplexed phenotypic analyses of EVs, and the ability to sort specific populations for functional analyses. However, currently available flow cytometers have significant limitations for the analysis of particles of exosome size. Indeed, the light-scatter intensity generated by exosomes on most flow cytometers is too low to be discriminated from optical and electronic noise, resulting in the common notion that only “the tip of the iceberg” of the EV population can be detected by flow cytometry.

**Methods**: To address these issues, we have developed a prototype nanoparticle analyser based on the technology of the CytoFLEX platform. Our current prototype can detect and resolve 30 nm-polystyrene and 50 nm-silica nanoparticles. It has enhanced fluorescence sensitivity due in parts to modifications that were made to enhance size resolution. And, it has very minimal background noise due to enhancements in noise filtering and coincidence reduction.

**Results**: In this poster, we will demonstrate the VSSC-based size resolution and fluorescence sensitivity of our prototype using a variety of NIST-traceable size standards and fluorescent nanoparticles. We will demonstrate the resolution of bead mixes including particles between 40 and 300 nm, as well as decades of separation for 40–100 nm fluorescently labelled nanoparticles.

**Summary/Conclusion**: Ultimately, we have built upon the already exquisite sensitivity of the CytoFLEX platform in order to provide the EV field with an easy-to-use, multiparametric instrument that can effectively detect and resolve exosomes and other biological nanoparticles.

This Prototype Nanoparticle Analyser is for Research Use Only. The results from this prototype may not reflect the performance of the final product. The Beckman Coulter product and service marks mentioned herein are trademarks or registered trademarks of Beckman Coulter, Inc. in the United States and other countries.

IP 03

A novel platform for a scalable, selective, and easy method to isolate extracellular vesicles

Victoria Portnoy; Frank Hsiung

System Biosciences (SBI), Palo Alto, USA

**Background**: Extracellular vesicles (EVs) are small natural nanoparticles present in many biological fluids, such as plasma, urine, milk and saliva. As major mediators of extracellular signalling and cell–cell communication, extracellular vesicles are now being studied as promising sources of biomarkers and are attractive targets in both research and diagnostic applications. Due to the insight that extracellular vesicles can offer into the diagnosis and treatment of certain diseases, primarily cancers and neurodegenerative diseases, there is a great need to isolate EVs from biological fluids. The current approaches to EV isolation, including ultracentrifugation and polymer-based precipitation, have limitations when it comes to scalability, selectivity and ease of use. The aim of our work is to develop a total EV isolation method that will overcome these limitations.

**Methods**: Our novel column chromatography-based isolation platform, designed to be polymer-free, works in wide range of settings, while offering highly efficient recovery of isolated EVs in their native form with minimal contamination. High binding capacity allows isolation of EVs ranging from micrograms to milligrams of protein equivalent and will be compatible with biofluid volumes ranging from ~100 µL to >10 mL, thereby providing flexibility for various input amounts. Scaling up to 25–100 mL volume of starting material is possible as well. An additional advantage of our approach is its adaptability to a 96-well plate format for high-throughput processing of samples.

**Results**: Data will be presented confirming isolation of exosomes via nanoparticle tracking analysis (NTA), and an additional fluorescent NTA analysis for more accurate quantification. The presence of canonical EV markers (CD63, CD9 and TSG101) and the absence of common contaminants (Immunoglobulins, albumin and lipoproteins) will be shown via immunoblotting analysis. In addition, morphological appearance of EVs will be documented using transmission electron microscopy (TEM), while functionality of isolated exosomes will be shown via uptake studies, mass spectrometry and NGS analysis.

**Summary/Conclusion**: The principle of our novel isolation chromatography-based platform along with isolation strategy and results will be presented.

IP 04

A protocol for rapid extraction of high quality RNA from urinary EVs used for the detection of TMPRSS2:ERG fusion transcripts in prostate cancer subjects

Martin Schlumpberger ^1^; Nicole Pickavé^1^; Karolin Spitzer^1^; Daniel Enderle^2^; Mikkel Noerholm^2^; Markus Sprenger-Haussels^1^

^1^QIAGEN GmbH, Hilden, Germany; ^2^Exosome Diagnostics GmbH, Martinsried, Germany

**Background**: Efficient isolation of urinary exosomes and other extracellular vesicles (EVs) and their nucleic acid content from urine presents particular challenges due to the significant variability in major and minor constituents of this biofluid, many of which are potent inhibitors of qRT-PCR.

We present optimized workflows for isolation of both intact mRNA (and other long RNAs) as well as miRNA (and other short RNA species) from urine, and demonstrate their use for miRNA and mRNA biomarker detection, including a research cohort of individuals with prostate cancer.

**Methods**: In this research study, intact EVs from urine were bound to an affinity membrane in spin column format, lysed *in situ* for RNA isolation and separation into long and short RNA fractions. For analysing clinical samples, qRT-PCR was used to quantify prostate cancer specific TMPRSS2:ERG (T2:E) fusion transcripts and compared to expression of KLK3 (PSA) in 20 mL urine from 16 individuals scheduled for radical prostatectomy.

**Results**: Applying the extraction to a research study, T2:E fusion transcripts from prostate cancer can be detected consistently in urine from 10 out of 16 samples, which is the expected frequency for this population.

**Summary/Conclusion**: The novel workflow to isolate exoRNA from urinary EVs is shown to avoid co-purification of inhibitors from the samples and recover RNA with high reproducibility.

IP 05

Affinity purification of membrane vesicles

Oleg Guryev; Tatyana Chernenko; Majid Mehrpouyan; Gulam Shaikh; Marybeth Sharkey

BD Biosciences, San Jose, USA

**Background**: In this work, we describe a new affinity method for purification of membrane vesicles. EVs and liposomes can be considered as membrane vesicles – all of them have bilayer lipid membrane. EVs are nanosized (20–5000 nm), membrane-bound vesicles released from cells that can transport cargo – including DNA, RNA and proteins – between cells as a form of intercellular communication. Liposomes are artificially prepared nano-/micro-size (50–5000 nm) vesicles of single or multiple lipid bilayers. In the last decades, they have become very important biomaterials with growing application in life science research, pharmacology and biotechnology. We here implement liposomes as a model system to assess methods and protocols of EVs purification.

**Methods**: First, we modify membrane vesicles with amphiphilic reagent. Second, we apply principles of affinity chromatography for separation of the labelled vesicles from the solution. Hydrophilic part of the reagent, PEG, helps to keep the molecule in aqueous environment, a hydrophobic molecule from hydrophobic part can rapidly anchor to the phospholipid membrane of the vesicles and an affinity probe is designed to interact with insoluble beads.

**Results**: We have prepared liposomes composed of 42 mol% PMPC, 14 mol% DOPS, 13.5 mol% DOPE, 30 mol% cholesterol and 0.5 mol% DHPE-TRITC by BD centrifugation procedure using sequential centrifugation in 200 nm and 80 nm devices. Size of the liposomes determined by dynamic light scattering (DLS) was 121.5 ± 1.9 nm. These liposomes were modified with amphiphilic reagent with affinity probe, following by the binding with insoluble beads. Size of the liposomes after elution from the beads was 125.0 ± 2.4 nm.

**Summary/Conclusion**: It can be concluded that amphiphilic reagent with affinity probe can be used for modification of membrane vesicles, and liposomes, in particular. Following the modification, the liposomes can be isolated. Isolation of liposomes does not affect their size. We believe that the combination of vesicles labelling with amphiphilic reagent and affinity beads allows for purification of a broad range of EVs without altering their structure and functionality. Multiple elution options allow to choose the most appropriate one.

IP 06

Fluorescence and 3D light scatter activated sorting of small particles

Oliver Kenyon

Apogee Flow Systems Ltd, Hemel Hempstead, United Kingdom

**Background**: Scattered light measurements from individual extracellular vesicles (EVs) offer excellent size resolution but the complex relationship between particle size and the amount of light scattered at different collection angles makes it difficult to infer particle size from a flow cytometer’s data. When comparing data between flow cytometers the difficulties are compounded by differences in light scatter illumination and collection angles. Fluorescent probes are an equally important tool for the study of EVs but the small size of EVs means that their fluorescence is weak and when the measured signals are close to the flow cytometer’s noise limit, small differences in the fluorescence sensitivity of the flow cytometer may give significantly different results. Standardization of EV enumeration is therefore a challenging task.

**Methods**: Apogee has developed a range of samples containing a continuum of particle size and of known refractive index which offer a “snapshot” of a flow cytometer’s light scatter performance and which allow a particle size calibration to be performed. Furthermore Apogee has developed a high speed actuator capable of sorting particles flowing in a liquid shortly after they have passed through a flow cytometer’s laser(s).

**Results**: We present data showing the limitations of a 2 dimensional calibration solution (2 light scatter angle ranges) and the benefits offered by a 3 dimensional solution (3 light scatter angle ranges). High resolution scattered light and fluorescence measurements may be used to trigger the novel high speed sorting actuator.

**Summary/Conclusion**: Light scatter and fluorescence flow cytometer signals may be used to trigger a novel actuator so that EVs and other small particles may be sorted to a high level of purity in liquid while minimizing aerosol biohazards. The ability to physically sort small particles of interest within a well-defined size range offers a potentially powerful means to validate and standardize EV analyses.

IP 07

Highly efficient and easy to use exosome isolation spin column with porous glass filter

Shuji Yamazaki ^1^; Keita Aoki^2^; Hiroshi Yukawa^2^; Daisuke Onoshima^2^; Naoto Kihara^1^; Kumiko Takahashi^1^; Tetsuya Kotani^1^; Hidefumi Odaka^1^; Kenji Ishikawa^2^; Masaru Hori^2^; Yoshinobu Baba^2^

^1^Asahi Glass Co., Ltd., Chiyoda-ku, Japan; ^2^Nagoya University, Nagoya, Japan

**Background**: Exosome is one kind of extracellular vesicles that contain some kinds of RNAs. The clinical applications of exosomes are expected for diagnosis and treatment of diseases. However, existing isolation methods were inefficient and complicated. We developed highly efficient and easy to use exosome isolation spin column with a porous glass filter.

**Methods**: The spin column with porous glass filter was developed by Asahi Glass Co., Ltd. Biological solution: serum and cell culture medium were passed through the filter using a conventional desk-top centrifugation. The capture efficiency of exosome was evaluated, and the captured exosome in the filter was observed using SEM. miRNA profiles from captured exosome was compared with ultracentrifuge (UC) and commercially available isolation kit.

**Results**: Capture efficiency of exosome from cell culture medium with our spin column was more than 90% and the exosome-like vesicles in the filter was observed clearly by SEM. Coverage of RNA species with our spin column was clearly better than UC and other isolation kit.

**Summary/Conclusion**: We developed exosome isolation method using porous glass filter. The capture efficiency and miRNA coverage of exosome were evaluated. The result showed our method was highly effective and easy to use.

IP 08

Liquid biopsy on a chip: microfluidic isolation and RNAseq analyses of cancer derived exosomes

Navneet Dogra

Icahn School of Medicine at Mount Sinai, New York City, USA

**Background**: Exosomes are an exciting target for “liquid biopsies”. However, isolation of exosomes and detection of their surface biomarkers remains an ongoing challenge. We have developed a nanoscale DLD (deterministic lateral displacement) device that brings capabilities with size-based sorting of colloidal particles at the tens of nanometres scale. Furthermore, we have successfully demonstrated on-chip separation of exosomes and detection of important biomarker on exosomes derived from cancer cells.

**Methods**: Nanofluidic pillar array is manufactured in SiO_2_ mask using optical contact lithography and deep ultra violet lithography. Exosomes are derived from prostate cancer cell lines and patients.

**Results**: We demonstrate precise size-based separation and exosomes. Once isolated, we performed small RNAseq analyses of exosomes derived from cancer cells and patient samples.

**Summary/Conclusion**: These exciting preliminary results indicates the potential of our nanoDLD technology for sorting of exosomes and detection of biomarkers from plasma, urine, serum or circulating tumour-derived exosomes.

IP 09

Microfluidic resistive pulse sensing (MRPS) validated as a rapid and practical method for evaluating EV enrichment techniques

Jean-Luc Fraikin ^1^; Jancy Johnson^2^; Ian Dixon^2^; Bill Kalionis^3^; Gregor Lichtfuss^4^

^1^Spectradyne LLC, Torrance, USA; ^2^Exopharm Pty Ltd, Melbourne, Australia; ^3^The Royal Women’s Hospital, Parkville, Australia, Melbourne, Australia; ^4^Exopharm Pty Ltd, Melbourn, Australia

**Background**: Delivering commercial value for extracellular vesicles (EVs) as therapeutics requires improved techniques for their isolation and enrichment. However, the development of these techniques is hindered by a lack of practical technologies for accurate EV quantification. In this study, we validated microfluidic resistive pulse sensing (MRPS) as a rapid, practical tool for characterizing the size exclusion chromatography (SEC) method of EV purification.

**Methods**: DMSC25 mesenchymal stem/stromal cells were cultured to 70% confluence in growth media. Cells were then cultured for 2 days in chemically defined, vesicle-free medium. Conditioned medium (50 ml) was then concentrated by sequential ultracentrifugation and resuspended in SEC buffer and applied to a GE NAP-5 column for further purification. Fractions were collected and total EV concentration measured using MRPS on the size range of 65–400 nm. UV absorption, an orthogonal technique to MRPS, was used to quantify the total protein in each fraction. Results of each of the techniques were compared.

**Results**: As expected, MRPS measurements showed a clear peak in total particle concentration in column fractions 3–5, in which EVs are known to elute. Importantly, however, particle size distributions obtained by MRPS showed that each eluted fraction contained a broad range of particle sizes spanning the full measured range of 65–400 nm, and that elution in different fractions did not significantly affect the size distribution profiles. Significant differences were observed between the two techniques for measurements of the non-EV fractions: a peak in total protein was detected in fractions 7 and 8, while no corresponding peak in particle concentration was observed, suggesting the protein in these fractions was not bound in the form of solid particles.

**Summary/Conclusion**: MRPS was validated as a practical tool for characterizing EV purification methods. In addition to demonstrating good agreement with orthogonal techniques, MRPS provided important insight about the limitations of SEC as a size-separation technique – broad particle size distributions were observed in each fraction. MRPS is therefore an important tool for accurate characterization of EV purification methods as they are being developed.

IP 10

Setting Benchmarks for Robust and Reproducible Nanoparticle Tracking Analyses

Agnieszka Siupa ^1^; Duncan Griffiths^2^; Pauline Carnell-Morris^1^

^1^Malvern Panalytical, Malvern, United Kingdom; ^2^Malvern Panalytical, Westborough, USA

**Background**: Nanoparticle Tracking Analysis (NTA) data has become the predominant technique for size and concentration of extracellular vesicles (EV). As the field has matured, the requirement for more robust results has increased; however, there remains concern about the reproducibility and operator-dependence of NTA.

**Methods**: A multi-round interlaboratory comparison (ILC) of NanoSight instruments was recently completed to establish a benchmark for repeatability and reproducibility for the NTA technique. Following refinement of the analytical methods, the size and concentration was proven to be robust and reproducible for multiple sample types in monomodal, binary or multimodal mixtures.

Additional improvements for concentration data were introduced in the final round of testing, further compensating for variability between both instruments and users.

The recent introduction of the Sample Assistant autosampler also eliminated operator-dependent variability from almost all the analytical steps and was shown to improve the repeatability and reproducibility of data, while enabling walk-away analysis of up to 96 samples in a single run.

**Results**: With sufficiently detailed methods, percentage coefficients of variation (%CV) were less than 5% for monodisperse samples. Application of the concentration calibration resulted in sizing accuracy above 97%, and concentration CV less than 9%.

Measurements of exosome samples utilizing the Sample Assistant were very reproducible, even while requiring only a fraction of the time of manual analyses. Concentration linearity with dilutions compared well to an experienced user.

**Summary/Conclusion**: The ILC process show highly reproducible results are available if methods are sufficiently specific to eliminate the variability. This is further aided by developments in the software and hardware that further improve the robustness of NTA analyses.

**Funding**: This work received funding from the European Commission under FP7 Capacities Programme under grant Agreement No. 262163 (QualityNano) and from European Union’s Horizon 2020 research and innovation programmes under grant agreement No 646002 (NanoFASE) and under grant agreement No 721058 (B-SMART).

IP 11

Nanoflow cytometry: quantitative and multiparameter analysis of single extracellular vesicles (40–150 nm)

Ling Ma ^1^; Jinyan Han^1^; Shaobin Zhu^2^; Ye Tian^3^; Xiaomei Yan^3^

^1^NanoFCM Inc., Xiamen, China, Xiamen, China (People’s Republic); ^2^nanoFCM, Inc, Xiamen, China (People’s Republic); ^3^Department of Chemical Biology, Xiamen University, Xiamen, China, Xiamen, China (People’s Republic)

**Background**: Extracellular vesicles (EVs) are nano-sized vesicles derived from cells, which play important roles in intercellular communication by delivering proteins, nucleic acids and lipids between cells. Compared with microvesicles with sizes ranging from 100 to 1000 nm, single exosome characterization remains more challenging due to the extremely small size (30–150 nm), heterogeneity, and the trace amount of molecular content. Here, we use Flow NanoAnalyzer for the quantitative and multiparameter analysis of EVs at single particle level.

**Methods**: EVs were prepared from cultured medium and human plasma by differential ultracentrifugation. Sizing analysis of EVs was performed by using S16-Exo (NanoFCM) as size standards. To validate the fluorescence capacity of the instrument, both intrinsically fluorescent and labelled EVs were characterized.

**Results**: We have demonstrated the sensitivity of Flow NanoAnalyzer by detecting single silica nanoparticles, the fluorescence sensitivity of single R-PE molecule has also been verified. The size and concentration of EVs can be acquired directly from the software, both intrinsic and labelled fluorescence could be detected individually.

**Summary/Conclusion**: The Flow NanoAnalyzer platform enables quantitative and multiparameter analysis of single EVs down to 40 nm, which is distinctively sensitive, yet high-throughput, and shows great potential in liquid biopsy applications.

